# Supplementary material for: AI powered quantification of nuclear morphology in cancers enables prediction of genome instability and prognosis
Source: NPJ Precis Oncol. 2024 Jun 19;8:134. doi: 10.1038/s41698-024-00623-9 (PMC11187064; doi:10.1038/s41698-024-00623-9)
Supplement: Supplementary file 1 — Reporting Summary [file 41698_2024_623_MOESM1_ESM.pdf]

nature portfolio

Corresponding author(s): John Abel

Last updated by author(s): May 7, 2024

Reporting Summary

Nature Portfolio wishes to improve the reproducibility of the work that we publish. This form provides structure for consistency and transparency in reporting. For further information on Nature Portfolio policies, see our Editorial Policies and the Editorial Policy Checklist.

Please do not complete any field with "not applicable" or n/a. Refer to the help text for what text to use if an item is not relevant to your study.
For final submission: please carefully check your responses for accuracy; you will not be able to make changes later.

Statistics

For all statistical analyses, confirm that the following items are present in the figure legend, table legend, main text, or Methods section.

n/a	Confirmed	
☐	☒	The exact sample size (*n*) for each experimental group/condition, given as a discrete number and unit of measurement
☐	☒	A statement on whether measurements were taken from distinct samples or whether the same sample was measured repeatedly
☐	☒	The statistical test(s) used AND whether they are one- or two-sided<br>*Only common tests should be described solely by name; describe more complex techniques in the Methods section.*
☐	☒	A description of all covariates tested
☐	☒	A description of any assumptions or corrections, such as tests of normality and adjustment for multiple comparisons
☐	☒	A full description of the statistical parameters including central tendency (e.g. means) or other basic estimates (e.g. regression coefficient) AND variation (e.g. standard deviation) or associated estimates of uncertainty (e.g. confidence intervals)
☐	☒	For null hypothesis testing, the test statistic (e.g. *F*, *t*, *r*) with confidence intervals, effect sizes, degrees of freedom and *P* value noted<br>*Give P values as exact values whenever suitable.*
☒	☐	For Bayesian analysis, information on the choice of priors and Markov chain Monte Carlo settings
☒	☐	For hierarchical and complex designs, identification of the appropriate level for tests and full reporting of outcomes
☐	☒	Estimates of effect sizes (e.g. Cohen's *d*, Pearson's *r*), indicating how they were calculated

Our web collection on statistics for biologists contains articles on many of the points above.

Software and code

Policy information about availability of computer code

Data collection

TCGA histopathology images were downloaded from https://www.cancer.gov/about-nci/organization/ccg/research/structural-genomics/tcga

Machine learning inference was performed on a proprietary machine learning platform built in the Python languange, and machine learning training was performed in Python via PyTorch using the TorchVision library.

PathExplore (PathAI, Boston, MA) models specific to breast cancer (BRCA), lung adenocarcinoma (LUAD), and prostate adenocarcinoma (PRAD) were used to assign cell classes to each segmented nucleus.

Data analysis

Data analyses in this study were performed in Python. All source code for reproducing correlational analyses and molecular predictions can be found at https://github.com/Path-AI/nuclear-features.

For manuscripts utilizing custom algorithms or software that are central to the research but not yet described in published literature, software must be made available to editors and reviewers. We strongly encourage code deposition in a community repository (e.g. GitHub). See the Nature Portfolio guidelines for submitting code & software for further information.

Data

Policy information about availability of data

All manuscripts must include a data availability statement. This statement should provide the following information, where applicable:

- Accession codes, unique identifiers, or web links for publicly available datasets
- A description of any restrictions on data availability
- For clinical datasets or third party data, please ensure that the statement adheres to our policy

Histopathology images from the Cancer Genome Atlas dataset are available at https://www.cancer.gov/about-nci/organization/ccg/research/structural-genomics/tcga. Images and annotations of nuclei from the (training set, validation set, test set, OOD-2 dataset, and TCGA datasets) can be found at https://github.com/Path-AI/nuclear-features. Images and annotations of nuclei from the OOD-1 dataset will be shared upon written request. Access to feature tables, cell-, tissue-, and nuclei-type heatmaps, as well as usage of cell- and tissue-type classification models, are available upon reasonable request to academic investigators without relevant conflicts of interest for non-commercial use who agree not to distribute the data. Access requests can be made to publications@pathai.com.

Model parameters for nuclei, cell, and tissue models, and codes model training, inference, and feature extractions are not disclosed. Access requests for such a code will not be considered to safeguard PathAI's intellectual property. All source code for reproducing correlational analyses and molecular predictions can be found at https://github.com/Path-AI/nuclear-features.

Gene expression data was acquired from the Genomic Data Commons (GDC)-processed TCGA BRCA cohort (release 18.0) from the UCSC Xena data portal: https://gdc.xenahubs.net

Aneuploidy score was obtained from the dataset published by Taylor et al: https://gdc.cancer.gov/about-data/publications/pancan-aneuploidy.

Homologous recombination deficiency (HRD) score was obtained from the dataset published by Marquard et al: https://biomarkerres.biomedcentral.com/articles/10.1186/s40364-015-0033-4.

Research involving human participants, their data, or biological material

Policy information about studies with human participants or human data. See also policy information about sex, gender (identity/presentation), and sexual orientation and race, ethnicity and racism.

Reporting on sex and gender	Sex data for each individual in the TCGA cohorts were obtained from TCGA. No sex- or gender-based analyses were performed as these were outside the scope of the current study.
Reporting on race, ethnicity, or other socially relevant groupings	Analyses on race, ethnicity, and other socially relevant groupings were not performed as these were outside the scope of the current study.
Population characteristics	Patient characteristics (including age, sex, tumor stage, whole genome doublings, and survival data) are provided in Table 3 in our manuscript for each TCGA dataset used.
Recruitment	N/A
Ethics oversight	N/A

Note that full information on the approval of the study protocol must also be provided in the manuscript.

Field-specific reporting

Please select the one below that is the best fit for your research. If you are not sure, read the appropriate sections before making your selection.

☒ Life sciences ☐ Behavioural & social sciences ☐ Ecological, evolutionary & environmental sciences

For a reference copy of the document with all sections, see nature.com/documents/nr-reporting-summary-flat.pdf

Life sciences study design

All studies must disclose on these points even when the disclosure is negative.

Sample size	No statistical methods were used to predetermine sample size. Sample size was determined by the number of cases available in the datasets used.
Data exclusions	Histopathology images were excluded if they did not meet basic quality control criteria as determined by board-certified pathologists.<br><br>TCGA slides were selected to be the DX1 (primary diagnostic) slide for each case, and only 40X magnification slides were used. No outlier exclusion was performed.

Replication	To assess model performance, evaluation was replicated on two out of distribution datasets, which contained tissue not seen in the training, validation, or test sets.
Randomization	We followed standard approaches for randomizing whole-slide images into training, validation, and test sets. Training data variation and number of annotations were selected to exceed previously used standards in the field and exhibit wide variation in tissue morphology as subjectively assessed by study pathologists.<br><br>Predictive and classification models were developed using iterative stratified cross-validation as described in the Methods, as well as in the code.
Blinding	Investigators were not blinded because no animal or human research subjects were used.

Reporting for specific materials, systems and methods

We require information from authors about some types of materials, experimental systems and methods used in many studies. Here, indicate whether each material, system or method listed is relevant to your study. If you are not sure if a list item applies to your research, read the appropriate section before selecting a response.

Materials & experimental systems

n/a	Involved in the study
☒	☐ Antibodies
☒	☐ Eukaryotic cell lines
☒	☐ Palaeontology and archaeology
☒	☐ Animals and other organisms
☒	☐ Clinical data
☒	☐ Dual use research of concern
☒	☐ Plants

Methods

n/a	Involved in the study
☒	☐ ChIP-seq
☒	☐ Flow cytometry
☒	☐ MRI-based neuroimaging

Plants

Seed stocks	Plants were not used in this study.
Novel plant genotypes	Plants were not used in this study.
Authentication	Plants were not used in this study.

This checklist template is licensed under a Creative Commons Attribution 4.0 International License, which permits use, sharing, adaptation, distribution and reproduction in any medium or format, as long as you give appropriate credit to the original author(s) and the source, provide a link to the Creative Commons license, and indicate if changes were made. The images or other third party material in this article are included in the article's Creative Commons license, unless indicated otherwise in a credit line to the material. If material is not included in the article's Creative Commons license and your intended use is not permitted by statutory regulation or exceeds the permitted use, you will need to obtain permission directly from the copyright holder. To view a copy of this license, visit http://creativecommons.org/licenses/by/4.0/
