# Supplementary material for: AI powered quantification of nuclear morphology in cancers enables prediction of genome instability and prognosis
Source: NPJ Precis Oncol. 2024 Jun 19;8:134. doi: 10.1038/s41698-024-00623-9 (PMC11187064; doi:10.1038/s41698-024-00623-9)
Supplement: Supplementary file 2 — Supplementary information [file 41698_2024_623_MOESM2_ESM.pdf]

Supplementary Figure 1

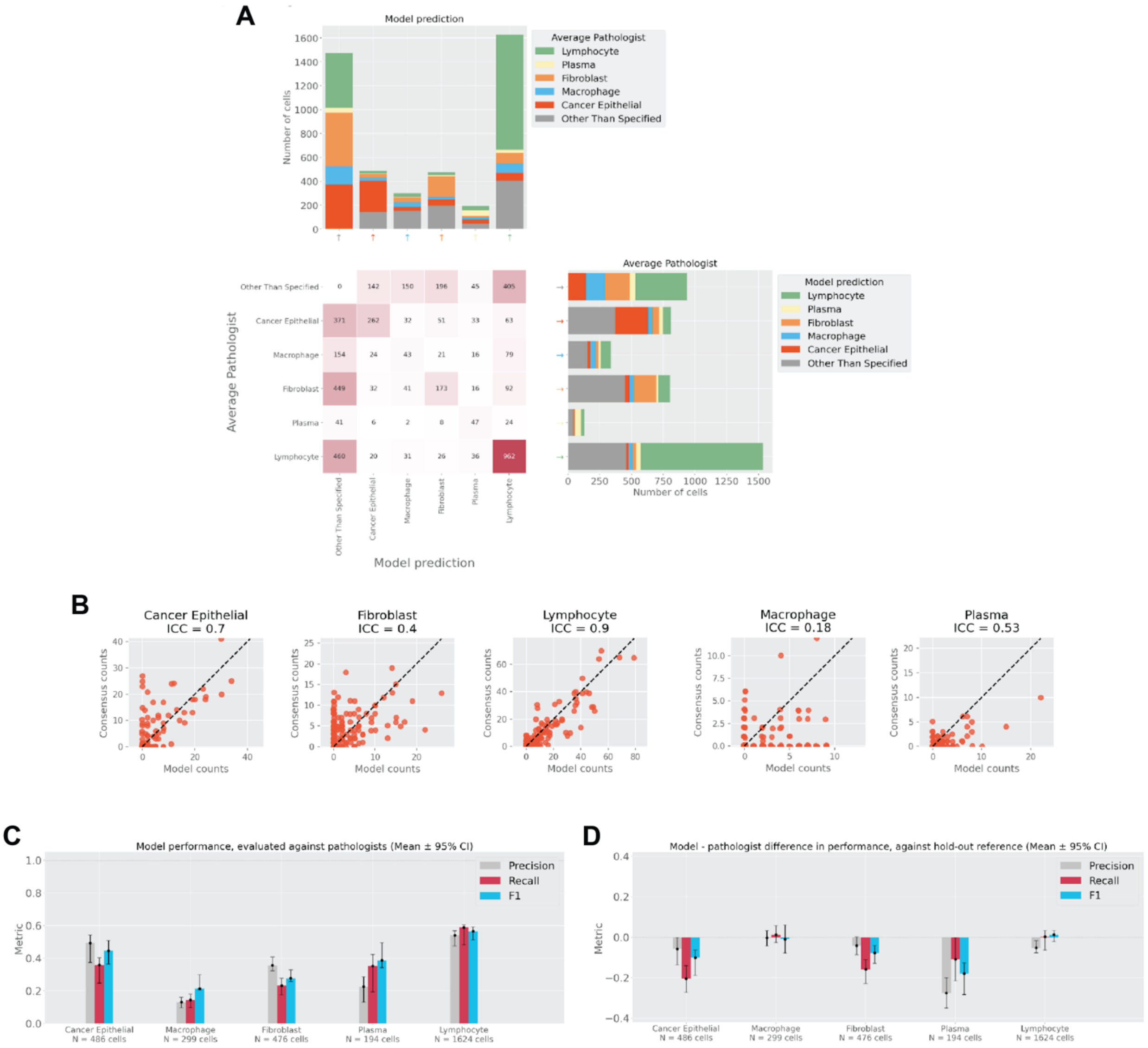

Supplementary Figure 1. Performance of cell classification model in breast cancer. A) Comparison of model-predicted cell types to average pathologist annotations. The bar graph at the top left depicts the breakdown of average pathologist annotations for each class of model prediction (precision). The bar graph at the bottom right shows the breakdown of model predictions for each class of average pathologist annotation (recall). “Other Than Specified” refers to predictions of classes other than those listed, or background class. **B)** Agreement between model-derived cell counts and pathologist consensus counts. **C**) Precision, Recall, and F1 scores of model predictions compared to pathologists’ annotations in nested pairwise fashion[32]. **D**) Difference in the nested pairwise metric comparing mean difference between model and individual pathologist performance. Positive values indicate that the model out-performed pathologists when evaluated against held-out pathologists, while negative values indicate the model under-performed pathologists. Confidence intervals were obtained by bootstrapping.

Supplementary Figure 2

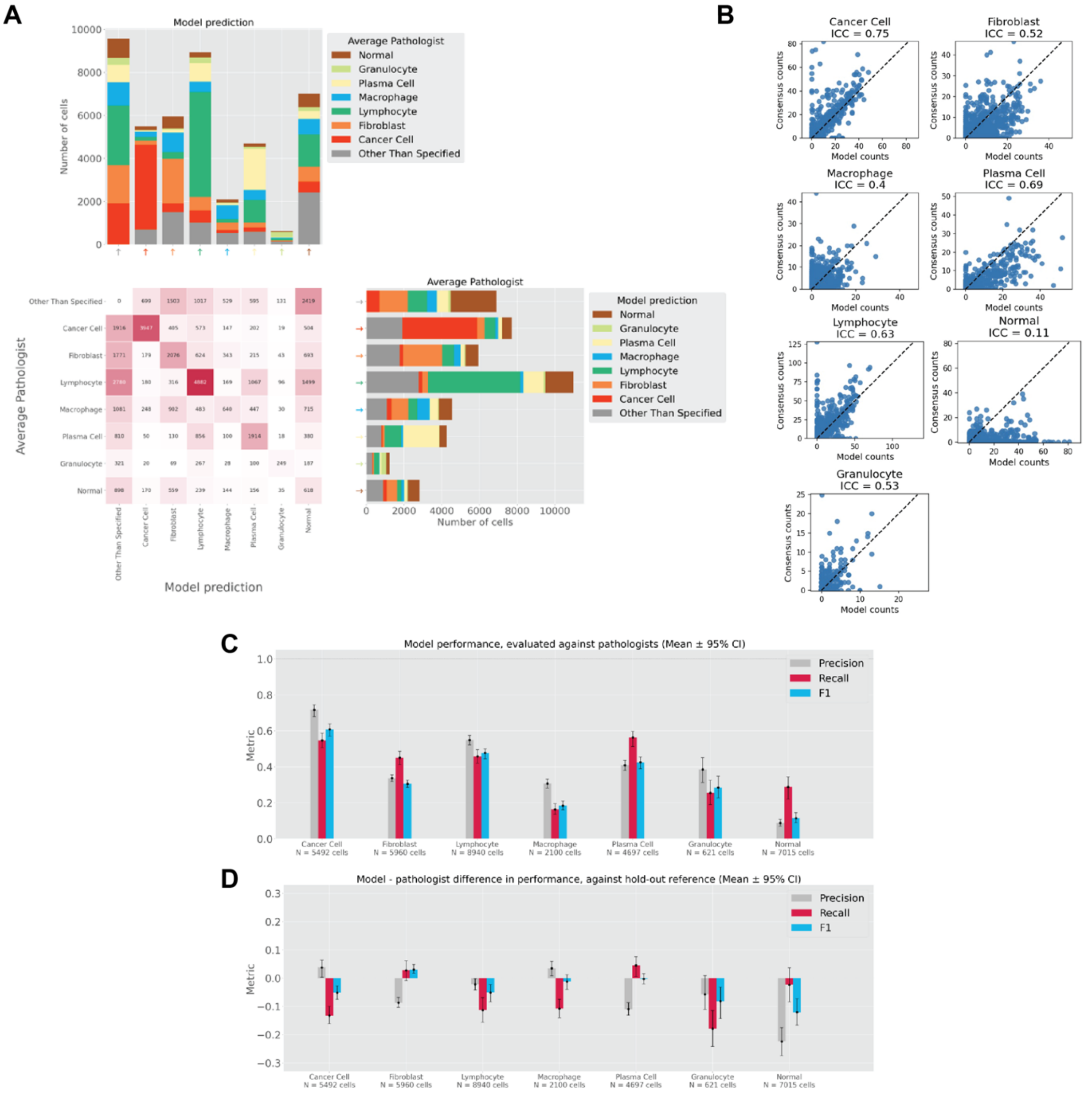

Supplementary Figure 2. Performance of cell classification model in NSCLC. A) Comparison of model-predicted cell types to average pathologist annotations. The bar graph at the top left depicts the breakdown of average pathologist annotations for each class of model prediction (precision). The bar graph at the bottom right shows the breakdown of model predictions for each class of average pathologist annotation (recall). “Other Than Specified” refers to predictions of classes other than those listed, or background class. **B)** Agreement between model-derived cell counts and pathologist consensus counts. **C**) Precision, Recall, and F1 scores of model predictions compared to pathologists’ annotations in nested pairwise fashion[32]. **D**) Difference in the nested pairwise metric comparing mean difference between model and individual pathologist performance. Positive values indicate that the model out-performed pathologists when evaluated against held-out pathologists, while negative values indicate the model under-performed pathologists. Confidence intervals were obtained by bootstrapping.

Supplementary Figure 3

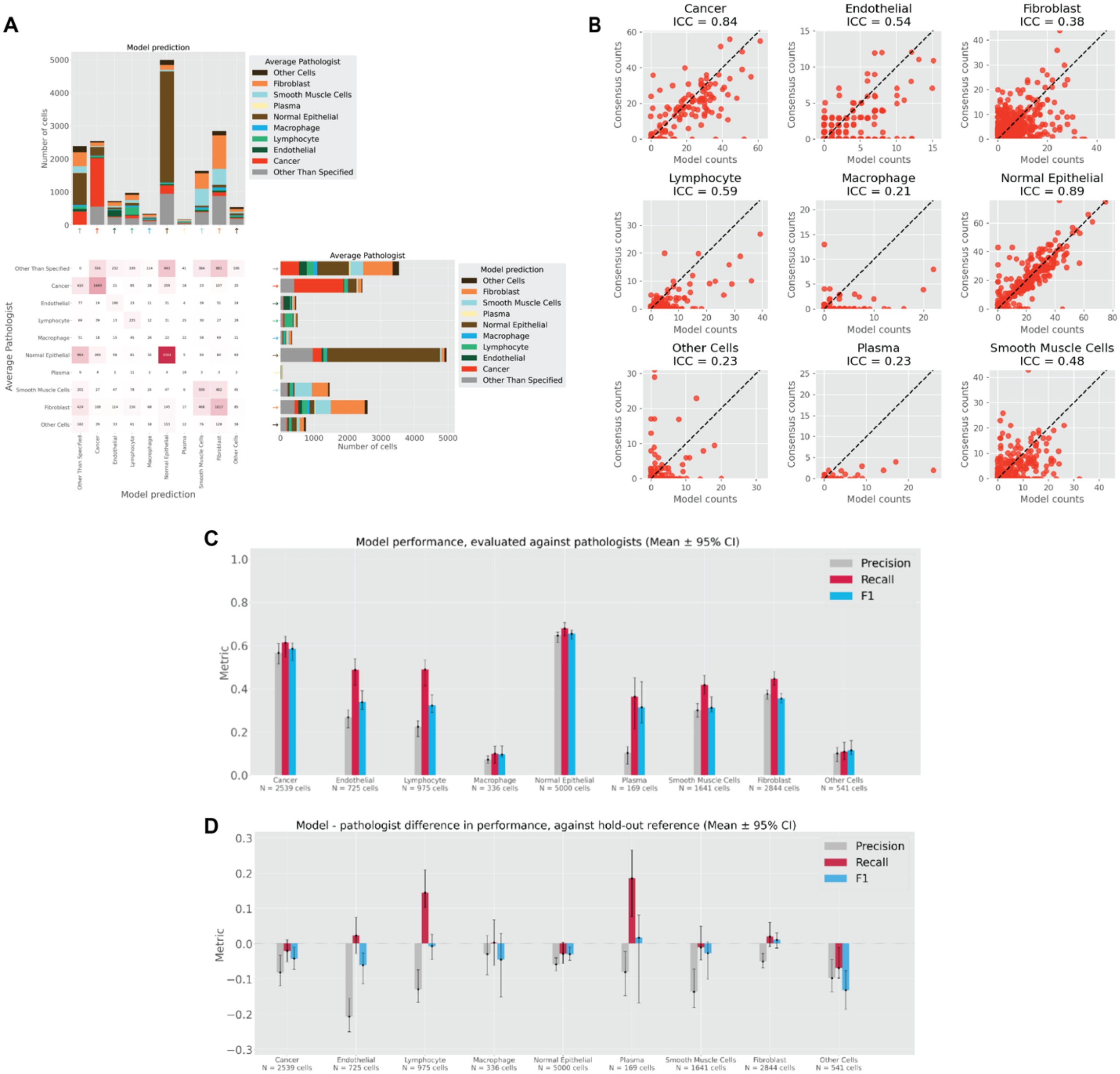

Supplementary Figure 3. Performance of cell classification model in prostate cancer. A) Comparison of model-predicted cell types to average pathologist annotations. The bar graph at the top left depicts the breakdown of average pathologist annotations for each class of model prediction (precision). The bar graph at the bottom right shows the breakdown of model predictions for each class of average pathologist annotation (recall). “Other Than Specified” refers to predictions of classes other than those listed, or background class. **B)** Agreement between model-derived cell counts and pathologist consensus counts. **C**) Precision, Recall, and F1 scores of model predictions compared to pathologists’ annotations in nested pairwise fashion[32]. **D**) Difference in the nested pairwise metric comparing mean difference between model and individual pathologist performance. Positive values indicate that the model out-performed pathologists when evaluated against held-out pathologists, while negative values indicate the model under-performed pathologists. Confidence intervals were obtained by bootstrapping.

Supplementary Figure 4

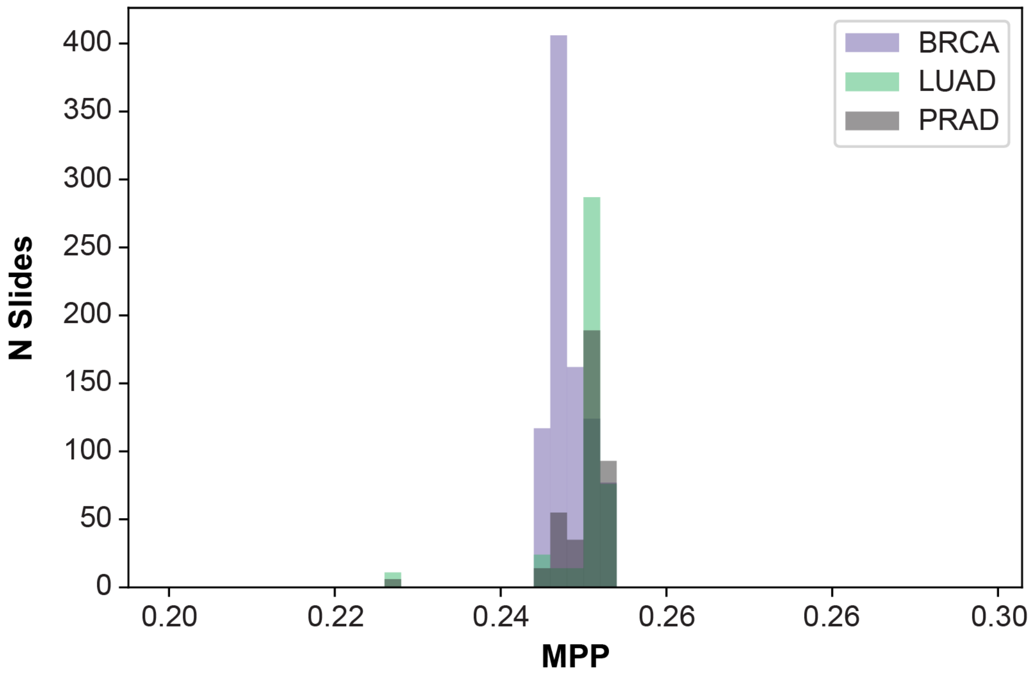

Supplementary Figure 4. Distribution of pixel sizes (in MPP) across the three TCGA datasets (BRCA, LUAD, PRAD) used in this study.

Supplementary Figure 5

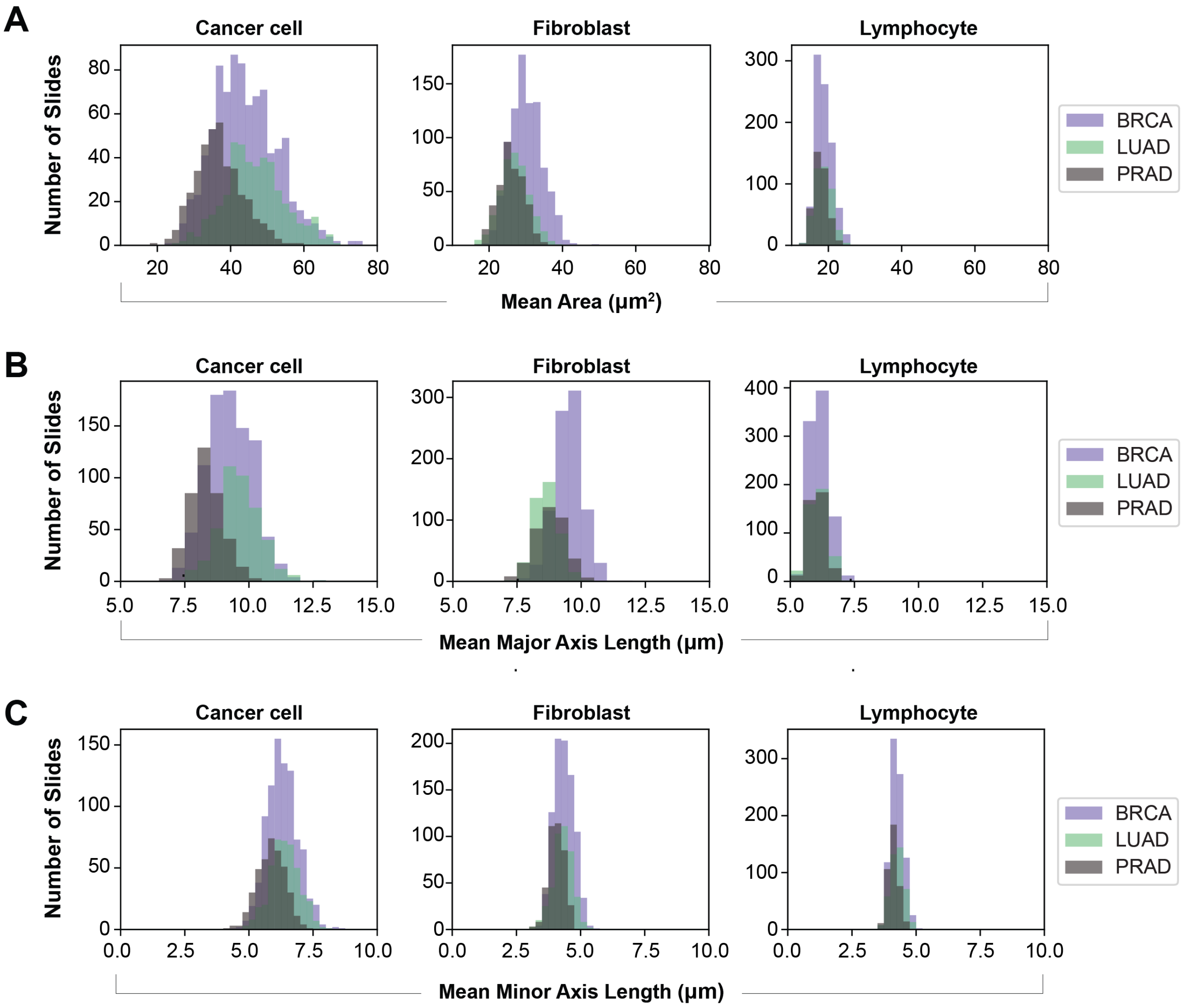

Supplementary Figure 5. Distributions of nuclear size features in BRCA, LUAD, and PRAD datasets. A) Distribution of area. B) Distribution of major axis length. C) Distribution of minor axis length.

Supplementary Figure 6

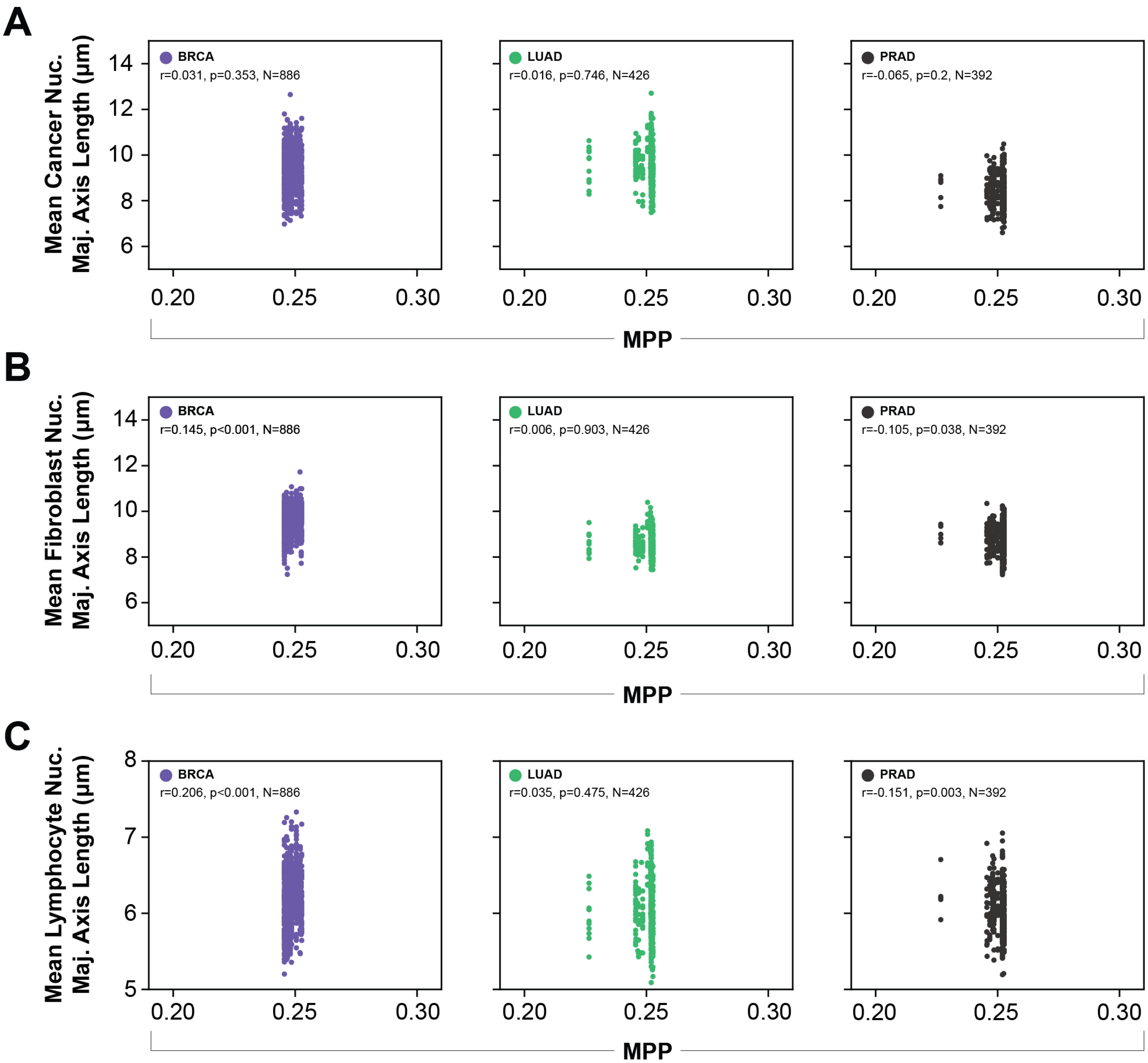

Supplementary Figure 6. Pearson correlation between nuclear size (using major axis length as a representative feature) and MPP for each cell type within BRCA, LUAD, and PRAD datasets. A) Correlation between major axis length and MPP in cancer epithelial cells. B) Correlation between major axis length and MPP in fibroblasts. C) Correlation between major axis length and MPP in lymphocytes.

Supplementary Table 1. Summary of agreement (ICC) between model-derived cell counts and pathologists' cell counts.

	BRCA (n=177 frames)			LUAD (n=834 frames)			PRAD (n=409 frames)		
	**Number of Cells**	**Model vs. Consensus [95% CI]**	**ΔICC [95% CI]**	**Number of Cells**	**Model vs. Consensus [95% CI]**	**ΔICC [95% CI]**	**Number of Cells**	**Model vs. Consensus [95% CI]**	**ΔICC [95% CI]**
**Cancer Cells**	486	0.7 [0.615, 0.768]	-0.018 [-0.209, -0.011]	5492	0.745 [0.704, 0.780]	-0.0199 [-0.0634, 0.0322]	2539	0.839 [0.805, 0.867]	0.002 [-0.030, 0.068]
**Fibroblasts**	476	0.401 [0.266, 0.52]	-0.058 [-0.093, -0.027]	5960	0.517 [0.442, 0.581]	0.00818 [-0.0435, 0.0505]	2844	0.37 [0.282, 0.451]	0.038 [-0.024, 0.068]
**Lymphocytes**	1624	0.898 0.865, 0.923]	0.043 [-0.025, 0.075]	8940	0.631 [0.579, 0.676]	-0.120 [-0.188, -0.0137]	975	0.59 [0.432, 0.698]	0.022 [-0.026, 0.063]
**Macrophages**	299	0.18 [0.039, 0.315]	0.049 [0.04, 0.087]	2100	0.398 [0.336, 0.457]	0.00524 [-0.0570, 0.0833]	336	0.214 [0.117, 0.307]	-0.003 [-0.076, 0.076]
**Plasma Cells**	194	0.531 [0.403, 0.636]	-0.102 [-0.169, -0.033]	4697	0.688 [0.593, 0.758]	0.0441 [0.0190, 0.0783]	169	0.228 [0.135, 0.318]	0.076 [-0.006, 0.097]

Supplementary Table 2. Summary table of cell model performance characteristics.

	Model vs. Avg Pathologist [95% CI]	Δrecall [95% CI]	Model vs. Avg Pathologist [95% CI]	ΔPrecision [95% CI]	Model vs. Avg Pathologist [95% CI]	ΔF1 [95% CI]
**BRCA**						
**Cancer Cells (N = 486 cells)**	0.36 [0.404, 0.248]	-0.204 [-0.141, -0.271]	-0.057 [0, -0.137]	-0.057 [0, -0.137]	0.446 [0.508, 0.364]	-0.101 [-0.064, -0.189]
**Fibroblasts (N = 476 cells)**	0.233 [0.278, 0.176]	-0.159 [-0.112, -0.228]	-0.042 [0.002, -0.087]	-0.002 [0.032, -0.043]	0.278 [0.328, 0.257]	-0.077 [-0.041, -0.129]
**Lymphocytes (N = 1,624 cells)**	0.589 [0.603, 0.483]	0.003 [0.033, -0.064]	-0.052 [-0.016, -0.077]	-0.042 [0.002, -0.087]	0.566 [0.592, 0.513]	0.013 [0.033, -0.021]
**Macrophages (N = 299 cells)**	0.144 [0.18, 0.099]	0.013 [0.057, -0.026]	-0.002 [0.032, -0.043]	-0.275 [-0.2, -0.35]	0.213 [0.3, 0.205]	-0.009 [0.061, -0.078]
**Plasma Cells (N = 194 cells)**	0.354 [0.423, 0.194]	-0.108 [-0.001, -0.216]	-0.275 [-0.2, -0.35]	-0.052 [-0.016, -0.077]	0.387 [0.495, 0.342]	-0.179 [-0.127, -0.282]
**LUAD**						
	**Model vs. Avg Pathologist [95% CI]**	**Δrecall [95% CI]**	**Model vs. Avg Pathologist [95% CI]**	**ΔPrecision [95% CI]**	**Model vs. Avg Pathologist [95% CI]**	**ΔF1 [95% CI]**
**Cancer Cells (N = 5,492 cells)**	0.547 [0.506, 0.586]	-0.132 [-0.161, -0.0991]	0.717 [0.679, 0.745]	0.0377 [0.00369, 0.0640]	0.608 [0.570, 0.638]	-0.051 [-0.0743, -0.0273]
**Fibroblasts (N = 5,960 cells)**	0.451 [0.412, 0.486]	0.0284 [-0.00804, 0.0623]	0.337 [0.319, 0.356]	-0.0863 [-0.103, -0.0675]	0.306 [0.285, 0.326]	0.0308 [0.0171, 0.0485]
**Lymphocytes (N = 8,940 cells)**	0.485 [0.420, 0.495]	-0.113 [-0.156, -0.0691]	0.549 [0.521, 0.575]	-0.0218 [-0.0414, -0.00144]	0.477 [0.445, 0.500]	-0.0509 [-0.0836, -0.0226]
**Macrophages (N = 2,100 cells)**	0.164 [0.136, 0.195]	-0.108 [-0.140, -0.0748]	0.307 [0.281, 0.333]	0.0349 [0.00816, 0.0595]	0.185 [0.161, 0.210]	-0.0121 [-0.0393, 0.0125]
**Plasma Cells (N = 4,697 cells)**	0.563 [0.513, 0.597]	0.0449 [0.00601, 0.0754]	0.409 [0.380, 0.435]	-0.11 [-0.131, -0.0868]	0.425 [0.389, 0.454]	-0.00294 [-0.0208, 0.0159]
**PRAD**						
	**Model vs. Avg Pathologist [95% CI]**	**Δrecall [95% CI]**	**Model vs. Avg Pathologist [95% CI]**	**ΔPrecision [95% CI]**	**Model vs. Avg Pathologist [95% CI]**	**ΔF1 [95% CI]**
**Cancer Cells (N = 2,539 cells)**	0.615 [0.553, 0.641]	-0.02 [-0.050, 0.008]	0.567 [0.514, 0.609]	-0.082 [-0.122, -0.037]	0.588 [0.533, 0.611]	-0.042 [-0.075, -0.013]
**Fibroblasts (N = 2,844 cells)**	0.451 [0.422, 0.484]	0.02 [-0.004, 0.062]	0.380 [0.355, 0.399]	-0.04 [-0.067, -0.027]	0.359 [0.339, 0.382]	0.014 [-0.009, 0.033]
**Lymphocytes (N = 975 cells)**	0.491 [0.423, 0.534]	0.14 [0.106, 0.211]	0.226 [0.183, 0.251]	-0.128 [-0.165, -0.073]	0.325 [0.296, 0.373]	-0.006 [-0.045, 0.029]
**Macrophages (N = 336 cells)**	0.115 [0.069, 0.150]	0.01 [-0.048, 0.083]	0.079 [0.060, 0.096]	-0.025 [-0.08, 0.028]	0.099 [0.089, 0.137]	-0.042 [-0.142, 0.032]
**Plasma Cells (N = 169 cells)**	0.365 [0.217, 0.452]	0.18 [0.071, 0.259]	0.104 [0.051, 0.137]	-0.082 [-0.147, -0.025]	0.315 [0.239, 0.424]	0.016 [-0.174, 0.075]

Supplementary Table 3. nuHIFs extracted from the nuclear segmentation model.

nuHIF Name	Descriptor
MEAN[CELL TYPE X_NUCLEUS_AREA]_H & E	Slide-level mean of the area of each nucleus of cell type X, in μm^2.
MEAN[CELL TYPE X_NUCLEUS_CIRCULARITY]_H & E	Slide-level mean of the circularity of each nucleus of cell type X.
MEAN[CELL TYPE X_NUCLEUS_ECCENTRICITY]_H & E	Slide-level mean of the eccentricity of each nucleus of cell type X.
MEAN[CELL TYPE X_NUCLEUS_MAJOR_AXIS_LENGTH]_H & E	Slide-level mean of the major axis length of each nucleus of cell type X, in µm.
MEAN[CELL TYPE X_NUCLEUS_MEAN_GRAYSCALE_CHANNEL_GRAY]_H & E	Slide-level mean of the (mean grayscale intensity value of each nucleus) of cell type X.
MEAN[CELL TYPE X_NUCLEUS_MEAN_HSV_CHANNEL_SATURATION]_H & E	Slide-level mean of the (mean saturation value in HSV colorspace of each nucleus) of cell type X.
MEAN[CELL TYPE X_NUCLEUS_MEAN_LAB_CHANNEL_A]_H & E	Slide-level mean of the (mean A value in LAB colorspace of each nucleus) of cell type X.
MEAN[CELL TYPE X_NUCLEUS_MEAN_LAB_CHANNEL_B]_H & E	Slide-level mean of the (mean B value in LAB colorspace of each nucleus) of cell type X.
MEAN[CELL TYPE X_NUCLEUS_MINOR_AXIS_LENGTH]_H & E	Slide-level mean of the minor axis length of each nucleus of cell type X, in µm.
MEAN[CELL TYPE X_NUCLEUS_PERIMETER]_H & E	Slide-level mean of the perimeter length of each nucleus of cell type X, in µm.
MEAN[CELL TYPE X_NUCLEUS_SOLIDITY]_H & E	Slide-level mean of the solidity of each nucleus of cell type X.
MEAN[CELL TYPE X_NUCLEUS_STD_GRAYSCALE_CHANNEL_GRAY]_H & E	Slide-level mean of the (standard deviation of the grayscale intensity value of each nucleus) of cell type X.
MEAN[CELL TYPE X_NUCLEUS_STD_HSV_CHANNEL_SATURATION]_H & E	Slide-level mean of the (standard deviation of the saturation value in HSV colorspace of each nucleus) of cell type X.
MEAN[CELL TYPE X_NUCLEUS_STD_LAB_CHANNEL_A]_H & E	Slide-level mean of the (standard deviation of the A value in LAB colorspace of each nucleus) of cell type X.
MEAN[CELL TYPE X_NUCLEUS_STD_LAB_CHANNEL_B]_H & E	Slide-level mean of the (standard deviation of the B value in LAB colorspace of each nucleus) of cell type X.
STD[CELL TYPE X_NUCLEUS_AREA]_H & E	Slide-level standard deviation of the area of each nucleus of cell type X, in μm^2.
STD[CELL TYPE X_NUCLEUS_CIRCULARITY]_H & E	Slide-level standard deviation of the circularity of each nucleus of cell type X.
STD[CELL TYPE X_NUCLEUS_ECCENTRICITY]_H & E	Slide-level standard deviation of the eccentricity of each nucleus of cell type X.
STD[CELL TYPE X_NUCLEUS_MAJOR_AXIS_LENGTH]_H & E	Slide-level standard deviation of the major axis length of each nucleus of cell type X, in µm.
STD[CELL TYPE X_NUCLEUS_MEAN_GRAYSCALE_CHANNEL_GRAY]_H & E	Slide-level standard deviation of the (mean grayscale intensity value of each nucleus) of cell type X.
STD[CELL TYPE X_NUCLEUS_MEAN_HSV_CHANNEL_SATURATION]_H & E	Slide-level standard deviation of the (mean saturation value in HSV colorspace of each nucleus) of cell type X.
STD[CELL TYPE X_NUCLEUS_MEAN_LAB_CHANNEL_A]_H & E	Slide-level standard deviation of the (mean A value in LAB colorspace of each nucleus) of cell type X.
STD[CELL TYPE X_NUCLEUS_MEAN_LAB_CHANNEL_B]_H & E	Slide-level standard deviation of the (mean B value in LAB colorspace of each nucleus) of cell type X.
STD[CELL TYPE X_NUCLEUS_MINOR_AXIS_LENGTH]_H & E	Slide-level standard deviation of the minor axis length of each nucleus of cell type X, in µm.
STD[CELL TYPE X_NUCLEUS_PERIMETER]_H & E	Slide-level standard deviation of the perimeter length of each nucleus of cell type X, in µm.
STD[CELL TYPE X_NUCLEUS_SOLIDITY]_H & E	Slide-level standard deviation of the solidity of each nucleus of cell type X.
STD[CELL TYPE X_NUCLEUS_STD_GRAYSCALE_CHANNEL_GRAY]_H & E	Slide-level standard deviation of the (standard deviation of the grayscale intensity value of each nucleus) of cell type X.
STD[CELL TYPE X_NUCLEUS_STD_HSV_CHANNEL_SATURATION]_H & E	Slide-level standard deviation of the (standard deviation of the saturation value in HSV colorspace of each nucleus) of cell type X.
STD[CELL TYPE X_NUCLEUS_STD_LAB_CHANNEL_A]_H & E	Slide-level standard deviation of the (standard deviation of the A value in LAB colorspace of each nucleus) of cell type X.
STD[CELL TYPE X_NUCLEUS_STD_LAB_CHANNEL_B]_H & E	Slide-level standard deviation of the (standard deviation of the B value in LAB colorspace of each nucleus) of cell type X.

Supplementary Table 4. Top nuHIF Mean Importance for WGD Prediction

BRCA	Mean Importance Score
Standard deviation of cancer cell nucleus major axis length	0.0685
Mean of cancer cell nucleus major axis length	0.0655
Standard deviation of cancer cell nucleus minor axis length	0.0572
Standard deciation of cancer cell nucleus area	0.0521
Mean of cancer cell nucleus perimeter	0.0519
**LUAD**	**Mean Importance Score**
Mean cancer cell nucleus perimeter	0.0479
Standard deviation of cancer cell nucleus minor axis length	0.0441
Mean cancer cell nucleus major axis length	0.0425
Mean of the cancer cell nucleus saturation (in HSV colorspace) standard deviation	0.0367
Mean cancer cell nucleus eccentricity	0.0355
**PRAD**	**Mean Importance Score**
Standard deviation of cancer cell nucleus major axis length	0.0618
Mean of the cancer cell nucleus saturation (in HSV colorspace) mean value	0.0544
Mean of the cancer cell nucleus grayscale value mean value	0.0481
Slide-level standard deviation of the cancer cell nucleus grayscale value standard deviation	0.046
Mean cancer nucleus circularity	0.0436

Supplementary Table 5. Association between PFS and CAF nuHIFs.

	N	coef	coef upper 95%	coef lower 95%	HR	HR upper 95%	HR lower 95%	HR p	corrected HR p
MEAN[FIBROBLAST_NUCLEUS_AREA]_H & E	869	0.223	0.368	0.078	1.250	1.444	1.081	0.003	0.014
MEAN[FIBROBLAST_NUCLEUS_CIRCULARITY]_H & E	869	0.238	0.392	0.084	1.269	1.480	1.088	0.002	0.014
MEAN[FIBROBLAST_NUCLEUS_ECCENTRICITY]_H & E	869	-0.233	-0.080	-0.386	0.792	0.923	0.680	0.003	0.014
MEAN[FIBROBLAST_NUCLEUS_MAJOR_AXIS_LENGTH]_H & E	869	0.160	0.306	0.014	1.173	1.358	1.014	0.032	0.088
MEAN[FIBROBLAST_NUCLEUS_MEAN_GRAYSCALE_CHANNEL_GRAY]_H & E	869	0.065	0.236	-0.106	1.067	1.266	0.900	0.456	0.707
MEAN[FIBROBLAST_NUCLEUS_MEAN_HSV_CHANNEL_SATURATION]_H & E	869	0.094	0.271	-0.084	1.098	1.311	0.920	0.301	0.564
MEAN[FIBROBLAST_NUCLEUS_MEAN_LAB_CHANNEL_A]_H & E	869	0.226	0.374	0.078	1.254	1.454	1.081	0.003	0.014
MEAN[FIBROBLAST_NUCLEUS_MEAN_LAB_CHANNEL_B]_H & E	869	-0.087	0.093	-0.267	0.916	1.097	0.765	0.341	0.569
MEAN[FIBROBLAST_NUCLEUS_MINOR_AXIS_LENGTH]_H & E	869	0.259	0.414	0.103	1.295	1.513	1.108	0.001	0.014
MEAN[FIBROBLAST_NUCLEUS_PERIMETER]_H & E	869	0.192	0.336	0.047	1.211	1.400	1.048	0.009	0.029
MEAN[FIBROBLAST_NUCLEUS_SOLIDITY]_H & E	869	0.234	0.392	0.075	1.263	1.481	1.078	0.004	0.014
MEAN[FIBROBLAST_NUCLEUS_STD_GRAYSCALE_CHANNEL_GRAY]_H & E	869	-0.100	0.062	-0.263	0.904	1.064	0.769	0.225	0.450
MEAN[FIBROBLAST_NUCLEUS_STD_HSV_CHANNEL_SATURATION]_H & E	869	-0.064	0.110	-0.238	0.938	1.116	0.788	0.471	0.707
MEAN[FIBROBLAST_NUCLEUS_STD_LAB_CHANNEL_A]_H & E	869	-0.048	0.095	-0.191	0.953	1.100	0.826	0.511	0.730
MEAN[FIBROBLAST_NUCLEUS_STD_LAB_CHANNEL_B]_H & E	869	-0.004	0.143	-0.151	0.996	1.154	0.860	0.956	0.984
STD[FIBROBLAST_NUCLEUS_AREA]_H & E	869	0.221	0.370	0.073	1.248	1.447	1.076	0.003	0.014
STD[FIBROBLAST_NUCLEUS_CIRCULARITY]_H & E	869	-0.039	0.098	-0.176	0.962	1.103	0.839	0.579	0.788
STD[FIBROBLAST_NUCLEUS_ECCENTRICITY]_H & E	869	0.212	0.372	0.051	1.236	1.451	1.052	0.010	0.029
STD[FIBROBLAST_NUCLEUS_MAJOR_AXIS_LENGTH]_H & E	869	0.104	0.244	-0.035	1.110	1.277	0.965	0.143	0.307
STD[FIBROBLAST_NUCLEUS_MEAN_GRAYSCALE_CHANNEL_GRAY]_H & E	869	0.036	0.202	-0.131	1.037	1.224	0.878	0.672	0.809
STD[FIBROBLAST_NUCLEUS_MEAN_HSV_CHANNEL_SATURATION]_H & E	869	0.034	0.215	-0.148	1.034	1.240	0.863	0.716	0.809
STD[FIBROBLAST_NUCLEUS_MEAN_LAB_CHANNEL_A]_H & E	869	0.001	0.138	-0.136	1.001	1.148	0.873	0.984	0.984
STD[FIBROBLAST_NUCLEUS_MEAN_LAB_CHANNEL_B]_H & E	869	0.073	0.218	-0.072	1.076	1.243	0.930	0.324	0.569
STD[FIBROBLAST_NUCLEUS_MINOR_AXIS_LENGTH]_H & E	869	0.247	0.404	0.089	1.280	1.498	1.093	0.002	0.014
STD[FIBROBLAST_NUCLEUS_PERIMETER]_H & E	869	0.140	0.280	0.000	1.150	1.323	1.000	0.050	0.124
STD[FIBROBLAST_NUCLEUS_SOLIDITY]_H & E	869	-0.039	0.109	-0.188	0.961	1.116	0.828	0.604	0.788
STD[FIBROBLAST_NUCLEUS_STD_GRAYSCALE_CHANNEL_GRAY]_H & E	869	0.014	0.162	-0.135	1.014	1.176	0.874	0.858	0.919
STD[FIBROBLAST_NUCLEUS_STD_HSV_CHANNEL_SATURATION]_H & E	869	0.037	0.216	-0.142	1.038	1.242	0.867	0.687	0.809
STD[FIBROBLAST_NUCLEUS_STD_LAB_CHANNEL_A]_H & E	869	0.125	0.268	-0.018	1.133	1.308	0.982	0.087	0.202
STD[FIBROBLAST_NUCLEUS_STD_LAB_CHANNEL_B]_H & E	869	-0.028	0.132	-0.189	0.972	1.141	0.828	0.728	0.809

Supplementary Table 6. Association between OS and CAF nuHIFs.

	N	coef	coef upper 95%	coef lower 95%	HR	HR upper 95%	HR lower 95%	HR p	corrected HR p
MEAN[FIBROBLAST_NUCLEUS_AREA]_H & E	869	0.229	0.389	0.069	1.257	1.475	1.071	0.005	0.038
MEAN[FIBROBLAST_NUCLEUS_CIRCULARITY]_H & E	869	0.219	0.398	0.040	1.245	1.489	1.041	0.016	0.062
MEAN[FIBROBLAST_NUCLEUS_ECCENTRICITY]_H & E	869	-0.201	-0.024	-0.379	0.818	0.977	0.685	0.026	0.079
MEAN[FIBROBLAST_NUCLEUS_MAJOR_AXIS_LENGTH]_H & E	869	0.203	0.365	0.042	1.225	1.440	1.043	0.014	0.062
MEAN[FIBROBLAST_NUCLEUS_MEAN_GRAYSCALE_CHANNEL_GRAY]_H & E	869	-0.025	0.173	-0.223	0.975	1.189	0.800	0.805	0.862
MEAN[FIBROBLAST_NUCLEUS_MEAN_HSV_CHANNEL_SATURATION]_H & E	869	0.194	0.397	-0.009	1.214	1.488	0.991	0.061	0.131
MEAN[FIBROBLAST_NUCLEUS_MEAN_LAB_CHANNEL_A]_H & E	869	0.271	0.439	0.103	1.311	1.551	1.109	0.002	0.037
MEAN[FIBROBLAST_NUCLEUS_MEAN_LAB_CHANNEL_B]_H & E	869	-0.207	0.006	-0.420	0.813	1.006	0.657	0.057	0.131
MEAN[FIBROBLAST_NUCLEUS_MINOR_AXIS_LENGTH]_H & E	869	0.258	0.432	0.084	1.294	1.541	1.087	0.004	0.037
MEAN[FIBROBLAST_NUCLEUS_PERIMETER]_H & E	869	0.214	0.372	0.055	1.238	1.451	1.056	0.008	0.050
MEAN[FIBROBLAST_NUCLEUS_SOLIDITY]_H & E	869	0.284	0.470	0.097	1.328	1.599	1.102	0.003	0.037
MEAN[FIBROBLAST_NUCLEUS_STD_GRAYSCALE_CHANNEL_GRAY]_H & E	869	-0.086	0.094	-0.267	0.917	1.098	0.766	0.347	0.520
MEAN[FIBROBLAST_NUCLEUS_STD_HSV_CHANNEL_SATURATION]_H & E	869	-0.007	0.193	-0.207	0.993	1.213	0.813	0.944	0.944
MEAN[FIBROBLAST_NUCLEUS_STD_LAB_CHANNEL_A]_H & E	869	0.036	0.197	-0.125	1.037	1.218	0.883	0.660	0.791
MEAN[FIBROBLAST_NUCLEUS_STD_LAB_CHANNEL_B]_H & E	869	0.110	0.280	-0.060	1.116	1.323	0.941	0.206	0.343
STD[FIBROBLAST_NUCLEUS_AREA]_H & E	869	0.205	0.370	0.039	1.227	1.448	1.040	0.015	0.062
STD[FIBROBLAST_NUCLEUS_CIRCULARITY]_H & E	869	-0.045	0.111	-0.201	0.956	1.117	0.818	0.570	0.712
STD[FIBROBLAST_NUCLEUS_ECCENTRICITY]_H & E	869	0.177	0.362	-0.008	1.194	1.436	0.992	0.061	0.131
STD[FIBROBLAST_NUCLEUS_MAJOR_AXIS_LENGTH]_H & E	869	0.132	0.289	-0.024	1.142	1.335	0.976	0.097	0.182
STD[FIBROBLAST_NUCLEUS_MEAN_GRAYSCALE_CHANNEL_GRAY]_H & E	869	-0.039	0.154	-0.233	0.962	1.167	0.792	0.692	0.791
STD[FIBROBLAST_NUCLEUS_MEAN_HSV_CHANNEL_SATURATION]_H & E	869	-0.040	0.173	-0.254	0.961	1.189	0.776	0.712	0.791
STD[FIBROBLAST_NUCLEUS_MEAN_LAB_CHANNEL_A]_H & E	869	-0.055	0.104	-0.214	0.947	1.109	0.808	0.497	0.678
STD[FIBROBLAST_NUCLEUS_MEAN_LAB_CHANNEL_B]_H & E	869	0.015	0.187	-0.156	1.016	1.205	0.855	0.860	0.890
STD[FIBROBLAST_NUCLEUS_MINOR_AXIS_LENGTH]_H & E	869	0.196	0.374	0.017	1.216	1.453	1.018	0.031	0.086
STD[FIBROBLAST_NUCLEUS_PERIMETER]_H & E	869	0.131	0.284	-0.022	1.139	1.328	0.978	0.095	0.182
STD[FIBROBLAST_NUCLEUS_SOLIDITY]_H & E	869	-0.110	0.057	-0.276	0.896	1.059	0.759	0.197	0.343
STD[FIBROBLAST_NUCLEUS_STD_GRAYSCALE_CHANNEL_GRAY]_H & E	869	0.052	0.224	-0.121	1.053	1.251	0.886	0.558	0.712
STD[FIBROBLAST_NUCLEUS_STD_HSV_CHANNEL_SATURATION]_H & E	869	0.083	0.289	-0.122	1.087	1.334	0.885	0.427	0.610
STD[FIBROBLAST_NUCLEUS_STD_LAB_CHANNEL_A]_H & E	869	0.191	0.350	0.031	1.210	1.419	1.032	0.019	0.063
STD[FIBROBLAST_NUCLEUS_STD_LAB_CHANNEL_B]_H & E	869	0.115	0.301	-0.071	1.122	1.352	0.931	0.226	0.357

Supplementary Table 7. Association between CAF nucleus area and gene expression levels

	Spearman rho	Spearman p	Spearman adj p
ARSI	0.255	2.91E-14	9.41E-10
PTPRN	0.253	3.95E-14	9.41E-10
IGFL3	0.245	3.00E-13	3.92E-09
TNFRSF12A	0.244	3.30E-13	3.92E-09
PPAPDC1A	0.241	6.80E-13	6.48E-09
RCN3	0.239	1.12E-12	8.91E-09
MMP14	0.238	1.43E-12	9.71E-09
MIR4435-1HG	0.235	2.49E-12	1.48E-08
BMP1	0.235	2.79E-12	1.48E-08
GJB2	0.234	3.12E-12	1.49E-08
RP11-145A3	0.229	9.85E-12	3.91E-08
TPM2	0.228	1.32E-11	4.83E-08
IL11	0.225	2.21E-11	7.51E-08
NXN	0.224	2.62E-11	7.81E-08
NNMT	0.222	3.93E-11	1.06E-07
ADAMTS14	0.222	4.00E-11	1.06E-07
SERPINH1	0.221	5.00E-11	1.25E-07
LINC01561	0.218	9.00E-11	2.10E-07
LOXL2	0.218	9.24E-11	2.10E-07
TAX1BP3	0.218	1.03E-10	2.14E-07
SUGCT	0.217	1.05E-10	2.14E-07
CERCAM	0.217	1.08E-10	2.14E-07
COL5A3	0.217	1.12E-10	2.14E-07
MRC2	0.216	1.29E-10	2.28E-07
UBTD1	0.214	2.18E-10	3.61E-07
P3H1	0.214	2.20E-10	3.61E-07
HHIPL1	0.213	2.64E-10	4.19E-07
MFAP2	0.212	3.14E-10	4.83E-07
COL7A1	0.212	3.25E-10	4.84E-07
PPIC	0.210	4.26E-10	6.15E-07
SCARF2	0.210	4.51E-10	6.32E-07
ANGPTL4	0.210	5.02E-10	6.83E-07
LINC00632	0.209	5.71E-10	7.56E-07
PTK7	0.208	6.10E-10	7.80E-07
CLEC11A	0.208	6.39E-10	7.80E-07
COL1A1	0.208	7.36E-10	8.76E-07
TLL2	0.206	9.12E-10	1.02E-06
PDLIM7	0.206	9.37E-10	1.02E-06
PLOD1	0.206	9.77E-10	1.02E-06
SRPX2	0.206	1.00E-09	1.02E-06
COL5A1	0.205	1.09E-09	1.06E-06
CHST6	0.205	1.13E-09	1.08E-06
FNDC4	0.205	1.27E-09	1.19E-06
UCN2	0.204	1.57E-09	1.39E-06
COL6A1	0.204	1.57E-09	1.39E-06
APCDD1L	0.203	1.73E-09	1.47E-06
NPTX2	0.203	1.73E-09	1.47E-06
COL6A2	0.202	2.05E-09	1.72E-06
TMEM92	0.201	2.51E-09	2.06E-06

RNF144A-AS1	0.199	3.83E-09	2.99E-06
RP11-400N13	0.198	4.46E-09	3.37E-06
C5orf46	0.197	4.99E-09	3.62E-06
HOMER3	0.197	5.02E-09	3.62E-06
EGLN3	0.197	5.30E-09	3.77E-06
P4HA3	0.197	5.42E-09	3.80E-06
SORCS2	0.197	5.54E-09	3.83E-06
CD276	0.196	5.94E-09	3.92E-06
RP11-417E7	0.196	6.11E-09	3.93E-06
COL16A1	0.194	8.26E-09	4.98E-06
CHPF	0.194	8.46E-09	5.04E-06
ADAMTS7	0.193	1.05E-08	6.02E-06
FAM127A	0.193	1.12E-08	6.21E-06
WNT5A	0.192	1.27E-08	6.92E-06
LGALS1	0.192	1.30E-08	6.94E-06
COL1A2	0.192	1.32E-08	6.98E-06
CKAP4	0.191	1.64E-08	8.21E-06
CILP2	0.190	1.67E-08	8.29E-06
PCSK9	0.190	1.76E-08	8.63E-06
SDC1	0.189	2.09E-08	9.96E-06
CAMK2A	0.189	2.26E-08	1.06E-05
FHL3	0.189	2.29E-08	1.07E-05
CTHRC1	0.188	2.44E-08	1.10E-05
SPHK1	0.188	2.62E-08	1.14E-05
EDN2	0.188	2.68E-08	1.15E-05
CTD-2171N6	0.187	3.03E-08	1.28E-05
RPSAP52	0.187	3.13E-08	1.30E-05
NKAIN4	0.187	3.21E-08	1.30E-05
P4HA2	0.186	3.60E-08	1.42E-05
GPX8	0.186	3.71E-08	1.45E-05
THBS2	0.185	4.07E-08	1.56E-05
TMEM158	0.185	4.43E-08	1.63E-05
SPON2	0.184	4.83E-08	1.72E-05
FAM20C	0.183	6.02E-08	2.08E-05
PODNL1	0.183	6.11E-08	2.09E-05
GDNF	0.183	6.41E-08	2.16E-05
MMP13	0.182	6.75E-08	2.25E-05
S100A10	0.182	7.69E-08	2.53E-05
WNT5A-AS1	0.182	7.69E-08	2.53E-05
HTRA1	0.181	8.06E-08	2.61E-05
MYL3	0.181	8.67E-08	2.74E-05
HSD11B1L	0.181	8.95E-08	2.79E-05
PCOLCE	0.181	9.20E-08	2.84E-05
AF015262	0.180	9.23E-08	2.84E-05
PITX3	0.179	1.13E-07	3.28E-05
PRRX2	0.179	1.16E-07	3.32E-05
RP11-863P13	0.179	1.17E-07	3.34E-05
RP1-79C4	0.179	1.19E-07	3.37E-05
FST	0.179	1.23E-07	3.44E-05
KIFC3	0.179	1.26E-07	3.48E-05
ADM	0.178	1.34E-07	3.61E-05
THY1	0.178	1.37E-07	3.66E-05

DACT3	0.178	1.50E-07	3.91E-05
FKBP9P1	0.177	1.52E-07	3.91E-05
ANXA2	0.177	1.62E-07	4.04E-05
HMGA2	0.177	1.68E-07	4.16E-05
GLIS1	0.177	1.77E-07	4.33E-05
SMTN	0.176	1.92E-07	4.60E-05
FAM189A1	0.176	1.93E-07	4.60E-05
CD99	0.176	1.96E-07	4.60E-05
CA9	0.175	2.11E-07	4.81E-05
LINC00511	0.175	2.23E-07	4.94E-05
TMEM240	0.175	2.23E-07	4.94E-05
SLC10A3	0.175	2.23E-07	4.94E-05
HOXA11	0.175	2.28E-07	4.99E-05
COPZ2	0.175	2.32E-07	5.02E-05
VSNL1	0.175	2.44E-07	5.15E-05
RPLP0P2	0.174	2.53E-07	5.29E-05
COL18A1	0.174	2.57E-07	5.34E-05
RP5-907D15	0.174	2.66E-07	5.49E-05
GRP	0.174	2.83E-07	5.77E-05
CCNJL	0.173	2.99E-07	5.94E-05
FSCN1	0.173	3.02E-07	5.97E-05
NKX3-2	0.173	3.10E-07	6.05E-05
DDIT4	0.173	3.14E-07	6.11E-05
MYL9	0.173	3.16E-07	6.12E-05
SMCO2	0.173	3.34E-07	6.34E-05
ZFHX4-AS1	0.172	3.41E-07	6.38E-05
FLNC	0.172	3.45E-07	6.40E-05
FBLIM1	0.172	3.51E-07	6.49E-05
APCDD1L-AS1	0.172	3.53E-07	6.49E-05
EMILIN1	0.172	3.69E-07	6.71E-05
PPAPDC3	0.172	3.75E-07	6.73E-05
KCNMB4	0.172	3.78E-07	6.73E-05
ACTN1-AS1	0.172	3.79E-07	6.73E-05
SPSB1	0.172	3.84E-07	6.77E-05
MMP2	0.171	4.02E-07	7.03E-05
PAX1	0.171	4.04E-07	7.05E-05
LMNA	0.171	4.13E-07	7.13E-05
PLAUR	0.171	4.40E-07	7.50E-05
MDFI	0.171	4.42E-07	7.52E-05
EFEMP2	0.171	4.58E-07	7.71E-05
ACTN1	0.171	4.62E-07	7.71E-05
PPP1R13L	0.170	4.63E-07	7.71E-05
MPZL2	0.170	4.79E-07	7.91E-05
RP3-428L16	0.170	4.82E-07	7.94E-05
COL13A1	0.170	4.83E-07	7.94E-05
GPC1	0.170	5.28E-07	8.38E-05
HOXA11-AS	0.170	5.40E-07	8.55E-05
ITGA5	0.169	5.46E-07	8.62E-05
FLJ16779	0.169	5.89E-07	9.19E-05
ZNF469	0.169	5.94E-07	9.24E-05
IGF2-AS	0.169	6.05E-07	9.35E-05
COL5A2	0.169	6.22E-07	9.49E-05

RARRES2	0.169	6.29E-07	9.58E-05
S100A11	0.168	6.88E-07	0.000102413
PTH1R	0.168	7.01E-07	0.000104047
BGN	0.168	7.23E-07	0.00010654
LOX	0.167	7.39E-07	0.000107616
WLS	0.167	7.50E-07	0.000108534
S100A2	0.167	7.73E-07	0.000110919
PDGFRL	0.167	7.86E-07	0.00011149
CDC42EP5	0.167	7.96E-07	0.00011195
MIR31HG	0.167	7.97E-07	0.00011195
RP13-463N16	0.167	8.14E-07	0.0001134
MAP7D1	0.167	8.22E-07	0.00011383
COL3A1	0.167	8.31E-07	0.000114353
FN1	0.167	8.36E-07	0.000114479
FGF16	0.167	8.46E-07	0.000115438
SDCBP2	0.166	8.74E-07	0.000118941
AC027612	0.166	9.07E-07	0.000122043
C1QTNF3	0.166	9.28E-07	0.000124163
RFX8	0.166	9.43E-07	0.000125418
PPP1R3G	0.166	9.51E-07	0.0001261
LINC00152	0.166	9.57E-07	0.000126568
ISLR	0.166	9.67E-07	0.000127171
PXDN	0.166	9.79E-07	0.000128077
BEAN1	0.166	9.98E-07	0.000130213
ARSE	0.165	1.04E-06	0.000133968
AEBP1	0.165	1.05E-06	0.000135049
LINC01426	0.165	1.05E-06	0.000135049
VLDLR	0.165	1.06E-06	0.00013582
CHSY3	0.165	1.08E-06	0.000136906
AP000695	0.165	1.08E-06	0.000136906
TMEM92-AS1	0.165	1.10E-06	0.000137757
ZYX	0.165	1.12E-06	0.000139895
SPARC	0.164	1.19E-06	0.000147201
KRT75	0.164	1.21E-06	0.000147662
LYPD1	0.164	1.21E-06	0.000147662
SYDE1	0.164	1.22E-06	0.000147662
CNIH3	0.164	1.22E-06	0.000148295
GXYLT2	0.164	1.24E-06	0.000149467
VWA1	0.164	1.27E-06	0.00015166
ACTB	0.164	1.27E-06	0.00015166
RRBP1	0.164	1.27E-06	0.00015166
TNC	0.164	1.28E-06	0.00015166
PLIN3	0.164	1.28E-06	0.00015166
SEPW1	0.164	1.28E-06	0.00015166
DCLK2	0.164	1.30E-06	0.00015343
ITGA11	0.163	1.36E-06	0.000158649
SYNDIG1	0.163	1.36E-06	0.000159305
PLS3	0.163	1.42E-06	0.000164099
P3H3	0.163	1.43E-06	0.000164509
LINC01503	0.163	1.44E-06	0.000165111
TP53I3	0.163	1.51E-06	0.000171542
DRD4	0.163	1.52E-06	0.000171626

IGLON5	0.163	1.54E-06	0.000173139
SPON1	0.162	1.60E-06	0.000178843
EFNB1	0.162	1.61E-06	0.000178843
TMEM45A	0.162	1.65E-06	0.00018149
RP11-13P5	0.162	1.75E-06	0.000190245
PMEPA1	0.162	1.77E-06	0.000190667
GABRB2	0.161	1.87E-06	0.000196436
CORIN	0.161	1.94E-06	0.000202416
POSTN	0.161	1.97E-06	0.000204338
TGFBI	0.161	1.98E-06	0.000204492
PTGFRN	0.161	2.02E-06	0.000208228
RHBDL2	0.161	2.03E-06	0.000209483
PRKCDBP	0.161	2.04E-06	0.000210087
OXT	0.161	2.08E-06	0.000212846
VKORC1	0.161	2.10E-06	0.000213933
KLKP1	0.160	2.12E-06	0.000214574
CSMD2	0.160	2.14E-06	0.000215215
AC012487	0.160	2.16E-06	0.000215905
IFNWP19	0.160	2.22E-06	0.00022029
LINC01057	0.160	2.22E-06	0.00022029
IGFL2	0.160	2.29E-06	0.000225059
RP11-62F24	0.160	2.42E-06	0.000235519
GPX7	0.159	2.47E-06	0.00024002
TGFB1I1	0.159	2.66E-06	0.000255413
MXRA8	0.159	2.66E-06	0.000255413
COL11A1	0.159	2.68E-06	0.00025581
FAT3	0.159	2.80E-06	0.000264179
MGST3	0.158	2.90E-06	0.000271511
RPL39L	0.158	2.94E-06	0.00027314
NREP	0.158	3.14E-06	0.000286673
MMP11	0.158	3.23E-06	0.000292925
GALNT2	0.157	3.41E-06	0.000304618
FOXQ1	0.157	3.43E-06	0.000305096
RP1-198K11	0.157	3.43E-06	0.000305096
NKD2	0.157	3.45E-06	0.000305642
RP11-91J19	0.157	3.79E-06	0.000327472
TM6SF2	0.156	3.87E-06	0.0003326
PTRF	0.156	3.95E-06	0.000336125
FAM101A	0.156	4.02E-06	0.000340589
CCIN	0.156	4.03E-06	0.000340589
SCT	0.156	4.08E-06	0.000343885
GAS1	0.156	4.22E-06	0.000351168
RP11-22N19	0.156	4.23E-06	0.000351168
PQLC2L	0.156	4.23E-06	0.000351168
EVA1A	0.156	4.29E-06	0.000355076
DPYSL3	0.155	4.43E-06	0.00036244
SMYD3	0.155	4.44E-06	0.00036244
CLDN14	0.155	4.71E-06	0.000378954
KRT16	0.155	4.83E-06	0.000385706
LGALS7B	0.155	4.85E-06	0.00038709
KIF26B	0.155	4.94E-06	0.000392429
KLK4	0.154	5.09E-06	0.000401458

TGFB2	0.154	5.12E-06	0.000401544
CSDC2	0.154	5.19E-06	0.000404812
CD248	0.154	5.20E-06	0.000404982
FKBP10	0.154	5.46E-06	0.000420115
LINC01429	0.154	5.69E-06	0.000433295
NXPH4	0.153	5.84E-06	0.000443102
CSPG4	0.153	5.84E-06	0.000443102
RP11-815M8	0.153	5.93E-06	0.000447681
TNFRSF1A	0.153	5.94E-06	0.000447801
AGPAT4	0.153	5.99E-06	0.000449538
CALB2	0.153	6.12E-06	0.000457837
ADAMTS16	0.153	6.45E-06	0.000479711
FIBIN	0.153	6.48E-06	0.000479711
CYS1	0.153	6.69E-06	0.000492312
GLIS2	0.152	6.91E-06	0.000504438
C8orf58	0.152	7.23E-06	0.000523655
CLCA2	0.152	7.33E-06	0.0005292
ROS1	0.152	7.38E-06	0.000531318
RP11-380J14	0.152	7.40E-06	0.000531318
MFRP	0.152	7.64E-06	0.000544321
CEBPD	0.152	7.70E-06	0.00054745
GAP43	0.151	7.84E-06	0.000554414
JMJD1C-AS1	0.151	8.41E-06	0.000590974
PLAU	0.151	8.46E-06	0.000593255
NMB	0.151	8.49E-06	0.000594689
CLDN1	0.151	8.50E-06	0.000594689
CMTM3	0.151	8.54E-06	0.00059639
SMIM3	0.151	8.62E-06	0.000600459
AP2M1	0.151	8.78E-06	0.000609354
TUBB2A	0.151	8.85E-06	0.00061367
KRT7	0.150	9.03E-06	0.000623414
CLTB	0.150	9.29E-06	0.000636409
EFNA4	0.150	9.37E-06	0.000640104
ABCG4	0.150	9.56E-06	0.000652584
RP4-665J23	0.150	9.66E-06	0.000658003
FKBP9	0.150	9.73E-06	0.000660991
KANK4	0.150	9.76E-06	0.000662049
RP11-100E13	0.149	1.03E-05	0.000695207
MMP8	0.149	1.04E-05	0.00069998
H19	0.149	1.06E-05	0.000707565
PITX2	0.149	1.08E-05	0.000714887
TAGLN	0.149	1.10E-05	0.000722336
SH3GL1	0.149	1.10E-05	0.000726459
GLIS3	0.149	1.14E-05	0.000744884
ELFN1	0.149	1.14E-05	0.000744884
FGF5	0.149	1.15E-05	0.000749113
COL8A1	0.148	1.19E-05	0.000769017
FKBP14	0.148	1.19E-05	0.000769017
CHPF2	0.148	1.20E-05	0.000769254
CNTN1	0.148	1.21E-05	0.000775595
DEPDC7	0.148	1.21E-05	0.000776344
RAB7B	0.148	1.23E-05	0.000779484

NUDT11	0.148	1.26E-05	0.000795971
MAGEL2	0.148	1.28E-05	0.000808989
CREG2	0.148	1.31E-05	0.000825548
MYL6	0.148	1.31E-05	0.000825548
EMP1	0.148	1.31E-05	0.000825548
COL4A5	0.148	1.31E-05	0.000825653
PMP22	0.147	1.35E-05	0.000843561
SPP1	0.147	1.37E-05	0.000852709
RP11-531H8	0.147	1.38E-05	0.0008547
TNFAIP6	0.147	1.40E-05	0.000868516
C1QTNF6	0.147	1.40E-05	0.000868516
CST6	0.147	1.41E-05	0.000868516
FBLN1	0.147	1.42E-05	0.000871396
FAM109A	0.147	1.43E-05	0.000875046
EN1	0.147	1.44E-05	0.000876825
OLFML2B	0.147	1.46E-05	0.000887224
ASPN	0.147	1.48E-05	0.000895688
TIMP2	0.147	1.49E-05	0.000898139
KDELC1	0.147	1.51E-05	0.000904271
SPOCD1	0.146	1.56E-05	0.000925196
AJ011932	0.146	1.56E-05	0.000925196
DMBT1	0.146	1.57E-05	0.000925196
TSPAN6	0.146	1.61E-05	0.000952028
CYP27C1	0.146	1.62E-05	0.000953635
PNMA6A	0.146	1.66E-05	0.000967295
HEPH	0.146	1.67E-05	0.000968363
RAET1L	0.146	1.69E-05	0.000981694
AC093673	0.146	1.70E-05	0.000983771
RP11-107N15	0.146	1.72E-05	0.000986536
RP11-395A13	0.146	1.73E-05	0.000992606
CPXM1	0.146	1.73E-05	0.000995547
TMEM114	0.146	1.74E-05	0.000996505
ANTXR1	0.145	1.76E-05	0.001005611
GPR87	0.145	1.77E-05	0.001007175
PAEP	0.145	1.81E-05	0.001022015
RPL17P50	0.145	1.81E-05	0.001022015
TUBB6	0.145	1.82E-05	0.001027953
RP3-495K2	0.145	1.89E-05	0.001056275
COL8A2	0.145	1.91E-05	0.001065865
RP11-401O9	0.145	1.94E-05	0.001083947
TGFB2-AS1	0.145	1.98E-05	0.001101788
NETO1	0.145	1.99E-05	0.00110217
FLNA	0.145	1.99E-05	0.00110217
CTSK	0.144	2.02E-05	0.001113692
HCFC1R1	0.144	2.08E-05	0.00113735
TPST1	0.144	2.10E-05	0.001142647
BCL7C	0.144	2.10E-05	0.001142647
DGKI	0.144	2.11E-05	0.001146329
MIR210HG	0.144	2.17E-05	0.001167693
RP11-420L9	0.144	2.21E-05	0.00118351
HMGN1P13	0.144	2.21E-05	0.001185299
FOSL1	0.144	2.22E-05	0.001187814

WNT7B	0.144	2.27E-05	0.001208985
TMEM215	0.143	2.32E-05	0.001234081
FAM225B	0.143	2.34E-05	0.001238025
DSG4	0.143	2.40E-05	0.001265809
PLA2G5	0.143	2.46E-05	0.001289542
TRO	0.143	2.49E-05	0.001301994
VASN	0.143	2.54E-05	0.001315265
MSX1	0.143	2.55E-05	0.001319463
WDR13	0.143	2.57E-05	0.00132812
PTPRD	0.142	2.65E-05	0.001361938
CDC42EP1	0.142	2.66E-05	0.001363706
RP4-598P13	0.142	2.66E-05	0.001364424
BARX1	0.142	2.66E-05	0.001364424
MAP3K6	0.142	2.67E-05	0.001366679
LAMB1	0.142	2.67E-05	0.001366679
MYH4	0.142	2.69E-05	0.001372372
TREM1	0.142	2.72E-05	0.001384553
PDPN	0.142	2.73E-05	0.001388287
DACT1	0.142	2.74E-05	0.001389899
RAB3IL1	0.142	2.75E-05	0.001391157
SALL1	0.142	2.76E-05	0.001396541
HSPA8P4	0.142	2.76E-05	0.001397183
GIP	0.142	2.80E-05	0.001411979
SPOCK1	0.142	2.93E-05	0.001464973
AKR1B15	0.142	2.93E-05	0.001466936
ACTA1	0.142	2.96E-05	0.001478462
EYA4	0.142	2.98E-05	0.001485226
C9orf3	0.141	3.01E-05	0.001491192
ANGPTL2	0.141	3.09E-05	0.001525809
NEFM	0.141	3.12E-05	0.00153394
LRRC15	0.141	3.14E-05	0.001537286
SPINK1	0.141	3.17E-05	0.001544797
AGRN	0.141	3.19E-05	0.001551387
RP11-703I16	0.141	3.20E-05	0.001552555
DPYSL4	0.141	3.26E-05	0.001574475
C11orf24	0.141	3.27E-05	0.001577699
OSMR	0.141	3.28E-05	0.001581089
ADAM12	0.141	3.36E-05	0.001611716
LPPR5	0.141	3.36E-05	0.001611716
MYH13	0.141	3.38E-05	0.001617659
CCDC107	0.141	3.39E-05	0.001624412
LINC00857	0.140	3.42E-05	0.001634862
NR2F1-AS1	0.140	3.50E-05	0.001666067
ADAMTS12	0.140	3.51E-05	0.001669483
HSD17B6	0.140	3.53E-05	0.001674327
LRRC17	0.140	3.63E-05	0.001715205
NBL1	0.140	3.66E-05	0.001726061
PCDH7	0.140	3.68E-05	0.001727909
RP11-25L3	0.140	3.70E-05	0.001735049
ZNHIT1	0.140	3.79E-05	0.001769475
P4HA1	0.140	3.82E-05	0.001777891
LBP	0.139	3.89E-05	0.001803935

HOXC13-AS	0.139	4.01E-05	0.001849383
PSORS1C1	0.139	4.02E-05	0.001849409
DBN1	0.139	4.05E-05	0.001859544
CHST3	0.139	4.08E-05	0.001868217
WWTR1	0.139	4.10E-05	0.001872778
OLFML3	0.139	4.10E-05	0.001872778
RP11-191N8	0.139	4.14E-05	0.001886732
BCAR1	0.139	4.14E-05	0.001886732
WFDC1	0.139	4.15E-05	0.001886732
DIRC3	0.139	4.17E-05	0.001889826
RP11-91I8	0.139	4.24E-05	0.001921198
RAI14	0.138	4.45E-05	0.001997682
SHANK1	0.138	4.47E-05	0.002002119
ARHGEF38-IT1	0.138	4.60E-05	0.002053792
AC093850	0.138	4.75E-05	0.00210553
CTD-2269F5	0.138	4.75E-05	0.00210553
DES	0.138	4.83E-05	0.00212919
SMO	0.138	4.91E-05	0.002160304
GPR1	0.138	4.97E-05	0.002177274
SCG5	0.138	4.99E-05	0.002184276
DUOXA1	0.137	5.15E-05	0.002232473
FUT3	0.137	5.15E-05	0.002232473
SMOX	0.137	5.17E-05	0.002232998
RP11-88G17	0.137	5.22E-05	0.002249985
LAMB3	0.137	5.37E-05	0.002307105
SHF	0.137	5.42E-05	0.0023255
F2RL1	0.137	5.48E-05	0.002340777
LL21NC02-21A1	0.137	5.59E-05	0.002381606
ELN	0.137	5.62E-05	0.002388598
CNN3	0.136	5.74E-05	0.002431096
RP11-390P2	0.136	5.78E-05	0.002442604
PRICKLE1	0.136	5.81E-05	0.002453783
FEZ1	0.136	6.07E-05	0.002549477
COL10A1	0.136	6.16E-05	0.002575157
AL589743	0.136	6.16E-05	0.002575157
MUM1L1	0.136	6.27E-05	0.002612407
LINC01119	0.136	6.27E-05	0.002612407
VSTM2L	0.136	6.30E-05	0.002621986
METTL11B	0.136	6.33E-05	0.002629361
RP5-1172A22	0.136	6.44E-05	0.002662358
ACTA2	0.136	6.44E-05	0.002662358
C14orf37	0.135	6.49E-05	0.002677349
SPSB4	0.135	6.49E-05	0.002677349
FSTL1	0.135	6.52E-05	0.002682696
TWIST2	0.135	6.52E-05	0.002682696
IGFL1	0.135	6.64E-05	0.00272834
INAFM1	0.135	6.71E-05	0.002752209
BBOX1-AS1	0.135	6.77E-05	0.002772783
TEAD2	0.135	6.83E-05	0.002793762
KRT1	0.135	6.88E-05	0.002809471
MGST1	0.135	6.94E-05	0.002821578
LRRC36	0.135	7.01E-05	0.002838117

SPANXB2	0.135	7.01E-05	0.002838117
AP001471	0.135	7.01E-05	0.002838211
KRT79	0.135	7.13E-05	0.00287359
KRT12	0.135	7.18E-05	0.002889283
B4GALT2	0.135	7.29E-05	0.002926156
SLC2A12	0.134	7.40E-05	0.00296071
FAM3A	0.134	7.43E-05	0.002964298
ADAMTS2	0.134	7.51E-05	0.002983654
CD55	0.134	7.62E-05	0.003020979
RIN2	0.134	7.98E-05	0.003136933
COL6A3	0.134	7.99E-05	0.003137056
CTC-327F10	0.134	8.02E-05	0.003147811
RP11-395G23	0.134	8.12E-05	0.003176693
C9orf16	0.133	8.29E-05	0.003238178
CDH15	0.133	8.50E-05	0.003297183
MIR6815	0.133	8.83E-05	0.003404224
RP11-86H7	0.133	8.87E-05	0.003417541
ACKR4	0.133	8.90E-05	0.003423202
NCKAP5L	0.133	9.28E-05	0.003545712
HRH1	0.133	9.36E-05	0.003564432
FLG	0.132	9.44E-05	0.003588164
JOSD2	0.132	9.50E-05	0.00359797
RP11-999E24	0.132	9.53E-05	0.003606656
AP000432	0.132	9.57E-05	0.003615915
ZNF593	0.132	9.85E-05	0.0037001
KLK12	0.132	9.97E-05	0.00373581
GFPT2	0.132	0.000100658	0.003760022
VTCN1	0.132	0.000101172	0.003773324
RP11-713C5	0.132	0.000102653	0.003819573
KISS1	0.132	0.000103149	0.003829061
PCDHB7	0.132	0.000103501	0.00383667
SFRP2	0.132	0.000103515	0.00383667
MRGPRF	0.132	0.000105884	0.00389862
SLN	0.131	0.000109135	0.00398909
UCHL1	0.131	0.000113237	0.004097243
COL12A1	0.131	0.000113314	0.004097243
LINC00941	0.131	0.000113385	0.004097243
SLC29A4	0.131	0.000114728	0.00413951
RP11-752L20	0.131	0.000117525	0.004224427
FJX1	0.131	0.000119292	0.004278262
CTD-3247H4	0.130	0.000120193	0.00430733
GUK1	0.130	0.000122376	0.004362576
LINC01048	0.130	0.00012264	0.00436871
UBE2E3	0.130	0.000122843	0.004372687
FAM101B	0.130	0.000123675	0.004395731
SOX11	0.130	0.000124474	0.004417541
AL591893	0.130	0.000124923	0.004423568
CNN2	0.130	0.000125088	0.004424518
SBSN	0.130	0.000125992	0.004431783
LOXL1	0.130	0.000126809	0.004448155
RP11-1060J15	0.130	0.000127029	0.00445182
RP11-791G15	0.130	0.000127553	0.004463879

EHD2	0.130	0.000127819	0.004466337
PAM	0.130	0.000128698	0.004490471
KCNG2	0.130	0.000131205	0.004561231
LINC01060	0.130	0.000133449	0.004625757
RRAS	0.129	0.000136429	0.004708472
AC106786	0.129	0.000137256	0.004730159
RP1-65J11	0.129	0.000138327	0.004763619
C4orf26	0.129	0.000138497	0.004766032
MYH1	0.129	0.000139355	0.004781755
COLGALT1	0.129	0.000140828	0.004818408
DIRC1	0.129	0.000141062	0.004822934
LINC01096	0.129	0.000144825	0.004921752
CENPB	0.129	0.000145288	0.004921752
CLTCL1	0.129	0.000145437	0.004921752
KRT17	0.129	0.000145445	0.004921752
SERPINB2	0.129	0.000151194	0.005067489
C1QTNF1	0.129	0.000151973	0.005090024
PRDM6	0.128	0.000154163	0.005141665
CRYGN	0.128	0.000156117	0.005188686
DAPK3	0.128	0.000156238	0.005189084
WISP1	0.128	0.000157269	0.005216044
KRBOX1	0.128	0.000158693	0.005255954
NTM	0.128	0.000159628	0.005275921
GLT8D2	0.128	0.000165231	0.005402329
CCDC8	0.128	0.000165626	0.005406626
SSC5D	0.128	0.000168138	0.005469896
CNN1	0.128	0.000169826	0.005503606
MIR99AHG	0.128	0.000170521	0.005513516
NUPR1	0.127	0.000172139	0.005550633
PTTG1IP	0.127	0.00017219	0.005550633
CNPY4	0.127	0.000176127	0.005648766
TRIP6	0.127	0.0001798	0.005747218
GLMP	0.127	0.000184764	0.00588612
COMP	0.127	0.000185764	0.005914017
CHRDL2	0.127	0.000187985	0.005976754
FNDC1	0.127	0.000188603	0.005992384
CORO6	0.127	0.00018888	0.005993183
RP11-439M11	0.127	0.000190825	0.006034824
RXFP3	0.127	0.000191426	0.006049792
XXyac-YX65C7_A	0.127	0.000192292	0.006069117
PDLIM2	0.126	0.000193191	0.006089412
SNAI2	0.126	0.000198629	0.00620619
LIMK1	0.126	0.00019872	0.00620619
INF2	0.126	0.000199776	0.006226909
PGF	0.126	0.000200767	0.006234482
ZNF365	0.126	0.000200959	0.00623522
DLX5	0.126	0.000202483	0.006269453
LMX1A	0.126	0.000203236	0.006277246
AC112715	0.126	0.00020428	0.006297235
UROD	0.126	0.000206921	0.006355613
NANOS3	0.126	0.000206974	0.006355613
DFNA5	0.126	0.000209158	0.006403695

ANXA2P1	0.126	0.000210639	0.006434963
BCAT1	0.126	0.000211874	0.00645869
NRP2	0.126	0.000216888	0.006587843
MARVELD1	0.125	0.000217231	0.006594056
MYPN	0.125	0.000217676	0.006603357
TM4SF19	0.125	0.000218432	0.006622059
WBP5	0.125	0.000218825	0.006629772
FBN2	0.125	0.00022208	0.00670703
CTGF	0.125	0.000225136	0.00676503
PHLDA3	0.125	0.000226454	0.006791776
UNC5B-AS1	0.125	0.000227742	0.006821813
FAM57A	0.125	0.00022858	0.006838305
TMED1	0.125	0.000231244	0.006899007
LZTS2	0.125	0.000231469	0.006899007
RP11-184M15	0.125	0.000233867	0.006952795
ST6GAL2-IT1	0.125	0.000234052	0.006953958
FAM132A	0.125	0.000234867	0.0069669
GRAMD3	0.125	0.000235171	0.006969822
GIPC1	0.125	0.000237265	0.00701773
CCDC102A	0.125	0.000241472	0.007101417
KIF7	0.125	0.00024155	0.007101417
EFNA3	0.125	0.000241916	0.007105375
FOXF2	0.125	0.000242343	0.00711156
AC011893	0.124	0.000245972	0.007213603
CHMP4BP1	0.124	0.000247131	0.007229798
EMX2	0.124	0.000248135	0.007254701
NUCB1	0.124	0.000249031	0.007271983
POR	0.124	0.000252591	0.007330981
LZTS3	0.124	0.000253181	0.007343641
SH3PXD2B	0.124	0.000255668	0.007389272
PRRX1	0.124	0.000255685	0.007389272
LCN1	0.124	0.000256757	0.007409298
RDH8	0.124	0.000257608	0.007417144
NKAIN3	0.124	0.000257681	0.007417144
ARL4D	0.124	0.000262157	0.007518977
EGFL6	0.124	0.000262226	0.007518977
TIMM8BP2	0.124	0.000264119	0.007553842
KCNJ2-AS1	0.124	0.000264235	0.007553842
SLC39A13	0.124	0.000265908	0.007592575
CALD1	0.124	0.000266736	0.007608583
RP11-732A21	0.124	0.000267008	0.007610281
AC006262	0.124	0.000270799	0.007695321
ST20-MTHFS	0.123	0.00027528	0.007808664
LINC00922	0.123	0.0002757	0.007815926
CACNA1G	0.123	0.000278838	0.007890798
C17orf51	0.123	0.000280067	0.007911485
CTD-2532N20	0.123	0.00028066	0.007923534
HAGLROS	0.123	0.000281399	0.007939699
SLC44A3	0.123	0.000282692	0.007966733
GDF6	0.123	0.000282927	0.007968625
RP11-229O3	0.123	0.000287673	0.008083173
MOSPD3	0.123	0.000287988	0.008087277

FAP	0.123	0.000289651	0.008129188
ST6GAL2	0.123	0.000289852	0.00813001
NIPAL4	0.123	0.000290696	0.008140073
EDIL3	0.123	0.000292538	0.008181264
NDN	0.123	0.000295422	0.00824281
KRT80	0.123	0.000295636	0.00824281
AGPAT2	0.123	0.00029749	0.008276035
KRT87P	0.123	0.000297834	0.00827604
APLN	0.123	0.00029793	0.00827604
SLC16A10	0.123	0.000298012	0.00827604
IQCJ-SCHIP1	0.123	0.000302549	0.008359254
TRIP10	0.123	0.000302921	0.008363593
IGFBP6	0.122	0.000309266	0.008499363
FBXL19	0.122	0.000309807	0.008509332
RP11-323P17	0.122	0.000311063	0.008529058
RFPL1	0.122	0.000311516	0.008531657
AC007750	0.122	0.000311911	0.008537571
MAGED1	0.122	0.000321799	0.008744719
SH3PXD2A	0.122	0.00032202	0.008744719
RP11-473E2	0.122	0.000325507	0.008813476
RP5-1011O1	0.122	0.000328157	0.008875154
DUSP23	0.122	0.000330164	0.0089193
GAL	0.122	0.000332966	0.008973063
AP2S1	0.122	0.000333704	0.00897928
RP11-513O13	0.122	0.00033634	0.009024702
LUM	0.122	0.000337274	0.009039927
GRIN2D	0.122	0.000338526	0.009039927
HS3ST3A1	0.122	0.00033894	0.009039927
TTYH3	0.122	0.000339884	0.009058576
TSPAN9	0.121	0.000348827	0.009232639
FAM129B	0.121	0.000349599	0.00924167
GAREML	0.121	0.000349859	0.00924167
PGLS	0.121	0.000350115	0.009243295
DNM3OS	0.121	0.000351823	0.009267857
PCYT1B	0.121	0.000359502	0.009418044
CTC-537E7	0.121	0.000359716	0.009418457
GRIA3	0.121	0.000360167	0.009425091
CCDC67	0.121	0.000360498	0.009428589
CCDC124	0.121	0.000363417	0.009493839
MCAM	0.121	0.000364838	0.009510747
EMC10	0.121	0.000369315	0.009591684
RP11-96B2	0.121	0.000378333	0.009798194
AL589787	0.121	0.000381357	0.00983924
C3orf80	0.121	0.000383869	0.009871956
PGM2L1	0.121	0.000384088	0.009871956
MYOZ1	0.120	0.000386426	0.009910788
GDF5	0.120	0.000388796	0.009943706
TENM3	0.120	0.000388963	0.009943706
RP1-93H18	0.120	0.000389859	0.009955399
PDIA5	0.120	0.000392598	0.010004518
CTD-2263F21	0.120	0.000395922	0.010072966
RP11-231D20	0.120	0.000396539	0.010080538

LAMC2	0.120	0.000396815	0.010080538
RP11-573D15	0.120	0.000396855	0.010080538
LINC01559	0.120	0.000398776	0.010119738
CD109	0.120	0.000406943	0.010271049
LINC01212	0.120	0.000408949	0.01031622
WISP2	0.120	0.000409899	0.010323782
TMSB10	0.120	0.000411322	0.010354135
RP11-783L4	0.120	0.00042018	0.010543692
PMF1-BGLAP	0.120	0.000422705	0.010591456
FERMT2	0.120	0.000422751	0.010591456
DOK5	0.120	0.000423814	0.010612505
FTSJ1	0.120	0.000429502	0.010726744
RP13-631K18	0.120	0.000430463	0.010745101
KRT6A	0.119	0.000432348	0.010783464
SLC1A6	0.119	0.000432453	0.010783464
ADAMTS6	0.119	0.000433183	0.010796027
RP11-362K2	0.119	0.000435358	0.010833237
RP1-293L8	0.119	0.000436232	0.010849311
FKBP7	0.119	0.000436515	0.010850666
SLC16A1	0.119	0.000440571	0.010934374
PTPRD-AS1	0.119	0.000449743	0.011109913
LRP1	0.119	0.000453085	0.011169285
INSRR	0.119	0.000455963	0.011234426
C6orf15	0.119	0.000456425	0.011236163
TSPO	0.119	0.000456741	0.011236163
KRT6B	0.119	0.000458045	0.011256592
GBX2	0.119	0.000461709	0.011311629
AP000892	0.119	0.00046479	0.011363733
FLJ26245	0.119	0.000467777	0.011419172
LTBP1	0.119	0.000468039	0.011419724
ANXA8	0.119	0.000468683	0.011429589
PKM	0.119	0.000474971	0.011553348
ZNF358	0.119	0.00047655	0.011585831
INHBA	0.118	0.00048459	0.011733381
RP11-167H9	0.118	0.000485201	0.011736964
FHOD3	0.118	0.000485575	0.011739338
BPIFB4	0.118	0.000487977	0.011785435
AC004160	0.118	0.000489799	0.011817449
RP5-952N6	0.118	0.000492847	0.011878956
XXYLT1	0.118	0.000494042	0.01190174
PCDHB17P	0.118	0.000495914	0.011940787
AKR1C2	0.118	0.000496167	0.011940846
LAMA4	0.118	0.000498715	0.011971926
SH3RF3	0.118	0.000499368	0.011978653
ABCA13	0.118	0.000499498	0.011978653
GABRA5	0.118	0.000503647	0.012047809
RP11-372M18	0.118	0.000505311	0.012075487
RP11-443C10	0.118	0.000514692	0.012232155
AC007282	0.118	0.000516827	0.012264539
RNASE1	0.118	0.000517229	0.012266495
RPL7P9	0.118	0.000517425	0.012266495
UPP1	0.118	0.000519026	0.012281333

NGFRAP1	0.118	0.000519082	0.012281333
SLED1	0.118	0.000526324	0.012409515
CDR1	0.118	0.000527595	0.012433324
GJA1	0.118	0.000529211	0.012459077
DUOX2	0.118	0.000531139	0.012492124
MLK7-AS1	0.118	0.000531682	0.012498596
OSMR-AS1	0.118	0.000531939	0.012498596
TPM1	0.117	0.000538791	0.012622236
EPGN	0.117	0.000540049	0.012639267
ANXA2P2	0.117	0.000541594	0.012656772
SHOX2	0.117	0.000545455	0.012715807
AC108142	0.117	0.000550002	0.012811277
CALU	0.117	0.000555235	0.012912195
RASAL2-AS1	0.117	0.000563247	0.013054883
NOTUM	0.117	0.000564387	0.013070628
TNKS1BP1	0.117	0.000564835	0.013070628
MSRB2	0.117	0.000564897	0.013070628
NCS1	0.117	0.000565642	0.013077586
ADAT3	0.117	0.000572472	0.013201088
FAM180A	0.117	0.000572647	0.013201088
VIM	0.117	0.000573471	0.0132137
C10orf55	0.117	0.000573769	0.01321417
GGN	0.117	0.000578902	0.013288326
SLC22A2	0.117	0.000580075	0.013294651
RP4-580N22	0.117	0.000587761	0.013419599
DCBLD1	0.117	0.000591505	0.013466353
KCTD11	0.117	0.000592578	0.013484332
HIC1	0.117	0.000594131	0.013513224
PERM1	0.117	0.000600197	0.013618674
TMEM53	0.116	0.000608009	0.013745888
VAT1L	0.116	0.000608469	0.013747426
MAFB	0.116	0.000609635	0.01376722
CSMD1	0.116	0.000618855	0.013896382
GLP2R	0.116	0.000626398	0.014045882
HOXA11-AS1_5	0.116	0.000627003	0.014052828
RP11-14N7	0.116	0.000630406	0.01412246
RP11-513N24	0.116	0.000636109	0.014236828
CDK15	0.116	0.000639731	0.014285823
ANKRD1	0.116	0.000639798	0.014285823
VEPH1	0.116	0.000641149	0.014309276
SH3RF3-AS1	0.116	0.000649738	0.01444681
PXDC1	0.116	0.000652283	0.014476364
RP11-626G11	0.116	0.000654885	0.01451382
LINC00116	0.116	0.000655366	0.014515487
SELM	0.116	0.000656142	0.014517117
KRTAP2-3	0.116	0.000656253	0.014517117
PRDX5	0.116	0.000658049	0.0145501
MIR503HG	0.116	0.000659318	0.014561259
AHNAK2	0.116	0.000660172	0.014569355
PLIN2	0.116	0.00066045	0.014569355
EDNRA	0.116	0.000661253	0.014580331
KRT81	0.116	0.000665052	0.01463698

VCL	0.115	0.000671236	0.014739025
SEC16B	0.115	0.000671628	0.014740838
VSTM4	0.115	0.000674173	0.014777601
GS1-257G1	0.115	0.000674234	0.014777601
CDH11	0.115	0.000678579	0.014838699
NACAD	0.115	0.000681009	0.014878174
SEMA3D	0.115	0.000686355	0.014966197
VLDLR-AS1	0.115	0.000686609	0.014966197
RBP1	0.115	0.000691534	0.015032257
TSPAN15	0.115	0.000698345	0.015152647
CWH43	0.115	0.000709041	0.015349773
RP11-200A1	0.115	0.000710462	0.015367012
TMEM161A	0.115	0.000710483	0.015367012
BRI3	0.115	0.000720077	0.015518159
SPANXD	0.115	0.000721757	0.015533275
RP11-465K1	0.115	0.000723133	0.015555423
COMMD1	0.115	0.000730396	0.015648473
NPC2	0.115	0.000736732	0.015728607
RFPL4AP6	0.115	0.000737002	0.015728607
LINC00704	0.115	0.000737108	0.015728607
OBP2A	0.115	0.000739541	0.015768468
REP15	0.115	0.000739638	0.015768468
MXRA5	0.114	0.000753678	0.016024744
TRPM8	0.114	0.00076025	0.016135662
DZIP1	0.114	0.000762294	0.016171846
SFN	0.114	0.000769022	0.016271083
RAB20	0.114	0.000779649	0.01643024
YIPF2	0.114	0.00078008	0.016432056
BOK	0.114	0.000786912	0.016546697
CSTB	0.114	0.000791276	0.016609128
RP5-1198O20	0.114	0.000796769	0.01669097
RPS20P14	0.114	0.000796927	0.01669097
CRISPLD2	0.114	0.000798142	0.016709053
FAM155A	0.114	0.00080812	0.016858672
RP11-20I20	0.114	0.000813035	0.0169067
ROR2	0.114	0.000813127	0.0169067
GPR161	0.114	0.000813483	0.0169067
FSTL3	0.114	0.000814867	0.016925272
EXTL3	0.114	0.000820794	0.01701871
RP4-639F20	0.114	0.000826708	0.017096667
LAMA1	0.113	0.000831881	0.01716637
RP4-756H11	0.113	0.000840836	0.017313661
MMP16	0.113	0.000854711	0.017508525
GREM1	0.113	0.00085543	0.017508918
VCAN	0.113	0.000864943	0.017640532
AC114803	0.113	0.000869735	0.01769819
WWTR1-AS1	0.113	0.00087016	0.01769819
WNT5B	0.113	0.00087118	0.017708789
NTMT1	0.113	0.000872829	0.017727169
FLJ22447	0.113	0.000886841	0.017965783
TRNP1	0.113	0.000891051	0.018012675
TMED9	0.113	0.000891095	0.018012675

TWF2	0.113	0.000896014	0.018082407
THBS3	0.113	0.000898867	0.018116943
SCX	0.113	0.000899848	0.018129034
DTX4	0.113	0.000906805	0.018244242
PPL	0.113	0.000914711	0.018343134
HTRA3	0.113	0.000918151	0.01839662
PLA2G4E	0.113	0.000921044	0.01843133
MT1X	0.113	0.00092269	0.018456504
CHCHD2	0.112	0.000925984	0.01850685
HSD11B2	0.112	0.000937994	0.018723318
SV2C	0.112	0.000939422	0.01873612
MIR210	0.112	0.000944143	0.018806649
CRYAB	0.112	0.000944549	0.018806873
ADAMTS7P3	0.112	0.00094903	0.018857772
RP4-668J24	0.112	0.00094918	0.018857772
NOX4	0.112	0.000951796	0.018887995
CTD-2297D10	0.112	0.000952348	0.01888946
TGM5	0.112	0.000955167	0.018915489
GAPDH	0.112	0.000957647	0.018940977
HHLA3	0.112	0.000963099	0.019032987
SLC24A2	0.112	0.000965838	0.019063389
LRP3	0.112	0.000974403	0.019192672
PGRMC1	0.112	0.000982873	0.019304265
HIF1A-AS1	0.112	0.000983097	0.019304265
ADAMTS20	0.112	0.000983716	0.019304265
EEF1G	0.112	0.000990296	0.019409395
APCDD1	0.112	0.000997382	0.019524171
OXCT2P1	0.112	0.001005038	0.019655164
CNFN	0.112	0.001008607	0.01970197
PDLIM4	0.112	0.001008947	0.01970197
ZNF503	0.112	0.001013062	0.019756663
SCGB3A2	0.112	0.001017121	0.019795707
FKBP1A	0.112	0.00102545	0.019909949
ULBP2	0.111	0.001032435	0.02002378
HAPLN1	0.111	0.001033989	0.020043067
TIMM8AP1	0.111	0.001036963	0.02008436
RP11-398G24	0.111	0.001040009	0.020125564
FZD9	0.111	0.00104217	0.020152421
PRADC1	0.111	0.00105418	0.020295067
KLHDC8A	0.111	0.001061961	0.020402583
LTBR	0.111	0.001067273	0.020479869
LINC00887	0.111	0.001076109	0.020616188
CTD-3035K23	0.111	0.00107699	0.020617971
EPHB4	0.111	0.001077068	0.020617971
KB-1980E6	0.111	0.001078059	0.020628654
GGT6	0.111	0.0010807	0.020645999
RHOV	0.111	0.001083062	0.020657985
C1QTNF2	0.111	0.001083623	0.020660415
RP11-230G5	0.111	0.001088525	0.020711184
RPS26P47	0.111	0.00109356	0.020775019
SNHG23	0.111	0.001098451	0.020842991
GJA3	0.111	0.00110429	0.020926429

LIMCH1	0.111	0.001104605	0.020926429
SLC12A4	0.111	0.001107117	0.020957336
C1orf122	0.111	0.001115478	0.021063336
TSR3	0.111	0.001127143	0.021243554
HILS1	0.111	0.001138699	0.021351497
FAM58A	0.111	0.001139425	0.021356707
RNU6-403P	0.110	0.001145839	0.02143911
COL22A1	0.110	0.001146292	0.02143911
ST8SIA2	0.110	0.00115035	0.021485378
NID1	0.110	0.001151575	0.021486129
RP11-635L1	0.110	0.001151718	0.021486129
RP11-19E11	0.110	0.001151744	0.021486129
PEPD	0.110	0.001152949	0.021500194
RP1-240K6	0.110	0.001163001	0.021641326
USE1	0.110	0.001168699	0.02170787
RP11-308N19	0.110	0.001169019	0.02170787
SGCD	0.110	0.001181607	0.021876903
RPLP1	0.110	0.001181815	0.021876903
PLOD3	0.110	0.001182053	0.021876903
HOXA-AS2	0.110	0.001183375	0.021882678
ERCC1	0.110	0.001183568	0.021882678
KIAA1644	0.110	0.001188216	0.021943073
NALCN	0.110	0.001199387	0.022097957
ACTRT3	0.110	0.001203852	0.022163061
RP11-89C3	0.110	0.00120587	0.022175335
RP11-626H12	0.110	0.001225394	0.022438226
KLK11	0.110	0.001234668	0.022590673
FBLN2	0.110	0.001236991	0.022624493
LINC00601	0.110	0.001238082	0.02263575
RP1-191J18	0.110	0.001242214	0.022685165
ADAMTSL2	0.110	0.001245756	0.022741139
MYLK-AS1	0.110	0.001248154	0.022758733
MYHAS	0.110	0.00125293	0.02280218
EVA1B	0.110	0.001254938	0.022822739
CTB-13F3	0.109	0.001267569	0.023007048
RPL37	0.109	0.001268457	0.023014395
PEX6	0.109	0.001273334	0.02306773
FLG-AS1	0.109	0.001280666	0.02316531
B3GNT7	0.109	0.001283159	0.023192799
LAMB2	0.109	0.001288356	0.023260239
FAM207A	0.109	0.001295001	0.023344816
VWC2	0.109	0.001297905	0.023379423
PHC2	0.109	0.00130611	0.02348287
SLPI	0.109	0.001308371	0.023505066
ODF3L1	0.109	0.001308826	0.023505066
CLDN6	0.109	0.001316905	0.023605654
IER5L	0.109	0.001319217	0.023629312
FNDC5	0.109	0.001334197	0.023799176
NOTCH3	0.109	0.001336915	0.023829802
HOXD11	0.109	0.001349873	0.023978239
LINC00545	0.109	0.001351957	0.023986306
HAS2-AS1	0.109	0.001357354	0.02404116

TPTE2P4	0.109	0.001363842	0.024138123
CCNA1	0.109	0.001364936	0.024148512
SERPINF1	0.109	0.001367317	0.024161259
LINC00460	0.109	0.001367502	0.024161259
LINC01137	0.109	0.001367685	0.024161259
MYO10	0.109	0.001368803	0.024172031
GABRQ	0.109	0.001381556	0.024346425
CYB5R3	0.109	0.001383232	0.024346425
IFNE	0.109	0.001383279	0.024346425
CLIP2	0.109	0.001390962	0.024396553
EMX2OS	0.109	0.001392078	0.024396553
NINJ2	0.109	0.00139497	0.024434812
NOG	0.108	0.001403899	0.024555082
MRPS6	0.108	0.001406867	0.024588941
HHIP	0.108	0.001407433	0.024589798
LAMC1	0.108	0.001409858	0.024607348
RP11-381O6	0.108	0.001411119	0.024615662
PTP4A3	0.108	0.001411936	0.024615662
ANKRD2	0.108	0.001419815	0.024715466
CLDN2	0.108	0.001424509	0.024779073
RNF217	0.108	0.001433848	0.024887014
SGIP1	0.108	0.001441445	0.024955176
QPRT	0.108	0.001446251	0.025024953
RP11-66N11	0.108	0.001446526	0.025024953
RILP	0.108	0.001453623	0.025081827
RP11-438L7	0.108	0.001457532	0.025096839
LINC01050	0.108	0.001461111	0.025122146
BAIAP2L1	0.108	0.001462678	0.025140016
FOXL1	0.108	0.00146662	0.025197206
PLEKHA4	0.108	0.001468337	0.02520089
KLK8	0.108	0.001473266	0.025249094
ITFG3	0.108	0.00148648	0.025438939
MVP	0.108	0.00148755	0.025446519
FAM46B	0.108	0.001491194	0.025460557
SRPX	0.108	0.001492554	0.025460557
FNDC1-IT1	0.108	0.001495897	0.025490197
RP11-166A12	0.108	0.001498002	0.025516935
RP11-323C15	0.108	0.001499585	0.025534764
TAGLN2	0.108	0.001512165	0.025684695
SLAMF9	0.108	0.001522901	0.025830205
MBD3	0.108	0.00153066	0.025916028
C1orf143	0.108	0.001531125	0.025916028
C9orf170	0.108	0.001542221	0.026074312
SLC30A10	0.108	0.001543595	0.026088287
ARHGEF4	0.108	0.001546769	0.026132659
NDUFA4L2	0.108	0.001548398	0.02614164
CTD-2078B5	0.107	0.001554613	0.02620283
OPCML	0.107	0.001554774	0.02620283
LZTS1	0.107	0.001557612	0.026241385
FOXL2	0.107	0.001562121	0.026308044
RAET1G	0.107	0.001562805	0.026310254
SMPD1	0.107	0.001575217	0.026463091

KB-176G8	0.107	0.001578733	0.026475465
PALM2	0.107	0.001581181	0.026488541
CTD-2186M15	0.107	0.001582294	0.026492751
PAPPA	0.107	0.001582545	0.026492751
ITPRIP	0.107	0.001589436	0.02658008
PRAF2	0.107	0.001593531	0.026620525
GGCX	0.107	0.00159576	0.026648416
CLDN8	0.107	0.001604417	0.026780467
P3H4	0.107	0.001607286	0.026797555
ITGB6	0.107	0.001609284	0.026817829
LYNX1	0.107	0.001609979	0.026820039
RP11-1069G10	0.107	0.001611126	0.026821382
SH2D5	0.107	0.001618722	0.026937411
GALNT10	0.107	0.001629516	0.027042895
PIANP	0.107	0.001629603	0.027042895
AVEN	0.107	0.001630585	0.027049769
MRPS21	0.107	0.001631837	0.02706111
ERI3	0.107	0.001639274	0.027156071
BAG2	0.107	0.001647167	0.027267858
CASP14	0.107	0.001657754	0.027390885
MEGF6	0.107	0.001668778	0.02752039
FAM133A	0.107	0.001681572	0.027670454
TMEM2	0.107	0.001681944	0.027670454
TMPRSS11E	0.107	0.001687288	0.027720059
PSG5	0.107	0.00169796	0.027876166
CSRP2	0.107	0.001711625	0.028032863
FAM19A5	0.107	0.001714942	0.02806789
RCAN1	0.107	0.001716408	0.028082232
SPRR2F	0.106	0.001721618	0.028157791
LIF	0.106	0.001724629	0.028174955
BEND6	0.106	0.001725033	0.028174955
AHDC1	0.106	0.001728443	0.02821129
GPC6	0.106	0.001734596	0.028292337
CDH3	0.106	0.00174895	0.028477692
CTC-518P12	0.106	0.001766076	0.028697686
MIR6133	0.106	0.001773387	0.028806654
NDNF	0.106	0.001775791	0.028835866
RP1-37C10	0.106	0.001785933	0.028910828
SLC11A1	0.106	0.001786035	0.028910828
AL603650	0.106	0.001786082	0.028910828
PCBP4	0.106	0.001786379	0.028910828
TMEM261	0.106	0.001787081	0.028910828
RHOC	0.106	0.001797961	0.029047322
RP11-964E11	0.106	0.001799264	0.029058506
TBC1D3	0.106	0.001801085	0.02907382
SLC4A11	0.106	0.001812202	0.029219098
RP11-1105O14	0.106	0.001819581	0.029317043
STON1	0.106	0.00184214	0.029575983
SNHG18	0.106	0.001844122	0.029575983
CYR61	0.106	0.001844346	0.029575983
PMF1	0.106	0.001849593	0.029620228
KLK7	0.106	0.001852273	0.029653175

TRPV4	0.106	0.001857572	0.029718028
AKR1C1	0.106	0.001859073	0.029732056
TPM4	0.106	0.001871205	0.029885948
LINC01301	0.106	0.001871837	0.029886013
RNU6-18P	0.106	0.001896985	0.030206515
SNTA1	0.105	0.001909717	0.030309394
TBC1D2	0.105	0.001916763	0.030369153
ZFP42	0.105	0.001923705	0.030458874
PDE9A	0.105	0.001933568	0.030584871
MYH2	0.105	0.001933969	0.030584871
DZIP1L	0.105	0.001936028	0.030603129
RP11-130L8	0.105	0.00193836	0.030629815
RP5-864K19	0.105	0.001947361	0.03074979
F2RL3	0.105	0.001947889	0.03074979
MRPS26	0.105	0.001949236	0.030760857
C1orf53	0.105	0.001952651	0.030804535
TNNI2	0.105	0.001957074	0.030843662
RP11-479G22	0.105	0.001976545	0.031078543
TEX36	0.105	0.001979865	0.031099937
ARF5	0.105	0.001989825	0.031225497
FAM214B	0.105	0.001990881	0.031231775
ENO1	0.105	0.001997015	0.031310955
C10orf10	0.105	0.001998445	0.031310955
NLGN2	0.105	0.001998575	0.031310955
SPTLC1P5	0.105	0.002003276	0.031343636
LINC01142	0.105	0.002026336	0.031608127
CAPN5	0.105	0.00203016	0.031649893
SSR2	0.105	0.00203355	0.031692366
CLDN10	0.105	0.002040142	0.031753545
AC093627	0.105	0.002041508	0.03176443
RP11-357P18	0.105	0.002049602	0.03182797
SERPINB7	0.105	0.002052732	0.03185581
SLC38A5	0.105	0.002058554	0.031935757
KCNJ12	0.105	0.002060356	0.0319533
KLK10	0.105	0.002073221	0.032110988
PDGFRB	0.105	0.002096462	0.032328675
RP11-679B19	0.104	0.002116266	0.032523844
ATP5EP2	0.104	0.002125456	0.032644009
HSPB3	0.104	0.002131561	0.032716674
LY6D	0.104	0.002139323	0.032806415
RP1-140K8	0.104	0.0021568	0.033003638
AC004538	0.104	0.002173573	0.033211659
RP11-9N12	0.104	0.002184087	0.033291883
SEMA5B	0.104	0.002189321	0.033338755
PRKCSH	0.104	0.00218959	0.033338755
CPNE1	0.104	0.002190775	0.033346134
TPRA1	0.104	0.002194813	0.033396927
ANXA1	0.104	0.002200853	0.033449779
ENC1	0.104	0.002208171	0.033546584
FTH1P27	0.104	0.002213594	0.033596831
ZFP36L2	0.104	0.002222643	0.033669795
GCKR	0.104	0.002227339	0.033719481

MAD1L1	0.104	0.002246008	0.033958054
MYH8	0.104	0.002246663	0.033958054
LA16c-444G7	0.104	0.002268226	0.034164702
SCEL	0.103	0.002324028	0.034785186
ALDH1B1	0.103	0.002328243	0.034818409
MMP28	0.103	0.002346668	0.034979158
DKKL1	0.103	0.002348584	0.034979158
NAP1L3	0.103	0.002357521	0.035058226
C7orf73	0.103	0.002362189	0.035102644
AC007796	0.103	0.002384295	0.035342927
KLF14	0.103	0.002398072	0.035436851
GRHL3	0.103	0.002399209	0.035442655
ASIP	0.103	0.002408163	0.035533544
LINC01105	0.103	0.002408346	0.035533544
FAT1	0.103	0.002414267	0.035576821
KCNJ15	0.103	0.00242238	0.035625087
AKR1B1P2	0.103	0.002442228	0.035822606
FAM8A3P	0.103	0.002451266	0.035899894
CH17-360D5	0.103	0.002452445	0.035906116
HSPB7	0.103	0.002454731	0.035928546
RP11-171N4	0.103	0.002467797	0.036053302
KRT6C	0.103	0.002482124	0.036166361
RP11-66B24	0.103	0.002482135	0.036166361
C20orf141	0.103	0.002482624	0.036166361
RP11-126K15	0.103	0.002488776	0.036226442
CNTN6	0.103	0.002503013	0.036344822
APP	0.103	0.002513449	0.036440805
PLOD2	0.103	0.00252561	0.036562264
RP11-1020M18	0.103	0.002538089	0.036695202
TMUB1	0.103	0.002541118	0.036719006
LMCD1	0.103	0.002546959	0.036758797
CTC-546K23	0.103	0.002550733	0.036768699
NDUFS6	0.102	0.002560955	0.036871405
PARVA	0.102	0.002565608	0.036927232
RUSC2	0.102	0.002573802	0.03702279
WTAPP1	0.102	0.002583023	0.037131082
RPL29P19	0.102	0.002611787	0.037456066
PDAP1	0.102	0.002616984	0.037508002
FHL2	0.102	0.002621715	0.037558453
FBN1	0.102	0.002622081	0.037558453
YIF1B	0.102	0.002631264	0.037667326
RAD51AP2	0.102	0.002643355	0.037817686
RP11-94A24	0.102	0.002647335	0.037863255
MGAT1	0.102	0.00264872	0.037871679
TCF7L2	0.102	0.002668167	0.03804695
PPAN	0.102	0.002676234	0.038116323
LL22NC03-N14H11	0.102	0.002686472	0.038227851
ANXA3	0.102	0.002689364	0.038246145
MXRA5Y	0.102	0.002697615	0.038306296
RABGEF1	0.102	0.002708154	0.03838612
ITPA	0.102	0.002708159	0.03838612
RP11-895K13	0.102	0.002712685	0.038417197

MZT2B	0.102	0.002717086	0.038445237
CYP11A1	0.102	0.002739124	0.03871106
MSANTD3	0.102	0.00274008	0.038711753
FIS1	0.102	0.002754514	0.03880191
RN7SKP231	0.102	0.002760551	0.038837595
DNM1	0.102	0.002777497	0.039010273
AKR1B10	0.102	0.002779393	0.039013893
TBX5	0.102	0.002798899	0.03918376
FHOD1	0.102	0.002803515	0.03923515
GLYATL2	0.102	0.002806005	0.039248641
TNFRSF19	0.102	0.002813245	0.039315266
FETUB	0.102	0.002816761	0.039330872
RP11-767I20	0.102	0.002817476	0.039330872
ZNF628	0.101	0.002825425	0.039404549
RP11-411K7	0.101	0.002833618	0.039480955
GAS6	0.101	0.002843285	0.039549392
UNC5CL	0.101	0.002855881	0.039678253
VMO1	0.101	0.002867972	0.039801567
PRKD1	0.101	0.00286957	0.039810375
ATP13A5	0.101	0.002871485	0.039813751
ARHGAP22	0.101	0.002872615	0.039817815
NAT14	0.101	0.002879969	0.039873342
RP11-14D22	0.101	0.002887321	0.03994029
SPRR2C	0.101	0.002892187	0.039984385
RNF144A	0.101	0.002897716	0.040025958
FAM71E1	0.101	0.002901423	0.04005393
C2orf54	0.101	0.002908562	0.040129218
RP11-469N6	0.101	0.002921346	0.04025896
HDGFRP2	0.101	0.002924438	0.040261436
STOML1	0.101	0.002930378	0.040325086
RTN4RL2	0.101	0.002932035	0.040327688
NTNG2	0.101	0.002939254	0.040400529
CCND2-AS1	0.101	0.00294516	0.040458366
APOPT1	0.101	0.002955917	0.040547661
INA	0.101	0.002964277	0.040615543
RP1-78O14	0.101	0.002974276	0.040682315
RP11-279O9	0.101	0.002976993	0.040707788
GLRX5	0.101	0.002979796	0.040734425
CCL26	0.101	0.002990065	0.04082793
MYO1B	0.101	0.002993119	0.040846211
CTD-2308L22	0.101	0.00300055	0.040917093
CASC10	0.101	0.003005791	0.04094875
RP11-98D18	0.101	0.003015155	0.041036693
ST14	0.101	0.003019847	0.041041056
ATAD3A	0.101	0.003023577	0.041073562
CPZ	0.101	0.003031917	0.041128195
RP11-86K22	0.101	0.003056087	0.041374421
SCPEP1	0.101	0.003067362	0.041475369
ALKBH4	0.101	0.00306796	0.041475369
DIRAS3	0.101	0.003072315	0.041522456
SERPINB11	0.101	0.003079011	0.041558042
DKK3	0.101	0.003085292	0.041615182

DPM3	0.101	0.003089918	0.04165398
ANGPT2	0.101	0.003103134	0.041793596
MIR9-1	0.100	0.003105933	0.041803261
VWA5A	0.100	0.003122	0.041951669
AP000240	0.100	0.003122571	0.041951669
SLC35D3	0.100	0.003123708	0.041955116
VASP	0.100	0.003128729	0.041998865
RP11-230B22	0.100	0.00314262	0.042137831
CTD-2287O16	0.100	0.003145857	0.042157498
SSTR2	0.100	0.003151762	0.042224742
ARPC1B	0.100	0.003153993	0.042236261
SMAD5-AS1	0.100	0.003154395	0.042236261
S100A16	0.100	0.00316106	0.042301712
RP11-832A4	0.100	0.003162796	0.042313056
EPS15P1	0.100	0.003179212	0.042449205
S100A9	0.100	0.003184797	0.042483746
MARCKSL1	0.100	0.003185733	0.042483746
RPL39P40	0.100	0.003189793	0.042507065
RPS4XP23	0.100	0.003198335	0.042585152
FZD2	0.100	0.003220322	0.042818062
ZFPM2	0.100	0.003227175	0.042873261
NUDT10	0.100	0.003239871	0.043029929
IGF2	0.100	0.003265809	0.04325381
ANKRD35	0.100	0.003269391	0.043277177
CXCL8	0.100	0.003275163	0.043292226
RP11-429E11	0.100	0.003287369	0.043370507
RASGRF2-AS1	0.100	0.003298291	0.043462435
SEC61A1	0.100	0.003323534	0.043678238
AL139099	0.100	0.003339141	0.043786694
IL13RA2	0.100	0.003354234	0.043924297
DSTN	0.100	0.003362095	0.044014983
ZSWIM4	0.100	0.003378359	0.04417932
RBMS1	0.100	0.003392639	0.044305236
RP11-685B14	0.100	0.003406799	0.044392784
TMED3	0.099	0.00341537	0.04448734
WT1	0.099	0.003415924	0.04448734
JHDM1D-AS1	0.099	0.00342002	0.04451634
MAN1B1	0.099	0.003431157	0.044636908
TRIM29	0.099	0.003445213	0.044782229
RP11-127L20	0.099	0.003450638	0.044816895
SEMA7A	0.099	0.003471452	0.045013576
RHEBP2	0.099	0.003475164	0.045037185
AURKAIP1	0.099	0.003478844	0.04506647
MME	0.099	0.003480313	0.045067117
CTC-441N14	0.099	0.003483648	0.045085787
LINC01265	0.099	0.003491095	0.04515762
RP11-677M24	0.099	0.003524347	0.045476591
FAM212A	0.099	0.00353283	0.045524371
KRT16P1	0.099	0.003534784	0.045537232
MN1	0.099	0.003562423	0.04579371
IL17D	0.099	0.003577963	0.045911461
ADRA1D	0.099	0.003602749	0.046081438

CDH2	0.099	0.00360416	0.046081965
RP11-676J12	0.099	0.003606978	0.046093254
CTB-178M22	0.099	0.003641163	0.046428249
BFSP1	0.099	0.003644032	0.046428249
MTHFD1L	0.099	0.00367019	0.046663149
EPN1	0.099	0.00370054	0.046923757
LKAAEAR1	0.099	0.003702414	0.04693502
LCAT	0.099	0.00371925	0.047085782
HHIP-AS1	0.099	0.003734342	0.047205779
AP001626	0.099	0.003737621	0.047230479
RP11-524D16__A	0.099	0.003741196	0.047250587
SHISA6	0.098	0.003754713	0.047357279
HOXA3	0.098	0.003757401	0.047357279
CCDC71L	0.098	0.003759979	0.047374736
CCK	0.098	0.003762115	0.047376589
STX4	0.098	0.003769254	0.047428865
NLRX1	0.098	0.003779053	0.047476911
CASC15	0.098	0.003793081	0.047585514
HAND2	0.098	0.003793693	0.047585514
DCN	0.098	0.003798529	0.047633627
LAMB4	0.098	0.003815361	0.047781805
BCL2L12	0.098	0.003824157	0.047827157
AGPAT4-IT1	0.098	0.003843266	0.048005046
PPIB	0.098	0.003847006	0.04802657
LINC01235	0.098	0.003848326	0.048030456
CEMIP	0.098	0.003868355	0.048179427
C7orf50	0.098	0.003922104	0.048708752
CCDC85B	0.098	0.003924949	0.048718668
FAM127B	0.098	0.003926018	0.048719249
MFI2	0.098	0.003928666	0.048722705
SULF2	0.098	0.003928783	0.048722705
CD82	0.098	0.003936008	0.048766975
FZD1	0.098	0.003945862	0.048850942
RP11-9N20	0.098	0.003975291	0.049125894
SNORD113-3	0.098	0.004004814	0.049413802
PDLIM3	0.098	0.004006932	0.049427131
TMEM256	0.098	0.004009567	0.049446821
RP11-513G11	0.098	0.004017021	0.049525923
RP11-313P22	0.098	0.004026732	0.049594301
YBX3	0.098	0.004032757	0.049650933
IGF2BP2	0.098	0.00403412	0.049650933
RPL36	0.098	0.004037847	0.049679808
SULF1	0.098	0.004057347	0.049881073
NPAS2	0.098	0.004063604	0.049919342
KRT3	0.098	0.004065312	0.049927439
SAMD14	0.098	0.004073606	0.049990628
RP11-849N15	0.098	0.004112304	0.050394724
RP11-82L18	0.098	0.004120275	0.050433391
NAA10	0.097	0.004125519	0.050475328
EIF5A2	0.097	0.004129502	0.050507388
MIF	0.097	0.004132676	0.050520265
RRAD	0.097	0.004144646	0.050640597

NXT1	0.097	0.004148948	0.050656159
RPL3P4	0.097	0.004163441	0.050805073
LINC01482	0.097	0.004173296	0.05089925
JPH2	0.097	0.004181834	0.050951189
RP11-499E18	0.097	0.00419512	0.051074309
MMP19	0.097	0.004205779	0.051118119
ATG101	0.097	0.004216784	0.051204887
LINC01505	0.097	0.004219458	0.051204887
ID3	0.097	0.004223352	0.051234225
MSRB1	0.097	0.004247372	0.051473173
DMKN	0.097	0.004256977	0.051529779
SLC6A8	0.097	0.004261488	0.051550863
LRRC3	0.097	0.004262441	0.051550863
KCNB2	0.097	0.004264433	0.051561861
KIRREL	0.097	0.00429879	0.051793189
NENF	0.097	0.004305958	0.051866425
ADAM23	0.097	0.004316804	0.051970784
SLC15A2	0.097	0.004327833	0.052037789
SERPINB5	0.097	0.004346646	0.052198109
BNC2	0.097	0.004358341	0.052257822
RECK	0.097	0.00436384	0.052299096
RP11-445N18	0.097	0.004372984	0.052329681
ITGB5	0.097	0.00438991	0.052479475
RNF128	0.097	0.004395847	0.052537265
SEMA4B	0.097	0.004406662	0.052574171
MSRB3	0.097	0.004410981	0.052599347
KRT17P3	0.097	0.004430532	0.052779627
DCBLD2	0.097	0.004431761	0.052781068
GRID1	0.097	0.004438432	0.052847297
SLC1A7	0.097	0.004450636	0.052923057
C1orf86	0.097	0.004469722	0.053113635
DMPK	0.097	0.004472291	0.053130906
AK5	0.097	0.004479125	0.053167489
RP11-290H9	0.097	0.004479354	0.053167489
TMEM59L	0.097	0.004488863	0.053204175
TRIOBP	0.097	0.004519906	0.053469833
RP11-215E13	0.097	0.004521824	0.053479238
RP11-466H18	0.096	0.00453013	0.053564174
GDA	0.096	0.00454116	0.053667949
SHOX	0.096	0.004549407	0.053711849
MTFP1	0.096	0.00455395	0.05373781
IGFBP3	0.096	0.004561619	0.053762987
RP11-290O12	0.096	0.004576047	0.053856535
AB019441	0.096	0.004592682	0.053974398
DEFB1	0.096	0.004593156	0.053974398
MFGE8	0.096	0.004597007	0.054006328
NDUFS4	0.096	0.004600465	0.054033627
B4GALT7	0.096	0.004638661	0.054384182
GNG5	0.096	0.004657238	0.054539038
PHLDB1	0.096	0.00466581	0.054599145
SMIM24	0.096	0.004674694	0.054675026
CAV2	0.096	0.004676693	0.054675026

RNF121	0.096	0.004676886	0.054675026
ARHGAP28	0.096	0.004698578	0.054847834
PIK3R2	0.096	0.00470305	0.054886584
LCN2	0.096	0.004730388	0.055097066
LINGO2	0.096	0.004736875	0.055119263
CTB-102L5	0.096	0.004743096	0.055178176
C1orf94	0.096	0.00474829	0.055210087
SYT14	0.096	0.004748426	0.055210087
FNDC3B	0.096	0.004761054	0.055245587
AP001434	0.096	0.004790458	0.055417814
PNCK	0.096	0.004801611	0.055492918
NRK	0.096	0.004806991	0.055541617
MAOB	0.096	0.00483862	0.055785275
KLK2	0.096	0.00488933	0.056233785
GADD45B	0.096	0.004892271	0.056254034
RP11-89M22	0.096	0.00490385	0.05633277
POPDC3	0.096	0.004906775	0.056338094
PBXIP1	0.096	0.004912242	0.056366502
LAMC3	0.096	0.004915493	0.056371348
RP4-673M15	0.096	0.004946307	0.056683772
RP13-128O4	0.096	0.004951576	0.056716865
DHDH	0.096	0.004954197	0.056733243
RP11-576I22	0.095	0.004966078	0.056855621
OLFM2	0.095	0.004969565	0.056868207
LHPP	0.095	0.00497246	0.056887668
RNF112	0.095	0.005016248	0.057231365
RP11-14J7	0.095	0.005018124	0.057231365
NDUFA11	0.095	0.005027106	0.057320075
MYLPF	0.095	0.005030148	0.05734104
CASC18	0.095	0.005044858	0.05742626
ZNF362	0.095	0.005049165	0.057461548
RP11-91K9	0.095	0.005051097	0.057469807
MEG9	0.095	0.005060123	0.057558747
RPS2	0.095	0.005079253	0.057693675
AKR1E2	0.095	0.005093737	0.057811084
TSPAN18	0.095	0.005108206	0.05792584
LINC00964	0.095	0.005143492	0.058228926
NSFL1C	0.095	0.005150942	0.05827171
NFE2	0.095	0.005171659	0.058408969
RN7SKP51	0.095	0.005177551	0.058418527
ABC7-42391500H16	0.095	0.005178639	0.058418527
DIRAS1	0.095	0.005193876	0.058548811
KIAA1549L	0.095	0.005210257	0.05865537
PCSK1N	0.095	0.00521561	0.058691179
SH3RF2	0.095	0.005216364	0.058691179
RP11-114M1	0.095	0.005220582	0.058723459
CPN2	0.095	0.005224803	0.058723459
NRP1	0.095	0.005224838	0.058723459
TBX5-AS1	0.095	0.005234899	0.058767704
ESM1	0.095	0.005235504	0.058767704
GNAS-AS1	0.095	0.005245831	0.058869744
MT1P3	0.095	0.005248466	0.058883568

ADAMTS4	0.095	0.005253245	0.058883568
GPR162	0.095	0.005254646	0.058885416
TMEM217	0.095	0.005259378	0.058924587
TWIST1	0.095	0.005263051	0.058942911
UGT1A6	0.095	0.005272267	0.059012447
SHBG	0.095	0.005273416	0.059012447
RP11-183I6	0.095	0.00527705	0.059039243
MFAP5	0.095	0.005278625	0.059043
TSHZ3	0.095	0.005291016	0.059139925
SLC52A2	0.095	0.005294906	0.059156546
ADAD2	0.095	0.005312172	0.059265101
DPP6	0.095	0.005313414	0.059265101
PTGIR	0.095	0.005317427	0.059282096
FABP5	0.095	0.00532343	0.059321246
TMEM35	0.095	0.005335769	0.059400567
ART5	0.095	0.005345814	0.059459382
FKBP8	0.095	0.005358975	0.059550142
CTD-2154B17	0.095	0.005366596	0.059607017
ISM1	0.095	0.005378222	0.059694381
PRSS22	0.095	0.005390613	0.059804038
DOHH	0.095	0.005420482	0.059995657
OSTC	0.095	0.005422319	0.060002037
XKR5	0.095	0.005425837	0.06001308
SALL4	0.095	0.005436329	0.060115168
GJC2	0.094	0.0054568	0.060285551
KRT16P2	0.094	0.00546095	0.060317413
MXRA7	0.094	0.005491999	0.060573718
R3HDML	0.094	0.005500493	0.060627633
R3HCC1	0.094	0.005505641	0.060654367
RBPMS	0.094	0.005521413	0.060723095
LINC00882	0.094	0.005522607	0.060723095
TUBA1A	0.094	0.005524111	0.060723095
WNT11	0.094	0.005531866	0.060734478
UNC93A	0.094	0.005537266	0.060751755
SLC15A1	0.094	0.005548339	0.060831205
TXNDC17	0.094	0.005576389	0.061040374
ZP1	0.094	0.005600485	0.061158024
STEAP1	0.094	0.005601092	0.061158024
FGL1	0.094	0.005601262	0.061158024
DEXI	0.094	0.005603683	0.061170434
FAM26E	0.094	0.005607657	0.061199786
GPR3	0.094	0.005625058	0.061327752
NKX2-8	0.094	0.005634492	0.061354322
RP11-244F12	0.094	0.005644164	0.061429298
AC010729	0.094	0.005653646	0.061490335
IGFL1P1	0.094	0.005665109	0.061572828
MYO1E	0.094	0.00566995	0.061603014
PDZK1IP1	0.094	0.005673574	0.061608593
GPR153	0.094	0.00568443	0.061642151
SYCE1L	0.094	0.005688607	0.061659379
IDH2	0.094	0.005705447	0.061782568
MRPL28	0.094	0.005709053	0.061782568

SLC43A2	0.094	0.005729902	0.061963884
TCF7L1	0.094	0.005731012	0.061963884
VWCE	0.094	0.005737588	0.061995114
RP11-557L19	0.094	0.005742101	0.062013392
ZNF474	0.094	0.005754266	0.062130682
KLC3	0.094	0.005757988	0.062156776
SIX5	0.094	0.005772903	0.062275439
KLK9	0.094	0.005782529	0.062351035
NAA60	0.094	0.005805259	0.062553636
CNTN3	0.094	0.005844843	0.062880581
BX842568	0.094	0.00585044	0.06289872
FGFRL1	0.094	0.005860388	0.062976696
PLA2G15	0.094	0.005862506	0.062985241
METRNL	0.094	0.005865413	0.063002258
RNU6-285P	0.094	0.005868188	0.063017852
B4GALT1-AS1	0.094	0.005942071	0.063549865
MPZL1	0.094	0.005947398	0.063581757
EIF4EBP1	0.094	0.005950033	0.063595652
PTX3	0.093	0.005959413	0.063632561
RP11-38H17	0.093	0.005961503	0.063632561
COL23A1	0.093	0.005973044	0.063727192
RUNX1	0.093	0.005976451	0.063735414
RP11-42I10	0.093	0.005976491	0.063735414
GABRE	0.093	0.00600383	0.063955364
KCNG1	0.093	0.006009315	0.063985162
NDUFS5	0.093	0.006018924	0.064073153
RAB40A	0.093	0.006031413	0.064163082
NRN1	0.093	0.006043582	0.064235141
PNLIP	0.093	0.006053269	0.064283601
RP11-150C16	0.093	0.006054232	0.064283601
EIF3K	0.093	0.006071217	0.064413866
PPEF1	0.093	0.006086045	0.064528063
ALKBH7	0.093	0.006124184	0.064773825
CTNNBIP1	0.093	0.006141326	0.064897482
TAF7L	0.093	0.006144384	0.064911824
HOMER2P2	0.093	0.006146757	0.064911824
DKFZp434J0226	0.093	0.006165688	0.065010681
RP11-71E19	0.093	0.006192014	0.06518724
MYH9	0.093	0.006210897	0.06533142
ADORA2B	0.093	0.006214654	0.06535335
PQLC1	0.093	0.006227383	0.065443859
MON1A	0.093	0.006259261	0.0656485
MCTS1	0.093	0.006262041	0.065663196
PPP1R14B	0.093	0.00627202	0.065724135
RP11-309M7	0.093	0.006274172	0.065724135
MMP12	0.093	0.006274753	0.065724135
RP11-175B9	0.093	0.006293438	0.065876392
REXO2	0.093	0.006297167	0.065900936
RP11-1124B17	0.093	0.006300858	0.065907419
KDELR3	0.093	0.006313753	0.066016492
MASP1	0.093	0.006328723	0.066114962
CADM4	0.093	0.00635258	0.06632054

GAA	0.093	0.006391695	0.06658748
SIL1	0.093	0.00639408	0.066593232
LINC00445	0.093	0.00641011	0.066701836
ACOT9	0.093	0.006452749	0.067052131
TNFRSF21	0.093	0.006463205	0.067107707
FZD8	0.093	0.006466173	0.06710927
NRG1	0.093	0.006471517	0.067120852
ARHGAP23	0.093	0.006475668	0.067149284
RFXANK	0.093	0.006498445	0.067297551
DUTP6	0.093	0.006508583	0.067329339
NOS2	0.092	0.006518123	0.067374469
GALNT15	0.092	0.006521868	0.067374469
PPP1R14A	0.092	0.006522182	0.067374469
ITGB1	0.092	0.006536692	0.06747357
RP11-115I9	0.092	0.006552048	0.067555434
MIR4768	0.092	0.006557389	0.067555434
PDGFC	0.092	0.006563379	0.0675879
RASGRF2	0.092	0.006577643	0.067705514
EPHB2	0.092	0.00658629	0.067765224
SYT8	0.092	0.006591621	0.067805432
ZDHHC1	0.092	0.006611544	0.067937003
ITGB1BP1	0.092	0.006613322	0.067940617
TGM7	0.092	0.006654554	0.068218153
UNC5B	0.092	0.006663231	0.068232574
ADAM33	0.092	0.006674911	0.068286253
LYPLA2	0.092	0.006687242	0.068346194
AC138647	0.092	0.006731408	0.068635573
ATE1-AS1	0.092	0.006762556	0.068849991
C1orf116	0.092	0.006780874	0.069006987
CADPS	0.092	0.006792858	0.069069906
ERCC2	0.092	0.006803159	0.069159884
RP11-809F4	0.092	0.00682628	0.069320949
RGCC	0.092	0.006833849	0.069353444
MUC20	0.092	0.006838327	0.069375097
PLXNA2	0.092	0.006856006	0.069489467
TRAPPC3	0.092	0.00685858	0.069499315
SHC1	0.092	0.006864572	0.06951998
ISLR2	0.092	0.006864855	0.06951998
AC007193	0.092	0.006866464	0.069521488
ZNF428	0.092	0.00688543	0.06961867
DPP4	0.092	0.006886295	0.06961867
RP11-615I2	0.092	0.006892034	0.069661907
LINC01449	0.092	0.006895852	0.069685709
AC016999	0.092	0.006914228	0.069797362
LSR	0.092	0.006925806	0.069869804
SERPINE1	0.092	0.006972412	0.070225379
SRSF12	0.092	0.006977202	0.070225379
HSPBP1	0.092	0.006978746	0.070225379
HABP2	0.092	0.006996141	0.070370693
MTSS1L	0.092	0.00700449	0.070422171
OAZ3	0.092	0.007017887	0.070511798
ARSF	0.092	0.007021558	0.070511798

GPR110	0.092	0.007022014	0.070511798
SNHG24	0.092	0.007030683	0.070554221
KRT78	0.092	0.007045512	0.070658367
BMP7	0.092	0.007057788	0.070736955
BTG3	0.092	0.007065595	0.070800148
B3GALT6	0.092	0.00710028	0.071021264
FRRS1L	0.091	0.007126549	0.071164783
RN7SKP20	0.091	0.007136676	0.071212749
HSPB2	0.091	0.007149432	0.071295228
TMEM5-AS1	0.091	0.007164681	0.071413113
LINC01133	0.091	0.007166718	0.071413113
PUSL1	0.091	0.007167323	0.071413113
CTA-293F17	0.091	0.007168751	0.071413113
MLIP	0.091	0.007179382	0.071489115
SDC2	0.091	0.00718885	0.071549942
MEG3	0.091	0.007189998	0.071549942
AC007099	0.091	0.007191785	0.071552782
NPR2	0.091	0.007202171	0.071626183
SLC16A3	0.091	0.007209355	0.071682666
GPR156	0.091	0.007216686	0.071695559
ST3GAL3	0.091	0.007220988	0.071695559
MYL6B	0.091	0.00722119	0.071695559
LAMTOR1	0.091	0.007225645	0.071724845
CRHR1	0.091	0.007257016	0.071875798
CTD-2024P10	0.091	0.007262443	0.071880377
FAM229B	0.091	0.007276949	0.071988394
AC144836	0.091	0.007290969	0.072018961
LIX1	0.091	0.007313907	0.072179749
SPRR3	0.091	0.007335897	0.072320915
NDUFA13	0.091	0.007368022	0.072533439
UG0898H09	0.091	0.007375846	0.072555408
KLHDC8B	0.091	0.007384463	0.072579421
IVL	0.091	0.0073867	0.072579421
C10orf25	0.091	0.007398545	0.072653709
RP11-572P18	0.091	0.007404349	0.07267902
F3	0.091	0.007417845	0.072738252
XG	0.091	0.007423578	0.072764507
HOXA1	0.091	0.007428815	0.072779472
SLC38A4	0.091	0.007428834	0.072779472
KERA	0.091	0.007429689	0.072779472
DDRGK1	0.091	0.007447441	0.072893398
RP11-680C21	0.091	0.007450363	0.072907016
AC006115	0.091	0.007454199	0.07291459
COL4A6	0.091	0.007480796	0.073084689
LOXL4	0.091	0.007485922	0.073119773
OST4	0.091	0.007495263	0.073177997
EPYC	0.091	0.007496492	0.073177997
RP11-483A20	0.091	0.007504235	0.073223563
LINC01405	0.091	0.007508757	0.073252673
MYL7	0.091	0.007553681	0.073550422
FBL	0.091	0.007553692	0.073550422
RPL29P14	0.091	0.007638057	0.074026273

RP11-121L11	0.091	0.007648333	0.074068154
CTC-451P13	0.091	0.007656879	0.074092704
NCLN	0.091	0.007657091	0.074092704
RP11-221N13	0.091	0.007664371	0.07413302
FAM195B	0.091	0.007674773	0.074203497
IL1RAP	0.091	0.007683647	0.074261654
ASIC4	0.091	0.007695523	0.074298535
RP11-423E7	0.091	0.007702093	0.074346899
S100A3	0.091	0.007715324	0.074449792
LINC01186	0.090	0.007780962	0.074898135
HRNR	0.090	0.007782436	0.074898135
RP3-473L9	0.090	0.00778725	0.074901684
TMEM190	0.090	0.007798576	0.074901684
KHDRBS3	0.090	0.007802029	0.074901684
IL4R	0.090	0.00781431	0.074974244
SPINT2	0.090	0.007867748	0.075359354
MIR4290	0.090	0.007869086	0.075359354
ZNF524	0.090	0.007936946	0.075830075
LIPH	0.090	0.007952705	0.075950166
RPS15A	0.090	0.007962928	0.075986853
LINC00162	0.090	0.007977618	0.076050843
RP6-191P20	0.090	0.007983771	0.076094272
RP11-536K7	0.090	0.008006023	0.076260576
RUNX2	0.090	0.008015199	0.076308701
SRM	0.090	0.008019465	0.076312298
S100A1	0.090	0.008022505	0.076325981
RPL24	0.090	0.008025403	0.076327473
RP11-177H13	0.090	0.008028523	0.076327473
AC009166	0.090	0.008056811	0.076511681
PDE4C	0.090	0.008058089	0.076511681
FAM20A	0.090	0.008060828	0.076522432
SNRPB	0.090	0.008090788	0.076655679
MRPS36P5	0.090	0.008101733	0.076704733
MMP15	0.090	0.008176141	0.077262912
KCP	0.090	0.008212693	0.077500675
MIR181A2HG	0.090	0.008278416	0.077948805
SPRR1A	0.090	0.00827982	0.077948805
ARL2	0.090	0.008287967	0.077994663
ASB10	0.090	0.008302212	0.078097854
CREB5	0.090	0.008386167	0.078649892
STEAP3	0.090	0.008387864	0.078649892
COX7A1	0.090	0.008392076	0.078649892
RP11-479J7	0.090	0.008402555	0.078700204
ACVR1	0.090	0.008402598	0.078700204
C19orf81	0.090	0.008409808	0.078722356
GLIPR2	0.090	0.008412356	0.078722356
GREM2	0.090	0.00844353	0.07888053
SNORA73B	0.090	0.008444409	0.07888053
NFIL3	0.090	0.00844922	0.078904118
RP11-490G2	0.090	0.0084648	0.079003137
PLAC9	0.090	0.008472049	0.079039818
FAM89A	0.089	0.008532602	0.079449119

MUC12	0.089	0.008541097	0.079505048
SLITRK3	0.089	0.008544821	0.079505048
RP1-232P20	0.089	0.008576165	0.079667842
AC004125	0.089	0.008580323	0.079675567
TREX2	0.089	0.00858881	0.079723107
DMWD	0.089	0.008598108	0.079747246
GAPDHP24	0.089	0.008601423	0.079762459
HUNK	0.089	0.00860315	0.079762939
GPR78	0.089	0.008605909	0.079772992
NOTCH1	0.089	0.008637329	0.08004866
RP5-1059L7	0.089	0.008646772	0.080077207
COL4A2	0.089	0.008647135	0.080077207
STX10	0.089	0.0086579	0.080110366
ETHE1	0.089	0.008672329	0.080170224
OAF	0.089	0.008685854	0.080248528
LINC01091	0.089	0.008698847	0.080336632
B3GNT5	0.089	0.008703257	0.080346972
ECH1	0.089	0.00870796	0.080374811
NPM3	0.089	0.008711415	0.08038613
TSTA3	0.089	0.008717163	0.08038613
BMP8B	0.089	0.008736227	0.080495318
GMPSP1	0.089	0.008784564	0.080878102
CTBP2P8	0.089	0.008822603	0.081150297
THOC6	0.089	0.008832678	0.081150297
PTPN21	0.089	0.008834271	0.081150297
SAPCD2P3	0.089	0.008836748	0.081150297
CCM2	0.089	0.008842866	0.081150297
OBP2B	0.089	0.008846876	0.081169364
RAB23	0.089	0.008858297	0.081208501
FAM225A	0.089	0.008860821	0.081208501
RNH1	0.089	0.008868982	0.081246974
RP11-95H3	0.089	0.008891466	0.081374681
CYP26A1	0.089	0.008898793	0.081426096
NSDHL	0.089	0.008904503	0.081448484
RBM42	0.089	0.008911281	0.081479798
KRT42P	0.089	0.008912576	0.081479798
ALPK2	0.089	0.008938597	0.081634365
GPAA1	0.089	0.008962155	0.081754564
ZIC4	0.089	0.008972716	0.081788237
LINC00520	0.089	0.008991196	0.081941007
RPS16P9	0.089	0.009078022	0.082560746
MID1	0.089	0.009102215	0.082715358
PRADC1P1	0.089	0.009140502	0.082968302
ANKRD65	0.089	0.009143714	0.082981642
PROM1	0.089	0.009157055	0.083076533
ZNF503-AS2	0.089	0.009172312	0.083123454
ZFP36L1	0.089	0.009175048	0.083123454
JUN	0.089	0.009197509	0.083258711
C21orf2	0.089	0.009198718	0.083258711
HAS2	0.089	0.009208868	0.083334744
CLMP	0.088	0.009284052	0.083728749
RNU6-725P	0.088	0.00928779	0.083744889

SLC22A16	0.088	0.009300881	0.083775982
NDUFAB1	0.088	0.009328572	0.08392367
LY6H	0.088	0.009334187	0.083931476
RP11-477I4	0.088	0.00933516	0.083931476
CTB-40H15	0.088	0.009346973	0.083962334
SDC3	0.088	0.009357984	0.08404539
RND3	0.088	0.009387418	0.084198599
NUAK1	0.088	0.009397919	0.084276915
RAB25	0.088	0.009426938	0.084425867
BOP1	0.088	0.009448709	0.084541357
GYG2	0.088	0.009453618	0.084569396
RP11-1081M5	0.088	0.009471285	0.084607844
AC002454	0.088	0.009473584	0.084607844
GPR143P	0.088	0.009473904	0.084607844
AC005256	0.088	0.009492448	0.084741674
CYTH3	0.088	0.00950038	0.084783578
NTAN1	0.088	0.009507758	0.084814755
AC091801	0.088	0.009511478	0.084832056
ANKFN1	0.088	0.009524337	0.084883157
HAS1	0.088	0.009528719	0.084906323
RP11-279O17	0.088	0.009546015	0.085028624
UCHL3	0.088	0.009550015	0.085032445
ZNF462	0.088	0.009562524	0.085112006
TCEB2	0.088	0.009568724	0.085150991
TPT1P5	0.088	0.009598131	0.085283075
AGT	0.088	0.009599651	0.085283075
RP11-467L19	0.088	0.009614711	0.085384921
PCDHB12	0.088	0.009616743	0.085384921
CATSPERD	0.088	0.009620455	0.085388261
PCDHB16	0.088	0.009640303	0.085516613
RPS19	0.088	0.009676402	0.08570383
MUC1	0.088	0.009677682	0.08570383
AC006145	0.088	0.009720178	0.08598494
ADAMTS5	0.088	0.00977614	0.086351669
YKT6	0.088	0.009781558	0.086383513
AC009060	0.088	0.009796174	0.086496544
GPX1	0.088	0.009814893	0.086632608
MVB12A	0.088	0.009815221	0.086632608
AC007392	0.088	0.009838277	0.086691511
RABAC1	0.088	0.009844788	0.086732842
TMEM41A	0.088	0.009849818	0.086761103
ATP6V0D2	0.088	0.00987828	0.08692988
AC010235	0.088	0.00987993	0.08692988
PERP	0.088	0.009912132	0.087087277
PALLD	0.088	0.009912447	0.087087277
GDNF-AS1	0.088	0.009937739	0.087212952
FZD7	0.088	0.00999896	0.087524438
SLC25A1	0.088	0.010025086	0.087704769
TEX29	0.088	0.010030829	0.08773183
KRT86	0.088	0.010039231	0.08773183
FABP6	0.088	0.010050325	0.087752283
AC084357	0.088	0.010050784	0.087752283

SCN2A	0.088	0.010052912	0.087754771
NEFL	0.088	0.010066158	0.087838202
KCNA1	0.087	0.01008165	0.087925058
PDLIM1	0.087	0.010086448	0.087950794
SPATA20P1	0.087	0.010104675	0.088061364
CH17-13I23	0.087	0.010108322	0.088071358
CDKN1A	0.087	0.01010952	0.088071358
KLK5	0.087	0.010141265	0.088267182
LRRC8A	0.087	0.010193697	0.088561696
OGN	0.087	0.010204609	0.088603506
HSD3B7	0.087	0.010205951	0.088603506
RP11-963H4	0.087	0.010222712	0.088694317
AL158048	0.087	0.010255589	0.08887244
RP11-157D23	0.087	0.010269252	0.088878935
TMEM120A	0.087	0.010269401	0.088878935
PEMT	0.087	0.010280027	0.088922418
ST3GAL6-AS1	0.087	0.010289681	0.088957462
S100A11P2	0.087	0.010309676	0.089106988
HMCN1	0.087	0.010326646	0.089208218
SNX7	0.087	0.010328052	0.089208218
MYO1H	0.087	0.010339173	0.089271896
RP11-167P20	0.087	0.010345782	0.089296586
SLC20A2	0.087	0.010366202	0.08945662
ARMC5	0.087	0.010375767	0.089506729
CGB8	0.087	0.010384247	0.089515029
LEMD1	0.087	0.010451901	0.089902964
RPS4Y1	0.087	0.01045687	0.089913148
DTD1	0.087	0.010459083	0.089913148
RP11-38M8	0.087	0.010468853	0.08991461
UBE2QL1	0.087	0.010469121	0.08991461
MAPK10	0.087	0.010477413	0.089959935
RP11-252K23	0.087	0.010493702	0.090083091
DENND2C	0.087	0.010498757	0.090086346
RGS20	0.087	0.010528477	0.090268184
ZNF205	0.087	0.010564288	0.09039622
MMP3	0.087	0.010573764	0.090461048
ATP5J	0.087	0.010613742	0.090705308
MYL1	0.087	0.010640567	0.090833485
MEG8	0.087	0.010669934	0.090934053
SPTLC3	0.087	0.010680482	0.09094928
TMEM242	0.087	0.010694924	0.0910397
GLIS3-AS1	0.087	0.01070511	0.091110125
FLJ20021	0.087	0.010710142	0.091136668
ARHGEF40	0.087	0.010723233	0.091151611
ATRAID	0.087	0.010761964	0.091398014
C12orf54	0.087	0.010784974	0.091494183
ABCC6	0.087	0.010789751	0.091503469
RP11-118A3	0.087	0.010797506	0.091536639
CTC-260F20	0.087	0.010863797	0.091869684
ACTBL2	0.087	0.01089407	0.092043976
ADK	0.087	0.010934613	0.092271935
HSD17B2	0.087	0.010948665	0.092374146

SERTAD1	0.086	0.010977868	0.092505824
NUPR1L	0.086	0.010990904	0.092566536
NKX1-2	0.086	0.010996587	0.092581657
AC073150	0.086	0.011001406	0.092605862
RP3-416H24	0.086	0.011016588	0.092700888
TENM4	0.086	0.011033229	0.092808127
RP11-435J9	0.086	0.011042638	0.092854466
ADPRHL2	0.086	0.0110449	0.0928571
PRSS27	0.086	0.011067319	0.092956586
NGEF	0.086	0.011070396	0.092956586
ZNF787	0.086	0.0110926	0.09306099
SWI5	0.086	0.011177653	0.093586129
RPL7P16	0.086	0.011180739	0.093586129
CPA5	0.086	0.011190719	0.093620298
B3GAT3	0.086	0.01122278	0.093706604
IL36B	0.086	0.011226667	0.09370706
EDN1	0.086	0.011232392	0.093717877
RP11-109J4	0.086	0.011233866	0.093717877
RP11-166D19	0.086	0.011241782	0.093750965
ZNF579	0.086	0.011243738	0.093750965
GAL3ST1	0.086	0.011259286	0.093814905
C19orf70	0.086	0.011338303	0.094176725
SPRY3	0.086	0.01139117	0.094460355
RP11-625H11	0.086	0.011398051	0.094460355
BEX4	0.086	0.011420429	0.094594917
URGCP-MRPS24	0.086	0.011428111	0.094642088
RP11-541P9	0.086	0.011449272	0.094800849
RP11-185E8	0.086	0.011464845	0.094847346
MMP1	0.086	0.011495009	0.094981398
LINC01443	0.086	0.011544775	0.095296234
ARSJ	0.086	0.011556684	0.09535866
PIGFP1	0.086	0.011578883	0.095517081
PCDHB14	0.086	0.011583513	0.095518923
FXYD5	0.086	0.011587858	0.09553313
RP11-329B9	0.086	0.01159099	0.095542415
C11orf87	0.086	0.011605989	0.095632948
PINX1	0.086	0.011660601	0.095950145
COMMD5	0.086	0.011675646	0.096024176
RP11-359D14	0.086	0.011696616	0.096113654
WDR83OS	0.086	0.011699342	0.09611947
EXT2	0.086	0.011729301	0.096204
SLC25A47	0.086	0.011777275	0.096476827
MPST	0.086	0.011783817	0.096513815
LARP6	0.086	0.011798193	0.096598341
TACSTD2	0.086	0.011864855	0.096998841
ZFP92	0.086	0.011879949	0.09704253
LRRC43	0.085	0.0119573	0.097424558
AP000679	0.085	0.011975321	0.09752883
SNHG19	0.085	0.011978117	0.097534926
RN7SL228P	0.085	0.012007559	0.097707847
OPN4	0.085	0.012043636	0.097865471
TNFSF9	0.085	0.012053099	0.097865471

FAM19A3		0.085	0.012157751	0.098525983
AC016735		0.085	0.012165421	0.09855061
RP11-173C1		0.085	0.012166997	0.09855061
AC006483		0.085	0.012204638	0.09871541
RP11-142C4		0.085	0.012205998	0.09871541
TRPC2		0.085	0.012218052	0.098779349
ATP10A		0.085	0.012235107	0.09888189
ADAMTSL4		0.085	0.01223904	0.09888189
	1-Dec	0.085	0.01226573	0.099047122
DAP		0.085	0.012297612	0.099186849
IFNGR2		0.085	0.0123316	0.099387934
PDZD2		0.085	0.012332977	0.099387934
MBLAC1		0.085	0.012359536	0.099517771
AP000696		0.085	0.012389461	0.099623985
CIB2		0.085	0.012403631	0.099721092
FAM85B		0.085	0.012413976	0.099787416
CDC42EP2		0.085	0.012419772	0.09981716
HOXA13		0.085	0.012442022	0.099913236
LRFN3		0.085	0.012445551	0.099923176
RP11-290M5		0.085	0.012463017	0.100029679
TRIML2		0.085	0.012469457	0.100047648
LINC01082		0.085	0.012472925	0.100058612
AK1		0.085	0.012506759	0.100262479
KLHL4		0.085	0.012573431	0.100593781
RSU1		0.085	0.012580336	0.100632125
NFKBIB		0.085	0.012590931	0.100699965
SFRP4		0.085	0.012611287	0.100824961
HSPA12B		0.085	0.012670412	0.101070812
APELA		0.085	0.012678649	0.101112862
RP4-630A11		0.085	0.012692809	0.101191898
KLK3		0.085	0.012704462	0.101250908
SCIN		0.085	0.012720892	0.101328908
MFSD2A		0.085	0.012734759	0.10139057
FAM162A		0.085	0.012766558	0.101609771
BACE2		0.085	0.012785821	0.101661154
PORCN		0.085	0.012791826	0.101665211
ARHGEF38		0.085	0.012807661	0.1017126
ZIC1		0.085	0.0128249	0.101784955
COPE		0.085	0.012860927	0.102019879
KLF17		0.085	0.01289271	0.10210301
ATP13A4		0.085	0.012892844	0.10210301
C16orf91		0.085	0.012945204	0.102380268
RP11-1152H15		0.085	0.012954638	0.102407233
KDELR1		0.084	0.012996109	0.102715845
LINC00163		0.084	0.013021386	0.102796214
B4GALT1		0.084	0.013060003	0.102964539
PLEKHM2		0.084	0.01306419	0.102980496
PF4V1		0.084	0.013075374	0.103034556
GATA6-AS1		0.084	0.013118018	0.103285148
TMEM204		0.084	0.01313026	0.103326258
SSPN		0.084	0.013131918	0.103326258
RP11-401P9		0.084	0.013153955	0.103394418

KRT82		0.084	0.013158363	0.103394418
IFNWP2		0.084	0.013167111	0.103394418
ACTR1A		0.084	0.013175315	0.103394418
KLK13		0.084	0.013181689	0.103410361
LGALS12		0.084	0.013221226	0.103579606
ST5		0.084	0.013262204	0.103768519
UNC93B6		0.084	0.013273092	0.103819602
RP5-1121A15		0.084	0.013289821	0.103911299
AC005330		0.084	0.013290832	0.103911299
GJA5		0.084	0.013291361	0.103911299
LINC01213		0.084	0.013309638	0.103951117
MSC		0.084	0.013311321	0.103951117
TPTEP1		0.084	0.013315701	0.103951117
PPP1R14C		0.084	0.01333477	0.103977584
PPP4C		0.084	0.01334125	0.10399406
RP11-680A11		0.084	0.013348593	0.104034269
RP11-817I4		0.084	0.013400318	0.104216981
TMEM169		0.084	0.013400483	0.104216981
SYT3		0.084	0.013423117	0.104328826
GSDMC		0.084	0.013452179	0.104413587
LRRC32		0.084	0.013461312	0.104434255
MACC1		0.084	0.01347951	0.104528697
GRAMD2		0.084	0.013484954	0.104540718
OXGR1		0.084	0.013552089	0.10489929
RNU6-44P		0.084	0.013564504	0.104975761
HAUS7		0.084	0.013574474	0.104987247
NCK2		0.084	0.013600001	0.105150528
DMRT1		0.084	0.013607758	0.105176353
DMRTA1		0.084	0.013617104	0.105200941
JPH3		0.084	0.013637148	0.105220341
IRX6		0.084	0.013646588	0.105220341
RP11-547C5		0.084	0.01365354	0.105256901
RP11-59D5__B		0.084	0.013680502	0.105417943
RPS26P4		0.084	0.01368107	0.105417943
RP11-376P6		0.084	0.013703712	0.105558258
SLC25A5		0.084	0.013729851	0.10564456
RP11-152P17		0.084	0.013741993	0.105659764
AC093901		0.084	0.013744712	0.105659764
RP11-134O21		0.084	0.01374573	0.105659764
INHBE		0.084	0.01376407	0.105749532
TNNC2		0.084	0.013768881	0.105769434
	11-Sep	0.084	0.013779585	0.105817527
RP11-247C2		0.084	0.013786236	0.105851531
CCDC9		0.084	0.013822008	0.106074894
P2RX6		0.084	0.013830922	0.106109106
NUTF2		0.084	0.013861174	0.106232691
PCAT6		0.084	0.013863868	0.106232691
C2CD4A		0.084	0.01387729	0.106293611
ZNF812		0.084	0.013914173	0.106507607
AC037459		0.084	0.013924173	0.106532784
SPEG		0.084	0.013926675	0.106534814
RP11-326A19		0.084	0.013933907	0.106573017

NRGN		0.084	0.013954855	0.106664718
BAX		0.084	0.013970543	0.106739939
LAYN		0.084	0.014027067	0.106993449
LINC01354		0.084	0.014034295	0.10701493
PLXDC2		0.084	0.01403665	0.107015129
HES4		0.083	0.014148987	0.107683344
RP1-138O17		0.083	0.014150904	0.107683344
PVRL4		0.083	0.014152356	0.107683344
ARHGEF25		0.083	0.014157887	0.107683344
ACTG2		0.083	0.014177043	0.107757743
CTD-2330J20		0.083	0.014185241	0.107774648
SDF4		0.083	0.014251527	0.108099613
RNU6-1234P		0.083	0.014294843	0.108323475
SLC19A3		0.083	0.014296961	0.108323475
KDM5D		0.083	0.0143175	0.108428731
NHSL1		0.083	0.014335004	0.108490901
MRVI1		0.083	0.014365114	0.108600691
LY6K		0.083	0.014367797	0.108600691
RNU6-373P		0.083	0.014384903	0.108626888
RP11-768F21		0.083	0.014404687	0.108740305
IFITM3		0.083	0.014409055	0.108740305
CTD-2532K18		0.083	0.014422067	0.108786791
ETV5		0.083	0.014440841	0.10887667
MRPS24		0.083	0.01448115	0.109025583
RPL35A		0.083	0.014481194	0.109025583
FTL		0.083	0.014502986	0.109172392
CMB9-22P13		0.083	0.014529029	0.109313954
ARL2BPP2		0.083	0.014533268	0.109313954
PADI1		0.083	0.014558449	0.109468779
CDH10		0.083	0.014561483	0.109474309
EGFEM1P		0.083	0.014565918	0.109490372
MUCL1		0.083	0.014574285	0.109535975
OR11J5P		0.083	0.014587678	0.109589089
AMDHD2		0.083	0.014635925	0.10977656
CORO2B		0.083	0.014636103	0.10977656
ARPC1A		0.083	0.014646585	0.109836706
BBC3		0.083	0.014700684	0.110142132
AP000473		0.083	0.014703858	0.110144799
KLK15		0.083	0.014727221	0.110267783
KRT17P6		0.083	0.01473914	0.110330256
GTF2E2		0.083	0.01477644	0.110532039
ERMN		0.083	0.014791232	0.110607944
RPS3		0.083	0.014810736	0.110719027
MYL4		0.083	0.014857047	0.11099554
NCKAP5-IT1		0.083	0.014882977	0.11113696
	4-Mar	0.083	0.014886729	0.111147556
TCEAL8		0.083	0.014901473	0.111195626
KCNH5		0.083	0.014902722	0.111195626
AP1M1		0.083	0.014971531	0.111518474
RIN1		0.083	0.015004111	0.11167382
BTBD19		0.083	0.015010024	0.111700378
VPS16		0.083	0.01503616	0.11182498

LINC00326	0.083	0.015050236	0.111868005
GALNT5	0.083	0.01505134	0.111868005
FAM181B	0.083	0.015069266	0.11194883
RP11-297K8	0.083	0.015108773	0.112119013
DNAH2	0.083	0.015132737	0.112210318
AGTRAP	0.083	0.015136128	0.112217994
RNF122	0.083	0.015154334	0.112300523
ZBTB18	0.083	0.015157018	0.112302941
SOX21	0.083	0.015165164	0.112322621
UGT1A7	0.083	0.015165436	0.112322621
LINC00626	0.083	0.015170498	0.112332916
FLRT3	0.083	0.015197678	0.112481709
NAPRT	0.083	0.015205223	0.112494106
RNU6-1304P	0.083	0.015228171	0.112602407
XIRP2	0.083	0.015250549	0.112697891
RP11-927P21	0.083	0.015258142	0.112719024
RP11-44N22	0.083	0.015261619	0.112727224
RNU6-860P	0.083	0.015264911	0.112734056
VAX1	0.083	0.015275569	0.112795274
ACO1	0.082	0.015294912	0.112885599
FAM171A2	0.082	0.015320464	0.113039152
AC124861	0.082	0.015374225	0.113312942
TEKT3	0.082	0.015387575	0.113393782
SPECC1	0.082	0.015399299	0.113445074
RPL7P6	0.082	0.015419547	0.113576674
TYRO3	0.082	0.015426402	0.113609594
TMEM160	0.082	0.015428826	0.113609876
RP11-396O20	0.082	0.015445125	0.113631954
RDH10	0.082	0.015447877	0.113631954
PLEKHG2	0.082	0.01544846	0.113631954
RAB32	0.082	0.015450723	0.113631954
EEF1D	0.082	0.015484311	0.113789733
CTB-63M22	0.082	0.015493384	0.113821287
SLC22A1	0.082	0.015524042	0.113993762
BRK1	0.082	0.015537626	0.114013039
SLC39A3	0.082	0.015553532	0.114087181
DLX3	0.082	0.015563813	0.114110232
FGF1	0.082	0.015606515	0.114229519
RP13-614K11	0.082	0.015615512	0.114243041
RP5-857K21	0.082	0.01562048	0.114243952
NUDC	0.082	0.015629176	0.11427887
GRN	0.082	0.015657035	0.114458577
ZBTB47	0.082	0.015662301	0.114479497
GSTA2	0.082	0.015688837	0.114620685
NUTF2P5	0.082	0.015732792	0.11480093
SLC38A8	0.082	0.015746694	0.114856159
MMP27	0.082	0.015750007	0.114856159
NAMPT	0.082	0.015795608	0.114959877
LINC01540	0.082	0.01581932	0.11507355
RP11-93H12	0.082	0.015820813	0.11507355
SLC43A3	0.082	0.015849267	0.115169107
AL139815	0.082	0.015858304	0.115169107

RP11-864N7	0.082	0.015859363	0.115169107
AC113189	0.082	0.015860629	0.115169107
TLCD2	0.082	0.01586445	0.115170924
AMOTL2	0.082	0.015865715	0.115170924
LBX1	0.082	0.015879403	0.115252716
RP11-65J21	0.082	0.0159496	0.115603652
CRISPLD1	0.082	0.01598433	0.115780344
AC004471	0.082	0.015993945	0.115819315
CDC34	0.082	0.016017392	0.115964306
TUBB2B	0.082	0.016037104	0.116009947
RP11-427J23	0.082	0.016041803	0.116024746
RP3-467K16	0.082	0.016049501	0.116045178
RPL37P6	0.082	0.016094287	0.116257062
IL12A-AS1	0.082	0.016095177	0.116257062
SPRR4	0.082	0.016095892	0.116257062
RILPL1	0.082	0.016176388	0.116739519
S100A7	0.082	0.016239938	0.117072842
TEAD4	0.082	0.016246586	0.117096876
CLCA3P	0.082	0.016260425	0.117161156
RP6-91H8	0.082	0.016353979	0.117675018
LINC00629	0.082	0.016368951	0.117695576
GNG12-AS1	0.082	0.016430332	0.117903783
CD24	0.082	0.016443696	0.117928612
DPCD	0.082	0.016457281	0.117990504
IGSF3	0.082	0.016471641	0.118051977
RNA5SP125	0.082	0.016484283	0.118094784
TRIM6	0.082	0.016486703	0.118094784
FBXO17	0.082	0.016526962	0.11832027
LRG1	0.082	0.01655808	0.118499128
APOOP2	0.081	0.016600733	0.118608325
PRL	0.081	0.016617016	0.118689144
BICC1	0.081	0.01666144	0.118863753
RP11-40C11	0.081	0.016716069	0.119027956
HMGB3P7	0.081	0.016718591	0.119027956
PLEKHS1	0.081	0.016748051	0.119161958
CALCA	0.081	0.016748276	0.119161958
IL1RL2	0.081	0.016807049	0.119437378
DDR2	0.081	0.016828289	0.119463237
CTIF	0.081	0.016889502	0.119737471
GMDS	0.081	0.016909041	0.119844711
AC000403	0.081	0.016915684	0.119844711
HOXA10	0.081	0.016918135	0.119844711
MIR30C1	0.081	0.016932831	0.119901867
HIST2H2AC	0.081	0.016946406	0.11998015
TXNRD3	0.081	0.016955391	0.1200217
RP13-401N8	0.081	0.016972444	0.120058981
JAM3	0.081	0.016972665	0.120058981
RP1-20N18	0.081	0.016983035	0.12009666
C1R	0.081	0.01699069	0.120132955
CD151	0.081	0.017022394	0.120339256
RP11-661C8	0.081	0.017041495	0.120456408
MIR5689	0.081	0.01705683	0.120546913

RAET1E	0.081	0.017076862	0.120659515
AC002985	0.081	0.017100716	0.120767469
TF	0.081	0.017124789	0.120883702
ETS2	0.081	0.017163953	0.121016669
LINC00346	0.081	0.017168662	0.121019429
C8B	0.081	0.017169427	0.121019429
MTMR11	0.081	0.017184461	0.121082468
RP11-114F10	0.081	0.017288755	0.121590599
MMP23B	0.081	0.017350447	0.121898474
RP11-2B6	0.081	0.017382286	0.122021619
LAPTM4A	0.081	0.017383347	0.122021619
AC019117	0.081	0.017448153	0.122242306
NID2	0.081	0.017480332	0.122413732
AC107399	0.081	0.01751542	0.12255133
MRAS	0.081	0.017530006	0.122599352
FRMPD4	0.081	0.017613218	0.122946609
RNU6-952P	0.081	0.017629266	0.123022573
NMRK2	0.081	0.017644063	0.123071729
GADD45GIP1	0.081	0.017699185	0.123357271
SESN2	0.081	0.01770226	0.123357271
AC245100	0.081	0.017728278	0.123478315
TMEM11	0.081	0.017760604	0.123649218
RP11-961A15	0.081	0.01776389	0.123654017
STAC2	0.081	0.017826384	0.123901392
CYB5R2	0.081	0.017827924	0.123901392
HOXD13	0.081	0.017828044	0.123901392
ASMTL	0.081	0.017867404	0.123975942
KRT83	0.081	0.017875297	0.123978227
GSTP1	0.081	0.017883234	0.123997663
PMVK	0.081	0.017894219	0.124035504
PYGL	0.081	0.017925958	0.124106032
RNA5SP104	0.081	0.017932184	0.124107132
TIMP3	0.081	0.017947074	0.124143968
RP11-803D5	0.081	0.017948965	0.124143968
SLC2A14	0.080	0.017958241	0.12419009
PCDHB11	0.080	0.017975299	0.124290004
VCAN-AS1	0.080	0.01805683	0.124673912
ACTG1	0.080	0.018059616	0.124673912
IL20RB	0.080	0.018065048	0.124693336
CLEC18B	0.080	0.018075766	0.124749238
PLXDC1	0.080	0.018079664	0.124758067
AP1S1	0.080	0.018139739	0.125009601
TMEM39B	0.080	0.018184667	0.125210517
ALDH1L2	0.080	0.0182829	0.125723313
PCDHB9	0.080	0.018298861	0.125805067
MIR4731	0.080	0.018305206	0.125805067
CLCN3P1	0.080	0.018306882	0.125805067
CD63	0.080	0.018323287	0.12587382
NECAB2	0.080	0.018332537	0.125901045
PFDN2	0.080	0.018364702	0.126049241
KRT5	0.080	0.018395784	0.126208008
LINC00226	0.080	0.018473129	0.126592767

RASD1	0.080	0.018516166	0.126812571
KCNE2	0.080	0.018533153	0.126876311
CTB-113I20	0.080	0.01856347	0.127011322
AC005932	0.080	0.018575995	0.127047417
C9orf69	0.080	0.01857682	0.127047417
SNX12	0.080	0.018613653	0.127166377
KLK6	0.080	0.018619545	0.127175255
IL1R1	0.080	0.018707256	0.127627918
UBE2E2	0.080	0.018728401	0.127735581
ARFRP1	0.080	0.018754654	0.127856865
RCN1	0.080	0.018779389	0.127941103
AC106802	0.080	0.01880006	0.128022654
RAB6B	0.080	0.018827807	0.12812001
GAS2L2	0.080	0.018834876	0.12814475
AC092638	0.080	0.018836824	0.12814475
SERPINB4	0.080	0.01884387	0.128156074
GSG1L	0.080	0.018848977	0.128172507
LINC01545	0.080	0.018856602	0.128204372
GOLM1	0.080	0.018859405	0.128204372
LINC01420	0.080	0.018861739	0.128204372
MUC21	0.080	0.018896746	0.128369034
BAD	0.080	0.018914826	0.128418594
CXADR	0.080	0.018945045	0.128532147
CLCNKB	0.080	0.018958797	0.1285705
DENND2A	0.080	0.018964805	0.128592613
RP11-298E9	0.080	0.018971416	0.128592613
MMP9	0.080	0.018973072	0.128592613
GRB10	0.080	0.019021533	0.128839777
RP11-141O11	0.080	0.019030683	0.128839777
MIR507	0.080	0.019030966	0.128839777
AC113208	0.080	0.019073385	0.129042335
ESYT3	0.080	0.019077988	0.129048049
FERMT1	0.080	0.019100648	0.1291463
HMOX2	0.080	0.019117261	0.129185272
APBA2	0.080	0.019127864	0.129220253
PSCA	0.080	0.019193836	0.129512356
RP4-597N16	0.080	0.019200474	0.129512356
RP11-483E23	0.080	0.019286069	0.129810289
MYO3B	0.080	0.019294572	0.129830824
ANXA2P3	0.080	0.01930025	0.129850685
LAMTOR4	0.080	0.019337284	0.129952985
SLC2A1	0.080	0.019384848	0.130162434
LINC00519	0.080	0.019391489	0.130184407
MALSU1	0.080	0.019413697	0.130245973
RP11-281O15	0.079	0.019456463	0.130459378
RP11-411G2	0.079	0.019466046	0.130462541
RP11-654E17	0.079	0.019466064	0.130462541
JDP2	0.079	0.019473966	0.130462541
SPACA6P	0.079	0.019520707	0.130561718
MTND1P36	0.079	0.019566191	0.130806371
RP3-449M8	0.079	0.019582927	0.130865171
TPPP3	0.079	0.019600855	0.130948231

PRSS3	0.079	0.019637579	0.131083245
IBSP	0.079	0.019730207	0.131450324
TMEM40	0.079	0.019734986	0.13145709
MIR137HG	0.079	0.019778472	0.13159951
ARL4C	0.079	0.019781559	0.131601665
SF3B5	0.079	0.019791439	0.131630622
WTIP	0.079	0.019800744	0.131671738
NGF	0.079	0.019830814	0.131818869
CTD-2600O9	0.079	0.019841125	0.131850604
RP11-69I8	0.079	0.019849989	0.131884572
LAMTOR2	0.079	0.019856885	0.131884572
AKR1C3	0.079	0.019871567	0.13192405
BSPRY	0.079	0.019914126	0.132059328
ZNF561-AS1	0.079	0.019938848	0.132186459
SMAD6	0.079	0.019961407	0.132317594
ABCG8	0.079	0.019975301	0.132387615
EIF5A	0.079	0.019985624	0.132395872
PHLDA2	0.079	0.019998225	0.132429516
GUCA1A	0.079	0.020043048	0.132600397
CTRB2	0.079	0.020099685	0.132938158
RP11-90L1	0.079	0.020116031	0.132990864
CDKN1C	0.079	0.020141883	0.13309295
NKX6-1	0.079	0.020143201	0.13309295
LINC00972	0.079	0.020193478	0.133225837
FAM41AY2	0.079	0.020194134	0.133225837
EYA2	0.079	0.020196329	0.133225837
AQP9	0.079	0.020210382	0.133270311
RP11-815J4	0.079	0.020211465	0.133270311
ANKH	0.079	0.020248153	0.133456793
RBAKDN	0.079	0.020304592	0.133699265
KIAA1024	0.079	0.020331936	0.133805324
AC093875	0.079	0.020356185	0.133890902
GYPA	0.079	0.020366028	0.133918654
B3GALTL	0.079	0.020422186	0.134190406
GTF3C6	0.079	0.020427547	0.134193488
ILVBL	0.079	0.020430765	0.134196116
CYP26C1	0.079	0.020540694	0.134713803
PLEKHN1	0.079	0.020559082	0.134790045
EPPIN	0.079	0.02056667	0.134790045
RN7SKP197	0.079	0.020569127	0.134790045
ZIC5	0.079	0.020613118	0.134965762
PSMG3	0.079	0.020621236	0.135000357
RHBDD2	0.079	0.020638182	0.135092731
AC026877	0.079	0.020751003	0.135506235
ASZ1	0.079	0.020752165	0.135506235
SLURP1	0.079	0.020809699	0.135693253
NRG4	0.079	0.02082482	0.135742339
SOD2P1	0.079	0.020825777	0.135742339
FAM50A	0.079	0.020850432	0.135766657
CTC-332L22	0.079	0.020890793	0.135942861
SOWAHB	0.079	0.020907818	0.1359573
FDX1L	0.079	0.020914902	0.1359573

RNVU1-7	0.079	0.020915508	0.1359573
PEBP4	0.079	0.020994602	0.136338955
WNT2	0.079	0.021007338	0.136403065
TMEM233	0.078	0.021058277	0.136618417
AC100800	0.078	0.021060584	0.136618417
LSM7	0.078	0.021135148	0.136990157
HRASLS	0.078	0.02114193	0.136996831
GPR123	0.078	0.021183773	0.137174654
SPACA3	0.078	0.021216119	0.137328094
AMOTL1	0.078	0.021264836	0.137587331
MRPL51	0.078	0.021287645	0.137697496
RPS9	0.078	0.021305469	0.137794072
ATP5O	0.078	0.021339298	0.137956665
ISY1	0.078	0.021374693	0.138021578
LINC01014	0.078	0.021376768	0.138021578
FADS3	0.078	0.021378319	0.138021578
RP11-30P6	0.078	0.021409709	0.138149331
PPP1R3A	0.078	0.02141474	0.138163074
MT1L	0.078	0.021440818	0.138275133
CHST14	0.078	0.02145182	0.138289909
NLRP2	0.078	0.021461428	0.1383144
RP11-179B15	0.078	0.021471331	0.138333904
RNASE7	0.078	0.021473168	0.138333904
RP5-1177I5	0.078	0.021510261	0.138535387
DTNB	0.078	0.02152417	0.138562267
FUT6	0.078	0.021549017	0.138627822
RP11-383H13	0.078	0.021549935	0.138627822
SLC39A7	0.078	0.021550809	0.138627822
FRMD6	0.078	0.021604268	0.138840436
LPAR1	0.078	0.021640733	0.139018506
AC112721	0.078	0.021649221	0.139033439
PCDHB6	0.078	0.021657654	0.139033439
RP11-651H2	0.078	0.021682129	0.139134301
GHRHR	0.078	0.02170939	0.139235508
RP11-432I5	0.078	0.021709595	0.139235508
EHHADH	0.078	0.021741391	0.139369809
CCDC92	0.078	0.02174224	0.139369809
LINC01343	0.078	0.021769883	0.139490679
DECR2	0.078	0.021853731	0.139914996
C18orf63	0.078	0.021866631	0.13995324
PTGER1	0.078	0.021901187	0.140064028
ADCY2	0.078	0.021903822	0.140064028
RP11-1008C21	0.078	0.021903917	0.140064028
C1QL2	0.078	0.021935805	0.140124084
LINC00543	0.078	0.021954336	0.140128851
FRMD5	0.078	0.021992101	0.14028687
RP11-553A21	0.078	0.02199429	0.14028687
AC091057	0.078	0.02200069	0.140290111
MIR199A2	0.078	0.022070961	0.14062524
AADAC	0.078	0.022137621	0.140974528
SLC1A1	0.078	0.02215686	0.141078176
SLC39A1	0.078	0.022174834	0.141154881

RP11-575F12	0.078	0.022216842	0.141365604
B3GNT9	0.078	0.022237074	0.141450079
OPN3	0.078	0.02226557	0.141548369
NXF3	0.078	0.022267746	0.141548369
STX8	0.078	0.022272314	0.141548369
RP11-753H16	0.078	0.022309133	0.141706732
PDZRN3	0.078	0.022347608	0.141837621
PADI3	0.078	0.022353132	0.141853783
NHS-AS1	0.078	0.02236316	0.141889691
RP11-432J24	0.078	0.022384202	0.141918581
RP11-138H8	0.078	0.022403562	0.141965735
EFS	0.078	0.022481725	0.142423134
SNRPGP20	0.078	0.022509293	0.142521952
C19orf47	0.078	0.022516523	0.142529827
PDZD3	0.078	0.022563883	0.14263145
GSTM5	0.078	0.022565263	0.14263145
AC008992	0.078	0.022575676	0.142657782
MIB2	0.078	0.022601964	0.142710443
KLK14	0.078	0.022601988	0.142710443
DIO3	0.078	0.022616963	0.142767127
ALG1L12P	0.077	0.022722774	0.14313154
ROR1-AS1	0.077	0.022776868	0.143226023
COL11A2	0.077	0.022811765	0.143388669
TMEM187	0.077	0.022833875	0.143468774
RN7SKP224	0.077	0.022836559	0.143468774
RNF217-AS1	0.077	0.022862795	0.143557855
RP11-118K6	0.077	0.02291029	0.143818158
C19orf68	0.077	0.022938178	0.143936675
AC124789	0.077	0.022941258	0.143936675
ITGB1BP2	0.077	0.023002702	0.1442082
DEFA3	0.077	0.023037417	0.144368824
RP11-57A1	0.077	0.023045292	0.144381324
RP11-367F23	0.077	0.023069529	0.144497785
AP000797	0.077	0.02314541	0.144816927
TRIM15	0.077	0.02319021	0.145021027
IDH3B	0.077	0.023205804	0.145061403
BNIP3P19	0.077	0.023241899	0.145212815
UBXN6	0.077	0.023244537	0.145212815
SNX21	0.077	0.023294577	0.145387343
CCDC23	0.077	0.023334609	0.145501354
TPI1	0.077	0.0234005	0.145742858
AKT3	0.077	0.023415791	0.145819022
SYNPO	0.077	0.023458523	0.146008765
DRAXIN	0.077	0.023469443	0.146038565
MIR3663HG	0.077	0.023476733	0.14604576
NUMBL	0.077	0.023487631	0.146056355
AC093620	0.077	0.023522425	0.146234503
CLDN18	0.077	0.023568216	0.146461827
MEIS1-AS2	0.077	0.023608847	0.146637933
AGXT	0.077	0.02362843	0.146721149
NT5E	0.077	0.023659352	0.146855721
SHB	0.077	0.023686271	0.146946204

CH25H	0.077	0.023777653	0.147321227
ATP6V1G3	0.077	0.023844264	0.147591128
C4B	0.077	0.023856316	0.147591128
F13A1	0.077	0.023856418	0.147591128
OR7E90P	0.077	0.023861501	0.147591128
QSOX1	0.077	0.023913881	0.147857513
SSC4D	0.077	0.023939307	0.14792565
WDR86	0.077	0.023956886	0.147980274
SBSPON	0.077	0.023978722	0.148065394
EFNB3	0.077	0.023989269	0.148092925
C15orf54	0.077	0.024016725	0.148138189
TMEFF1	0.077	0.024122314	0.148433264
MAP4K4	0.077	0.024154441	0.148573365
AC004870	0.077	0.02417795	0.148678491
RP5-858L17	0.077	0.024187141	0.148678491
MCEMP1	0.077	0.024207874	0.148717122
ADAMTSL4-AS1	0.077	0.024209038	0.148717122
ZNF532	0.077	0.024221676	0.148756385
RP11-396F22	0.077	0.024251774	0.148883633
AC012462	0.077	0.024257545	0.148888791
CYSTM1	0.077	0.024261147	0.148888791
DGCR6	0.077	0.024264987	0.148888791
RBMS3-AS2	0.077	0.024281118	0.148908914
TRHDE	0.077	0.02428173	0.148908914
KCNE5	0.077	0.024284031	0.148908914
WT1-AS	0.077	0.024296386	0.14893189
RP11-271M24	0.077	0.024297159	0.14893189
RAP2B	0.077	0.024303237	0.148949976
RP11-346C4	0.077	0.024330283	0.149058193
DSEL	0.077	0.024364407	0.149175982
MPG	0.077	0.024405713	0.149328225
RPL8	0.076	0.024578399	0.150076333
ECHDC3	0.076	0.024633087	0.150231782
CD9	0.076	0.024635401	0.150231782
PRSS12	0.076	0.024645742	0.150275603
FAM81B	0.076	0.024690461	0.150487814
LINC00242	0.076	0.024700594	0.150501671
RP11-676B18	0.076	0.024728192	0.150643336
C1orf123	0.076	0.02475207	0.150711688
TMEM91	0.076	0.024757771	0.15071383
PON2	0.076	0.024836937	0.15101615
MIR511	0.076	0.024847539	0.151027041
C5orf66-AS1	0.076	0.024938951	0.151327228
TPRN	0.076	0.024959641	0.151433482
CLDN9	0.076	0.02505668	0.151813956
CDC37	0.076	0.0250739	0.151875189
FBXL15	0.076	0.025114533	0.15192576
ENAH	0.076	0.02512462	0.15192576
PNLDC1	0.076	0.025193745	0.152133261
PUF60	0.076	0.025252525	0.152395085
RP11-302B13	0.076	0.025272493	0.152492402
COPS6	0.076	0.02529452	0.152552084

GAD1	0.076	0.025331254	0.152657429
RP11-539E19	0.076	0.02543774	0.153105427
PSG4	0.076	0.025508582	0.153415483
RPL18	0.076	0.025544576	0.153535023
WNT3A	0.076	0.025551485	0.153557172
RP11-6F2	0.076	0.025559629	0.153574673
CDCP1	0.076	0.025567295	0.153574673
ZC4H2	0.076	0.025645173	0.153925979
HIF1A-AS2	0.076	0.025649404	0.153931974
UFSP1	0.076	0.025657985	0.153961012
DNAJB5	0.076	0.025667679	0.153983442
TRIM16	0.076	0.025692092	0.154052281
AC003092	0.076	0.025735097	0.154182683
NDUFB7	0.076	0.02575212	0.154218057
RP3-326L13	0.076	0.025807795	0.15445437
RP11-4C20	0.076	0.025811742	0.15445596
RPL28	0.076	0.025817889	0.15445596
PHKG1	0.076	0.025824276	0.15445596
PVRL1	0.076	0.025842352	0.154502471
CITED4	0.076	0.025853129	0.154512108
PITX1	0.076	0.02588113	0.15462491
AC113617	0.076	0.025881743	0.15462491
MIR3921	0.076	0.026012813	0.15521326
GPRIN2	0.076	0.026019939	0.155236329
DDX49	0.076	0.026026171	0.155254063
DYNC1I1	0.076	0.026055071	0.155387539
RP11-619A14	0.076	0.026075493	0.155450932
LRRN4CL	0.076	0.026130394	0.155700271
ATP5D	0.076	0.026138176	0.15572716
JUNB	0.076	0.026142013	0.1557293
AC008427	0.076	0.026171576	0.1557621
ZFPM2-AS1	0.076	0.026172283	0.1557621
IRF6	0.076	0.026183176	0.1557621
CA5BP1	0.076	0.026206126	0.155875133
RN7SL675P	0.076	0.026299181	0.156158972
RPL15P3	0.076	0.026314717	0.156212269
NT5DC2	0.076	0.026343931	0.156327236
RP11-453D21	0.076	0.02636586	0.156395023
LINC00160	0.076	0.026367405	0.156395023
TIMM8B	0.076	0.026368489	0.156395023
AL513318	0.076	0.026450558	0.156770498
RN7SL689P	0.076	0.026457319	0.156785209
NUDT1	0.076	0.026464123	0.156806021
RP11-371I20	0.075	0.026477683	0.156847338
CHCHD10	0.075	0.026514797	0.156930563
RNU7-102P	0.075	0.026544494	0.157008771
SERPINB3	0.075	0.026580044	0.157160488
AC021749	0.075	0.026645724	0.157366702
PFKP	0.075	0.026677794	0.157475022
RP11-395L14	0.075	0.02680631	0.157940857
AC144831	0.075	0.026851431	0.158132037
RN7SL420P	0.075	0.026853333	0.158132037

GLB1L2	0.075	0.026870616	0.158191203
AJUBA	0.075	0.026892119	0.158277306
C8orf82	0.075	0.026894123	0.158277306
AFF2	0.075	0.026908795	0.158333252
MESP2	0.075	0.026938122	0.158463363
LUCAT1	0.075	0.026940136	0.158463363
CPA4	0.075	0.027017574	0.15884039
LTBP3	0.075	0.027042675	0.15896166
KRTAP19-3	0.075	0.027069961	0.159029292
RP4-794I6	0.075	0.027154438	0.159291097
SPTB	0.075	0.027211213	0.159467018
GATA6	0.075	0.02725042	0.159618223
SPRED3	0.075	0.027304381	0.159739183
RNU6-733P	0.075	0.027312959	0.159746643
CTD-2566J3	0.075	0.027318113	0.159746643
RP4-800M22	0.075	0.027320556	0.159746643
PTPN14	0.075	0.027322656	0.159746643
EIF1AY	0.075	0.027397808	0.160068133
CTA-941F9	0.075	0.027432877	0.160128905
RP11-71N10	0.075	0.027433226	0.160128905
ZNF668	0.075	0.027445186	0.160128905
LA16c-335H7	0.075	0.027448556	0.160128905
TMEM43	0.075	0.027469741	0.160177678
FANCD2OS	0.075	0.027475426	0.160177678
RP11-315D16	0.075	0.027489431	0.160202609
FASTK	0.075	0.027491463	0.160202609
SDSL	0.075	0.027501213	0.160239815
ANO7P1	0.075	0.027539622	0.160345912
POLR2J	0.075	0.027609578	0.16069429
UPK1B	0.075	0.027667438	0.160932715
GAST	0.075	0.027682016	0.160949798
ECE2	0.075	0.027689843	0.160949798
RPL7P15	0.075	0.027698389	0.160949798
RP4-564O4	0.075	0.027700789	0.160949798
AMTN	0.075	0.027717771	0.160984695
RP1-122P22	0.075	0.02773865	0.161071527
PARL	0.075	0.027752125	0.161130131
ZFHX4	0.075	0.027837731	0.161528708
EMD	0.075	0.027935516	0.16187916
ESRRA	0.075	0.027994008	0.162020974
MTMR2	0.075	0.028016087	0.162129059
PLXNA1	0.075	0.028039826	0.162204571
PCED1CP	0.075	0.028065698	0.162240836
FAM89B	0.075	0.028069467	0.162240836
BARHL2	0.075	0.028076862	0.162244199
RP11-543P15	0.075	0.028087044	0.162244937
FAM160B2	0.075	0.028088889	0.162244937
SURF4	0.075	0.028102589	0.162274735
LCE3D	0.075	0.028115529	0.16231226
PRPH2	0.075	0.028115942	0.16231226
PANO1	0.075	0.028126392	0.16231226
IKBIP	0.075	0.028128121	0.16231226

DEFB135	0.075	0.028161506	0.162457367
RASL10B	0.075	0.028275494	0.162995645
CYP1B1-AS1	0.075	0.028289668	0.163038724
TIMP1	0.075	0.028306218	0.163055182
ABHD17AP1	0.075	0.028312048	0.163069042
S100A12	0.075	0.028321938	0.163106282
CDKL5	0.075	0.028335033	0.163161964
PCDHGC5	0.075	0.028384164	0.163326395
MIR3156-1	0.075	0.028432967	0.163488124
LINC01370	0.075	0.028468677	0.16361502
SGCE	0.074	0.028578647	0.164088637
NPY	0.074	0.028585452	0.164107919
SLC35B4	0.074	0.028602996	0.164144835
PVRL3	0.074	0.028614679	0.164165451
LINC01396	0.074	0.028625485	0.16419481
CROCC	0.074	0.02863626	0.16419481
RP11-742B18	0.074	0.02867423	0.164293471
CDC20P1	0.074	0.02867718	0.164293471
PBDC1	0.074	0.028705367	0.164400014
MRPL47	0.074	0.028723726	0.164485382
OR2AT2P	0.074	0.0287329	0.164515713
RPL22L1	0.074	0.028779747	0.164645685
IGFL4	0.074	0.028784868	0.164645685
C12orf75	0.074	0.028787042	0.164645685
EEF1GP8	0.074	0.028800265	0.164666292
SEC14L2	0.074	0.028809176	0.164677704
ZNRF1	0.074	0.028836744	0.164756192
TPGS1	0.074	0.028900153	0.165019428
RP11-108K14	0.074	0.028903736	0.165019428
ACTN2	0.074	0.029061563	0.165682913
SORCS3	0.074	0.029065674	0.165686518
RIT1	0.074	0.029092109	0.165779284
CRNN	0.074	0.029099352	0.165779284
SEC13	0.074	0.029144006	0.16592844
RP11-763K15	0.074	0.02914513	0.16592844
ADAM15	0.074	0.029186124	0.166055595
TOM1	0.074	0.029186205	0.166055595
YIF1A	0.074	0.029226142	0.166203399
AC008045	0.074	0.029312698	0.166536545
C11orf96	0.074	0.0294726	0.167265434
MIER2	0.074	0.029486756	0.167325833
IGSF9	0.074	0.029592084	0.167758127
IQSEC3	0.074	0.029614872	0.16777299
RPL32P33	0.074	0.02965995	0.167928491
TSPY1	0.074	0.029681422	0.16801011
AC016772	0.074	0.029701113	0.168021723
SUMO3	0.074	0.029716721	0.168070094
CTSLP4	0.074	0.029731961	0.16810107
RP5-882O7	0.074	0.029732817	0.16810107
FOXC2-AS1	0.074	0.029736316	0.16810107
CD8BP	0.074	0.0297693	0.168248901
TMEM130	0.074	0.029769532	0.168248901

AP000647	0.074	0.029781661	0.168297484
RP11-745C15	0.074	0.029811214	0.168404543
CNRIP1	0.074	0.029824365	0.168450312
SMARCE1P4	0.074	0.029858003	0.1685609
MIR3129	0.074	0.029880509	0.168615997
RNU1-139P	0.074	0.029932211	0.168727829
ZPBP	0.074	0.029945227	0.168776001
RP11-259O2	0.074	0.029990926	0.168934036
KRT71	0.074	0.030004297	0.168961537
RN7SL310P	0.074	0.030005599	0.168961537
PCDHB5	0.074	0.030027355	0.169044072
RP11-766F14	0.074	0.030031598	0.169047973
RPL7P7	0.074	0.030074607	0.169230069
AC087762	0.074	0.030080108	0.169230937
C1QTNF4	0.074	0.030094586	0.169249471
PXN	0.074	0.030142386	0.169391316
MIR99B	0.074	0.030155911	0.169447331
EPHA2	0.074	0.030169335	0.169502761
RP11-313E19	0.074	0.030188799	0.169560962
RPL18A	0.074	0.030190374	0.169560962
RP11-440G5	0.074	0.030261261	0.169785928
LRFN1	0.074	0.030261476	0.169785928
GLRX3	0.074	0.030334692	0.170050654
EPHB3	0.074	0.030357047	0.170140589
CTD-2538C1	0.074	0.030401673	0.170271462
MESP1	0.074	0.030446567	0.170425048
UGT1A3	0.074	0.030464014	0.170474632
RPS26P3	0.074	0.030476933	0.170526892
COMMD6	0.074	0.030488148	0.1705696
LCN15	0.074	0.030586468	0.171059381
CCDC80	0.074	0.030591891	0.171069623
CAPG	0.074	0.030678024	0.171309914
RPL10AP6	0.074	0.030727718	0.17148688
C1orf233	0.073	0.030772472	0.171693272
RP11-57C13	0.073	0.030775515	0.171693272
SRI	0.073	0.030789497	0.171751158
AHSG	0.073	0.030824647	0.171879681
C1RL	0.073	0.030904512	0.172162651
RHCE	0.073	0.030919512	0.172194058
RP11-545E8	0.073	0.030929408	0.172198797
LRIG3	0.073	0.030963737	0.172288453
TKT	0.073	0.030971035	0.172293078
OR14K1	0.073	0.031010166	0.172436799
AC110810	0.073	0.031018767	0.172444356
TSPAN10	0.073	0.031057098	0.172574572
VTA1P1	0.073	0.031095892	0.17269775
TBX18	0.073	0.031098536	0.17269775
RP11-115C21	0.073	0.031104233	0.17269775
KRT23	0.073	0.031129345	0.172817033
NDST3	0.073	0.031176837	0.172974982
AC241585	0.073	0.031183219	0.172974982
SLC24A3	0.073	0.031198644	0.17302024

GOLIM4	0.073	0.031207192	0.173027351
RP11-689K5	0.073	0.031247017	0.17317804
TLDC1	0.073	0.031250952	0.17317804
RP11-114J13	0.073	0.031263559	0.17317804
ALG3	0.073	0.031307988	0.173323902
PWWP2B	0.073	0.031321732	0.173340426
EGFLAM	0.073	0.031329922	0.173340426
MIR1245A	0.073	0.03135427	0.173418889
CSPG5	0.073	0.031365198	0.173418889
TIMM13	0.073	0.031404742	0.173577075
AC079922	0.073	0.0314139	0.173587403
TUBB3	0.073	0.031447485	0.173655059
MRPL41	0.073	0.031453237	0.173655059
RP11-320G24	0.073	0.031496974	0.173784223
LINC00961	0.073	0.031561999	0.173976797
SLC25A2	0.073	0.031563654	0.173976797
AC090095	0.073	0.031563703	0.173976797
CTB-1144G6	0.073	0.031605807	0.174094363
CALN1	0.073	0.031676849	0.17425194
SCN2B	0.073	0.03169817	0.174344602
KRT14	0.073	0.031720953	0.174344602
C1orf106	0.073	0.031722979	0.174344602
LINC00565	0.073	0.031740657	0.174417409
RP11-54A9	0.073	0.031763766	0.174468101
TPRXL	0.073	0.031801698	0.174595902
GRM6	0.073	0.031821122	0.174622028
SIAE	0.073	0.031827078	0.174634592
COX7B	0.073	0.03183136	0.174637967
PCDHB8	0.073	0.03184726	0.17464601
TBC1D8B	0.073	0.031878493	0.174752035
TIGD5	0.073	0.03193607	0.17491021
REN	0.073	0.032013365	0.175252935
PDE10A	0.073	0.032041513	0.175363149
GLB1	0.073	0.032060413	0.175380913
LINC00327	0.073	0.032073296	0.175396992
RP11-358M14	0.073	0.032117894	0.175562831
PHYHIP	0.073	0.032122013	0.175565196
NPR3	0.073	0.032137649	0.175610351
RP13-188A5	0.073	0.032193375	0.175733398
CTU2	0.073	0.032247638	0.175948935
RPL13AP25	0.073	0.032292909	0.176051731
AC090617	0.073	0.032294829	0.176051731
INTS4P1	0.073	0.032391596	0.176370702
PPFIBP1	0.073	0.032408321	0.176377996
KRT4	0.073	0.03241061	0.176377996
CTD-2288F12	0.073	0.032502746	0.176758612
STUB1	0.073	0.032633955	0.177227045
RN7SKP202	0.073	0.032648749	0.177244679
HSPB1	0.073	0.03265628	0.177264718
MRPL4	0.073	0.032666464	0.177299793
MECR	0.073	0.032681835	0.177354349
ERICH5	0.073	0.032705668	0.17745191

GOLT1A	0.073	0.032716911	0.177456245
MT1M	0.073	0.032729014	0.177456245
HECTD3	0.073	0.032732549	0.177456245
CHSY1	0.073	0.03273792	0.17746516
RP11-335I12	0.073	0.032749024	0.177505152
MICAL2	0.073	0.032795835	0.177637576
NFKBIL1	0.073	0.032830186	0.177709182
NUBP2	0.073	0.032831442	0.177709182
SLC6A2	0.073	0.03285532	0.177798016
HLX	0.073	0.03288541	0.17789015
TMEM159	0.073	0.032893588	0.17789015
SAMD1	0.073	0.032920561	0.177908492
ANXA8L1	0.073	0.032944693	0.177978322
LRRC4	0.073	0.032957769	0.178028772
VGLL1	0.073	0.03297409	0.178096733
FSCN1P1	0.073	0.033008567	0.17820587
RRAGA	0.073	0.033055709	0.1783604
RP11-546K22	0.073	0.033056612	0.1783604
LINC00853	0.073	0.033072185	0.178424211
OR1C1	0.073	0.033106784	0.17852734
SURF6	0.072	0.033111799	0.17852734
RANBP3L	0.072	0.033116288	0.17852734
KDELR2	0.072	0.033138699	0.178580767
ANKRD53	0.072	0.033172066	0.178703216
ST3GAL2	0.072	0.033172678	0.178703216
CDH17	0.072	0.033208696	0.178836563
NEFH	0.072	0.033292043	0.179088778
ITIH5	0.072	0.033406174	0.179450509
RP11-197K6	0.072	0.033425407	0.179475971
SHFM1P1	0.072	0.033436652	0.17951611
MEDAG	0.072	0.033444626	0.179518448
USP26	0.072	0.03345437	0.179550515
LRPAP1	0.072	0.03346642	0.17959495
TEDDM2P	0.072	0.033491299	0.179694004
RP11-373D23	0.072	0.033521323	0.179740801
SYT16	0.072	0.033592103	0.179960113
LINC01023	0.072	0.033594922	0.179960113
AC092765	0.072	0.033607944	0.179963728
TCEAL7	0.072	0.033633193	0.180023387
GJB6	0.072	0.033653239	0.180090206
SLC17A4	0.072	0.033687539	0.180207911
AC073508	0.072	0.033700262	0.180218238
TMEM9	0.072	0.033738722	0.180365263
KRTAP20-4	0.072	0.03376182	0.180468483
GRIN2A	0.072	0.03378492	0.180501872
RP11-213L8	0.072	0.033806051	0.180583312
NFIA-AS2	0.072	0.033840651	0.180707329
TIMM22	0.072	0.03386784	0.180771448
CTNNAL1	0.072	0.033873738	0.180782668
JRKL	0.072	0.033897062	0.180870792
BSG	0.072	0.033920353	0.180964904
GPC2	0.072	0.033926905	0.180964904

SLC25A37	0.072	0.033958247	0.181030832
RBMXP4	0.072	0.034009023	0.181220376
RP5-1070A16	0.072	0.034014026	0.18122676
RNA5SP133	0.072	0.034035904	0.181258453
YTHDF3-AS1	0.072	0.034039003	0.181258453
SNX3	0.072	0.034047888	0.181260033
HOXC13	0.072	0.034055758	0.181260033
GNAI1	0.072	0.034063489	0.181267237
HYAL2	0.072	0.034068906	0.181275812
FKBP1C	0.072	0.034081689	0.181279578
MTRNR2L6	0.072	0.03409735	0.181285387
MRPS12	0.072	0.034113154	0.181349166
YBX1	0.072	0.034119969	0.181365152
CLIC4	0.072	0.034127017	0.181382374
RP3-460G2	0.072	0.034146451	0.181439945
FTH1P20	0.072	0.034201843	0.181577434
EIF1P5	0.072	0.034231267	0.181693143
RN7SKP42	0.072	0.034253381	0.181770002
TSNAX-DISC1	0.072	0.034360722	0.182217803
CD177P1	0.072	0.034367607	0.182234027
PCBP3	0.072	0.034390919	0.182296749
TMEM105	0.072	0.034396355	0.182305275
STYXL1	0.072	0.034424869	0.182415805
RGS4	0.072	0.034513313	0.182721825
MEX3D	0.072	0.034534481	0.182752639
GPR124	0.072	0.03454015	0.18276233
ZIM2-AS1	0.072	0.034562876	0.182860079
NUTF2P3	0.072	0.034570389	0.182861385
UBL4A	0.072	0.034580182	0.182892875
SLC25A6	0.072	0.034631569	0.183047035
COX6B1P1	0.072	0.034636233	0.183047035
RP11-369F10	0.072	0.0346404	0.183048744
CDSN	0.072	0.03465497	0.183105422
RP11-339B21	0.072	0.03466685	0.18312756
CEBPB	0.072	0.034700798	0.183245915
SLFN12	0.072	0.034720559	0.183309618
ACTN4	0.072	0.034736328	0.183352219
KRT76	0.072	0.034763223	0.183416963
RP11-419J16	0.072	0.034767424	0.183416963
CPPED1	0.072	0.034810876	0.183521635
HYI-AS1	0.072	0.034825486	0.183521635
AC009229	0.072	0.034826652	0.183521635
EPS8L1	0.072	0.034830077	0.183521635
FAM171A1	0.072	0.034860099	0.183634252
VEGFC	0.072	0.034902432	0.183780889
ANXA13	0.072	0.034991729	0.184165739
RP11-284H19	0.072	0.034994855	0.184165739
LPCAT1	0.072	0.035038516	0.184334408
TMC5	0.072	0.035047718	0.184362455
PTGR1	0.072	0.035051656	0.184362806
ALDH1A3	0.072	0.035069783	0.184412087
RN7SL408P	0.072	0.035109285	0.18448256

COX6B1	0.072	0.035181238	0.184714743
PROC	0.072	0.035184855	0.184714743
PRPF31	0.072	0.035188378	0.184714743
OPN1SW	0.072	0.035223237	0.184836611
HNRNPA1P25	0.072	0.035294142	0.185067339
KRBOX1-AS1	0.072	0.035294405	0.185067339
RPS10	0.072	0.035337256	0.185108613
RP11-574H6	0.072	0.035400525	0.185349667
ITPRIPL2	0.072	0.035423432	0.185429398
OR5B3	0.072	0.035428077	0.185429398
RP1L1	0.072	0.035429885	0.185429398
ENDOG	0.072	0.035484658	0.185608744
LYSMD4	0.072	0.035518087	0.185688248
FIBCD1	0.072	0.035532551	0.185723094
TRIM17	0.072	0.035543813	0.185761569
CDKN2A	0.072	0.035596706	0.185907009
RP11-428J1	0.072	0.035598965	0.185907009
OR13Z3P	0.072	0.035612419	0.18595688
RAB43	0.071	0.035640185	0.186027066
VANGL2	0.071	0.035641484	0.186027066
FNTB	0.071	0.035729883	0.186386324
OR7E161P	0.071	0.035734174	0.186388296
ALDH16A1	0.071	0.035741603	0.186401216
NAA38	0.071	0.035759359	0.186437982
SHH	0.071	0.035783314	0.186500326
RNU6-927P	0.071	0.035855235	0.186774281
SEL1L2	0.071	0.035934394	0.187063872
PURG	0.071	0.035939746	0.187071104
SCAND1	0.071	0.035987029	0.187203151
SRA1	0.071	0.036006975	0.187216616
ROMO1	0.071	0.036039705	0.187305003
ERGIC3	0.071	0.036093372	0.18744074
S100A13	0.071	0.036156897	0.187647873
AC093865	0.071	0.036222805	0.187867375
ENPEP	0.071	0.036233985	0.187904616
TMEM37	0.071	0.036240476	0.187917817
PSMB6	0.071	0.036278077	0.188051366
RP11-499O7	0.071	0.036290576	0.188095686
RP11-36B6	0.071	0.036328281	0.188240546
FAM189A2	0.071	0.036343884	0.188247742
HSD17B14	0.071	0.036350979	0.188247742
S100A6	0.071	0.036408862	0.188421697
EIF2B3	0.071	0.036428468	0.188461726
AC098650	0.071	0.036462824	0.188578011
C11orf74	0.071	0.036547619	0.188929825
RP11-235G24	0.071	0.036553782	0.188929825
RP11-649A16	0.071	0.036573654	0.188966525
CTC-526N19	0.071	0.036594201	0.188970668
RPL23AP82	0.071	0.036622361	0.189024096
LSAMP	0.071	0.036695802	0.189299618
EIF2S2P2	0.071	0.036705692	0.189299618
RPS3AP25	0.071	0.03672181	0.189355748

RP11-26O3	0.071	0.036737139	0.189359278
TMEM147	0.071	0.036758944	0.189375305
PTCHD4	0.071	0.036774555	0.189378279
AC090616	0.071	0.036777713	0.189378279
FAM47C	0.071	0.036878886	0.189745037
TMEM79	0.071	0.036879707	0.189745037
NKD1	0.071	0.036914896	0.189864551
P4HB	0.071	0.036979003	0.190021663
TTTY10	0.071	0.03698933	0.190021663
MVD	0.071	0.037005825	0.190085896
RP11-510P12	0.071	0.037029932	0.190168143
MAP1S	0.071	0.03712942	0.190503944
RP11-442J21	0.071	0.037253204	0.190862559
PHYHIPL	0.071	0.037286099	0.190915276
IGHD2OR15-2A	0.071	0.037289818	0.190915276
RP11-667F14	0.071	0.037291553	0.190915276
SIX3	0.071	0.037362266	0.191133473
LAGE3	0.071	0.03748146	0.191584198
MIR8063	0.071	0.0375378	0.191743113
RP11-174G17	0.071	0.037552183	0.191796011
CNGB1	0.071	0.037626151	0.191988505
ZNF865	0.071	0.037657457	0.192107083
CRMP1	0.071	0.037697213	0.192268703
MIR4740	0.071	0.037723996	0.192364107
NUDT8	0.071	0.037741247	0.192407549
AC060834	0.071	0.037763065	0.192454781
CAPNS1	0.071	0.037803333	0.192583095
RP1-301L19	0.071	0.037808546	0.192589051
SPSB2	0.071	0.037868445	0.19277046
KLF16	0.071	0.037924722	0.192933212
OR2T6	0.071	0.037990532	0.193144222
BCAS2P3	0.071	0.038042816	0.193306859
MEIS2	0.071	0.038082319	0.19344567
LINC01564	0.071	0.038132123	0.193657351
RP6-43L17	0.071	0.038156168	0.193696299
HNF1B	0.071	0.03819525	0.193771322
GBAP1	0.071	0.038240574	0.193939283
CHI3L2	0.071	0.038252735	0.193945706
RP11-416N13	0.070	0.038281319	0.193994432
MIR8062	0.070	0.038289345	0.193994432
SIRT2	0.070	0.038300236	0.193994432
KCTD15	0.070	0.038337187	0.194098565
RP11-514O12	0.070	0.038342955	0.194107133
DEFA4	0.070	0.03844856	0.194455727
ZNF697	0.070	0.038471273	0.194487987
SNORD114-9	0.070	0.038531304	0.194688142
LINC00284	0.070	0.038604032	0.194931534
RNF175	0.070	0.038628888	0.195015695
PINLYP	0.070	0.03865925	0.195044925
KANK2	0.070	0.038673071	0.195093991
CTC-360J11	0.070	0.038835769	0.195571742
ZNF598	0.070	0.038905981	0.195758686

RNU1-3	0.070	0.038950603	0.195933711
TMEM8C	0.070	0.039002955	0.195969377
LINC00452	0.070	0.03902828	0.196026706
PRKG1	0.070	0.039040037	0.196031613
RP11-139K1	0.070	0.039054177	0.196081942
RP11-719J20	0.070	0.039063865	0.196109908
CTD-2620I22	0.070	0.039148102	0.196383394
WASF1	0.070	0.039157696	0.196383394
RP11-634B7	0.070	0.039163566	0.196383394
RN7SKP263	0.070	0.039172868	0.196403427
AL354978	0.070	0.039222428	0.196533044
GBGT1	0.070	0.039254233	0.196609671
CAPN2	0.070	0.039268862	0.19662187
TFAP2B	0.070	0.039269054	0.19662187
PRICKLE2	0.070	0.039275342	0.196632681
ANKRD34C	0.070	0.039281045	0.196640567
SLC8B1	0.070	0.03937003	0.196941121
PYGB	0.070	0.039462655	0.197284534
LINC01198	0.070	0.039467734	0.197284821
WBP1LP2	0.070	0.039573555	0.197627105
CCDC184	0.070	0.039645773	0.197816601
PIN4	0.070	0.039666164	0.197882099
DYTN	0.070	0.039680106	0.19791019
AC079305	0.070	0.039711245	0.197982568
CTC-455F18	0.070	0.039743051	0.198017692
FAM210CP	0.070	0.039785088	0.19810991
RP11-78O9	0.070	0.039788948	0.19810991
AP3S1	0.070	0.039790862	0.19810991
BACH1-IT2	0.070	0.039806543	0.198124203
RNU6-977P	0.070	0.039844781	0.19825828
LINC00316	0.070	0.039903498	0.198463231
PNMAL2	0.070	0.03991266	0.198481901
MFSD12	0.070	0.039958774	0.19865517
NME2	0.070	0.04000196	0.19878687
RP5-881L22	0.070	0.04003634	0.198916209
PES1	0.070	0.040056557	0.198995892
PTPRS	0.070	0.040159973	0.199344592
RPS20	0.070	0.040285878	0.199760074
CHRNA3	0.070	0.040303349	0.199780954
PTPLA	0.070	0.040404086	0.200137848
NANOGNB	0.070	0.040560811	0.200684576
HS6ST2	0.070	0.040621885	0.200882415
ERF	0.070	0.040628904	0.200896266
MT-TP	0.070	0.040653489	0.200955239
IDH3G	0.070	0.04081141	0.201407452
RN7SL143P	0.070	0.040816749	0.201407452
CTA-363E6	0.070	0.040859568	0.201513786
MUC4	0.070	0.040885748	0.201622028
MICALL1	0.070	0.040916906	0.201692515
AC018464	0.070	0.04101192	0.201912914
RN7SKP155	0.070	0.041012567	0.201912914
ZNF837	0.070	0.041087201	0.202175858

C4BPB	0.069	0.041121322	0.202239281
ABRACL	0.069	0.041129026	0.202256286
RPS8	0.069	0.041153214	0.20233345
SAMD4A	0.069	0.04117241	0.202349
PTP4A2P2	0.069	0.041173371	0.202349
AP001885	0.069	0.041199274	0.202425351
LGMN	0.069	0.041215343	0.202450817
SPATA12	0.069	0.04123255	0.202483114
NME4	0.069	0.041234672	0.202483114
GNG11	0.069	0.041319375	0.202669196
RN7SL318P	0.069	0.041407068	0.203015691
SHC3	0.069	0.041414556	0.203031502
C9orf89	0.069	0.041422209	0.203037943
C1orf105	0.069	0.041438391	0.203064746
RP13-143G15	0.069	0.041484638	0.203186843
TNFSF11	0.069	0.041492729	0.203205575
RP1-170O19	0.069	0.041563274	0.203465608
HOTAIRM1	0.069	0.041593347	0.203540632
RP11-328C8	0.069	0.041599453	0.203540632
UBA52	0.069	0.041603881	0.203540632
CPQ	0.069	0.041631489	0.203592046
CAPNS2	0.069	0.041636904	0.20359762
RP11-608O21	0.069	0.041774769	0.204055114
RP11-424I19	0.069	0.041777593	0.204055114
RP1-292L20	0.069	0.041789036	0.204069954
YAP1	0.069	0.041794874	0.204076735
SPANXN3	0.069	0.041891413	0.204485225
DTX1	0.069	0.041970408	0.20480785
SUN5	0.069	0.041986595	0.204865849
PIWIL1	0.069	0.041996857	0.204873943
TIMM23	0.069	0.04200685	0.204893675
BHMT2	0.069	0.042009506	0.204893675
ZNF454	0.069	0.042030976	0.204935431
PLD3	0.069	0.042038393	0.204950613
RAB13	0.069	0.04215483	0.205350118
PCDHB10	0.069	0.042161267	0.205360469
FUOM	0.069	0.042173976	0.205362697
NTN4	0.069	0.042194684	0.205381618
CRTAP	0.069	0.04224035	0.205493479
LINC00547	0.069	0.042252562	0.205510904
DKKL1P1	0.069	0.042305334	0.205704557
FHAD1	0.069	0.042400342	0.206045995
OPRK1	0.069	0.042401512	0.206045995
KRT15	0.069	0.042434366	0.206100502
DKK1	0.069	0.042480475	0.206240327
MFSD10	0.069	0.042525394	0.206343094
ATP1A1	0.069	0.042560286	0.206459434
CTD-2541J13	0.069	0.042607644	0.206626033
AGAP2-AS1	0.069	0.042628461	0.206680501
RP4-718N17	0.069	0.042631895	0.206680501
AZIN2	0.069	0.04272433	0.206941969
LYPD4	0.069	0.04272844	0.206941969

MIR22HG	0.069	0.042737968	0.206941969
C3orf30	0.069	0.042768873	0.207023795
AC009228	0.069	0.042769141	0.207023795
RP11-556E13	0.069	0.04277048	0.207023795
HIST1H3A	0.069	0.042772254	0.207023795
MMP10	0.069	0.042777055	0.207025994
LAMP2	0.069	0.042781545	0.207026688
RP11-156K13	0.069	0.04279746	0.207031821
RIPK4	0.069	0.0428048	0.207031821
HOXB9	0.069	0.042857446	0.207204504
MRPL55	0.069	0.042891385	0.207302925
C4orf48	0.069	0.042895213	0.207302925
PANX3	0.069	0.042965286	0.20751226
WI2-1896O14	0.069	0.042994522	0.20758279
DSE	0.069	0.042998866	0.20758279
RP11-8P13	0.069	0.043036807	0.207713216
AC004699	0.069	0.043062702	0.207775031
G0S2	0.069	0.043077239	0.207797669
TBL3	0.069	0.043085603	0.207797669
SPATS2L	0.069	0.043116527	0.207797669
NPPB	0.069	0.043117211	0.207797669
SLC9A5	0.069	0.043119751	0.207797669
RP11-85O21	0.069	0.043183345	0.208019942
RP11-378I6	0.069	0.043188192	0.208022249
PCDHB2	0.069	0.043197969	0.208048305
LMAN2	0.069	0.043221651	0.208073486
RPL3L	0.069	0.043299594	0.208284999
TCHHL1	0.069	0.043306013	0.208294835
SEC22A	0.069	0.043538036	0.209009781
BHMT	0.069	0.043570117	0.209121635
CALM2	0.069	0.043588236	0.209187515
DTX2	0.069	0.043595941	0.209192842
SP6	0.069	0.043619864	0.209233885
RP1-127D3	0.069	0.043763435	0.209626987
ERHP2	0.069	0.043798686	0.209753646
POLR2H	0.069	0.04393244	0.210165153
KRT8P23	0.069	0.044017161	0.210386666
RPS26P34	0.069	0.044024446	0.210386666
RNA5SP93	0.069	0.044025889	0.210386666
CSRP1	0.069	0.04412186	0.210708093
FOLR3	0.068	0.04416679	0.21080982
PFN2	0.068	0.044172741	0.21080982
CTC-573N18	0.068	0.044213146	0.210932536
SEMA6B	0.068	0.044238234	0.21100995
CLPSL1	0.068	0.044272509	0.211088878
PSENEN	0.068	0.044300857	0.211181754
AL139188	0.068	0.044368028	0.211369218
LINC01080	0.068	0.044374526	0.211369218
RP5-1093G23	0.068	0.044379519	0.211369218
RP5-1110E20	0.068	0.044387507	0.211383243
TSPAN4	0.068	0.044396931	0.211401856
UGT1A10	0.068	0.044472907	0.211662952

NPM1P11	0.068	0.04456666	0.21200323
ALDOA	0.068	0.044574018	0.212017052
CTB-32O4	0.068	0.04460469	0.212099399
SLC38A10	0.068	0.044675777	0.212301793
CTSB	0.068	0.044686091	0.212302371
AC092614	0.068	0.044687494	0.212302371
CLCN1	0.068	0.044697752	0.212329925
PABPC4L	0.068	0.044707485	0.212354981
FADS2P1	0.068	0.044756414	0.212478174
RN7SL303P	0.068	0.044759952	0.212478174
RP11-10A14	0.068	0.044760189	0.212478174
PEX14	0.068	0.044774695	0.212525853
MYLK2	0.068	0.044833184	0.2126763
CTD-2129N1	0.068	0.044841713	0.212695574
PTCHD1	0.068	0.044895021	0.21289046
MPP6	0.068	0.045017051	0.213314804
AC109642	0.068	0.04502705	0.213340958
AL035610	0.068	0.045052079	0.213438313
ASL	0.068	0.045083244	0.213530296
NALCN-AS1	0.068	0.045088789	0.213530296
ITGB8	0.068	0.045098395	0.213530296
CDH23	0.068	0.045134528	0.213680138
BCRP9	0.068	0.045162642	0.213770737
MIR3689C	0.068	0.045168626	0.213777817
SMTNL2	0.068	0.0452292	0.214018626
PRR5	0.068	0.04527141	0.214123896
S100A5	0.068	0.045327527	0.214320841
RP11-366L20	0.068	0.045341805	0.21436313
C7orf57	0.068	0.045410995	0.214605026
ORM1	0.068	0.045442119	0.214724996
AC092699	0.068	0.045453059	0.214739891
ERP27	0.068	0.045484546	0.214842879
NEU2	0.068	0.045494616	0.214850974
RN7SL448P	0.068	0.045635052	0.215258059
DCTN3	0.068	0.045659697	0.215352978
DCHS1	0.068	0.045669431	0.21537135
SNAI1	0.068	0.045672636	0.21537135
RP11-87N24	0.068	0.045680143	0.215385426
GBE1	0.068	0.045719672	0.21546683
RPL35	0.068	0.045720028	0.21546683
PRRG3	0.068	0.045729495	0.215490124
AC079987	0.068	0.045837475	0.215913503
RP11-400K9	0.068	0.045861678	0.215943203
FAM224A	0.068	0.045878467	0.215978427
EGFR	0.068	0.045981305	0.216398381
RNU6-503P	0.068	0.046166336	0.216981376
GDPD5	0.068	0.046168964	0.216981376
ZNF622	0.068	0.046224104	0.217091397
DUSP10	0.068	0.04623298	0.217096437
FOSL2	0.068	0.046241317	0.217096437
SERINC2	0.068	0.046245276	0.217096437
RP11-61J19	0.068	0.046249115	0.217096437

TMEM132B	0.068	0.046365963	0.217435185
RP11-383C5	0.068	0.046409427	0.21757474
ARFGAP1	0.068	0.046432034	0.217628593
RP11-141M3	0.068	0.046450516	0.217681662
TINAGL1	0.068	0.046511906	0.217895458
APMAP	0.068	0.046570829	0.218116716
CPTP	0.068	0.046618098	0.218237865
SYNGR4	0.068	0.046629422	0.218261839
MLF1	0.068	0.046650901	0.218261839
DAW1	0.068	0.046656808	0.218261839
CTD-3023L14	0.068	0.046734114	0.218537621
RP11-456O19	0.068	0.046965325	0.21927434
TMEM54	0.068	0.046986753	0.219331383
PPP2R3A	0.068	0.047052146	0.219477215
RP11-406O23	0.068	0.04706211	0.219477215
RPL21P133	0.068	0.047072517	0.219495084
MYLK	0.068	0.047157421	0.219783393
SNRPGP9	0.068	0.047280933	0.220219872
SHISA2	0.068	0.047311057	0.220283872
CTD-2325P2	0.067	0.047352988	0.220414461
AC068799	0.067	0.04737429	0.220470521
C9orf92	0.067	0.047381774	0.220483805
RRP1	0.067	0.047413756	0.220546434
C10orf90	0.067	0.047479652	0.220719561
CACNA1A	0.067	0.047516518	0.220788665
FOXC2	0.067	0.047523392	0.220797638
MCU	0.067	0.047580256	0.220997157
MAD2L2	0.067	0.047586277	0.221003571
TMEM132A	0.067	0.047596417	0.221014968
CAPSL	0.067	0.047605531	0.221021958
PPP1R14BP3	0.067	0.047609139	0.221021958
C17orf64	0.067	0.047655952	0.221154633
LINC00942	0.067	0.047692139	0.221222024
MGMT	0.067	0.047701481	0.221222024
AC011738	0.067	0.047834619	0.221590655
RP4-590F24	0.067	0.047862041	0.22165747
SCN1A	0.067	0.047906901	0.221843652
RP3-523K23	0.067	0.04792293	0.221896302
SEC11A	0.067	0.047963325	0.221974589
SYPL2	0.067	0.047965676	0.221974589
RPL36A	0.067	0.047974584	0.221974589
RBPMS2	0.067	0.047995436	0.222016185
TMEM220-AS1	0.067	0.048013944	0.222080228
P2RX6P	0.067	0.048079072	0.222273534
LINC00844	0.067	0.048108235	0.222340883
FGF18	0.067	0.048210282	0.222642403
RPS28P7	0.067	0.048252229	0.222749724
RNU6-302P	0.067	0.048278301	0.22282225
THEG	0.067	0.048291377	0.22282225
MRPL36	0.067	0.04829601	0.22282225
ANPEP	0.067	0.048384359	0.223143396
RP4-541C22	0.067	0.048432705	0.223336747

RP11-467J12	0.067	0.048442372	0.223346064
NANOGP9	0.067	0.048448085	0.223350782
SCAF1	0.067	0.048479832	0.223436287
RPS19BP1	0.067	0.048480706	0.223436287
KIAA1210	0.067	0.048520283	0.223546006
CD58	0.067	0.048523287	0.223546006
RP11-115J23	0.067	0.048558653	0.223622412
GPSM1	0.067	0.048563712	0.223624086
ZSWIM2	0.067	0.048590261	0.223724708
U82695	0.067	0.04864701	0.223793445
RP11-54I5	0.067	0.04864748	0.223793445
UQCRC1	0.067	0.048742369	0.224056828
RP11-249L21	0.067	0.048749016	0.224065757
WNT9A	0.067	0.048819849	0.2241966
TLL1	0.067	0.048827978	0.224212313
RP1-41C23	0.067	0.048854086	0.224253628
RP4-761I2	0.067	0.048856963	0.224253628
RXFP1	0.067	0.048860518	0.224253628
DRAP1	0.067	0.049036586	0.224693618
LAMP5	0.067	0.049189716	0.225135363
CTD-2367A17	0.067	0.04921999	0.225252276
CTB-14A14	0.067	0.04922682	0.225261889
C4orf47	0.067	0.049236106	0.225282737
MIR219A1	0.067	0.04930336	0.225512175
AC000077	0.067	0.04941361	0.225769571
DMRTA2	0.067	0.049437476	0.225829221
EFNA1	0.067	0.049445632	0.225829221
CDH16	0.067	0.049465721	0.225844059
RP11-114G11	0.067	0.049476109	0.225844059
SLC35C1	0.067	0.049545654	0.226004295
ZMAT3	0.067	0.049589653	0.226112693
HDGFL1	0.067	0.049593155	0.226112693
LANCL3	0.067	0.049626364	0.226239273
IL36RN	0.067	0.049635168	0.226239273
RP11-316O14	0.067	0.049647584	0.226249006
RBPJP7	0.067	0.049673657	0.226328067
SLC27A1	0.067	0.049764447	0.226568333
MBNL1-AS1	0.067	0.049775549	0.226592403
RNA5SP519	0.067	0.049807207	0.226654676
NDUFS7	0.067	0.049939437	0.227029434
AMPD2	0.067	0.049963051	0.227078185
USP2	0.067	0.049970028	0.227078185
RP11-181B18	0.067	0.049975316	0.227078185
MAPK7	0.067	0.049976558	0.227078185
NTRK1	0.067	0.049987251	0.227099553
GAPDHP59	0.067	0.049995566	0.227099553
C11orf39	0.067	0.050031961	0.227137234
KRT127P	0.067	0.050100726	0.227399977
TUFM	0.067	0.050130986	0.227454366
RP11-201E8	0.067	0.05018719	0.227622635
LINC00087	0.067	0.050214231	0.227723593
ARPC4	0.067	0.050260206	0.227893944

SERTAD4-AS1	0.067	0.050275067	0.227912681
ENPP6	0.067	0.050334612	0.228079328
RP11-429A20	0.067	0.050410555	0.228255754
FAM25BP	0.067	0.050412244	0.228255754
RFPL3	0.067	0.050458113	0.22830786
PLTP	0.067	0.050552051	0.228580892
KCNJ6	0.067	0.050562685	0.228585573
RNU6-529P	0.067	0.05059843	0.228702065
C4orf17	0.067	0.050625269	0.228779955
MTND1P17	0.067	0.050649066	0.228802339
EGFL7	0.067	0.050675442	0.228812504
NR1H2	0.067	0.050689239	0.228812504
YBX1P6	0.066	0.050726522	0.228935096
RP11-746M1	0.066	0.050771548	0.229116592
RPS15AP17	0.066	0.050864106	0.229382138
RP11-568K15	0.066	0.050899246	0.229460151
PODN	0.066	0.050900676	0.229460151
AHRR	0.066	0.050954724	0.229638591
RHOD	0.066	0.050985769	0.229715071
HCAR2	0.066	0.051027514	0.229792681
ETV1	0.066	0.051071112	0.229902095
GOLGA6L2	0.066	0.051124186	0.230020382
SLC35E4	0.066	0.051129226	0.230020382
RP5-884M6	0.066	0.051149997	0.23006147
TPST2	0.066	0.051252747	0.230267906
TRPC4	0.066	0.051253859	0.230267906
FURIN	0.066	0.051257913	0.230267906
NCCRP1	0.066	0.0513807	0.230619979
RP11-571O6	0.066	0.051405245	0.230643205
MYL6P4	0.066	0.05146482	0.230806264
FAM131B	0.066	0.051465818	0.230806264
SIM2	0.066	0.051595212	0.231190627
COMMD2	0.066	0.051664224	0.231456306
TCTEX1D4	0.066	0.051713717	0.231612675
TCP10L	0.066	0.051805579	0.232002286
LINC01423	0.066	0.05184179	0.232077162
ITGAV	0.066	0.051906273	0.232256675
SNCAIP	0.066	0.051978013	0.232381191
SMCO4	0.066	0.05205136	0.232574309
GLI2	0.066	0.052070041	0.232574309
ETFB	0.066	0.052108482	0.2327211
IGF2BP1	0.066	0.052142936	0.232834391
SNORD114-12	0.066	0.052197396	0.232924707
KIF1C	0.066	0.052253509	0.233065918
PDF	0.066	0.052273846	0.233134795
AC011242	0.066	0.052361846	0.23339613
KRT8P50	0.066	0.05241752	0.233556857
HFE	0.066	0.052463913	0.23369798
LINC00636	0.066	0.052498818	0.233809726
RP11-474O21	0.066	0.052644845	0.234262921
ADAMTSL5	0.066	0.052753009	0.234590809
STX1B	0.066	0.052805007	0.23473422

TDRP	0.066	0.052842425	0.234813039
TDRD1	0.066	0.052907887	0.234979296
FAM86FP	0.066	0.05302594	0.235365
AC012075	0.066	0.053055038	0.235420724
RP11-710C12	0.066	0.053058266	0.235420724
CGB7	0.066	0.05317355	0.235756534
VPS37B	0.066	0.053210257	0.23585341
CHRD	0.066	0.053215587	0.235855085
PLCD3	0.066	0.05326101	0.235998872
MIR485	0.066	0.053275639	0.236019913
UQCC3	0.066	0.053277554	0.236019913
TTTY9B	0.066	0.053308369	0.236134458
RP11-686D22	0.066	0.053411077	0.236307608
KXD1	0.066	0.053478248	0.236513001
PGM1	0.066	0.053512198	0.236575278
RPL7P32	0.066	0.053519701	0.236586484
AC008166	0.066	0.053583214	0.236661338
RPL21P119	0.066	0.053586324	0.236661338
RP11-29G8	0.066	0.053610967	0.236748219
RP11-770E5	0.066	0.053620803	0.236769702
REREP1Y	0.066	0.053631589	0.236773424
GLP1R	0.066	0.053646467	0.236795207
RN7SKP36	0.066	0.053660933	0.236837108
RN7SKP113	0.066	0.053700384	0.236967313
C16orf62	0.066	0.053734914	0.237056596
APOBEC1	0.066	0.053772485	0.237175602
RPL10	0.066	0.053784383	0.237206113
C11orf88	0.066	0.053890867	0.237437284
GNL1	0.066	0.053901912	0.237437284
ATP1B3	0.066	0.053911188	0.237437284
AC005523	0.066	0.053918255	0.237437284
DBET	0.066	0.053918587	0.237437284
LINC01139	0.066	0.053924198	0.237437284
RP11-517I3	0.066	0.054000597	0.237667882
MT2P1	0.066	0.054029833	0.237730238
FUNDC2	0.066	0.05404159	0.237730238
P3H2	0.066	0.054070642	0.237807965
MEGF8	0.066	0.054081582	0.237821776
RP11-364M6	0.066	0.054089412	0.237821776
UBE2Q2P6	0.066	0.054183075	0.237994773
LINC00229	0.066	0.054190649	0.238006089
SNORD113-4	0.066	0.054209852	0.238046527
RASAL1	0.066	0.054282029	0.238165842
P2RY1	0.065	0.05434725	0.23833115
OR7E36P	0.065	0.054351367	0.23833115
TMEM200A	0.065	0.054354735	0.23833115
LIMK2	0.065	0.054428118	0.23843521
AP000474	0.065	0.054476833	0.238571955
RP11-123B3	0.065	0.054555117	0.238792076
DCTPP1	0.065	0.054568034	0.238826663
PSMA6P3	0.065	0.054637956	0.239044822
MMP7	0.065	0.054819296	0.239596093

P2RY11	0.065	0.054869733	0.239693722
MIR4322	0.065	0.05487545	0.239693722
ZNF90P3	0.065	0.054982308	0.240022215
VCY1B	0.065	0.055098125	0.240285541
PPDPF	0.065	0.055178592	0.240487168
FAT2	0.065	0.055179704	0.240487168
TUFT1	0.065	0.055207247	0.24056318
GAPDHP2	0.065	0.055343273	0.241002073
NAGLU	0.065	0.055343391	0.241002073
PLA2G2A	0.065	0.055352537	0.241009791
RSU1P2	0.065	0.055522235	0.24151589
STK38L	0.065	0.05557593	0.241624962
ETV2	0.065	0.055577749	0.241624962
LINC01272	0.065	0.055630447	0.241699624
TFPT	0.065	0.055651033	0.241744966
AC003075	0.065	0.055669721	0.241759989
PGK1	0.065	0.0556876	0.241788397
EIF3KP1	0.065	0.05568875	0.241788397
ZP4	0.065	0.055729012	0.241792799
USP2-AS1	0.065	0.055744621	0.241792799
ITGB3	0.065	0.055775947	0.241868434
FOLH1	0.065	0.05583189	0.242057174
RNU6-83P	0.065	0.0558398	0.242057174
RP11-792A8	0.065	0.055886333	0.242192757
SYNDIG1L	0.065	0.05589934	0.242227084
LHX9	0.065	0.055948927	0.242375802
RELB	0.065	0.056030066	0.242594903
LMO7-AS1	0.065	0.05604295	0.242628634
FAM173A	0.065	0.056084599	0.242764811
LRP4	0.065	0.056309894	0.243474473
SI	0.065	0.056318037	0.243487577
RP11-425D10	0.065	0.056335429	0.243533267
VN1R10P	0.065	0.056352811	0.243533267
SNORA11B	0.065	0.056371977	0.243551423
RP11-567G11	0.065	0.0563737	0.243551423
TMEM200C	0.065	0.056422397	0.24369543
ATP2B2	0.065	0.056477643	0.243867696
RNU4-6P	0.065	0.056494796	0.243897538
ZNF556	0.065	0.056537227	0.243941141
MT1H	0.065	0.056543643	0.243941141
AL391384	0.065	0.056558925	0.243953262
EVPLL	0.065	0.056623958	0.244079036
LINC01444	0.065	0.056642678	0.244108275
CALM1P1	0.065	0.056688079	0.244134472
RPL13AP20	0.065	0.056703604	0.244179256
DVL1	0.065	0.056781467	0.244443627
PSG8	0.065	0.056800484	0.244485917
RP11-414C23	0.065	0.056820983	0.244552047
BCAR1P2	0.065	0.056862649	0.244671679
RNU6-238P	0.065	0.056890558	0.244718803
PPIAP11	0.065	0.056903063	0.244750492
G6PD	0.065	0.056913123	0.244771655

MGC32805	0.065	0.056963747	0.244809993
CHCHD6	0.065	0.056979939	0.244837923
QPCTL	0.065	0.057031237	0.244869472
DDX55P1	0.065	0.057031763	0.244869472
MIR8053	0.065	0.057043022	0.244869472
CLEC18A	0.065	0.057048978	0.244869472
CUEDC2	0.065	0.057135881	0.245071918
MICA	0.065	0.057148767	0.245084616
RNF216P1	0.065	0.057221474	0.245145973
RP1-231P7P	0.065	0.057227342	0.245145973
RP11-414H17	0.065	0.057236929	0.245145973
ARHGEF17	0.065	0.057315857	0.245351639
ADCY8	0.065	0.057332865	0.245402388
AC007403	0.065	0.057413962	0.245639128
GADD45A	0.065	0.057432221	0.245695174
TMEM132D	0.065	0.057459922	0.245769529
SCG2	0.065	0.057468319	0.24578337
RPS5	0.065	0.057483281	0.245803216
RN7SKP28	0.065	0.057489475	0.245807633
RP11-729I10	0.065	0.05750981	0.245850436
OSR1	0.065	0.057535387	0.245893564
RIMBP2	0.065	0.057612064	0.246154997
SOBP	0.065	0.057702693	0.246409591
EPPK1	0.065	0.057748392	0.246507541
GALNS	0.065	0.057751859	0.246507541
CoTC_ribozyme	0.065	0.057756685	0.246507541
RAB11FIP5	0.065	0.057790291	0.246563824
B9D2	0.065	0.057805395	0.246582855
AC017060	0.065	0.057828827	0.246616544
FABP5P10	0.065	0.057882535	0.246779295
RP11-642D21	0.065	0.057933731	0.246842887
ARMCX2	0.065	0.05794952	0.246888076
RN7SL269P	0.065	0.05797261	0.246964356
KRT16P4	0.065	0.057995451	0.246995052
RP11-315O6	0.064	0.058201558	0.24757579
HOXA-AS3	0.064	0.058270339	0.247767294
RP11-305M3	0.064	0.058279923	0.24778592
RP5-933B4	0.064	0.05832576	0.24793654
HOXA10-AS	0.064	0.058331922	0.247940603
CABP7	0.064	0.058350169	0.247973902
LINC01193	0.064	0.058398496	0.24811286
CFAP58	0.064	0.058423219	0.248173623
CAHM	0.064	0.058449862	0.248201805
SNHG9	0.064	0.058536891	0.24836851
FABP3	0.064	0.058552596	0.248413011
ORM2	0.064	0.058609871	0.248611713
LINC00456	0.064	0.058647257	0.248748145
USH1G	0.064	0.058667365	0.248773813
HIST2H3D	0.064	0.058723491	0.248943954
CTD-2382E5	0.064	0.058760755	0.248995414
HMGN2P26	0.064	0.058781178	0.249027856
NUTM2F	0.064	0.058822262	0.249053475

FAM114A1	0.064	0.058846359	0.249063063
CTC-806A22	0.064	0.058857316	0.249084539
DGAT1	0.064	0.058899561	0.24919689
MAP1B	0.064	0.058931575	0.24931019
LINC01563	0.064	0.059047013	0.249554716
RNF7	0.064	0.059073608	0.249622813
LINC00900	0.064	0.059102166	0.249685888
LPAR3	0.064	0.059104262	0.249685888
AC008132	0.064	0.05927228	0.250054171
RP1-142L7	0.064	0.05932237	0.250142117
RRAGC	0.064	0.05942801	0.250453749
LINC00615	0.064	0.05947485	0.250562466
ALDH7A1	0.064	0.059483941	0.250578602
JAG1	0.064	0.059496969	0.25059913
NDUFB2	0.064	0.059566143	0.250858314
GNG12	0.064	0.059604609	0.250909381
GSN	0.064	0.059623369	0.250943988
COL26A1	0.064	0.059636423	0.250976754
EREG	0.064	0.059659498	0.250985154
ZBED1	0.064	0.059682792	0.251038801
CTD-2337A12	0.064	0.059703172	0.251080173
TBC1D3D	0.064	0.059742406	0.251178633
RNU2-64P	0.064	0.059784713	0.251312139
BUD31	0.064	0.059904747	0.251636555
RPS4XP7	0.064	0.059928901	0.251673907
KCNK2	0.064	0.060068341	0.252015022
PTHLH	0.064	0.060071922	0.252015022
RP11-215A19	0.064	0.060082002	0.252027905
MUC15	0.064	0.060243827	0.252439973
AL365502	0.064	0.060265324	0.252463419
SGMS2	0.064	0.060295148	0.252496125
MIR6072	0.064	0.06029603	0.252496125
GCK	0.064	0.060296326	0.252496125
USP9Y	0.064	0.060326921	0.252563186
LINC00705	0.064	0.060331563	0.252563186
ZNF536	0.064	0.060338554	0.252570252
GNB1L	0.064	0.060400535	0.25269644
KCNS1	0.064	0.060452883	0.252826328
ZNF502	0.064	0.060476655	0.252837221
AC073109	0.064	0.060507082	0.25292003
NME7	0.064	0.060530398	0.252950889
UGT2B7	0.064	0.060548337	0.253003653
IFNA1	0.064	0.060582899	0.253089212
LRRTM1	0.064	0.060585942	0.253089212
DLX1	0.064	0.060612575	0.253116164
WBSCR22	0.064	0.060648759	0.253156744
RNF24	0.064	0.060655002	0.253160615
RARRES1	0.064	0.060682762	0.253229691
PCDHGC3	0.064	0.060687503	0.253229691
HSPG2	0.064	0.060706167	0.253263194
RP11-564P9	0.064	0.060790128	0.253524644
HOXC8	0.064	0.060811695	0.253570181

BDH2	0.064	0.060811695	0.253570181
CDH24	0.064	0.060845321	0.253665976
TM4SF1	0.064	0.060879868	0.253734686
TMEM38A	0.064	0.060883112	0.253734686
RP11-443P15	0.064	0.060917205	0.253773537
EMC6	0.064	0.060924924	0.253773537
GNB2	0.064	0.060939873	0.253787265
AP001476	0.064	0.060949015	0.253787265
RN7SKP97	0.064	0.061021954	0.25402435
RP3-454B23	0.064	0.06104895	0.254092312
PLD1	0.064	0.061207129	0.254483801
RP11-451M19	0.064	0.061220995	0.254507193
LINC01266	0.064	0.061228787	0.254507193
TLN2	0.064	0.061467824	0.255140203
OMD	0.064	0.061471093	0.255140203
ASPH	0.064	0.061523742	0.255267971
RP11-290L7	0.064	0.061524367	0.255267971
RP11-1059L18	0.064	0.061545114	0.255309569
ADCYAP1R1	0.064	0.06158648	0.255436669
IL17RD	0.064	0.06165598	0.255591378
TSPAN2	0.064	0.061747678	0.255915188
UQCRH	0.064	0.061923226	0.256352999
CD164L2	0.064	0.061925825	0.256352999
CORO1C	0.064	0.061955752	0.256388497
RP5-1009N12	0.064	0.062072065	0.256712941
SCHIP1	0.063	0.062139687	0.256903375
SPP2	0.063	0.062175468	0.257006686
SAMD11	0.063	0.062188665	0.257038927
RPS26P43	0.063	0.062309695	0.257338176
LINC00702	0.063	0.062315314	0.257339066
OR3A2	0.063	0.062331898	0.257385237
NWD1	0.063	0.062390899	0.257571711
MIR4634	0.063	0.062408316	0.25760803
SNORD114-24	0.063	0.06241814	0.25760803
RP11-195F19	0.063	0.062462055	0.257766252
PGAM1P5	0.063	0.062604263	0.258174148
TPM4P1	0.063	0.062613753	0.258190928
PNMAL1	0.063	0.062634258	0.258218576
CHODL	0.063	0.062660886	0.258227354
CPNE2	0.063	0.062674356	0.258227354
LUZP2	0.063	0.062678457	0.258227354
MTHFD2P5	0.063	0.062679097	0.258227354
SIRT4	0.063	0.062687649	0.258227354
RP11-302L19	0.063	0.06270757	0.25826474
AC007969	0.063	0.062719192	0.258290267
SP8	0.063	0.062768639	0.258426865
TMSB10P1	0.063	0.062800328	0.258465044
RP11-865I6	0.063	0.062805047	0.258465044
RP11-386B13	0.063	0.062853917	0.258633018
RP11-295K3	0.063	0.062880673	0.258686864
SCTR	0.063	0.062900125	0.258722193
CFI	0.063	0.062911434	0.258727846

RNU6-358P	0.063	0.062992835	0.258924634
RP11-793I11	0.063	0.063032587	0.258979281
YWHAQP9	0.063	0.063057777	0.259057428
PTS	0.063	0.063083182	0.259097
DERA	0.063	0.063101132	0.259123719
CTD-2175A23	0.063	0.063106558	0.259123719
JMJD4	0.063	0.063140932	0.259175488
DGUOK	0.063	0.063179975	0.259223781
CTD-2201E9	0.063	0.063199325	0.259223781
RP11-295G20	0.063	0.063270348	0.2593884
CADM1	0.063	0.063290055	0.259407527
RN7SL443P	0.063	0.063348759	0.259617178
LGALS3	0.063	0.063411061	0.25974702
ASPG	0.063	0.063446017	0.259845509
RPL36AP40	0.063	0.06346802	0.259868587
NEXN	0.063	0.063517879	0.260020172
MEA1	0.063	0.063559146	0.260129885
KCNQ3	0.063	0.063598637	0.260246781
BACH1-AS1	0.063	0.063604266	0.260247456
UTF1	0.063	0.063630447	0.260332213
FOXC1	0.063	0.063654819	0.260374316
ZNF667-AS1	0.063	0.063705637	0.260476147
SERPINA7	0.063	0.063765683	0.260505075
RP11-61K9	0.063	0.063846207	0.260744579
RNU6-961P	0.063	0.063861787	0.260785847
RP11-20J15	0.063	0.06388363	0.260830316
SOX9	0.063	0.063906103	0.260883681
HNRNPA1P73	0.063	0.063917187	0.260883681
ARL4A	0.063	0.063945064	0.260904138
SLC27A5	0.063	0.063945535	0.260904138
FGF11	0.063	0.063985389	0.26095497
HEYL	0.063	0.064067416	0.261155618
AC016734	0.063	0.064125514	0.261281761
DBI	0.063	0.064153949	0.261329125
GLG1	0.063	0.064163561	0.261334168
RP11-563H6	0.063	0.064177151	0.261356576
A4GALT	0.063	0.064191931	0.261394418
RNF187	0.063	0.064228512	0.261521019
PPIE	0.063	0.064239396	0.261536671
SLC16A4	0.063	0.064273395	0.26159195
RPS16	0.063	0.064321225	0.261697163
EPHX4	0.063	0.064368294	0.261866298
FAM47E-STBD1	0.063	0.064376589	0.261877676
SNORD114-2	0.063	0.064400066	0.261950801
PDE3A	0.063	0.064416498	0.261995266
RNU6-760P	0.063	0.064440719	0.262047247
RP5-1136G13	0.063	0.064531914	0.262330271
UQCRQ	0.063	0.064624749	0.262581206
MAP1A	0.063	0.064631487	0.262581206
PI3	0.063	0.064687807	0.262629681
TEAD3	0.063	0.064741186	0.26279386
ZNF843	0.063	0.064773261	0.262823077

HOXA5	0.063	0.064776834	0.262823077
RP11-128A17	0.063	0.064795531	0.262833784
CREB3L1	0.063	0.064898985	0.263133657
KIAA1614	0.063	0.064900814	0.263133657
SOX21-AS1	0.063	0.064916794	0.263166255
GAS2L1	0.063	0.064919905	0.263166255
AP001439	0.063	0.06497502	0.263322438
INO80B	0.063	0.065021006	0.263419145
RNU6-34P	0.063	0.065106447	0.263651111
CLUH	0.063	0.065116813	0.263651111
RP11-62H20	0.063	0.065117014	0.263651111
OR7E39P	0.063	0.065153609	0.263754437
SPRR2D	0.063	0.065198403	0.263890911
AC007229	0.063	0.065220282	0.263934604
TAAR7P	0.063	0.065249686	0.264031163
MINOS1P3	0.063	0.065343981	0.264121247
RP11-674P19	0.063	0.065429592	0.264399726
RP11-351J23	0.063	0.065459479	0.264413657
RPS15	0.063	0.06547292	0.264413657
LYVE1	0.063	0.065485178	0.264413657
TMEM8A	0.063	0.065495355	0.264413657
RNA5SP22	0.063	0.065500273	0.264413657
AC015933	0.063	0.065529809	0.264445649
C8orf48	0.063	0.065544922	0.264445649
UBTFL3	0.063	0.065556423	0.264445649
CGB5	0.063	0.065603357	0.264507315
S100A8	0.063	0.065656592	0.26459862
RP1-249H1	0.063	0.065703832	0.2647218
PID1	0.063	0.065769813	0.264965223
FTH1P3	0.063	0.065798525	0.265030169
HDHD3	0.063	0.065802628	0.265030169
FHL1	0.063	0.065820569	0.265047078
OR6C6	0.063	0.065825565	0.265047078
DEFB4A	0.063	0.065904031	0.265236713
ARID3C	0.063	0.065985214	0.26538404
WDR1	0.063	0.065992059	0.265389159
RPL13P12	0.063	0.066025369	0.265411762
LINC01549	0.063	0.066025543	0.265411762
CLDN5	0.063	0.066060145	0.265528444
RP13-81N3	0.063	0.066179569	0.26585141
TEAD1	0.063	0.066220988	0.265972931
SPRR2B	0.063	0.066272266	0.266111559
MAGIX	0.063	0.066290644	0.26614048
KNCN	0.063	0.06631021	0.266185938
SHMT2	0.063	0.066318734	0.266185938
RP11-128A6	0.062	0.066378462	0.266358329
BLACAT1	0.062	0.066404066	0.266438622
RP11-387D10	0.062	0.066419781	0.266479229
RP11-684N3	0.062	0.066456346	0.266558562
PCSK5	0.062	0.066503868	0.266681799
CLDN17	0.062	0.066629838	0.267008725
RPL13	0.062	0.066657875	0.267030877

NCBP2-AS2	0.062	0.066664379	0.267032287
pRNA	0.062	0.066771294	0.267224993
CTA-298G8	0.062	0.066779597	0.267224993
CUTA	0.062	0.066851203	0.267444119
GCNT2	0.062	0.06698399	0.267727971
BCAP31	0.062	0.067007258	0.267753561
ALS2CR11	0.062	0.067092545	0.267926365
RPS23P4	0.062	0.067118632	0.267968343
SUPT20HL1	0.062	0.06715305	0.268013645
TCF15	0.062	0.067178151	0.268027657
NANOGP1	0.062	0.067179524	0.268027657
KB-1448A5	0.062	0.067184439	0.268027657
RP11-296E3	0.062	0.067294894	0.268362548
SFRP5	0.062	0.067318081	0.268410068
SH2D7	0.062	0.067429751	0.268697854
SPATA3	0.062	0.067449301	0.268719543
SUPT20HL2	0.062	0.067477244	0.26875219
FLJ43879	0.062	0.067551361	0.268867514
IFNA2	0.062	0.067572255	0.268924738
MT1JP	0.062	0.067577031	0.268924738
PENK	0.062	0.067643623	0.269155716
SNORD114-28	0.062	0.067646375	0.269155716
TMEM182	0.062	0.06789721	0.269725617
AP004782	0.062	0.068041338	0.270208023
RPL7	0.062	0.068112021	0.27044362
RNA5SP64	0.062	0.068156856	0.270531425
HSD17B1	0.062	0.068203188	0.270670215
MOGAT2	0.062	0.068238713	0.270730153
SOX1	0.062	0.068246713	0.270730153
NRBF2P4	0.062	0.068320847	0.270869523
BAHCC1	0.062	0.068480034	0.271291041
SLC35C2	0.062	0.068511129	0.271341577
ZDHHC8	0.062	0.068530756	0.271359688
PARD6G	0.062	0.068554964	0.271432976
GGCT	0.062	0.068652032	0.271645424
LINC00609	0.062	0.06865425	0.271645424
SEC61B	0.062	0.068676691	0.271711643
UCN	0.062	0.068713694	0.271769389
RP11-715J22	0.062	0.068722447	0.271769389
PCOLCE2	0.062	0.068846951	0.272039159
RP11-154H12	0.062	0.06886156	0.272039159
YPEL4	0.062	0.069049297	0.272664858
RNU1-140P	0.062	0.069101014	0.272823842
RN7SL384P	0.062	0.069171033	0.273043131
ABHD11-AS1	0.062	0.069173755	0.273043131
RPS2P28	0.062	0.069184682	0.273043532
AC097461	0.062	0.069218548	0.273106767
COX5B	0.062	0.069237227	0.273139844
GPC6-AS2	0.062	0.06926203	0.273185714
NFIA	0.062	0.069355915	0.273422245
NWD2	0.062	0.06938669	0.273520931
MIR3119-2	0.062	0.069405802	0.273550261

RP11-783K16	0.062	0.069465601	0.273717949
HABP4	0.062	0.069471152	0.273717949
AC019100	0.062	0.069547093	0.273852191
NRBP1	0.062	0.069564903	0.273852191
RP11-427M20	0.062	0.069574223	0.273852191
RNU6-883P	0.062	0.069597776	0.273914681
DAB2IP	0.062	0.069607353	0.273914681
EXTL1	0.062	0.069629934	0.273958269
ANO1-AS2	0.062	0.069676611	0.274066098
RP11-555G19	0.062	0.069680357	0.274066098
OLIG2	0.062	0.069710432	0.274116474
NOMO1	0.062	0.069736178	0.274138783
COL27A1	0.062	0.069795767	0.274203002
PLS1-AS1	0.062	0.069822155	0.274284046
CIB1	0.062	0.069841035	0.274312962
C1orf177	0.062	0.069854104	0.274341667
TMEM31	0.062	0.069866977	0.274348278
FCN2	0.062	0.069912073	0.274491891
LINC00933	0.062	0.069929698	0.27451221
CRYM	0.062	0.070027976	0.274670176
RP11-256I9	0.062	0.07003103	0.274670176
RP11-353N4	0.062	0.070032351	0.274670176
FAM127C	0.062	0.07012993	0.274926182
CCR10	0.062	0.070244268	0.275238483
RNU6-1276P	0.062	0.070257426	0.27524475
CXADRP1	0.062	0.07028327	0.275323351
RP11-466L17	0.062	0.070310542	0.275384885
KRT39	0.062	0.07037121	0.275554521
IFI27L2	0.062	0.070424481	0.275659761
CLPSL2	0.062	0.070431239	0.275659761
ACE2	0.062	0.070433946	0.275659761
H1FX	0.062	0.070438602	0.275659761
RP1-309H15	0.062	0.070480356	0.275777844
UGT1A2P	0.062	0.070622985	0.2761771
RP11-540D14	0.062	0.070672683	0.276303387
CTD-2194D22	0.061	0.070808879	0.276544999
WASH6P	0.061	0.070811842	0.276544999
MAP6D1	0.061	0.070814577	0.276544999
AL096700	0.061	0.070828458	0.276549185
CD99L2	0.061	0.070939239	0.276882601
RP5-905G11	0.061	0.070942057	0.276882601
RPN1	0.061	0.07107685	0.277321992
RP1-205F14P	0.061	0.071078791	0.277321992
AP000351	0.061	0.07112326	0.277450039
ROBO3	0.061	0.071174431	0.277621959
BCHE	0.061	0.0711826	0.277621959
RPL21P42	0.061	0.071279899	0.277823322
CDA	0.061	0.071279938	0.277823322
CTD-2119L1	0.061	0.071300383	0.277867877
MYDGF	0.061	0.07139383	0.278034435
RP11-480C22	0.061	0.07140013	0.278034435
RP1-107N3	0.061	0.071407173	0.278034435

MIR346	0.061	0.071525644	0.278256348
CCS	0.061	0.071562836	0.278311695
CBY1	0.061	0.071631548	0.278496212
RFWD2P1	0.061	0.071637036	0.278496212
PTRHD1	0.061	0.071727593	0.278774441
GLYATL1	0.061	0.071728012	0.278774441
PFKFB4	0.061	0.071756781	0.278774441
ADA	0.061	0.071770083	0.278785886
SLC6A7	0.061	0.071844587	0.279029775
AC022538	0.061	0.071890641	0.279102077
LPHN2	0.061	0.071894044	0.279102077
NLGN4X	0.061	0.071898364	0.279102077
DMGDH	0.061	0.071941981	0.279114188
CD207	0.061	0.071954228	0.279114188
AP001596	0.061	0.071964044	0.279129532
SOX6	0.061	0.072007785	0.279196096
RP11-333I13	0.061	0.072015253	0.279196096
SPTLC1P4	0.061	0.07206867	0.279349316
RP11-394A14	0.061	0.072073496	0.279349316
TNS4	0.061	0.072093664	0.279382014
NEUROD1	0.061	0.072225774	0.279689155
AC005082	0.061	0.072232949	0.279694199
TOMM40	0.061	0.072264284	0.279747303
COL9A3	0.061	0.072287568	0.279755641
RP11-776B3	0.061	0.072327739	0.279856465
HOXD9	0.061	0.072350413	0.279921457
SLITRK4	0.061	0.07246666	0.280159457
BOC	0.061	0.072470752	0.280159457
RNU6ATAC41P	0.061	0.072504279	0.280250651
ADCK5	0.061	0.072513166	0.280255179
RPL32	0.061	0.072620032	0.280577128
LRRTM4	0.061	0.072651266	0.280592067
NES	0.061	0.072681193	0.280654061
C1orf35	0.061	0.072760652	0.280824276
PMS2P10	0.061	0.072821993	0.280935242
DGCR6L	0.061	0.072853735	0.280978609
AP000275	0.061	0.07291598	0.281083554
MRGPRF-AS1	0.061	0.072916357	0.281083554
DMRTC2	0.061	0.072929852	0.281090074
SF3B6	0.061	0.072953919	0.281120024
RN7SKP62	0.061	0.073066288	0.281365467
KPTN	0.061	0.07307762	0.281385639
IL1RN	0.061	0.073157695	0.281478521
RN7SL496P	0.061	0.073159605	0.281478521
ARHGEF37	0.061	0.073177177	0.281496601
GAPDHP51	0.061	0.073222791	0.281626573
GYPB	0.061	0.073305967	0.281739112
CARHSP1	0.061	0.073311206	0.281739112
KIAA1462	0.061	0.073355429	0.281886316
NEDD9	0.061	0.07338287	0.281964758
RP11-332M4	0.061	0.073405214	0.281980546
MIR205HG	0.061	0.073447172	0.282037009

AC004009	0.061	0.073547792	0.282238391
LDOC1	0.061	0.07368616	0.282616412
RP11-402N8	0.061	0.073707029	0.282647743
MAP7D2	0.061	0.073875213	0.283135608
SPRR2E	0.061	0.07390328	0.283214636
CCKAR	0.061	0.073941312	0.283251204
AMPH	0.061	0.073950363	0.283251204
LDHA	0.061	0.073962137	0.283251204
PSMD4	0.061	0.074027232	0.283370437
CLLU1OS	0.061	0.074071124	0.283470102
UBE2U	0.061	0.074089247	0.283493899
RP11-449O16	0.061	0.074112909	0.283561659
ACTBP9	0.061	0.074125785	0.283576113
RP11-108M9	0.061	0.074148867	0.283608103
ACTR3BP6	0.061	0.074191176	0.283638042
AL356693	0.061	0.074198383	0.283638042
EDF1	0.061	0.07421454	0.283665655
PTN	0.061	0.074335715	0.28391277
HMGN1P14	0.061	0.074340768	0.28391277
VGLL2	0.061	0.074400733	0.283955742
ASS1	0.061	0.074407483	0.283958751
GLTSCR2	0.061	0.07441727	0.283973346
MTAPP1	0.061	0.074479218	0.28414986
RN7SL555P	0.061	0.074487392	0.28414986
RAD51B	0.061	0.074528114	0.284282433
CIB4	0.061	0.074552076	0.284307688
INTS1	0.061	0.074566339	0.284307688
GAD2	0.061	0.074596235	0.284307688
RP11-793H4	0.061	0.074602526	0.284307688
LONP1	0.061	0.074607255	0.284307688
RP11-395G17	0.061	0.074642773	0.284355412
RP11-384C12	0.061	0.074665546	0.28439667
GS1-174L6	0.061	0.074719948	0.284503771
AL109659	0.061	0.074774392	0.284583663
CER1	0.061	0.074857069	0.284753338
C2orf76	0.061	0.074939092	0.284891773
CDKN2B	0.061	0.074948936	0.284904471
TSPY14P	0.061	0.074960378	0.284904471
PRKAR1B	0.061	0.074972028	0.284926013
LINC01063	0.061	0.075086818	0.285248455
NCAM1	0.061	0.075111961	0.285275706
C8orf88	0.061	0.075132615	0.285331396
ZNF667	0.061	0.075245192	0.285553649
EVC2	0.061	0.075289196	0.285584466
LCE1A	0.061	0.075319037	0.285652156
BAALC	0.061	0.075352815	0.285712007
RN7SKP238	0.061	0.075361529	0.285722303
RPS3AP47	0.061	0.075381134	0.285773881
CCDC127	0.061	0.075400827	0.285803041
CLDN7	0.060	0.075564202	0.286285599
UQCR11	0.060	0.07560926	0.286365186
PCDHB3	0.060	0.075618544	0.286377577

WWC2-AS1	0.060	0.075686964	0.286591114
RP11-2L8	0.060	0.075710109	0.286633177
ANKRD54P1	0.060	0.075735193	0.286705352
C11orf73	0.060	0.07577228	0.286720063
NAALADL2	0.060	0.075780949	0.286720063
SNORA36B	0.060	0.076094396	0.287516682
HRCT1	0.060	0.07614703	0.287594438
AC005083	0.060	0.076151205	0.287594438
NTPCR	0.060	0.076210699	0.287773485
RPL7P10	0.060	0.0762619	0.287829902
COL4A1	0.060	0.076277675	0.287843817
INHBB	0.060	0.076329437	0.288010397
RNU7-56P	0.060	0.076441207	0.288293836
IRS4	0.060	0.076443416	0.288293836
ACTBP7	0.060	0.076445396	0.288293836
FLJ33581	0.060	0.076451407	0.288293836
RPL39	0.060	0.076528759	0.288560087
ZNF705D	0.060	0.076574526	0.28866669
NDUFB11	0.060	0.076582208	0.288672801
PCNPP3	0.060	0.076684943	0.288880808
LLGL1	0.060	0.076704061	0.288903228
ZNF521	0.060	0.076704882	0.288903228
ABHD17A	0.060	0.076730604	0.288957735
CACNA1S	0.060	0.076761946	0.288984363
HTR1A	0.060	0.07682537	0.289164428
RNA5SP432	0.060	0.076864959	0.28927004
CDH22	0.060	0.076920778	0.289418366
TRADD	0.060	0.076925842	0.289418366
C19orf60	0.060	0.076933887	0.289425769
TMPRSS13	0.060	0.077080822	0.289800855
EIF6	0.060	0.077171054	0.290060786
PLA2G16	0.060	0.077187644	0.290071701
EMP3	0.060	0.077190853	0.290071701
KRT84	0.060	0.077210546	0.290111033
JMJD8	0.060	0.077244769	0.290135071
PGLYRP4	0.060	0.077315802	0.29031218
LEPROT	0.060	0.077330567	0.290344731
PCDHB4	0.060	0.077364632	0.290449735
SCRIB	0.060	0.077465247	0.290689986
IRAK1	0.060	0.077477566	0.290713311
MIR181A1HG	0.060	0.077552719	0.290880718
IRX1	0.060	0.077569649	0.290921305
HECW1	0.060	0.077635859	0.291053691
RNU6ATAC39P	0.060	0.077647725	0.291053691
CTC-535M15	0.060	0.077714599	0.291193083
EEF1B2P6	0.060	0.077778519	0.291270945
PAPSS2	0.060	0.077779072	0.291270945
SH3TC2	0.060	0.07789924	0.291606673
LECT2	0.060	0.077905459	0.291606673
AC016682	0.060	0.077980588	0.291796157
BNIP3L	0.060	0.078010127	0.291860825
PKP2	0.060	0.078017061	0.291863842

MOGS	0.060	0.078087176	0.291989916
ITPKC	0.060	0.078087546	0.291989916
RN7SKP45	0.060	0.078147052	0.292107582
SEC14L3	0.060	0.078156078	0.29210857
NMT2	0.060	0.078245168	0.292326843
SRGAP2	0.060	0.078278262	0.292404611
C20orf166-AS1	0.060	0.07836729	0.292546916
C10orf107	0.060	0.078378203	0.292546916
FLJ31356	0.060	0.078418826	0.29263131
ARVCF	0.060	0.078540638	0.292902275
HN1	0.060	0.078588157	0.292976148
C21orf33	0.060	0.078677957	0.293175534
CSGALNACT1	0.060	0.078708966	0.293231535
DRD2	0.060	0.078797351	0.293468989
RP11-445L6	0.060	0.078926382	0.293742816
SURF2	0.060	0.078952625	0.293794567
PRSS56	0.060	0.078963524	0.293804597
HECW1-IT1	0.060	0.079000205	0.293867409
RPL31P28	0.060	0.079003051	0.293867409
CTD-2353N24	0.060	0.079052756	0.293952367
SVOPL	0.060	0.079087724	0.294021625
NCKAP5	0.060	0.07921	0.294330245
RP11-213H15	0.060	0.07921019	0.294330245
HSPE1P1	0.060	0.079260355	0.294388435
GPC4	0.060	0.079260564	0.294388435
COX7BP2	0.060	0.079281927	0.29442634
GCSHP2	0.060	0.079338918	0.294587562
LINC00303	0.060	0.079364751	0.294620641
RP11-490M8	0.060	0.079426298	0.294797092
PTPLB	0.060	0.079433335	0.294800236
XXbac-BPG27H4	0.060	0.079475408	0.294887447
RP11-344F13	0.060	0.079537062	0.295026791
RP3-340H11	0.060	0.079551176	0.295053646
RP11-575H3	0.060	0.079596558	0.295123337
RBMX2	0.060	0.079676178	0.295172474
DCUN1D5	0.060	0.079701577	0.295197684
SMIM23	0.060	0.079748649	0.295303135
RPL36A-HNRNPH2	0.060	0.079788986	0.295406564
FKBP2	0.060	0.079815014	0.295472684
SLC17A5	0.060	0.07987098	0.295595242
RNA5SP45	0.060	0.079879173	0.29559971
RNU6-433P	0.060	0.079925596	0.295682487
AC007016	0.060	0.079955393	0.295716099
RP11-461F16	0.060	0.080002599	0.2958117
PHGDH	0.060	0.080003296	0.2958117
POFUT2	0.060	0.080035672	0.2958117
TTC23	0.060	0.080035955	0.2958117
MIR663B	0.060	0.080121997	0.29597993
NINJ1	0.060	0.080124073	0.29597993
TMEM110	0.060	0.080143918	0.296030275
ZFYVE21	0.060	0.080161403	0.2960719
RP11-325D8	0.060	0.080187318	0.296121685

CP	0.060	0.080221822	0.296180212
EXT1	0.060	0.080299315	0.296351452
FAM43A	0.060	0.080342488	0.296424794
C19orf48	0.060	0.080362755	0.296424794
TSSC2	0.060	0.080468894	0.2966514
SCARA3	0.059	0.080608375	0.296893904
BTBD16	0.059	0.080648468	0.296995636
EVC	0.059	0.080655577	0.296998853
AC007391	0.059	0.080692289	0.297088098
RP11-348J12	0.059	0.080895142	0.297509164
RP11-447E20	0.059	0.080899821	0.297509164
AC005158	0.059	0.080900355	0.297509164
AC002456	0.059	0.080939197	0.297606046
PLEKHG5	0.059	0.080973847	0.297679762
WDR46	0.059	0.081123771	0.297916714
LINC01208	0.059	0.081193008	0.298022757
RNA5SP239	0.059	0.081196449	0.298022757
ACTG1P14	0.059	0.081255855	0.29811615
BMP2	0.059	0.081281472	0.298135335
CMSS1	0.059	0.081316752	0.298211515
PLXNB3	0.059	0.081386393	0.298397626
TULP2	0.059	0.08139461	0.298397626
MAF	0.059	0.081409266	0.298397626
RP11-632L2	0.059	0.081416459	0.298397626
TOMM7	0.059	0.081418072	0.298397626
PRAME	0.059	0.081423889	0.298397626
CCDC89	0.059	0.081542384	0.298724223
PTPRZ1	0.059	0.081600084	0.298836387
SMIM20	0.059	0.081852251	0.299529556
RP11-426C22	0.059	0.081865903	0.299542984
PLB1	0.059	0.0818685	0.299542984
RP11-227H15	0.059	0.081973185	0.299764809
FAM132B	0.059	0.081994751	0.299797638
RP11-91A15	0.059	0.082114425	0.300166071
LINC01254	0.059	0.082153495	0.300262798
TGFB3	0.059	0.082216881	0.300425301
SPRY1	0.059	0.082257315	0.300503885
PNLIPRP2	0.059	0.082334024	0.300668805
RPS2P1	0.059	0.082370427	0.300778681
PRRG1	0.059	0.082554263	0.301126741
SMIM12	0.059	0.082581371	0.30117949
RPPH1	0.059	0.082605712	0.301198489
BTBD2	0.059	0.082611877	0.301198489
TMC7	0.059	0.082620982	0.301208628
RNF144B	0.059	0.082700928	0.301384737
SLC22A17	0.059	0.082718186	0.301424563
C19orf52	0.059	0.082772109	0.301551843
RP11-643C9	0.059	0.082859392	0.301777492
PROCR	0.059	0.082877517	0.301820422
EEPD1	0.059	0.082898495	0.301873736
OR7G3	0.059	0.082909067	0.301889155
RP11-182L21	0.059	0.083004907	0.302076466

CTB-109A12	0.059	0.083090032	0.302278544
LDLRAD2	0.059	0.083104715	0.302294212
SMOC1	0.059	0.08317939	0.302434142
NTF4	0.059	0.08319719	0.302475767
SLC6A14	0.059	0.083211609	0.302482009
ENTPD3	0.059	0.083300525	0.302708874
CTC-277H1	0.059	0.083325881	0.302745934
SVIL	0.059	0.083336318	0.302750481
RNA5SP289	0.059	0.083370931	0.30285025
LINC00634	0.059	0.08342982	0.302974611
RP11-799N11	0.059	0.08344929	0.303022212
FMO4	0.059	0.08361939	0.303465147
AC091939	0.059	0.083622243	0.303465147
SHISA4	0.059	0.083779731	0.303763524
ST3GAL4-AS1	0.059	0.083796876	0.303797962
ALDH1L1	0.059	0.083806281	0.303808933
RP11-440G9	0.059	0.083909434	0.303992915
PPP2R1A	0.059	0.083914029	0.303992915
BHLHA9	0.059	0.083920861	0.303992915
C1orf198	0.059	0.084157973	0.304550701
ACADS	0.059	0.084207637	0.304675633
MPV17	0.059	0.084272482	0.304826118
PRR5L	0.059	0.084286263	0.304852814
AC004946	0.059	0.084342262	0.305032189
DOCK5	0.059	0.084384555	0.305138804
KB-1410C5	0.059	0.084398942	0.305167659
SERPINB10	0.059	0.084456706	0.305330161
AC022537	0.059	0.084568325	0.305548142
CETN4P	0.059	0.084608026	0.305587555
PRR32	0.059	0.084609998	0.305587555
POMGNT2	0.059	0.084642246	0.305587631
OR2T27	0.059	0.084645599	0.305587631
AL138714	0.059	0.084654113	0.305587631
RP1-32I10	0.059	0.084656353	0.305587631
CHAC1	0.059	0.084701826	0.305625268
EXOSC4	0.059	0.084796156	0.305884006
GGH	0.059	0.084816734	0.305888733
FCGBP	0.059	0.084839889	0.305902749
CTD-2207P18	0.059	0.084861834	0.30595871
CAMTA1	0.059	0.084928203	0.306160963
RPL5P34	0.059	0.084937216	0.306160963
TRIM54	0.059	0.085203852	0.306565224
R3HDM4	0.059	0.085217919	0.306592673
PGBD4P7	0.059	0.085329103	0.30687678
TTTY15	0.059	0.085420737	0.307104234
NDP	0.059	0.085424589	0.307104234
RP11-301O19	0.059	0.085440531	0.307130137
DDX3P1	0.059	0.085546948	0.307404896
SRD5A1P1	0.059	0.085581967	0.307507531
AC023157	0.059	0.085603817	0.307562838
CTD-2006M22	0.058	0.085732049	0.307930642
RP11-436K8	0.058	0.085743881	0.307932353

CENPP	0.058	0.085826411	0.308037263
C5orf38	0.058	0.085841796	0.308069264
OR2W3	0.058	0.085849878	0.308075055
TBC1D26	0.058	0.085861257	0.308092678
GPI	0.058	0.085910653	0.30819869
RNU7-159P	0.058	0.08601368	0.30830804
IL1RAPL2	0.058	0.086035155	0.308317791
RP11-315I14	0.058	0.086093132	0.308333223
DGCR14	0.058	0.086093157	0.308333223
TMEM246	0.058	0.08610227	0.308333223
TYRP1	0.058	0.086115118	0.308333223
CD59	0.058	0.086200764	0.308590182
RBM17P3	0.058	0.086291881	0.308707536
B3GNT8	0.058	0.086338788	0.308782567
RP11-817J15	0.058	0.086378065	0.308853462
UROC1	0.058	0.086411145	0.308895999
SEPHS2P1	0.058	0.086455716	0.308991926
C12orf57	0.058	0.086464729	0.30900095
TRPC6P	0.058	0.086526359	0.309050162
AP5S1	0.058	0.086529264	0.309050162
HEXB	0.058	0.086529666	0.309050162
AZGP1	0.058	0.086548068	0.309050162
AP5Z1	0.058	0.086549879	0.309050162
LINC00911	0.058	0.086648034	0.309238358
NBEAP1	0.058	0.086700352	0.30937871
RN7SL616P	0.058	0.086795139	0.309600966
MRPS15	0.058	0.086814705	0.309647566
EVI5L	0.058	0.086835173	0.309674188
EPHX1	0.058	0.086870068	0.309719911
TFDP3	0.058	0.086880509	0.309719911
NKAIN2	0.058	0.086930189	0.309866152
SSNA1	0.058	0.087153179	0.310436351
ACAD9	0.058	0.08716926	0.310470414
ALKBH3	0.058	0.087190099	0.310521414
MAP3K13	0.058	0.087434801	0.310965396
AP000697	0.058	0.087443933	0.310965396
PYCRL	0.058	0.087491802	0.311070718
RP11-64K10	0.058	0.087559984	0.311151721
C11orf53	0.058	0.087574088	0.311151721
TEX26	0.058	0.087583445	0.311151721
RNU6-1216P	0.058	0.087587678	0.311151721
MAMDC2	0.058	0.087600612	0.311151721
RPL17P41	0.058	0.08767273	0.311262118
SNORD114-6	0.058	0.087681049	0.311262118
RP11-297J22	0.058	0.087689428	0.311262118
DGKB	0.058	0.087692172	0.311262118
PLEKHF1	0.058	0.087775119	0.311401157
NBAT1	0.058	0.087777112	0.311401157
PWP2	0.058	0.087800265	0.311460093
RP11-1152H14	0.058	0.087823723	0.311520104
MYBPHL	0.058	0.087831245	0.311523587
RP11-360O19	0.058	0.087874174	0.311606231

DGCR2	0.058	0.087925457	0.31164886
PHF23	0.058	0.087936662	0.311665381
RN7SKP181	0.058	0.087950028	0.311666366
CTB-118P15	0.058	0.088130853	0.311973689
PACSIN3	0.058	0.088131502	0.311973689
MGP	0.058	0.088139156	0.311973689
LINC01218	0.058	0.088212688	0.31210948
EVX1	0.058	0.088226089	0.312133707
RP11-331K21	0.058	0.088251219	0.312176232
ROM1	0.058	0.088314526	0.312307389
LMO1	0.058	0.088355973	0.312430762
PFN1	0.058	0.088444727	0.312567996
AP000230	0.058	0.088454664	0.312570876
AC004231	0.058	0.088523764	0.312699053
APOBR	0.058	0.088621303	0.312997168
DPY19L1	0.058	0.088643959	0.31304999
AC005703	0.058	0.088649405	0.31304999
TSPY17P	0.058	0.088749076	0.313316847
SPANXC	0.058	0.08876437	0.313316847
ELSPBP1	0.058	0.088764445	0.313316847
RP11-790J24	0.058	0.088915794	0.313665099
CLEC2A	0.058	0.088927082	0.31366899
RGS2	0.058	0.088930068	0.31366899
POMC	0.058	0.088953889	0.313694298
RP11-556O5	0.058	0.089000594	0.313824437
RIN3	0.058	0.089073144	0.313957102
POP7	0.058	0.089077673	0.313957102
AC013275	0.058	0.089094794	0.313994211
FAM43B	0.058	0.089131001	0.314052097
RPS27AP2	0.058	0.089220015	0.314249495
TMEM174	0.058	0.089251575	0.314279591
AP4M1	0.058	0.089254955	0.314279591
CYYR1-AS1	0.058	0.089315133	0.314421748
FOXRED1	0.058	0.089331993	0.314426909
KRT18P53	0.058	0.089464212	0.314737186
OR4F3	0.058	0.089490744	0.314783047
S1PR2	0.058	0.089511025	0.31480886
KCND2	0.058	0.089543992	0.314855055
SSBP4	0.058	0.089598994	0.314978691
MUC7	0.058	0.089606122	0.314980497
CDK2AP1	0.058	0.089624747	0.31499044
HM13	0.058	0.089693761	0.315113975
RP11-707G18	0.058	0.089838214	0.315433418
KB-1184D12	0.058	0.089846938	0.315433418
RP11-381N20	0.058	0.089855071	0.315433418
CTC-498J12	0.058	0.089887149	0.315502635
LAD1	0.058	0.089918065	0.315567077
CD81-AS1	0.058	0.089922881	0.315567077
RP1-53O8	0.058	0.089925386	0.315567077
MIR6726	0.058	0.090036021	0.315769189
RP11-798L4	0.058	0.090053439	0.315807021
UBALD1	0.058	0.090099495	0.315922008

RNU1-14P	0.058	0.090112473	0.315925084
CATSPER1	0.058	0.090220577	0.316230159
SHC2	0.058	0.090254482	0.31632572
KCNJ2	0.058	0.090271231	0.316335498
OFD1P1Y	0.058	0.090284328	0.316335498
CDY13P	0.058	0.090284328	0.316335498
RNU6-894P	0.058	0.090284328	0.316335498
MIA	0.058	0.090556322	0.317032129
C14orf166	0.058	0.09056729	0.317032129
C1QL4	0.058	0.090575853	0.317032129
RSPH6A	0.058	0.090588094	0.317042823
AC006077	0.058	0.090661871	0.317182842
PNPLA4P1	0.058	0.090780831	0.317447102
MIR1267	0.058	0.090798576	0.317447102
RP11-556K13	0.058	0.090803067	0.317447102
RP11-334G8	0.058	0.090894599	0.317649125
AC079756	0.058	0.091033596	0.317968993
RP11-10B2	0.058	0.091037126	0.317968993
IFNA5	0.058	0.091084605	0.318025922
RP11-255M6	0.058	0.091122132	0.318032666
RN7SL251P	0.058	0.091137259	0.318043547
RP11-813I20	0.058	0.091138605	0.318043547
ARHGEF19	0.058	0.091158811	0.318067449
C16orf13	0.058	0.091183245	0.318080522
WNT7A	0.058	0.091191835	0.318080522
TNFRSF10C	0.057	0.091228142	0.318146204
MFSD3	0.057	0.091321454	0.318308468
RP1-144C9	0.057	0.091342892	0.318336597
RP11-696F12	0.057	0.09135077	0.318340758
HS6ST1	0.057	0.091376806	0.318399876
PCDHA7	0.057	0.091381105	0.318399876
RP11-14C10	0.057	0.091401371	0.31844719
ATOX1	0.057	0.091419993	0.318483784
PCDH17	0.057	0.091494111	0.318636197
PDXK	0.057	0.091665871	0.318948745
FPGS	0.057	0.091700001	0.319020887
MBOAT7	0.057	0.091857721	0.319404597
RP11-438P9	0.057	0.091901279	0.319465247
TPT1P4	0.057	0.091923995	0.319466081
PCDHGA9	0.057	0.091935005	0.319466081
SRPK3	0.057	0.091935291	0.319466081
TOP1MT	0.057	0.092007842	0.319648223
RNU1-119P	0.057	0.092106318	0.319897003
RP5-998H6	0.057	0.092132758	0.319950311
RP11-332E4	0.057	0.09216919	0.320022017
RP11-322E11	0.057	0.092262025	0.320227626
DSG3	0.057	0.092305596	0.320355506
RAB31	0.057	0.092321596	0.320372325
LRAT	0.057	0.092381127	0.32044738
LCE2A	0.057	0.092501755	0.32073584
WWTR1-IT1	0.057	0.092567503	0.32082418
AC069368	0.057	0.092588523	0.320846447

GS1-44D20	0.057	0.092658296	0.321032171
AC000089	0.057	0.092778094	0.321271555
RNU7-93P	0.057	0.092791368	0.321271555
RP5-1116H23	0.057	0.092802128	0.321271555
CTB-111H14	0.057	0.092805637	0.321271555
NDUFB5	0.057	0.092815428	0.321282099
RP11-416L21	0.057	0.092890053	0.321517047
RNU6-308P	0.057	0.092925364	0.321615894
CTD-2014N11	0.057	0.092940131	0.321628367
ARMCX6	0.057	0.092945713	0.321628367
RP3-390M24	0.057	0.092960727	0.321628367
RP1-16A9	0.057	0.092969486	0.321628367
WDR41	0.057	0.093006222	0.321685353
CYP4Z2P	0.057	0.093030485	0.321722546
CTD-2337I7	0.057	0.093046331	0.321753984
OR10R3P	0.057	0.093064434	0.321793219
LINC01220	0.057	0.093085842	0.321800355
RP3-481F12	0.057	0.093121282	0.321872946
TBXA2R	0.057	0.093390461	0.322545867
KCNK1	0.057	0.093601263	0.323039665
RN7SKP43	0.057	0.093705185	0.323210954
SLC7A10	0.057	0.093758214	0.32330636
HBB	0.057	0.093762779	0.32330636
SYCP1	0.057	0.093779572	0.32330636
RP11-101E5	0.057	0.093886336	0.323460974
SAMD5	0.057	0.093949651	0.323608884
S100A4	0.057	0.094022184	0.323690201
GNA11	0.057	0.094031213	0.323690201
NDUFA1	0.057	0.09405981	0.323730784
MIR548H4	0.057	0.094165823	0.323978593
RNU7-57P	0.057	0.094165823	0.323978593
LINC00479	0.057	0.094240319	0.324136627
RP11-292B8	0.057	0.094245785	0.324136627
DHRS3	0.057	0.094332158	0.32434
RP11-431K24	0.057	0.094402061	0.324486645
PRR7	0.057	0.09444602	0.324567842
EGR1	0.057	0.094491013	0.324675237
RP3-523C21	0.057	0.094592681	0.324929258
PRR35	0.057	0.094606519	0.324929258
AC016710	0.057	0.0946102	0.324929258
ATP5F1P1	0.057	0.09461086	0.324929258
RP11-4N23	0.057	0.09462461	0.324946738
AC000111	0.057	0.094659216	0.325009499
CD163L1	0.057	0.094670182	0.325009499
KRT9	0.057	0.094677765	0.325012103
KCNJ4	0.057	0.094741836	0.32513022
TRIM51BP	0.057	0.094746306	0.32513022
ELOF1	0.057	0.094822674	0.325321964
RP11-432I13	0.057	0.094866679	0.325391231
AC004054	0.057	0.094877024	0.325391231
COL25A1	0.057	0.09499229	0.325757398
COX7A2L	0.057	0.095242551	0.326386314

RP11-170M17	0.057	0.095267424	0.326448061
USB1	0.057	0.095291324	0.326506467
RP11-763E3	0.057	0.095324249	0.32655443
KCNS2	0.057	0.095325892	0.32655443
MRGPRE	0.057	0.095390669	0.326647917
ROR1	0.057	0.095463872	0.326839037
MLLT11	0.057	0.095498586	0.326852522
NXF2B	0.057	0.095590535	0.326932985
CPAMD8	0.057	0.095622179	0.326981442
SPRR2A	0.057	0.095640272	0.327019833
IFITM10	0.057	0.095657048	0.327053712
NPM2	0.057	0.095693105	0.327153507
PIGQ	0.057	0.095739902	0.32721954
STYK1	0.057	0.095910259	0.327613706
BGLAP	0.057	0.096013547	0.32787246
TMEM133	0.057	0.096057238	0.327927609
RN7SKP297	0.057	0.096105485	0.327951273
FXYD1	0.057	0.096205038	0.32822044
RP11-47P18	0.057	0.096244167	0.328306902
C2orf71	0.057	0.096298051	0.328379773
RP11-212D19	0.057	0.096300004	0.328379773
UPK2	0.057	0.096351894	0.328439141
MIR4644	0.057	0.096422438	0.328609049
MRPL12	0.057	0.096474125	0.328714633
RP11-20A20	0.057	0.096577538	0.328949323
AC113171	0.057	0.096587714	0.328960458
IFNA11P	0.057	0.096608003	0.328975669
NEXN-AS1	0.057	0.096613078	0.328975669
RP11-457I16	0.057	0.096626717	0.328975669
MYO3A	0.057	0.096713927	0.329178461
ALX3	0.057	0.096780733	0.32935877
AC011294	0.057	0.096845724	0.329485947
EIF3B	0.057	0.096947299	0.329691821
CTD-2015A6	0.057	0.096959073	0.329691821
RNA5SP503	0.057	0.096963165	0.329691821
FAS-AS1	0.056	0.096994032	0.329691821
TMEM222	0.056	0.097005033	0.329691821
HPGDS	0.056	0.097008009	0.329691821
KNDC1	0.056	0.097017017	0.329691821
RP11-515C16	0.056	0.097040482	0.329720134
RP11-114H7	0.056	0.097040735	0.329720134
MID1IP1	0.056	0.097046147	0.329720134
RP11-150D5	0.056	0.097065324	0.329728233
PLEKHH3	0.056	0.0970693	0.329728233
LSM4	0.056	0.097086392	0.329757907
AC092043	0.056	0.097091883	0.329757907
RP11-70D24	0.056	0.097178731	0.329911713
MIR512-1	0.056	0.097366229	0.330430481
PAQR7	0.056	0.097387385	0.330478731
SPNS2	0.056	0.097402311	0.330505831
RP11-361D15	0.056	0.09741837	0.330513229
CYP1B1	0.056	0.097622978	0.331057732

PRSS8	0.056	0.097642585	0.331058448
RPL7P12	0.056	0.097646998	0.331058448
RNU7-186P	0.056	0.097659837	0.331058448
CLDN23	0.056	0.097693888	0.331059713
C16orf45	0.056	0.097715391	0.331061962
SPAG7	0.056	0.097742774	0.331080638
AC010127	0.056	0.097745498	0.331080638
SCUBE3	0.056	0.097784136	0.331164408
RN7SL679P	0.056	0.097801332	0.331188049
HNRNPA1P74	0.056	0.097809704	0.331188049
RRAS2	0.056	0.09785736	0.331294603
AC005682	0.056	0.097882998	0.33130943
AL050316	0.056	0.09793817	0.331426832
MT2A	0.056	0.097968276	0.331458059
TIMM50	0.056	0.098038164	0.331600287
CACNA1C	0.056	0.098079071	0.331667985
RGS6	0.056	0.098129656	0.33180325
CTC-786C10	0.056	0.098186391	0.331883567
RN7SL208P	0.056	0.098197455	0.331883567
MRPS17P1	0.056	0.098227174	0.331956688
RP11-51B23	0.056	0.098244061	0.331990202
RP13-212L9	0.056	0.098281206	0.332048194
RRP9	0.056	0.098404585	0.332344006
EPHX3	0.056	0.098469702	0.332516768
RN7SL705P	0.056	0.09848578	0.332547481
RP11-55L4	0.056	0.098493304	0.332549312
RP11-701B6	0.056	0.098528188	0.332596359
LINC00475	0.056	0.098575089	0.332687504
AF013593	0.056	0.098576145	0.332687504
RP11-395D3	0.056	0.09863683	0.332772816
AKAP12	0.056	0.098655875	0.332772816
SLC35B2	0.056	0.09866403	0.332772816
LHCGR	0.056	0.098670698	0.332772816
IL13RA1	0.056	0.098707826	0.33282532
RP11-557H15	0.056	0.098768554	0.332912239
CTD-2282P23	0.056	0.098831549	0.333077424
RP11-598F17	0.056	0.09886217	0.333135032
RNA5SP410	0.056	0.098872598	0.333135032
TRAPPC5	0.056	0.098893799	0.333145775
RN7SL356P	0.056	0.09897848	0.333360303
EXOC3-AS1	0.056	0.099089802	0.333564095
HEPHL1	0.056	0.099146464	0.333625644
RP11-74C1	0.056	0.099147206	0.333625644
KRTAP3-1	0.056	0.099148327	0.333625644
ANAPC15	0.056	0.099166384	0.333662829
RP11-626G3	0.056	0.099402219	0.334273204
RNU6-923P	0.056	0.099403015	0.334273204
RABEP2	0.056	0.099403939	0.334273204
SGK223	0.056	0.099449458	0.334363808
ZBTB17	0.056	0.099465984	0.334363808
RP4-798P15	0.056	0.099502418	0.334462675
LINC01468	0.056	0.0995341	0.334494049

C11orf98	0.056	0.099557535	0.33450299
RNU6ATAC31P	0.056	0.099563575	0.33450299
AC107983	0.056	0.099609076	0.334608651
AC004000	0.056	0.099619655	0.334620589
RP11-742D12	0.056	0.099656334	0.334649383
RP11-549L6	0.056	0.099677165	0.334695736
THEGL	0.056	0.09970658	0.33472371
COMTD1	0.056	0.099763601	0.33479714
RP11-298C3	0.056	0.099770926	0.334798133
ZNF890P	0.056	0.099795949	0.334858508
MTND4P30	0.056	0.099811101	0.334885758
NOP16	0.056	0.099825599	0.334910807
ATP13A4-AS1	0.056	0.100032228	0.33539826
AC098587	0.056	0.100034272	0.33539826
DYSF	0.056	0.100092543	0.335451941
CXCL5	0.056	0.100105048	0.335470247
AC016708	0.056	0.100145887	0.335559881
RN7SL560P	0.056	0.100153527	0.335561873
SLC35F6	0.056	0.100248117	0.33569249
OR51E2	0.056	0.100248898	0.33569249
WASH4P	0.056	0.100291228	0.335763412
COX5BP1	0.056	0.10030429	0.335783538
OXCT2	0.056	0.100326229	0.335833378
KRT8P12	0.056	0.100362885	0.335886639
SNRPD2	0.056	0.100379532	0.335886639
AC005592	0.056	0.100386238	0.335886639
ADAMTS3	0.056	0.100392443	0.335886639
ZNF662	0.056	0.100398559	0.335886639
DPM2	0.056	0.100557574	0.336183993
RNU6-382P	0.056	0.100652841	0.336398054
ARSK	0.056	0.100760173	0.336573856
SNORA33	0.056	0.100876999	0.336754611
LSM3P4	0.056	0.100877192	0.336754611
ZNF185	0.056	0.100907246	0.33683133
SDHA	0.056	0.100946942	0.336916609
RP5-968J1	0.056	0.101115545	0.337321522
MIR539	0.056	0.101299042	0.337651368
NKX2-2	0.056	0.101316869	0.337654365
MEGF10	0.056	0.10160629	0.33833481
ICAM5	0.056	0.101679601	0.338502543
GOLGA6L6	0.056	0.101685092	0.338502543
RNU2-57P	0.056	0.101716057	0.338517643
RPS17	0.056	0.101718059	0.338517643
SNTB1	0.056	0.101802665	0.338728205
GCNT6	0.056	0.101964679	0.339111826
ENOX1-AS1	0.056	0.101982041	0.339111826
CLTA	0.056	0.102114897	0.339435107
C19orf26	0.056	0.10214831	0.339498782
NKX2-1	0.056	0.10217624	0.339544223
MIR649	0.056	0.10234806	0.339809179
RNF126P1	0.056	0.102361606	0.339809179
LRRN3	0.056	0.102374272	0.339809179

RPL41P1	0.056	0.102377263	0.339809179
RP11-11N5	0.056	0.102396816	0.339850393
RP11-1398P2	0.056	0.102430537	0.339914938
ISPD	0.056	0.102507898	0.34007609
LINC00578	0.056	0.102514801	0.34007609
TPD52L2	0.056	0.102534387	0.340098652
RP11-109E12	0.056	0.102581329	0.340202004
ZNF771	0.056	0.102672214	0.340380714
SIK1	0.056	0.102755831	0.340504804
CD99P1	0.056	0.102792183	0.340545584
BEST3	0.056	0.10288642	0.340786671
RN7SL181P	0.056	0.102920072	0.340832538
PEF1	0.056	0.102955128	0.340895708
RN7SL401P	0.055	0.103056792	0.341161175
RP11-97N19	0.055	0.103082613	0.341195743
SCARNA2	0.055	0.1031333	0.341248415
MIR7855	0.055	0.103175246	0.341339777
RP11-706C16	0.055	0.103234902	0.341400426
SOSTDC1	0.055	0.103236192	0.341400426
BAMBI	0.055	0.103262286	0.341414405
MAMLD1	0.055	0.103352084	0.341663753
SCRN2	0.055	0.103436376	0.341777742
AC093897	0.055	0.103443974	0.341777742
RP11-72K17	0.055	0.103459787	0.341806277
AC010980	0.055	0.103483898	0.341808853
DHRS7B	0.055	0.103489274	0.341808853
AC079780	0.055	0.103502356	0.341813945
RP11-280K24	0.055	0.103524843	0.341855212
ADAMTS10	0.055	0.103598504	0.341965078
B4GALNT1	0.055	0.103608375	0.341965078
ACSF2	0.055	0.103689114	0.342169246
BHLHE41	0.055	0.103705035	0.342189256
RP11-34F13	0.055	0.103730846	0.342210634
CCDC3	0.055	0.103740255	0.342210634
OR7E89P	0.055	0.103759516	0.342236511
HOXD1	0.055	0.103802417	0.342320849
RP11-556O9	0.055	0.103903323	0.342465743
RP11-451J24	0.055	0.103903818	0.342465743
CTB-78H18	0.055	0.104100684	0.342931807
LRRTM3	0.055	0.104133342	0.342937261
UBE2SP1	0.055	0.104226538	0.34307812
CTC-552D5	0.055	0.104474836	0.343444436
PTGER3	0.055	0.104534803	0.343564509
RP11-344B2	0.055	0.104540216	0.343564509
HMBS	0.055	0.104583584	0.343659608
TMEM255B	0.055	0.104595285	0.343674344
RP11-247A12	0.055	0.104640108	0.343721676
LINC01093	0.055	0.104645224	0.343721676
RP11-120I21	0.055	0.104645774	0.343721676
AF064858	0.055	0.10466062	0.343723028
RP11-385F7	0.055	0.104696539	0.343794031
MEIS3	0.055	0.104751002	0.343868009

RP11-93O17	0.055	0.10479783	0.343944043
PARP4P3	0.055	0.104867445	0.344050979
TRIM10	0.055	0.104926341	0.344152229
PANX1	0.055	0.105037744	0.344391619
PPP1R15A	0.055	0.105106326	0.344484109
RP11-439H9	0.055	0.105145473	0.344555075
RP11-49G2	0.055	0.105161782	0.344584809
CSNK1G2	0.055	0.105202565	0.344694726
GRIP1	0.055	0.105265233	0.344876334
CFL1	0.055	0.105328991	0.344962357
PHLDA1	0.055	0.105393838	0.34509521
SOD3	0.055	0.105397252	0.34509521
RYK	0.055	0.10545542	0.345200633
DSCAM	0.055	0.10546569	0.345200633
F11R	0.055	0.105545076	0.345365532
SMARCA1	0.055	0.105567116	0.345413921
ESRRG	0.055	0.105577718	0.345424881
WBSCR16	0.055	0.105604251	0.345487956
FBN3	0.055	0.105683464	0.345627954
RP11-697E14	0.055	0.105688854	0.345627954
PLS3-AS1	0.055	0.105747812	0.345720144
RP11-58C22	0.055	0.105847986	0.345839265
NDUFV1	0.055	0.105849602	0.345839265
AP2A1	0.055	0.105912457	0.345903992
RPL31	0.055	0.105993079	0.346107127
KRTAP19-5	0.055	0.105996988	0.346107127
SNRPGP11	0.055	0.106021322	0.34616285
CTC-293G12	0.055	0.106046296	0.346196926
TBX15	0.055	0.106116351	0.346354403
DMRT2	0.055	0.106185093	0.346415725
RP11-193J6	0.055	0.106258775	0.346510556
UBTD2	0.055	0.106267342	0.346514768
U8	0.055	0.106307626	0.346552313
RP1-300G12	0.055	0.106310642	0.346552313
RP11-254I22	0.055	0.106315238	0.346552313
GS1-57L11	0.055	0.106371901	0.346665836
LRCH2	0.055	0.106395255	0.346718221
LINC01377	0.055	0.106425271	0.346756565
RP11-21L1	0.055	0.106490017	0.34688462
VCY	0.055	0.106557853	0.347050953
ZNF219	0.055	0.106574466	0.347064758
ADH1C	0.055	0.106617585	0.34711024
FAM126A	0.055	0.106748089	0.347463862
SPTSSA	0.055	0.106838376	0.347543974
RP11-403P13	0.055	0.10686322	0.347583601
SPATA16	0.055	0.106954612	0.347785774
FLJ40288	0.055	0.106988862	0.34783654
TMEM239	0.055	0.10701204	0.34783654
TMEM234	0.055	0.107085277	0.347966875
PALM2-AKAP2	0.055	0.107233007	0.348288121
GNAT3	0.055	0.107454802	0.348858204
RNA5SP443	0.055	0.107469343	0.348864574

RPS20P2	0.055	0.107531911	0.348898615
DUSP27	0.055	0.107532037	0.348898615
RP11-665C16	0.055	0.107640426	0.349166463
TFG	0.055	0.107645058	0.349166463
MIR608	0.055	0.107702923	0.349330368
SERPINA3	0.055	0.107738158	0.349420856
RP11-565A3	0.055	0.107926776	0.349748338
KRTAP1-5	0.055	0.107986184	0.349866882
ELOVL1	0.055	0.107995044	0.349866882
TPSAB1	0.055	0.108032131	0.349945338
RNU7-119P	0.055	0.108106033	0.350082373
LRRC42	0.055	0.108170734	0.350233934
MIR3913-2	0.055	0.10818141	0.350238325
LL22NC03-102D1	0.055	0.108215524	0.350266604
MIR873	0.055	0.108216425	0.350266604
MIR6075	0.055	0.108219562	0.350266604
ATOH1	0.055	0.108276756	0.350427907
AC006288	0.055	0.108370407	0.350672518
MSN	0.055	0.10839575	0.350681429
DNAJB2	0.055	0.108435837	0.350713279
RP11-146G7	0.055	0.108441859	0.350713279
TLX3	0.055	0.108458479	0.350713279
RP11-185B14	0.055	0.108497478	0.350808511
RP11-96H17	0.055	0.108552666	0.350889248
FAM198A	0.055	0.108566653	0.350889248
ISOC2	0.055	0.108710259	0.351186564
KRT8P38	0.055	0.108747583	0.351235666
PCDHA5	0.055	0.108904933	0.351568333
AL596330	0.055	0.109046139	0.351889726
RP4-797C5	0.055	0.109060634	0.351912658
RP11-168P13	0.055	0.109095039	0.351999826
LINC00506	0.055	0.109166834	0.352103443
PPP1R12C	0.055	0.109171511	0.352103443
CBLC	0.055	0.109222841	0.352192291
FOLR1	0.055	0.109228638	0.352192291
PKIA	0.055	0.109238222	0.352194805
OR51B8P	0.055	0.109244207	0.352194805
RP11-717I24	0.055	0.109256019	0.352209045
KDM4D	0.055	0.109284369	0.352268284
KIFAP3	0.055	0.10930339	0.352268284
AP000320	0.055	0.109303981	0.352268284
RNU6-1	0.055	0.109382434	0.352473423
RP11-245P10	0.054	0.109427571	0.352547313
HMGB1P36	0.054	0.109558311	0.352784362
RP11-382D12	0.054	0.109560407	0.352784362
HPD	0.054	0.109589863	0.352831501
PTGS2	0.054	0.109604918	0.352856121
RP11-433C9	0.054	0.109762149	0.353276889
FOXP2	0.054	0.109765289	0.353276889
DDA1	0.054	0.10981757	0.353296849
VMP1	0.054	0.109835935	0.353296849
TFEB	0.054	0.109848827	0.353296849

CTD-3001H11	0.054	0.10986744	0.353319146
SYNPR	0.054	0.109887332	0.353346546
TOMM40L	0.054	0.109890798	0.353346546
AC002465	0.054	0.109919102	0.353365985
PSMD9	0.054	0.109943857	0.35337401
CFDP1	0.054	0.109966742	0.353423715
OR7E158P	0.054	0.110067584	0.353628495
RP11-589N15	0.054	0.110120827	0.35372797
ZFY-AS1	0.054	0.110129815	0.353732984
FAM118B	0.054	0.110233971	0.353924318
SLC25A39P2	0.054	0.110279963	0.353993177
MAP2K2	0.054	0.110329385	0.354018586
PGLYRP1	0.054	0.110337528	0.354018586
C11orf68	0.054	0.110442943	0.354197098
RPL7P35	0.054	0.110484875	0.354197098
GSC	0.054	0.11048888	0.354197098
PAF1	0.054	0.110497923	0.35419872
IFITM2	0.054	0.110521266	0.354249701
NFYC	0.054	0.110552069	0.354324589
B3GNT4	0.054	0.110571141	0.354338026
LINC01239	0.054	0.110587014	0.354365049
AKR1B1	0.054	0.1108519	0.354998887
PFKL	0.054	0.110862559	0.35500915
SRXN1	0.054	0.11087763	0.355013798
GARS	0.054	0.110890864	0.355013798
SC5D	0.054	0.11090128	0.355013798
HMGB1P15	0.054	0.111024221	0.355296394
RP11-119J18	0.054	0.111051773	0.355328369
PSMC1P9	0.054	0.111063458	0.355341888
GJB4	0.054	0.111086166	0.355388535
RP11-46A10	0.054	0.111118122	0.355445148
RP11-634H22	0.054	0.111204437	0.355581865
ARMC10	0.054	0.111296334	0.355800141
TBC1D25	0.054	0.111332717	0.355866871
C1QTNF1-AS1	0.054	0.111349825	0.355866871
TMEM177	0.054	0.111358288	0.355866871
RP3-470B24	0.054	0.111362039	0.355866871
RAB11B	0.054	0.111396285	0.355922139
AGRP	0.054	0.11144654	0.355942886
WT1-AS_2	0.054	0.111453906	0.355942886
ATP6V0E1	0.054	0.111458775	0.355942886
GNAS	0.054	0.11150283	0.356006253
RP11-483K5	0.054	0.111510509	0.356006904
RPS20P35	0.054	0.111625533	0.356326359
VAX2	0.054	0.111665878	0.356405509
FBXW9	0.054	0.111672778	0.356405509
OR7E102P	0.054	0.111722312	0.356471402
RP11-459D20	0.054	0.111777259	0.356619475
ARSA	0.054	0.111823069	0.356737344
RP11-1007G5	0.054	0.111877958	0.356821234
RP11-479I16	0.054	0.111907202	0.356866708
TMOD1	0.054	0.111916233	0.356868097

FRK	0.054	0.111941169	0.356868097
RN7SL815P	0.054	0.112051274	0.357182655
MED10	0.054	0.112127342	0.35734325
CTA-331P3	0.054	0.112131666	0.35734325
FABP5P1	0.054	0.112148155	0.357347974
DIO3OS	0.054	0.112172425	0.357378923
SNORD114-3	0.054	0.112224873	0.357520671
SLC39A4	0.054	0.112271309	0.357572931
TARID	0.054	0.112299532	0.357618916
WDR91	0.054	0.112379131	0.357724961
RPL7P44	0.054	0.112455633	0.357891104
PITPNM3	0.054	0.112459277	0.357891104
RALA	0.054	0.112518889	0.35798173
SGTA	0.054	0.112527442	0.35798173
C20orf85	0.054	0.112587333	0.358090535
PYGO1	0.054	0.112763794	0.358374564
NPHP3-AS1	0.054	0.112778933	0.358398762
DAG1	0.054	0.112849498	0.358437028
LINC00302	0.054	0.112917077	0.358437028
RP11-352D3	0.054	0.112955768	0.358437028
OAZ1	0.054	0.112974544	0.358437028
HOXA4	0.054	0.113021665	0.358437028
PLEKHG4B	0.054	0.113035117	0.358437028
RP11-3G21	0.054	0.113125029	0.358437028
RN7SL418P	0.054	0.113166697	0.358437028
MROH9	0.054	0.113169187	0.358437028
AC005754	0.054	0.113223825	0.358437028
AC022819	0.054	0.113330422	0.358437028
SCN5A	0.054	0.113351364	0.358437028
RPL17P1	0.054	0.113375686	0.358437028
BBS9	0.054	0.113481059	0.358437028
C19orf33	0.054	0.11350215	0.358437028
RNY3P8	0.054	0.113536364	0.358437028
RP11-995C19	0.054	0.113560353	0.358437028
DDAH2	0.054	0.113597412	0.358437028
MYH10	0.054	0.113723359	0.358437028
AC100757	0.054	0.113770065	0.358437028
RHBDF1	0.054	0.113827148	0.358571704
ARPC5	0.054	0.113920872	0.358805905
RP9P	0.054	0.113957177	0.358815183
NDUFB6	0.054	0.114038707	0.35892613
RGMA	0.054	0.114142346	0.359089605
RP11-123J14	0.054	0.114191421	0.35919654
TBX2	0.054	0.114216079	0.359226652
LINC01583	0.054	0.114296814	0.359355708
CCL7	0.054	0.114303563	0.359355708
LGI3	0.054	0.114326385	0.35936
RPS6	0.054	0.114348967	0.359383546
CCT3	0.054	0.114410496	0.359505752
RP11-336A10	0.054	0.114517573	0.359763769
SNORD115-1	0.054	0.11453747	0.359763769
DDOST	0.054	0.114672723	0.360044682

AC024592	0.054	0.114691138	0.360069371
SUGT1P	0.054	0.114829176	0.360375772
AC016730	0.054	0.114916938	0.360526249
DARS-AS1	0.054	0.11494828	0.360577074
AL022344	0.054	0.115197466	0.361168435
LRP12	0.054	0.115255175	0.361187035
CTD-2026G22	0.054	0.115274619	0.361187035
AC079776	0.054	0.115356874	0.361304064
RNA5SP305	0.054	0.115362104	0.361304064
SRF	0.054	0.115432007	0.361380412
AC004012	0.054	0.115459721	0.361423722
snoMBII-202	0.054	0.115515505	0.361546755
SLC2A9	0.054	0.115546132	0.361618848
PEA15	0.054	0.115599555	0.361678494
AP003900	0.054	0.115655155	0.361724539
CALML6	0.054	0.115655852	0.361724539
RHOQ	0.054	0.115668199	0.361739399
LINC00982	0.054	0.115693193	0.361770055
RP11-59H7	0.054	0.115742818	0.361893208
FGFBP1	0.054	0.115961318	0.362275481
RP11-561I11	0.054	0.115985982	0.362303456
SLC38A11	0.054	0.115993094	0.362303456
RNU6-806P	0.054	0.116032336	0.362363755
C2CD4B	0.054	0.116051549	0.362375876
AC010969	0.054	0.11608427	0.362445623
MIR152	0.053	0.116347425	0.363029274
RP11-93M12	0.053	0.116380988	0.363086422
ARF4	0.053	0.11649238	0.363315599
RP11-594C13	0.053	0.116492589	0.363315599
TSPY11P	0.053	0.116548077	0.363417262
SLC25A18	0.053	0.116742265	0.363728768
SLC45A2	0.053	0.11677699	0.363728768
RNU6-229P	0.053	0.116777308	0.363728768
MTCL1P1	0.053	0.116782542	0.363728768
RP11-538I12	0.053	0.11679983	0.363728768
ABHD17AP9	0.053	0.116925251	0.363930593
ACADVL	0.053	0.116997428	0.364031914
THEM7P	0.053	0.117005771	0.364034091
SLC39A14	0.053	0.117028875	0.364082188
TRIM58	0.053	0.117055844	0.364090005
ROPN1	0.053	0.117133004	0.364239591
ALDH1A2	0.053	0.117222172	0.364398463
SIVA1	0.053	0.117232671	0.364398463
CEBPE	0.053	0.117425695	0.364698284
PRKRIRP6	0.053	0.117432661	0.364698284
SYCE1	0.053	0.117433659	0.364698284
GAPDHP28	0.053	0.11752721	0.364917427
RP11-265D20	0.053	0.11755507	0.364936083
AFAP1	0.053	0.117555757	0.364936083
RAC1P2	0.053	0.117604518	0.365014686
LINC00555	0.053	0.11769481	0.365247021
SNORD28	0.053	0.117729006	0.365259994

NCAM1-AS1	0.053	0.117865399	0.365609962
PDGFRA	0.053	0.117937538	0.365786085
RPP40	0.053	0.117973042	0.365811379
AC060835	0.053	0.118118314	0.366013074
ABCB5	0.053	0.118219566	0.366126957
AP001347	0.053	0.118224253	0.366126957
BOLA3	0.053	0.118284961	0.366244546
RP11-115A14	0.053	0.118292982	0.366244546
XPR1	0.053	0.118337569	0.366329162
CALML3	0.053	0.118345182	0.366329162
BMS1P19	0.053	0.118358136	0.366329162
RP11-354K1	0.053	0.118379023	0.36634559
MAN1A2P1	0.053	0.118379462	0.36634559
AC098591	0.053	0.118432948	0.366415877
RP1-161N10	0.053	0.118553314	0.366740627
LINC00222	0.053	0.118588173	0.366800812
TMPRSS12	0.053	0.118603509	0.366805651
FOXCUT	0.053	0.118664803	0.366918689
WIZ	0.053	0.118755731	0.367104512
MED8	0.053	0.11876556	0.367111068
NTSR1	0.053	0.118805564	0.367163233
CCHCR1	0.053	0.118842271	0.367213169
RPS15AP1	0.053	0.1188732	0.367229285
RP11-541F9	0.053	0.119069027	0.367605128
GLYATL1P1	0.053	0.119069349	0.367605128
ALK	0.053	0.119096511	0.367632998
RP11-659P15	0.053	0.119145477	0.367736481
ANXA4	0.053	0.119251448	0.367992013
TMEM102	0.053	0.119267667	0.368018217
GABRR1	0.053	0.119396793	0.368297347
SUSD5	0.053	0.11945619	0.3683546
RP11-15J10	0.053	0.119469492	0.3683546
H2BFM	0.053	0.119580868	0.368554844
PCDH18	0.053	0.119598515	0.368561926
ZDHHC12	0.053	0.119700108	0.36870761
SLCO6A1	0.053	0.119720457	0.368746442
FAM84A	0.053	0.119754119	0.368786716
SLC9A4	0.053	0.119837899	0.368988705
DEFA6	0.053	0.119893646	0.369051043
RNA5SP293	0.053	0.119966216	0.369203737
PFKFB2	0.053	0.119969703	0.369203737
AC005281	0.053	0.12010983	0.369539462
SRD5A1	0.053	0.120144867	0.369599508
NAT2	0.053	0.120202952	0.369682681
RPL18AP3	0.053	0.120276339	0.369836735
AC016747	0.053	0.12030782	0.369863444
MKRN4P	0.053	0.120402683	0.369994172
BACH1-IT1	0.053	0.120420763	0.369994172
SLC25A3P1	0.053	0.120534316	0.370301284
GGT2	0.053	0.12053634	0.370301284
ITGBL1	0.053	0.120614346	0.370445569
RP11-84A12	0.053	0.120646478	0.370496473

PPP4R1-AS1	0.053	0.120769799	0.370755653
SNORD114-11	0.053	0.120823814	0.370897571
RP11-227F19	0.053	0.120860039	0.37096095
KIAA2013	0.053	0.12093932	0.371113942
PSMB4	0.053	0.120956637	0.371113942
MIR3648-2	0.053	0.121035877	0.371309223
GALE	0.053	0.121064775	0.371373957
HTR3C	0.053	0.121080073	0.371396963
GPR85	0.053	0.121159853	0.371569884
FBLN7	0.053	0.121242239	0.371798604
PGAM1	0.053	0.121279044	0.371851737
RP11-205M3	0.053	0.121291107	0.371852692
CTD-2544H17	0.053	0.12142722	0.372126259
POLR3D	0.053	0.121441487	0.372144676
TANC1	0.053	0.121486632	0.372210817
PDE11A	0.053	0.121610686	0.372426433
CPA1	0.053	0.121617127	0.372426433
CEP170B	0.053	0.121797743	0.372630574
GJB5	0.053	0.121818619	0.372630574
ZSWIM5P3	0.053	0.121865382	0.372672952
AC133785	0.053	0.121912009	0.372735702
CCL20	0.053	0.121942427	0.372769703
AC114812	0.053	0.121959591	0.372798245
RP11-277E18	0.053	0.122004076	0.372891616
PPP2CB	0.053	0.122018572	0.372891616
GS1-184P14	0.053	0.122087669	0.373046091
SNORA40	0.053	0.122170222	0.373187419
LINC00622	0.053	0.122190771	0.373193528
DDX43P3	0.053	0.122209842	0.373219237
RP11-384O8	0.053	0.122241648	0.373248577
RP11-619L19	0.053	0.122367199	0.373397143
CTD-2336O2	0.053	0.122461564	0.373589347
PRDM5	0.053	0.122522773	0.373704267
C14orf1	0.053	0.122596469	0.373809348
RP11-69H14	0.053	0.122652656	0.373956727
GOLGA2P3Y	0.053	0.122669588	0.373984412
LINC01587	0.053	0.122700393	0.374054383
CLCN4	0.053	0.122724059	0.374078421
RP1-200K18	0.053	0.122762257	0.374120395
PALM	0.053	0.122843653	0.374248032
LINC01554	0.053	0.122862979	0.374248032
RP1-130H16	0.053	0.122873926	0.374248032
TUT1	0.053	0.122896511	0.37427967
SSBP1	0.053	0.122931714	0.37427967
LRWD1	0.053	0.122948057	0.374282333
RN7SKP145	0.053	0.123029051	0.37438532
LRRC4C	0.053	0.123053525	0.37441492
LPP-AS2	0.053	0.123064896	0.374422626
IQSEC3P1	0.052	0.123072908	0.374423085
ARNTL2-AS1	0.052	0.123121272	0.374457698
TMEM132C	0.052	0.123123597	0.374457698
CTC-573M9	0.052	0.123141509	0.374471622

RPS4X	0.052	0.1231439	0.374471622
TECRP1	0.052	0.1231596	0.374495451
MIR125B2	0.052	0.123174773	0.374496546
RP11-318E3	0.052	0.123183549	0.374496546
RNU2-1	0.052	0.123306559	0.374697375
RP3-431A14	0.052	0.123396295	0.374832282
HPN	0.052	0.123500712	0.375053778
SPDYE8P	0.052	0.123538722	0.37512137
AC145676	0.052	0.123648288	0.375387853
AC000081	0.052	0.123741453	0.375545415
LINC01241	0.052	0.123815194	0.375697391
RPS2P5	0.052	0.123881984	0.375768174
LINC01013	0.052	0.123927546	0.375830979
AVPI1	0.052	0.123934521	0.375830979
PSMB5	0.052	0.123938131	0.375830979
RASL11B	0.052	0.123996571	0.375881804
NYNRIN	0.052	0.124021987	0.375917722
RPS23P8	0.052	0.124099864	0.376064942
GRASP	0.052	0.124110839	0.376064942
CLRN3	0.052	0.124227528	0.376278644
EBPL	0.052	0.124281725	0.376417534
CDK6	0.052	0.124304506	0.376431023
UPK3B	0.052	0.124386348	0.37661466
ATP5L	0.052	0.124542558	0.376967805
ITGB1P1	0.052	0.124561046	0.376989766
SERPINH1P1	0.052	0.124589036	0.377012646
RPL36AP29	0.052	0.124650423	0.377126373
CDHR4	0.052	0.124655874	0.377126373
DOK4	0.052	0.12468959	0.377182359
PCDHGA3	0.052	0.124745986	0.377303636
HTR1F	0.052	0.124803541	0.377374024
FTLP2	0.052	0.124954922	0.377735834
SPINK8	0.052	0.12503437	0.377856089
ZBTB7C	0.052	0.125049076	0.377876551
RP11-475O6	0.052	0.125094859	0.377918989
SMPX	0.052	0.125128744	0.377973406
RN7SKP172	0.052	0.125137534	0.377975986
KLF8	0.052	0.125294574	0.378378333
SHFM1	0.052	0.125330177	0.378437859
ADCYAP1	0.052	0.125349526	0.378448297
ARMC6	0.052	0.125451609	0.378708487
DNPH1	0.052	0.125542474	0.378833162
SOX9-AS1	0.052	0.125551928	0.378833162
MED27	0.052	0.125603258	0.378867699
RP11-851M3	0.052	0.125656819	0.378943666
RP11-37O16	0.052	0.125832287	0.379365828
C3orf22	0.052	0.125839373	0.379365828
SERINC5	0.052	0.125850279	0.379365828
TRMT61A	0.052	0.125857857	0.379365828
RN7SL782P	0.052	0.125908875	0.379365828
RP1-95L4	0.052	0.125914293	0.379365828
MPRIPP1	0.052	0.125924253	0.379365828

NEGR1-IT1	0.052	0.125965987	0.379431474
RP11-15K3	0.052	0.125969943	0.379431474
RAC3	0.052	0.12599675	0.379444658
CTD-2201G3	0.052	0.126006188	0.379444658
RPS17P13	0.052	0.12606223	0.379517436
RAB40C	0.052	0.126103099	0.379592485
MRAP2	0.052	0.126132935	0.379658299
TCEB1P28	0.052	0.126374232	0.380096341
PPARG	0.052	0.126397581	0.380104943
MAP4	0.052	0.126416997	0.380104943
XGY1	0.052	0.126478947	0.380169976
MYO16-AS1	0.052	0.126486519	0.380169976
TMEM117	0.052	0.126496585	0.380176238
DYNLT1	0.052	0.126527154	0.380244116
HAGLR	0.052	0.126551749	0.38027267
LINC01573	0.052	0.126610516	0.380374633
RNU4-73P	0.052	0.126731231	0.380517428
C1orf50	0.052	0.126769054	0.380517428
RPS10P16	0.052	0.126782575	0.380517428
ELMOD1	0.052	0.126796681	0.380517428
S100A7A	0.052	0.126801858	0.380517428
RPL31P1	0.052	0.126821502	0.380536518
HOXC11	0.052	0.1268242	0.380536518
RP11-231N3	0.052	0.126849029	0.3805492
CTA-503F6	0.052	0.126889878	0.380613695
RP5-1021I20	0.052	0.126953269	0.380659995
KB-68A7	0.052	0.127056738	0.380910855
LPAR2	0.052	0.127060927	0.380910855
TNFSF4	0.052	0.127232507	0.381173062
NHLH2	0.052	0.127348095	0.381435614
PSMC4	0.052	0.127419841	0.381530539
RNU6-614P	0.052	0.127521931	0.381620892
SAA1	0.052	0.12752213	0.381620892
CCBE1	0.052	0.127538879	0.381632316
CD81	0.052	0.127612176	0.381722466
AC010091	0.052	0.127765661	0.38207042
DEFB123	0.052	0.12776861	0.38207042
RP11-299L17	0.052	0.127788431	0.382105702
GPR111	0.052	0.127893307	0.382347282
USH1C	0.052	0.127972051	0.38255868
RP11-810P12	0.052	0.128010822	0.382650563
LINC00960	0.052	0.1280233	0.382663844
RNU6-714P	0.052	0.128061872	0.382755118
TRAPPC1	0.052	0.128119998	0.382847999
RNU6-1184P	0.052	0.128195125	0.382872323
RP4-715N11	0.052	0.128219466	0.382872323
RP11-64D24	0.052	0.128225381	0.382872323
UNC93B5	0.052	0.128299249	0.382995673
SLC2A1-AS1	0.052	0.12831123	0.382995673
RP11-800A3	0.052	0.128386796	0.383197212
AC004461	0.052	0.128472173	0.383379962
FKSG62	0.052	0.128535748	0.383506453

ARGFXP1	0.052	0.128577461	0.383598015
CTD-2315E11	0.052	0.128695706	0.383758477
UBE2M	0.052	0.128746547	0.383763294
UBE2Q2L	0.052	0.128751256	0.383763294
RP11-115A15	0.052	0.128755287	0.383763294
RPS11P6	0.052	0.128758304	0.383763294
LINC01473	0.052	0.128774781	0.383778016
AC002472	0.052	0.128897059	0.383998263
TCEB3CL	0.052	0.128976903	0.384128536
ABHD8	0.052	0.128981115	0.384128536
RP11-785G17	0.052	0.129037483	0.384176295
RBP4	0.052	0.129098797	0.384232575
TDRD10	0.052	0.129112859	0.384232575
FGF14	0.052	0.12914076	0.384267587
MIR3197	0.052	0.12917231	0.384313443
COL17A1	0.052	0.129275819	0.384511192
LINC01326	0.052	0.129353945	0.38458957
AC009303	0.052	0.129418331	0.384633079
OR8G5	0.052	0.129528158	0.38481926
ATP2A1-AS1	0.052	0.129615217	0.38492587
RNA5-8S5	0.052	0.129736989	0.385127372
RP4-682E18	0.052	0.129769256	0.385159133
ALPK3	0.052	0.129772976	0.385159133
CH17-437K3	0.052	0.129783144	0.385159133
TP53TG1	0.052	0.129891914	0.385338917
PPFIA2	0.052	0.129974685	0.385520602
ASGR1	0.052	0.129999418	0.385562297
MIR4263	0.052	0.130014992	0.385562297
MIR3713	0.052	0.130036447	0.385583729
AC008914	0.052	0.130155423	0.385816414
MSL3P1	0.052	0.130196282	0.385913512
FOXN3-AS1	0.052	0.13024284	0.386003467
RP11-223A3	0.052	0.130332903	0.386225507
UBXN1	0.052	0.130342087	0.386225507
RNU6-365P	0.051	0.130438156	0.386341918
RP11-442N24__B	0.051	0.13045836	0.386354595
METTL9	0.051	0.130646866	0.386719595
SAT2	0.051	0.130837947	0.387093339
TNKS2-AS1	0.051	0.13083815	0.387093339
CLEC2L	0.051	0.130850908	0.387098284
ARTN	0.051	0.130856077	0.387098284
RN7SL58P	0.051	0.130891547	0.387147043
C20orf27	0.051	0.130896946	0.387147043
CTD-2195M15	0.051	0.130924194	0.387181784
PLK3	0.051	0.13093308	0.387181784
DSTNP4	0.051	0.131096914	0.387383897
TRGV8	0.051	0.13111008	0.387383897
MIR1302-1	0.051	0.131243544	0.387594486
AC104651	0.051	0.131304195	0.387725518
AC003989	0.051	0.131429714	0.388012703
NPM1P22	0.051	0.131512331	0.388099497
SOHLH2	0.051	0.131547247	0.388154434

CACNA2D3	0.051	0.131617866	0.38829064
CTD-3007L5	0.051	0.131656997	0.388324857
H1F0	0.051	0.13169229	0.388388025
HEBP2	0.051	0.131815533	0.388633055
ELOVL4	0.051	0.131857475	0.38873265
CTD-2522B17	0.051	0.131884317	0.388735701
DEFT1P	0.051	0.131887726	0.388735701
PGBD5	0.051	0.131919355	0.388735701
AC004069	0.051	0.131922482	0.388735701
TNNT2	0.051	0.131974525	0.388790761
MIR933	0.051	0.131975145	0.388790761
DSC3	0.051	0.132000767	0.388823125
GS1-72M22	0.051	0.132031128	0.388852676
TCF3	0.051	0.132045149	0.388852676
TTTY8	0.051	0.132080011	0.38893129
MB21D2	0.051	0.13212733	0.388974333
EXOC3	0.051	0.13214068	0.388974333
RP11-463H12	0.051	0.13217447	0.389041067
GSTO1	0.051	0.13224231	0.389188868
VENTXP1	0.051	0.1322492	0.389188868
RNU6-1326P	0.051	0.132272057	0.389208036
DPP10-AS2	0.051	0.132272057	0.389208036
UNC93B2	0.051	0.132305958	0.38925124
ABCC6P2	0.051	0.132311259	0.38925124
SMIM10	0.051	0.132425763	0.38941972
PGAM4	0.051	0.132551217	0.389668342
RP3-333A15	0.051	0.13259921	0.389761313
LINC00552	0.051	0.132642851	0.389828813
MTRNR2L1	0.051	0.132734525	0.389979541
ADTRP	0.051	0.132909814	0.390385182
RP11-21B23	0.051	0.132926881	0.390411233
IBA57	0.051	0.132947649	0.390422574
RBP2	0.051	0.132950135	0.390422574
RNU1-131P	0.051	0.132985762	0.390439706
LINC01350	0.051	0.133043839	0.390562064
LINC00602	0.051	0.133079224	0.390641863
PRSS48	0.051	0.133213391	0.390963408
DLGAP4	0.051	0.133299709	0.391156741
CCDC33	0.051	0.133304547	0.391156741
SALRNA3	0.051	0.133312179	0.391156741
ZNF350-AS1	0.051	0.133330122	0.391161373
HDHD1P1	0.051	0.133521245	0.391627721
CTU1	0.051	0.133521971	0.391627721
TSC22D1	0.051	0.133540388	0.391657618
FDXR	0.051	0.133551156	0.39166508
SNCB	0.051	0.133645586	0.39182138
RP11-138M12	0.051	0.133701058	0.391863401
RP11-637O11	0.051	0.13372879	0.391870232
DPPA2P4	0.051	0.133744528	0.391870232
ZP3	0.051	0.133792327	0.391962057
LINC01455	0.051	0.133871455	0.392169749
AL121652	0.051	0.1339012	0.392178772

NOP56P3	0.051	0.133907472	0.392178772
RN7SKP33	0.051	0.133930257	0.392197266
RPS2P7	0.051	0.133961248	0.392215673
RP11-431M3	0.051	0.134021953	0.392345169
NME1-NME2	0.051	0.134060548	0.39237749
ARHGAP1	0.051	0.134161611	0.392550722
AC023469	0.051	0.134166348	0.392550722
SKI	0.051	0.134484097	0.393243601
POM121L8P	0.051	0.13453389	0.393243601
ZNF213	0.051	0.134551772	0.393243601
AL451164	0.051	0.134578044	0.393272089
RP11-183C12	0.051	0.134685447	0.393513513
MRGPRX3	0.051	0.134755283	0.393645109
HMX1	0.051	0.13478928	0.393720271
RP11-315A17	0.051	0.134825873	0.39376822
ZDHHC14	0.051	0.134837438	0.39376822
RUVBL2	0.051	0.134873982	0.393822767
RP11-377K22	0.051	0.134891953	0.393849672
RP1-244F24	0.051	0.134908005	0.393849672
KRT40	0.051	0.135034624	0.394044625
HMGB3P18	0.051	0.135256252	0.394401113
RP5-1125N11	0.051	0.135316353	0.394401113
P2RY2	0.051	0.135344312	0.394401113
RP11-642A1	0.051	0.135387246	0.394401113
VN1R40P	0.051	0.135392009	0.394401113
NEK6	0.051	0.135393706	0.394401113
PLN	0.051	0.135440249	0.394484633
MIR934	0.051	0.135449774	0.394488251
RPL21P132	0.051	0.135559672	0.394643148
NTN1	0.051	0.135560744	0.394643148
STAU2-AS1	0.051	0.135565708	0.394643148
OR8G3P	0.051	0.135569247	0.394643148
LRP4-AS1	0.051	0.135694907	0.394898643
AC063932	0.051	0.135702707	0.394898643
RP3-342P20	0.051	0.135758208	0.394975941
HIST2H2BB	0.051	0.135809207	0.395076052
OR1E2	0.051	0.135860359	0.395200715
ACTA2-AS1	0.051	0.135897883	0.395212819
COX4I1	0.051	0.135898601	0.395212819
RP11-115F18	0.051	0.135914308	0.395212819
RP11-624M8	0.051	0.135933303	0.395219793
APLP2	0.051	0.136543253	0.396392903
RN7SKP111	0.051	0.136569827	0.396392903
AC008163	0.051	0.136643064	0.396508818
MIR1262	0.051	0.136710208	0.396572538
SPATA2L	0.051	0.136733025	0.396600716
AC006355	0.051	0.136755697	0.396642324
ATP5E	0.051	0.136884872	0.396920293
AC007559	0.051	0.136944565	0.396936164
FTCD	0.051	0.137015313	0.39706308
MRPL2	0.051	0.137040896	0.397081748
UBE2J2	0.051	0.137065613	0.397081748

RTP1	0.051	0.137085894	0.397116349
CINP	0.051	0.13712084	0.397187278
AC092309	0.051	0.137135398	0.397187278
SH3BGRL3	0.051	0.137149472	0.397190068
PLAT	0.051	0.137169286	0.397190068
RP3-429O6	0.051	0.137259442	0.39735219
RP3-512B11	0.051	0.137275767	0.39735219
ARHGEF5	0.051	0.137286189	0.397358208
AC008598	0.051	0.13731344	0.397388783
GCM2	0.051	0.137397063	0.397582474
AC074212	0.051	0.137490519	0.397745331
BRINP3	0.051	0.137536856	0.397745331
SNORD72	0.051	0.137553165	0.397768341
EPO	0.051	0.137582186	0.397828108
DESI2	0.051	0.137633008	0.39790259
AC073465	0.051	0.137743451	0.398013931
RNF215	0.051	0.1378418	0.398216148
NDUFB10	0.051	0.13795159	0.398388332
RP11-346M5	0.051	0.1380149	0.398479604
PIN1	0.051	0.13802235	0.398479604
LRRIQ4	0.050	0.138075425	0.398552621
PRSS42	0.050	0.138225017	0.398791065
APOA1BP	0.050	0.138396519	0.399116621
RPS17P11	0.050	0.138668097	0.399609455
RP5-940F7	0.050	0.138714983	0.39969385
CTD-2193P3	0.050	0.138730952	0.39969385
RP11-679B17	0.050	0.138764458	0.399742021
ZFAND2B	0.050	0.138792775	0.399799413
RP11-724O16	0.050	0.138836936	0.399902434
IER2	0.050	0.138894093	0.39997031
SFTPC	0.050	0.138913995	0.399979253
DNAJA1P4	0.050	0.138982462	0.400055465
ZBTB11-AS1	0.050	0.139018993	0.400136434
AC010086	0.050	0.139076612	0.400190933
ALDH3A1	0.050	0.139124475	0.400222916
FAM69C	0.050	0.139170404	0.400231561
RN7SL446P	0.050	0.139192729	0.400231561
OR4G11P	0.050	0.139194902	0.400231561
RP11-101E14	0.050	0.13929827	0.400298092
UNC5D	0.050	0.139325986	0.400298092
HIST1H3C	0.050	0.139472892	0.400477077
HOTAIR	0.050	0.139473687	0.400477077
NFATC4	0.050	0.13954144	0.400550877
CTSV	0.050	0.139553458	0.400561232
COL14A1	0.050	0.139617811	0.400627592
LMF2	0.050	0.139636139	0.400629541
RNU6-1235P	0.050	0.139647021	0.400636629
C15orf43	0.050	0.139687779	0.400668598
ANP32BP2	0.050	0.139699616	0.400668598
FAM86B1	0.050	0.139700228	0.400668598
MIR8059	0.050	0.139748375	0.400712429
PKDCC	0.050	0.139778573	0.400748501

ST6GALNAC6	0.050	0.139826565	0.400766733
NEGR1	0.050	0.139827006	0.400766733
RPL37P3	0.050	0.139910381	0.400907007
DNAH11	0.050	0.139945413	0.400961329
TMEM45B	0.050	0.140069215	0.401195364
STX1A	0.050	0.140099496	0.401257964
BTF3L4P1	0.050	0.140145238	0.401316574
FAM197Y9	0.050	0.140275904	0.401473499
PRNP	0.050	0.140306596	0.401513886
RPS6KA2-IT1	0.050	0.140306876	0.401513886
RPS6P12	0.050	0.140318936	0.401524271
DLL4	0.050	0.140367829	0.401607771
RP11-437J19	0.050	0.140421842	0.401653084
CHRNE	0.050	0.140500081	0.401777086
ASNSP1	0.050	0.140540536	0.401785688
RP11-261B23	0.050	0.140592252	0.401862558
APOBEC3AP1	0.050	0.140617352	0.401871144
MDGA1	0.050	0.140793055	0.402180221
GRM5	0.050	0.140823102	0.402241926
AC010084	0.050	0.140846897	0.402256593
SYT5	0.050	0.140901354	0.402326196
MAB21L1	0.050	0.140939267	0.402326196
ATP1B1	0.050	0.140939644	0.402326196
RP11-193E15	0.050	0.1409896	0.402379656
KCNAB1-AS2	0.050	0.141051708	0.402501591
RNA5SP94	0.050	0.141124224	0.402594994
CCDC61	0.050	0.141177416	0.402698496
GALK1	0.050	0.141246633	0.402871805
RN7SKP284	0.050	0.141371945	0.403010499
HOXD-AS2	0.050	0.141373155	0.403010499
DAPL1	0.050	0.141503292	0.403193375
HPDL	0.050	0.141560655	0.403332695
RPL39P5	0.050	0.141570898	0.403337749
MTATP6P3	0.050	0.141837441	0.403822689
RFNG	0.050	0.141876548	0.403894464
RP11-368I23	0.050	0.141911197	0.403968954
RP1-283K11	0.050	0.142069282	0.404265339
GPRC5B	0.050	0.142119545	0.404320385
RN7SKP234	0.050	0.142154615	0.404371848
RP11-505K9	0.050	0.14226972	0.404602614
GPM6B	0.050	0.142338753	0.404750598
RPS15AP9	0.050	0.142546918	0.405081017
EPHA5	0.050	0.142560826	0.405081017
C1QTNF7	0.050	0.14256552	0.405081017
CCKBR	0.050	0.142653096	0.405254034
AC009297	0.050	0.142695616	0.405280913
RP11-276H7	0.050	0.14270818	0.405280913
NRXN2	0.050	0.14272873	0.405280913
RP11-745A24	0.050	0.142763546	0.405353489
RETN	0.050	0.142802302	0.405415191
SEC23A	0.050	0.142894472	0.405562298
RPL17P11	0.050	0.14293983	0.40556388

SERPINE2	0.050	0.142952689	0.405576202
RP11-748L13	0.050	0.143069769	0.405787512
MT3	0.050	0.143105171	0.40585142
CCL24	0.050	0.14314618	0.40588338
WFDC6	0.050	0.143222375	0.406026909
AC093698	0.050	0.143279555	0.406068156
AAMDC	0.050	0.143302966	0.406086176
PCDHA8	0.050	0.143350563	0.406148558
RP1-276N6	0.050	0.14338667	0.406226694
AC079250	0.050	0.143404406	0.406226918
MICU3	0.050	0.143412338	0.406226918
PQBP1	0.050	0.143446763	0.406276106
AC073410	0.050	0.143496871	0.406393855
LSM5	0.050	0.143530609	0.406465234
RNU6-491P	0.050	0.143551074	0.406493245
TRPC1	0.050	0.14356221	0.406493245
ANO10	0.050	0.143566105	0.406493245
COX20	0.050	0.14362483	0.406611174
C3P1	0.050	0.143691974	0.406777084
FRMD8	0.050	0.143842514	0.406962235
CLYBL	0.050	0.143857515	0.406962235
UTY	0.050	0.143897939	0.407019631
RP11-54D18	0.050	0.143974651	0.407093368
LINC00868	0.050	0.14400117	0.40713254
RPL35P5	0.050	0.144101003	0.407329721
C14orf2	0.050	0.144140663	0.40739347
IGFBP7	0.050	0.144178529	0.407436075
CYGB	0.050	0.144227074	0.407436075
TMEM88B	0.050	0.144399283	0.407761452
PTPRK	0.050	0.144479619	0.407892773
RNU6-1159P	0.050	0.144480044	0.407892773
RPL22P11	0.050	0.144497621	0.407918215
KCTD10	0.050	0.14457778	0.40804776
AL136303	0.050	0.144587977	0.408049275
C4orf3	0.050	0.144673883	0.408077171
FDPS	0.050	0.144713061	0.408115169
AC104308	0.050	0.144787474	0.408276673
DHRS7C	0.050	0.144797972	0.408282103
RNA5SP346	0.050	0.144877617	0.408313288
AC092171	0.050	0.144973954	0.408506339
ZNF414	0.050	0.145006251	0.408506339
RP11-51J9	0.050	0.145030402	0.408526316
MIR3668	0.050	0.145105416	0.408635283
TRIM60P8Y	0.050	0.145105416	0.408635283
CTSZ	0.050	0.145201358	0.408753644
RP11-24N18	0.050	0.145243661	0.408823363
SLIRP	0.050	0.145247446	0.408823363
PAK4	0.050	0.145392734	0.409159759
IGSF21	0.050	0.145428733	0.409236885
SOX4	0.050	0.145524209	0.409384613
VEGFA	0.050	0.145639721	0.409588604
PCDH12	0.050	0.145677282	0.409621673

AP000568	0.050	0.145748375	0.409676457
SLC6A3	0.050	0.145765495	0.409700396
ARID5B	0.050	0.145982127	0.41009147
RNA5SP425	0.049	0.146063833	0.410270481
MIR181A1	0.049	0.146133984	0.410351819
RPA2P2	0.049	0.146151463	0.410368369
OPCML-IT1	0.049	0.146158243	0.410368369
RP11-600K15	0.049	0.146188127	0.41037762
LEP	0.049	0.146197154	0.41037762
TTTY23	0.049	0.146216414	0.41037762
AL133260	0.049	0.146262562	0.410382253
AL356253	0.049	0.146274799	0.410382253
LINC01043	0.049	0.146311071	0.410458701
OR2V2	0.049	0.146395742	0.410599493
RNU7-28P	0.049	0.146449798	0.410702734
A2ML1-AS2	0.049	0.14652496	0.410865131
RP13-383K5	0.049	0.146575392	0.410958156
RNU2-61P	0.049	0.14660363	0.41096475
MIEF2	0.049	0.146707613	0.411207832
MIR4253	0.049	0.146750614	0.411288903
KRTDAP	0.049	0.146761817	0.411288903
RP11-750B16	0.049	0.14690401	0.411395147
CAPZB	0.049	0.146979178	0.4115324
ZNF90P2	0.049	0.146998957	0.4115324
MIR6089	0.049	0.147004865	0.4115324
AC016576	0.049	0.147017073	0.411542386
PVR	0.049	0.147085653	0.411613397
BID	0.049	0.147169593	0.411799907
AC005498	0.049	0.147187776	0.411802398
AC073135	0.049	0.147211813	0.411809229
KRBA1	0.049	0.14726258	0.411890705
RBMS3	0.049	0.14732643	0.411912687
CTD-2292M14	0.049	0.147330617	0.411912687
AL035467	0.049	0.147333899	0.411912687
BABAM1	0.049	0.147376089	0.411942073
KRT74	0.049	0.147423378	0.412022157
PACERR	0.049	0.147459937	0.412055528
HS1BP3	0.049	0.14751153	0.412151335
TMEM256-PLSCR3	0.049	0.147691451	0.412441589
TMEM191A	0.049	0.147745337	0.412514196
AFAP1L1	0.049	0.147944478	0.412947193
SAT1	0.049	0.148105933	0.413206097
HEY2	0.049	0.14817221	0.41336679
TSSC4	0.049	0.148223621	0.413378791
GRIN2B	0.049	0.148232196	0.413378791
RP11-306G20	0.049	0.148249969	0.413378791
RPL21P120	0.049	0.148348171	0.413542777
TDGF1P7	0.049	0.148412899	0.41367479
RP11-353M9	0.049	0.1484606	0.413731006
CTD-2650P22	0.049	0.148464976	0.413731006
RPL21P75	0.049	0.148507775	0.413769733
DEFA8P	0.049	0.148529844	0.413782807

MIR502	0.049	0.148668682	0.414096926
MIR181A2	0.049	0.148743485	0.414193001
RP11-449J1	0.049	0.148746658	0.414193001
RNF222	0.049	0.148837081	0.414396332
PLIN4	0.049	0.1488567	0.414404906
FAM131C	0.049	0.148880893	0.41444563
RP11-610J23	0.049	0.148989888	0.414556729
STARD4-AS1	0.049	0.149074093	0.414668371
MIR1272	0.049	0.14924284	0.415010539
RP11-398J5	0.049	0.149289127	0.415072577
AC007486	0.049	0.149343436	0.4151615
PDRG1	0.049	0.149344824	0.4151615
KPNA7	0.049	0.14935051	0.4151615
AL590731	0.049	0.149367353	0.415168891
DCAF12L1	0.049	0.149418621	0.415287155
CTSD	0.049	0.149440556	0.415312613
TEX22	0.049	0.149477781	0.415354625
HP	0.049	0.149531835	0.415384496
RP11-361D14	0.049	0.149532139	0.415384496
AXL	0.049	0.149603718	0.415413728
GPX1P1	0.049	0.149719421	0.415613851
VGLL4	0.049	0.149834631	0.415860953
RPS24P1	0.049	0.149847444	0.415872282
PPY2	0.049	0.149865569	0.415884895
TES	0.049	0.149869453	0.415884895
RNU1-98P	0.049	0.149899522	0.415898968
CHL1-AS2	0.049	0.149979352	0.416028424
FUNDC2P2	0.049	0.150055473	0.416110162
NR2E1	0.049	0.150072107	0.416132058
TCEAL5	0.049	0.150113572	0.416187003
AC016700	0.049	0.150163294	0.416187003
AC115099	0.049	0.150168722	0.416187003
AC093388	0.049	0.150173546	0.416187003
MIR4473	0.049	0.15017481	0.416187003
RP11-91G21	0.049	0.150232801	0.41626378
MAGOH3P	0.049	0.150236576	0.41626378
NF1P1	0.049	0.150236976	0.41626378
ZNF688	0.049	0.150256789	0.41626378
C19orf53	0.049	0.150259426	0.41626378
PLEKHA8P1	0.049	0.150314334	0.41626378
LINC01270	0.049	0.150385825	0.416310202
RP11-585F1	0.049	0.15042157	0.416325436
ARSH	0.049	0.150556214	0.416583398
AC011404	0.049	0.150580959	0.416621317
DND1P1	0.049	0.150643524	0.416721795
OR51A6P	0.049	0.150692296	0.416784088
RP11-345K20	0.049	0.150799561	0.41700811
RN7SL189P	0.049	0.150995105	0.417306556
KRTCAP2	0.049	0.151061929	0.417467011
SLC4A2	0.049	0.151083419	0.417502175
RNU2-8P	0.049	0.151236927	0.417853643
LINC00205	0.049	0.151306089	0.418020483

RASL11A	0.049	0.151412136	0.418216723
CRISP2	0.049	0.151439612	0.41822316
BICD1	0.049	0.151458003	0.41822316
TRPM1	0.049	0.151467262	0.41822316
RP11-359P5	0.049	0.151518865	0.418292893
CASC20	0.049	0.151528068	0.418294052
RNF181	0.049	0.151885669	0.41891989
NOP56P1	0.049	0.151886717	0.41891989
TRIM43CP	0.049	0.151897976	0.418926683
RP11-535M15	0.049	0.151909437	0.418929744
MTND1P34	0.049	0.151925474	0.418929744
ASPRV1	0.049	0.151949632	0.418947847
ANAPC10P1	0.049	0.152022448	0.419024443
NDUFAF2	0.049	0.152030201	0.419024443
RN7SL1	0.049	0.152098214	0.41910505
RP11-757C15	0.049	0.152442867	0.419821697
RNVU1-2	0.049	0.152470322	0.419848754
RNU6ATAC17P	0.049	0.152538031	0.419955813
ALDH18A1	0.049	0.152550849	0.419955813
RNU7-121P	0.049	0.15255329	0.419955813
POLR3GP1	0.049	0.152673873	0.420210517
CDPF1	0.049	0.152681105	0.420210517
TLR8-AS1	0.049	0.152860621	0.420534472
PAPL	0.049	0.152954294	0.420629209
SART1	0.049	0.153051461	0.420815536
TBC1D19	0.049	0.153067975	0.420815536
CLDN16	0.049	0.15313456	0.420926595
GRPEL2P1	0.049	0.153140188	0.420926595
RP11-326I19	0.049	0.153144562	0.420926595
GATAD2A	0.049	0.153379488	0.42116602
KRT20	0.049	0.153382002	0.42116602
HNRNPCL1	0.049	0.153417783	0.421239984
CPEB1	0.049	0.153492153	0.421419887
DYNLL1	0.049	0.153526044	0.421488639
CTD-3157E16	0.049	0.153670341	0.421763229
SHARPIN	0.049	0.153698423	0.42180246
PRR18	0.049	0.153764101	0.421923306
C1orf216	0.049	0.153814424	0.422012764
LLNLR-304G9	0.049	0.153943336	0.422244833
RP11-60L3	0.049	0.153972769	0.422276925
FAM150A	0.049	0.154030214	0.422398004
AC079163	0.049	0.154130334	0.422502393
AC140132	0.049	0.154176115	0.422566946
CHST8	0.049	0.154246233	0.422686154
TMEM121	0.049	0.154287162	0.422700238
RNU2-52P	0.049	0.154319267	0.422700238
CLN3	0.049	0.154321661	0.422700238
RP11-766H1	0.048	0.154340292	0.422725004
UQCRHP3	0.048	0.154502137	0.423070965
TSPY18P	0.048	0.154547026	0.423100746
RP11-395B7	0.048	0.154548548	0.423100746
GAREM	0.048	0.154665225	0.423302416

ADRM1	0.048	0.154666652	0.423302416
AC241952	0.048	0.154696245	0.423334755
CDCA4P3	0.048	0.154726584	0.423369127
AC073063	0.048	0.154770676	0.423441118
DVL3	0.048	0.154812396	0.423446461
APOD	0.048	0.154860154	0.423539941
C17orf49	0.048	0.154877451	0.423562925
RP11-619J20	0.048	0.154896174	0.423589807
CCDC12	0.048	0.155005559	0.423772676
LINC00668	0.048	0.155024668	0.423772676
FAM35CP	0.048	0.155099467	0.423891389
PPIH	0.048	0.155117541	0.423891389
MTND5P24	0.048	0.15522743	0.424057405
ENOX1	0.048	0.155318785	0.42419299
RN7SL216P	0.048	0.155321594	0.42419299
PIK3CD-AS2	0.048	0.155391797	0.424336054
MIR548I4	0.048	0.155433181	0.424369515
WNK3	0.048	0.155448602	0.424369515
RNU6-974P	0.048	0.155511506	0.424465887
MAGEA4	0.048	0.155524237	0.424465887
AP001048	0.048	0.155528465	0.424465887
SH2D3A	0.048	0.155573246	0.424552505
GALNT18	0.048	0.155591878	0.424552505
DAGLB	0.048	0.155649375	0.424674198
MSRA	0.048	0.155676298	0.424698994
PCDHGA2	0.048	0.155768244	0.424901154
TULP1	0.048	0.155911831	0.425146729
RPS10-NUDT3	0.048	0.155937945	0.425169252
BACH1	0.048	0.156133566	0.425411447
TXLNGY	0.048	0.15613396	0.425411447
AC046143	0.048	0.15616646	0.42547566
RP11-229C3	0.048	0.156200535	0.425495476
RP11-2K6	0.048	0.156237413	0.425547254
WBP1L	0.048	0.156321903	0.425704345
TCF4	0.048	0.156408062	0.425841581
SUB1P3	0.048	0.156432327	0.425883298
HSPB6	0.048	0.15665571	0.426199102
SLC5A1	0.048	0.156736251	0.426320813
SDS	0.048	0.156773078	0.42637228
RPL6P27	0.048	0.156797424	0.426389798
PHEX-AS1	0.048	0.156841569	0.426412457
PTMS	0.048	0.156866475	0.426431485
OR7E16P	0.048	0.156877934	0.426438295
AC003666	0.048	0.156892819	0.426454417
AC104655	0.048	0.156901889	0.426454732
AC005076	0.048	0.156955426	0.426527222
HSPE1P6	0.048	0.15697841	0.426541005
AC027348	0.048	0.157008493	0.426596731
RN7SKP118	0.048	0.157121858	0.42675723
Z97356	0.048	0.157139657	0.42675723
OR6K3	0.048	0.157156551	0.42675723
RP11-345J18	0.048	0.157199067	0.426810712

COPS8	0.048	0.157346461	0.426950593
TSC22D2	0.048	0.157348067	0.426950593
KCTD9P5	0.048	0.157373156	0.42698025
HES7	0.048	0.157397791	0.427022763
RN7SL70P	0.048	0.157443269	0.427091725
CTD-2256P15	0.048	0.15746181	0.427091725
KLF9	0.048	0.157472577	0.427091725
SLC39A2	0.048	0.157485982	0.427091725
RP11-46B11	0.048	0.157553453	0.427201726
RP11-63E5	0.048	0.157584949	0.42722658
CHST13	0.048	0.157587925	0.42722658
MYOZ3	0.048	0.157617745	0.42722658
RNA5SP31	0.048	0.157686363	0.42722658
LHB	0.048	0.157698409	0.42722658
RHEB	0.048	0.157830616	0.427434754
PRELID2	0.048	0.157832065	0.427434754
CTD-2036A2	0.048	0.157836837	0.427434754
GHR	0.048	0.157905648	0.427475262
TRNAU1AP	0.048	0.157921913	0.427494996
SPINT1	0.048	0.157983122	0.427603913
CXCL2	0.048	0.158047242	0.427639154
AL133232	0.048	0.158060896	0.427639154
HSPA8P13	0.048	0.158080652	0.427639154
COX4I2	0.048	0.158091893	0.427639154
CXXC1P1	0.048	0.158195195	0.427772812
CTD-2552K11	0.048	0.158315377	0.427854874
LINC00473	0.048	0.158342114	0.427877867
CTSL	0.048	0.158413056	0.4279246
SYF2P1	0.048	0.158438012	0.42796774
AQP4	0.048	0.158502858	0.428049426
LMOD1	0.048	0.158540464	0.428108553
GNA12	0.048	0.158553064	0.428108553
HIGD2A	0.048	0.158563887	0.428113506
ARHGAP20	0.048	0.158577619	0.428126311
KRT8P9	0.048	0.158586991	0.428127345
BNIP3P34	0.048	0.158689062	0.428321822
MEIS1	0.048	0.158832551	0.428547353
MTND1P6	0.048	0.159064641	0.428809172
TUBB4BP5	0.048	0.159178961	0.428995947
RP11-322L20	0.048	0.159306258	0.429217581
MZT2A	0.048	0.159351936	0.42924352
KRTAP19-1	0.048	0.159534934	0.429639258
RAB34	0.048	0.159594982	0.429659137
CCNO	0.048	0.159596443	0.429659137
OOSP1P2	0.048	0.159734274	0.429840155
OR10D4P	0.048	0.159752425	0.429840155
CD24P2	0.048	0.159859535	0.430040144
LMOD3	0.048	0.159967336	0.430219906
RP11-1053O12	0.048	0.159985931	0.430245622
MIR4484	0.048	0.160090386	0.430406295
RP11-266O8	0.048	0.160133196	0.43044803
LHX4-AS1	0.048	0.1601335	0.43044803

RP11-269F20	0.048	0.160299394	0.430796707
ANKRD30BP3	0.048	0.160322788	0.430804677
ADAM21	0.048	0.160377037	0.430859495
BMS1P10	0.048	0.160416352	0.430916503
BCL2L10	0.048	0.160567164	0.431200017
HADHA	0.048	0.160650056	0.431349657
RPS4Y2	0.048	0.160695619	0.43138201
AL158111	0.048	0.160698335	0.43138201
RNY4P34	0.048	0.160727992	0.431394326
AC006227	0.048	0.160816641	0.431577965
EFCAB1	0.048	0.160937608	0.431744669
FAR2P4	0.048	0.161037976	0.43190425
SDHAF1	0.048	0.161080553	0.431928059
STAC	0.048	0.161111842	0.431956411
ATP13A5-AS1	0.048	0.161170307	0.432064516
RN7SL230P	0.048	0.161241685	0.432167918
RP11-383J24	0.048	0.161245174	0.432167918
NPSR1	0.048	0.161274546	0.432197996
RP11-1259L22	0.048	0.161523468	0.432620039
LRRN1	0.048	0.161585154	0.432713867
CMTM2	0.048	0.161621956	0.432784536
MIR379	0.048	0.161692727	0.432904576
TRIM28	0.048	0.161758429	0.433010971
SAMD4B	0.048	0.161759741	0.433010971
MIR103A2	0.048	0.161828038	0.433112902
TM2D2	0.048	0.16184635	0.433112902
VENTXP5	0.048	0.161877	0.433149949
FGGY	0.048	0.16206713	0.433444026
RP11-124A7	0.048	0.16214606	0.433596089
HGH1	0.048	0.162175387	0.433622745
SSSCA1	0.048	0.162202994	0.433622745
ACKR3	0.048	0.162282055	0.433691343
SLC6A17	0.048	0.162296193	0.433691343
RP13-444H2	0.048	0.162312187	0.433709746
AC008625	0.048	0.1623396	0.433758661
RP3-404F18	0.048	0.162473623	0.434019365
RNU1-47P	0.048	0.162670184	0.434408102
IGFL1P2	0.048	0.162793773	0.43450292
RP11-2F9	0.048	0.162818855	0.43450292
POLR2I	0.048	0.1628958	0.434635018
RP11-162I7	0.048	0.162915408	0.434635018
SNRPB2	0.048	0.162995732	0.434804397
AC145123	0.048	0.163011476	0.434822042
S100A14	0.047	0.163054294	0.434861989
AL356213	0.047	0.163057005	0.434861989
SPG21	0.047	0.163089769	0.434909094
AL121761	0.047	0.163111753	0.434943367
RP11-345N11	0.047	0.163233051	0.435144997
VSIG2	0.047	0.163252195	0.435171673
RBMS2	0.047	0.163267561	0.435188276
HOMER1	0.047	0.163287379	0.435216745
CTD-2169H9	0.047	0.163303225	0.435234622

LINC00310	0.047	0.163323058	0.435235686
OR10Q1	0.047	0.163323444	0.435235686
RPS12P2	0.047	0.163381957	0.435273953
RP11-363E6	0.047	0.163598231	0.435600559
RP11-351M16	0.047	0.163707121	0.435829077
SPNS1	0.047	0.163709287	0.435829077
AC012668	0.047	0.163724379	0.435844894
PROK1	0.047	0.163775486	0.43590637
FTH1P19	0.047	0.163861253	0.436014297
RGS17	0.047	0.163875828	0.436028718
CLEC19A	0.047	0.163912398	0.436042332
SNRPD2P1	0.047	0.163969758	0.436112596
EIF4A1P9	0.047	0.163973615	0.436112596
MRPL9P1	0.047	0.164003826	0.436116015
RP11-346D19	0.047	0.164018479	0.436116015
RNU1-89P	0.047	0.164037627	0.436116015
FIGNL2	0.047	0.164052895	0.436116015
MIR7641-1	0.047	0.164099801	0.436116015
AC106878	0.047	0.16410605	0.436116015
RP11-329E24	0.047	0.164305522	0.436377287
SLC34A2	0.047	0.164380939	0.436446146
UROS	0.047	0.164510378	0.436642945
AL158154	0.047	0.164625324	0.436827138
CD36	0.047	0.164706456	0.436945045
MS4A10	0.047	0.164732066	0.436964305
RN7SL380P	0.047	0.164761679	0.437005833
RP11-312P21	0.047	0.164829554	0.437120007
OFD1P11Y	0.047	0.164851234	0.43713433
CEACAMP4	0.047	0.164907386	0.437185864
USP12-AS2	0.047	0.165060496	0.437348331
RPL21P32	0.047	0.165076234	0.437365699
ECHS1	0.047	0.165102137	0.437409994
RP5-884C9	0.047	0.1652109	0.437492292
FAM155A-IT1	0.047	0.165210906	0.437492292
AC003088	0.047	0.165269161	0.437560416
C11orf91	0.047	0.165449225	0.437866763
NANOGNBP3	0.047	0.165514063	0.438014019
GPX3	0.047	0.165535907	0.438047485
MIR208A	0.047	0.165645223	0.438264241
CES1P1	0.047	0.165654761	0.438264241
LATS2	0.047	0.165709855	0.438264241
ANKRD37	0.047	0.16571904	0.438264241
GYS1	0.047	0.165739917	0.438270781
ARSFP1	0.047	0.165815061	0.438362416
RHBDD3	0.047	0.165827289	0.438362416
ARHGAP42	0.047	0.165829795	0.438362416
GS1-594A7	0.047	0.16586668	0.438435584
AC004492	0.047	0.165885509	0.43846102
NAV2-AS5	0.047	0.165904891	0.438487915
IL17RC	0.047	0.165978391	0.438633493
HSPD1P14	0.047	0.166162506	0.438879582
HIST1H2BH	0.047	0.166189791	0.438879582

RN7SKP162	0.047	0.166233759	0.43894302
MGC45922	0.047	0.166246395	0.438952049
C1orf110	0.047	0.166272639	0.438971543
FAM188B	0.047	0.16630418	0.439007271
HOXD10	0.047	0.166428129	0.43924549
UNC13D	0.047	0.166431312	0.43924549
RNU1-34P	0.047	0.166550412	0.439428898
FUCA2	0.047	0.166565391	0.439428898
RP11-845M18	0.047	0.166656694	0.439572375
BCKDK	0.047	0.166671203	0.4395863
RP11-404J24	0.047	0.166749642	0.439700839
LAMP1	0.047	0.166782939	0.439759234
RP11-390C19	0.047	0.166862806	0.439848067
HOXC-AS3	0.047	0.166930536	0.439891042
RP4-633O19__A	0.047	0.166931078	0.439891042
BMP8A	0.047	0.166940236	0.439891042
DKFZP434L187	0.047	0.167027053	0.439971157
KIF26A	0.047	0.167029594	0.439971157
AC007461	0.047	0.16711548	0.440124362
RP4-655L22	0.047	0.167194469	0.440259356
CIC	0.047	0.16728695	0.440451538
PSMB2	0.047	0.167292165	0.440451538
OSBPL3	0.047	0.16738301	0.440512396
CIR1P3	0.047	0.167385045	0.440512396
LMO7DN	0.047	0.167401555	0.440512396
FLYWCH2	0.047	0.167477764	0.440615559
KRT77	0.047	0.167573649	0.440794773
KLHDC3	0.047	0.167749795	0.441018181
RN7SL749P	0.047	0.167828639	0.441112087
AC112497	0.047	0.16783796	0.441112087
LINC00862	0.047	0.167857844	0.441128153
CAV1	0.047	0.16795315	0.441305565
RN7SL135P	0.047	0.167972759	0.44133274
PROS1	0.047	0.168028063	0.441453695
MIR153-1	0.047	0.168162908	0.441650887
SAMD13	0.047	0.168164963	0.441650887
CTC-301O7	0.047	0.16821791	0.441692722
AC109309	0.047	0.168230331	0.441692722
RP11-78H24	0.047	0.16828437	0.441697393
ID1	0.047	0.168287755	0.441697393
MCAT	0.047	0.168365146	0.441763517
FAM195CP	0.047	0.168365628	0.441763517
C1orf159	0.047	0.16838509	0.441782442
RP11-158I23	0.047	0.168399073	0.441787428
RP11-59E19	0.047	0.168421453	0.441787984
MIRLET7DHG	0.047	0.168501645	0.44186917
PPM1F	0.047	0.168538951	0.441912051
RP11-707M1	0.047	0.168542782	0.441912051
DUX4L4	0.047	0.168579772	0.441976705
TSPYL5	0.047	0.168596127	0.441995251
RPS4XP6	0.047	0.168666009	0.442087021
ARHGEF35	0.047	0.16866814	0.442087021

LIMS2	0.047	0.168681917	0.442087021
FLII	0.047	0.168744038	0.442115322
CFAP52	0.047	0.168806496	0.442225997
HOXA9	0.047	0.168867316	0.44231665
PLEC	0.047	0.168885102	0.44233891
RP11-3J1	0.047	0.168948297	0.442407111
LINC01346	0.047	0.169001501	0.442497772
SEC1P	0.047	0.169033106	0.442547004
MIR4450	0.047	0.169042985	0.442547004
RIMS3	0.047	0.169066429	0.442547004
MPV17L2	0.047	0.169258762	0.442879191
METTL24	0.047	0.169466533	0.443236188
RP11-805F19	0.047	0.169472118	0.443236188
CLCA1	0.047	0.169529125	0.44325601
STRA6	0.047	0.169555212	0.44325601
GAPDHP27	0.047	0.16961798	0.443323691
LINC00400	0.047	0.169620966	0.443323691
RP11-358H9	0.047	0.169624113	0.443323691
GAL3ST4	0.047	0.169708344	0.443446497
RP11-725D20	0.047	0.169802154	0.443568295
AL080286	0.047	0.169824128	0.443568295
MRPS21P3	0.047	0.169837263	0.443568295
METTL7AP1	0.047	0.169874739	0.44363789
DPPA2P1	0.047	0.169944527	0.443771479
HSFX1	0.047	0.170193435	0.444132035
ZSCAN18	0.047	0.170212316	0.444132035
ZNF608	0.047	0.170225096	0.444134839
RP11-659E9	0.047	0.170234812	0.444134839
FAM182A	0.047	0.170390641	0.44445885
KRT33B	0.047	0.170649728	0.444978625
ILK	0.047	0.170701868	0.44502882
RP11-276H19	0.047	0.170778893	0.44512047
C4BPA	0.047	0.170820257	0.445203916
RARRES2P9	0.047	0.170993079	0.445483693
RP11-102K13	0.047	0.171068795	0.445583455
RP3-513G18	0.047	0.171157176	0.445716159
PAGR1	0.047	0.171211296	0.445783972
CES1	0.047	0.1712635	0.445895518
RP13-349O20	0.047	0.171307229	0.445920871
RN7SL483P	0.047	0.171309973	0.445920871
C1S	0.047	0.171315544	0.445920871
RNU6-770P	0.047	0.171419181	0.446019257
MIR1915	0.047	0.171460864	0.446019257
IFT57	0.047	0.171483056	0.446042018
GS1-24F4	0.047	0.171488345	0.446042018
WI2-2221J1	0.047	0.171566617	0.446100898
WWC3	0.047	0.171646575	0.446234248
MAML2	0.047	0.171700764	0.446343952
CGB	0.047	0.171914012	0.446783185
MIR412	0.047	0.171939141	0.44682411
ABCG5	0.047	0.171970604	0.446849219
C14orf180	0.047	0.17197695	0.446849219

EBP	0.047	0.172035099	0.446927162
PSAT1	0.047	0.172046091	0.44693134
TUBB8	0.046	0.172202737	0.447167534
RMST_8	0.046	0.172213097	0.447170056
NIFKP9	0.046	0.172334516	0.447412155
AADACL2-AS1	0.046	0.172358769	0.447430284
RP11-318G8	0.046	0.172381078	0.447459864
BRE	0.046	0.172405871	0.447499833
RP11-15K2	0.046	0.172418712	0.447508775
CARD18	0.046	0.172455451	0.44755667
XKR8	0.046	0.172593117	0.447801852
RP4-758J18	0.046	0.172613262	0.447818488
DECR1	0.046	0.17269093	0.447995584
OR10Z1	0.046	0.172753296	0.448108558
LINC01309	0.046	0.172877466	0.448351413
C8orf4	0.046	0.172882371	0.448351413
SUPT5H	0.046	0.172899611	0.448351413
FBXO24	0.046	0.172903403	0.448351413
MIR1972-1	0.046	0.173006386	0.448562715
MURC	0.046	0.173010489	0.448562715
AC023672	0.046	0.173019821	0.448562715
C19orf73	0.046	0.173022563	0.448562715
RNU6-826P	0.046	0.173032036	0.448562856
RNU6-247P	0.046	0.173069892	0.448587741
AC118754	0.046	0.173268795	0.448887812
RP11-536O18	0.046	0.173307802	0.44892254
MEIKIN	0.046	0.173321596	0.44892254
RP11-397O8	0.046	0.173399663	0.448995867
RP11-448K10	0.046	0.173449261	0.449011198
LL22NC03-32F9	0.046	0.173450102	0.449011198
RPS26	0.046	0.1734732	0.449046583
RN7SKP266	0.046	0.173557441	0.449197514
PRAC1	0.046	0.173579028	0.449222865
MTND4P28	0.046	0.173632925	0.449320649
SLC7A15P	0.046	0.173785512	0.449582105
RPL7P23	0.046	0.173801107	0.449582105
RP11-217L21	0.046	0.173857289	0.449649802
BOLA1	0.046	0.173899084	0.449709056
EMILIN2	0.046	0.174005764	0.449911646
ASB12	0.046	0.174018286	0.449915665
B3GNTL1P1	0.046	0.174175558	0.450204021
AF196972	0.046	0.174277495	0.450371625
RP11-731N10	0.046	0.174304998	0.450416372
STK19	0.046	0.174379179	0.450487726
RP11-738B7	0.046	0.17438272	0.450487726
RP11-405K6	0.046	0.174389363	0.450487726
SLC22A14	0.046	0.174419134	0.450496117
RN7SL542P	0.046	0.174420988	0.450496117
APOB	0.046	0.17447781	0.450520719
SERTM1	0.046	0.174499855	0.450534491
RP5-859I17	0.046	0.174530172	0.450534491
RP11-144A16	0.046	0.174538919	0.450534491

EEFSEC	0.046	0.174539025	0.450534491
PDHA1	0.046	0.174644782	0.450683085
RP11-982M15	0.046	0.174658468	0.450683476
IRX4	0.046	0.174663986	0.450683476
RP11-781P6	0.046	0.174673321	0.450683476
RP4-794H19	0.046	0.174708884	0.450726396
RNU6-140P	0.046	0.174781758	0.450821731
SLC6A11	0.046	0.174783699	0.450821731
APOH	0.046	0.174974594	0.451069825
ABCD1	0.046	0.175153389	0.451310889
APEX2	0.046	0.175184376	0.451365123
ANKRD7	0.046	0.175193392	0.451365123
ABCC1	0.046	0.175272484	0.45146181
ST3GAL4	0.046	0.175278057	0.45146181
TNFAIP8L3	0.046	0.175309061	0.45146181
ACSL4	0.046	0.17532108	0.45146181
LPL	0.046	0.17533519	0.45146181
EXOC3L1	0.046	0.175346165	0.451465662
LRRC34P1	0.046	0.175447448	0.451509599
RP11-102N11	0.046	0.175472668	0.451509599
LINC00619	0.046	0.175476992	0.451509599
DNAH9	0.046	0.17553519	0.451601919
RP4-800F24	0.046	0.175541318	0.451601919
SEC14L6	0.046	0.175782883	0.451801021
SIX3-AS1	0.046	0.175801683	0.451801021
EGLN3-AS1	0.046	0.175803789	0.451801021
BCRP7	0.046	0.17587991	0.45193593
POT1-AS1	0.046	0.175924393	0.451977079
TUSC1	0.046	0.17594605	0.452008335
BIN3	0.046	0.175986923	0.452014257
TUBB	0.046	0.176002646	0.452014257
CTC-465D4	0.046	0.176043193	0.452053941
C1orf137	0.046	0.176049226	0.452053941
EEF1A1P27	0.046	0.17611821	0.452101767
FKRP	0.046	0.176136678	0.452101767
RN7SKP242	0.046	0.176149079	0.452101767
FBXO32	0.046	0.176153285	0.452101767
AC025279	0.046	0.176218162	0.452156734
DEFA5	0.046	0.176249673	0.45217858
RN7SL178P	0.046	0.176335625	0.452204222
TRHDE-AS1	0.046	0.176411424	0.452354221
RP11-402C9	0.046	0.176449871	0.452424119
RNA5SP193	0.046	0.176482094	0.452433669
TH	0.046	0.176545254	0.452499853
RP11-237D3	0.046	0.176608642	0.452598781
LINC00927	0.046	0.176618867	0.452598781
ANO3	0.046	0.176641444	0.45262291
LHFPL5	0.046	0.176710407	0.452702198
LCE3A	0.046	0.176844084	0.452892814
CRLF1	0.046	0.176951414	0.453100269
CTD-2003C8	0.046	0.177145886	0.453451958
KRT16P6	0.046	0.177288279	0.453628348

PRAC2	0.046	0.177290992	0.453628348
RNA5SP28	0.046	0.177353184	0.453763099
TXNRD2	0.046	0.177392953	0.453835514
USP17L17	0.046	0.177434955	0.453899162
ERVV-1	0.046	0.177478302	0.453961284
FGFBP3	0.046	0.177568919	0.4541199
MATN3	0.046	0.177604796	0.454161882
MAEL	0.046	0.177636622	0.454181384
C14orf80	0.046	0.177650178	0.454181384
TST	0.046	0.177851508	0.454476542
AL159140	0.046	0.177902348	0.454508911
RP13-644M16	0.046	0.177973977	0.45459437
BNIP3P20	0.046	0.178077336	0.454712056
RNA5SP270	0.046	0.178122915	0.454804058
ID4	0.046	0.178164224	0.454821751
TRAM2	0.046	0.178187143	0.454821751
PDCL3P1	0.046	0.178244149	0.454894122
THOC5	0.046	0.178310415	0.45494858
AL161757	0.046	0.178315818	0.45494858
KRTAP3-4P	0.046	0.178336953	0.45494858
LIPK	0.046	0.178351607	0.45494858
MIR205	0.046	0.178415303	0.454989594
RHOBTB2	0.046	0.178455106	0.455028456
CTD-2023N9	0.046	0.178459204	0.455028456
AC090841	0.046	0.17854045	0.455055524
RP11-470M17	0.046	0.178586378	0.455055524
RP11-6C14	0.046	0.178622694	0.455055524
CES3	0.046	0.178663297	0.45513347
RNVU1-10	0.046	0.178672402	0.45513347
AC090825	0.046	0.178727702	0.455152619
RBMY1E	0.046	0.178727702	0.455152619
TREM2	0.046	0.178844618	0.455328627
RP4-712E4	0.046	0.178894325	0.455375726
RNU6-771P	0.046	0.178904619	0.455375726
SPANXN1	0.046	0.17902539	0.455618373
PRRT3-AS1	0.046	0.179088538	0.455681686
NKX2-5	0.046	0.179124861	0.45574976
HSPB1P2	0.046	0.17914121	0.45576701
RP11-945A11	0.046	0.17929157	0.455979048
RP11-359E19	0.046	0.179308747	0.455998382
ENKD1	0.046	0.179340854	0.456025004
SPESP1	0.046	0.179398429	0.456066601
RP11-82O18	0.046	0.179402603	0.456066601
AC024198	0.046	0.17942036	0.456078058
RP11-681N23	0.046	0.179439474	0.456078058
STOML2	0.046	0.179465427	0.456078058
REC114	0.046	0.1796473	0.456330638
NAV2-AS2	0.046	0.179651232	0.456330638
LXN	0.046	0.179659769	0.456330638
SEPN1	0.046	0.179786813	0.456555921
ZC3HC1	0.046	0.179812905	0.456555921
NTAN1P3	0.046	0.179830194	0.456555921

CRB3	0.046	0.179834738	0.456555921
MCM8-AS1	0.046	0.179874192	0.456622175
TMEM214	0.046	0.179889598	0.456622175
RP11-454P21	0.046	0.179920488	0.456655648
RNU6-1133P	0.046	0.17997588	0.456655648
DYRK3	0.046	0.179979814	0.456655648
CTB-11I22	0.046	0.180014466	0.456655648
OR4Q1P	0.046	0.180019634	0.456655648
AC092156	0.046	0.180027431	0.456655648
SHKBP1	0.046	0.180067399	0.456732705
SULT1E1	0.046	0.180092576	0.456747916
CACNG7	0.046	0.180152366	0.456807972
C19orf43	0.046	0.180155548	0.456807972
RP11-522M21	0.046	0.180173804	0.456807972
ZNF653	0.046	0.180185946	0.456814439
IFFO1	0.046	0.180273249	0.457011447
AL358852	0.046	0.180295125	0.457042575
RP4-760C5	0.046	0.180360876	0.45709838
RNU6-55P	0.046	0.180365126	0.45709838
SLC22A31	0.046	0.180446955	0.457208444
MIR4437	0.046	0.180494323	0.457219901
DDN	0.046	0.180564732	0.457336551
TNFRSF10D	0.046	0.180574518	0.457337015
SRD5A2	0.046	0.180591846	0.457353109
RP4-545L17	0.046	0.180656439	0.457386213
OR13G1	0.046	0.180714571	0.457386213
SHPK	0.046	0.180718789	0.457386213
EIF3KP3	0.046	0.18091549	0.457665163
NPM1P47	0.046	0.180982081	0.457784989
SLC9B2	0.046	0.180991712	0.457785038
RP11-488L1	0.046	0.181097016	0.457919799
IRGC	0.046	0.1811005	0.457919799
RP11-1L9	0.046	0.18112734	0.45793353
OR13C9	0.046	0.181146391	0.457939338
MIR4665	0.046	0.181148868	0.457939338
CEACAM19	0.046	0.1812378	0.458049322
ARNTL2	0.046	0.181291905	0.458104259
MIR1252	0.046	0.18143856	0.458266373
OR8B9P	0.046	0.181466471	0.458266373
RARRES2P4	0.045	0.181494107	0.458301247
CYP11B2	0.045	0.18155468	0.458360842
WNT6	0.045	0.181564304	0.458360842
ANKS3	0.045	0.181590322	0.458398381
AC055714	0.045	0.181758314	0.45865407
CHCHD2P6	0.045	0.181795135	0.458661626
GLYATL1P4	0.045	0.181878177	0.458711711
LCE1C	0.045	0.181966311	0.458853527
FP671120	0.045	0.181984337	0.458853527
MAGED4	0.045	0.1819954	0.458853527
FGF19	0.045	0.181998759	0.458853527
TNFRSF11B	0.045	0.182106886	0.458972569
RP11-144M17	0.045	0.182145201	0.458997353

CARD14	0.045	0.182155269	0.458997353
ERICH2	0.045	0.182191839	0.459065216
DCD	0.045	0.182229613	0.459087532
TACC2	0.045	0.182290612	0.459144056
CACNG2	0.045	0.182339248	0.459193706
GSTA5	0.045	0.18240173	0.45926869
OR6C4	0.045	0.182406604	0.45926869
DLL1	0.045	0.182417238	0.45926869
SLC39A11	0.045	0.182481832	0.459325371
RNU4-13P	0.045	0.182503658	0.459325371
LIMA1	0.045	0.1825057	0.459325371
HIST1H4C	0.045	0.182507261	0.459325371
RBM46	0.045	0.18252044	0.459334268
IL1F10	0.045	0.182550207	0.459360633
RP11-655G22	0.045	0.182563512	0.459369843
RP11-141J10	0.045	0.182647897	0.459533619
SNORD116-22	0.045	0.182669783	0.45954013
HS6ST1P1	0.045	0.182798579	0.459724792
DDX54	0.045	0.182801102	0.459724792
RP11-25J3	0.045	0.18282169	0.459752291
ALPL	0.045	0.182989842	0.460053695
RBMS1P1	0.045	0.183016554	0.460057945
PTPRG	0.045	0.183061241	0.460099394
RP11-131L23	0.045	0.183091281	0.460114418
RPL21P2	0.045	0.18316987	0.460239061
FEZ2	0.045	0.183191863	0.460270038
RP11-63B19	0.045	0.183363012	0.460569007
LINC00244	0.045	0.183387361	0.460569007
RP11-380D11	0.045	0.183406711	0.460584222
MEPCE	0.045	0.183418365	0.460584222
AC021052	0.045	0.183429157	0.460584222
RBMS3-AS3	0.045	0.183442629	0.460584222
LINC01452	0.045	0.183510119	0.460638095
CEND1	0.045	0.183641741	0.460865486
RASAL2	0.045	0.183726582	0.460922126
RP11-180I4	0.045	0.183737644	0.460922126
JUND	0.045	0.183741732	0.460922126
PRKRIP1	0.045	0.183850517	0.461053079
SERPINB13	0.045	0.183904459	0.461087475
MTND4P17	0.045	0.183968669	0.461151357
CTC-439O9	0.045	0.18400766	0.461200549
RP11-51O6	0.045	0.184022541	0.461213575
PJA1	0.045	0.18408015	0.461284594
NR0B1	0.045	0.184188424	0.461337999
TREML3P	0.045	0.184222551	0.46136996
RP11-547C13	0.045	0.184230245	0.46136996
MICU1	0.045	0.184352843	0.461580548
KRTAP2-2	0.045	0.184387595	0.461580548
UPP2-IT1	0.045	0.184402308	0.461580548
ARL6IP4	0.045	0.184528833	0.461689375
RP11-434H6	0.045	0.184552175	0.461689375
ANTXR2	0.045	0.184651539	0.461842182

SVOP	0.045	0.184719009	0.461924644
WFDC2	0.045	0.184730108	0.461924644
SCYL1	0.045	0.184736635	0.461924644
TPTE2P1	0.045	0.184780521	0.461946453
SPDYE9P	0.045	0.184831822	0.46200222
EHD1	0.045	0.18509327	0.462364271
AL157702	0.045	0.185296487	0.46267777
GALNT13	0.045	0.185316526	0.462679292
RPL23AP53	0.045	0.185341475	0.462694985
RNU6-570P	0.045	0.185346931	0.462694985
RP5-836J3	0.045	0.185351956	0.462694985
RNU6-310P	0.045	0.185377547	0.462710362
CD300LD	0.045	0.185409016	0.462740407
LINC01007	0.045	0.185435181	0.462759423
SNORA35	0.045	0.185526917	0.462920621
GOT2P5	0.045	0.185652077	0.463104356
QKI	0.045	0.185665889	0.463109578
RPS28	0.045	0.185702789	0.463109578
TPT1P1	0.045	0.185717423	0.463121824
TCEAL4	0.045	0.185853353	0.463388004
RPS6P23	0.045	0.185926977	0.463498778
SLC17A1	0.045	0.185988159	0.463627031
AXIN1	0.045	0.186084467	0.463820012
MARK2P6	0.045	0.186262996	0.464043734
RP11-556I14	0.045	0.186313624	0.464074
RPS10L	0.045	0.186358802	0.464137983
MIR4799	0.045	0.186393914	0.46414304
MAP2K3	0.045	0.186506294	0.464344182
C11orf94	0.045	0.186558121	0.464391555
RP11-1007I13	0.045	0.186706569	0.464642943
RP11-145H9	0.045	0.186756259	0.464714751
MACROD2-IT1	0.045	0.18686199	0.464844314
RP11-121E16	0.045	0.186966525	0.465005345
VAT1	0.045	0.18709438	0.465221285
AL139147	0.045	0.187109693	0.465229619
RNU6ATAC	0.045	0.187337663	0.465664854
OR52D1	0.045	0.187343403	0.465664854
RP11-274H2	0.045	0.187396671	0.465748644
RP11-413P11	0.045	0.187484289	0.465829695
MIR1261	0.045	0.187513329	0.465844104
RP11-475J5	0.045	0.187524484	0.465847518
CITF22-92A6	0.045	0.187570081	0.465902996
HNRNPA1P69	0.045	0.187613937	0.465907784
AC093585	0.045	0.187638685	0.465907784
LINC01123	0.045	0.187639131	0.465907784
CEBPA-AS1	0.045	0.187656351	0.465907784
EEF1B2P5	0.045	0.187731852	0.465980459
RP11-380D23	0.045	0.187797691	0.465980459
ZNF133	0.045	0.187806432	0.465980459
RP1-137D17	0.045	0.187807621	0.465980459
RNU4-2	0.045	0.187917038	0.466129435
ANGPT1	0.045	0.187966689	0.46616796

RNASE12	0.045	0.188034634	0.466311968
TYR	0.045	0.188133343	0.466411259
ACAN	0.045	0.188230214	0.46657732
SERPINA15P	0.045	0.18826288	0.466610959
AC008063	0.045	0.18839068	0.466854828
LINC01192	0.045	0.188412812	0.46686109
RP11-66A2	0.045	0.188437206	0.466897242
MORN5	0.045	0.188456179	0.466919962
FOXO1B	0.045	0.18851726	0.467047
RPS24	0.045	0.188567356	0.467146813
IQCA1	0.045	0.188665919	0.467342368
Six3os1_4	0.045	0.18879085	0.467545945
RNA5SP325	0.045	0.188818969	0.467545945
AC096587	0.045	0.18883637	0.467545945
RP11-380L11	0.045	0.188876042	0.467575142
VEGFB	0.045	0.188921512	0.467581078
ANGPTL6	0.045	0.188937529	0.467581078
AC096669	0.045	0.189122533	0.467889403
RP11-231E6	0.045	0.189178969	0.467927003
RP1-39J2	0.045	0.189289122	0.467961225
SNORD113-7	0.045	0.189322383	0.46797058
MIR4758	0.045	0.189322383	0.46797058
RP11-96G10	0.045	0.189334512	0.467976273
GHc-1077H7	0.045	0.189366718	0.468031586
NOTCH4	0.045	0.189387164	0.468057831
LSAMP-AS1	0.045	0.189506076	0.468230228
CHI3L1	0.045	0.189527006	0.468234534
AC156455	0.045	0.189613158	0.468309966
AC108463	0.045	0.189669139	0.468325587
RNU6-223P	0.045	0.189748345	0.468401979
RNU7-158P	0.045	0.189790952	0.468401979
AADAT	0.045	0.189844237	0.468458188
RPLP2	0.045	0.189878051	0.468464956
RN7SKP174	0.045	0.19028183	0.469124791
RPS12	0.045	0.19038376	0.469277584
RFKP1	0.045	0.190427965	0.469290732
DMP1	0.045	0.190481623	0.469374392
RPLP1P6	0.045	0.190497097	0.469388238
RP11-756G12	0.045	0.190783825	0.469778784
OR7E97P	0.045	0.190847202	0.469861965
VDAC2P1	0.045	0.190868818	0.469888281
C9orf135	0.045	0.190982517	0.470025027
CH17-478G19	0.045	0.191145659	0.470280726
RN7SL299P	0.045	0.19118881	0.470338297
KLHL31	0.044	0.191304296	0.470480997
PABPC1L2B-AS1	0.044	0.191306087	0.470480997
CYCSP23	0.044	0.191330986	0.470517937
RP11-785F11	0.044	0.191353244	0.470527094
PPP1R3F	0.044	0.191354469	0.470527094
HSD17B10	0.044	0.191393685	0.470593024
TCN1	0.044	0.191401043	0.470593024
RP11-670E13	0.044	0.191467185	0.470629838

OR7E23P	0.044	0.191472611	0.470629838
FKBPL	0.044	0.191508951	0.470629838
RPL22P19	0.044	0.191512977	0.470629838
RP4-719C8	0.044	0.191513254	0.470629838
RNU7-173P	0.044	0.191515624	0.470629838
ADAM11	0.044	0.191649571	0.470839477
RP11-673F18	0.044	0.191714525	0.470926179
RPL23AP68	0.044	0.191763302	0.470948837
RP11-14I2	0.044	0.191867432	0.471083111
PKP1	0.044	0.191934762	0.471224131
RNU6-432P	0.044	0.191997594	0.471354092
RP11-214O14	0.044	0.192103951	0.471469385
ILDR2	0.044	0.19222595	0.471695879
RN7SL554P	0.044	0.192261932	0.471708265
AP003464	0.044	0.1922913	0.471734715
ASB17	0.044	0.19233267	0.471766532
RP11-248M19	0.044	0.192400964	0.471906516
NT5C1A	0.044	0.192592565	0.472134996
RNU5A-1	0.044	0.192626187	0.472134996
CLSTN1	0.044	0.192646862	0.472144914
HSPB2-C11orf52	0.044	0.192680731	0.472203621
RP11-507E23	0.044	0.192736463	0.472266301
NUDT18	0.044	0.192778191	0.472320947
DRP2	0.044	0.192793887	0.472335107
UGP2	0.044	0.192975213	0.472682086
CTD-2017D15	0.044	0.19299218	0.472691181
ADAMTSL1	0.044	0.192998776	0.472691181
QARS	0.044	0.193033563	0.47270262
GPR56	0.044	0.193047217	0.47270262
TRPV6	0.044	0.193103692	0.472777953
AWAT2	0.044	0.193127978	0.472788806
C6orf226	0.044	0.193200643	0.472942383
RP11-107E21	0.044	0.193266369	0.473070271
FAM90A23P	0.044	0.193412271	0.473128026
RP11-231G15	0.044	0.193428663	0.473128026
LINC01356	0.044	0.193441144	0.473128026
LMNTD1	0.044	0.193467844	0.473128026
RP11-547M24	0.044	0.193492441	0.473145991
RP11-810P8	0.044	0.193665861	0.473491381
EI24	0.044	0.193730083	0.473539467
RP11-839D17	0.044	0.193746207	0.473544961
OR9A4	0.044	0.19378207	0.473562453
MIR4474	0.044	0.19379371	0.4735666
RP11-317N12	0.044	0.19388035	0.473636555
RNU6ATAC32P	0.044	0.193894353	0.473636555
SNX8	0.044	0.193918435	0.473636555
RNU6-12P	0.044	0.194096156	0.473787173
GUSBP12	0.044	0.194102826	0.473787173
SNRPEP5	0.044	0.194146418	0.473804815
ZSCAN1	0.044	0.194325392	0.474111748
RP4-604K5	0.044	0.194392341	0.474226505
AC116562	0.044	0.19453669	0.474428951

EIF4A3P1	0.044	0.194634399	0.474556695
LINC01519	0.044	0.194637296	0.474556695
C11orf70	0.044	0.194743204	0.474735605
MYCNOS	0.044	0.1947776	0.474758542
MIR4528	0.044	0.19478986	0.474758542
NR2F1	0.044	0.194919278	0.474864445
MIR409	0.044	0.194969635	0.474921914
MIR582	0.044	0.194970473	0.474921914
CH507-210P18	0.044	0.195145425	0.475222525
RPLP0	0.044	0.195153534	0.475222525
OR8A1	0.044	0.195169811	0.475222525
LINC00470	0.044	0.195243027	0.475352195
RP5-1121H13	0.044	0.195358105	0.475488697
SLC25A1P4	0.044	0.195360031	0.475488697
PHBP7	0.044	0.195464596	0.475648495
RP11-247L20	0.044	0.195514027	0.475695864
CLDN4	0.044	0.195529564	0.475709366
PEX16	0.044	0.195556565	0.475750755
PRRC2CP1	0.044	0.195581259	0.475786526
RP11-471L13	0.044	0.195626633	0.475859314
KIAA1217	0.044	0.195631162	0.475859314
CREM	0.044	0.195697279	0.475905619
SNCG	0.044	0.195704647	0.475905619
RP11-95M15	0.044	0.195852925	0.476179859
AC023449	0.044	0.195963553	0.476300663
HAO2-IT1	0.044	0.196028459	0.476387764
LCE3E	0.044	0.196083301	0.476470285
SIRT6	0.044	0.196092428	0.476470285
LA16c-60D12	0.044	0.196115258	0.476501448
RP11-713C19	0.044	0.196148867	0.476517538
USMG5	0.044	0.196192158	0.476518584
AC079355	0.044	0.196257606	0.476595134
ANKRD30BL	0.044	0.19635271	0.476735463
LYZL4	0.044	0.196424093	0.476838284
RP11-1021N1	0.044	0.196580964	0.477003383
SEMA6C	0.044	0.196674868	0.477179517
PTPRH	0.044	0.196700467	0.477193009
LINC00836	0.044	0.196996631	0.477595242
MYPOP	0.044	0.197021141	0.477613821
PCDHGA12	0.044	0.197052163	0.477633365
TMSB4X	0.044	0.19705247	0.477633365
TMPRSS5	0.044	0.197100399	0.477673934
MIR370	0.044	0.197109119	0.477673934
TMEM72-AS1	0.044	0.197134012	0.477673934
RPS3AP44	0.044	0.197140724	0.477673934
GPR115	0.044	0.197173039	0.477673934
SF3A2	0.044	0.197214869	0.47773524
PPP1R16A	0.044	0.197307603	0.477747485
RNU6-1339P	0.044	0.197308103	0.477747485
RP5-999L4	0.044	0.197334384	0.477747485
XXbac-B33L19	0.044	0.197335557	0.477747485
PROM2	0.044	0.197340296	0.477747485

TMPRSS11D	0.044	0.197355754	0.477760623
RP11-329L6	0.044	0.197612445	0.478265647
OR51S1	0.044	0.197616351	0.478265647
THAP7-AS1	0.044	0.197624623	0.478265647
LINC01394	0.044	0.197675646	0.478340513
MIR4713	0.044	0.19776084	0.478473738
MKL1	0.044	0.197791174	0.478498515
DNM3	0.044	0.197833638	0.478552626
AL390718	0.044	0.197898012	0.4786306
RN7SL271P	0.044	0.19790607	0.4786306
ZNF511	0.044	0.197920679	0.478641626
ZNF423	0.044	0.197954399	0.478674561
IGFBP1	0.044	0.197974486	0.4786946
KRTAP20-1	0.044	0.197982788	0.4786946
REEP6	0.044	0.198035381	0.478756013
PRDX6	0.044	0.198207715	0.479043886
SLC44A2	0.044	0.19822278	0.479050578
RP11-452F19	0.044	0.198302507	0.479175723
MYL6P3	0.044	0.198387873	0.479290528
IGFBP2	0.044	0.198412026	0.47929446
RPL7AL2	0.044	0.198580368	0.479581452
RP11-44L9	0.044	0.198589777	0.479581452
FEZF1-AS1	0.044	0.198641247	0.479604921
HBEGF	0.044	0.198677995	0.479669329
RNU4-35P	0.044	0.198776948	0.479798196
RPS27AP1	0.044	0.198889884	0.479986244
LINC01151	0.044	0.198932769	0.480026221
RP11-115C10	0.044	0.198944667	0.480026221
MYO9B	0.044	0.198955697	0.480026221
LBX1-AS1	0.044	0.199119776	0.480271379
CTD-2228K2	0.044	0.199138947	0.480271379
KRT90P	0.044	0.199139127	0.480271379
RNU6-824P	0.044	0.199168149	0.480292847
SNORD114-1	0.044	0.199239295	0.480361067
MRPL15P1	0.044	0.199246916	0.480361067
RN7SL837P	0.044	0.199308045	0.480484118
RNA5SP164	0.044	0.199497792	0.480745401
AC051649	0.044	0.199617946	0.480866114
RNA5SP358	0.044	0.199647739	0.480889235
AP000525	0.044	0.199666286	0.480901267
LINC00359	0.044	0.199771081	0.481113333
AC007274	0.044	0.19980826	0.48117854
EOGT	0.044	0.199822342	0.481188123
C15orf39	0.044	0.199860518	0.481214305
RNASEK	0.044	0.199867042	0.481214305
TMEM219	0.044	0.199921343	0.481231896
URB1-AS1	0.044	0.199924605	0.481231896
CHL1	0.044	0.200006825	0.481316006
RP11-540K16	0.044	0.200146011	0.481553629
NCALD	0.044	0.200180142	0.481570538
RPL7P45	0.044	0.20024053	0.481674464
AC007318	0.044	0.200246801	0.481674464

CIAPIN1	0.044	0.200362869	0.481822263
RP3-388N13	0.044	0.200368945	0.481822263
SND1	0.044	0.200471679	0.481923213
TUSC3	0.044	0.200580724	0.482063791
RP11-744A16	0.044	0.200594867	0.482073455
ATL1	0.044	0.200664028	0.482166674
MED16	0.044	0.200710211	0.482253316
RNU6-132P	0.044	0.200750892	0.482326729
AC091705	0.044	0.200787775	0.48239101
MEF2BNBP1	0.044	0.200854769	0.482478948
C7orf65	0.044	0.20091426	0.48251147
UQCRHP4	0.044	0.200980926	0.482635939
LMNB2	0.044	0.201033522	0.482673222
MROH5	0.044	0.20107981	0.482691805
AC005094	0.044	0.201185489	0.482814149
MIR323B	0.044	0.201201208	0.482814149
TIMM44	0.044	0.201205826	0.482814149
ATP8B5P	0.044	0.201298902	0.482839586
RPL31P54	0.044	0.201323264	0.482859464
RP11-22L13	0.044	0.20139852	0.483005554
EMILIN3	0.043	0.201559261	0.483220726
MIR8068	0.043	0.201673829	0.483420243
MGARP	0.043	0.201729292	0.483482338
RPL12	0.043	0.201788485	0.483567981
RNU6-7	0.043	0.201846252	0.483567981
RPL21P131	0.043	0.20188341	0.483597822
KCTD16	0.043	0.20188917	0.483597822
MOB2	0.043	0.201930331	0.483647768
AL050303	0.043	0.201944435	0.483657228
RP11-129M6	0.043	0.202015799	0.483706523
RP11-683L23	0.043	0.202167356	0.483826173
ANKRD45	0.043	0.202238952	0.483837591
RP13-126C7	0.043	0.202251638	0.483837591
AL162711	0.043	0.202270802	0.483837591
VPS25P1	0.043	0.202272628	0.483837591
AC020931	0.043	0.202277049	0.483837591
LINC01038	0.043	0.202283875	0.483837591
LTC4S	0.043	0.202327843	0.48389623
RP11-264A11	0.043	0.202339021	0.48389623
SPRY4	0.043	0.202340235	0.48389623
SNRPBP1	0.043	0.202396804	0.483932176
RP11-268J15	0.043	0.202572827	0.484153122
TMEM115	0.043	0.202583871	0.484153122
OR52A5	0.043	0.202919625	0.484700987
RNU6-694P	0.043	0.202951073	0.484751793
AC092326	0.043	0.203060724	0.48495513
RP11-259K5	0.043	0.203078577	0.484959056
NPHS2	0.043	0.203169737	0.485110004
DPYD-AS2	0.043	0.203229108	0.485196889
SYT17	0.043	0.203382118	0.485437426
AC006153	0.043	0.203390298	0.485437426
RNU6-33P	0.043	0.203502536	0.485630589

UBL5	0.043	0.203698632	0.485952452
GOLPH3	0.043	0.203784238	0.486075319
RP11-66H6	0.043	0.203787908	0.486075319
AP001331	0.043	0.203815987	0.486075319
PKN3	0.043	0.203827245	0.486075319
TMEM109	0.043	0.203839117	0.486075319
MIR449A	0.043	0.203856881	0.486075319
USP54	0.043	0.203864809	0.486075319
MXI1	0.043	0.204017257	0.486249807
RP5-856G1	0.043	0.204035564	0.486269106
PRND	0.043	0.204110829	0.486399803
LINC00998	0.043	0.204178751	0.486488637
RPUSD1	0.043	0.204256512	0.486649569
HSPD1P16	0.043	0.20431269	0.486736369
RP11-977B10	0.043	0.204319669	0.486736369
CAND2	0.043	0.204344042	0.486736369
IFNWP15	0.043	0.204566742	0.487023255
CRYBB2	0.043	0.204688347	0.487208574
RP11-106M7	0.043	0.204783282	0.487319544
ARRDC3	0.043	0.204818429	0.48737468
CICP26	0.043	0.20496965	0.487548867
RP11-130C6	0.043	0.204974141	0.487548867
C11orf97	0.043	0.205014514	0.487548867
PAX3	0.043	0.205023756	0.487548867
RP11-14K3	0.043	0.205040651	0.487548867
CC2D1A	0.043	0.205053675	0.487548867
CHAT	0.043	0.205080951	0.487589381
RP11-500C11	0.043	0.205212439	0.487853291
RNU6-1032P	0.043	0.205285976	0.487924327
BAIAP2L2	0.043	0.205538459	0.488214063
RN7SKP207	0.043	0.205575692	0.488253801
RP11-523L1	0.043	0.205713823	0.488484431
KCTD10P1	0.043	0.205741988	0.488526955
PHEX	0.043	0.205820556	0.488689147
XXcos-LUCA11	0.043	0.205854015	0.488744226
UBAC2	0.043	0.205892496	0.488786856
RP11-977P2	0.043	0.205924565	0.488814258
RP11-875O11	0.043	0.206025251	0.488980148
OR4F7P	0.043	0.206069977	0.48900884
LRRC71	0.043	0.206330863	0.489497172
RNA5SP105	0.043	0.206333852	0.489497172
AC007003	0.043	0.206341941	0.489497172
C1orf204	0.043	0.206381467	0.489529433
RSL24D1P4	0.043	0.206404373	0.489529433
ANKRD6	0.043	0.206421139	0.489529433
CLDN24	0.043	0.206462394	0.489554139
AC005178	0.043	0.206493803	0.48960424
RP3-380B8	0.043	0.206544063	0.489650279
ASTN1	0.043	0.206710342	0.489892072
STK25	0.043	0.206736556	0.489911014
AC016757	0.043	0.206746624	0.489911014
TEX41	0.043	0.206817185	0.489973699

RP11-51I5	0.043	0.206824517	0.489973699
RPL13A	0.043	0.206860656	0.490010567
WNT4	0.043	0.20689587	0.490043154
PDCD5	0.043	0.206896461	0.490043154
WI2-85898F10	0.043	0.206923948	0.490043154
MIR3162	0.043	0.206930824	0.490043154
RN7SKP148	0.043	0.206982436	0.490075163
RP5-915N17	0.043	0.206990824	0.490075163
SHISA5	0.043	0.207150565	0.490307124
DKFZP434A062	0.043	0.207252204	0.490485341
FAM3C2	0.043	0.207287651	0.490485341
OR7H2P	0.043	0.207464305	0.490732677
RP11-662I13	0.043	0.207535025	0.490826827
WI2-89927D4	0.043	0.207570966	0.490827293
ZNF488	0.043	0.207653553	0.490884004
BCO1	0.043	0.20768056	0.490884004
RP11-444P10	0.043	0.207699552	0.490884004
CTD-2262B20	0.043	0.207703497	0.490884004
AC079949	0.043	0.207764765	0.490940003
AC007312	0.043	0.207768423	0.490940003
DKFZp779M0652	0.043	0.207812919	0.490959011
RNU6-319P	0.043	0.207813306	0.490959011
CCDC144NL-AS1	0.043	0.207945234	0.491173801
CYP1D1P	0.043	0.208034144	0.491299756
NPAS4	0.043	0.208098953	0.491368229
RP11-594N15	0.043	0.208104406	0.491368229
AC093326	0.043	0.208159047	0.491433495
PACRG	0.043	0.208194617	0.49143505
HIST1H4J	0.043	0.208370301	0.491703529
VSIG8	0.043	0.208449542	0.491769164
KLHL30	0.043	0.20849957	0.491841596
LINC00645	0.043	0.208590595	0.492003975
AC011524	0.043	0.20871363	0.492170735
RP1-290F12	0.043	0.208742027	0.49219053
CISD1	0.043	0.208812806	0.492259937
PGP	0.043	0.208971494	0.49251214
NOV	0.043	0.209012913	0.492557373
RORB-AS1	0.043	0.209072325	0.492627894
CXCL1	0.043	0.209118625	0.492696748
CTD-2007H13	0.043	0.209122236	0.492696748
KCNK3	0.043	0.2091495	0.492736606
RPL3	0.043	0.209296273	0.493009228
LENG9	0.043	0.209353613	0.49309552
RPL21P65	0.043	0.209470121	0.493165099
LINC01065	0.043	0.209508309	0.493165099
RNA5SP135	0.043	0.209531893	0.493165099
GFRA2	0.043	0.209638299	0.493351283
TRAPPC13P1	0.043	0.209684872	0.493394518
RHOT2	0.043	0.209898414	0.493670704
AP000997	0.043	0.209966748	0.493739589
CHL1-AS1	0.043	0.209970306	0.493739589
S100B	0.043	0.209979536	0.493739589

ARL5AP3	0.043	0.210073551	0.493911883
RP11-325D15	0.043	0.210141168	0.493975795
RASIP1	0.043	0.210145629	0.493975795
TMEM47	0.043	0.210152593	0.493975795
NINL	0.043	0.210275387	0.494191259
AC243772	0.043	0.210286104	0.494192059
RNA5SP148	0.043	0.210414334	0.49439583
SNORD113-2	0.043	0.210452352	0.494436966
MEIS1-AS3	0.043	0.210567286	0.494560022
RP11-838N2	0.043	0.210594216	0.494583947
RN7SKP137	0.043	0.210621279	0.49461366
RNU6-1049P	0.043	0.210788311	0.494908302
TTPA	0.043	0.21087822	0.495070585
CTD-2613O8	0.043	0.210909893	0.495105639
MARK2P15	0.043	0.210913942	0.495105639
RP11-448P19	0.043	0.210926664	0.495111101
RXRG	0.043	0.211011001	0.49516264
RP11-114H20	0.043	0.211028136	0.495178452
EMG1	0.043	0.211056148	0.495219783
AC019048	0.043	0.211092578	0.495256464
BX284613	0.043	0.211116278	0.49528767
TM4SF19-AS1	0.043	0.211230323	0.495506408
RP11-318I4	0.043	0.211286039	0.495588287
CCDC153	0.043	0.211303548	0.495604949
RN7SL824P	0.043	0.211334405	0.495619502
RNA5SP154	0.043	0.211370233	0.495619502
NLGN1	0.043	0.21137566	0.495619502
OR2S1P	0.043	0.211490931	0.49575148
SALL3	0.043	0.211577314	0.495880751
RP11-392M9	0.043	0.211647061	0.495883337
PDE1B	0.043	0.21164754	0.495883337
ECM2	0.043	0.21165894	0.495883337
CYP21A2	0.043	0.211899525	0.496342826
UQCR10	0.043	0.211969311	0.496457456
AC006195	0.042	0.212034057	0.496514809
MIR216B	0.042	0.212035499	0.496514809
ARG2	0.042	0.212126522	0.49662978
RCAN2	0.042	0.21218382	0.49663425
CTD-2021J15	0.042	0.212201209	0.49663425
FGF13-AS1	0.042	0.212237663	0.496659152
NAALADL1	0.042	0.212243133	0.496659152
TRIM42	0.042	0.212489629	0.497056614
AC006538	0.042	0.212501766	0.497056614
AC104134	0.042	0.212519934	0.497062657
B3GAT3P1	0.042	0.212543577	0.497093544
MEMO1P5	0.042	0.212592339	0.497183174
NAGK	0.042	0.212638592	0.497211576
RP11-572F4	0.042	0.212646242	0.497211576
MUC19	0.042	0.212690361	0.497257474
THBS1	0.042	0.212697194	0.497257474
BMPER	0.042	0.212723066	0.497293548
RP11-169L17	0.042	0.21283487	0.497384022

MRO	0.042	0.212940556	0.497582175
CCDC28B	0.042	0.212982744	0.497631924
TSPY15P	0.042	0.213154074	0.497836853
RP11-223J6	0.042	0.213320179	0.49801631
MIAT_exon1	0.042	0.21335639	0.49801631
IGHD5OR15-5B	0.042	0.21335639	0.49801631
IGHD1OR15-1A	0.042	0.21335639	0.49801631
RNU2-43P	0.042	0.21341406	0.498126511
SNAP91	0.042	0.21344743	0.498155571
GSC2	0.042	0.213520539	0.498292566
BRD7P6	0.042	0.21360739	0.498357948
HSPD1P21	0.042	0.213873839	0.498601143
NAALADL2-AS1	0.042	0.213879131	0.498601143
MXD4	0.042	0.21390251	0.49863124
FAM3C	0.042	0.213934757	0.498635568
PHB2	0.042	0.213945842	0.498635568
RN7SL163P	0.042	0.213992589	0.498694772
SCNM1	0.042	0.214034542	0.498701899
ADRA2A	0.042	0.214042002	0.498701899
AC009108	0.042	0.214048003	0.498701899
SBNO2	0.042	0.214076885	0.498742161
CPNE9	0.042	0.21412175	0.498782006
PDZD11	0.042	0.214124276	0.498782006
CAP1	0.042	0.214167237	0.498857681
RNU6-809P	0.042	0.21420111	0.498912182
MIR711	0.042	0.214347158	0.499097183
PON3	0.042	0.214393835	0.499165779
KIRREL-IT1	0.042	0.214530186	0.499312377
SC22CB-109E1	0.042	0.214634772	0.499506978
PTGIS	0.042	0.2148593	0.499907371
RP11-90C1	0.042	0.215097518	0.500241686
YBX1P2	0.042	0.215156952	0.500339353
IFITM9P	0.042	0.21517103	0.500339353
MIR4310	0.042	0.215211634	0.500375407
RNU1-18P	0.042	0.215253617	0.500409235
SLC35A2	0.042	0.215264944	0.500411141
ULBP3	0.042	0.215345688	0.500463617
NYAP1	0.042	0.215407385	0.500484629
ERP29	0.042	0.215440927	0.50050266
NFIA-AS1	0.042	0.215598006	0.500703166
VPS72	0.042	0.215611339	0.500703166
OR6F1	0.042	0.215644839	0.500721203
RP11-552C15	0.042	0.215748715	0.50085124
AC003681	0.042	0.215808332	0.500940804
PGBD4P6	0.042	0.2158792	0.500996194
RPS7P12	0.042	0.215885214	0.500996194
RP11-399O19	0.042	0.215899699	0.500996194
RNU4ATAC13P	0.042	0.215905828	0.500996194
CTD-2588E21	0.042	0.21598798	0.501129384
AL354931	0.042	0.215994793	0.501129384
LDLRAD4-AS1	0.042	0.216034439	0.50117598
RP1-37E16	0.042	0.216072316	0.501224023

TXN2	0.042	0.216077679	0.501224023
SMURF1	0.042	0.216144475	0.501318979
TENM2	0.042	0.216227118	0.501424138
AC079135	0.042	0.216270181	0.501499582
ACSL1	0.042	0.216496969	0.501903287
C6orf118	0.042	0.216577243	0.501994259
AC022616	0.042	0.216580932	0.501994259
RP11-972L6	0.042	0.216617001	0.502025198
MFAP4	0.042	0.216654963	0.502025198
SYT12	0.042	0.216702967	0.502045372
AL137852	0.042	0.216705834	0.502045372
PLA2G2E	0.042	0.216786129	0.502126137
SEMG1	0.042	0.216848256	0.502126137
RP11-73M7	0.042	0.216848646	0.502126137
SNAPC1	0.042	0.216855778	0.502126137
LINC01204	0.042	0.216856467	0.502126137
RP11-416I2	0.042	0.216890834	0.502126137
AC007967	0.042	0.216942131	0.502126137
NACAP1	0.042	0.216952876	0.502126137
TMEM208	0.042	0.216971607	0.502126137
MYLK-AS2	0.042	0.217018197	0.502182766
AL132671	0.042	0.217089078	0.502211563
RP11-275O4	0.042	0.217115	0.502211563
SPPL2B	0.042	0.217140851	0.502246967
GAPDHP25	0.042	0.217300159	0.502407854
RPS2P32	0.042	0.2173268	0.502408673
DDX11L10	0.042	0.217402001	0.502460576
ATP5G3	0.042	0.217662822	0.502794997
ASB4	0.042	0.217720277	0.50285369
HIGD1AP4	0.042	0.217751698	0.502878346
WI2-89031B12	0.042	0.217980159	0.503236683
CNIH4	0.042	0.218100871	0.503391818
DBIP1	0.042	0.218129746	0.503423452
RNU6-559P	0.042	0.218173985	0.503423452
RP11-380I20	0.042	0.21823553	0.503483027
ALX4	0.042	0.218247179	0.503485512
KRT8	0.042	0.218260931	0.50349285
RGN	0.042	0.218285878	0.503513372
SLC45A3	0.042	0.218371162	0.503649557
MEMO1P1	0.042	0.218548147	0.503981035
KB-1582A10	0.042	0.218557211	0.503981035
PAUPAR	0.042	0.218627189	0.504117991
HPGD	0.042	0.218647117	0.504139536
RHOG	0.042	0.218749196	0.504214329
ANKS6	0.042	0.218786062	0.504214329
CFD	0.042	0.218798352	0.504214329
CCDC140	0.042	0.218802737	0.504214329
CXCL16	0.042	0.218803063	0.504214329
KCNA4	0.042	0.21881099	0.504214329
AC010872	0.042	0.218814859	0.504214329
TRBVB	0.042	0.218828914	0.504214329
CTD-2272G21	0.042	0.218865826	0.504214329

AL359771	0.042	0.218998271	0.504345422
LINC00906	0.042	0.219021315	0.504345422
CPT1C	0.042	0.219037692	0.504345422
ECSIT	0.042	0.219097966	0.504422045
OR2T8	0.042	0.219146131	0.504508545
RPL27	0.042	0.219218756	0.504591537
COX8C	0.042	0.219280194	0.504625948
CTD-2311M21	0.042	0.219281891	0.504625948
UTP11L	0.042	0.219305533	0.504635409
DAZ2	0.042	0.219317789	0.504635409
LCE3B	0.042	0.219317789	0.504635409
RNU6-472P	0.042	0.219361778	0.504646607
OLFML1	0.042	0.219385151	0.504668497
SFTPA2	0.042	0.219428553	0.504730396
ST8SIA1	0.042	0.219439336	0.504730396
SLC12A9	0.042	0.219484086	0.504764095
MYEOV2	0.042	0.219489629	0.504764095
HPSE2	0.042	0.219511599	0.504764095
OR2M4	0.042	0.219521478	0.504764095
RP4-718P11	0.042	0.219545447	0.504769057
RP11-140C5	0.042	0.219762914	0.50497652
WWC2-AS2	0.042	0.219817899	0.505078496
RPL21P127	0.042	0.21988876	0.505192569
AC073283	0.042	0.219922489	0.505221319
AC226118	0.042	0.220041716	0.505446456
CTD-2636A23	0.042	0.220187437	0.505683627
RNY3P2	0.042	0.220208392	0.505707368
RNU2-56P	0.042	0.220265165	0.505806081
RP11-497E19	0.042	0.220366481	0.505910866
MIR3126	0.042	0.220435348	0.505990582
RPL34P31	0.042	0.220437957	0.505990582
AL135745	0.042	0.220497226	0.506012534
ATRN	0.042	0.220585617	0.506134372
NDUFB9	0.042	0.220623323	0.506172118
XAGE1A	0.042	0.220664535	0.506242283
STX19	0.042	0.220680963	0.506255587
RPL23AP4	0.042	0.220745445	0.506354733
RNF145	0.042	0.220915856	0.506623626
TMEM185A	0.042	0.221027046	0.506805408
PSMD8	0.042	0.221095967	0.50691463
PLCL1	0.042	0.2212799	0.507142455
MIR376B	0.042	0.221291169	0.507142455
OR51D1	0.042	0.22131465	0.507171863
RP3-510D11	0.042	0.221401343	0.50734612
RP11-372K20	0.042	0.221453624	0.507395496
DLX2-AS1	0.042	0.221557789	0.507455937
RP11-278H7	0.042	0.221559517	0.507455937
RP11-666A20	0.042	0.221634079	0.507488764
RALY	0.042	0.221645819	0.507491248
SNORD116-10	0.042	0.221665218	0.507511264
PRMT2	0.042	0.221723563	0.507563095
ELL2P3	0.042	0.221731606	0.507563095

GTPBP6	0.042	0.221741141	0.507563095
ABL1	0.042	0.221752929	0.507565684
OR10B1P	0.042	0.221808787	0.50762035
COL5A1-AS1	0.042	0.22190577	0.507744707
POLR1E	0.042	0.222039909	0.50797842
NUTF2P2	0.042	0.22211177	0.508045204
RPL39P6	0.042	0.222126713	0.508054364
EBNA1BP2	0.042	0.222141564	0.508054364
AL122003	0.042	0.22219297	0.508117786
RP11-74B21	0.042	0.222258537	0.508139301
AP002967	0.042	0.222324382	0.508189831
RN7SKP200	0.042	0.222374218	0.508215081
RPS23P1	0.042	0.222378111	0.508215081
RP11-450H5	0.042	0.222437131	0.508284314
CSRNP3	0.042	0.22259942	0.50834244
TTTY14	0.042	0.22273352	0.508452165
RP11-248E9	0.042	0.222738068	0.508452165
MGAT4C	0.042	0.222847024	0.508613682
RP11-761B3	0.042	0.222872899	0.508613682
RPL10A	0.042	0.222883967	0.508614571
PCDHA6	0.041	0.223034405	0.50886034
Z69720	0.041	0.22309683	0.50890525
RPSAP58	0.041	0.22315558	0.508990508
GALR1	0.041	0.223219868	0.509046627
RNA5SP461	0.041	0.223222937	0.509046627
RP11-587P21	0.041	0.22328427	0.509113364
ASS1P11	0.041	0.223341553	0.509214484
RAC1	0.041	0.22337462	0.509246232
PCDHGB2	0.041	0.223463173	0.509350908
RP11-628M2	0.041	0.223524796	0.509429769
APLF	0.041	0.223588405	0.509489736
THEM6	0.041	0.223634176	0.509525166
TOR4A	0.041	0.22363584	0.509525166
KCNMB2	0.041	0.223725603	0.50958294
MTATP6P9	0.041	0.223836777	0.509811782
OR2D2	0.041	0.223881111	0.509819689
FAM167B	0.041	0.224042662	0.510084082
FAM46D	0.041	0.224072835	0.510105488
EIF3G	0.041	0.224109753	0.510141629
PXDNL	0.041	0.224190614	0.510227285
GTF2H2	0.041	0.224288176	0.510424931
RP11-227L6	0.041	0.224404127	0.510591217
SUN2	0.041	0.224574573	0.510765495
RN7SK	0.041	0.224578121	0.510765495
RP1-169K13	0.041	0.224665649	0.51077218
EFHC2	0.041	0.224858725	0.51103963
RP11-10D7	0.041	0.224956454	0.511154336
REEP4	0.041	0.225096515	0.511360445
IFNA13	0.041	0.225133553	0.511406443
RP11-1182P23	0.041	0.225230433	0.511515247
RP11-295M3	0.041	0.225234089	0.511515247
MIR3675	0.041	0.225250578	0.511515247

RNU6-1076P	0.041	0.225397631	0.511678407
RP11-172E9	0.041	0.225451295	0.511751446
CRH	0.041	0.225666319	0.512099531
RN7SL98P	0.041	0.225697772	0.512114574
UGT1A8	0.041	0.225731456	0.512114574
RNU1-148P	0.041	0.225741695	0.512114574
RP11-473M14	0.041	0.225773543	0.512114574
CNGA1	0.041	0.225800548	0.512114574
MMP20	0.041	0.22592249	0.512281186
RP11-203I2	0.041	0.226069816	0.512544191
MIR181B1	0.041	0.22608034	0.512544191
CTD-2595P9	0.041	0.22616946	0.512615763
PGAM1P2	0.041	0.226398093	0.513029462
SUSD6	0.041	0.226402109	0.513029462
LRRC14B	0.041	0.226421025	0.513047916
SRIP3	0.041	0.226477058	0.513112117
AC016751	0.041	0.226522792	0.513180849
PTDSS2	0.041	0.226534581	0.513183147
MIR7158	0.041	0.226673105	0.513329158
LINC01116	0.041	0.226674461	0.513329158
PTCD1	0.041	0.226701772	0.513329158
REG1A	0.041	0.226709589	0.513329158
AC114765	0.041	0.226776391	0.513377785
AC131213	0.041	0.226890116	0.513515196
SLC22A3	0.041	0.226897563	0.513515196
HMGB1P16	0.041	0.226913711	0.513515196
CNN2P3	0.041	0.226968408	0.513564126
CTD-3094K11	0.041	0.226972332	0.513564126
LGR5	0.041	0.227002823	0.513595138
PKP3	0.041	0.227007606	0.513595138
LA16c-4G1	0.041	0.227040835	0.513622124
RPS28P1	0.041	0.227068634	0.513649328
RP13-60M5	0.041	0.227119871	0.513654641
RP11-335M9	0.041	0.227145692	0.513654641
AC018359	0.041	0.22715254	0.513654641
CTD-2031P19	0.041	0.227174625	0.51368019
RP11-406O16	0.041	0.227326036	0.513883811
MIR4735	0.041	0.227353503	0.513913873
RN7SL180P	0.041	0.227458407	0.514080886
CCDC64B	0.041	0.227488705	0.514097487
CCDC82	0.041	0.227750815	0.514470122
RP11-63N8	0.041	0.227828286	0.51459631
RP11-140M13	0.041	0.227981858	0.514845861
RNU1-46P	0.041	0.228057056	0.514966501
AC010150	0.041	0.228091201	0.514994769
SERPING1	0.041	0.228158523	0.515119436
AC121332	0.041	0.228168048	0.515119436
TTC7A	0.041	0.228288136	0.515270066
RN7SKP286	0.041	0.228299682	0.515270066
TLE1	0.041	0.228382529	0.515408204
BCYRN1	0.041	0.228483499	0.515550876
PHPT1	0.041	0.228497232	0.515550876

RP11-702M1	0.041	0.228499873	0.515550876
DPH3P2	0.041	0.228571468	0.515621912
HMGB3P30	0.041	0.228585488	0.515621912
KRT16P3	0.041	0.228788089	0.51590788
MTND4P24	0.041	0.228802689	0.515916375
OR10H1	0.041	0.228865997	0.516010264
RP11-99H20	0.041	0.229108361	0.516336721
AP001282	0.041	0.229179195	0.516367975
RP11-843F11	0.041	0.229194628	0.516367975
FLYWCH1	0.041	0.229200707	0.516367975
MT-TA	0.041	0.229205219	0.516367975
C6	0.041	0.229306517	0.516465595
RPL35AP35	0.041	0.229396091	0.516600026
TBCAP1	0.041	0.229398743	0.516600026
AC234582	0.041	0.229429175	0.516644128
PRSS45	0.041	0.229626945	0.51698222
RNF26	0.041	0.229638244	0.51698222
MIR7108	0.041	0.229681866	0.51698222
TTC22	0.041	0.229745335	0.517063664
NOC4L	0.041	0.229832942	0.517186559
PDSS1	0.041	0.22988556	0.517238418
SMYD1	0.041	0.229930036	0.517238418
CDH12	0.041	0.230028808	0.517398356
RP11-176N18	0.041	0.230078396	0.517450043
CLIC4P3	0.041	0.230144724	0.517545812
CMTM7	0.041	0.230199538	0.517595761
AC107079	0.041	0.230221814	0.51759799
HILPDA	0.041	0.230222265	0.51759799
BCRP2	0.041	0.230276892	0.517623066
FIZ1	0.041	0.230356748	0.517729264
FCN3	0.041	0.230502978	0.517904378
RP11-530C5	0.041	0.230515307	0.517904378
MIR34C	0.041	0.230522828	0.517904378
SYN2	0.041	0.230525726	0.517904378
RP11-388K12	0.041	0.230589254	0.517958516
BANF1	0.041	0.230666503	0.518083166
RNA5SP88	0.041	0.230759292	0.518180169
AC007881	0.041	0.230789972	0.518189037
AC096951	0.041	0.230919472	0.518309142
PSMB1	0.041	0.231038088	0.518504446
CHCHD2P2	0.041	0.231044433	0.518504446
MIR4674	0.041	0.23110351	0.518526729
RNU6-829P	0.041	0.231155312	0.518618526
TTLL11-IT1	0.041	0.231167058	0.518620447
AL132988	0.041	0.231213769	0.518651949
TGFB1	0.041	0.231241085	0.518664367
SNORD114-20	0.041	0.231310748	0.518768171
RP11-525J21	0.041	0.231320042	0.518768171
XXbac-BCX196D17	0.041	0.231398573	0.518868385
RP11-15I11	0.041	0.23148018	0.518931822
LHX5	0.041	0.231594677	0.519101916
AC023798	0.041	0.231596673	0.519101916

ZMAT4	0.041	0.23162145	0.519101916
SPRN	0.041	0.231717229	0.51924327
RP11-704M14	0.041	0.231955878	0.519618111
PRKACA	0.041	0.231974887	0.519625056
SLC5A6	0.041	0.232074846	0.519677873
RP11-174B4	0.041	0.232110118	0.519683555
RN7SKP240	0.041	0.232270844	0.519945643
AL023806	0.041	0.232333742	0.520015382
PRTFDC1	0.041	0.232454239	0.52011172
ZDHHC9	0.041	0.232685297	0.520530889
FAM110A	0.041	0.232757428	0.520643342
LINC01582	0.041	0.23283951	0.520696258
RP5-1097P24	0.041	0.232841285	0.520696258
MIR8061	0.041	0.232860098	0.520696258
RP11-699A5	0.041	0.232862166	0.520696258
RNU6-37P	0.041	0.232895155	0.520696258
IMPA2	0.041	0.232921228	0.520696258
FBXO10	0.041	0.232952285	0.520696258
AP000859	0.041	0.232965582	0.520696258
EXOSC7	0.041	0.232984415	0.520696258
RPL7P20	0.041	0.233007125	0.52071276
HNF4A	0.041	0.233029238	0.520713312
SNORD3B-2	0.041	0.233075354	0.520791926
RP11-678D18	0.041	0.233109411	0.520794723
TIMMDC1	0.041	0.233136541	0.520806474
MIR5690	0.041	0.233181528	0.520851145
ANKRD62P1	0.041	0.233239097	0.520864561
RN7SL861P	0.041	0.233261693	0.520883166
C10orf120	0.041	0.233436143	0.521111772
RP6-74O6	0.041	0.233491845	0.521172842
TCEB1P2	0.041	0.23368771	0.521499605
PPAN-P2RY11	0.041	0.233717157	0.521519398
PRR14	0.041	0.233838943	0.521717673
RP5-1185K9	0.041	0.233840199	0.521717673
TMEM74B	0.041	0.233853762	0.521723492
SSR4P1	0.041	0.233888772	0.52175272
RNU6-1161P	0.041	0.23397962	0.521887846
RP11-698N11	0.041	0.23398222	0.521887846
CTD-2281E23	0.041	0.234013489	0.521892179
PPP1R35	0.041	0.234038952	0.521892179
NMRAL1	0.041	0.234163401	0.522028631
MYH3	0.040	0.234539612	0.522497175
USP9YP2	0.040	0.234563821	0.522497175
ATP8A2	0.040	0.234589978	0.522497175
GLDC	0.040	0.234609995	0.522497175
RARRES2P7	0.040	0.234616777	0.522497175
OR2AT4	0.040	0.23467369	0.522497175
PLA2R1	0.040	0.234676737	0.522497175
TRIM11	0.040	0.23471808	0.522528285
HMGN2P35	0.040	0.234731126	0.522528285
RP11-1437A8	0.040	0.234741117	0.522528285
SAA2-SAA4	0.040	0.234757013	0.522539246

RPS15AP5	0.040	0.234791345	0.522566819
AC006273	0.040	0.234875346	0.522680503
RAB24	0.040	0.235120791	0.52308006
RNU6-1290P	0.040	0.235157172	0.523112126
TBCB	0.040	0.235182674	0.523135725
FABP7	0.040	0.23525555	0.523233215
RP11-75C9	0.040	0.235284767	0.523273762
RNU4-80P	0.040	0.235315346	0.523285343
Z97055	0.040	0.235322936	0.523285343
CETN2	0.040	0.235439169	0.523494925
SNORD123	0.040	0.235532026	0.523607345
SYPL1	0.040	0.235723074	0.523954957
MIR3672	0.040	0.235967325	0.524277655
RP11-5K16	0.040	0.235978633	0.524278321
FOXE1	0.040	0.236140939	0.524555631
GATSL3	0.040	0.236155982	0.524555631
BVES	0.040	0.236221085	0.524596738
RNU6-901P	0.040	0.236324608	0.524781178
KRT8P36	0.040	0.236451882	0.524962417
RP11-44F21	0.040	0.236594161	0.525146925
JAG2	0.040	0.236601145	0.525146925
CHODL-AS1	0.040	0.236620722	0.525152518
RNU6-526P	0.040	0.236681769	0.525236547
CLIC1	0.040	0.236789169	0.525400046
SLC44A1	0.040	0.236808237	0.525400046
SNORD115-46	0.040	0.23685519	0.525435171
RP11-789A21	0.040	0.236863399	0.525435171
SDCBPP1	0.040	0.236903885	0.525466528
RP11-397C12	0.040	0.236908536	0.525466528
ATP5J2	0.040	0.236977733	0.525509126
RN7SKP226	0.040	0.236987286	0.525509126
HMGB3	0.040	0.237006568	0.525509126
ATP12A	0.040	0.237007077	0.525509126
NPW	0.040	0.237118865	0.525708042
RP11-21M7	0.040	0.23721989	0.5258425
UBOX5	0.040	0.237223675	0.5258425
ARPIN	0.040	0.237340636	0.526019821
FRRS1	0.040	0.237349969	0.526019821
ZC3H3	0.040	0.237401845	0.526041578
OR9H1P	0.040	0.237452334	0.526093611
OR11K1P	0.040	0.237497263	0.526130618
C4A	0.040	0.237830188	0.526549756
PGC	0.040	0.238017969	0.52691382
FAM83H	0.040	0.23806914	0.526956311
MT-TK	0.040	0.238112237	0.526978231
RNU6-1247P	0.040	0.23819824	0.527095088
PSMD2	0.040	0.238514447	0.527672221
LA16c-380F5	0.040	0.238596346	0.527776681
RPL26	0.040	0.238620092	0.527776681
TUBA4A	0.040	0.238628153	0.527776681
RNU6-160P	0.040	0.238717924	0.527912398
RP11-291H24	0.040	0.238731153	0.527912398

PNKP	0.040	0.238788539	0.527939999
RAB40AL	0.040	0.238798753	0.527939999
RP11-87O11	0.040	0.238800462	0.527939999
LINC00607	0.040	0.238929811	0.528125034
AC002386	0.040	0.238949797	0.528136264
RNU6-989P	0.040	0.239130541	0.528341602
RP11-293K19	0.040	0.239185129	0.528378889
HNRNPA0	0.040	0.239260985	0.528383198
MED15	0.040	0.239286523	0.528383198
RNU11	0.040	0.239288954	0.528383198
CTD-2046J7	0.040	0.239503572	0.528680615
RP11-13K12	0.040	0.23955614	0.528724236
OR1I1	0.040	0.239625139	0.528788709
AC253572	0.040	0.239659744	0.528830366
FGF8	0.040	0.239724847	0.528900508
MIR362	0.040	0.239945287	0.529190748
RP11-65M17	0.040	0.239977337	0.529236924
UACA	0.040	0.240010259	0.529260516
XPNPEP1	0.040	0.240089019	0.529383783
TMSB4Y	0.040	0.240099504	0.529383783
SNORD100	0.040	0.240164339	0.529479814
RNU6-202P	0.040	0.240184643	0.529497963
MRPS11	0.040	0.24027527	0.529673238
VN1R78P	0.040	0.240326784	0.529719269
HBZ	0.040	0.240346437	0.529727296
AC004967	0.040	0.24039088	0.529780387
AC000067	0.040	0.240401741	0.529780387
ANKRD9	0.040	0.240474985	0.529868245
GPR21	0.040	0.240540056	0.529881891
TP63	0.040	0.24071449	0.530097553
C19orf12	0.040	0.240791658	0.530157259
RP11-632K5	0.040	0.240827712	0.530203922
BCRP4	0.040	0.240971241	0.530432643
RP11-193H5	0.040	0.240983046	0.530432643
RP3-455H14	0.040	0.241033231	0.53050763
LPP	0.040	0.241072197	0.530521464
RNA5SP140	0.040	0.241222402	0.530706767
ZNF282	0.040	0.241276329	0.530748301
SACS-AS1	0.040	0.241283561	0.530748301
RP11-545D22	0.040	0.241461246	0.531034113
DNM1P28	0.040	0.241586172	0.531157574
AC013701	0.040	0.241594084	0.531157574
SGSM3	0.040	0.241599238	0.531157574
PLA2G12B	0.040	0.241624525	0.531160519
MTND1P35	0.040	0.241630248	0.531160519
RNU6-183P	0.040	0.241673395	0.531189177
ADIPOQ-AS1	0.040	0.241727137	0.531238415
PKMP4	0.040	0.241749608	0.531251262
CEACAM18	0.040	0.241800594	0.531314274
OBSCN	0.040	0.241884176	0.531399864
AC093616	0.040	0.241923547	0.531437332
MTND5P10	0.040	0.242060638	0.531713957

AC010311	0.040	0.242083889	0.531740505
RP11-483L5	0.040	0.242116946	0.531788591
RP1-135L22	0.040	0.242175011	0.531882533
GRXCR2	0.040	0.242383571	0.532177868
GAPDHP14	0.040	0.242402402	0.532194331
CTD-2162K18	0.040	0.242539672	0.532352686
SCARNA17	0.040	0.242554375	0.532352686
AP000479	0.040	0.242595148	0.532352686
LINC01165	0.040	0.242623794	0.532352686
CYCSP6	0.040	0.242626278	0.532352686
COMMD4	0.040	0.242643402	0.532352686
RP11-345F18	0.040	0.242654779	0.532352686
PCDHGA5	0.040	0.242670296	0.532352686
VGLL3	0.040	0.242688374	0.532352686
TARBP2	0.040	0.242694327	0.532352686
OR5M1	0.040	0.242700522	0.532352686
RP11-127O4	0.040	0.242702227	0.532352686
MT1E	0.040	0.242709098	0.532352686
PLEKHG3	0.040	0.242872952	0.532589441
KANK1	0.040	0.242941339	0.532690353
PRCC	0.040	0.243067118	0.532865874
RP11-671C19	0.040	0.24310926	0.532865874
ENSAP3	0.040	0.243110894	0.532865874
CACTIN	0.040	0.243129363	0.53288183
CTC-512J14	0.040	0.243141063	0.53288295
SLC25A5P7	0.040	0.243265778	0.532976066
SAA2	0.040	0.243292679	0.532976066
AC078986	0.040	0.24338303	0.533069835
C8G	0.040	0.243794222	0.533847697
RP4-806M20	0.040	0.24380779	0.533852862
PHYH	0.040	0.243852173	0.533900954
MIR6810	0.040	0.243958288	0.534050771
RNVU1-18	0.040	0.24402783	0.534050771
TMEM44	0.040	0.244055299	0.534050771
RPS5P7	0.040	0.244057699	0.534050771
FZD10	0.040	0.244078318	0.534050771
PSD	0.040	0.244088943	0.534050771
RP13-492C18	0.040	0.244264355	0.534336691
RNU1-114P	0.040	0.244276414	0.534338527
RP11-709A23	0.040	0.244287819	0.534338934
ADH4	0.040	0.244397468	0.534482607
NXF2	0.040	0.24483324	0.535015953
HPN-AS1	0.040	0.244853058	0.535034713
SLC4A3	0.040	0.244989918	0.535255501
CLEC16A	0.040	0.245001681	0.535255501
DGAT2	0.040	0.245009005	0.535255501
LINC00869	0.040	0.245079367	0.535370764
PABPC1P8	0.040	0.245085535	0.535370764
TXNDC2	0.040	0.245285085	0.535683819
RP11-503L19	0.040	0.245368475	0.535712358
GABRA3	0.040	0.245383992	0.535712358
RP11-873E20	0.040	0.245410281	0.535712358

CTC-286N12	0.040	0.245489313	0.53585955
LCE2B	0.040	0.245541214	0.535948277
CCDC181	0.040	0.245803119	0.536200482
KEAP1	0.040	0.245816466	0.536205038
GJB3	0.040	0.245845337	0.536226233
RNA5S16	0.040	0.245983934	0.536370005
GJB1	0.040	0.246002954	0.536370005
RIPPLY1	0.040	0.246018107	0.536374657
PCDHGA7	0.039	0.246170557	0.536619926
S1PR5	0.039	0.246190384	0.536619926
RP4-710M16	0.039	0.246209802	0.536619926
RP11-513O17	0.039	0.24621149	0.536619926
SLC35G2	0.039	0.246249402	0.536657832
RPPH1-3P	0.039	0.246329944	0.536710565
UBASH3B	0.039	0.246455261	0.536812831
WFDC9	0.039	0.246579514	0.536921408
CATSPERB	0.039	0.246584541	0.536921408
AC103881	0.039	0.246696644	0.53703966
NAV1	0.039	0.246775651	0.537074354
NLRP12	0.039	0.246816911	0.537075598
HDAC8	0.039	0.246824505	0.537075598
RP11-162A23	0.039	0.246896689	0.537116509
MMD	0.039	0.246899694	0.537116509
OR13D3P	0.039	0.246933051	0.537147537
GABRD	0.039	0.246968204	0.537167404
MIR892A	0.039	0.246986014	0.53718161
PCDHB18P	0.039	0.247027534	0.537222099
PRR16	0.039	0.247042977	0.537222099
RP11-309J13	0.039	0.24704968	0.537222099
MIR921	0.039	0.247144189	0.53732933
RP11-753N8	0.039	0.247370701	0.537576401
RNU6-173P	0.039	0.247398479	0.537602862
RP13-43E11	0.039	0.247411762	0.537602862
HS1BP3-IT1	0.039	0.247416741	0.537602862
TMPRSS11A	0.039	0.24749621	0.537608434
FBXL12	0.039	0.247593439	0.537765995
LINC01324	0.039	0.247672643	0.53781539
SETP2	0.039	0.247728717	0.537878746
MMP24-AS1	0.039	0.247740583	0.537878746
NUDT22	0.039	0.247876376	0.538012493
AC102948	0.039	0.247897637	0.53801268
AP001172	0.039	0.247975461	0.538072574
WDR63	0.039	0.247978189	0.538072574
GRAMD4P1	0.039	0.248061886	0.538169884
MIR4760	0.039	0.24808819	0.538202433
AC009500	0.039	0.248242394	0.538438859
MROH6	0.039	0.248334248	0.538586959
LUZP4	0.039	0.248344599	0.538586959
CELA2A	0.039	0.248376155	0.538630868
RPL12P38	0.039	0.248436834	0.538737927
RNU6-1255P	0.039	0.248481009	0.538749037
CTD-2576N18	0.039	0.248500489	0.538749037

RP1-155D22	0.039	0.248513206	0.538749037
TARDBPP1	0.039	0.248627406	0.538884613
RP11-554D15	0.039	0.248678034	0.538917446
CTD-2541M15	0.039	0.248706578	0.538954781
RP11-521D12	0.039	0.248743728	0.539010762
RP11-106D4	0.039	0.248781537	0.539059654
RP11-401N18	0.039	0.24883031	0.539059654
HORMAD2	0.039	0.248831492	0.539059654
ULK4P1	0.039	0.248834201	0.539059654
MRPL11	0.039	0.248915441	0.539162075
LDHB	0.039	0.248939902	0.539176749
OTOS	0.039	0.24897333	0.539176749
C16orf47	0.039	0.248994683	0.539176749
UQCRFS1	0.039	0.249031479	0.539176749
RNU6-1189P	0.039	0.249035424	0.539176749
RP11-350O14	0.039	0.249054183	0.539192853
MLNR	0.039	0.24909874	0.539196568
RPS4XP20	0.039	0.249101184	0.539196568
AC004448	0.039	0.24912641	0.539226664
RP1-43E13	0.039	0.249253552	0.539313441
MTND1P1	0.039	0.249270073	0.539313441
KRTAP13-3	0.039	0.24932945	0.53934749
RPL29	0.039	0.249359598	0.539363711
RP11-120E11	0.039	0.249449484	0.539518462
RPL7P40	0.039	0.249527438	0.539579719
SLC25A15P5	0.039	0.249611261	0.539736472
RP11-195L15	0.039	0.249658576	0.539765264
DUOXA2	0.039	0.249703568	0.539838032
DCTN1	0.039	0.24973075	0.539847787
CTD-2325B11	0.039	0.249827948	0.53998008
IP6K3	0.039	0.249886414	0.540061725
WDR70	0.039	0.249912083	0.540092693
RP11-313E4	0.039	0.249944041	0.540115486
MIR605	0.039	0.24996967	0.540115486
UGT8	0.039	0.249990106	0.540115486
AC012360	0.039	0.250142142	0.540320188
TBL1YP1	0.039	0.250212347	0.540398305
NME2P1	0.039	0.25030119	0.540514228
EEF1GP4	0.039	0.250663479	0.541004646
PCAT7	0.039	0.250734188	0.541052139
POLR2F	0.039	0.250777347	0.541062385
LY6E	0.039	0.250877205	0.541196151
RP1-309I22	0.039	0.250946249	0.541239628
NEFMP1	0.039	0.25094863	0.541239628
CYHR1	0.039	0.250975146	0.541239628
RP1-45C12	0.039	0.25098607	0.541239628
LINC01508	0.039	0.250988272	0.541239628
RP11-418H16	0.039	0.251009802	0.541245056
FRAT2	0.039	0.251013518	0.541245056
BBOX1	0.039	0.251037225	0.54127167
CDCA4	0.039	0.251116689	0.541358062
BPIFB1	0.039	0.251134126	0.541358062

MIR146B	0.039	0.251180632	0.541381502
RP11-849I19	0.039	0.251190468	0.541381502
AC098870	0.039	0.251281981	0.541480721
ZNF784	0.039	0.251373053	0.541556169
RN7SKP108	0.039	0.251373847	0.541556169
DLX2	0.039	0.251442354	0.541654757
PLXNB2	0.039	0.251466383	0.541672592
MIR7854	0.039	0.25147338	0.541672592
RPL21P17	0.039	0.251515527	0.541673502
RNU6-1196P	0.039	0.251519296	0.541673502
RN7SL670P	0.039	0.251671755	0.541902974
C1orf145	0.039	0.251682738	0.541902974
LINC01315	0.039	0.251726387	0.541946845
CTSE	0.039	0.251741576	0.541946845
UGT1A1	0.039	0.251795959	0.541999734
MRAP	0.039	0.251874382	0.542119539
HSPA2	0.039	0.25201623	0.542302308
HMGN1P19	0.039	0.252059483	0.542330263
RP11-126L15	0.039	0.252267343	0.542622019
C1orf21	0.039	0.252284332	0.542624789
OC90	0.039	0.252291417	0.542624789
HMGB4	0.039	0.252393062	0.542755698
TRI-TAT2-2	0.039	0.252526725	0.542934738
DEFB108P3	0.039	0.252575018	0.542989545
MLF2	0.039	0.252628807	0.543043344
RGS3	0.039	0.252634249	0.543043344
RNU6-255P	0.039	0.252729365	0.543198766
CH17-335B8	0.039	0.252892708	0.543427226
PTF1A	0.039	0.253003797	0.543567839
AQP8	0.039	0.253025955	0.543590923
RNU6-938P	0.039	0.253080811	0.543684249
PDCL3P5	0.039	0.253154099	0.543738869
MIR3666	0.039	0.253283097	0.543868511
ATP11B	0.039	0.253286256	0.543868511
RP5-1155K23	0.039	0.253292196	0.543868511
FAM138E	0.039	0.253349362	0.543934863
POMT2	0.039	0.25335736	0.543934863
CRCT1	0.039	0.253542624	0.544185424
SLC5A12	0.039	0.253638638	0.544317911
AC099778	0.039	0.253726856	0.544433635
CHST9	0.039	0.253843493	0.544585767
RP11-162J8	0.039	0.25388283	0.544629277
TGFBR1	0.039	0.253961961	0.544741773
RP11-1090M7	0.039	0.253993516	0.544784922
NRN1L	0.039	0.254005653	0.54478642
CTB-127C13	0.039	0.254082588	0.544804224
SDC4P	0.039	0.254131343	0.544878231
ZFP64P1	0.039	0.254146216	0.544878231
DPP10-AS1	0.039	0.254151424	0.544878231
DYNLL1P7	0.039	0.25419186	0.544915862
RP11-429D19	0.039	0.254258425	0.545034027
ARPC2	0.039	0.254300511	0.545069334

HOXA7	0.039	0.254321115	0.545069334
RBM3	0.039	0.254343563	0.545069334
SNORD114-4	0.039	0.254400048	0.545092274
MDH2	0.039	0.254510127	0.545205496
GAPDHP56	0.039	0.254551377	0.545222865
RN7SKP280	0.039	0.254696514	0.545457569
RP11-168A11	0.039	0.254814828	0.545661877
LMO4	0.039	0.254881806	0.545738421
RN7SKP156	0.039	0.25488511	0.545738421
RPL10P15	0.039	0.25495595	0.545841366
AC002310	0.039	0.25498106	0.545858599
LINC00864	0.039	0.254986922	0.545858599
RPL23AP12	0.039	0.255022222	0.545860562
C20orf203	0.039	0.255231769	0.546174392
DUSP14	0.039	0.255303421	0.54626839
AC079398	0.039	0.255311365	0.54626839
CTD-3065J16	0.039	0.255315984	0.54626839
ZNF22	0.039	0.255393423	0.54640953
HMGB1P5	0.039	0.255503327	0.546521915
OLFM4	0.039	0.255526894	0.546547778
CDY9P	0.039	0.255560433	0.546562165
MRPL20P1	0.039	0.255565377	0.546562165
C8A	0.039	0.255610204	0.546627775
NAP1L1P2	0.039	0.255910843	0.546974153
CALHM3	0.039	0.25592492	0.546974153
RP11-87C7	0.039	0.255967412	0.546974153
RPS2P46	0.039	0.255997044	0.547012349
TMEM139	0.039	0.256067531	0.547094129
MYCN	0.039	0.256153589	0.547175592
DAD1	0.039	0.256193892	0.5472041
ADAM9	0.039	0.256198471	0.5472041
RNU6-1124P	0.039	0.256314257	0.547396041
C17orf74	0.039	0.256515919	0.54763076
NELFB	0.039	0.256546191	0.547665654
MIR3180-1	0.039	0.256559979	0.547665654
RP1-182D15	0.039	0.256628601	0.547772616
ANKRD34A	0.039	0.256744936	0.547892467
ZCCHC24	0.039	0.256765344	0.547892467
CTD-2073O6	0.039	0.256806434	0.547892467
AADACL2	0.039	0.256822966	0.547892467
RP11-554F20	0.039	0.256850394	0.547892467
MSLN	0.039	0.256857308	0.547892467
CTC-304I17	0.039	0.257004564	0.548094238
RNA5SP330	0.039	0.257146228	0.548340679
ANXA5	0.039	0.257148027	0.548340679
HTR2A	0.039	0.257174176	0.548371887
NMUR2	0.039	0.257234016	0.548446104
RPS3AP39	0.039	0.257266559	0.548446104
RPL7P47	0.039	0.257300128	0.548493117
RNU6-1010P	0.039	0.257400771	0.548615608
CDO1	0.039	0.257473431	0.548715192
RN7SL632P	0.039	0.257499108	0.548720805

AC016724	0.039	0.257556735	0.548760268
RNASE10	0.039	0.257592818	0.548760268
AVP	0.039	0.257616424	0.548774373
MATN4	0.039	0.257798798	0.549040082
RP1-37N7	0.039	0.257858702	0.549086634
ARAP3	0.039	0.257886056	0.549086634
MUC5AC	0.039	0.258121942	0.549335505
ZAN	0.039	0.25812354	0.549335505
MT-RNR1	0.039	0.258140983	0.549335505
AOC4P	0.038	0.258307924	0.549510072
FTH1P7	0.038	0.258358085	0.549559601
MRPS34	0.038	0.258409214	0.549559601
RP11-397O4	0.038	0.258439201	0.549582159
RNU6-129P	0.038	0.258556817	0.549677541
MIR659	0.038	0.258571307	0.549677541
RP11-44N21	0.038	0.258717052	0.549829207
CHRNA1	0.038	0.258986251	0.550178787
HNRNPUL2-BSCL2	0.038	0.258991925	0.550178787
RP11-636O21	0.038	0.259008371	0.550189184
DPF1	0.038	0.259021223	0.550191945
AC006050	0.038	0.259092336	0.550293911
CUBN	0.038	0.259170977	0.550378451
ATP2B4	0.038	0.259237221	0.550405275
RPL18AP14	0.038	0.259389075	0.550544667
ANXA11	0.038	0.259400456	0.550544667
GFOD2	0.038	0.259418469	0.550544667
STARD13-AS	0.038	0.259445445	0.550577383
RP11-54A4	0.038	0.259494323	0.550607507
MIR301A	0.038	0.259565646	0.550664204
NKAIN1P1	0.038	0.259578853	0.550664204
HIST1H2AE	0.038	0.259649639	0.550723765
POLR2L	0.038	0.259653183	0.550723765
ATF3	0.038	0.259681146	0.550741374
LCMT1	0.038	0.259684612	0.550741374
DLX4	0.038	0.259704533	0.55074367
LINC00885	0.038	0.259821614	0.55074367
OR13J1	0.038	0.259822703	0.55074367
OSBPL5	0.038	0.259824459	0.55074367
SPRR1B	0.038	0.260026548	0.551024889
RN7SL786P	0.038	0.260082843	0.551073359
RNU4-59P	0.038	0.260093457	0.551073359
CDK16	0.038	0.260115878	0.551091588
FAS	0.038	0.260198256	0.551168045
OR11H4	0.038	0.260297826	0.551280886
OR9M1P	0.038	0.260333814	0.551327782
RP11-42O15	0.038	0.260343121	0.551327782
XXbac-BPG308K3	0.038	0.260401803	0.551369842
AC133106	0.038	0.260579156	0.551582375
RP4-625H18	0.038	0.26070305	0.551759888
SETD9	0.038	0.260709357	0.551759888
LAMA5	0.038	0.260728591	0.551776077
ZNF736P8Y	0.038	0.260757813	0.551788882

LRRC52	0.038	0.260783643	0.551818582
TMEM189	0.038	0.260847266	0.551819459
RP11-548K23	0.038	0.26093149	0.551917648
RP11-449P1	0.038	0.260938599	0.551917648
RPS4XP22	0.038	0.261028571	0.552060433
UXS1	0.038	0.261037177	0.552060433
AP003419	0.038	0.261068842	0.552060433
NOL12	0.038	0.261077305	0.552060433
AC005518	0.038	0.261244046	0.552298953
FTH1	0.038	0.261272669	0.552298953
RP11-45P15	0.038	0.261287987	0.552298953
RP11-1028N23	0.038	0.26133462	0.552371365
HIST1H3I	0.038	0.261415451	0.552414699
LINC00366	0.038	0.261499549	0.552521112
RNU6-966P	0.038	0.261509877	0.552521112
ABHD17AP3	0.038	0.261546251	0.552573451
PRAP1	0.038	0.261602936	0.552646167
ADAMTS9	0.038	0.261612593	0.552646167
PLEKHG6	0.038	0.261624905	0.552646167
RPL11	0.038	0.261704592	0.552711835
RP11-483F11	0.038	0.261719967	0.552718218
CHMP6	0.038	0.261785458	0.552776748
LINC01114	0.038	0.26184719	0.5528659
PCDHB15	0.038	0.26193399	0.553000138
FAM216B	0.038	0.262106317	0.553192305
SMAD1-AS1	0.038	0.262144962	0.553249351
MIR6776	0.038	0.262258622	0.553342108
TSSK1A	0.038	0.262310398	0.553402319
MIR4718	0.038	0.262339981	0.553440213
IFNGR1	0.038	0.262368994	0.553469083
C9orf84	0.038	0.262441486	0.55353176
RP11-347D21	0.038	0.262460109	0.553546525
RP11-714L20	0.038	0.262500823	0.553580664
MFAP3L	0.038	0.262548258	0.553609858
SCGB3A1	0.038	0.262578887	0.553649931
RALB	0.038	0.262873476	0.554050318
RNU7-195P	0.038	0.262918876	0.554121484
KRTAP21-3	0.038	0.262955171	0.554152625
TMEM186	0.038	0.262956922	0.554152625
RP11-401H2	0.038	0.263027233	0.554276273
CEACAMP2	0.038	0.263054607	0.55427988
RP11-119F19	0.038	0.263063859	0.55427988
OR10V2P	0.038	0.263207334	0.554508589
RPL21P67	0.038	0.263286628	0.554591345
GCNT3	0.038	0.263333566	0.554602802
RN7SKP39	0.038	0.26337289	0.554661094
RN7SKP101	0.038	0.263652773	0.55498058
RP11-57C19	0.038	0.263948384	0.555455537
DDIT4L	0.038	0.264035667	0.555556636
RP11-524H19	0.038	0.264043085	0.555556636
MIR770	0.038	0.264095517	0.555576043
AC035144	0.038	0.264098969	0.555576043

OR2J2	0.038	0.264157106	0.555649258
IFNA6	0.038	0.264206803	0.555704709
RCC1	0.038	0.264262494	0.555772755
SIGLEC31P	0.038	0.264318834	0.555817612
AL133269	0.038	0.264402965	0.55588058
NRCAM	0.038	0.264430479	0.55588058
LL22NC03-22D1	0.038	0.264472423	0.555886923
GAPDHP74	0.038	0.264521461	0.555886923
USP9YP5	0.038	0.264557476	0.555886923
RAX	0.038	0.264564043	0.555886923
NDUFC2	0.038	0.264579575	0.555886923
FGF13	0.038	0.264598772	0.555886923
TRBV25OR9-2	0.038	0.264703508	0.555988269
RN7SKP244	0.038	0.265017865	0.556524282
RN7SKP294	0.038	0.265173698	0.556730339
ATP10B	0.038	0.265210518	0.556732503
RPL17P46	0.038	0.265214309	0.556732503
RP11-547D24	0.038	0.265316634	0.556858598
HOTTIP	0.038	0.265402696	0.557014683
MIR5096	0.038	0.265531607	0.557162475
RNA5SP42	0.038	0.265636671	0.557333822
RP11-83A16	0.038	0.265722254	0.557403042
RP5-1136A10	0.038	0.265728181	0.557403042
RP1-118J21	0.038	0.265754216	0.55740398
SNORD126	0.038	0.265763739	0.55740398
C4orf51	0.038	0.265832583	0.557474724
DUSP5P1	0.038	0.265878634	0.557546745
CYP4Z1	0.038	0.265943731	0.557658701
ARHGAP40	0.038	0.266000525	0.557738758
NSMCE1	0.038	0.266005331	0.557738758
RP11-550I24	0.038	0.266087579	0.557837536
IGHD4OR15-4A	0.038	0.266123291	0.557863292
USP9YP16	0.038	0.266123291	0.557863292
OR7E38P	0.038	0.266195939	0.557982738
AC116311	0.038	0.266237242	0.558028478
RP11-57P19	0.038	0.266262032	0.558055879
KIAA0020	0.038	0.266340545	0.558195869
KLC4	0.038	0.266357478	0.558206793
KIF25	0.038	0.26640638	0.558235587
RP11-312B18	0.038	0.266499269	0.558383174
RNA5SP65	0.038	0.266525859	0.558384112
GAPDHP64	0.038	0.266535467	0.558384112
HIGD1AP18	0.038	0.266947724	0.559025602
RP11-319E12	0.038	0.267095931	0.559250059
MIIP	0.038	0.267108444	0.559250059
IGFBPL1	0.038	0.267160589	0.559250059
ZDHHC15	0.038	0.267207189	0.559282589
RNA5SP40	0.038	0.267211357	0.559282589
RP11-699F16	0.038	0.26725387	0.559322409
INPP5A	0.038	0.267307611	0.559338715
RNU6-1128P	0.038	0.26732654	0.559338715
AC022142	0.038	0.267454099	0.559502828

LINC00635	0.038	0.267502132	0.559571506
TNIP2	0.038	0.267547217	0.559623073
ZFP36	0.038	0.267550284	0.559623073
TBC1D10B	0.038	0.267611625	0.559692531
PKD1	0.038	0.267671795	0.55973024
LINC01451	0.038	0.267683458	0.55973024
RP11-272L14	0.038	0.267815753	0.559912634
AC093270	0.038	0.267909417	0.559949316
OR52E8	0.038	0.267917883	0.559949316
TXNDC12	0.038	0.267941851	0.559974836
RNASE4	0.038	0.268021204	0.560075937
RNU6-1064P	0.038	0.268034654	0.560075937
SOX18	0.038	0.268065847	0.560111085
ADSSL1	0.038	0.268277556	0.560436474
TDRKH	0.038	0.268339248	0.560436474
PABPC5	0.038	0.268409933	0.560534942
MIR520F	0.038	0.268433189	0.560536003
RP11-382M14	0.038	0.268445749	0.560536003
RP11-245G13	0.038	0.268694174	0.560867833
ARSB	0.038	0.268769468	0.56092897
AC010646	0.038	0.268804966	0.560966354
FAM98A	0.038	0.268818456	0.560969926
HYPM	0.038	0.268861493	0.560987797
RP11-277J24	0.038	0.268872587	0.560987797
RP11-248I9	0.038	0.268966581	0.561106935
CTD-2227E11	0.038	0.269088076	0.561286637
RP11-436D23	0.038	0.269361294	0.561659749
HORMAD2-AS1	0.038	0.269419173	0.561709004
DNAAF3	0.038	0.269420297	0.561709004
TSKU	0.038	0.26953576	0.56181255
ADM5	0.038	0.269572513	0.561847824
RP11-11A5	0.038	0.269581256	0.561847824
AMOT	0.038	0.269633934	0.561905591
RP11-734J24	0.038	0.269644367	0.561905591
LGI2	0.038	0.269702244	0.561952435
RP1-269M15	0.038	0.269874614	0.562237795
NIFKP3	0.038	0.269898624	0.562263221
DNAH3	0.038	0.26992123	0.562285721
CARM1	0.038	0.269937386	0.562294781
CBX3P7	0.038	0.269963695	0.562308126
PCDHGB4	0.038	0.269967405	0.562308126
ASB11	0.038	0.270033224	0.562384051
RP11-893F2	0.038	0.27005109	0.562384051
RPL39P26	0.038	0.270154725	0.562545254
LINC00482	0.038	0.270175744	0.562545254
SCGB1A1	0.038	0.270272853	0.562698251
IGSF10	0.038	0.270391932	0.562847751
NBPF14	0.038	0.270439697	0.562872689
SLC25A6P1	0.038	0.270503107	0.562872689
DNMBP-AS1	0.038	0.2705399	0.562872689
RP11-136I13	0.038	0.270615294	0.56288343
HIST2H3C	0.038	0.270713318	0.562953832

RP11-114H23	0.038	0.270797469	0.563052084
RN7SL124P	0.038	0.270835214	0.563081402
DMD-AS3	0.037	0.270885124	0.563111422
SLC13A3	0.037	0.270926705	0.563118404
RP11-505E24	0.037	0.2709476	0.563118404
CTB-193M12	0.037	0.271116868	0.563415462
RP11-311F12	0.037	0.271143723	0.563415462
CPN1	0.037	0.271208829	0.563415462
TMEM100	0.037	0.271369879	0.563532377
RP11-356J5	0.037	0.271387393	0.563532377
ZNF768	0.037	0.271390728	0.563532377
RN7SL183P	0.037	0.271421253	0.563532377
RN7SKP72	0.037	0.271454379	0.56355685
RNU6-791P	0.037	0.271580816	0.563711572
CYB5P4	0.037	0.271588085	0.563711572
RP11-588P7	0.037	0.271630892	0.563721202
TP53I13	0.037	0.271634028	0.563721202
MT1XP1	0.037	0.271680136	0.563735823
GALNT8	0.037	0.271714241	0.563737292
LMTK3	0.037	0.271783512	0.563797785
CYP24A1	0.037	0.271848806	0.563800397
RP11-170L9	0.037	0.271851092	0.563800397
CYCSP17	0.037	0.27186085	0.563800397
PSMA7	0.037	0.271900612	0.563800397
AC055876	0.037	0.272070462	0.563906084
RP11-680N20	0.037	0.272098222	0.563906084
AF241734	0.037	0.272228474	0.56404855
RPS12P5	0.037	0.272279592	0.564114327
FRMD6-AS2	0.037	0.272397096	0.564308678
MIR3160-1	0.037	0.272486029	0.564429045
PCDHA1	0.037	0.272516419	0.564457662
SVIL-AS1	0.037	0.272546592	0.564478813
LSM2	0.037	0.272705741	0.564633996
MEX3A	0.037	0.272797293	0.564646531
CTD-2535L24	0.037	0.272834632	0.564666422
RPL31P41	0.037	0.272842471	0.564666422
AC009238	0.037	0.272991792	0.564926357
RAB42P1	0.037	0.273261837	0.565187556
HIST1H4L	0.037	0.273263708	0.565187556
TDH	0.037	0.273320896	0.565263551
CTD-2296D1	0.037	0.273418106	0.565389256
CCZ1B	0.037	0.273519443	0.565453186
CBX3P6	0.037	0.273545555	0.565482623
ADD3-AS1	0.037	0.273595993	0.56551134
FAM83B	0.037	0.273610699	0.56551134
RP11-445O3	0.037	0.273640596	0.56551134
RNU6-301P	0.037	0.273766856	0.565690725
OS9	0.037	0.273844811	0.565701553
URM1	0.037	0.273876212	0.565701553
EGF	0.037	0.273902821	0.565705535
CTC-501O10	0.037	0.273917079	0.565710452
RNA5SP336	0.037	0.274011922	0.565830028

GS1-393G12	0.037	0.2740225	0.565830028
ADAM19	0.037	0.274043325	0.565837761
RP11-482H16	0.037	0.274094774	0.565868522
MIR7852	0.037	0.27410774	0.565868522
AC112198	0.037	0.27413802	0.565896822
RP11-495P10	0.037	0.274218664	0.565989699
RP11-486P11	0.037	0.274401575	0.566258075
KRTAP17-1	0.037	0.274408138	0.566258075
RP13-514E23	0.037	0.274452578	0.566300709
OLIG1	0.037	0.27467259	0.566607392
HOXD12	0.037	0.274716466	0.566656958
MIR411	0.037	0.274732312	0.566656958
RBMY2TP	0.037	0.274840178	0.566756718
B4GALNT4	0.037	0.274869838	0.566792776
YBX1P9	0.037	0.274891279	0.566792776
KCNK12	0.037	0.274954636	0.566852998
CDR2L	0.037	0.274958279	0.566852998
SNRNP70	0.037	0.274997699	0.566890409
SNTG1	0.037	0.275022298	0.566890409
AC135048	0.037	0.275029168	0.566890409
RNU4-40P	0.037	0.275035939	0.566890409
RN7SKP143	0.037	0.275092048	0.566945009
MIR8089	0.037	0.275110045	0.566945009
AL512635	0.037	0.275170922	0.567045928
GAPDHP44	0.037	0.275184182	0.567048719
RNA5SP155	0.037	0.275242739	0.567112585
RP11-791N19	0.037	0.275302078	0.567155133
PNMA2	0.037	0.275304539	0.567155133
MYH16	0.037	0.275487987	0.567184675
RPL6P25	0.037	0.275502109	0.567184675
RP11-356K23	0.037	0.275540031	0.567184675
GGT5	0.037	0.275576706	0.567184675
MIR5579	0.037	0.275611377	0.567184675
RP11-63A1	0.037	0.275638361	0.567184675
AC018742	0.037	0.275680868	0.567184675
MAGEA10	0.037	0.275803254	0.567293526
PRICKLE2-AS1	0.037	0.275849389	0.567341073
RP11-158I9	0.037	0.275869291	0.567355848
RP11-373J21	0.037	0.275982393	0.567426748
BPIFA1	0.037	0.275997076	0.567426748
ENTPD7	0.037	0.275998674	0.567426748
CLIP3	0.037	0.276023013	0.567451462
CKS1B	0.037	0.276058681	0.567454462
LPAR4	0.037	0.276090714	0.567454462
DOCK6	0.037	0.27626118	0.567696044
GSTA4	0.037	0.276411914	0.567858786
ADAMTS18	0.037	0.276452897	0.567869495
RP4-669K10	0.037	0.276570185	0.567903364
RP11-154F14	0.037	0.276575359	0.567903364
SCGB1C1	0.037	0.276594233	0.567914287
PRR20A	0.037	0.276606648	0.567914287
ENKUR	0.037	0.276770809	0.568130477

CBLN2	0.037	0.27683507	0.568143264
PCDHB13	0.037	0.276836683	0.568143264
PRPF18	0.037	0.276889811	0.568203327
AC109465	0.037	0.276942449	0.568262377
EIF4G1	0.037	0.276967582	0.568289465
LINC01085	0.037	0.277016134	0.568357468
LPCAT2	0.037	0.277024592	0.568357468
RP11-218E20	0.037	0.277062264	0.568363895
RNU6-210P	0.037	0.277063126	0.568363895
RP11-532M24	0.037	0.27714806	0.56851282
PLEKHG1	0.037	0.277284378	0.568686502
RN7SL783P	0.037	0.277292432	0.568686502
CTD-2524L6	0.037	0.277341373	0.568762382
NTF3	0.037	0.27735611	0.56876261
AC011591	0.037	0.277451014	0.568864758
RP11-157G21	0.037	0.277500702	0.568896318
KB-7G2	0.037	0.277614345	0.569052653
SNX19P1	0.037	0.277664342	0.56910615
RP11-467L24	0.037	0.277732738	0.569221839
LINC00973	0.037	0.277806761	0.569349052
CTBP2P3	0.037	0.277961493	0.56956813
FMN2	0.037	0.277980324	0.56958221
UNC79	0.037	0.27802404	0.569640197
RN7SL424P	0.037	0.278032545	0.569640197
RP11-578B16	0.037	0.278061214	0.56965298
RCL1	0.037	0.278071993	0.56965298
TMEM184B	0.037	0.278130853	0.569719072
RP11-287F9	0.037	0.278192759	0.569772362
RPS14	0.037	0.278264591	0.569870471
RNU6-447P	0.037	0.278380828	0.570034983
HOXC12	0.037	0.278412594	0.570051013
OR6K6	0.037	0.278496643	0.570174079
MIR1266	0.037	0.278516233	0.570179218
ATP6V0CP2	0.037	0.278523096	0.570179218
AC079150	0.037	0.278610001	0.570260331
RNU6ATAC25P	0.037	0.278793892	0.570479879
RPL10AP2	0.037	0.278866467	0.570531473
RNU6-1066P	0.037	0.278916013	0.570531473
AC079613	0.037	0.278917587	0.570531473
FAM83C-AS1	0.037	0.278920483	0.570531473
DUSP15	0.037	0.278922771	0.570531473
RP3-436N22	0.037	0.27895875	0.570580561
FGF14-AS1	0.037	0.278972618	0.570584422
GAPDHP57	0.037	0.279008355	0.570609854
NDUFA9	0.037	0.279089545	0.570652031
CSDAP1	0.037	0.279123621	0.570697206
KRTAP21-1	0.037	0.279181627	0.570781906
USP17L8	0.037	0.279291688	0.57079246
RPL31P35	0.037	0.279314025	0.57079246
RNU5B-3P	0.037	0.279336868	0.570814649
ACADL	0.037	0.279382138	0.570828742
FBXW5	0.037	0.279578604	0.571112596

WDR18	0.037	0.279892072	0.571517764
RP4-612B15	0.037	0.279894505	0.571517764
QTRT1	0.037	0.279904966	0.571517764
AC009302	0.037	0.27996908	0.571542536
RING1	0.037	0.280263971	0.571850405
ATAD3C	0.037	0.280345551	0.571914365
HAPLN2	0.037	0.280371795	0.571914365
MOB3B	0.037	0.280403032	0.571914365
RP11-478J18	0.037	0.280403392	0.571914365
CHRM4	0.037	0.280494105	0.572050389
AC006534	0.037	0.280661183	0.572268608
QRFP	0.037	0.280673252	0.572268717
AP000354	0.037	0.280773919	0.572332257
LCE3C	0.037	0.280800095	0.572332257
PCDH15	0.037	0.280806602	0.572332257
BANF1P5	0.037	0.280820754	0.572332257
AL160471	0.037	0.280848619	0.572332257
PRDX1	0.037	0.280927306	0.572443624
AC084882	0.037	0.281042568	0.572556009
LINC00901	0.037	0.281075735	0.572557341
RP11-6F6	0.037	0.281079287	0.572557341
RPL10P3	0.037	0.281149059	0.572650482
ECSCR	0.037	0.281209827	0.572700779
MYO1C	0.037	0.281308925	0.572810954
LSM3	0.037	0.28132406	0.572810954
RNVU1-3	0.037	0.28145458	0.573001202
CTD-2535I10	0.037	0.281477652	0.573001202
RP3-398G3	0.037	0.281693917	0.573343428
RNU4-28P	0.037	0.281781294	0.573447749
CCDC36	0.037	0.281872233	0.5735838
AC009498	0.037	0.281912363	0.57361711
CACNA1C-IT2	0.037	0.28191915	0.57361711
AL359955	0.037	0.281952337	0.57361711
FABP5P4	0.037	0.281975879	0.57361711
WI2-87327B8	0.037	0.281976532	0.57361711
RP11-404J23	0.037	0.281988402	0.57361711
RP11-115H13	0.037	0.282045293	0.57361711
RPL39P33	0.037	0.282052849	0.57361711
RP11-663N22	0.037	0.28223225	0.573784948
AC091320	0.037	0.282264754	0.573809879
RPS6KA2	0.037	0.282605724	0.574203256
RP11-5P18	0.037	0.282610673	0.574203256
AC017083	0.037	0.282642332	0.574208666
LL21NC02-1C16	0.037	0.28265862	0.574208666
AC104012	0.037	0.282661561	0.574208666
MAFK	0.037	0.282782561	0.574331985
CYCTP	0.037	0.282818465	0.574369891
MRPS2	0.037	0.282855326	0.574369891
ARHGEF2	0.037	0.282858267	0.574369891
BANF1P2	0.037	0.282897366	0.574369891
RNA5SP274	0.037	0.282926029	0.574369891
AC002523	0.037	0.282933128	0.574369891

GLYAT	0.037	0.282952377	0.574369891
IL25	0.037	0.282954217	0.574369891
COLEC10	0.037	0.282980239	0.574390549
AC139099	0.037	0.283018784	0.574444305
MRGPRG-AS1	0.037	0.283079918	0.574519421
COL24A1	0.037	0.283234301	0.574622388
AC010543	0.037	0.283248237	0.574622388
UGT2B17	0.037	0.283276762	0.574622388
XRCC4	0.037	0.283289239	0.574622388
CH17-353B19	0.037	0.283331368	0.574622388
RP11-553A10	0.037	0.283336743	0.574622388
AC013439	0.037	0.283418978	0.57466881
RP11-34N19	0.037	0.283534102	0.574800091
CTC-444N24	0.037	0.283534832	0.574800091
MIR4466	0.037	0.283546016	0.574800091
RP11-3B12	0.037	0.283764488	0.575002181
LDHAP7	0.037	0.283782044	0.575013292
RP11-431D12	0.036	0.284017946	0.575229436
RP11-680G10	0.036	0.284273204	0.575494342
RP11-96L14	0.036	0.284467168	0.575694855
RP11-210H10__A	0.036	0.284474424	0.575694855
RPL3P9	0.036	0.284561615	0.57579855
RP3-431P23	0.036	0.284580639	0.57579855
PRKAA1	0.036	0.28461287	0.575836739
HOXA2	0.036	0.284623694	0.575836739
RP4-593M8	0.036	0.284692531	0.575922663
AC093702	0.036	0.284848806	0.57610697
RAB9AP4	0.036	0.284854033	0.57610697
GALNT1	0.036	0.28491365	0.576139124
HNRNPA1P65	0.036	0.284967813	0.576214743
AP001891	0.036	0.285097986	0.576382597
PRKCA	0.036	0.285101069	0.576382597
AC002979	0.036	0.285122986	0.576382597
AL008721	0.036	0.285167926	0.576448054
RP11-65N13	0.036	0.285234386	0.576509001
OCSTAMP	0.036	0.285258115	0.576532494
OSTCP4	0.036	0.285305023	0.576602833
AC010983	0.036	0.285336624	0.576620524
CTA-992D9	0.036	0.28542783	0.57675313
RBM43P1	0.036	0.285446049	0.576765473
RNU6-241P	0.036	0.285592781	0.576908043
MLST8	0.036	0.285600191	0.576908043
RP11-70P17	0.036	0.285601399	0.576908043
RGMB	0.036	0.285650505	0.576958296
TPSB2	0.036	0.285734601	0.576995332
LINC01132	0.036	0.285741531	0.576995332
CLPS	0.036	0.285758967	0.577006076
PRRG2	0.036	0.285874774	0.577134286
AC002066	0.036	0.285883051	0.577134286
SCN10A	0.036	0.285907674	0.577159531
GLRXP3	0.036	0.286034754	0.577318185
AC093381	0.036	0.286187958	0.577435246

SNORA23	0.036	0.28618932	0.577435246
EEF2KMT	0.036	0.286213993	0.577435246
AC048346	0.036	0.286259722	0.577472107
NRSN2-AS1	0.036	0.286416073	0.577644156
PTPRU	0.036	0.286426699	0.577644156
NRBF2	0.036	0.286508499	0.577784658
MIR4469	0.036	0.286582713	0.577909851
KCNJ13	0.036	0.286605282	0.577930893
LNP1	0.036	0.286667943	0.577983834
AC010967	0.036	0.286855342	0.578239277
CPA2	0.036	0.286949854	0.578331882
SNORD114-5	0.036	0.28700186	0.578371569
AC055733	0.036	0.287006399	0.578371569
GS1-25M2	0.036	0.287006711	0.578371569
RP11-736P16	0.036	0.287025233	0.578371569
RP11-65F13	0.036	0.287313905	0.578566773
LINC00415	0.036	0.287319693	0.578566773
RNU6-937P	0.036	0.287331661	0.578566773
MIR4477B	0.036	0.28734396	0.578566773
NKAPL	0.036	0.287350251	0.578566773
RP11-219I21	0.036	0.287487273	0.578655918
TRAPPC2B	0.036	0.287509662	0.578676527
RP5-935K16	0.036	0.287689054	0.578933735
PRELID1P1	0.036	0.28773677	0.578986825
TRPM4	0.036	0.287897545	0.579187985
KRT19	0.036	0.288020303	0.579361531
RNU5F-1	0.036	0.2881783	0.579630386
TBC1D1	0.036	0.288231431	0.57966381
ZNF93	0.036	0.288352299	0.579686587
RNU1-43P	0.036	0.288646436	0.580078137
GPR4	0.036	0.288684801	0.580078137
MAGI3	0.036	0.288731754	0.580078137
RP11-430G17	0.036	0.288783964	0.58013809
ZEB1	0.036	0.288851024	0.580219395
RP11-382F24	0.036	0.288872035	0.580219395
HOXC-AS1	0.036	0.28900339	0.580432
OR7E83P	0.036	0.289090423	0.580484404
RP11-333B11	0.036	0.289136085	0.580527142
YIPF7	0.036	0.289164517	0.580533647
MIR3141	0.036	0.289188082	0.580533647
AC013477	0.036	0.289241109	0.580556212
RN7SL582P	0.036	0.289346041	0.580581558
ANP32AP1	0.036	0.289378209	0.580617529
AP000908	0.036	0.289401554	0.580619564
CTD-3203P2	0.036	0.28953274	0.580742013
AC091814	0.036	0.289543769	0.580742013
TBKBP1	0.036	0.289561614	0.580742013
RSPO2	0.036	0.289621103	0.580742013
C17orf102	0.036	0.289634005	0.580743433
RP11-111I12	0.036	0.289700522	0.5808279
NRM	0.036	0.289729671	0.580843941
RPS27	0.036	0.28974511	0.580843941

CTC-551A13	0.036	0.289897861	0.580945993
RNA5SP377	0.036	0.289972246	0.580945993
CRHR2	0.036	0.28998926	0.580945993
SUMF1	0.036	0.290024862	0.58096459
RP1-166H4	0.036	0.290216084	0.581274293
C10orf71-AS1	0.036	0.290302548	0.581325238
MIR4274	0.036	0.290403994	0.581476628
RN7SL734P	0.036	0.290414776	0.581476628
SEC63P2	0.036	0.290519115	0.581587738
CTD-2383M3	0.036	0.290581709	0.581615258
CTSLP1	0.036	0.290790183	0.581880538
MTND6P16	0.036	0.290808409	0.581880538
RP13-884E18	0.036	0.290882156	0.581947594
SUMO2P2	0.036	0.290942177	0.582018778
MIR548AS	0.036	0.291016359	0.582067774
FAM78B	0.036	0.291099716	0.582187208
CYP8B1	0.036	0.291152531	0.582243937
RP3-417G15	0.036	0.291230637	0.58232678
FTH1P23	0.036	0.291327105	0.58238068
RP11-7F17	0.036	0.291376346	0.582382369
RN7SKP107	0.036	0.291376486	0.582382369
KREMEN2	0.036	0.291380718	0.582382369
CSMD2-AS1	0.036	0.291468883	0.582433343
AC093117	0.036	0.291542917	0.582510991
RP11-540O11	0.036	0.291603629	0.582547236
C1orf140	0.036	0.29167921	0.582547236
AL390065	0.036	0.291688037	0.582547236
AC099754	0.036	0.291706736	0.582547236
OTOR	0.036	0.291779723	0.582602217
RP11-252C24	0.036	0.291791335	0.582602217
RP11-95L3	0.036	0.291803072	0.582602217
PIAS3	0.036	0.291803507	0.582602217
TMSB4XP1	0.036	0.291893215	0.582697551
RNA5SP198	0.036	0.291963685	0.582813799
CHCHD5	0.036	0.292065563	0.582968298
HIST3H2A	0.036	0.292279179	0.58332134
GS1-519E5	0.036	0.292358965	0.583382791
NOP10	0.036	0.292388156	0.583402798
GLIPR1	0.036	0.292585636	0.58363394
RBFOX2	0.036	0.292593908	0.58363394
MAGEC1	0.036	0.292714389	0.583676292
LEF1-AS1	0.036	0.292753362	0.583698691
HELT	0.036	0.292755611	0.583698691
DHCR24	0.036	0.292820073	0.583764822
RP1-261D10	0.036	0.292934202	0.583868284
RAB42	0.036	0.293007963	0.583943855
TXNP6	0.036	0.293039526	0.583957887
RNA5SP401	0.036	0.293157296	0.584097134
NDUFA3	0.036	0.293216769	0.584097134
COX6B2	0.036	0.293319875	0.584199523
MIR320A	0.036	0.293369352	0.584248468
RN7SL372P	0.036	0.293481047	0.584446473

NAPA-AS1	0.036	0.293622871	0.584664571
PINK1	0.036	0.293625886	0.584664571
FRMD7	0.036	0.293793273	0.584872606
RNU6-219P	0.036	0.293944251	0.585060436
POU4F2	0.036	0.293975694	0.585064576
RP3-413H6	0.036	0.294028724	0.585145664
RPS27P26	0.036	0.29406271	0.585164397
FAM159B	0.036	0.294178608	0.585282795
PLAGL1	0.036	0.294228578	0.585308844
RNU6-994P	0.036	0.294304756	0.585415524
CABYRP1	0.036	0.294341355	0.585429772
HSPD1P3	0.036	0.294402647	0.585498366
RP11-168E17	0.036	0.294488925	0.585587812
RPSAP34	0.036	0.294515931	0.585587812
CASP1P1	0.036	0.294534629	0.585587812
CCDC71	0.036	0.294544208	0.585587812
RP11-775B15	0.036	0.294545985	0.585587812
ATP6V1F	0.036	0.294647454	0.585740641
RPTN	0.036	0.294784499	0.585864884
SEZ6L2	0.036	0.295374383	0.586524733
TIMM8A	0.036	0.295392798	0.586536846
NARFL	0.036	0.295472524	0.586608212
RP11-956J14	0.036	0.295492246	0.586608212
RNU6-1308P	0.036	0.295508295	0.586608212
VPS13A-AS1	0.036	0.295514957	0.586608212
RP11-449D8	0.036	0.295596139	0.586696003
AC005062	0.036	0.295803197	0.586963721
LINC01234	0.036	0.295817293	0.586963721
RP11-443O13	0.036	0.295945882	0.587135048
COMMD7	0.036	0.295952949	0.587135048
UBA1	0.036	0.296056777	0.587267643
CORT	0.036	0.296133385	0.587335846
NUP93	0.036	0.296174023	0.58735344
HIST1H1A	0.036	0.296325701	0.587513429
YBX1P1	0.036	0.296500405	0.587658126
RP11-736I10	0.036	0.296628458	0.587797332
RP11-542C16	0.036	0.296632349	0.587797332
RNY4P14	0.036	0.296668467	0.587842546
HMCN2	0.036	0.296762745	0.587900372
RPS27P22	0.036	0.296805462	0.587900372
RNF183	0.036	0.29682013	0.587900372
RP1-167F1	0.036	0.296838515	0.587912336
PELP1	0.036	0.296891569	0.587923411
ASS1P9	0.036	0.296918172	0.587923411
CLCA4	0.036	0.296949177	0.587950166
TLN1	0.036	0.29701334	0.588001395
DNAJC5G	0.036	0.297027726	0.588001395
KLHL7	0.036	0.29707158	0.588031591
RSPO4	0.035	0.297307139	0.588360789
PYCARD	0.035	0.29735985	0.588406563
DEFB129	0.035	0.297426638	0.5884666
RP11-307N16	0.035	0.297446644	0.588479384

RNF151	0.035	0.297748825	0.588743103
RN7SKP116	0.035	0.297755388	0.588743103
TMEM51	0.035	0.297775865	0.588743103
RNU6-428P	0.035	0.297778662	0.588743103
RPS14P8	0.035	0.297837317	0.588788723
TSKS	0.035	0.297866106	0.588812246
DAB2	0.035	0.297938218	0.588890418
CDH4	0.035	0.297958263	0.588902723
ATP6V0C	0.035	0.297969173	0.588902723
RP11-136I14	0.035	0.298031596	0.58892834
SRPRB	0.035	0.298083371	0.588951432
AC124997	0.035	0.298210324	0.589030684
AP005118	0.035	0.298237961	0.589042853
RP11-50D9	0.035	0.298283923	0.589063858
UQCC2	0.035	0.298364169	0.589160847
AC144835	0.035	0.298391431	0.589160847
CCDC86	0.035	0.298398202	0.589160847
AC108868	0.035	0.298401355	0.589160847
RTN2	0.035	0.298518861	0.589304392
SLC22A18AS	0.035	0.298568404	0.589361162
CHST12	0.035	0.298590255	0.589361162
TMEM81	0.035	0.298751125	0.589438153
PPP2R4	0.035	0.298755955	0.589438153
VSX1	0.035	0.298767657	0.589438153
CTD-2643K12	0.035	0.29877226	0.589438153
GSTT2B	0.035	0.298819497	0.589450697
RNA5SP338	0.035	0.298978092	0.5896
AC005040	0.035	0.299013582	0.589602126
NXPH2	0.035	0.299039621	0.589616679
CLN6	0.035	0.299051164	0.589616679
AL049791	0.035	0.299102351	0.589674132
RNA5SP431	0.035	0.299168154	0.589679376
NPFFR2	0.035	0.29927778	0.589805081
C17orf82	0.035	0.299280597	0.589805081
RP11-179B2	0.035	0.299292611	0.589805081
C10orf35	0.035	0.299373038	0.589865947
RNU7-163P	0.035	0.299550753	0.59013777
NLGN4Y	0.035	0.299560558	0.59013777
ZHX3	0.035	0.299620007	0.590181648
PCMTD1P1	0.035	0.299801505	0.590453303
IMPDH2	0.035	0.299872463	0.590459144
MIR374A	0.035	0.2998979	0.590484818
PCDHA12	0.035	0.300063522	0.590713238
TUBB4B	0.035	0.300108998	0.59072951
TMEM108	0.035	0.30012149	0.590729687
RWDD1	0.035	0.300164326	0.590789584
ZNF580	0.035	0.300231647	0.59087325
FGF3	0.035	0.300406289	0.591083377
RNU4-41P	0.035	0.30041264	0.591083377
AC012005	0.035	0.300538251	0.591207934
PPP1R1B	0.035	0.300639613	0.591260802
LINC00871	0.035	0.300817348	0.59139801

AC231645	0.035	0.300926458	0.591531817
CTC-367J11	0.035	0.301004206	0.591633734
ZMYM4-AS1	0.035	0.301015572	0.591633734
RP11-496N12	0.035	0.301107912	0.591766384
MIR8058	0.035	0.301122818	0.591771259
ATP2B3	0.035	0.301346906	0.591998938
CDC37L1	0.035	0.301350542	0.591998938
RP1-43O17	0.035	0.301386155	0.592020062
AL161626	0.035	0.301426496	0.592027911
TMEM123	0.035	0.301452304	0.592027911
TARS	0.035	0.301481712	0.592061252
GYPC	0.035	0.301631593	0.592209081
HIST1H3D	0.035	0.301664661	0.592249591
KB-1433G4	0.035	0.301714716	0.592267878
FSHR	0.035	0.30177667	0.592267878
OR4F8P	0.035	0.301817781	0.592267878
RP11-359E8	0.035	0.301830725	0.592267878
GET4	0.035	0.301861628	0.592267878
RP1-102K2	0.035	0.30202036	0.592532694
AC011530	0.035	0.302133067	0.592680571
RP11-328L11	0.035	0.302201518	0.592790432
DIRC3-AS1	0.035	0.302233391	0.592828537
GADD45G	0.035	0.302343824	0.592922758
CHID1	0.035	0.302422744	0.592974345
RP11-410K21	0.035	0.30243223	0.592974345
UXT	0.035	0.302536538	0.59309882
EIF3KP2	0.035	0.302557766	0.59309882
RP11-20D14	0.035	0.302577547	0.59309882
RP11-35J1	0.035	0.302582886	0.59309882
THRSP	0.035	0.302847502	0.593488274
OACYLP	0.035	0.302868803	0.593488274
PSAPL1	0.035	0.302966746	0.593509264
GRXCR1	0.035	0.303014337	0.593578079
ATG10-IT1	0.035	0.303045783	0.593615263
MTND3P8	0.035	0.303165462	0.593727599
LYPD3	0.035	0.303327209	0.59394639
RNU2-17P	0.035	0.303330863	0.59394639
AC069213	0.035	0.30338319	0.593952476
RP11-137F15	0.035	0.303404518	0.593952476
PMM1	0.035	0.303468453	0.593993318
AC139712	0.035	0.303662025	0.594235816
HIST2H2AB	0.035	0.303917309	0.594539951
BRWD1-AS2	0.035	0.304049037	0.594675516
RP11-152P23	0.035	0.304155009	0.594809502
RNA5SP484	0.035	0.304179992	0.594814231
RP11-52M17	0.035	0.304206511	0.594831626
PRH1	0.035	0.304216279	0.594831626
NAMA	0.035	0.304270559	0.594888913
RP11-347E10	0.035	0.304316332	0.594905141
YEATS2	0.035	0.304391204	0.595027084
RP11-628O18	0.035	0.304449414	0.595116447
EMC8	0.035	0.30451733	0.595144549

RPS3P7	0.035	0.304556549	0.595144549
CYCSP55	0.035	0.304576864	0.595144549
RP11-58O9	0.035	0.304688919	0.595218177
CTD-2503I6	0.035	0.304906892	0.595380221
KRTAP8-3P	0.035	0.304924446	0.595385233
HOXB5	0.035	0.304968319	0.595422076
SLC19A1	0.035	0.305098985	0.595522366
ODF3L2	0.035	0.305196925	0.595551029
TBCAP2	0.035	0.305256188	0.595610938
AC112504	0.035	0.305364078	0.595662513
PTGES2	0.035	0.305436775	0.595726168
LTBP4	0.035	0.3056566	0.59598407
KLF7P1	0.035	0.305674015	0.595984602
CTC-548K16	0.035	0.305693506	0.595984602
WFDC5	0.035	0.305757553	0.596012378
TMEM248	0.035	0.305970523	0.596254423
PPBPP2	0.035	0.306014418	0.596265402
RP11-151F5	0.035	0.306018211	0.596265402
BAALC-AS2	0.035	0.306121844	0.596265402
LINC01310	0.035	0.306122655	0.596265402
STARD13-IT1	0.035	0.306134407	0.596265402
ASPHD1	0.035	0.306157532	0.596270515
ST20-AS1	0.035	0.306166575	0.596270515
RPS10P28	0.035	0.306179704	0.596271703
SPRY2	0.035	0.30621799	0.596297499
AC092570	0.035	0.306250093	0.59632222
CTC-575D19	0.035	0.306369688	0.596470961
CCDC134	0.035	0.306434569	0.596548506
AC004945	0.035	0.306467366	0.596587968
AC134878	0.035	0.306516459	0.596659148
RP11-91A18	0.035	0.306618592	0.596760398
LINC00687	0.035	0.306685985	0.596778812
GALNT12	0.035	0.306703235	0.596778812
RP11-184J23	0.035	0.306801741	0.596946096
AL591704	0.035	0.306876751	0.596954092
RN7SKP227	0.035	0.306913724	0.596954092
HAND2-AS1	0.035	0.306993671	0.597002474
PEAK1	0.035	0.307021492	0.597007822
AC023271	0.035	0.307193131	0.597268422
RP11-646J21	0.035	0.307335088	0.597441857
INTS10	0.035	0.307354472	0.597441857
GUCY1A2	0.035	0.307386591	0.597449445
MIR7845	0.035	0.307461359	0.597548173
RAB38	0.035	0.307539967	0.597649992
COX6A1P3	0.035	0.307608325	0.597685284
MATN2	0.035	0.307648552	0.597739061
MPDU1	0.035	0.307663585	0.597743884
AC092569	0.035	0.307699256	0.597779717
EPHA1	0.035	0.307707131	0.597779717
RP11-223I10	0.035	0.30783299	0.597869899
SMAGP	0.035	0.307841425	0.597869899
CCDC101	0.035	0.307872541	0.597899366

IKBKG	0.035	0.307990541	0.598037568
TET1P1	0.035	0.308091046	0.59810826
KB-1562D12	0.035	0.308120762	0.598119729
CTD-3010D24	0.035	0.30817909	0.59817654
CTB-54I1	0.035	0.308218748	0.59817654
RP11-295G24	0.035	0.308250152	0.59817654
DAAM2	0.035	0.308250246	0.59817654
SUMO2P7	0.035	0.308303566	0.598201088
RP11-532F12	0.035	0.308317207	0.598201088
MIR583	0.035	0.308320704	0.598201088
SNRPEP8	0.035	0.308383353	0.598263705
AL359538	0.035	0.308484063	0.59838596
AC020594	0.035	0.30849943	0.59838939
RP11-769N22	0.035	0.308514057	0.59838939
MIR450B	0.035	0.308523524	0.59838939
RNA5SP508	0.035	0.308634893	0.598474
CDH13	0.035	0.308729043	0.598582474
VGF	0.035	0.308940986	0.598833272
MIR3678	0.035	0.309009831	0.598926494
RP11-323I14__A	0.035	0.309094055	0.59900808
HSPE1P28	0.035	0.309123727	0.599025431
LINC00331	0.035	0.309242099	0.599124332
PRSS35	0.035	0.309258956	0.599132619
CRP	0.035	0.309359027	0.59925336
LINC00278	0.035	0.309414597	0.599263511
BCRP1	0.035	0.309559041	0.59944576
SFTPD	0.035	0.309657643	0.599612318
C8orf34	0.035	0.309710958	0.599647555
CBX1P3	0.035	0.30976814	0.599683561
GLRA2	0.035	0.30988787	0.599787631
RARS2	0.035	0.309973762	0.599837266
RN7SL78P	0.035	0.310048943	0.599955348
ABCB8	0.035	0.310164729	0.60004016
BRAT1	0.035	0.310291127	0.600165846
ZMYND15	0.035	0.310369068	0.60019506
SLC3A2	0.035	0.310383928	0.60019506
NRSN2	0.035	0.310387056	0.60019506
RP11-343H5	0.035	0.310423035	0.600240261
MITF	0.035	0.310567498	0.600446465
RPF2	0.035	0.310767837	0.600563616
IL22RA1	0.035	0.310792827	0.600563616
DCAF4	0.035	0.310804628	0.600563616
RP1-10C16	0.035	0.310883712	0.600667689
LINC01479	0.035	0.311025826	0.600892963
MIR6124	0.035	0.311050925	0.600892963
PARK7	0.035	0.311078949	0.600892963
VPS9D1	0.034	0.311155454	0.600933142
FAM45B	0.034	0.311198946	0.600984194
RP11-408N14	0.034	0.31146108	0.601256565
HYI	0.034	0.311470218	0.601256565
OPN1MW	0.034	0.311861369	0.601726682
RNU6-708P	0.034	0.311931806	0.601776759

PRYP2	0.034	0.311983143	0.601807728
RNU6-436P	0.034	0.312107728	0.601886306
RN7SKP229	0.034	0.31211991	0.601886306
CSN1S1	0.034	0.312121023	0.601886306
EXOSC3P2	0.034	0.312167523	0.601892558
CPE	0.034	0.312177046	0.601892558
OTP	0.034	0.312181538	0.601892558
FOLH1B	0.034	0.312200091	0.601892558
SLC12A8	0.034	0.312320998	0.602031433
DLEU2_6	0.034	0.312434865	0.602150183
MIR124-2	0.034	0.31252073	0.602291297
RNU4-45P	0.034	0.312731256	0.602575109
AIM1L	0.034	0.312788491	0.602612251
BNIP3P16	0.034	0.312890731	0.602687328
RP11-467N20	0.034	0.312923458	0.602705057
RP11-9D8	0.034	0.312925245	0.602705057
HES2	0.034	0.313474191	0.603468636
HIST1H2AJ	0.034	0.313479517	0.603468636
TUSC8	0.034	0.313486416	0.603468636
MED14P1	0.034	0.313560421	0.603516803
RP11-413E6	0.034	0.313632702	0.603603859
CYMP	0.034	0.313726828	0.603671642
ALDH1L1-AS1	0.034	0.313730781	0.603671642
AEN	0.034	0.313757412	0.603671642
IL1R2	0.034	0.313760518	0.603671642
AP000358	0.034	0.313769322	0.603671642
CTA-520D8	0.034	0.313824467	0.603693255
IRX3	0.034	0.31385956	0.603712508
ADRA1B	0.034	0.313934127	0.603811237
RP11-807H17	0.034	0.314067954	0.60398026
HSPB9	0.034	0.314069228	0.60398026
DCAF12L2	0.034	0.314173442	0.604156277
SSX4	0.034	0.314226479	0.604188378
RP11-58O15	0.034	0.314261749	0.604204115
AC007879	0.034	0.31439468	0.604313319
NAMPTP3	0.034	0.314555266	0.604548794
PLCB3	0.034	0.314622813	0.60461206
TELO2	0.034	0.314627021	0.60461206
RNY3P13	0.034	0.314631848	0.60461206
AL589988	0.034	0.314845013	0.604866777
PLVAP	0.034	0.314846447	0.604866777
TTC29	0.034	0.314945908	0.605006606
GSTM4	0.034	0.314974886	0.605013467
CRELD1	0.034	0.315020954	0.605065788
RPL7P59	0.034	0.315027534	0.605065788
DNAJC15	0.034	0.315069132	0.605096882
EPB41L4A-AS2	0.034	0.315085456	0.605103831
AL049871	0.034	0.315156455	0.605206222
RP5-933K21	0.034	0.315330373	0.605354495
AC009313	0.034	0.315559442	0.605672184
SNAP47	0.034	0.315618654	0.605723591
RP11-90E5	0.034	0.31562438	0.605723591

MIR548B	0.034	0.315681206	0.605735015
NOX5	0.034	0.315765733	0.605821881
FBLN5	0.034	0.315777357	0.605821881
RNU5E-5P	0.034	0.315915524	0.606062542
RP11-1038A11	0.034	0.315977681	0.606157369
JAKMIP2-AS1	0.034	0.316104264	0.606305283
RPL7P61	0.034	0.316252772	0.606489664
FBXL22	0.034	0.316302611	0.606557736
RP11-554A11	0.034	0.316367943	0.606557736
ORAI3	0.034	0.316388141	0.606557736
MIR126	0.034	0.316390154	0.606557736
LPHN1	0.034	0.316439888	0.606628666
CTD-2218K11	0.034	0.316460707	0.606644157
RN7SL456P	0.034	0.31650186	0.606698628
RP11-104H15	0.034	0.316551385	0.606758775
ATG9A	0.034	0.316823152	0.607192317
EPPIN-WFDC6	0.034	0.316900166	0.607279862
RP1-90L14	0.034	0.316907084	0.607279862
RP11-184I16	0.034	0.317123044	0.607586285
RNU6-528P	0.034	0.317127954	0.607586285
IL17B	0.034	0.317156879	0.607603154
SNORD114-16	0.034	0.317165096	0.607603154
MIR1290	0.034	0.317199192	0.607644033
RNU6-916P	0.034	0.31722358	0.607649135
LOH12CR2	0.034	0.317267812	0.607702154
SERPINF2	0.034	0.31739531	0.607897472
GSTA7P	0.034	0.317579149	0.608176201
RP5-1068B5	0.034	0.317639226	0.608217884
RP3-486I3	0.034	0.317671052	0.60825437
STMN2	0.034	0.31781846	0.608487691
KCNIP3	0.034	0.317850822	0.608525188
CALML5	0.034	0.317872491	0.608542212
ACPP	0.034	0.317900343	0.608571069
AC073370	0.034	0.31799856	0.608661232
CTDNEP1	0.034	0.318095562	0.60875319
PIK3R3	0.034	0.318101125	0.60875319
RP11-492I21	0.034	0.31810525	0.60875319
AL121819	0.034	0.318122576	0.60875319
ARL2BPP5	0.034	0.318123294	0.60875319
PDGFA	0.034	0.318202873	0.60877044
RN7SKP69	0.034	0.318206685	0.60877044
RNU7-14P	0.034	0.318250791	0.608777028
ATOH7	0.034	0.318280241	0.60878446
FOXO6	0.034	0.318314713	0.608825945
AC013463	0.034	0.318362142	0.608867756
FLRT2	0.034	0.318443017	0.608899503
SLC46A2	0.034	0.3184525	0.608899503
HYMAI	0.034	0.318452975	0.608899503
RPL35P9	0.034	0.318533059	0.608901224
VWC2L-IT1	0.034	0.318663923	0.609053601
RP13-942N8	0.034	0.318775863	0.609128268
LINC00165	0.034	0.318827712	0.609147658

CTD-2375G15	0.034	0.318828245	0.609147658
KCND1	0.034	0.318862095	0.609187894
ORAI1	0.034	0.318929234	0.609267284
RP11-856B14	0.034	0.319097731	0.609477203
NLRP10	0.034	0.319103104	0.609477203
TTYH1	0.034	0.319141062	0.609525257
GAR1	0.034	0.319378215	0.609733686
LINC01138	0.034	0.319426297	0.609779462
RP5-1030M6	0.034	0.319576772	0.609990499
FAM83A	0.034	0.319611608	0.610006272
SLC17A7	0.034	0.319635547	0.610006272
MT1P1	0.034	0.319720264	0.610117679
DNAJB8	0.034	0.319755932	0.610125831
OR8G1	0.034	0.319762968	0.610125831
BATF3	0.034	0.319781348	0.610136458
AC093159	0.034	0.319938526	0.610195297
ZNF734P	0.034	0.319940306	0.610195297
MCL1	0.034	0.320000726	0.610261655
RP11-116N8	0.034	0.320101159	0.61039261
NR1D1	0.034	0.320307488	0.610675502
LINC01020	0.034	0.320340775	0.610714519
MIR548AG1	0.034	0.320458181	0.610913893
SMG9	0.034	0.320475982	0.610923375
SOX17	0.034	0.32050021	0.610945106
AC025038	0.034	0.320656055	0.611094597
CASKP1	0.034	0.320657762	0.611094597
SNORD114-27	0.034	0.320686452	0.611104456
RPL7P1	0.034	0.320699666	0.611105185
ALDOAP1	0.034	0.320914025	0.611317986
TRAF7	0.034	0.320946635	0.611331662
PKMYT1	0.034	0.321067901	0.611399804
LINC00113	0.034	0.321093271	0.611399804
ADAM7	0.034	0.321117797	0.611399804
FAM25E	0.034	0.321133143	0.611399804
TRIM60P3Y	0.034	0.321136698	0.611399804
KRTAP24-1	0.034	0.321221242	0.611456495
DUXAP4	0.034	0.321272784	0.611512224
RP11-501H19	0.034	0.321317681	0.611548801
CTD-2381F24	0.034	0.321365485	0.611568573
TBC1D10A	0.034	0.3213932	0.611594771
RNA5SP328	0.034	0.321415612	0.611612983
RNU6-290P	0.034	0.321487076	0.611675655
RP11-575M22	0.034	0.321617748	0.61182651
TUBA1C	0.034	0.32165733	0.611877367
TCEB1P7	0.034	0.321780451	0.61196934
SULT4A1	0.034	0.322028533	0.612308656
RP11-691G17	0.034	0.322038329	0.612308656
RNF150	0.034	0.322290482	0.612567994
TMEM125	0.034	0.322306515	0.612574022
NDUFS8	0.034	0.322332704	0.61258243
RPRM	0.034	0.322336664	0.61258243
SNORA57	0.034	0.32236925	0.612591976

RPL24P2	0.034	0.322380274	0.612591976
RN7SKP19	0.034	0.322475232	0.612683225
CLIC3	0.034	0.322477833	0.612683225
STEAP2	0.034	0.322491199	0.612683225
ITGA2	0.034	0.322503496	0.612683225
CDKL1	0.034	0.322518466	0.612683225
RPL30P11	0.034	0.322531207	0.612683225
RNF148	0.034	0.322565933	0.612700315
RP11-89K22	0.034	0.322604089	0.612714337
RNA5SP515	0.034	0.32261191	0.612714337
ZCCHC16	0.034	0.322673273	0.612806443
C2orf61	0.034	0.322749688	0.612863079
SPRR2G	0.034	0.32276973	0.612863079
NDUFB11P1	0.034	0.322818985	0.612885538
FGFBP2	0.034	0.322923822	0.613010176
AL139377	0.034	0.322930054	0.613010176
AC019084	0.034	0.322935002	0.613010176
RP1-45I4	0.034	0.322964826	0.613024861
MIR4771-1	0.034	0.322990477	0.613024861
SELENBP1	0.034	0.322996959	0.613024861
GNPTG	0.034	0.322998832	0.613024861
EIF4EBP2P1	0.034	0.323075714	0.613130659
HCRTR2	0.034	0.323210557	0.613288813
DLC1	0.034	0.323235709	0.613312103
MIR1343	0.034	0.323293555	0.613348555
RP5-1186P10	0.034	0.323324962	0.613359274
RP1-27K12	0.034	0.323436384	0.61343339
FAM9A	0.034	0.32344131	0.61343339
CYP4A27P	0.034	0.323581013	0.613644041
RP5-1051H14	0.034	0.323632705	0.613649767
TWSG1	0.034	0.32375536	0.613809025
EXD3	0.034	0.323789129	0.613814355
AC128653	0.034	0.3238102	0.613814355
TMEM141	0.034	0.323835499	0.613814355
LINC01332	0.034	0.323896075	0.613880313
TMEM178A	0.034	0.323921817	0.61389206
DUSP9	0.034	0.324070394	0.613990798
RASL10A	0.034	0.324504719	0.614593639
ZNF114	0.034	0.324532902	0.614604017
PEG3	0.034	0.324559745	0.614604017
MTHFD2P6	0.034	0.324562633	0.614604017
RP4-706L14	0.034	0.324595687	0.614604017
TRMT112P3	0.034	0.324683199	0.614687233
ATP6V1E1P1	0.034	0.324705519	0.614705055
ATPIF1	0.034	0.324758963	0.614757357
AC005901	0.034	0.324849001	0.614903203
LYARP1	0.034	0.32486183	0.614903203
FANK1	0.034	0.324932739	0.614975738
PCOLCE-AS1	0.034	0.32495526	0.614975738
RP4-777G9	0.034	0.324987366	0.614975738
GPX6	0.034	0.325043204	0.615050961
ADH6	0.034	0.325066863	0.615062272

RPS7	0.034	0.32514178	0.61513973
C4orf19	0.034	0.325203596	0.615187915
AC105443	0.034	0.325215419	0.615187915
PRG4	0.034	0.325218916	0.615187915
H2AFB2	0.034	0.325287198	0.615292639
RP11-403D15	0.033	0.325480002	0.615535103
RNU1-138P	0.033	0.325559917	0.615637345
COL4A2-AS2	0.033	0.325697863	0.615751514
CYP4F36P	0.033	0.325849739	0.615988413
LDHAP4	0.033	0.325930096	0.61606829
USP5	0.033	0.325969666	0.616085505
PIGPP4	0.033	0.326001356	0.616105177
NGB	0.033	0.326039533	0.61612843
CTB-108O6	0.033	0.326086433	0.616132504
RP11-672A2	0.033	0.326088877	0.616132504
PRB2	0.033	0.326093435	0.616132504
RN7SKP166	0.033	0.326230242	0.616266884
CIART	0.033	0.326286065	0.616319726
RP11-744D14	0.033	0.326445099	0.616444817
RP11-676J15	0.033	0.326476074	0.61644662
SORBS1	0.033	0.326486509	0.61644662
NKX2-4	0.033	0.326522679	0.616478857
RP11-804L24	0.033	0.32657269	0.616548837
RP11-816J8	0.033	0.32672376	0.616760702
OR2W5	0.033	0.326806805	0.616807649
NFIC	0.033	0.326921347	0.616904247
FAM183A	0.033	0.326942283	0.616904247
CLEC5A	0.033	0.327025732	0.616957181
RP11-12O16	0.033	0.327039636	0.616957181
RP11-491H19	0.033	0.327073968	0.616957181
EXOC3L2	0.033	0.327151994	0.617055484
WIF1	0.033	0.32721008	0.617116163
TVP23C	0.033	0.327374108	0.617278856
RP11-803A13	0.033	0.327404084	0.617310939
SLC6A1-AS1	0.033	0.327434549	0.617331671
RBMY2FP	0.033	0.327441003	0.617331671
RNU1-33P	0.033	0.327551129	0.617417091
NDUFA5P6	0.033	0.327645355	0.617465381
RN7SKP257	0.033	0.327649962	0.617465381
PER1	0.033	0.327680315	0.617465381
RP11-832N8	0.033	0.327687799	0.617465381
RPS4XP12	0.033	0.327693429	0.617465381
CCDC103	0.033	0.327722795	0.617478407
ZKSCAN7P1	0.033	0.327778132	0.617478407
VPS28	0.033	0.327799716	0.617494644
CTF1	0.033	0.327824572	0.617507012
CTD-2126E3	0.033	0.327843989	0.617507012
SRP68P1	0.033	0.327845179	0.617507012
VIM-AS1	0.033	0.328150518	0.617862214
AL392167	0.033	0.328405494	0.618049096
OR2T33	0.033	0.328589176	0.61832148
PPIAP6	0.033	0.328626627	0.618367523

LINC00662	0.033	0.328716149	0.618462671
RNU6-960P	0.033	0.329063093	0.618968718
AIMP2	0.033	0.329088983	0.618980709
GOLGA2P7	0.033	0.329095461	0.618980709
SCAP	0.033	0.329180541	0.619018503
GALNT3	0.033	0.329219011	0.619039229
CTC-431G16	0.033	0.329279964	0.619039229
FAM183DP	0.033	0.329367831	0.619132262
RP11-589M2	0.033	0.32937152	0.619132262
RP11-94M14	0.033	0.329412303	0.619160967
LL0XNC01-250H12	0.033	0.329436491	0.619181996
KB-1732A1	0.033	0.329512509	0.619300432
SMARCA4	0.033	0.329638578	0.619401927
GNAT1	0.033	0.329677275	0.619438993
AC005915	0.033	0.329793317	0.619583698
WDYHV1	0.033	0.329858799	0.619682274
RPL6P21	0.033	0.329970984	0.619772591
RP11-324L17	0.033	0.330068494	0.619827228
MRPS33P2	0.033	0.330078033	0.619827228
PDIA6	0.033	0.330079115	0.619827228
RPL29P22	0.033	0.330111611	0.619863811
BX088702	0.033	0.330287541	0.620023046
PLIN1	0.033	0.330427419	0.620261181
FEZF1	0.033	0.330505682	0.62029284
DLEU1-AS1	0.033	0.330588547	0.620368035
RN7SL266P	0.033	0.330617043	0.620385303
RN7SL290P	0.033	0.330664809	0.620413365
LPPR2	0.033	0.330707755	0.620424102
RP11-380M21	0.033	0.330709612	0.620424102
MET	0.033	0.330933058	0.620730781
LURAP1L	0.033	0.330972476	0.620770532
CACNA2D1	0.033	0.331023588	0.6208175
CTXN3	0.033	0.331067946	0.620876243
RP11-432L12	0.033	0.331128895	0.620941645
MED19	0.033	0.33116118	0.620962467
SPA17P1	0.033	0.33120728	0.620990833
LINC00313	0.033	0.331340836	0.621167881
RP11-1223D19	0.033	0.331527089	0.621394752
EPHA3	0.033	0.331669449	0.621616027
GPR1-AS	0.033	0.331697351	0.621616027
PWRN1	0.033	0.331845912	0.621796572
ARHGAP10	0.033	0.331868214	0.621813896
AC073626	0.033	0.332033505	0.622025714
PCDHGB6	0.033	0.332050437	0.622027894
RFC2	0.033	0.332158603	0.622113247
SUSD2	0.033	0.33231258	0.62230375
BRD9	0.033	0.332427889	0.622387315
RP5-1050K3	0.033	0.332500946	0.622387315
PARD3-AS1	0.033	0.332508617	0.622387315
SMYD2	0.033	0.332530408	0.622387315
MARCO	0.033	0.332568669	0.622416221
RNU7-35P	0.033	0.332659939	0.622513651

RP4-736H5	0.033	0.332766176	0.622597449
SH3D19	0.033	0.333268816	0.623297457
FGF14-AS2	0.033	0.333275097	0.623297457
FGF14-IT1	0.033	0.333545376	0.623543607
RN7SKP189	0.033	0.333562483	0.623543607
HNRNPMP1	0.033	0.333651658	0.623658851
MTRNR2L11	0.033	0.333747195	0.623709937
TMED11P	0.033	0.333795808	0.62373246
RP11-100E5	0.033	0.333811673	0.623734813
MIR4449	0.033	0.333951291	0.623862927
OR2T12	0.033	0.334092299	0.624017175
RRP7A	0.033	0.334163357	0.624100945
RP4-640H8	0.033	0.334262829	0.624237767
FOS	0.033	0.334287835	0.624259987
C11orf31	0.033	0.334344749	0.62434179
MIR3173	0.033	0.334408256	0.624410903
IGFBP4	0.033	0.334466533	0.624410903
PLK2	0.033	0.33460313	0.624477779
PIGCP2	0.033	0.33461691	0.624477779
RNU6-1270P	0.033	0.334632403	0.624477779
C17orf97	0.033	0.334675959	0.624477779
RN7SL414P	0.033	0.334683602	0.624477779
RN7SL519P	0.033	0.334692922	0.624477779
AL161891	0.033	0.334798475	0.62457685
RP11-459F1	0.033	0.334880133	0.624680249
RP1-17K7	0.033	0.334941906	0.624771008
GPAA1P1	0.033	0.334989201	0.624834756
RPS3AP33	0.033	0.335057409	0.624893496
CTD-2514C3	0.033	0.335073251	0.624893496
AP000477	0.033	0.335093029	0.624893496
LCE1B	0.033	0.335113626	0.624893496
PELI2	0.033	0.335137268	0.624893496
C2orf74	0.033	0.335174815	0.624893496
KLHL13	0.033	0.335175637	0.624893496
LINC01460	0.033	0.335335106	0.625040913
RN7SKP191	0.033	0.335335953	0.625040913
PPFIA4	0.033	0.335404817	0.625120341
RNU6-1134P	0.033	0.335470381	0.625218072
OR5AM1P	0.033	0.335486277	0.625223231
CSPG4P12	0.033	0.335594003	0.62529615
TMCC1P1	0.033	0.335745187	0.625363161
BST1	0.033	0.335773744	0.62536649
C4A-AS1	0.033	0.335865169	0.625464336
PFKM	0.033	0.33593017	0.625525028
RP11-27K13	0.033	0.335932915	0.625525028
CTD-2306M5	0.033	0.335937161	0.625525028
STXBP6	0.033	0.336094467	0.625744544
AC007204	0.033	0.336132529	0.625790945
RP11-728C8	0.033	0.336157968	0.625813843
MIR193A	0.033	0.336258638	0.625878933
SUCLG1	0.033	0.336357152	0.625968707
RN7SL668P	0.033	0.336359442	0.625968707

RGPD4	0.033	0.336385637	0.625992996
VENTX	0.033	0.336521722	0.626172842
UNC5C	0.033	0.336567888	0.626227331
AC092174	0.033	0.336702566	0.626357904
MRPL24	0.033	0.336814174	0.626398016
RNU6-659P	0.033	0.336835809	0.626398016
RUVBL1	0.033	0.336840019	0.626398016
AKR1A1	0.033	0.336910961	0.626422331
QRFPR	0.033	0.336994323	0.626479402
AC010930	0.033	0.337072665	0.626492264
RNU6-1331P	0.033	0.337074858	0.626492264
C17orf59	0.033	0.337178594	0.626611709
AC092066	0.033	0.337195101	0.626617937
ACAP3	0.033	0.337238575	0.626653364
RPL7P2	0.033	0.33724048	0.626653364
CTB-66B24	0.033	0.337311145	0.626735766
RP13-213K19	0.033	0.337528994	0.627042689
RP11-527N22	0.033	0.337644318	0.627152488
SPATA8	0.033	0.337770661	0.627226711
MIRLET7A1	0.033	0.337781121	0.627226711
HEY1	0.033	0.337788357	0.627226711
RP11-1151B14	0.033	0.337799256	0.627226711
CERS5	0.033	0.337862458	0.627319608
QRSL1P2	0.033	0.337958853	0.627449666
AP000261	0.033	0.33797213	0.627449857
LINC00330	0.033	0.338091574	0.627490122
GNRH2	0.033	0.338094855	0.627490122
AC018495	0.033	0.338143428	0.627523264
OR4K12P	0.033	0.338212515	0.627587217
CTBP2	0.033	0.338213544	0.627587217
ADI1	0.033	0.338314725	0.627669955
RP11-350J20	0.033	0.338354706	0.627695229
RP11-67L3	0.033	0.338549417	0.627999375
THAP3	0.033	0.338584451	0.628005017
RNY4P30	0.033	0.338587625	0.628005017
NANOS1	0.033	0.338676598	0.628121118
ZBTB45P2	0.033	0.338769337	0.628219725
RBMY2AP	0.033	0.338849104	0.628318717
RBPMS-AS1	0.033	0.338988746	0.628489784
CCNE1	0.033	0.339042307	0.628489784
RNY1P7	0.033	0.339060124	0.628489784
PACS2	0.033	0.339166568	0.628613696
ATAD3B	0.033	0.339226776	0.628700819
GSTM3	0.033	0.339275113	0.628728864
DDX6P2	0.033	0.339306981	0.628728864
RP1-153G14	0.033	0.339317769	0.628728864
RP5-1051D14	0.033	0.339330297	0.628728864
AC016575	0.033	0.339350446	0.628728864
TCTEX1D1	0.033	0.339361026	0.628728864
TMEFF2	0.033	0.339413829	0.628734332
C11orf86	0.033	0.339461107	0.628753195
MIR449C	0.033	0.339465062	0.628753195

DNAJC4	0.033	0.339536762	0.628819006
RN7SL50P	0.033	0.339572823	0.628841981
RPL36AP6	0.033	0.339588086	0.628841981
RP3-495O10	0.033	0.339659419	0.628939743
ABCA4	0.033	0.339705092	0.628999861
SLC16A8	0.033	0.339789968	0.629094798
RPL41	0.033	0.339878177	0.629150907
RP11-3G20	0.033	0.339892348	0.629150907
RNA5SP60	0.033	0.339986593	0.629227538
MTUS2-AS1	0.032	0.340125874	0.629313835
RNU2-26P	0.032	0.340191353	0.629313835
HNRNPH3P1	0.032	0.340207942	0.629313835
PDPR	0.032	0.340237522	0.629313835
GTDC1	0.032	0.340275434	0.629321921
AL157407	0.032	0.340374567	0.629448838
PAGE4	0.032	0.340398996	0.629448838
RNU6-976P	0.032	0.340449802	0.629463538
RP11-18A15	0.032	0.340470956	0.629463538
RP3-429G5	0.032	0.340556516	0.629548408
AGBL1	0.032	0.340778667	0.62964137
CLEC18C	0.032	0.34079574	0.629648487
PAK1IP1	0.032	0.340845281	0.62971559
F8A1	0.032	0.340987145	0.629807169
RNA5SP397	0.032	0.340990625	0.629807169
RP11-419C19	0.032	0.34100064	0.629807169
SPEM1	0.032	0.341088444	0.629902757
RNA5SP26	0.032	0.341092072	0.629902757
AC072052	0.032	0.341121885	0.629933388
CITED1	0.032	0.341149005	0.629959043
TADA3	0.032	0.341181161	0.629993996
RP11-1072A3	0.032	0.341239934	0.629997988
IRF5P1	0.032	0.341262689	0.629997988
KANK3	0.032	0.341326442	0.630017999
RP11-107D24	0.032	0.341414211	0.630099627
ZNF212	0.032	0.341436815	0.630099627
SNORD115-17	0.032	0.341732177	0.630412438
AL137251	0.032	0.341742202	0.630412438
AC080003	0.032	0.34181283	0.630450179
LRRC75B	0.032	0.341832738	0.630450179
C19orf83	0.032	0.341865129	0.630474754
RN7SL509P	0.032	0.341967384	0.630590081
IL17RE	0.032	0.342082595	0.630729269
RNU7-165P	0.032	0.342123528	0.630731488
RP11-435F13	0.032	0.342310294	0.630867262
RN7SKP3	0.032	0.342314412	0.630867262
TMPRSS4	0.032	0.342379259	0.630958712
RNF6P1	0.032	0.342395103	0.630963497
RNU2-28P	0.032	0.342608297	0.631169056
NRBF2P1	0.032	0.342617499	0.631169056
PFDN5	0.032	0.342634636	0.631169056
HAS3	0.032	0.342652427	0.631169056
GNAI2	0.032	0.342685229	0.631205066

GMPPA	0.032	0.342793658	0.631282715
ST20	0.032	0.342809786	0.631288004
RNU6-1143P	0.032	0.342867841	0.631321684
WFDC8	0.032	0.342921132	0.63138922
NDUFB4	0.032	0.342931034	0.63138922
AC006386	0.032	0.342955421	0.631409711
MIR557	0.032	0.342976938	0.631424918
ABC7-481722F1	0.032	0.343124909	0.631624088
RP11-420B22	0.032	0.343160345	0.631641564
BACH2	0.032	0.343164986	0.631641564
EGFLAM-AS1	0.032	0.343206387	0.631676415
FIBP	0.032	0.343303012	0.631749323
AC106869	0.032	0.343333714	0.631764277
DNAJC8P1	0.032	0.343350492	0.631770741
AC019050	0.032	0.343481359	0.631828043
RP4-730D4	0.032	0.343558406	0.63185964
TSPAN9-IT1	0.032	0.343571275	0.63185964
MLIP-AS1	0.032	0.343614269	0.631865513
RTKN	0.032	0.343645449	0.631866111
RNU6-958P	0.032	0.343661344	0.631866111
RP11-372H2	0.032	0.343667662	0.631866111
AMH	0.032	0.343778369	0.631923288
SYN3	0.032	0.343814846	0.631965949
RMST_4	0.032	0.343858398	0.631997219
HNRNPA1P32	0.032	0.343881995	0.632016199
DSG1-AS1	0.032	0.343939738	0.632021562
MTND5P3	0.032	0.343986529	0.632061977
DIAPH2	0.032	0.344012906	0.632067304
SNORD115-5	0.032	0.344055688	0.632086413
RNU6-955P	0.032	0.344072374	0.632086413
RP11-470B22	0.032	0.34409273	0.632086413
RANGAP1	0.032	0.344154495	0.632126731
TTTY8B	0.032	0.344205971	0.63215795
RP1-317E23	0.032	0.344234225	0.63215795
RNU2-71P	0.032	0.344285608	0.632207674
LINC00605	0.032	0.344291483	0.632207674
CTD-2231H16	0.032	0.344545587	0.6324602
HOXC6	0.032	0.344593992	0.632483034
RPL37A	0.032	0.34460264	0.632483034
CHST7	0.032	0.344785965	0.632679213
ST8SIA5	0.032	0.344800673	0.632679213
DHRSX	0.032	0.344895097	0.632779336
RP1-99E18	0.032	0.344925603	0.632786826
CTC-339O9	0.032	0.345015182	0.632853381
RCVRN	0.032	0.345123166	0.632976697
RP11-387M24	0.032	0.345170366	0.632991761
AGMO	0.032	0.345230352	0.633028643
RP11-655B23	0.032	0.345278014	0.633091664
RP5-1065P14	0.032	0.345355625	0.633172698
HTR5A-AS1	0.032	0.345475524	0.633283149
TPT1-AS1	0.032	0.345557056	0.633383848
GNAI2P1	0.032	0.345601477	0.633438192

C20orf173	0.032	0.345648836	0.633438192
RP11-26E5	0.032	0.34576617	0.6335598
ARHGAP23P1	0.032	0.345794558	0.633564772
FMOD	0.032	0.34581899	0.633571358
RNU6-644P	0.032	0.345843253	0.633581709
RNU5A-8P	0.032	0.345891155	0.633581709
PSORS1C2	0.032	0.345915374	0.633601704
SLC22A12	0.032	0.345964194	0.633666757
OR2E1P	0.032	0.346021568	0.633697493
BICD2	0.032	0.346024264	0.633697493
RNY3	0.032	0.346027663	0.633697493
RP11-113O24	0.032	0.346191211	0.633887541
CHMP4B	0.032	0.346212547	0.633902237
LINC01483	0.032	0.346309863	0.633982928
RP11-3B7	0.032	0.346348228	0.634016262
OR1R1P	0.032	0.346419388	0.63408025
TBC1D24	0.032	0.346454361	0.63408025
AC068570	0.032	0.346456219	0.63408025
TMEM183AP1	0.032	0.346483154	0.634092131
CTXN1	0.032	0.346600789	0.63424735
RP11-299H22	0.032	0.346750926	0.634400214
IFNWP4	0.032	0.346786103	0.63441583
KCNK5	0.032	0.346957924	0.634559543
AVPR1A	0.032	0.346986786	0.634587959
LINC01331	0.032	0.347095225	0.634694967
RPL41P5	0.032	0.347098602	0.634694967
GPR176	0.032	0.347112055	0.634695199
RP11-113I22	0.032	0.347226771	0.634855445
RPL32P14	0.032	0.347239682	0.634855445
RNU6-1222P	0.032	0.3472633	0.634863422
RP11-25P11	0.032	0.347270705	0.634863422
SNHG6	0.032	0.34734137	0.634912787
REEP2	0.032	0.347364362	0.634912787
RP11-192C21	0.032	0.347432715	0.634988983
CTD-2272D18	0.032	0.347506538	0.635026431
MIR183	0.032	0.347664902	0.635267073
SEMA3E	0.032	0.347729074	0.635335581
SRC	0.032	0.347775593	0.635348993
ACKR2	0.032	0.347776434	0.635348993
RP11-774D14	0.032	0.347886829	0.635496469
SNORD96B	0.032	0.34789719	0.635496469
GGT1	0.032	0.347917603	0.635509384
RP11-725G5	0.032	0.347987731	0.635545951
PPP6R2P1	0.032	0.348161405	0.635686534
CYCSP11	0.032	0.348290384	0.635802766
RP4-569D19	0.032	0.348291813	0.635802766
OVOL3	0.032	0.348348562	0.635857617
MIR3943	0.032	0.348450037	0.635862053
RP11-484O2	0.032	0.348455614	0.635862053
GLB1L	0.032	0.348458478	0.635862053
RN7SL468P	0.032	0.348471149	0.635862053
RAB44	0.032	0.348555319	0.63596588

CHCHD2P9	0.032	0.348578464	0.63596588
CLDN11	0.032	0.348597855	0.63596588
MIR6737	0.032	0.348605222	0.63596588
RP1-180M12	0.032	0.348608168	0.63596588
LINC00934	0.032	0.348737096	0.636074857
AC140912	0.032	0.348748036	0.636074857
DENND5B-AS1	0.032	0.348871322	0.636202262
LONRF3	0.032	0.348923675	0.63623952
PMPCA	0.032	0.349057826	0.636330269
MYRF	0.032	0.349061763	0.636330269
RP11-280G9	0.032	0.349246751	0.636479893
RP11-45I20	0.032	0.349338228	0.636590596
ITGA7	0.032	0.349395738	0.636653089
MIR4803	0.032	0.349491984	0.63673102
KIAA1161	0.032	0.349502416	0.63673102
NR1H4	0.032	0.349541242	0.63673102
ACPT	0.032	0.34954898	0.63673102
RNA5SP97	0.032	0.349627141	0.636821901
RP11-282M16	0.032	0.349638984	0.636821901
RFPL1S	0.032	0.349810461	0.63701242
SLC52A3	0.032	0.349866516	0.637017071
AQP7P4	0.032	0.349974909	0.637092649
HTR7	0.032	0.350138964	0.637220804
NPY4R	0.032	0.35037677	0.637526221
SH3PXD2A-AS1	0.032	0.350380105	0.637526221
RP11-204I15	0.032	0.350400346	0.637526221
SCAMP3	0.032	0.350475574	0.637553629
CTC-575I10	0.032	0.350495867	0.637553629
TCOF1	0.032	0.350634361	0.637732479
LINC00556	0.032	0.350647765	0.637732504
RNU1-27P	0.032	0.350706236	0.637746261
RP11-1029M24	0.032	0.350752148	0.637751872
EFEMP1	0.032	0.350857246	0.637845564
RPL37P23	0.032	0.351011937	0.638078073
EXOSC5	0.032	0.3510726	0.638098581
FREM2	0.032	0.351174728	0.63822784
ATF4P2	0.032	0.351205803	0.638229935
AC067959	0.032	0.351221763	0.638229935
CRIP2	0.032	0.351287472	0.638229935
LINC00337	0.032	0.351296486	0.638229935
AC007568	0.032	0.351347363	0.638244733
RNU6-163P	0.032	0.351369921	0.638244733
GRM2	0.032	0.351371636	0.638244733
NAB2	0.032	0.351693442	0.638588059
OR5D18	0.032	0.351695858	0.638588059
CTD-2378E21	0.032	0.351721544	0.638588059
RP11-123O10	0.032	0.351843242	0.638735967
RPL35P6	0.032	0.351884519	0.638762206
TMEM261P1	0.032	0.35208769	0.638910635
OSCAR	0.032	0.35211385	0.638910635
LRRC3-AS1	0.032	0.352163435	0.63897071
PDP1	0.032	0.352197008	0.63897071

ARHGEF7-AS1	0.032	0.352219494	0.63897071
SERPINB8	0.032	0.352339311	0.639027584
KCNK9	0.032	0.352441636	0.639149577
RNU1-4	0.032	0.352499855	0.639149577
HOXB7	0.032	0.352520707	0.639149577
RP5-1099C19	0.032	0.352531515	0.639149577
GOLGA8DP	0.032	0.352538018	0.639149577
WDR34	0.032	0.35255354	0.639149577
AC097108	0.032	0.352574995	0.639149577
F12	0.032	0.352602511	0.639149577
MOGAT3	0.032	0.352616859	0.639149577
RNU2-48P	0.032	0.352621292	0.639149577
CYP46A1	0.032	0.352801266	0.639378459
AMMECR1	0.032	0.352872632	0.639421771
AC007405	0.032	0.352894723	0.639421771
LSM10	0.032	0.352897272	0.639421771
CTD-2377O17	0.032	0.352986604	0.639500788
ILF2	0.032	0.352989611	0.639500788
GCG	0.032	0.353015476	0.639523319
RP11-822E23	0.032	0.3530816	0.639594453
GFRA3	0.032	0.353157646	0.639634895
HSFY3P	0.032	0.353209269	0.639702224
ASNSP6	0.032	0.353221683	0.639702224
VPS51	0.032	0.353375563	0.639921769
STAP2	0.032	0.353383217	0.639921769
TEX26-AS1	0.032	0.353672055	0.640225891
LINC01035	0.032	0.35368021	0.640225891
RN7SL503P	0.032	0.35373222	0.640254765
MIR4308	0.032	0.353769697	0.640254765
TMEM129	0.032	0.353772276	0.640254765
AL645730	0.032	0.353781409	0.640254765
KMT2E-AS1	0.032	0.353927465	0.640395995
EDARADD	0.032	0.353947044	0.640407091
PDE6A	0.032	0.353962373	0.640407449
SIM1	0.032	0.354001569	0.64040843
AC002553	0.032	0.354049077	0.640470046
SGSH	0.032	0.354076453	0.640495241
ABO	0.032	0.354097456	0.640502634
CAMP	0.032	0.354279284	0.640701621
RPS3AP5	0.032	0.354705474	0.641316438
MESDC2	0.032	0.354741561	0.641334769
CH507-216K13	0.032	0.354742544	0.641334769
AC022431	0.032	0.354779678	0.641353212
RPL26L1	0.032	0.354856657	0.641443677
RP11-115H18	0.032	0.354899457	0.641448006
RBCK1	0.032	0.354948183	0.641487387
XXbac-BPG55C20	0.032	0.35496308	0.641489969
ZBTB8OSP2	0.032	0.355023807	0.641548154
RP11-175G14	0.032	0.355065607	0.641548154
RNF212	0.031	0.355112218	0.641548154
RP11-161I6	0.031	0.355116509	0.641548154
RP11-484K9	0.031	0.355163852	0.641590323

RNU6-383P	0.031	0.355334812	0.641753872
GS1-600G8	0.031	0.355492444	0.641870566
AC036178	0.031	0.355848348	0.642163886
RP11-1079J22	0.031	0.355870381	0.642179314
RP11-744J10	0.031	0.35591048	0.64222734
RP11-7G12	0.031	0.356097648	0.642325836
AC009158	0.031	0.356113525	0.642325836
ITGA1	0.031	0.356244208	0.642440094
VN1R31P	0.031	0.356273835	0.642469197
ADCK4	0.031	0.356344792	0.64252417
SLC9A1	0.031	0.356487579	0.642708632
RP11-195C7	0.031	0.356643352	0.642845737
RP11-542B15	0.031	0.356654704	0.642845737
AL359253	0.031	0.356672701	0.642845737
RP11-459O1	0.031	0.356685104	0.642845737
RNA5SP27	0.031	0.356851378	0.643047948
PPIA	0.031	0.356857966	0.643047948
OR13C2	0.031	0.356899187	0.643047948
MAGEH1	0.031	0.356905316	0.643047948
RP3-412A9	0.031	0.356919293	0.643048805
C19orf24	0.031	0.357002309	0.643174041
KRT17P7	0.031	0.357052863	0.643226049
RP11-447L10	0.031	0.357071693	0.643226049
RP11-434D2	0.031	0.357191204	0.643319675
OR9A3P	0.031	0.357221096	0.643327885
ERRFI1	0.031	0.357257137	0.643327885
SEC61G	0.031	0.357263702	0.643327885
RNU6-753P	0.031	0.35728142	0.643327885
GOLGA2P9	0.031	0.357331971	0.643354242
UHRF2P1	0.031	0.357405466	0.643405531
PTENP1-AS	0.031	0.357418218	0.643405531
RNU6-376P	0.031	0.357428004	0.643405531
AC005701	0.031	0.357560488	0.643554167
KCNQ4	0.031	0.357564624	0.643554167
SARS2	0.031	0.357604036	0.643576459
CTA-313A17	0.031	0.357686093	0.643592462
AC005276	0.031	0.357750807	0.643600836
CA5AP1	0.031	0.357752715	0.643600836
AL136137	0.031	0.35779317	0.643624992
CTC-340A15	0.031	0.357838082	0.643681471
LYPD2	0.031	0.357851913	0.643682041
RP11-78F17	0.031	0.357917155	0.643750767
ILF2P1	0.031	0.357961603	0.643805663
ZNF503-AS1	0.031	0.358071085	0.643881732
CTD-2083E4	0.031	0.358109201	0.643925961
AP000290	0.031	0.358153509	0.643981319
PCDHB19P	0.031	0.35821126	0.644057642
FMO10P	0.031	0.358236525	0.644057642
RP11-414H23	0.031	0.358272203	0.644097471
THPO	0.031	0.358293442	0.644098463
RP11-775J23	0.031	0.358432024	0.644223159
RP11-177F15	0.031	0.358475958	0.644244913

LHX8	0.031	0.35852035	0.644276071
SMARCA5-AS1	0.031	0.358575988	0.644283312
RP11-314C16	0.031	0.358652582	0.644283312
SDAD1P1	0.031	0.358673184	0.644283312
ROBO4	0.031	0.35879137	0.644416482
LIX1L	0.031	0.358823139	0.644416482
RP4-734C18	0.031	0.35885643	0.644418037
GSTZ1	0.031	0.358954525	0.644432757
CAGE1	0.031	0.358972874	0.644432757
SC22CB-1D7	0.031	0.359156081	0.644664456
AHR	0.031	0.359181302	0.644685429
RP11-393K10	0.031	0.359228885	0.644746537
MIR365B	0.031	0.359251366	0.644747369
CRYBB2P1	0.031	0.359414185	0.6449188
C14orf178	0.031	0.359419651	0.6449188
AC002429	0.031	0.359476866	0.644980696
OR2F2	0.031	0.359492418	0.644980696
RNA5SP376	0.031	0.359508316	0.644980696
NREP-AS1	0.031	0.359568943	0.645040869
LINC00315	0.031	0.359765949	0.645199867
RP11-547I7	0.031	0.359827441	0.645285848
SDR9C7	0.031	0.359880533	0.645330829
TFAMP2	0.031	0.360083269	0.645598775
CRISP3	0.031	0.360220191	0.64574392
RP11-42F12	0.031	0.360232016	0.64574392
MIR6771	0.031	0.360354392	0.645890359
TRAPPC6A	0.031	0.360405496	0.645935396
LPCAT2BP	0.031	0.360577367	0.646193164
KRTAP1-3	0.031	0.360712472	0.646386218
SNHG3	0.031	0.360899064	0.646568427
MIR148A	0.031	0.361031587	0.646666093
MPP4	0.031	0.361050939	0.646676435
TTTY7B	0.031	0.361170653	0.646710891
CDC20B	0.031	0.361194268	0.646714264
FAU	0.031	0.361234093	0.646761255
OR7G2	0.031	0.361324864	0.646850822
AP000318	0.031	0.361441455	0.64700031
RNA5SP192	0.031	0.361467427	0.647008762
AC092597	0.031	0.361494707	0.647033274
SF3B4	0.031	0.361552925	0.647064526
TCEA2	0.031	0.361583696	0.64709528
RP11-864I4	0.031	0.36181928	0.647322296
RP11-472K22	0.031	0.36189577	0.647348724
NOTCH2	0.031	0.361966892	0.647416148
MIR5088	0.031	0.362009743	0.647468476
RP11-1127D7	0.031	0.362059912	0.647485854
PPP1R2P2	0.031	0.362079109	0.647485854
ZBED3	0.031	0.362180752	0.647579783
AP001059	0.031	0.362336577	0.647785442
LINC01524	0.031	0.362388335	0.647805635
MIR501	0.031	0.362415122	0.647805635
RP11-254F7	0.031	0.362601795	0.647995124

RP11-16N2	0.031	0.362603524	0.647995124
LTBP2	0.031	0.362658553	0.648069147
AC067969	0.031	0.362713136	0.648142368
FILIP1L	0.031	0.36279514	0.648232933
RP11-187A9	0.031	0.362942228	0.648342666
RP11-305O4	0.031	0.362969141	0.648342666
CARM1P1	0.031	0.362969929	0.648342666
OR52T1P	0.031	0.363082394	0.648411337
LINC01021	0.031	0.363093526	0.648411337
RP11-69E9	0.031	0.363095631	0.648411337
CTB-164L20	0.031	0.363110272	0.648411337
SOCS5	0.031	0.363122329	0.648411337
MAGI2-AS3	0.031	0.363214321	0.648485235
AC006466	0.031	0.363225883	0.648485235
RN7SKP211	0.031	0.36325598	0.648485235
RP11-403A3	0.031	0.363426409	0.648686789
FBXL6	0.031	0.363470765	0.648717337
RP11-96K14	0.031	0.363512797	0.648768042
AC005019	0.031	0.363591313	0.648883856
LIPF	0.031	0.363620956	0.64890322
RNU4-71P	0.031	0.363651566	0.64890322
CTB-1H10	0.031	0.363725694	0.64897777
LINC00577	0.031	0.363837196	0.649030829
RP11-116D17	0.031	0.363860872	0.649048754
RPL6P9	0.031	0.363894552	0.649053876
RP11-321P16	0.031	0.3639029	0.649053876
RP11-451G4	0.031	0.363941795	0.649063515
AC068492	0.031	0.364006282	0.649069016
RNU6-1172P	0.031	0.364022141	0.649069016
MIR4722	0.031	0.364077187	0.649142864
OR9P1P	0.031	0.364140056	0.649206351
MMP24	0.031	0.364201485	0.649243176
AC104088	0.031	0.364201607	0.649243176
MPRIP	0.031	0.364326587	0.649269897
AC108022	0.031	0.364376884	0.64929624
CTB-43I4	0.031	0.364419029	0.649314818
GS1-115M3	0.031	0.364594064	0.649514316
CENPBD1P1	0.031	0.364661866	0.649597386
RNU6-71P	0.031	0.364819767	0.649761455
SENP3	0.031	0.364909113	0.649809165
RP11-605F14	0.031	0.364935486	0.649809165
RP11-348F1	0.031	0.36496066	0.649809165
PSMC3	0.031	0.364963203	0.649809165
CTD-2034I21	0.031	0.365094195	0.649934261
RNU6-706P	0.031	0.36518627	0.649952409
AC087269	0.031	0.365383694	0.650228675
RP11-589M4	0.031	0.365541928	0.650439566
LHFPL2	0.031	0.365603698	0.650500871
LDLR	0.031	0.365681021	0.65058984
AC003045	0.031	0.365889156	0.650838575
RNU1-38P	0.031	0.365999184	0.650912738
AP000251	0.031	0.366085428	0.650993192

AC012501	0.031	0.366175473	0.651093818
RPL23AP67	0.031	0.366236143	0.651093818
MIR1258	0.031	0.366237709	0.651093818
PABPC4	0.031	0.366259388	0.651108053
FXYD6	0.031	0.366273883	0.651109519
RN7SL14P	0.031	0.366306749	0.65114364
C11orf1	0.031	0.366368692	0.651229443
NUTM2E	0.031	0.366390899	0.651244611
INHBA-AS1	0.031	0.366509341	0.651357901
FAM8A4P	0.031	0.366601105	0.651385916
ACOT1	0.031	0.366658143	0.651385916
RP11-155N3	0.031	0.366697259	0.651385916
LINC00551	0.031	0.36670999	0.651385916
PCDHA11	0.031	0.366777406	0.651413238
XYLT1	0.031	0.366900718	0.651445738
ATP6V1G1P3	0.031	0.366994759	0.651556576
OR2M3	0.031	0.367081375	0.651578313
RP11-1082L8	0.031	0.367084832	0.651578313
RP11-69E11	0.031	0.367217434	0.651649348
CDK4	0.031	0.367269123	0.651662564
C9orf50	0.031	0.367376786	0.651763711
TMEM134	0.031	0.367422185	0.651763711
SNAP25-AS1	0.031	0.36774657	0.652041712
ITCH-IT1	0.031	0.367756618	0.652041712
PSMG1	0.031	0.3678819	0.652222233
AC026471	0.031	0.367913211	0.652222233
CTD-2540M10	0.031	0.367927873	0.652223947
RP11-272J7	0.031	0.367977469	0.652279679
RNA5SP166	0.031	0.368004088	0.652279679
RPL7AP34	0.031	0.368101599	0.652316155
PPP1R42	0.031	0.368130662	0.652316155
RNA5SP179	0.031	0.368255209	0.65238884
RN7SL649P	0.031	0.36835067	0.652408723
MIR4497	0.031	0.368359168	0.652408723
AC074183	0.031	0.36836489	0.652408723
CITF22-49E9	0.031	0.368395015	0.652408723
RNU6-364P	0.031	0.368401961	0.652408723
KBTBD13	0.031	0.368421669	0.65241177
BAI1	0.031	0.368687986	0.652648307
RP11-115J16	0.031	0.368786022	0.652694136
TPRX1P1	0.031	0.368809805	0.652694136
SPANXA2-OT1	0.031	0.368868955	0.652727357
RP11-165D7	0.031	0.368869692	0.652727357
RN7SL346P	0.031	0.368905883	0.652742894
MIR378G	0.031	0.369100137	0.653038085
POF1B	0.031	0.369182647	0.653084447
RAB9BP1	0.031	0.369210816	0.653085049
PEG10	0.031	0.369230845	0.653085049
ANG	0.031	0.369236381	0.653085049
MGAT2	0.031	0.369276882	0.653128258
GRIK2	0.031	0.369309769	0.653128258
RP11-137D19	0.031	0.369317812	0.653128258

UPK1A	0.031	0.369366495	0.653128258
AP000344	0.031	0.36961152	0.653311774
RAB4A	0.031	0.369618487	0.653311774
HIGD1B	0.031	0.369625193	0.653311774
LAMA5-AS1	0.031	0.369657566	0.653320502
RNA5SP382	0.031	0.369916343	0.653656575
NPM1P2	0.031	0.369979705	0.653744284
RP4-753F5	0.031	0.37003693	0.653748381
MAPT-AS1	0.031	0.370094035	0.653776514
RP11-212P7	0.031	0.37043073	0.654152924
SH2D4A	0.031	0.370471896	0.65417711
EPHB6	0.031	0.370512482	0.654184746
RP11-1057B6	0.030	0.370621835	0.654281154
AC004895	0.030	0.370626981	0.654281154
AC141930	0.030	0.37066061	0.654313527
MTRF1LP2	0.030	0.370678249	0.654313527
BRMS1	0.030	0.370686535	0.654313527
RP13-49I15	0.030	0.370725058	0.654338198
RP11-474D1	0.030	0.370741728	0.654338198
RP11-506M13	0.030	0.370793436	0.65438096
CLPB	0.030	0.370829085	0.654385289
PPM1G	0.030	0.370840187	0.654385289
MIR449B	0.030	0.370850848	0.654385289
RP11-21C17	0.030	0.370879933	0.654388121
TEKT4P2	0.030	0.370974008	0.654483349
RP11-351I24	0.030	0.371018166	0.654525088
RNA5SP222	0.030	0.371044567	0.654529511
DEFB136	0.030	0.371072688	0.654529511
RTN4RL1	0.030	0.371095583	0.654529511
NDP-AS1	0.030	0.371102	0.654529511
RP11-72J9	0.030	0.371130032	0.654529511
AMIGO3	0.030	0.371347558	0.65480082
RP11-529G21	0.030	0.371450384	0.654918492
PRR23D2	0.030	0.37162595	0.655145769
MIR3910-1	0.030	0.371639474	0.655145769
PDIA4	0.030	0.371719627	0.655259759
AC106827	0.030	0.371747007	0.655259759
RP11-809C18	0.030	0.371821996	0.655259759
PPP1CB	0.030	0.371826721	0.655259759
DDX26B-AS1	0.030	0.372087224	0.655515371
C3orf14	0.030	0.37210303	0.655515371
CACFD1	0.030	0.372110642	0.655515371
HSF1	0.030	0.372277838	0.655712892
AL365510	0.030	0.372438352	0.65586283
MIR1976	0.030	0.37244559	0.65586283
PLP1	0.030	0.372630563	0.656104784
AC108683	0.030	0.372651868	0.656104784
RP11-542G1	0.030	0.372778463	0.656296973
RS1	0.030	0.372788587	0.656296973
LAMA2	0.030	0.372808429	0.656307646
CA13	0.030	0.372876142	0.656341668
IMMP2L	0.030	0.37291044	0.656341668

IL34	0.030	0.372975819	0.656429242
ISY1-RAB43	0.030	0.373004667	0.656434737
RP11-680F8	0.030	0.373032994	0.656460332
GALNT9	0.030	0.373233003	0.656715244
FABP4	0.030	0.373494404	0.65693249
AC006004	0.030	0.373576849	0.657032221
HAUS1P3	0.030	0.373578696	0.657032221
AC007228	0.030	0.373776179	0.657307704
AC016994	0.030	0.373871836	0.657426396
OLFM3	0.030	0.373934212	0.65748753
TSPY24P	0.030	0.374063543	0.657520736
ESRG	0.030	0.374139297	0.657605355
AC019186	0.030	0.374184196	0.657622972
AC005152	0.030	0.374217794	0.657622972
SFRP1	0.030	0.374231988	0.657622972
OR52J1P	0.030	0.374348065	0.657795111
RN7SKP104	0.030	0.374509563	0.6579596
FAM168A	0.030	0.374546144	0.6579596
RN7SL656P	0.030	0.374629344	0.658019248
GJD4	0.030	0.374667063	0.658019248
PLGRKT	0.030	0.374678732	0.658019248
OR11H1	0.030	0.374781068	0.658110147
SSXP4	0.030	0.375164833	0.658624735
OR7E110P	0.030	0.375189067	0.658624735
ARL5AP1	0.030	0.37519088	0.658624735
RP11-468H14	0.030	0.375304188	0.658624735
LINC00923	0.030	0.375351844	0.658624735
SNRPGP2	0.030	0.375375368	0.658624735
EXTL2	0.030	0.375377134	0.658624735
ARHGEF7-AS2	0.030	0.375393756	0.658624735
ISM2	0.030	0.375403769	0.658624735
GRK4	0.030	0.37542806	0.658624735
RP5-944M2	0.030	0.375431464	0.658624735
B4GALT4	0.030	0.375438363	0.658624735
ENDOU	0.030	0.375484754	0.658656209
HOXC10	0.030	0.375496655	0.658656209
RBMY2JP	0.030	0.375679364	0.65877949
AP000619	0.030	0.375684074	0.65877949
ASCL5	0.030	0.375815483	0.658913202
ZBTB45	0.030	0.375903966	0.659022686
TSTD1	0.030	0.375914278	0.659022686
MIR4477A	0.030	0.375973112	0.659068715
C15orf52	0.030	0.376004142	0.659068715
RNU6-281P	0.030	0.376027596	0.659068715
RP11-753A21	0.030	0.3760374	0.659068715
FAM74A7	0.030	0.376178129	0.659156913
STRA8	0.030	0.376181779	0.659156913
SPIN2A	0.030	0.376345276	0.659248056
TBC1D20	0.030	0.376515296	0.659445225
CAMKK1	0.030	0.376565727	0.659448275
AC005477	0.030	0.37658202	0.659448275
UBE2FP2	0.030	0.376586268	0.659448275

RP11-339D20	0.030	0.376651645	0.659538509
RP11-403P14	0.030	0.37676375	0.659607741
RHEBP3	0.030	0.376852759	0.659633588
RP11-77E14	0.030	0.376935774	0.659656924
RP11-511B7	0.030	0.376936669	0.659656924
AC005357	0.030	0.376940878	0.659656924
RN7SKP57	0.030	0.377049535	0.659779501
AC067960	0.030	0.37711136	0.659858288
PKMP5	0.030	0.377143826	0.659890852
TUB-AS1	0.030	0.377211175	0.659935959
RP11-17M15	0.030	0.37725252	0.65998036
CRTC1	0.030	0.377291983	0.65998036
ALOX15B	0.030	0.377322397	0.660009322
TEKT1	0.030	0.377402865	0.660077348
ARMC10P1	0.030	0.37765918	0.660380136
KRT8P3	0.030	0.377753976	0.660392193
RP11-85A1	0.030	0.377798783	0.660406027
GFI1B	0.030	0.377873932	0.660457967
RP11-358H18	0.030	0.377875151	0.660457967
RP11-435O11	0.030	0.377885869	0.660457967
GULP1	0.030	0.377887262	0.660457967
KRT34	0.030	0.3779117	0.660457967
FAM86DP	0.030	0.377927165	0.66046076
GEM	0.030	0.377951108	0.660478368
MIRLET7F2	0.030	0.378023698	0.660517369
SNORD114-10	0.030	0.378044467	0.660517369
LA16c-380H5	0.030	0.378068472	0.660517369
CGREF1	0.030	0.378072907	0.660517369
KCNAB3	0.030	0.3781486	0.660605339
OR7E29P	0.030	0.378252062	0.660689146
GSN-AS1	0.030	0.378286406	0.660700673
MIR4535	0.030	0.378335508	0.660737972
MIR320B1	0.030	0.378372842	0.660754715
AL121657	0.030	0.378500614	0.660808238
ZHX1-C8orf76	0.030	0.378564485	0.660871296
RP11-357K6	0.030	0.378646432	0.660946988
RP11-100N3	0.030	0.378662666	0.660946988
RP11-430L3	0.030	0.378714486	0.660946988
IMP4	0.030	0.378742342	0.660946988
RNU6-488P	0.030	0.378768469	0.660946988
LINC00395	0.030	0.378775495	0.660946988
CTC-246B18	0.030	0.378776972	0.660946988
OR5BD1P	0.030	0.378821746	0.660957003
OR51L1	0.030	0.37896747	0.661137917
AL121760	0.030	0.379019116	0.661156042
CKLF	0.030	0.379179953	0.661363936
RP11-570G20	0.030	0.379245077	0.661452692
PET117	0.030	0.379320659	0.661491661
LINC00518	0.030	0.379322627	0.661491661
ENO4	0.030	0.379358581	0.661530137
HEG1	0.030	0.379459618	0.661657876
RPL21P66	0.030	0.379757961	0.662065699

TBC1D3P6	0.030	0.379773192	0.662065699
COX8A	0.030	0.37984051	0.662065699
ILF2P2	0.030	0.379841994	0.662065699
LINC01143	0.030	0.379846415	0.662065699
AC231759	0.030	0.379937671	0.662200521
FBXW4P1	0.030	0.380011024	0.662237713
ADD3	0.030	0.380052844	0.662237713
ST7-OT3_2	0.030	0.380056342	0.662237713
EEF1GP2	0.030	0.380235052	0.662423231
RP11-521C20	0.030	0.38024046	0.662423231
CDK14	0.030	0.380357237	0.662541356
MIR129-2	0.030	0.380364773	0.662541356
AC006033	0.030	0.380495187	0.662687203
RNU6-29P	0.030	0.380639469	0.662879264
TMEM238	0.030	0.380754716	0.663035587
C2orf72	0.030	0.380778747	0.663035587
CHTF18	0.030	0.380846181	0.66308028
CTC-254B4	0.030	0.380908033	0.663139486
NR2F6	0.030	0.380980731	0.663217561
TMC1	0.030	0.381132864	0.663358147
CBR3	0.030	0.38115037	0.663358147
ZNF517	0.030	0.381158986	0.663358147
RP11-747H12	0.030	0.381190406	0.663365545
BSND	0.030	0.381191094	0.663365545
RP4-610C12	0.030	0.38128398	0.663455375
CXCL6	0.030	0.381299272	0.663455375
SLC25A43	0.030	0.381306383	0.663455375
CTB-147C22	0.030	0.381312364	0.663455375
RP11-355F22	0.030	0.381434352	0.663505823
RBX1	0.030	0.381440438	0.663505823
KB-1183D5	0.030	0.381534848	0.663551586
RP11-326G21	0.030	0.38165793	0.663717173
LTF	0.030	0.381674433	0.663721639
OR8T1P	0.030	0.382175581	0.664200301
AC062017	0.030	0.382189263	0.664200301
KARSP1	0.030	0.382257973	0.664232559
CTGLF10P	0.030	0.382367902	0.66433349
RP5-1158E12	0.030	0.38240194	0.66433349
SLC7A14	0.030	0.382468289	0.664374397
OSBPL10-AS1	0.030	0.38250958	0.664395586
LINC00677	0.030	0.382515934	0.664395586
PGAM1P12	0.030	0.382738586	0.664640575
RP11-617I14	0.030	0.382745704	0.664640575
CYP39A1	0.030	0.382747119	0.664640575
HNRNPA1P62	0.030	0.382826602	0.664754359
KCNMA1-AS1	0.030	0.382863682	0.66475813
LINC01118	0.030	0.382890396	0.66475813
AC009474	0.030	0.382908136	0.66475813
MIR499A	0.030	0.382957968	0.664763819
NRTN	0.030	0.382984697	0.664763819
RN7SL831P	0.030	0.382999542	0.664763819
LL0XNC01-37G1	0.030	0.383097722	0.664863809

CTNNB1	0.030	0.383170569	0.664963733
SERBP1	0.030	0.383248973	0.665075563
DEGS1	0.030	0.383321721	0.665129103
ZNF71	0.030	0.38337599	0.665148666
RP11-834C11	0.030	0.383393882	0.665148666
AC016716	0.030	0.383404511	0.665148666
RP11-30L3	0.030	0.38341679	0.665148666
DUSP6	0.030	0.383554334	0.665266116
DIO2	0.030	0.383706474	0.665373903
OPLAH	0.030	0.383749438	0.665373903
OTOG	0.030	0.383756183	0.665373903
AC010641	0.030	0.383792758	0.665413094
RP11-875H7	0.030	0.383857754	0.665477331
MIR569	0.030	0.383945635	0.66551227
OR8D4	0.030	0.383955624	0.66551227
LINC00561	0.030	0.383961748	0.66551227
GYG2P1	0.030	0.383990255	0.665524646
ITGB4	0.030	0.383996835	0.665524646
SH3D21	0.030	0.384041909	0.665539937
RP11-287D1	0.030	0.384047282	0.665539937
IER5	0.030	0.38404758	0.665539937
NANOGNBP1	0.030	0.384087483	0.665584869
RNASET2	0.030	0.384261649	0.665765564
RPLP0P11	0.030	0.384324553	0.665826106
PCDHA10	0.030	0.384447967	0.66594302
GSDMD	0.030	0.384608734	0.666124592
AC134873	0.030	0.384642646	0.666159101
AC068196	0.030	0.384683913	0.666182123
MIR6786	0.030	0.384698815	0.666183706
BAG3	0.030	0.38472736	0.666208914
MIR6888	0.030	0.384842889	0.666287844
EN2	0.030	0.384901748	0.666356577
LINC01006	0.030	0.384910572	0.666356577
HMGB1P42	0.030	0.385199121	0.666662253
RP11-515O17	0.030	0.385367152	0.666859821
ESAM	0.030	0.385473691	0.666921924
AC068580	0.030	0.385475187	0.666921924
RNU6-985P	0.030	0.38568703	0.667076946
RNU6-284P	0.030	0.38568703	0.667076946
STK32A	0.030	0.385762507	0.667128211
ZNF513	0.030	0.385824137	0.667178187
RP11-1C8	0.030	0.385857666	0.667178187
CFL1P4	0.030	0.385907932	0.667210117
RP11-124N14	0.030	0.386003421	0.667302538
PCDHGA8	0.030	0.386089438	0.667402143
CTD-2015G9	0.030	0.38611709	0.667402143
AL034395	0.030	0.386219579	0.667530842
PPP1R1A	0.030	0.386323356	0.667647093
RP11-48G14	0.030	0.386328894	0.667647093
RP11-77O7	0.029	0.386589883	0.667904246
RP11-26L20	0.029	0.386675671	0.667961584
AC078917	0.029	0.386710026	0.667989935

C8orf74	0.029	0.386737024	0.667989935
SHROOM2	0.029	0.386741422	0.667989935
LINC00535	0.029	0.386759387	0.667989935
TNFRSF4	0.029	0.386827766	0.668040605
OXER1	0.029	0.386917121	0.668055144
CLEC4GP1	0.029	0.38692436	0.668055144
LAGE3P1	0.029	0.387071713	0.668251875
RP11-357D18	0.029	0.387232092	0.668456065
RP11-109P6	0.029	0.387346108	0.668569086
RRP12	0.029	0.387426805	0.668622547
C20orf194	0.029	0.387551663	0.668813793
RP11-392O17	0.029	0.387584983	0.668847058
MIR548A1	0.029	0.387644099	0.668865873
CHEK2P5	0.029	0.387680077	0.668865873
PDE7B	0.029	0.387680148	0.668865873
RNU6-620P	0.029	0.387886279	0.669124554
HSD17B7	0.029	0.387978947	0.669235932
SERAC1	0.029	0.388000875	0.669249517
RP3-422G23	0.029	0.38806151	0.669305625
AC068522	0.029	0.388083924	0.669320045
FAM90A26	0.029	0.388179553	0.669379543
IGF2R	0.029	0.388200659	0.669379543
AC073091	0.029	0.38820275	0.669379543
AP000692	0.029	0.388228604	0.669399889
FH	0.029	0.388279088	0.669462699
MIR6858	0.029	0.388343879	0.669499653
ATP2C1	0.029	0.388429362	0.669552125
RHOQP3	0.029	0.388755432	0.669895978
LDHBP3	0.029	0.388799425	0.669943755
AC068706	0.029	0.388811291	0.669943755
NXF4	0.029	0.388947456	0.670105646
AC092625	0.029	0.389061819	0.670181462
GAS7	0.029	0.389151769	0.670287921
OR4A44P	0.029	0.389308493	0.670479842
RP11-339D23	0.029	0.389325136	0.670479842
PNP	0.029	0.389444031	0.670509841
C3orf33	0.029	0.389466912	0.670515494
FMO9P	0.029	0.389536632	0.670562806
IL22RA2	0.029	0.389586519	0.670576569
WDR24	0.029	0.38965029	0.670646054
LRP5	0.029	0.389727692	0.670673536
RN7SKP194	0.029	0.389807497	0.670762397
RP11-419M24	0.029	0.389836002	0.670787213
EIF4EBP3	0.029	0.389862038	0.670807778
RP11-482E14	0.029	0.389937916	0.670889861
RP11-138B9	0.029	0.390085847	0.67107165
ACAA1	0.029	0.390167283	0.671116921
RP11-395N6	0.029	0.390188342	0.671126767
CACNA1C-AS3	0.029	0.39022864	0.67117184
RP11-157P1	0.029	0.3904389	0.671398334
RP4-726F1	0.029	0.390542297	0.671425358
SNORA74A	0.029	0.390558259	0.671425358

FUT11	0.029	0.390565252	0.671425358
ARF1	0.029	0.390596316	0.671425358
STK25P1	0.029	0.390624151	0.671425358
AC073427	0.029	0.390699667	0.671496989
AC010096	0.029	0.390839152	0.671639772
AC009965	0.029	0.390955907	0.671779591
MROH2A	0.029	0.390974413	0.671779591
TPBGL	0.029	0.390996965	0.671779591
RARRES2P6	0.029	0.391036608	0.671785179
SLFN13	0.029	0.39117595	0.671840968
EPOR	0.029	0.39119678	0.671840968
MIR130B	0.029	0.391201473	0.671840968
LINC01179	0.029	0.391207292	0.671840968
IFRD2	0.029	0.391213081	0.671840968
MIR3922	0.029	0.39129355	0.671867536
RNU6-800P	0.029	0.391302808	0.671867536
CTD-2014B16	0.029	0.391326522	0.671867536
PIRT	0.029	0.391350686	0.671867536
MIR4304	0.029	0.391368402	0.671867536
AC002486	0.029	0.391549286	0.67203787
RN7SL708P	0.029	0.391586842	0.672051692
RP11-184D12	0.029	0.391803405	0.672278012
LINC01338	0.029	0.39187366	0.672348318
CALML4	0.029	0.391896617	0.672348318
SV2A	0.029	0.391900847	0.672348318
SCNN1A	0.029	0.392258158	0.672743223
RP11-190J1	0.029	0.392315315	0.672784269
FABP5P3	0.029	0.392438375	0.672810025
C19orf67	0.029	0.392533314	0.672948567
MIR132	0.029	0.392646742	0.673022597
AC007319	0.029	0.392692076	0.673075377
AMIGO2	0.029	0.392802665	0.673216471
RP11-764K9	0.029	0.392817797	0.673218179
RPS2P52	0.029	0.393218274	0.673591726
OTX2-AS1	0.029	0.393305547	0.673666233
ARMCX1	0.029	0.393385491	0.673718104
RP11-12M9	0.029	0.3934591	0.673783791
RP11-83C7	0.029	0.39347321	0.673783791
PABPC5-AS1	0.029	0.393615979	0.67391293
RP13-34L8	0.029	0.39370803	0.674016259
AL591668	0.029	0.393774501	0.674081595
RNU6-1320P	0.029	0.393985466	0.67432155
CPSF4	0.029	0.394031599	0.67433072
DACT3-AS1	0.029	0.394042967	0.67433072
RP11-2C24	0.029	0.394061616	0.67433072
CTD-2325M2	0.029	0.394133179	0.67438049
RP3-471C18	0.029	0.394206109	0.674432586
PRKAB2	0.029	0.394249335	0.674433859
NUDT14	0.029	0.394271476	0.674447511
MTX1	0.029	0.394315458	0.674474297
RP5-1039K5	0.029	0.394554833	0.674733257
TMEM132E	0.029	0.394676753	0.674898381

CTD-2231E14	0.029	0.394858653	0.675079336
RP11-182E7	0.029	0.39486762	0.675079336
HCG16	0.029	0.395004805	0.67522134
RP11-411H5	0.029	0.395026109	0.67522134
MRPL34	0.029	0.395149554	0.6752632
RNU6-1101P	0.029	0.395173559	0.6752632
MRPL57P7	0.029	0.395187839	0.6752632
CCDC54	0.029	0.395232226	0.675290591
RTN4R	0.029	0.3952586	0.675311426
RNU6-206P	0.029	0.395286735	0.675325252
SLC40A1	0.029	0.39543785	0.675453277
RNASEH2CP1	0.029	0.395483875	0.675478232
RNU6-1103P	0.029	0.395584597	0.675611395
SCN3B	0.029	0.395597068	0.675611395
FRG2DP	0.029	0.395755827	0.675748854
RP11-47J17	0.029	0.395836379	0.67576526
AL035697	0.029	0.395851758	0.675767292
PAM16	0.029	0.395946702	0.675813453
OR4F29	0.029	0.396009178	0.675813453
LL22NC03-80A10	0.029	0.396015734	0.675813453
RNU6-15P	0.029	0.396018637	0.675813453
MIR6854	0.029	0.396080129	0.675833111
RNU6-148P	0.029	0.396146432	0.675869843
RNA5S9	0.029	0.396153075	0.675869843
CCT5	0.029	0.396236565	0.675963854
CBLN1	0.029	0.396339359	0.675983417
RN7SKP223	0.029	0.396358441	0.675983417
LINC01282	0.029	0.396404158	0.675983417
MKKS	0.029	0.396459904	0.676030069
SHC4	0.029	0.39650099	0.67604293
RP11-508N22	0.029	0.396530208	0.676053131
AL359851	0.029	0.396562444	0.676063886
SLC39A12	0.029	0.39661409	0.676091106
MAGEA2B	0.029	0.396645245	0.676091106
NEUROD2	0.029	0.396651849	0.676091106
AC007271	0.029	0.396722683	0.676163442
AL593844	0.029	0.396759299	0.676201651
RP11-20O24	0.029	0.396837989	0.676311562
UBXN8	0.029	0.396876054	0.676352231
AACS	0.029	0.396901025	0.676370585
RP11-568A7	0.029	0.396974855	0.67646774
XKR3	0.029	0.397027677	0.67646774
RP11-282C5	0.029	0.397126763	0.676513812
IQCJ	0.029	0.397245766	0.676691731
CALR	0.029	0.397321414	0.676747979
RP11-156J23	0.029	0.397579945	0.677067259
RP11-125D12	0.029	0.397804683	0.677269361
RNU6-1334P	0.029	0.397815675	0.677269361
LRRC29	0.029	0.397838129	0.677269361
AC004108	0.029	0.397844173	0.677269361
RNU6-1050P	0.029	0.397892902	0.677309598
AC073321	0.029	0.397926951	0.677319142

RNU6-704P	0.029	0.397953298	0.677339781
NRG2	0.029	0.398011502	0.677368956
RP11-321F6	0.029	0.398167941	0.677559831
RP11-415H23	0.029	0.398251653	0.677653858
RP11-128P17	0.029	0.398337765	0.677683626
KCNS3	0.029	0.398372034	0.677704248
RNU2-65P	0.029	0.398380873	0.677704248
RP11-292I17	0.029	0.39845233	0.677798198
ZNF575	0.029	0.398490161	0.677798198
AC113331	0.029	0.398573528	0.677910914
RP11-889D3	0.029	0.398641606	0.677976943
TNNT3	0.029	0.398685088	0.678003809
RP11-92G12	0.029	0.398699847	0.6780047
RP11-293A21	0.029	0.398727526	0.678027559
SLC38A3	0.029	0.398926224	0.678195941
CTC-350I8	0.029	0.399028931	0.678322122
PWRN4	0.029	0.399076207	0.678342951
CTC-369M3	0.029	0.399095856	0.678342951
RP11-378I13	0.029	0.399112562	0.678342951
PRSS16	0.029	0.399247836	0.678465537
TMEM221	0.029	0.39925575	0.678465537
RN7SKP93	0.029	0.39945045	0.678602972
SNORA70C	0.029	0.399504272	0.678645717
CTAGE13P	0.029	0.399726486	0.678904728
ESYT2	0.029	0.399794011	0.678927023
RP11-524L6	0.029	0.399832463	0.678927023
DLG4	0.029	0.399837139	0.678927023
LINC00592	0.029	0.399915693	0.678977179
AC009081	0.029	0.399927495	0.678977179
SNORD113-5	0.029	0.400089231	0.679058117
HNRNPA1P66	0.029	0.400158288	0.67912692
MROH7-TTC4	0.029	0.40038247	0.679289538
RN7SKP47	0.029	0.400471516	0.679370845
RPS23P6	0.029	0.400484888	0.679370845
SPATA31E1	0.029	0.400528822	0.679392628
RP11-48F14	0.029	0.40056285	0.679401954
RP11-169F17	0.029	0.400670607	0.67948793
APH1A	0.029	0.400835379	0.679624747
AC073264	0.029	0.400935353	0.679651452
RP11-545H22	0.029	0.400942318	0.679651452
AC098830	0.029	0.40108197	0.679798255
LINC00839	0.029	0.401168457	0.679848068
TBC1D7	0.029	0.401211061	0.679882202
RNU7-8P	0.029	0.401254233	0.679882202
RSL24D1P11	0.029	0.40127735	0.679887454
AL353751	0.029	0.40137482	0.679966062
AC152010	0.029	0.401381052	0.679966062
RP11-647K16	0.029	0.401462451	0.679976702
AC008834	0.029	0.401479838	0.679976702
UST	0.029	0.401498941	0.679976702
DVL2	0.029	0.401620078	0.68002195
SULT1C3	0.029	0.401620482	0.68002195

RP11-879F14	0.029	0.401674358	0.680029046
MGC39584	0.029	0.40167504	0.680029046
GNAL	0.029	0.401704733	0.68003149
RP11-10E18	0.029	0.40172061	0.68003196
AGAP3	0.029	0.40195815	0.68022438
RNU6-1299P	0.029	0.40202949	0.68022438
FRG2FP	0.029	0.40204238	0.68022438
RP11-111D3	0.029	0.4020475	0.68022438
RN7SL643P	0.029	0.402074493	0.680245884
SERF2	0.029	0.402218415	0.680414285
RN7SL865P	0.029	0.40222332	0.680414285
ANKRD33	0.029	0.402249985	0.680414285
CTD-3066C23	0.029	0.402259748	0.680414285
C16orf95	0.029	0.40246312	0.680513939
HIST1H2BL	0.029	0.402463797	0.680513939
TATDN1P1	0.029	0.402476917	0.680513939
AC069240	0.029	0.402494206	0.680513939
AL022341	0.029	0.402504413	0.680513939
RPL21P38	0.029	0.402571037	0.68054636
TINCR	0.029	0.402580745	0.68054636
MIR542	0.029	0.402739004	0.680693089
VMA21	0.028	0.40312061	0.681023882
C6orf203	0.028	0.40315596	0.681059444
KLF5	0.028	0.403171945	0.681062291
HIST1H1B	0.028	0.403297292	0.681153237
FUT9	0.028	0.403350191	0.681218423
FGF23	0.028	0.403418176	0.681293796
FAM71E2	0.028	0.403423429	0.681293796
MIR6077	0.028	0.403482711	0.681339851
MIR655	0.028	0.403492772	0.681339851
BTF3L4P3	0.028	0.403535998	0.681351496
LRRC31	0.028	0.403543431	0.681351496
SLC5A3	0.028	0.403565855	0.681365201
RP11-158I13	0.028	0.403660749	0.681452945
RNU6-984P	0.028	0.403874169	0.68166527
SNORD116-3	0.028	0.403878483	0.68166527
SNORD114-18	0.028	0.403958447	0.681695169
HMGN1P15	0.028	0.404002193	0.681739387
FARP1	0.028	0.404030169	0.681748794
SNRPGP12	0.028	0.404064757	0.681748794
RNU2-3P	0.028	0.404131859	0.681748794
MIR3166	0.028	0.404307639	0.681748794
RP11-116O11	0.028	0.404312964	0.681748794
MIR375	0.028	0.404329412	0.681748794
RNU6-1307P	0.028	0.404329616	0.681748794
ARMC2	0.028	0.404375612	0.681789764
SLC37A3	0.028	0.404436591	0.681809998
EDDM3DP	0.028	0.404442557	0.681809998
RPL23P11	0.028	0.404444875	0.681809998
NT5C3B	0.028	0.404672858	0.681881053
AC027347	0.028	0.404673147	0.681881053
RPP25L	0.028	0.404757898	0.681968078

ATP5F1P7	0.028	0.404782069	0.681968078
RNU6-788P	0.028	0.404831567	0.682027345
ACOX3	0.028	0.40486298	0.68205614
LEMD2	0.028	0.404915064	0.682119757
SNORD6	0.028	0.404962112	0.682150757
AC087897	0.028	0.405001247	0.682192552
SIRPA	0.028	0.40506641	0.682205803
GP6	0.028	0.405221357	0.68234898
MRPS21P9	0.028	0.405223057	0.68234898
CENPW	0.028	0.40524184	0.682355484
ADAM30	0.028	0.405257687	0.682355484
RASA3	0.028	0.405269901	0.682355484
AC010295	0.028	0.405477329	0.682500273
RP13-30A9	0.028	0.405532961	0.682557103
RNU6-2	0.028	0.405558683	0.682567944
IFNA8	0.028	0.405568065	0.682567944
CTD-3076M17	0.028	0.405587014	0.682575714
CTD-2007A10	0.028	0.405651808	0.682660636
OTX2	0.028	0.40574842	0.68273032
MT1B	0.028	0.405922607	0.682899187
AC072061	0.028	0.405937942	0.682900865
RN7SL65P	0.028	0.40601565	0.682942806
CCDC169	0.028	0.406064202	0.682942806
TMPRSS11F	0.028	0.406076367	0.682942806
WFDC3	0.028	0.406092502	0.682942806
RNU1-95P	0.028	0.406130786	0.682942806
L1TD1	0.028	0.406139643	0.682942806
RAG1	0.028	0.406159191	0.682942806
RP11-516A11	0.028	0.406179012	0.682942806
EFNA2	0.028	0.406192304	0.682942806
FARP1-AS1	0.028	0.406350545	0.683088289
NOXA1	0.028	0.406522094	0.68313555
RP11-512N4	0.028	0.406687407	0.68331691
GPX1P2	0.028	0.406807352	0.683476361
HMGN5	0.028	0.406811008	0.683476361
RP11-238F2	0.028	0.406837366	0.683496533
POLR3H	0.028	0.406872824	0.683531993
CTAGE11P	0.028	0.406906156	0.683563878
LCMT1-AS1	0.028	0.40707783	0.683779919
KSR1P1	0.028	0.407097197	0.683788334
RP5-1056H1	0.028	0.407209122	0.683879206
CTD-2060C23	0.028	0.407217262	0.683879206
RP3-340B19	0.028	0.407223094	0.683879206
RP6-1O2	0.028	0.407282699	0.683919291
AC073900	0.028	0.407297237	0.683919291
SUCLA2-AS1	0.028	0.407332909	0.683919291
RNU6-929P	0.028	0.407343213	0.683919291
CCAR2	0.028	0.407360128	0.683919291
PABPC1L2A	0.028	0.407491289	0.684036191
RNA5SP249	0.028	0.407524991	0.684036191
RNU5E-9P	0.028	0.407532007	0.684036191
KRT16P5	0.028	0.407554845	0.684050415

FERD3L	0.028	0.407747876	0.684302058
AC006007	0.028	0.407782189	0.684335529
AC091565	0.028	0.408020887	0.68463014
RNU6-349P	0.028	0.408076767	0.68466777
YDJC	0.028	0.408115577	0.684677888
CH17-189H20	0.028	0.408165433	0.684692244
RPL5P20	0.028	0.408167262	0.684692244
COMT	0.028	0.408250599	0.684783803
DPYD-AS1	0.028	0.408281912	0.684794049
MTRNR2L10	0.028	0.408304453	0.684801782
GLDCP1	0.028	0.408339061	0.684801798
RN7SL267P	0.028	0.408368256	0.684801798
UBL5P2	0.028	0.408468781	0.684908557
ICAM1	0.028	0.408537455	0.684999591
OR1F1	0.028	0.408593155	0.685039239
RPL30P15	0.028	0.408614445	0.685039239
DGAT2L6	0.028	0.408618635	0.685039239
AQPEP	0.028	0.408646238	0.6850614
STOML3	0.028	0.408752847	0.68516777
RN7SL221P	0.028	0.408882212	0.685336376
FBXL8	0.028	0.408918669	0.68534591
AL132709	0.028	0.408925947	0.68534591
CTC-340D7	0.028	0.40893107	0.68534591
RP11-345E19	0.028	0.409053462	0.685478668
PTP4A1P3	0.028	0.409117186	0.685537214
GALNT6	0.028	0.409162695	0.685565231
MIR5190	0.028	0.409180248	0.685570522
RP11-613D13	0.028	0.409205494	0.685588703
RABL6	0.028	0.409250156	0.685616814
FLAD1	0.028	0.409282104	0.685620582
FMR1-AS1_2	0.028	0.40932554	0.685623113
CTD-2542O7	0.028	0.409384548	0.685647496
RP11-49E18	0.028	0.409490326	0.685776425
RPL7P55	0.028	0.409524366	0.685782548
RP11-81F13	0.028	0.409541943	0.685782548
PRAMEF17	0.028	0.409558502	0.685782548
GUCY1B3	0.028	0.409565977	0.685782548
DUSP21	0.028	0.409698459	0.685883136
TMEM203	0.028	0.409773604	0.685911224
MIR6814	0.028	0.409774924	0.685911224
RP11-114O8	0.028	0.409786843	0.685911224
OR7E148P	0.028	0.410008213	0.686137075
IL11RA	0.028	0.410103329	0.686223913
SLCO4A1-AS1	0.028	0.410183278	0.686285358
RP11-758N13	0.028	0.410366574	0.686471456
LMO7	0.028	0.410481681	0.686543441
OR7E41P	0.028	0.410509926	0.686566571
SNORA27	0.028	0.410560797	0.686627539
SPSB3	0.028	0.410678394	0.686755849
E2F6P3	0.028	0.410686257	0.686755849
PTGES	0.028	0.410785942	0.686816493
RP1-124C6	0.028	0.410853347	0.686816493

AC019129	0.028	0.410870705	0.686816493
RP4-609E1	0.028	0.41087567	0.686816493
RP11-33H15	0.028	0.410908291	0.686846916
SPHKAP	0.028	0.410952577	0.686896834
SLC6A10P	0.028	0.411197032	0.687233078
RNU6-1252P	0.028	0.41154848	0.687684051
FES	0.028	0.411565383	0.687684051
GOLGA8CP	0.028	0.411566568	0.687684051
RP1-232L22__A	0.028	0.411570286	0.687684051
HIST1H3PS1	0.028	0.411846301	0.687917626
ERO1L	0.028	0.411852062	0.687917626
RP11-126O22	0.028	0.411866612	0.687917626
AL592549	0.028	0.411897015	0.687920157
ITGA10	0.028	0.412009615	0.688032961
RNU6-405P	0.028	0.412022342	0.688032961
DAZ1	0.028	0.412063443	0.688077469
BVES-AS1	0.028	0.412171701	0.688209047
RP11-536G4	0.028	0.412270659	0.688209047
POMP	0.028	0.412338847	0.688209047
AC096664	0.028	0.412358989	0.688209047
CNTNAP3	0.028	0.412504974	0.688353841
ACEA_U3	0.028	0.412513156	0.688353841
SERPINA11	0.028	0.412548109	0.68835583
MFSD6L	0.028	0.412578304	0.688382094
IGHVIII-26-1	0.028	0.41265089	0.688406737
AL138498	0.028	0.412798676	0.68854026
UBAP1	0.028	0.412803212	0.68854026
EML1	0.028	0.412859955	0.688586674
TMEM51-AS1	0.028	0.41300838	0.688761865
DCLK3	0.028	0.413216727	0.688916339
RPS26P13	0.028	0.413337581	0.688924144
PPIAP16	0.028	0.413655512	0.689237722
CD2BP2	0.028	0.413858064	0.689478733
ACSM4	0.028	0.413873139	0.689479729
FTLP14	0.028	0.41395406	0.689576605
RP11-547L9	0.028	0.413960248	0.689576605
RP11-217E22	0.028	0.414201492	0.689804456
ADAMTS8	0.028	0.414205341	0.689804456
RP11-434I12	0.028	0.414247496	0.689804456
KIAA0930	0.028	0.414317192	0.689855265
OR7E101P	0.028	0.414323017	0.689855265
BNIP3P1	0.028	0.414588817	0.690165033
OR7E62P	0.028	0.414642021	0.690168672
RN7SL177P	0.028	0.4147082	0.690190108
RP11-651J20	0.028	0.414733165	0.690190108
RN7SL275P	0.028	0.414738681	0.690190108
IFRD1	0.028	0.414748796	0.690190108
RFPL4AP3	0.028	0.41482479	0.690292453
RP11-293I14	0.028	0.414868154	0.690340492
C8orf59P2	0.028	0.414990964	0.690398262
TPSD1	0.028	0.414998379	0.690398262
KRT10	0.028	0.415033343	0.690398275

NPR1	0.028	0.415096209	0.690448716
AC092289	0.028	0.4151375	0.690475073
RP11-643N23	0.028	0.415185194	0.690528294
MC3R	0.028	0.415354859	0.690739434
RN7SL292P	0.028	0.415412366	0.690741708
TCEB1P18	0.028	0.415565859	0.690959607
SOX12	0.028	0.415619685	0.690987494
FAM223A	0.028	0.415669357	0.691020632
PLA2G4A	0.028	0.415736449	0.691071253
INTS9	0.028	0.41583194	0.691147367
SFTA3	0.028	0.415897824	0.691190972
RPL17P25	0.028	0.415940111	0.691190972
RP11-793K1	0.028	0.41605496	0.691260751
RP4-796I8	0.028	0.416073062	0.691260751
CTD-3064M3	0.028	0.416227505	0.691394649
RP11-480G7	0.028	0.416335601	0.691453445
RP11-68E19	0.028	0.416563371	0.69168719
LSM1P2	0.028	0.416603425	0.691729581
PIEZO1	0.028	0.4168324	0.692037393
AP1B1	0.028	0.41692114	0.692112344
LINC00970	0.028	0.417056998	0.692193666
ALAS2	0.028	0.417069797	0.692193666
MIR3140	0.028	0.417207708	0.692282492
AL356140	0.028	0.417247869	0.692282492
MIR3193	0.028	0.417257713	0.692282492
RNU1-13P	0.028	0.417270739	0.692282492
C9orf66	0.028	0.417347437	0.692361503
FAM50B	0.028	0.417481747	0.692439602
RP11-341D18	0.028	0.41760018	0.692587798
RN7SL339P	0.028	0.417684472	0.692679354
NDUFS5P2	0.028	0.417727615	0.692726781
PFDN1	0.028	0.417810726	0.692782226
RP11-359N11	0.028	0.41782612	0.692782226
RNU6-391P	0.028	0.417829075	0.692782226
AC007304	0.028	0.41788288	0.692816548
PLD2	0.028	0.417960198	0.692919463
ZNF101P1	0.028	0.418177566	0.693135521
SNRPCP20	0.028	0.418177841	0.693135521
OR51B5	0.028	0.418299197	0.693218032
HNF1A	0.028	0.418375002	0.693317544
FAM90A14P	0.028	0.418402526	0.693339032
NMS	0.028	0.418519954	0.693365486
ABCF3	0.028	0.418538913	0.693365486
SNORD115-35	0.028	0.418549515	0.693365486
CHMP1A	0.028	0.418634468	0.693433861
CHCHD2P3	0.028	0.418705534	0.693527456
NACAP7	0.028	0.418836143	0.693668138
IGKV3D-34	0.028	0.418848727	0.693668138
JUP	0.028	0.41894574	0.693780554
ANGPTL3	0.028	0.419049573	0.693859405
TMEM237	0.028	0.419066197	0.693859405
RP1-105O18	0.028	0.419163531	0.693972311

IRAK2	0.028	0.419315669	0.694127677
TMEM107	0.028	0.419337066	0.69413897
ZNF714	0.028	0.419401573	0.694163739
PPIAL4G	0.027	0.419515543	0.694269973
FIGN	0.027	0.4195262	0.694269973
RP4-745K6	0.027	0.419601143	0.694310658
AC011357	0.027	0.419658928	0.694320573
RP5-1103B4	0.027	0.419672538	0.694320573
TGM1	0.027	0.419694606	0.694320573
MTND2P15	0.027	0.419804601	0.694406062
RP11-7D5	0.027	0.419925806	0.694485949
GAPDHP17	0.027	0.41996023	0.694502578
LINC01430	0.027	0.419969467	0.694502578
ZNF705B	0.027	0.420117867	0.694610624
KRT8P22	0.027	0.420227215	0.694732353
RP11-696L21	0.027	0.420324409	0.694807394
AC009413	0.027	0.420363254	0.694817776
PDCD6	0.027	0.420389424	0.694817776
WFDC12	0.027	0.420407663	0.694817776
KLHL40	0.027	0.420418222	0.694817776
TNS1	0.027	0.420502561	0.694823656
AK4	0.027	0.420523993	0.694823656
PHLDB2	0.027	0.420538491	0.694823656
RP4-612B18	0.027	0.420584673	0.694865632
BANF1P3	0.027	0.420593076	0.694865632
PARVB	0.027	0.420759785	0.694993453
OR7G1	0.027	0.420781033	0.694993453
RP11-118M12	0.027	0.420867004	0.695019847
NPY2R	0.027	0.420874995	0.695019847
LINC01571	0.027	0.420983015	0.695102084
RP13-192B19	0.027	0.420983255	0.695102084
SLC6A18	0.027	0.421157946	0.695244168
ST13P11	0.027	0.421202425	0.695251662
SSMEM1	0.027	0.42121728	0.695251662
AMACR	0.027	0.421220877	0.695251662
RNY3P12	0.027	0.421347053	0.695411724
CH17-408M7	0.027	0.421444224	0.695475696
MTND6P8	0.027	0.421688912	0.695737278
RP11-301L8	0.027	0.421987979	0.695967235
CD1A	0.027	0.422008581	0.695967235
RNU6-13P	0.027	0.422048957	0.695967235
RP11-81B10	0.027	0.422125799	0.696021653
RP11-275F13	0.027	0.422175075	0.696078802
CCND2-AS2	0.027	0.422284936	0.696211736
NOC2L	0.027	0.42235594	0.696284234
RARRES2P1	0.027	0.42238256	0.696284234
GUCY1A3	0.027	0.422391015	0.696284234
RN7SL516P	0.027	0.422402007	0.696284234
GOLGA6L1	0.027	0.422452944	0.696319999
MIR1237	0.027	0.422534287	0.696420597
GAPDHP40	0.027	0.422543221	0.696420597
MIR8067	0.027	0.422569166	0.696432367

H6PD	0.027	0.42259423	0.696432367
LINC01214	0.027	0.422614008	0.696440862
LINC00642	0.027	0.422639236	0.696458338
PKHD1	0.027	0.422671026	0.696486627
AC084117	0.027	0.422736898	0.696571072
RP11-96B5	0.027	0.42276577	0.696594545
CPNE8	0.027	0.422821216	0.696613604
FAM96AP2	0.027	0.422864341	0.696637681
LRRC23	0.027	0.422970155	0.696738477
SLC6A1	0.027	0.42319631	0.697013133
AC068858	0.027	0.423217348	0.697013133
LINC01073	0.027	0.42322995	0.697013133
MTND1P22	0.027	0.423267918	0.697020796
CTD-2314B22	0.027	0.423329713	0.697020796
STOM	0.027	0.423351971	0.697020796
RBMX2P3	0.027	0.423356553	0.697020796
ALS2CL	0.027	0.42335735	0.697020796
ARMC9	0.027	0.423370957	0.697020796
RP9	0.027	0.423375703	0.697020796
DSG2-AS1	0.027	0.423450232	0.697071208
RP11-573N10	0.027	0.423522627	0.697153581
CTC-436K13	0.027	0.423529546	0.697153581
RP11-542F9	0.027	0.423672383	0.697364597
RN7SKP77	0.027	0.423782974	0.697447501
RP11-749H20	0.027	0.423829867	0.697479184
CYP2J2	0.027	0.423985717	0.697627271
CHIAP3	0.027	0.423993092	0.697627271
KB-1958F4	0.027	0.424278663	0.697955834
RP11-202D18	0.027	0.42433774	0.697955834
CBR1	0.027	0.424357295	0.697955834
EVX1-AS	0.027	0.424368636	0.697955834
RNF208	0.027	0.424432077	0.697992515
ZNF711	0.027	0.424449823	0.697992946
SKOR1	0.027	0.424496881	0.69804623
FAH	0.027	0.424532537	0.698080761
BPESC1	0.027	0.424567993	0.698101993
RP11-404G16	0.027	0.424574765	0.698101993
LINC00681	0.027	0.424700017	0.698283831
PRX	0.027	0.425038221	0.69856826
RP11-802H3	0.027	0.425057029	0.69856826
TMSB10P2	0.027	0.425063439	0.69856826
RP4-777L9	0.027	0.425078353	0.69856826
EHBP1L1	0.027	0.425156529	0.698672624
MIR30A	0.027	0.42520405	0.698724072
RN7SKP21	0.027	0.425358496	0.69885875
KRT17P1	0.027	0.425502957	0.698952519
RP11-92G19	0.027	0.425540428	0.69898158
RN7SL546P	0.027	0.42555901	0.69898158
CTD-2339M3	0.027	0.425742922	0.699015129
RP11-320A16	0.027	0.425759599	0.699015129
ZNF560	0.027	0.425822961	0.699044194
RP1-315G1	0.027	0.425993038	0.699246999

NDEL1	0.027	0.426005227	0.699246999
LINC00378	0.027	0.426026287	0.699254288
RP11-711C17	0.027	0.426062019	0.699254288
SNORA69	0.027	0.426068395	0.699254288
FLG2	0.027	0.426185658	0.699398538
DLG2-AS1_2	0.027	0.426325797	0.69953047
ARF4P3	0.027	0.426336132	0.69953047
U6atac	0.027	0.426374674	0.69953047
FEV	0.027	0.426376255	0.69953047
CRX	0.027	0.426440143	0.699535737
RP11-212D3	0.027	0.426531252	0.699604089
MIR100HG	0.027	0.4266432	0.699691321
RP11-686G8	0.027	0.426697864	0.699756875
MSTO1	0.027	0.426749997	0.699818274
RP4-668E10	0.027	0.426849993	0.699943628
PSPHP1	0.027	0.427044049	0.700107635
MYO9A	0.027	0.427230178	0.700316355
BMS1P8	0.027	0.427347226	0.70040911
HES1	0.027	0.427396247	0.700419829
LINC01570	0.027	0.427680397	0.700716717
FAM181A	0.027	0.427835512	0.700886435
SNORD114-21	0.027	0.427857568	0.700886435
MFI2-AS1	0.027	0.428022367	0.700965499
SLC25A26	0.027	0.428038289	0.700965499
JADE3	0.027	0.428209956	0.701101983
PPEF1-AS1	0.027	0.42835133	0.701237027
RP11-298H24	0.027	0.428391275	0.701278314
MIR4783	0.027	0.428493799	0.701422036
APOL5	0.027	0.428545383	0.701453875
PNMA6B	0.027	0.428644215	0.701475381
KRT8P14	0.027	0.428711304	0.701531427
FGF9	0.027	0.428808843	0.701545051
RPS3AP32	0.027	0.428858302	0.701545051
CHRFAM7A	0.027	0.428863117	0.701545051
CTD-2318B16	0.027	0.428919992	0.701608672
RP11-753G20	0.027	0.428955038	0.701608672
PIGZ	0.027	0.428958878	0.701608672
RN7SKP296	0.027	0.428969852	0.701608672
PAX9	0.027	0.429039211	0.701663708
MTND3P2	0.027	0.429089143	0.701697178
RN7SKP268	0.027	0.429169204	0.701755817
JAZF1	0.027	0.429227719	0.701780814
ADAM20P3	0.027	0.429260381	0.701808527
CTD-2267G17	0.027	0.429460122	0.702018508
GPR125	0.027	0.429538139	0.702093931
AC135983	0.027	0.429597232	0.70212544
AL591034	0.027	0.429635416	0.702156543
RN7SL493P	0.027	0.429714183	0.702236233
RP11-145E5	0.027	0.429717966	0.702236233
FGB	0.027	0.42973421	0.702236233
NIPA2P4	0.027	0.429785903	0.702281991
AC066614	0.027	0.430037126	0.702558737

TSEN34		0.027	0.430229316	0.702699777
LCE5A		0.027	0.430294221	0.702699777
MPC2		0.027	0.430295115	0.702699777
AL354720		0.027	0.430297971	0.702699777
CICP27		0.027	0.430378488	0.702699777
OR2H2		0.027	0.430390414	0.702699777
KRT8P4		0.027	0.430399448	0.702699777
PC		0.027	0.430410438	0.702699777
LIPE-AS1		0.027	0.430535044	0.702830935
RNU6-1181P		0.027	0.430591268	0.702887219
PDE4A		0.027	0.430646249	0.702888576
GJC3		0.027	0.430662548	0.702888576
FDX1P2		0.027	0.430699853	0.702888576
SDHAF2		0.027	0.430734138	0.702888576
RP11-481F24		0.027	0.43077055	0.702888576
RP11-403I13		0.027	0.430776968	0.702888576
MPP7		0.027	0.430839939	0.702943159
MTND5P20		0.027	0.4308904	0.70298804
NDRG1		0.027	0.430986998	0.703086752
RN7SL190P		0.027	0.431023114	0.703091094
NAALADL2-AS2		0.027	0.431056573	0.703091094
LINC01533		0.027	0.431076941	0.703091094
STARD8		0.027	0.431105305	0.703091094
MRPL9		0.027	0.431136004	0.703091094
AL078471		0.027	0.43125765	0.703188308
RN7SL283P		0.027	0.431327809	0.703188308
AL357973		0.027	0.431422556	0.703196721
MIA-RAB4B		0.027	0.431423524	0.703196721
UNC93B8		0.027	0.431548131	0.703241416
ATP5G1P6		0.027	0.431579376	0.703241416
OR6K2		0.027	0.431607404	0.703241416
RN7SL706P		0.027	0.43161268	0.703241416
TMEM216		0.027	0.431641122	0.703241416
LDHAL6B		0.027	0.431652098	0.703241416
AL138744		0.027	0.431676482	0.703248017
	5-Sep	0.027	0.431709926	0.703278446
LINC00493		0.027	0.431784818	0.703301069
RP11-203F10		0.027	0.431829692	0.703301069
C9orf40		0.027	0.431837951	0.703301069
MAP3K11		0.027	0.431881805	0.703337133
HMGB3P5		0.027	0.431991148	0.70344641
IGDCC4		0.027	0.432046999	0.703466631
LINC01436		0.027	0.432117108	0.703556731
AC104135		0.027	0.432229145	0.703618877
ARRDC1		0.027	0.432309272	0.703725262
RANBP17		0.027	0.432412734	0.703845566
PLP2		0.027	0.432492502	0.703909945
TNIK		0.027	0.432662958	0.703971752
CBWD5		0.027	0.432717436	0.703971752
RN7SL843P		0.027	0.432726752	0.703971752
RP11-1217F2		0.027	0.43276614	0.703986644
RPS2P55		0.027	0.43278025	0.703986644

EEF1GP1	0.027	0.432905505	0.704118247
COQ9	0.027	0.432931859	0.704137063
SMPDL3B	0.027	0.43303765	0.704257647
MAPK4	0.027	0.433077255	0.704257647
LINC00387	0.027	0.433086318	0.704257647
PRDX4	0.027	0.43309472	0.704257647
OR4B1	0.027	0.433214374	0.704356024
LLNLR-276E7	0.027	0.433285727	0.704416902
AC008781	0.027	0.433296188	0.704416902
KRT13	0.027	0.433394946	0.704481267
MAGEA11	0.027	0.433448913	0.704496857
PRRT4	0.027	0.433477949	0.704520007
AC090079	0.027	0.433495057	0.704523769
AC007106	0.027	0.433605752	0.704635618
CTC-228N24	0.027	0.433608262	0.704635618
RP11-761N21	0.027	0.433679132	0.704678653
COX20P1	0.027	0.433747854	0.704742233
RELT	0.027	0.433765453	0.704746784
AC107993	0.027	0.433933503	0.70491865
FAM228A	0.027	0.433945239	0.70491865
MME-AS1	0.027	0.43399586	0.704976836
OR52I2	0.027	0.434147592	0.705093807
MT-TC	0.027	0.434208958	0.705093807
MARK2P13	0.027	0.434217716	0.705093807
GAGE12J	0.027	0.434250038	0.705093807
CXorf22	0.027	0.434269357	0.705093807
RPSAP23	0.027	0.434307026	0.705093807
RP11-373N22	0.027	0.434319546	0.705093807
RP11-626A5	0.027	0.434461931	0.705228805
UBE2E1-AS1	0.027	0.434725479	0.705416184
RP11-6J21	0.027	0.434879555	0.705570041
RP1-52J10	0.027	0.434953659	0.705605266
VDR	0.027	0.435123183	0.705704933
DNAJC22	0.027	0.435174945	0.705704933
RP11-20G6	0.027	0.435377056	0.705872245
CNN2P10	0.027	0.435396923	0.705880425
AC005215	0.027	0.43546886	0.705946147
PIK3C2G	0.027	0.435471517	0.705946147
AC011286	0.027	0.43550561	0.705953823
SGCA	0.027	0.435516308	0.705953823
RP11-685F15	0.027	0.435767219	0.706143463
NTHL1	0.027	0.435781566	0.706143463
RP11-367H1	0.027	0.435835877	0.706183416
AC096649	0.027	0.436116561	0.70642665
RP11-260M19	0.027	0.436224196	0.706548199
RPS6P16	0.027	0.43634785	0.706682352
CCNY	0.027	0.436388674	0.70668836
CYCSP1	0.027	0.436419022	0.70668836
SUMF2	0.027	0.436445032	0.706689588
SNORD115	0.026	0.436642969	0.706889925
RP4-601P9	0.026	0.436750833	0.707016482
RPL17P26	0.026	0.436789949	0.707027142

RN7SL220P	0.026	0.436792912	0.707027142
EEF1A1P6	0.026	0.436801953	0.707027142
AL512655	0.026	0.43688582	0.707066771
RP11-507F16	0.026	0.437103448	0.707322843
MT-TT	0.026	0.437145412	0.707366716
MIR19B2	0.026	0.437213038	0.707405272
GABARAPL1	0.026	0.437238024	0.707405272
CES5A	0.026	0.437239272	0.707405272
SLC2A8	0.026	0.437358931	0.707440877
RNU4-42P	0.026	0.437411114	0.707440877
LINC01271	0.026	0.437443861	0.707440877
CX3CL1	0.026	0.437444383	0.707440877
CTA-14H9	0.026	0.437484371	0.707440877
NCOA7	0.026	0.43760693	0.707584629
AC004791	0.026	0.437635673	0.707607081
RP5-1185H19	0.026	0.43767552	0.707647486
RP11-339A11	0.026	0.437755668	0.707688572
PNLIPRP1	0.026	0.437767978	0.707688572
NXNL1	0.026	0.437775227	0.707688572
AAED1	0.026	0.43783436	0.70773612
AC133104	0.026	0.437856791	0.707748359
RPS7P9	0.026	0.437986051	0.707909242
CRLS1	0.026	0.438065731	0.707990207
RP11-463J17	0.026	0.438065874	0.707990207
ACN9	0.026	0.43810672	0.708028477
LARP7P4	0.026	0.438119286	0.708028477
ARHGAP5-AS1	0.026	0.43830944	0.708215397
EMC1	0.026	0.438436974	0.708311152
SLC25A11	0.026	0.43852287	0.708392251
FAM96B	0.026	0.438600056	0.708399381
MIR5706	0.026	0.438644234	0.708420107
TPI1P4	0.026	0.438710715	0.708479426
FEM1AP1	0.026	0.438827736	0.708541616
LIMS1	0.026	0.438904998	0.708541616
RP5-1112D6	0.026	0.43893117	0.708541616
TMEM98	0.026	0.438937632	0.708541616
AL445464	0.026	0.438947346	0.708541616
RP11-120J4	0.026	0.438996101	0.708541616
LEFTY2	0.026	0.439024761	0.708541616
RP1-313L4	0.026	0.439026593	0.708541616
PDCD5P1	0.026	0.439086258	0.708605354
RP11-193H22	0.026	0.439178873	0.708658767
DUSP7	0.026	0.439244325	0.708675627
PCNXL2	0.026	0.43926372	0.708675627
VN1R20P	0.026	0.439311564	0.708728807
RP11-5N19	0.026	0.43936621	0.708748915
NBR2	0.026	0.439411458	0.708769923
RPS29	0.026	0.43949229	0.708835378
AC004988	0.026	0.439493025	0.708835378
XX-DJ76P10__A	0.026	0.439496687	0.708835378
CDRT7	0.026	0.439621346	0.708940402
AC009365	0.026	0.439639437	0.708945572

SGK2	0.026	0.439668297	0.708963173
RPP21	0.026	0.439685674	0.708963173
RN7SL184P	0.026	0.439740849	0.708968071
CBX3P3	0.026	0.439742704	0.708968071
TBC1D22A	0.026	0.439759096	0.708970498
RP1-292B18	0.026	0.43979613	0.709006204
BEND3P3	0.026	0.439970675	0.709184298
RHOBTB1	0.026	0.440037966	0.709204028
LINC01230	0.026	0.440122144	0.70928467
MIR520C	0.026	0.440124534	0.70928467
MIR6087	0.026	0.440188152	0.70928467
SNX29	0.026	0.4401988	0.70928467
RN7SKP291	0.026	0.440262804	0.709287579
NANOS2	0.026	0.44028341	0.709287579
FADS6	0.026	0.440344635	0.709335318
IQCF5-AS1	0.026	0.440345547	0.709335318
ACTBP8	0.026	0.440469384	0.709439924
RPL19P11	0.026	0.440482247	0.709439924
RP11-740C1	0.026	0.440755524	0.709736056
MIR143HG	0.026	0.440827246	0.709827548
RP11-100F15	0.026	0.440892731	0.709860996
IHH	0.026	0.440966914	0.709908438
RPS4XP19	0.026	0.441029738	0.709977086
KB-1466C5	0.026	0.441161483	0.710097602
MBL1P	0.026	0.441183471	0.710097602
MIR6853	0.026	0.441203692	0.710097602
PSMA3P	0.026	0.441426374	0.710221622
CMAHP	0.026	0.441429623	0.710221622
B3GNT3	0.026	0.441730962	0.710427482
RP11-295M18	0.026	0.441743219	0.710427482
RP11-120E13	0.026	0.441924197	0.710523532
FER1L6-AS2	0.026	0.441930808	0.710523532
AL163953	0.026	0.442271267	0.710974909
RPS24P7	0.026	0.442364907	0.71103427
RP5-1195D24	0.026	0.44236791	0.71103427
RP11-818F20	0.026	0.4424746	0.711157756
HK3	0.026	0.442511777	0.711193507
SNRPD2P2	0.026	0.442716946	0.711372983
IGHD1OR15-1B	0.026	0.442927086	0.711561132
RP11-282K24	0.026	0.443448078	0.712025542
RNU4-53P	0.026	0.443627496	0.712177596
RP11-116B13	0.026	0.44368474	0.712213451
USP17L16P	0.026	0.443731536	0.712264564
AC004819	0.026	0.443851541	0.712378996
AC084262	0.026	0.444018531	0.71253094
RP11-232M24	0.026	0.444106869	0.712602852
HIGD1A	0.026	0.444132964	0.712620716
RPL21P18	0.026	0.44428112	0.712707654
RP11-395M19	0.026	0.444306863	0.712707654
RNU4-1	0.026	0.444396399	0.712827269
AC092155	0.026	0.444467659	0.712878587
SCRG1	0.026	0.444479143	0.712878587

KCNF1	0.026	0.444582321	0.71298142
MPLKIP	0.026	0.44461487	0.712985603
SRRM3	0.026	0.444761355	0.713088404
ACAT2	0.026	0.444808562	0.713088404
RP11-305F5	0.026	0.444819278	0.713088404
TNFSF15	0.026	0.444827771	0.713088404
CTD-3093M3	0.026	0.444831575	0.713088404
TGM4	0.026	0.444910923	0.713100203
RP11-123G9	0.026	0.444991921	0.713182024
ADAMTS17	0.026	0.445043661	0.713240946
BCL6B	0.026	0.445079811	0.71327488
RPL27A	0.026	0.445138763	0.71329735
RP1-213J1P__B	0.026	0.445241204	0.713393791
RN7SL366P	0.026	0.445253346	0.713393791
TARS2P1	0.026	0.445261841	0.713393791
RN7SL732P	0.026	0.445287212	0.713393791
AL445204	0.026	0.445313226	0.713407533
LURAP1	0.026	0.445340729	0.713407533
RP11-481A20	0.026	0.445382436	0.713407533
LINC01427	0.026	0.445387272	0.713407533
NDUFA3P3	0.026	0.445456843	0.713494974
THUMPD3P1	0.026	0.445524647	0.71357958
MIR8070	0.026	0.445562837	0.713586452
RP11-407P15	0.026	0.445573887	0.713586452
RP11-408E5	0.026	0.445800958	0.713806826
LURAP1L-AS1	0.026	0.445816403	0.713806826
OFD1P9Y	0.026	0.44584863	0.713834427
SLC9A7P1	0.026	0.445932249	0.713934969
AADACL3	0.026	0.44595472	0.713934969
ALDH7A1P1	0.026	0.446134158	0.714128818
CCDC6	0.026	0.44613705	0.714128818
RNU4-90P	0.026	0.446144121	0.714128818
OR2T4	0.026	0.446167449	0.714128818
CYP4A22-AS1	0.026	0.446188373	0.714138309
RN7SL591P	0.026	0.446385535	0.714357846
RP11-359H3	0.026	0.446454245	0.714386695
LINC01428	0.026	0.446500279	0.714397447
HHIPL2	0.026	0.446596021	0.714502626
RP11-173M11	0.026	0.446611737	0.714503769
SYN1	0.026	0.446731477	0.714538674
NT5CP2	0.026	0.44681303	0.714574087
RP11-84O12	0.026	0.446839613	0.714574087
OBFC1	0.026	0.446872482	0.714574087
AL928970	0.026	0.446945469	0.714597497
TREH	0.026	0.447021733	0.71467956
RP11-815D16	0.026	0.447037106	0.714680146
BZW2	0.026	0.447205186	0.714908084
TCTE1	0.026	0.447221412	0.714908084
RP11-659O3	0.026	0.447224714	0.714908084
RP11-338O1	0.026	0.44730072	0.714957592
RN7SL111P	0.026	0.447365909	0.715013797
MIR4435-2	0.026	0.4474422	0.71508167

MRPL23	0.026	0.447453418	0.71508167
RP6-114E22	0.026	0.447469355	0.715083145
RNA5SP48	0.026	0.447550101	0.715156774
OLFML2A	0.026	0.447569068	0.715156774
AC079340	0.026	0.447624765	0.715156774
AP001065	0.026	0.447753592	0.715156774
YAP1P2	0.026	0.447758574	0.715156774
RP3-497J21	0.026	0.44776834	0.715156774
OR2M2	0.026	0.447906877	0.715278327
NUDT19P4	0.026	0.447954291	0.715313319
RN7SL863P	0.026	0.448119938	0.715465955
AC021914	0.026	0.448170334	0.71547201
CKM	0.026	0.448207758	0.71547201
AC068499	0.026	0.448293922	0.715528648
PCP4L1	0.026	0.44837483	0.715593728
SKINTL	0.026	0.44845234	0.715669468
ATP6V0E2	0.026	0.448597272	0.715828802
RNU6-115P	0.026	0.448637708	0.715869342
MIR4761	0.026	0.448660197	0.715871302
AP002414	0.026	0.448668998	0.715871302
LINC00458	0.026	0.448928839	0.716165929
EFNB2	0.026	0.449134299	0.716376853
RARRES2P3	0.026	0.449351599	0.716624317
MIR7152	0.026	0.449550217	0.716844867
FTLP19	0.026	0.449595251	0.716844867
RPL7AP69	0.026	0.449614989	0.716852339
RP11-679I18	0.026	0.449673375	0.716897431
MINOS1P4	0.026	0.450072261	0.717397238
AC116176	0.026	0.450107382	0.717397238
MIR1302-3	0.026	0.450167975	0.717404016
KRTAP10-9	0.026	0.450228979	0.717422984
KRTAP3-2	0.026	0.450264382	0.717437603
MIR3179-3	0.026	0.450294999	0.717437603
AC018738	0.026	0.450593077	0.717835044
OSGIN1	0.026	0.450818751	0.717923881
MSX2	0.026	0.450854831	0.717923881
RPL5P8	0.026	0.450857423	0.717923881
TMSB4XP4	0.026	0.450872555	0.717923881
AC099339	0.026	0.450905097	0.717923881
USP12-AS1	0.026	0.451046221	0.718004558
RP11-401F2	0.026	0.451117459	0.718069958
AL160269	0.026	0.451182556	0.718103955
RN7SL72P	0.026	0.451219255	0.718103955
RP11-778D12	0.026	0.451225406	0.718103955
DUTP1	0.026	0.451339186	0.718214241
RP11-20J1	0.026	0.451602717	0.718482183
CTD-2019O4	0.026	0.451774462	0.718671724
BRAFP1	0.026	0.451848315	0.718728866
AC008154	0.026	0.451895075	0.718779239
RNF113B	0.026	0.451972017	0.718853607
RP11-429G19	0.026	0.452020453	0.718906636
RWDD2B	0.026	0.452066358	0.718920719

RASGEF1C	0.026	0.452083975	0.718920719
RP11-560B16	0.026	0.452166492	0.718970839
IGHVII-31-1	0.026	0.452333247	0.719175289
GABRP	0.026	0.452357199	0.719175289
RASD2	0.026	0.452411764	0.719175289
RP11-80P20	0.026	0.45241307	0.719175289
OR51A9P	0.026	0.452471378	0.71919864
ZNF649	0.026	0.452475866	0.71919864
RP11-368J21	0.026	0.452547132	0.71921037
APOBEC3B-AS1	0.026	0.452591027	0.71921037
MRPS5P3	0.026	0.452639241	0.719242289
MAPT-IT1	0.026	0.452761893	0.719326116
RN7SL464P	0.026	0.452833297	0.719360956
YARS	0.026	0.453043145	0.71941778
RP11-624J12	0.026	0.453051363	0.71941778
UGT2B11	0.026	0.453066893	0.71941778
AC026797	0.026	0.453201909	0.719475464
RP11-208P4	0.026	0.453206678	0.719475464
RNF223	0.026	0.453259631	0.719524267
DPRXP7	0.026	0.453284555	0.719524267
RN7SL598P	0.026	0.45328503	0.719524267
AC105398	0.026	0.453389287	0.719617809
KRT8P24	0.026	0.453438323	0.71965007
RNU4-66P	0.026	0.453500274	0.71965007
C1orf210	0.026	0.453641113	0.719746735
TCEB3B	0.026	0.453704528	0.719746735
STXBP5-AS1	0.026	0.453710252	0.719746735
RP5-827O9	0.026	0.453737876	0.719746735
RP13-347D8	0.026	0.453740002	0.719746735
SAC3D1	0.026	0.453740872	0.719746735
CTD-2066L21	0.026	0.453757647	0.719746735
GAPDHP32	0.026	0.453835419	0.71982215
XRCC6P1	0.026	0.45400991	0.720026971
RP13-565O16	0.026	0.454046184	0.720036547
ATP5F1P3	0.025	0.454122388	0.720109438
SLC26A10	0.025	0.454185099	0.720136951
THRB-IT1	0.025	0.454202675	0.720140844
GFER	0.025	0.454342324	0.720338278
FAM35DP	0.025	0.45451159	0.720446308
RN7SL371P	0.025	0.454515042	0.720446308
MEIS3P1	0.025	0.454573999	0.720489729
MIR8074	0.025	0.454728195	0.720601467
TMPRSS15	0.025	0.454735278	0.720601467
AC127391	0.025	0.454757153	0.720612155
RN7SKP6	0.025	0.454840878	0.72069456
PPP1R11P2	0.025	0.454866489	0.72069456
NTRK3-AS1	0.025	0.454884816	0.72069456
RP11-25I15	0.025	0.45493253	0.720698231
RP11-79P5	0.025	0.454995337	0.720725809
STK40	0.025	0.455127916	0.720846252
GNG10P1	0.025	0.455131914	0.720846252
XKRYP1	0.025	0.455397831	0.721197607

RHOA	0.025	0.45544032	0.721197607
RPS21	0.025	0.455645907	0.721341632
SERPINA12	0.025	0.4557413	0.721341632
OR4F28P	0.025	0.455753413	0.721341632
NRBP2	0.025	0.455909318	0.721453736
GTF2IRD1	0.025	0.456190319	0.72175452
RP5-1185I7	0.025	0.456354608	0.721894543
ATP5C1	0.025	0.456432103	0.721993151
PRPS1	0.025	0.456531216	0.722077738
RP11-755O11	0.025	0.456585697	0.722077738
RP11-1102P22	0.025	0.456595273	0.722077738
RP11-63H19	0.025	0.45662801	0.722077738
AC023158	0.025	0.45663192	0.722077738
HENMT1	0.025	0.456668011	0.722077738
AP000620	0.025	0.456700829	0.722077738
RP11-445J9	0.025	0.456765158	0.722088299
SCARNA18	0.025	0.456907572	0.722141095
CRABP1	0.025	0.456912572	0.722141095
LINC00319	0.025	0.456976925	0.7221594
AC009009	0.025	0.457147723	0.72223676
SSXP5	0.025	0.457353061	0.722394264
RP11-464C19	0.025	0.457385319	0.7223973
ULBP1	0.025	0.457435442	0.722452508
MIR497HG	0.025	0.457462804	0.722461672
AP000662	0.025	0.457552428	0.722517472
RNU6-567P	0.025	0.457679887	0.722608923
AC092620	0.025	0.457791937	0.722660027
RPL13AP2	0.025	0.457794438	0.722660027
RNU6ATAC5P	0.025	0.457905002	0.722714791
NME3	0.025	0.457959447	0.722752819
AC011431	0.025	0.458019381	0.722777605
MCC	0.025	0.458020679	0.722777605
RP11-521M14	0.025	0.458242325	0.722958458
MIR381	0.025	0.458301295	0.722958458
MIR652	0.025	0.458330983	0.722960944
HPS6	0.025	0.458334196	0.722960944
RPS17P2	0.025	0.458369622	0.722969802
AL359218	0.025	0.458413898	0.722969802
MTND2P19	0.025	0.458423148	0.722969802
MRPL54	0.025	0.458430891	0.722969802
VN1R36P	0.025	0.458473614	0.723013239
TSPY6P	0.025	0.458573054	0.723093484
MOSPD1	0.025	0.458585229	0.723093484
FAM3D	0.025	0.458686908	0.723205925
RP11-453N3	0.025	0.458727871	0.723222627
RNA5SP361	0.025	0.458762652	0.72322958
BLID	0.025	0.458868585	0.72330504
RP11-111M22	0.025	0.458916524	0.72330504
POLR2G	0.025	0.458931104	0.72330504
TSPY19P	0.025	0.458932013	0.72330504
KRTAP20-2	0.025	0.458970511	0.723341778
AC068491	0.025	0.459145307	0.723449288

PTPRG-AS1	0.025	0.459153813	0.723449288
DSPP	0.025	0.459168139	0.723449288
HIGD1AP8	0.025	0.459227466	0.723449288
ZNF777	0.025	0.459245632	0.723449288
RPS20P32	0.025	0.45927405	0.723449288
SYNM	0.025	0.459353043	0.723537633
ESPNL	0.025	0.459404258	0.723594372
MIR5000	0.025	0.459477787	0.723626638
GSTA6P	0.025	0.459500712	0.723626638
FOXO3	0.025	0.459529109	0.723631859
PIWIL2	0.025	0.459534415	0.723631859
C7orf61	0.025	0.459696987	0.723733834
HS3ST1	0.025	0.459705544	0.723733834
CTD-3107M8	0.025	0.459785222	0.723811423
NENFP3	0.025	0.459920595	0.723928819
AC087073	0.025	0.460058189	0.724049678
RP11-972K6	0.025	0.460299184	0.724320796
FBXL21	0.025	0.460302911	0.724320796
SMIM8	0.025	0.460404028	0.724380967
CYP7B1	0.025	0.460406351	0.724380967
C11orf52	0.025	0.460442783	0.724380967
TMEM212-IT1	0.025	0.460446836	0.724380967
SNORD88	0.025	0.460559541	0.724470508
MIR3150A	0.025	0.460614972	0.724495022
EGR4	0.025	0.46073431	0.724610936
RNVU1-17	0.025	0.460807501	0.724675687
RP11-649G15	0.025	0.460858277	0.724675687
HDGF	0.025	0.46087887	0.724675687
RN7SKP278	0.025	0.460906223	0.724677932
HMGB1P1	0.025	0.461116393	0.724828887
RP11-472G21	0.025	0.461193718	0.72485739
MAP1LC3A	0.025	0.461227039	0.72485739
MIR30B	0.025	0.461275983	0.724864434
SHROOM2P1	0.025	0.461350163	0.724885334
RP11-606P2	0.025	0.461387047	0.724895457
RP11-268P4	0.025	0.461561722	0.725002478
GUSBP1	0.025	0.46160328	0.725043844
TFRC	0.025	0.461667777	0.725073412
RHOJ	0.025	0.461731281	0.725101416
RNU4-91P	0.025	0.461762937	0.72510331
AL121594	0.025	0.461804605	0.725132648
RP11-707P20	0.025	0.461848541	0.725148673
EIF3I	0.025	0.461859302	0.725148673
LINC01034	0.025	0.461867953	0.725148673
ZNF90P1	0.025	0.461936142	0.725207918
AC024896	0.025	0.46200246	0.725288126
LRP10	0.025	0.46220988	0.725398556
GUSB	0.025	0.462231173	0.725408068
RNU4-76P	0.025	0.462253675	0.725419479
PTPRF	0.025	0.462305367	0.725476696
RNY3P14	0.025	0.462375752	0.725500096
MIR3926-2	0.025	0.462379654	0.725500096

OXCT1-AS1	0.025	0.462381211	0.725500096
PIP	0.025	0.462570144	0.725698073
HSD17B12	0.025	0.462712058	0.725851822
MIR3917	0.025	0.46273821	0.725868939
ABLIM1	0.025	0.462759662	0.725878682
RP11-671M22	0.025	0.462873404	0.725948243
RNU7-87P	0.025	0.462901933	0.725948243
RP11-105N14	0.025	0.463005644	0.726023554
TIE1	0.025	0.463019704	0.726023554
FTO-IT1	0.025	0.463077641	0.726090496
RPSAP8	0.025	0.463098587	0.726099434
AC078941	0.025	0.463188822	0.72614355
RNU4-69P	0.025	0.463261585	0.726153626
TSLP	0.025	0.463318239	0.726153626
RP5-836N17	0.025	0.4633499	0.726153626
RNU6-95P	0.025	0.463362535	0.726153626
RP11-263G22	0.025	0.463414835	0.726153626
SLC12A2	0.025	0.463425411	0.726153626
AC105914	0.025	0.463426011	0.726153626
RP3-404K8	0.025	0.463483824	0.726153626
RP4-644L1	0.025	0.46357883	0.726254693
SNORA7	0.025	0.463761539	0.726456424
AC034243	0.025	0.464010932	0.726542475
RNY3P16	0.025	0.464029351	0.726542475
RN7SKP250	0.025	0.46403656	0.726542475
DDX43P2	0.025	0.464055724	0.726542475
AC012499	0.025	0.464065635	0.726542475
RP11-696D21	0.025	0.464067622	0.726542475
RP11-278J20	0.025	0.464180882	0.726599486
PXYLP1	0.025	0.464186395	0.726599486
RNU2-36P	0.025	0.464224684	0.726599486
CTA-929C8	0.025	0.464234128	0.726599486
CT45A11P	0.025	0.464378033	0.726660419
RP11-784B15	0.025	0.464501349	0.7267676
SBK1	0.025	0.4647832	0.727083412
RP11-618G20	0.025	0.464824776	0.727083412
PRDX2P2	0.025	0.464850775	0.727083412
GLI4	0.025	0.464898007	0.727083412
C16orf90	0.025	0.465032715	0.727192866
GPRC5C	0.025	0.465091277	0.727260564
AC007115	0.025	0.465108561	0.727263714
EMID1	0.025	0.465139352	0.727267161
RP4-718D20	0.025	0.465210236	0.727351057
BCAM	0.025	0.465235842	0.727367214
AC079612	0.025	0.465354995	0.727457982
KRTAP12-1	0.025	0.465517607	0.727621258
snoU109	0.025	0.465589932	0.727658059
KCNN4	0.025	0.465618241	0.727678424
MIR589	0.025	0.466073442	0.728150889
EIF1P7	0.025	0.46619402	0.728261648
DHCR7	0.025	0.466296969	0.728313017
TNN	0.025	0.466350796	0.728341933

AQP5	0.025	0.466418586	0.728341933
STEAP3-AS1	0.025	0.466455109	0.728341933
MIR548F3	0.025	0.466455701	0.728341933
RP13-582O9	0.025	0.466538381	0.72840593
CTA-331F8	0.025	0.466568532	0.72840593
DNM1P35	0.025	0.466626097	0.72841695
SOCS2P1	0.025	0.466716329	0.728475984
MRPS21P6	0.025	0.466763066	0.728487499
RP11-239A17	0.025	0.466844423	0.7285906
BTD	0.025	0.466862492	0.728594924
C1orf234	0.025	0.467037402	0.728700748
MIR6777	0.025	0.46712151	0.728784228
ELOVL3	0.025	0.46720522	0.728843203
HSPA8P6	0.025	0.467225216	0.728847601
GDF11	0.025	0.467277427	0.728847601
RP1-145M24	0.025	0.467284555	0.728847601
RAP1BP3	0.025	0.467308544	0.728859203
RP11-472G23	0.025	0.4673226	0.728859203
RNF149	0.025	0.467460198	0.729029703
CAPN6	0.025	0.467462534	0.729029703
RNU7-96P	0.025	0.46758418	0.729052306
RN7SKP76	0.025	0.467627781	0.729075092
RP11-442P12	0.025	0.46773046	0.729145819
AC114737	0.025	0.467844316	0.729243045
CFAP221	0.025	0.467925748	0.729323309
RARSP1	0.025	0.468022433	0.729401323
ZBTB7B	0.025	0.468094845	0.729442567
SLC25A6P5	0.025	0.468172078	0.729487224
RP11-524P6	0.025	0.468184768	0.729487224
RNU6-1243P	0.025	0.468387539	0.729662415
RN7SL634P	0.025	0.468404449	0.729662415
PPIAP14	0.025	0.468585978	0.729825841
PON1	0.025	0.468660405	0.729917891
HR	0.025	0.468728292	0.729952011
CIDEC	0.025	0.468879838	0.730106693
LL22NC03-13G6	0.025	0.468888937	0.730106693
STEAP1B	0.025	0.468935501	0.730131455
EPHB1	0.025	0.469109258	0.730330368
RP11-477N12	0.025	0.469170194	0.730401361
RNU6-362P	0.025	0.469263705	0.73049828
MIR5704	0.025	0.469278464	0.73049828
CTC-308K20	0.025	0.469531001	0.73079002
BPIFA2	0.025	0.4695426	0.73079002
SNORD115-39	0.025	0.469573229	0.730813808
TMEM151B	0.025	0.469620303	0.730833598
AVPR1B	0.025	0.4696281	0.730833598
RNU6-674P	0.025	0.469714017	0.730891187
AC007367	0.025	0.469715392	0.730891187
NDUFA6	0.025	0.46973037	0.730891187
DMTN	0.025	0.469829401	0.730981048
RP11-93I21	0.025	0.469834166	0.730981048
ALDH7A1P2	0.025	0.469916059	0.730983153

RP11-520A21	0.025	0.469927608	0.730983153
RP11-454H19	0.025	0.470076256	0.731158305
LSM1	0.025	0.470086263	0.731158305
CTD-2061E19	0.025	0.47019731	0.731235504
RAP1BP1	0.025	0.470257353	0.731265164
SKOR2	0.025	0.470443971	0.731356411
SYNPO2L	0.025	0.470486143	0.731374226
RP11-149P14	0.025	0.47053745	0.731417106
SH3BP4	0.025	0.470544442	0.731417106
AC090666	0.025	0.470656087	0.731542898
ERI1	0.025	0.470789756	0.731666947
RP11-138I17	0.025	0.470815542	0.731666947
FZD3	0.025	0.471048252	0.731818082
PLBD1	0.025	0.471138022	0.731838184
FAM83F	0.025	0.471185768	0.73187106
AC243945	0.025	0.471209743	0.731877982
PRSS50	0.025	0.471320438	0.732002168
CCER2	0.025	0.471471143	0.732212349
UQCRFS1P1	0.025	0.471569155	0.732284548
SPTLC1P3	0.025	0.471609862	0.732284548
LCTL	0.025	0.471679854	0.732325481
SLC2A2	0.025	0.471739444	0.732325481
RP1-169P22	0.025	0.471757412	0.732325481
RAB11B-AS1	0.025	0.471757585	0.732325481
ZNF391	0.025	0.471804693	0.732340598
IPO13	0.025	0.471815124	0.732340598
RPP25	0.025	0.47193156	0.732425848
CTD-2651C21	0.025	0.471992893	0.732448928
AC024067	0.024	0.472038705	0.732448928
RP11-384C21	0.024	0.472136972	0.732489412
AC004552	0.024	0.472144577	0.732489412
RNU6-338P	0.024	0.472378329	0.732665604
AC009495	0.024	0.472472165	0.732715698
AC004540	0.024	0.472578304	0.732735443
MAMSTR	0.024	0.472587397	0.732735443
CTC-251D13	0.024	0.472666529	0.732780113
RP11-46P12	0.024	0.472752192	0.732815903
SNORD116-11	0.024	0.472785576	0.732843789
MAGEA2	0.024	0.47285971	0.732887155
DSCR4-IT1	0.024	0.472890833	0.732891579
SPATA31A7	0.024	0.472946508	0.732926279
KCNJ16	0.024	0.47298785	0.732966498
YES1	0.024	0.473108586	0.733129742
RPS6KA4	0.024	0.473176663	0.733205847
AC092013	0.024	0.473188488	0.733205847
CACNA2D3-AS1	0.024	0.473314764	0.733268156
RNA5SP450	0.024	0.473318502	0.733268156
SLC6A16	0.024	0.473370141	0.733272611
AC097381	0.024	0.473395765	0.733288454
ZSCAN5C	0.024	0.473499716	0.733401768
OBSL1	0.024	0.473580859	0.733479742
RP11-180P8	0.024	0.473601165	0.73348734

OR8R1P	0.024	0.473775581	0.733614332
GUSBP5	0.024	0.47386718	0.733689874
TRPM3	0.024	0.474045035	0.733912261
KRI1	0.024	0.474074982	0.733934768
RP11-165A20	0.024	0.474102408	0.733944808
RP11-378A12	0.024	0.474161742	0.733973651
GPAA1P2	0.024	0.474279592	0.734059464
PTPRM	0.024	0.474355055	0.734081494
RNA5SP308	0.024	0.474380027	0.734081494
MIR885	0.024	0.474395113	0.734081494
ZNHIT2	0.024	0.474400955	0.734081494
GRID2IP	0.024	0.474729077	0.734421425
NDUFA5P2	0.024	0.474772049	0.734440963
FRG2B	0.024	0.47483494	0.734490538
DEGS2	0.024	0.474861779	0.734508198
RN7SKP221	0.024	0.474989449	0.734644956
FLT4	0.024	0.475029289	0.734648004
DMRT3	0.024	0.475047857	0.734652865
AC008906	0.024	0.475173822	0.734752237
RP1-121G13	0.024	0.475278121	0.734841939
COPS7A	0.024	0.475359338	0.734882288
ATP6V0D1	0.024	0.475435027	0.73492816
RP11-478H13	0.024	0.475764244	0.735283242
AC013437	0.024	0.475802632	0.735317152
AC010904	0.024	0.475827742	0.735317152
GSS	0.024	0.475832503	0.735317152
RP11-10C8	0.024	0.475872989	0.735355856
RP11-132G19	0.024	0.47598839	0.735486457
AL138994	0.024	0.476062877	0.735577687
GSTA9P	0.024	0.476085673	0.735584287
RP11-211C9	0.024	0.47609895	0.735584287
RN7SL465P	0.024	0.476154433	0.735623692
CRYBA4	0.024	0.476187015	0.735646456
RNF11	0.024	0.476332986	0.735756345
RP11-34P13	0.024	0.476527847	0.735952058
CTB-164N12	0.024	0.476536954	0.735952058
KRTAP1-4	0.024	0.47657992	0.735970684
WRAP53	0.024	0.476696017	0.736054504
RP5-1007F24	0.024	0.476720192	0.736064481
NIPAL2	0.024	0.476781637	0.736079469
NOD1	0.024	0.47681142	0.736079469
RNA5SP150	0.024	0.476846632	0.736095176
UBE2A	0.024	0.476902128	0.736095176
MIR3935	0.024	0.476907822	0.736095176
SNCA	0.024	0.476939362	0.736096147
RP11-179A10	0.024	0.476964991	0.736104205
NUTF2P7	0.024	0.476986899	0.736104205
RNU6-830P	0.024	0.47699095	0.736104205
RPL23AP62	0.024	0.477125705	0.736243091
TC2N	0.024	0.477228148	0.736327102
RN7SKP152	0.024	0.477267989	0.736358986
RP11-758M4	0.024	0.477375331	0.736433532

CTD-2062F14	0.024	0.477389902	0.736433532
OR4H6P	0.024	0.477517731	0.736559154
NAMPTP2	0.024	0.477570919	0.736593483
RP4-743O11	0.024	0.477670139	0.736689557
RP11-29B9	0.024	0.477714576	0.736689557
SNRPEP3	0.024	0.477760047	0.736718181
RN7SL191P	0.024	0.477959052	0.73697733
TCEAL3	0.024	0.478195411	0.737187781
AC243654	0.024	0.478283638	0.73728686
RP11-685A21	0.024	0.478387891	0.737328244
MIR4721	0.024	0.478420109	0.73735404
PPA2	0.024	0.478436462	0.737355382
CHADL	0.024	0.478452399	0.737356083
MTND4P4	0.024	0.478717925	0.737622084
USP17L3	0.024	0.478740045	0.737632303
MRPS22	0.024	0.478878051	0.737786588
C10orf99	0.024	0.478902143	0.737786588
RP11-131K5	0.024	0.479042119	0.737869132
MRPL45P1	0.024	0.479083938	0.737869132
SNX18P27	0.024	0.479100375	0.737869132
TSC22D3	0.024	0.479101983	0.737869132
ZP2	0.024	0.479151899	0.737884939
USP43	0.024	0.479215667	0.737935419
AL139429	0.024	0.479440587	0.73815966
SYNE4	0.024	0.479454282	0.73815966
RN7SKP228	0.024	0.479600998	0.738298327
PPP1R7	0.024	0.479660025	0.738306057
BNIP3P18	0.024	0.479682017	0.738306057
TMEM38B	0.024	0.479688887	0.738306057
RNU6-232P	0.024	0.479738111	0.738329598
SNORD15A	0.024	0.479773403	0.738340696
CTD-2332E11	0.024	0.479857504	0.738389845
AC004453	0.024	0.479898081	0.738389845
RP11-49K4	0.024	0.479904112	0.738389845
PIGC	0.024	0.47994733	0.738393626
MIR4757	0.024	0.480037453	0.738435104
COLEC11	0.024	0.480056706	0.738435104
NDUFA3P4	0.024	0.480209587	0.738537909
CTD-2542C24	0.024	0.480211686	0.738537909
RNA5SP186	0.024	0.480232787	0.738546512
GTF2H5	0.024	0.480272784	0.738572491
CLEC14A	0.024	0.480489594	0.738798318
ABCA9-AS1	0.024	0.480561386	0.738837147
RBFOX1	0.024	0.48061372	0.738869871
EIF4A2P5	0.024	0.4806626	0.738869871
GATA1	0.024	0.480695796	0.738876834
FBXL13	0.024	0.480763571	0.738930124
AC091185	0.024	0.480789101	0.738930124
CASP3P1	0.024	0.480792525	0.738930124
FER1L5	0.024	0.480838706	0.738960203
FAM66E	0.024	0.480913013	0.739002098
PPIAP13	0.024	0.480916938	0.739002098

CH17-140K24	0.024	0.480960212	0.739033363
CYBRD1	0.024	0.481149865	0.739243614
AC004386	0.024	0.481167238	0.739243614
LDHAP3	0.024	0.481186683	0.739249642
CTPS1	0.024	0.481267215	0.73933422
RRS1	0.024	0.481467257	0.739537557
BLVRB	0.024	0.481526801	0.739585403
TCHH	0.024	0.481636542	0.739702157
RNU6-547P	0.024	0.48171138	0.73974553
CTC-470E21	0.024	0.481823715	0.739894182
GRM7	0.024	0.481856211	0.739901098
C2CD4D	0.024	0.481904919	0.73992112
RP11-13J12	0.024	0.481912637	0.73992112
CDY10P	0.024	0.481971454	0.73992112
PPAP2C	0.024	0.482113276	0.739945163
RP11-98L5	0.024	0.482167051	0.739945163
C12orf77	0.024	0.482167578	0.739945163
WDR38	0.024	0.482172304	0.739945163
RFPL4B	0.024	0.482373328	0.740102809
RASSF7	0.024	0.482448043	0.740184677
CTC-205M6	0.024	0.482536949	0.740257054
RP5-1017F8	0.024	0.482550283	0.740257054
RN7SKP49	0.024	0.482604731	0.740259234
RP11-136C24	0.024	0.482643676	0.740270204
MIR6081	0.024	0.482721373	0.740328631
RN7SL577P	0.024	0.482743947	0.740328631
ASXL3	0.024	0.482899637	0.740379948
LINC01586	0.024	0.483077607	0.740578015
MAS1LP1	0.024	0.483175223	0.740606237
AC104777	0.024	0.483189317	0.740606237
KB-1517D11	0.024	0.483228892	0.74064306
LINC01177	0.024	0.483354057	0.740735064
RP11-1263C18	0.024	0.483365262	0.740735064
FABP7P2	0.024	0.483372724	0.740735064
RNU6-429P	0.024	0.48339019	0.740735064
RN7SKP295	0.024	0.483395172	0.740735064
AC106765	0.024	0.483397789	0.740735064
FAM108A8P	0.024	0.483502244	0.740832396
RPL23AP10	0.024	0.483571943	0.740832396
LARGE-AS1	0.024	0.483574196	0.740832396
ZNF32	0.024	0.48360179	0.740832396
RP11-434E6	0.024	0.483717077	0.740832396
RP11-647P12	0.024	0.483728264	0.740832396
RP11-56I23	0.024	0.483732451	0.740832396
LINC01442	0.024	0.483914854	0.74102671
C17orf107	0.024	0.483932682	0.741030184
RP11-263F14	0.024	0.483955209	0.741039534
CYP4A22	0.024	0.48407212	0.741124561
RNA5SP137	0.024	0.484100992	0.74114494
RNA5-8SP4	0.024	0.484141607	0.741183296
OR5BA1P	0.024	0.484217927	0.741234572
RP11-541G9	0.024	0.484224716	0.741234572

ZNF571-AS1	0.024	0.484336057	0.741309476
OR9K2	0.024	0.484348548	0.741309476
EGLN3P1	0.024	0.484484233	0.741442601
SLC6A20	0.024	0.484497177	0.741442601
COX6CP4	0.024	0.484524814	0.741442601
RN7SKP68	0.024	0.484585818	0.741442601
OR13C7	0.024	0.484591204	0.741442601
AC012066	0.024	0.484628109	0.741475247
RP5-849H19	0.024	0.484846228	0.741718287
RP11-467P22	0.024	0.484849254	0.741718287
RN7SL645P	0.024	0.484894528	0.741741154
FBXW4	0.024	0.48489535	0.741741154
FAM86JP	0.024	0.484989624	0.741813888
RP11-109D9	0.024	0.485056252	0.741863564
CAMK2D	0.024	0.485147795	0.741935845
NTM-IT	0.024	0.485240717	0.741935845
RP4-724P12	0.024	0.485343163	0.742000793
MEF2BNB	0.024	0.485375022	0.742000793
RP11-112L6	0.024	0.485390014	0.742000793
PGAM1P1	0.024	0.485393267	0.742000793
DLG5-AS1	0.024	0.48540783	0.742000793
PRDM16	0.024	0.485445765	0.742025186
DLK1	0.024	0.485454947	0.742025186
PLXND1	0.024	0.485500791	0.742071443
RP11-343P9	0.024	0.485680535	0.742281994
TFDP2	0.024	0.485696313	0.742281994
LINC01098	0.024	0.485752287	0.742330322
RNU7-54P	0.024	0.48580664	0.742372151
MUL1	0.024	0.48582281	0.742373043
RP11-96L7	0.024	0.485947862	0.742492664
PICK1	0.024	0.485975998	0.742509692
HOPX	0.024	0.486208566	0.742628932
C2CD4C	0.024	0.486266033	0.742645257
AP002490	0.024	0.486317465	0.742676176
RP11-274J16	0.024	0.486372871	0.742736974
IRF3	0.024	0.486664982	0.743034729
STK11	0.024	0.486666009	0.743034729
PALM3	0.024	0.486681389	0.743034729
COA6	0.024	0.486698864	0.743034729
RP4-647C14	0.024	0.48671666	0.743034729
RP3-486D24	0.024	0.486736391	0.743034729
PDLIM1P2	0.024	0.486770668	0.743034729
OR52E3P	0.024	0.486819353	0.743085229
PTGES3L-AARSD1	0.024	0.486846389	0.743092274
MIR4695	0.024	0.486868138	0.743092274
HNRNPA1P33	0.024	0.486874134	0.743092274
GPR88	0.024	0.486911168	0.743092274
HSPA12A	0.024	0.486917582	0.743092274
AC018634	0.024	0.486940577	0.743103556
RAET1M	0.024	0.486987936	0.743120024
MIR938	0.024	0.487011991	0.743120024
RP11-789C17	0.024	0.487071746	0.743184662

PPP1R1C	0.024	0.487882261	0.743978462
RNA5SP110	0.024	0.488042587	0.744189162
ST8SIA6-AS1	0.024	0.488108964	0.744218895
RNU6-707P	0.024	0.488309648	0.74443522
RP11-174M13	0.024	0.48835928	0.74443522
KCNQ1	0.024	0.488359915	0.74443522
B3GALT1	0.024	0.488403133	0.744464624
RP11-673P17	0.024	0.488415739	0.744464624
FGF7	0.024	0.488510791	0.744536751
ELP5	0.024	0.488555715	0.744536751
RP11-214N1	0.024	0.488644109	0.744536751
RN7SL714P	0.024	0.488708161	0.744597059
RP11-298P3	0.024	0.488911874	0.744846966
HOXB8	0.024	0.489176999	0.745028574
GOLGA2P10	0.024	0.489276526	0.745068198
C11orf95	0.024	0.489285793	0.745068198
RP11-455B2	0.024	0.489291747	0.745068198
GOLGA6L16P	0.024	0.489359949	0.745100585
CEL	0.024	0.489519212	0.745211632
ME3	0.024	0.489607103	0.745262472
LRGUK	0.024	0.489631137	0.745275237
RP11-57A19	0.024	0.489782902	0.745452002
GPNMB	0.024	0.489841634	0.745452002
PRAMEF10	0.024	0.489856832	0.745452002
LGALS14	0.024	0.489913628	0.745505625
ADARB1	0.024	0.489923374	0.745505625
TK1	0.024	0.489986229	0.745529811
PTPRA	0.024	0.490078631	0.745598945
RP11-1057N3	0.024	0.490118573	0.745612077
RP11-456H18	0.024	0.490167989	0.745645626
MAF1	0.024	0.49018822	0.745645626
RPL5P22	0.024	0.49025546	0.745701226
RNU6-1229P	0.024	0.490271919	0.745702445
SLC9A3	0.023	0.490319136	0.745738636
SNRPGP19	0.023	0.490343948	0.745740555
CTSA	0.023	0.490509504	0.745788055
RP11-54H7	0.023	0.490707673	0.745974542
CTD-2298J14	0.023	0.490745854	0.745974542
RNA5SP476	0.023	0.490758593	0.745974542
RP11-240L7	0.023	0.490921561	0.746166306
RP11-490G8	0.023	0.491154577	0.746282311
RP11-7K24	0.023	0.491262818	0.746304825
AC006272	0.023	0.491310051	0.746304825
RP11-121P10	0.023	0.491312874	0.746304825
RNU6-796P	0.023	0.491339284	0.746304825
RP11-318C24	0.023	0.491444962	0.746366366
RPL4P5	0.023	0.491576222	0.746470499
GPT2	0.023	0.491733824	0.746552086
FTH1P15	0.023	0.491792432	0.746560788
ANKRD62P1-PARP4P3	0.023	0.491868957	0.74662936
AC024568	0.023	0.491996805	0.746704433
LINC01170	0.023	0.492110236	0.746805194

TSPEAR	0.023	0.492245463	0.746939009
TMPRSS11B	0.023	0.492462959	0.747116592
LINC00858	0.023	0.49261736	0.74726808
FXYD4	0.023	0.492656766	0.747277423
NR1H5P	0.023	0.492806914	0.747457562
AC131011	0.023	0.492875848	0.747492234
RNU6-992P	0.023	0.492879192	0.747492234
EPDR1	0.023	0.493089703	0.747640618
RP11-286O18	0.023	0.49332725	0.747818102
NTN5	0.023	0.493756379	0.748063518
AC020571	0.023	0.49378649	0.748063518
SNORA14A	0.023	0.493787576	0.748063518
OR13Z1P	0.023	0.49385784	0.748117641
AC104169	0.023	0.49394937	0.748170369
HYAL3	0.023	0.494056946	0.748209805
AL513023	0.023	0.494147685	0.748299635
RPL38	0.023	0.494344728	0.748506516
AC107057	0.023	0.494411652	0.748560014
GNAI3	0.023	0.494464836	0.748613285
RP11-340L3	0.023	0.494501322	0.748639896
MIR1286	0.023	0.49459081	0.748690488
TSPAN17	0.023	0.494722361	0.748741362
MARK4	0.023	0.494842864	0.748854924
TFE3	0.023	0.494859093	0.748854924
RPUSD2	0.023	0.494876013	0.748854924
RNA5SP165	0.023	0.495086019	0.749146424
LINC01107	0.023	0.495193798	0.749240581
RP11-208K4	0.023	0.495318153	0.749364311
RNA5SP161	0.023	0.495356319	0.749364311
GS1-21A4	0.023	0.495480284	0.749392489
PSMC1P8	0.023	0.495559614	0.749392489
PYY3	0.023	0.495561686	0.749392489
RSPO1	0.023	0.495649099	0.749490153
RP11-806O11	0.023	0.495682431	0.749490153
RP11-304M2	0.023	0.495693814	0.749490153
MMP17	0.023	0.495713432	0.749490153
RP13-497K6	0.023	0.495782385	0.74953621
MIR28	0.023	0.49578617	0.74953621
OR52N1	0.023	0.495798366	0.74953621
RNA5SP373	0.023	0.495823882	0.749550992
SNORA36C	0.023	0.495942025	0.749682001
RP11-941H19	0.023	0.496174703	0.749879339
CTA-392E5	0.023	0.496296191	0.749994217
OR7E163P	0.023	0.496306039	0.749994217
AC005159	0.023	0.496362329	0.750031684
RP11-469H8	0.023	0.496568943	0.750193191
NKX6-2	0.023	0.496602477	0.750193191
TTTY25P	0.023	0.496649095	0.75022698
WNK2	0.023	0.496741889	0.750281374
KCNH1-IT1	0.023	0.496764304	0.750282029
RP11-21J18	0.023	0.496786859	0.750283912
MIR548H1	0.023	0.496814223	0.750283912

RP1-28O10	0.023	0.496987511	0.750428767
BLCAP	0.023	0.497085537	0.750529193
BOLA2B	0.023	0.497215796	0.750607255
RP11-831A10	0.023	0.497276357	0.750632568
BIN1	0.023	0.497402996	0.750757578
RP11-586L23	0.023	0.497410195	0.750757578
AC022909	0.023	0.497573825	0.750943496
AL607076	0.023	0.497580676	0.750943496
WDR81	0.023	0.497608	0.750960936
CCDC172	0.023	0.497681711	0.75098959
OR4A3P	0.023	0.497705827	0.75098959
RP11-484L7	0.023	0.497759518	0.751046811
LINC01365	0.023	0.497853625	0.751108965
HSPE1P24	0.023	0.497863794	0.751108965
LINC01323	0.023	0.497987667	0.751224459
OR7E91P	0.023	0.498083361	0.751321221
RNU2-5P	0.023	0.498131423	0.751369921
C9orf142	0.023	0.498282196	0.751502142
WWC1	0.023	0.498463473	0.751667301
GSTM1	0.023	0.498510199	0.75167939
PGLYRP3	0.023	0.498544364	0.75168331
VN1R108P	0.023	0.498597477	0.751739594
RP13-258O15	0.023	0.498620124	0.751749942
FOXJ1	0.023	0.49866961	0.751753533
RP11-435O5	0.023	0.49871621	0.751799612
RP4-753D10	0.023	0.498773776	0.751834984
RP11-1K3	0.023	0.498871143	0.751914172
KRTAP6-2	0.023	0.498919444	0.751924901
ZFAT-AS1	0.023	0.498925625	0.751924901
RPL32P35	0.023	0.499030101	0.751928718
HIBADH	0.023	0.499036303	0.751928718
LINC01269	0.023	0.499071063	0.751928718
EIF4A1P7	0.023	0.499111855	0.751928718
NUTM2B	0.023	0.499128538	0.751928718
STK19B	0.023	0.499194944	0.751973862
BEGAIN	0.023	0.499321808	0.75211739
RP1-276J11	0.023	0.499381411	0.75218338
RPL21P136	0.023	0.499531999	0.75233882
CD93	0.023	0.499588658	0.752343773
TTTY16	0.023	0.499623406	0.752343773
FAM109B	0.023	0.499657725	0.75235838
AC005779	0.023	0.499682171	0.75235838
SLC15A4	0.023	0.499688269	0.75235838
AC006126	0.023	0.499835449	0.75235838
FOCAD-AS1	0.023	0.499883158	0.75235838
RPS26P6	0.023	0.499895984	0.75235838
RN7SL587P	0.023	0.499909151	0.75235838
ACVR2A	0.023	0.499984041	0.752359224
AC073850	0.023	0.500057749	0.752369307
NDRG4	0.023	0.500076401	0.752373603
MIR4798	0.023	0.500152119	0.752452698
RNU7-73P	0.023	0.50017637	0.752452698

ACVRL1	0.023	0.50021357	0.752484893
AC018696	0.023	0.500294247	0.752541861
RNU2-50P	0.023	0.500336964	0.752561447
NF1P8	0.023	0.500457787	0.752634734
AL590226	0.023	0.50050653	0.752634734
UPP2	0.023	0.500588603	0.752692451
RP11-1033H12	0.023	0.500617673	0.752712397
FAM195A	0.023	0.500675925	0.752776218
RP11-154D6	0.023	0.500772182	0.752849643
RNU6-296P	0.023	0.500830398	0.752884898
RNF126	0.023	0.500846542	0.752884898
RP1-163M9	0.023	0.500858864	0.752884898
TRIM9	0.023	0.500928908	0.752942659
FOXO1	0.023	0.501009891	0.753006152
MIR762HG	0.023	0.501018581	0.753006152
GDF15	0.023	0.501088448	0.75306363
MPTX1	0.023	0.501159745	0.753123251
RP11-475I24	0.023	0.50121056	0.753128323
AL121865	0.023	0.501435582	0.753323843
SCARNA5	0.023	0.501654387	0.753573133
CTD-2001E22	0.023	0.50173089	0.753573133
AC106876	0.023	0.501733073	0.753573133
RNU6-1026P	0.023	0.501896989	0.753734592
OR6P1	0.023	0.501925597	0.753734592
CAPN15	0.023	0.502215024	0.753968728
ARC	0.023	0.502228943	0.753968728
SLC22A11	0.023	0.502295066	0.753996198
MIR7846	0.023	0.502353702	0.753998157
FREM3	0.023	0.502360296	0.753998157
RP11-555J4	0.023	0.502382425	0.753998157
VPS18	0.023	0.50245345	0.754068078
RP11-139F9	0.023	0.502479117	0.754077652
MIR3189	0.023	0.502491496	0.754077652
KCNMB2-AS1	0.023	0.502531219	0.754096748
RP11-374A4	0.023	0.502578771	0.754101614
ATP6V0CP3	0.023	0.502602066	0.754101614
RP11-275I14	0.023	0.502710041	0.754109182
SEPHS2	0.023	0.502738595	0.754109182
RP11-37N22	0.023	0.502768526	0.754109182
AURKC	0.023	0.502940928	0.754299329
RP11-424N24	0.023	0.502960429	0.754299329
ZBTB45P1	0.023	0.503228559	0.754541826
CTD-2550O8	0.023	0.503347148	0.754648314
AC127383	0.023	0.503362959	0.754648314
KRT18P48	0.023	0.503600416	0.754821465
DUSP1	0.023	0.503614407	0.754821465
RP11-563K23	0.023	0.503622195	0.754821465
GALNT16	0.023	0.503672614	0.754827425
ATOH8	0.023	0.503758711	0.754885194
LINC01515	0.023	0.504088528	0.755209196
SDK2	0.023	0.504101781	0.755209196
AP000281	0.023	0.504177112	0.755225552

RPL7AP38	0.023	0.504263566	0.755225552
PLEKHJ1	0.023	0.504268829	0.755225552
BLOC1S2	0.023	0.50427127	0.755225552
ELF3	0.023	0.504406953	0.755326475
COTL1	0.023	0.504426487	0.755326475
AKAP6	0.023	0.504433812	0.755326475
RBM17P1	0.023	0.504485032	0.755361548
DBIL5P2	0.023	0.504500562	0.755361548
AL118496	0.023	0.504540151	0.755366962
KCNE4	0.023	0.504602313	0.75543628
RP11-469A15	0.023	0.504652118	0.755447724
MIR203A	0.023	0.504673405	0.755447724
RPS6P7	0.023	0.504750307	0.755521196
RP11-692M12	0.023	0.504754214	0.755521196
RP11-520H14	0.023	0.504828772	0.7555268
SLITRK5	0.023	0.504837275	0.7555268
ACSM1	0.023	0.50504935	0.755725452
RP4-790G17	0.023	0.505127334	0.755794651
MORF4L1P5	0.023	0.505211587	0.755873221
RP13-324D24	0.023	0.505462123	0.756084546
NDUFC1	0.023	0.505583206	0.756167923
RPS20P1	0.023	0.505645115	0.756221245
RP11-725M22	0.023	0.505763305	0.75636603
RP11-463C14	0.023	0.505826092	0.756412423
KRTAP19-4	0.023	0.506000795	0.756576228
RNA5SP49	0.023	0.506043134	0.756576228
RP11-983G14	0.023	0.506073755	0.756576228
ERICD	0.023	0.506153002	0.756616208
LINC01257	0.023	0.506172683	0.756621882
WDR4	0.023	0.506235827	0.756692518
RP11-481H12	0.023	0.506297101	0.756760357
AL732325	0.023	0.506454007	0.756908114
RP11-778J15	0.023	0.506457157	0.756908114
GABARAP	0.023	0.506459524	0.756908114
RNU6-666P	0.023	0.506654047	0.757127555
RP3-382I10	0.023	0.506740211	0.757185042
RNA5SP428	0.023	0.506839299	0.757285585
CTD-2114J12	0.023	0.506886435	0.757332253
ARHGAP6	0.023	0.507073911	0.757446028
STX6	0.023	0.507203692	0.757592368
RP11-98N22	0.023	0.507219897	0.757592813
RP11-855C21	0.023	0.507292097	0.757629373
RP11-394B2	0.023	0.507416417	0.757743758
RP5-1031J8	0.023	0.507441349	0.757757231
RP11-577H5	0.023	0.507561622	0.757841789
KCNK16	0.023	0.507661689	0.757923266
CTD-2076M15	0.023	0.507663932	0.757923266
GCNT1P2	0.023	0.507834897	0.758017282
AP001092	0.023	0.507838315	0.758017282
RP11-370B11	0.023	0.508032027	0.758140102
RNU6-1123P	0.023	0.508127997	0.758244574
RP11-400D2	0.023	0.508190992	0.758282289

GPER1	0.023	0.508314013	0.758411531
SNORD42	0.023	0.50832538	0.758411531
FLT1	0.023	0.508399475	0.758462546
MRPS16P1	0.023	0.508421869	0.758462546
RP13-487K5	0.023	0.508565647	0.758603653
RN7SL25P	0.023	0.50870574	0.758717992
RP11-352E6	0.023	0.50877958	0.758740054
RNU4-7P	0.023	0.508781435	0.758740054
SNORD8	0.023	0.508784536	0.758740054
USP32P1	0.023	0.508894099	0.758784649
RNU6-865P	0.022	0.509033092	0.758872743
RNU4-72P	0.022	0.509087635	0.758872743
FAM163B	0.022	0.509194142	0.758994411
RFX4	0.022	0.509325377	0.759108509
RP11-446F17	0.022	0.509388938	0.759165972
PDE8B	0.022	0.509441734	0.759220899
COA4	0.022	0.509680904	0.759407706
CASC17	0.022	0.509694643	0.759407706
CDIPT-AS1	0.022	0.509839634	0.75951758
AP001634	0.022	0.509855083	0.75951758
PHBP12	0.022	0.509911912	0.75951758
RNU7-55P	0.022	0.51011089	0.759742679
RP11-143N13	0.022	0.510174399	0.759768912
FAM170A	0.022	0.51023386	0.759822013
ESX1	0.022	0.51024658	0.759822013
RN7SL127P	0.022	0.510285843	0.759822013
CTTNBP2NL	0.022	0.510597794	0.760182592
RP11-129M16	0.022	0.510664146	0.760223069
GLYCAM1	0.022	0.510672867	0.760223069
HGD	0.022	0.510741035	0.76027702
RP11-3P22	0.022	0.510889991	0.760388221
LINGO1	0.022	0.510912745	0.760390029
LRRC38	0.022	0.510939343	0.760391918
RP11-236F9	0.022	0.510948824	0.760391918
AC023513	0.022	0.511106477	0.760490012
KLF6	0.022	0.511128038	0.760496566
RNA5SP306	0.022	0.511350783	0.760726367
ANAPC11	0.022	0.511517652	0.760857027
RP11-33N16	0.022	0.511581628	0.760857027
RP11-6I2	0.022	0.511603557	0.760857027
TRIL	0.022	0.511629049	0.760857027
HSPE1P14	0.022	0.511647066	0.760857027
MTHFD2P4	0.022	0.511674023	0.760857307
EDA	0.022	0.511690551	0.760858129
MSMO1	0.022	0.511840281	0.760989124
ETFA	0.022	0.511868753	0.760989124
RNU1-101P	0.022	0.511983353	0.761079657
RP11-425I13	0.022	0.512113171	0.761218006
PRG3	0.022	0.512265136	0.761306279
RNU1-42P	0.022	0.512267133	0.761306279
RP1-261G23	0.022	0.512293164	0.761306279
CTD-2130O13	0.022	0.51229565	0.761306279

RPS23	0.022	0.512466512	0.761465153
AC002076	0.022	0.512524868	0.761514849
SSBP3	0.022	0.512703606	0.76168691
RP11-88E18	0.022	0.512718378	0.76168691
RNU6ATAC19P	0.022	0.512769912	0.761725854
ITGA2B	0.022	0.512951314	0.761784763
LINC00703	0.022	0.512956572	0.761784763
RP5-986I17	0.022	0.513035615	0.761784763
RP11-627D16	0.022	0.513038723	0.761784763
DIP2C	0.022	0.513101235	0.761784763
PELO	0.022	0.513135728	0.761784763
OR13C3	0.022	0.513145401	0.761784763
RP4-669H2	0.022	0.513166935	0.761784763
AC091487	0.022	0.513172795	0.761784763
DNAH12	0.022	0.51322828	0.761784763
LINC00323	0.022	0.513235921	0.761784763
RP11-613E4	0.022	0.513369379	0.761927187
CLU	0.022	0.513417694	0.761928841
MIR188	0.022	0.513418488	0.761928841
LINC00301	0.022	0.513550148	0.762035422
SNED1	0.022	0.513554306	0.762035422
OOSP2	0.022	0.513612166	0.762093914
HOXC-AS2	0.022	0.513807981	0.762153849
LINC01183	0.022	0.513832518	0.762153849
FAHD2P1	0.022	0.513874219	0.762153849
PRSS38	0.022	0.513898886	0.762153849
ZMAT5	0.022	0.513903316	0.762153849
CEP112	0.022	0.51391177	0.762153849
RNU6-680P	0.022	0.513922163	0.762153849
AP001464	0.022	0.513948176	0.762168694
RP11-54O7	0.022	0.514000892	0.762199405
H2AFX	0.022	0.514188673	0.762382913
MRPL37	0.022	0.514220384	0.762406196
RP11-235D19	0.022	0.514255465	0.76241074
RPS11	0.022	0.514329268	0.762420616
SNORD115-20	0.022	0.514361117	0.762424977
MARCKS	0.022	0.514460719	0.762548882
RNU6-1332P	0.022	0.514808518	0.762945678
RP11-260O18	0.022	0.514982891	0.762999031
RP11-63E9	0.022	0.514988701	0.762999031
RP11-323K10	0.022	0.515022056	0.763024713
AC092684	0.022	0.515150959	0.763120734
STARD13	0.022	0.515333744	0.76329654
CYP1A1	0.022	0.5154141	0.76336808
RP11-63E16	0.022	0.515643784	0.763637018
DTYMK	0.022	0.515693614	0.763687067
CASC9	0.022	0.515886676	0.763827481
RP11-711G10	0.022	0.515942636	0.763827481
BTF3P14	0.022	0.516065344	0.763912202
AC091114	0.022	0.516085189	0.763912202
BPIFB3	0.022	0.516173282	0.763912202
NACC1	0.022	0.516174349	0.763912202

BOLA2P3	0.022	0.51622026	0.763920654
HDAC2	0.022	0.516259438	0.763954893
ZGPAT	0.022	0.516350536	0.763994749
VENTXP3	0.022	0.516383078	0.764017025
RP11-219A15	0.022	0.516501755	0.764123539
RNU6-574P	0.022	0.516535043	0.764149049
RP11-79C6	0.022	0.5165739	0.764162004
ARL2BPP4	0.022	0.516591934	0.764162004
RN7SKP32	0.022	0.516628668	0.764177766
AC079907	0.022	0.516638386	0.764177766
SNHG5	0.022	0.516650725	0.764177766
GOLGA6C	0.022	0.516768027	0.764272784
RP11-161M6	0.022	0.516795201	0.764272784
RN7SL128P	0.022	0.516874692	0.764316894
SNORA4	0.022	0.516885394	0.764316894
DYNLL1P3	0.022	0.516886332	0.764316894
AC011648	0.022	0.51703851	0.764490178
RNU6-1014P	0.022	0.517087	0.764514406
CDH9	0.022	0.517151337	0.764585792
RP3-337H4	0.022	0.517266924	0.764709206
LINC00311	0.022	0.517440786	0.76489501
MAGEA5	0.022	0.517508521	0.764923913
LINC01510	0.022	0.517554599	0.764960308
RN7SL314P	0.022	0.51765352	0.765067003
RP11-308B16	0.022	0.517829365	0.765161343
NSMF	0.022	0.517835407	0.765161343
VTRNA1-1	0.022	0.517845571	0.765161343
LINC01497	0.022	0.517845876	0.765161343
RP11-710E1	0.022	0.518127975	0.765364465
ZBTB48	0.022	0.51821033	0.765402333
TCF21	0.022	0.518228908	0.765402333
AC008592	0.022	0.518367761	0.765457519
APRT	0.022	0.518391475	0.765468804
PROKR1	0.022	0.518443206	0.765493611
RP11-734E19	0.022	0.518524878	0.765570847
CTC-830E23	0.022	0.518546429	0.765578932
OR10G9	0.022	0.518604631	0.7655874
SSR3	0.022	0.518624677	0.7655874
CTD-3006G17	0.022	0.518657555	0.7655874
ZNF660	0.022	0.518700417	0.765616412
RP11-137L10	0.022	0.518717915	0.765618512
MIR660	0.022	0.51874526	0.765635145
KY	0.022	0.518793007	0.765658161
ARL3	0.022	0.518838109	0.765700998
FSIP2	0.022	0.519114079	0.765858426
FAHD2A	0.022	0.519129483	0.765858426
CICP2	0.022	0.519157031	0.765858426
DHRS1	0.022	0.519174808	0.765858426
RNU6-551P	0.022	0.519262845	0.765858426
GINM1	0.022	0.519362502	0.765858426
RP11-157F20	0.022	0.51936382	0.765858426
TBL1Y	0.022	0.519427939	0.765890876

MESTIT1	0.022	0.519433119	0.765890876
AL356310	0.022	0.519496539	0.765960673
RP11-165M1	0.022	0.51954038	0.7660016
AP003068	0.022	0.519654028	0.766079836
AC002044	0.022	0.519772891	0.766175844
RMRP	0.022	0.519841676	0.766175844
RNU6-446P	0.022	0.519846775	0.766175844
MRGBP	0.022	0.519884333	0.766175844
RP11-383H17	0.022	0.519996529	0.766238826
RP5-860P4	0.022	0.520023103	0.766238826
RP11-236B18	0.022	0.520063124	0.766238826
NFE2L3	0.022	0.520087399	0.766238826
LINC01066	0.022	0.520146078	0.766252185
RP11-231L11	0.022	0.520193058	0.766252185
PTPRT	0.022	0.520197593	0.766252185
CXorf51B	0.022	0.520209086	0.766252185
TSC22D4	0.022	0.520559606	0.766544108
PPIAP26	0.022	0.520575277	0.766544108
RP11-356O9	0.022	0.520587849	0.766544108
RP11-252M24	0.022	0.520591054	0.766544108
C12orf45	0.022	0.520644002	0.766544108
SLC35G5	0.022	0.52066411	0.766544108
FRDAP	0.022	0.520667772	0.766544108
CHCHD2P7	0.022	0.520795518	0.766618084
POLD1	0.022	0.520866729	0.766675641
AC067717	0.022	0.521002946	0.766849821
CXCL3	0.022	0.521029654	0.766849821
SOWAHC	0.022	0.521055092	0.766857974
IL1RL1	0.022	0.521178714	0.766976469
RP11-1018J11	0.022	0.521193674	0.766976469
FUT4	0.022	0.521293523	0.767042963
KLF7	0.022	0.521359882	0.76706951
CYP26B1	0.022	0.521554371	0.767293949
DEFA11P	0.022	0.521746801	0.767471881
LILRA2	0.022	0.521753512	0.767471881
USP9YP35	0.022	0.521831405	0.76750245
RNA5SP385	0.022	0.522092303	0.767815088
TECTB	0.022	0.522124578	0.767815088
AC003658	0.022	0.52224037	0.767866823
PPP1R8P1	0.022	0.522261776	0.76787459
ENTHD1	0.022	0.522300321	0.767907556
RNU7-136P	0.022	0.522401116	0.767986924
RP11-51E20	0.022	0.522437399	0.767986924
RP11-486G15	0.022	0.522460065	0.767986924
RPL30P13	0.022	0.522561113	0.768039186
RP5-907C10	0.022	0.52259949	0.768039186
FGG	0.022	0.522838051	0.768210138
RP11-528A4	0.022	0.522876228	0.768210138
GAPDHP66	0.022	0.522877265	0.768210138
GLIS2-AS1	0.022	0.522994895	0.768312088
C8orf89	0.022	0.5230234	0.768322642
RP11-52B19	0.022	0.523051898	0.768322642

MTMR8	0.022	0.523057357	0.768322642
RP11-368J22	0.022	0.523066608	0.768322642
RP11-227D2	0.022	0.52311504	0.768346389
RP11-265G8	0.022	0.52328014	0.768524565
ECI2	0.022	0.523284757	0.768524565
AC092662	0.022	0.523322921	0.768556915
RNASE13	0.022	0.523407987	0.768563348
SNORD56B	0.022	0.523530394	0.768673945
LCE1F	0.022	0.523547864	0.768673945
AP000237	0.022	0.523656154	0.768772871
FTH1P10	0.022	0.523706695	0.768772871
RN7SL188P	0.022	0.523712092	0.768772871
RP11-744H18	0.022	0.523760651	0.768820456
AC079199	0.022	0.523925813	0.768971691
RP11-389O22	0.022	0.523928263	0.768971691
SQRDL	0.022	0.523997516	0.769002239
CDC42P2	0.022	0.524153205	0.769183322
RN7SL552P	0.022	0.524199337	0.769227317
RPL7A	0.022	0.524396436	0.769374316
OR6C72P	0.022	0.524460651	0.769380144
RP11-368E13	0.022	0.524465025	0.769380144
GAPDHP45	0.022	0.524506678	0.769387787
CIDEA	0.022	0.524547548	0.769387787
SHROOM1	0.022	0.524580469	0.769387787
ERCC8	0.022	0.524656298	0.769447437
RN7SL791P	0.022	0.524697125	0.769459924
RN7SL309P	0.022	0.524760943	0.76951882
RP11-220C2	0.022	0.524784272	0.76951882
RP11-139K4	0.022	0.524785758	0.76951882
OR8X1P	0.022	0.524826522	0.769540435
ARHGEF9-IT1	0.022	0.524832814	0.769540435
TCTN3	0.022	0.524994218	0.76964684
CTD-2026J24	0.022	0.524996697	0.76964684
LY6G6D	0.022	0.525002342	0.76964684
OR51B3P	0.022	0.525115779	0.769765749
AC099342	0.022	0.525181555	0.769791091
ZNF300P1	0.022	0.525226089	0.769832676
RBM20	0.022	0.52540457	0.770002102
SNORD114-31	0.022	0.525432013	0.770016016
TRGJ1	0.022	0.525593156	0.77010999
AC005753	0.022	0.525684845	0.770173253
RP3-483K16	0.022	0.525718255	0.77019851
LINC01049	0.022	0.525805065	0.7702783
RNU6-8	0.022	0.526011163	0.770508366
RP3-406A7	0.022	0.526026823	0.770508366
ZIM2	0.022	0.52610014	0.770566449
MTRNR2L2	0.022	0.526115015	0.770566449
RP11-595B24	0.022	0.526150423	0.770577415
APOBEC4	0.022	0.52615486	0.770577415
MRPL14	0.022	0.526252866	0.770627325
RN7SL498P	0.022	0.526253661	0.770627325
RP11-49I11	0.022	0.526350572	0.77066729

RN7SL293P	0.022	0.526429423	0.77066729
NR5A1	0.022	0.526457165	0.77066729
MIRLET7C	0.022	0.526480944	0.77066729
RP11-383G10	0.022	0.526500947	0.77066729
MAFF	0.022	0.526507491	0.77066729
KLF8P1	0.022	0.52654112	0.770692827
RP11-157B13	0.022	0.526600704	0.770720101
SCARF1	0.022	0.526624483	0.770720101
TMSB4XP8	0.022	0.526674422	0.770769503
RP11-84N19	0.022	0.526733558	0.770813493
EIF2B2	0.022	0.527032274	0.771174724
AC002395	0.022	0.527100404	0.771250721
SEMA4F	0.022	0.527178589	0.771282267
AL159977	0.022	0.527203413	0.771282267
SH2B1	0.022	0.527216421	0.771282267
CLDN3	0.022	0.527441653	0.77151301
THOP1	0.022	0.527479494	0.771520971
RBPMSLP	0.022	0.527549752	0.771552649
RNU4-81P	0.022	0.527656441	0.771630949
FDX1P1	0.022	0.527670778	0.771630949
C8orf31	0.022	0.527700499	0.771630949
AC016866	0.022	0.527764213	0.771653042
TNNI1	0.022	0.527943298	0.771820415
RPS27P15	0.021	0.527980877	0.771820415
ZFP37	0.021	0.52800727	0.771820415
CUX2	0.021	0.528010347	0.771820415
RBMY1F	0.021	0.528039733	0.771820415
SNORD105B	0.021	0.528041408	0.771820415
CAP2P1	0.021	0.528056947	0.771820415
RP11-506H20	0.021	0.528182607	0.771885328
RP4-651E10	0.021	0.528235461	0.771901441
AC011322	0.021	0.528300178	0.771962711
TNNT1	0.021	0.528352896	0.772016056
UGT1A9	0.021	0.528459891	0.772077647
RYR1	0.021	0.528498506	0.772094399
RPL26P37	0.021	0.528609148	0.772167774
CHRNB1	0.021	0.528640383	0.772175536
AC068483	0.021	0.528739818	0.772265421
RP11-428F18	0.021	0.528766784	0.772265421
RPL7AP72	0.021	0.528800492	0.772267295
VN1R2	0.021	0.528910685	0.772367039
RNU6-1337P	0.021	0.528917442	0.772367039
CREB3L2	0.021	0.528959587	0.77238122
CTSLP8	0.021	0.528991903	0.772385173
RP11-501M7	0.021	0.529040293	0.772428021
RP11-242G20	0.021	0.529121053	0.772455071
LINC00652	0.021	0.529192242	0.772531461
RNU6-1224P	0.021	0.529235202	0.772543394
FTLP3	0.021	0.529252783	0.772543394
RP11-462D18	0.021	0.529265299	0.772543394
OR4N1P	0.021	0.529425316	0.772729598
RPL7P5	0.021	0.529449204	0.772740783

ZNF316	0.021	0.529553216	0.772828274
TTLL2	0.021	0.529704529	0.772863328
RP11-108B14	0.021	0.529731406	0.772863328
GLI3	0.021	0.529756415	0.772863328
POFUT1	0.021	0.52976035	0.772863328
MIR365A	0.021	0.529872391	0.772882925
RP11-225N10	0.021	0.529876642	0.772882925
SPATA31D5P	0.021	0.529895888	0.772882925
CYTL1	0.021	0.529913842	0.772882925
CTD-2194F4	0.021	0.529919833	0.772882925
PPP1R14BP2	0.021	0.529971916	0.772911548
HTR6	0.021	0.5300444	0.77296992
ISX	0.021	0.530215704	0.773099174
FAR2P2	0.021	0.530221737	0.773099174
MIR549A	0.021	0.530346858	0.773195469
RP11-718O11	0.021	0.530361408	0.773195469
PIH1D3	0.021	0.530532027	0.773280376
RNU5B-6P	0.021	0.530659456	0.773351388
AC093160	0.021	0.53066321	0.773351388
DEFA1	0.021	0.53066321	0.773351388
ZNF717	0.021	0.53069744	0.773377607
ATG10	0.021	0.530752589	0.773424117
RP11-84A19	0.021	0.530761833	0.773424117
AC092651	0.021	0.530998482	0.773589586
RNU6ATAC10P	0.021	0.531005328	0.773589586
RP11-799D4	0.021	0.531071364	0.773631498
NCSTNP1	0.021	0.53111225	0.773650695
RPS7P13	0.021	0.531150711	0.773673827
AC005618	0.021	0.531276631	0.773795477
RP11-55C6	0.021	0.531319992	0.773811304
ACYP2	0.021	0.531342579	0.773820536
ZBTB22	0.021	0.531421054	0.773887496
AL033403	0.021	0.531786785	0.774183379
LHFP	0.021	0.5318275	0.774192011
RPSAP53	0.021	0.53183286	0.774192011
OR2A14	0.021	0.531941043	0.774286144
RP11-738O11	0.021	0.532070039	0.774406374
SLC2A13	0.021	0.532109667	0.774433236
AC079325	0.021	0.532121016	0.774433236
MAP2	0.021	0.532306189	0.774601312
EEF1B2P3	0.021	0.532403388	0.774702153
TSPAN11	0.021	0.532441768	0.774710661
ACP6	0.021	0.532585615	0.774868128
MAP2K6	0.021	0.532592072	0.774868128
RP11-445K13	0.021	0.5325988	0.774868128
AC113618	0.021	0.532660474	0.774930961
RNU6-227P	0.021	0.532697256	0.774940353
RPL21P39	0.021	0.532777273	0.774999732
HSF2BP	0.021	0.53278689	0.774999732
RP11-483M24	0.021	0.532917129	0.775095316
MIR5583-1	0.021	0.532951105	0.775120233
RPL21P129	0.021	0.533162539	0.775339465

PQLC2	0.021	0.533179506	0.775339465
RP11-275N1	0.021	0.533212526	0.775339465
RP11-489D6	0.021	0.533259191	0.775339465
RP11-128D14	0.021	0.533297815	0.775339465
RN7SKP186	0.021	0.533314508	0.775339465
DPP10	0.021	0.533329754	0.775339465
TPT1P11	0.021	0.533411405	0.775434497
BX322559	0.021	0.533495468	0.775509359
C6orf223	0.021	0.533619514	0.775547653
RNU6-949P	0.021	0.53366786	0.775570584
MIR548AZ	0.021	0.533735044	0.775610443
RP11-142A22	0.021	0.533744142	0.775610443
SEMA5A	0.021	0.533776245	0.775633427
RP11-91J3	0.021	0.533818443	0.775671079
CTA-126B4	0.021	0.534018731	0.775843759
CTA-31J9	0.021	0.534135972	0.775857829
RP11-1H15	0.021	0.534165424	0.775857829
RP11-451F20	0.021	0.534165858	0.775857829
DNAJB11	0.021	0.534170665	0.775857829
MIMT1	0.021	0.53417185	0.775857829
RP11-629G13	0.021	0.534524821	0.776084187
CXCL14	0.021	0.534526416	0.776084187
HRASLS5	0.021	0.534633164	0.776144879
CFB	0.021	0.534642509	0.776144879
RP11-678G15	0.021	0.534804276	0.776227319
RP11-451B8	0.021	0.534878189	0.776287283
CH507-236L23	0.021	0.53494602	0.776338415
RNMTL1	0.021	0.535021431	0.776400539
NAT16	0.021	0.535126584	0.776482157
BMS1P3	0.021	0.535163413	0.776511939
USP17L4	0.021	0.535293975	0.77664979
RP11-283G6	0.021	0.535299776	0.77664979
RNU6-1118P	0.021	0.535314046	0.77664979
WDR45	0.021	0.535354806	0.77664979
AC006427	0.021	0.535372567	0.77664979
ABCD1P2	0.021	0.535417749	0.776691677
RP11-128M1	0.021	0.535491896	0.776775577
RP6-149D17	0.021	0.535546255	0.776807111
RP11-960B9	0.021	0.535609763	0.776830763
RN7SKP112	0.021	0.535641843	0.776830763
TAS2R2P	0.021	0.535644115	0.776830763
CACNA1C-AS2	0.021	0.535704436	0.776850268
HOOK2	0.021	0.535760608	0.776857775
AC092610	0.021	0.535853087	0.77692091
DEFT1P2	0.021	0.535929903	0.776963872
HMGN1P7	0.021	0.535958007	0.776963872
RP11-476H24	0.021	0.536173205	0.777122502
LINC00507	0.021	0.536350971	0.777193249
TCEB2P4	0.021	0.536434149	0.77726648
RNU6-641P	0.021	0.536506418	0.777347546
BCAR1P1	0.021	0.536528868	0.777356425
ZNF883	0.021	0.536796317	0.777460109

AC012512	0.021	0.536933429	0.77758287
RP11-567C20	0.021	0.537000093	0.777605649
RP11-745H7	0.021	0.537027421	0.777605649
C2orf80	0.021	0.537134258	0.777689415
STAT3	0.021	0.537186814	0.777694583
RP11-330O11	0.021	0.537246266	0.777705529
ABCC6P1	0.021	0.537265314	0.777705529
AC007359	0.021	0.537290442	0.777705529
RAC1P4	0.021	0.537353726	0.777715359
RN7SL369P	0.021	0.537567354	0.777920164
RP11-953B20	0.021	0.537574251	0.777920164
ZBTB8B	0.021	0.537620304	0.777920164
ADCY10	0.021	0.537759469	0.778026982
USP9YP15	0.021	0.537780385	0.778033608
RP11-307C12	0.021	0.537812121	0.77803756
BPGM	0.021	0.537822576	0.77803756
RP11-715H19	0.021	0.537832124	0.77803756
RP13-415G19	0.021	0.537880088	0.77805968
OR10AG1	0.021	0.537979911	0.77810603
ARRDC3-AS1	0.021	0.538001768	0.77810603
POU5F1	0.021	0.538020157	0.77810603
HNMT	0.021	0.538234726	0.778289015
MIR5588	0.021	0.538464834	0.778482255
MIR1249	0.021	0.538537769	0.778482255
CYC1	0.021	0.538547819	0.778482255
ATP5G1P5	0.021	0.538575086	0.778482255
CRAT	0.021	0.538583896	0.778482255
AC092628	0.021	0.538666161	0.778482255
MIR4643	0.021	0.538666171	0.778482255
SLC2A5	0.021	0.538669402	0.778482255
bP-21264C1	0.021	0.538675798	0.778482255
ANO6	0.021	0.538682391	0.778482255
FAM49A	0.021	0.538695741	0.778482255
C8orf59	0.021	0.53870837	0.778482255
HSPB1P1	0.021	0.538752029	0.778517033
AC116345	0.021	0.538860694	0.778579566
RP11-292D4	0.021	0.539045378	0.77870249
PTPRR	0.021	0.539048127	0.77870249
PTOV1	0.021	0.53906022	0.77870249
BDH2P1	0.021	0.539134281	0.778712752
AC011313	0.021	0.539171593	0.778712752
RN7SKP261	0.021	0.539196086	0.778712752
RSU1P3	0.021	0.539205904	0.778712752
RNU4-83P	0.021	0.539398206	0.77881278
ZNF486	0.021	0.539595614	0.778986431
SFTA2	0.021	0.539648581	0.778986431
RNA5SP345	0.021	0.539649055	0.778986431
C20orf62	0.021	0.539695099	0.779028898
RN7SL445P	0.021	0.539826051	0.779090504
SYNC	0.021	0.539828556	0.779090504
OSBPL9	0.021	0.539881652	0.779104285
LARGE	0.021	0.53991091	0.779104285

DSG2	0.021	0.540024338	0.779165536
RPL30P3	0.021	0.540031148	0.779165536
CHST11	0.021	0.54004204	0.779165536
RNU6-981P	0.021	0.540051515	0.779165536
RNA5SP202	0.021	0.540098329	0.779185869
RP11-864G5	0.021	0.540134419	0.779214332
PIAS4	0.021	0.540337952	0.779374248
RP11-83M16	0.021	0.540421758	0.779416379
CNN2P1	0.021	0.540529429	0.779471207
RP4-763G1	0.021	0.540584343	0.779471207
RP11-835E18	0.021	0.540590704	0.779471207
DAZAP1	0.021	0.540637198	0.77949105
FABP5P7	0.021	0.540713301	0.779577175
CTD-2644I21	0.021	0.540793025	0.779611229
RNU6-1327P	0.021	0.540885244	0.77968346
GPR98	0.021	0.540936889	0.779720803
ZNF32-AS3	0.021	0.540943892	0.779720803
ZNF774	0.021	0.541000887	0.779779355
XXbac-BPG13B8	0.021	0.541046918	0.779822103
RNU6-46P	0.021	0.54124623	0.779992238
RNA5SP67	0.021	0.54132148	0.780005389
LINC01117	0.021	0.541413192	0.780090337
SOX7	0.021	0.541609597	0.780312313
SCNN1D	0.021	0.541794483	0.780437851
RNU6-1260P	0.021	0.541801859	0.780437851
SLX1B	0.021	0.541896529	0.780527006
MIR941-4	0.021	0.541914957	0.780529944
SLC35A4	0.021	0.542086893	0.780649568
ADAMTS1	0.021	0.542123449	0.780649568
RP11-19P22	0.021	0.542133626	0.780649568
RPL23AP73	0.021	0.542329954	0.780797096
RP11-32D16	0.021	0.542410977	0.780866536
MYBBP1A	0.021	0.542433326	0.780875106
RNU5D-2P	0.021	0.542511451	0.780940364
PAMR1	0.021	0.542530361	0.780943981
GSTK1	0.021	0.542568381	0.780975106
CYB5A	0.021	0.542678103	0.780991419
BNIPL	0.021	0.542910259	0.781168861
CCDC167	0.021	0.542934889	0.781168861
SOX30	0.021	0.542947239	0.781168861
TP53INP2	0.021	0.542949016	0.781168861
AC013268	0.021	0.54301885	0.781201753
HSPA8P16	0.021	0.543069077	0.781216965
RP11-435M3	0.021	0.543121249	0.781251456
PADI2	0.021	0.543158042	0.781257189
MRPS17	0.021	0.543280505	0.781315525
TMEM229A	0.021	0.54335412	0.781374035
RP11-286B14	0.021	0.543393985	0.781389875
RP11-307O1	0.021	0.543428405	0.781389875
RNU7-161P	0.021	0.543447167	0.781389875
RP11-159M11	0.021	0.543475143	0.78140651
RP11-1017G21	0.021	0.543577662	0.781466559

RP11-396N11	0.021	0.543585815	0.781466559
LYRM4	0.021	0.543609557	0.781466559
ARL10	0.021	0.543615356	0.781466559
RP11-552E10	0.021	0.543667054	0.7814937
AC112719	0.021	0.543738623	0.781541059
HTT-AS	0.021	0.543868004	0.781604132
WASH1	0.021	0.543891577	0.781604132
MIR548AV	0.021	0.543960512	0.78167961
AC092168	0.021	0.544253184	0.781935035
SEC63P1	0.021	0.544356452	0.78205981
CTD-2306A12	0.021	0.544551629	0.782245828
RP11-132M7	0.021	0.544593947	0.782267065
RP11-18M17	0.021	0.544629965	0.782287573
NFE4	0.021	0.544670485	0.782300274
CASC8	0.021	0.544671659	0.782300274
TSSC1-IT1	0.021	0.544778835	0.782407019
CHMP2A	0.021	0.544837127	0.782443548
AC002069	0.021	0.544896104	0.782477159
RP11-7C6	0.021	0.544944427	0.782477159
CYP51A1-AS1	0.021	0.544959106	0.782477159
GPD1	0.021	0.544998245	0.782509765
RP11-63N3	0.021	0.545034623	0.782538405
OR6M1	0.021	0.545148759	0.782641684
KAZN	0.021	0.545425831	0.78288811
BCL7B	0.021	0.545428736	0.78288811
SPATA31B1P	0.021	0.545442747	0.78288811
RP5-1166A24	0.021	0.545621627	0.783074208
RNU6-1092P	0.021	0.545667243	0.783074208
AC239798	0.021	0.545698749	0.783074208
PRR22	0.021	0.545706866	0.783074208
RN7SL504P	0.021	0.54578326	0.783117505
RNU4-4P	0.021	0.54585228	0.783169349
RP11-144C9	0.021	0.545893427	0.783170087
CTD-2350C19	0.021	0.545972408	0.783170087
MIR29A	0.021	0.545990832	0.783170087
RPE65	0.021	0.54601742	0.783170087
RP4-534P7	0.021	0.546024406	0.783170087
GYLTL1B	0.021	0.546042095	0.783170087
CTB-51J22	0.021	0.546129696	0.783170087
DCTN2	0.021	0.546179412	0.783170087
RP11-567C2	0.021	0.546229939	0.783170087
ERMP1	0.021	0.54623536	0.783170087
RNU7-181P	0.021	0.546250518	0.783170087
FAM184B	0.021	0.54625389	0.783170087
RP11-804N13	0.021	0.546256466	0.783170087
MTUS2-AS2	0.021	0.546435994	0.78327536
RNA5SP72	0.021	0.546558558	0.783306106
RP11-174O3	0.021	0.546568351	0.783306106
RP11-555M1	0.021	0.546577249	0.783306106
TBX22	0.021	0.546582765	0.783306106
RP11-23A22	0.021	0.546598034	0.783306106
RNU6-194P	0.021	0.546605464	0.783306106

MLXIP	0.021	0.546656158	0.783331613
AC092470	0.021	0.54673877	0.783355717
ALCAM	0.021	0.546772832	0.783380954
ZNF209P	0.021	0.546915269	0.783502581
FOXN3	0.021	0.546953865	0.783522475
PART1	0.021	0.546975014	0.783529205
ABCF2P2	0.021	0.547020805	0.783547666
RN7SL553P	0.021	0.547069758	0.783570653
RP11-510N19	0.021	0.547162224	0.783627403
RP11-166J5	0.021	0.547177101	0.783627403
AC092431	0.021	0.547240982	0.783627403
SLC36A1	0.021	0.547241107	0.783627403
POLD2P1	0.021	0.547251353	0.783627403
RNU6-78P	0.021	0.54725746	0.783627403
OR5I1	0.021	0.547316745	0.783660755
LINC01228	0.021	0.54732938	0.783660755
NAMPTP1	0.020	0.547346568	0.783660755
RNU6-1082P	0.020	0.54743169	0.783711948
LYG1	0.020	0.547484411	0.783763866
NPM1P28	0.020	0.547677113	0.783969039
MIR2115	0.020	0.547705442	0.783986028
C2orf81	0.020	0.547780522	0.784022807
RP13-204A15	0.020	0.547821718	0.784034646
RNU6-577P	0.020	0.547914891	0.784120868
AC092796	0.020	0.547982417	0.78417038
HMG20B	0.020	0.548086174	0.784224605
KCMF1	0.020	0.548145131	0.78426184
CLIP4	0.020	0.548297996	0.78440986
RP11-452N17	0.020	0.54833896	0.784414487
MIR619	0.020	0.54846593	0.784414487
C19orf18	0.020	0.548484011	0.784416791
EEF1DP5	0.020	0.548598107	0.784487425
C17orf77	0.020	0.548613938	0.784487425
RN7SL557P	0.020	0.548615757	0.784487425
DRD5P1	0.020	0.548658144	0.784500928
RP11-218F4	0.020	0.548789466	0.784641586
ACTBP2	0.020	0.548848848	0.78467747
RNA5SP335	0.020	0.548887652	0.78467747
RBMY1A3P	0.020	0.54889694	0.78467747
RNA5SP422	0.020	0.549168634	0.784924511
GGTLC3	0.020	0.549286056	0.784981746
RP11-263K19	0.020	0.5493078	0.784981746
RN7SL702P	0.020	0.549345982	0.784981746
PKLR	0.020	0.549357015	0.784981746
C16orf59	0.020	0.549445522	0.784995142
LRRC28	0.020	0.549491433	0.785020143
RNU6-271P	0.020	0.549501336	0.785020143
RP11-739P6	0.020	0.549603899	0.785080823
RP11-191L17	0.020	0.549676971	0.785152269
PPIAP28	0.020	0.549690664	0.785152269
AL645922	0.020	0.549970346	0.785410425
ETNK2	0.020	0.549991475	0.785417048

CABP5	0.020	0.550015807	0.785428245
FAM27C	0.020	0.550125604	0.785487629
ZNF436	0.020	0.550139854	0.785487629
THUMPD2	0.020	0.55016574	0.785501041
POM121L2	0.020	0.550187618	0.785508729
SNRPG	0.020	0.550380875	0.785562703
RP11-452C13	0.020	0.550381979	0.785562703
CROT	0.020	0.550441048	0.785564991
RP11-314O13	0.020	0.550441449	0.785564991
TRIM47	0.020	0.55069709	0.785648689
HEPACAM	0.020	0.550698046	0.785648689
GKN1	0.020	0.551079704	0.786010075
RP5-1029F21	0.020	0.55110783	0.786021401
RP1-100J12	0.020	0.551297805	0.786151099
MIR4297	0.020	0.551316999	0.786154931
OR1A1	0.020	0.551392187	0.786192471
SGK1	0.020	0.551545509	0.78631598
RP11-1275H24	0.020	0.551593696	0.786326331
GHET1	0.020	0.551610124	0.786326331
RP11-178D12	0.020	0.551834724	0.78654243
RN7SL67P	0.020	0.551883193	0.78654243
CXorf51A	0.020	0.551886009	0.78654243
LL0XNC01-220B11	0.020	0.551886009	0.78654243
LIMS3L	0.020	0.551886009	0.78654243
OR4A14P	0.020	0.551906789	0.786542941
MIR128-1	0.020	0.551956435	0.786568429
RP4-684O24	0.020	0.551972329	0.786568429
RPF2P1	0.020	0.551997271	0.786568429
PCA3_1	0.020	0.552003342	0.786568429
KCNC3	0.020	0.552048294	0.786603962
RP11-347H15	0.020	0.552075572	0.786603962
RP11-480I12	0.020	0.552125357	0.786624618
RNA5SP369	0.020	0.552173269	0.786669347
AC005725	0.020	0.552305432	0.786810565
KPRP	0.020	0.552347187	0.78682298
RPL5	0.020	0.552558612	0.787074516
OR7E85P	0.020	0.552613453	0.787074516
RP11-121G22	0.020	0.552618622	0.787074516
PLCH1	0.020	0.552648125	0.787076986
SLC25A10	0.020	0.552690521	0.787076986
ORAI2	0.020	0.552875804	0.787222815
RP1-117P20	0.020	0.552907554	0.787244487
PABPC1P11	0.020	0.552990449	0.787338976
RP13-476E20	0.020	0.553081952	0.787398641
HMGN2P16	0.020	0.553109694	0.787404133
NDUFA5P4	0.020	0.553179244	0.787442999
RP11-381P6	0.020	0.553255523	0.787504506
CDRT1	0.020	0.553461349	0.787674987
AP005138	0.020	0.553528425	0.787699113
MAP7D3	0.020	0.553557631	0.787699113
RN7SL797P	0.020	0.553790387	0.787861978
AC010677	0.020	0.553800101	0.787861978

AC073330		0.020	0.55381209	0.787861978
AL590126		0.020	0.553919785	0.787881888
CILP		0.020	0.553930708	0.787881888
DUX4L26		0.020	0.553987314	0.787910347
	2-Mar	0.020	0.554101559	0.788011489
RYR3		0.020	0.554163028	0.788042594
RNF130		0.020	0.554452489	0.788336529
RP11-629O19		0.020	0.554505669	0.788353063
RP11-79L21		0.020	0.554530329	0.788353063
RPS6KA2-AS1		0.020	0.5545614	0.788373704
C5AR1		0.020	0.55462525	0.788440938
DEPDC1-AS1		0.020	0.554682	0.788498079
CNNM1		0.020	0.554758758	0.78856012
ADAP1		0.020	0.554789616	0.788560363
AC079610		0.020	0.554792044	0.788560363
TOR1AIP2		0.020	0.554880046	0.788607812
RPL36P1		0.020	0.554891658	0.788607812
MIR4486		0.020	0.55497029	0.78862941
LINC00640		0.020	0.554991207	0.78862941
RP11-321E2		0.020	0.555034507	0.78862941
RP11-452C8		0.020	0.555060662	0.788631783
SAR1A		0.020	0.55507411	0.788631783
RP11-90C4		0.020	0.555100229	0.788645366
AL671883		0.020	0.555149304	0.788652676
RNU6-861P		0.020	0.555163408	0.788652676
RP11-627G18		0.020	0.55517346	0.788652676
DPPA5P2		0.020	0.555188169	0.788652676
AC005863		0.020	0.555217623	0.788670994
ZNF736P9Y		0.020	0.555239795	0.788678966
LRFN4		0.020	0.555316554	0.788705979
RP11-587D21		0.020	0.555344169	0.788709608
CTC-264K15		0.020	0.555416028	0.788788143
AL391416		0.020	0.555582816	0.788977959
NSUN5		0.020	0.555695704	0.789052487
C10orf71		0.020	0.555739461	0.789071852
CFTR		0.020	0.55574834	0.789071852
RPL24P4		0.020	0.555773381	0.789083882
C20orf24		0.020	0.555893205	0.789193774
MTND1P7		0.020	0.555900492	0.789193774
LINC00390		0.020	0.555946856	0.789224295
RP5-875O13		0.020	0.556000651	0.789265387
ZNF471		0.020	0.556053357	0.789316679
DNM1P49		0.020	0.556076815	0.789326452
MIR3186		0.020	0.556106124	0.78934453
TAF6L		0.020	0.556168812	0.789386458
RP11-215G15		0.020	0.556225666	0.789420102
COX17P1		0.020	0.556344445	0.789476989
FCER1A		0.020	0.55645618	0.78955533
CYP2C8		0.020	0.556577756	0.789660961
RUNX1T1		0.020	0.556710689	0.789802508
RPLP0P1		0.020	0.556765705	0.789833504
PARK2		0.020	0.556984292	0.790096526

RP11-353H3	0.020	0.557064253	0.790186418
TMEM30A	0.020	0.557217235	0.790309269
RN7SL413P	0.020	0.55732821	0.790359095
RP11-139J15	0.020	0.557355833	0.790359095
RN7SKP94	0.020	0.557751736	0.790705349
FKSG63	0.020	0.557762128	0.790705349
CH507-39O4	0.020	0.557788743	0.790719542
SBK3	0.020	0.558058039	0.791010741
CXCL17	0.020	0.558256969	0.791128383
ZNF300	0.020	0.558312889	0.791128383
PSG10P	0.020	0.55847706	0.791271365
MIR4500HG	0.020	0.558496752	0.791275725
CH17-118O6	0.020	0.55872536	0.791411275
RP11-88I21	0.020	0.559055045	0.791607432
INTS5	0.020	0.559079916	0.791607432
RP11-346J10	0.020	0.559175349	0.791695483
RP11-5P4	0.020	0.559415058	0.791900166
AF121898	0.020	0.559538556	0.791979572
OR4M2	0.020	0.559636473	0.792068592
RP11-713D19	0.020	0.559696445	0.792068592
DOK6	0.020	0.559713246	0.792068592
RALYL	0.020	0.559739279	0.792068592
CTB-25B13	0.020	0.559771707	0.792068592
CTD-2589H19	0.020	0.559808659	0.792068592
LUZP4P1	0.020	0.559846025	0.792068592
RP11-309P22	0.020	0.559984364	0.792181523
AK2	0.020	0.560016984	0.792185342
AL450226	0.020	0.560072374	0.792185342
RP3-399L15	0.020	0.56019316	0.792227418
RP11-374A22	0.020	0.560271543	0.792305376
RNU6-777P	0.020	0.560314398	0.792318478
RP11-664D7	0.020	0.560320587	0.792318478
OR9Q1	0.020	0.560391294	0.792345889
RN7SKP279	0.020	0.56044901	0.792365858
CTC-369A16	0.020	0.560464044	0.792365858
RPL21P106	0.020	0.560503054	0.792397488
SLC25A6P3	0.020	0.560546739	0.792435724
GPR39	0.020	0.560822129	0.792613298
MMD2	0.020	0.560872598	0.792614954
AL031587	0.020	0.560935487	0.792653903
RP11-25D10	0.020	0.560950717	0.792653903
AC093763	0.020	0.560995478	0.792676402
JTB	0.020	0.561025115	0.792676402
ZNF846	0.020	0.561113092	0.792690928
AC004593	0.020	0.561313662	0.792808094
TAF6	0.020	0.561342821	0.792808094
CLCN5	0.020	0.561427508	0.792904188
SH3BP5L	0.020	0.561560074	0.792942132
ZMIZ1-AS1	0.020	0.561570386	0.792942132
TSSK1B	0.020	0.561663556	0.792942132
RPS29P20	0.020	0.561692762	0.792942132
DDX11L9	0.020	0.561695684	0.792942132

CFTRP3	0.020	0.561745947	0.792954186
AC012307	0.020	0.561803596	0.792990449
AL109947	0.020	0.561838237	0.792990449
UGGT2	0.020	0.561977756	0.793116858
FNDC8	0.020	0.562013431	0.793143703
RN7SL168P	0.020	0.562102646	0.793152097
RP11-572B2	0.020	0.562178392	0.793188703
MYO1D	0.020	0.562319675	0.7932395
AC015884	0.020	0.562338338	0.7932395
SNORD53	0.020	0.562344929	0.7932395
RP11-706P11	0.020	0.562366017	0.7932395
SLC52A1	0.020	0.562480719	0.793307036
AC004985	0.020	0.562493653	0.793307036
PROX1-AS1	0.020	0.56256224	0.793307036
AC006377	0.020	0.562631556	0.793381291
POLR3GL	0.020	0.562749426	0.793424178
LINC00628	0.020	0.56281576	0.793424178
RP11-706J10	0.020	0.562878538	0.793424178
RP4-663N10	0.020	0.563151666	0.79371521
MIR6879	0.020	0.563238861	0.793802887
RPL7P37	0.020	0.563247207	0.793802887
C18orf12	0.020	0.563458511	0.793936169
RNU2-54P	0.020	0.563667894	0.794019361
AC002978	0.020	0.563706275	0.794019361
BEST2	0.020	0.563719949	0.794019361
OR6K1P	0.020	0.563720318	0.794019361
PIGR	0.020	0.563865757	0.794128746
RP11-237P21	0.020	0.564093875	0.794235824
AC012354	0.020	0.564103502	0.794235824
BECN1P1	0.020	0.564140798	0.794235824
CTD-2050E21	0.020	0.564141254	0.794235824
TBC1D3P7	0.020	0.564163625	0.794235824
E2F6P2	0.020	0.564166101	0.794235824
RP11-244H18	0.020	0.564188095	0.794235824
GAPDHP65	0.020	0.564248337	0.794297152
RP11-6K23	0.020	0.564282256	0.794321422
RP11-6B4	0.020	0.564373053	0.794378798
CFAP45	0.020	0.564782375	0.794640095
NHS	0.020	0.564808964	0.794640095
AC013549	0.020	0.564880204	0.794693374
RP4-788P17	0.020	0.565041901	0.794850418
PMEL	0.020	0.565134718	0.794887569
API5P2	0.020	0.56527708	0.795063832
RN7SL499P	0.020	0.565331764	0.795074496
AC013734	0.020	0.565358303	0.795074496
RNU6ATAC23P	0.020	0.565404141	0.795080692
FAM205CP	0.020	0.5654961	0.795137067
HBA1	0.020	0.565519644	0.795146698
RP11-43P8	0.020	0.565562757	0.795156031
MALL	0.020	0.565656713	0.795187376
LINC00261	0.020	0.565745587	0.795187376
RN7SL547P	0.020	0.565761232	0.795187376

RPL7AP71	0.020	0.565915825	0.79532807
HNRNPA1P38	0.020	0.566193848	0.795565685
RP5-984P4	0.020	0.566216853	0.795565685
DPPA5P1	0.020	0.566222968	0.795565685
RP11-376M2	0.020	0.566276159	0.795589824
RP11-242P2	0.020	0.566323053	0.795589824
RP11-993B23	0.020	0.56633594	0.795589824
UGT2B28	0.020	0.566486032	0.795730262
BAK1	0.020	0.566589521	0.79585182
RP11-321M21	0.020	0.566613538	0.79585182
FAM69B	0.020	0.566643519	0.79585759
MGAM	0.020	0.566849142	0.795981995
GNB2L1	0.020	0.56689357	0.795981995
RNA5SP53	0.020	0.56689622	0.795981995
MKRN5P	0.020	0.566901997	0.795981995
RP11-244G12	0.020	0.566918326	0.795981995
AC007394	0.020	0.566932648	0.795981995
LINC00407	0.020	0.567003261	0.796034203
DYNAP	0.019	0.567090622	0.796086451
APOBEC3B	0.019	0.567186226	0.796126799
SORBS3	0.019	0.567407154	0.796334875
COQ3	0.019	0.56746193	0.7963495
HSPB11	0.019	0.567611836	0.796410533
AC114273	0.019	0.567616558	0.796410533
KDF1	0.019	0.56776139	0.796490205
RN7SKP40	0.019	0.567762875	0.796490205
KRT18P54	0.019	0.567901753	0.796638101
RPL7AP14	0.019	0.568113717	0.796724243
RPL35AP19	0.019	0.568163141	0.796744398
MAGEB17	0.019	0.568334054	0.796843845
MRPS17P9	0.019	0.568349579	0.796843845
RP11-617F23	0.019	0.56841832	0.796913409
RP11-266L9	0.019	0.568600948	0.797059067
TRIM51GP	0.019	0.56863044	0.797059067
RP11-538P18	0.019	0.568653706	0.797059067
ACOT2	0.019	0.568753961	0.797176131
AC067968	0.019	0.568824149	0.797232936
RNU6-406P	0.019	0.568827968	0.797232936
MTUS2	0.019	0.568942098	0.797322504
KC6	0.019	0.569044239	0.797388922
MAL2	0.019	0.569061901	0.797388922
RP11-53L24	0.019	0.569210457	0.797481655
CTC-370J7	0.019	0.56935715	0.797481655
RNU6-207P	0.019	0.569404039	0.797481655
RP11-118H4	0.019	0.569430252	0.797481655
HAUS5	0.019	0.569448512	0.797481655
TRAV31	0.019	0.569460195	0.797481655
CTC-207P7	0.019	0.569490303	0.797481655
AP002802	0.019	0.569511351	0.797481655
SLC4A9	0.019	0.569602253	0.797484373
RN7SL618P	0.019	0.569610167	0.797484373
KRTAP1-1	0.019	0.5696705	0.797545398

RCC2P3	0.019	0.569739859	0.797619055
AP005273	0.019	0.569844602	0.797652862
RN7SKP220	0.019	0.569952425	0.79768986
GPATCH3	0.019	0.569954298	0.79768986
MT1G	0.019	0.57014124	0.797852643
BCL3	0.019	0.570319308	0.798013852
DNAJC17	0.019	0.570361603	0.798013852
COLEC12	0.019	0.570376073	0.798013852
NEDD4	0.019	0.570388688	0.798013852
MROH3P	0.019	0.570396079	0.798013852
LINC01278	0.019	0.570407239	0.798013852
PSMA2	0.019	0.570570503	0.798143631
ADH1A	0.019	0.570583793	0.798143631
SLC16A2	0.019	0.570656597	0.79822082
RP11-115N4	0.019	0.570682354	0.79822082
AC020900	0.019	0.570708734	0.79822082
SLC30A2	0.019	0.570732751	0.79822082
ZNF280A	0.019	0.570756294	0.79822082
CTSF	0.019	0.570797254	0.798231225
RP1-59M18	0.019	0.570855328	0.798286849
RP11-504E21	0.019	0.570897053	0.798286849
GSTM3P2	0.019	0.570904075	0.798286849
AC007126	0.019	0.571036338	0.798401469
XXyac-YRM2039	0.019	0.571129807	0.79842781
PPP1R26P1	0.019	0.571131225	0.79842781
SNORD81	0.019	0.571155763	0.79842781
OR7E46P	0.019	0.57130893	0.798548171
RP11-549A6	0.019	0.571332577	0.798557789
MIR27A	0.019	0.571411605	0.798621373
PPIAP17	0.019	0.571467959	0.798653261
LIMS3	0.019	0.571720385	0.798823275
POM121L9P	0.019	0.571756689	0.798823275
MIRLET7A2	0.019	0.571764569	0.798823275
FLOT1	0.019	0.571848811	0.798904207
GSR	0.019	0.571939826	0.798984486
RP11-220H4	0.019	0.572058618	0.799056685
RPL7AP65	0.019	0.57208113	0.799064695
RNU4-65P	0.019	0.572118493	0.799091264
RP4-704D23	0.019	0.572143105	0.799091264
SLC6A9	0.019	0.572150486	0.799091264
MIR5694	0.019	0.572184571	0.79911198
ACTBP11	0.019	0.572198876	0.79911198
AL008707	0.019	0.572296835	0.799225351
KCNAB1	0.019	0.572340001	0.799253718
MRPL43	0.019	0.572386826	0.799257282
MRPS36P4	0.019	0.572474126	0.799349493
GBA	0.019	0.57248643	0.799349493
TCEAL2	0.019	0.5725888	0.79942212
RP11-262K1	0.019	0.572917153	0.799786771
TEX9	0.019	0.572954083	0.799814881
PDLIM1P1	0.019	0.573079363	0.799872545
ACSM6	0.019	0.573126336	0.799914664

CUEDC1	0.019	0.573257894	0.800051389
TGM6	0.019	0.573285528	0.800066267
RNU1-70P	0.019	0.573374157	0.800079078
RNU6-1004P	0.019	0.573379744	0.800079078
AC107020	0.019	0.573419926	0.800079078
SMIM2-IT1	0.019	0.573420518	0.800079078
BANF1P4	0.019	0.573521261	0.800114154
OR2G1P	0.019	0.573576713	0.800144639
RP11-217B1	0.019	0.573887882	0.800463535
ATP11AUN	0.019	0.573891796	0.800463535
SNRPGP10	0.019	0.573917831	0.800463535
LPO	0.019	0.573937111	0.800463535
HBA2	0.019	0.574013367	0.800532253
SPAG16	0.019	0.574036548	0.800532253
GSPT2	0.019	0.574056272	0.800532253
RP11-485O14	0.019	0.574120538	0.800598433
GS1-69O6	0.019	0.574203679	0.800667485
HBE1	0.019	0.574255849	0.80069958
UBE2Q2P12	0.019	0.574313301	0.80075001
RP11-61K12	0.019	0.574353881	0.800759705
LSP1	0.019	0.574379716	0.800772283
RP11-343N15	0.019	0.574470707	0.800794688
ZNF541	0.019	0.574538306	0.800801782
PAICSP3	0.019	0.57454494	0.800801782
SSX2	0.019	0.574578411	0.800801782
RNU6-660P	0.019	0.574635151	0.800801782
SLC26A2	0.019	0.574777346	0.800896371
RP11-38C18	0.019	0.574788226	0.800896371
MIR876	0.019	0.575013193	0.800994784
GAPDHP70	0.019	0.575018265	0.800994784
MRPL23-AS1	0.019	0.575318377	0.801283435
OR10V3P	0.019	0.575355317	0.801311451
LINC00570	0.019	0.575408708	0.801329454
ZNF395	0.019	0.575504437	0.801398108
OR9G2P	0.019	0.575815639	0.801645683
SNORD31	0.019	0.575830946	0.801645683
RP11-399N22	0.019	0.575851904	0.801651427
RNU6-552P	0.019	0.575930756	0.801723709
OR51P1P	0.019	0.575960406	0.801723709
GRIK1	0.019	0.575994287	0.801723709
RP4-560B9	0.019	0.576035754	0.801723709
ZSCAN16-AS1	0.019	0.57603923	0.801723709
LINGO4	0.019	0.576090709	0.801723709
HMBOX1-IT1	0.019	0.576139748	0.801723709
RPS3AP50	0.019	0.576169956	0.801723709
RP1-223B1	0.019	0.57617316	0.801723709
GRIN3B	0.019	0.576193007	0.801726985
RNU6-1288P	0.019	0.576248489	0.801726985
CCDC120	0.019	0.576271481	0.801726985
KCNH1	0.019	0.576290174	0.801726985
AC010987	0.019	0.576421363	0.801806563
RBMY2QP	0.019	0.576434726	0.801806563

ALG9-IT1	0.019	0.576656086	0.802008932
NEK4P3	0.019	0.57666441	0.802008932
MRPL33	0.019	0.576756256	0.802066404
MIR92B	0.019	0.576785726	0.802083966
FGA	0.019	0.576900793	0.802197135
MIR4458HG	0.019	0.577021657	0.80231835
AP000445	0.019	0.577045786	0.802323192
C2orf82	0.019	0.577103921	0.802323192
SNORD115-9	0.019	0.577106797	0.802323192
RPS17P15	0.019	0.577209522	0.802323192
AC090044	0.019	0.577243337	0.802323192
DDT	0.019	0.577323687	0.802373809
MIR4254	0.019	0.577342127	0.802373809
RBBP9	0.019	0.577347942	0.802373809
PGCP1	0.019	0.577459651	0.802484084
GRID1-AS1	0.019	0.577644424	0.802597249
HOXB6	0.019	0.577662554	0.802597249
RP11-1100L3	0.019	0.577677236	0.802597249
MPND	0.019	0.577721156	0.802619665
LINC01312	0.019	0.577732155	0.802619665
RP11-370K11	0.019	0.577833595	0.802697408
UNC45A	0.019	0.577870847	0.802725743
STAB1	0.019	0.577910793	0.802756461
TRGVB	0.019	0.57792667	0.802756461
RP11-104F15	0.019	0.577957028	0.802775216
CTD-2331D11	0.019	0.578140566	0.802984631
PPBP	0.019	0.578187354	0.802984631
ZFP57	0.019	0.578192095	0.802984631
AC092635	0.019	0.578378413	0.803046436
RP11-61G23	0.019	0.57838767	0.803046436
DLEC1P1	0.019	0.578429678	0.803080407
HTN3	0.019	0.578480729	0.803081053
OFD1P3Y	0.019	0.5785095	0.803097586
WSCD1	0.019	0.578559434	0.803135764
RP11-293B20	0.019	0.578806231	0.803283943
AL354682	0.019	0.578944589	0.803373753
RP11-1041F24	0.019	0.579081185	0.803469661
RP5-1126H10	0.019	0.579122466	0.803503531
METTL5	0.019	0.579286903	0.803661443
OR9K1P	0.019	0.579371737	0.80372397
AC131097	0.019	0.579394925	0.80372397
NDUFB1	0.019	0.579598646	0.803930009
SNORD116-30	0.019	0.57967963	0.804004959
TMEM200B	0.019	0.57969884	0.804004959
AHSA1	0.019	0.579703326	0.804004959
SUMO2P18	0.019	0.579924915	0.804195194
VWA5B2	0.019	0.579943375	0.804197377
LRRC74B	0.019	0.579988047	0.804223507
FRMPD4-AS1	0.019	0.580051533	0.804277107
JKAMPP1	0.019	0.580128152	0.804359927
AC090559	0.019	0.580162812	0.80436115
RP4-608O15	0.019	0.580208048	0.804374352

IFT43	0.019	0.580256779	0.804374352
CBWD1	0.019	0.580293435	0.804401754
RNU4-11P	0.019	0.580352917	0.80443738
EEF1A1P21	0.019	0.580398931	0.804452801
AC141002	0.019	0.580448495	0.804452801
UBE2E2-AS1	0.019	0.580634427	0.804663656
RP11-1037D22	0.019	0.58072278	0.804739266
DNAJB5P1	0.019	0.580760524	0.804768154
RIPPLY2	0.019	0.580795755	0.80479356
RP11-560O20	0.019	0.580982941	0.805006096
KCNQ1OT1	0.019	0.581038391	0.805059506
AC004862	0.019	0.581117504	0.805108547
GGTLC1	0.019	0.581121475	0.805108547
VPS37D	0.019	0.581165276	0.805118201
CYP2C61P	0.019	0.581270379	0.805134092
RP1-146I3	0.019	0.581325367	0.805134092
CTD-2044J15	0.019	0.58133317	0.805134092
RP11-810K23	0.019	0.581395644	0.805156301
RN7SL748P	0.019	0.581465216	0.805229235
RP11-416H1	0.019	0.581535204	0.805255914
RN7SL199P	0.019	0.581582559	0.805289258
RNA5SP302	0.019	0.581593101	0.805289258
RNU6-371P	0.019	0.581810118	0.805402427
AP006547	0.019	0.581887506	0.805467175
HSBP1P2	0.019	0.581913822	0.80547575
RNU6ATAC12P	0.019	0.581953496	0.805507255
AC087430	0.019	0.582056528	0.805597884
RP11-130N24	0.019	0.582151418	0.805687547
RNU6-928P	0.019	0.582274592	0.805811186
USF1	0.019	0.582340796	0.805840056
RP11-246E12	0.019	0.582399806	0.805867389
PCDHGA6	0.019	0.58246669	0.805913109
RNU6-1233P	0.019	0.582499475	0.805935058
RP11-276E15	0.019	0.582555408	0.805989031
AC068062	0.019	0.58264633	0.806046266
RP11-567B20	0.019	0.582681397	0.806046266
RP4-741O10	0.019	0.582797488	0.806072675
RP5-866L20	0.019	0.582802036	0.806072675
NPAS1	0.019	0.582841074	0.806098454
AC142472	0.019	0.58287968	0.806098454
RNU6-524P	0.019	0.582919186	0.806106173
RN7SKP282	0.019	0.58307459	0.806135916
CNTNAP3B	0.019	0.583098306	0.806135916
L3MBTL3	0.019	0.583113219	0.806135916
SOCS2-AS1	0.019	0.583134588	0.806135916
OR6C74	0.019	0.583135502	0.806135916
ANKRD30BP2	0.019	0.583302821	0.806216028
AC093874	0.019	0.583329176	0.806216028
RP11-588P8	0.019	0.583330074	0.806216028
RP6-22P16	0.019	0.583342507	0.806216028
NDST1	0.019	0.583378674	0.806222081
OR5K1	0.019	0.583384038	0.806222081

RP5-1100E15	0.019	0.583454665	0.806270011
MIR4533	0.019	0.583503912	0.806270011
RP11-34P1	0.019	0.583554151	0.806270011
RNU4-54P	0.019	0.583695738	0.806403827
RNASE9	0.019	0.583701798	0.806403827
SCMH1	0.019	0.583752205	0.80642278
RN7SKP79	0.019	0.583766313	0.80642278
LINC01207	0.019	0.583858249	0.806502997
RPSAP14	0.019	0.583892059	0.806526308
JOSD1	0.019	0.584001288	0.806630397
DEFB4B	0.019	0.584077716	0.806665778
AC126177	0.019	0.584117956	0.806697961
RSL24D1P6	0.019	0.58415314	0.806723159
CTD-3030D20	0.019	0.584342725	0.806914786
CTB-95B16	0.019	0.584431525	0.80695954
C2orf40	0.019	0.584437905	0.80695954
RP11-440G2	0.019	0.584442908	0.80695954
SUGP1	0.019	0.584523997	0.807003907
NKIRAS2	0.019	0.584525874	0.807003907
RPIAP1	0.019	0.58467473	0.807115833
CTD-2008L17	0.019	0.584857663	0.80722798
METTL21C	0.019	0.585016728	0.807344358
RP11-272L13	0.019	0.585022428	0.807344358
TRIM39-RPP21	0.019	0.58504369	0.807344358
ECI1	0.019	0.585200502	0.807443777
OR7E28P	0.019	0.585320266	0.807562234
SOX5P	0.019	0.585360154	0.807570479
GRM1	0.019	0.585567327	0.807692521
SLC44A5	0.019	0.585623202	0.807695691
OR10H5	0.019	0.585647262	0.807695691
AP1S2P1	0.019	0.585705803	0.807695691
NEU1	0.019	0.585709057	0.807695691
OPA3	0.019	0.585733852	0.807695691
SNORD115-33	0.019	0.585756841	0.807695691
SNX17	0.019	0.585776304	0.807695691
IRX2	0.019	0.585811995	0.807702513
OR7E31P	0.019	0.585869408	0.807734905
AC022716	0.019	0.585904099	0.80775765
RP11-747H7	0.019	0.585995726	0.807792141
RP11-115M14	0.019	0.586069662	0.807866068
RTCB	0.019	0.586155057	0.807912986
RP11-978I15	0.019	0.586168208	0.807912986
RPL31P50	0.019	0.586235813	0.807956722
CTB-77H17	0.019	0.586275487	0.807956722
DBX2	0.019	0.586362561	0.8079938
RNA5SP156	0.019	0.586497929	0.808063436
AP000289	0.019	0.58660372	0.808162435
SLC22A6	0.019	0.586771447	0.808321183
SNORA50	0.019	0.586818751	0.808321183
RP11-31F15	0.019	0.586849724	0.808337676
MIR3689A	0.019	0.586904337	0.808366144
RIC1	0.019	0.587000144	0.808474721

RNU6-435P	0.019	0.587035853	0.808487492
MIR606	0.019	0.587095815	0.808489587
TUBB8P9	0.018	0.587393503	0.808841486
RNA5SP122	0.018	0.587419083	0.808841486
AC010518	0.018	0.587419283	0.808841486
RP11-12P19	0.018	0.58753673	0.808979817
RP11-113I24	0.018	0.587556353	0.808983447
COX7C	0.018	0.587617398	0.80900806
BNIP1	0.018	0.58763621	0.80900806
GAPDHP63	0.018	0.58765904	0.80900806
ZNF518B	0.018	0.587697539	0.809037508
RP4-533D7	0.018	0.587899213	0.809236314
C19orf25	0.018	0.587905628	0.809236314
AC098477	0.018	0.587945671	0.809236314
OR7E106P	0.018	0.587972497	0.809236314
DTNBP1	0.018	0.587977883	0.809236314
CTC-255N20	0.018	0.588111397	0.809373292
GAPDHP36	0.018	0.588190582	0.809449421
NRON	0.018	0.588227592	0.809449421
RP11-362A1	0.018	0.588251692	0.809449421
RNU6-1283P	0.018	0.588336531	0.809519384
KALRN	0.018	0.588406986	0.80956955
IGKV2-36	0.018	0.588496694	0.809600308
LRRC37A7P	0.018	0.588525198	0.809615251
LINC01242	0.018	0.588569732	0.809639868
AC012362	0.018	0.588602767	0.809639868
TOPORS-AS1	0.018	0.588621592	0.809639868
AC001226	0.018	0.588627623	0.809639868
VN1R35P	0.018	0.588749466	0.809751374
MT-CO3	0.018	0.588760164	0.809751374
LINC00559	0.018	0.588883963	0.809804715
NPAP1	0.018	0.588982088	0.80988834
GSTM2P1	0.018	0.589012794	0.80988834
AC013271	0.018	0.589093155	0.809951524
AC090957	0.018	0.589108908	0.809951524
RP11-122M14	0.018	0.589290957	0.810154699
RP11-169K16	0.018	0.589391081	0.810244739
MTND3P1	0.018	0.589452305	0.810275801
RPS26P31	0.018	0.58955725	0.810319458
CTC-265F19	0.018	0.589596362	0.810319458
RP11-109M19	0.018	0.589598557	0.810319458
JRKL-AS1	0.018	0.589677472	0.810381145
RP11-575B7	0.018	0.589809959	0.810516444
ATP10D	0.018	0.58990433	0.810622739
PUS1	0.018	0.589923176	0.810625248
RP3-425P12	0.018	0.589962607	0.810630072
HDAC3	0.018	0.590113155	0.810745954
FLJ42102	0.018	0.590321033	0.81095232
RP11-474N24	0.018	0.590415158	0.810997108
YWHAQ	0.018	0.590443048	0.811012028
RP11-391L3	0.018	0.590531076	0.81108379
RNY4	0.018	0.590546383	0.81108379

RPL4	0.018	0.590616889	0.811153007
RPL13P	0.018	0.590707514	0.811208621
TRPV3	0.018	0.590739818	0.811208621
RNU1-122P	0.018	0.590772458	0.811208621
OR3A1	0.018	0.590825404	0.811208621
MIR4438	0.018	0.590859362	0.811208621
CARD9	0.018	0.590917488	0.811219178
MIR8069-1	0.018	0.590951548	0.811219178
RNU6-386P	0.018	0.591038609	0.811315306
SLC25A39	0.018	0.591112142	0.81134609
INGX	0.018	0.591216259	0.811395463
DEFB113	0.018	0.59124066	0.811396269
LINC00028	0.018	0.59126712	0.811396269
RNU6V	0.018	0.591453662	0.811616443
KRT18P47	0.018	0.591481557	0.811616443
RNU6-98P	0.018	0.591563135	0.811616443
OR14A16	0.018	0.591583541	0.811616443
PEX11G	0.018	0.591665709	0.811616443
ZNF649-AS1	0.018	0.591671656	0.811616443
RNA5SP114	0.018	0.591679625	0.811616443
SLC38A7	0.018	0.591686845	0.811616443
AL365274	0.018	0.591733126	0.811616443
TMA7	0.018	0.591780074	0.811616443
CDY2A	0.018	0.591882615	0.811678472
HIST1H4A	0.018	0.592004538	0.811715693
RP11-797E24	0.018	0.592040886	0.811715693
RP11-375I20	0.018	0.592090403	0.811715693
CTNNA2	0.018	0.59209474	0.811715693
RPS17P14	0.018	0.592096123	0.811715693
AC234771	0.018	0.592276212	0.811884126
XAGE3	0.018	0.592288281	0.811884126
RN7SKP1	0.018	0.592388239	0.811916303
NRSN1	0.018	0.592395383	0.811916303
GNG7	0.018	0.592396992	0.811916303
GCSAML	0.018	0.592513094	0.812005327
RP1-53C18	0.018	0.592590039	0.812048361
PHBP11	0.018	0.592731962	0.812126705
AC007511	0.018	0.592755129	0.812126705
TSSK5P	0.018	0.592860611	0.812154409
ARL2-SNX15	0.018	0.59288887	0.812156088
AC016993	0.018	0.592895942	0.812156088
GLT8D1	0.018	0.593014204	0.812195522
RAB5C	0.018	0.59302705	0.812195522
SRGAP3-AS2	0.018	0.593104764	0.812255243
PCDHGA11	0.018	0.593136433	0.812275257
CNTN4-AS2	0.018	0.593162678	0.812287842
RBMS3-AS1	0.018	0.593187525	0.812298512
DANCR	0.018	0.593247718	0.81234629
MIR2113	0.018	0.593274083	0.81234629
METTL7B	0.018	0.593279076	0.81234629
GOT1	0.018	0.593365624	0.812385308
MIR424	0.018	0.593387367	0.812385308

PRKX	0.018	0.59342496	0.812411074
SNORD114-14	0.018	0.593477727	0.81242046
NMI	0.018	0.593481274	0.81242046
AL133243	0.018	0.593562537	0.812446561
TICAM1	0.018	0.593591642	0.812446561
RGPD6	0.018	0.593620828	0.812446561
BMS1P2	0.018	0.593621888	0.812446561
SCARNA8	0.018	0.593631817	0.812446561
ANKMY2	0.018	0.59367329	0.812446561
PEX5	0.018	0.593787833	0.812525677
ARSEP1	0.018	0.593859927	0.812540254
DUSP12P1	0.018	0.593885998	0.812540254
RFX2	0.018	0.593892934	0.812540254
LINC01375	0.018	0.593953127	0.812599263
RP11-421P23	0.018	0.594165865	0.81274134
SPTSSB	0.018	0.594177279	0.81274134
RP11-367G18	0.018	0.594208288	0.81274134
TMEM259	0.018	0.594210557	0.81274134
CFAP74	0.018	0.594348216	0.812882936
RP11-1026M7	0.018	0.594371742	0.812890473
RP11-219G10	0.018	0.594453043	0.812944939
TCAM1P	0.018	0.594529026	0.812944939
OLIG3	0.018	0.594538196	0.812944939
AC106795	0.018	0.594651329	0.813013371
RAB43P1	0.018	0.594683163	0.813013371
COBLL1	0.018	0.594685624	0.813013371
RNU6-267P	0.018	0.594822633	0.813013371
RP11-29O6	0.018	0.594849795	0.813013371
RP11-1101K5	0.018	0.594851429	0.813013371
NOS2P1	0.018	0.59493969	0.813013371
SUMO2P5	0.018	0.59504402	0.81306408
RP11-588D3	0.018	0.595098776	0.813115569
RNU6-370P	0.018	0.595192698	0.81322057
RNU6-108P	0.018	0.595218423	0.813224004
LINC01159	0.018	0.595273718	0.813261277
CLPP	0.018	0.595400062	0.813370819
HNRNPA1P71	0.018	0.59542221	0.813370819
AC092275	0.018	0.595601127	0.813421285
RP11-321E8	0.018	0.595603157	0.813421285
MYBPH	0.018	0.595752263	0.813506089
LINC01185	0.018	0.595774348	0.813506089
RP11-269C4	0.018	0.595790216	0.813506089
RP11-281N10	0.018	0.595795224	0.813506089
GLUD1P8	0.018	0.595811607	0.813506089
RP1-309F20	0.018	0.595846346	0.81352852
SPAG11A	0.018	0.595920244	0.813536341
MIR135A1	0.018	0.596109712	0.813656529
AKT1S1	0.018	0.596138372	0.81367233
AL121897	0.018	0.596177956	0.813703037
GNL2	0.018	0.596242527	0.813744529
RNA5SP498	0.018	0.596301563	0.813801781
RNU6-855P	0.018	0.596384467	0.813891602

RPL29P12	0.018	0.59654745	0.814032438
DHX32	0.018	0.596686505	0.814118089
TUBB7P	0.018	0.596687428	0.814118089
AP001043	0.018	0.596713134	0.814118089
TNNI3K	0.018	0.596771437	0.814144817
LCT	0.018	0.596792183	0.814144817
DISC2	0.018	0.596823519	0.814164172
XRCC6P3	0.018	0.596906021	0.814230075
RNA5SP162	0.018	0.596954516	0.814238702
OR14I1	0.018	0.59696075	0.814238702
MIR4327	0.018	0.596963634	0.814238702
ZNF92P1Y	0.018	0.597010244	0.8142417
HOXB13-AS1_1	0.018	0.597034217	0.8142417
PYY	0.018	0.597071758	0.814269582
RP1-171K16	0.018	0.597135069	0.81428679
RP13-279N23	0.018	0.597152765	0.81428679
AL157399	0.018	0.597375363	0.814520367
LINC00032	0.018	0.597402391	0.8145339
RNU6-1303P	0.018	0.597432313	0.814551379
HCG9	0.018	0.597538945	0.814650121
RP11-361M4	0.018	0.597603845	0.814668641
MIR4489	0.018	0.597662686	0.814725534
RMI2	0.018	0.597687144	0.814735557
AL359987	0.018	0.597799268	0.814818435
LINC01500	0.018	0.598011861	0.815014906
HIST1H2BG	0.018	0.598056684	0.815029349
CENPVP3	0.018	0.598145641	0.815103932
CAP1P2	0.018	0.59820662	0.815122361
RP11-156K23	0.018	0.598256787	0.815138778
PTGER4P2	0.018	0.598317712	0.815193098
CEP89	0.018	0.598367558	0.815213477
TRIO	0.018	0.598380077	0.815213477
RP11-117L6	0.018	0.598501589	0.815285736
ACAT1	0.018	0.598580781	0.815328317
MTX1P1	0.018	0.598729526	0.815432975
MAGT1	0.018	0.598867282	0.815597267
CYCSP49	0.018	0.599118779	0.815836452
BTBD11	0.018	0.599136005	0.815836452
KLF2P4	0.018	0.599179945	0.815836452
FBXL18	0.018	0.599225211	0.815851437
KRT2	0.018	0.599367044	0.815978112
RP11-365D23	0.018	0.599369649	0.815978112
RP11-330L19	0.018	0.599441124	0.816028766
MIR383	0.018	0.599579981	0.816112225
CTB-83J4	0.018	0.599610728	0.816119689
NLGN3	0.018	0.599766654	0.816242343
RP11-673E1	0.018	0.599769396	0.816242343
SMOC2	0.018	0.599830836	0.816279309
RNU1-31P	0.018	0.599930355	0.816344762
GPR32P1	0.018	0.599950144	0.816348366
CYCSP27	0.018	0.599997934	0.81639007
SMARCB1	0.018	0.60003176	0.816412605

AL591379	0.018	0.600100205	0.816412605
BTBD3	0.018	0.600235373	0.816503202
AC007292	0.018	0.600273364	0.81650824
RN7SL278P	0.018	0.60046874	0.816610738
SHISA9	0.018	0.600520762	0.816658168
SDHCP1	0.018	0.600622149	0.816713106
RP11-1136G4	0.018	0.600697796	0.816713106
RP13-685P2	0.018	0.600737714	0.816743316
MIR6821	0.018	0.600988665	0.817014455
CSPG4P4Y	0.018	0.601106039	0.817017445
CDC42BPG	0.018	0.601269496	0.817093111
RP11-444C12	0.018	0.601310804	0.817125932
OR1M1	0.018	0.601343153	0.817134769
RP11-379F4	0.018	0.601386982	0.817136192
RP5-1048B16	0.018	0.601475379	0.817212919
RNU7-95P	0.018	0.601494927	0.817212919
AC020934	0.018	0.601640092	0.817333829
RN7SL700P	0.018	0.601652565	0.817333829
AL354833	0.018	0.601724682	0.817342665
GPR150	0.018	0.601788331	0.817357617
LINC00492	0.018	0.601902946	0.817405197
ASB18	0.018	0.601911052	0.817405197
RP11-296P7	0.018	0.601985823	0.817471187
MIR519B	0.018	0.602028712	0.817476943
RP4-765H13	0.018	0.602159306	0.817578444
RN7SKP247	0.018	0.602343413	0.817682105
RP11-62I21	0.018	0.602346686	0.817682105
CICP12	0.018	0.602373085	0.817682105
BTBD6	0.018	0.602389653	0.817682105
RNU6-121P	0.018	0.60250954	0.817803445
NDUFB8	0.018	0.602538825	0.817803445
RP5-928E24	0.018	0.602571511	0.817803445
MIR4787	0.018	0.60260933	0.817803445
CCT4P2	0.018	0.602624461	0.817803445
RP4-539M6	0.018	0.602868431	0.817918387
KRT18P36	0.018	0.602873479	0.817918387
VN1R66P	0.018	0.602875542	0.817918387
RP11-323D18	0.018	0.602890018	0.817918387
AC005264	0.018	0.602955628	0.817960798
GDI2	0.018	0.603174893	0.818162717
RNU7-77P	0.018	0.603190745	0.818162717
LINC01548	0.018	0.603205884	0.818162717
DUXAP6	0.018	0.603216903	0.818162717
TMEM207	0.018	0.603287277	0.818200961
RBPJP6	0.018	0.603337189	0.818223917
RP4-800G7	0.018	0.603623862	0.81841848
RP5-908M14	0.018	0.603712226	0.8184976
IRF2BP1	0.018	0.603729747	0.818498055
AC026241	0.018	0.603813749	0.818575909
AC004911	0.018	0.603991271	0.818738338
RN7SL653P	0.018	0.603999155	0.818738338
TMSB15A	0.018	0.604060782	0.8187837

WFDC11	0.018	0.60425256	0.818872253
CCNB1IP1	0.018	0.604290166	0.818884872
ZNF863P	0.018	0.604336407	0.818907468
RP11-61G24	0.018	0.604392183	0.818953213
SNORD113-9	0.018	0.60442442	0.818954019
PCDHA3	0.018	0.604693205	0.819174653
AC010928	0.018	0.604746647	0.819177997
GLRX2	0.018	0.604835187	0.819247418
PRSS58	0.018	0.604850126	0.819247418
SLC25A5P8	0.018	0.604897114	0.819287762
CASC19	0.018	0.6049639	0.819308319
MIR8082	0.018	0.604987997	0.819314164
AC144838	0.018	0.605002621	0.819314164
RP11-234N17	0.018	0.605107308	0.819432635
LINC00347	0.018	0.605142025	0.819456349
LINC01487	0.018	0.605214861	0.819484951
RP11-366F6	0.018	0.605249179	0.819484951
PGAM3P	0.018	0.605499715	0.819800862
GJC1	0.018	0.605586	0.819824462
RP11-127B16	0.018	0.605631842	0.819863218
SNORD115-15	0.018	0.605667446	0.819882049
RN7SKP86	0.018	0.605680181	0.819882049
GPKOW	0.018	0.60582975	0.819998259
RNU6-188P	0.018	0.605832053	0.819998259
FUS	0.018	0.60590834	0.820036102
CTD-3080F16	0.018	0.605920823	0.820036102
KCNJ9	0.018	0.605974286	0.820047091
RP11-91P17	0.018	0.606139853	0.820224537
LINC01456	0.018	0.606227688	0.820273476
OSTCP2	0.018	0.606294491	0.820317256
RP11-458D21	0.018	0.606325016	0.820335252
AC009506	0.018	0.606443327	0.8204021
CES1P2	0.018	0.606518937	0.820411173
RNU6-1162P	0.018	0.606617516	0.820497911
STK32C	0.018	0.606668909	0.82050007
PMS2P5	0.018	0.606696393	0.82050007
RNU6-841P	0.018	0.606704241	0.82050007
LINC00604	0.018	0.606722478	0.82050007
LOXHD1	0.018	0.60675631	0.82050834
RP11-713H12	0.018	0.606776122	0.82050834
RPL7P58	0.018	0.606780277	0.82050834
DEFB122	0.018	0.606866362	0.820554855
BRI3P1	0.018	0.606971861	0.820604316
SNX18P26	0.018	0.607128026	0.82074555
RN7SL713P	0.018	0.607243001	0.820863136
AC013402	0.018	0.607283722	0.820863136
KLRG2	0.018	0.607360251	0.8209293
TAAR8	0.017	0.607612904	0.821035701
RP11-404H14	0.017	0.607627154	0.821035701
SIPA1L2	0.017	0.607633492	0.821035701
PRAMEF5	0.017	0.607635719	0.821035701
MED11	0.017	0.607690793	0.821063529

PDLIM1P4	0.017	0.607765728	0.821084168
HCG15	0.017	0.607775028	0.821084168
PAK3	0.017	0.607844371	0.821131265
LINC00693	0.017	0.607946102	0.821201291
RP11-98L12	0.017	0.608072829	0.821266679
OR11G1P	0.017	0.608085528	0.821266679
EHF	0.017	0.608140115	0.821266679
C5orf49	0.017	0.608168005	0.821266679
RPL7P18	0.017	0.608483973	0.821502119
RP11-509E10	0.017	0.608576711	0.821584598
CTD-2036J7	0.017	0.60861558	0.821613782
OR7E37P	0.017	0.608653508	0.821629238
FGD1	0.017	0.608857196	0.821699518
RP13-526J3	0.017	0.608864967	0.821699518
CYP2E1	0.017	0.608871342	0.821699518
GNG5P3	0.017	0.60897033	0.821766587
DCTN6	0.017	0.609025435	0.821817663
RPL34P27	0.017	0.609077973	0.821841987
CCDC85A	0.017	0.609114394	0.821844564
CCT8L1P	0.017	0.609198918	0.821897281
VN1R21P	0.017	0.609205236	0.821897281
GTF3C5	0.017	0.609250679	0.821928511
MIR548X2	0.017	0.609498002	0.82209003
RP11-148O7	0.017	0.609498002	0.82209003
PRG2	0.017	0.609503455	0.82209003
LGR6	0.017	0.609608697	0.822154534
LINC01433	0.017	0.609608995	0.822154534
RP11-444K7	0.017	0.609620328	0.822154534
OR2A20P	0.017	0.609653004	0.822175321
CTC-326K19	0.017	0.609729563	0.822255286
DCHS2	0.017	0.609794264	0.822265896
AC008065	0.017	0.609852626	0.822304824
RP1-257I9	0.017	0.610103522	0.82250341
KIAA0226L	0.017	0.61023737	0.822660568
MAGEE2	0.017	0.61033147	0.822717563
SMIM7	0.017	0.610398506	0.822761353
CRYBG3	0.017	0.610541669	0.822842327
ERVMER61-1	0.017	0.61055275	0.822842327
DEDD	0.017	0.610587989	0.822842327
TIPRL	0.017	0.610596794	0.822842327
NELFCD	0.017	0.610618998	0.822848966
RNU6-1065P	0.017	0.610713725	0.822887305
RNU1-67P	0.017	0.61076238	0.822890804
COPS8P3	0.017	0.610843695	0.8229422
MIR6825	0.017	0.610922283	0.823012324
RP11-461C20	0.017	0.610930306	0.823012324
YJEFN3	0.017	0.611014135	0.823078691
DNAH10	0.017	0.611071194	0.823103372
RP11-1129I3	0.017	0.611084304	0.823103372
U91319	0.017	0.611340231	0.823354952
AP003064	0.017	0.61146478	0.823441034
RBMY2NP	0.017	0.611693662	0.823557132

RP11-149I23	0.017	0.611753127	0.823586396
KANSL1-AS1	0.017	0.611754172	0.823586396
RP1-76B20	0.017	0.611918093	0.823690841
ATP6V0A4	0.017	0.612030162	0.823771669
THRB-AS1	0.017	0.612047812	0.823772146
RP11-383D22	0.017	0.612179271	0.823855952
SLC25A51P2	0.017	0.612231228	0.823879314
SMIM2-AS1	0.017	0.612280897	0.823903367
AC008622	0.017	0.612291064	0.823903367
RNA5SP235	0.017	0.61251813	0.824079123
LINC00521	0.017	0.612561772	0.824097362
IGFBP5	0.017	0.612566293	0.824097362
MATK	0.017	0.612684759	0.82414034
BNIP3P33	0.017	0.61274328	0.824193713
MIR3182	0.017	0.612781989	0.824193713
RNU4-16P	0.017	0.612882216	0.824266266
STRC	0.017	0.612988971	0.824363284
CTD-2055G21	0.017	0.613052233	0.824376492
MIR4650-1	0.017	0.613090125	0.824376492
MIR6769B	0.017	0.613105442	0.824376492
DPRXP2	0.017	0.613129724	0.824376492
AC068831	0.017	0.613140314	0.824376492
RP5-1085F17	0.017	0.613217022	0.824390637
MIR2278	0.017	0.613481847	0.824621003
MIR147A	0.017	0.613485078	0.824621003
RN7SKP185	0.017	0.613509577	0.824621003
CTD-3096P4	0.017	0.613596064	0.824706235
NR2C2AP	0.017	0.613818172	0.824837125
AL160279	0.017	0.613862801	0.824839712
SNRPE	0.017	0.61393214	0.824863068
TMEM136	0.017	0.613967829	0.824887748
HLA-N	0.017	0.614131353	0.825060899
EPAS1	0.017	0.614321312	0.825148572
LDHAL6CP	0.017	0.614331131	0.825148572
RP4-753D4	0.017	0.614369864	0.825148572
COL18A1-AS1	0.017	0.614462262	0.825218747
TNMD	0.017	0.614523501	0.825237616
RP11-624G19	0.017	0.614688071	0.825334572
EIF4EP4	0.017	0.614690128	0.825334572
RN7SKP102	0.017	0.614701687	0.825334572
PPIAP23	0.017	0.614722438	0.825334572
RPL31P53	0.017	0.614902232	0.825434526
RNU6ATAC28P	0.017	0.614905922	0.825434526
RN7SL229P	0.017	0.614929397	0.825434526
MAGEF1	0.017	0.614995903	0.825482559
PPOX	0.017	0.61504314	0.825494143
LINC00684	0.017	0.615121363	0.825573003
RP5-1125M8	0.017	0.615340423	0.825730259
AC003003	0.017	0.61539758	0.82578369
AP1B1P2	0.017	0.615453672	0.82581143
RP1-125I3	0.017	0.615461226	0.82581143
TNFAIP8L1	0.017	0.61547027	0.82581143

KRTAP13-1	0.017	0.61563598	0.82589417
RP11-240B13	0.017	0.615799844	0.825991261
MIR4470	0.017	0.615826038	0.825991261
RP11-443G13	0.017	0.615829754	0.825991261
SLC2A4RG	0.017	0.615976694	0.826124988
ZG16B	0.017	0.616086225	0.82619507
MTND5P21	0.017	0.616153302	0.82619507
SMARCD3	0.017	0.616173088	0.82619507
AC016745	0.017	0.616229235	0.82619507
RP6-65G23	0.017	0.616236509	0.82619507
TOP3BP1	0.017	0.616240859	0.82619507
TRIM51	0.017	0.616241914	0.82619507
MIR4460	0.017	0.61633796	0.826285446
AL357514	0.017	0.616361371	0.826285446
CENPT	0.017	0.616426788	0.826349883
CTD-2299E8	0.017	0.616498354	0.826403497
AC108078	0.017	0.616501486	0.826403497
MEF2BNB-MEF2B	0.017	0.616538662	0.826406812
HAPLN3	0.017	0.616680919	0.826550969
RP11-569A11	0.017	0.616783142	0.826618192
SPATA31C2	0.017	0.61697569	0.826744226
RP11-1O7	0.017	0.616981925	0.826744226
RNU6-1310P	0.017	0.617039498	0.826775641
GAPDHP19	0.017	0.617117349	0.826826728
ALG1L2	0.017	0.617160085	0.826826728
AL138925	0.017	0.617249039	0.826889783
COA3	0.017	0.617340873	0.826889783
SLC34A1	0.017	0.617375068	0.826889783
AMHR2	0.017	0.617377396	0.826889783
RP11-253M7	0.017	0.617385111	0.826889783
RP11-259P15	0.017	0.61743654	0.826911212
AC006987	0.017	0.617517562	0.826911212
RNU6-139P	0.017	0.617517742	0.826911212
RNU7-196P	0.017	0.617602774	0.826948758
RP5-1024C24	0.017	0.617711074	0.827026468
GFOD1	0.017	0.617712906	0.827026468
HARS	0.017	0.617777165	0.827033731
H19_3	0.017	0.617876253	0.827033731
RNA5SP391	0.017	0.617883826	0.827033731
CTB-158D10	0.017	0.618011641	0.827124162
AP000641	0.017	0.618165834	0.827260788
RP5-1057B20	0.017	0.618198882	0.827281769
AC068058	0.017	0.618285816	0.827305123
DLGAP5P1	0.017	0.618323682	0.827332547
MIR5192	0.017	0.618366656	0.827349415
C10orf11	0.017	0.618420693	0.827354494
MIR328	0.017	0.61846148	0.827354494
CTC-507E12	0.017	0.618476703	0.827354494
SLC41A3	0.017	0.61847998	0.827354494
TSR2	0.017	0.618510779	0.827354494
TMEM104	0.017	0.618526564	0.827354494
HSPD1P10	0.017	0.618531172	0.827354494

RP11-152C17	0.017	0.618632985	0.827360649
ZNF295-AS1	0.017	0.618657375	0.827360649
RP11-12G12	0.017	0.618743336	0.827372438
MIR1225	0.017	0.618789288	0.827372438
VARS	0.017	0.618802251	0.827372438
SLC41A2	0.017	0.618804947	0.827372438
RN7SL558P	0.017	0.618808093	0.827372438
RP11-390G14	0.017	0.618928754	0.827454291
VAC14	0.017	0.618935879	0.827454291
METTL12	0.017	0.619113427	0.827563075
RHBDF1P1	0.017	0.619122029	0.827563075
RP11-25G10	0.017	0.619236489	0.827563075
RN7SL819P	0.017	0.619265101	0.827563075
CCNG1P1	0.017	0.619273334	0.827563075
CHCHD4P5	0.017	0.619293989	0.827563075
RP11-349E4	0.017	0.619295264	0.827563075
GNAI2P2	0.017	0.619520691	0.827697968
ACTR3B	0.017	0.619523681	0.827697968
HIST1H1E	0.017	0.619532486	0.827697968
AC107083	0.017	0.619829757	0.827975313
NMBR	0.017	0.619901908	0.827991467
RP4-799D16	0.017	0.61991269	0.827991467
OR2B2	0.017	0.619935766	0.827991467
UBXN2A	0.017	0.62014179	0.828134003
RP11-307C18	0.017	0.620191318	0.828156332
SNORA41	0.017	0.620262743	0.828228487
TMEM30C	0.017	0.620324159	0.828271952
RNU6-647P	0.017	0.620330076	0.828271952
ST3GAL6	0.017	0.620419719	0.828335255
RP11-195E2	0.017	0.620437651	0.828335255
LOC124685	0.017	0.620599367	0.828335255
TRPC3	0.017	0.620617691	0.828335255
RPL5P15	0.017	0.6206244	0.828335255
RPL34P34	0.017	0.620630305	0.828335255
RPS4XP15	0.017	0.620772658	0.828444838
GPR149	0.017	0.620794736	0.828451088
RP11-771F20	0.017	0.620873412	0.828490213
KB-1742H10	0.017	0.620932471	0.828542034
KCNMB3P1	0.017	0.621069195	0.828631609
RP1-6P5	0.017	0.621377775	0.828904216
TAS1R1	0.017	0.621461882	0.828969754
ACR	0.017	0.621523518	0.829007655
TUSC7	0.017	0.621525108	0.829007655
CSF2RBP1	0.017	0.621571507	0.829046325
OR8Q1P	0.017	0.621702655	0.82914663
RP1-300I2	0.017	0.62200768	0.829480001
DUSP22	0.017	0.622018566	0.829480001
CCDC63	0.017	0.62206314	0.82948938
LRRN2	0.017	0.622067195	0.82948938
OR1F12	0.017	0.622224334	0.829584976
RANP2	0.017	0.622310709	0.829584976
RP11-136O12	0.017	0.622324748	0.829584976

AC114813	0.017	0.622383323	0.829584976
ARGFX	0.017	0.622434075	0.829584976
RIT2	0.017	0.622476272	0.829584976
CTD-2587M23	0.017	0.622482462	0.829584976
RP11-281J9	0.017	0.622489471	0.829584976
OR5K4	0.017	0.622552938	0.829635099
OR51V1	0.017	0.622663799	0.829681329
AC000370	0.017	0.622678529	0.829681329
RP11-531A24	0.017	0.622698374	0.829681329
PKD2	0.017	0.622709572	0.829681329
RP11-481G8	0.017	0.622735837	0.829693113
ALG1L5P	0.017	0.6228668	0.829795487
OR6R2P	0.017	0.62288503	0.829795487
RPL34P26	0.017	0.622899789	0.829795487
GTPBP8	0.017	0.623097685	0.829893762
TUBB3P2	0.017	0.623117422	0.829893762
KMO	0.017	0.623182659	0.829893762
SMUG1	0.017	0.623205062	0.829900391
CCT4	0.017	0.623392798	0.829997239
RP11-247I13	0.017	0.623396661	0.829997239
RP11-826N14	0.017	0.623417205	0.829997239
EEF1A1P20	0.017	0.623444162	0.830009926
MIR500B	0.017	0.623598812	0.830120352
DCST2	0.017	0.623610695	0.830120352
RP11-472F19	0.017	0.623631683	0.830120352
ODF2L	0.017	0.623739748	0.830217791
CTD-2026G6	0.017	0.623947667	0.830358306
Z84466	0.017	0.624036462	0.830403861
IFNA17	0.017	0.624100385	0.830442518
UGT1A5	0.017	0.62432079	0.830666171
HIF3A	0.017	0.624493855	0.830764631
RP1-63P18	0.017	0.62449945	0.830764631
RP11-72H11	0.017	0.624550623	0.830786296
RP11-95P13	0.017	0.624610629	0.830836546
RPL7P30	0.017	0.624623289	0.830836546
CHIA	0.017	0.624700147	0.830892518
MTND1P16	0.017	0.624752598	0.830892518
RNU6-1075P	0.017	0.625077408	0.831152487
AL591428	0.017	0.625111795	0.831152487
MAPK6	0.017	0.625211157	0.831183792
CNKSR2	0.017	0.625215935	0.831183792
RP11-456I15	0.017	0.625251502	0.831207874
RP11-882I15	0.017	0.625281073	0.831223983
RNPS1	0.017	0.625450605	0.831379736
AC010649	0.017	0.625526114	0.831442861
RN7SL654P	0.017	0.625562135	0.831458371
PLD5	0.017	0.625612139	0.831501628
MSGN1	0.017	0.625635529	0.831509511
RPL9P32	0.017	0.625671114	0.831511912
RAP1BP2	0.017	0.625687586	0.831511912
MIR5100	0.017	0.625689712	0.831511912
CTD-2007H18	0.017	0.625715918	0.831523536

DSCAM-IT1	0.017	0.625893411	0.831666587
PREP	0.017	0.625955935	0.831703259
RNU6-1107P	0.017	0.625990272	0.831725679
LINC01550	0.017	0.626066882	0.831781058
MIR454	0.017	0.62609884	0.831800314
AC104692	0.017	0.626200867	0.831843045
MEST	0.017	0.626226084	0.831853341
PRKAG3	0.017	0.626266902	0.831868856
LRTM1	0.017	0.62637985	0.831964781
USP17L5	0.017	0.626435801	0.832015893
MIR124-2HG	0.017	0.626511072	0.832092661
MIR1302-6	0.017	0.626537642	0.832095679
CCDC105	0.017	0.626566651	0.832095679
RN7SKP56	0.017	0.626573109	0.832095679
TSACC	0.017	0.626624819	0.832100844
GPIHBP1	0.017	0.626627142	0.832100844
TTC38	0.017	0.626639531	0.832100844
COL20A1	0.017	0.626764724	0.83224388
LYL1	0.017	0.626815321	0.832287861
CCT5P1	0.017	0.626887843	0.83232214
SNORD93	0.017	0.62693573	0.832350645
RGS16	0.017	0.627004686	0.832400061
FAM207BP	0.017	0.627025077	0.832400126
RP11-76K13	0.017	0.627050978	0.832400126
RNU6-145P	0.017	0.627057167	0.832400126
NDUFAF3	0.017	0.627173548	0.832482527
SBDS	0.017	0.627196012	0.832482527
RP11-449L23	0.017	0.627238461	0.832482527
RPS3AP41	0.017	0.627283943	0.832515532
XYLT2	0.017	0.627402084	0.832625921
CXADRP3	0.017	0.627470766	0.832693867
CA3	0.017	0.627613132	0.83281318
BANF2	0.017	0.627669573	0.832864869
MIR4635	0.017	0.627714301	0.832901015
RFWD2	0.017	0.627824777	0.832972703
RP11-69C13	0.017	0.6278375	0.832972703
CCDC94	0.017	0.627855777	0.832972703
RP11-76G10	0.017	0.627937908	0.833035256
SETP6	0.017	0.628002249	0.833068406
FAM58CP	0.017	0.628015371	0.833068406
RNU6-692P	0.017	0.628080595	0.83310852
ALKBH2	0.016	0.628208832	0.83313784
RP11-693L9	0.016	0.628215773	0.83313784
RPL7P56	0.016	0.628305741	0.833198316
PAFAH1B3	0.016	0.628418923	0.833203747
HRAS	0.016	0.628420861	0.833203747
HRK	0.016	0.628432297	0.833203747
MIR5687	0.016	0.628734932	0.83348898
AC012065	0.016	0.628777154	0.833500537
P3H2-AS1	0.016	0.62877865	0.833500537
BACE1	0.016	0.628878746	0.833586821
AL117190	0.016	0.629038987	0.833741467

CHML	0.016	0.629246011	0.833861178
AL732479	0.016	0.629278318	0.833861178
RNA5SP171	0.016	0.629278318	0.833861178
TBC1D3P1	0.016	0.629397935	0.833950075
AC009502	0.016	0.629489133	0.834024506
AC004832	0.016	0.629576527	0.834117093
RP11-1055B8	0.016	0.629605995	0.834118446
MIR6852	0.016	0.629773086	0.834181688
RCBTB2P1	0.016	0.629946268	0.834328445
C17orf50	0.016	0.630272562	0.83459814
RP1-293L6	0.016	0.630461401	0.834784865
KRT17P2	0.016	0.630483683	0.834784865
PSMD10P2	0.016	0.630513819	0.834801559
RP11-40H19	0.016	0.630640494	0.83483958
CAPS	0.016	0.630657357	0.83483958
CNGA2	0.016	0.630684006	0.83483958
MIR1273D	0.016	0.630747746	0.83483958
WI2-2373I1	0.016	0.630757282	0.83483958
RNU7-20P	0.016	0.630845264	0.83483958
CETN1	0.016	0.630879814	0.83483958
CAPN13	0.016	0.630916303	0.83483958
BX571818	0.016	0.630951763	0.83483958
ATP5F1P4	0.016	0.630963225	0.83483958
LGALS7	0.016	0.631020032	0.834891548
AC013480	0.016	0.631051471	0.834909951
SMS	0.016	0.631183513	0.834991866
DIAPH2-AS1	0.016	0.63124265	0.835000519
TMEM179	0.016	0.63126546	0.835005317
MIR1285-2	0.016	0.631316406	0.835005317
SLC30A3	0.016	0.631342137	0.835016161
MIR708	0.016	0.631379246	0.835018864
CCL11	0.016	0.631523703	0.835100592
RP11-13N12	0.016	0.631705405	0.835218292
TSGA10IP	0.016	0.631765745	0.835251697
PIN1P1	0.016	0.631802092	0.835253379
WDR78	0.016	0.631851281	0.83528787
RP11-192H6	0.016	0.632040819	0.83528787
ABHD5	0.016	0.632079766	0.83528787
MS4A15	0.016	0.632089722	0.83528787
RP11-26C10	0.016	0.632126329	0.83528787
OR8I4P	0.016	0.632126329	0.83528787
NOS2P3	0.016	0.632246451	0.835357403
PCDHGB7	0.016	0.632282627	0.835357403
RER1	0.016	0.632297014	0.835357403
SLC39A5	0.016	0.632301728	0.835357403
LINC00948	0.016	0.632356423	0.835360146
CTD-2206G10	0.016	0.632438312	0.835444301
BAGE2	0.016	0.632487107	0.835463265
RP11-698E18	0.016	0.632550711	0.835500935
RP11-789C1	0.016	0.632593832	0.835534719
AC004969	0.016	0.632651148	0.835587249
SHROOM4	0.016	0.632691563	0.835588361

RP11-526K17	0.016	0.632873484	0.835647557
RPS24P8	0.016	0.632888293	0.835647557
GOLT1B	0.016	0.632894126	0.835647557
RP11-341G5	0.016	0.632939232	0.835666477
AC012322	0.016	0.633417087	0.836065615
LINC01351	0.016	0.633568426	0.836186338
RPF2P2	0.016	0.633702457	0.836202969
COMMD9	0.016	0.633755615	0.83621588
ZNF732	0.016	0.633759178	0.83621588
OR5AH1P	0.016	0.634010674	0.836385518
RNA5SP146	0.016	0.634198646	0.836523194
LINC00574	0.016	0.634202857	0.836523194
PCDHAC1	0.016	0.634249313	0.836538134
AC013717	0.016	0.634315683	0.836556172
DGKG	0.016	0.63437238	0.836572879
AC093732	0.016	0.634487088	0.836623281
RP11-65L3	0.016	0.634545198	0.836650356
KRTAP4-16P	0.016	0.634650053	0.836714305
AC079121	0.016	0.634768608	0.836714305
TMLHE-AS1	0.016	0.634782867	0.836714305
MAPKAPK2	0.016	0.634821592	0.836714305
CTC-457E21	0.016	0.634834372	0.836714305
CARD6	0.016	0.634991346	0.836857744
RP4-796I17	0.016	0.635001197	0.836857744
SCb-64M4	0.016	0.635025773	0.836862078
RN7SL745P	0.016	0.63509428	0.836909056
C15orf59-AS1	0.016	0.635197752	0.836954666
C10orf91	0.016	0.635307692	0.83703544
UBE2L5P	0.016	0.635400833	0.837106455
RP11-174E22	0.016	0.635468688	0.837162918
RN7SL479P	0.016	0.635538829	0.837169452
RASSF1	0.016	0.635553307	0.837169452
RP11-449H15	0.016	0.635555129	0.837169452
GAGE12H	0.016	0.635571694	0.837169452
AC108137	0.016	0.635738141	0.837310693
LINC01252	0.016	0.635743791	0.837310693
GRAMD4P6	0.016	0.635851388	0.837413368
RNU6-170P	0.016	0.635864691	0.837413368
RP11-630C16	0.016	0.636059586	0.837580455
MIR6068	0.016	0.636102409	0.837613687
AC006373	0.016	0.636215496	0.837669959
LINC01547	0.016	0.636891484	0.838267975
CACNA1G-AS1	0.016	0.636968746	0.838274763
CTD-2280E9	0.016	0.636974062	0.838274763
GFRAL	0.016	0.637068734	0.838320742
RP11-609L23	0.016	0.637077267	0.838320742
RNU6-1324P	0.016	0.637107476	0.838334514
RPL17P22	0.016	0.637142911	0.838339061
RNU6-1217P	0.016	0.63714866	0.838339061
MAN1B1-AS1	0.016	0.637199503	0.838362958
IGSF8	0.016	0.63729543	0.838466007
RP11-409K15	0.016	0.637341234	0.838503107

RGMB-AS1	0.016	0.637511874	0.83868127
AC092646	0.016	0.637570377	0.838735066
GAPDHP68	0.016	0.637834128	0.838888795
AL021920	0.016	0.637838692	0.838888795
RBMY2OP	0.016	0.637838692	0.838888795
MIR378C	0.016	0.637838692	0.838888795
ATP2B2-IT1	0.016	0.637845759	0.838888795
MIR4266	0.016	0.638088051	0.839068434
DGUOK-AS1	0.016	0.638168024	0.83912726
RNU7-154P	0.016	0.638237013	0.839194805
KRT18P51	0.016	0.63827949	0.83920749
RP11-141O15	0.016	0.638293438	0.83920749
RNA5SP190	0.016	0.638405394	0.839304193
PSD2	0.016	0.639071383	0.839967236
TSPAN8	0.016	0.639324075	0.840198571
RN7SKP165	0.016	0.639335596	0.840198571
SLFN5	0.016	0.639466723	0.840205168
AC004754	0.016	0.639471017	0.840205168
SLC39A10	0.016	0.639478829	0.840205168
RNA5SP471	0.016	0.639554617	0.840214512
IGKV2-19	0.016	0.639582707	0.840214512
LINC00382	0.016	0.639682077	0.840214512
AC073621	0.016	0.639710902	0.840214512
RP11-10O22	0.016	0.639727197	0.840214512
RPL14P5	0.016	0.639753481	0.840214512
BNIP3P24	0.016	0.639804692	0.840249403
FDPSP7	0.016	0.639815333	0.840249403
ARHGAP36	0.016	0.639844925	0.84025433
AC134025	0.016	0.639927302	0.84025433
SEC14L4	0.016	0.63993891	0.84025433
AP000472	0.016	0.639942581	0.84025433
HADHB	0.016	0.639990887	0.840294591
PSMB3	0.016	0.640120589	0.840372217
ITLN2	0.016	0.640253765	0.840454387
COX7B2	0.016	0.640313337	0.84046064
TNFRSF10B	0.016	0.640343686	0.84046064
RP11-443F16	0.016	0.640344848	0.84046064
RP11-5G9	0.016	0.640364409	0.84046064
AC016821	0.016	0.640395814	0.840474877
WAC-AS1	0.016	0.640468337	0.840488666
RP11-486L19	0.016	0.640473999	0.840488666
SNORD104	0.016	0.640546319	0.84056041
SNX33	0.016	0.640652402	0.840676456
C1orf74	0.016	0.640757536	0.840718431
MIR4493	0.016	0.640772651	0.840718431
SULT1D1P	0.016	0.640835993	0.840778376
TBX2-AS1	0.016	0.640865789	0.840794307
CDC20	0.016	0.640904502	0.840800527
MPPE1P1	0.016	0.640940713	0.840823119
RP11-671P2	0.016	0.641266808	0.841053531
RNA5SP444	0.016	0.641292943	0.841053531
RP11-395E19	0.016	0.641420546	0.841105075

TBC1D23	0.016	0.641468651	0.841143508
RN7SKP264	0.016	0.641711376	0.841301138
ABCC9	0.016	0.6418081	0.841381623
PKIG	0.016	0.641998584	0.841515524
AQP7P1	0.016	0.642126098	0.841659503
SRGAP2D	0.016	0.642291148	0.841672833
RP11-445O16	0.016	0.642295318	0.841672833
MS4A2	0.016	0.642354633	0.841704242
AP001505	0.016	0.642377666	0.841711266
TMEM185AP1	0.016	0.642724541	0.841981921
EIF4E2P2	0.016	0.642761914	0.841981921
LINC01299	0.016	0.642783582	0.841981921
BLOC1S1	0.016	0.642796368	0.841981921
RHCG	0.016	0.64286235	0.842022032
RP11-20L24	0.016	0.642887195	0.842024674
RP11-142A23	0.016	0.642966087	0.842088432
RPS3AP30	0.016	0.643020718	0.842113668
RP11-58A18	0.016	0.643039285	0.842114827
RP11-613F7	0.016	0.64307495	0.842132774
VN1R33P	0.016	0.64313978	0.842173932
PRKD3	0.016	0.643260204	0.842260753
DUX4L16	0.016	0.643276854	0.842260753
KLK1	0.016	0.64329219	0.842260753
LINC01556	0.016	0.64338876	0.842273836
LAP3P1	0.016	0.64340333	0.842273836
FGF10-AS1	0.016	0.643408292	0.842273836
RN7SL375P	0.016	0.643428057	0.84227656
LINC00936	0.016	0.643575378	0.842353648
HCG4P8	0.016	0.643725937	0.842425367
RN7SL580P	0.016	0.643743989	0.842425367
MIR30E	0.016	0.643749548	0.842425367
RP11-245J24	0.016	0.64387845	0.842484318
CTD-3138B18	0.016	0.644130318	0.842644919
RP11-252M18	0.016	0.644225639	0.842644919
MIR15B	0.016	0.644233245	0.842644919
RNASEH2A	0.016	0.644287006	0.842644919
RP11-313C4	0.016	0.644385814	0.842650207
NFIB	0.016	0.644459381	0.842677127
ALX1	0.016	0.644475974	0.842677127
MPZL3	0.016	0.644486415	0.842677127
DPT	0.016	0.644635694	0.842722447
RP11-1260E13	0.016	0.6446682	0.842741811
RP1-89D4	0.016	0.644710457	0.842773918
NPBWR1	0.016	0.64480009	0.842844821
AC016577	0.016	0.644833428	0.842865266
RP11-1058G23	0.016	0.644855936	0.842871554
DLG1-AS1	0.016	0.644883175	0.842884024
LINC00375	0.016	0.644928742	0.842897318
RNU6-726P	0.016	0.645030439	0.84297546
RNA5SP256	0.016	0.645178192	0.843069383
GDE1	0.016	0.645257648	0.843069383
RP11-545G3	0.016	0.645283249	0.843069383

SGCZ	0.016	0.64536955	0.843126386
RP11-638F5	0.016	0.6454289	0.843164125
C7orf66	0.016	0.645483523	0.843205895
KRTAP10-2	0.016	0.645505845	0.843207677
AC103740	0.016	0.645648285	0.843216796
RP11-10L7	0.016	0.645676059	0.843216796
RN7SL870P	0.016	0.645725983	0.843216796
SERHL	0.016	0.645731236	0.843216796
RNU7-41P	0.016	0.645731632	0.843216796
RFTN1P1	0.016	0.64575299	0.843216796
RP11-35J6	0.016	0.645759084	0.843216796
RP11-711K1	0.016	0.645775141	0.843216796
AC016819	0.016	0.645850011	0.843271917
PRODH	0.016	0.645950799	0.843356509
MIR4771-2	0.016	0.645988385	0.843356509
RN7SKP53	0.016	0.646232952	0.843461825
KIRREL3	0.016	0.646240654	0.843461825
SLC18B1	0.016	0.646246152	0.843461825
CCDC51	0.016	0.64637324	0.843558347
ZBBX	0.016	0.646415086	0.843585983
AC091038	0.016	0.646661752	0.843737294
RP11-452G18	0.016	0.64666825	0.843737294
LINC01070	0.016	0.64674612	0.843737294
ZNF826P	0.016	0.646772615	0.843737294
VIM2P	0.016	0.64679794	0.843737294
CDC27P2	0.016	0.646811522	0.843737294
MIR6069	0.016	0.64684077	0.843746867
LINC01419	0.016	0.646859972	0.843746867
RPS3AP22	0.016	0.646897936	0.843746867
EEF1A1P25	0.016	0.646904401	0.843746867
CTD-2176I21	0.016	0.647050476	0.843826137
AC025594	0.016	0.647255438	0.843945556
TMEM244	0.016	0.647318686	0.843945556
WBSCR28	0.016	0.647348704	0.843945556
RP11-719C20	0.016	0.647409243	0.843945556
CTNNA1	0.016	0.647435064	0.843945556
KLHL2P1	0.016	0.647533299	0.844008441
STRN4	0.016	0.647605038	0.844022372
PHBP4	0.016	0.647675934	0.844055757
JPH1	0.016	0.64775854	0.84409412
RPP30	0.016	0.647809973	0.844138046
ZFP41	0.016	0.647873579	0.844174736
XXbac-BPG34I8	0.016	0.647931592	0.844221657
RP11-810B23	0.016	0.648113873	0.844320675
CHST4	0.016	0.64816563	0.844320675
AL136992	0.016	0.648210326	0.844320675
GRHPR	0.016	0.648333032	0.844354175
RNA5SP32	0.016	0.648349529	0.844354175
UCHL1-AS1	0.016	0.648370632	0.844354175
ACE3P	0.016	0.648389614	0.844354175
FAM74A4	0.016	0.648496163	0.84440842
RN7SL475P	0.016	0.648595112	0.844467999

FAM83C	0.016	0.648645799	0.844510907
AP001056	0.016	0.648827327	0.844654888
CTD-2102P23	0.016	0.648845322	0.844655228
GTF2F2P1	0.016	0.648897601	0.844677109
P4HA2-AS1	0.015	0.649313209	0.845033342
CEACAMP7	0.015	0.649771767	0.845581256
GJA6P	0.015	0.649787482	0.845581256
HIP1	0.015	0.649869439	0.845610837
MRPL52	0.015	0.649881233	0.845610837
FIG4	0.015	0.649940204	0.845618262
CTA-369K23	0.015	0.650127046	0.845722737
AC138031	0.015	0.650304851	0.845824198
ACTN3	0.015	0.650400395	0.845824198
RP11-64C1	0.015	0.650439959	0.845829459
DUS3L	0.015	0.650554818	0.845916876
AC021218	0.015	0.650631519	0.845916876
MIR6078	0.015	0.65064079	0.845916876
RPL23P4	0.015	0.650824925	0.846088496
RNU6-1093P	0.015	0.650850108	0.846088496
PCNPP5	0.015	0.650870299	0.846088754
RPL30P10	0.015	0.650971672	0.846197435
RN7SKP235	0.015	0.651052163	0.846255871
RP11-342M1	0.015	0.651168882	0.846292427
CDH12P2	0.015	0.651186902	0.846292427
MIR3689B	0.015	0.651320839	0.846397206
TOMM22P3	0.015	0.651347913	0.846409294
MIR4299	0.015	0.651399867	0.846453713
ATP8A2P3	0.015	0.651522859	0.846507992
ACY1	0.015	0.651530507	0.846507992
PFN1P11	0.015	0.651563657	0.84652797
RP13-585F24	0.015	0.651720661	0.846600968
TAF10	0.015	0.651782395	0.846600968
RP11-5O23	0.015	0.651784539	0.846600968
RP11-475C16	0.015	0.651815852	0.846600968
ABHD14A	0.015	0.651860331	0.846600968
AP006261	0.015	0.651868437	0.846600968
AC105235	0.015	0.651904254	0.846600968
AIG1	0.015	0.651971337	0.846641914
NASP	0.015	0.652012671	0.846650278
RP11-78A19	0.015	0.65213896	0.846755566
TNRC18	0.015	0.652139041	0.846755566
CTB-78O21	0.015	0.652428109	0.846922802
RP11-349P19	0.015	0.652444196	0.846922802
CTD-2152M20	0.015	0.652523641	0.846966545
AC008967	0.015	0.652554203	0.846983132
RNF103-CHMP3	0.015	0.652599388	0.846995583
RP4-593H12	0.015	0.652634932	0.846995583
CTA-276O3	0.015	0.652698924	0.84705555
RNA5SP91	0.015	0.652897746	0.847151991
ZSCAN5A	0.015	0.652967192	0.847195936
ARMS2	0.015	0.6532134	0.847414266
MORC4	0.015	0.653259883	0.847414266

RNU6-877P	0.015	0.653312002	0.847414266
ANO4	0.015	0.653316254	0.847414266
AC003090	0.015	0.653365204	0.847431403
ZNF676	0.015	0.653480635	0.847515786
AC074363	0.015	0.653513273	0.847525169
TRPM2-AS	0.015	0.653570724	0.847534958
NDUFS5P1	0.015	0.653584394	0.847534958
ATP5G1P4	0.015	0.653702978	0.847596421
RNA5SP151	0.015	0.653766983	0.847656333
C15orf59	0.015	0.653803257	0.84766641
NOL3	0.015	0.653810351	0.84766641
AC092865	0.015	0.654069405	0.847840715
RN7SL822P	0.015	0.654092843	0.847841324
AC012003	0.015	0.654201983	0.847841324
SSTR1	0.015	0.654217416	0.847841324
OR2A9P	0.015	0.654220981	0.847841324
MIR6829	0.015	0.654221885	0.847841324
FAR2P1	0.015	0.654253313	0.847841324
LINC00448	0.015	0.654294225	0.847855228
SNORA17	0.015	0.65454278	0.84811164
FGFR4	0.015	0.654581006	0.848134544
AC110813	0.015	0.654703498	0.848157544
CTD-3035D6	0.015	0.654705607	0.848157544
LINC01201	0.015	0.654786091	0.84819826
RP11-10G12	0.015	0.654807739	0.84819826
RNU6-765P	0.015	0.65494538	0.848283575
TALDO1	0.015	0.655002164	0.84829758
LEFTY1	0.015	0.655157615	0.848445607
AC006988	0.015	0.65528769	0.848542328
RN7SKP95	0.015	0.655451415	0.848657887
RP11-545I10	0.015	0.655561695	0.848710214
SPTLC1P2	0.015	0.65559554	0.848710214
PMS2L2	0.015	0.655668457	0.848758469
AC117444	0.015	0.655712494	0.848792405
TUBBP9	0.015	0.65589547	0.84898194
RP11-524K22	0.015	0.655932106	0.84898194
NDFIP1P1	0.015	0.655961438	0.84898194
RP11-526F3	0.015	0.655980871	0.84898194
RP11-369C8	0.015	0.655993935	0.84898194
RNU6-331P	0.015	0.656002231	0.84898194
AP001042	0.015	0.656189606	0.849065531
RP11-872D17	0.015	0.656190902	0.849065531
CBLN4	0.015	0.656293658	0.849100837
MIR450A2	0.015	0.656318484	0.849100837
XX-C2158C12	0.015	0.656421055	0.849100837
SZT2-AS1	0.015	0.656447961	0.849100837
DMBX1	0.015	0.656515417	0.849100837
RNA5SP178	0.015	0.656518542	0.849100837
RP11-284P20	0.015	0.656528402	0.849100837
E2F6P1	0.015	0.656535639	0.849100837
GTF2IRD1P1	0.015	0.656539809	0.849100837
NODAL	0.015	0.656557465	0.849100837

VENTXP2	0.015	0.656584212	0.849100837
DDC-AS1	0.015	0.65667057	0.849100837
RNA5SP360	0.015	0.6566796	0.849100837
RANBP20P	0.015	0.656736133	0.849100837
MIR3920	0.015	0.656736142	0.849100837
MIR483	0.015	0.656740452	0.849100837
CHRM3	0.015	0.656753032	0.849100837
ARFGAP3	0.015	0.656831999	0.849133781
RP11-567P19	0.015	0.656870024	0.849150467
RETSAT	0.015	0.656880565	0.849150467
RNU2-21P	0.015	0.657023711	0.849243311
KRTAP5-9	0.015	0.657082098	0.849272683
RCN1P1	0.015	0.657196221	0.849351297
MAGEB1	0.015	0.657214256	0.849351297
CSNK1G2P1	0.015	0.657274029	0.84938245
ABCC3	0.015	0.657383681	0.849407314
SMYD3-IT1	0.015	0.657532756	0.849467636
RP11-301G7	0.015	0.657536143	0.849467636
TAC1	0.015	0.65763387	0.849570844
RP11-166B14	0.015	0.657726943	0.849573047
RP3-324O17	0.015	0.657742872	0.849573047
ADIPOQ	0.015	0.657773213	0.849573047
RP11-168L22	0.015	0.657809827	0.849573047
ABCF2	0.015	0.65783006	0.849573047
RP11-213G6	0.015	0.657861413	0.849573047
IGFALS	0.015	0.657889124	0.849573047
CNPY2	0.015	0.657958071	0.84961474
CTD-2270L9	0.015	0.65797111	0.84961474
AF216667	0.015	0.658106342	0.849663453
RP11-412H9	0.015	0.658218676	0.849684352
AC011406	0.015	0.658221265	0.849684352
RP5-1052M9	0.015	0.658251935	0.849700913
RP5-988G17	0.015	0.658331016	0.849743948
AC097359	0.015	0.658393602	0.849743948
YBEY	0.015	0.658397493	0.849743948
RP11-962G15	0.015	0.658463843	0.849743948
SNORD114-17	0.015	0.658494625	0.849743948
TCEB1	0.015	0.658511319	0.849743948
RP11-396M11	0.015	0.658517215	0.849743948
AC093807	0.015	0.658689226	0.849919855
RN7SL776P	0.015	0.658942046	0.850092552
AC084368	0.015	0.658973365	0.850092552
AC104843	0.015	0.65900852	0.850092552
RND2	0.015	0.659021263	0.850092552
CFAP44-AS1	0.015	0.659036646	0.850092552
ADAMTS13	0.015	0.659171905	0.850137125
GRAMD4P7	0.015	0.659275318	0.850186712
RP11-295D22	0.015	0.659369663	0.850244997
MIR6511A2	0.015	0.65939986	0.850260915
ERICH1-AS1	0.015	0.659540232	0.850395869
RPS10P21	0.015	0.659695264	0.850480642
RP11-330M2	0.015	0.659771905	0.85051038

AC027811	0.015	0.659977943	0.85063784
OFD1P5Y	0.015	0.660032925	0.850662665
CTC-471C19	0.015	0.660083091	0.85068128
C1orf158	0.015	0.660171062	0.850748611
CASP16	0.015	0.660249253	0.850757295
AC114498	0.015	0.660305986	0.850784363
SLC17A3	0.015	0.660350271	0.850818406
GFAP	0.015	0.660413506	0.850830827
MIR648	0.015	0.660456195	0.850851209
HNRNPDLP1	0.015	0.660536578	0.850861097
HIST1H4B	0.015	0.660544191	0.850861097
REXO4	0.015	0.660600325	0.850887377
RP11-541E12	0.015	0.660759092	0.850918111
RPL12L3	0.015	0.660762178	0.850918111
TBRG4	0.015	0.660796231	0.850918111
RP11-301M17	0.015	0.660824728	0.850918111
CTD-3224I3	0.015	0.660825756	0.850918111
SRY	0.015	0.660825764	0.850918111
XKRYP3	0.015	0.660900749	0.850952148
POLE4	0.015	0.660969597	0.851017788
TUSC5	0.015	0.661043257	0.85102486
RP5-1189B24	0.015	0.661046564	0.85102486
TRABD2A	0.015	0.661169901	0.851152293
SELO	0.015	0.661258185	0.851228271
PGAM1P9	0.015	0.661306656	0.851266269
RP11-486I11	0.015	0.661361915	0.851292774
RP11-491H9	0.015	0.661385556	0.851300196
RP11-328J2	0.015	0.661420744	0.851322481
YIPF5	0.015	0.661449021	0.851330044
RP11-154D17	0.015	0.661462943	0.851330044
SMCP	0.015	0.6614881	0.851330044
ASCL4	0.015	0.661608235	0.851353347
HNRNPA1P40	0.015	0.661765592	0.851490203
DUXAP2	0.015	0.661871304	0.851495064
SLC47A2	0.015	0.661943224	0.851495064
RP11-211G3	0.015	0.661979267	0.851495064
KRT125P	0.015	0.662199806	0.851620051
CPS1	0.015	0.662206248	0.851620051
GAPDHP60	0.015	0.662273382	0.851664926
SLC9C2	0.015	0.662276906	0.851664926
SFTPA3P	0.015	0.662459698	0.851712084
CACYBPP1	0.015	0.662501841	0.851712084
RP1-142F18	0.015	0.662544716	0.851712084
GARSP1	0.015	0.66258149	0.851712084
THBD	0.015	0.662649576	0.851712084
AP001992	0.015	0.662652855	0.851712084
CEP131	0.015	0.662720503	0.851712084
MIR489	0.015	0.662745674	0.851712084
WDFY2	0.015	0.662755374	0.851712084
SUFU	0.015	0.662803322	0.851712084
RP11-553L6	0.015	0.662813625	0.851712084
TNFSF12-TNFSF13	0.015	0.662832184	0.851712084

RP11-364L4	0.015	0.66286296	0.851712888
CDC37L1-AS1	0.015	0.662882578	0.851712888
DIRAS2	0.015	0.662886458	0.851712888
RPS26P2	0.015	0.662937661	0.851755698
MAGED4B	0.015	0.663002237	0.851768708
RNU6-485P	0.015	0.663508057	0.85219058
DDX3P2	0.015	0.663516737	0.85219058
AC104037	0.015	0.66380682	0.852246369
VSTM5	0.015	0.663868046	0.852246369
MIR1263	0.015	0.663953559	0.852303135
AC002467	0.015	0.664010404	0.852303135
CNPY3	0.015	0.66402587	0.852303135
TUBB1P2	0.015	0.664096024	0.852370208
DUXAP8	0.015	0.664153965	0.852378717
UQCRBP3	0.015	0.664184759	0.852392216
AL590085	0.015	0.664264869	0.852472055
RP11-661A12	0.015	0.664308642	0.85250526
WI2-2118C23	0.015	0.664585391	0.85269958
MIR320D2	0.015	0.664637619	0.85274362
RP11-761E20	0.015	0.664686174	0.852755378
PTCD2P1	0.015	0.664700499	0.852755378
LINC00501	0.015	0.664824213	0.852825352
ARL2BPP8	0.015	0.664827387	0.852825352
VCX3A	0.015	0.664960744	0.852951397
TMPRSS9	0.015	0.665150498	0.853125865
PPME1	0.015	0.665209627	0.853152214
TAF13	0.015	0.665242169	0.853152214
AC079586	0.015	0.665253107	0.853152214
C19orf66	0.015	0.665289394	0.853152214
MIR3165	0.015	0.665354542	0.853152214
PPIAP22	0.015	0.665379087	0.853152214
CTD-2243E23	0.015	0.665403913	0.853152214
DEFA7P	0.015	0.665445917	0.853155801
KCNK7	0.015	0.665453254	0.853155801
RP11-375A20	0.015	0.665623606	0.85322717
GATM	0.015	0.66572044	0.853305363
ARID5A	0.015	0.665741082	0.853308857
RP5-1052I5	0.015	0.665890945	0.853435362
RPL23AP60	0.015	0.666065279	0.853521849
RP11-502H18	0.015	0.666130844	0.853566126
RPL34P3	0.015	0.666207565	0.853566126
PRICKLE2-AS3	0.015	0.666210629	0.853566126
MARK3P3	0.015	0.666258421	0.853586468
BX664724	0.015	0.666417491	0.853719994
HMGB3P6	0.015	0.666426706	0.853719994
C16orf86	0.015	0.666444653	0.853719994
RP11-676F20	0.015	0.666456199	0.853719994
DDX50P2	0.015	0.666605089	0.853864788
RP1-281E18	0.015	0.66665187	0.853878781
RP11-362B23	0.015	0.666781863	0.854010963
SERPINC1	0.015	0.666803328	0.854010963
RP11-455O6	0.015	0.666906581	0.854109921

RNU1-62P	0.015	0.666956118	0.854126579
SCGB2A1	0.015	0.666972711	0.854126579
CSNK2B	0.015	0.667266917	0.854367864
AC118550	0.015	0.667285095	0.854368171
AL390036	0.015	0.66734108	0.854408972
RNU6-797P	0.015	0.667352841	0.854408972
RP11-337L12	0.015	0.667438436	0.854469662
CTDP1	0.015	0.667454067	0.854469662
IFT22	0.015	0.667605869	0.854549148
AC026799	0.015	0.667747771	0.854638909
RNA5SP251	0.015	0.667904306	0.854747377
NAT8B	0.015	0.667979405	0.854820515
AC022395	0.015	0.668232336	0.854999941
METRN	0.015	0.668292722	0.855014707
TNFAIP1	0.015	0.668359515	0.855021168
RPS3AP6	0.015	0.668481576	0.855021168
RP11-211N11	0.015	0.668588692	0.855021168
DMAP1	0.015	0.668709819	0.855021168
PRDX2P4	0.015	0.668734542	0.855021168
RP11-16L9	0.015	0.668751954	0.855021168
LHX5-AS1	0.015	0.6687833	0.855021168
LRRC4B	0.015	0.668814406	0.855021168
LINC00391	0.015	0.668834992	0.855021168
CCDC81	0.015	0.668938491	0.855067668
TLE1P1	0.015	0.668944534	0.855067668
KRT17P5	0.015	0.668971216	0.855078825
DPYSL2	0.015	0.669099971	0.855198708
BARX1-AS1	0.015	0.669100919	0.855198708
RAD1P1	0.015	0.669198236	0.855208754
CHIT1	0.015	0.669212736	0.855208754
NDUFA2	0.015	0.669216517	0.855208754
GSTM2	0.015	0.669458384	0.855372905
OR7A15P	0.015	0.669470687	0.855372905
FAT4	0.015	0.669544319	0.855409652
IGSF11	0.015	0.6695889	0.855409652
DLGAP1-AS2	0.015	0.669631054	0.855409652
VWA3B	0.015	0.669638805	0.855409652
LINC00207	0.015	0.669881301	0.855622096
RNU1-59P	0.015	0.669947309	0.855683459
ABHD1	0.015	0.670150761	0.855851508
AL591925	0.015	0.670242483	0.855890339
TRABD2B	0.015	0.670253049	0.855890339
MIR5002	0.014	0.670404707	0.855988989
RNU6-266P	0.014	0.670430715	0.855988989
SMAD9	0.014	0.670438139	0.855988989
RP11-728L1	0.014	0.670707297	0.856143971
RNU4-29P	0.014	0.670765755	0.856154811
HIST3H2BB	0.014	0.670806927	0.856161473
RNA5SP475	0.014	0.670921262	0.856284453
RNU7-21P	0.014	0.670986779	0.856322177
RP11-344A5	0.014	0.671047246	0.856353451
KRTAP21-2	0.014	0.671099629	0.856374408

STARD3NL	0.014	0.671276668	0.856490157
E2F3-IT1	0.014	0.671312857	0.856508812
FOXR2	0.014	0.671430881	0.856544348
LBR	0.014	0.671458682	0.856544348
AQP7P2	0.014	0.671597941	0.856627423
PML	0.014	0.67160367	0.856627423
MIR519D	0.014	0.671658537	0.856668929
LRRC16A	0.014	0.671797271	0.856713756
RGAG4	0.014	0.671895276	0.856761696
RPL22P5	0.014	0.67191191	0.856761696
HMGN1P32	0.014	0.671913153	0.856761696
AC019181	0.014	0.671916271	0.856761696
RP11-385M4	0.014	0.671970603	0.856796779
MAPK6PS4	0.014	0.672068145	0.856796779
CTD-2335L22	0.014	0.672078251	0.856796779
FGF7P1	0.014	0.672148952	0.856841041
RP11-118F2	0.014	0.672276402	0.856886481
RNA5SP442	0.014	0.672285383	0.856886481
CC2D2A	0.014	0.672384582	0.85695792
RP11-193F5	0.014	0.672455124	0.857001959
FAM163A	0.014	0.672540329	0.857072818
RPL35AP3	0.014	0.672641184	0.857140307
FAM19A4	0.014	0.672653724	0.857140307
RP11-366H4	0.014	0.672671663	0.857140307
CA14	0.014	0.672695851	0.857148196
BRDT	0.014	0.672793978	0.857180285
DYNLL1P4	0.014	0.672811023	0.857180285
HIST2H3DP1	0.014	0.672905651	0.857212479
Vault	0.014	0.672915772	0.857212479
TOMM22P2	0.014	0.672986435	0.857252017
VDAC1P12	0.014	0.673108576	0.857331592
RP3-325F22	0.014	0.673112424	0.857331592
AC138649	0.014	0.67335223	0.85745693
FAM83E	0.014	0.673452129	0.857537664
GEMIN2	0.014	0.673455723	0.857537664
RN7SKP59	0.014	0.673513561	0.857570655
RNU6-1027P	0.014	0.673577004	0.857598747
RP11-462G2	0.014	0.673601822	0.857598747
AC243981	0.014	0.673747026	0.857730337
RPL15P21	0.014	0.673886391	0.857844344
HOXB-AS3	0.014	0.673940789	0.857844344
RP11-735G4	0.014	0.674113278	0.858004919
ATP11C	0.014	0.674146914	0.858004919
ELP3	0.014	0.674160876	0.858004919
RN7SKP230	0.014	0.674218458	0.858032326
N6AMT2	0.014	0.674231088	0.858032326
RP11-521H3	0.014	0.674250219	0.858032326
CYCSP5	0.014	0.674254474	0.858032326
BDKRB2	0.014	0.674351173	0.858119776
KLF2P2	0.014	0.674405265	0.858132502
RN7SKP241	0.014	0.674583286	0.858292864
ANP32E	0.014	0.674658405	0.858339945

THAP7	0.014	0.674898243	0.858550971
FAM104B	0.014	0.67490822	0.858550971
PPARGC1A	0.014	0.674914404	0.858550971
RP11-397G5	0.014	0.67493446	0.858553552
CCDC144NL	0.014	0.67496381	0.858567955
SLC2A4	0.014	0.675054295	0.858607315
CTC-297N7	0.014	0.675076831	0.858607315
HIGD1AP3	0.014	0.675181022	0.8586551
RP13-297E16	0.014	0.675196691	0.8586551
CCDC144A	0.014	0.67520039	0.8586551
RNU6-848P	0.014	0.675212607	0.8586551
RP11-697N18	0.014	0.675273429	0.858709519
RP1-81P11	0.014	0.675429084	0.858859185
ST13P13	0.014	0.675471867	0.858859185
CTA-253N17	0.014	0.675479018	0.858859185
NTF6G	0.014	0.675556762	0.858917512
FKBP1B	0.014	0.675831569	0.859166937
KATNA1	0.014	0.676125482	0.859357076
LINC00951	0.014	0.676208601	0.859385137
CHMP3	0.014	0.676219737	0.859385137
LINC00202-1	0.014	0.676301408	0.859443064
RP11-179G5	0.014	0.676463355	0.85949812
RP5-839B4	0.014	0.676471057	0.85949812
STK38	0.014	0.676550865	0.859554521
RNU6-745P	0.014	0.676566989	0.859554521
KDM1A	0.014	0.676603596	0.859574794
AC239800	0.014	0.676667948	0.859628408
IMPDH1P10	0.014	0.676762217	0.859684583
SAFB	0.014	0.677032291	0.85991704
RP4-575N6	0.014	0.677035488	0.85991704
IGFN1	0.014	0.677089168	0.859934241
LIPG	0.014	0.677116604	0.859934241
CDC42BPA	0.014	0.677157364	0.859934241
RP3-509I19	0.014	0.677302027	0.860072086
RPL26P20	0.014	0.677397591	0.860147571
RP11-744O11	0.014	0.677462607	0.860173619
KB-1458E12	0.014	0.677612937	0.860235596
RNU6-727P	0.014	0.677685123	0.860235596
ATP6V0B	0.014	0.677714903	0.860235596
THOC7	0.014	0.677929173	0.860380234
ANKRD20A18P	0.014	0.677937848	0.860380234
CCDC169-SOHLH2	0.014	0.678003259	0.860380234
GBP6	0.014	0.678003301	0.860380234
ENOX1-AS2	0.014	0.678134142	0.860486353
CTD-2523D13	0.014	0.678180254	0.860521939
RP11-103J8	0.014	0.678212127	0.860525776
OR4A17P	0.014	0.678219414	0.860525776
OR5D3P	0.014	0.678422975	0.860682553
MCF2L2	0.014	0.678498549	0.860719433
CTC-258N23	0.014	0.678537884	0.86072675
KRT18P12	0.014	0.678558534	0.86072675
TMEM257	0.014	0.678672672	0.860779829

DSC2	0.014	0.678728118	0.860827228
CTD-3074O7	0.014	0.678840511	0.860874219
RN7SL617P	0.014	0.678859935	0.860874219
AP000438	0.014	0.678889584	0.860874219
FOXL2NB	0.014	0.678913785	0.860874219
AL356214	0.014	0.678930097	0.860874219
AC000095	0.014	0.678985068	0.860874219
GCNT1	0.014	0.679066918	0.860874219
LA16c-83F12	0.014	0.679095477	0.860874219
RP11-361I14	0.014	0.679115819	0.860874219
RP11-492L8	0.014	0.679133889	0.860874219
RNU4-34P	0.014	0.679174904	0.860874219
RP11-728B21	0.014	0.679224237	0.860874219
SH3BGRL	0.014	0.679230942	0.860874219
RP11-147G7	0.014	0.679314342	0.860897009
MIR106A	0.014	0.679363263	0.860897009
CHCHD3	0.014	0.679429391	0.860897009
OR13C8	0.014	0.679434288	0.860897009
RPS3AP35	0.014	0.679436218	0.860897009
AC092669	0.014	0.679476346	0.860897009
MIR410	0.014	0.679524246	0.860897009
CTD-2315M5	0.014	0.679568501	0.860930174
RNA5SP52	0.014	0.679636387	0.860947827
PPP1R37	0.014	0.679636665	0.860947827
RP11-320M16	0.014	0.679822032	0.861136837
CNKSR3	0.014	0.679840762	0.861137659
PRR29	0.014	0.679923608	0.86119883
RAB15	0.014	0.679983644	0.861227024
OR5M4P	0.014	0.680119102	0.861284067
OR51B4	0.014	0.680154309	0.86130575
ZFY	0.014	0.680397513	0.86148518
HIST1H3F	0.014	0.680431421	0.86148518
RNU5F-2P	0.014	0.680703484	0.861689339
RNF186	0.014	0.680710515	0.861689339
LARP7P3	0.014	0.680831889	0.861751365
TMEM201	0.014	0.680975328	0.861853598
PNPLA3	0.014	0.680987336	0.861853598
POTEB3	0.014	0.681083159	0.861853598
MYH14	0.014	0.681193997	0.861929598
RP3-492P14	0.014	0.681306423	0.861992017
NIT2	0.014	0.681363824	0.861992017
SLC29A2	0.014	0.681367029	0.861992017
RP11-470P21	0.014	0.681375935	0.861992017
WFDC21P	0.014	0.681422306	0.861992017
RP5-1097F14	0.014	0.681434301	0.861992017
MTND5P16	0.014	0.68143829	0.861992017
ASB9	0.014	0.681554899	0.862041767
PHKG2	0.014	0.681568119	0.862041767
KDELC2	0.014	0.681608981	0.862057797
RP11-428O23	0.014	0.681672762	0.862105436
RRAGD	0.014	0.681872201	0.862266078
AC016912	0.014	0.681917271	0.862300177

GS1-115G20	0.014	0.681946882	0.862314725
AC005752	0.014	0.68205061	0.862422991
GRIK4	0.014	0.682148882	0.862497054
FAM87A	0.014	0.682156614	0.862497054
MGC27382	0.014	0.682207663	0.862515125
AC006041	0.014	0.682244451	0.862515125
RP1-84O15	0.014	0.682260522	0.862515125
H2AFJ	0.014	0.68233383	0.86253783
PSD3	0.014	0.682340644	0.86253783
TECPR2	0.014	0.682409177	0.862601568
LINC00086	0.014	0.682546653	0.862693454
AC016894	0.014	0.682702869	0.862743832
AC007098	0.014	0.682741752	0.862770077
GTF2F1	0.014	0.682781949	0.862775089
RNU6-261P	0.014	0.682867595	0.862796107
TEX36-AS1	0.014	0.682934162	0.862796107
ATG4A	0.014	0.682943508	0.862796107
C1GALT1C1	0.014	0.682973175	0.862810701
CTC-264O10	0.014	0.683046954	0.862816063
RP11-563P16	0.014	0.683081078	0.862816063
FOXP1-AS1	0.014	0.683174771	0.862816063
AP000280	0.014	0.683176697	0.862816063
AC108056	0.014	0.683200849	0.86281967
CTB-138E5	0.014	0.683215785	0.86281967
LINC01320	0.014	0.683331565	0.862897244
CTD-2333M24	0.014	0.683560831	0.86309522
CU463998	0.014	0.683636519	0.863111088
RP11-454K7	0.014	0.683677811	0.863111088
RP11-697G4	0.014	0.683682132	0.863111088
HMGB3P8	0.014	0.683841322	0.863205301
PHYHD1	0.014	0.683847381	0.863205301
EIF3IP1	0.014	0.683941458	0.863232538
AC114788	0.014	0.683994375	0.863276449
OR4F21	0.014	0.684076211	0.863313402
RNU1-49P	0.014	0.684091341	0.863313402
MTND5P26	0.014	0.684113908	0.863313402
TOMM5	0.014	0.684266989	0.863313402
PNLIPRP3	0.014	0.684269715	0.863313402
NHLH1	0.014	0.684373808	0.863313402
ADRA2C	0.014	0.684374514	0.863313402
AL137795	0.014	0.684513099	0.863450628
AF241725	0.014	0.684576274	0.863461711
RP11-786A20	0.014	0.684626054	0.86350163
CHRNA5	0.014	0.684697931	0.863534311
TLE2	0.014	0.684706358	0.863534311
CEACAM1	0.014	0.68475612	0.863574202
HIF1A	0.014	0.68494314	0.86368249
RN7SKP149	0.014	0.684950791	0.86368249
RP11-175K6	0.014	0.684990501	0.863709694
RNU6ATAC6P	0.014	0.6850252	0.86373058
MIR601	0.014	0.68507398	0.863746352
PPP2R2A	0.014	0.685174334	0.863804277

AP000357	0.014	0.685263086	0.863864436
RP11-481N16	0.014	0.685280274	0.863864436
LRRC24	0.014	0.685294604	0.863864436
CTD-2223O18	0.014	0.685493209	0.864069052
PRSS36	0.014	0.685580197	0.86415583
RNU6-1041P	0.014	0.685658366	0.864224513
CDKN2A-AS1	0.014	0.685681882	0.864224513
LINC00364	0.014	0.685743277	0.864269894
AL732374	0.014	0.685864555	0.864331625
OR55B1P	0.014	0.685892599	0.864331625
RP11-1E6	0.014	0.685906945	0.864331625
RNA5SP90	0.014	0.686056822	0.864394358
HIST1H2BA	0.014	0.686105279	0.864395377
MRPL20	0.014	0.686156832	0.864402249
OVAAL	0.014	0.68627283	0.864473445
RP11-1E22	0.014	0.686286507	0.864473445
RP1-60O19	0.014	0.686322252	0.864473445
AC114760	0.014	0.686381609	0.864478916
KRT8P42	0.014	0.686421928	0.864478916
MTNR1B	0.014	0.686439178	0.864478916
MIR2116	0.014	0.686444994	0.864478916
AC108218	0.014	0.686567237	0.864530518
HNRNPA1P54	0.014	0.686608613	0.864559312
AC010975	0.014	0.686695817	0.864578135
MEIG1	0.014	0.686828034	0.864629832
AC004941	0.014	0.686884249	0.864629832
RPS26P30	0.014	0.686936683	0.864629832
MIR877	0.014	0.686936756	0.864629832
RNU7-47P	0.014	0.686978912	0.864629832
AC005412	0.014	0.687000682	0.864629832
RP11-702B10	0.014	0.687023325	0.864629832
SPANXN5	0.014	0.687037767	0.864629832
RPL34P29	0.014	0.687045996	0.864629832
SPPL2C	0.014	0.687073416	0.864629832
CTD-2307P3	0.014	0.687081808	0.864629832
RP11-543A18	0.014	0.687264876	0.864700549
RNA5SP46	0.014	0.687288842	0.864700549
RP11-346I3	0.014	0.68735787	0.864701755
HOXB13	0.014	0.687372051	0.864701755
MIR5688	0.014	0.687403767	0.864715122
RP11-19N8	0.014	0.687563256	0.864870063
MIR6085	0.014	0.687660234	0.864969205
DIAPH3-AS2	0.014	0.687712501	0.865007763
RNU6-1078P	0.014	0.687825868	0.865063313
RP11-319F12	0.014	0.688004601	0.865147481
MAGEB4	0.014	0.688031015	0.865147481
RNU6-1146P	0.014	0.688210485	0.865210838
RNU6-1335P	0.014	0.688223153	0.865210838
NPHP3-ACAD11	0.014	0.688233829	0.865210838
HDLBP	0.014	0.688295903	0.865266035
RNA5SP237	0.014	0.688372237	0.865297096
RP3-369A17	0.014	0.688459729	0.865334939

PGRMC2	0.014	0.688498567	0.865360918
MTND1P10	0.014	0.688668402	0.865505181
RNU6-798P	0.014	0.688675312	0.865505181
AC128709	0.014	0.688704209	0.865505181
FAM90A12P	0.014	0.688950177	0.865722919
POLD2	0.014	0.689003314	0.865731685
AC020703	0.014	0.689113006	0.86579047
IMPDH1P11	0.014	0.68913958	0.865801018
OR8S21P	0.014	0.689163285	0.865807961
RP11-32B5	0.014	0.689216843	0.865829568
LINC01248	0.014	0.689273282	0.86587763
RPL5P32	0.014	0.689326198	0.865921265
NCLP2	0.014	0.68935855	0.865939066
RNFT1P3	0.014	0.68950756	0.866034878
GAGE10	0.014	0.689544561	0.866035674
RP11-255G12	0.014	0.689646961	0.866045552
RNU6-1111P	0.014	0.689647872	0.866045552
MIR1293	0.014	0.689667669	0.866045552
CCNJP2	0.014	0.689852336	0.86617097
RP11-219B17	0.014	0.689881063	0.866184203
RNA5SP340	0.014	0.690060909	0.866250338
RP11-22P4	0.014	0.690367607	0.866475949
MIR4734	0.014	0.690368128	0.866475949
GPRASP2	0.014	0.690399627	0.866492649
RN7SL488P	0.014	0.690417933	0.866492791
KLHL35	0.014	0.69044109	0.86649902
TRIM6-TRIM34	0.014	0.690675182	0.866747125
NDUFA4	0.014	0.690710017	0.866767182
SPATA31D1	0.014	0.690727563	0.866767182
MRPL39	0.014	0.690769605	0.866797102
RP11-627G23	0.014	0.690821672	0.866836455
MTND4P27	0.014	0.690871171	0.866836455
HMGN1P34	0.014	0.690932524	0.866864501
SEPHS1	0.014	0.691050258	0.866917331
MORF4L2	0.014	0.69120973	0.866917331
KRT8P49	0.014	0.69121678	0.866917331
MAP1LC3P	0.014	0.691226113	0.866917331
RP11-112J1	0.014	0.691229463	0.866917331
PTH2R	0.014	0.691450144	0.867103812
CDC14B	0.014	0.691450977	0.867103812
OR1D2	0.014	0.691523835	0.867172346
AC079809	0.014	0.691554694	0.86718821
RN7SL319P	0.014	0.691617035	0.867233047
RP11-82L20	0.014	0.691626868	0.867233047
PEX11A	0.014	0.691646333	0.867234623
C4B-AS1	0.014	0.691740886	0.86726185
MIR1270	0.014	0.691827167	0.867324362
RNA5SP507	0.013	0.692093355	0.86755773
RNU6-43P	0.013	0.69225723	0.867703637
RNA5SP96	0.013	0.692353191	0.86780108
RP11-654C22	0.013	0.692599763	0.867950241
RP11-209D20	0.013	0.692669537	0.867966676

VSIG10L	0.013	0.692681056	0.867966676
CTSG	0.013	0.692733313	0.867966676
C12orf80	0.013	0.692738382	0.867966676
RP5-1068H6	0.013	0.692787892	0.867966676
RNU1-21P	0.013	0.692938352	0.868100453
CTD-2020P22	0.013	0.693033065	0.868163288
MIR562	0.013	0.693133233	0.868207571
RP4-683L5	0.013	0.693235185	0.868312438
LINC01115	0.013	0.693299253	0.868355152
CBX2	0.013	0.693353046	0.868391552
RP11-661D19	0.013	0.693427684	0.868439356
SPINK6	0.013	0.693647223	0.868645779
RP11-27G22	0.013	0.693919651	0.868911748
PTP4A1P2	0.013	0.693935404	0.868911748
RP11-153F1	0.013	0.694021129	0.868931239
RP11-722E23	0.013	0.694072468	0.868940852
RP11-798K3	0.013	0.694083541	0.868940852
RN7SL284P	0.013	0.694106983	0.868947358
AC078819	0.013	0.694309353	0.868995122
B4GALT3	0.013	0.694344742	0.868995122
RN7SL34P	0.013	0.694346722	0.868995122
RP11-692P14	0.013	0.694349583	0.868995122
RNU2-60P	0.013	0.69435196	0.868995122
ZNF197-AS1	0.013	0.694354993	0.868995122
TLE6	0.013	0.694364105	0.868995122
RP11-114O18	0.013	0.694373635	0.868995122
KRT18P66	0.013	0.694382664	0.868995122
TSPY5P	0.013	0.694400578	0.868995122
KCNQ1-AS1	0.013	0.69444094	0.869022797
AC080037	0.013	0.69448637	0.869036883
MIR767	0.013	0.69448869	0.869036883
TFPI2	0.013	0.694609377	0.869135065
RPL32P31	0.013	0.69461964	0.869135065
AC004019	0.013	0.694689192	0.869176796
MTCH1	0.013	0.694705415	0.869176796
NCRUPAR_1	0.013	0.694718194	0.869176796
AC000041	0.013	0.694740632	0.869176796
RP1-37J18	0.013	0.694851762	0.869176796
RPS29P24	0.013	0.694889649	0.869176796
PAPPA2	0.013	0.694890169	0.869176796
RRM1-AS1	0.013	0.695130091	0.869294319
MTHFD2P1	0.013	0.695132209	0.869294319
RP1-144F13	0.013	0.695173175	0.869294319
OR52U1P	0.013	0.695291557	0.869335263
RP11-1157N2__A	0.013	0.695348288	0.869335263
SUB1P1	0.013	0.695356046	0.869335263
CTC-436P18	0.013	0.695365993	0.869335263
RP11-387A1	0.013	0.695481073	0.869434031
AC105009	0.013	0.695499618	0.869434031
RP11-438O11	0.013	0.695511117	0.869434031
BYSL	0.013	0.695520612	0.869434031
RN7SL792P	0.013	0.695536272	0.869434031

RP11-459A10	0.013	0.69562519	0.869485222
MIR4717	0.013	0.695697933	0.869485222
RP11-280O1	0.013	0.695717076	0.869485222
RNA5SP219	0.013	0.695770888	0.869499097
RPL9	0.013	0.695855267	0.869558912
RP11-64D22	0.013	0.695992476	0.869596645
MIR325HG	0.013	0.696003249	0.869596645
RP11-366N18	0.013	0.696013272	0.869596645
SLC5A7	0.013	0.696109771	0.869694396
LITAF	0.013	0.696186752	0.869721007
RP3-481A17	0.013	0.696197653	0.869721007
ARHGAP44	0.013	0.696371294	0.869884219
PELI1	0.013	0.696715793	0.870138808
AC016292	0.013	0.696751337	0.870138808
DNMBP	0.013	0.696757799	0.870138808
C16orf89	0.013	0.696805462	0.870152698
RNU6-618P	0.013	0.696858189	0.87018745
AC010744	0.013	0.696893991	0.870187609
FER	0.013	0.696926643	0.870187609
WIBG	0.013	0.696941589	0.870187609
TAAR6	0.013	0.696978931	0.870187609
MIR4482	0.013	0.696997857	0.870187609
RNU6-39P	0.013	0.697052113	0.870209724
RPS6P8	0.013	0.697087741	0.870217219
OR52E7P	0.013	0.697158627	0.870251453
RP11-226M10	0.013	0.697279736	0.870349473
RP5-1073F15	0.013	0.697308705	0.870349473
URAD	0.013	0.697516945	0.870493398
RN7SL823P	0.013	0.697543607	0.870503862
PTRH1	0.013	0.69760241	0.870554434
KRT8P19	0.013	0.697770145	0.870617734
OR5P1P	0.013	0.69784433	0.870618392
UNC93B7	0.013	0.697877219	0.87062363
AL009174	0.013	0.697932762	0.870670116
RP11-762E8	0.013	0.698152044	0.870807333
RP11-646E18	0.013	0.698241435	0.870807333
CTD-2582M21	0.013	0.698251675	0.870807333
DEFA9P	0.013	0.698253957	0.870807333
LUZP1	0.013	0.698353118	0.870852353
TMEM173	0.013	0.698420629	0.870913737
EML3	0.013	0.698536252	0.870997323
FGF17	0.013	0.698544236	0.870997323
ELAVL2	0.013	0.698720213	0.871150461
RP11-794M8	0.013	0.698845437	0.871196254
RP11-543C4	0.013	0.6988459	0.871196254
ARMCX3-AS1	0.013	0.698891336	0.871204169
RP11-411G9	0.013	0.698931467	0.871231391
MIR4666B	0.013	0.699063068	0.871281415
CR383657	0.013	0.699063068	0.871281415
RPS18	0.013	0.699238633	0.871477426
RN7SL92P	0.013	0.699482994	0.871667928
FAM21EP	0.013	0.699788343	0.871979996

CD44	0.013	0.699895958	0.872022832
RP11-325L12	0.013	0.700039678	0.872133453
EIF3LP2	0.013	0.70021827	0.872287496
RPL9P28	0.013	0.700326042	0.872358088
CNN2P4	0.013	0.70033192	0.872358088
BMS1P14	0.013	0.700362684	0.872358088
LLNLR-285B5	0.013	0.700403871	0.872358088
KCNA10	0.013	0.700412234	0.872358088
FGFR1	0.013	0.700496443	0.872360216
CYP7A1	0.013	0.700563294	0.872420657
MIR4654	0.013	0.700653818	0.872441076
RP11-15P13	0.013	0.700666022	0.872441076
RP11-97I14	0.013	0.700899554	0.872656865
RP1-184J9	0.013	0.70099827	0.872716298
RP11-437L7	0.013	0.701202871	0.872769825
ZNF696	0.013	0.701227069	0.872769825
TXN	0.013	0.701274843	0.872781879
RNA5SP344	0.013	0.701311255	0.872783969
RP1-66H9	0.013	0.701349824	0.872783969
PACSIN2	0.013	0.701388614	0.872789611
GFOD1-AS1	0.013	0.701391009	0.872789611
RP11-235E17	0.013	0.701521422	0.872865385
NDUFB8P1	0.013	0.701613763	0.872907152
DEFB109P3	0.013	0.701726582	0.872983645
AADACP1	0.013	0.701730234	0.872983645
ENG	0.013	0.701754668	0.872991239
ZSCAN23	0.013	0.7018335	0.872998096
RP11-110G21	0.013	0.702206258	0.873267649
PSKH1	0.013	0.702209093	0.873267649
FTH1P16	0.013	0.702233397	0.873267649
RPL23AP40	0.013	0.702233559	0.873267649
AC006461	0.013	0.702320193	0.873306977
RNU6-120P	0.013	0.702380124	0.873318683
LINC01485	0.013	0.702384617	0.873318683
RENBP	0.013	0.702464298	0.873344028
COX5A	0.013	0.702488605	0.873344028
INAFM2	0.013	0.702570036	0.873344028
CTD-2351A16	0.013	0.702678151	0.873387241
RP11-785D18	0.013	0.702766384	0.873430107
LINC01209	0.013	0.702934737	0.87359217
EHD4	0.013	0.703007227	0.873659461
RP11-327L3	0.013	0.703085107	0.87371065
OR8B6P	0.013	0.703119632	0.873712562
RP11-93N20	0.013	0.703218187	0.873739508
RPL9P2	0.013	0.703218402	0.873739508
TLR12P	0.013	0.703326616	0.873797349
RNA5SP366	0.013	0.703382327	0.873829202
RP1-257C22	0.013	0.703446545	0.873857212
SNORD113-1	0.013	0.703522903	0.873889868
KRT18P50	0.013	0.703586395	0.873907894
NECAB1	0.013	0.703592462	0.873907894
TPT1P14	0.013	0.703661201	0.87397048

PIM1	0.013	0.703810552	0.87401922
AIM1	0.013	0.703872172	0.874061286
CYCSP20	0.013	0.70388113	0.874061286
C2orf27AP3	0.013	0.703932177	0.874075183
CRIP1P1	0.013	0.703947378	0.874075183
PKD1L2	0.013	0.704089888	0.874195473
SNRPCP9	0.013	0.704123319	0.874195473
LDHD	0.013	0.704138137	0.874195473
TTC1	0.013	0.704175845	0.874195473
CRELD2	0.013	0.704337229	0.874238013
C4orf27	0.013	0.704353854	0.874238013
RP11-252I13	0.013	0.704383197	0.874238248
PFKFB3	0.013	0.704390756	0.874238248
ZNF385D-AS1	0.013	0.704431914	0.874251866
TMBIM1	0.013	0.704571218	0.874276924
SLC16A11	0.013	0.704572219	0.874276924
RP11-883G14	0.013	0.704585643	0.874276924
MED28P4	0.013	0.704619212	0.874276924
RP11-217E13	0.013	0.704623623	0.874276924
AL080272	0.013	0.704633531	0.874276924
CTD-2128A3	0.013	0.704675802	0.874276924
SERGEF	0.013	0.704704649	0.874276924
AC004221	0.013	0.7047228	0.874276924
MIR4663	0.013	0.704744309	0.874276924
RP13-444K19	0.013	0.70475234	0.874276924
AL445384	0.013	0.704776802	0.874284498
SIPA1L1	0.013	0.704841333	0.874302321
FLJ31662	0.013	0.704846717	0.874302321
MTND2P26	0.013	0.704858302	0.874302321
AC010468	0.013	0.70486567	0.874302321
EIF4A1P12	0.013	0.704882955	0.874302321
RNA5SP483	0.013	0.704970706	0.874342851
RPL31P12	0.013	0.705039936	0.874360406
RP1-46F2	0.013	0.70515422	0.874360704
MR1	0.013	0.705168685	0.874360704
RNA5SP55	0.013	0.705220415	0.874402081
RP11-176D17	0.013	0.705311821	0.874492648
POLR2E	0.013	0.705410888	0.874579809
ITGA3	0.013	0.705542753	0.87469128
TIMM17B	0.013	0.705563852	0.87469128
CPB2-AS1	0.013	0.705694349	0.874764548
CTA-85E5	0.013	0.705696422	0.874764548
RP13-52K8	0.013	0.705784322	0.874850738
APBA1	0.013	0.705914456	0.874965729
TSEN15	0.013	0.705940024	0.874965729
CCDC130	0.013	0.705950575	0.874965729
AC079298	0.013	0.705971045	0.874968329
GPR37	0.013	0.706085317	0.875041644
RN7SKP281	0.013	0.706290261	0.875176101
VN2R11P	0.013	0.706319798	0.875176101
RNA5SP334	0.013	0.706359194	0.875176101
ZNF135	0.013	0.706429925	0.875211548

OTX1	0.013	0.706462374	0.875211548
RNA5SP365	0.013	0.706476211	0.875211548
OR2A1	0.013	0.70655317	0.875279817
MIR4265	0.013	0.706612683	0.875330774
UCP1	0.013	0.706782581	0.875495695
PPIAP33	0.013	0.707107416	0.875668978
RP11-758I14	0.013	0.707149755	0.875668978
PTPRD-AS2	0.013	0.707150261	0.875668978
FAM213A	0.013	0.707179016	0.875668978
ERAS	0.013	0.707179875	0.875668978
ZCCHC17	0.013	0.707222061	0.875698448
SLITRK1	0.013	0.70734835	0.875752478
LINC01553	0.013	0.707356597	0.875752478
SPTAN1	0.013	0.707413342	0.875764293
AL590683	0.013	0.70744995	0.875764293
DBNDD1	0.013	0.707499039	0.875764293
RNASEK-C17orf49	0.013	0.707500257	0.875764293
AC090696	0.013	0.707545103	0.875764293
SNORD66	0.013	0.707593904	0.875764293
RNU6-295P	0.013	0.707632184	0.875764293
RP11-416K24	0.013	0.707661386	0.875764293
KCNIP2	0.013	0.707707812	0.875793601
MOXD1	0.013	0.707815457	0.87580716
PRR9	0.013	0.707843135	0.87580716
GPRIN1	0.013	0.707921889	0.875843708
RNU7-43P	0.013	0.707965032	0.875843708
FAM224B	0.013	0.707968443	0.875843708
RASA2-IT1	0.013	0.708081012	0.875942191
RNU6-178P	0.013	0.708216624	0.876057035
PANK2	0.013	0.70822903	0.876057035
RP11-264E20	0.013	0.708332355	0.876119752
RP11-420M1	0.013	0.708394177	0.876170293
AC117507	0.013	0.708483721	0.876230919
TRABD	0.013	0.708509398	0.876230919
CDIP1	0.013	0.708753635	0.876354303
SALL4P5	0.013	0.708821817	0.876354303
CTC-518B2	0.013	0.708835928	0.876354303
RARA-AS1	0.013	0.708837357	0.876354303
XX-FW80269A6	0.013	0.708881939	0.876386671
RP11-123O22	0.013	0.708910869	0.876396098
TFAP2E	0.013	0.708926367	0.876396098
ATF7	0.013	0.708994244	0.87645726
RP11-108M12	0.013	0.709022126	0.876468978
B3GNTL1P2	0.013	0.709057561	0.876490032
DYNLL1P2	0.013	0.709177673	0.876546114
RNA5SP76	0.013	0.709179608	0.876546114
RP11-460I19	0.013	0.70928212	0.876546499
PGAP1	0.013	0.709347081	0.876546499
STX5	0.013	0.709360903	0.876546499
RP4-781L3	0.013	0.70938322	0.876551335
RP11-545A16	0.013	0.709432299	0.876580084
DCAF15	0.013	0.709576377	0.876638204

KLC1	0.013	0.709612156	0.876638204
AC016738	0.013	0.709625448	0.876638204
RPL32P9	0.013	0.709664006	0.876638204
KLF2P1	0.013	0.709673296	0.876638204
MTND6P4	0.013	0.709701858	0.87664257
MEMO1	0.013	0.709750399	0.876663873
HAUS4P1	0.013	0.709792862	0.876675865
RN7SL274P	0.013	0.709811371	0.876675865
PTH	0.013	0.70981533	0.876675865
RP11-390F4	0.013	0.709906544	0.876723241
RP11-413N13	0.013	0.709983415	0.87676978
PAQR6	0.013	0.710031795	0.87680679
MIR4471	0.013	0.710077084	0.876839982
RP11-435I10	0.013	0.710119035	0.876869051
AC017048	0.013	0.710290962	0.87705861
SNAI3-AS1	0.013	0.710356343	0.87709085
SLC18A3	0.013	0.710401955	0.87709085
OR6N1	0.013	0.710457944	0.87709085
RN7SL492P	0.013	0.710504688	0.87709085
RNU6-945P	0.013	0.710544529	0.87709085
OR7E33P	0.013	0.710722828	0.87709085
RP11-554K11	0.013	0.710784539	0.87709085
FGD5P1	0.013	0.710800771	0.87709085
RN7SL807P	0.013	0.710857994	0.87709085
UPK3BL	0.013	0.710898722	0.87709085
ALDH2	0.013	0.710920563	0.87709085
C6orf52	0.013	0.710953505	0.87709085
TBC1D3C	0.013	0.710981877	0.87709085
CNN2P6	0.013	0.711050917	0.87709085
ASNSP4	0.013	0.71105128	0.87709085
ZNF736P7Y	0.013	0.711059663	0.87709085
RP11-346K17	0.013	0.711062599	0.87709085
KCTD17	0.013	0.711241185	0.877179511
LINC00307	0.013	0.711248407	0.877179511
RP11-12A2	0.013	0.711254506	0.877179511
SERPINA2	0.013	0.711288762	0.877199044
CYP51A1	0.013	0.711364891	0.87723041
TLE4	0.013	0.711403143	0.87723041
TM4SF1-AS1	0.013	0.711427489	0.877233838
C2orf73	0.013	0.711705204	0.877451536
YWHAEP2	0.013	0.71171458	0.877451536
RN7SL419P	0.013	0.711778948	0.877475217
RNU2-42P	0.013	0.711800547	0.877475217
MIR651	0.013	0.711816212	0.877475217
PCDHGB9P	0.013	0.71185876	0.877493003
MIR580	0.013	0.71193202	0.877515174
RP3-468O1	0.013	0.711971143	0.877520991
RP11-257I8	0.013	0.712144694	0.877586029
DRAXINP1	0.013	0.71215734	0.877586029
ENSA	0.013	0.712165498	0.877586029
RPS27P12	0.013	0.712205496	0.877586029
COL18A1-AS2	0.013	0.712315614	0.877615926

SNUPN	0.013	0.712345444	0.877615926
RND1	0.013	0.712400001	0.877637735
RP11-424G14	0.013	0.712476841	0.877709694
LSM3P2	0.013	0.712621404	0.877815485
MPC1L	0.013	0.712695016	0.877815485
AC017002	0.013	0.712702692	0.877815485
RN7SL150P	0.013	0.712758178	0.87782922
MRPL15	0.013	0.712833659	0.877876779
RP11-20B7	0.013	0.712863956	0.87789139
MYCBP2-AS1	0.013	0.712976535	0.877947836
RNU6-1309P	0.013	0.713038445	0.877975504
AC012168	0.013	0.713119349	0.878001583
CDY4P	0.013	0.713270384	0.878142138
OR2W4P	0.013	0.713343798	0.878180306
TMEM258	0.013	0.713410791	0.878180306
EGFLAM-AS4	0.013	0.713421535	0.878180306
AC013403	0.013	0.713424031	0.878180306
DNER	0.013	0.713430458	0.878180306
RP11-122C5	0.013	0.71350392	0.878183887
RPL36AP51	0.013	0.713516372	0.878183887
ADSS	0.013	0.713543999	0.878183887
AL031721	0.013	0.713686854	0.878337006
CDCA7	0.012	0.713832035	0.878368236
GS1-164F24	0.012	0.713844917	0.878368236
RP11-813N20	0.012	0.713868496	0.878368236
SNORD109B	0.012	0.713917878	0.878368236
ADRB3	0.012	0.713970036	0.878368236
TMEM86B	0.012	0.714002349	0.878368236
TECRL	0.012	0.714031653	0.878368236
BLOC1S4	0.012	0.714063878	0.878368236
TIAM2	0.012	0.714136411	0.878368236
RP11-764I5	0.012	0.714193698	0.878378967
ARHGAP31-AS1	0.012	0.714254951	0.878404663
RP11-5A19	0.012	0.714267226	0.878404663
LRRC34	0.012	0.714310418	0.878404663
CCDC122	0.012	0.714332018	0.878404663
RP11-90P16	0.012	0.714376452	0.878413943
RP11-181E10	0.012	0.714544239	0.878506853
SNORD17	0.012	0.714658407	0.878571883
POLR3KP1	0.012	0.71467092	0.878571883
CTD-2170G1	0.012	0.714742251	0.878614215
TMEM86A	0.012	0.714788914	0.878621681
TBX4	0.012	0.714804166	0.878621681
RNA5SP229	0.012	0.714840564	0.878621681
LINC00659	0.012	0.714916529	0.878679858
NUDT4P2	0.012	0.714924794	0.878679858
AL031681	0.012	0.715021041	0.878775473
AQP1	0.012	0.715049403	0.878787653
RP11-164A6	0.012	0.715109136	0.878838386
CCDC178	0.012	0.71514067	0.878840086
DDC	0.012	0.715147425	0.878840086
AC090051	0.012	0.715218383	0.878876264

CCDC34P1	0.012	0.715232223	0.878876264
PCDH10	0.012	0.715319239	0.878915159
SNORA74B	0.012	0.715481056	0.879057321
PNPT1P2	0.012	0.715521929	0.879057321
DEFB132	0.012	0.715539156	0.879057321
AC006326	0.012	0.715563754	0.879057321
RPL34	0.012	0.715613298	0.879057321
RPL37P2	0.012	0.715637968	0.879057321
PPP3CA	0.012	0.715816795	0.879164906
RP1-47A17	0.012	0.71581742	0.879164906
PCDHGA10	0.012	0.71584412	0.879164906
C9orf85	0.012	0.715882864	0.879164906
GIF	0.012	0.715910035	0.879164906
RP11-12K22	0.012	0.715951497	0.879164906
Z95114	0.012	0.715956266	0.879164906
RP11-420N3	0.012	0.715965524	0.879164906
SMPD2	0.012	0.716038666	0.879189219
RP1-232N11	0.012	0.716104009	0.879221614
TCEB3CL2	0.012	0.716263288	0.879352217
ACTBP12	0.012	0.716344722	0.879403806
IDH1-AS1	0.012	0.716566713	0.879500439
FDPSP4	0.012	0.716574895	0.879500439
MTND6P12	0.012	0.716593306	0.879500439
AC133555	0.012	0.71661152	0.879500439
CTC-458I2	0.012	0.716662567	0.879500439
ARSD	0.012	0.716749304	0.879500439
PPP2R3B	0.012	0.716940672	0.879618403
VTI1BP2	0.012	0.716944313	0.879618403
AC073578	0.012	0.716982655	0.879633279
RP11-386I14	0.012	0.717013911	0.879635074
RP11-350D17	0.012	0.717045935	0.879635074
HIST1H2BE	0.012	0.717124465	0.879635074
GGT7	0.012	0.717152462	0.879635074
RP11-43P5	0.012	0.717196287	0.879635074
AC098973	0.012	0.717197817	0.879635074
SDCBP	0.012	0.717199393	0.879635074
RP11-208G20	0.012	0.71723494	0.879635074
RP5-1139I1	0.012	0.717276129	0.879662938
MYL12A	0.012	0.717494473	0.879885399
SLC2A3	0.012	0.71768795	0.880036153
AC003958	0.012	0.717709792	0.880036153
ETV5-AS1	0.012	0.717872565	0.88011268
ECE1	0.012	0.717883079	0.88011268
YOD1	0.012	0.718021884	0.880237536
CTD-2061E9	0.012	0.718090074	0.88025411
LHX2	0.012	0.718146298	0.88025411
ZNF576	0.012	0.718241951	0.880348699
RP1-199J3	0.012	0.718321981	0.880385785
BHMG1	0.012	0.718334762	0.880385785
BMS1P17	0.012	0.718353766	0.880385785
ABT1P1	0.012	0.718364633	0.880385785
AC006499	0.012	0.718510248	0.880541584

RP11-412H8	0.012	0.718602483	0.880563986
PLA2G1B	0.012	0.718656636	0.880566846
EZR	0.012	0.718694406	0.880566846
RP11-11N6	0.012	0.718735412	0.880566846
CBX1P1	0.012	0.718798867	0.880566846
LINC00971	0.012	0.718808194	0.880566846
SNORD99	0.012	0.718879416	0.88060366
PUS3	0.012	0.718893714	0.88060366
RPL23AP46	0.012	0.719031673	0.88073018
OR52M1	0.012	0.719166513	0.88077945
NPIPP1	0.012	0.719169018	0.88077945
PTGDR2	0.012	0.719221405	0.88077945
RP11-568A19	0.012	0.71925844	0.88077945
LINC01415	0.012	0.719272503	0.88077945
ALG1L10P	0.012	0.719295574	0.88077945
GEMIN8P3	0.012	0.719296023	0.88077945
JAZF1-AS1	0.012	0.719312986	0.88077945
RNU6-799P	0.012	0.719317179	0.88077945
RN7SL727P	0.012	0.719353863	0.880782211
RP11-663P9	0.012	0.719455726	0.880795304
DPP7	0.012	0.719457024	0.880795304
KALP	0.012	0.719621771	0.880864055
RBBP4P4	0.012	0.719628537	0.880864055
LL0XNC01-240C2	0.012	0.719678043	0.880864055
RN7SL438P	0.012	0.719680525	0.880864055
RP11-506B6	0.012	0.719719446	0.880867504
RP4-683M8	0.012	0.7197792	0.880895903
RP11-643M14	0.012	0.719779641	0.880895903
RP11-73E6	0.012	0.719814476	0.8809159
RN7SKP217	0.012	0.719857704	0.88092353
USMG5P1	0.012	0.719980999	0.880953905
RN7SL451P	0.012	0.720165735	0.881057069
RPS25	0.012	0.720168017	0.881057069
MIR4431	0.012	0.720189649	0.881057069
FGF7P2	0.012	0.720245735	0.881060227
SNRPEP2	0.012	0.720246895	0.881060227
RP11-122D10	0.012	0.720296982	0.881069332
CTD-2373N4	0.012	0.72030861	0.881069332
RP13-122B23	0.012	0.720456973	0.881160791
PRMT1	0.012	0.720458613	0.881160791
RP11-284F21	0.012	0.720511342	0.881202652
AC104667	0.012	0.720697945	0.881317714
TNNI3	0.012	0.720754092	0.881344961
AL590007	0.012	0.720757236	0.881344961
MIR4529	0.012	0.720891514	0.881430232
RP11-244J10	0.012	0.720936609	0.881446259
AC130709	0.012	0.721192285	0.881632828
MIR4705	0.012	0.721196275	0.881632828
MIR4293	0.012	0.721316695	0.881757404
MIR7702	0.012	0.721368266	0.881761545
KIAA1919	0.012	0.721548605	0.881900414
RNPEPL1	0.012	0.72156251	0.881900414

RNU6-823P	0.012	0.721564873	0.881900414
MIR6504	0.012	0.721581818	0.881900414
AL109615	0.012	0.721647459	0.881958007
RP11-361H10	0.012	0.721690642	0.88198815
MIR4301	0.012	0.721767261	0.882020926
MIR125B1	0.012	0.721784677	0.882020926
ARHGAP24	0.012	0.721791538	0.882020926
OR10AE3P	0.012	0.721962979	0.88211726
RNU6-344P	0.012	0.722115357	0.882245908
KLHL5	0.012	0.722145355	0.882245908
AC010894	0.012	0.722159673	0.882245908
BCAR3	0.012	0.722194853	0.882245908
AL513123	0.012	0.722197939	0.882245908
RP11-378J18	0.012	0.722254171	0.882269343
PRELID1	0.012	0.722404313	0.882407484
CTD-2060F24	0.012	0.722452505	0.88242706
RP11-823E8	0.012	0.722610968	0.882507917
CAST	0.012	0.722641617	0.882516277
RN7SKP60	0.012	0.722696592	0.882532494
RP11-420O16	0.012	0.722755834	0.882542614
TMEM196	0.012	0.722857583	0.88256174
RNU6-119P	0.012	0.722864151	0.88256174
ZNF849P	0.012	0.722933956	0.882609601
AL589663	0.012	0.72303568	0.882663616
HMGB3P11	0.012	0.72304422	0.882663616
LINC00588	0.012	0.723062996	0.882663616
SNORD115-42	0.012	0.723067922	0.882663616
DEFB117	0.012	0.723095856	0.882663616
APOE	0.012	0.723372925	0.882888685
AC012494	0.012	0.723456114	0.882909742
TRIM43	0.012	0.723538481	0.882909742
RPL24P8	0.012	0.723660688	0.883033799
RP11-394O4	0.012	0.723686887	0.883033799
AC007682	0.012	0.723698537	0.883033799
RP11-200K12	0.012	0.723806477	0.883078389
RASSF9	0.012	0.723898542	0.883082916
GJB7	0.012	0.723906744	0.883082916
LINC01108	0.012	0.723911453	0.883082916
PLEKHO1	0.012	0.724049619	0.883082916
CTD-2021H9	0.012	0.724081332	0.883082916
FAM212B-AS1	0.012	0.724099263	0.883082916
RNU6-127P	0.012	0.724101215	0.883082916
AD000091	0.012	0.724266528	0.883218985
RASSF1-AS1	0.012	0.724279731	0.883218985
RP11-501C14	0.012	0.72430722	0.883218985
XXYLT1-AS1	0.012	0.724329089	0.883218985
RNU6-243P	0.012	0.724483072	0.883292937
RP11-119B16	0.012	0.724513973	0.883308
MIR4259	0.012	0.724559526	0.883340924
SNRPCP16	0.012	0.724695043	0.883438399
BAIAP3	0.012	0.724738816	0.883446433
HIST1H3B	0.012	0.724967823	0.883635125

AKR1B10P1		0.012	0.724995601	0.883640118
ANKRD34C-AS1		0.012	0.725015909	0.883640118
RP11-15I20		0.012	0.72502758	0.883640118
AL008708		0.012	0.725153016	0.883747508
AL589947		0.012	0.725207859	0.883769374
MIR6765		0.012	0.725261268	0.883805582
CFC1		0.012	0.725286582	0.883805582
AC104304		0.012	0.725324491	0.883805582
SPIN1		0.012	0.725358588	0.883805582
GRIK1-AS1		0.012	0.725370972	0.883805582
RP11-364B6		0.012	0.725414914	0.883805582
CTD-2266L18		0.012	0.725446411	0.883805582
CAPZA3		0.012	0.725463391	0.883805582
COX6B1P7		0.012	0.7255921	0.883861716
RP11-532L16		0.012	0.725629508	0.883861716
MUC13		0.012	0.725654793	0.883861716
RNU6-665P		0.012	0.725814625	0.883965968
CKLF-CMTM1		0.012	0.725891661	0.884024472
RAI2		0.012	0.725904364	0.884024472
AL355361		0.012	0.725942314	0.884024472
SMCO4P1		0.012	0.726119129	0.88404817
OR4A6P		0.012	0.726141562	0.88404817
RP13-650J16		0.012	0.726175832	0.88404817
ZCCHC12		0.012	0.726179111	0.88404817
RNU6-781P		0.012	0.726209165	0.884056156
AC055873		0.012	0.726222795	0.884056156
RNU6-240P		0.012	0.726302761	0.884097326
FBXO39		0.012	0.726455407	0.884141331
RP11-102M11		0.012	0.726471276	0.884141331
	8-Sep	0.012	0.72649414	0.884141331
WDR74		0.012	0.726535951	0.884141331
OR1A2		0.012	0.726545874	0.884141331
HIST1H2BPS2		0.012	0.726555974	0.884141331
LINC01448		0.012	0.726578163	0.884141331
CNTROB		0.012	0.726580222	0.884141331
AC128677		0.012	0.726589785	0.884141331
RNU6-337P		0.012	0.726667778	0.88415207
COX6CP5		0.012	0.726732632	0.8841596
BEST4		0.012	0.726733322	0.8841596
IFNA4		0.012	0.726734748	0.8841596
WFDC10B		0.012	0.726901646	0.884249213
LRRC26		0.012	0.726919803	0.884249213
RP11-40F6		0.012	0.727112255	0.884429012
RNU6-608P		0.012	0.727206165	0.884484601
POP5		0.012	0.727301399	0.884577842
MIR6804		0.012	0.727372446	0.884641663
AL050335		0.012	0.727406252	0.884660187
AC093106		0.012	0.727613862	0.884756539
SLC27A6		0.012	0.727684071	0.884769703
KRT126P		0.012	0.727685191	0.884769703
SPRED1		0.012	0.72774349	0.884774504
AP000654		0.012	0.727763793	0.884774504

RP11-305A4	0.012	0.727889716	0.884841459
MRPL17	0.012	0.727928473	0.884850383
CTNNBL1	0.012	0.727934215	0.884850383
YEATS2-AS1	0.012	0.728092141	0.884997177
RP3-354J5	0.012	0.728120734	0.885009346
MLIP-IT1	0.012	0.728168722	0.885011072
RP11-94H6	0.012	0.728177901	0.885011072
SPAG17	0.012	0.728355705	0.885104339
HOXD3	0.012	0.728396732	0.885104339
POU6F2	0.012	0.728440481	0.885104339
OR8A2P	0.012	0.728470704	0.885118481
LINC01144	0.012	0.728504032	0.885136394
MIR6850	0.012	0.72858188	0.885153358
WEE2	0.012	0.728592334	0.885153358
RPSA	0.012	0.728645176	0.885173372
RPS12P24	0.012	0.728645979	0.885173372
SP5	0.012	0.728720679	0.885173807
NDUFAF4P1	0.012	0.72877925	0.885222376
RP11-65D17	0.012	0.728918437	0.885269021
FOXB2	0.012	0.728949218	0.885269021
RP3-426I6	0.012	0.728980839	0.885269021
D4S234E	0.012	0.72900259	0.885269021
RP11-739B23	0.012	0.729049695	0.885302513
RNU6-750P	0.012	0.729349622	0.88544722
RP11-73M11	0.012	0.729352082	0.88544722
POLR3KP2	0.012	0.729394108	0.88544722
MBNL3	0.012	0.72941055	0.88544722
RNU1-83P	0.012	0.729454124	0.885475289
NPM1P10	0.012	0.72950516	0.88548112
CTB-32H22	0.012	0.729626763	0.885551031
AC004052	0.012	0.72970711	0.885558938
RP11-1081K18	0.012	0.730034599	0.885866082
WWC2	0.012	0.730078471	0.885896747
PSMD13	0.012	0.730263242	0.885962948
IDUA	0.012	0.730319087	0.885997518
ATG5	0.012	0.730328943	0.885997518
RP11-321C24	0.012	0.730503618	0.886119038
AL583860	0.012	0.730522139	0.886119038
AC108066	0.012	0.730668256	0.886204816
TNXA	0.012	0.730684358	0.886204816
SULT1B1	0.012	0.730696636	0.886204816
RP3-527G5	0.012	0.730876787	0.886354693
HMGA1	0.012	0.730935943	0.886368837
CHP2	0.012	0.730956505	0.886368837
CRB3P1	0.012	0.730970243	0.886368837
ROBO2P1	0.012	0.731078602	0.886437479
TUBB8P3	0.012	0.731090415	0.886437479
RP11-92J19	0.012	0.731238026	0.886535828
RP11-815J21	0.012	0.731319819	0.886612423
AC000367	0.012	0.731445782	0.886742561
SLC25A13	0.012	0.731607206	0.886809793
PAICSP2	0.012	0.731721049	0.886846905

RP11-394D2	0.012	0.731809847	0.886912161
ARHGEF7-IT1	0.012	0.731941663	0.886940528
DNAJB3	0.012	0.731960742	0.886940528
OR56A5	0.012	0.731961764	0.886940528
OR51K1P	0.012	0.732027089	0.88699573
GABRA1	0.012	0.732187255	0.887054395
RNU6-1045P	0.012	0.732510175	0.88733276
RNU6-1135P	0.012	0.7325675	0.887365446
RNA5SP41	0.012	0.732574422	0.887365446
SRGAP2C	0.012	0.732702866	0.887453319
RP11-471J7	0.012	0.732881383	0.887577068
CD300LB	0.012	0.732892792	0.887577068
HMGB3P23	0.012	0.732898216	0.887577068
RP11-930O11	0.012	0.733000613	0.887655933
GDF3	0.012	0.73306929	0.887655933
A2ML1	0.012	0.733157559	0.887720964
RP11-314N14	0.012	0.733166148	0.887720964
CTC-278L1	0.012	0.733274057	0.887760129
CSTF2	0.012	0.733287495	0.887760129
TAS2R7	0.012	0.733320267	0.887760129
BMP4	0.012	0.733373023	0.887778497
MIR518A1	0.012	0.733381427	0.887778497
DPY30	0.012	0.733473359	0.887867217
SNORD115-36	0.012	0.733867354	0.88818902
RP11-359E10	0.012	0.733869744	0.88818902
RP11-692C23	0.012	0.733963027	0.888249143
CBS	0.012	0.734003224	0.888249143
RP11-146E13	0.012	0.734012672	0.888249143
AC004692	0.012	0.734112615	0.888337733
D2HGDH	0.012	0.734123183	0.888337733
BNIP3	0.012	0.734152388	0.888344848
OR52K1	0.012	0.734192626	0.888344848
TMC2	0.012	0.734248432	0.888344848
CATSPERG	0.012	0.734251525	0.888344848
SLC28A1	0.012	0.734646391	0.888627722
LINC01280	0.012	0.734757623	0.88872159
RP11-366I13	0.012	0.734791329	0.888739788
AC097523	0.012	0.734898672	0.888830889
AKIRIN1P1	0.012	0.735035369	0.888830889
RP11-104D21	0.012	0.735120464	0.888860738
RP11-559M23	0.012	0.73515261	0.888860738
MIR6784	0.012	0.735265341	0.888974472
IGSF23	0.012	0.735306022	0.88900109
RP11-449M6	0.012	0.735361053	0.88903066
C9orf135-AS1	0.012	0.73547015	0.889109243
ISCUP1	0.012	0.735524627	0.889152531
MIR103A1	0.012	0.735630073	0.88925743
SNX18	0.012	0.735673655	0.889279777
GYG1P2	0.011	0.735739738	0.889290459
AC104389	0.011	0.735759001	0.889290459
KRT8P48	0.011	0.735769427	0.889290459
HDAC6	0.011	0.735931349	0.889378091

GLRA4	0.011	0.735939086	0.889378091
PPP1R2P6	0.011	0.735953974	0.889378091
AF096876	0.011	0.736084709	0.889468372
HIST1H2AD	0.011	0.736160645	0.889514995
B4GALT5	0.011	0.736421707	0.889717027
RN7SL538P	0.011	0.73646265	0.889717027
AC002407	0.011	0.736488314	0.889717027
OR2AT1P	0.011	0.736495974	0.889717027
HNRNPA1P64	0.011	0.736599446	0.889748851
KLHL29	0.011	0.736606185	0.889748851
RP11-497K21	0.011	0.736640894	0.889756685
RP11-465B22	0.011	0.736759791	0.889832595
RP11-326K13	0.011	0.736849571	0.889895895
CYP51A1P3	0.011	0.736910617	0.889913773
ALDH1L1-AS2	0.011	0.736978169	0.889913773
TSPEAR-AS2	0.011	0.737008374	0.889913773
RP4-798A17	0.011	0.737013855	0.889913773
UBE2S	0.011	0.737097102	0.88992728
HMGN2P38	0.011	0.737147279	0.88992728
ISYNA1	0.011	0.737167125	0.88992728
RN7SL174P	0.011	0.737194558	0.88992728
AC005371	0.011	0.737206211	0.88992728
BMS1P13	0.011	0.737259892	0.88992728
RP11-447H19	0.011	0.737299415	0.88992728
RP11-430K21	0.011	0.737305205	0.88992728
CTD-2151A2	0.011	0.737325805	0.88992728
RP11-118N24	0.011	0.737347162	0.88992728
RP11-93L14	0.011	0.737367923	0.88992728
RPL9P7	0.011	0.73739327	0.88992728
RP11-340E6	0.011	0.737467796	0.889965507
NR1I3	0.011	0.737549401	0.890018885
RP11-736E3	0.011	0.737626225	0.890031375
FLJ26850	0.011	0.737680626	0.890031375
C3orf36	0.011	0.737703006	0.890031375
NEK4P1	0.011	0.737707613	0.890031375
RAB12	0.011	0.737762401	0.890059206
RDH5	0.011	0.737869938	0.890112498
AL117380	0.011	0.73804308	0.890186081
CAPN1	0.011	0.738219778	0.890354108
SNORA53	0.011	0.738245242	0.890362273
RP4-614C15	0.011	0.738345289	0.890460386
SOHLH1	0.011	0.738635803	0.890652879
AC006390	0.011	0.738696411	0.89070341
HIST1H2AL	0.011	0.738800393	0.890752158
RN7SL771P	0.011	0.738830354	0.890752158
RP1-202I21	0.011	0.738853452	0.890756739
SOCS1	0.011	0.738946416	0.890777768
MIR377	0.011	0.73898129	0.890777768
AL356806	0.011	0.739017261	0.890777768
RP11-64C12	0.011	0.739029695	0.890777768
GOLGA7B	0.011	0.739265501	0.890934181
RNU6-695P	0.011	0.739278527	0.890934181

SLC35F3	0.011	0.739280636	0.890934181
AC092287	0.011	0.739345022	0.89095988
SND1-IT1	0.011	0.739353582	0.89095988
RBM7	0.011	0.739517832	0.891062206
TTC39DP	0.011	0.739662991	0.891109914
MIR124-3	0.011	0.739669394	0.891109914
RP3-450M14	0.011	0.739679725	0.891109914
OR10AA1P	0.011	0.739688398	0.891109914
MTND4P29	0.011	0.739752423	0.891163777
RP3-522P13	0.011	0.739770531	0.891163777
RP1-23E21	0.011	0.739824653	0.891206433
RNU6-936P	0.011	0.739845002	0.891208405
TMSB15B	0.011	0.739959827	0.891245889
MIR5697	0.011	0.739962178	0.891245889
AC011376	0.011	0.74007956	0.891267258
TRIM60P13	0.011	0.740104882	0.891267258
ATN1	0.011	0.740116613	0.891267258
MIR7850	0.011	0.740118422	0.891267258
AL161422	0.011	0.740170856	0.89128934
AGBL4-IT1	0.011	0.740174187	0.89128934
EMP2	0.011	0.740284069	0.891376583
KNOP1P4	0.011	0.740361206	0.891401855
AC104581	0.011	0.740388466	0.891412141
RNF17	0.011	0.740454379	0.891446428
PFN3	0.011	0.740740793	0.8916327
RBM10	0.011	0.740788161	0.891645423
TMEM164	0.011	0.740855822	0.891659259
RNU7-182P	0.011	0.741024663	0.891772327
RNA5SP409	0.011	0.741024663	0.891772327
BEX2	0.011	0.741081922	0.891796167
MBL2	0.011	0.741105183	0.891801626
F2RL2	0.011	0.741154955	0.891837614
ALOX12P2	0.011	0.741173745	0.891837614
RP11-595O22	0.011	0.741323264	0.891951372
PIGU	0.011	0.741546157	0.892089478
BIRC7	0.011	0.741550432	0.892089478
RPSAP15	0.011	0.741596484	0.892122344
TMEM126A	0.011	0.741653048	0.892145322
AQP4-AS1	0.011	0.741673665	0.892147589
KCNMB1	0.011	0.741750038	0.892185247
AC021205	0.011	0.741789109	0.892185247
ROPN1L	0.011	0.741849917	0.892185247
RPL21P111	0.011	0.741963951	0.892185247
GPR75-ASB3	0.011	0.741972512	0.892185247
RP11-95M5	0.011	0.742126604	0.892185247
RRM2P3	0.011	0.742140365	0.892185247
IGHVII-67-1	0.011	0.742161769	0.892185247
LINC01541	0.011	0.742167369	0.892185247
RNASEH1	0.011	0.742193355	0.892185247
TAS2R13	0.011	0.742194405	0.892185247
RP11-212E8	0.011	0.742273092	0.892201877
AC008271	0.011	0.74228079	0.892201877

RP1-40E16		0.011	0.742377885	0.892273546
GCNT1P3		0.011	0.742497098	0.892352828
RP11-572C15		0.011	0.742637697	0.89240235
PGD		0.011	0.742653687	0.89240235
KRTAP19-9P		0.011	0.742672786	0.892402785
FECHP1		0.011	0.742759866	0.89243498
RP3-419C19		0.011	0.742789255	0.89243498
MRPS21P7		0.011	0.742845224	0.892452374
AC107072		0.011	0.742942148	0.892546304
CTC-305H11		0.011	0.742964591	0.892549829
SNRPCP5		0.011	0.743001303	0.892549829
RP11-626E13		0.011	0.743059745	0.892576666
ERC2		0.011	0.743061126	0.892576666
RPS10P14		0.011	0.743119879	0.892624729
AC123023		0.011	0.743147735	0.892635675
KLF4		0.011	0.743211574	0.892687811
NAV2-AS3		0.011	0.743266113	0.892687811
OPCML-IT2		0.011	0.743445673	0.892858437
RP1-19N1		0.011	0.74360707	0.892984252
KCTD21-AS1		0.011	0.743612213	0.892984252
RNA5SP315		0.011	0.743706121	0.89304134
RNU7-80P		0.011	0.743756147	0.893051154
DPF3		0.011	0.744138932	0.893341542
FTH1P21		0.011	0.744320961	0.893459042
TMPRSS3		0.011	0.744353784	0.893475923
CD24P4		0.011	0.744401975	0.893495364
RPL15		0.011	0.744407501	0.893495364
	11-Mar	0.011	0.744441526	0.893508313
DEFB108B		0.011	0.744460116	0.893508313
MTND4P19		0.011	0.744493332	0.893508313
RP3-446N13		0.011	0.744681005	0.893581756
RP4-620E11		0.011	0.744691803	0.893581756
PPP2R2B-IT1		0.011	0.744754595	0.893581756
RNU6-956P		0.011	0.744799129	0.893581756
LDHC		0.011	0.744812927	0.893581756
SDHCP3		0.011	0.744834088	0.893581756
BCL2L1		0.011	0.744843888	0.893581756
RNU6-172P		0.011	0.744963206	0.893625866
RP11-317J19		0.011	0.745043269	0.893664267
NDUFS3		0.011	0.745129899	0.893664267
MIR6738		0.011	0.745216902	0.893742972
LINC01327		0.011	0.745263203	0.893742972
HS2ST1		0.011	0.745283665	0.893742972
RP5-891H21		0.011	0.745448707	0.893827046
IPMKP1		0.011	0.745491664	0.893827046
MYL12B		0.011	0.74550193	0.893827046
RPL35P2		0.011	0.745680113	0.893827046
ETV4		0.011	0.745707813	0.893827046
AP006748		0.011	0.745711176	0.893827046
TACR1		0.011	0.745737363	0.893827046
AC017081		0.011	0.745864724	0.893898475
GLA		0.011	0.745869473	0.893898475

SUMO2P13	0.011	0.745993114	0.893979167
AC004158	0.011	0.746263311	0.894116286
TIMM9P3	0.011	0.746276494	0.894116286
CDIPT	0.011	0.746325984	0.894153087
RP11-353P15	0.011	0.7463523	0.894162122
ACTL6B	0.011	0.746408108	0.894206489
NABP1	0.011	0.746775553	0.894511689
AC140542	0.011	0.746897235	0.894592796
RNA5SP157	0.011	0.747004989	0.894592796
RP11-482L11	0.011	0.747140712	0.894611592
MAP3K10	0.011	0.747190042	0.894648167
RP1-149M18	0.011	0.747367998	0.894793757
RNA5SP191	0.011	0.747411673	0.894801059
CTC-575N7	0.011	0.747519493	0.894832487
TIMM10	0.011	0.747533851	0.894832487
VWDE	0.011	0.747569427	0.894832487
MIR1229	0.011	0.747569443	0.894832487
RPL21P5	0.011	0.74767617	0.894892767
ICMT	0.011	0.747721706	0.894924778
KB-1907C4	0.011	0.74785637	0.89498328
GAS6-AS2	0.011	0.747879366	0.89498328
UCA1	0.011	0.747890381	0.89498328
PAPSS1	0.011	0.747902126	0.89498328
RP11-686D16	0.011	0.747948627	0.895016439
RPL7AP31	0.011	0.747986623	0.895034442
PLCD1	0.011	0.748048624	0.895034442
FAM230B	0.011	0.748092501	0.895034442
CCDC13	0.011	0.748114013	0.895034442
TCF7L1-IT1	0.011	0.748143066	0.895046717
CTD-2616J11	0.011	0.748162134	0.895047046
RNA5SP204	0.011	0.748280418	0.8951211
SLC16A12-AS1	0.011	0.748329934	0.895132876
RP11-395N21	0.011	0.748334928	0.895132876
APBB1	0.011	0.74835034	0.895132876
SNORA5	0.011	0.748365441	0.895132876
DIO2-AS1	0.011	0.748423087	0.895134386
AC073089	0.011	0.748609104	0.895266937
TIGD7	0.011	0.748716258	0.89536611
AP000962	0.011	0.748755182	0.89536611
RP11-363G10	0.011	0.748776854	0.89536611
LINC01410	0.011	0.748798638	0.89536611
NT5M	0.011	0.748840037	0.89536611
EIF4A1P6	0.011	0.748842426	0.89536611
RN7SL336P	0.011	0.74887996	0.895384422
RP11-574O7	0.011	0.749007743	0.895410569
IFNWP5	0.011	0.749011214	0.895410569
KLHL25	0.011	0.749041658	0.895424488
ZNF408	0.011	0.749124464	0.895495505
ITGB3BP	0.011	0.749145166	0.895495505
RP11-60A14	0.011	0.749248612	0.895495505
AC011379	0.011	0.749435055	0.895557591
RPL5P14	0.011	0.749558011	0.895561167

LINC00189	0.011	0.749559017	0.895561167
RNU7-97P	0.011	0.749569672	0.895561167
OR1E3	0.011	0.749633411	0.895587586
NAIF1	0.011	0.74963834	0.895587586
RP11-255M2	0.011	0.749726635	0.895587586
RN7SL621P	0.011	0.749728899	0.895587586
DCLRE1B	0.011	0.749767965	0.895587586
SLC50A1	0.011	0.749795713	0.895587586
RP11-316E14	0.011	0.749798631	0.895587586
RN7SL518P	0.011	0.749893778	0.895627917
LGALS13	0.011	0.749971065	0.895681234
GPAM	0.011	0.750006113	0.895690172
OR10A3	0.011	0.750034969	0.895690172
CLUHP1	0.011	0.750083805	0.895721789
KCNJ14	0.011	0.750186293	0.895758582
AC008756	0.011	0.750327596	0.895867134
AC015909	0.011	0.750448581	0.895895855
Z99916	0.011	0.750541314	0.89595787
RNU6-99P	0.011	0.750568408	0.895967757
PCDHGB5	0.011	0.750592243	0.895973753
R3HDM2P1	0.011	0.75062149	0.895986208
RP11-718M20	0.011	0.750732626	0.896052881
RPS16P8	0.011	0.750752133	0.896052881
RAB7A	0.011	0.750752602	0.896052881
MIR4658	0.011	0.751001796	0.896238005
LINC01291	0.011	0.751132187	0.896331713
RP11-379P1	0.011	0.751212059	0.896354171
ACTC1	0.011	0.751342219	0.896380617
RNU6-28P	0.011	0.75134944	0.896380617
DUOX1	0.011	0.751353856	0.896380617
CTD-2308N23	0.011	0.751375826	0.896380617
SYT14P1	0.011	0.751639604	0.896476877
RN7SL435P	0.011	0.751712358	0.896476877
NPVF	0.011	0.751740301	0.896476877
C1QBPP	0.011	0.751795797	0.896489194
PFN1P12	0.011	0.751884093	0.896544265
SCN8A	0.011	0.751895156	0.896544265
AL358815	0.011	0.751954638	0.896544265
RP11-146F11	0.011	0.752016613	0.896544265
MAN2A1	0.011	0.7520419	0.896544265
PEX10	0.011	0.752068016	0.896544265
RNU6-723P	0.011	0.752119695	0.896544265
BRIX1	0.011	0.752133101	0.896544265
KB-1440D3	0.011	0.752189553	0.896544265
MRPS9	0.011	0.752288565	0.896605378
RP11-148E17	0.011	0.752354116	0.896661065
RP11-556N21	0.011	0.752435628	0.896724847
CDRT8	0.011	0.752468364	0.896729908
ARL14	0.011	0.752606307	0.896782101
TMEM202	0.011	0.752787509	0.896928069
AC099665	0.011	0.752809509	0.896928069
G6PC	0.011	0.75285466	0.896943388

KHDC1	0.011	0.75294287	0.897003604
RNY4P18	0.011	0.753156164	0.897048936
MIR8060	0.011	0.753162805	0.897048936
AL356476	0.011	0.753162805	0.897048936
RN7SL123P	0.011	0.753169271	0.897048936
MIR6500	0.011	0.753375214	0.897198297
C19orf35	0.011	0.753390577	0.897198297
MIR6515	0.011	0.753412189	0.897198297
PROL1	0.011	0.753445379	0.897198297
RP11-344H11	0.011	0.753582934	0.897294792
GNAS-AS1_2	0.011	0.753737707	0.897380552
PF4	0.011	0.753826502	0.897427757
RP11-266J6	0.011	0.754182813	0.897739742
RP11-384F7	0.011	0.754280222	0.897788378
PABPC1P4	0.011	0.75431425	0.897806443
CD300A	0.011	0.754375661	0.897850464
COL15A1	0.011	0.754388939	0.897850464
H2AFY2	0.011	0.754441223	0.897867816
AC234917	0.011	0.754462275	0.897870434
SNORA54	0.011	0.754557822	0.897939269
SMURF2P1	0.011	0.75460569	0.897973795
RP11-621K7	0.011	0.754641827	0.89799436
SAE1	0.011	0.75494503	0.898242947
AC004485	0.011	0.754997131	0.898269867
RNU7-127P	0.011	0.755022039	0.898269867
AC010900	0.011	0.755043097	0.898269867
RP11-793A3	0.011	0.755120572	0.898326079
TMC4	0.011	0.755128069	0.898326079
THAP8	0.011	0.755188255	0.898375238
RNU6-343P	0.011	0.755298992	0.898462089
RNU7-34P	0.011	0.755395675	0.89855282
LINC00533	0.011	0.755466178	0.898561819
SNORD50	0.011	0.755472545	0.898561819
MIR6806	0.011	0.755499244	0.898565618
SURF6P1	0.011	0.755550632	0.898565618
MIR3144	0.011	0.755574692	0.898565618
PSG11	0.011	0.755681124	0.898595613
NUDT15	0.011	0.755767363	0.898618699
FAM46A	0.011	0.755770269	0.898618699
MIR6827	0.011	0.755790247	0.898620018
SPX	0.011	0.756049545	0.898838563
VAMP3	0.011	0.75617012	0.898958529
OR5A2	0.011	0.756261714	0.898984437
ADH7	0.011	0.756266623	0.898984437
PIFO	0.011	0.756309233	0.898986075
MRGPRG	0.011	0.756324628	0.898986075
KRT8P6	0.011	0.756537744	0.899194509
TPBG	0.011	0.756751121	0.899315435
RNU6-177P	0.011	0.756870943	0.899410973
AC064853	0.011	0.757068226	0.899484261
TMPRSS11BNL	0.011	0.757096639	0.899484261
HSD17B7P1	0.011	0.757104991	0.899484261

RNU6-1106P		0.011	0.757114116	0.899484261
RP11-322A17		0.011	0.757145773	0.899484261
RPL7AP58		0.011	0.757240935	0.899518273
ADH5P2		0.011	0.757253816	0.899518273
RP11-561E1		0.011	0.757273459	0.899518273
CLCN7		0.011	0.757292377	0.899518273
RP4-673D20		0.011	0.757379404	0.899518273
RP5-968D22		0.011	0.757392121	0.899518273
SLCO1B7		0.011	0.757551599	0.899636205
TMEM150A		0.011	0.757615193	0.899666862
TSPAN19		0.011	0.757653713	0.899676272
RN7SKP65		0.011	0.757757225	0.899738295
RP11-296C13		0.011	0.757781458	0.899738295
RNU4-58P		0.011	0.757795136	0.899738295
RPS3AP15		0.011	0.757807588	0.899738295
RP11-622C24		0.011	0.757852495	0.899740896
RP11-25K19		0.011	0.757867695	0.899740896
VWA2		0.010	0.757960491	0.89975129
HSPE1P19		0.010	0.758005956	0.89975129
RPEP6		0.010	0.758087338	0.899819261
HOXC9		0.010	0.758112587	0.899819261
	12-Sep	0.010	0.758121391	0.899819261
RP5-1177M21		0.010	0.758154809	0.899820394
RPSAP41		0.010	0.758166301	0.899820394
DHODH		0.010	0.758339086	0.899930805
AL356756		0.010	0.758357851	0.899930805
CH17-12M21		0.010	0.758390937	0.899930805
GCNT1P4		0.010	0.758551571	0.899991301
SOX2OT_exon4		0.010	0.758626822	0.899991301
PRSS23		0.010	0.75863419	0.899991301
OXTR		0.010	0.758677835	0.899991301
NPS		0.010	0.758697331	0.899991301
PRSS29P		0.010	0.75875058	0.899991301
LCN8		0.010	0.758843703	0.899991301
PIGX		0.010	0.758866556	0.899991301
MEX3B		0.010	0.75889815	0.899991301
RP11-525G12		0.010	0.758905841	0.899991301
PCA3		0.010	0.758968022	0.899991301
AC090311		0.010	0.758968051	0.899991301
AC126917		0.010	0.759016562	0.899991301
ZEB1-AS1		0.010	0.759023697	0.899991301
RP1-40G4P		0.010	0.759041102	0.899991301
AC068137		0.010	0.759068627	0.900001531
MED14OS		0.010	0.759195398	0.900060509
ASCC2		0.010	0.75921286	0.900060509
RNU6-1178P		0.010	0.759286757	0.90012571
RP11-488C13		0.010	0.75945027	0.900274735
CDH8		0.010	0.759494939	0.900305278
FAM53A		0.010	0.75957834	0.900346045
DDO		0.010	0.759670238	0.900401026
OR10H3		0.010	0.759819291	0.900446238
AC010890		0.010	0.759836468	0.900446238

STOX2		0.010	0.759865689	0.900446238
AL354932		0.010	0.759871341	0.900446238
LINC01140		0.010	0.759999602	0.900544884
RP11-405A12		0.010	0.760210577	0.900638751
KIRREL3-AS1		0.010	0.760227635	0.900638751
RP11-561B11		0.010	0.76027712	0.900672049
RP11-142A12		0.010	0.760404473	0.900725758
RP3-499B10		0.010	0.760417017	0.900725758
HDHD1P3		0.010	0.7604612	0.900741272
PLSCR4		0.010	0.760467939	0.900741272
RP11-156F23		0.010	0.760524517	0.900763483
LINC00334		0.010	0.760553476	0.900775381
RP1-117B12		0.010	0.760641226	0.900818722
RP11-359J6		0.010	0.760739979	0.900884255
NAGPA		0.010	0.760976305	0.901009755
RNU6-1291P		0.010	0.761013259	0.901009755
AC096553		0.010	0.761018445	0.901009755
AL138919		0.010	0.761028232	0.901009755
PCDH9-AS1		0.010	0.761035136	0.901009755
	2-Mar	0.010	0.761119432	0.901048102
MIR3681		0.010	0.761143201	0.901048102
RP11-881L2		0.010	0.761277456	0.901139838
PCAT4		0.010	0.761331382	0.901181273
RP11-26M5		0.010	0.761407867	0.901217199
SOX2		0.010	0.761628527	0.901309588
HAAO		0.010	0.761673708	0.901309588
MEGF9		0.010	0.761723649	0.901309588
RP11-77G22		0.010	0.762085479	0.90159635
LSM1P1		0.010	0.762090221	0.90159635
RP11-390M11		0.010	0.762098512	0.90159635
RORB		0.010	0.76223096	0.901598867
RP11-190G13		0.010	0.762285717	0.901598867
AL355821		0.010	0.762306738	0.901598867
ZBTB20-AS3		0.010	0.762310647	0.901598867
RNU6-716P		0.010	0.76232972	0.901598867
CCER1		0.010	0.762370986	0.901598867
RNF216		0.010	0.762395032	0.901598867
PHKA2-AS1		0.010	0.762632354	0.901739271
AC008641		0.010	0.762635854	0.901739271
AC006989		0.010	0.762707947	0.901776318
RP11-250B2		0.010	0.762786789	0.901776318
LINC01152		0.010	0.762789684	0.901776318
RN7SL169P		0.010	0.762791982	0.901776318
OR5H8		0.010	0.76283471	0.901776318
RNU6-500P		0.010	0.762873227	0.901796058
RP11-448N11		0.010	0.762945373	0.901821144
FZD5		0.010	0.763107817	0.901891101
RTN4IP1		0.010	0.763124057	0.901891101
TEX40		0.010	0.763283922	0.90194582
RP11-14C22		0.010	0.763320463	0.90194582
T		0.010	0.763387029	0.902000448
OR2M7		0.010	0.763472402	0.902056564

RP11-475B2	0.010	0.763540714	0.902114896
RPL5P27	0.010	0.763675035	0.902207424
MORN1	0.010	0.763675858	0.902207424
RNU6-1062P	0.010	0.763723354	0.902222865
EEF1GP5	0.010	0.763864502	0.902299486
RP4-563E14	0.010	0.763867455	0.902299486
RP11-471M2	0.010	0.763990375	0.902377046
RECQL4	0.010	0.763996999	0.902377046
KCNK15-AS1	0.010	0.764008903	0.902377046
RP11-579D7	0.010	0.764203179	0.90251698
CCDC147-AS1	0.010	0.764241142	0.902539435
LINC00881	0.010	0.76441952	0.902663482
UFL1-AS1	0.010	0.764421992	0.902663482
AC019322	0.010	0.764447041	0.902670681
SNORD3A	0.010	0.764603424	0.902728612
ANKRD54	0.010	0.764647735	0.902728612
DPYD-IT1	0.010	0.764770891	0.902739744
KATNBL1P3	0.010	0.764795432	0.902740002
CSNK1E	0.010	0.764810236	0.902740002
U47924	0.010	0.76487524	0.902740002
NOSIP	0.010	0.764997751	0.902776941
RP11-533K11	0.010	0.765052455	0.902776941
RP11-524C21	0.010	0.765072083	0.902776941
C1QL1	0.010	0.765142355	0.902776941
RP11-672F9	0.010	0.765157102	0.902776941
RNU1-104P	0.010	0.765200461	0.902776941
AC079741	0.010	0.765324385	0.902823439
RBM22P7	0.010	0.765362162	0.902823439
GPM6BP2	0.010	0.765362162	0.902823439
CTD-2081C10	0.010	0.765531559	0.902930202
RPS6KC1	0.010	0.765546623	0.902930202
RP11-125O18	0.010	0.76558286	0.90293732
RNU6-1081P	0.010	0.765666678	0.902957044
RRAGB	0.010	0.765675419	0.902957044
RP11-244O19	0.010	0.765703346	0.90296762
HYDIN	0.010	0.765899852	0.903154627
COL6A4P2	0.010	0.766062441	0.903205903
RP11-227F8	0.010	0.766086182	0.903205903
OR5H15	0.010	0.766212549	0.903211736
ZNF587P1	0.010	0.766647589	0.903489862
RP11-288C17	0.010	0.766665147	0.903489862
SLCO1B3	0.010	0.766702296	0.903489862
RNU7-2P	0.010	0.766748896	0.903489862
TRIT1	0.010	0.766749384	0.903489862
RP11-608B3	0.010	0.766770771	0.903489862
MIR6511B1	0.010	0.766836233	0.90352059
TRHR	0.010	0.766887363	0.90355848
MIR6880	0.010	0.767007115	0.90363251
AC079584	0.010	0.767031729	0.903639156
AC012146	0.010	0.767178929	0.903767127
FABP5P11	0.010	0.7672177	0.903767127
THAP4	0.010	0.767235234	0.903767127

MIR204	0.010	0.767301227	0.903776715
MIR190A	0.010	0.767320161	0.903776715
POP4	0.010	0.767338254	0.903776715
RP11-21C4	0.010	0.767396945	0.903780362
SRGAP3-AS4	0.010	0.767617612	0.903945675
RP11-576C2	0.010	0.767666817	0.903945675
SLC25A5P2	0.010	0.767696661	0.903945675
SNORD114-30	0.010	0.767733016	0.903945675
SSU72	0.010	0.767972315	0.904143408
RP11-417D4	0.010	0.768037063	0.904153432
SOCS3	0.010	0.768142954	0.904237454
MPZ	0.010	0.768147126	0.904237454
FLJ37505	0.010	0.768297753	0.904325361
MIR548AI	0.010	0.768421235	0.904353742
RNU6-1313P	0.010	0.768439395	0.904353742
OR4K5	0.010	0.768474656	0.904353742
TET1	0.010	0.768637828	0.904353742
DYNLL1P6	0.010	0.768638293	0.904353742
RPL26P19	0.010	0.768738817	0.904353742
AP000469	0.010	0.768739606	0.904353742
RN7SKP115	0.010	0.768812022	0.904381718
RNA5SP149	0.010	0.768820354	0.904381718
HTN1	0.010	0.768918875	0.9043896
CTBP2P1	0.010	0.769066253	0.904451511
PABPC1P13	0.010	0.769078899	0.904451511
MANBA	0.010	0.769098989	0.904451511
AC016995	0.010	0.769310189	0.904600444
OR6U2P	0.010	0.769346953	0.904605102
RPS20P24	0.010	0.769373512	0.904605102
RP13-786C16	0.010	0.769377592	0.904605102
OSBPL6	0.010	0.769409118	0.904605102
KCNJ8	0.010	0.769458375	0.904640682
FAM181A-AS1	0.010	0.769600278	0.904673523
MTRNR2L9	0.010	0.769638228	0.904689602
HTR1D	0.010	0.769651947	0.904689602
MIR6074	0.010	0.769792815	0.904788194
RP11-586K2	0.010	0.76990764	0.904900824
CTD-2196P11	0.010	0.770135633	0.905079451
RPS3A	0.010	0.770272917	0.905196116
TMPRSS2	0.010	0.770394431	0.905298166
THG1L	0.010	0.770397771	0.905298166
RP11-598G2	0.010	0.770642626	0.905474192
RAPSN	0.010	0.770740318	0.90548667
RP11-559N14	0.010	0.770870401	0.905584795
OR7E15P	0.010	0.7709166	0.905584795
RNU6-410P	0.010	0.770926901	0.905584795
GPR112	0.010	0.771171403	0.905766996
RP11-7M8	0.010	0.771173095	0.905766996
AC004866	0.010	0.771174696	0.905766996
SATB1-AS1	0.010	0.771295293	0.905851188
RN7SL33P	0.010	0.771305839	0.905851188
RP11-509M23	0.010	0.771365191	0.90589095

BMP10	0.010	0.771503871	0.905972061
CTD-2534I21	0.010	0.771544417	0.905997336
RN7SL223P	0.010	0.771616602	0.906037422
RP11-674E16	0.010	0.771754246	0.906142807
RPSAP37	0.010	0.771840371	0.906186886
MRPL35P2	0.010	0.772029587	0.906308285
DNTTIP1	0.010	0.772033958	0.906308285
ATP2B2-IT2	0.010	0.772052133	0.906308285
RP11-134C1	0.010	0.772056601	0.906308285
MRPL13	0.010	0.772187728	0.906411603
AC008169	0.010	0.772216533	0.906411603
OR4A16	0.010	0.772232118	0.906411603
MTAP	0.010	0.772295123	0.906411603
RP11-402P6	0.010	0.772315898	0.906411603
RP11-602N24	0.010	0.772377109	0.906448342
CERS3	0.010	0.772385266	0.906448342
AC134772	0.010	0.772477565	0.906511987
A2ML1-AS1	0.010	0.772603573	0.906592844
RP11-421K23	0.010	0.772752452	0.906684961
RBPMS2P1	0.010	0.772752485	0.906684961
ANAPC2	0.010	0.772774898	0.906684961
RN7SL304P	0.010	0.77294978	0.906792378
RP11-461L18	0.010	0.7732249	0.906996116
CELF5	0.010	0.773232899	0.906996116
RNA5SP127	0.010	0.773301091	0.907053765
ERMAP	0.010	0.773395642	0.907104928
LINC00437	0.010	0.773420894	0.907104928
GEMIN4	0.010	0.77346086	0.907107125
SLC16A9	0.010	0.773592554	0.907172221
RP11-686O6	0.010	0.773629347	0.907193029
BIK	0.010	0.773709204	0.907264335
RP11-59N23	0.010	0.773778814	0.907276391
CTB-55B8	0.010	0.773795683	0.907276391
RYKP1	0.010	0.773832203	0.907287231
AKR1C6P	0.010	0.773892516	0.907300582
SCN1B	0.010	0.77398726	0.907311049
SUMO2P16	0.010	0.774019311	0.907311049
PCBP2P3	0.010	0.774113751	0.907311049
RNA5SP511	0.010	0.774128807	0.907311049
RP11-85B7	0.010	0.774159272	0.907311049
ELL2P4	0.010	0.774163576	0.907311049
RP11-87P13	0.010	0.774278208	0.907338176
RNU6-702P	0.010	0.77434755	0.907341501
RN7SKP140	0.010	0.774351476	0.907341501
RP11-467C18	0.010	0.77435367	0.907341501
FTH1P6	0.010	0.774384642	0.907341501
RP11-105L10	0.010	0.774550149	0.907468449
AC106883	0.010	0.774550149	0.907468449
AC084125	0.010	0.774687753	0.907607341
RP11-144G6	0.010	0.774761377	0.907671269
CAMKMT	0.010	0.774810307	0.907706265
UBE2HP1	0.010	0.7749755	0.90779254

RP11-37J13	0.010	0.775002589	0.90779254
RNU6-1225P	0.010	0.775003057	0.90779254
RPL5P2	0.010	0.775017374	0.90779254
CTA-339C12	0.010	0.775132999	0.907879066
AC231657	0.010	0.775155566	0.907879066
RP4-815D20	0.010	0.775167494	0.907879066
GLTSCR1	0.010	0.775206865	0.907902852
RP11-736G13	0.010	0.775269049	0.907931202
TMSB4XP2	0.010	0.775343689	0.907994737
CT47B1	0.010	0.775428286	0.908002128
VDAC1P2	0.010	0.775457878	0.908002128
RP11-100M12	0.010	0.775485242	0.908002128
RNVU1-6	0.010	0.775604268	0.908060481
TOMM22P6	0.010	0.775609605	0.908060481
FOXA2	0.010	0.775646514	0.908060481
OGFR	0.010	0.77570683	0.908108772
FTH1P2	0.010	0.775737374	0.908122208
ATP6AP1L	0.010	0.775771267	0.908139563
RNU6-681P	0.010	0.775938602	0.908290801
TMEM220	0.010	0.776028613	0.908373839
AC021451	0.010	0.776269071	0.908553165
CTD-2290C23	0.010	0.776332062	0.908570172
LINC00505	0.010	0.77636627	0.908570172
LINC00560	0.010	0.776386645	0.908570172
RP11-415D17	0.010	0.776408178	0.908570172
AC037445	0.010	0.776424328	0.908570172
RP11-398K22	0.010	0.776497339	0.908616907
SLC17A8	0.010	0.776503356	0.908616907
RP11-563N4	0.010	0.776659295	0.908746485
AKR7A2P1	0.010	0.776671335	0.908746485
RN7SKP180	0.010	0.77685067	0.908903926
ARHGAP31	0.010	0.776871122	0.908903926
PKN1	0.010	0.776985821	0.908980464
RAB1A	0.010	0.77708336	0.909005261
RN7SL766P	0.010	0.777109345	0.909013331
ZNF783	0.010	0.777261285	0.909131926
RP11-745L13	0.010	0.777359713	0.909131926
FAHD2B	0.010	0.77736344	0.909131926
RP11-369K16	0.010	0.777544914	0.909234347
RP11-220D10	0.010	0.77761973	0.909234347
PSMC2	0.010	0.777651085	0.909234347
RP11-156L14	0.010	0.777708474	0.909234347
RN7SL432P	0.010	0.777740419	0.909234347
HSPA8P5	0.010	0.777765921	0.909234347
LINC00616	0.010	0.777861418	0.909312398
AC002398	0.010	0.777909028	0.90931342
NAP1L4P2	0.010	0.777919569	0.90931342
KCTD5	0.010	0.77798315	0.909327252
AC005176	0.010	0.77798868	0.909327252
RNA5SP363	0.010	0.7780997	0.909390062
BAI2	0.010	0.778220184	0.909440316
SNORD83	0.010	0.778261884	0.909440316

AC004938	0.010	0.778267756	0.909440316
UBE2Q2	0.010	0.778272536	0.909440316
KCNRG	0.010	0.778302272	0.909440316
KLF10	0.010	0.778351693	0.909440316
AC092107	0.010	0.778353769	0.909440316
CLDN12	0.010	0.778489072	0.909440316
RP11-587H10	0.010	0.77849812	0.909440316
RP11-138H10	0.010	0.77851831	0.909440316
TP53BP2	0.010	0.778539753	0.909440316
ZNF192P2	0.010	0.778845104	0.909606798
ECM1	0.010	0.778858102	0.909606798
DNAJA1P2	0.010	0.778987514	0.909668453
AL360074	0.010	0.779093914	0.909748347
RN7SL2	0.010	0.779293798	0.909854756
CD3EAP	0.010	0.779299664	0.909854756
MFHAS1	0.010	0.779396448	0.909912818
STON1-GTF2A1L	0.010	0.77957361	0.909951531
RP11-424I3	0.010	0.779723357	0.910037109
CDC26	0.010	0.779797867	0.910085441
HMGCS2	0.010	0.77996505	0.91019811
RP11-486M23	0.010	0.78003863	0.91024893
AC073094	0.010	0.780067433	0.910260239
RN7SKP203	0.010	0.780103548	0.910280078
RPL36AP26	0.010	0.780234079	0.91036922
CCDC58	0.010	0.780259697	0.91036922
RP11-802F5	0.010	0.780306444	0.91036922
RN7SL757P	0.010	0.780351906	0.91036922
MIR4427	0.009	0.78037405	0.91036922
MIR581	0.009	0.780398721	0.91036922
MYBPC2	0.009	0.780433299	0.91036922
RPL7L1P1	0.009	0.780441413	0.91036922
RP3-445O10	0.009	0.780493643	0.91036922
FTLP15	0.009	0.780498589	0.91036922
SNORA65	0.009	0.780513056	0.91036922
RP11-710N8	0.009	0.780551784	0.91036922
KRT38	0.009	0.780639551	0.91036922
RRM2P4	0.009	0.78064213	0.91036922
TRAF3IP2	0.009	0.780643637	0.91036922
KLHDC4	0.009	0.780696034	0.91036922
CYP2C60P	0.009	0.780750256	0.910387867
BCL2L12P1	0.009	0.780770467	0.910389146
TONSL	0.009	0.78080209	0.91040373
CTRC	0.009	0.780974196	0.910537528
CHRNA2	0.009	0.781018957	0.910557211
SGMS1	0.009	0.781120282	0.910564037
SUMO4	0.009	0.781209634	0.910609175
CHEK2P2	0.009	0.781226843	0.910609175
HBM	0.009	0.781287198	0.910657238
CPSF1	0.009	0.781347944	0.910661182
CES5AP1	0.009	0.781566767	0.91073133
RP11-48L13	0.009	0.781641562	0.91073133
RP5-1059H15	0.009	0.781652411	0.91073133

C1orf185	0.009	0.781663196	0.91073133
SLC6A6P1	0.009	0.781736672	0.91073133
AC005363	0.009	0.781755289	0.91073133
RP13-77O11	0.009	0.781757691	0.91073133
ATXN1	0.009	0.781789485	0.91073133
KIFC2	0.009	0.781805421	0.91073133
CTC-470C15	0.009	0.782112822	0.910830041
MSANTD3-TMEFF1	0.009	0.782127458	0.910830041
RNA5SP147	0.009	0.782170719	0.910830041
MPV17L	0.009	0.782174895	0.910830041
MIR892B	0.009	0.782230893	0.910865523
RNU7-194P	0.009	0.782484953	0.911049989
RAB30-AS1	0.009	0.782625796	0.911147151
BCAP29	0.009	0.782715985	0.911208824
SNORD116-17	0.009	0.782732516	0.911208824
TMEM89	0.009	0.782838544	0.911208824
BMP5	0.009	0.782850931	0.911208824
SSX4B	0.009	0.782873924	0.911208824
RFC4	0.009	0.782890499	0.911208824
AC025166	0.009	0.782908337	0.911208824
ERC1	0.009	0.782992862	0.911208824
BRSK1	0.009	0.783032299	0.911208824
C20orf187	0.009	0.783034856	0.911208824
RNU6-737P	0.009	0.78303526	0.911208824
RP4-725G10	0.009	0.783042429	0.911208824
RN7SL460P	0.009	0.78307558	0.911208824
RP1-27C22	0.009	0.783161881	0.911222801
AP001607	0.009	0.783170385	0.911222801
U2	0.009	0.783182385	0.911222801
CDR2	0.009	0.78321858	0.911222801
CTD-2008N3	0.009	0.783226485	0.911222801
DIAPH3-AS1	0.009	0.783279841	0.911247154
CDT1	0.009	0.783338159	0.911263684
RP11-561C5	0.009	0.783402602	0.91127811
RP4-568F9	0.009	0.783589066	0.911355225
CAMK2N2	0.009	0.783621811	0.911366784
AC008173	0.009	0.783656412	0.911366784
RP1-67A8	0.009	0.783706054	0.911380008
RPL10AP3	0.009	0.783763808	0.911385009
C11orf72	0.009	0.783799752	0.911385009
RP11-259N19	0.009	0.783806033	0.911385009
NDUFA8	0.009	0.783931093	0.911463666
CNTNAP1	0.009	0.783988589	0.911508264
SNORD39	0.009	0.78404988	0.91154563
RNU7-4P	0.009	0.784080016	0.91154563
OR11H2	0.009	0.7841043	0.91154563
LINC00993	0.009	0.784118432	0.91154563
KCNK13	0.009	0.784192981	0.91154563
MCIDAS	0.009	0.78429195	0.911616959
AC126763	0.009	0.784452908	0.911658447
OR7E13P	0.009	0.784461261	0.911658447
RP11-346C16	0.009	0.78448036	0.911658447

DNASE1L1	0.009	0.784500226	0.911658447
SFPQP1	0.009	0.784838021	0.911939312
RP11-363G2	0.009	0.784903731	0.911973988
NDUFAF5	0.009	0.784906161	0.911973988
MVK	0.009	0.785181575	0.912186773
RP11-92C4	0.009	0.785185062	0.912186773
IGF1	0.009	0.7852265	0.912212663
RP11-796G6	0.009	0.785403165	0.912282959
MOCS1	0.009	0.785421744	0.912282959
RP11-61L19	0.009	0.78548053	0.912282959
RNU1-35P	0.009	0.785509204	0.912282959
CTD-2210P24	0.009	0.785514552	0.912282959
AC093906	0.009	0.785585594	0.912282959
AC041039	0.009	0.785585694	0.912282959
RP11-301G21	0.009	0.785589535	0.912282959
LINC00940	0.009	0.785686039	0.912323702
RP11-113E21	0.009	0.785711033	0.912330482
PRKG1-AS1	0.009	0.785779775	0.912348637
ZNF582-AS1	0.009	0.785784137	0.912348637
CNOT7P2	0.009	0.785954646	0.912413151
OR6C70	0.009	0.786032561	0.912452792
OR2T34	0.009	0.786046268	0.912452792
MIR1193	0.009	0.786239133	0.912587702
RP11-45L9	0.009	0.786395822	0.912725084
RP5-1065J22	0.009	0.786504019	0.912806173
RP11-255L13	0.009	0.786537754	0.912823081
RNU6-1122P	0.009	0.786576144	0.912845391
CYP4F29P	0.009	0.786609146	0.912855221
DCSTAMP	0.009	0.786632129	0.912855221
ZKSCAN7	0.009	0.786674566	0.912855221
RP6-102O10	0.009	0.786677443	0.912855221
CXCL12	0.009	0.786680448	0.912855221
PCCB	0.009	0.787152657	0.913158437
AC107081	0.009	0.787224905	0.913197763
TMEM184A	0.009	0.787261058	0.913217459
SNORD113	0.009	0.787396843	0.913307903
OR4K17	0.009	0.787409222	0.913307903
RP11-310P5	0.009	0.787415733	0.913307903
ACTBP14	0.009	0.787489085	0.913370739
NFKB2	0.009	0.787512329	0.913375455
TRMT2A	0.009	0.787562754	0.913411695
AC026951	0.009	0.787747603	0.913578485
RP1-27O5	0.009	0.787830544	0.913578485
MTND4P1	0.009	0.787840836	0.913578485
MIR4498	0.009	0.787926973	0.913589394
RP3-417O22	0.009	0.787974897	0.913611676
SMAP1	0.009	0.788076717	0.913696288
COX6CP1	0.009	0.788202851	0.913714404
DUXAP10	0.009	0.788209372	0.913714404
TMLHE	0.009	0.788241895	0.913714404
RP11-2P2	0.009	0.788244842	0.913714404
CTD-3073N11	0.009	0.78832256	0.913714404

CRK	0.009	0.788344452	0.913717542
RP11-98E6	0.009	0.788441186	0.913735589
RN7SKP289	0.009	0.788467906	0.913735589
KCTD14	0.009	0.788482322	0.913735589
NCAPH2	0.009	0.788513007	0.913735589
FLJ43681	0.009	0.788551092	0.913735589
SNORA8	0.009	0.788628848	0.913735589
PDC	0.009	0.788629215	0.913735589
RP11-214N9	0.009	0.788639115	0.913735589
SNORD115-40	0.009	0.78869406	0.913735589
MED13P1	0.009	0.788705142	0.913735589
LINC01285	0.009	0.788712048	0.913735589
RNU6-389P	0.009	0.788723663	0.913735589
RNU6ATAC30P	0.009	0.788858386	0.913735589
POM121L10P	0.009	0.788863385	0.913735589
DNAJC19P6	0.009	0.788876415	0.913735589
AC092104	0.009	0.7888868	0.913735589
ENPP7P14	0.009	0.78889721	0.913735589
RP3-455E7	0.009	0.788929469	0.913750731
RP11-461K13	0.009	0.788970162	0.91377564
AC105399	0.009	0.789223936	0.913936209
RP11-624L12	0.009	0.789295445	0.913974572
POU5F1P5	0.009	0.789475813	0.914009323
RNU6ATAC11P	0.009	0.789619625	0.914127707
LINC00051	0.009	0.789944424	0.914343953
RP11-417O11	0.009	0.789980471	0.914343953
RP3-520B18	0.009	0.790038555	0.914343953
RP11-673C5	0.009	0.790065778	0.914343953
RNU1-117P	0.009	0.790167063	0.914343953
RP11-123M21	0.009	0.790183504	0.914343953
RP11-478C6	0.009	0.790201207	0.914343953
AL442643	0.009	0.790228773	0.914343953
RNU6-892P	0.009	0.790526083	0.914576864
AC064847	0.009	0.790669791	0.914629768
AC013262	0.009	0.790745737	0.914652456
CTSLP7	0.009	0.790749639	0.914652456
RP11-446H18	0.009	0.790900207	0.914743053
OR4K11P	0.009	0.791088071	0.914759202
RP11-142L1	0.009	0.791163496	0.914759202
ETV6	0.009	0.791168738	0.914759202
RP11-449G16	0.009	0.791176522	0.914759202
CTD-2555C10	0.009	0.79117969	0.914759202
RP11-690C23	0.009	0.791198064	0.914759202
NARSP2	0.009	0.791281654	0.914782002
BTF3P8	0.009	0.791344191	0.914782002
CARSP1	0.009	0.791387385	0.914782002
NELFE	0.009	0.791434655	0.914802913
RP11-425L10	0.009	0.791457377	0.914802913
ACOT11	0.009	0.791600866	0.914892424
MED15P1	0.009	0.791619127	0.914892424
RP11-386J22	0.009	0.791703966	0.914949369
SORT1	0.009	0.791758669	0.914949369

RNU6ATAC2P	0.009	0.791782498	0.914949369
RN7SKP11	0.009	0.792146467	0.915206554
SLC8A1-AS1	0.009	0.792222805	0.915272548
AC007285	0.009	0.792338188	0.915338436
CTD-2333K2	0.009	0.792366328	0.915338436
RP11-160A9	0.009	0.792429076	0.915372145
SPATA22	0.009	0.792446622	0.915372145
FIRRE	0.009	0.792462768	0.915372145
IGLV3-7	0.009	0.792567827	0.915471295
MIR4445	0.009	0.792630382	0.915521347
CTD-3088G3	0.009	0.792808806	0.91563861
CCDC17	0.009	0.792901842	0.915686849
RP11-370F5	0.009	0.793042649	0.915686849
AC124312	0.009	0.793121145	0.915686849
RP1-287H17	0.009	0.793130028	0.915686849
PDCD6IPP2	0.009	0.793130226	0.915686849
RP11-146P2	0.009	0.793144712	0.915686849
EEF1A1P19	0.009	0.793173811	0.915686849
FABP5P5	0.009	0.793177419	0.915686849
RP11-12K11	0.009	0.793432611	0.915853611
RNU6-333P	0.009	0.79369938	0.916022982
OLA1	0.009	0.793884163	0.916214041
OR5AP2	0.009	0.793932172	0.916224637
RP11-69G7	0.009	0.793980651	0.91623358
RP4-603I14	0.009	0.794152513	0.916279506
HSPE1P11	0.009	0.794330023	0.916373317
RP11-386I23	0.009	0.794483122	0.916505539
RPS13	0.009	0.794662084	0.916650499
HIST1H2AH	0.009	0.794735351	0.916707691
MIR541	0.009	0.79480344	0.916719624
RP11-326L2	0.009	0.794931661	0.916800906
FER1L6	0.009	0.794955564	0.916801494
MMACHC	0.009	0.795089669	0.916894326
RP11-342F17	0.009	0.795252013	0.917005429
LINC01397	0.009	0.795340497	0.917037531
CBX1P4	0.009	0.795447248	0.917129033
RP11-317P15	0.009	0.795557967	0.917198934
RPL6P8	0.009	0.795584907	0.917198934
RP5-875H18	0.009	0.795687795	0.917273226
AC011994	0.009	0.795771769	0.917303339
NCOR2	0.009	0.795812701	0.91732832
RP11-680L20	0.009	0.795873492	0.917335358
GPR128	0.009	0.795876589	0.917335358
CTD-2579N5	0.009	0.795927965	0.917350173
OR6R1P	0.009	0.796100997	0.917460793
TRIM50	0.009	0.79628619	0.917628168
GS1-542M4	0.009	0.796463943	0.91770142
RNU6-918P	0.009	0.796497969	0.917718424
VHLL	0.009	0.796636158	0.917811034
HMGB1P28	0.009	0.796709295	0.917873091
RP11-322N21	0.009	0.796734754	0.917880219
RNA5SP255	0.009	0.796817293	0.917908698

RP11-326L9	0.009	0.796993189	0.917996297
VAMP7	0.009	0.797017722	0.917996297
RNU6-35P	0.009	0.797084735	0.917996297
KCNMA1-AS2	0.009	0.797143907	0.917996297
RP11-284B18	0.009	0.797180082	0.918015759
RP11-356B19	0.009	0.797227459	0.91804812
AC097470	0.009	0.797296886	0.918083673
TRIM48	0.009	0.797414583	0.918135632
RN7SL259P	0.009	0.79747178	0.918135632
RNU7-169P	0.009	0.797476953	0.918135632
RP11-674I16	0.009	0.797515772	0.91814186
RP11-354B3	0.009	0.797520917	0.91814186
TEPP	0.009	0.797550728	0.918153985
LINC00392	0.009	0.797600196	0.918188739
HMGB1P46	0.009	0.797621036	0.918190537
AC005488	0.009	0.797769956	0.918287296
LINC01077	0.009	0.797818537	0.918287296
AC093267	0.009	0.797848149	0.918287296
BNIP3P3	0.009	0.797871554	0.918287296
RNU6-11P	0.009	0.797881705	0.918287296
RPL39P3	0.009	0.797883748	0.918287296
BASP1	0.009	0.797929712	0.918287296
RNA5SP479	0.009	0.798110259	0.918420733
AC010731	0.009	0.798195284	0.918452001
AL160165	0.009	0.79830438	0.918510314
RP11-785H5	0.009	0.798321071	0.918510314
CEACAMP9	0.009	0.798321313	0.918510314
VSTM1	0.009	0.798343057	0.918510314
RPL14	0.009	0.79838096	0.918510314
RP11-565F4	0.009	0.798439607	0.918524395
UBTFL2	0.009	0.798478377	0.918524395
AQP7	0.009	0.798485407	0.918524395
RP13-146A14	0.009	0.798561807	0.918524395
NKPD1	0.009	0.798589316	0.918524395
EEF1A1P37	0.009	0.798610631	0.918524395
MTND3P5	0.009	0.798651389	0.918524395
RP11-285C1	0.009	0.79869197	0.918535302
RNU6-1148P	0.009	0.798739859	0.918568196
BEND5	0.009	0.79876605	0.918576136
snoZ6	0.009	0.798880047	0.918611242
SNORD88A	0.009	0.798889284	0.918611242
AC004066	0.009	0.798893015	0.918611242
AC011239	0.009	0.798918335	0.918618178
TMEM39A	0.009	0.799126721	0.918746882
AC011363	0.009	0.799202738	0.918767743
RP4-594L9	0.009	0.799422523	0.918931507
AL160037	0.009	0.799460957	0.918931507
MIR4298	0.009	0.799488319	0.91894078
LINC00211	0.009	0.799572844	0.919015755
COX6CP11	0.009	0.799609595	0.91902171
RP11-93K7	0.009	0.799670221	0.91902171
RP11-269F19	0.009	0.799678141	0.91902171

ARMC4P1	0.009	0.799693802	0.91902171
RNLS	0.009	0.799896295	0.919099176
SLC5A8	0.009	0.799961672	0.919138222
FRG1	0.009	0.799968874	0.919138222
HDDC3	0.009	0.800034282	0.919191198
SLC35F1	0.009	0.800128808	0.919229055
ZNF626	0.009	0.800185357	0.919229055
EI24P3	0.009	0.800229328	0.919229055
RP11-546B8	0.009	0.800240959	0.919229055
RP11-228O6	0.009	0.800252405	0.919229055
MRPS21P1	0.009	0.80037133	0.919296125
SAV1	0.009	0.800376533	0.919296125
CMPK1	0.009	0.800401855	0.91930304
AL445523	0.009	0.800477373	0.919347666
PCED1A	0.009	0.80071629	0.919398926
CHMP1B2P	0.009	0.800736293	0.919398926
OR10P1	0.009	0.800767057	0.919409314
CTC-232H10	0.009	0.800904689	0.919525772
GAPDHP53	0.009	0.800944623	0.919549454
OR2AL1P	0.009	0.801062527	0.919618314
CTD-2012K14	0.009	0.801111384	0.919620754
NAT9	0.009	0.801161197	0.919620754
KDM5B	0.009	0.801207633	0.919651891
RP11-81A22	0.009	0.801323304	0.919690742
RP5-899B16	0.009	0.801334314	0.919690742
CTA-29F11	0.009	0.801336813	0.919690742
TTLL1	0.009	0.801424625	0.919745798
NPIPB9	0.009	0.801600247	0.919788948
AL355531	0.009	0.801604473	0.919788948
MIR4739	0.009	0.801611659	0.919788948
RP11-154P18	0.009	0.801662536	0.919819355
TESK1	0.009	0.801832899	0.919904359
AL354806	0.009	0.801917045	0.919960829
TAZ	0.009	0.801962951	0.919960829
MIR3134	0.009	0.80196745	0.919960829
GORASP2	0.009	0.801971396	0.919960829
MGRN1	0.009	0.801978996	0.919960829
RP11-188I24	0.009	0.802119754	0.920022124
MIR4694	0.009	0.802150244	0.920022124
PLCE1-AS2	0.009	0.802171758	0.920022124
ANKRD66	0.009	0.802187346	0.920022124
HIST1H2BM	0.009	0.80221753	0.920022124
BMPR2	0.009	0.802225603	0.920022124
MIR4417	0.009	0.802291787	0.920033602
AL080243	0.009	0.802293563	0.920033602
AC069394	0.009	0.802338273	0.920040568
GABRA6	0.009	0.802391136	0.920056881
RNA5SP63	0.009	0.80242913	0.920075665
RP5-991G20	0.009	0.802465472	0.920075665
MIR3972	0.009	0.802548725	0.920122286
ZNF707	0.009	0.802602308	0.920128109
TMEM65	0.009	0.802637362	0.920128109

RP11-797M17	0.009	0.802639249	0.920128109
RP11-620J15	0.009	0.802685087	0.920128109
OR8J3	0.009	0.802724388	0.920128867
AGPAT1	0.009	0.802760689	0.920148332
RN7SL325P	0.009	0.802904846	0.920289003
CCNYL1	0.009	0.802960473	0.920289003
RP11-62J1	0.008	0.803076642	0.920289003
ABCA17P	0.008	0.803100287	0.920293955
AC007567	0.008	0.803234025	0.920370794
PRDX3P3	0.008	0.803308128	0.920370794
RP4-565E6	0.008	0.803370442	0.920370794
AC013251	0.008	0.803640453	0.920562714
PRKY	0.008	0.803775337	0.920646641
AC104331	0.008	0.803965201	0.92080731
CKMT2	0.008	0.803980287	0.92080731
LINC00083	0.008	0.804017247	0.920813
CTD-2576F9	0.008	0.804126018	0.920915427
RP11-363H12	0.008	0.804166229	0.920922276
PROSP	0.008	0.80417067	0.920922276
RP11-177B4	0.008	0.804219563	0.920956123
RPS3AP49	0.008	0.804239388	0.920956681
AC118758	0.008	0.804482408	0.921105525
AC004004	0.008	0.804530545	0.92112202
RP11-28O3	0.008	0.804588934	0.921157624
CTD-2027I19	0.008	0.804625552	0.921176776
RP11-324C10	0.008	0.804676459	0.921176776
NANOGP11	0.008	0.804757705	0.921217979
RP11-447B18	0.008	0.804815033	0.921240719
NFKBIE	0.008	0.804835599	0.921240719
RP11-351E7	0.008	0.804932891	0.921261573
RNU6-730P	0.008	0.804973975	0.921261573
RPL11P5	0.008	0.804984478	0.921261573
CTA-360L10	0.008	0.805009769	0.921261573
RP3-354N19	0.008	0.805018112	0.921261573
RP11-799M12	0.008	0.805052965	0.9212655
RP11-745K9	0.008	0.805070212	0.9212655
CA4	0.008	0.805143515	0.921268961
PSTK	0.008	0.805213041	0.921268961
RP1-111B22	0.008	0.805260191	0.921277148
RP11-885N19	0.008	0.805287265	0.921277148
RP11-60H5	0.008	0.805292984	0.921277148
RP11-173G21	0.008	0.805447045	0.921391631
TRIM2	0.008	0.805511477	0.921413402
HPS1	0.008	0.805532614	0.921413402
KIAA1755	0.008	0.805566856	0.921413402
AC067742	0.008	0.805601262	0.921430627
LINC01097	0.008	0.805622076	0.921432305
TSPYL6	0.008	0.805704587	0.921482418
RP11-80H18	0.008	0.805775966	0.921525326
RP5-978I12	0.008	0.805784551	0.921525326
AC002368	0.008	0.805858196	0.921525326
ADCK1	0.008	0.805932125	0.921549772

CTB-105N12	0.008	0.80597574	0.921552007
IQCA1-AS1	0.008	0.805996347	0.921552007
RP11-394G3	0.008	0.805997624	0.921552007
CACNB3	0.008	0.806124501	0.921652822
LRRC19	0.008	0.806170816	0.921683649
RP11-588K22	0.008	0.806289403	0.9217971
RP11-723G8	0.008	0.806367661	0.92186444
RP11-1394O16	0.008	0.806430984	0.921892575
MIR5692B	0.008	0.806551512	0.921954101
RPEP1	0.008	0.806562235	0.921954101
CYP4A43P	0.008	0.80661325	0.92196816
CHCHD1	0.008	0.806686896	0.921982683
CTC-281M20	0.008	0.8067422	0.921982683
BDKRB1	0.008	0.806745255	0.921982683
MORF4L1	0.008	0.806745255	0.921982683
CDX2	0.008	0.806792343	0.921982683
RP5-1148A21	0.008	0.806824338	0.921982683
AC073072	0.008	0.806829902	0.921982683
AL008723	0.008	0.806854384	0.921982683
RN7SKP195	0.008	0.806858257	0.921982683
BRF1	0.008	0.806880586	0.921986076
RPL7AP7	0.008	0.806981886	0.922043909
SNRPF	0.008	0.806989277	0.922043909
PRIMA1	0.008	0.807133491	0.922164438
LINC01184	0.008	0.807232895	0.922220498
RNU6-274P	0.008	0.807260493	0.922220498
RP11-405P11	0.008	0.807481465	0.922362867
RP11-23F23	0.008	0.80756217	0.922366586
RP11-352B15	0.008	0.807889446	0.922629739
CLASRP	0.008	0.807946514	0.92265474
RP11-510C10	0.008	0.807950083	0.92265474
MYOM3	0.008	0.808040012	0.922703924
NT5C1B-RDH14	0.008	0.808105682	0.92274393
RP11-131M6	0.008	0.808151957	0.922755589
AC073628	0.008	0.808174017	0.922755589
LINC00491	0.008	0.808215363	0.922780676
MIR5187	0.008	0.808371516	0.92289259
MICG	0.008	0.8084587	0.922947878
FTH1P26	0.008	0.808551213	0.922986188
RP11-101E13	0.008	0.808574395	0.922986188
DCUN1D3	0.008	0.808576934	0.922986188
DRC1	0.008	0.808656327	0.923024923
FAM131A	0.008	0.808711496	0.923024923
RP11-406A20	0.008	0.808893449	0.923167167
SORBS2	0.008	0.808902769	0.923167167
RP11-274E6	0.008	0.808950721	0.923189739
H2AFZP4	0.008	0.809016741	0.923189739
UBE2L3	0.008	0.809085167	0.923220471
AES	0.008	0.809375925	0.923447217
RP11-30L8	0.008	0.809499296	0.923447217
EIF1P3	0.008	0.809510217	0.923447217
GPR137B	0.008	0.80952999	0.923447217

AC108488	0.008	0.809538879	0.923447217
ZNF723P	0.008	0.809560594	0.923447217
PLSCR5	0.008	0.80957813	0.923447217
MIR381HG	0.008	0.809658173	0.923469228
AC002451	0.008	0.809697653	0.923469228
RNU1-7P	0.008	0.809698457	0.923469228
RP11-260G13	0.008	0.809729742	0.923469228
RN7SL646P	0.008	0.809775437	0.923499228
CTAG2	0.008	0.809872679	0.923499559
RNA5SP469	0.008	0.810028434	0.923593292
MRPS25	0.008	0.810146327	0.923634333
RP11-119H12	0.008	0.810177661	0.923634333
RP11-418I22	0.008	0.810211098	0.923634333
RNY1P5	0.008	0.810319405	0.923699345
RP4-694B14	0.008	0.81046597	0.923844305
AC007742	0.008	0.810500875	0.923857758
RP11-63A11	0.008	0.810516568	0.923857758
RP11-92A5	0.008	0.810561197	0.923886518
CCNB3P1	0.008	0.810628768	0.923919313
ZNF835	0.008	0.810659889	0.923932673
MAS1	0.008	0.810724985	0.923975199
EHD4-AS1	0.008	0.810736002	0.923975199
RP11-409C19	0.008	0.81079969	0.923981273
RP11-675M1	0.008	0.810817878	0.923981273
ULK4	0.008	0.810916912	0.923990351
CTD-2572N17	0.008	0.810924697	0.923990351
RP11-614F17	0.008	0.811097837	0.923990351
RP11-11H9	0.008	0.811150966	0.923990351
RP11-101P17	0.008	0.811156064	0.923990351
RNU6-324P	0.008	0.811270107	0.923990351
RP11-294L13	0.008	0.811315476	0.923990351
RN7SL400P	0.008	0.811327948	0.923990351
RP11-58O3	0.008	0.811373297	0.923990351
LPP-AS1	0.008	0.811425485	0.92400922
AL035106	0.008	0.811462736	0.924029546
EPB41L4A-AS1	0.008	0.811492007	0.9240381
TMCO5B	0.008	0.811584922	0.9240381
OR6C3	0.008	0.811589625	0.9240381
UGT2A3	0.008	0.811679291	0.924069077
ABC14-1080714F14	0.008	0.811716133	0.924075061
SPINK13	0.008	0.811883111	0.924176811
RP11-120J1	0.008	0.811883129	0.924176811
PPIL6	0.008	0.811944785	0.924224906
RP11-646D13	0.008	0.812202738	0.924244323
RPL31P33	0.008	0.812237008	0.924244323
RP11-426J5	0.008	0.812319049	0.924244323
CTD-2086L14	0.008	0.812366754	0.924244323
RP11-370P15	0.008	0.812368547	0.924244323
RP13-1039J1	0.008	0.812369367	0.924244323
CCDC68	0.008	0.812409903	0.924268363
DAND5	0.008	0.812450479	0.924292446
CTD-2314G24	0.008	0.812529026	0.924359725

CTD-2016O11	0.008	0.81260132	0.924409526
CTD-2299N12	0.008	0.812670843	0.924410658
OR4F17	0.008	0.812823493	0.924465772
MIR3156-2	0.008	0.812847627	0.924465772
STARD4	0.008	0.812856462	0.924465772
SC22CB-1E7	0.008	0.812891206	0.924465772
MTND5P4	0.008	0.813153441	0.924540069
AC092675	0.008	0.813189347	0.924558821
RP5-1132H15	0.008	0.813469862	0.924767374
LYPLA2P1	0.008	0.813523712	0.924784443
RN7SL120P	0.008	0.813558856	0.924800556
RPS4XP10	0.008	0.813576721	0.924800556
PCNXL3	0.008	0.813597174	0.924801733
NOP56	0.008	0.813689563	0.924829031
SEPT7P5	0.008	0.813698862	0.924829031
LINC01010	0.008	0.813785868	0.924839103
RP11-4H14	0.008	0.813804816	0.924839103
HIGD1AP15	0.008	0.814023556	0.924994799
NONO	0.008	0.814051938	0.924994799
MIR4478	0.008	0.814134865	0.925026782
SCD5	0.008	0.814144764	0.925026782
MIR524	0.008	0.814170233	0.925033653
LA16c-60H5	0.008	0.814263414	0.925073317
GABRA2	0.008	0.814398105	0.925164545
PLG	0.008	0.81440199	0.925164545
CTD-2154H6	0.008	0.814610787	0.925288881
MLLT1	0.008	0.814628007	0.925288881
RAD21L1	0.008	0.81467521	0.925320428
HOXC5	0.008	0.814742843	0.925322074
RP11-775L16	0.008	0.814836088	0.925370747
RP11-619L12	0.008	0.814859076	0.925374788
MIR4804	0.008	0.814895184	0.925393728
AC079917	0.008	0.814933684	0.925409585
RP11-288H12	0.008	0.815039242	0.92546047
FZD10-AS1	0.008	0.815093088	0.925486066
CDK2AP2P2	0.008	0.815193547	0.925578064
AL139333	0.008	0.81523474	0.925580926
DUS2	0.008	0.815313993	0.925597422
SALL4P6	0.008	0.815457663	0.925701397
MIR525	0.008	0.81551639	0.925745998
KATNBL1P2	0.008	0.815594862	0.92581301
MT-RNR2	0.008	0.815754999	0.925899214
LGI4	0.008	0.815768569	0.925899214
CTC-527H23	0.008	0.815786593	0.925899214
MAPRE1P3	0.008	0.815787446	0.925899214
RP11-201K10	0.008	0.815842629	0.925923183
MORN2	0.008	0.815847448	0.925923183
LINC00266-4P	0.008	0.815933511	0.925954663
SLIT3	0.008	0.816037442	0.925991551
CCNT2P1	0.008	0.816165005	0.925991551
MIR656	0.008	0.81617079	0.925991551
CDH19	0.008	0.816372339	0.926121654

MIR3115	0.008	0.816430012	0.926157115
TCFL5	0.008	0.816464122	0.926159594
RP11-446H13	0.008	0.816573413	0.926165307
AC005150	0.008	0.816623423	0.926165307
RP11-24C14	0.008	0.816644174	0.926165307
SNORD14	0.008	0.816801663	0.926299803
BCAR4	0.008	0.816905821	0.926312872
LINC01567	0.008	0.816913033	0.926312872
RP11-103C3	0.008	0.816921664	0.926312872
AQP10	0.008	0.816926402	0.926312872
SLC25A5P6	0.008	0.816978344	0.926345767
MIR4524B	0.008	0.817018309	0.926369027
AC002115	0.008	0.817092401	0.926430981
SLITRK6	0.008	0.817137325	0.926459861
ST6GALNAC2P1	0.008	0.817342557	0.926645205
TEX38	0.008	0.817417878	0.926653073
RP11-45H22	0.008	0.817443933	0.926653073
AC007242	0.008	0.817598063	0.926805735
OR51A10P	0.008	0.817643162	0.926834798
RP11-756H20	0.008	0.8177639	0.926887264
RXRA	0.008	0.817788191	0.926887264
RP11-478B11	0.008	0.817812401	0.926887264
AC104002	0.008	0.81781532	0.926887264
AC091178	0.008	0.817956887	0.926954105
AC096775	0.008	0.817977569	0.926954105
MIR1305	0.008	0.817988461	0.926954105
EHBP1	0.008	0.817996118	0.926954105
RNU4-61P	0.008	0.818001429	0.926954105
LINC01471	0.008	0.818062765	0.927001554
RP11-240E2	0.008	0.81815558	0.927084671
AC011998	0.008	0.818196914	0.927087394
TMEM191B	0.008	0.818220497	0.927092059
CUTC	0.008	0.818379332	0.927184295
AC004637	0.008	0.818435701	0.927225603
ARHGAP42P5	0.008	0.818468584	0.927240801
UQCRB	0.008	0.81868916	0.927387303
KCNE1	0.008	0.818705019	0.927387303
RDH13	0.008	0.81871972	0.927387303
LRRC1	0.008	0.818753676	0.927387303
LYPD5	0.008	0.818806733	0.927403288
S100A11P1	0.008	0.818990609	0.92752976
RPS12P15	0.008	0.819107366	0.927567324
SRGAP2B	0.008	0.819311449	0.927732255
VSIG4	0.008	0.819434561	0.927815664
RP11-430H12	0.008	0.819464949	0.927817833
THADA	0.008	0.819563198	0.927864167
ASS1P5	0.008	0.819580709	0.927864167
RPSAP42	0.008	0.8195838	0.927864167
RPS27A	0.008	0.81968547	0.927898221
RNU6-973P	0.008	0.819857781	0.928041986
ZDHHC4	0.008	0.819958052	0.928111371
RP5-1101C3	0.008	0.820121852	0.928174638

ZFAND3	0.008	0.820155497	0.928174638
PLA2G3	0.008	0.820177746	0.928174638
CTD-2156G10	0.008	0.820186565	0.928174638
EFHD1	0.008	0.82026038	0.928174638
AC016739	0.008	0.820337241	0.928187626
AC009299	0.008	0.820487993	0.92830244
RP11-573E11	0.008	0.820497187	0.92830244
AC007679	0.008	0.820612826	0.928318144
RSG1	0.008	0.820615395	0.928318144
COX7A2	0.008	0.82065574	0.928318144
RP11-19D2	0.008	0.820695101	0.928318144
OR6C64P	0.008	0.820717518	0.928318144
RP11-487E1	0.008	0.820720073	0.928318144
C4orf22	0.008	0.820782672	0.928318144
AC100848	0.008	0.820892029	0.928318144
RN7SL398P	0.008	0.820900084	0.928318144
AC024908	0.008	0.820939809	0.928318144
TMEM101	0.008	0.820944539	0.928318144
ZNF205-AS1	0.008	0.820956006	0.928318144
STT3A	0.008	0.820957705	0.928318144
RP11-277P12	0.008	0.821024812	0.928348062
AL611946	0.008	0.821139415	0.928433562
RP11-289K10	0.008	0.821248706	0.92853509
CTD-2525P14	0.008	0.821284722	0.928553767
SLC26A3	0.008	0.821337394	0.928569233
RASL12	0.008	0.821439375	0.928598806
RGL1	0.008	0.821461039	0.928598806
MIRLET7I	0.008	0.821537039	0.928662677
OR51F3P	0.008	0.821757036	0.928704007
AC104695	0.008	0.821875089	0.928758242
AC006019	0.008	0.822015212	0.928894547
RP11-1046B16	0.008	0.822160473	0.9289944
RP11-509J21	0.008	0.822162092	0.9289944
ARHGDIG	0.008	0.822253077	0.929039806
RNF2P1	0.008	0.822260797	0.929039806
GALNTL5	0.008	0.822336189	0.929075107
PRKACG	0.008	0.8223532	0.929075107
OLAH	0.008	0.822383334	0.929075107
CECR5	0.008	0.822395692	0.929075107
MFSD2B	0.008	0.822433934	0.929075107
RP11-335E8	0.008	0.822525616	0.929102593
RP11-384J4	0.008	0.822530953	0.929102593
C22orf46	0.008	0.822579254	0.92911308
TUBAP	0.008	0.822612464	0.929128556
MTRNR2L3	0.008	0.822662502	0.929163037
MIR6859-2	0.008	0.822771954	0.929207926
C6orf132	0.008	0.822799796	0.929207926
RP11-420C9	0.008	0.822937958	0.929274579
ATCAY	0.008	0.823059472	0.929279072
RP11-691H4	0.008	0.823082175	0.929279072
RP11-163I18	0.008	0.823087593	0.929279072
CTBP2P4	0.008	0.823096933	0.929279072

RP1-209A6	0.008	0.823313061	0.929409198
SPAG11B	0.008	0.823382943	0.929409198
MIR607	0.008	0.823492222	0.92943895
TSPY25P	0.008	0.823561694	0.92943895
RP11-19C24	0.008	0.823613056	0.929443157
CTD-2215L10	0.008	0.823675195	0.929469234
MOS	0.008	0.823792049	0.929579073
LYPLAL1-AS1	0.008	0.823896135	0.929608425
OR8B12	0.008	0.823948423	0.929614838
GRB14	0.008	0.82394989	0.929614838
PCDHGA1	0.008	0.824043411	0.929630035
TGFA	0.008	0.824051921	0.929630035
AL354680	0.008	0.824230682	0.929769896
RP11-478P10	0.008	0.824476805	0.929911118
RP3-420J14	0.008	0.824497624	0.929912578
LRRC40	0.008	0.82457611	0.92992323
CCDC60	0.008	0.824637623	0.92992323
AL590422	0.008	0.824647522	0.92992323
RP11-102G14	0.008	0.824704509	0.929926472
C15orf38-AP3S2	0.008	0.824705195	0.929926472
RMDN2-AS1	0.008	0.824821798	0.929981549
SPG20-AS1	0.008	0.824950157	0.930029348
HMGB3P3	0.008	0.82497168	0.930029348
AL512640	0.008	0.824972177	0.930029348
RN7SKP139	0.008	0.825213451	0.930147273
ING4	0.008	0.82521349	0.930147273
CEACAM20	0.008	0.825241465	0.930150741
UMODL1-AS1	0.008	0.82529123	0.930150741
CTD-2382H12	0.008	0.825300073	0.930150741
BX470187	0.008	0.825376559	0.930198991
ITFG1	0.008	0.825415911	0.930221329
SOCS2	0.008	0.825570711	0.930351756
MOK	0.008	0.825609838	0.930371222
PLGLA	0.008	0.825712427	0.930371222
LINC01219	0.008	0.825724293	0.930371222
AL121932	0.008	0.825742778	0.930371222
RP11-797D24	0.008	0.825754311	0.930371222
FADS1	0.007	0.825763796	0.930371222
RPL35AP25	0.007	0.82590388	0.930462009
RNU6-423P	0.007	0.825931357	0.930462009
MIR3928	0.007	0.825942057	0.930462009
AL158839	0.007	0.826051167	0.930484733
RBBP8NL	0.007	0.826051312	0.930484733
AC004673	0.007	0.826055967	0.930484733
KRT8P8	0.007	0.82605963	0.930484733
LZTR1	0.007	0.826142765	0.930519767
NTF6B	0.007	0.826148509	0.930519767
LINC00454	0.007	0.826384951	0.930674845
RP11-439K3	0.007	0.826415063	0.930674845
ASNA1	0.007	0.826432443	0.930674845
VAMP5	0.007	0.826460944	0.930674845
LINC01004	0.007	0.82647796	0.930674845

MIR7111	0.007	0.826525402	0.930674845
RP11-252A24	0.007	0.826541344	0.930674845
LINC01039	0.007	0.826567133	0.93068188
AC069067	0.007	0.826691192	0.930799561
IGLL4P	0.007	0.82676292	0.930818211
ERVMER34-1	0.007	0.826766388	0.930818211
Z98941	0.007	0.826809407	0.930844639
OR52E6	0.007	0.826835421	0.930851923
RP11-34C15	0.007	0.826859353	0.930856862
MTND4P7	0.007	0.827006393	0.930962544
GPR116	0.007	0.827011869	0.930962544
RP11-3K24	0.007	0.827094657	0.931025783
RP11-403B2	0.007	0.827126692	0.931025783
GVINP2	0.007	0.82718789	0.931050661
ACTR6P1	0.007	0.827218093	0.931062652
MAP4K5	0.007	0.827440755	0.931200903
ZNF581	0.007	0.827455223	0.931200903
AC084149	0.007	0.827458236	0.931200903
CCT7P2	0.007	0.82750519	0.93122757
RP5-898I4	0.007	0.827536164	0.93122757
LINC00675	0.007	0.827542052	0.93122757
HS3ST4	0.007	0.827634032	0.93122757
AP006564	0.007	0.827689711	0.93122757
RN7SL497P	0.007	0.827694757	0.93122757
MTHFSD	0.007	0.827735645	0.93122757
RP11-558A11	0.007	0.827757386	0.93122757
HPX	0.007	0.82792374	0.93132956
LINC01311	0.007	0.82792757	0.93132956
APOA4	0.007	0.827944098	0.93132956
CTD-2195M18	0.007	0.828032238	0.931376621
RP11-384P7	0.007	0.828092646	0.931408663
MIR890	0.007	0.828239711	0.931530079
PLBD1-AS1	0.007	0.828285473	0.931559549
AC016909	0.007	0.828441785	0.931608056
MPO	0.007	0.828462528	0.931608056
OR7E86P	0.007	0.828463535	0.931608056
UGDH	0.007	0.828719635	0.93180151
COX6CP17	0.007	0.828808315	0.931851384
RP11-644C3	0.007	0.828854334	0.931858947
PSG2	0.007	0.828885532	0.931858947
AC026904	0.007	0.828940268	0.931858947
FLJ13224	0.007	0.829076593	0.931955076
AC069154	0.007	0.829107505	0.931955893
RP11-93G23	0.007	0.829209738	0.932004819
TMCO5A	0.007	0.829329923	0.932014001
AC005105	0.007	0.829348914	0.932014001
RPS26P56	0.007	0.829354891	0.932014001
RNU6-1198P	0.007	0.829390484	0.932032008
AL137797	0.007	0.829534787	0.932084207
MORF4L2P1	0.007	0.829620082	0.932123641
RN7SL83P	0.007	0.829687305	0.932123641
LINC01028	0.007	0.829746665	0.932123641

RNU6-409P	0.007	0.829763553	0.932123641
RP11-32P22	0.007	0.829793043	0.932123641
FAM122C	0.007	0.829813746	0.932123641
RP11-680F20	0.007	0.82985221	0.932123641
APOLD1	0.007	0.829862712	0.932123641
BDNF	0.007	0.829881169	0.932123641
AC013722	0.007	0.829918863	0.932123641
NATD1	0.007	0.82993893	0.932123641
TMEM198	0.007	0.829950853	0.932123641
MED4-AS1	0.007	0.829969844	0.932123641
AC096566	0.007	0.830042033	0.932128478
RP11-20L19	0.007	0.830070426	0.932128478
ITGA6	0.007	0.830096055	0.932128478
RNA5SP95	0.007	0.830102616	0.932128478
SLFNL1	0.007	0.830236485	0.932220502
FRMPD1	0.007	0.830300452	0.932247274
TMEM263	0.007	0.830334523	0.932247274
RP11-45A16	0.007	0.830384755	0.932247478
CTD-2144E22	0.007	0.830457479	0.932275182
RNU6-1153P	0.007	0.830468156	0.932275182
KLHL9	0.007	0.830594726	0.932342733
SNAP25	0.007	0.830608351	0.932342733
AC015883	0.007	0.830676868	0.932356044
MIR519C	0.007	0.830778866	0.932436753
RP11-261C10	0.007	0.830788284	0.932436753
MIR6823	0.007	0.830818664	0.932448876
ARL13B	0.007	0.830843874	0.932455197
RNU6-96P	0.007	0.830875044	0.932466064
C7orf62	0.007	0.830892714	0.932466064
ZBED1P1	0.007	0.830942555	0.932500025
ARF1P1	0.007	0.831048596	0.932597052
CH17-38B12	0.007	0.831231077	0.932703487
AL139151	0.007	0.831280526	0.932703487
ZNF529-AS1	0.007	0.831329016	0.932707457
RAD51L3-RFFL	0.007	0.831362399	0.932707457
VDAC1P7	0.007	0.831465363	0.932801
RP1-67K17	0.007	0.831549249	0.932873135
CDCA4P2	0.007	0.831694134	0.932973946
FZR1	0.007	0.831746485	0.932973946
TUBB8P7	0.007	0.831784776	0.932983527
LAMTOR3P1	0.007	0.831906684	0.93301822
TEX261	0.007	0.831941605	0.933027593
RNU6-1305P	0.007	0.832090444	0.933103702
SIGLEC15	0.007	0.83210265	0.933103702
LRRC9	0.007	0.832123329	0.933103702
ARMC4	0.007	0.832216947	0.933125342
RP1-309K20	0.007	0.83222344	0.933125342
OR7H1P	0.007	0.832224687	0.933125342
ATP6AP2	0.007	0.83231824	0.933163153
PRR19	0.007	0.832365109	0.933163153
RP11-477L16	0.007	0.832375968	0.933163153
EFNA5	0.007	0.832466945	0.933196865

RP11-285G1	0.007	0.8324688	0.933196865
OR5B15P	0.007	0.832576201	0.933223146
MIR93	0.007	0.832586236	0.933223146
RN7SKP132	0.007	0.832678157	0.933260286
RNA5SP258	0.007	0.83271923	0.933269294
GOLGA8J	0.007	0.832725385	0.933269294
RN7SKP288	0.007	0.832750664	0.933275664
ABHD4	0.007	0.832851304	0.933322566
UGT1A13P	0.007	0.83292822	0.933369157
AC010136	0.007	0.832962067	0.933369157
DGCR5	0.007	0.832990867	0.933369157
RP11-461F11	0.007	0.833082409	0.933422448
COL21A1	0.007	0.833144927	0.933422448
RP1-35C21	0.007	0.833165952	0.933422448
Z73964	0.007	0.833172843	0.933422448
NHP2L1	0.007	0.833195216	0.933422448
CTD-2265D6	0.007	0.833283383	0.933433395
RP11-541M12	0.007	0.833673996	0.933698929
RP11-496I2	0.007	0.833684141	0.933698929
LRP6	0.007	0.833716471	0.933698929
FKBP1AP2	0.007	0.833803047	0.933745254
LRRC3C	0.007	0.8338478	0.933745254
NAV2-IT1	0.007	0.83389145	0.933745254
AC097463	0.007	0.833903066	0.933745254
ZDHHC11	0.007	0.833914678	0.933745254
REG3G	0.007	0.833943183	0.933748204
MIR6870	0.007	0.833956605	0.933748204
MCCD1P2	0.007	0.833976129	0.933748204
DEF8	0.007	0.834319224	0.933982606
MTRNR2L5	0.007	0.834370619	0.933990581
HINT1	0.007	0.834427934	0.933990581
RP11-307P5	0.007	0.834586403	0.934106526
RP11-393J16	0.007	0.83469841	0.934106526
RP11-428F8	0.007	0.834725638	0.934106526
EDAR	0.007	0.834745712	0.934106526
RPS4XP13	0.007	0.834746413	0.934106526
PSPN	0.007	0.834771991	0.934106526
RN7SL155P	0.007	0.834784622	0.934106526
SLC25A48	0.007	0.834844198	0.934106526
RPL18AP16	0.007	0.834856029	0.934106526
STX17-AS1	0.007	0.834866218	0.934106526
RNU5E-3P	0.007	0.834892274	0.934106526
SDHAP1	0.007	0.834904165	0.934106526
RP5-843L14	0.007	0.834966077	0.934144275
RP11-492D6	0.007	0.83505892	0.934144275
HNRNPCP10	0.007	0.835077333	0.934144275
MYADM	0.007	0.835172365	0.934194192
FCF1	0.007	0.835304554	0.93428635
SNORD115-8	0.007	0.835405081	0.934293848
OR2L9P	0.007	0.835426059	0.93429537
PRPF19	0.007	0.835524167	0.934361208
UCK1	0.007	0.835583443	0.934405557

RP4-551E13	0.007	0.835634231	0.934418471
RP11-70J12	0.007	0.835724811	0.93442483
SLC16A1-AS1	0.007	0.835778337	0.93442483
NAA50	0.007	0.835800941	0.93442483
CELSR3-AS1	0.007	0.835940786	0.93442483
RP11-560I19	0.007	0.83598287	0.93442483
RP11-689C9	0.007	0.836006212	0.93442483
LAPTM4B	0.007	0.836046173	0.93442483
EPS8L2	0.007	0.8360466	0.93442483
TRMT1	0.007	0.836082853	0.93442483
C12orf5	0.007	0.836102046	0.93442483
DMD	0.007	0.8361174	0.93442483
RP11-47G4	0.007	0.836130433	0.93442483
CTB-140J7	0.007	0.836146537	0.93442483
LRRC6P1	0.007	0.836150029	0.93442483
RP11-528A10	0.007	0.836174425	0.934429637
EIF1P6	0.007	0.836195244	0.934429637
RP11-106E7	0.007	0.836213189	0.934429637
PRR15L	0.007	0.836237252	0.934434602
RP1-97J1	0.007	0.836415336	0.934569478
RP11-843B15	0.007	0.836427244	0.934569478
GCHFR	0.007	0.836436445	0.934569478
RP11-104O19	0.007	0.836682618	0.934734889
RNU6-561P	0.007	0.836753031	0.934791626
AC007096	0.007	0.836885408	0.934798895
RN7SL741P	0.007	0.836921818	0.934798895
SLC12A7	0.007	0.836975642	0.93479912
XX-FYM637E10_5	0.007	0.837082752	0.934874903
AC151960	0.007	0.837182389	0.934964256
LINC00446	0.007	0.837231741	0.934997447
CH17-340M24	0.007	0.837277266	0.935024613
RNU6-1031P	0.007	0.837360183	0.935024613
AC093824	0.007	0.837416268	0.935024613
MIR8066	0.007	0.837426064	0.935024613
DIRC2	0.007	0.837549048	0.935103524
IGHVII-62-1	0.007	0.837605249	0.935129517
RN7SL810P	0.007	0.837676501	0.935165222
RN7SKP254	0.007	0.837743193	0.935173915
MIR5692C1	0.007	0.837809726	0.935204346
RP11-439I14	0.007	0.838107375	0.935454854
SMIM9	0.007	0.838369017	0.935631296
ARPC5L	0.007	0.838433218	0.935659096
PROSER2	0.007	0.838502356	0.935696725
RP11-321G12	0.007	0.838512948	0.935696725
RRP36	0.007	0.838536925	0.935696725
CBWD3	0.007	0.838895867	0.935822156
NAMA_1	0.007	0.838908776	0.935822156
FANCC	0.007	0.838913367	0.935822156
RNU6-494P	0.007	0.838980855	0.935875519
PVRL2	0.007	0.839008124	0.935879947
AC109351	0.007	0.839024124	0.935879947
AC092380	0.007	0.839353975	0.936104809

PROK2	0.007	0.839383213	0.936104809
AC090060	0.007	0.839481705	0.936149176
MIR6088	0.007	0.83962699	0.936223507
THA1P	0.007	0.839711901	0.936236356
GEMIN7	0.007	0.839736802	0.936236356
MED6P1	0.007	0.839824661	0.936272872
TEX33	0.007	0.839848187	0.936272872
RP11-268G13	0.007	0.839926529	0.936319768
DCXR	0.007	0.840017723	0.936368034
RNF7P1	0.007	0.840031852	0.936368034
XX-C2158C6	0.007	0.840067974	0.936386383
CTD-2147F2	0.007	0.840101312	0.936401628
SIX2	0.007	0.840143248	0.936426456
AC010492	0.007	0.840215242	0.936440955
GARNL3	0.007	0.840266215	0.936453937
TAGLN2P1	0.007	0.840337517	0.936489574
THOC7-AS1	0.007	0.840413455	0.936530373
RN7SL117P	0.007	0.840565129	0.9366358
RPL23	0.007	0.84073378	0.9366358
RP11-755B10	0.007	0.840750007	0.9366358
CTC-458G6	0.007	0.840772975	0.9366358
RPL30P12	0.007	0.840800789	0.9366358
RNU6-820P	0.007	0.840801714	0.9366358
RP11-286O1	0.007	0.841001308	0.93680726
VDAC1P3	0.007	0.841169699	0.936934663
RPS3AP46	0.007	0.841525946	0.937221875
HAP1	0.007	0.841576391	0.937256139
RP3-405J10	0.007	0.841608855	0.93725978
NUDT4P1	0.007	0.841624898	0.93725978
AC093859	0.007	0.841638698	0.93725978
RP11-69L16	0.007	0.84167805	0.937270422
HOXA6	0.007	0.841703497	0.937270422
RP11-223J15	0.007	0.841713496	0.937270422
ERICH1	0.007	0.841798929	0.937270422
BPIFB2	0.007	0.841825501	0.937270422
MAGEA6	0.007	0.841904087	0.937270422
SHANK3	0.007	0.842042835	0.937312146
HNRNPA1P3	0.007	0.842050208	0.937312146
MGLL	0.007	0.842100076	0.937312146
ACSM5P1	0.007	0.842159409	0.937312146
TOLLIP	0.007	0.842196192	0.937312146
RNU6-1098P	0.007	0.842197409	0.937312146
RP3-332B22	0.007	0.842244327	0.937315531
MTND1P3	0.007	0.842423955	0.937412655
AC010196	0.007	0.842473896	0.937444611
PRICKLE2-AS2	0.007	0.842523724	0.937453079
AC128686	0.007	0.842585649	0.937455921
AC012358	0.007	0.842619128	0.937455921
AL390335	0.007	0.842639683	0.937455921
RP11-363J17	0.007	0.842656991	0.937455921
CALML3-AS1	0.007	0.84266121	0.937455921
RP3-508D13	0.007	0.842702102	0.937479516

RNU6-837P	0.007	0.842759611	0.937492061
RP11-1198D22	0.007	0.842795633	0.937492061
AC091962	0.007	0.842862905	0.937505116
ZNF710	0.007	0.84289812	0.937522391
ATP5G1P3	0.007	0.843014583	0.937541696
POU6F2-AS1	0.007	0.843115717	0.937611139
CSN3	0.007	0.84341738	0.937883208
RP11-335L23	0.007	0.843537752	0.937961294
RP11-510J16	0.007	0.843568439	0.937961294
RNU6-530P	0.007	0.843729336	0.937982679
PFN4	0.007	0.84373436	0.937982679
CTD-2130F23	0.007	0.843962245	0.938179123
CYP4F60P	0.007	0.844208581	0.938388268
CTC-448F2	0.007	0.844259182	0.938421458
RP11-503P10	0.007	0.844314929	0.938421458
MUC17	0.007	0.844317255	0.938421458
WASH2P	0.007	0.844531451	0.93849757
RN7SKP110	0.007	0.844531749	0.93849757
MIR3940	0.007	0.844561691	0.93849757
KCNE3	0.007	0.844660539	0.938531054
RN7SL52P	0.007	0.844719993	0.938540548
NPB	0.007	0.845090763	0.938815744
CDC14C	0.007	0.845106248	0.938815744
CYP4B1	0.007	0.845123908	0.938815744
UBAC1	0.007	0.845138885	0.938815744
MIR613	0.007	0.845205097	0.938815744
CPA3	0.007	0.845218394	0.938815744
SLC31A1	0.007	0.8452479	0.938815744
OR2C3	0.007	0.845267184	0.938815744
RPL19P18	0.007	0.845380581	0.938828799
RN7SL241P	0.007	0.845392928	0.938828799
RP11-538C21	0.007	0.845448025	0.938828799
RN7SKP44	0.007	0.84545252	0.938828799
USP17L15	0.007	0.845616245	0.938966821
OR4A10P	0.007	0.845652307	0.938984973
POU5F1P3	0.007	0.845685465	0.938999898
RP11-289F5	0.007	0.845733401	0.93900347
MIR5739	0.007	0.845742986	0.93900347
WDR5	0.007	0.845762659	0.93900347
GPR97	0.007	0.845791923	0.939008646
CLDND2	0.007	0.845929413	0.939118906
RNU6-1194P	0.007	0.845948237	0.939118906
RP11-91N2	0.007	0.846004697	0.93913712
DPY19L2P1	0.007	0.846006236	0.93913712
ZBTB20-AS2	0.007	0.846132326	0.939211418
AC090018	0.007	0.846191579	0.939255298
BET1	0.007	0.846341628	0.939356173
TIMP4	0.007	0.846366424	0.939361802
DDX11L8	0.007	0.846425983	0.939400907
REG1P	0.007	0.846441105	0.939400907
CTD-2009A10	0.007	0.846476412	0.939418201
CTA-228A9	0.007	0.84659522	0.939509057

AP000745	0.007	0.846607457	0.939509057
NAB1	0.007	0.846617459	0.939509057
MOV10L1	0.007	0.846650802	0.939524168
LMO3	0.007	0.846691872	0.939547851
PGAM1P4	0.007	0.846741852	0.939581421
AL133244	0.007	0.846766455	0.939586831
OR4D12P	0.007	0.846916696	0.939665971
ATP5C1P1	0.007	0.847101157	0.939804952
AC112219	0.007	0.847268732	0.939968971
C15orf32	0.007	0.84732907	0.939981333
TECR	0.007	0.847469444	0.940019997
RP11-191A15	0.007	0.847472623	0.940019997
CTD-2203K17	0.007	0.847499387	0.94002779
OR7G15P	0.007	0.847581119	0.940034765
RP11-157E16	0.007	0.847638464	0.940034765
KNOP1	0.007	0.847678618	0.940034765
AC087392	0.007	0.84768357	0.940034765
FRMPD3-AS1	0.007	0.847715538	0.940034765
OR5AR1	0.007	0.847725359	0.940034765
KRTAP5-14P	0.007	0.847742524	0.940034765
CHCHD4P4	0.007	0.847807976	0.940073423
CDH26	0.007	0.847859997	0.940087339
ZC3H12B	0.007	0.847918177	0.940087339
RALGAPA2	0.007	0.848108978	0.940100741
UBE2I	0.007	0.848144915	0.940100741
MORF4	0.007	0.848151557	0.940100741
CUBNP2	0.007	0.848153396	0.940100741
OTUD5	0.007	0.848157321	0.940100741
AC092660	0.007	0.848243378	0.940174246
SMIM4	0.007	0.848294654	0.940183941
AP001601	0.007	0.848371061	0.940206364
CPO	0.007	0.848435689	0.940234231
C9orf153	0.007	0.848600791	0.940329677
RN7SKP89	0.007	0.848666381	0.940380478
RP11-17E15	0.006	0.848761048	0.940421363
AL356479	0.006	0.848762515	0.940421363
OLA1P1	0.006	0.848907787	0.940538562
RP11-308B5	0.006	0.849015656	0.940582157
RPL5P6	0.006	0.84902613	0.940582157
RPS27L	0.006	0.849154692	0.94068082
RNU6-164P	0.006	0.849247071	0.940719396
PIK3R1	0.006	0.849268521	0.940719396
AL139340	0.006	0.849545345	0.940916612
RP11-442G21	0.006	0.849584844	0.940938478
AC116038	0.006	0.84979905	0.941131946
UBTFL11	0.006	0.849882732	0.94119517
SPANXA2	0.006	0.849895661	0.94119517
BCL6	0.006	0.850080823	0.941310196
HBG2	0.006	0.850150168	0.941310196
RNU6-1242P	0.006	0.850156227	0.941310196
PCDHGA4	0.006	0.850179266	0.941310196
LAMTOR5	0.006	0.850266588	0.941315161

CTD-2571L23	0.006	0.850376941	0.941377082
CTB-161C1	0.006	0.85051539	0.941474523
RN7SL38P	0.006	0.850523502	0.941474523
AC087653	0.006	0.850813119	0.941641913
RAI1	0.006	0.850899277	0.941650625
IL4I1	0.006	0.851014867	0.941722887
SDR16C5	0.006	0.851027181	0.941722887
ATP5SL	0.006	0.851045692	0.941722887
RNU6-629P	0.006	0.851370677	0.941816029
UFC1	0.006	0.851443433	0.941858004
ATP5LP2	0.006	0.851539073	0.941876296
RN7SL87P	0.006	0.851667456	0.941974546
POLR3DP1	0.006	0.851748791	0.941997774
KRASP1	0.006	0.851882046	0.941998224
yR211F11	0.006	0.851891853	0.941998224
BEND7	0.006	0.85197053	0.941998224
VN1R5	0.006	0.851980917	0.941998224
RPL6	0.006	0.851994082	0.941998224
AC025300	0.006	0.852035852	0.941998224
RP1-34H18	0.006	0.852045097	0.941998224
RP11-307B6	0.006	0.852059847	0.941998224
SETD1A	0.006	0.852084437	0.941998224
ZNF702P	0.006	0.85215852	0.942045351
RN7SKP71	0.006	0.852166625	0.942045351
THEM4	0.006	0.852292559	0.942140831
HAUS8P1	0.006	0.852493845	0.942182078
FAM92A1P1	0.006	0.852709568	0.942182078
OR4A8	0.006	0.852716654	0.942182078
RNU7-116P	0.006	0.852727618	0.942182078
COLCA1	0.006	0.852770506	0.942182078
PIH1D2	0.006	0.852826616	0.942182078
STAT5A	0.006	0.852848459	0.942182078
TPTE	0.006	0.852860358	0.942182078
AL133391	0.006	0.852864056	0.942182078
RP11-546O6	0.006	0.85290587	0.942182078
TMEM167A	0.006	0.852929409	0.942182078
RN7SL549P	0.006	0.853013463	0.942182078
RNU6-673P	0.006	0.853013571	0.942182078
PPP4R4	0.006	0.853027421	0.942182078
AC097711	0.006	0.853055883	0.942182078
RP11-297A16	0.006	0.853070094	0.942182078
PPP2R2D	0.006	0.853080177	0.942182078
AL360297	0.006	0.853101391	0.942182078
C14orf119	0.006	0.853189401	0.94223558
DNAH6	0.006	0.853235662	0.94226482
RBMY2GP	0.006	0.853291079	0.94229111
FAM90A24P	0.006	0.853362987	0.942339879
RNU6-1190P	0.006	0.853426094	0.942385464
CTBP2P2	0.006	0.853551758	0.942397531
AC008067	0.006	0.853593007	0.942401212
SNORD115-11	0.006	0.853722058	0.942474166
RNU4ATAC17P	0.006	0.853808453	0.942525848

SYTL4	0.006	0.853899215	0.942563515
TLX2	0.006	0.854012356	0.942575601
RP11-139E19	0.006	0.854020188	0.942575601
RP11-510D21	0.006	0.854047452	0.942575601
RN7SL306P	0.006	0.854091012	0.942575601
GPC5-AS2	0.006	0.854118307	0.942583882
RP11-756P10	0.006	0.85430665	0.942748043
FAM13A	0.006	0.854481461	0.942860071
SPATA33	0.006	0.854565441	0.942860071
RP4-686C3	0.006	0.854569797	0.942860071
OR1K1	0.006	0.854578662	0.942860071
OR7A18P	0.006	0.854587122	0.942860071
ST13P16	0.006	0.854591624	0.942860071
RP11-555K2	0.006	0.854624944	0.942860071
CNGA3	0.006	0.854632539	0.942860071
RP11-293A10	0.006	0.854645729	0.942860071
IL6STP1	0.006	0.854728953	0.942908203
RTN4	0.006	0.854793396	0.942940778
RAB4B-EGLN2	0.006	0.854798079	0.942940778
CRPP1	0.006	0.854857926	0.942984955
SH3GL3	0.006	0.854909786	0.943003387
C7orf60	0.006	0.854914234	0.943003387
RP11-273G15	0.006	0.855239914	0.943190438
TMEM161BP1	0.006	0.855285427	0.943190438
RN7SKP91	0.006	0.855347467	0.943190438
RP1-69B13	0.006	0.855366279	0.943190438
AC092022	0.006	0.855370893	0.943190438
LINC00865	0.006	0.855378764	0.943190438
CCDC106	0.006	0.855420762	0.943212591
RNU6-1086P	0.006	0.85545416	0.943227579
RP11-115D7	0.006	0.855581333	0.943240954
HSPE1P27	0.006	0.855610657	0.943240954
RP11-277J6	0.006	0.855627798	0.943240954
RN7SL193P	0.006	0.855822778	0.943371025
C12orf50	0.006	0.855887814	0.943371025
MIR101-1	0.006	0.855911993	0.943371025
RP11-239L20	0.006	0.855977779	0.943371025
DMBT1P1	0.006	0.855987766	0.943371025
GHDC	0.006	0.856070937	0.943405306
KIF3C	0.006	0.856253964	0.943585172
ACTR3P3	0.006	0.856305321	0.943619933
OR4K2	0.006	0.856329845	0.943625125
SPTLC1P1	0.006	0.856359462	0.943631989
SEMA3A	0.006	0.856496154	0.943663434
OR1L3	0.006	0.856521169	0.943663434
AL596243	0.006	0.856626329	0.943734582
RP11-136K14	0.006	0.856656158	0.94374261
SMPD4P1	0.006	0.856723025	0.943765322
RNU7-115P	0.006	0.856734493	0.943765322
TNP1	0.006	0.856900035	0.943907511
IFI16	0.006	0.856903835	0.943907511
BARHL1	0.006	0.856969152	0.943907511

AC003986	0.006	0.856974929	0.943907511
E4F1	0.006	0.857107475	0.943909397
LHFPL3-AS1	0.006	0.857128448	0.943909397
MIR7-3	0.006	0.857142745	0.943909397
MIR4716	0.006	0.857204796	0.943931533
RPL37P10	0.006	0.85743257	0.944019137
COX6A1	0.006	0.857440608	0.944019137
PCDH1	0.006	0.85753708	0.944052192
DDX11L1	0.006	0.857550166	0.944052192
OR14L1P	0.006	0.857574485	0.944052192
OGDHL	0.006	0.857592695	0.944052192
DHRS4L2	0.006	0.857679005	0.944052192
HMGB3P20	0.006	0.85770665	0.944052192
AP000577	0.006	0.857707487	0.944052192
PPARGC1B	0.006	0.857728315	0.944052192
SNHG15	0.006	0.857728315	0.944052192
RP11-479A21	0.006	0.857830212	0.944110578
FDPSP1	0.006	0.858095585	0.944276738
RP11-503L23	0.006	0.858138567	0.944276738
AC007237	0.006	0.858211317	0.944276738
RNU6-390P	0.006	0.858240652	0.944276738
AKR1B1P4	0.006	0.858264099	0.944276738
CEACAMP10	0.006	0.858320228	0.944289056
HMGB3P22	0.006	0.858425579	0.944294358
GLTPD2	0.006	0.858427723	0.944294358
RP11-168K9	0.006	0.858762472	0.944513781
VASH2	0.006	0.858835584	0.944547243
SIN3B	0.006	0.8589308	0.944589849
CYP4X1	0.006	0.858953103	0.944592566
RPL36AP39	0.006	0.859029591	0.944647331
PRM2	0.006	0.859042571	0.944647331
LPHN3	0.006	0.859120379	0.944655055
CTD-2015H3	0.006	0.859126591	0.944655055
CTC-459M5	0.006	0.859169056	0.944655055
MINOS1-NBL1	0.006	0.859211963	0.944655055
SLC16A7	0.006	0.859277368	0.944655055
GOLPH3L	0.006	0.859285947	0.944655055
RN7SL788P	0.006	0.8593507	0.944655055
RP11-145G20	0.006	0.859366946	0.944655055
RN7SL214P	0.006	0.859656361	0.944864154
CRYBB1	0.006	0.859820008	0.94490128
RP11-394I13	0.006	0.859829017	0.94490128
RNU6-1047P	0.006	0.859933866	0.944971484
AL109805	0.006	0.859992006	0.944971484
ARHGEF26-AS1	0.006	0.859994179	0.944971484
OR4C1P	0.006	0.860017039	0.944971484
MIR602	0.006	0.860076387	0.944971484
PITPNA-AS1	0.006	0.860110873	0.944971484
CCDC154	0.006	0.860128034	0.944971484
HSD3B1	0.006	0.860170478	0.944971484
DEFB118	0.006	0.860181119	0.944971484
CTBP1	0.006	0.860268325	0.944971484

UBDP1	0.006	0.860275753	0.944971484
FOXO3B	0.006	0.86028377	0.944971484
TPD52L3	0.006	0.860319439	0.944971484
RPL18AP15	0.006	0.860331085	0.944971484
RBM38	0.006	0.860385882	0.944971484
RP11-224P11	0.006	0.860428609	0.944971484
RP11-461M2	0.006	0.860606458	0.945047184
RN7SKP136	0.006	0.860616592	0.945047184
RBBP4P3	0.006	0.860906695	0.945244136
MRPL18	0.006	0.860958264	0.945244136
NAA20	0.006	0.861043656	0.945276403
RP11-164H5	0.006	0.861163411	0.945294061
AR	0.006	0.86124577	0.945345755
AC009411	0.006	0.861312054	0.945353403
AOC3	0.006	0.861312286	0.945353403
RP6-24A23	0.006	0.861348672	0.94536541
AC005255	0.006	0.861362924	0.94536541
RP11-384P14	0.006	0.8614252	0.945385113
MIR3167	0.006	0.861440425	0.945385113
DNAJC8	0.006	0.861475359	0.945401301
MIR548X	0.006	0.861675484	0.945512355
RNU6-342P	0.006	0.861745347	0.945539069
OR4G1P	0.006	0.861842748	0.945565183
AC000003	0.006	0.861995943	0.945667906
LACRT	0.006	0.862273668	0.945873806
RNU1-142P	0.006	0.862346507	0.945916576
SNRPGP13	0.006	0.862484109	0.945963768
DUX4L17	0.006	0.862545469	0.946009281
RP11-215A21	0.006	0.86269173	0.946065086
SLC25A30-AS1	0.006	0.86269922	0.946065086
SPATA17	0.006	0.862702462	0.946065086
AL450327	0.006	0.86272296	0.946065086
RN7SL100P	0.006	0.86274842	0.946065086
RP11-722P11	0.006	0.862866151	0.946121332
RP11-923I11	0.006	0.862906797	0.946122336
DLX6	0.006	0.862965081	0.946143099
VN1R107P	0.006	0.862965466	0.946143099
D21S2088E	0.006	0.863094281	0.946256811
AC062031	0.006	0.863206448	0.946256811
CDH18	0.006	0.863210203	0.946256811
VKORC1L1	0.006	0.863225337	0.946256811
RNU2-10P	0.006	0.863232324	0.946256811
CDRT15L2	0.006	0.86324506	0.946256811
MUTYH	0.006	0.863251096	0.946256811
AC095056	0.006	0.863329211	0.946256811
HCG17	0.006	0.863351668	0.946256811
RN7SL836P	0.006	0.863375958	0.946256811
NPM1P12	0.006	0.863586534	0.946256811
NANOG	0.006	0.863615539	0.946256811
ODF4	0.006	0.863637444	0.946256811
ACA64	0.006	0.863665223	0.946256811
CTB-167G5	0.006	0.863747653	0.946303585

CRYBA1	0.006	0.863843906	0.946365499
AC009800	0.006	0.863942255	0.94641176
TBATA	0.006	0.863954539	0.94641176
NPRL3	0.006	0.863956517	0.94641176
TMEM63C	0.006	0.86398228	0.94641176
HMGB1P12	0.006	0.864010319	0.94641176
MARCKSL1P2	0.006	0.864199847	0.946494184
TRIM16L	0.006	0.864239919	0.946516306
SLC7A11-AS1	0.006	0.864320751	0.946546076
RN7SL712P	0.006	0.864387174	0.946546076
RNU6-690P	0.006	0.864419912	0.946546076
RN7SKP119	0.006	0.864420859	0.946546076
CCT8	0.006	0.864422851	0.946546076
MORN3	0.006	0.864433586	0.946546076
SOCS5P3	0.006	0.86463554	0.946605948
ANO7	0.006	0.864643581	0.946605948
RP3-355L5	0.006	0.864662047	0.946605948
TMEM249	0.006	0.864753446	0.946665112
RNU4-70P	0.006	0.864865834	0.946688129
LINC01378	0.006	0.864873857	0.946688129
RP4-587D13	0.006	0.864896878	0.94669157
RNU6-755P	0.006	0.865026587	0.946746513
RNA5SP295	0.006	0.865063606	0.946765272
ST6GAL1	0.006	0.865103112	0.946772614
RN7SL681P	0.006	0.865158843	0.946772614
RPL21P112	0.006	0.865382349	0.94695956
RP11-10G15	0.006	0.86540019	0.94695956
PNMT	0.006	0.865468934	0.947012404
RP11-504A18	0.006	0.865488251	0.947012404
BLOC1S5-TXNDC5	0.006	0.865799775	0.947266221
RNU2-37P	0.006	0.865841942	0.947268835
DSP	0.006	0.865878834	0.947287436
OR1L6	0.006	0.865914618	0.947304824
RN7SKP171	0.006	0.865967457	0.947312801
RP11-277K23	0.006	0.865999145	0.947312801
PCYT1B-AS1	0.006	0.866063361	0.947312801
RP11-155D18	0.006	0.866151333	0.947346183
RP4-761J14	0.006	0.866199298	0.947376888
CTB-30L5	0.006	0.866344051	0.947489447
C12orf56	0.006	0.866361894	0.947489447
RN7SL263P	0.006	0.866514573	0.947634663
FTH1P5	0.006	0.866611723	0.947654015
NPM1P45	0.006	0.86663507	0.947654015
CYB561D2	0.006	0.86675608	0.947657059
GSTA12P	0.006	0.866791127	0.947657059
CTD-2146O16	0.006	0.866841834	0.947657059
RP11-390D11	0.006	0.866921414	0.947709647
SLC25A1P5	0.006	0.867063793	0.947725389
PPP1R32	0.006	0.867342577	0.947856863
FTMT	0.006	0.867425283	0.947856863
CTD-2530H12	0.006	0.86752735	0.947856863
DRD5	0.006	0.867531293	0.947856863

RNU6-182P	0.006	0.867539799	0.947856863
HCG19P	0.006	0.86760032	0.947886138
RN7SL415P	0.006	0.867751081	0.948007357
AC010145	0.006	0.867955196	0.948117282
RP11-539D10	0.006	0.867963413	0.948117282
HPRT1	0.006	0.868087298	0.948170485
NUTM2A	0.006	0.868155564	0.948170485
UBE2V2P1	0.006	0.868157946	0.948170485
NSA2	0.006	0.868181846	0.948170485
RP11-354E11	0.006	0.868217751	0.948170485
COG4	0.006	0.86830906	0.948170485
TRIML1	0.006	0.868755259	0.948450626
HMGB3P27	0.006	0.86876686	0.948450626
MRP63P3	0.006	0.868780661	0.948450626
RP11-530I17	0.006	0.868794076	0.948450626
RASSF8-AS1	0.006	0.868873828	0.948515949
RP4-697K14	0.006	0.86889431	0.948516567
RP3-523E19	0.006	0.86892273	0.94852585
ULK4P2	0.006	0.868954243	0.94853851
LRRC41	0.006	0.868990315	0.948556145
OR7E99P	0.006	0.869018612	0.948565293
LPCAT3	0.006	0.869097777	0.948623248
ARL5AP2	0.006	0.869111542	0.948623248
BEX1	0.006	0.869147081	0.948633761
NPIPA2	0.006	0.869352373	0.948741497
KIF2B	0.006	0.869547058	0.948810315
MANF	0.006	0.869548715	0.948810315
LA16c-360A4	0.006	0.869649952	0.948867917
GSTTP2	0.006	0.869734084	0.948933044
RPSAP51	0.006	0.869789081	0.948949571
FP325317	0.006	0.869853635	0.948998261
MAPK6PS2	0.006	0.86990821	0.949014324
RP11-758P17	0.006	0.869977279	0.949067934
SDHDP1	0.006	0.870120388	0.949137092
AC011754	0.006	0.870417543	0.94938074
LINC00114	0.006	0.870469894	0.949403487
ENPP1	0.006	0.87062701	0.949499671
CTD-2377D24	0.006	0.87067109	0.949499671
CCDC22	0.006	0.870756009	0.949525974
RNU6-811P	0.006	0.870869373	0.949584368
AC104698	0.006	0.870921649	0.949589608
RP11-513G19	0.006	0.870940428	0.949589608
MIR4490	0.006	0.871116904	0.949659117
RNU6-842P	0.006	0.871126918	0.949659117
LA16c-425C2	0.006	0.87113732	0.949659117
NSUN5P1	0.006	0.87118861	0.949680371
RP4-654H19	0.006	0.871196697	0.949680371
RN7SL48P	0.006	0.871260766	0.949699001
Pinc	0.006	0.87135611	0.949699001
VN1R81P	0.006	0.87137331	0.949699001
REV3L-IT1	0.006	0.871424422	0.949732973
CTNS	0.006	0.871515882	0.94975655

RP11-201M22	0.006	0.871726681	0.949850768
MIR4446	0.006	0.871743039	0.949850768
TCEB2P3	0.005	0.871770627	0.949850768
RP4-605O3	0.005	0.871953282	0.949945907
TUBA4B	0.005	0.871958873	0.949945907
A1BG	0.005	0.872170061	0.950097778
SKIL	0.005	0.872198019	0.950097778
LILRA6	0.005	0.872239742	0.950121496
BST2	0.005	0.87233093	0.950155633
MIR3919	0.005	0.872377107	0.950184199
HTRA2	0.005	0.872478041	0.950250672
PYROXD2	0.005	0.872499979	0.950252835
PTPN3	0.005	0.872650966	0.950259164
SULT2B1	0.005	0.872660859	0.950259164
EEF1A1P2	0.005	0.872680433	0.950259164
MIR3912	0.005	0.87271364	0.950259164
FBXO46	0.005	0.872734419	0.950259164
KCNA7	0.005	0.87273616	0.950259164
AC010524	0.005	0.872747322	0.950259164
RP11-126K1	0.005	0.872765392	0.950259164
RP11-385D13	0.005	0.872803683	0.950259164
RP11-293P20	0.005	0.872812018	0.950259164
AL121756	0.005	0.872828168	0.950259164
TSNAXIP1	0.005	0.872937036	0.950280415
CDH6	0.005	0.872948651	0.950280415
RPSAP35	0.005	0.872964258	0.950280415
RP11-227H4	0.005	0.873081532	0.95036923
RP11-447G11	0.005	0.873186362	0.950372137
RNU6-1340P	0.005	0.873188198	0.950372137
MEP1AP4	0.005	0.873223642	0.950388995
CT45A9	0.005	0.873270507	0.950418281
RP13-16D10	0.005	0.873326028	0.950456989
CHN1	0.005	0.873377574	0.950469649
AC012640	0.005	0.873456581	0.950512192
DNAL4	0.005	0.873714027	0.950599185
RP11-525A16	0.005	0.873716775	0.950599185
RP11-520D19	0.005	0.873763215	0.950599185
RNF2	0.005	0.873788893	0.950599185
PDZD7	0.005	0.873802661	0.950599185
FAM71D	0.005	0.873815952	0.950599185
RP11-508N12	0.005	0.873923076	0.950672293
RPL26P31	0.005	0.874262875	0.950808509
AC009542	0.005	0.874265231	0.950808509
MIR663A	0.005	0.874272443	0.950808509
RP11-492C2	0.005	0.874272443	0.950808509
VWFP1	0.005	0.874505431	0.950936554
ELL	0.005	0.874533742	0.950940391
RP11-680B3	0.005	0.874557782	0.950940391
ABCA12	0.005	0.874588825	0.950940391
MCFD2P1	0.005	0.874654599	0.950990197
RP11-474J18	0.005	0.874803161	0.951086588
KRTAP10-7	0.005	0.874837159	0.951101839

MANBAL	0.005	0.874877158	0.951123614
PRKG2	0.005	0.874964511	0.951193263
RN7SL41P	0.005	0.875018564	0.951193263
RP6-201G10	0.005	0.875021111	0.951193263
MIR302E	0.005	0.87513674	0.951297245
RP11-552M6	0.005	0.875361037	0.951430198
UGT2A1	0.005	0.875373828	0.951430198
DUPD1	0.005	0.875636626	0.95161726
RNU4-30P	0.005	0.875653016	0.95161726
RP11-552J9	0.005	0.875683266	0.95161726
HSD3BP5	0.005	0.875710863	0.95161726
PAQR9-AS1	0.005	0.875773975	0.951642416
RP11-368N21	0.005	0.875876779	0.951709623
GALP	0.005	0.87593581	0.951709702
C4orf45	0.005	0.876117214	0.951885084
BRK1P2	0.005	0.876175485	0.951911669
RP11-98G7	0.005	0.876207749	0.951911669
CTC-329H14	0.005	0.876243281	0.951911669
RP11-218E15	0.005	0.87628295	0.951911669
NDUFA9P1	0.005	0.876330145	0.951911669
AC112693	0.005	0.876423982	0.951979497
GPR108	0.005	0.876453819	0.951990195
UCP2	0.005	0.876517964	0.952038155
ROPN1B	0.005	0.876597161	0.952059039
C12orf10	0.005	0.876666919	0.95209432
RP3-366N23	0.005	0.876702026	0.95210624
PES1P2	0.005	0.876734563	0.95210624
RP11-147I11	0.005	0.876740575	0.95210624
TRIM51JP	0.005	0.876913736	0.952227521
RP11-382N13	0.005	0.87693223	0.952227521
AC105461	0.005	0.876980568	0.952253175
SPATA18	0.005	0.876995843	0.952253175
FAM160A1	0.005	0.877078938	0.95230097
DDX19A	0.005	0.877116826	0.95230097
GJA4	0.005	0.87711984	0.95230097
RPL7AP3	0.005	0.877210295	0.952364369
YWHABP1	0.005	0.877224615	0.952364369
FAM156A	0.005	0.877238224	0.952364369
MATN1	0.005	0.877289475	0.952389394
RP5-1042I8	0.005	0.877324357	0.952389394
RPS27P5	0.005	0.877341412	0.952389394
BEAN1-AS1	0.005	0.877358445	0.952389394
AP001482	0.005	0.877361258	0.952389394
RBMX2P5	0.005	0.877393268	0.952402434
TUBB4BP3	0.005	0.87747998	0.952462305
HNRNPA1P41	0.005	0.877635993	0.952579066
AC133749	0.005	0.877764476	0.95269681
CACNA1B	0.005	0.877937305	0.952819252
SUCLA2P1	0.005	0.878028659	0.952853259
HM13-AS1	0.005	0.87809308	0.952878564
OR5W2	0.005	0.878116135	0.952878564
RAB2A	0.005	0.878167912	0.952878564

AC012603	0.005	0.878198651	0.952878564
SACS	0.005	0.878203224	0.952878564
CTD-3252C9	0.005	0.878266099	0.952915516
RPL23AP35	0.005	0.8783035	0.952929494
RP11-787B4	0.005	0.87868416	0.953169063
ZMYND19	0.005	0.878749739	0.953201399
RPS4XP2	0.005	0.878830279	0.953267052
MAP1LC3C	0.005	0.878896607	0.953313109
MPDZ	0.005	0.878994376	0.953319292
LINC00115	0.005	0.879023234	0.953319292
OR4A50P	0.005	0.879030395	0.953319292
ZNF99	0.005	0.879101678	0.953330788
MIR4679-1	0.005	0.879109221	0.953330788
AC078916	0.005	0.879277229	0.953416137
R3HDM2P2	0.005	0.879284787	0.953416137
RP11-1042B17	0.005	0.879288017	0.953416137
WDPCP	0.005	0.879322587	0.953431915
CLDN19	0.005	0.879394903	0.953466912
MUC22	0.005	0.879457756	0.953491647
RP11-120B7	0.005	0.879518066	0.953523209
TTLL11	0.005	0.879543129	0.953523209
MIR4789	0.005	0.879546929	0.953523209
MRPL22	0.005	0.879716295	0.953590158
RP11-495O10	0.005	0.879739427	0.953590158
RNA5SP459	0.005	0.879746118	0.953590158
RNU6-9	0.005	0.879748839	0.953590158
SNORA77	0.005	0.879790548	0.953600679
AC104395	0.005	0.879991099	0.953666403
MRPL42P4	0.005	0.879999393	0.953666403
CCDC148-AS1	0.005	0.8801325	0.953778848
LINC01173	0.005	0.880335945	0.953900897
RP11-267L5	0.005	0.880589762	0.954057116
RPL13P6	0.005	0.880729394	0.954142936
RP11-31A23	0.005	0.880733268	0.954142936
TM4SF4	0.005	0.88073962	0.954142936
CTD-2309O5	0.005	0.880830752	0.954161828
MIR568	0.005	0.880905992	0.954161828
USP9YP4	0.005	0.880915295	0.954161828
RP11-153M7	0.005	0.880923015	0.954161828
EEF1DP6	0.005	0.881021891	0.954209978
RNU1-115P	0.005	0.881149709	0.954263891
DARS	0.005	0.881151813	0.954263891
TMEM27	0.005	0.881223772	0.954319451
MIR670HG	0.005	0.881243191	0.954319451
EEF1E1P1	0.005	0.88128292	0.954333567
ARHGEF16	0.005	0.881325894	0.954333567
ARL6IP5	0.005	0.881410782	0.954333567
TRAPPC2P8	0.005	0.881425882	0.954333567
RP5-1077I2	0.005	0.881520296	0.954402527
PRDX3P4	0.005	0.881589398	0.95442716
RP11-546M21	0.005	0.881704651	0.95442716
FAM90A25P	0.005	0.881767651	0.95442716

VN2R19P	0.005	0.881784492	0.95442716
RP11-15B24	0.005	0.881862149	0.95442716
RP11-463D19	0.005	0.881863682	0.95442716
MIR3649	0.005	0.882010973	0.954521499
SPAM1	0.005	0.88207482	0.954528359
RP11-279E1	0.005	0.882123489	0.954537682
PDE1C	0.005	0.882126137	0.954537682
LGALSL	0.005	0.882254566	0.9545899
RP1-7G5	0.005	0.882276461	0.954591903
RP11-575A19	0.005	0.882317125	0.954614214
RPSAP64	0.005	0.88234935	0.954619472
RP11-402K9	0.005	0.882436642	0.954620377
RNA5SP505	0.005	0.882436959	0.954620377
RP11-36D19	0.005	0.882438257	0.954620377
RNU6-1292P	0.005	0.882443083	0.954620377
LENG1	0.005	0.882488591	0.954633408
RP11-44N11	0.005	0.882586859	0.954667492
ENTPD6	0.005	0.882610564	0.954671451
RP11-506K19	0.005	0.882676436	0.954686563
TVP23C-CDRT4	0.005	0.882709818	0.954686563
AC004984	0.005	0.882835351	0.954686563
RNU4-22P	0.005	0.882859067	0.954686563
TESC-AS1	0.005	0.882863428	0.954686563
LSS	0.005	0.882996758	0.954749932
RNF40	0.005	0.883042448	0.954749932
GPR113	0.005	0.883043956	0.954749932
HULC	0.005	0.883243814	0.954900986
HMGB1P45	0.005	0.883334268	0.954955423
C21orf140	0.005	0.883454444	0.954958496
RP3-442L6	0.005	0.883471277	0.954958496
AC091167	0.005	0.883574001	0.955041167
MSNP1	0.005	0.883617404	0.955066405
SLFN11	0.005	0.883843379	0.95510974
ATP6V1D	0.005	0.883846397	0.95510974
TAF12	0.005	0.883857605	0.95510974
SERPINB12	0.005	0.883872074	0.95510974
CTD-2560K21	0.005	0.883881071	0.95510974
RP11-314N13	0.005	0.883883903	0.95510974
AC097499	0.005	0.883898144	0.95510974
RP11-83J16	0.005	0.884008494	0.955207309
OR8G7P	0.005	0.884068406	0.955234773
AC211469	0.005	0.88429813	0.955404028
RP1-81D8	0.005	0.884412684	0.955448962
RP1-149L1	0.005	0.88448104	0.955480803
RP11-527H14	0.005	0.884482282	0.955480803
SLC25A28	0.005	0.884529739	0.955510397
ZNF629	0.005	0.884672885	0.955572963
SPTLC1	0.005	0.884687976	0.955572963
RN7SKP125	0.005	0.884733477	0.955589609
RNA5SP145	0.005	0.884860469	0.955640394
RP11-53B5	0.005	0.884866804	0.955640394
RP1-190J20	0.005	0.884891087	0.955640639

RP11-499A10	0.005	0.884935667	0.955667113
TP53AIP1	0.005	0.884969924	0.955667829
FRS3	0.005	0.885059658	0.955692664
NUTM2A-AS1	0.005	0.8851929	0.955755917
ASB1	0.005	0.885198506	0.955755917
SLC22A18	0.005	0.88524637	0.955785929
CFHR4	0.005	0.885368628	0.95580956
RPS12P10	0.005	0.885373244	0.95580956
RP3-389A20	0.005	0.885408738	0.95580956
RNU6-699P	0.005	0.885484654	0.955869846
TSTD3	0.005	0.885520198	0.95588655
MFAP1P1	0.005	0.885547229	0.955894064
HFE2	0.005	0.885607084	0.955937008
RP6-186E3	0.005	0.885642925	0.955947327
RP11-735A19	0.005	0.885657051	0.955947327
AC013409	0.005	0.885825839	0.956035143
RP3-522D1	0.005	0.885851847	0.956035143
SMAD1-AS2	0.005	0.885853305	0.956035143
RPL19	0.005	0.885867786	0.956035143
CTSL3P	0.005	0.88587866	0.956035143
RN7SKP117	0.005	0.885907236	0.956044319
C1QTNF9	0.005	0.886070066	0.956138815
RP11-601H16	0.005	0.886143392	0.956138815
RP11-113C12	0.005	0.886377826	0.956292145
MIR8076	0.005	0.886516099	0.956397994
TEX37	0.005	0.886574665	0.956417848
MAGEA3	0.005	0.886712857	0.956540032
NCAPD2P1	0.005	0.886728095	0.956540032
HIST1H4E	0.005	0.88680665	0.95658144
PEX3	0.005	0.886871426	0.956629647
RP11-147K16	0.005	0.886917835	0.956636374
ARHGEF10	0.005	0.887017221	0.956721906
RNU6-1275P	0.005	0.88720819	0.956834923
PSMA6P1	0.005	0.887278625	0.956834923
AC067956	0.005	0.88730055	0.956834923
TMBIM7P	0.005	0.887357866	0.956850958
CCDC137	0.005	0.887398982	0.956873629
HMHB1	0.005	0.887449067	0.95690597
APOOP5	0.005	0.887595861	0.956982063
RN7SKP232	0.005	0.887636027	0.956999232
RPS27P20	0.005	0.888094043	0.957303037
SLC35F5	0.005	0.88810563	0.957303037
OR4F5	0.005	0.888118813	0.957303037
LRRC37A5P	0.005	0.88830838	0.957442365
ESPNP	0.005	0.888347149	0.957449281
AC004549	0.005	0.888449948	0.957486608
RNU6-864P	0.005	0.888473663	0.9574905
RNA5SP267	0.005	0.888563007	0.957515371
pk	0.005	0.888565561	0.957515371
OR13C6P	0.005	0.888729901	0.957593299
LINC00499	0.005	0.8889125	0.957700871
SNORA11C	0.005	0.888949925	0.957700871

RN7SL666P	0.005	0.888967918	0.957700871
TIGD2	0.005	0.889012151	0.957700871
NEUROG2	0.005	0.88907036	0.957700871
RBAK-RBAKDN	0.005	0.889070408	0.957700871
MTND1P30	0.005	0.889071038	0.957700871
SLC26A9	0.005	0.889396843	0.957900171
SNRPA	0.005	0.889480355	0.957913693
CLIC4P1	0.005	0.889544102	0.957943799
MIR548H2	0.005	0.889607242	0.957975132
SNORD12C	0.005	0.889717886	0.958072617
NFATC1	0.005	0.889781578	0.95810842
ZNF652P1	0.005	0.889791369	0.95810842
MIR1972-2	0.005	0.889882636	0.958156176
HS3ST2	0.005	0.88991407	0.958156176
SLC35G1	0.005	0.889985712	0.958179958
TIMM17BP1	0.005	0.890049384	0.958179958
TTTY20	0.005	0.890079108	0.958179958
TAF9P1	0.005	0.890079108	0.958179958
FAM197Y7	0.005	0.890079108	0.958179958
CTD-2553L13	0.005	0.890217906	0.958255577
NPHP1	0.005	0.890260635	0.958255577
AC022201	0.005	0.890467719	0.958372658
RPL7L1P2	0.005	0.890651016	0.958429373
RP4-765C7	0.005	0.890652899	0.958429373
ATP5I	0.005	0.890712538	0.958471894
MBD3L3	0.005	0.890786368	0.958480971
SNORD113-8	0.005	0.890811648	0.958480971
PDCL3	0.005	0.890882173	0.958481188
ITSN1	0.005	0.890923179	0.958501971
RPS24P17	0.005	0.891113916	0.958608774
RN7SL461P	0.005	0.891121524	0.958608774
AC018450	0.005	0.891165617	0.958618077
ARL9	0.005	0.891211312	0.958640404
MICALL2	0.005	0.891244336	0.958654275
MIR187	0.005	0.891383202	0.958760338
RP11-155G14	0.005	0.891485413	0.958816879
SHMT1	0.005	0.891515865	0.958816879
CTD-2639E6	0.005	0.891516297	0.958816879
RP11-356O24	0.005	0.891850938	0.959029158
TOPORSLP	0.005	0.891858848	0.959029158
RP11-481J13	0.005	0.891860601	0.959029158
PSMC1P2	0.005	0.891874765	0.959029158
RP11-272B17	0.005	0.891993055	0.959069741
PCSK7	0.005	0.892027877	0.959069869
MIR8485	0.005	0.892053586	0.959069869
OMP	0.005	0.892146308	0.959126255
CTA-989H11	0.005	0.892232521	0.959138518
XAGE1B	0.005	0.892297928	0.959138518
RPS26P11	0.005	0.892359325	0.959138759
RP5-991C6	0.005	0.892410289	0.959158722
RP11-83N9	0.005	0.892418176	0.959158722
SYT13	0.005	0.892455279	0.959162332

RNU6-705P	0.005	0.892586969	0.959162332
CTD-2246P4	0.005	0.892587139	0.959162332
EEF1A1P31	0.005	0.892624473	0.959163996
PSORS1C3	0.005	0.892656123	0.959176364
ARIH2OS	0.005	0.892754959	0.959260923
RP13-580F15	0.005	0.892798919	0.959264876
C1orf229	0.005	0.892952919	0.959293348
MIR141	0.005	0.892984884	0.959293348
HNRNPA1P22	0.005	0.892993405	0.959293348
UOX	0.005	0.893071457	0.959319516
CTTNBP2	0.005	0.893237715	0.959388514
RP11-43A14	0.005	0.893747956	0.959760802
RP11-3N2	0.005	0.894074681	0.959943047
RNU6-245P	0.005	0.894176686	0.960008859
RPL26P30	0.005	0.894252442	0.960068551
AC006366	0.005	0.894370092	0.96010829
RP11-69C17	0.005	0.894417129	0.960115502
CALM1P2	0.005	0.894484662	0.960122613
RN7SL16P	0.005	0.894497043	0.960122613
AL928874	0.005	0.894504864	0.960122613
LINC01525	0.005	0.894505749	0.960122613
RP11-703G6	0.005	0.894601839	0.960183932
CYCSP44	0.005	0.894644442	0.960208019
RARS	0.005	0.894703065	0.960237236
LIPJ	0.005	0.894803301	0.960237236
MYLK3	0.005	0.894840847	0.960237236
TPI1P3	0.005	0.894856258	0.960237236
SIPA1	0.005	0.894907371	0.960237236
AC107613	0.004	0.894993557	0.960253336
ARMCX7P	0.004	0.895027346	0.960253336
AWAT1	0.004	0.895059017	0.960253336
RPL7L1P8	0.004	0.895066386	0.960253336
LINC00368	0.004	0.895069742	0.960253336
PLEK2	0.004	0.895101316	0.960265579
UBXN10-AS1	0.004	0.895217945	0.960304175
SCARNA3	0.004	0.895245144	0.960311722
FLJ41941	0.004	0.895523239	0.960498373
AC007251	0.004	0.895563443	0.960501725
RPL37AP8	0.004	0.895611925	0.960532092
MIR1255B2	0.004	0.895705961	0.960589683
GTF2F2	0.004	0.895773909	0.960619292
AC007743	0.004	0.895799381	0.960624977
ELOVL5	0.004	0.895924249	0.960671255
AC073310	0.004	0.895949348	0.960671255
ZSWIM7	0.004	0.896037006	0.960682558
AC144525	0.004	0.89620731	0.960737948
LINC01305	0.004	0.896236115	0.960747202
PIGK	0.004	0.896274623	0.960766858
MYBPC1	0.004	0.896352618	0.960802252
RP4-775D17	0.004	0.896368162	0.960802252
RNU6-1236P	0.004	0.89641437	0.960805531
RP11-593F23	0.004	0.896442327	0.960805531

OR4X2	0.004	0.896472089	0.960805531
ENDOV	0.004	0.896512682	0.960827415
LRRC59	0.004	0.89656669	0.960830565
RNU6-911P	0.004	0.89659426	0.960830565
RP11-545N8	0.004	0.896608477	0.960830565
C11orf84	0.004	0.896812537	0.960994592
RP5-1024N4	0.004	0.896875606	0.961021744
GS1-383H3	0.004	0.897107621	0.961248727
MIR551A	0.004	0.897137409	0.96124963
AC017074	0.004	0.897163393	0.96124963
RP11-753B14	0.004	0.897375596	0.96134121
ALG1L3P	0.004	0.897439733	0.961388294
RNU6-1210P	0.004	0.897558237	0.961410943
MLK4	0.004	0.897561807	0.961410943
RP11-152O14	0.004	0.897733751	0.961475928
RNU4-85P	0.004	0.897767527	0.961475928
RP11-1147P1	0.004	0.89777011	0.961475928
AC079772	0.004	0.897814534	0.961475928
RP11-58E21	0.004	0.897846156	0.961475928
CASC23	0.004	0.897873698	0.961475928
SMR3A	0.004	0.897925174	0.961475928
RNU6-1019P	0.004	0.897925291	0.961475928
AC012454	0.004	0.898026106	0.961527897
AC019198	0.004	0.898060474	0.961527897
RP11-397A16	0.004	0.898072817	0.961527897
PI4K2A	0.004	0.898153855	0.96156934
OR2AF1P	0.004	0.898202445	0.96157082
RP11-494M8	0.004	0.898237603	0.96157082
AL160291	0.004	0.898256186	0.96157082
RLN3	0.004	0.898369919	0.961588669
TRIM35	0.004	0.898389825	0.961588669
RPL31P56	0.004	0.898393884	0.961588669
IGKV3-34	0.004	0.898395011	0.961588669
CTD-2001J20	0.004	0.89841419	0.961588669
YIPF1	0.004	0.898570051	0.961677833
EDA2R	0.004	0.898578263	0.961677833
DOCK4-AS1	0.004	0.89863484	0.961716772
GM140	0.004	0.89872862	0.961784993
FBXL7	0.004	0.899026472	0.961962971
RP11-659F24	0.004	0.899110788	0.962031576
RPL35P1	0.004	0.899136693	0.96203768
AC107890	0.004	0.899166821	0.962048303
AC104946	0.004	0.899202194	0.962064537
CTB-113P19	0.004	0.899297921	0.962068491
DEAF1	0.004	0.899313914	0.962068491
RP5-934G17	0.004	0.899331424	0.962068491
SSX3	0.004	0.899348188	0.962068491
SQSTM1P1	0.004	0.899359667	0.962068491
RP11-618M23	0.004	0.89938769	0.962068491
TSPY10	0.004	0.899429482	0.962091588
RP11-183A22	0.004	0.899555787	0.962147261
RP1-278O22	0.004	0.899575918	0.962147261

RNU6-579P	0.004	0.899576738	0.962147261
NKX3-1	0.004	0.899733646	0.962177571
MIR26B	0.004	0.899771533	0.962177571
RP11-632C17__A	0.004	0.899790707	0.962177571
RP11-145M9	0.004	0.89993682	0.96226687
RN7SL521P	0.004	0.900046615	0.962341059
RP11-283C24	0.004	0.90017587	0.962400969
AP000860	0.004	0.900183475	0.962400969
THSD1	0.004	0.90049494	0.962669132
SRGAP3-AS1	0.004	0.900608055	0.962702262
FAM185BP	0.004	0.900624209	0.962702262
DLGAP1-AS4	0.004	0.900689584	0.962720981
FLJ36000	0.004	0.90070515	0.962720981
RP11-300A12	0.004	0.900769912	0.962744668
IQGAP1	0.004	0.90091475	0.962836972
SNX33P1	0.004	0.900953296	0.962856561
RP11-187E13	0.004	0.900980891	0.962864446
RN7SKP144	0.004	0.901009204	0.962873098
CLPTM1LP1	0.004	0.901266812	0.963105172
RN7SKP133	0.004	0.901290976	0.963108713
TRPM6	0.004	0.901329891	0.963108713
KRTAP11-1	0.004	0.90136115	0.963119545
RP3-380B4	0.004	0.901469545	0.963202515
CYP4F62P	0.004	0.901510615	0.963202515
FAM196B	0.004	0.901635624	0.963239942
AC083829	0.004	0.901767731	0.963316251
PSMC1P11	0.004	0.901888374	0.963380304
MIR3116-1	0.004	0.902156693	0.963491595
RP11-64J4	0.004	0.902201854	0.963491595
SRP68P2	0.004	0.902222769	0.963491595
MIR4456	0.004	0.902269781	0.963506791
HDAC4	0.004	0.902424738	0.96355917
CTD-2318H23	0.004	0.90244022	0.96355917
SPATS1	0.004	0.902461031	0.96355979
PTPLAD2	0.004	0.902586482	0.963585725
RNU6-56P	0.004	0.902648717	0.963608967
COX6CP15	0.004	0.902772063	0.963675842
GGT3P	0.004	0.902851257	0.963738778
SNRPCP6	0.004	0.903093965	0.963933042
ST13P20	0.004	0.903259908	0.963963906
RPS4XP5	0.004	0.903267508	0.963963906
KBTBD12	0.004	0.903295532	0.963963906
CDH20	0.004	0.903315658	0.963963906
TFAP2D	0.004	0.903453999	0.964037025
AP000428	0.004	0.903454523	0.964037025
RPLP0P7	0.004	0.903673566	0.964163463
RP11-296I10	0.004	0.903719681	0.964168752
CTD-2540B15	0.004	0.903771388	0.964202319
AC096554	0.004	0.903797531	0.964208517
AF124730	0.004	0.903817688	0.964208517
RP11-219O3	0.004	0.903910111	0.964263917
AC010723	0.004	0.903944362	0.964278857

MIR1202	0.004	0.903997734	0.964297659
CTD-2308B18	0.004	0.904002481	0.964297659
CTC-432M15	0.004	0.9042761	0.964531018
PCDH19	0.004	0.904282004	0.964531018
CRYGB	0.004	0.904541769	0.964663728
OR1AC1P	0.004	0.90456098	0.964663728
CCDC110	0.004	0.904689081	0.964679328
RPL31P7	0.004	0.904694181	0.964679328
CDX1	0.004	0.904714148	0.964679328
EEF1E1	0.004	0.904764789	0.964679328
RP11-53M11	0.004	0.904869475	0.964679328
MAFA	0.004	0.904886913	0.964679328
GALNT11	0.004	0.9051905	0.964863673
NEIL2	0.004	0.905203479	0.964863673
ABCC8	0.004	0.905238494	0.964863673
ETF1P3	0.004	0.905368991	0.964863673
RN7SL845P	0.004	0.905377099	0.964863673
GRM5-AS1	0.004	0.905601966	0.964964302
AC023137	0.004	0.905683151	0.965004682
RP11-318A15	0.004	0.905891387	0.965120577
MIR487B	0.004	0.905942995	0.965120577
ALDOC	0.004	0.905959357	0.965120577
ADIPOR1P2	0.004	0.905967807	0.965120577
CTB-46B19	0.004	0.90597859	0.965120577
TPI1P1	0.004	0.906061906	0.96514654
GTF2A1L	0.004	0.906072121	0.96514654
SCN4B	0.004	0.90617917	0.965192784
AC068286	0.004	0.906208137	0.965202052
ACTR3	0.004	0.906357882	0.965296783
AL080276	0.004	0.906401703	0.965321867
AL645937	0.004	0.90646849	0.965336654
CH17-76K2	0.004	0.906517853	0.965336654
PCK1	0.004	0.906575002	0.965336654
FAM195DP	0.004	0.906585282	0.965336654
RN7SKP270	0.004	0.906587337	0.965336654
DNAH5	0.004	0.906676372	0.965398515
ENTPD2	0.004	0.906779799	0.965411857
MIR4508	0.004	0.906799276	0.965411857
SEMA6D	0.004	0.906810524	0.965411857
TMEM61	0.004	0.906837354	0.96541884
CELF2-AS2	0.004	0.906937634	0.965470871
CTD-2384B9	0.004	0.907011969	0.965518407
RP11-563N12	0.004	0.907094502	0.965538736
ABCC4	0.004	0.907112158	0.965538736
RP11-589P10	0.004	0.907155868	0.965563682
RP11-472F14	0.004	0.907224413	0.96561506
BCAS2	0.004	0.907274448	0.965629394
E2F3	0.004	0.907340231	0.965652012
HLFP1	0.004	0.90758335	0.965781268
MIR4773-1	0.004	0.907687668	0.965869484
MIR548U	0.004	0.90779683	0.965904195
CTD-2182N23	0.004	0.907913746	0.965960731

AL354936	0.004	0.907914253	0.965960731
RPL34P6	0.004	0.908010031	0.966007143
AC093590	0.004	0.90804333	0.966007143
DPRXP6	0.004	0.908121515	0.966051761
CTB-92J24	0.004	0.908307573	0.966163367
AARSP1	0.004	0.908396496	0.966214796
RP11-302F12	0.004	0.908812374	0.96650605
YWHAEP5	0.004	0.908860554	0.966522698
MCHR1	0.004	0.908878511	0.966522698
OR9N1P	0.004	0.909086559	0.966581148
AL161651	0.004	0.909104293	0.966581148
RNA5SP510	0.004	0.909104293	0.966581148
MT-CO1	0.004	0.90954389	0.967024887
RNU6-198P	0.004	0.909581667	0.967043464
RP11-332H18	0.004	0.909689282	0.9671147
RP11-106J23	0.004	0.90973015	0.96713656
AC024569	0.004	0.909911939	0.967199626
RP11-413M3	0.004	0.909931628	0.967199626
DNAH14	0.004	0.910011029	0.967224749
CLEC2B	0.004	0.910031811	0.967224749
RP4-811H24	0.004	0.910036497	0.967224749
GRINA	0.004	0.910253053	0.967357799
PTGER2	0.004	0.910262578	0.967357799
SZRD1	0.004	0.910341812	0.967370147
CTD-2007F2	0.004	0.910495718	0.967438018
CTA-326K9	0.004	0.910533636	0.967438018
RP11-697K23	0.004	0.910536736	0.967438018
ZNF788	0.004	0.910541848	0.967438018
RNASE2	0.004	0.910628878	0.96748732
RNA5SP89	0.004	0.910702241	0.96754368
RN7SL363P	0.004	0.910888107	0.967633221
DHRS4	0.004	0.911008187	0.96769603
APOC4-APOC2	0.004	0.911180803	0.967793049
LINC01572	0.004	0.911233307	0.967827232
MIR7154	0.004	0.911449852	0.967856992
ECT2L	0.004	0.911474123	0.967856992
CYBA	0.004	0.911536915	0.967856992
RP11-459K23	0.004	0.91155319	0.967856992
MIR3121	0.004	0.911656399	0.967856992
RP11-78E6	0.004	0.911662532	0.967856992
MFSD7	0.004	0.911708402	0.967856992
MRPL48	0.004	0.911741315	0.967858933
ZMYM3	0.004	0.911768597	0.967858933
KRT8P7	0.004	0.911846812	0.967896056
RP11-137P24	0.004	0.91194404	0.967977687
EIF3EP2	0.004	0.912267587	0.968105751
AC121157	0.004	0.912280524	0.968105751
RP11-85D18	0.004	0.912488299	0.968202375
RNU6-68P	0.004	0.912531044	0.968202375
PFN1P9	0.004	0.912535864	0.968202375
LIN28B	0.004	0.912557975	0.968202375
MTND4P18	0.004	0.912562299	0.968202375

A3GALT2	0.004	0.91260372	0.968217443
RNU5A-7P	0.004	0.912637489	0.968217443
CUBNP3	0.004	0.912734992	0.968217864
OR8I1P	0.004	0.912758511	0.968217864
RPS2P24	0.004	0.912767388	0.968217864
MRPL30P1	0.004	0.912768117	0.968217864
GNAQP1	0.004	0.912780189	0.968217864
RNU6-162P	0.004	0.912802802	0.968220286
RP11-359P18	0.004	0.912931318	0.968291909
AC008085	0.004	0.913122988	0.968408937
SCGB1D4	0.004	0.913345221	0.968601488
AL355490	0.004	0.913601238	0.968756099
CTD-3093B17	0.004	0.913741981	0.968801185
RP11-34H11	0.004	0.913832256	0.968801185
ZGLP1	0.004	0.913966704	0.968801185
POLR2LP	0.004	0.914011481	0.968801185
RSPRY1	0.004	0.91402172	0.968801185
GM2AP2	0.004	0.914236827	0.968935144
MIR548F4	0.004	0.914331516	0.968936589
RN7SL236P	0.004	0.914358738	0.968936589
RP11-94H18	0.004	0.914373254	0.968936589
RPS18P9	0.004	0.914504312	0.96898923
FAM124B	0.004	0.914603954	0.969073249
KRT8P25	0.004	0.914650153	0.96907908
RP11-755E23	0.004	0.914719286	0.969085435
AF038458	0.004	0.914728748	0.969085435
TRIM77	0.004	0.914823814	0.969085435
VN1R25P	0.004	0.914861632	0.969085435
AC104183	0.004	0.91489312	0.969085435
RNU6-1191P	0.004	0.914909637	0.969085435
WBP1LP6	0.004	0.914957578	0.969102979
DHRS11	0.004	0.915010766	0.969137277
RSPH14	0.004	0.915030656	0.969137277
ACTR3BP1	0.004	0.915105909	0.969195427
RP11-11P22	0.004	0.915173771	0.969219488
RNA5SP169	0.004	0.915208536	0.969219488
RNA5SP212	0.004	0.915223362	0.969219488
IFNA10	0.004	0.915226506	0.969219488
RHOQP2	0.004	0.91562759	0.969510788
RNU6-1114P	0.004	0.915696498	0.969521598
ZFAS1	0.004	0.915892917	0.969632361
RP11-332L8	0.004	0.915996932	0.969702214
RP11-543H23	0.004	0.916022094	0.969702214
BPIFB6	0.004	0.916063357	0.969713493
RPL7L1P7	0.004	0.916091224	0.96971984
RP3-518E13	0.004	0.916126209	0.96971984
RP11-297M9	0.004	0.916130434	0.96971984
RP11-887P2	0.004	0.916151602	0.969720694
AL354749	0.004	0.916382196	0.969835448
RP11-447M12	0.004	0.916628167	0.969964226
BCAN	0.004	0.916702368	0.969980309
GAPDHP73	0.004	0.916799901	0.970041753

AC005392	0.004	0.916803969	0.970041753
SFXN3	0.004	0.91694028	0.970057805
PRMT8	0.004	0.917018958	0.970057805
RP1-203C2	0.004	0.91702456	0.970057805
PCDHA4	0.004	0.917040073	0.970057805
CTD-2588J1	0.004	0.917042127	0.970057805
LINC00558	0.004	0.917075182	0.970057805
ABCC11	0.004	0.917101492	0.970057805
RPS11P1	0.004	0.917153502	0.970069729
MIR6128	0.004	0.917263873	0.970104402
ZNF677	0.004	0.917345917	0.970104402
CNN2P2	0.004	0.917374499	0.97010958
CTB-37A13	0.004	0.917412579	0.970128309
TPRKBP1	0.004	0.917512999	0.970144855
RP11-458K10	0.004	0.917517987	0.970144855
MIR6788	0.004	0.917530074	0.970144855
FAM229A	0.004	0.917607253	0.970174267
RP4-677H15	0.004	0.917651137	0.970186705
CTD-2091N23	0.004	0.917697778	0.970192943
CTD-2515A14	0.004	0.917864446	0.970299294
AC008277	0.004	0.91793588	0.970312324
RNA5-8SP2	0.003	0.918241977	0.970502536
RPL7P38	0.003	0.91829604	0.970502536
EPHA5-AS1	0.003	0.918296277	0.970502536
RP11-565H13	0.003	0.918592759	0.970602753
CTD-2320J21	0.003	0.918594383	0.970602753
OR4A7P	0.003	0.918634816	0.970602753
CAMKV	0.003	0.918640759	0.970602753
MAPRE1P1	0.003	0.918643807	0.970602753
RN7SKP206	0.003	0.918697506	0.970602753
OCRL	0.003	0.918839507	0.970615014
RNU7-164P	0.003	0.91889979	0.970615014
VPS4A	0.003	0.918902793	0.970615014
CSDE1	0.003	0.919007694	0.970615114
OR7E155P	0.003	0.919023942	0.970615114
RP11-343B18	0.003	0.919046721	0.970615114
RP11-379P15	0.003	0.91905268	0.970615114
RP11-11N9	0.003	0.919054943	0.970615114
RNU6-787P	0.003	0.919105095	0.970625391
C22orf42	0.003	0.919208563	0.970669732
ZNF354C	0.003	0.919451588	0.970788549
MAP3K4	0.003	0.919468928	0.970788549
RP5-872K7	0.003	0.919543091	0.970813414
KRT8P45	0.003	0.919556497	0.970813414
RPL17P2	0.003	0.919770618	0.970921865
CTRB1	0.003	0.919776416	0.970921865
CYCSP32	0.003	0.919793891	0.970921865
OR4L1	0.003	0.919926202	0.970967692
DDX41	0.003	0.920013428	0.970967692
RP11-184C23	0.003	0.920035619	0.970967692
U52111	0.003	0.920038573	0.970967692
RPUSD3	0.003	0.920251441	0.971125042

RP11-331F4	0.003	0.920439601	0.971242254
RNF10P1	0.003	0.920444084	0.971242254
RPS14P4	0.003	0.920643299	0.971430941
RP11-781E19	0.003	0.920687281	0.971455828
FLJ37453	0.003	0.920799896	0.971500958
FAM83A-AS1	0.003	0.920861463	0.971500958
RNU6-345P	0.003	0.920956329	0.97150823
MECOM	0.003	0.920987203	0.97150823
SNX31	0.003	0.921071949	0.971517334
SLC51B	0.003	0.921110021	0.971520184
MT-TD	0.003	0.921198377	0.971520184
RP11-351I21	0.003	0.921199724	0.971520184
FTH1P11	0.003	0.921217441	0.971520184
RP11-244B22	0.003	0.921352829	0.971641451
RNA5SP139	0.003	0.921381701	0.971650384
RP11-157I4	0.003	0.921497591	0.971720352
CRIP1P3	0.003	0.92158139	0.971774899
SYCE3	0.003	0.921638256	0.971784212
RP11-98H4	0.003	0.92165695	0.971784212
RP13-46H24	0.003	0.921695577	0.971784212
RP11-45O22	0.003	0.921817412	0.971794049
RSPH10B2	0.003	0.921852274	0.971794049
NDUFV2	0.003	0.921865697	0.971794049
LACTBL1	0.003	0.921877271	0.971794049
AGBL3	0.003	0.921885211	0.971794049
CTC-270D5	0.003	0.922107278	0.971884627
PTPN20CP	0.003	0.922165156	0.971884627
EEF1A1P11	0.003	0.922226922	0.971884627
RP11-297L6	0.003	0.922231291	0.971884627
RP11-71J2	0.003	0.922242197	0.971884627
CTD-2506P8	0.003	0.922259012	0.971884627
PYDC1	0.003	0.922266622	0.971884627
IGSF22	0.003	0.922320591	0.971884627
EID2	0.003	0.922341407	0.971884627
MIR3123	0.003	0.922358633	0.971884627
RP5-1096D14	0.003	0.922407599	0.971884627
CTC-313D10	0.003	0.922464183	0.971884627
AC084809	0.003	0.922484075	0.971884627
GMPPB	0.003	0.92255477	0.971884627
MYCBP	0.003	0.922572984	0.971884627
RP1-34M23	0.003	0.922635226	0.971884627
CITED2	0.003	0.92264454	0.971884627
AREG	0.003	0.922742551	0.971942246
DGCR9	0.003	0.922947813	0.972096586
OR8K2P	0.003	0.922999055	0.972129058
DYRK4	0.003	0.923031387	0.972131393
RP11-475O23	0.003	0.923162758	0.972131393
NOTCH2NL	0.003	0.923206385	0.972131393
RPL36AP33	0.003	0.92321893	0.972131393
OR51G2	0.003	0.923225797	0.972131393
AP001412	0.003	0.923344813	0.972170662
MIR3688-1	0.003	0.923380264	0.972170662

RP11-45D17	0.003	0.923408508	0.972170662
YBX1P4	0.003	0.923445455	0.972170662
KCNK15	0.003	0.923454251	0.972170662
AC000110	0.003	0.923456344	0.972170662
FAM24A	0.003	0.923581413	0.972235167
LINC00251	0.003	0.923589726	0.972235167
NLRP9	0.003	0.92372497	0.972291575
OOEP	0.003	0.923814386	0.97231305
RNA5SP138	0.003	0.923827034	0.97231305
GANAB	0.003	0.923888414	0.972356164
OR2A4	0.003	0.924058215	0.972491893
ZNF839P1	0.003	0.924115522	0.972509224
RP11-6N17	0.003	0.92427879	0.97259508
RP3-430A16	0.003	0.924302805	0.972598862
PIP5K1C	0.003	0.924471815	0.972737093
AC091947	0.003	0.924579264	0.972782305
RPS4XP9	0.003	0.924624996	0.972808524
ZNF648	0.003	0.924645035	0.972808524
RP11-206P5	0.003	0.924714024	0.972838126
TRPM5	0.003	0.924745238	0.972849476
RP11-73B8	0.003	0.924909602	0.972937854
LL22NC03-86D4	0.003	0.924912982	0.972937854
MIR6770-1	0.003	0.924998938	0.972937854
RNU11-2P	0.003	0.9250831	0.972968505
TRIM43B	0.003	0.92522389	0.973023002
PPP4R1	0.003	0.925249168	0.973023002
AC026449	0.003	0.92531599	0.973063031
C1orf87	0.003	0.925389044	0.973106443
CTA-345G4	0.003	0.925400208	0.973106443
RP11-706F1	0.003	0.925418567	0.973106443
RP11-182J23	0.003	0.925519204	0.973147809
RP11-798K23	0.003	0.925655658	0.973263565
IFT52	0.003	0.925801427	0.9733586
LINC01154	0.003	0.925974353	0.973450759
ANKRD30BP1	0.003	0.926075644	0.973496473
PCYOX1L	0.003	0.92610859	0.973509619
FAM231C	0.003	0.926150414	0.973510611
CTTN	0.003	0.926187552	0.973527903
SNX22	0.003	0.926246557	0.973527903
RP11-160H22	0.003	0.926251254	0.973527903
FAM171B	0.003	0.926289509	0.973527903
RP11-160O5	0.003	0.926373679	0.973534838
CTB-5E10	0.003	0.926420217	0.973534838
AMMECR1LP1	0.003	0.926420463	0.973534838
RP11-63D14	0.003	0.926422873	0.973534838
NPTN	0.003	0.926439194	0.973534838
DHDDS	0.003	0.9265251	0.973603631
PFDN6	0.003	0.926657867	0.97369117
ACD	0.003	0.926706462	0.97369117
CRYBB3	0.003	0.926712305	0.97369117
LCE1E	0.003	0.926876215	0.97369117
LINC01551	0.003	0.926924668	0.97369117

RP11-604N13	0.003	0.926935243	0.97369117
RNU4-9P	0.003	0.926983761	0.973698901
AC018361	0.003	0.927019144	0.973714592
NIM1K	0.003	0.92709957	0.97375612
LINC01044	0.003	0.927362969	0.973902502
RP11-168F9	0.003	0.927461242	0.973964161
RN7SL808P	0.003	0.927505135	0.973988779
TDRD9	0.003	0.927664528	0.974024565
ZIK1	0.003	0.927679719	0.974024565
PSMD5	0.003	0.927680587	0.974024565
RPL9P5	0.003	0.927740568	0.974024565
PDZRN4	0.003	0.927779079	0.974040212
AC099512	0.003	0.927810666	0.974051902
RP11-356N1	0.003	0.928047252	0.974239913
RNA5SP349	0.003	0.928181663	0.974281531
RP11-62E14	0.003	0.928193045	0.974281531
GCFC2	0.003	0.928256869	0.974296821
RP11-177A2	0.003	0.928288641	0.974296821
STEAP2-AS1	0.003	0.928315283	0.974296821
UCN3	0.003	0.928383035	0.974330641
RHPN1	0.003	0.928410493	0.974337987
RN7SL340P	0.003	0.928439518	0.974346978
MIR3661	0.003	0.928704854	0.974555293
FKSG56	0.003	0.928761246	0.974555293
RNU6-235P	0.003	0.928779943	0.974555293
FARSBP1	0.003	0.928801715	0.974555293
OR4F14P	0.003	0.928860646	0.974595655
RP11-388E23	0.003	0.928993742	0.974614812
SERPIND1	0.003	0.928999311	0.974614812
RN7SL240P	0.003	0.929052583	0.974614812
AP001442	0.003	0.929071785	0.974614812
CTC-510F12	0.003	0.92908968	0.974614812
RP11-729L2	0.003	0.929158178	0.974620344
RP11-287J9	0.003	0.929249504	0.974638643
MRPL21	0.003	0.929307118	0.97464138
PDSS1P1	0.003	0.92933096	0.97464138
OR2A3P	0.003	0.92933397	0.97464138
LINC01384	0.003	0.929500128	0.974790047
PPP1R1AP2	0.003	0.929656252	0.974893505
RP5-1033H22	0.003	0.929734193	0.9749537
RP13-33H18	0.003	0.9297769	0.974955623
LDHAL6FP	0.003	0.929861912	0.975001834
RP11-408E1	0.003	0.92999243	0.975098471
BANF1P1	0.003	0.929997885	0.975098471
AS3MT	0.003	0.93003597	0.975098471
OSTM1-AS1	0.003	0.930105056	0.975123813
EGFR-AS1	0.003	0.930133224	0.975123813
FTH1P8	0.003	0.930141328	0.975123813
RPL36AP55	0.003	0.930142038	0.975123813
AC104405	0.003	0.930338506	0.97530601
RPS10P2	0.003	0.930366159	0.97530601
RN7SL571P	0.003	0.930439053	0.975349315

MUC16	0.003	0.930489444	0.97538067
PPP1R3C	0.003	0.930533286	0.975405159
SNORD115-23	0.003	0.930690927	0.975505992
RN7SKP159	0.003	0.930690927	0.975505992
RP11-201A3	0.003	0.930765657	0.975541382
TCEB3C	0.003	0.930892024	0.975609419
CTD-2025J6	0.003	0.930951507	0.975634104
LIPC	0.003	0.931030305	0.975634104
GACAT3	0.003	0.931032729	0.975634104
PPP2R5B	0.003	0.931075891	0.975634104
DPH3	0.003	0.931094123	0.975634104
AP001465	0.003	0.931127607	0.975641627
TMEM256P1	0.003	0.93115654	0.975650479
RNU6-469P	0.003	0.931247142	0.975723945
RP11-366M4	0.003	0.93127471	0.975731364
MBD3L1	0.003	0.931313448	0.97573892
UBE2R2	0.003	0.931322896	0.97573892
CLSTN2-AS1	0.003	0.931366401	0.975763035
ALG1L8P	0.003	0.931448609	0.975827697
AC008686	0.003	0.931545123	0.975864411
AKT3-IT1	0.003	0.931628258	0.975930035
CRYAA	0.003	0.931662169	0.975944093
FCF1P6	0.003	0.931876921	0.97596949
HYDIN2	0.003	0.931889167	0.97596949
RP11-861L17	0.003	0.931899467	0.97596949
RPS20P23	0.003	0.931900934	0.97596949
RP11-192P3	0.003	0.931941941	0.97596949
PRPF19P1	0.003	0.931988632	0.975985545
RP11-79N23	0.003	0.932019004	0.975995891
GAPDHP52	0.003	0.932129491	0.97604721
FDPSP6	0.003	0.932205629	0.976062557
AL161740	0.003	0.932338639	0.976158903
RP1-69E11	0.003	0.932419493	0.976188417
CACNG8	0.003	0.932448814	0.976188417
RP11-685N10	0.003	0.932534712	0.976239274
AC024937	0.003	0.932557148	0.976239274
ZNF691	0.003	0.932590145	0.976250444
PCAT19	0.003	0.932610551	0.976250444
KRTAP8-2P	0.003	0.932721927	0.976278599
CTD-3118D7	0.003	0.932739939	0.976278599
RNU6-970P	0.003	0.932778277	0.97629727
AC093166	0.003	0.932899909	0.976358448
GPR173	0.003	0.932926631	0.976358448
KIAA1522	0.003	0.93293792	0.976358448
MSANTD2P1	0.003	0.932967712	0.976358448
UBE3AP2	0.003	0.933025935	0.976363374
GABRG1	0.003	0.933147931	0.976448127
MIR3660	0.003	0.933184492	0.976464931
AC105921	0.003	0.933250711	0.976498855
EIF5A2P1	0.003	0.933304621	0.976526268
MTND1P14	0.003	0.933472011	0.976594136
DERL2	0.003	0.933601375	0.976666279

SLC4A4		0.003	0.933644363	0.976672167
BAGE5		0.003	0.933716042	0.976690688
DUSP3		0.003	0.933930949	0.976829758
RPS3AP26		0.003	0.933933121	0.976829758
GOLGA6L17P		0.003	0.934040481	0.976829758
RN7SKP269		0.003	0.934045073	0.976829758
	2-Sep	0.003	0.934068601	0.976829758
SNORD38A		0.003	0.934086389	0.976829758
PARD3		0.003	0.934092484	0.976829758
FBXO16		0.003	0.934105945	0.976829758
NUTF2P6		0.003	0.934211851	0.976860858
NME1		0.003	0.934343041	0.976906849
GPR53P		0.003	0.934406775	0.976906849
TOMM22P1		0.003	0.934427476	0.976907046
RP11-100K18		0.003	0.934448498	0.97690758
RPL21P28		0.003	0.934576235	0.976966054
OR6C66P		0.003	0.934578386	0.976966054
CDKN2B-AS		0.003	0.934586482	0.976966054
RBM22P5		0.003	0.934666554	0.976981325
PCDHGC4		0.003	0.934796038	0.976981325
PKMP2		0.003	0.934801544	0.976981325
NDUFAF4		0.003	0.934831148	0.976985936
MROH7		0.003	0.934939806	0.977078053
GAPDHP67		0.003	0.935033708	0.977111866
OR10D1P		0.003	0.935165607	0.977206818
RP11-516J2		0.003	0.935305745	0.97728893
RP11-196O2		0.003	0.935394787	0.977344592
LARP1BP3		0.003	0.935400058	0.977344592
RP11-90D11		0.003	0.935453139	0.977378612
WASF3		0.003	0.935473887	0.977378849
RP11-326E7		0.003	0.935573889	0.977419005
XXbac-B461K10		0.003	0.935744277	0.977554128
CUL7		0.003	0.935808302	0.977563015
RNU6-997P		0.003	0.935855722	0.977563015
TRAPPC9		0.003	0.935863026	0.977563015
MAGI2		0.003	0.93586954	0.977563015
MIR6772		0.003	0.935890638	0.977563015
RP1-217P22		0.003	0.936027206	0.977678138
MYLK4		0.003	0.936303865	0.977881325
OR9I2P		0.003	0.936372801	0.977931877
SLC25A15P3		0.003	0.936489709	0.97800553
SNORA31		0.003	0.936504928	0.97800553
KRT8P28		0.003	0.936567336	0.978041347
RP11-27M5		0.003	0.936587118	0.978041347
AC104637		0.003	0.936627268	0.978041347
RP11-307P22		0.003	0.936791922	0.978041347
CNDP1		0.003	0.936829536	0.978041347
AC018616		0.003	0.93685359	0.978041347
RNU6-1042P		0.003	0.93690328	0.978041347
WDR25		0.003	0.936973196	0.978041347
RHBDL1		0.003	0.936991005	0.978041347
RNU6-36P		0.003	0.93704741	0.978055445

OR51E1	0.003	0.93709395	0.978055445
RNU6-646P	0.003	0.937136223	0.978055445
RNA5SP259	0.003	0.937165157	0.978055445
ARNT	0.003	0.937219049	0.978055445
RP11-701P16	0.003	0.937230404	0.978055445
RRNAD1	0.003	0.937408003	0.978173255
ESRRAP1	0.003	0.937425449	0.978173255
C10orf67	0.003	0.937473573	0.978202039
LEMD1-AS1	0.003	0.937674584	0.978283512
MIR4436B2	0.003	0.937736519	0.978283512
IFITM8P	0.003	0.93785284	0.978362002
TAB3P1	0.003	0.938100962	0.978533907
TPT1	0.003	0.938107382	0.978533907
BMP7-AS1	0.003	0.938120356	0.978533907
RP11-483I24	0.003	0.93816939	0.978563622
ATP8A2P1	0.003	0.938305764	0.978617918
AC007677	0.003	0.938331948	0.978617918
AC090945	0.003	0.938369971	0.978617918
CYP4A26P	0.003	0.938385825	0.978617918
RN7SL738P	0.003	0.938540563	0.978757858
LINC01348	0.003	0.93870275	0.978905561
SNORA49	0.003	0.938738883	0.978921807
TMEM127	0.003	0.938779438	0.978942664
RPL37P21	0.003	0.938879177	0.979025235
C1orf112	0.003	0.938940617	0.979045834
AC093724	0.003	0.938952347	0.979045834
TMEM69	0.003	0.938960601	0.979045834
RP11-486A14	0.003	0.939024884	0.979091426
AC005510	0.003	0.939189663	0.97913313
IGHVIII-76-1	0.003	0.93920879	0.97913313
ERLIN1	0.003	0.939295566	0.97920216
RP11-214N16	0.003	0.939328362	0.979214916
MTUS1	0.003	0.93942156	0.979269204
KRTAP6-1	0.003	0.939485659	0.979293975
GOLGA6L3	0.003	0.939500853	0.979293975
RNU6-887P	0.003	0.939555959	0.979302133
CTD-2207A17	0.003	0.939661041	0.979316214
CD320	0.003	0.93978087	0.979316214
AKAP14	0.003	0.939850761	0.979316214
XAB2	0.003	0.939921643	0.979324246
IAPP	0.003	0.939988423	0.979324246
RPL7P13	0.003	0.940021543	0.97933733
EIF3FP3	0.003	0.940149317	0.979441363
DGAT2L7P	0.003	0.940200287	0.979441363
PRSS37	0.003	0.940207152	0.979441363
AL022400	0.003	0.940221019	0.979441363
RP11-204L24	0.003	0.940224224	0.979441363
CCDC155	0.003	0.940311296	0.979446376
RPL21	0.003	0.94037265	0.979488862
LINC00398	0.003	0.940396976	0.979492778
MT-TR	0.003	0.940498516	0.979512854
RP1-303A1	0.003	0.940694883	0.979649058

MTND1P15	0.003	0.940711572	0.979649058
SRIP2	0.003	0.940788315	0.979682527
MSLNL	0.003	0.940792203	0.979682527
NPM1P50	0.003	0.940825514	0.979682527
RP11-48B14	0.003	0.940867129	0.979682527
ASCC3	0.003	0.94089885	0.979694137
MAST4-AS1	0.003	0.941056986	0.97969621
NOMO3	0.003	0.941057888	0.97969621
SNORD115-47	0.003	0.941076624	0.97969621
APOM	0.003	0.941123068	0.97969621
MIR3651	0.003	0.94113098	0.97969621
RP11-1057B8	0.003	0.941198287	0.97969621
PGA5	0.003	0.941206238	0.97969621
RP11-306K13	0.003	0.94122299	0.97969621
ST8SIA6	0.003	0.941240393	0.97969621
RP11-740D6	0.003	0.941258384	0.97969621
CTC-448D22	0.003	0.941311852	0.979698192
RP11-320M2	0.003	0.941387703	0.979722452
LINC00265-2P	0.003	0.941416804	0.979722452
KAAG1	0.003	0.94149924	0.979722452
ODF1	0.002	0.941602434	0.979722452
CKS1BP7	0.002	0.941620499	0.979722452
RPL21P50	0.002	0.941633183	0.979722452
HCN1	0.002	0.941740552	0.979722452
RP11-263E1	0.002	0.94178245	0.979722452
RPL21P1	0.002	0.941782665	0.979722452
MIR15A	0.002	0.942026168	0.97978068
IRAK3	0.002	0.942027375	0.97978068
RN7SL311P	0.002	0.942068555	0.97978068
AC073133	0.002	0.942077855	0.97978068
RNA5SP119	0.002	0.942128445	0.97978068
RP11-544D21	0.002	0.942139795	0.97978068
RP11-313I2	0.002	0.942173801	0.97978068
RP11-319G6	0.002	0.942195991	0.97978068
TCIRG1	0.002	0.942213374	0.97978068
PSAT1P3	0.002	0.942299209	0.979845518
RP5-926E3	0.002	0.942325546	0.979851512
RN7SKP216	0.002	0.94248014	0.979869569
SFR1	0.002	0.942549743	0.979876631
ABCB10P3	0.002	0.942613688	0.979876631
RP11-665E10	0.002	0.942672288	0.979876631
MIR206	0.002	0.942676766	0.979876631
BIN2P2	0.002	0.942678887	0.979876631
RP4-612C19	0.002	0.94272648	0.979884211
PLS1	0.002	0.942759219	0.979884211
MIR874	0.002	0.942791992	0.979884211
RNU7-48P	0.002	0.942823095	0.979884211
AL590708	0.002	0.942829885	0.979884211
PLEKHA6	0.002	0.942870912	0.979884211
CASKIN2	0.002	0.942871347	0.979884211
SLC10A4	0.002	0.943031361	0.979991227
RPL29P11	0.002	0.943082567	0.979991227

RP11-36C20	0.002	0.943097779	0.979991227
GSTO2	0.002	0.943139067	0.979991288
RNU1-52P	0.002	0.943142246	0.979991288
PTGS1	0.002	0.94320339	0.979998579
AC079248	0.002	0.94323618	0.980003687
LYPLA2P2	0.002	0.943303596	0.980003687
KRT18P60	0.002	0.943315536	0.980003687
RP5-998N21	0.002	0.943658465	0.980167529
RP11-15G16	0.002	0.943716095	0.980184636
DAPK2	0.002	0.943853598	0.980272278
TRDD1	0.002	0.943862223	0.980272278
LINC00883	0.002	0.944021372	0.980337044
AFP	0.002	0.94403688	0.980337044
GHc-602D8	0.002	0.944068669	0.980337044
RP11-138J23	0.002	0.944108612	0.980350964
USP17L7	0.002	0.944246808	0.980350964
RN7SL597P	0.002	0.944247751	0.980350964
UBE2V1P10	0.002	0.944258848	0.980350964
KRT18P9	0.002	0.94439462	0.980350964
RN7SL51P	0.002	0.944419312	0.980350964
CTC-367F4	0.002	0.944608562	0.980448189
TMCC1	0.002	0.944628594	0.980448189
KCTD21	0.002	0.944852887	0.980616877
TESK2	0.002	0.944945475	0.980645389
MRPS10P2	0.002	0.944959751	0.980645389
AL355098	0.002	0.94496272	0.980645389
AC023669	0.002	0.944991344	0.980653726
PAGE2	0.002	0.945024278	0.980666535
GJD3	0.002	0.945145501	0.980686675
SLAMF6P1	0.002	0.945167232	0.980686675
RP11-179A9	0.002	0.945278812	0.980759716
BCAP31P1	0.002	0.945330518	0.980768944
RP11-351C8	0.002	0.945349485	0.980768944
HERC2P3	0.002	0.945447112	0.980789436
AC003991	0.002	0.945451609	0.980789436
YWHAEP3	0.002	0.945495668	0.980795659
MIR133B	0.002	0.945498795	0.980795659
MAP3K7CL	0.002	0.945553615	0.980831163
SNORD3B-1	0.002	0.945611543	0.980856554
AL049798	0.002	0.945703478	0.980901162
CTD-2247C11	0.002	0.945852446	0.980976548
PPAP2A	0.002	0.945936599	0.980976548
GMEB2	0.002	0.945939642	0.980976548
TSC22D1-AS1	0.002	0.945961813	0.980976548
MRPS21P8	0.002	0.945966399	0.980976548
RP13-824C8	0.002	0.945997211	0.980976548
RNA5SP301	0.002	0.946083576	0.981004675
SDHD	0.002	0.946106142	0.981004675
OR5M7P	0.002	0.946170532	0.981024737
RAD23BP2	0.002	0.946273597	0.981086545
CICP4	0.002	0.946313072	0.981106114
AL513327	0.002	0.946478931	0.981192636

RN7SKP10		0.002	0.946574648	0.981244396
RNU6-22P		0.002	0.946590668	0.981244396
RP5-836E8		0.002	0.946815367	0.981405672
MIR6792		0.002	0.946817905	0.981405672
AC010095		0.002	0.946873397	0.981411383
BFAR		0.002	0.947046496	0.981495275
RP11-24J11		0.002	0.947173912	0.98157965
RP11-33E15		0.002	0.947257114	0.981593406
AP000350		0.002	0.947289233	0.981605333
AC004051		0.002	0.947331024	0.981605924
MTND1P33		0.002	0.947390139	0.981633195
RILPL2		0.002	0.947460397	0.981633195
LMF1		0.002	0.947550397	0.981654613
SEPT10P1		0.002	0.947605155	0.981654613
CEACAMP6		0.002	0.947605741	0.981654613
DOC2GP		0.002	0.947625384	0.981654613
DLL3		0.002	0.947681398	0.981654613
AC084010		0.002	0.947687183	0.981654613
RP11-463J7		0.002	0.947839861	0.981722196
	4-Sep	0.002	0.947877791	0.981722196
AL589675		0.002	0.947891515	0.981722196
HTR1B		0.002	0.947896716	0.981722196
POLR2KP1		0.002	0.947965945	0.981772546
RP11-464F9		0.002	0.948013012	0.981799942
RNU6-1102P		0.002	0.948054505	0.981821564
RABL3		0.002	0.94811519	0.98184534
REPS1		0.002	0.948207368	0.981873121
DHRS12		0.002	0.948284763	0.981916273
SNRPCP2		0.002	0.948300939	0.981916273
GSTA1		0.002	0.948310891	0.981916273
RNU6-212P		0.002	0.948367681	0.981933898
C1orf146		0.002	0.948379521	0.981933898
HTT-AS1_1		0.002	0.948410382	0.981933898
KCNV2		0.002	0.948462506	0.981945172
PLSCR2		0.002	0.948498087	0.981960664
GHSR		0.002	0.948634501	0.982059196
ZNF311		0.002	0.948676531	0.982060014
KRTAP19-6		0.002	0.948724729	0.982088562
RP11-453O22		0.002	0.948774474	0.982097366
KATNB1		0.002	0.948817413	0.982120467
RP11-666O2		0.002	0.948859591	0.982142781
FKSG68		0.002	0.948896292	0.982159423
VN1R80P		0.002	0.949048359	0.98225278
TRIB2		0.002	0.949075268	0.982254168
AC140725		0.002	0.949175728	0.982283881
LONRF2P1		0.002	0.949257974	0.982302411
RNA5SP79		0.002	0.949262998	0.982302411
XRN2		0.002	0.949324865	0.982302411
PPIAP24		0.002	0.949327055	0.982302411
MNX1-AS2		0.002	0.949455613	0.982321494
AC016549		0.002	0.94949865	0.982321494
TSNARE1		0.002	0.949506631	0.982321494

ROBO1	0.002	0.949529243	0.98232355
GAL3ST3	0.002	0.949590772	0.982331185
CTC-400I9	0.002	0.949610488	0.982331185
MS4A14	0.002	0.949696662	0.982331185
NAP1L1P1	0.002	0.949751888	0.982331185
CTC-209H22	0.002	0.949785939	0.982331185
MTATP8P2	0.002	0.949815191	0.982331185
RPGRIP1L	0.002	0.949868431	0.982331185
AC007136	0.002	0.94989274	0.982331185
UGT3A2	0.002	0.949921367	0.982331185
SNX9	0.002	0.949944967	0.982331185
TBC1D3K	0.002	0.950015789	0.982331185
PRLHR	0.002	0.950022694	0.982331185
LAP3P2	0.002	0.950058776	0.982331185
OR2S2	0.002	0.950084007	0.982331185
ASCC1	0.002	0.950114565	0.982331185
ZDHHC3	0.002	0.950128916	0.982331185
AC239395	0.002	0.950145686	0.982331185
AC063956	0.002	0.950145686	0.982331185
AC017101	0.002	0.950170034	0.982331185
CTC-265N9	0.002	0.950176014	0.982331185
CTC-788C1	0.002	0.950220547	0.982334577
PRDX4P1	0.002	0.95033785	0.982421242
AL035045	0.002	0.950381765	0.982421242
S100G	0.002	0.950632729	0.98249462
AL157882	0.002	0.950643534	0.98249462
TTC28	0.002	0.950673386	0.982504152
SARNP	0.002	0.950743403	0.982533873
RP11-506K6	0.002	0.950818122	0.982589769
ATP5G1	0.002	0.95088083	0.982594459
HES3	0.002	0.950886497	0.982594459
ATP5G2	0.002	0.950905184	0.982594459
RP11-399H11	0.002	0.950968128	0.98263818
HMGCS1	0.002	0.951016953	0.982645776
CCDC160	0.002	0.951037374	0.982645776
RP11-696N14	0.002	0.951102629	0.982653807
RP11-734C14	0.002	0.951169203	0.982653807
RNU6-1300P	0.002	0.951185335	0.982653807
PPIAP15	0.002	0.951238211	0.982653807
HYOU1	0.002	0.951277896	0.982653807
AC125238	0.002	0.951303975	0.982653807
AK3	0.002	0.951308775	0.982653807
RN7SL233P	0.002	0.951317812	0.982653807
GPX4	0.002	0.951334	0.982653807
AC022384	0.002	0.951462263	0.982713758
NIT1	0.002	0.951594514	0.98273108
SEPW1P	0.002	0.951695439	0.982750069
RPA3	0.002	0.951970726	0.982885164
EDDM3CP	0.002	0.95202294	0.982917767
RN7SL167P	0.002	0.952064997	0.982939881
SPDYC	0.002	0.95224326	0.983059993
RP11-178L8	0.002	0.952309977	0.98310756

RP4-551N11	0.002	0.952372616	0.983150914
AGGF1P3	0.002	0.952493068	0.983232636
RNU6-1301P	0.002	0.952557952	0.983278302
RP11-480C16	0.002	0.952792496	0.983406262
AL137190	0.002	0.952823309	0.983406262
AC099537	0.002	0.952826451	0.983406262
GAPDHP69	0.002	0.95287027	0.983430176
RNU6-881P	0.002	0.952936762	0.983434868
RP11-789F5	0.002	0.953010282	0.983472751
RN7SL752P	0.002	0.953054233	0.983472751
LRRN4	0.002	0.953123494	0.983475101
HIVEP1	0.002	0.953126826	0.983475101
RP11-529E15	0.002	0.953140944	0.983475101
KRT18P62	0.002	0.953183001	0.98349719
DCST1	0.002	0.953209906	0.983503644
RP11-551G24	0.002	0.953289057	0.983564002
RP4-553F4	0.002	0.953700344	0.983732621
ADAM18	0.002	0.953770811	0.983763433
MIR182	0.002	0.953771527	0.983763433
ADAM3B	0.002	0.953861712	0.983771233
bP-2168N6	0.002	0.953951257	0.983777604
RNU6-645P	0.002	0.953959539	0.983777604
RPS7P15	0.002	0.954010331	0.983777604
RP11-479O17	0.002	0.954068226	0.983777604
PGM2	0.002	0.954095104	0.983777604
RP4-581F12	0.002	0.954303894	0.983907684
HDHD1	0.002	0.95448574	0.983988665
AC022486	0.002	0.954567259	0.984051404
RNU6-387P	0.002	0.95458841	0.984051908
OR4X7P	0.002	0.954655203	0.984099462
HCFC2P1	0.002	0.954839384	0.984206054
CORO7-PAM16	0.002	0.955023043	0.984306794
AC025750	0.002	0.955031357	0.984306794
MKX	0.002	0.95514567	0.984306794
LINC01297	0.002	0.955411124	0.984516446
RNU6-822P	0.002	0.955461024	0.984525263
FADD	0.002	0.955502402	0.984546599
LINC01333	0.002	0.955546984	0.984549936
RNA5SP310	0.002	0.955685224	0.984621725
OAZ2	0.002	0.95571427	0.984621725
INSIG2	0.002	0.955877415	0.984733711
VDAC1	0.002	0.956140191	0.98484156
OR56A3	0.002	0.956219217	0.984880358
GMCL1P1	0.002	0.956269683	0.984894774
RNF182	0.002	0.956295941	0.984894774
C20orf96	0.002	0.95635729	0.984894774
RNU7-107P	0.002	0.956389029	0.984906164
AL157883	0.002	0.956463188	0.984938774
SUMO2P15	0.002	0.956526968	0.984943163
DISP2	0.002	0.956627035	0.984943163
RP11-874G11	0.002	0.956637403	0.984943163
TPMTP4	0.002	0.956651907	0.984943163

CDC123	0.002	0.956675329	0.984943163
AC068057	0.002	0.956734043	0.984943163
TTC23L	0.002	0.956734065	0.984943163
TMPRSS4-AS1	0.002	0.956779617	0.984943163
RBMY2EP	0.002	0.956871734	0.984943163
FLJ22763	0.002	0.956987192	0.984943163
RP11-421F16	0.002	0.956991635	0.984943163
RP11-298D21	0.002	0.957017791	0.984943163
TCP11	0.002	0.957030608	0.984943163
KCTD8	0.002	0.957141774	0.984943163
RP11-328D5	0.002	0.957147458	0.984943163
LINC01560	0.002	0.957237898	0.984943163
MACROD1	0.002	0.957312734	0.984943163
NXNL2	0.002	0.957318391	0.984943163
MIR6728	0.002	0.957319905	0.984943163
EIF5AP4	0.002	0.95737061	0.984943163
RP11-334N17	0.002	0.957381545	0.984943163
TCP11X2	0.002	0.9573973	0.984943163
WASH7P	0.002	0.957410632	0.984943163
SSX6	0.002	0.957417725	0.984943163
CA10	0.002	0.957431305	0.984943163
GGTLC2	0.002	0.957519198	0.985000826
AC091654	0.002	0.957547894	0.985000826
EIF3LP1	0.002	0.957682433	0.985053899
C8orf76	0.002	0.957691275	0.985053899
CYP21A1P	0.002	0.957722329	0.985053899
NIP7P3	0.002	0.957729706	0.985053899
RN7SKP198	0.002	0.95775361	0.985053899
CTD-2227C6	0.002	0.95777286	0.985053899
PLA2G12AP2	0.002	0.957915137	0.985135804
RP11-4O3	0.002	0.958046183	0.985195362
RN7SL541P	0.002	0.958080603	0.985195362
RNU6-1028P	0.002	0.95808723	0.985195362
AC009264	0.002	0.95812173	0.985195362
C17orf98	0.002	0.958171764	0.985195362
KYNU	0.002	0.958182521	0.985195362
SMG1P3	0.002	0.958224728	0.985195362
RP11-369J21	0.002	0.958235633	0.985195362
RP11-87M18	0.002	0.958241962	0.985195362
AXDND1	0.002	0.958345779	0.985233502
RNA5SP326	0.002	0.95836213	0.985233502
LDB2	0.002	0.958378328	0.985233502
KCNIP2-AS1	0.002	0.958382492	0.985233502
SOAT2	0.002	0.958567298	0.985376236
GVQW1	0.002	0.95861591	0.985376236
TNFSF12	0.002	0.958635491	0.985376236
RP5-1025A1	0.002	0.958678205	0.985376236
AGKP1	0.002	0.958868868	0.985499516
OR7E7P	0.002	0.958990566	0.985593921
SNTN	0.002	0.959069761	0.985609775
HSBP1	0.002	0.959070633	0.985609775
DPPA3P3	0.002	0.959147095	0.985638643

OR7A11P	0.002	0.959168917	0.985638643
RPL12P23	0.002	0.959224984	0.985664095
MTND4P16	0.002	0.959235955	0.985664095
AC007358	0.002	0.959294062	0.985695111
KIAA1549	0.002	0.95933216	0.985695111
UQCRHP2	0.002	0.959344191	0.985695111
ZNF72P	0.002	0.959465732	0.98575133
ELF5	0.002	0.95951036	0.985775916
RPL21P104	0.002	0.959653284	0.985850437
HSPE1P7	0.002	0.959665693	0.985850437
SPG20	0.002	0.959802135	0.985919036
TCEB1P20	0.002	0.959912273	0.985944521
TOMM22	0.002	0.960036693	0.986040145
RNU6-381P	0.002	0.960063649	0.986046567
RP11-399K21	0.002	0.960108847	0.986051896
MRGPRX1	0.002	0.960132525	0.986051896
CTB-56J15	0.002	0.960148445	0.986051896
RP11-720L8	0.002	0.960230493	0.986111601
AMELX	0.002	0.960319276	0.986160249
RPL39P25	0.002	0.960409229	0.986197737
AC007562	0.002	0.960470566	0.986230551
RP11-288A5	0.002	0.960625893	0.986287017
GIPC3	0.002	0.960717321	0.986287017
RP11-472K17	0.002	0.960735019	0.986287017
SSTR4	0.002	0.960738923	0.986287017
RN7SKP100	0.002	0.960760072	0.986287017
RP1-91J24	0.002	0.96077406	0.986287017
RP11-379J5	0.002	0.960803656	0.98629614
RP4-656G21	0.002	0.960851865	0.986303112
SOX14	0.002	0.96105969	0.986430615
RAD1	0.002	0.961061128	0.986430615
RPL5P7	0.002	0.96109993	0.986430615
ACTBP1	0.002	0.961105392	0.986430615
AC118063	0.002	0.961146806	0.986430615
HIGD1AP1	0.002	0.961199325	0.986447169
OR1D4	0.002	0.961222972	0.986450181
OR2N1P	0.002	0.961328088	0.9865204
UFD1L	0.002	0.961340095	0.9865204
CHCHD2P1	0.002	0.961357348	0.9865204
CTD-2244C20	0.002	0.961374249	0.9865204
VTA1P2	0.002	0.961422336	0.986527234
AC104441	0.002	0.961470842	0.986549343
AC069286	0.002	0.961588235	0.986631751
RP11-368P15	0.002	0.961627279	0.986631751
FOXS1	0.002	0.961627771	0.986631751
FBXO2	0.002	0.961708287	0.986648485
AC130689	0.002	0.961737165	0.986648485
FAM90A1	0.002	0.961737882	0.986648485
SIGMAR1	0.002	0.961745716	0.986648485
A2M	0.002	0.961942437	0.986784536
RNU6-972P	0.002	0.962179631	0.986953847
C4BPAP2	0.002	0.962310808	0.987007532

AC002463	0.002	0.962325609	0.987007532
OPHN1	0.002	0.962368964	0.987025155
RNU6-1018P	0.002	0.962448841	0.987025155
MIR744	0.002	0.962459623	0.987025155
KCNJ6-AS1	0.002	0.962461055	0.987025155
RANBP1	0.002	0.962479079	0.987025155
CTB-60E11	0.002	0.962613387	0.987111374
CTD-2311A18	0.002	0.962687012	0.987144365
CTD-2303H24	0.002	0.96273402	0.987171314
BAALC-AS1	0.002	0.962898655	0.987318871
HSPE1P21	0.002	0.963050237	0.987345382
POTEF	0.002	0.963101999	0.987345382
RP11-135J2	0.002	0.963110842	0.987345382
MARK2P17	0.002	0.963118753	0.987345382
RP11-119K6	0.002	0.963154497	0.987345382
MIR526A1	0.002	0.963175682	0.987345382
AC091633	0.002	0.963223346	0.987345382
C16orf82	0.002	0.963227903	0.987345382
TGFBR3L	0.002	0.963250217	0.987345382
RP11-427G18	0.002	0.963256203	0.987345382
RP11-28B23	0.002	0.963610632	0.987559898
KRT19P6	0.002	0.963725643	0.987612958
RN7SL841P	0.002	0.963745351	0.987612958
NF1P3	0.002	0.963798351	0.987646019
RNU4-48P	0.002	0.963890709	0.987719411
CCDC70	0.002	0.963957256	0.98776015
AC099759	0.002	0.963992683	0.98776015
FO082796	0.002	0.964058193	0.9877673
EDN3	0.002	0.964058908	0.9877673
RP11-357J22	0.002	0.96406188	0.9877673
DEFB119	0.002	0.964217414	0.987862903
RP11-96P7	0.002	0.964413111	0.988038572
ZNF705G	0.002	0.964473641	0.988061649
PLXNB1	0.002	0.964516992	0.988084807
ABLIM2	0.002	0.964593603	0.988132636
MIR99A	0.002	0.964636903	0.988132636
PABPC1P3	0.002	0.964665427	0.988132636
RNU4ATAC5P	0.002	0.96479308	0.988159142
SNAPIN	0.001	0.964902664	0.988224828
CTD-2626G11	0.001	0.964945059	0.988225045
ARHGAP39	0.001	0.965006265	0.988238802
HIST1H3G	0.001	0.965060704	0.988253951
MIR134	0.001	0.965119192	0.988276574
RP11-144G16	0.001	0.965253984	0.9883721
ZNF285	0.001	0.965402395	0.988460312
MIR4442	0.001	0.965562066	0.988577237
RNMTL1P1	0.001	0.965690012	0.988577237
CERS1	0.001	0.965697844	0.988577237
AC097532	0.001	0.965732806	0.988577237
RP5-892F13	0.001	0.965745045	0.988577237
RNA5SP92	0.001	0.965924085	0.988596878
RAPGEF4	0.001	0.965966132	0.988596878

RP11-678L1	0.001	0.966052076	0.988596878
DSG1	0.001	0.966079526	0.988596878
GALNT14	0.001	0.966393191	0.988596878
MIR520H	0.001	0.966449346	0.988596878
RNU1-40P	0.001	0.966449346	0.988596878
NDUFA5P7	0.001	0.966449346	0.988596878
RNU1-41P	0.001	0.966449346	0.988596878
RNU1-97P	0.001	0.966449346	0.988596878
RNU6-256P	0.001	0.966449346	0.988596878
RNU6-546P	0.001	0.966449346	0.988596878
AC093680	0.001	0.966449346	0.988596878
RNU1-86P	0.001	0.966449346	0.988596878
PRR20FP	0.001	0.966449346	0.988596878
SNORD115-18	0.001	0.966449346	0.988596878
RNU1-45P	0.001	0.966449346	0.988596878
RNU1-128P	0.001	0.966449346	0.988596878
SNORD115-10	0.001	0.966449346	0.988596878
SNORD115-27	0.001	0.966449346	0.988596878
SNORD115-29	0.001	0.966449346	0.988596878
SNORD115-19	0.001	0.966449346	0.988596878
USP17L23	0.001	0.966449346	0.988596878
SNORD115-34	0.001	0.966449346	0.988596878
SNORD115-16	0.001	0.966449346	0.988596878
FANK1-AS1	0.001	0.966644377	0.988711703
IL36G	0.001	0.966704213	0.988725423
RP1-200G19	0.001	0.966722799	0.988725423
MIR3915	0.001	0.96677847	0.988725423
AC120104	0.001	0.966782349	0.988725423
GAPDHP33	0.001	0.966895764	0.988756485
HTR3A	0.001	0.966935165	0.988775547
C2orf70	0.001	0.967004286	0.988806706
ERG	0.001	0.967013989	0.988806706
ZNF880	0.001	0.96702792	0.988806706
RN7SL35P	0.001	0.967281899	0.988917785
CTD-2197M16	0.001	0.967343089	0.988937886
DIP2A-IT1	0.001	0.967390534	0.988965161
YWHAH	0.001	0.967471981	0.989027196
TAS2R40	0.001	0.967544316	0.989033073
KRT18P11	0.001	0.967552105	0.989033073
DEPTOR	0.001	0.967560795	0.989033073
RP11-65G9	0.001	0.967613539	0.98906576
RN7SL494P	0.001	0.967716152	0.98910985
RP4-537K23	0.001	0.967739744	0.98910985
PTCSC2	0.001	0.967764105	0.989113523
C1orf68	0.001	0.967833295	0.989157397
MEF2A	0.001	0.967848571	0.989157397
LA16c-313F4	0.001	0.967933468	0.989189602
AC005631	0.001	0.96796316	0.989189602
INO80E	0.001	0.967987018	0.989192759
PI4KAP1	0.001	0.968049276	0.989213931
MTND5P18	0.001	0.968079049	0.98922313
CTD-2033D24	0.001	0.968124972	0.989227608

AC108032	0.001	0.968181847	0.989264499
RP11-255E6	0.001	0.968208849	0.989270865
RNU6-947P	0.001	0.968319085	0.989341051
AC005514	0.001	0.968389932	0.989356156
SERPINA4	0.001	0.968393728	0.989356156
C7orf49	0.001	0.96846985	0.989362866
RP11-459C13	0.001	0.968527395	0.989362866
CDK5RAP2	0.001	0.9686575	0.9894672
CTD-2216M2	0.001	0.968671083	0.9894672
MIR4754	0.001	0.968736449	0.9894862
NMNAT1P1	0.001	0.968760188	0.9894862
CSF3	0.001	0.968772787	0.9894862
CTB-60B18	0.001	0.968906901	0.989587208
RPL21P43	0.001	0.968913236	0.989587208
RP11-140K17	0.001	0.969011907	0.989609147
LINC00351	0.001	0.969017113	0.989609147
RP11-368L12	0.001	0.969029209	0.989609147
RP11-161D15	0.001	0.969043052	0.989609147
REEP3	0.001	0.96908157	0.989609147
RP1-229K20	0.001	0.969097928	0.989609147
PLEKHM1	0.001	0.96910421	0.989609147
AC127904	0.001	0.969148185	0.989609147
RP11-272D12	0.001	0.969201463	0.989609147
MIR191	0.001	0.969226519	0.989609147
ZNF229	0.001	0.969255728	0.989609147
FAM189B	0.001	0.969287948	0.989609147
TUBBP6	0.001	0.969344587	0.989627051
RP11-720N19	0.001	0.969347333	0.989627051
RP11-258F22	0.001	0.96936782	0.989627051
CCT7	0.001	0.969415085	0.989631666
DDX43	0.001	0.969419794	0.989631666
RP11-68I18	0.001	0.969465125	0.98963361
MRPL46	0.001	0.969534385	0.989669813
MIR34B	0.001	0.969593631	0.989678124
MIR1231	0.001	0.969656306	0.989678124
RP11-472I20	0.001	0.969675756	0.989678124
RNU6-1186P	0.001	0.969727267	0.989678124
PTGES3P5	0.001	0.9697991	0.989685623
DOCK4	0.001	0.969820012	0.989685623
RP11-702C7	0.001	0.969884111	0.989723797
FAM106CP	0.001	0.969905387	0.989723797
TEX30	0.001	0.969933654	0.989723797
RP11-35J23	0.001	0.969974548	0.989723797
RP11-111A24	0.001	0.969986131	0.989723797
NPM1P48	0.001	0.970084756	0.989786128
XIRP1	0.001	0.970167242	0.989827879
RFX1	0.001	0.970218415	0.989831055
MIR5091	0.001	0.970293922	0.989831055
HMGB1P21	0.001	0.970315836	0.989831055
PCCA	0.001	0.970491859	0.989919707
AC068292	0.001	0.970493448	0.989919707
RN7SKP90	0.001	0.970539634	0.989924367

OR10J2P	0.001	0.970795472	0.990021622
RN7SL156P	0.001	0.970821801	0.990021622
TRMT112P2	0.001	0.970830097	0.990021622
ALG1L	0.001	0.970854554	0.990021622
KRTAP4-9	0.001	0.970857872	0.990021622
AC074121	0.001	0.970906954	0.990021622
CNOT10-AS1	0.001	0.970914418	0.990021622
RP5-930J4	0.001	0.971307654	0.990273613
RP11-262I2	0.001	0.97134867	0.990290759
RP11-647O20	0.001	0.97138287	0.990304428
GJD2	0.001	0.971413669	0.990314628
RP11-89K21	0.001	0.971454218	0.990334769
AC079233	0.001	0.97158826	0.990407817
GFY	0.001	0.971644262	0.990443706
AP000705	0.001	0.971739329	0.990478386
OR2T5	0.001	0.971742866	0.990478386
PLXNA4	0.001	0.971800781	0.990478386
MYO15A	0.001	0.97182386	0.990478386
DCTN5	0.001	0.971873501	0.990481713
GH2	0.001	0.97190334	0.990481713
LINC01398	0.001	0.971910311	0.990481713
AL109750	0.001	0.972167763	0.990659527
ZNF123P	0.001	0.972167992	0.990659527
RNU6-815P	0.001	0.972254796	0.990726785
RNU6-228P	0.001	0.972380114	0.990796499
ABCF1	0.001	0.972429143	0.990819648
RP11-485B17	0.001	0.972485249	0.990829537
NCSTN	0.001	0.972507526	0.990829537
AC005588	0.001	0.972666379	0.990911737
RP11-384D10	0.001	0.972697434	0.990911737
OR4A46P	0.001	0.972706774	0.990911737
C9orf9	0.001	0.972778385	0.990949495
RP11-434C1	0.001	0.972797113	0.990949495
OSBPL10	0.001	0.972835867	0.990956406
LZTS1-AS1	0.001	0.972884576	0.990956406
KLHL26	0.001	0.972894655	0.990956406
RP11-98C8	0.001	0.972919509	0.990956406
AL031655	0.001	0.972967565	0.990956406
TCAP	0.001	0.972972605	0.990956406
TMEM246-AS1	0.001	0.973090074	0.991026643
AC090707	0.001	0.973134926	0.991043355
PDE4D	0.001	0.9731481	0.991043355
RP11-315F22	0.001	0.973169952	0.991044418
TMEM171	0.001	0.973583176	0.991251936
MIR130A	0.001	0.973611326	0.991251936
RP11-123C21	0.001	0.973638745	0.991251936
MAGEB10	0.001	0.973723508	0.991251936
RN7SL390P	0.001	0.973758894	0.991251936
CCDC37	0.001	0.973777317	0.991251936
RPS12P26	0.001	0.97385573	0.991281263
SNORD74	0.001	0.973860421	0.991281263
PTP4A2P1	0.001	0.973904531	0.99128379

AKR1D1	0.001	0.974070691	0.99141054
OR5BC1P	0.001	0.974145639	0.991423261
AC105402	0.001	0.974266711	0.991504106
RP11-248B24	0.001	0.974404351	0.991613428
RP11-514D23	0.001	0.974415774	0.991613428
OR2T1	0.001	0.974465832	0.991643181
FAM99B	0.001	0.974492531	0.991649163
RP1-212G6	0.001	0.974622056	0.991756754
GTF2IP2	0.001	0.974719196	0.991759558
RP11-463I20	0.001	0.97471957	0.991759558
CDY8P	0.001	0.974741587	0.991759558
HIST1H2AA	0.001	0.974798825	0.991771024
LINC00424	0.001	0.974820521	0.991771024
TICAM2	0.001	0.974936129	0.991827523
MIR1-1HG	0.001	0.975033902	0.991869222
RP11-85G20	0.001	0.975213549	0.991953639
AC018463	0.001	0.975279697	0.991953639
AGBL1-AS1	0.001	0.975282073	0.991953639
RN7SL423P	0.001	0.975405976	0.991998715
PRDM9	0.001	0.975440062	0.991998715
RP5-831C21	0.001	0.975511839	0.992008164
RNU1-75P	0.001	0.975549109	0.992010601
STARP1	0.001	0.975570317	0.992010601
PPIAP20	0.001	0.975576722	0.992010601
RPL3P7	0.001	0.975662224	0.992068369
GRIFIN	0.001	0.975675192	0.992068369
RP11-254F19	0.001	0.975699209	0.992071609
RNU7-61P	0.001	0.975885619	0.99217642
RNA5SP19	0.001	0.976042794	0.992228653
KCNK4	0.001	0.976061994	0.992228653
RP11-490P13	0.001	0.97611774	0.992264142
RP11-565P22	0.001	0.97631244	0.992419699
RP11-294L11	0.001	0.976448191	0.992476813
RP5-1106H14	0.001	0.976464482	0.992476813
ZFAND5	0.001	0.976517596	0.992501142
AL133475	0.001	0.976766422	0.992673152
MIR16-2	0.001	0.976895964	0.992747366
RNA5SP99	0.001	0.976932542	0.992753396
SNORD78	0.001	0.977011142	0.992782055
MIR625	0.001	0.977098303	0.992790755
RP11-18B3	0.001	0.977166999	0.992790755
USF2	0.001	0.97732944	0.992790755
LINC00333	0.001	0.977357921	0.992790755
RP11-507J18	0.001	0.977413384	0.992790755
RMST_5	0.001	0.977440316	0.992790755
MIR421	0.001	0.977460087	0.992790755
RP11-90B22	0.001	0.977583689	0.992790755
MIR4792	0.001	0.977589068	0.992790755
TRAJ46	0.001	0.977612632	0.992790755
CTC-543D15	0.001	0.97762387	0.992790755
DGCR11	0.001	0.977647826	0.992790755
RP11-848D3	0.001	0.977660944	0.992790755

RP11-886D15	0.001	0.977669448	0.992790755
RP1-86D1	0.001	0.977719093	0.992790755
CHRAC1	0.001	0.977724047	0.992790755
C17orf96	0.001	0.977746696	0.992790755
AP006288	0.001	0.978031055	0.992996875
CTD-2189E23	0.001	0.978080219	0.993008603
AC062022	0.001	0.978208841	0.993075681
RP1-49C23	0.001	0.978324624	0.993140935
HRSP12	0.001	0.978359523	0.993140935
SNRPEP4	0.001	0.978377381	0.993140935
ANKRD50	0.001	0.978453605	0.993175973
KIT	0.001	0.978476254	0.993177796
OR51I1	0.001	0.978567228	0.993227801
HOTAIR_5	0.001	0.978588725	0.993228454
RP11-326J18	0.001	0.978688315	0.993308366
RNU6-1228P	0.001	0.978798543	0.993313272
AC104458	0.001	0.978899908	0.993313272
RP11-662G23	0.001	0.978901709	0.993313272
MIR3689F	0.001	0.979002917	0.993352477
SAP30L-AS1	0.001	0.979028117	0.993356884
PTCHD3P2	0.001	0.979221685	0.993384211
CUX1	0.001	0.979292074	0.993384211
RNU4-84P	0.001	0.979302769	0.993384211
ASB6	0.001	0.979348698	0.993384211
AC090625	0.001	0.979376387	0.993384211
IL9RP4	0.001	0.979384628	0.993384211
ANK3	0.001	0.979388771	0.993384211
PLCL2-AS1	0.001	0.97943721	0.993412185
AL158155	0.001	0.979467221	0.993421468
OR10J1	0.001	0.979849996	0.99370389
RP11-95J11	0.001	0.979891207	0.993724523
RNU2-49P	0.001	0.97993318	0.993745929
ADH5P4	0.001	0.9801796	0.993901764
EVA1C	0.001	0.980198953	0.993901764
GNA15	0.001	0.980232928	0.993901764
FP236383	0.001	0.980366781	0.99399516
RP11-650J17	0.001	0.980590178	0.994112798
ALKBH6	0.001	0.980601437	0.994112798
GNB4	0.001	0.980688992	0.994170409
KRTAP22-1	0.001	0.980765733	0.994170409
RNU6ATAC15P	0.001	0.980813121	0.994193728
CUL4AP1	0.001	0.980867241	0.994227426
RP11-556I13	0.001	0.980917044	0.994251274
CDC42EP3	0.001	0.980948171	0.994251274
TTC3-AS1	0.001	0.981016023	0.994251274
MIR5092	0.001	0.981089242	0.994291655
CTD-2593A12	0.001	0.981185929	0.994320848
HIST1H2APS1	0.001	0.981243627	0.994354991
MIR6801	0.001	0.981441866	0.994437652
RP11-723C11	0.001	0.981453915	0.994437652
KRTAP4-2	0.001	0.981476228	0.994437652
POLRMT	0.001	0.981492236	0.994437652

RN7SL383P	0.001	0.981664787	0.99451085
OR7E1P	0.001	0.98176202	0.994561997
MBL3P	0.001	0.981857214	0.994561997
RN7SL455P	0.001	0.981937871	0.994592948
RNU6-205P	0.001	0.98206599	0.994638109
RP11-958F21	0.001	0.982076206	0.994638109
ACTRT1	0.001	0.982128646	0.994638109
AC109496	0.001	0.982254982	0.99471978
RP11-1B20	0.001	0.982282732	0.99471978
RP13-820C6	0.001	0.982351281	0.99471978
ADAM17	0.001	0.982403966	0.99471978
RP11-127L21	0.001	0.982420765	0.99471978
RNA5SP100	0.001	0.982435985	0.99471978
GRK5	0.001	0.982443172	0.99471978
OSTCP6	0.001	0.982474301	0.99471978
BCL10	0.001	0.982489347	0.99471978
WARS2	0.001	0.982599122	0.99471978
LMAN2L	0.001	0.982617854	0.99471978
WBSCR17	0.001	0.982627002	0.99471978
SFT2D1	0.001	0.982980289	0.994869108
RNF39	0.001	0.983013397	0.994869108
SNF8	0.001	0.983046068	0.994869108
LMO7DN-IT1	0.001	0.983188068	0.99494939
C2orf91	0.001	0.983267897	0.994967213
AC008869	0.001	0.983303873	0.994967213
FTSJ3	0.001	0.983349263	0.994967213
NPC1L1	0.001	0.98335754	0.994967213
MDK	0.001	0.983393697	0.994967213
NAP1L5	0.001	0.983452942	0.994990728
RN7SL397P	0.001	0.983479613	0.994990728
BOLA3P4	0.001	0.983553291	0.995021973
FAM182B	0.001	0.983636777	0.995021973
LINC01552	0.001	0.983645079	0.995021973
RP13-714J12	0.001	0.983709716	0.995021973
SNORA30	0.001	0.9837533	0.995021973
AE000659	0.001	0.983756219	0.995021973
CCNHP1	0.001	0.983849667	0.995046493
ST7	0.001	0.984032766	0.995190975
RP5-964N17	0.001	0.984063613	0.995201038
SH2D4B	0.001	0.984175129	0.995271549
HDX	0.001	0.984261039	0.995273589
RSL24D1P5	0.001	0.98434554	0.995273589
MIR4479	0.001	0.984366551	0.995273589
MIR4676	0.001	0.984431705	0.995273589
PRSS54	0.001	0.98446568	0.995273589
RNA5SP474	0.001	0.984478702	0.995273589
MAGED2	0.001	0.98451763	0.995273589
CACNA1C-AS1	0.001	0.984607494	0.995273589
AC009110	0.001	0.98462645	0.995273589
OR7E125P	0.001	0.984697276	0.995273589
AC138942	0.001	0.984704395	0.995273589
RP11-628E19	0.001	0.984761548	0.995273589

DNAJA1P6	0.001	0.98477444	0.995273589
RNA5SP47	0.001	0.984835924	0.995273589
OR2A5	0.001	0.984846647	0.995273589
RP11-554D13	0.001	0.984940363	0.995273589
UPK1A-AS1	0.001	0.98497655	0.995273589
ATP1B2	0.001	0.984982895	0.995273589
PTMAP5	0.001	0.984985509	0.995273589
GDF2	0.001	0.98499076	0.995273589
AC007327	0.001	0.98508774	0.995273589
AL355794	0.001	0.985088209	0.995273589
EIF4H	0.001	0.98513842	0.995273589
RP11-448G4	0.001	0.985270805	0.995343681
RNA5SP309	0.001	0.985298735	0.995343681
RP11-601I15	0.001	0.985313077	0.995343681
TAOK2	0.001	0.985355372	0.995343681
RPL30	0.001	0.985378897	0.995343681
RP11-203H2	0.001	0.985378956	0.995343681
F10	0.001	0.985505235	0.995369692
CDC25B	0.001	0.98555882	0.995370818
DEFB105B	0.001	0.985617693	0.995370818
RNA5SP106	0.001	0.985617693	0.995370818
RNU6-906P	0.001	0.985622121	0.995370818
TUBB1P1	0.001	0.985656709	0.995374922
ASRGL1	0.001	0.98574005	0.995416873
OR2X1P	0.001	0.985766382	0.995422358
AP001062	0.001	0.985950035	0.995517802
KDM4A	0.001	0.986072889	0.995563002
RNU6-128P	0.001	0.986154532	0.995624325
RPEL1	0.001	0.986207719	0.995656916
RNU6-268P	0.001	0.986248783	0.995677268
OR6J1	0.001	0.98631464	0.995701543
RPL3P3	0.001	0.986459588	0.995805655
KIAA1211L	0.001	0.986641858	0.995888404
RP11-716O23	0.001	0.986704475	0.995888404
RPL7P41	0.001	0.986741641	0.995888404
MIR4719	0.001	0.986774797	0.995888404
FKSG48	0.001	0.986845577	0.995888404
MIR6822	0.001	0.986889909	0.995888404
SPICE1	0.001	0.986904997	0.995888404
RNU6-122P	0.001	0.986906516	0.995888404
LINC01352	0.001	0.986917944	0.995888404
SNORD114-15	0.001	0.987094895	0.995963012
SOST	0.001	0.987097185	0.995963012
RN7SKP109	0.001	0.987126984	0.995963012
AC100767	0.001	0.987138261	0.995963012
MIR550A1	0.001	0.987207515	0.995966948
RSF1-IT2	0.001	0.987252803	0.995966948
CTC-379B2	0.001	0.987267633	0.995966948
THNSL2	0.001	0.987306679	0.995985241
AC010240	0.001	0.987378704	0.996028965
GK2	0.001	0.987425201	0.996028965
RP11-88E10	0.001	0.987437815	0.996028965

KIAA1045	0.001	0.987502281	0.996028965
RN7SKP178	0.001	0.987542277	0.996028965
RP11-1070A24	0.001	0.987559153	0.996028965
ASAP3	0.001	0.987655213	0.996071355
SYNCRIP	0.001	0.987673511	0.996071355
RP11-120C12	0.001	0.987673661	0.996071355
MRPL16	0.001	0.987684838	0.996071355
SNORD15B	0.001	0.987820597	0.99616103
CTB-162E12	0.001	0.987828843	0.99616103
AC083826	0.001	0.987836506	0.99616103
OR2AM1P	0.001	0.987909348	0.9961923
APCS	0.001	0.98798968	0.996217625
OR5AW1P	0.001	0.988192689	0.996260347
AC005775	0.001	0.988215924	0.996260347
RNU6-843P	0.001	0.988218065	0.996260347
RNU6-808P	0.001	0.988281691	0.996260347
RNU6-656P	0.000	0.988307848	0.996260347
AC091729	0.000	0.988344881	0.996260347
TRAIP	0.000	0.988446393	0.996260347
RP11-404K5	0.000	0.988450993	0.996260347
RPL22	0.000	0.988470791	0.996260347
ANP32B	0.000	0.988570561	0.996260347
OR6C75	0.000	0.988581626	0.996260347
PRICKLE3	0.000	0.988633734	0.996260347
TMEM213	0.000	0.988712102	0.996260347
TP53	0.000	0.988778379	0.996260347
RNU6-717P	0.000	0.988813523	0.996260347
MIR190B	0.000	0.988868461	0.996260347
PTCD2P2	0.000	0.988876001	0.996260347
AKAP8L	0.000	0.988884249	0.996260347
CLPTM1L	0.000	0.988922153	0.996260347
RP11-349K16	0.000	0.989023651	0.996260347
RP11-460B17	0.000	0.98902758	0.996260347
TXNDC5	0.000	0.989059827	0.996260347
MIR4287	0.000	0.989064564	0.996260347
HNRNPA3P7	0.000	0.989195099	0.996267392
HAUS8	0.000	0.989197067	0.996267392
HSD17B8	0.000	0.989233228	0.996282744
AC073071	0.000	0.98925975	0.996288387
SPERT	0.000	0.989326875	0.996300513
RNA5SP280	0.000	0.989496729	0.996391685
RN7SL623P	0.000	0.989508764	0.996391685
GS1-5L10	0.000	0.989542769	0.996403758
SNAPC4	0.000	0.989675882	0.996468615
RP11-168P6	0.000	0.98972074	0.996468615
FCF1P5	0.000	0.98973162	0.996468615
BRICD5	0.000	0.98987499	0.996526037
RP11-75C23	0.000	0.989914194	0.996526037
SYCN	0.000	0.990133947	0.996578742
AL021395	0.000	0.990224432	0.996591176
HNRNPA1P29	0.000	0.990239085	0.996591176
RASGRP4	0.000	0.990373852	0.996668369

EXOGP1	0.000	0.990374643	0.996668369
TDRD5	0.000	0.990390407	0.996668369
OTUB1	0.000	0.990439642	0.996696856
XXbac-BPG294E21	0.000	0.990480856	0.996717272
RP11-589B3	0.000	0.990546438	0.996741146
RP4-800O15	0.000	0.990723347	0.996888804
PCDH9	0.000	0.990865311	0.996935636
AC095030	0.000	0.990891049	0.99694047
RP11-204E9	0.000	0.991185667	0.99709982
ALG1L9P	0.000	0.991216481	0.99709982
ARL2BP	0.000	0.991216916	0.99709982
CYP4F27P	0.000	0.991263652	0.997104713
RP11-496H1	0.000	0.991356339	0.997132848
PGBD4P5	0.000	0.991425625	0.997132848
FAM58BP	0.000	0.991438185	0.997132848
GOLGA8A	0.000	0.991450885	0.997132848
GRWD1	0.000	0.99149489	0.997132848
TMIE	0.000	0.991500985	0.997132848
TMCO1	0.000	0.991751515	0.997300566
RP11-21L19	0.000	0.991888789	0.997388951
RP4-813F11	0.000	0.991898669	0.997388951
HSFY2	0.000	0.991979754	0.997424784
RNA5SP117	0.000	0.992009531	0.997430495
PPARD	0.000	0.992078289	0.997430495
PCNAP3	0.000	0.992096332	0.997430495
RP11-475D12	0.000	0.992125637	0.997430495
AC005355	0.000	0.992132032	0.997430495
RIMS2	0.000	0.992162587	0.997440159
RP11-202G11	0.000	0.992428819	0.997612803
SDCCAG3P2	0.000	0.992438631	0.997612803
PDGFB	0.000	0.992439049	0.997612803
MIR3118-3	0.000	0.992661046	0.99777278
RP11-439C8	0.000	0.992776026	0.997790669
GYG1	0.000	0.992853838	0.997790669
AP3B2	0.000	0.992858413	0.997790669
RP11-272P10	0.000	0.992882489	0.997790669
DCTN1-AS1	0.000	0.992917637	0.997790669
SNRPGP16	0.000	0.992931303	0.997790669
PCSK1	0.000	0.992974092	0.997790669
ZNF444	0.000	0.993009384	0.997790669
TEX15	0.000	0.993027625	0.997790669
RP1-34L19	0.000	0.993147451	0.997861559
EBF4	0.000	0.993326141	0.997941742
PDLIM5	0.000	0.993371019	0.99795987
MIR1911	0.000	0.99357605	0.997973589
AC073069	0.000	0.993600929	0.997973589
SETP1	0.000	0.993616689	0.997973589
GPR35	0.000	0.993773176	0.997973589
SAMD12-AS1	0.000	0.993830249	0.997973589
GPR12	0.000	0.993831058	0.997973589
BSCL2	0.000	0.99384594	0.997973589
KRT18P18	0.000	0.993853796	0.997973589

RP11-216N21	0.000	0.993873551	0.997973589
RP11-118E9	0.000	0.99388757	0.997973589
RP4-559A3	0.000	0.993961214	0.997992207
AL161935	0.000	0.993972594	0.997992207
CTD-2110K23	0.000	0.993989928	0.997992207
Z98052	0.000	0.99403215	0.998006786
XXbac-BPGBPG34I8	0.000	0.994187119	0.998021877
AC073218	0.000	0.994294324	0.998061054
RP11-190A12	0.000	0.994309969	0.998061054
LINC00371	0.000	0.994347781	0.998062283
CXorf36	0.000	0.994353105	0.998062283
GABRB3	0.000	0.994414539	0.998090119
RN7SKP122	0.000	0.994425084	0.998090119
SALL1P1	0.000	0.99450732	0.998090119
RP5-1054A22	0.000	0.994527532	0.998090119
RN7SL170P	0.000	0.994555787	0.998090809
RP11-166J22	0.000	0.994570132	0.998090809
RP11-164O23	0.000	0.994700056	0.99820016
RNU1-11P	0.000	0.994788373	0.998267753
RP1-131F15	0.000	0.994934702	0.998351488
AC010132	0.000	0.99501082	0.998385799
CORO7	0.000	0.995186621	0.998498882
RNU6-654P	0.000	0.995205667	0.998498882
HES6	0.000	0.995244135	0.998498882
CYCSP35	0.000	0.995244821	0.998498882
RP11-852E15	0.000	0.995272921	0.998498882
TBC1D28	0.000	0.995275699	0.998498882
AC141928	0.000	0.995302826	0.998498882
PCNAP1	0.000	0.995384742	0.998498892
AC004816	0.000	0.995508003	0.998534633
PSMF1	0.000	0.995560463	0.998550582
PPM1AP1	0.000	0.995573402	0.998550582
OR2K2	0.000	0.995635827	0.998552308
RP11-163G10	0.000	0.995681798	0.998554158
PDK4	0.000	0.995776142	0.998610382
SLC9A2	0.000	0.995802656	0.998612282
RNA5SP517	0.000	0.995867219	0.998615972
AC116050	0.000	0.996069279	0.99875348
RP11-582E3	0.000	0.996301615	0.998839412
RN7SL687P	0.000	0.996440992	0.99891368
MED21	0.000	0.996448015	0.99891368
YBX1P10	0.000	0.996472415	0.99891368
LINC00443	0.000	0.996501234	0.99891368
RNU4ATAC14P	0.000	0.996501706	0.99891368
RP11-769N19	0.000	0.996589694	0.99895983
FOXH1	0.000	0.996759547	0.999045982
RP11-60C6	0.000	0.996856203	0.999049484
GRK1	0.000	0.996873425	0.999049484
AC098795	0.000	0.996920916	0.999049484
RNU7-46P	0.000	0.996968615	0.999049484
RN7SL101P	0.000	0.997013312	0.999049484
REG1B	0.000	0.997021354	0.999049484

GJA1P1	0.000	0.997046972	0.999049484
SNHG12	0.000	0.997053227	0.999049484
RPS29P22	0.000	0.997141321	0.999049484
FKBP4P2	0.000	0.997260656	0.999065119
COLGALT2	0.000	0.997278493	0.999065119
RNA5SP481	0.000	0.997318246	0.999065119
AC138744	0.000	0.99732697	0.999065119
CCNC	0.000	0.997488945	0.999107496
CTC-529P8	0.000	0.997586909	0.999168552
KATNBL1P5	0.000	0.997612023	0.999168552
AC004878	0.000	0.997666707	0.999168552
RNU6-987P	0.000	0.997679191	0.999168552
RP11-236L14	0.000	0.997700019	0.999168552
PNKD	0.000	0.997864535	0.999261241
ZBTB8A	0.000	0.997948193	0.999269059
RP11-527N12	0.000	0.998282067	0.999409117
PSG3	0.000	0.998300669	0.999409117
CKB	0.000	0.998346441	0.999409117
RGS7	0.000	0.998346961	0.999409117
CTD-2138O14	0.000	0.998353807	0.999409117
C3orf56	0.000	0.998374215	0.999409117
GNGT1	0.000	0.99838486	0.999409117
RP11-249C24	0.000	0.998516712	0.99948205
RP11-154H23	0.000	0.998704385	0.999606879
HCCAT5	0.000	0.998785222	0.999660234
RP11-436H11	0.000	0.998900455	0.999698081
PMS2P6	0.000	0.998958626	0.999735291
YWHAE	0.000	0.999016578	0.99977228
HNRNPFP1	0.000	0.999095875	0.99980962
RP11-46D6	0.000	0.999156009	0.999827781
MIR1208	0.000	0.999208899	0.999832579
RP11-406H21	0.000	0.99927254	0.999832579
RN7SL447P	0.000	0.999321475	0.999832579
CASC6	0.000	0.999339967	0.999832579
MTCO3P2	0.000	0.999373238	0.999832579
RPL24P7	0.000	0.999382343	0.999832579
LINC01537	0.000	0.999391726	0.999832579
CCDC129	0.000	0.999433304	0.999846197
MOCS2	0.000	0.999476699	0.999854581
MIR101-2	0.000	0.999538346	0.999895249
RN7SKP153	0.000	0.999676666	0.99996483
CTC-353G13	0.000	0.999749598	0.99996483
GOPC	0.000	0.999771682	0.99996483
OR5D16	0.000	0.999778311	0.99996483
UBTFL10	0.000	0.99981786	0.99996483
RPL10AP1	0.000	0.999860555	0.999983411
RP11-168O16	0.000	0.99987843	0.999983411
RP11-109E10	0.000	0.99990178	0.999985765
IFT46	0.000	0.999993464	0.999993464
RN7SL107P	0.000	0.99998868	0.999993464
BANCR	0.000	0.999963819	0.999993464
SNORD116-9	0.000	0.999935375	0.999993464

LINC01078	0.000	0.999806373	0.99996483
PGAM5P1	0.000	0.9997653	0.99996483
PRKRA	0.000	0.999709374	0.99996483
RNU6-426P	0.000	0.999672831	0.99996483
UBTFL8	0.000	0.999609514	0.99994544
AQP7P3	0.000	0.999447325	0.999846197
RIOK3P1	0.000	0.999375619	0.999832579
AF127577	0.000	0.999364497	0.999832579
RNU6-193P	0.000	0.999361366	0.999832579
COMMD3-BMI1	0.000	0.999189988	0.999832579
EEF1B2	0.000	0.999148602	0.999827781
ZBTB9	0.000	0.999055865	0.999790588
IGKV2D-38	0.000	0.998896811	0.999698081
SNORD38	0.000	0.99882066	0.999660234
PHBP14	0.000	0.998815555	0.999660234
AC010376	0.000	0.998700735	0.999606879
RNU7-148P	0.000	0.998661426	0.999605896
SLC9A3P1	0.000	0.99850704	0.99948205
AL391559	0.000	0.998401881	0.999409117
SNRPC	0.000	0.998352105	0.999409117
LAS1L	0.000	0.998179124	0.999375177
LINC00459	0.000	0.998119193	0.999336181
AC004074	0.000	0.998061746	0.999299672
RP13-436F16	0.000	0.998048694	0.999299672
RP11-61N20	0.000	0.998041001	0.999299672
SNORA76	0.000	0.997968228	0.999269059
TASP1	0.000	0.997938171	0.999269059
RNU6-1054P	0.000	0.997897477	0.999261241
PDXP	0.000	0.997886756	0.999261241
CTF2P	0.000	0.997869506	0.999261241
AL121931	0.000	0.997729131	0.999176696
ACMSD	0.000	0.997688892	0.999168552
AC083875	0.000	0.997654041	0.999168552
AP001604	0.000	0.997492209	0.999107496
RP11-90J7	0.000	0.997482956	0.999107496
RN7SL97P	0.000	0.997424499	0.999102711
FTH1P4	0.000	0.997414436	0.999102711
OR1E1	0.000	0.997345016	0.999065119
CICP24	0.000	0.997327336	0.999065119
ESPN	0.000	0.997203549	0.999049484
RP5-845O24	0.000	0.997187495	0.999049484
MIR5682	0.000	0.997178049	0.999049484
SOD1	0.000	0.997176535	0.999049484
DCPS	0.000	0.997174792	0.999049484
RP11-62L18	0.000	0.997123086	0.999049484
RFPL3S	0.000	0.997112918	0.999049484
RP11-123C5	0.000	0.997071217	0.999049484
SNORA43	0.000	0.996929323	0.999049484
CTD-3012A18	0.000	0.996840637	0.999049484
SALL2	0.000	0.996825346	0.999049484
RN7SKP258	0.000	0.996800267	0.999049484
MIR6867	0.000	0.996755701	0.999045982

DNLZ	0.000	0.996730793	0.999045982
MED31	0.000	0.996619252	0.998968433
EIF5B	0.000	0.996574373	0.99895983
RP11-54J7	0.000	0.996410935	0.99891368
AP005901	0.000	0.996301785	0.998839412
RNU6-581P	0.000	0.996285754	0.998839412
RP11-351A20	0.000	0.996228596	0.998829113
HSD3BP1	0.000	0.996220831	0.998829113
OR2Q1P	0.000	0.996205704	0.998829113
RNU6-1187P	0.000	0.99618995	0.998829113
RN7SL202P	0.000	0.995998213	0.998703249
ELP6	0.000	0.995963935	0.998689903
MOG	0.000	0.995869238	0.998615972
LINC00508	0.000	0.995865145	0.998615972
RP11-108A14	0.000	0.995779794	0.998610382
RP11-85L21	0.000	0.995663366	0.998554158
SMAD1	0.000	0.99563802	0.998552308
RP11-231I13	0.000	0.995626244	0.998552308
MIRLET7F1	0.000	0.995515569	0.998534633
ATP1B4	0.000	0.995439758	0.99850065
MT-TQ	0.000	0.995417041	0.998498892
SNORD51	0.000	0.995403723	0.998498892
RP11-197B12	0.000	0.995401199	0.998498892
RP11-320J16	0.000	0.995379943	0.998498892
MIR487A	0.000	0.995312206	0.998498882
RPL37P25	0.000	0.995280931	0.998498882
PAX8-AS1	0.000	0.994965715	0.998361574
GAPDHP23	0.000	0.994844636	0.998282145
KB-1991G8	0.000	0.99482578	0.998282145
LINC01251	0.000	0.994519157	0.998090119
RNU7-62P	0.000	0.994515478	0.998090119
RP11-718B12	0.000	0.994514214	0.998090119
ZNF877P	0.000	0.994259068	0.998052031
RNA5SP141	0.000	0.994210417	0.998024229
VWA8-AS1	0.000	0.994186741	0.998021877
RNU6-1323P	0.000	0.994185664	0.998021877
RP5-1180C18	0.000	0.99415323	0.998021877
AC091042	0.000	0.994106246	0.998021877
GDF10	0.000	0.994105511	0.998021877
RP13-395E19	0.000	0.994046359	0.998006786
DCT	0.000	0.993966323	0.997992207
RP11-61L23	0.000	0.993837661	0.997973589
TUBGCP2	0.000	0.993831561	0.997973589
OR2A12	0.000	0.993795385	0.997973589
RNU5F-8P	0.000	0.993761371	0.997973589
RP11-221J22	0.000	0.993714209	0.997973589
RP11-438B23	0.000	0.993704363	0.997973589
RP5-1136G2	0.000	0.993629373	0.997973589
NUP62CL	0.000	0.993622857	0.997973589
EVX2	0.000	0.993614825	0.997973589
MTCO2P3	0.000	0.993563117	0.997973589
AC004812	0.000	0.993499958	0.997973589

STXBP1	0.000	0.993498681	0.997973589
CTAG1B	0.000	0.993425265	0.997972266
MIR1273F	0.000	0.993406998	0.997972266
NPTX1	0.000	0.993332021	0.997941742
TRIM8	0.000	0.993291285	0.997941742
RPL13AP	0.000	0.993264949	0.99793751
RP1-297M16	0.000	0.993200764	0.997894074
OR52A4	0.000	0.993055945	0.997790669
ALG1	0.000	0.993042933	0.997790669
RP5-1139B12	0.000	0.992990649	0.997790669
ARMC3	0.000	0.992988444	0.997790669
OR13Z2P	0.000	0.992939487	0.997790669
DEFB133	0.000	0.992858342	0.997790669
RP11-760L24	0.000	0.99280888	0.997790669
RNU1-19P	0.000	0.992785938	0.997790669
OGFR-AS1	0.000	0.992693766	0.997784611
AC005324	0.000	0.992589144	0.997721565
AF001548	0.000	0.992470855	0.997623719
CNTN4	0.000	0.992319667	0.997555961
TRBV24OR9-2	0.000	0.992191802	0.997448475
CTD-2586B10	0.000	0.99212747	0.997430495
AC107871	0.000	0.99209524	0.997430495
CTD-2515C13	0.000	0.991923175	0.997388951
RNA5SP438	0.000	0.991921293	0.997388951
ASPSCR1	0.000	0.991603378	0.997172655
ITIH3	0.000	0.991565473	0.99715559
AC005783	0.000	0.991545205	0.99715559
CCDC171	0.000	0.991462213	0.997132848
DNMT3L	0.000	0.99142107	0.997132848
SERPINA7P1	0.000	0.991374948	0.997132848
RP1-93L7	0.000	0.991362155	0.997132848
CTD-2124B20	0.000	0.99124862	0.997104713
IL9RP2	0.000	0.991187967	0.99709982
RP11-20E24	0.000	0.991093043	0.997059448
RN7SL273P	0.000	0.990966684	0.996953386
AC090578	0.000	0.990946207	0.996953386
NAT6	0.000	0.990941122	0.996953386
CABP1	0.000	0.990838299	0.996929518
DDX18P6	0.000	0.990795055	0.996907068
U51244	0.000	0.990774706	0.996907068
MZF1-AS1	0.000	0.99073504	0.996888804
RNA5SP427	0.000	0.990538147	0.996741146
RP11-74C3	0.000	0.990250925	0.996591176
HUNK-AS1	0.000	0.990231216	0.996591176
FTLP5	0.000	0.990203198	0.996591176
MMAB	0.000	0.990118975	0.996578742
RP11-665J16	0.000	0.990057666	0.996544085
PROX1	0.000	0.990043165	0.996544085
KRTAP9-11P	0.000	0.990025528	0.996544085
MIR4703	0.000	0.990012545	0.996544085
SALL4P1	0.000	0.990011646	0.996544085
PRAMEF2	0.000	0.989998404	0.996544085

RP11-557F20	0.000	0.989908843	0.996526037
MAPRE3	0.000	0.989872812	0.996526037
MORC2	0.000	0.989822272	0.996517753
WIPI2	0.000	0.989793517	0.996509868
AOC1	0.000	0.989720318	0.996468615
ME1	0.000	0.989583517	0.996403758
RP11-88H10	0.000	0.989568269	0.996403758
CTD-2514K5	0.000	0.989444475	0.99636908
NOS3	0.000	0.989433206	0.99636908
RP11-8L18	0.000	0.989334546	0.996300513
RP11-713P14	0.000	0.989333006	0.996300513
OR6C5P	0.000	0.989196107	0.996267392
RP11-281H11	0.000	0.989164722	0.996267392
RP11-529H20	0.000	0.989143042	0.996267392
TSC2	0.000	0.989121694	0.996267392
AC011225	0.000	0.989052984	0.996260347
SDHAP3	0.000	0.988994911	0.996260347
RPL31P61	0.000	0.988981854	0.996260347
RP11-45A17	0.000	0.988979662	0.996260347
RP11-22D3	0.000	0.988932874	0.996260347
DEFB104B	0.000	0.988900165	0.996260347
SCGB2A2	0.000	0.988854145	0.996260347
C19orf57	0.000	0.988788835	0.996260347
FRMD1	0.000	0.98878106	0.996260347
CFL2	0.000	0.988766616	0.996260347
RP11-416N4	0.000	0.988683581	0.996260347
CCL22	0.000	0.988673381	0.996260347
RP11-117L5	0.000	0.988665085	0.996260347
FAM107A	0.000	0.988648983	0.996260347
RNA5SP225	0.000	0.988599076	0.996260347
AMY2A	0.000	0.988558604	0.996260347
C16orf93	0.000	0.98843158	0.996260347
PSRC1	0.000	0.988398033	0.996260347
MAGEA13P	0.000	0.988382202	0.996260347
CTD-2057D4	0.000	0.988341717	0.996260347
LINC00583	0.000	0.988309778	0.996260347
RNA5SP390	0.000	0.988297714	0.996260347
TGM2	-0.001	0.988281708	0.996260347
CTC-273B12	-0.001	0.98821472	0.996260347
KRTAP4-8	-0.001	0.988208945	0.996260347
TMCC3	-0.001	0.988150571	0.996260347
CRADD	-0.001	0.987997214	0.996217625
WISP3	-0.001	0.987981964	0.996217625
AC008175	-0.001	0.987862207	0.996165855
SLC37A4	-0.001	0.987538018	0.996028965
AL162419	-0.001	0.987492176	0.996028965
RP11-317M11	-0.001	0.987480573	0.996028965
OVCH1-AS1	-0.001	0.987441668	0.996028965
RNU7-185P	-0.001	0.987264838	0.995966948
OSTCP5	-0.001	0.987249662	0.995966948
SNORD115-32	-0.001	0.987192308	0.995966948
RP11-44N12	-0.001	0.987084672	0.995963012

FARS2	-0.001	0.987008249	0.995937328
CTC-347C20	-0.001	0.986946849	0.995896472
WBP2P1	-0.001	0.986908465	0.995888404
PPIEL	-0.001	0.986884733	0.995888404
FKSG66	-0.001	0.986854918	0.995888404
FAM73B	-0.001	0.986791289	0.995888404
RP11-23B5	-0.001	0.986757436	0.995888404
RP11-358M11	-0.001	0.986698968	0.995888404
FAM32A	-0.001	0.986628788	0.995888404
TCERG1L	-0.001	0.986592234	0.995888404
TPRKB	-0.001	0.986544705	0.995870471
RP11-345M22	-0.001	0.98634089	0.995706937
LOC440461	-0.001	0.986294975	0.995701543
MIR378A	-0.001	0.986013545	0.99552419
AC091895	-0.001	0.985986316	0.995517802
RP11-367G12	-0.001	0.985974452	0.995517802
AC005027	-0.001	0.985875793	0.995469522
AC105252	-0.001	0.985866148	0.995469522
ZMYND10-AS1	-0.001	0.985802024	0.995437243
CTD-2544L4	-0.001	0.985704425	0.995402003
PNPLA1	-0.001	0.985631745	0.995370818
RP11-925D8	-0.001	0.985623318	0.995370818
MDGA2	-0.001	0.985497806	0.995369692
GOLGA2P8	-0.001	0.985475897	0.995369692
RNU6-801P	-0.001	0.985442294	0.995369446
PITRM1-AS1	-0.001	0.985395887	0.995343681
RPS26P15	-0.001	0.985370433	0.995343681
NEBL-AS1	-0.001	0.985287846	0.995343681
CHRM3-AS1	-0.001	0.985134783	0.995273589
DACT2	-0.001	0.985102274	0.995273589
RESP18	-0.001	0.985058155	0.995273589
RP11-138I1	-0.001	0.985043011	0.995273589
ATP4A	-0.001	0.985010982	0.995273589
GS1-531I17	-0.001	0.985008227	0.995273589
SNORA71	-0.001	0.984944463	0.995273589
CECR7	-0.001	0.984922776	0.995273589
RP1-258N20	-0.001	0.984903332	0.995273589
RPL21P12	-0.001	0.984889033	0.995273589
RP11-430H10	-0.001	0.98487918	0.995273589
RMST_3	-0.001	0.984870326	0.995273589
SNORD115-3	-0.001	0.984814758	0.995273589
RPL3P6	-0.001	0.984587395	0.995273589
RNU4ATAC4P	-0.001	0.984545612	0.995273589
NLRP5	-0.001	0.984516919	0.995273589
RIOK3	-0.001	0.984487571	0.995273589
MIR23C	-0.001	0.984442913	0.995273589
RP11-470F18	-0.001	0.984420247	0.995273589
AC073973	-0.001	0.984412799	0.995273589
GYG1P1	-0.001	0.98433347	0.995273589
RP11-759A24	-0.001	0.984274488	0.995273589
RP11-63M22	-0.001	0.984256971	0.995273589
TSPAN3	-0.001	0.984097676	0.995214354

RP11-174I12	-0.001	0.983869013	0.995046493
TUBB4BP1	-0.001	0.983814148	0.995033265
OR4K14	-0.001	0.983782091	0.995021973
OR1S2	-0.001	0.983762262	0.995021973
MAFA-AS1	-0.001	0.983739803	0.995021973
BMP15	-0.001	0.983723064	0.995021973
RP11-492A10	-0.001	0.983637072	0.995021973
RP11-281P23	-0.001	0.983616071	0.995021973
HMP19	-0.001	0.983584446	0.995021973
DUX4L9	-0.001	0.983478751	0.994990728
RAB18	-0.001	0.983388034	0.994967213
OR5B10P	-0.001	0.983380777	0.994967213
NANOGP7	-0.001	0.983372959	0.994967213
AF196779	-0.001	0.983274199	0.994967213
RPS4XP17	-0.001	0.98314053	0.994922424
RP11-59C5	-0.001	0.9831345	0.994922424
NIPSNAP3B	-0.001	0.98304389	0.994869108
NUP210P2	-0.001	0.983041626	0.994869108
OR5G4P	-0.001	0.983023027	0.994869108
RN7SL391P	-0.001	0.983000858	0.994869108
MIR10B	-0.001	0.982969166	0.994869108
CCDC157	-0.001	0.9828997	0.994869108
NCOR1P1	-0.001	0.982860761	0.994869108
POU3F2	-0.001	0.982734267	0.994764934
RNU6-696P	-0.001	0.982695464	0.994746798
STAU2	-0.001	0.98265009	0.994722009
CPSF3	-0.001	0.982606092	0.99471978
CTAGE16P	-0.001	0.982600198	0.99471978
AC092786	-0.001	0.982565705	0.99471978
RP11-753D20	-0.001	0.982544423	0.99471978
AC092316	-0.001	0.982505755	0.99471978
CTD-2526M8	-0.001	0.982484705	0.99471978
CCDC144B	-0.001	0.982403911	0.99471978
ELAC2	-0.001	0.982149568	0.994638148
AC135584	-0.001	0.982124974	0.994638109
AL121875	-0.001	0.982124974	0.994638109
AC073255	-0.001	0.98208049	0.994638109
CDCA7L	-0.001	0.981959642	0.994593846
RPL19P13	-0.001	0.981900039	0.99457578
RP11-981G7	-0.001	0.98186555	0.994561997
RPL41P2	-0.001	0.98186163	0.994561997
MIR1224	-0.001	0.981815242	0.994561997
CTD-3046C4	-0.001	0.981814543	0.994561997
NACA3P	-0.001	0.981689768	0.99451085
RGS19	-0.001	0.981678674	0.99451085
SPATA45	-0.001	0.981591967	0.994475231
ZNF695	-0.001	0.98157696	0.994475231
CTC-774J1	-0.001	0.981564871	0.994475231
EEF2K	-0.001	0.981481781	0.994437652
RNU5F-6P	-0.001	0.981377715	0.994427395
NMNAT1P3	-0.001	0.981322394	0.994392495
AJ006995	-0.001	0.98126696	0.994357479

MAST1	-0.001	0.981189057	0.994320848
RP11-399F2	-0.001	0.981160803	0.994320848
ADAM21P1	-0.001	0.98109762	0.994291655
RNU1-63P	-0.001	0.981008514	0.994251274
RP11-690G19	-0.001	0.980992733	0.994251274
RNU6-273P	-0.001	0.980971518	0.994251274
AP001198	-0.001	0.980769242	0.994170409
FLJ12825	-0.001	0.9807613	0.994170409
RNF11B	-0.001	0.980751605	0.994170409
RP1-4G17	-0.001	0.980608042	0.994112798
RNA5SP160	-0.001	0.980558601	0.994112798
FASN	-0.001	0.980483392	0.994071069
RP11-583F24	-0.001	0.980432592	0.994040726
ZNF692	-0.001	0.980316561	0.993965403
RP11-530N7	-0.001	0.980232533	0.993901764
RNF133	-0.001	0.980115437	0.99386727
METTL1	-0.001	0.980013393	0.993784953
PPP4R1L	-0.001	0.979995534	0.993784953
RNA5SP81	-0.001	0.979735389	0.993608819
FOXO4	-0.001	0.979632695	0.993525827
LLNLF-65H9	-0.001	0.979618406	0.993525827
SULT1A2	-0.001	0.979508555	0.993442236
RPL31P2	-0.001	0.979350691	0.993384211
SALRNA1	-0.001	0.979323035	0.993384211
AC005668	-0.001	0.979301806	0.993384211
HSP90B3P	-0.001	0.979280158	0.993384211
AC007163	-0.001	0.979265465	0.993384211
AMER1	-0.001	0.979246774	0.993384211
AC024996	-0.001	0.979226777	0.993384211
NOMO2	-0.001	0.979226302	0.993384211
RNU6-231P	-0.001	0.979149689	0.993384211
C17orf105	-0.001	0.978957178	0.99332723
RPS26P54	-0.001	0.978934612	0.993325495
YIPF3	-0.001	0.978897446	0.993313272
DIP2B	-0.001	0.978876974	0.993313272
GUCY2F	-0.001	0.978821045	0.993313272
AC136896	-0.001	0.978801109	0.993313272
SLC41A1	-0.001	0.978761984	0.993313272
RP11-592P9	-0.001	0.978731194	0.99330955
AC106753	-0.001	0.978724267	0.99330955
AC005042	-0.001	0.978501952	0.993182714
KLHL2	-0.001	0.978443151	0.993175973
TMEM99	-0.001	0.978352554	0.993140935
MIR8054	-0.001	0.978306208	0.993140935
OR7E104P	-0.001	0.978201659	0.993075681
RNU6-1214P	-0.001	0.97811631	0.993024077
RP11-640I15	-0.001	0.978047817	0.992996875
RP11-342F21	-0.001	0.97804201	0.992996875
RNA5SP485	-0.001	0.977831143	0.992840383
RP11-631M6	-0.001	0.97776142	0.992790755
RP11-403H13	-0.001	0.977713288	0.992790755
RPL21P33	-0.001	0.977656864	0.992790755

RPL30P5	-0.001	0.977623243	0.992790755
MAPK11	-0.001	0.977617337	0.992790755
FAM220A	-0.001	0.977608191	0.992790755
OR5AK4P	-0.001	0.97754803	0.992790755
RP11-139F4	-0.001	0.977518088	0.992790755
EXO5	-0.001	0.977502788	0.992790755
AC099799	-0.001	0.977411382	0.992790755
SCRT2	-0.001	0.977334333	0.992790755
CTD-2368P22	-0.001	0.977322471	0.992790755
RP5-1106E3	-0.001	0.977264557	0.992790755
RP11-105N13	-0.001	0.977248261	0.992790755
AC068718	-0.001	0.977149263	0.992790755
ATP5F1	-0.001	0.977119944	0.992790755
RP11-478L17	-0.001	0.977096214	0.992790755
AFAP1L2	-0.001	0.977059403	0.992790755
RP13-346H10	-0.001	0.977023279	0.992782055
RPL36AP41	-0.001	0.976975135	0.992775497
RP11-986E7	-0.001	0.976905763	0.992747366
RP11-689E3	-0.001	0.976791742	0.992673856
ASMTL-AS1	-0.001	0.976770206	0.992673152
AC004159	-0.001	0.976752885	0.992673152
TUBBP10	-0.001	0.97668018	0.992645205
PCDHB1	-0.001	0.97647282	0.992476813
MIR4684	-0.001	0.97644792	0.992476813
ESD	-0.001	0.976410019	0.992476813
LGI1	-0.001	0.976266764	0.99239445
MIR519E	-0.001	0.976060495	0.992228653
RP4-747G18	-0.001	0.976040167	0.992228653
RP11-770J1	-0.001	0.976017609	0.992228653
AC009312	-0.001	0.975984413	0.992228653
MIR1298	-0.001	0.975836397	0.992147555
RHPN2	-0.001	0.975833383	0.992147555
SRRT	-0.001	0.97573931	0.992091203
RP11-49O14	-0.001	0.975485992	0.992003061
MRPL27	-0.001	0.975464499	0.992002385
CLLU1	-0.001	0.975438959	0.991998715
HMGN1P24	-0.001	0.975435556	0.991998715
HIST1H3H	-0.001	0.975326877	0.991968334
SPIN2B	-0.001	0.9752916	0.991953639
ANKRD30B	-0.001	0.975268877	0.991953639
BPIFC	-0.001	0.975259564	0.991953639
TUNAR	-0.001	0.975219659	0.991953639
TGIF2LY	-0.001	0.975075772	0.991882397
MIR4733	-0.001	0.975041995	0.991869222
RP11-440D17	-0.001	0.974974549	0.991842981
AC011196	-0.001	0.974938529	0.991827523
CYP1A2	-0.001	0.974868298	0.991798445
LCN1P1	-0.001	0.974807283	0.991771024
RLF	-0.001	0.97474678	0.991759558
RP11-545P7	-0.001	0.97473314	0.991759558
NKX2-6	-0.001	0.974639908	0.991756754
CYP4F44P	-0.001	0.974184746	0.991441875

RP11-334G22	-0.001	0.974142463	0.991423261
TOR1B	-0.001	0.974123147	0.991423261
SNCA-AS1	-0.001	0.973976835	0.991336198
IL6	-0.001	0.973883628	0.991283698
CR848007	-0.001	0.973789984	0.991251936
RP11-378G13	-0.001	0.973739902	0.991251936
MLXIPL	-0.001	0.97371553	0.991251936
LINC00920	-0.001	0.973686788	0.991251936
RPL34P18	-0.001	0.973680261	0.991251936
PSKH2	-0.001	0.973561658	0.991251936
RNU6-1001P	-0.001	0.973506947	0.991218047
OR52B4	-0.001	0.973453263	0.991184576
HIGD1AP13	-0.001	0.97341305	0.991164821
RCOR2	-0.001	0.973383693	0.991156119
HSF4	-0.001	0.97330313	0.991095274
RP11-107F6	-0.001	0.97328997	0.991095274
RP11-430L16	-0.001	0.973263472	0.991095274
RNA5SP304	-0.001	0.973191224	0.991044889
OR4D2	-0.001	0.973086854	0.991026643
OR4P4	-0.001	0.972979495	0.990956406
RP11-318G21	-0.001	0.972971958	0.990956406
RN7SL173P	-0.001	0.972806258	0.990949495
MT-TY	-0.001	0.972630755	0.99089788
AC022872	-0.001	0.972544061	0.990830752
RNU2-16P	-0.001	0.972522065	0.990829537
RNU6-457P	-0.001	0.972460851	0.990829537
UBL5P1	-0.001	0.97238562	0.990796499
RP11-118H15	-0.001	0.972294899	0.990746451
EEF1B2P8	-0.001	0.972071611	0.990603702
CTC-391G2	-0.001	0.971969761	0.990521104
RNU6-718P	-0.001	0.97184572	0.99047947
ABALON	-0.001	0.971823859	0.990478386
RP11-165H23	-0.001	0.971796953	0.990478386
RN7SL37P	-0.001	0.97167936	0.990458285
RP11-525E9	-0.001	0.971558602	0.990398783
FARSA	-0.001	0.971522966	0.990383654
RN7SL794P	-0.001	0.971311059	0.990273613
TOLLIP-AS1	-0.001	0.971301762	0.990273613
RNU2-53P	-0.001	0.971282197	0.990273613
AL159997	-0.001	0.971281437	0.990273613
SDK1	-0.001	0.971026566	0.990089536
MIR154	-0.001	0.970990509	0.990073968
AC011752	-0.001	0.970918385	0.990021622
TMEM95	-0.001	0.970888266	0.990021622
RP11-434H14	-0.001	0.970871866	0.990021622
ELMO2P1	-0.001	0.970855178	0.990021622
PRM3	-0.001	0.970658346	0.989989615
RN7SL578P	-0.001	0.970641686	0.989989615
AP000640	-0.001	0.970552802	0.989924367
AF131216	-0.001	0.970506664	0.989919707
CYP4F3	-0.001	0.970461705	0.989919707
E2F6P4	-0.001	0.970362466	0.989857422

OR7E59P	-0.001	0.970303065	0.989831055
DUSP5P2	-0.001	0.970286427	0.989831055
PAWRP1	-0.001	0.970243419	0.989831055
RNA5-8SP6	-0.001	0.9701894	0.989829282
CTD-2192J16	-0.001	0.97010869	0.989789344
CETP	-0.001	0.970002884	0.989723797
AL138885	-0.001	0.969960344	0.989723797
RP4-814D15	-0.001	0.969810584	0.989685623
AP001032	-0.001	0.969750324	0.989678124
DCAF13P3	-0.001	0.969749196	0.989678124
SIAH1	-0.001	0.969701581	0.989678124
RP11-75A9	-0.001	0.969659774	0.989678124
DRAM2	-0.001	0.969654557	0.989678124
AC140479	-0.001	0.969608494	0.989678124
LINC01205	-0.001	0.96947814	0.98963361
ANKK1	-0.001	0.969434677	0.989631666
MKRN2OS	-0.001	0.969280546	0.989609147
SUPT3H	-0.001	0.969242231	0.989609147
RP11-747D18	-0.001	0.969169522	0.989609147
RNA5SP298	-0.001	0.969149676	0.989609147
RPL36AP10	-0.001	0.969006831	0.989609147
RP11-90K18	-0.001	0.968718592	0.9894862
PGBD3	-0.001	0.968523342	0.989362866
OR52Z1	-0.001	0.968514096	0.989362866
AP001052	-0.001	0.968485477	0.989362866
CTD-2529P6	-0.001	0.968454355	0.989362866
CSNK2A3	-0.001	0.968396189	0.989356156
RNU1-15P	-0.001	0.968275693	0.989317939
ZNF519P4	-0.001	0.968108776	0.989227608
TRIB3	-0.001	0.968011834	0.989196894
OR8I2	-0.001	0.967957375	0.989189602
PIH1D1	-0.001	0.967947399	0.989189602
ENPP3	-0.001	0.967732328	0.98910985
NLGN1-AS1	-0.001	0.967650749	0.989082567
RP11-5L12	-0.001	0.967492811	0.989027262
RP11-10L12	-0.001	0.967321783	0.988937332
RP11-162O12	-0.001	0.967255213	0.98891173
SCARNA10	-0.001	0.96724959	0.98891173
SLIT1-AS1	-0.001	0.967208463	0.98890639
LINC00968	-0.001	0.967113044	0.988830058
NANOGP2	-0.001	0.96707552	0.988812919
RPL37P15	-0.001	0.967069431	0.988812919
E2F4	-0.001	0.966872936	0.988754371
HPRTP4	-0.001	0.966860745	0.988754371
RP11-654D12	-0.001	0.966806596	0.988728989
MIR1276	-0.001	0.96676791	0.988725423
MTND5P11	-0.001	0.966697928	0.988725423
FCF1P9	-0.001	0.966511358	0.988596878
RNU6-53P	-0.001	0.966507365	0.988596878
AMD1	-0.001	0.966500284	0.988596878
TBC1D3J	-0.001	0.966285266	0.988596878
KRT18P6	-0.001	0.966283683	0.988596878

RP11-607P23	-0.001	0.966163952	0.988596878
TRIM46	-0.001	0.966163771	0.988596878
TBC1D3F	-0.001	0.965937965	0.988596878
RP11-241N4	-0.001	0.965863936	0.988596878
RPS15AP3	-0.001	0.965786429	0.988577237
RP1-28O17	-0.001	0.96578603	0.988577237
CTC-463A16	-0.001	0.965771543	0.988577237
RN7SL376P	-0.001	0.965704951	0.988577237
PPP1R14BP5	-0.001	0.965635032	0.988577237
MSL3	-0.001	0.96562397	0.988577237
GAPDHP48	-0.001	0.965608482	0.988577237
RNU6-259P	-0.001	0.96554134	0.988577237
NUDT19	-0.001	0.965378888	0.988457493
RN7SL622P	-0.001	0.965307286	0.988405429
RP11-669I1	-0.001	0.96515333	0.988290283
APBA3	-0.001	0.965076349	0.988253951
CYP2C58P	-0.001	0.965020056	0.988238802
RP11-411B10	-0.001	0.964965123	0.988225045
ZNF273	-0.001	0.964942711	0.988225045
ADPRH	-0.002	0.96481778	0.988159142
CADPS2	-0.002	0.964814298	0.988159142
OR4C7P	-0.002	0.964796534	0.988159142
CTB-1048E9	-0.002	0.964755377	0.988159142
AC011523	-0.002	0.964688164	0.988132636
HMGA1P1	-0.002	0.96467852	0.988132636
RNASE11	-0.002	0.964633077	0.988132636
SPATA7	-0.002	0.964430369	0.988038572
RNA5SP177	-0.002	0.964205013	0.987862903
IFT172	-0.002	0.964105647	0.987790893
CTD-2568A17	-0.002	0.963986293	0.98776015
ALG1L7P	-0.002	0.963703566	0.987612638
RP11-310I9	-0.002	0.963650641	0.98757965
LINC00994	-0.002	0.963534593	0.987503219
AL049868	-0.002	0.963521418	0.987503219
RP5-965G21	-0.002	0.963457091	0.987466286
SBF2	-0.002	0.963454479	0.987466286
SIGLEC27P	-0.002	0.963320553	0.987368842
SOD1P2	-0.002	0.963287597	0.987356312
AL137164	-0.002	0.963138967	0.987345382
IKBKGP1	-0.002	0.963133963	0.987345382
RP11-438J1	-0.002	0.963089753	0.987345382
CFL1P2	-0.002	0.963070626	0.987345382
AC083760	-0.002	0.962984313	0.987345382
KRT18P34	-0.002	0.962975256	0.987345382
AC114783	-0.002	0.962669318	0.987144365
TCEB2P2	-0.002	0.962591691	0.98711038
RP11-692E14	-0.002	0.962487859	0.987025155
PRKCZ	-0.002	0.962391161	0.987025155
RP5-1165K10	-0.002	0.962309762	0.987007532
MAGEA7P	-0.002	0.962297816	0.987007532
HOXA10-HOXA9	-0.002	0.962190376	0.986953847
CTD-2158P22	-0.002	0.962121548	0.986925758

OR7E4P	-0.002	0.96206295	0.986886905
AC079054	-0.002	0.961809072	0.986668979
ABCC13	-0.002	0.961799878	0.986668979
RNU4-20P	-0.002	0.961747663	0.986648485
VIPR2	-0.002	0.96148531	0.986549343
IGFBP7-AS1	-0.002	0.961419048	0.986527234
OR5L2	-0.002	0.961162483	0.986430615
LINC01255	-0.002	0.961159441	0.986430615
RP11-629N8	-0.002	0.96104495	0.986430615
EEF1A1P32	-0.002	0.961015823	0.986430615
RP11-318C4	-0.002	0.960835264	0.986303112
BAI3	-0.002	0.960750627	0.986287017
MIR217HG	-0.002	0.960707781	0.986287017
RP1-137F1	-0.002	0.96070562	0.986287017
GAS5	-0.002	0.960704265	0.986287017
RP11-847H18	-0.002	0.96054901	0.986268571
GPR123-AS1	-0.002	0.960498397	0.986237865
MRVI1-AS1	-0.002	0.960417903	0.986197737
TMED10	-0.002	0.960347411	0.986167877
SLC26A8	-0.002	0.960305194	0.986160249
RNA5SP350	-0.002	0.960151652	0.986051896
RP5-1193P9	-0.002	0.959922889	0.985944521
RP11-488I4	-0.002	0.959905183	0.985944521
ZSCAN4	-0.002	0.959836532	0.985919609
AC104654	-0.002	0.959815273	0.985919036
UBE2D3P3	-0.002	0.95971972	0.985863409
RNA5SP416	-0.002	0.959704649	0.985863409
AC144573	-0.002	0.959632227	0.985850437
RNU6-1035P	-0.002	0.959560586	0.985806251
TTC7B	-0.002	0.959448347	0.98575133
RP11-188P20	-0.002	0.959448343	0.98575133
MIR4764	-0.002	0.959348923	0.985695111
RP1-73A14	-0.002	0.959169795	0.985638643
LSM3P3	-0.002	0.95907962	0.985609775
LINC01029	-0.002	0.95900211	0.985593921
DLK2	-0.002	0.958859588	0.985499516
MTG1	-0.002	0.958772995	0.985443511
TRBV22-1	-0.002	0.958686851	0.985376236
CCL28	-0.002	0.958669432	0.985376236
RP11-1012E15	-0.002	0.958643528	0.985376236
RP11-625B21	-0.002	0.958628517	0.985376236
MED9	-0.002	0.958289126	0.985222584
IGKV2-38	-0.002	0.958223904	0.985195362
RP11-862P13	-0.002	0.958177558	0.985195362
C7orf55	-0.002	0.95810855	0.985195362
PAXBP1P1	-0.002	0.958101113	0.985195362
RP11-764E7	-0.002	0.957892568	0.985133866
RCC2P1	-0.002	0.957799384	0.985059303
AF246928	-0.002	0.957773447	0.985053899
RNF138P1	-0.002	0.957610918	0.985035641
RAB29	-0.002	0.957556391	0.985000826
RPL31P60	-0.002	0.957438294	0.984943163

SNX5	-0.002	0.957417158	0.984943163
RP5-1056L3	-0.002	0.957412372	0.984943163
RP11-493G17	-0.002	0.957373064	0.984943163
AL158035	-0.002	0.957351859	0.984943163
RP13-157F18	-0.002	0.957274324	0.984943163
RP11-490O24	-0.002	0.957183726	0.984943163
RP11-210L7	-0.002	0.957147786	0.984943163
RP11-101E19	-0.002	0.957111229	0.984943163
RP11-254A24	-0.002	0.957111035	0.984943163
MTRNR2L7	-0.002	0.957089214	0.984943163
AC064875	-0.002	0.957053249	0.984943163
AFAP1-AS1	-0.002	0.957035329	0.984943163
UBALD2	-0.002	0.957027313	0.984943163
PYGM	-0.002	0.956989025	0.984943163
CTD-2545H1	-0.002	0.956927557	0.984943163
KRT8P37	-0.002	0.956877961	0.984943163
FNBP1L	-0.002	0.956700999	0.984943163
AC006335	-0.002	0.956696589	0.984943163
MIR3648-1	-0.002	0.956631002	0.984943163
OR2AH1P	-0.002	0.956581595	0.984943163
RP11-138C24	-0.002	0.956482736	0.984938774
KARS	-0.002	0.95644996	0.984938774
RPL26P9	-0.002	0.956345803	0.984894774
EEF1A1P4	-0.002	0.956317696	0.984894774
RP1-290I10	-0.002	0.956285263	0.984894774
AC211476	-0.002	0.956186898	0.98486837
RN7SL868P	-0.002	0.956134714	0.98484156
RPS4XP8	-0.002	0.956130779	0.98484156
RNA5SP435	-0.002	0.956117609	0.98484156
GUCY2C	-0.002	0.956081893	0.98484156
AATBC	-0.002	0.955979219	0.984782244
RNA5SP449	-0.002	0.95591143	0.984733711
RNU6-713P	-0.002	0.955900529	0.984733711
SPTY2D1-AS1	-0.002	0.955803021	0.984685929
AC009478	-0.002	0.955720027	0.984621725
RPL35AP21	-0.002	0.955612307	0.984574641
CAD	-0.002	0.955583321	0.984566075
FLT1P1	-0.002	0.955535929	0.984549936
RP4-562J12	-0.002	0.955459392	0.984525263
EEF1DP8	-0.002	0.955370945	0.984496343
RNU6-539P	-0.002	0.955222878	0.984365061
RP11-367B6	-0.002	0.955138777	0.984306794
RP4-764D2	-0.002	0.955120567	0.984306794
ULK4P3	-0.002	0.955112628	0.984306794
LINC00664	-0.002	0.955091937	0.984306794
RNU6-325P	-0.002	0.955066379	0.984306794
SCARB2	-0.002	0.95506606	0.984306794
FAM26D	-0.002	0.954940551	0.984287091
RP11-909N17	-0.002	0.954841265	0.984206054
MIP	-0.002	0.954762909	0.984167887
PDIA2	-0.002	0.954684381	0.98410824
RP11-534C12	-0.002	0.954485739	0.983988665

LDHBP2	-0.002	0.954445756	0.983988665
RP11-375A5	-0.002	0.954409848	0.983974323
ENDOD1	-0.002	0.954340447	0.983924071
RN7SKP192	-0.002	0.954267478	0.983891438
MIR521-2	-0.002	0.95423082	0.983874941
AL033528	-0.002	0.95421207	0.983874941
CNTNAP4	-0.002	0.95409369	0.983777604
IGLCOR22-2	-0.002	0.954037753	0.983777604
USHBP1	-0.002	0.954013324	0.983777604
HOXD8	-0.002	0.953999839	0.983777604
RP11-465O11	-0.002	0.95396377	0.983777604
RALY-AS1	-0.002	0.953934155	0.983777604
SAA4	-0.002	0.95384812	0.983771233
ALDH3B1	-0.002	0.953844981	0.983771233
HNRNPAB	-0.002	0.953808877	0.983771233
MRTO4	-0.002	0.95367664	0.983729475
RP11-641A6	-0.002	0.953645667	0.983718832
MIR6499	-0.002	0.953630837	0.983718832
FAM205A	-0.002	0.953622432	0.983718832
SNRNP25	-0.002	0.953505691	0.983638354
MAGOH	-0.002	0.953472632	0.983625556
OR9A1P	-0.002	0.953456852	0.983625556
KRTAP4-7	-0.002	0.95345484	0.983625556
PI15	-0.002	0.953445663	0.983625556
B3GNT6	-0.002	0.953353046	0.983587408
RP1-125N5	-0.002	0.953345844	0.983587408
RP11-314C9	-0.002	0.953076718	0.983472751
RP3-500L14	-0.002	0.953065054	0.983472751
TSPEAR-AS1	-0.002	0.953044004	0.983472751
RHOXF1	-0.002	0.95291916	0.983434868
AC005358	-0.002	0.952913399	0.983434868
AC005480	-0.002	0.952762962	0.983404667
ANKRD18CP	-0.002	0.952710972	0.983372317
XRCC6P5	-0.002	0.952685814	0.983367662
KRTAP5-10	-0.002	0.95267091	0.983367662
AC000374	-0.002	0.952438231	0.983197339
RNA5SP118	-0.002	0.95222257	0.983059943
ANAPC13	-0.002	0.952129042	0.982984694
KCNQ5	-0.002	0.951894612	0.982827885
CLEC3B	-0.002	0.95187939	0.982827885
RP11-309M23	-0.002	0.951833303	0.982807196
RP11-226P1	-0.002	0.951805667	0.982799967
CTD-2560E9	-0.002	0.951756502	0.982770508
H3F3AP1	-0.002	0.951729437	0.982763868
SEPT4-AS1	-0.002	0.95167628	0.982750069
SNORD23	-0.002	0.951647081	0.982742748
HIST2H3A	-0.002	0.951622734	0.982738914
RP11-356I18	-0.002	0.951580421	0.98273108
ZNF343	-0.002	0.951537975	0.982715308
LSM11	-0.002	0.951522318	0.982715308
CYP4F11	-0.002	0.951514988	0.982715308
RNU4ATAC	-0.002	0.951474573	0.982713758

TCP1	-0.002	0.951427071	0.982707317
DNASE2	-0.002	0.951372272	0.982672028
RP11-200A13	-0.002	0.951333048	0.982653807
RPS7P3	-0.002	0.951309559	0.982653807
HSPE1P12	-0.002	0.951216314	0.982653807
MIR4419B	-0.002	0.951162266	0.982653807
RNA5SP144	-0.002	0.951109202	0.982653807
RNFT1P2	-0.002	0.951008556	0.982645776
AC092670	-0.002	0.950849025	0.982594459
CYP11B1	-0.002	0.950694241	0.982504385
MIR593	-0.002	0.950636976	0.98249462
RANGRF	-0.002	0.950600325	0.98249462
RNU6-827P	-0.002	0.950490025	0.982421242
AC007333	-0.002	0.950490025	0.982421242
RNU6-478P	-0.002	0.950490025	0.982421242
MIR548AE1	-0.002	0.950490025	0.982421242
RNU6-1325P	-0.002	0.950490025	0.982421242
WNT2B	-0.002	0.950481166	0.982421242
TUBB4AP1	-0.002	0.950400449	0.982421242
RP11-80K6	-0.002	0.950218432	0.982334577
MTA1	-0.002	0.950021068	0.982331185
OR4A41P	-0.002	0.949969485	0.982331185
TAB2	-0.002	0.949954969	0.982331185
TTLL4	-0.002	0.94990322	0.982331185
HHLA1	-0.002	0.949788896	0.982331185
CNKSR1	-0.002	0.949784503	0.982331185
RNU6-564P	-0.002	0.949769121	0.982331185
FOXE3	-0.002	0.949735155	0.982331185
TRAPPC2P7	-0.002	0.949704999	0.982331185
OR7A5	-0.002	0.949643638	0.982331185
AC109829	-0.002	0.949603771	0.982331185
EMC4	-0.002	0.949475322	0.982321494
CTD-2006K23	-0.002	0.949442989	0.982321494
RCC2P6	-0.002	0.949438499	0.982321494
EIF2D	-0.002	0.949364436	0.982302411
RP11-379B8	-0.002	0.94934418	0.982302411
RP5-1004I9	-0.002	0.949298551	0.982302411
RP11-164P12	-0.002	0.949274423	0.982302411
RP11-259K15	-0.002	0.949181531	0.982283881
MIR3201	-0.002	0.949177082	0.982283881
HIGD1AP6	-0.002	0.949090948	0.982254168
OR7E55P	-0.002	0.94901233	0.982236836
AC005077	-0.002	0.948989171	0.982234211
RP11-244N9	-0.002	0.948756843	0.982097366
GS1-388B5	-0.002	0.948663982	0.982060014
CYCSP28	-0.002	0.948523033	0.981965145
RP11-155O18	-0.002	0.948455195	0.981945172
VAV2	-0.002	0.948392596	0.981933898
RP11-139G7	-0.002	0.948190803	0.981873121
CTC-235G5	-0.002	0.948189229	0.981873121
ART2BP	-0.002	0.948118693	0.98184534
RP11-65E10	-0.002	0.947875431	0.981722196

PHKG1P4	-0.002	0.947826983	0.981722196
RPS8P3	-0.002	0.947750021	0.981698353
ATP5G1P7	-0.002	0.947675188	0.981654613
AP000282	-0.002	0.947653373	0.981654613
AC021192	-0.002	0.947614024	0.981654613
DPCR1	-0.002	0.947509297	0.981654613
PPFIA3	-0.002	0.947459528	0.981633195
PARP3	-0.002	0.947459093	0.981633195
RP11-536I6	-0.002	0.947402177	0.981633195
RPL27AP8	-0.002	0.947311823	0.981605924
HMGN1P10	-0.002	0.947241854	0.981593406
RAPGEF4-AS1	-0.002	0.947229632	0.981593406
RP11-298E10	-0.002	0.947182009	0.98157965
RNA5SP18	-0.002	0.947059375	0.981495275
FTX	-0.002	0.946953457	0.981428218
PPA1	-0.002	0.946926068	0.981421187
RN7SL813P	-0.002	0.946902846	0.981418477
CTD-2553C6	-0.002	0.946875395	0.981411383
AC006509	-0.002	0.946828673	0.981405672
KRTAP10-12	-0.002	0.94661691	0.981250241
CYCSP48	-0.002	0.94650138	0.98119455
MTND2P20	-0.002	0.946407117	0.981139543
RPL17P12	-0.002	0.946399796	0.981139543
ATP5HP2	-0.002	0.946354062	0.981127254
MIR4480	-0.002	0.946199153	0.981030718
RP11-449L13	-0.002	0.946172786	0.981024737
ECM1P2	-0.002	0.946112242	0.981004675
RP1-140A9	-0.002	0.946023324	0.980976548
TMEM151A	-0.002	0.946021579	0.980976548
RP4-778K6	-0.002	0.945959688	0.980976548
RP3-469D22	-0.002	0.945921017	0.980976548
AC098614	-0.002	0.945882694	0.980976548
XXbac-BPG300A18	-0.002	0.945859244	0.980976548
RP11-514F8	-0.002	0.945654783	0.980872016
RNU6-950P	-0.002	0.945619281	0.980856554
RP11-567N4	-0.002	0.945435877	0.980789436
RP5-827E24	-0.002	0.945377633	0.980776783
RNU6-993P	-0.002	0.945331262	0.980768944
LINC00888	-0.002	0.945267581	0.980759716
RNU4-63P	-0.002	0.945158297	0.980686675
MYOF	-0.002	0.945154126	0.980686675
FAM25C	-0.002	0.945125168	0.980686675
ARRDC1-AS1	-0.002	0.945076316	0.980686675
CTA-221G9	-0.002	0.944942867	0.980645389
TTC25	-0.002	0.944773776	0.980556138
RAB3B	-0.002	0.944663802	0.980463365
DEFB126	-0.002	0.944597111	0.980448189
RNA5SP269	-0.002	0.944541141	0.980421518
RP11-572N21	-0.002	0.944452585	0.980350964
RP4-678D15	-0.002	0.944439299	0.980350964
B3GNT2	-0.002	0.944429081	0.980350964
ATP6V1C2	-0.002	0.944414737	0.980350964

TNFRSF11A	-0.002	0.944363446	0.980350964
RP11-334A14	-0.002	0.944323603	0.980350964
FSIP2-AS1	-0.002	0.944304609	0.980350964
OR5B17	-0.002	0.944280683	0.980350964
SSFA2	-0.002	0.944249565	0.980350964
RAB9A	-0.002	0.944248696	0.980350964
AC135724	-0.002	0.94419544	0.980350964
POLR3F	-0.002	0.944132208	0.980350964
SNRPGP6	-0.002	0.944067825	0.980337044
PRR26	-0.002	0.943949416	0.980298703
GABRG3	-0.002	0.943933843	0.980298703
PDILT	-0.002	0.943919709	0.980298703
RN7SL344P	-0.002	0.943744285	0.980192539
SLC25A6P4	-0.002	0.943697802	0.980184636
RP1-20I3	-0.002	0.943624808	0.980153945
RP11-575I8	-0.002	0.943619868	0.980153945
MMRN2	-0.002	0.943567172	0.980136831
UQCRHL	-0.002	0.943536748	0.980126604
RP3-449H6	-0.002	0.943517401	0.980126604
RPL13AP23	-0.002	0.943474897	0.980105109
AC010754	-0.002	0.943472409	0.980105109
SAPCD2P4	-0.002	0.943407187	0.980077524
STARD6	-0.002	0.943313074	0.980003687
AL121578	-0.002	0.94328842	0.980003687
PINK1-AS	-0.002	0.943207737	0.979998579
RP11-704C2	-0.002	0.943159567	0.979991288
RP11-153K11	-0.002	0.94308051	0.979991227
RNU1-146P	-0.002	0.943069629	0.979991227
LBH	-0.002	0.942996949	0.979991227
RP11-1149M3	-0.002	0.942834205	0.979884211
FLJ20019	-0.002	0.942760799	0.979884211
AC078889	-0.002	0.942664538	0.979876631
RP11-640M9	-0.002	0.942621103	0.979876631
TMEM206	-0.002	0.942600156	0.979876631
MARK1	-0.002	0.942571908	0.979876631
RP11-453M23	-0.002	0.942486928	0.979869569
OR4C14P	-0.002	0.942479071	0.979869569
NKX2-3	-0.002	0.942436886	0.979869569
NPSR1-AS1	-0.002	0.942432939	0.979869569
RP11-362J17	-0.002	0.942396544	0.979869569
OR4N2	-0.002	0.942387337	0.979869569
GAPDHP72	-0.002	0.942216283	0.97978068
IGLVVI-25-1	-0.002	0.942202293	0.97978068
AP001605	-0.002	0.942169369	0.97978068
MIR4749	-0.002	0.942019244	0.97978068
KRTAP22-2	-0.002	0.942013367	0.97978068
TMEM108-AS1	-0.002	0.941955542	0.97978068
ACVR1C	-0.002	0.941912213	0.97978068
RP11-355N15	-0.002	0.941853319	0.979745507
RP5-1119A7	-0.002	0.941844317	0.979745507
AKR1B1P1	-0.002	0.941790014	0.979722452
NKX2-1-AS1	-0.002	0.941783131	0.979722452

RP11-259P6	-0.002	0.941774143	0.979722452
AC023115	-0.002	0.941771321	0.979722452
RP13-1016M1	-0.002	0.941737019	0.979722452
RGR	-0.002	0.941711001	0.979722452
ASAP1-IT2	-0.002	0.941603693	0.979722452
RP11-390K5	-0.002	0.94158499	0.979722452
GUCA2B	-0.002	0.941552455	0.979722452
AC009035	-0.002	0.941517897	0.979722452
TBX20	-0.003	0.941499802	0.979722452
TP73-AS1	-0.003	0.941432897	0.979722452
AL353782	-0.003	0.941410431	0.979722452
ZPLD1	-0.003	0.941314149	0.979698192
RPS15AP34	-0.003	0.941271104	0.97969621
AC024610	-0.003	0.941269918	0.97969621
RPL5P17	-0.003	0.941237414	0.97969621
MIR7975	-0.003	0.941126668	0.97969621
LINC01005	-0.003	0.941094193	0.97969621
ZNF806	-0.003	0.941042828	0.97969621
MGAT5	-0.003	0.940968374	0.97969621
RNU6-458P	-0.003	0.940923529	0.97969621
RAB6C-AS1	-0.003	0.940858291	0.979682527
MIR4307	-0.003	0.940839232	0.979682527
INSC	-0.003	0.940671406	0.979649058
RNU6-839P	-0.003	0.940523406	0.979517357
RP3-393E18	-0.003	0.940467682	0.97950216
RP11-248J23	-0.003	0.940439142	0.979493855
AC091878	-0.003	0.940432973	0.979493855
RP11-715C4	-0.003	0.940288812	0.979444377
ZBTB16	-0.003	0.940269791	0.979444377
RP11-849F2	-0.003	0.940260983	0.979444377
RP11-498D10	-0.003	0.939983979	0.979324246
APOO	-0.003	0.939964657	0.979324246
RP11-158N24	-0.003	0.939932105	0.979324246
RN7SL378P	-0.003	0.939894126	0.979324246
ATG4B	-0.003	0.939857339	0.979316214
RP11-52L5	-0.003	0.939834552	0.979316214
RN7SKP161	-0.003	0.939826494	0.979316214
TRIM7	-0.003	0.939815194	0.979316214
AC002530	-0.003	0.939780856	0.979316214
RP11-4L24	-0.003	0.939736293	0.979316214
SLC13A2	-0.003	0.939694022	0.979316214
GDI2P1	-0.003	0.93969264	0.979316214
AL133168	-0.003	0.939674934	0.979316214
AC018635	-0.003	0.939658067	0.979316214
RP11-1221G12	-0.003	0.939585756	0.979311758
MDH1B	-0.003	0.93952757	0.979293975
MIR6754	-0.003	0.939509447	0.979293975
MIR3137	-0.003	0.939353855	0.979220059
TUBB8P4	-0.003	0.939194473	0.97913313
RP11-297H3	-0.003	0.939183789	0.97913313
RP11-543B16	-0.003	0.939151974	0.97913313
AC073115	-0.003	0.939101112	0.979128035

RNU7-24P	-0.003	0.939049385	0.979095537
RNA5SP215	-0.003	0.938373969	0.978617918
FSTL5	-0.003	0.938328055	0.978617918
OR7E66P	-0.003	0.938322936	0.978617918
AC027088	-0.003	0.938190021	0.97856371
CTD-2372A4	-0.003	0.938073195	0.978533907
MIR3196	-0.003	0.938057608	0.978533907
AC017053	-0.003	0.937786287	0.978314003
AC104000	-0.003	0.937736519	0.978283512
GOLGA2P6	-0.003	0.937713565	0.978283512
CCNT2-AS1	-0.003	0.937699477	0.978283512
PPM1L	-0.003	0.937696002	0.978283512
AL353147	-0.003	0.93762367	0.978283512
KCNK6	-0.003	0.937542228	0.978230813
SUMO2P20	-0.003	0.937502223	0.978210502
ABC7-43041300I9	-0.003	0.937334367	0.978121073
RNU6-67P	-0.003	0.937257214	0.978061992
PARP1P1	-0.003	0.937224294	0.978055445
EIF3M	-0.003	0.937210796	0.978055445
RP11-177H2	-0.003	0.937165355	0.978055445
RP11-51M24	-0.003	0.937107707	0.978055445
RNU6-1044P	-0.003	0.937013423	0.978043312
AL132772	-0.003	0.93696148	0.978041347
GRID2	-0.003	0.936954459	0.978041347
RP1-193H18	-0.003	0.936938881	0.978041347
SERPINA10	-0.003	0.936905713	0.978041347
RP11-382E9	-0.003	0.936897825	0.978041347
RP11-307I14	-0.003	0.936874605	0.978041347
RP11-195M16	-0.003	0.936824964	0.978041347
CTA-481E9	-0.003	0.936774172	0.978041347
FAM218A	-0.003	0.936732561	0.978041347
FAM71A	-0.003	0.936730622	0.978041347
GMNN	-0.003	0.9367204	0.978041347
SLC35F4	-0.003	0.936675619	0.978041347
OR13I1P	-0.003	0.936670077	0.978041347
RP11-243M5	-0.003	0.936449715	0.977990759
RP1-96H9	-0.003	0.936245903	0.977842232
FGF10	-0.003	0.936207373	0.977823434
S100P	-0.003	0.936205706	0.977823434
NME8	-0.003	0.935896462	0.977563015
RP11-284G10	-0.003	0.935792378	0.977563015
RP11-748H22	-0.003	0.935717108	0.977547187
MIR646HG	-0.003	0.935531142	0.977395786
MIR3147	-0.003	0.935506729	0.97739172
RP11-156G14	-0.003	0.935259776	0.977262337
RP11-162N7	-0.003	0.935222983	0.977245332
LINC01574	-0.003	0.935158008	0.977206818
RNU6-504P	-0.003	0.935032838	0.977111866
AC010745	-0.003	0.934984377	0.977103193
OR5BN1P	-0.003	0.934806223	0.976981325
LARP1BP1	-0.003	0.934797175	0.976981325
RN7SL615P	-0.003	0.934781245	0.976981325

KDM8	-0.003	0.934777733	0.976981325
MIR520A	-0.003	0.934777619	0.976981325
ACAA2	-0.003	0.934736477	0.976981325
BNIP3P42	-0.003	0.934734616	0.976981325
AL139318	-0.003	0.934528543	0.976966054
AC010878	-0.003	0.934393954	0.976906849
KB-1205A7	-0.003	0.934386434	0.976906849
RPL17P17	-0.003	0.934359359	0.976906849
RP11-613C6	-0.003	0.934260233	0.976860858
HIST1H2AM	-0.003	0.934255647	0.976860858
NAV2	-0.003	0.934251416	0.976860858
NDUFA5P12	-0.003	0.934246642	0.976860858
AC007560	-0.003	0.93420674	0.976860858
RPL18AP7	-0.003	0.934107429	0.976829758
DCUN1D2-AS	-0.003	0.93404344	0.976829758
PWRN3	-0.003	0.933949987	0.976829758
MIR9-3	-0.003	0.933906344	0.976829758
OR11J2P	-0.003	0.933728356	0.976690688
PDSS2	-0.003	0.933712101	0.976690688
AC093822	-0.003	0.933649129	0.976672167
RP11-523J2	-0.003	0.933602487	0.976666279
ZNF48	-0.003	0.933501507	0.976603542
MIR6762	-0.003	0.933407336	0.976547925
AL162511	-0.003	0.933401664	0.976547925
ZPR1	-0.003	0.933400336	0.976547925
AP003176	-0.003	0.933396303	0.976547925
ORMDL2	-0.003	0.933257918	0.976498855
NDRG2	-0.003	0.933047335	0.976364314
RNU6-805P	-0.003	0.933025533	0.976363374
OR5M12P	-0.003	0.932980228	0.976358448
LONRF2P2	-0.003	0.932954798	0.976358448
CTC-320C6	-0.003	0.932875459	0.976358448
SIRT3	-0.003	0.932713449	0.976278599
TAF1D	-0.003	0.93271041	0.976278599
CEBPA	-0.003	0.932684794	0.976278599
DHX37	-0.003	0.932558885	0.976239274
EVPL	-0.003	0.932430807	0.976188417
RNU4ATAC7P	-0.003	0.932408059	0.976188417
RPL23AP54	-0.003	0.932232361	0.976069088
AARS2	-0.003	0.932178563	0.976055675
SEPHS1P1	-0.003	0.9321586	0.976055675
RP11-360P21	-0.003	0.932124604	0.97604721
MISP	-0.003	0.932105192	0.97604721
NF2	-0.003	0.931952809	0.97596949
BOK-AS1	-0.003	0.931942599	0.97596949
BCAT2	-0.003	0.931860338	0.97596949
RP11-100N21	-0.003	0.931855996	0.97596949
CASK-AS1	-0.003	0.931831758	0.97596949
EGFLAM-AS2	-0.003	0.931827044	0.97596949
RP11-43D2	-0.003	0.931727022	0.975969096
PSG7	-0.003	0.931705996	0.975968537
SPHK2	-0.003	0.931544294	0.975864411

TCP11L1	-0.003	0.931515642	0.975864411
RNU6-113P	-0.003	0.931099942	0.975634104
REXO1	-0.003	0.931084573	0.975634104
MIR31	-0.003	0.9310435	0.975634104
ALOX15	-0.003	0.931022445	0.975634104
PSMD12P	-0.003	0.930888897	0.975609419
LINC01101	-0.003	0.930857086	0.975609419
KB-1299A7	-0.003	0.93072681	0.975522134
RP11-309L24	-0.003	0.930570616	0.975422822
RP11-99A1	-0.003	0.930377264	0.97530601
IPO9-AS1	-0.003	0.930034545	0.975098471
C17orf47	-0.003	0.929832261	0.974992209
PRR34	-0.003	0.929754596	0.9749537
MIR4255	-0.003	0.929622803	0.974879893
RP11-90K6	-0.003	0.92951666	0.974790047
C1orf228	-0.003	0.929313628	0.97464138
SLC25A5-AS1	-0.003	0.929223354	0.974632678
MIR3663	-0.003	0.92919113	0.974620344
MIR23A	-0.003	0.929172243	0.974620344
U95743	-0.003	0.929160314	0.974620344
RASSF8	-0.003	0.929104002	0.974614812
KCNQ1DN	-0.003	0.929075367	0.974614812
RP11-328K22	-0.003	0.929053369	0.974614812
RP11-175I6	-0.003	0.929037245	0.974614812
SPNS3	-0.003	0.928963386	0.974614812
RPL7L1P3	-0.003	0.928901357	0.974614812
AP005271	-0.003	0.928800534	0.974555293
RP11-242F24	-0.003	0.928765632	0.974555293
LINC00462	-0.003	0.928662484	0.974538022
UBXN7-AS1	-0.003	0.928491707	0.974380278
RP4-669B10	-0.003	0.928330353	0.974296821
RP11-550F7	-0.003	0.928298945	0.974296821
AC018866	-0.003	0.928224005	0.974292555
PLEKHH2	-0.003	0.9281857	0.974281531
AL353707	-0.003	0.928168547	0.974281531
PPP2CA	-0.003	0.928166172	0.974281531
MTND4P21	-0.003	0.928051118	0.974239913
ACOT12	-0.003	0.927900086	0.974124306
GS1-259H13	-0.003	0.927743724	0.974024565
RP11-264M12	-0.003	0.927717438	0.974024565
RPS3AP52	-0.003	0.9277161	0.974024565
AC008938	-0.003	0.92767874	0.974024565
ELANE	-0.003	0.927662041	0.974024565
PXMP2	-0.003	0.927635458	0.974024565
AC079140	-0.003	0.927382079	0.973902502
RP11-63A2	-0.003	0.927381446	0.973902502
KRAS	-0.003	0.927375104	0.973902502
SLFNL1-AS1	-0.003	0.927243194	0.973842542
MTND2P6	-0.003	0.927221569	0.973841306
UNGP1	-0.003	0.92716746	0.97380595
RP11-468I15	-0.003	0.927043824	0.973719041
ZAR1	-0.003	0.926970764	0.973698901

RP11-134E15	-0.003	0.926935513	0.97369117
HPCA	-0.003	0.926901279	0.97369117
RP11-195B3	-0.003	0.926850781	0.97369117
RP11-986G18	-0.003	0.926822306	0.97369117
PTPN13	-0.003	0.926817537	0.97369117
RNU6-175P	-0.003	0.926795378	0.97369117
RPL4P4	-0.003	0.926781091	0.97369117
COX5BP8	-0.003	0.926759865	0.97369117
RSL24D1P8	-0.003	0.926720937	0.97369117
C1orf61	-0.003	0.926646341	0.97369117
SNHG11	-0.003	0.926381923	0.973534838
RP11-7O11	-0.003	0.92635025	0.973534838
CCDC109B	-0.003	0.926283869	0.973527903
RP11-391A7	-0.003	0.926245523	0.973527903
RNU6-1052P	-0.003	0.926143731	0.973510611
GAPDHP34	-0.003	0.926065954	0.973496473
AC008850	-0.003	0.925991279	0.973450759
RP11-42H13	-0.003	0.925948939	0.973449222
KRT8P21	-0.003	0.925929711	0.973449222
MCOLN3	-0.003	0.925923336	0.973449222
EFCAB8	-0.003	0.925702048	0.973275601
RP11-597M12	-0.003	0.925670165	0.973263565
ZNF286B	-0.003	0.925499914	0.973147809
MIR7515HG	-0.003	0.925486105	0.973147809
RP11-3M1	-0.003	0.925257495	0.973023002
OR52Y1P	-0.003	0.925253232	0.973023002
RP11-14O19	-0.003	0.925240365	0.973023002
CISD1P1	-0.003	0.925230014	0.973023002
RP11-510M2	-0.003	0.925081136	0.972968505
MIR1183	-0.003	0.925013101	0.972937854
ZNF736P4Y	-0.003	0.924998938	0.972937854
TTLL12	-0.003	0.924965027	0.972937854
BMPR1A	-0.003	0.92496199	0.972937854
STK11IP	-0.003	0.924960689	0.972937854
DUXAP1	-0.003	0.924789211	0.972874246
MS4A7	-0.003	0.924667014	0.972810159
MIR4504	-0.003	0.924553357	0.972776538
C11orf98P1	-0.003	0.92454594	0.972776538
AC013460	-0.003	0.92447502	0.972737093
ALOX12	-0.003	0.92424539	0.972581423
EEF1DP1	-0.003	0.924232811	0.972581423
NDUFAF4P4	-0.003	0.924201857	0.97257859
RP11-707O23	-0.003	0.924084339	0.972497897
ZNF565	-0.003	0.924053237	0.972491893
FOXK1	-0.003	0.923786052	0.972312891
ZFAND6	-0.003	0.92375092	0.9722974
AP000547	-0.003	0.923720417	0.972291575
RP11-173D14	-0.003	0.923640043	0.972245156
MIS18A	-0.003	0.923619066	0.972244563
RNU6-1090P	-0.003	0.923512644	0.972197
RP11-496D24	-0.003	0.923467213	0.972170662
AL138807	-0.003	0.92342138	0.972170662

RP11-316N24	-0.003	0.923372224	0.972170662
CTSO	-0.003	0.92325084	0.97213627
RP11-674N23	-0.003	0.923209588	0.972131393
RPL36AP43	-0.003	0.92317143	0.972131393
LRRC14	-0.003	0.92314892	0.972131393
DNAJC30	-0.003	0.923141981	0.972131393
HMGN2P30	-0.003	0.923091419	0.972131393
LINC00396	-0.003	0.923050286	0.972131393
CYP2A13	-0.003	0.922908263	0.972076426
RP11-46I1	-0.003	0.922760462	0.971942246
RP11-526I8	-0.003	0.922741449	0.971942246
C6orf1	-0.003	0.92263153	0.971884627
CTD-2383I20	-0.003	0.922614971	0.971884627
PRSS53	-0.003	0.922587729	0.971884627
ZNF470	-0.003	0.922580357	0.971884627
OR8K4P	-0.003	0.922562583	0.971884627
RP11-640N11	-0.003	0.922549207	0.971884627
ABCC12	-0.003	0.922489415	0.971884627
RPL7AP61	-0.003	0.922443352	0.971884627
LA16c-312E8	-0.003	0.922438327	0.971884627
RN7SKP184	-0.003	0.922227394	0.971884627
SMC1B	-0.003	0.922223018	0.971884627
LINC00349	-0.003	0.922182503	0.971884627
RP3-365O12	-0.003	0.92209191	0.971884627
CXorf49	-0.003	0.922073793	0.971884627
OR4A12P	-0.003	0.922023147	0.971884627
RP1-146A15	-0.003	0.921963604	0.971855176
AL390966	-0.003	0.921817412	0.971794049
AC003968	-0.003	0.921810945	0.971794049
AC064837	-0.003	0.92178008	0.971794049
FTLP8	-0.003	0.921712646	0.971784212
RP11-196E1	-0.003	0.921700852	0.971784212
AP000936	-0.003	0.921626725	0.971784212
MYCL	-0.003	0.921509258	0.971720352
ADCK2	-0.003	0.921501453	0.971720352
SLC2A6	-0.003	0.921213538	0.971520184
OR2T3	-0.003	0.921195351	0.971520184
SNRPGP1	-0.003	0.921187724	0.971520184
RN7SL329P	-0.003	0.921042919	0.97150823
TMEM14A	-0.003	0.921042269	0.97150823
NET1	-0.003	0.921011484	0.97150823
RP11-533F5	-0.003	0.920997395	0.97150823
SLC16A13	-0.003	0.920926068	0.97150823
ZNF256	-0.003	0.920918263	0.97150823
RP11-159D23	-0.003	0.92087284	0.971500958
RN7SKP15	-0.003	0.920870683	0.971500958
AC118562	-0.003	0.920853963	0.971500958
RP11-121A13	-0.003	0.920769683	0.971499727
RP3-418C23	-0.003	0.920712356	0.971460763
AL138810	-0.003	0.920431856	0.971242254
RP11-680H20	-0.003	0.920413589	0.971242254
RP11-61D1	-0.003	0.92020802	0.971100737

AC007050	-0.003	0.920101484	0.971009824
RP13-554M15	-0.003	0.920041174	0.970967692
CDHR3	-0.003	0.920036838	0.970967692
RPL6P29	-0.003	0.919984449	0.970967692
HOXD4	-0.003	0.919921216	0.970967692
RP11-6J24	-0.003	0.919898173	0.970967692
SUMO1P2	-0.003	0.919832779	0.970941396
MKI67P1	-0.003	0.919664531	0.970849867
RP11-481K9	-0.003	0.919609615	0.970813414
SESN1	-0.003	0.919598113	0.970813414
CNTNAP2	-0.003	0.919570367	0.970813414
CLYBL-AS2	-0.003	0.919484147	0.970788549
CACTIN-AS1	-0.003	0.919402816	0.97076724
RNU6-908P	-0.003	0.919352037	0.970735146
NMU	-0.003	0.919312347	0.970714757
MACC1-AS1	-0.003	0.919259601	0.970680583
RP11-379J13	-0.003	0.919243437	0.970680583
LINC00290	-0.003	0.919195566	0.970669732
SCNN1G	-0.003	0.919125814	0.970625391
ACTL10	-0.003	0.919118232	0.970625391
MANSC1	-0.003	0.918971716	0.970615114
IGF2BP3	-0.003	0.918932572	0.970615014
SCARB1	-0.003	0.918926201	0.970615014
FOCAD	-0.003	0.918873751	0.970615014
RP11-207F8	-0.003	0.918798688	0.970602753
RNU6-556P	-0.003	0.918787839	0.970602753
RP11-262D11	-0.003	0.918783372	0.970602753
CCDC152	-0.003	0.918759315	0.970602753
LA16c-2F2	-0.003	0.918746621	0.970602753
CBX3	-0.003	0.918730707	0.970602753
LINC01018	-0.003	0.918696673	0.970602753
RPS2P48	-0.003	0.918672189	0.970602753
AL357140	-0.003	0.918576225	0.970602753
RP11-28P17	-0.003	0.918462118	0.97057012
RP11-618K16	-0.003	0.918451619	0.97057012
RNU6-609P	-0.003	0.91835855	0.970503743
OR2T7	-0.003	0.91834636	0.970503743
RN7SKP87	-0.003	0.91833264	0.970503743
RN7SL526P	-0.003	0.918285149	0.970502536
EEF1B2P7	-0.003	0.918230836	0.970502536
LY6G6C	-0.003	0.918185792	0.970493447
ASPA	-0.004	0.918160287	0.970488027
RP11-363I22	-0.004	0.917984106	0.970323339
MIR548AJ2	-0.004	0.917953312	0.970312324
NCRUPAR_2	-0.004	0.91790036	0.970299422
ATP13A2	-0.004	0.917879866	0.970299294
HSPA13	-0.004	0.917797084	0.970254855
RNU6-511P	-0.004	0.917781907	0.970254855
LINCMD1	-0.004	0.917692402	0.970192943
HIST1H2BF	-0.004	0.917619002	0.970174267
SLC6A21P	-0.004	0.91761363	0.970174267
ANKRD20A4	-0.004	0.917528735	0.970144855

RGS21	-0.004	0.91743795	0.970133598
AC005597	-0.004	0.917349234	0.970104402
RNU6-1220P	-0.004	0.917311783	0.970104402
MRPS5	-0.004	0.917297379	0.970104402
DLEU2	-0.004	0.917292611	0.970104402
AC012175	-0.004	0.917284482	0.970104402
RP11-280O24	-0.004	0.917195678	0.970092795
RP11-376N17	-0.004	0.917153215	0.970069729
AC137675	-0.004	0.917100853	0.970057805
CTC-394G3	-0.004	0.917002583	0.970057805
AC092484	-0.004	0.916986858	0.970057805
FRMD6-AS1	-0.004	0.916941147	0.970057805
MIR4780	-0.004	0.9168952	0.970057805
OR6L2P	-0.004	0.916890084	0.970057805
TRIAP1	-0.004	0.91682154	0.970041753
LINC00376	-0.004	0.916666803	0.969964226
RNA5SP136	-0.004	0.916661337	0.969964226
RP11-76N22	-0.004	0.916655175	0.969964226
RNU6-959P	-0.004	0.91661531	0.969964226
PANX2	-0.004	0.916569765	0.969964226
RNU7-30P	-0.004	0.916550522	0.969964226
AC006925	-0.004	0.916512098	0.969951374
RP11-31E13	-0.004	0.916341376	0.969813798
RP1-90K10	-0.004	0.916325255	0.969813798
AC111155	-0.004	0.916300269	0.969813393
KB-1073A2	-0.004	0.916218415	0.96974831
MUPP	-0.004	0.916211843	0.96974831
RP5-945I17	-0.004	0.916032341	0.969702214
CLNS1AP1	-0.004	0.915905278	0.969632361
DBNL	-0.004	0.915838754	0.96960504
RP11-598C10	-0.004	0.915752513	0.969535288
RP11-806H10	-0.004	0.915737796	0.969535288
AC025263	-0.004	0.915698869	0.969521598
RN7SL517P	-0.004	0.915649251	0.969512169
NAGS	-0.004	0.915550616	0.969450837
AC083811	-0.004	0.915497758	0.96941642
RP11-256K9	-0.004	0.915458154	0.969396036
CNP	-0.004	0.91533831	0.969290682
RP11-669M2	-0.004	0.915314573	0.969287097
AC093390	-0.004	0.915230379	0.969219488
RP11-810H18	-0.004	0.914920666	0.969085435
RNU2-18P	-0.004	0.914907675	0.969085435
NOVA2	-0.004	0.914904187	0.969085435
TPSP2	-0.004	0.914867937	0.969085435
RP11-320P11	-0.004	0.914845514	0.969085435
SPARCL1	-0.004	0.914754285	0.969085435
AF067845	-0.004	0.914692284	0.969085435
AL360001	-0.004	0.91463528	0.96907908
RP11-1007J8	-0.004	0.914449933	0.968953167
AC011453	-0.004	0.914444596	0.968953167
OR4C3	-0.004	0.91443923	0.968953167
RP11-687D19	-0.004	0.914362539	0.968936589

RP11-116D2	-0.004	0.914324455	0.968936589
ITGA9	-0.004	0.914321062	0.968936589
RN7SKP204	-0.004	0.914249825	0.968935144
CYP51A1P1	-0.004	0.914239101	0.968935144
ZACN	-0.004	0.914159043	0.968903613
CLEC4G	-0.004	0.914103592	0.968866402
AC004744	-0.004	0.914018011	0.968801185
IQUB	-0.004	0.913990522	0.968801185
TMEM165	-0.004	0.913926418	0.968801185
RN7SL73P	-0.004	0.913924298	0.968801185
DNAJC25-GNG10	-0.004	0.913921868	0.968801185
CAMK1	-0.004	0.913909523	0.968801185
HMGA1P6	-0.004	0.913849648	0.968801185
ATP2A1	-0.004	0.913800794	0.968801185
KB-1683C8	-0.004	0.913796806	0.968801185
RNU1-36P	-0.004	0.913778357	0.968801185
CTC-338M12	-0.004	0.91376744	0.968801185
PSMA6P2	-0.004	0.913762343	0.968801185
WI2-8325B5	-0.004	0.913741817	0.968801185
RP11-354E23	-0.004	0.913613054	0.968756099
AC019064	-0.004	0.91358715	0.968756099
HSPA8P14	-0.004	0.913565973	0.968756099
AL117187	-0.004	0.913519643	0.968743322
RNU6-627P	-0.004	0.913474056	0.968716548
GTF2F2P2	-0.004	0.913295507	0.968570333
PIN4P1	-0.004	0.913093635	0.968399371
EPHA7	-0.004	0.913012599	0.96833499
AC013470	-0.004	0.913002171	0.96833499
TM4SF19-TCTEX1D2	-0.004	0.912857715	0.968235405
LINC00238	-0.004	0.912846673	0.968235405
AL033522	-0.004	0.912707097	0.968217864
CTD-2046I8	-0.004	0.912694486	0.968217864
SEC11B	-0.004	0.912631615	0.968217443
GS1-54N10	-0.004	0.912468917	0.968202375
MIR586	-0.004	0.912453632	0.968202375
TAAR5	-0.004	0.912406763	0.968188332
DNAJC19P3	-0.004	0.912317624	0.968115313
RP11-296A18	-0.004	0.912288287	0.968105751
OR7E136P	-0.004	0.912244778	0.968105751
RP11-114O13	-0.004	0.912224566	0.968105751
RPL13AP3	-0.004	0.912190625	0.968105751
RP3-503A6	-0.004	0.912178242	0.968105751
TMEM223	-0.004	0.912042729	0.967996146
AL445074	-0.004	0.912041043	0.967996146
TPT1P7	-0.004	0.912015446	0.967996146
KAT8	-0.004	0.911989894	0.967996146
IZUMO1	-0.004	0.911831864	0.967896056
STX18	-0.004	0.911771196	0.967858933
LA16c-306E5	-0.004	0.911695999	0.967856992
LINC00397	-0.004	0.911691495	0.967856992
RP11-789C2	-0.004	0.911661202	0.967856992
BOLA2P1	-0.004	0.911654402	0.967856992

NHP2	-0.004	0.911627855	0.967856992
GUSBP8	-0.004	0.911610848	0.967856992
SPINT3	-0.004	0.911591784	0.967856992
VPS25	-0.004	0.911575023	0.967856992
RP13-578N3	-0.004	0.911573791	0.967856992
COL6A4P1	-0.004	0.911555069	0.967856992
RP11-708L7	-0.004	0.911431941	0.967856992
RGPD3	-0.004	0.911422696	0.967856992
OR7E162P	-0.004	0.911417447	0.967856992
NRG3-AS1	-0.004	0.911359392	0.967856992
AKR7A2	-0.004	0.911283155	0.967856992
AC010887	-0.004	0.911083383	0.967711158
KRTAP4-1	-0.004	0.911082529	0.967711158
MYCBP2-AS2	-0.004	0.911058446	0.967711158
MED25	-0.004	0.910975278	0.967682656
AC009878	-0.004	0.910924844	0.967650665
RNU6-464P	-0.004	0.910869252	0.967633221
AC087393	-0.004	0.910845234	0.967630843
RP11-383I23	-0.004	0.910839845	0.967630843
HAT1	-0.004	0.910794715	0.967620341
WDR54	-0.004	0.910586445	0.967463819
BMP6P1	-0.004	0.910457529	0.967434758
HNRNPDLP2	-0.004	0.91043785	0.967434758
BEST1	-0.004	0.9103561	0.967370147
LINC01158	-0.004	0.910303858	0.967357799
RP11-515E23	-0.004	0.910284019	0.967357799
LRFN5	-0.004	0.910282592	0.967357799
POTEB	-0.004	0.91019472	0.967349741
RP11-972P1	-0.004	0.910133555	0.967306321
RP4-646P19	-0.004	0.910025698	0.967224749
FAM60DP	-0.004	0.909885282	0.967193535
KRTAP13-2	-0.004	0.909878172	0.967193535
SNN	-0.004	0.909875949	0.967193535
CYP2C56P	-0.004	0.909866254	0.967193535
RP11-459D22	-0.004	0.909821432	0.967193535
OR9R1P	-0.004	0.909686274	0.9671147
KIF1A	-0.004	0.909106233	0.966581148
AL163952	-0.004	0.909104293	0.966581148
RP11-545I5	-0.004	0.909026134	0.966581148
AP000593	-0.004	0.908942166	0.96653617
TAS2R62P	-0.004	0.90894066	0.96653617
RP11-507K2	-0.004	0.908888909	0.966522698
RNA5SP250	-0.004	0.908660393	0.966365999
RP11-1J11	-0.004	0.908570487	0.966291961
COLQ	-0.004	0.908567285	0.966291961
MTCL1	-0.004	0.908511879	0.966272784
CYP2D7	-0.004	0.908481358	0.966261901
PRKRIRP4	-0.004	0.908452041	0.966252297
RP11-699A7	-0.004	0.9083938	0.966214796
OR4F1P	-0.004	0.908187972	0.966057724
RP11-570L14	-0.004	0.908169619	0.966057724
RP11-157H4	-0.004	0.908142652	0.966052669

CTD-2343P13	-0.004	0.90805929	0.966007143
ZNF286A	-0.004	0.908043975	0.966007143
FABP1	-0.004	0.907985576	0.966007143
C16orf71	-0.004	0.907885129	0.965960731
CPS1-IT1	-0.004	0.907800273	0.965904195
LINC01203	-0.004	0.907767183	0.965904195
GPR64	-0.004	0.90770681	0.965869484
CTD-2026D23	-0.004	0.907542897	0.965759798
PRDX2P1	-0.004	0.907532001	0.965759798
ZNF92P3	-0.004	0.907509685	0.965759798
CTD-2334D19	-0.004	0.90747889	0.965756421
ABCD1P3	-0.004	0.907443054	0.965739863
RP11-704J17	-0.004	0.907327148	0.965652012
RP11-19F9	-0.004	0.907278429	0.965629394
AC139451	-0.004	0.907069663	0.965536661
RP11-217B7	-0.004	0.907066497	0.965536661
AC236972	-0.004	0.906947042	0.965470871
HDAC7	-0.004	0.906885823	0.96544886
NXPE2P1	-0.004	0.906763327	0.965411857
RP3-335N17	-0.004	0.906729889	0.965411857
MIR1238	-0.004	0.906719317	0.965411857
SPACA5B	-0.004	0.906598006	0.965336654
RP11-1191J2	-0.004	0.906569903	0.965336654
RP11-438E8	-0.004	0.906533333	0.965336654
OR4C50P	-0.004	0.906501933	0.965336654
TM9SF4	-0.004	0.906347929	0.965296783
OR5A1	-0.004	0.906245717	0.965220492
PGAM4P2	-0.004	0.906162054	0.965192784
UQCRC2	-0.004	0.906095225	0.96514654
RP11-127I20	-0.004	0.906079647	0.96514654
MYCLP1	-0.004	0.905989793	0.965120577
RP4-614N24	-0.004	0.905947987	0.965120577
MIR4444-2	-0.004	0.905888074	0.965120577
RN7SL39P	-0.004	0.905862337	0.965120577
AC100802	-0.004	0.905698644	0.965004682
EIF4A1P4	-0.004	0.905620223	0.964964302
MAPK3	-0.004	0.905587248	0.964964302
RP11-577N1	-0.004	0.905579189	0.964964302
CTNND2	-0.004	0.905445332	0.964864295
CWC15	-0.004	0.905434517	0.964864295
DOT1L	-0.004	0.90540423	0.964863673
EIF1P1	-0.004	0.905389415	0.964863673
AC115617	-0.004	0.905382155	0.964863673
RNU6-1279P	-0.004	0.90536717	0.964863673
MIR3942	-0.004	0.905353058	0.964863673
AC021654	-0.004	0.905265377	0.964863673
AZGP1P1	-0.004	0.905254046	0.964863673
MLN	-0.004	0.905225233	0.964863673
EBF1	-0.004	0.905117383	0.96483864
TUBBP8	-0.004	0.905067811	0.964807391
NT5C	-0.004	0.905015713	0.964773448
BTF3P15	-0.004	0.90498049	0.964757494

AC072031	-0.004	0.904870686	0.964679328
KIAA1456	-0.004	0.904866023	0.964679328
RPL13AP17	-0.004	0.904819281	0.964679328
RP11-500G22	-0.004	0.904808916	0.964679328
MKX-AS1	-0.004	0.904796506	0.964679328
TMEM72	-0.004	0.904743362	0.964679328
TUBB1	-0.004	0.904712628	0.964679328
OR8V1P	-0.004	0.904692196	0.964679328
NAALAD2	-0.004	0.904588716	0.964663728
RP11-342I1	-0.004	0.90458392	0.964663728
FOXD4L6	-0.004	0.904426132	0.964576738
RP11-535A19	-0.004	0.904391445	0.964561344
MIR5684	-0.004	0.90438012	0.964561344
CHORDC2P	-0.004	0.90436073	0.964561344
CTB-2L9	-0.004	0.904328072	0.964558553
ECM1P1	-0.004	0.904066252	0.964344085
SLC30A1	-0.004	0.903874952	0.964248009
RP11-489M13	-0.004	0.90369448	0.964163463
AC006133	-0.004	0.903678451	0.964163463
RP11-756A22	-0.004	0.903566559	0.964091774
CTB-55O6	-0.004	0.903516802	0.964060282
RP11-203H19	-0.004	0.903490838	0.964054175
TXNL4A	-0.004	0.90334552	0.963963906
SCN9A	-0.004	0.903342058	0.963963906
VN1R96P	-0.004	0.903325105	0.963963906
RNU6-715P	-0.004	0.903303852	0.963963906
NGFR	-0.004	0.903240417	0.963963906
MALRD1	-0.004	0.903237763	0.963963906
RP1-154J13	-0.004	0.903171348	0.963963906
AC069542	-0.004	0.903039495	0.963896505
MIR4662B	-0.004	0.902875909	0.963743493
OR10A5	-0.004	0.902725703	0.963647953
RP11-329N22	-0.004	0.902669611	0.963609673
TRIM74	-0.004	0.902623662	0.963603819
MIR548AX	-0.004	0.90257688	0.963585725
AC113607	-0.004	0.902570898	0.963585725
RP11-285A15	-0.004	0.902549638	0.963585725
RP11-416A17	-0.004	0.902520857	0.963585725
CYB5D2	-0.004	0.902419548	0.96355917
ZNF965P	-0.004	0.902395725	0.96355917
RPS2P4	-0.004	0.902365465	0.96355917
RP3-332O11	-0.004	0.902322856	0.963541864
VPS9D1-AS1	-0.004	0.902235321	0.963491595
RNU6-1160P	-0.004	0.902224063	0.963491595
MIR138-1	-0.004	0.902192361	0.963491595
WDR86-AS1	-0.004	0.902186974	0.963491595
RP11-335E14	-0.004	0.902071938	0.963468339
HIGD2B	-0.004	0.902069274	0.963468339
SLIT2	-0.004	0.902016937	0.963452807
RPS2P44	-0.004	0.901999446	0.963452807
C10orf53	-0.004	0.901976065	0.963452365
LIPI	-0.004	0.901872895	0.963380304

IQCG	-0.004	0.901868181	0.963380304
RPL31P63	-0.004	0.90175913	0.963316251
SNRPCP17	-0.004	0.901683165	0.963269124
TRIM51HP	-0.004	0.901618796	0.963239942
NTNG1	-0.004	0.901585204	0.96322929
IGHVII-78-1	-0.004	0.901551383	0.963214764
NXNP1	-0.004	0.901519695	0.963202515
LL22NC03-33B6	-0.004	0.901517732	0.963202515
AL034375	-0.004	0.90133079	0.963108713
RP11-142A5	-0.004	0.901233517	0.963091203
CMC2	-0.004	0.90089097	0.962833163
RP11-297C4	-0.004	0.900787954	0.962744668
RNU6-1000P	-0.004	0.900765862	0.962744668
RP11-448A19	-0.004	0.900677387	0.962720981
RP11-208C17	-0.004	0.900626997	0.962702262
AC010103	-0.004	0.900586766	0.962702262
MIR181B2	-0.004	0.900535382	0.962690758
AC073834	-0.004	0.900493587	0.962669132
CIZ1	-0.004	0.900334546	0.962540875
RP11-15J23	-0.004	0.900140497	0.962398228
CYP2W1	-0.004	0.900108134	0.96238523
RP11-212J19	-0.004	0.900020558	0.962334803
RP11-125O5	-0.004	0.899883718	0.962231693
RN7SKP58	-0.004	0.8998129	0.962177571
COX7A2P2	-0.004	0.89979309	0.962177571
C11orf49	-0.004	0.899683933	0.962147675
FTX_2	-0.004	0.899680774	0.962147675
MRPL40	-0.004	0.899673559	0.962147675
RP11-780O17	-0.004	0.899659958	0.962147675
LINC01475	-0.004	0.899654772	0.962147675
IGF2BP2-AS1	-0.004	0.899582538	0.962147261
MAGEC3	-0.004	0.899507243	0.962147261
CTB-78F1	-0.004	0.899373762	0.962068491
TSHZ2	-0.004	0.899354978	0.962068491
AC093787	-0.004	0.899349314	0.962068491
LCA5	-0.004	0.898988004	0.961943421
DEFB103A	-0.004	0.898987189	0.961943421
RNY3P5	-0.004	0.898844642	0.961833238
TRBV26OR9-2	-0.004	0.898779362	0.961784993
RPL31P47	-0.004	0.898759625	0.961784993
PLAC1	-0.004	0.898742763	0.961784993
AL031320	-0.004	0.898551263	0.961677833
RP11-268F1	-0.004	0.898546139	0.961677833
CTC-539A10	-0.004	0.898410427	0.961588669
UCK2	-0.004	0.898315057	0.961588669
OR4D7P	-0.004	0.898234157	0.96157082
EQTN	-0.004	0.898193043	0.96157082
F9	-0.004	0.898112597	0.961546783
FLT3LG	-0.004	0.898074768	0.961527897
PMS2P1	-0.004	0.897987471	0.961520892
BTF3L4P4	-0.004	0.897903924	0.961475928
RN7SL817P	-0.004	0.897842777	0.961475928

LINC01164	-0.004	0.897828057	0.961475928
HBG1	-0.004	0.897809707	0.961475928
ALOXE3	-0.004	0.897708716	0.961475928
ALKBH3-AS1	-0.004	0.897648229	0.961460266
RNU4-18P	-0.004	0.897620577	0.961452271
RP3-406P24	-0.004	0.897549275	0.961410943
OR5AL2P	-0.004	0.897531373	0.961410943
PCBD1	-0.004	0.897490073	0.961410943
FBP2	-0.004	0.897357271	0.96134121
FGF20	-0.004	0.897344417	0.96134121
SNORD115-38	-0.004	0.897304581	0.961330003
RNU6-20P	-0.004	0.897274425	0.961319319
RP11-404I7	-0.004	0.897218713	0.961281255
GBAS	-0.004	0.897169012	0.96124963
RP11-485F13	-0.004	0.896830089	0.960994592
UGT2B4	-0.004	0.896716625	0.96091625
RP11-364P22	-0.004	0.896616491	0.960830565
RNF25	-0.004	0.896534285	0.960828947
RNU6-321P	-0.004	0.896457202	0.960805531
CTD-2517M22	-0.004	0.896423679	0.960805531
POLR2J3	-0.004	0.896363624	0.960802252
RNU6-667P	-0.004	0.896199384	0.960737948
CYP2C18	-0.004	0.896115299	0.960682558
AKAP13	-0.004	0.896100514	0.960682558
RP11-527F13	-0.004	0.896093586	0.960682558
NDUFB1P1	-0.004	0.89607318	0.960682558
MIR300	-0.004	0.896027821	0.960682558
LRRC47	-0.004	0.895988187	0.960676033
CTD-2033C11	-0.004	0.89596356	0.960671255
MTG2	-0.004	0.895960106	0.960671255
RN7SKP88	-0.004	0.895929403	0.960671255
RASGRF1	-0.004	0.895831366	0.960637648
RP4-742J24	-0.004	0.895726792	0.960590392
MRPS35P3	-0.004	0.895667488	0.960570052
RNA5SP24	-0.004	0.895540151	0.960498373
DPRXP1	-0.004	0.895520212	0.960498373
RP4-529N6	-0.004	0.895395134	0.960407721
FLJ37035	-0.004	0.895383822	0.960407721
AL357936	-0.004	0.895336641	0.960388239
MIR548O	-0.004	0.895208205	0.960304175
MED28P2	-0.004	0.895199536	0.960304175
DYRK1B	-0.004	0.89516611	0.960304175
RP11-687F6	-0.004	0.895033814	0.960253336
AL049758	-0.004	0.894933765	0.960237236
CFL1P1	-0.005	0.894914065	0.960237236
COPS3	-0.005	0.894874975	0.960237236
TRIM60	-0.005	0.89487383	0.960237236
MED15P5	-0.005	0.894861721	0.960237236
EXOSC1	-0.005	0.894819255	0.960237236
COX6B1P4	-0.005	0.894765332	0.960237236
RP11-442N1	-0.005	0.894735801	0.960237236
SPINK14	-0.005	0.894524549	0.960122613

RP11-187O7		-0.005	0.894410873	0.960115502
CTC-506B8		-0.005	0.894364087	0.96010829
RP11-778D9		-0.005	0.894351055	0.96010829
RNU4-27P		-0.005	0.894339843	0.96010829
RP11-300M6		-0.005	0.894142333	0.959993618
PLCE1		-0.005	0.894075076	0.959943047
RP11-656G20		-0.005	0.894040409	0.959943047
RP11-12M5		-0.005	0.894016765	0.959943047
RNA5SP115		-0.005	0.893980415	0.959927972
RNU4-33P		-0.005	0.893955773	0.959923154
PHLDB3		-0.005	0.893784426	0.959760802
MIR6859-1		-0.005	0.893767022	0.959760802
SEMA4G		-0.005	0.893695032	0.959729724
RNU6-66P		-0.005	0.893635281	0.959687198
CTD-3187F8		-0.005	0.893613535	0.959685483
HTR3B		-0.005	0.893522527	0.959609385
AL117374		-0.005	0.893309633	0.959402379
HAMP		-0.005	0.893276579	0.959388514
CYP4F25P		-0.005	0.893259483	0.959388514
	7-Sep	-0.005	0.893233829	0.959388514
RPLP0P6		-0.005	0.893221306	0.959388514
RNASEH1-AS1		-0.005	0.893111625	0.959319516
MIR4642		-0.005	0.893109084	0.959319516
CTD-3214K23		-0.005	0.893065721	0.959319516
ANGPTL1		-0.005	0.893006695	0.959293348
RP11-215C7		-0.005	0.892991342	0.959293348
RPL7P19		-0.005	0.892990005	0.959293348
RP11-395C17		-0.005	0.892983389	0.959293348
RP11-95P9		-0.005	0.892927615	0.959293348
CTD-3233P19		-0.005	0.892875372	0.959293348
RP11-540E16		-0.005	0.892780763	0.959264876
RNU7-124P		-0.005	0.892602786	0.959162332
CTA-204B4		-0.005	0.892590451	0.959162332
SELV		-0.005	0.89255876	0.959162332
MIR4661		-0.005	0.892555273	0.959162332
RP11-707A18		-0.005	0.892492568	0.959162332
SMIM22		-0.005	0.892488121	0.959162332
STC2		-0.005	0.892359025	0.959138759
HMGN2P27		-0.005	0.892358267	0.959138759
RN7SL291P		-0.005	0.892298685	0.959138518
JAGN1		-0.005	0.892295557	0.959138518
PCMTD1P3		-0.005	0.892280153	0.959138518
LLPH		-0.005	0.892247201	0.959138518
RP11-288D15		-0.005	0.892235882	0.959138518
RP11-20F24		-0.005	0.892144416	0.959126255
PCDHA9		-0.005	0.892052666	0.959069869
RN7SL242P		-0.005	0.891964272	0.959060444
GSTM5P1		-0.005	0.891929138	0.959044318
TTLL7-IT1		-0.005	0.891916004	0.959044318
TMEM60		-0.005	0.891830147	0.959029158
RP11-529O20		-0.005	0.891791509	0.959029158
RP11-24B13		-0.005	0.891776095	0.959029158

RP11-419L9	-0.005	0.891753585	0.959029158
FXYD3	-0.005	0.891405784	0.958762975
AC004595	-0.005	0.891360475	0.958757545
RN7SL665P	-0.005	0.891170428	0.958618077
IFT20	-0.005	0.891066921	0.958593337
CFAP20	-0.005	0.891034546	0.958580161
ZFPL1	-0.005	0.89094174	0.958501971
RP11-467P9	-0.005	0.89087247	0.958481188
MIR450A1	-0.005	0.890841721	0.958480971
RP11-152L7	-0.005	0.890834977	0.958480971
RP5-1061H20	-0.005	0.890808362	0.958480971
MIR4748	-0.005	0.890781588	0.958480971
ANTXRL	-0.005	0.890646919	0.958429373
TBC1D3G	-0.005	0.890592705	0.958429373
EPB41L2	-0.005	0.890533413	0.95838741
DMRTC1	-0.005	0.890527632	0.95838741
TFPI	-0.005	0.89047946	0.958372658
AC012627	-0.005	0.890468119	0.958372658
RP11-540H22	-0.005	0.890310313	0.958255577
RP11-182L7	-0.005	0.890304022	0.958255577
RP11-876F14	-0.005	0.89029925	0.958255577
ZNF876P	-0.005	0.890294731	0.958255577
FAHD2CP	-0.005	0.890236034	0.958255577
RPL17P49	-0.005	0.890157052	0.958242206
SLC22A23	-0.005	0.890040523	0.958179958
RP5-1016A21	-0.005	0.890006457	0.958179958
MIR329-1	-0.005	0.889916191	0.958156176
LINC01015	-0.005	0.889837488	0.958136418
POLL	-0.005	0.889558033	0.957943799
EEF1A1P41	-0.005	0.88948985	0.957913693
CEACAMP1	-0.005	0.889466871	0.957913693
PGAP3	-0.005	0.889430296	0.957913693
RP11-393K19	-0.005	0.889289885	0.957806634
MIR6797	-0.005	0.889289141	0.957806634
RNA5SP74	-0.005	0.88925066	0.957806634
OR13K1P	-0.005	0.889200482	0.95777532
RNU6-47P	-0.005	0.889184508	0.95777532
AC104076	-0.005	0.889175624	0.95777532
MRPS28	-0.005	0.889012583	0.957700871
VPS45	-0.005	0.8889772	0.957700871
RP11-521O16	-0.005	0.88887887	0.957688812
RN7SL655P	-0.005	0.888841693	0.957670422
PISRT1	-0.005	0.888784777	0.957630762
RNU6-412P	-0.005	0.888705116	0.957588258
HOXA11-AS1_4	-0.005	0.888669443	0.957571483
OR7E140P	-0.005	0.888597264	0.957515371
LRRC20	-0.005	0.88858886	0.957515371
RNY1P10	-0.005	0.888587546	0.957515371
AC019196	-0.005	0.888446813	0.957486608
OGFOD3	-0.005	0.888386071	0.957461099
AL139022	-0.005	0.888355003	0.957449281
NDUFAF4P2	-0.005	0.888257528	0.957409222

CTC-490E21	-0.005	0.88815352	0.957318782
OR6B1	-0.005	0.888067631	0.957303037
ATHL1	-0.005	0.888043072	0.957303037
HNRNPRP2	-0.005	0.88799231	0.957275006
RN7SL342P	-0.005	0.887960471	0.95726235
CTB-23I7	-0.005	0.887870522	0.957187047
MIR4282	-0.005	0.887788949	0.957120771
KRT8P20	-0.005	0.887683389	0.95702863
HIRIP3	-0.005	0.887600009	0.956982063
ISL1	-0.005	0.887538367	0.956958929
MIR6730	-0.005	0.887498242	0.956937329
RP6-105D16	-0.005	0.887322944	0.956834964
PKN2-AS1	-0.005	0.887302816	0.956834923
RNU6-758P	-0.005	0.887274002	0.956834923
CCDC102B	-0.005	0.887257059	0.956834923
PTP4A1P1	-0.005	0.887251203	0.956834923
RAB11AP1	-0.005	0.887207149	0.956834923
RP1-137H15	-0.005	0.887062734	0.956749328
VIS1	-0.005	0.886910657	0.956636374
AC008836	-0.005	0.886791224	0.95658144
ARHGAP29	-0.005	0.886571652	0.956417848
AP000567	-0.005	0.886436248	0.956333511
RP11-16C1	-0.005	0.886328289	0.956260362
CBWD7	-0.005	0.886195555	0.956138815
RN7SL551P	-0.005	0.886188237	0.956138815
CTD-2001C12	-0.005	0.886162809	0.956138815
CREB3	-0.005	0.88613345	0.956138815
SPATC1L	-0.005	0.88612504	0.956138815
NIFKP8	-0.005	0.886099447	0.956138815
MEPE	-0.005	0.886097203	0.956138815
TMEM212	-0.005	0.88605181	0.956138815
DNAH10OS	-0.005	0.885742721	0.955996749
ATP5LP3	-0.005	0.885676859	0.955947327
MIR889	-0.005	0.885392421	0.95580956
DLX6-AS1	-0.005	0.885341726	0.95580956
RNU6-215P	-0.005	0.885330842	0.95580956
RN7SL337P	-0.005	0.885297498	0.95580956
RP11-16B13	-0.005	0.885147224	0.95574388
RP11-340F16	-0.005	0.885137439	0.95574388
MIR1185-2	-0.005	0.885016593	0.955667829
TMEM88	-0.005	0.885014382	0.955667829
SLC25A33	-0.005	0.884982905	0.955667829
GPT	-0.005	0.884870796	0.955640394
PRAMEF9	-0.005	0.884763579	0.955589609
POMZP3	-0.005	0.884763432	0.955589609
RPL12P41	-0.005	0.884637249	0.955561513
OR6C7P	-0.005	0.884609237	0.955552927
HMGB3P9	-0.005	0.884576417	0.955539147
SCO2	-0.005	0.884370644	0.955425216
LINC01467	-0.005	0.884330972	0.955404028
RN7SL237P	-0.005	0.884316926	0.955404028
RPL21P11	-0.005	0.884282082	0.955404028

B3GALNT2P1	-0.005	0.884242136	0.955394742
RP1-267D11	-0.005	0.884074024	0.955234773
RP11-67L2	-0.005	0.883797251	0.95510974
RP11-790L1	-0.005	0.883750846	0.95510974
TRMT112P1	-0.005	0.883719187	0.95510974
PDK3	-0.005	0.88370629	0.95510974
LINC01341	-0.005	0.883658007	0.955088617
KRT8P40	-0.005	0.883477465	0.954958496
COQ2	-0.005	0.883453676	0.954958496
AGBL4	-0.005	0.883402745	0.954958496
RP11-216P16	-0.005	0.883398745	0.954958496
TBCCD1	-0.005	0.883370479	0.954958496
LCN1P2	-0.005	0.883317326	0.954955423
RNA5SP343	-0.005	0.883224289	0.954900986
AC087738	-0.005	0.883155775	0.954849155
SYT11	-0.005	0.883006672	0.954749932
CTA-963H5	-0.005	0.882967448	0.954749932
SQLE	-0.005	0.882885121	0.954686563
MIR544B	-0.005	0.882866972	0.954686563
AC003664	-0.005	0.882823757	0.954686563
DLG1	-0.005	0.882817881	0.954686563
KRT18P26	-0.005	0.882808517	0.954686563
RP11-16O13	-0.005	0.882732774	0.954686563
LINC01362	-0.005	0.882692954	0.954686563
CTD-2313J23	-0.005	0.882640882	0.954682563
RP11-17A4	-0.005	0.882541206	0.954639791
RNU6-60P	-0.005	0.882515261	0.954633408
RP11-624D11	-0.005	0.882508374	0.954633408
AL592205	-0.005	0.882362071	0.954619472
CRTC3	-0.005	0.882192937	0.954544903
RP11-211F2	-0.005	0.882178992	0.954544903
LINC01090	-0.005	0.882152783	0.954544828
PDZD4	-0.005	0.882077438	0.954528359
NLE1	-0.005	0.88206925	0.954528359
USP22	-0.005	0.881958925	0.95448686
OR5F2P	-0.005	0.88188648	0.954430145
RGS10	-0.005	0.881855928	0.95442716
CCL16	-0.005	0.88185387	0.95442716
SLC7A13	-0.005	0.881853191	0.95442716
CDKL3	-0.005	0.881846448	0.95442716
LSMEM1	-0.005	0.881814989	0.95442716
MIR500A	-0.005	0.881797667	0.95442716
AF104455	-0.005	0.881793368	0.95442716
RP11-295K2	-0.005	0.881717836	0.95442716
CECR5-AS1	-0.005	0.881671703	0.95442716
SLC25A5P9	-0.005	0.881662169	0.95442716
AC005391	-0.005	0.881436565	0.954333567
SCCPDH	-0.005	0.881409489	0.954333567
RNU6-463P	-0.005	0.881381903	0.954333567
MIR4710	-0.005	0.881381364	0.954333567
AL139187	-0.005	0.881326664	0.954333567
RP11-332H21	-0.005	0.881115766	0.954263891

OR2B4P	-0.005	0.881099976	0.954263891
AC117479	-0.005	0.880995783	0.954203401
AC007795	-0.005	0.880937365	0.954161828
AC011901	-0.005	0.880923318	0.954161828
RP11-553N16	-0.005	0.880910474	0.954161828
RP11-1236K1	-0.005	0.880866516	0.954161828
AC013399	-0.005	0.880840582	0.954161828
C12orf49	-0.005	0.880600307	0.954057116
RP11-575M4	-0.005	0.880531064	0.954025502
GLT1D1	-0.005	0.88046858	0.953979505
SNORD65	-0.005	0.880441875	0.953972271
CSNK2A1	-0.005	0.880420649	0.953970976
RNA5SP85	-0.005	0.880311986	0.953896639
RBMY3AP	-0.005	0.880234902	0.953834812
PPIAL4F	-0.005	0.880194296	0.953812512
CCDC148	-0.005	0.880143204	0.953778848
RP11-439M15	-0.005	0.879962903	0.953666403
FAM86EP	-0.005	0.879923499	0.953649254
NANP	-0.005	0.879861466	0.953603724
SKIDA1	-0.005	0.879838635	0.953600679
NHLRC4	-0.005	0.879828296	0.953600679
MRPS5P4	-0.005	0.879818698	0.953600679
SNORD116-5	-0.005	0.879625541	0.953543317
MAGI2-AS2	-0.005	0.879607313	0.953543317
RP11-385H1	-0.005	0.879593016	0.953543317
RP11-527D7	-0.005	0.879422895	0.953475556
AC021037	-0.005	0.879379251	0.953466912
RNU6-171P	-0.005	0.879183473	0.953367894
RNF167	-0.005	0.879176569	0.953367894
BANP	-0.005	0.879070612	0.953330788
NPAP1P2	-0.005	0.879038571	0.953319292
RP11-99A14	-0.005	0.879022289	0.953319292
FAM210B	-0.005	0.87896595	0.953319292
CYP2G1P	-0.005	0.878912773	0.953313109
SLC2A3P4	-0.005	0.878699916	0.953169063
C6orf47	-0.005	0.87862398	0.953130109
RAF1P1	-0.005	0.878604558	0.953130109
RP11-251P6	-0.005	0.878492898	0.953031325
OR6K5P	-0.005	0.878447051	0.953003296
VAPA	-0.005	0.878438361	0.953003296
AC019171	-0.005	0.878431244	0.953003296
RNA5SP419	-0.005	0.878318999	0.952929494
RP11-133F8	-0.005	0.878212034	0.952878564
TMEM167AP1	-0.005	0.878132013	0.952878564
MIR548Q	-0.005	0.878052836	0.952857784
FARSB	-0.005	0.878012883	0.952853259
CALCP	-0.005	0.877990348	0.952853259
CCDC151	-0.005	0.877904797	0.952805683
OR6D1P	-0.005	0.877844496	0.952761948
IQCF4	-0.005	0.877508419	0.952462305
RP11-203P2	-0.005	0.877494796	0.952462305
FAM106DP	-0.005	0.877111829	0.95230097

MIR6856		-0.005	0.876819545	0.952148577
PLEKHA8		-0.005	0.876792631	0.952141061
RP11-664H17		-0.005	0.876669627	0.95209432
MGME1		-0.005	0.876568333	0.95204944
RP11-124G5		-0.005	0.876541666	0.952042187
RNU6-857P		-0.005	0.876341551	0.951911669
ZNF492		-0.005	0.876330586	0.951911669
FUT8-AS1		-0.005	0.876317782	0.951911669
OR7D2		-0.005	0.87627836	0.951911669
RNA5SP62		-0.005	0.876264644	0.951911669
AC007179		-0.005	0.875930643	0.951709702
RP11-122F24		-0.005	0.875895772	0.951709623
RP11-336F14		-0.005	0.875835427	0.951687479
STRADB		-0.005	0.875736233	0.951623117
RP11-363N22		-0.005	0.875704812	0.95161726
RNU6-722P		-0.005	0.875638908	0.95161726
KRT31		-0.005	0.875476171	0.951492483
RP11-758H6		-0.005	0.875402354	0.951433968
RP11-463M14		-0.005	0.875378909	0.951430198
RP11-813P10		-0.005	0.875349005	0.951430198
GFRA4		-0.005	0.875279578	0.951409085
CYP27A1		-0.005	0.875164648	0.95130587
AF238380		-0.005	0.875020497	0.951193263
RP11-19O2		-0.005	0.874754827	0.95105575
AIFM1P1		-0.005	0.874717906	0.951037318
ISCA1P6		-0.005	0.874585366	0.950940391
AF001550		-0.005	0.874492778	0.950936554
RP11-144I2		-0.005	0.874417999	0.950884902
RP4-802A10		-0.005	0.8743845	0.950870185
PTTG1		-0.005	0.874307823	0.950808509
ZNF98		-0.005	0.874301012	0.950808509
RP11-57B24		-0.005	0.874291272	0.950808509
	10-Sep	-0.005	0.874265652	0.950808509
RNU105C		-0.005	0.874216496	0.950808509
RP11-566K11		-0.005	0.874172199	0.950808509
HTATSF1P		-0.005	0.874154488	0.950808509
SNORA74		-0.005	0.874089545	0.950808509
UBE2V1P9		-0.005	0.873945442	0.95067491
AC068724		-0.005	0.873842252	0.950606084
RNA5SP80		-0.005	0.873788388	0.950599185
RP4-655C5		-0.005	0.873711514	0.950599185
MRPL42P3		-0.005	0.873703866	0.950599185
RFKP2		-0.005	0.873634062	0.95059673
AC115286		-0.005	0.873597085	0.950578213
ZNF738		-0.005	0.873566449	0.950566595
OCA2		-0.005	0.873556899	0.950566595
AC098848		-0.005	0.873501818	0.9505397
RNU2-12P		-0.005	0.87341473	0.950488367
AC233287		-0.005	0.87336292	0.950469649
GAPDHP1		-0.005	0.873175799	0.950372137
H2BFWT		-0.005	0.873125665	0.95036923
OR7E121P		-0.005	0.873119461	0.95036923

TSSC1	-0.005	0.873103094	0.95036923
RP11-284L19	-0.005	0.872944164	0.950280415
HTR3E-AS1	-0.005	0.872898623	0.950280415
AC097724	-0.005	0.872844975	0.950259164
CCDC58P5	-0.005	0.872820788	0.950259164
AL158077	-0.005	0.872703547	0.950259164
AC067735	-0.005	0.872634033	0.950259164
HSPA8P17	-0.005	0.872557426	0.950259164
RP11-14N9	-0.005	0.872535459	0.950259164
AL590764	-0.005	0.872419213	0.95020833
LRRC75A-AS1	-0.005	0.872311574	0.950155633
CYTH2	-0.005	0.872264689	0.950126941
RP13-262C2	-0.005	0.872183535	0.950097778
RP11-343J18	-0.005	0.872173847	0.950097778
RP11-426M1	-0.005	0.871988149	0.949956072
ERN2	-0.005	0.871950026	0.949945907
SPATA31D3	-0.005	0.871883619	0.94992911
PRELP	-0.005	0.871847906	0.949911931
RNU6-874P	-0.005	0.871771826	0.949850768
RP11-327I22	-0.005	0.871755871	0.949850768
RP11-173A6	-0.006	0.871585645	0.94975655
RNU2-24P	-0.006	0.871571588	0.94975655
RP4-706G24	-0.006	0.87155106	0.94975655
AC069063	-0.006	0.871534335	0.94975655
IARS2P1	-0.006	0.871527631	0.94975655
SUN1	-0.006	0.871476746	0.94975655
RP11-809H16	-0.006	0.871359086	0.949699001
ZNF885P	-0.006	0.871341975	0.949699001
FAM197Y8	-0.006	0.871337811	0.949699001
RPS29P6	-0.006	0.871295322	0.949699001
RP11-467D6	-0.006	0.871259137	0.949699001
C17orf70	-0.006	0.87106261	0.949642882
ZBED3-AS1	-0.006	0.871033798	0.949633208
RP11-11L12	-0.006	0.871004806	0.949623338
CHEK2P3	-0.006	0.87095393	0.949589608
SNORD115-31	-0.006	0.87090312	0.949589608
GABRA4	-0.006	0.870836573	0.949570343
DUXAP7	-0.006	0.87079372	0.949545355
MIR1303	-0.006	0.870733505	0.949523175
RNU6-615P	-0.006	0.870692016	0.949499671
HGFAC	-0.006	0.87067369	0.949499671
BTG4	-0.006	0.870656431	0.949499671
C19orf44	-0.006	0.870582291	0.949488717
SLC20A1P1	-0.006	0.87048421	0.949403487
OR4C11	-0.006	0.870423487	0.94938074
RNU6-642P	-0.006	0.870399744	0.94938074
LA16c-352F7	-0.006	0.870265161	0.94927327
RP11-256L11	-0.006	0.870100816	0.949137092
NPAS3	-0.006	0.870090412	0.949137092
AC007950	-0.006	0.870083246	0.949137092
RP11-269F21	-0.006	0.869901083	0.949014324
RNU6-849P	-0.006	0.869767414	0.948947669

AP3B1	-0.006	0.86965447	0.948867917
LINC01492	-0.006	0.869561833	0.948810315
SUMO2P8	-0.006	0.869552149	0.948810315
BPI	-0.006	0.869528236	0.948810315
WFIKKN2	-0.006	0.869450725	0.948797765
VDAC3P1	-0.006	0.869379242	0.948741497
LINC01287	-0.006	0.869362165	0.948741497
PCBP3-OT1	-0.006	0.869252237	0.948668109
CLSTN3	-0.006	0.869237911	0.948668109
TNNC1	-0.006	0.869195978	0.948650187
PSAT1P1	-0.006	0.86916101	0.948633761
AC007064	-0.006	0.868705155	0.948440511
RP11-456P18	-0.006	0.868682554	0.948437577
LINC01432	-0.006	0.868659793	0.948434469
RPL30P14	-0.006	0.868494134	0.948275335
RNU6-422P	-0.006	0.868429031	0.94822599
TUBBP5	-0.006	0.868420155	0.94822599
RPL23AP77	-0.006	0.86833838	0.948170485
BRI3BPP1	-0.006	0.868336461	0.948170485
FDFT1	-0.006	0.868315077	0.948170485
MIR6729	-0.006	0.868261798	0.948170485
RP11-338L22	-0.006	0.868256211	0.948170485
RNU6-953P	-0.006	0.868251739	0.948170485
LINC00890	-0.006	0.86824748	0.948170485
RP11-63A23	-0.006	0.868217756	0.948170485
RP11-94D20	-0.006	0.86806049	0.948170485
CTD-2538G9	-0.006	0.86799105	0.948117282
PMCHL1	-0.006	0.867988629	0.948117282
RNU6-995P	-0.006	0.867963413	0.948117282
AL353763	-0.006	0.867935066	0.948117282
RNU6-473P	-0.006	0.867786807	0.948024641
AC096750	-0.006	0.867728821	0.948004785
HMGB1P9	-0.006	0.867553622	0.947856863
RNU6-573P	-0.006	0.867539261	0.947856863
RN7SKP124	-0.006	0.867538446	0.947856863
RP11-538D16	-0.006	0.867528477	0.947856863
AC009134	-0.006	0.867478359	0.947856863
BOLA3-AS1	-0.006	0.867455159	0.947856863
EXOC4	-0.006	0.867454299	0.947856863
SNORD91A	-0.006	0.867268106	0.94780583
RNU7-103P	-0.006	0.867263694	0.94780583
RNU7-81P	-0.006	0.867209322	0.947785084
RPL18AP17	-0.006	0.867170143	0.947764013
PRSS30P	-0.006	0.867132246	0.947744343
RP11-157J24	-0.006	0.867113354	0.947744343
GBX1	-0.006	0.867075106	0.947725389
MIDN	-0.006	0.867056839	0.947725389
SLC25A12	-0.006	0.867052542	0.947725389
RP11-407P2	-0.006	0.867018481	0.947725389
AC007253	-0.006	0.866965026	0.947713817
AC117490	-0.006	0.866945396	0.947713817
CRISP1	-0.006	0.866853412	0.947657059

FAM150B	-0.006	0.866845692	0.947657059
RP11-180K7	-0.006	0.86682646	0.947657059
PSMD7	-0.006	0.866820527	0.947657059
CTA-407F11	-0.006	0.866773013	0.947657059
RP11-260N14	-0.006	0.8667302	0.947657059
ASIC3	-0.006	0.866704524	0.947657059
MIR3138	-0.006	0.866651653	0.947654015
PHOX2A	-0.006	0.866623123	0.947654015
MIR3180-2	-0.006	0.866615396	0.947654015
RP11-767C6	-0.006	0.866588883	0.947654015
LINC00374	-0.006	0.866351302	0.947489447
BRD7P2	-0.006	0.866100922	0.947312801
ANKRD60	-0.006	0.866087677	0.947312801
KRT18P8	-0.006	0.866074133	0.947312801
ZNF259P1	-0.006	0.866040856	0.947312801
AL359853	-0.006	0.866002496	0.947312801
MTND1P23	-0.006	0.865978923	0.947312801
IGKV2OR2-8	-0.006	0.865836445	0.947268835
AC063976	-0.006	0.865739937	0.947222513
SNRPCP8	-0.006	0.865715661	0.947217712
TTL	-0.006	0.865529185	0.947035437
CSNK1A1L	-0.006	0.865189587	0.946772614
AMZ2P2	-0.006	0.865187556	0.946772614
TMEM175	-0.006	0.8651641	0.946772614
LINC01128	-0.006	0.865152503	0.946772614
LINC00567	-0.006	0.865025829	0.946746513
LINC00682	-0.006	0.865010453	0.946746513
CTD-2228A4	-0.006	0.864955516	0.946733996
C2orf43	-0.006	0.864848002	0.946688129
MTCYBP3	-0.006	0.864810198	0.946683721
HEXA	-0.006	0.864791313	0.946683721
RPL35AP	-0.006	0.864679526	0.946605948
AL354923	-0.006	0.864622016	0.946605948
PPP1R18	-0.006	0.864609225	0.946605948
RP11-120A1	-0.006	0.864530092	0.946572903
AC097372	-0.006	0.864485717	0.946546076
RP11-524O1	-0.006	0.864469535	0.946546076
LINC01578	-0.006	0.864459352	0.946546076
RP11-301A5	-0.006	0.864365812	0.946546076
GAS2L1P2	-0.006	0.864260962	0.946517587
RN7SL754P	-0.006	0.864132026	0.946441669
CYP4F34P	-0.006	0.864111052	0.946440463
YPEL5P1	-0.006	0.864064975	0.94641176
ERGIC2	-0.006	0.864056565	0.94641176
WI2-2994D6	-0.006	0.864025848	0.94641176
RPL12P8	-0.006	0.863997166	0.94641176
MKNK1	-0.006	0.863824699	0.946365499
RP4-791M13	-0.006	0.863691274	0.946263585
RNU1-55P	-0.006	0.863659136	0.946256811
RNA5SP205	-0.006	0.863649028	0.946256811
FOXD1	-0.006	0.86364072	0.946256811
RP11-231E4	-0.006	0.863602145	0.946256811

AC133141	-0.006	0.863578619	0.946256811
RNF216-IT1	-0.006	0.863563107	0.946256811
RAMP1	-0.006	0.863534883	0.946256811
RNU6-701P	-0.006	0.863532883	0.946256811
RNU1-150P	-0.006	0.863518309	0.946256811
ZNF969P	-0.006	0.863481977	0.946256811
HDDC2	-0.006	0.863472629	0.946256811
RN7SL531P	-0.006	0.863411955	0.946256811
AC098612	-0.006	0.863364719	0.946256811
EPB42	-0.006	0.863335727	0.946256811
RP11-490D19	-0.006	0.86329517	0.946256811
RBMY2DP	-0.006	0.86315833	0.946256811
TERF1P2	-0.006	0.862898542	0.946122336
RP11-9G10	-0.006	0.862864011	0.946121332
AL139021	-0.006	0.862796588	0.94608862
RP11-349N19	-0.006	0.862755262	0.946065086
RP11-103J17	-0.006	0.86270874	0.946065086
RNA5SP321	-0.006	0.862610143	0.946058428
LMF1-AS1	-0.006	0.862465707	0.945963768
INHA	-0.006	0.862443193	0.945962461
SGSM2	-0.006	0.862386534	0.945922099
SPATA6	-0.006	0.862361638	0.945916576
RNU6-505P	-0.006	0.862282926	0.945873806
FAM110B	-0.006	0.862281373	0.945873806
AC010423	-0.006	0.862175128	0.94582091
AL360157	-0.006	0.862092556	0.945752111
ENTPD4	-0.006	0.861950452	0.945639781
FMR1NB	-0.006	0.861867798	0.945570884
ARL2BPP6	-0.006	0.861799094	0.945539069
PHB	-0.006	0.861798091	0.945539069
TPRG1L	-0.006	0.861780065	0.945539069
RP11-2N1	-0.006	0.861740249	0.945539069
ENO1-IT1	-0.006	0.86163588	0.945490682
LINC01393	-0.006	0.86156909	0.945439174
EEF2	-0.006	0.861547028	0.945436748
AC007364	-0.006	0.861494876	0.945401301
FTH1P14	-0.006	0.861422778	0.945385113
DYNC1H1	-0.006	0.861272805	0.945353403
SLC37A2	-0.006	0.861178827	0.945294061
HAUS1P2	-0.006	0.861155701	0.945294061
SOAT1	-0.006	0.861142352	0.945294061
LINC01527	-0.006	0.861133843	0.945294061
CTC-384G19	-0.006	0.861106829	0.945294061
NQO2	-0.006	0.861004607	0.945255323
TPM3	-0.006	0.86097457	0.945244136
VTRNA2-2P	-0.006	0.860963514	0.945244136
OR5BQ1P	-0.006	0.860947393	0.945244136
AL354873	-0.006	0.86089556	0.945244136
WDR77	-0.006	0.860855711	0.945244136
MTHFD2P3	-0.006	0.860821549	0.94522866
CFHR2	-0.006	0.860681252	0.945096396
RNA5SP387	-0.006	0.860554278	0.945022333

HNRNPA1P8	-0.006	0.860551067	0.945022333
MIR519A1	-0.006	0.86048302	0.944987659
DAB1-AS1	-0.006	0.860478199	0.944987659
SLC1A3	-0.006	0.86040948	0.944971484
RP11-548H3	-0.006	0.860392747	0.944971484
DNAH17	-0.006	0.860370008	0.944971484
RNASEH1P1	-0.006	0.860359071	0.944971484
SGCB	-0.006	0.860357136	0.944971484
KRT72	-0.006	0.860336844	0.944971484
AGO3	-0.006	0.860288061	0.944971484
MIR548AO	-0.006	0.860278879	0.944971484
CEP72	-0.006	0.860268755	0.944971484
KB-1836B5	-0.006	0.860217687	0.944971484
ARMC2-AS1	-0.006	0.860093792	0.944971484
C17orf112	-0.006	0.859812679	0.94490128
MIR3659	-0.006	0.859775759	0.94490128
AF212831	-0.006	0.859740045	0.944890715
AC008694	-0.006	0.859715161	0.94488517
AC010982	-0.006	0.859677628	0.944865723
AC097382	-0.006	0.859650769	0.944864154
NPAP1P3	-0.006	0.859523394	0.944761613
RP11-356C4	-0.006	0.859483637	0.944739716
TMEM254	-0.006	0.859392325	0.944661149
STEAP4	-0.006	0.859360583	0.944655055
CH17-333M13	-0.006	0.859352652	0.944655055
RP11-360D2	-0.006	0.859336134	0.944655055
HIST2H2BF	-0.006	0.859285518	0.944655055
LINC00308	-0.006	0.859240864	0.944655055
CICP18	-0.006	0.859230618	0.944655055
FAM173B	-0.006	0.859186005	0.944655055
MIR6718	-0.006	0.859130625	0.944655055
RP11-373E16	-0.006	0.858872225	0.944547243
RP11-385G16	-0.006	0.858855756	0.944547243
AC013448	-0.006	0.858807508	0.944541502
RP11-655M14	-0.006	0.85867425	0.94443856
RNA5SP287	-0.006	0.858637324	0.944419756
SRSF4	-0.006	0.858536654	0.944330837
MIR2355	-0.006	0.858483663	0.944294358
CCDC37-AS1	-0.006	0.858474816	0.944294358
MIR7-2	-0.006	0.858464958	0.944294358
PDYN	-0.006	0.858447286	0.944294358
RP11-3L21	-0.006	0.858386264	0.944294358
AC108485	-0.006	0.858371664	0.944294358
RP11-477N3	-0.006	0.858289205	0.944276738
TMX4	-0.006	0.858284059	0.944276738
GPX5	-0.006	0.858277723	0.944276738
PDIA3P2	-0.006	0.858230957	0.944276738
WNT8A	-0.006	0.858224504	0.944276738
HSCB	-0.006	0.858204295	0.944276738
RP11-550A5	-0.006	0.858120252	0.944276738
MIR4637	-0.006	0.858053531	0.944276738
TUBB3P1	-0.006	0.857993638	0.944235113

RP11-106E15	-0.006	0.857971212	0.944232252
RNU6-679P	-0.006	0.857840831	0.944110578
KCTD9P2	-0.006	0.857759179	0.944064345
AC106736	-0.006	0.857707401	0.944052192
SCARNA4	-0.006	0.857620425	0.944052192
AFMID	-0.006	0.857532793	0.944052192
MIR585	-0.006	0.857508889	0.944052192
PPRC1	-0.006	0.857435463	0.944019137
SPG7	-0.006	0.857396018	0.944019137
RPS24P13	-0.006	0.857390308	0.944019137
CBX1P2	-0.006	0.857275762	0.943946749
LINC00937	-0.006	0.857272483	0.943946749
SPDYE4	-0.006	0.857222304	0.943931533
RNA5SP227	-0.006	0.857165514	0.943912646
GLOD5	-0.006	0.8571213	0.943909397
LYPD8	-0.006	0.8571187	0.943909397
CEACAM7	-0.006	0.857102894	0.943909397
SOX15	-0.006	0.857070176	0.943909397
DNM1P50	-0.006	0.857002301	0.943907511
OR13D2P	-0.006	0.856998749	0.943907511
KCNMA1	-0.006	0.85694842	0.943907511
AL162853	-0.006	0.856674244	0.94374261
HAGHL	-0.006	0.856627327	0.943734582
RP1-249F5	-0.006	0.856523119	0.943663434
NPC1	-0.006	0.856492724	0.943663434
LINC00408	-0.006	0.856418475	0.943635458
RP3-380C13	-0.006	0.856414595	0.943635458
H2AFY	-0.006	0.8563757	0.943631989
FBXO25	-0.006	0.856055078	0.943405306
MC1R	-0.006	0.856000215	0.943371025
AC004022	-0.006	0.855985967	0.943371025
LINC00112	-0.006	0.855933327	0.943371025
RP5-1099E6	-0.006	0.855887818	0.943371025
SP9	-0.006	0.855878816	0.943371025
EIF2B4	-0.006	0.855839915	0.943371025
MIR548L	-0.006	0.855816631	0.943371025
MIR4525	-0.006	0.855644533	0.943240954
RP11-131P10	-0.006	0.855601471	0.943240954
RP11-338K13	-0.006	0.855565433	0.943240954
NUCB1-AS1	-0.006	0.855548988	0.943240954
RP11-114H24	-0.006	0.855535381	0.943240954
RP11-552O4	-0.006	0.855489619	0.943240954
RP11-98G13	-0.006	0.855380867	0.943190438
IL10RB-AS1	-0.006	0.855372764	0.943190438
AC010209	-0.006	0.855371994	0.943190438
RP11-323H21	-0.006	0.855331384	0.943190438
RP11-5G18	-0.006	0.855288967	0.943190438
MGAT4B	-0.006	0.855272055	0.943190438
RP11-401E5	-0.006	0.855261987	0.943190438
RP11-308D13	-0.006	0.855238831	0.943190438
SULT6B1	-0.006	0.854979734	0.943053794
RDX	-0.006	0.854683702	0.942880123

RPL3P1	-0.006	0.854621786	0.942860071
AC026992	-0.006	0.854571986	0.942860071
AC010504	-0.006	0.854472463	0.942860071
KRT18P41	-0.006	0.854296798	0.942748043
MIR620	-0.006	0.854081627	0.942575601
RPL21P71	-0.006	0.854074559	0.942575601
PYCR1	-0.006	0.85407101	0.942575601
SULT1A4	-0.006	0.854044131	0.942575601
RP5-1022J11	-0.006	0.854003796	0.942575601
LMAN1L	-0.006	0.853901946	0.942563515
CTD-2142D14	-0.006	0.853861852	0.942562948
TIGD6	-0.006	0.853760413	0.942494661
OR51B6	-0.006	0.853691996	0.942462824
CEP170	-0.006	0.853686729	0.942462824
CHRNA4	-0.006	0.853596613	0.942401212
SAR1P3	-0.006	0.853553704	0.942397531
LINC00410	-0.006	0.853527463	0.942397531
PLEKHG4	-0.006	0.853500385	0.942397531
RNA5SP264	-0.006	0.853491262	0.942397531
KCTD9	-0.006	0.853443841	0.942385464
WDR45BP1	-0.006	0.853299038	0.94229111
RP11-399E6	-0.006	0.853124316	0.942185549
EEF1A1P36	-0.006	0.853092363	0.942182078
RP11-108H9	-0.006	0.852954434	0.942182078
MIR508	-0.006	0.852941789	0.942182078
BNIP3P17	-0.006	0.852910479	0.942182078
RP11-618L22	-0.006	0.852902076	0.942182078
TMEM189-UBE2V1	-0.006	0.852871863	0.942182078
TRPC6	-0.006	0.852782073	0.942182078
TRAF2	-0.006	0.852695558	0.942182078
RP11-82L7	-0.006	0.852694973	0.942182078
MID1IP1-AS1	-0.006	0.852674962	0.942182078
GOLGA6D	-0.006	0.852631283	0.942182078
ANKRD30A	-0.006	0.852624331	0.942182078
LINC00838	-0.006	0.852598075	0.942182078
PTCHD1-AS	-0.006	0.852571343	0.942182078
LINC01168	-0.006	0.852523351	0.942182078
ANKRD20A9P	-0.006	0.852509438	0.942182078
KLF3P1	-0.006	0.852489553	0.942182078
MIR96	-0.006	0.852460539	0.942182078
NOS2P2	-0.006	0.85245956	0.942182078
PTEN	-0.006	0.852455296	0.942182078
PTMAP8	-0.006	0.852424014	0.942182078
RNA5SP98	-0.006	0.852253639	0.942119675
MIR518E	-0.006	0.852065906	0.941998224
FAM90A2P	-0.006	0.852030397	0.941998224
LACTB	-0.006	0.852017634	0.941998224
KIAA0125P2	-0.006	0.851983458	0.941998224
RTL1	-0.006	0.851967198	0.941998224
AC008264	-0.006	0.851931512	0.941998224
AP1B1P1	-0.006	0.851805901	0.941998224
RPL21P103	-0.006	0.851767572	0.941997774

MYEOV	-0.006	0.851766491	0.941997774
RNU6-1338P	-0.006	0.851711953	0.941997774
CALM2P4	-0.006	0.851650166	0.941974546
LRRK1	-0.006	0.851512367	0.941868631
RP11-882G5	-0.006	0.851484122	0.941859263
NDUFS2	-0.006	0.851478114	0.941859263
RP11-272D20	-0.006	0.851385712	0.941816029
IGKV2D-19	-0.006	0.851345137	0.941814895
F2	-0.006	0.851340357	0.941814895
RNU6-289P	-0.006	0.851293497	0.941801519
PLBD2	-0.006	0.851263182	0.941789857
NUDT5	-0.006	0.851236209	0.941781893
TTTY1	-0.006	0.851231817	0.941781893
MEG3_1	-0.006	0.851201352	0.941781893
OTOP3	-0.006	0.85114111	0.941742308
RP11-541G7	-0.006	0.851115345	0.941735678
RNU4ATAC18P	-0.006	0.851084012	0.941722887
MAGI2-IT1	-0.006	0.85107498	0.941722887
TBX3	-0.006	0.851054254	0.941722887
RP11-218D6	-0.006	0.850900077	0.941650625
AC091043	-0.006	0.850896844	0.941650625
DEFB110	-0.006	0.85083715	0.941646627
CTD-2328D6	-0.006	0.85075049	0.941594478
EXOC6B	-0.006	0.850724187	0.941587247
MAP3K19	-0.006	0.850671085	0.941550355
FABP5P6	-0.006	0.850653479	0.941550355
METTL13	-0.006	0.850610317	0.941526856
RPS10P3	-0.006	0.850585702	0.941521492
RNU6-731P	-0.006	0.850395943	0.941377082
RP4-789D17	-0.006	0.850336549	0.941355093
GNB5	-0.006	0.850280713	0.941315161
FBXW10	-0.006	0.850274734	0.941315161
SYAP1	-0.006	0.850216936	0.941310196
MRPL51P2	-0.006	0.850210648	0.941310196
AL359314	-0.006	0.850166078	0.941310196
RN7SL279P	-0.006	0.85014221	0.941310196
UBE2D3P2	-0.006	0.850109712	0.941310196
AP000221	-0.006	0.850051649	0.941310196
RPS18P1	-0.006	0.849991999	0.94127997
MIR6851	-0.006	0.849745156	0.941094143
ABL2	-0.006	0.849461097	0.940845182
RPL7P22	-0.006	0.849356038	0.940750698
RP11-154D9	-0.006	0.84932928	0.940742939
RPS3AP14	-0.006	0.849295871	0.940727811
BMPR1B	-0.006	0.849261246	0.940719396
RP1-161C16	-0.006	0.849184671	0.940692151
TTC39CP1	-0.006	0.849115246	0.940659002
REXO1L8P	-0.006	0.848951245	0.940542952
RP11-548N1	-0.006	0.848944507	0.940542952
PRR31	-0.006	0.848808355	0.940450275
STMN1	-0.007	0.848687425	0.940381918
CHRND	-0.007	0.848590932	0.940329677

CRACR2B	-0.007	0.848527831	0.940292584
RNA5SP221	-0.007	0.848466469	0.940246464
ST6GALNAC1	-0.007	0.848416505	0.940234231
RN7SL638P	-0.007	0.848331087	0.940183941
RN7SKP246	-0.007	0.848328307	0.940183941
RP11-190D6	-0.007	0.848315336	0.940183941
RNA5SP388	-0.007	0.848153971	0.940100741
RFT1	-0.007	0.848076892	0.940100741
AC092506	-0.007	0.848029346	0.940100741
PLA2G2F	-0.007	0.847988515	0.940088661
RNU6-793P	-0.007	0.847967583	0.940087339
RP11-472M19	-0.007	0.84795336	0.940087339
SLC7A8	-0.007	0.847951973	0.940087339
ZNF202	-0.007	0.847922883	0.940087339
mascRNA-menRNA	-0.007	0.847916226	0.940087339
AMD1P1	-0.007	0.847816863	0.940073423
HK1	-0.007	0.847673913	0.940034765
PRCP	-0.007	0.84766878	0.940034765
CTAGE6	-0.007	0.847647467	0.940034765
RP11-313A24	-0.007	0.847591875	0.940034765
AC091517	-0.007	0.847535211	0.940034765
RNU6-191P	-0.007	0.847469566	0.940019997
LINC01017	-0.007	0.847453091	0.940019997
WDR11-AS1	-0.007	0.847358821	0.939981333
PIP5KL1	-0.007	0.847345827	0.939981333
CEACAMP8	-0.007	0.847336954	0.939981333
RP3-449O17	-0.007	0.847040007	0.939759002
RP5-988G15	-0.007	0.846976597	0.939710541
NACAP2	-0.007	0.846861597	0.939626728
ENY2	-0.007	0.846832149	0.939615943
TP53TG3D	-0.007	0.846804144	0.939606761
MIR323A	-0.007	0.84630679	0.939339396
ABCB9	-0.007	0.846272353	0.939323064
MYBL2	-0.007	0.846060685	0.939153784
FOXI1	-0.007	0.846035099	0.939147271
AC092265	-0.007	0.845950392	0.939118906
RP11-311B14	-0.007	0.845767545	0.93900347
RP11-1110J8	-0.007	0.845575124	0.938943052
RP11-955H22	-0.007	0.845449656	0.938828799
SLC26A6	-0.007	0.845440713	0.938828799
PCDH11Y	-0.007	0.845370842	0.938828799
RNU4-62P	-0.007	0.845359242	0.938828799
CASP12	-0.007	0.84528307	0.938815744
NLRP8	-0.007	0.845235339	0.938815744
AL133249	-0.007	0.845225631	0.938815744
GSTA3	-0.007	0.845175498	0.938815744
HMCES	-0.007	0.845108105	0.938815744
AC117401	-0.007	0.844857566	0.93862771
RP1-149A16	-0.007	0.844840072	0.93862771
PCDHGB1	-0.007	0.844823996	0.93862771
NADK2	-0.007	0.844691743	0.938531054
YPEL5P3	-0.007	0.844678952	0.938531054

FAM212B	-0.007	0.844654554	0.938531054
AC106706	-0.007	0.844637419	0.938531054
RP11-804H8	-0.007	0.844563081	0.93849757
RABIF	-0.007	0.844542564	0.93849757
MROH1	-0.007	0.844523329	0.93849757
HMGN1P26	-0.007	0.844480867	0.93849757
BCL9L	-0.007	0.84442502	0.938497432
RP11-408H20	-0.007	0.844399318	0.938490766
CTD-2293H3	-0.007	0.844284779	0.938421458
CD164	-0.007	0.84408865	0.938276855
AC138783	-0.007	0.84398103	0.938179123
DUXA	-0.007	0.843964239	0.938179123
RP3-512E2	-0.007	0.843745227	0.937982679
CPSF3L	-0.007	0.843742907	0.937982679
HPCAL4	-0.007	0.843721865	0.937982679
MIR4759	-0.007	0.843721314	0.937982679
CTD-2278B20	-0.007	0.84371838	0.937982679
RN7SL790P	-0.007	0.843676426	0.937982679
RP11-382B18	-0.007	0.843562816	0.937961294
AC114271	-0.007	0.843486503	0.93793588
AC020956	-0.007	0.843419442	0.937883208
PHBP8	-0.007	0.843316864	0.937812932
OR7C1	-0.007	0.843033588	0.937541696
AC023380	-0.007	0.84300098	0.937541696
HINT1P1	-0.007	0.842991503	0.937541696
RP11-164C12	-0.007	0.842988778	0.937541696
OR7E156P	-0.007	0.842969738	0.937541696
RP11-507B12	-0.007	0.842831483	0.937492061
AC103794	-0.007	0.842828056	0.937492061
USP17L13	-0.007	0.842815034	0.937492061
CITF22-62D4	-0.007	0.842808435	0.937492061
AL589863	-0.007	0.842634766	0.937455921
RNU1-93P	-0.007	0.842540555	0.937453079
RPL29P25	-0.007	0.842516838	0.937453079
RP11-748C4	-0.007	0.842425495	0.937412655
AC100756	-0.007	0.842344795	0.937366657
RNU6-474P	-0.007	0.842279171	0.937315531
CASP4	-0.007	0.84226326	0.937315531
C2-AS1	-0.007	0.842229055	0.937315531
RAB11FIP3	-0.007	0.842179104	0.937312146
KRT33A	-0.007	0.842145604	0.937312146
RP4-633H17	-0.007	0.842110684	0.937312146
RP11-436J20	-0.007	0.842081596	0.937312146
CTB-26E19	-0.007	0.842046917	0.937312146
AC003005	-0.007	0.841981727	0.937312146
TIPARP-AS1	-0.007	0.841954839	0.937305014
LINC00237	-0.007	0.841896471	0.937270422
AC007131	-0.007	0.841881044	0.937270422
TMEM253	-0.007	0.841876505	0.937270422
WEE2-AS1	-0.007	0.841856167	0.937270422
PPIHP1	-0.007	0.841836601	0.937270422
DNAJC1	-0.007	0.841757924	0.937270422

RP1-140C12	-0.007	0.84174721	0.937270422
RN7SL795P	-0.007	0.841497468	0.937212074
RP11-597K23	-0.007	0.841452775	0.937184213
RP11-240D10	-0.007	0.84130642	0.93704312
MIR222HG	-0.007	0.841298349	0.93704312
AC092666	-0.007	0.841088288	0.936865894
OR7E117P	-0.007	0.841015979	0.93680726
CRYGGP	-0.007	0.840948664	0.936776097
RP5-906A24	-0.007	0.840803053	0.9366358
USP32P3	-0.007	0.840789867	0.9366358
AC005616	-0.007	0.840759813	0.9366358
HCRTR1	-0.007	0.840715891	0.9366358
GRHL1	-0.007	0.840689797	0.9366358
TREML4	-0.007	0.84068818	0.9366358
CNOT7P1	-0.007	0.840657062	0.9366358
FAIM	-0.007	0.840609518	0.9366358
LMCD1-AS1	-0.007	0.840589021	0.9366358
ABCC2	-0.007	0.840397724	0.936530373
RP11-145E17	-0.007	0.840333453	0.936489574
DDX10	-0.007	0.84023718	0.936443492
LSM3P5	-0.007	0.840211728	0.936440955
CSTA	-0.007	0.840211135	0.936440955
AC005375	-0.007	0.840017352	0.936368034
RNU6-413P	-0.007	0.839929573	0.936319768
ELK3	-0.007	0.839844813	0.936272872
CSHL1	-0.007	0.839824258	0.936272872
RP11-360F5	-0.007	0.839722493	0.936236356
RNU6-684P	-0.007	0.839682162	0.936236356
PMPCAP1	-0.007	0.839680905	0.936236356
RPL12P2	-0.007	0.839605718	0.936221707
PSMC1P10	-0.007	0.839597016	0.936221707
RP11-112N19	-0.007	0.839516385	0.93616593
RP11-167P23	-0.007	0.839480295	0.936149176
DRC7	-0.007	0.839402609	0.936104809
RP11-807E13	-0.007	0.83939308	0.936104809
PMM2	-0.007	0.839260392	0.936033878
NPEPL1	-0.007	0.839250574	0.936033878
EEF1A1P9	-0.007	0.839209169	0.936020587
RP5-875H3	-0.007	0.839193728	0.936020587
KLF4P1	-0.007	0.839051445	0.935888503
RP11-214J9	-0.007	0.838896957	0.935822156
HINT2P1	-0.007	0.838893892	0.935822156
RP11-465I4	-0.007	0.838881115	0.935822156
SSBP2	-0.007	0.838826723	0.935822156
CTD-2194M22	-0.007	0.838793586	0.935822156
RP3-406C18	-0.007	0.838753	0.935818613
TTLL10	-0.007	0.838703639	0.935785461
TMEM17	-0.007	0.838692705	0.935785461
PNMA5	-0.007	0.838652181	0.935771892
PCDH8	-0.007	0.838635825	0.935771892
RSL24D1P2	-0.007	0.838545523	0.935696725
RP11-640I2	-0.007	0.83841905	0.935659096

MAEA	-0.007	0.838349999	0.935631296
C11orf44	-0.007	0.838336241	0.935631296
RP11-291L19	-0.007	0.83831364	0.935631296
RPL5P26	-0.007	0.838179158	0.935507095
LINC01314	-0.007	0.838112711	0.935454854
SERF1AP1	-0.007	0.838036624	0.935413774
MTND2P5	-0.007	0.838013711	0.935410121
SNRPCP13	-0.007	0.837768379	0.935180111
MTND4P25	-0.007	0.837724693	0.935173915
RP11-338N12	-0.007	0.837717435	0.935173915
AC106900	-0.007	0.837643121	0.935149877
RP11-860B13	-0.007	0.837562333	0.935103524
RP4-675C20	-0.007	0.837488566	0.935065005
LINC00550	-0.007	0.837432756	0.935024613
CALM3	-0.007	0.837381369	0.935024613
RN7SL147P	-0.007	0.837364658	0.935024613
RBM15B	-0.007	0.837337424	0.935024613
RPL7AP33	-0.007	0.837331925	0.935024613
RNU6-990P	-0.007	0.837082348	0.934874903
RP11-160N1	-0.007	0.836955812	0.934798895
RP4-635G19	-0.007	0.836942912	0.934798895
MYF5	-0.007	0.836935631	0.934798895
AL357115	-0.007	0.836912076	0.934798895
AIPL1	-0.007	0.836870763	0.934798895
ETF1	-0.007	0.836842542	0.934798895
RP11-458I4	-0.007	0.836823398	0.934798895
ANKRD13B	-0.007	0.836784952	0.934798895
RNU2-63P	-0.007	0.836672864	0.934734889
MLLT4-AS1	-0.007	0.836650152	0.934734889
RNU6-889P	-0.007	0.836630704	0.934734889
LINC01372	-0.007	0.836512584	0.934632624
CHRNA7	-0.007	0.83638046	0.934569478
RNU6-543P	-0.007	0.836122822	0.93442483
RN7SKP106	-0.007	0.836085535	0.93442483
ZNF90	-0.007	0.836015038	0.93442483
RAVER1	-0.007	0.836006508	0.93442483
RPL5P4	-0.007	0.835978786	0.93442483
AC097721	-0.007	0.835974521	0.93442483
RP11-315I20	-0.007	0.835963005	0.93442483
RNA5SP455	-0.007	0.835892484	0.93442483
RN7SL718P	-0.007	0.835789859	0.93442483
GSTO3P	-0.007	0.83575098	0.93442483
RP11-463H24	-0.007	0.835689605	0.93442483
KDELC1P1	-0.007	0.835685099	0.93442483
RP11-739N20	-0.007	0.835603183	0.934405691
UGT2A2	-0.007	0.835519539	0.934361208
RP11-369K17	-0.007	0.835387445	0.934293848
AL132640	-0.007	0.835359143	0.93428635
FRAS1	-0.007	0.835345064	0.93428635
DUSP4	-0.007	0.835331418	0.93428635
CTD-2249K22	-0.007	0.835198284	0.934194192
SPTLC2	-0.007	0.835190701	0.934194192

RNA5SP278	-0.007	0.835094816	0.934144275
MIR571	-0.007	0.835064022	0.934144275
SCAANT1	-0.007	0.835054419	0.934144275
MIR4636	-0.007	0.835005703	0.934144275
EID3	-0.007	0.834983472	0.934144275
ITM2C	-0.007	0.834886258	0.934106526
RNU6-233P	-0.007	0.83488621	0.934106526
FAM86KP	-0.007	0.834769213	0.934106526
LINC01339	-0.007	0.834725671	0.934106526
TTTY11	-0.007	0.834711029	0.934106526
MTND4LP1	-0.007	0.834631283	0.934106526
HIST1H2AB	-0.007	0.834559596	0.934106526
RP11-20G13	-0.007	0.834414364	0.933990581
RP11-113D19	-0.007	0.834389836	0.933990581
OR11I1P	-0.007	0.834362814	0.933990581
AC004520	-0.007	0.834322758	0.933982606
TXNP7	-0.007	0.834311965	0.933982606
CTD-2376I4	-0.007	0.8342297	0.933944288
RNU4-60P	-0.007	0.834192647	0.933924759
AC010480	-0.007	0.834136171	0.933883484
RP5-827C21	-0.007	0.834065152	0.933825924
UBE2CP3	-0.007	0.833882882	0.933745254
IFITM1	-0.007	0.833843493	0.933745254
RP11-316A16	-0.007	0.833758272	0.933723787
BTF3P12	-0.007	0.833701137	0.933698929
CCSER1	-0.007	0.833620142	0.933678868
FLJ44511	-0.007	0.833619273	0.933678868
NDUFS5P6	-0.007	0.833496262	0.933584029
PRDM14	-0.007	0.833463471	0.933569255
NPNT	-0.007	0.83345008	0.933569255
BCO2	-0.007	0.833393821	0.93353515
KCNJ5	-0.007	0.833275339	0.933433395
KRTAP12-4	-0.007	0.833275322	0.933433395
RP11-222A5	-0.007	0.833224893	0.933433395
RP11-737F9	-0.007	0.833189447	0.933422448
ABHD17AP6	-0.007	0.833189272	0.933422448
RN7SKP55	-0.007	0.833083814	0.933422448
GAPDHP31	-0.007	0.832983904	0.933369157
RP11-157P23	-0.007	0.832969258	0.933369157
MIR4801	-0.007	0.832811981	0.93330046
RP11-18B16	-0.007	0.832799294	0.93330046
MIR34A	-0.007	0.832662367	0.933260286
SPTBN5	-0.007	0.832659119	0.933260286
MKLN1-AS	-0.007	0.832567505	0.933223146
RP11-137H2	-0.007	0.832565706	0.933223146
CD68	-0.007	0.832484415	0.933196865
MAN1A1	-0.007	0.832460979	0.933196865
MRPL42P6	-0.007	0.832370721	0.933163153
ZNF496	-0.007	0.832360422	0.933163153
RP11-177F11	-0.007	0.832285757	0.933163153
AL022393	-0.007	0.832192586	0.933125342
RP11-477J21	-0.007	0.832127019	0.933103702

RNU6-1261P	-0.007	0.832091175	0.933103702
MIR6082	-0.007	0.832043333	0.933103702
KARSP3	-0.007	0.831913657	0.93301822
CCNG2P1	-0.007	0.831905788	0.93301822
AC132192	-0.007	0.831883023	0.93301822
ANKRD20A3	-0.007	0.831840239	0.93301822
CICP20	-0.007	0.831756646	0.932973946
RP11-70K10	-0.007	0.831748292	0.932973946
MIR4452	-0.007	0.831724376	0.932973946
MTHFS	-0.007	0.831585456	0.93289178
RP11-322J23	-0.007	0.831349374	0.932707457
RN7SL864P	-0.007	0.831342257	0.932707457
DAB1	-0.007	0.831276345	0.932703487
RNU7-12P	-0.007	0.831273706	0.932703487
RP5-1027O11	-0.007	0.831255952	0.932703487
RP11-531F16	-0.007	0.831192281	0.932703487
AGL	-0.007	0.831075576	0.932605354
RN7SL335P	-0.007	0.830677221	0.932356044
RP11-307O10	-0.007	0.83062621	0.932342733
RNU6-480P	-0.007	0.83055298	0.932326454
GNG5P1	-0.007	0.83053902	0.932326454
AC099050	-0.007	0.83043961	0.932275182
MIR494	-0.007	0.830365	0.932247274
RNU6-1155P	-0.007	0.830363032	0.932247274
RP11-677O4	-0.007	0.830310219	0.932247274
Z82205	-0.007	0.830243287	0.932220502
RN7SKP212	-0.007	0.830127812	0.932134794
NEO1	-0.007	0.830091371	0.932128478
CTC-260E6	-0.007	0.830047512	0.932128478
RP11-284A20	-0.007	0.82998088	0.932123641
EIF2S2P5	-0.007	0.829950887	0.932123641
FGFR3P5	-0.007	0.829879276	0.932123641
HMGB1P25	-0.007	0.829779778	0.932123641
LA16c-359F1	-0.007	0.82977841	0.932123641
MUM1	-0.007	0.829762226	0.932123641
PGAM1P10	-0.007	0.829738102	0.932123641
NGRN	-0.007	0.829564243	0.932095315
HOXB-AS4	-0.007	0.829520738	0.932084207
RP11-140C18	-0.007	0.829501927	0.932084207
HIST1H2AI	-0.007	0.829471523	0.932079092
RNU7-6P	-0.007	0.829463987	0.932079092
DNAJA1P5	-0.007	0.829348639	0.932014001
RP11-2L13	-0.007	0.829295934	0.932014001
GRM7-AS3	-0.007	0.829284521	0.932014001
CTD-2015C24	-0.007	0.829257972	0.932014001
EIF4A2P1	-0.007	0.829201356	0.932004819
NSMCE2	-0.007	0.829132549	0.931962048
RP11-650L12	-0.007	0.82908721	0.931955076
LRRC30	-0.007	0.828962561	0.931858947
TNS2	-0.007	0.828950776	0.931858947
RP11-211G23	-0.007	0.82894492	0.931858947
RP11-384G23	-0.007	0.828937208	0.931858947

RNA5SP59	-0.007	0.828818873	0.931851384
GCSH	-0.007	0.828735385	0.93180151
Z97632	-0.007	0.828646764	0.93174586
CTD-2358C21	-0.007	0.828642412	0.93174586
RPL35AP4	-0.007	0.828552849	0.931684253
COX17	-0.007	0.828465526	0.931608056
RP11-186F10	-0.007	0.828422395	0.931608056
DDR1-AS1	-0.007	0.82837827	0.931608056
AC117481	-0.007	0.828336666	0.931595126
RP11-315O8	-0.007	0.828233886	0.931530079
ATP6V1B1	-0.007	0.828044602	0.931376621
EIF4A1P10	-0.007	0.828041198	0.931376621
HMGCR	-0.007	0.827862033	0.931303235
RP11-231G3	-0.007	0.82777522	0.93122757
MS4A18	-0.007	0.827753137	0.93122757
CYP2G2P	-0.007	0.827743322	0.93122757
MAK	-0.007	0.827736496	0.93122757
RP11-266N13	-0.007	0.827692048	0.93122757
AL590113	-0.007	0.827614816	0.93122757
RP1-278D1	-0.007	0.827570608	0.93122757
GAN	-0.007	0.827416076	0.931200903
TGM3	-0.007	0.827377608	0.931198179
AC018730	-0.007	0.827288093	0.931119435
VN1R53P	-0.007	0.827165232	0.93104716
OR14C36	-0.007	0.827125766	0.931025783
AP001046	-0.007	0.826991447	0.930962544
RNU6-143P	-0.007	0.826761552	0.930818211
AC107622	-0.007	0.826512783	0.930674845
MRPS18CP6	-0.007	0.826496842	0.930674845
IGLVIV-59	-0.007	0.826385649	0.930674845
KRT18P21	-0.007	0.826342476	0.930670949
CMTM8	-0.007	0.826188704	0.930519767
SAMD11P1	-0.007	0.82618491	0.930519767
XX-FW83128A1	-0.007	0.826171072	0.930519767
RNU6-562P	-0.007	0.826059913	0.930484733
RP11-23E19	-0.007	0.825890236	0.930462009
HMGB3P16	-0.007	0.825856828	0.930454029
MTND3P7	-0.008	0.82574574	0.930371222
IGBP1-AS2	-0.008	0.825679143	0.930371222
RPL21P47	-0.008	0.825664468	0.930371222
CTB-57H20	-0.008	0.825519957	0.930316572
RN7SL570P	-0.008	0.825314216	0.930150741
DOC2A	-0.008	0.825304067	0.930150741
RPL15P4	-0.008	0.825185743	0.930147273
AC013442	-0.008	0.825156917	0.930147273
WSCD2	-0.008	0.825100671	0.930108157
GIMD1	-0.008	0.825067032	0.930092251
CD70	-0.008	0.825004249	0.93004349
CH507-154B10	-0.008	0.824871198	0.929981549
FOSB	-0.008	0.82486821	0.929981549
RP11-298A8	-0.008	0.824850455	0.929981549
AL118525	-0.008	0.824766387	0.929951437

NTF6A	-0.008	0.824742769	0.929946823
PCTP	-0.008	0.82466327	0.92992323
SGCG	-0.008	0.824642312	0.92992323
IFT27	-0.008	0.824631382	0.92992323
CTD-2184D3	-0.008	0.824618494	0.92992323
RN7SL370P	-0.008	0.824593661	0.92992323
SERBP1P3	-0.008	0.824412134	0.929860198
HMGN1P5	-0.008	0.824352247	0.929814671
GUSBP6	-0.008	0.824315701	0.929795469
KRT18P15	-0.008	0.824273507	0.929769896
RP11-502F1	-0.008	0.824271853	0.929769896
RP11-15B17	-0.008	0.824260538	0.929769896
GXYLT1P6	-0.008	0.824159718	0.929729622
HRASLS2	-0.008	0.82402854	0.929630035
AC097493	-0.008	0.823979893	0.929614838
BCR	-0.008	0.823968177	0.929614838
AC018685	-0.008	0.823881899	0.929608425
HOOK3	-0.008	0.823852554	0.929603297
RP11-340C20	-0.008	0.823833273	0.929603297
RPL12P24	-0.008	0.82363456	0.929445402
LINC00896	-0.008	0.823608545	0.929443157
AC006942	-0.008	0.823570298	0.92943895
CUL4B	-0.008	0.823557562	0.92943895
CCDC58P3	-0.008	0.823524179	0.92943895
CTD-2501M5	-0.008	0.823513256	0.92943895
NXPE1	-0.008	0.823429114	0.929411755
AC008410	-0.008	0.823408337	0.92941033
AC018462	-0.008	0.82338782	0.929409198
BSPH1	-0.008	0.823382256	0.929409198
RP11-209A2	-0.008	0.823361003	0.929409198
MLLT10P2	-0.008	0.823267995	0.929384077
RP11-431J24	-0.008	0.823255986	0.929384077
OR1P1	-0.008	0.823240358	0.929384077
AK4P1	-0.008	0.82318891	0.929360884
TTTY21	-0.008	0.823091935	0.929279072
RP11-100G15	-0.008	0.82308616	0.929279072
MPHOSPH6	-0.008	0.822997403	0.929279072
CU013544	-0.008	0.822956373	0.929274579
AC006840	-0.008	0.82293281	0.929274579
MAPT	-0.008	0.822928558	0.929274579
RP11-802D6	-0.008	0.82291468	0.929274579
CHRNB3	-0.008	0.822790219	0.929207926
ANKS4B	-0.008	0.822779783	0.929207926
PSMB11	-0.008	0.822746309	0.929207926
MIR302A	-0.008	0.822573231	0.92911308
KRTAP12-3	-0.008	0.822467606	0.929075107
EEF1A1P16	-0.008	0.822463464	0.929075107
ASS1P7	-0.008	0.822447884	0.929075107
CHRNA9	-0.008	0.822414673	0.929075107
RP11-787D11	-0.008	0.822224022	0.929039806
CTD-2589M5	-0.008	0.822145441	0.9289944
CH17-248H7	-0.008	0.821847727	0.928749359

SEPT7P4	-0.008	0.821804045	0.92872203
RP11-667K14	-0.008	0.821768597	0.928704007
KRT8P29	-0.008	0.821749898	0.928704007
AC111200	-0.008	0.821720072	0.928704007
GM2A	-0.008	0.821677261	0.928688932
AC142293	-0.008	0.821661027	0.928688932
MIR941-5	-0.008	0.821640083	0.928688932
RNU6-111P	-0.008	0.821623821	0.928688932
CTD-2534J5	-0.008	0.821606672	0.928688932
CTD-2014D20	-0.008	0.821601317	0.928688932
DNAJB5-AS1	-0.008	0.821456901	0.928598806
LINC00680	-0.008	0.821446171	0.928598806
SKP1	-0.008	0.821421959	0.928598806
AC005513	-0.008	0.8213168	0.928567992
TRIM68	-0.008	0.821058366	0.928363962
AL355795	-0.008	0.820978861	0.928318144
MTHFD1P1	-0.008	0.82096001	0.928318144
LINC00599	-0.008	0.820932182	0.928318144
CLCNKA	-0.008	0.820913041	0.928318144
SOCS5P2	-0.008	0.820871222	0.928318144
MIR544A	-0.008	0.82080117	0.928318144
VWA8P1	-0.008	0.820790393	0.928318144
RPL21P116	-0.008	0.820790118	0.928318144
VCX3B	-0.008	0.820771394	0.928318144
CTAGE14P	-0.008	0.820626684	0.928318144
RPS18P6	-0.008	0.82056474	0.928318144
SDCCAG3	-0.008	0.820437459	0.928278967
AP1M2	-0.008	0.820323652	0.928187626
AC005758	-0.008	0.820286784	0.928174638
VTRNA3-1P	-0.008	0.820276076	0.928174638
RNU6-542P	-0.008	0.820250552	0.928174638
AC013727	-0.008	0.820238978	0.928174638
RP11-94P11	-0.008	0.820196842	0.928174638
TEKT4	-0.008	0.82017771	0.928174638
SCGB1D2	-0.008	0.820047611	0.928146568
RP11-179G8	-0.008	0.820034567	0.928146568
TAGLN3	-0.008	0.820002761	0.928139918
LINC00639	-0.008	0.819945723	0.928111371
AC010642	-0.008	0.819716888	0.927904555
TTC36	-0.008	0.81969181	0.927898221
PNPLA6	-0.008	0.819654053	0.927898221
OR10J6P	-0.008	0.81960896	0.927870596
OR5G3	-0.008	0.819532406	0.927864167
DHFRP1	-0.008	0.819443553	0.927815664
SNHG10	-0.008	0.819333948	0.927735675
RP11-466C23	-0.008	0.819308385	0.927732255
ACTR1B	-0.008	0.819148013	0.927591298
NXPE2	-0.008	0.819073605	0.927551147
RP5-890O15	-0.008	0.819035244	0.92752976
RNU6-1336P	-0.008	0.819023601	0.92752976
RP11-273P3	-0.008	0.819016042	0.92752976
OR8F1P	-0.008	0.818872187	0.927433312

STK35	-0.008	0.818829229	0.927406713
PARP10	-0.008	0.818797819	0.927403288
RP11-788M5	-0.008	0.818741911	0.927387303
ALAD	-0.008	0.818645443	0.927374986
MESTP4	-0.008	0.818635246	0.927374986
RP5-862P8	-0.008	0.81856332	0.927326068
RPS24P6	-0.008	0.818379771	0.927184295
RNA5SP331	-0.008	0.818285486	0.927121582
RP11-339A7	-0.008	0.818257133	0.927111514
MRPL50P2	-0.008	0.818179352	0.927087394
RP11-294O2	-0.008	0.817968701	0.926954105
TCEB1P14	-0.008	0.817825676	0.926887264
AC009127	-0.008	0.817825676	0.926887264
RP5-855F14	-0.008	0.817716352	0.926887264
AC096570	-0.008	0.81743762	0.926653073
AP000946	-0.008	0.817424822	0.926653073
RNU6ATAC33P	-0.008	0.817359167	0.926645205
SAGE3P	-0.008	0.817357833	0.926645205
USP35	-0.008	0.816929884	0.926312872
RP11-235P11	-0.008	0.816839664	0.926312872
RP11-54O15	-0.008	0.816664284	0.92616606
AGAP10	-0.008	0.816640153	0.926165307
RPS6KB2	-0.008	0.816638881	0.926165307
RNU7-9P	-0.008	0.816616314	0.926165307
TRIM49	-0.008	0.816590213	0.926165307
MXD3	-0.008	0.816578647	0.926165307
RP5-892G5	-0.008	0.816577558	0.926165307
RP11-429J17	-0.008	0.81644249	0.926157115
COX14	-0.008	0.816273254	0.926031306
UBE2CP4	-0.008	0.816271114	0.926031306
KHSRP	-0.008	0.816261379	0.926031306
TP53TG3	-0.008	0.816179884	0.925991551
PNRC1	-0.008	0.816136685	0.925991551
RNA5SP243	-0.008	0.81610897	0.925991551
ANKRD33B-AS1	-0.008	0.816107555	0.925991551
BNIP3P32	-0.008	0.816080365	0.925991551
RNU6-161P	-0.008	0.816070446	0.925991551
AUTS2	-0.008	0.816031929	0.925991551
CEP57L1P1	-0.008	0.815973934	0.925978473
RPS7P2	-0.008	0.815924751	0.925954663
AC069276	-0.008	0.815921777	0.925954663
RP11-125N22	-0.008	0.81577919	0.925899214
FRG2C	-0.008	0.815647381	0.925850561
CTC-425K20	-0.008	0.815437153	0.925700179
UBE2L2	-0.008	0.815327202	0.925597422
RNY1P1	-0.008	0.815273803	0.925580926
ACSM3	-0.008	0.815256328	0.925580926
COPRS	-0.008	0.815243607	0.925580926
FXR2	-0.008	0.815051114	0.92546047
RNA5SP129	-0.008	0.814978674	0.925422342
AC011525	-0.008	0.814948009	0.925409585
PVRL3-AS1	-0.008	0.814773801	0.925322074

OTOP2	-0.008	0.814764214	0.925322074
TCTN2	-0.008	0.814761547	0.925322074
RP3-525L6	-0.008	0.814752083	0.925322074
BBX	-0.008	0.814577983	0.925276196
TMX2-CTNND1	-0.008	0.814574091	0.925276196
MAPRE1	-0.008	0.814548309	0.925276196
CTD-3148I10	-0.008	0.814432489	0.925177125
FAM27B	-0.008	0.814318794	0.925114166
SEC63	-0.008	0.814253699	0.925073317
RN7SL672P	-0.008	0.814214204	0.925061543
CXCR2	-0.008	0.814092387	0.925011407
RP11-1D12	-0.008	0.814058349	0.924994799
RP11-675P14	-0.008	0.81392309	0.924907306
MIR1277	-0.008	0.813862434	0.924860446
AP002958	-0.008	0.813830688	0.924846437
RP11-300G22	-0.008	0.813767676	0.924839103
RYBP	-0.008	0.813762035	0.924839103
RP11-575N15	-0.008	0.813745218	0.924839103
AC027238	-0.008	0.813646971	0.924814192
AC008626	-0.008	0.813642059	0.924814192
RP11-42O4	-0.008	0.813489708	0.924767861
SNRPN	-0.008	0.81342427	0.924737616
MINOS1	-0.008	0.813314938	0.924635394
MYH11	-0.008	0.81327341	0.924610252
SULT1A1	-0.008	0.813214932	0.924565839
EEF1A1P28	-0.008	0.813068685	0.924465772
ECEL1P2	-0.008	0.813057309	0.924465772
RP11-686G23	-0.008	0.813055328	0.924465772
LINC01304	-0.008	0.813050782	0.924465772
RP5-1172N10	-0.008	0.813021351	0.924465772
LINC00536	-0.008	0.813018365	0.924465772
ZW10	-0.008	0.812992473	0.924465772
RP4-613B23	-0.008	0.812990769	0.924465772
C6orf25	-0.008	0.812983998	0.924465772
OR13F1	-0.008	0.812899491	0.924465772
OR4A5	-0.008	0.812878915	0.924465772
AMN	-0.008	0.812850665	0.924465772
C20orf196	-0.008	0.81283909	0.924465772
RP11-774O3	-0.008	0.812753928	0.924465772
PCDHA2	-0.008	0.812654103	0.924410658
RNU6-555P	-0.008	0.812631029	0.924409526
TBX6	-0.008	0.812622164	0.924409526
RNU6-728P	-0.008	0.812363232	0.924244323
HMGXB3	-0.008	0.812334081	0.924244323
LA16c-380A1	-0.008	0.81233364	0.924244323
ZDHHC20P3	-0.008	0.812270039	0.924244323
GCSAML-AS1	-0.008	0.812266085	0.924244323
CADM2-AS2	-0.008	0.812168724	0.924244323
AL136446	-0.008	0.812155231	0.924244323
ISM1-AS1	-0.008	0.812126229	0.924244323
CSNK1A1	-0.008	0.812125236	0.924244323
BTBD7P1	-0.008	0.812092727	0.924244323

USP31	-0.008	0.812048564	0.92423259
EIF3LP3	-0.008	0.812043774	0.92423259
RNU6-831P	-0.008	0.812024424	0.92423259
NRIP3	-0.008	0.812005782	0.92423259
TIMM9	-0.008	0.811971471	0.92423259
PRSS46	-0.008	0.811815089	0.924143535
C2orf66	-0.008	0.811814881	0.924143535
CPNE4	-0.008	0.811691474	0.924069077
PTP4A1P6	-0.008	0.811688962	0.924069077
KRTAP19-10P	-0.008	0.81160606	0.9240381
RNA5S17	-0.008	0.81160606	0.9240381
RP11-167N24	-0.008	0.811581586	0.9240381
APLNR	-0.008	0.811575442	0.9240381
B3GALT4	-0.008	0.811389515	0.923990351
LINC01072	-0.008	0.811376644	0.923990351
ATP5EP1	-0.008	0.811367562	0.923990351
FCF1P10	-0.008	0.811361178	0.923990351
COX6B1P3	-0.008	0.811351731	0.923990351
SAP30BP	-0.008	0.811307355	0.923990351
MIR3179-4	-0.008	0.811302823	0.923990351
RNA5SP283	-0.008	0.811298606	0.923990351
KTN1-AS1	-0.008	0.811289992	0.923990351
NDUFB3	-0.008	0.811285757	0.923990351
SUB1P2	-0.008	0.811261907	0.923990351
ZSCAN10	-0.008	0.811137767	0.923990351
GPC3	-0.008	0.811087143	0.923990351
RP11-314A20	-0.008	0.811047337	0.923990351
ADH1B	-0.008	0.810996896	0.923990351
AL109761	-0.008	0.81098219	0.923990351
RPL7P39	-0.008	0.810962564	0.923990351
RP4-597J3	-0.008	0.810919785	0.923990351
RNU6-557P	-0.008	0.810867092	0.923990351
MTND2P24	-0.008	0.810862336	0.923990351
RP11-346D14	-0.008	0.810818933	0.923981273
DHX34	-0.008	0.810784375	0.923981273
PCCA-AS1	-0.008	0.810589122	0.923896236
ZIC2	-0.008	0.81030611	0.923699345
GPX2	-0.008	0.810223588	0.923634333
RP11-389G6	-0.008	0.810203624	0.923634333
RP11-351O2	-0.008	0.810153514	0.923634333
HMGN3-AS1	-0.008	0.810107802	0.923634333
ANP32D	-0.008	0.810100088	0.923634333
RP11-466A19	-0.008	0.810032448	0.923593292
RP11-958J22	-0.008	0.809966147	0.923561916
NPM1P42	-0.008	0.809946917	0.923561916
ONECUT3	-0.008	0.809870372	0.923499559
RPS27AP14	-0.008	0.809859963	0.923499559
SCARNA21	-0.008	0.809839633	0.923499559
RARG	-0.008	0.809816132	0.923499559
BOLA3P1	-0.008	0.809718881	0.923469228
CDHR2	-0.008	0.809626074	0.923461563
PKD1P5	-0.008	0.809594107	0.923447217

TTTY7	-0.008	0.809520299	0.923447217
PABPC1	-0.008	0.809496553	0.923447217
RN7SL5P	-0.008	0.809476109	0.923447217
HNRNPA1P57	-0.008	0.80946492	0.923447217
RN7SL789P	-0.008	0.809398322	0.923447217
CTB-22K14	-0.008	0.809304696	0.923404599
RP11-34D15	-0.008	0.80925277	0.923367472
LBX2	-0.008	0.809179121	0.923305558
BNIP3P13	-0.008	0.80903885	0.923189739
KRTAP19-8	-0.008	0.809038837	0.923189739
RP11-35O7	-0.008	0.809017075	0.923189739
SLC7A3	-0.008	0.808976341	0.923189739
AC124853	-0.008	0.808739371	0.923024923
AC096546	-0.008	0.808721211	0.923024923
AC108025	-0.008	0.808702967	0.923024923
LINC00269	-0.008	0.808684912	0.923024923
CNTFR	-0.008	0.808589156	0.922986188
CIDECP	-0.008	0.808483421	0.922953976
HNRNPA1P18	-0.008	0.808439156	0.922947689
AC108448	-0.008	0.80833778	0.922876197
KRT18P10	-0.008	0.808267631	0.92281823
AC005540	-0.008	0.808158655	0.922755589
OR3D1P	-0.008	0.808051273	0.922703924
AC068138	-0.008	0.808045867	0.922703924
AL161643	-0.008	0.807845317	0.922601465
HMGA1P7	-0.008	0.807812575	0.922586195
AC018832	-0.008	0.807802448	0.922586195
MPL	-0.008	0.807664665	0.92246151
RP11-340A13	-0.008	0.807562187	0.922366586
PLCXD1	-0.008	0.80755817	0.922366586
AC074008	-0.008	0.807511506	0.922366586
RP11-425P5	-0.008	0.807409705	0.922303017
OPRD1	-0.008	0.807392779	0.922303017
AC079834	-0.008	0.807320997	0.922245926
RP11-468D11	-0.008	0.807279374	0.922220498
RP5-1069C8	-0.008	0.807242506	0.922220498
PRR13P7	-0.008	0.807207402	0.922220498
ACSL5	-0.008	0.807112352	0.922162409
RNU6-700P	-0.008	0.806981079	0.922043909
AL161652	-0.008	0.806825284	0.921982683
RP11-102D18	-0.008	0.806801212	0.921982683
CYCSP19	-0.008	0.806644236	0.92198145
MIR644A	-0.008	0.806601617	0.92196816
RN7SL690P	-0.008	0.806477748	0.921901779
RP11-299M14	-0.008	0.806457405	0.921900651
RNU7-69P	-0.008	0.806425924	0.921892575
AC087491	-0.008	0.806096865	0.92164335
TIPARP	-0.008	0.805937622	0.921549772
PSAP	-0.008	0.805887733	0.921536976
RP11-379I19	-0.008	0.805846236	0.921525326
SLC10A6	-0.008	0.805838226	0.921525326
RP11-550A9	-0.008	0.805819823	0.921525326

CTD-2330K9	-0.008	0.805671244	0.921466413
RPS3AP42	-0.008	0.805560443	0.921413402
NTN3	-0.008	0.805472586	0.921394088
PLA2G12AP1	-0.008	0.805451092	0.921391631
SLC25A14	-0.008	0.805374515	0.92134829
AC008265	-0.008	0.805227797	0.921268961
RNA5SP120	-0.008	0.805217602	0.921268961
BX470102	-0.008	0.805175102	0.921268961
AP004372	-0.008	0.805174258	0.921268961
BMS1P11	-0.008	0.805173701	0.921268961
RPL5P19	-0.008	0.805089369	0.9212655
CACNB4	-0.008	0.805027907	0.921261573
DKK4	-0.008	0.804979567	0.921261573
ZNF790-AS1	-0.008	0.804926891	0.921261573
CTD-2071N1	-0.008	0.804923141	0.921261573
RPS3AP12	-0.008	0.804820471	0.921240719
USP32P2	-0.008	0.804735558	0.921214768
SREBF1	-0.008	0.804683028	0.921176776
PLEKHA1	-0.008	0.804663588	0.921176776
SHROOM3	-0.008	0.804538495	0.92112202
RNU6ATAC20P	-0.008	0.804485408	0.921105525
ARF4P1	-0.008	0.804422533	0.921077822
RP11-841C19	-0.008	0.804368911	0.921038567
AUP1	-0.008	0.804341991	0.921029885
TBC1D3P5	-0.008	0.804293754	0.920996793
LINC01088	-0.008	0.803992945	0.92080731
RP11-397D12	-0.008	0.803954092	0.92080731
ESYT1	-0.008	0.803703121	0.920586064
SPEF1	-0.008	0.803663407	0.920562714
TTTY2B	-0.008	0.80366231	0.920562714
LIPE	-0.008	0.803578489	0.920531862
LINC00694	-0.008	0.803554123	0.920526091
F2R	-0.008	0.803492728	0.920477899
RP11-3J11	-0.008	0.803379911	0.920370794
PDHA2	-0.008	0.803369273	0.920370794
MIR6803	-0.008	0.803347044	0.920370794
MAST4-IT1	-0.008	0.803342751	0.920370794
NUBP1	-0.008	0.803289531	0.920370794
RP4-810F7	-0.008	0.803266885	0.920370794
WI2-1528O10	-0.008	0.803244256	0.920370794
KRT8P27	-0.008	0.803232054	0.920370794
MTND2P18	-0.008	0.803075212	0.920289003
SCOCP1	-0.008	0.80305615	0.920289003
SIGLEC20P	-0.008	0.803043348	0.920289003
AL391650	-0.008	0.803030334	0.920289003
YBX1P3	-0.008	0.803021553	0.920289003
CDC42P1	-0.009	0.802962496	0.920289003
RP5-826L7	-0.009	0.802925684	0.920289003
F11-AS1	-0.009	0.802706667	0.920128867
SPIDR	-0.009	0.802672417	0.920128109
ACOT7	-0.009	0.802649188	0.920128109
RP11-351O1	-0.009	0.802564092	0.920122286

HTR3E	-0.009	0.802525355	0.920122173
TMEM161B-AS1	-0.009	0.802457871	0.920075665
SMIM2	-0.009	0.802370267	0.920055103
SERPINE4P	-0.009	0.802333491	0.920040568
BCORP1	-0.009	0.802263099	0.920033602
DIXDC1	-0.009	0.802221802	0.920022124
RN7SL735P	-0.009	0.802162775	0.920022124
RP11-468N14	-0.009	0.80211276	0.920022124
ELL2P2	-0.009	0.80204718	0.920016885
C22orf15	-0.009	0.801833195	0.919904359
RP11-238I10	-0.009	0.801810993	0.919904359
LINC01450	-0.009	0.801795765	0.919904359
RP5-1181K21	-0.009	0.801698754	0.919838751
EEF1A1	-0.009	0.801616723	0.919788948
RP11-208N14	-0.009	0.801612053	0.919788948
TRIM32	-0.009	0.801547904	0.919788948
PTCSC3	-0.009	0.801526436	0.919788948
MIR4260	-0.009	0.801519888	0.919788948
CTD-2165H16	-0.009	0.80137924	0.919715875
KCNIP4-IT1	-0.009	0.801338031	0.919690742
AC007322	-0.009	0.801298512	0.919690742
LINC00904	-0.009	0.801159628	0.919620754
SNHG21	-0.009	0.801156975	0.919620754
ASB9P1	-0.009	0.801107369	0.919620754
RN7SL491P	-0.009	0.801031198	0.919604514
RNU6-317P	-0.009	0.801024466	0.919604514
KCNAB1-AS1	-0.009	0.80078395	0.919409314
LINC01016	-0.009	0.800728634	0.919398926
IMPDH1	-0.009	0.800720511	0.919398926
SYF2P2	-0.009	0.800711018	0.919398926
SAGE4P	-0.009	0.80065177	0.919398926
RP11-616K6	-0.009	0.800603836	0.919379826
RP11-1G11	-0.009	0.80058841	0.919379826
HNRNPCL2	-0.009	0.800577974	0.919379826
LINC01273	-0.009	0.800555712	0.919379826
KRTAP9-8	-0.009	0.800535153	0.919379826
DNAJB13	-0.009	0.800479316	0.919347666
ULK1	-0.009	0.800351633	0.919296125
XKR4	-0.009	0.800260237	0.919229055
OR51I2	-0.009	0.800224697	0.919229055
MIR563	-0.009	0.800163324	0.919229055
RNU6ATAC27P	-0.009	0.800116765	0.919229055
PMS2P11	-0.009	0.800083459	0.919225525
RP11-629E24	-0.009	0.799876138	0.919098189
CDK5	-0.009	0.799857474	0.919098189
RP11-541H12	-0.009	0.799839988	0.919098189
CD34	-0.009	0.799800931	0.919078294
CTB-161J7	-0.009	0.799748681	0.919040425
RNU6-1263P	-0.009	0.799736191	0.919040425
FOXP4-AS1	-0.009	0.799684474	0.91902171
RP11-467L13	-0.009	0.799672238	0.91902171
RP5-1066H13	-0.009	0.799443339	0.918931507

RP11-312B8	-0.009	0.799380894	0.918906011
RNU6-1104P	-0.009	0.799357233	0.918900991
OR4B2P	-0.009	0.799249329	0.918799126
DSTNP2	-0.009	0.799195063	0.918767743
AC078852	-0.009	0.799187688	0.918767743
IFNWP18	-0.009	0.799078934	0.918714119
AC098828	-0.009	0.799066062	0.918714119
AC079807	-0.009	0.799004383	0.918672761
PNPLA4	-0.009	0.798957559	0.9186411
LINC00239	-0.009	0.798884457	0.918611242
CSPG4P1Y	-0.009	0.798856841	0.918611242
UQCRBP2	-0.009	0.7986632	0.918524395
LINC01296	-0.009	0.798660126	0.918524395
ZYG11A	-0.009	0.798639087	0.918524395
RP11-171A24	-0.009	0.798638446	0.918524395
FLJ21408	-0.009	0.79862559	0.918524395
RP11-402F9	-0.009	0.798606339	0.918524395
RP11-484N16	-0.009	0.798447782	0.918524395
RN7SL720P	-0.009	0.798375495	0.918510314
PSMG3-AS1	-0.009	0.79833288	0.918510314
RP11-112L18	-0.009	0.798171498	0.918446821
RP11-302I18	-0.009	0.798153126	0.918446821
APEH	-0.009	0.798076887	0.918404521
RP11-744N12	-0.009	0.797958843	0.918290866
RP11-50C13	-0.009	0.797936459	0.918287296
TAB1	-0.009	0.797920856	0.918287296
AP000704	-0.009	0.797892599	0.918287296
RPS3AP53	-0.009	0.797877442	0.918287296
RHOT1P2	-0.009	0.797781814	0.918287296
FAM71F1	-0.009	0.797472679	0.918135632
ZNF299P	-0.009	0.797470579	0.918135632
RP11-521C22	-0.009	0.797343612	0.918093083
BSN-AS1	-0.009	0.797320636	0.918088824
RPL7AP8	-0.009	0.797273267	0.918078672
RP11-332M2	-0.009	0.797129861	0.917996297
CH17-431G21	-0.009	0.797128593	0.917996297
RP11-797H7	-0.009	0.797123534	0.917996297
BOLA3P3	-0.009	0.797110623	0.917996297
PDCD10	-0.009	0.797063674	0.917996297
LL09NC01-254D11	-0.009	0.797005137	0.917996297
RP11-143E21	-0.009	0.797001837	0.917996297
RNU6-1126P	-0.009	0.796868392	0.917923158
RNY4P16	-0.009	0.796849588	0.917923158
MIR6842	-0.009	0.796793985	0.91790405
RP11-415J8	-0.009	0.79676142	0.917888736
C6orf120	-0.009	0.796602928	0.91779495
RP11-470L19	-0.009	0.79653698	0.91774117
SCGB1D1	-0.009	0.796426589	0.917680582
SIK2	-0.009	0.796361834	0.917628168
NAALADL2-AS3	-0.009	0.796356883	0.917628168
ILDR1	-0.009	0.796355933	0.917628168
AC217773	-0.009	0.796308773	0.917628168

AC018737	-0.009	0.79624728	0.917607172
SRIP1	-0.009	0.796069309	0.917446474
SNRPCP15	-0.009	0.796001844	0.917390922
RP11-180N14	-0.009	0.795990847	0.917390922
CLCN2	-0.009	0.795900775	0.917341034
GABRG3-AS1	-0.009	0.795851138	0.917335358
MICAL3	-0.009	0.795732637	0.917280431
AGBL5-AS1	-0.009	0.795719262	0.917280431
OR1L1	-0.009	0.795687867	0.917273226
RP11-420A23	-0.009	0.795582205	0.917198934
KHDC1L	-0.009	0.795561424	0.917198934
RP1-48J14	-0.009	0.795348632	0.917037531
AL357075	-0.009	0.795333776	0.917037531
RP11-432M24	-0.009	0.795263028	0.917005429
AC005229	-0.009	0.795227857	0.917005429
DNAJC18	-0.009	0.795136012	0.916925567
MIR4675	-0.009	0.795010589	0.91682533
AL669914	-0.009	0.79497067	0.916801494
CTC-435M10	-0.009	0.794927053	0.916800906
ANKRD20A11P	-0.009	0.794856308	0.9167584
RP11-77A13	-0.009	0.794798931	0.916719624
PSPC1P2	-0.009	0.79476273	0.91671707
MIR4295	-0.009	0.794666522	0.916650499
RN7SL459P	-0.009	0.794648799	0.916650499
EEF1DP4	-0.009	0.794452463	0.916492369
RP11-5K23	-0.009	0.794316143	0.916373317
OSTN	-0.009	0.794315795	0.916373317
RP11-358N2	-0.009	0.794232239	0.916327096
CCT7P1	-0.009	0.794186227	0.916296208
TM7SF3	-0.009	0.79409347	0.91623358
AF196970	-0.009	0.794088038	0.91623358
U1	-0.009	0.794068618	0.91623358
DPP3P1	-0.009	0.794064854	0.91623358
IGLVI-42	-0.009	0.794027895	0.91623358
RP11-225H22	-0.009	0.793997866	0.91623358
CFL1P3	-0.009	0.793951057	0.916224637
CCND3P1	-0.009	0.793949346	0.916224637
AP003096	-0.009	0.793623242	0.915957305
MRPL3	-0.009	0.793580548	0.915930225
ABHD12B	-0.009	0.79355925	0.915927841
ZNF385D	-0.009	0.793546861	0.915927841
RTCA-AS1	-0.009	0.793516123	0.915922456
PPIAP2	-0.009	0.793437249	0.915853611
NQO1	-0.009	0.793379277	0.915831088
RP11-473M10	-0.009	0.793300025	0.915761799
HSPE1P4	-0.009	0.793245743	0.915721332
RP11-780O24	-0.009	0.793239821	0.915721332
RPS15AP38	-0.009	0.793158621	0.915686849
KITLG	-0.009	0.793093396	0.915686849
RAP1GAP2	-0.009	0.79303235	0.915686849
PSMC1P12	-0.009	0.793011189	0.915686849
PUS7	-0.009	0.793007932	0.915686849

RP11-1325J9	-0.009	0.792967911	0.915686849
RPS6P22	-0.009	0.792957518	0.915686849
RP11-1086F11	-0.009	0.792918001	0.915686849
RNU6-222P	-0.009	0.792854728	0.915669442
RP11-58K22	-0.009	0.792767021	0.915612555
CSPG4P3Y	-0.009	0.792718461	0.915578672
DPP3	-0.009	0.792690515	0.915568598
RP11-1114I9	-0.009	0.792375929	0.915338436
MCFD2	-0.009	0.792304525	0.915322554
MIR6752	-0.009	0.792286617	0.915322554
RNA5SP395	-0.009	0.792115603	0.915193097
CALCB	-0.009	0.792089857	0.915185552
AL049872	-0.009	0.792027089	0.91513523
IGKV2D-23	-0.009	0.79195238	0.915071108
RP11-316M20	-0.009	0.791879686	0.915009312
KRT8P11	-0.009	0.791823844	0.914966985
RP11-15E18	-0.009	0.791789388	0.914949369
RNU6-270P	-0.009	0.791743764	0.914949369
RP11-502M1	-0.009	0.791723593	0.914949369
ITGA9-AS1	-0.009	0.791624851	0.914892424
TSPAN12	-0.009	0.791489777	0.914802913
Evf-2_5p	-0.009	0.791483595	0.914802913
OR13C1P	-0.009	0.791394857	0.914782002
INPP5F	-0.009	0.791375327	0.914782002
ALB	-0.009	0.791373003	0.914782002
TUBBP1	-0.009	0.791325256	0.914782002
PDE4B	-0.009	0.791240685	0.914759202
ATF1P1	-0.009	0.791238972	0.914759202
PRMT5P1	-0.009	0.791216787	0.914759202
RNU6-225P	-0.009	0.791175239	0.914759202
STMN1P2	-0.009	0.791156067	0.914759202
ELK1	-0.009	0.791082489	0.914759202
RN7SL162P	-0.009	0.791074605	0.914759202
FAR1P1	-0.009	0.791054917	0.914759202
MIR3132	-0.009	0.791045668	0.914759202
RP13-465B17	-0.009	0.790980729	0.914759202
RNU6-1009P	-0.009	0.790935121	0.914759202
RP11-756D7	-0.009	0.790891974	0.914743053
RP13-612N21	-0.009	0.790891875	0.914743053
GS1-304P7	-0.009	0.790764263	0.914652456
LANCL2	-0.009	0.790687035	0.914629768
BNIP3P30	-0.009	0.790625762	0.914603317
ACOT4	-0.009	0.790602062	0.914598115
AC025442	-0.009	0.790572042	0.914585602
PAK1	-0.009	0.790552425	0.914585124
RP11-888D10	-0.009	0.790351363	0.914396939
AC025811	-0.009	0.790320696	0.91438367
HMGN2P34	-0.009	0.790311198	0.91438367
MTND4P10	-0.009	0.790261421	0.914359515
CLVS2	-0.009	0.790217838	0.914343953
RP11-57J16	-0.009	0.790180441	0.914343953
AKR1B1P8	-0.009	0.790166357	0.914343953

RP11-760D2	-0.009	0.79014133	0.914343953
GSTA10P	-0.009	0.790098776	0.914343953
TRAJ53	-0.009	0.790097005	0.914343953
MIR6794	-0.009	0.790077186	0.914343953
PRR36	-0.009	0.790046011	0.914343953
AC015818	-0.009	0.790032574	0.914343953
PITRM1	-0.009	0.789990075	0.914343953
RP11-266K4	-0.009	0.789866852	0.914325019
RN7SKP273	-0.009	0.789841562	0.914317966
LINC00476	-0.009	0.789684341	0.914158186
MAGEA9B	-0.009	0.789648882	0.914139357
RP11-317M22	-0.009	0.789569509	0.914091909
MSTN	-0.009	0.789478983	0.914009323
RP11-159J3	-0.009	0.789475769	0.914009323
LINC01277	-0.009	0.789464626	0.914009323
RHOB	-0.009	0.789458109	0.914009323
SNRPD1	-0.009	0.789419425	0.914009323
BCL11A	-0.009	0.789398401	0.914009323
AC137695	-0.009	0.789386657	0.914009323
POTEA	-0.009	0.789271764	0.913969372
LINC01361	-0.009	0.789153024	0.913876312
TPM3P2	-0.009	0.789140308	0.913876312
NOBOX	-0.009	0.789088647	0.913846201
MED22	-0.009	0.789042277	0.913814719
C1orf52	-0.009	0.789002336	0.913790683
RP11-445F12	-0.009	0.788840986	0.913735589
NBEAL1	-0.009	0.788836695	0.913735589
AE000661	-0.009	0.78878932	0.913735589
MRPS35P2	-0.009	0.788788421	0.913735589
RPS7P7	-0.009	0.788767477	0.913735589
RP11-457D20	-0.009	0.788733488	0.913735589
MIR5590	-0.009	0.788611795	0.913735589
LINC00572	-0.009	0.788547684	0.913735589
RP11-95H11	-0.009	0.788539405	0.913735589
RP11-493K23	-0.009	0.788402863	0.913735589
RP11-778J16	-0.009	0.788380938	0.913735589
RP11-393N4	-0.009	0.78832013	0.913714404
RP11-213N20	-0.009	0.788287043	0.913714404
RP11-903H12	-0.009	0.788285133	0.913714404
SORD2P	-0.009	0.788278041	0.913714404
COLCA2	-0.009	0.788218303	0.913714404
CPXCR1	-0.009	0.788143727	0.913714404
CAPZA2	-0.009	0.788115804	0.913714404
RP11-89N17	-0.009	0.787984556	0.913611676
C2orf48	-0.009	0.787921517	0.913589394
CKS1BP5	-0.009	0.787904513	0.913589394
KIF5A	-0.009	0.787896201	0.913589394
TEX264	-0.009	0.787819934	0.913578485
GATM-AS1	-0.009	0.787804357	0.913578485
EEF1DP3	-0.009	0.787779895	0.913578485
STARD9	-0.009	0.787591748	0.91342308
RN7SKP277	-0.009	0.787297001	0.91323691

LDHAP5	-0.009	0.787220553	0.913197763
PFN1P8	-0.009	0.787129193	0.913153459
OR5G1P	-0.009	0.787064108	0.913100194
RP11-514P8	-0.009	0.786969111	0.913012225
AC003688	-0.009	0.786967734	0.913012225
CTD-2588C8	-0.009	0.786881289	0.912954814
RP3-340N1	-0.009	0.786799167	0.912881773
PSMC1P7	-0.009	0.786785254	0.912881773
DRG2	-0.009	0.786780008	0.912881773
PIGT	-0.009	0.786729177	0.912867284
FLJ46066	-0.009	0.786714626	0.912867284
KIRREL3-AS3	-0.009	0.78643854	0.912752421
OR2AP1	-0.009	0.78639117	0.912725084
CTD-2043I16	-0.009	0.786237275	0.912587702
PRDM12	-0.009	0.786104869	0.912476338
ZNF204P	-0.009	0.786093235	0.912476338
chr22-38_28785274-29006793	-0.009	0.786011338	0.912452792
DKK2	-0.009	0.785938262	0.912413151
AC111194	-0.009	0.785916399	0.912413151
CTD-2134A5	-0.009	0.785889755	0.912404537
CGB2	-0.009	0.785850647	0.912381375
RNU6-838P	-0.009	0.785841423	0.912381375
SPATA31D2P	-0.009	0.785762747	0.912348637
HEBP1	-0.009	0.785678732	0.912323702
SRSF10P1	-0.009	0.785632523	0.912306046
FGF12	-0.009	0.785593487	0.912282959
RP4-755D9	-0.009	0.785524796	0.912282959
IER3	-0.009	0.785500266	0.912282959
RP3-438O4	-0.009	0.785476324	0.912282959
HNRNPA1P60	-0.009	0.785460919	0.912282959
RP11-630A13	-0.009	0.78545369	0.912282959
CLK3P2	-0.009	0.7854407	0.912282959
LRCOL1	-0.009	0.785351229	0.912282959
ALYREF	-0.009	0.785071154	0.912098938
CYP2F2P	-0.009	0.785021394	0.912063375
AC136932	-0.009	0.784934439	0.911984595
NDUFV2P1	-0.009	0.784602649	0.911688064
RP11-449H3	-0.009	0.784575095	0.911678289
MIR3180-4	-0.009	0.784549334	0.911670598
RPL7P25	-0.009	0.78451975	0.911658464
SNORD115-30	-0.009	0.784500594	0.911658447
MRPS18BP2	-0.009	0.784419513	0.911658447
MIR6071	-0.009	0.784371725	0.911642154
RP11-123O1	-0.009	0.784356631	0.911642154
RP11-430E17	-0.009	0.784352071	0.911642154
RP5-981O7	-0.009	0.784292626	0.911616959
AC020689	-0.009	0.784192031	0.91154563
MEIS3P2	-0.009	0.784170994	0.91154563
NDUFB4P2	-0.009	0.784147925	0.91154563
RP11-1J7	-0.009	0.784063506	0.91154563
AC004112	-0.009	0.783923129	0.911463666
RP11-807H7	-0.009	0.783863233	0.911429267

CHURC1	-0.009	0.783780854	0.911385009
GRM8	-0.009	0.783752738	0.911385009
SERPINA13P	-0.009	0.783684328	0.911376996
FAM172A	-0.009	0.783646989	0.911366784
PAK7	-0.009	0.783558637	0.911342089
GNMT	-0.009	0.783536629	0.911338747
RP11-555H23	-0.009	0.783515546	0.911336482
CTD-3118D11	-0.009	0.783509268	0.911336482
PSEN2	-0.009	0.783497605	0.911336482
USP17L14P	-0.009	0.783407961	0.91127811
MAGOHB	-0.009	0.783385572	0.91127811
H3F3A	-0.009	0.783307529	0.91125031
SNORA72	-0.009	0.783285683	0.911247154
RP11-12A20	-0.009	0.783216871	0.911222801
OR2M5	-0.009	0.783112738	0.911222801
TCF25	-0.009	0.783080546	0.911208824
RP5-1100H13	-0.009	0.783005353	0.911208824
MTCO1P3	-0.009	0.782986918	0.911208824
MORF4L1P4	-0.009	0.782916178	0.911208824
ICE2P2	-0.009	0.782820924	0.911208824
FAM53C	-0.009	0.782789816	0.911208824
KRT18	-0.009	0.782767163	0.911208824
RN7SKP34	-0.009	0.782752783	0.911208824
ERVV-2	-0.009	0.782556624	0.911088891
AC005034	-0.009	0.782553212	0.911088891
RNU6-586P	-0.009	0.782484258	0.911049989
EMR1	-0.009	0.782352311	0.910940092
ARHGAP35	-0.009	0.782325467	0.910931106
RBMX2P4	-0.009	0.782276523	0.910896386
MBD3L5	-0.009	0.782181298	0.910830041
AC069157	-0.009	0.782178231	0.910830041
PRAMEF4	-0.009	0.782113242	0.910830041
OR4E2	-0.009	0.782022224	0.910800686
CCDC173	-0.009	0.782013004	0.910800686
TAS2R38	-0.009	0.782012283	0.910800686
RBP3	-0.009	0.781981599	0.910800686
C16orf58	-0.009	0.781952198	0.910800686
PARM1	-0.009	0.781947166	0.910800686
PSMC5	-0.009	0.78187126	0.910758493
RP11-392P7	-0.009	0.78183855	0.910742665
AC005237	-0.009	0.781809697	0.91073133
RP11-617J18	-0.009	0.781788312	0.91073133
RP5-858B6	-0.009	0.781755937	0.91073133
GNB3	-0.009	0.781636846	0.91073133
AC110283	-0.009	0.781616313	0.91073133
RP11-1112C15	-0.009	0.781567706	0.91073133
SNHG7	-0.009	0.781539983	0.91073133
HNRNPUL2	-0.009	0.781526565	0.91073133
CTC-493L21	-0.009	0.781466364	0.910710054
CEP164	-0.009	0.781449832	0.910710054
RP11-212E4	-0.009	0.781416048	0.910695985
SS18L2	-0.009	0.78139155	0.910689719

AC090505	-0.009	0.781331878	0.910661182
COX5AP2	-0.009	0.781323632	0.910661182
RP11-96C21	-0.009	0.781174767	0.910593045
C1QBP	-0.009	0.781130764	0.910564037
RP11-344N17	-0.009	0.781114377	0.910564037
NDUFA5P8	-0.009	0.781071706	0.910562053
RN7SL215P	-0.009	0.781048433	0.910557211
RP11-74M13	-0.009	0.781036882	0.910557211
CYP4F22	-0.009	0.780869579	0.910437842
snoU2-30	-0.009	0.780843043	0.910429191
AC011233	-0.009	0.78071815	0.910372719
RP11-25O3	-0.009	0.78069324	0.91036922
MIR1323	-0.009	0.780693119	0.91036922
LINC00598	-0.009	0.780626276	0.91036922
NUDT4	-0.009	0.78061136	0.91036922
TCEB1P24	-0.009	0.780561197	0.91036922
RP11-192I3	-0.009	0.780559162	0.91036922
CTD-2651B20	-0.009	0.780489384	0.91036922
RNU6-38P	-0.010	0.780359112	0.91036922
AL360178	-0.010	0.780326578	0.91036922
RP13-512J5	-0.010	0.780314191	0.91036922
BET1L	-0.010	0.780140251	0.910300604
CH17-472G23	-0.010	0.779975968	0.91019811
LINC01523	-0.010	0.779973225	0.91019811
AC010148	-0.010	0.779830333	0.910095057
RP11-300M24	-0.010	0.779802985	0.910085441
AC087348	-0.010	0.779721106	0.910037109
CTC-529L17	-0.010	0.779710466	0.910037109
LINC01149	-0.010	0.779676418	0.910037109
HSPE1P22	-0.010	0.779556114	0.909951531
C22orf23	-0.010	0.779526756	0.909941443
GAPDHP21	-0.010	0.779483129	0.909912818
RP11-310J24	-0.010	0.779467987	0.909912818
MIR514B	-0.010	0.779455885	0.909912818
AL450063	-0.010	0.779441176	0.909912818
RN7SKP85	-0.010	0.779426457	0.909912818
RP11-441M10	-0.010	0.779386184	0.909912818
ZSCAN31	-0.010	0.779249806	0.909841154
CTD-2270N23	-0.010	0.779235562	0.909841154
YBX2	-0.010	0.779209586	0.909838803
RP11-774G5	-0.010	0.779150252	0.909791827
RNA5SP87	-0.010	0.779006394	0.909668453
RP1-154K9	-0.010	0.778984667	0.909668453
SNORD92	-0.010	0.778948831	0.909668146
RNA5SP291	-0.010	0.778906633	0.909641171
TAS2R39	-0.010	0.778852592	0.909606798
RNU6ATAC4P	-0.010	0.778798031	0.909603557
NT5C3AP1	-0.010	0.77879523	0.909603557
ATF4P1	-0.010	0.778759591	0.909603272
RP11-716D16	-0.010	0.77872139	0.90958096
RP11-30J20	-0.010	0.778670885	0.909544274
NACA	-0.010	0.77856279	0.909440316

RP1-34B20	-0.010	0.778544558	0.909440316
SNORD111B	-0.010	0.778523361	0.909440316
RPL27P12	-0.010	0.778493438	0.909440316
AC006000	-0.010	0.778479767	0.909440316
SNAPC2	-0.010	0.778466873	0.909440316
WBP1	-0.010	0.778425408	0.909440316
RN7SL426P	-0.010	0.778409558	0.909440316
GINS3	-0.010	0.778406979	0.909440316
CTD-2527I21	-0.010	0.778343316	0.909440316
RNU6-1151P	-0.010	0.778239709	0.909440316
LINC01195	-0.010	0.778089144	0.909390062
ATP5A1P8	-0.010	0.778037551	0.909362057
FOXP1-IT1	-0.010	0.777973664	0.909327252
LINC01232	-0.010	0.777918241	0.90931342
RP11-715L17	-0.010	0.777775559	0.909234347
SCD	-0.010	0.777762467	0.909234347
ENPP7P12	-0.010	0.777729177	0.909234347
RP11-793B23	-0.010	0.777719655	0.909234347
KRT8P2	-0.010	0.777704294	0.909234347
ADAMTS9-AS1	-0.010	0.77769371	0.909234347
AC133552	-0.010	0.777665956	0.909234347
RNU6-394P	-0.010	0.777653898	0.909234347
ATF6	-0.010	0.777561062	0.909234347
PCGF7P	-0.010	0.777493239	0.909234347
UGT1A12P	-0.010	0.77744554	0.909205617
LINC01141	-0.010	0.777337932	0.909131926
OR10H2	-0.010	0.777333381	0.909131926
RNU1-64P	-0.010	0.777292363	0.909131926
RP11-777J24	-0.010	0.777274999	0.909131926
SLC25A23	-0.010	0.777270914	0.909131926
RP13-131K19	-0.010	0.777067034	0.909005261
MIR5004	-0.010	0.777065565	0.909005261
MIR6817	-0.010	0.777046163	0.909005261
GAPDHS	-0.010	0.776901313	0.908903926
ZFPM1	-0.010	0.776891634	0.908903926
MIR4281	-0.010	0.776740559	0.908805154
AC011243	-0.010	0.776552106	0.908651626
RP11-92B11	-0.010	0.776425263	0.908570172
GEMIN2P2	-0.010	0.776300111	0.908557659
ENOX2	-0.010	0.776277195	0.908553165
AC073284	-0.010	0.776188075	0.908493511
FHP1	-0.010	0.776171028	0.908493511
SLC43A1	-0.010	0.776142439	0.908484749
SASH1	-0.010	0.775877968	0.908242147
RNU6-854P	-0.010	0.775643534	0.908060481
LINC00929	-0.010	0.775548178	0.908034639
LLPHP1	-0.010	0.775501345	0.908002128
EI24P4	-0.010	0.775495826	0.908002128
RNU6-352P	-0.010	0.775452879	0.908002128
NOL4	-0.010	0.775380645	0.907994737
MIR4325	-0.010	0.775366235	0.907994737
TTLL7	-0.010	0.775269199	0.907931202

RP11-437J2	-0.010	0.775064033	0.907824866
LINC00548	-0.010	0.775011209	0.90779254
SNORD19B	-0.010	0.774979459	0.90779254
XRCC1	-0.010	0.774891454	0.907779001
OR5M9	-0.010	0.774476821	0.907427183
RP11-141A19	-0.010	0.774380729	0.907341501
RN7SKP292	-0.010	0.774286549	0.907338176
FAM41C	-0.010	0.774281443	0.907338176
RP5-827L5	-0.010	0.774260286	0.907338176
RPS13P2	-0.010	0.774188398	0.907312454
AL079334	-0.010	0.774168148	0.907311049
OR9I3P	-0.010	0.774146663	0.907311049
PPP1R3B	-0.010	0.77412818	0.907311049
LCE6A	-0.010	0.774105436	0.907311049
ACTL6A	-0.010	0.774051656	0.907311049
STK31	-0.010	0.774034286	0.907311049
CTC-507E2	-0.010	0.773960175	0.907311049
RD3	-0.010	0.773916066	0.907305858
RP11-567E21	-0.010	0.773872875	0.90729989
PGAM1P13	-0.010	0.773843028	0.907287231
AL136234	-0.010	0.773793508	0.907276391
RDH16	-0.010	0.773730527	0.907267002
RP11-40C6	-0.010	0.773567469	0.907165141
RP11-916L7	-0.010	0.773548763	0.907165141
PIR	-0.010	0.773526079	0.907161276
RP11-703H8	-0.010	0.773458932	0.907107125
RP11-173L6	-0.010	0.773409842	0.907104928
RP11-637O19	-0.010	0.773366726	0.907104928
AKIRIN1P2	-0.010	0.773229499	0.906996116
HSD17B13	-0.010	0.77303249	0.906828039
BCKDHB	-0.010	0.77298305	0.906792378
AC005187	-0.010	0.772972864	0.906792378
ATP5J2P3	-0.010	0.772948108	0.906792378
AC018767	-0.010	0.772947466	0.906792378
CT867977	-0.010	0.772796299	0.906684961
ZNF319	-0.010	0.772786183	0.906684961
MBIP	-0.010	0.772717638	0.906684961
CTNNAP1	-0.010	0.772598249	0.906592844
CRIPT	-0.010	0.772564254	0.90659138
RP3-375P9	-0.010	0.772410859	0.906456041
COL9A2	-0.010	0.772309331	0.906411603
RNA5SP400	-0.010	0.772272731	0.906411603
RNU6-1108P	-0.010	0.772259111	0.906411603
RP11-787I22	-0.010	0.772139576	0.906383349
CLN5	-0.010	0.771966686	0.906292086
NMD3	-0.010	0.771858051	0.906186886
RP1-257A15	-0.010	0.771851984	0.906186886
GS1-256O22	-0.010	0.771763429	0.906142807
PDE2A	-0.010	0.771760284	0.906142807
SPATA17-AS1	-0.010	0.771579606	0.906016319
AL096864	-0.010	0.771483065	0.905969966
snoZ196	-0.010	0.771452446	0.905956347

MIR6090	-0.010	0.771377737	0.90589095
ARRDC2	-0.010	0.771252288	0.905832968
KIAA0355	-0.010	0.771177099	0.905766996
RP1-273P12	-0.010	0.771099436	0.905765127
OR8J2	-0.010	0.770898624	0.905584795
RP11-753C18	-0.010	0.770819053	0.905547447
RP11-354O24	-0.010	0.770748307	0.90548667
OR56A7P	-0.010	0.770743752	0.90548667
HIRAP1	-0.010	0.770731775	0.90548667
CTD-2265O21	-0.010	0.770711736	0.90548667
VN2R3P	-0.010	0.770574256	0.905416196
AC108925	-0.010	0.770556786	0.905416196
FAM170B-AS1	-0.010	0.770534502	0.90541416
ADORA3	-0.010	0.770520544	0.90541416
RP4-775C13	-0.010	0.770253165	0.905195239
AC004470	-0.010	0.770085298	0.905042628
MIR526B	-0.010	0.770067826	0.905042628
RNU6-399P	-0.010	0.769959728	0.904939713
RP11-413G15	-0.010	0.769790935	0.904788194
TK2	-0.010	0.769687139	0.90470864
PRLR	-0.010	0.769598219	0.904673523
POU4F3	-0.010	0.769596716	0.904673523
RP11-386D6	-0.010	0.769589019	0.904673523
SAFB2	-0.010	0.769535602	0.904673523
DSCR4	-0.010	0.769527809	0.904673523
TRIM41	-0.010	0.769407449	0.904605102
EEF1A1P22	-0.010	0.769263019	0.904567311
RNU6-416P	-0.010	0.769161954	0.904470801
RP11-453D16	-0.010	0.769126559	0.904451511
RP11-24I21	-0.010	0.769110491	0.904451511
SLC8A2	-0.010	0.769053829	0.904451511
RP11-254G11	-0.010	0.768959977	0.9043896
RP11-68L18	-0.010	0.768951446	0.9043896
VN2R17P	-0.010	0.768951386	0.9043896
SPACA4	-0.010	0.768905799	0.9043896
AC073387	-0.010	0.768878896	0.9043896
RN7SKP2	-0.010	0.76885649	0.9043896
RBM22P2	-0.010	0.768762118	0.904357886
SNORD116-8	-0.010	0.768724899	0.904353742
RP4-630C24	-0.010	0.768719851	0.904353742
AC018735	-0.010	0.76866644	0.904353742
LINC00685	-0.010	0.768664118	0.904353742
RP11-507A3	-0.010	0.768637382	0.904353742
TMEM14C	-0.010	0.768621136	0.904353742
SLC25A34	-0.010	0.768613625	0.904353742
RNU6-501P	-0.010	0.768583471	0.904353742
HORMAD1	-0.010	0.768569979	0.904353742
PDCD5P2	-0.010	0.768500623	0.904353742
RP11-467D10	-0.010	0.768475745	0.904353742
OR10Y1P	-0.010	0.768465411	0.904353742
RP11-89M20	-0.010	0.768439017	0.904353742
RP11-557C9	-0.010	0.768397057	0.904353742

VN1R1	-0.010	0.768331546	0.904342787
RNA5SP523	-0.010	0.768263908	0.904307872
HSPD1P8	-0.010	0.768241922	0.904304343
KCTD12	-0.010	0.768168822	0.904240644
RNU4-25P	-0.010	0.768037781	0.904153432
CDK2AP2P3	-0.010	0.768031443	0.904153432
RP11-266E8	-0.010	0.767952993	0.90414301
SMC2-AS1	-0.010	0.767841539	0.904034139
RPL30P1	-0.010	0.767747423	0.903945675
RP11-313F23	-0.010	0.767724888	0.903945675
MIR133A2	-0.010	0.767720085	0.903945675
RNU6-1226P	-0.010	0.767701457	0.903945675
TAAR1	-0.010	0.76769578	0.903945675
RP11-402L1	-0.010	0.767538788	0.903901139
MIR4648	-0.010	0.767522186	0.903901139
AP000478	-0.010	0.767398279	0.903780362
AL772307	-0.010	0.767357482	0.903777011
IQCK	-0.010	0.767309475	0.903776715
RNASE3	-0.010	0.767304782	0.903776715
HIST1H2BI	-0.010	0.767210728	0.903767127
OR8B4	-0.010	0.767194911	0.903767127
RP5-956O18	-0.010	0.766980601	0.903623626
SOX13	-0.010	0.766976659	0.903623626
MIR3939	-0.010	0.766791184	0.903489862
RP11-460E7	-0.010	0.766784807	0.903489862
RP11-543N12	-0.010	0.766763531	0.903489862
LRIT1	-0.010	0.766762118	0.903489862
RN7SKP75	-0.010	0.766708352	0.903489862
RP11-966I7	-0.010	0.766666848	0.903489862
RN7SL204P	-0.010	0.76666526	0.903489862
RN7SKP255	-0.010	0.766653741	0.903489862
KRT18P13	-0.010	0.766572406	0.903489862
AC024082	-0.010	0.766500551	0.903482694
RNU6-721P	-0.010	0.766454949	0.903451302
RP11-344N10	-0.010	0.766295729	0.903285979
MIR1273A	-0.010	0.766213782	0.903211736
RP4-568B10	-0.010	0.766212852	0.903211736
RP11-90D4	-0.010	0.766209475	0.903211736
FOXI2	-0.010	0.766152275	0.903211736
HMGA1P3	-0.010	0.766145914	0.903211736
RP11-348M3	-0.010	0.766095048	0.903205903
SLC16A14P1	-0.010	0.766091515	0.903205903
ZNF385A	-0.010	0.766077269	0.903205903
AC025458	-0.010	0.766008872	0.903205903
OR8S1	-0.010	0.765995372	0.903205903
RNU6-710P	-0.010	0.765954353	0.903196533
RAET1E-AS1	-0.010	0.765788395	0.903045556
ITGA8	-0.010	0.765649159	0.902957044
CCDC186	-0.010	0.765647075	0.902957044
CATIP	-0.010	0.765557866	0.902930202
EGLN1	-0.010	0.765508277	0.902930202
PELI3	-0.010	0.765502859	0.902930202

RP11-657O9	-0.010	0.765372565	0.902823439
INSL5	-0.010	0.765359147	0.902823439
MIR3177	-0.010	0.7652772	0.902822749
CCNB1IP1P1	-0.010	0.765264171	0.902822749
ANGPT4	-0.010	0.765198521	0.902776941
RPL13P2	-0.010	0.765184924	0.902776941
HCG14	-0.010	0.765169676	0.902776941
SST	-0.010	0.765137966	0.902776941
RP11-874J12	-0.010	0.765072562	0.902776941
VWF	-0.010	0.765057759	0.902776941
LL0XNC01-138C3	-0.010	0.765038255	0.902776941
XX-FW83563B9	-0.010	0.764922744	0.902740002
TMEM42	-0.010	0.764911076	0.902740002
RNU7-75P	-0.010	0.764908062	0.902740002
CTB-31C7	-0.010	0.764871051	0.902740002
CTD-2160D9	-0.010	0.764860156	0.902740002
RP11-564H18	-0.010	0.764768238	0.902739744
RNA5SP488	-0.010	0.764767782	0.902739744
RP5-1086K13	-0.010	0.764766481	0.902739744
AC010455	-0.010	0.764707329	0.902739744
TYROBP	-0.010	0.764681484	0.902739744
CLCF1	-0.010	0.764640235	0.902728612
EIF4E2	-0.010	0.764622319	0.902728612
LDHAP2	-0.010	0.764617242	0.902728612
GNA14-AS1	-0.010	0.764611167	0.902728612
FAM69A	-0.010	0.764563571	0.902728612
AC092048	-0.010	0.764528382	0.902728612
MAP3K1	-0.010	0.764283602	0.902544819
TMEM97P1	-0.010	0.764282016	0.902544819
RNU4-12P	-0.010	0.764161756	0.902490439
PTPN2P2	-0.010	0.764158848	0.902490439
MIR4261	-0.010	0.764141358	0.902490439
SNORD5	-0.010	0.763917799	0.902336574
RN7SL471P	-0.010	0.763800687	0.902265373
ADAMTS9-AS2	-0.010	0.763798691	0.902265373
RP11-257P3	-0.010	0.763726815	0.902222865
DRGX	-0.010	0.763620906	0.902187261
RPS26P8	-0.010	0.763460241	0.902056564
BCKDHA	-0.010	0.763321859	0.90194582
PPT1	-0.010	0.763295207	0.90194582
OR7E18P	-0.010	0.763261664	0.90194582
FAM83G	-0.010	0.763260226	0.90194582
MIR506	-0.010	0.763186021	0.901919571
MOBP	-0.010	0.763159235	0.901910295
RP11-179A18	-0.010	0.76309585	0.901891101
GSPT1	-0.010	0.763089496	0.901891101
RP3-393B19	-0.010	0.763000587	0.901834693
CXorf49B	-0.010	0.762979775	0.901832475
SPAG8	-0.010	0.762951254	0.901821144
RNA5SP66	-0.010	0.762903451	0.901809403
RP11-472F21	-0.010	0.762837594	0.901776318
KCNN2	-0.010	0.762837168	0.901776318

ADSL	-0.010	0.762760559	0.901776318
TMEM44-AS1	-0.010	0.762759726	0.901776318
TRMU	-0.010	0.762529913	0.901658775
RPL5P23	-0.010	0.762473711	0.901614705
RP11-1315F8	-0.010	0.762447756	0.901606398
RP11-545E17	-0.010	0.762432498	0.901606398
MIR6871	-0.010	0.762403527	0.901598867
AC092896	-0.010	0.762376255	0.901598867
GAFA3	-0.010	0.762371261	0.901598867
RP11-523L20	-0.010	0.76226547	0.901598867
RP4-705O1	-0.010	0.762251592	0.901598867
AC118345	-0.010	0.762246963	0.901598867
TBPL1	-0.010	0.762244756	0.901598867
CTB-33G10	-0.010	0.762186896	0.901598867
RP11-580P21	-0.010	0.762133336	0.901598867
BTF3P2	-0.010	0.761986144	0.901530594
CFHR1	-0.010	0.76197098	0.901530594
RNU6-147P	-0.010	0.761915821	0.901492182
RP11-78O22	-0.010	0.761837122	0.901421461
GPAT2	-0.010	0.761709499	0.901309588
RNU6-636P	-0.010	0.761696233	0.901309588
JTBP1	-0.010	0.761688252	0.901309588
RPL30P9	-0.010	0.761668625	0.901309588
RP1-172B20	-0.010	0.761649806	0.901309588
RP11-33A14	-0.010	0.761534627	0.901265057
HTR1DP1	-0.010	0.761529191	0.901265057
OR4H12P	-0.010	0.761457754	0.901218868
RP11-46F15	-0.010	0.761444544	0.901218868
GPR42	-0.010	0.761418499	0.901217199
RP11-196I18	-0.010	0.761378036	0.9012141
C12orf74	-0.010	0.761275357	0.901139838
RP11-325I22	-0.010	0.761261605	0.901139838
DNMT3B	-0.010	0.761129418	0.901048102
RNU6-1137P	-0.010	0.76107526	0.901034861
RP13-313G19	-0.010	0.760976227	0.901009755
MIR769	-0.010	0.760961453	0.901009755
MIR1273E	-0.010	0.760848069	0.900945053
RP11-555H7	-0.010	0.760790137	0.900898853
ATP6V1E1	-0.010	0.760778688	0.900898853
GOLGA6L7P	-0.010	0.76067985	0.900835449
ZBTB20	-0.010	0.760646813	0.900818722
RNU6-1265P	-0.010	0.760574548	0.900777938
LINC01289	-0.010	0.76051025	0.900763483
TRDMT1	-0.010	0.760382251	0.900725758
OR2L1P	-0.010	0.760372976	0.900725758
TMEM56	-0.010	0.76033816	0.900721957
RP11-54F2	-0.010	0.760230102	0.900638751
RN7SL764P	-0.010	0.760210056	0.900638751
MIR566	-0.010	0.760177145	0.900638751
CH17-125A10	-0.010	0.76014096	0.900638751
SOS1-IT1	-0.010	0.760103993	0.900623765
LINC00352	-0.010	0.760020865	0.900547673

RP11-443B20	-0.010	0.759947616	0.900505688
CRLF2	-0.010	0.759878539	0.900446238
ACOT6	-0.010	0.759809696	0.900446238
RP11-775H9	-0.010	0.759801759	0.900446238
RN7SL160P	-0.010	0.759765819	0.900446238
AC079112	-0.010	0.759697847	0.900411342
ELAC1	-0.010	0.759665664	0.900401026
RN7SKP245	-0.010	0.759586042	0.900346045
CASK	-0.010	0.759547569	0.900345257
GPN1	-0.010	0.759366696	0.900198069
RP11-602M11	-0.010	0.759190496	0.900060509
AK3P4	-0.010	0.759173912	0.900060509
AC073127	-0.010	0.759098328	0.90001434
OR8B7P	-0.010	0.759019122	0.899991301
DYRK2	-0.010	0.758994596	0.899991301
RN7SL798P	-0.010	0.758992183	0.899991301
MXD1	-0.010	0.758989191	0.899991301
HNRNPM	-0.010	0.758897734	0.899991301
BOD1P2	-0.010	0.758829999	0.899991301
AC008865	-0.010	0.758787752	0.899991301
AC026700	-0.010	0.758764767	0.899991301
SPC24	-0.010	0.758719818	0.899991301
AC023824	-0.010	0.758704499	0.899991301
MIR6509	-0.010	0.758697521	0.899991301
PCGF1	-0.010	0.758636684	0.899991301
PDE6H	-0.010	0.758591268	0.899991301
FAM185A	-0.010	0.758510741	0.899989812
KIAA2012	-0.010	0.758454455	0.899945448
HCG4P5	-0.010	0.758453372	0.899945448
KB-1043D8	-0.010	0.758404323	0.899930805
LINGO1-AS2	-0.010	0.758370349	0.899930805
RP4-547N15	-0.010	0.758285814	0.899902284
LINC00379	-0.010	0.758265605	0.899900724
AC068135	-0.010	0.758179025	0.899820394
ELMO3	-0.010	0.758007449	0.89975129
CLDN10-AS1	-0.010	0.757970615	0.89975129
RP11-651K21	-0.010	0.757960062	0.89975129
MRPL35P3	-0.010	0.757946669	0.89975129
VN2R1P	-0.011	0.757885344	0.899740896
CTD-2015H6	-0.011	0.757855359	0.899740896
SERTAD2	-0.011	0.757758127	0.899738295
USP6NL	-0.011	0.757660898	0.899676272
RNU6-1077P	-0.011	0.757608023	0.899666862
RP11-535E8	-0.011	0.757486557	0.899581395
SFXN2	-0.011	0.757414519	0.899518273
ATF7IP2	-0.011	0.757407041	0.899518273
RP11-310I24	-0.011	0.757352341	0.899518273
RP11-44M6	-0.011	0.7573468	0.899518273
ACTR3C	-0.011	0.757341815	0.899518273
PRPF40B	-0.011	0.757178134	0.899484261
RNU5A-6P	-0.011	0.757177246	0.899484261
AC006552	-0.011	0.757174757	0.899484261

LMO2	-0.011	0.757174396	0.899484261
LINC00698	-0.011	0.75717297	0.899484261
RP11-7N14	-0.011	0.757154171	0.899484261
RNU6-1056P	-0.011	0.757016372	0.899484261
DYNLT3	-0.011	0.756954234	0.899484261
SNORD114-7	-0.011	0.756821905	0.89937514
AC104066	-0.011	0.756752781	0.899315435
MIR3175	-0.011	0.756656495	0.899245887
MYL6P2	-0.011	0.756619994	0.899224945
CSF2	-0.011	0.756615505	0.899224945
LINC01247	-0.011	0.756612858	0.899224945
RP11-270F18	-0.011	0.756394847	0.899047102
AC064850	-0.011	0.75630624	0.898986075
TMEM231	-0.011	0.756207078	0.898958529
DAOA-AS1_2	-0.011	0.756207015	0.898958529
RP11-558O12	-0.011	0.755989951	0.89879015
RP11-347N5	-0.011	0.755956293	0.89877257
NPM1P26	-0.011	0.755908219	0.898737849
RPL17P19	-0.011	0.755763295	0.898618699
PCDHA13	-0.011	0.755694251	0.898595613
RNA5SP113	-0.011	0.755693407	0.898595613
ADAM20	-0.011	0.755627495	0.898583539
RNU7-187P	-0.011	0.755600478	0.898573846
RNU6-185P	-0.011	0.755573672	0.898565618
RN7SKP29	-0.011	0.755538735	0.898565618
NCMAP	-0.011	0.755477165	0.898561819
MIR7843	-0.011	0.755412999	0.89855282
RP11-713M15	-0.011	0.755214406	0.898383907
LINC01258	-0.011	0.755032157	0.898269867
RNA5SP23	-0.011	0.754908637	0.898222086
DNAJC2	-0.011	0.754895641	0.898222086
RP11-276M12	-0.011	0.75489365	0.898222086
Z99756	-0.011	0.754745322	0.898095077
FAM25G	-0.011	0.75449673	0.897889002
BHLHE22	-0.011	0.754438318	0.897867816
RNU2-69P	-0.011	0.754263286	0.897788378
LDHAL6EP	-0.011	0.754232698	0.897776685
DGKH	-0.011	0.754085218	0.897646006
RP11-78C3	-0.011	0.754078315	0.897646006
RP5-1189K21	-0.011	0.75405163	0.897646006
RP11-763F8	-0.011	0.753993834	0.897604528
TCERG1L-AS1	-0.011	0.753776228	0.897390336
LINC01288	-0.011	0.753749168	0.897380552
OFCC1	-0.011	0.753696672	0.897362917
MIR5699	-0.011	0.753682804	0.897362917
RN7SKP25	-0.011	0.753651496	0.897353995
CLDN25	-0.011	0.753534677	0.897259764
ARMC12	-0.011	0.753492784	0.897232313
SNX18P5	-0.011	0.753434594	0.897198297
UBE2R2-AS1	-0.011	0.753324637	0.897166675
AC012370	-0.011	0.753268382	0.897122112
AC022210	-0.011	0.753231309	0.897100391

AC126323	-0.011	0.753162805	0.897048936
HECW2	-0.011	0.75315562	0.897048936
CHST2	-0.011	0.75309341	0.897048936
HMGB3P13	-0.011	0.753081755	0.897048936
GABBR2	-0.011	0.753032044	0.897048936
TRIM51FP	-0.011	0.752993699	0.89704172
FLJ30679	-0.011	0.752940826	0.897003604
GPR101	-0.011	0.752822969	0.896928069
AARD	-0.011	0.752798973	0.896928069
RN7SL69P	-0.011	0.752787586	0.896928069
RP11-267A15	-0.011	0.752573107	0.896764978
AC005102	-0.011	0.752572691	0.896764978
KRT18P55	-0.011	0.752561508	0.896764978
OR7E2P	-0.011	0.752540137	0.896764978
RN7SKP167	-0.011	0.752445289	0.896724847
RP11-146E23	-0.011	0.752265522	0.896600351
TRIM60P14	-0.011	0.752199641	0.896544265
CTH	-0.011	0.752196115	0.896544265
FAM157B	-0.011	0.752180421	0.896544265
ZNF728	-0.011	0.752113097	0.896544265
RP5-965F6	-0.011	0.752075262	0.896544265
FAM90A11P	-0.011	0.752051215	0.896544265
OR4A15	-0.011	0.752024563	0.896544265
AC130360	-0.011	0.752008273	0.896544265
NUDT17	-0.011	0.751990082	0.896544265
TAS2R60	-0.011	0.75192496	0.896544265
CCDC144CP	-0.011	0.751766645	0.896476877
RNU6-1281P	-0.011	0.751757988	0.896476877
RP11-796A5	-0.011	0.751732175	0.896476877
MESDC1	-0.011	0.751695367	0.896476877
AC079354	-0.011	0.751692579	0.896476877
LINC00343	-0.011	0.75168984	0.896476877
RP11-44D19	-0.011	0.751659883	0.896476877
RNA5SP466	-0.011	0.751607479	0.896476877
POLR1C	-0.011	0.751558176	0.896475187
CTB-70D19	-0.011	0.75150646	0.89643595
SNORA76A	-0.011	0.751505864	0.89643595
SMG5	-0.011	0.751423481	0.896381869
PLAC8L1	-0.011	0.751403611	0.896380617
MIR3181	-0.011	0.75138529	0.896380617
MIR517B	-0.011	0.751369979	0.896380617
IGHVIII-67-3	-0.011	0.751303052	0.896380617
EIF4A2P2	-0.011	0.751287021	0.896380617
G3BP1	-0.011	0.751174417	0.896331713
RP11-358B23	-0.011	0.751161981	0.896331713
SERPINA6	-0.011	0.751155474	0.896331713
RP11-589F4	-0.011	0.751084062	0.896313721
RN7SL332P	-0.011	0.750929017	0.896173606
SECTM1	-0.011	0.750910878	0.896173606
SSU72P2	-0.011	0.750899749	0.896173606
HSD52	-0.011	0.750881101	0.896173606
OGFOD1P1	-0.011	0.750684296	0.89603872

RP3-526F5	-0.011	0.750470553	0.895895855
SSX2B	-0.011	0.750466025	0.895895855
RP11-357H3	-0.011	0.750448952	0.895895855
RP11-488I20	-0.011	0.750405138	0.895895855
LY96	-0.011	0.750391342	0.895895855
BLACE	-0.011	0.750333634	0.895867134
ACTL8	-0.011	0.750236825	0.895796462
RP11-107M16	-0.011	0.750158039	0.895747303
SNORD46	-0.011	0.750117865	0.895721789
CCDC182	-0.011	0.750113806	0.895721789
RP11-36I17	-0.011	0.750033735	0.895690172
KLHDC7A	-0.011	0.749907617	0.895627917
RP11-32A1	-0.011	0.749895551	0.895627917
RP11-739G5	-0.011	0.749861936	0.895627917
RP11-754I20	-0.011	0.749774217	0.895587586
RP5-979D14	-0.011	0.749748054	0.895587586
RN7SL91P	-0.011	0.749747048	0.895587586
RP11-259F16	-0.011	0.749693708	0.895587586
RNU4-88P	-0.011	0.749540979	0.895561167
RP13-735L24	-0.011	0.749510657	0.895561167
AC092332	-0.011	0.749488909	0.895561167
MIR4269	-0.011	0.749470183	0.895561167
ATP6V1E2	-0.011	0.749434598	0.895557591
RP1-30E17	-0.011	0.749367506	0.89552181
AC122714	-0.011	0.749334975	0.895505403
CD14	-0.011	0.749307889	0.895495505
RP11-263I1	-0.011	0.749289339	0.895495505
PPP1R2P5	-0.011	0.749279965	0.895495505
UMODL1	-0.011	0.749268089	0.895495505
AL442203	-0.011	0.74923811	0.895495505
PEX13	-0.011	0.74921514	0.895495505
LRRIQ1	-0.011	0.749207208	0.895495505
RP11-296E23	-0.011	0.749184874	0.895495505
ATP13A1	-0.011	0.74900895	0.895410569
RP1-140J1	-0.011	0.748950895	0.895405886
TM9SF1	-0.011	0.748914142	0.895384422
RNA5SP472	-0.011	0.748909959	0.895384422
KIR3DP1	-0.011	0.748840883	0.89536611
MIR8075	-0.011	0.748753988	0.89536611
MIR5586	-0.011	0.748561842	0.895232897
OR4S1	-0.011	0.748507475	0.895190355
HNRNPDP2	-0.011	0.748487403	0.895188829
FZD4	-0.011	0.748406217	0.895134386
RP11-90E9	-0.011	0.748393628	0.895134386
FAM167A-AS1	-0.011	0.748203294	0.895051322
BNIP3P29	-0.011	0.748184827	0.895051322
GRPEL2P2	-0.011	0.748104281	0.895034442
RP1-50A13	-0.011	0.748074454	0.895034442
LINC00867	-0.011	0.748046487	0.895034442
P2RX2	-0.011	0.748023787	0.895034442
RP11-13A1	-0.011	0.747824255	0.894980043
STRAP	-0.011	0.747809017	0.894980043

AGAP1-IT1	-0.011	0.747785822	0.894979025
RP11-286M16	-0.011	0.747650069	0.894884015
RNU7-174P	-0.011	0.747613898	0.89486321
RNU6-445P	-0.011	0.747547693	0.894832487
RP11-15M15	-0.011	0.747506117	0.894832487
CDCP2	-0.011	0.747453288	0.894828388
TJP1	-0.011	0.747407244	0.894801059
TMF1P1	-0.011	0.747319437	0.894758109
AC005262	-0.011	0.747211449	0.894651307
EXPH5	-0.011	0.747137503	0.894611592
ZFYVE1	-0.011	0.747087447	0.894592796
AL591509	-0.011	0.747085907	0.894592796
RPS29P28	-0.011	0.747085907	0.894592796
RP11-809N15	-0.011	0.747074692	0.894592796
MTRNR2L12	-0.011	0.747070073	0.894592796
RP11-277A4	-0.011	0.74703021	0.894592796
RP11-419L4	-0.011	0.747006609	0.894592796
C9orf114	-0.011	0.746998921	0.894592796
RP11-324D17	-0.011	0.746994441	0.894592796
RNU6-1003P	-0.011	0.746943632	0.894592796
RP11-548L20	-0.011	0.746881899	0.894592796
OR52E2	-0.011	0.746623435	0.89435197
RPS15AP16	-0.011	0.746507137	0.894235152
RP11-867O8	-0.011	0.746500509	0.894235152
DKC1	-0.011	0.746496372	0.894235152
CTD-2554C21	-0.011	0.746463699	0.894235152
CDC42P5	-0.011	0.74625294	0.894116286
LINC00708	-0.011	0.746251693	0.894116286
RP5-1102E8	-0.011	0.746218628	0.894116286
MRPL50P4	-0.011	0.746187103	0.894116286
PRAM1	-0.011	0.746072907	0.894007305
EIF4A3	-0.011	0.746042308	0.893993132
AC132812	-0.011	0.746014579	0.893982396
RP11-296L20	-0.011	0.745935552	0.893932679
DCAF13	-0.011	0.745932321	0.893932679
KLHL38	-0.011	0.745773455	0.893828384
RP11-141E13	-0.011	0.745753571	0.893827046
RP11-476J6	-0.011	0.745738829	0.893827046
ELAVL4	-0.011	0.745727677	0.893827046
TCF24	-0.011	0.745681009	0.893827046
FGFR3P6	-0.011	0.745676878	0.893827046
CLN8	-0.011	0.745646625	0.893827046
RNU6-1029P	-0.011	0.745635982	0.893827046
AKT1	-0.011	0.745605283	0.893827046
IFT140	-0.011	0.745580064	0.893827046
RP11-429K17	-0.011	0.745539377	0.893827046
CRABP2	-0.011	0.7454763	0.893827046
RP11-326L17	-0.011	0.745470116	0.893827046
NSUN2	-0.011	0.745432067	0.893827046
HAUS6P1	-0.011	0.745308116	0.893742972
RP11-789C7	-0.011	0.74530217	0.893742972
RP11-158A14	-0.011	0.745286069	0.893742972

AL021394		-0.011	0.745126426	0.893664267
MIR618		-0.011	0.745116424	0.893664267
RP11-570J4		-0.011	0.745115304	0.893664267
RP1-68D18		-0.011	0.745100663	0.893664267
SOX8		-0.011	0.745093526	0.893664267
DUXAP3		-0.011	0.74496654	0.893625866
PSMD10		-0.011	0.744892243	0.893581756
ADHFE1		-0.011	0.744880258	0.893581756
LRRC3B		-0.011	0.744877542	0.893581756
CTC-461H2		-0.011	0.744861484	0.893581756
RP11-130C19		-0.011	0.744815046	0.893581756
UBE2V1P12		-0.011	0.744770397	0.893581756
PPIAP30		-0.011	0.744731553	0.893581756
TYRL		-0.011	0.744706758	0.893581756
CTC-454I21		-0.011	0.74465996	0.893581756
RP11-390N6		-0.011	0.744536845	0.893515505
RP11-47A17		-0.011	0.744519633	0.893515505
OR2G3		-0.011	0.744475173	0.893508313
	15-Sep	-0.011	0.744309573	0.893459042
RP11-738G5		-0.011	0.744224188	0.893387912
RNU5F-7P		-0.011	0.744217595	0.893387912
KRT8P18		-0.011	0.744148046	0.893341542
RP11-899L11		-0.011	0.744142873	0.893341542
RP1-92O14		-0.011	0.744142237	0.893341542
PCP4		-0.011	0.744137892	0.893341542
KIF1B		-0.011	0.744128603	0.893341542
POT1		-0.011	0.743862544	0.893133874
WRN		-0.011	0.743858826	0.893133874
KIAA1614-AS1		-0.011	0.743729223	0.89304134
LINC00903		-0.011	0.743720176	0.89304134
FRMD4A		-0.011	0.743625431	0.892984252
EFCAB6-AS1		-0.011	0.743504607	0.8929067
RP11-998D10		-0.011	0.743442849	0.892858437
RP11-181G12		-0.011	0.743263199	0.892687811
LINC00856		-0.011	0.743256924	0.892687811
RNA5SP493		-0.011	0.742982905	0.892549829
RNU6-42P		-0.011	0.742812008	0.89243498
RP11-289I10		-0.011	0.742795727	0.89243498
RP1-18D14		-0.011	0.742790318	0.89243498
RNU6-1144P		-0.011	0.742762342	0.89243498
ERICH6		-0.011	0.742631856	0.89240235
RNU6-217P		-0.011	0.742603409	0.89240235
RP11-13E5		-0.011	0.742562059	0.892382307
AC010888		-0.011	0.742518793	0.892352828
AC138304		-0.011	0.74250954	0.892352828
RPS3AP54		-0.011	0.742478054	0.892352828
IFNL4		-0.011	0.742313134	0.892218236
RP11-570H19		-0.011	0.742242909	0.892201379
RP11-78A18		-0.011	0.742210756	0.892185247
AC008740		-0.011	0.742159511	0.892185247
TECTA		-0.011	0.742149598	0.892185247
MIR5189		-0.011	0.742128992	0.892185247

LL22NC01-81G9	-0.011	0.742088195	0.892185247
SLC10A5P1	-0.011	0.742086223	0.892185247
MTND4P22	-0.011	0.742086152	0.892185247
MIR3691	-0.011	0.742069619	0.892185247
RP11-128P10	-0.011	0.74206407	0.892185247
TTC39B	-0.011	0.742027824	0.892185247
U85056	-0.011	0.741941361	0.892185247
RNU6-62P	-0.011	0.741901165	0.892185247
RP11-172F4	-0.011	0.741837454	0.892185247
MIR4769	-0.011	0.741829356	0.892185247
SNAI1P1	-0.011	0.741774285	0.892185247
MIR128-2	-0.011	0.741766777	0.892185247
RP11-359P14	-0.011	0.741639609	0.892145322
MIR4447	-0.011	0.741546443	0.892089478
RP11-457D2	-0.011	0.741472495	0.892063317
AC007395	-0.011	0.741380422	0.891975076
PPP1R12BP2	-0.011	0.741348364	0.891959039
FADS2	-0.011	0.741225928	0.891856789
RAB4B	-0.011	0.741191267	0.891837614
RN7SL130P	-0.011	0.74108044	0.891796167
RP11-697E2	-0.011	0.740979184	0.891762662
SQSTM1	-0.011	0.740894604	0.891683403
FABP5P15	-0.011	0.740855809	0.891659259
BMP3	-0.011	0.740829445	0.891659259
SLC31A2	-0.011	0.740758869	0.8916327
AC026423	-0.011	0.7406524	0.89154961
ATL2	-0.011	0.74064707	0.89154961
ANO8	-0.011	0.740605817	0.891538602
RP11-21J7	-0.011	0.740530795	0.891470822
JMJD6	-0.011	0.740507638	0.891465479
RPS2P35	-0.011	0.7404827	0.891457991
AC092798	-0.011	0.740416996	0.891423956
BNIP3P7	-0.011	0.740333816	0.891391411
WI2-80269A6	-0.011	0.740306052	0.891380516
TNRC18P2	-0.011	0.740217379	0.891318815
VSTM2A	-0.011	0.740025824	0.891245889
AQP12A	-0.011	0.740025503	0.891245889
RN7SL63P	-0.011	0.74000269	0.891245889
PRKRIRP3	-0.011	0.739979961	0.891245889
NOX1	-0.011	0.739952057	0.891245889
RNU6-792P	-0.011	0.739894818	0.891245871
LINC01056	-0.011	0.739682289	0.891109914
EIF4EP3	-0.011	0.739656465	0.891109914
LL0XNC01-105G4	-0.011	0.739637007	0.891109914
RP3-326I13	-0.011	0.739494941	0.891057166
RP11-254A17	-0.011	0.739440241	0.891013798
AC138811	-0.011	0.739376788	0.89095988
AP002530	-0.011	0.739367643	0.89095988
NFE2L1	-0.011	0.739236229	0.890934181
B4GALNT3	-0.011	0.739168616	0.890889353
AC011518	-0.011	0.739121167	0.890854709
NRG1-IT3	-0.011	0.739038627	0.890777768

OFD1P6Y	-0.011	0.738991362	0.890777768
TMEM30B	-0.011	0.738964554	0.890777768
AP001350	-0.011	0.73892869	0.890777768
RP11-399J13	-0.011	0.738871559	0.890756739
RP11-463J10	-0.011	0.7388128	0.890752158
FOXA3	-0.011	0.738806134	0.890752158
LDLRAD4	-0.011	0.738730007	0.890721369
CRY1	-0.011	0.738585747	0.890615068
LINC01344	-0.011	0.738551394	0.890596193
NDUFA5P1	-0.011	0.738484527	0.890538108
AL583784	-0.011	0.738455998	0.890526253
FAM72D	-0.011	0.738423338	0.890509415
RP3-521E19	-0.011	0.738397669	0.890501007
AQP12B	-0.011	0.738192441	0.890343683
IMP3P1	-0.011	0.738022756	0.890184111
LPIN3	-0.011	0.738013741	0.890184111
RP11-204E4	-0.011	0.73797351	0.8901698
RP11-218I7	-0.011	0.737939274	0.890151047
ILF3-AS1	-0.011	0.737922678	0.890151047
AOC2	-0.011	0.737856341	0.890112498
RP11-451O18	-0.011	0.737826868	0.890105632
RP11-442O1	-0.011	0.737769696	0.890059206
DDX11	-0.011	0.737709252	0.890031375
SYT4	-0.011	0.737651921	0.890031375
EPB41L4A	-0.011	0.737627677	0.890031375
AP000673	-0.011	0.737588567	0.890031375
NOP2	-0.011	0.737500377	0.889982276
OR2A41P	-0.011	0.737463525	0.889965507
NCOA7-AS1	-0.011	0.737398748	0.88992728
RPL7AP28	-0.011	0.737373173	0.88992728
HEXIM1	-0.011	0.737289463	0.88992728
RP11-753E22	-0.011	0.737263866	0.88992728
RP11-474N8	-0.011	0.73720014	0.88992728
RNY1P15	-0.011	0.73719885	0.88992728
CTD-2325A15	-0.011	0.737165067	0.88992728
AKAP17BP	-0.011	0.737151121	0.88992728
CTD-2316B1	-0.011	0.736981783	0.889913773
TBX19	-0.011	0.736976834	0.889913773
RP11-2N5	-0.011	0.736929734	0.889913773
RPL36AP45	-0.011	0.736901362	0.889913773
H1FX-AS1	-0.011	0.736812506	0.889873696
RN7SL254P	-0.011	0.736715689	0.889801895
LINC01580	-0.011	0.73666195	0.889759554
RNU6-819P	-0.011	0.736615726	0.889748851
RP5-828K20	-0.011	0.736573454	0.889748851
AP002884	-0.011	0.736560038	0.889748851
OR6V1	-0.011	0.736450119	0.889717027
HARS2	-0.011	0.73642953	0.889717027
PCAT18	-0.011	0.73636221	0.889690834
RNU7-52P	-0.011	0.736303064	0.889641941
RP11-67H24	-0.011	0.73618183	0.889518026
POU3F4	-0.011	0.736107604	0.889473471

GALNTL6	-0.011	0.7360826	0.889468372
TAS2R12	-0.011	0.736043208	0.889463358
DOLPP1	-0.011	0.735908295	0.889378091
MED15P6	-0.011	0.735854896	0.889348622
COX6A1P2	-0.011	0.735846957	0.889348622
AC011306	-0.011	0.735754548	0.889290459
AFF2-IT1	-0.012	0.735685903	0.889279777
RP11-209E8	-0.012	0.735400219	0.889047269
AC010883	-0.012	0.735367813	0.88903066
RPS20P15	-0.012	0.735148723	0.888860738
OR1J2	-0.012	0.735123392	0.888860738
KRTAP10-13P	-0.012	0.735053273	0.888830889
LINC01307	-0.012	0.735025001	0.888830889
SMIM13	-0.012	0.735017618	0.888830889
FYN	-0.012	0.735000334	0.888830889
RNU1-17P	-0.012	0.734993926	0.888830889
SNX3P1Y	-0.012	0.734979421	0.888830889
RN7SKP38	-0.012	0.734964757	0.888830889
AC004510	-0.012	0.734927843	0.888830889
SCML2P1	-0.012	0.73466136	0.888627722
GJE1	-0.012	0.734571703	0.888564409
ZNF251	-0.012	0.734563772	0.888564409
RFC5P1	-0.012	0.734426092	0.888433402
OR52A1	-0.012	0.734405268	0.888430777
RP11-77P16	-0.012	0.734367531	0.888407692
RP11-167N4	-0.012	0.734364112	0.888407692
AC002075	-0.012	0.734329012	0.888406225
RP13-379L11	-0.012	0.734259628	0.888344848
STK3	-0.012	0.734221084	0.888344848
MARCKSL1P1	-0.012	0.734207567	0.888344848
RP11-529H2	-0.012	0.733983216	0.888249143
RP11-597M17	-0.012	0.733945339	0.888249143
P2RY4	-0.012	0.733698069	0.888026377
RPL31P20	-0.012	0.7336553	0.887997178
TTC9	-0.012	0.733655126	0.887997178
ZNF618	-0.012	0.733532987	0.887894263
VN1R13P	-0.012	0.73350057	0.88787759
AC007339	-0.012	0.733328973	0.887760129
RP11-586K12	-0.012	0.73332014	0.887760129
KCNK18	-0.012	0.733241543	0.887760129
PCSK4	-0.012	0.733205259	0.887745752
AL353805	-0.012	0.733075164	0.887655933
RP11-465K4	-0.012	0.733043019	0.887655933
RPL14P3	-0.012	0.733042848	0.887655933
EEF1A1P26	-0.012	0.733030447	0.887655933
RP3-395C13	-0.012	0.732792991	0.887517337
RIMKLB	-0.012	0.732736163	0.887471079
RP11-534I8	-0.012	0.732618492	0.887373692
MCTP2	-0.012	0.732604621	0.887373692
RNU6-27P	-0.012	0.73243779	0.887267643
RPS21P8	-0.012	0.732430783	0.887267643
AC011551	-0.012	0.732427791	0.887267643

MIR633	-0.012	0.732335001	0.887210823
RP11-324F21	-0.012	0.732174721	0.887054395
RP11-135D11	-0.012	0.73217312	0.887054395
TRPM2	-0.012	0.732138529	0.887054395
OR6L1P	-0.012	0.732137644	0.887054395
LINC01425	-0.012	0.732074006	0.887030012
MIR3670-2	-0.012	0.731962908	0.886940528
MT-TI	-0.012	0.731956459	0.886940528
MIR200B	-0.012	0.731902311	0.886940528
LRRC37A12P	-0.012	0.731827766	0.886912161
RN7SL164P	-0.012	0.73173668	0.886846905
MTND2P21	-0.012	0.73173654	0.886846905
TPRG1-AS1	-0.012	0.731687022	0.886846905
MIR4433B	-0.012	0.731685999	0.886846905
DISP1	-0.012	0.731612958	0.886809793
RP11-354P17	-0.012	0.731570701	0.88680371
MIR3664	-0.012	0.73154032	0.886789453
MIR3667	-0.012	0.731524352	0.886789453
ACSS2	-0.012	0.731522157	0.886789453
RP11-157D18	-0.012	0.731209518	0.886523833
CTD-2116N24	-0.012	0.731182887	0.886514112
THYN1	-0.012	0.731101069	0.886437479
PRR33	-0.012	0.730988623	0.886368837
CPEB1-AS1	-0.012	0.730974104	0.886368837
RN7SL854P	-0.012	0.730883907	0.886354693
CTD-2258A20	-0.012	0.730777882	0.886271248
AD001527	-0.012	0.730704498	0.886204816
MIR4277	-0.012	0.730623788	0.886197198
C7orf26	-0.012	0.730621333	0.886197198
RP4-667H12	-0.012	0.730404738	0.886021762
PPP1R36	-0.012	0.730397519	0.886021762
OR6N2	-0.012	0.730393576	0.886021762
EMR2	-0.012	0.730239837	0.88595712
DPH2	-0.012	0.73023532	0.88595712
RP11-692D12	-0.012	0.730232035	0.88595712
NANS	-0.012	0.730229161	0.88595712
RP11-213G21	-0.012	0.730210462	0.88595712
RP11-313H3	-0.012	0.730178914	0.88595712
SMAD9-IT1	-0.012	0.730020402	0.885866082
RP4-552O12	-0.012	0.729963168	0.885824543
CASC22	-0.012	0.729815761	0.885668228
AC117945	-0.012	0.729685165	0.885554871
LINC01003	-0.012	0.729663408	0.885551031
AC092846	-0.012	0.729660518	0.885551031
RP11-635N19	-0.012	0.729603474	0.885545991
CTD-3131K8	-0.012	0.72955011	0.885503787
OR11L1	-0.012	0.729512843	0.88548112
RNU6-396P	-0.012	0.729470855	0.885475289
GCSHP1	-0.012	0.729393825	0.88544722
LINC00686	-0.012	0.72937917	0.88544722
LARS2-AS1	-0.012	0.729295123	0.885442511
RP11-59O2	-0.012	0.729192751	0.885340789

ACOT8	-0.012	0.729174958	0.885340789
RP11-309H21	-0.012	0.729174259	0.885340789
FAM47B	-0.012	0.729162161	0.885340789
XPO6	-0.012	0.729142946	0.885340789
SLITRK2	-0.012	0.729130568	0.885340789
MTND6P11	-0.012	0.729003528	0.885269021
RPL7AP73	-0.012	0.728977136	0.885269021
DEFB107B	-0.012	0.728950525	0.885269021
AC093756	-0.012	0.728950525	0.885269021
RN7SL148P	-0.012	0.728873902	0.885269021
SLC31A1P1	-0.012	0.728830325	0.885261837
RP1-52D1	-0.012	0.728716757	0.885173807
KLF7-IT1	-0.012	0.728709159	0.885173807
OR10AK1P	-0.012	0.728695223	0.885173807
RP11-78L16	-0.012	0.728564974	0.885153358
RP13-104F24	-0.012	0.728562294	0.885153358
LRSAM1	-0.012	0.728433874	0.885104339
SNORD115-43	-0.012	0.728412002	0.885104339
TERC	-0.012	0.728404767	0.885104339
FBRSL1	-0.012	0.728354281	0.885104339
FABP5P14	-0.012	0.728311303	0.885104339
NPM1P38	-0.012	0.728285126	0.885096218
RPL12P11	-0.012	0.728225582	0.885046437
CH17-302M23	-0.012	0.728143651	0.885011072
CNBD1	-0.012	0.728049705	0.884968182
ERBB2	-0.012	0.727841106	0.884804951
C1orf168	-0.012	0.727797483	0.884774504
AC108057	-0.012	0.727785212	0.884774504
AL160010	-0.012	0.727719226	0.884769703
RNU6-45P	-0.012	0.727703217	0.884769703
SRMP1	-0.012	0.727698546	0.884769703
TECRP2	-0.012	0.727615515	0.884756539
RNU6-554P	-0.012	0.727607224	0.884756539
RP11-216M21	-0.012	0.727509084	0.884694884
LINC01511	-0.012	0.727474225	0.884675082
AC003988	-0.012	0.727466778	0.884675082
RNA5SP168	-0.012	0.727435488	0.884673152
CHST15	-0.012	0.727141891	0.884429012
EIF3D	-0.012	0.72713451	0.884429012
MIR1228	-0.012	0.727076623	0.884417386
RFTN2	-0.012	0.726897526	0.884249213
OR56B2P	-0.012	0.726890155	0.884249213
RP1-128O3	-0.012	0.726868966	0.884249213
ZMYND12	-0.012	0.726792163	0.884206865
WDR6	-0.012	0.726672867	0.88415207
PSMB7	-0.012	0.726668767	0.88415207
AC016910	-0.012	0.726615119	0.884149568
RN7SL308P	-0.012	0.726557597	0.884141331
RP4-753D5	-0.012	0.726423438	0.884141331
RP1-134O19	-0.012	0.726419243	0.884141331
RNU6-766P	-0.012	0.726402491	0.884141331
RN7SL8P	-0.012	0.726312303	0.884097326

RP11-362F19	-0.012	0.726248598	0.884064971
MIR4522	-0.012	0.726143118	0.88404817
AC135068	-0.012	0.726141059	0.88404817
LINC00671	-0.012	0.72611554	0.88404817
PRPF6	-0.012	0.726103944	0.88404817
IFT80	-0.012	0.726085501	0.88404817
ARL6IP1P1	-0.012	0.726003788	0.884038106
NOL6	-0.012	0.725974031	0.884024472
RP11-307L14	-0.012	0.725973211	0.884024472
SERPINA5	-0.012	0.725921576	0.884024472
LINC00370	-0.012	0.725768103	0.883931912
AE000662	-0.012	0.725718368	0.883893942
ALMS1-IT1	-0.012	0.725705214	0.883893942
RAP2CP1	-0.012	0.7256547	0.883861716
CERS4	-0.012	0.725648668	0.883861716
RNU4-67P	-0.012	0.725515923	0.883805582
AC139085	-0.012	0.725501325	0.883805582
MTND4P31	-0.012	0.725493996	0.883805582
FTH1P12	-0.012	0.725432743	0.883805582
RP11-461H3	-0.012	0.725415583	0.883805582
RNA5SP128	-0.012	0.725392161	0.883805582
GPC5-IT1	-0.012	0.725337356	0.883805582
KNOP1P5	-0.012	0.72517136	0.883747508
RP11-767C4	-0.012	0.725154155	0.883747508
ANKRD16	-0.012	0.72491453	0.88359278
ADRA1A	-0.012	0.724836885	0.883520751
MIR8073	-0.012	0.724806902	0.883506816
CYCS	-0.012	0.724730625	0.883446433
RP11-359D24	-0.012	0.724695127	0.883438399
OR8D2	-0.012	0.724616865	0.883388216
AL592310	-0.012	0.724482477	0.883292937
RP11-48D4	-0.012	0.724415065	0.883255243
NXT1P1	-0.012	0.724398542	0.883255243
RP11-2C15	-0.012	0.724367244	0.88324216
SLC15A3	-0.012	0.724329694	0.883218985
RP3-322G13	-0.012	0.724147998	0.883110481
RP3-433F14	-0.012	0.724106854	0.883082916
SPATA42	-0.012	0.724100363	0.883082916
RNU1-69P	-0.012	0.724044578	0.883082916
RP11-171L9	-0.012	0.724036089	0.883082916
RP11-473I1	-0.012	0.724004939	0.883082916
RP11-339F13	-0.012	0.723967048	0.883082916
KRT18P3	-0.012	0.723930165	0.883082916
RPL5P13	-0.012	0.72389708	0.883082916
DEFA10P	-0.012	0.723840938	0.883082916
MIR3680-2	-0.012	0.723763196	0.883048206
AC009234	-0.012	0.72374802	0.883048206
FABP9	-0.012	0.723714308	0.883033799
VPS26AP1	-0.012	0.723535179	0.882909742
MADCAM1	-0.012	0.723520473	0.882909742
MRPS36P2	-0.012	0.723513917	0.882909742
RNU6-1212P	-0.012	0.723492673	0.882909742

LL22NC03-22A12	-0.012	0.723484585	0.882909742
GAPDHP38	-0.012	0.723393422	0.882891077
PANK1	-0.012	0.723257503	0.882770433
ITPKB	-0.012	0.723251363	0.882770433
VN1R37P	-0.012	0.723207513	0.882754664
FAM21FP	-0.012	0.723115894	0.882665454
VN1R6P	-0.012	0.723087322	0.882663616
SLC9B1P2	-0.012	0.722940415	0.882609601
HMGB1P27	-0.012	0.722833325	0.88256174
RP1-120G22	-0.012	0.722792989	0.882542729
ENPP5	-0.012	0.722778692	0.882542729
MIR24-2	-0.012	0.722729016	0.882532494
ZNF277	-0.012	0.722721815	0.882532494
AC018717	-0.012	0.722688437	0.882532494
MIR548A3	-0.012	0.722616242	0.882507917
RP11-545P6	-0.012	0.722600364	0.882507917
OR51F2	-0.012	0.722589923	0.882507917
NOTCH2P1	-0.012	0.722572964	0.882507917
SPATA31D4	-0.012	0.722457395	0.88242706
RP11-476H16	-0.012	0.722321914	0.882329464
SALL4P2	-0.012	0.72222225	0.882252978
RP11-379L18	-0.012	0.722177419	0.882245908
LINC01445	-0.012	0.722109463	0.882245908
SDR16C6P	-0.012	0.721873749	0.882030864
IFFO2	-0.012	0.721864751	0.882030864
ALDH7A1P3	-0.012	0.721849666	0.882030864
BEND3	-0.012	0.721814852	0.882026784
RP11-439L18	-0.012	0.721758475	0.882020926
HSD3BP4	-0.012	0.721505589	0.881897773
TMEM230	-0.012	0.721375624	0.881761545
RP11-736K20	-0.012	0.721336368	0.881758821
VN1R12P	-0.012	0.721121486	0.881586658
RP11-189G24	-0.012	0.721087121	0.881567275
RP11-631N16	-0.012	0.721017439	0.881504713
AC009227	-0.012	0.720951121	0.881446259
RP11-136H13	-0.012	0.720900998	0.881430232
RP11-744K17	-0.012	0.720868303	0.881430232
ALPPL2	-0.012	0.720825836	0.881406215
AKIRIN2P1	-0.012	0.720663814	0.881298605
CHMP2B	-0.012	0.720661768	0.881298605
CNTN4-AS1	-0.012	0.720653192	0.881298605
AL512359	-0.012	0.720634519	0.881298605
LINC00358	-0.012	0.7204458	0.881160791
NUP210P1	-0.012	0.720391266	0.881146305
SUCLG2	-0.012	0.720309836	0.881069332
OR7A1P	-0.012	0.720207316	0.881057069
TERF1P1	-0.012	0.720196055	0.881057069
RP11-39M21	-0.012	0.720172929	0.881057069
GUSBP3	-0.012	0.720063537	0.881016958
RP5-834N19	-0.012	0.719993507	0.880953905
FAM90A8P	-0.012	0.719938575	0.880931956
DAP3P1	-0.012	0.719935286	0.880931956

ABC12-47964100C23	-0.012	0.719931702	0.880931956
AC004980	-0.012	0.719887677	0.880931956
NEK10	-0.012	0.719837008	0.880920838
SNORD14E	-0.012	0.719698133	0.880864055
PPIAP7	-0.012	0.719697037	0.880864055
ANKRD26P3	-0.012	0.719690887	0.880864055
AC005086	-0.012	0.719541154	0.880830374
TLR3	-0.012	0.719526887	0.880830374
OR4U1P	-0.012	0.719513866	0.880830374
RP5-1103G7	-0.012	0.719455456	0.880795304
RNY4P9	-0.012	0.719442337	0.880795304
REPS2	-0.012	0.719374499	0.880784835
RP11-794P6	-0.012	0.719333115	0.88077945
AC093602	-0.012	0.719307421	0.88077945
TRAJ43	-0.012	0.719301884	0.88077945
SNRPD3	-0.012	0.719188626	0.88077945
ARL5AP4	-0.012	0.719083866	0.880768627
RP11-1049H7	-0.012	0.719033984	0.88073018
RP11-222N13	-0.012	0.718830914	0.88057203
RNA5SP252	-0.012	0.718798096	0.880566846
OCIAD2	-0.012	0.71878058	0.880566846
AGTR2	-0.012	0.718718709	0.880566846
RP11-23E10	-0.012	0.718700967	0.880566846
AC007557	-0.012	0.718674425	0.880566846
RP1-158P9	-0.012	0.718673678	0.880566846
ARHGAP19-SLIT1	-0.012	0.718593097	0.880563986
FAM86B2	-0.012	0.718589979	0.880563986
RP11-759A9	-0.012	0.718577475	0.880563986
RPL9P18	-0.012	0.718361346	0.880385785
TBCA	-0.012	0.718144368	0.88025411
AC004057	-0.012	0.718124787	0.88025411
IFNA16	-0.012	0.718115747	0.88025411
RNU6-123P	-0.012	0.718110495	0.88025411
SUB1	-0.012	0.717992489	0.880224157
SIGLEC30P	-0.012	0.717849368	0.88011268
GOLGA2P11	-0.012	0.717841813	0.88011268
MIR521-1	-0.012	0.717779538	0.880076358
RNU6-287P	-0.012	0.717745163	0.880056867
DICER1-AS1	-0.012	0.717700196	0.880036153
ZNF117	-0.012	0.717545085	0.879902154
MYT1L	-0.012	0.717525283	0.879900527
AP002495	-0.012	0.717300159	0.879669757
TAAR4P	-0.012	0.717221997	0.879635074
LINC00393	-0.012	0.717207346	0.879635074
RP11-804M7	-0.012	0.717197137	0.879635074
PLAA	-0.012	0.717090024	0.879635074
VDAC2	-0.012	0.717058602	0.879635074
YY1P1	-0.012	0.716993376	0.879633279
AC073043	-0.012	0.716910119	0.879618403
RP11-153I24	-0.012	0.716896586	0.879618403
RP11-393I23	-0.012	0.716894424	0.879618403
FAM86C2P	-0.012	0.716755832	0.879500439

MIR3617	-0.012	0.71673123	0.879500439
RP11-756J15	-0.012	0.716722999	0.879500439
TMEM25	-0.012	0.716710543	0.879500439
FCF1P4	-0.012	0.716707415	0.879500439
CU104787	-0.012	0.716693206	0.879500439
MTATP6P7	-0.012	0.71666984	0.879500439
NMRK1	-0.012	0.716586513	0.879500439
PRR30	-0.012	0.716559234	0.879500439
PICALM	-0.012	0.716464122	0.879482377
FAUP2	-0.012	0.716462074	0.879482377
TSPY2	-0.012	0.716450939	0.879482377
AC128715	-0.012	0.716284235	0.879352217
CDK10	-0.012	0.716282589	0.879352217
AC012074	-0.012	0.716191211	0.879306012
FTLP17	-0.012	0.71609476	0.879221614
SIRPAP1	-0.012	0.716040704	0.879189219
RP11-49C9	-0.012	0.715993579	0.879176688
SUMO1	-0.012	0.715946896	0.879164906
PRPS2	-0.012	0.715904072	0.879164906
COX19	-0.012	0.71589306	0.879164906
KIF4B	-0.012	0.715885799	0.879164906
TCEB1P5	-0.012	0.715733049	0.87915144
TYK2	-0.012	0.7156239	0.879057321
RP11-707M3	-0.012	0.715616968	0.879057321
OFD1P4Y	-0.012	0.715592361	0.879057321
PRELID1P2	-0.012	0.715576242	0.879057321
AL390071	-0.012	0.715450541	0.879053813
MIR4747	-0.012	0.715292349	0.878904794
RP11-956E11	-0.012	0.715269535	0.878899436
RP11-519G16	-0.012	0.715172118	0.878847755
AC010323	-0.012	0.714826463	0.878621681
RP6-204F4	-0.012	0.714782177	0.878621681
C17orf104	-0.012	0.71470413	0.878590032
RP11-359B12	-0.012	0.714649511	0.878571883
SENP8	-0.012	0.714647473	0.878571883
RNU6-26P	-0.012	0.714514817	0.878493358
UBL5P4	-0.012	0.714480307	0.878473605
CALHM2	-0.012	0.714455097	0.878465287
AC112229	-0.012	0.714439522	0.878465287
MIR20B	-0.012	0.714370752	0.878413943
GPR6	-0.012	0.714321426	0.878404663
PTPN23	-0.012	0.714288004	0.878404663
SDHCP4	-0.012	0.714200464	0.878378967
SNORA70E	-0.012	0.714177825	0.878378967
NFIX	-0.012	0.714135596	0.878368236
TAF1A	-0.012	0.714125001	0.878368236
KYNUP2	-0.012	0.714098767	0.878368236
MBOAT2	-0.012	0.714076102	0.878368236
RP4-650F12	-0.012	0.714068557	0.878368236
LL22NC03-63E9	-0.012	0.714056007	0.878368236
RPL12P4	-0.012	0.714034132	0.878368236
SEMA3F	-0.012	0.71403291	0.878368236

ZNF438	-0.012	0.714028835	0.878368236
AC007056	-0.012	0.713952527	0.878368236
CYCSP45	-0.012	0.713872614	0.878368236
RP11-708H21	-0.012	0.713771979	0.878368236
MTATP6P1	-0.012	0.713760328	0.878368236
C14orf105	-0.012	0.713725541	0.878361922
RP11-312P12	-0.013	0.713531374	0.878183887
RP11-428P16	-0.013	0.713494962	0.878183887
CTD-2517O10	-0.013	0.713476376	0.878183887
RP11-392C20	-0.013	0.713390435	0.878180306
CRYGA	-0.013	0.713379886	0.878180306
RNU6-675P	-0.013	0.713201656	0.878080221
RP11-249M12	-0.013	0.71311329	0.878001583
OR11H13P	-0.013	0.713068223	0.87798403
LINC00891	-0.013	0.713042864	0.877975504
CASP6	-0.013	0.712983526	0.877947836
PHTF2	-0.013	0.712973752	0.877947836
HMGCL	-0.013	0.712954611	0.877947836
LINC00440	-0.013	0.712830804	0.877876779
C14orf132	-0.013	0.712728595	0.877815485
AC007611	-0.013	0.712716253	0.877815485
NF1P2	-0.013	0.712702297	0.877815485
KLB	-0.013	0.7126724	0.877815485
RNU6-1251P	-0.013	0.712517063	0.877713839
POLD4	-0.013	0.712500161	0.877713839
KCTD1	-0.013	0.712369872	0.877623319
RN7SKP67	-0.013	0.712339971	0.877615926
GPR179	-0.013	0.712310965	0.877615926
AC019172	-0.013	0.712247472	0.877586029
RAC1P6	-0.013	0.712242293	0.877586029
DLD	-0.013	0.712229413	0.877586029
APOOP4	-0.013	0.712226942	0.877586029
MIR5585	-0.013	0.71221015	0.877586029
MIR4284	-0.013	0.712188506	0.877586029
TG	-0.013	0.712071867	0.877586029
AC025540	-0.013	0.712022658	0.877558759
CUBNP1	-0.013	0.711973589	0.877520991
UBE2V1P1	-0.013	0.711931188	0.877515174
PHBP1	-0.013	0.711905756	0.877515174
RPS3AP17	-0.013	0.711825907	0.877475217
RN7SL563P	-0.013	0.711817534	0.877475217
RNU7-90P	-0.013	0.711705686	0.877451536
RPS3AP1	-0.013	0.711622354	0.877405972
RP1-69D17	-0.013	0.711506212	0.877285483
SMIM1	-0.013	0.711465407	0.877257882
GOT2P2	-0.013	0.711406289	0.87723041
BTBD9-AS1	-0.013	0.711382258	0.87723041
TUBG2	-0.013	0.71131199	0.877204975
UBE2H	-0.013	0.711252986	0.877179511
RP11-285F16	-0.013	0.711216615	0.877179511
RNU6-107P	-0.013	0.711115714	0.877121905
CTB-88F18	-0.013	0.711072121	0.87709085

KIF13B	-0.013	0.710999493	0.87709085
ISCA1	-0.013	0.710994611	0.87709085
FAM99A	-0.013	0.710983923	0.87709085
MC5R	-0.013	0.710969743	0.87709085
RP11-431N15	-0.013	0.71094991	0.87709085
ECEL1	-0.013	0.710944866	0.87709085
CCM2L	-0.013	0.710939278	0.87709085
AP5B1	-0.013	0.710909171	0.87709085
MIR551B	-0.013	0.710903844	0.87709085
RNU6-1317P	-0.013	0.710901774	0.87709085
ATP6AP1	-0.013	0.710871742	0.87709085
HOXC4	-0.013	0.710869301	0.87709085
RP11-94B19	-0.013	0.710851791	0.87709085
ZMAT2	-0.013	0.710802173	0.87709085
RP11-314N2	-0.013	0.710676122	0.87709085
AC068754	-0.013	0.710660354	0.87709085
RP11-362M19	-0.013	0.710649103	0.87709085
RP11-567M21	-0.013	0.710569221	0.87709085
C15orf26	-0.013	0.710566353	0.87709085
VNN3	-0.013	0.710461582	0.87709085
CTC-280C13	-0.013	0.710435255	0.87709085
LINC00959	-0.013	0.71041654	0.87709085
TXNDC9	-0.013	0.710353495	0.87709085
RP6-60B16	-0.013	0.709949393	0.876750498
MIR609	-0.013	0.709908913	0.876723241
MIR4422	-0.013	0.709903297	0.876723241
LINC01477	-0.013	0.709714746	0.87664257
FP325318	-0.013	0.709674398	0.876638204
AC137527	-0.013	0.709632616	0.876638204
AC104794	-0.013	0.709580489	0.876638204
RP5-967N21	-0.013	0.709547917	0.876638204
RP11-162K11	-0.013	0.709476438	0.876598291
PNKDP1	-0.013	0.709443297	0.876580084
HMGN1P17	-0.013	0.7093578	0.876546499
PODXL2	-0.013	0.709334479	0.876546499
OR7E122P	-0.013	0.709306227	0.876546499
HYAL1	-0.013	0.709267809	0.876546499
OR6C71P	-0.013	0.709262115	0.876546499
KIAA0922	-0.013	0.709256427	0.876546499
C5orf52	-0.013	0.709194952	0.876546114
ZNF479	-0.013	0.709177505	0.876546114
MYOM1	-0.013	0.70911334	0.876536232
MRI1	-0.013	0.708806063	0.876354303
RNU1-108P	-0.013	0.708798388	0.876354303
RN7SL140P	-0.013	0.708740122	0.876354303
RP5-844F9	-0.013	0.708719795	0.876354303
RPL5P21	-0.013	0.708600418	0.876243337
CTDSP1	-0.013	0.708571979	0.876230919
POM121L3P	-0.013	0.708557131	0.876230919
AC108938	-0.013	0.708536511	0.876230919
CCDC24	-0.013	0.708528091	0.876230919
ST7-AS1	-0.013	0.708452509	0.876219685

RPS17P1	-0.013	0.708334918	0.876119752
RP11-491F9	-0.013	0.708275041	0.876091196
NUDT16L1	-0.013	0.70813477	0.87598594
AC006478	-0.013	0.707983012	0.875843708
BX323860	-0.013	0.707983012	0.875843708
AC116165	-0.013	0.707983012	0.875843708
EPS8	-0.013	0.707840237	0.87580716
PITPNM2	-0.013	0.707833737	0.87580716
CTD-2277K2	-0.013	0.707798257	0.87580716
MRPS6P4	-0.013	0.707740234	0.875793601
VENTXP7	-0.013	0.707730278	0.875793601
RN7SL146P	-0.013	0.707658343	0.875764293
MIR2392	-0.013	0.707656104	0.875764293
RP11-157L10	-0.013	0.707616689	0.875764293
LRRC45	-0.013	0.707602204	0.875764293
TPTE2P3	-0.013	0.707567748	0.875764293
KRTAP3-3	-0.013	0.707562076	0.875764293
PRPH	-0.013	0.707511284	0.875764293
RNU1-78P	-0.013	0.707492286	0.875764293
CTD-2281M20	-0.013	0.707357635	0.875752478
XIRP2-AS1	-0.013	0.707353935	0.875752478
RP11-79C23	-0.013	0.707241334	0.875699546
RP11-134K1	-0.013	0.707158101	0.875668978
LINC00240	-0.013	0.707145717	0.875668978
RNU6-74P	-0.013	0.707128022	0.875668978
RP11-324P9	-0.013	0.707057319	0.875668978
KRT18P14	-0.013	0.707028671	0.875668978
APOA2	-0.013	0.706948066	0.875609589
LILRB5	-0.013	0.706907768	0.875582447
TGFA-IT1	-0.013	0.706857907	0.875543459
RP11-706O15	-0.013	0.7068493	0.875543459
RP11-788H18	-0.013	0.706736263	0.87546109
OR5M8	-0.013	0.706479685	0.875211548
CTC-463N11	-0.013	0.706448258	0.875211548
PTGFR	-0.013	0.706358409	0.875176101
VTRNA1-2	-0.013	0.706352179	0.875176101
RPL29P28	-0.013	0.706335495	0.875176101
RP11-615J4	-0.013	0.706202217	0.875118203
AL391827	-0.013	0.706166797	0.875097079
SUSD1	-0.013	0.706116569	0.875057604
RP4-751H13	-0.013	0.706054066	0.875025683
RNU6-1094P	-0.013	0.70600137	0.874983144
AC145124	-0.013	0.705828988	0.874883333
AC011366	-0.013	0.705664716	0.874764548
CH17-270A2	-0.013	0.705659411	0.874764548
RP11-580I1	-0.013	0.705557828	0.87469128
RP11-219D15	-0.013	0.705418846	0.874579809
Z83001	-0.013	0.705158175	0.874360704
RNU6-873P	-0.013	0.705127946	0.874360704
AATK	-0.013	0.705105401	0.874360704
RP11-131O15	-0.013	0.705088543	0.874360704
ACSM2B	-0.013	0.705068076	0.874360704

RP11-214F16	-0.013	0.705026624	0.874360406
AC122136	-0.013	0.705006897	0.874360406
AC079790	-0.013	0.704925119	0.874309079
AC004775	-0.013	0.704913909	0.874309079
GIGYF1	-0.013	0.704746018	0.874276924
UAP1	-0.013	0.704725739	0.874276924
PPIAP27	-0.013	0.704721809	0.874276924
RP11-422P22	-0.013	0.704714727	0.874276924
MTND1P4	-0.013	0.704569059	0.874276924
ZNF177	-0.013	0.704438441	0.874251866
INS-IGF2	-0.013	0.704332555	0.874238013
KRTAP2-4	-0.013	0.704332	0.874238013
RP13-582L3	-0.013	0.704331769	0.874238013
AC022173	-0.013	0.704296203	0.874238013
SH3TC1	-0.013	0.704229774	0.874220701
NAP1L4P1	-0.013	0.704191096	0.874195473
RP11-455B3	-0.013	0.704188564	0.874195473
SNORD114-23	-0.013	0.704119045	0.874195473
NDUFS5P3	-0.013	0.70406785	0.874195473
AC012671	-0.013	0.703929746	0.874075183
AL353997	-0.013	0.703794861	0.87401922
HMGB1P26	-0.013	0.703792739	0.87401922
IL23A	-0.013	0.703731096	0.873988915
CHEK2	-0.013	0.703705557	0.873979988
ZNF705A	-0.013	0.703701585	0.873979988
MIR4463	-0.013	0.703556292	0.873907894
RP11-465L10	-0.013	0.703511724	0.873889868
MAPK15	-0.013	0.703459918	0.873857212
SFTA1P	-0.013	0.703417286	0.873849838
AC093826	-0.013	0.703338341	0.873797349
LAMP5-AS1	-0.013	0.703276618	0.873766251
MIR4666A	-0.013	0.703265174	0.873766251
CTD-2374C24	-0.013	0.70319953	0.873739508
FBRS	-0.013	0.703143388	0.873714684
RPL30P4	-0.013	0.703123335	0.873712562
PDE4DIP	-0.013	0.703058691	0.87370062
RN7SKP22	-0.013	0.702785994	0.873430107
RP11-351D16	-0.013	0.702775451	0.873430107
AL024509	-0.013	0.702758	0.873430107
AURKB	-0.013	0.702661194	0.873387241
RP11-546M4	-0.013	0.702648408	0.873387241
SKP1P3	-0.013	0.702591275	0.873347636
RP11-293E1	-0.013	0.702562196	0.873344028
AL031601	-0.013	0.702535386	0.873344028
NOX3	-0.013	0.702523827	0.873344028
MIR548AB	-0.013	0.702499244	0.873344028
SSX7	-0.013	0.70247634	0.873344028
PPAP2B	-0.013	0.702421357	0.873341564
MT-TE	-0.013	0.702372585	0.873318683
SNORD36C	-0.013	0.702295172	0.873298664
RP11-782E2	-0.013	0.702273431	0.873294431
RP5-1028L10	-0.013	0.702205116	0.873267649

TRAPPC2L	-0.013	0.702166967	0.873267649
TXNDC15	-0.013	0.701990373	0.873102015
AARS	-0.013	0.701949872	0.873074442
RP11-212F11	-0.013	0.701942346	0.873074442
RP11-476M19	-0.013	0.701899159	0.873056966
RN7SL691P	-0.013	0.701831024	0.872998096
LINC01290	-0.013	0.701818386	0.872998096
RP4-666O22	-0.013	0.701793627	0.872998096
MIR373	-0.013	0.701676088	0.872961889
AC233702	-0.013	0.701576293	0.872883337
RPS3P1	-0.013	0.701543538	0.872865385
NPM1P46	-0.013	0.701532572	0.872865385
MIR646	-0.013	0.701500406	0.872865385
FNTAL1	-0.013	0.701444908	0.872833877
RP4-813D12	-0.013	0.701335504	0.872783969
RNU6-213P	-0.013	0.701314778	0.872783969
RP11-12A16	-0.013	0.701253655	0.872778317
ACTG1P12	-0.013	0.701228507	0.872769825
RP11-4K16	-0.013	0.701186855	0.872769825
KAZALD1	-0.013	0.701156221	0.872769825
RN7SKP205	-0.013	0.701112626	0.872739631
RP11-24J19	-0.013	0.701107876	0.872739631
GTPBP4	-0.013	0.701101163	0.872739631
TRAF6P1	-0.013	0.701038909	0.872716298
OVCH2	-0.013	0.701032688	0.872716298
VN1R70P	-0.013	0.701029812	0.872716298
FAM71BP1	-0.013	0.701015019	0.872716298
MIR4777	-0.013	0.700787594	0.872540279
NRXN1	-0.013	0.700709437	0.872465775
DCAF6	-0.013	0.700671282	0.872441076
RNU6-668P	-0.013	0.700621152	0.872441076
MIR6745	-0.013	0.700599057	0.872441076
AC005487	-0.013	0.700476812	0.872358579
RP5-1119O21	-0.013	0.700468604	0.872358579
SLC29A1	-0.013	0.700439785	0.872358088
ARAFP1	-0.013	0.700422442	0.872358088
HNRNPH1P3	-0.013	0.700392493	0.872358088
LINC01069	-0.013	0.700254698	0.872310059
ANKRD26P1	-0.013	0.700187679	0.872272203
RP11-338L18	-0.013	0.700172501	0.872272203
CDRT15P1	-0.013	0.699999316	0.872105981
RNF19BPY	-0.013	0.699977192	0.87210123
MC4R	-0.013	0.699868772	0.872011772
SPATA41	-0.013	0.699826776	0.871982259
IL27RA	-0.013	0.699826776	0.871982259
CTD-2516K3	-0.013	0.699732105	0.871932732
RP11-10H3	-0.013	0.699522385	0.871694208
F8	-0.013	0.699436414	0.871632688
RP11-129G17	-0.013	0.699431382	0.871632688
RNA5SP500	-0.013	0.699396405	0.871628443
BNC1	-0.013	0.699289072	0.871517483
MRPS16P3	-0.013	0.699027429	0.871281415

PLXNA3	-0.013	0.698985471	0.871253102
SLC36A2	-0.013	0.698961998	0.871246645
PRB1	-0.013	0.698866695	0.871196254
AC005926	-0.013	0.698857526	0.871196254
AC006452	-0.013	0.698767247	0.871163491
RP11-300I6	-0.013	0.698739376	0.871151548
MAGEB5	-0.013	0.698666802	0.871106672
RP11-617O8	-0.013	0.698560811	0.870997323
PSMC1P5	-0.013	0.698541516	0.870997323
NIFKP1	-0.013	0.698341282	0.870852353
AC011380	-0.013	0.698284108	0.870811899
RPSAP9	-0.013	0.698262163	0.870807333
RP11-301G19	-0.013	0.698255374	0.870807333
RPL36AP53	-0.013	0.698216409	0.870807333
HDAC1P1	-0.013	0.698215525	0.870807333
RP11-588H23	-0.013	0.698168271	0.870807333
CACNA1I	-0.013	0.698127154	0.870807333
MIR1254-2	-0.013	0.698122526	0.870807333
CA2	-0.013	0.698106136	0.870807333
RARRES3	-0.013	0.69785474	0.870618392
FAM133CP	-0.013	0.697831806	0.870618392
MT-ND6	-0.013	0.697799373	0.870617734
RNA5SP185	-0.013	0.697794809	0.870617734
TMEM97P2	-0.013	0.697775964	0.870617734
RP11-528M18	-0.013	0.697749893	0.870617734
DYNLL1P5	-0.013	0.697732173	0.870617734
OXLD1	-0.013	0.697708041	0.870617734
MIR617	-0.013	0.697697793	0.870617734
AL035419	-0.013	0.697506698	0.870493398
CTD-2564P9	-0.013	0.697470258	0.870480752
PGM3	-0.013	0.697346796	0.870349473
ATP6V0E2-AS1	-0.013	0.697346392	0.870349473
RP11-615I16	-0.013	0.697339301	0.870349473
OR9Q2	-0.013	0.697268086	0.870349473
SLC3A1	-0.013	0.697112931	0.870217219
RP11-8J23	-0.013	0.697098481	0.870217219
CDKL4	-0.013	0.69703527	0.870209724
RNU6-932P	-0.013	0.696993643	0.870187609
ZNF232	-0.013	0.696951284	0.870187609
CENPUP1	-0.013	0.696869833	0.87018745
SNORD116-26	-0.013	0.696782331	0.870146628
SERBP1P5	-0.013	0.696738769	0.870138808
RP1-92C8	-0.013	0.696718337	0.870138808
PABPC1P6	-0.013	0.696707193	0.870138808
MIR491	-0.013	0.696670808	0.870138808
A4GNT	-0.013	0.696640477	0.870138808
AP000435	-0.013	0.696486845	0.869982925
RP11-169I9	-0.013	0.696399863	0.869897091
ORC5	-0.013	0.696298202	0.869815728
TUSC2	-0.013	0.696204115	0.869721007
SDR42E1	-0.013	0.6961494	0.869721007
RNU6-425P	-0.013	0.695969863	0.869596645

CNDP2	-0.013	0.695959834	0.869596645
HHAT	-0.013	0.695951759	0.869596645
EFHB	-0.013	0.695947721	0.869596645
RNU6-565P	-0.013	0.695841439	0.869558912
GSTA11P	-0.013	0.695743246	0.869487368
RPL3P13	-0.013	0.695723273	0.869485222
AL022336	-0.013	0.695689412	0.869485222
RP11-573C10	-0.013	0.695688629	0.869485222
AC004129	-0.013	0.695640887	0.869485222
RNU6-514P	-0.013	0.695595195	0.869484865
RP11-44F14	-0.013	0.695340189	0.869335263
MIR3611	-0.013	0.695314527	0.869335263
AC007317	-0.013	0.695280968	0.869335263
RP4-781B1	-0.013	0.695210362	0.869300425
EFCAB10	-0.013	0.695187226	0.869294319
CTD-2320B12	-0.013	0.695180676	0.869294319
THOC3	-0.013	0.695176733	0.869294319
RNU6-234P	-0.013	0.695065729	0.869279331
RN7SL392P	-0.013	0.695056824	0.869279331
CTB-167B5	-0.013	0.695000833	0.86924382
AC111186	-0.013	0.694938594	0.869188802
RP11-108F13	-0.013	0.694931714	0.869188802
RP11-618P17	-0.013	0.694892495	0.869176796
RP11-289H16	-0.013	0.694856027	0.869176796
RP11-158L12	-0.013	0.694853496	0.869176796
CATIP-AS1	-0.013	0.694827114	0.869176796
MIR95	-0.013	0.694819698	0.869176796
MIR212	-0.013	0.694766715	0.869176796
TM2D3	-0.013	0.694621897	0.869135065
C1DP3	-0.013	0.694287312	0.868995122
RP11-433B3	-0.013	0.694231348	0.868995122
AC099326	-0.013	0.694226956	0.868995122
LRP11	-0.013	0.694173709	0.868995122
RP11-279L11	-0.013	0.694041387	0.868933759
RP11-141J13	-0.013	0.693987318	0.868911748
RP11-1072N2	-0.013	0.693978157	0.868911748
MIR361	-0.013	0.693955694	0.868911748
LINC00612	-0.013	0.693867202	0.868875565
CCT6P2	-0.013	0.69367978	0.86866371
RP3-347M6	-0.013	0.693599124	0.868608385
SIGLEC28P	-0.013	0.693567087	0.868591104
RP11-844G16	-0.013	0.693378254	0.868400287
GH1	-0.013	0.693305751	0.868355152
PSMG4	-0.013	0.69311115	0.868202745
CARS2	-0.013	0.693082528	0.868189726
RNU6-187P	-0.013	0.693043195	0.868163288
AC084872	-0.013	0.693037777	0.868163288
LINC00846	-0.013	0.692873737	0.868042336
ALG14	-0.013	0.69279512	0.867966676
MIR339	-0.013	0.692788537	0.867966676
RP11-484L8	-0.013	0.69277386	0.867966676
RN7SL399P	-0.013	0.692768097	0.867966676

RNU6-795P	-0.013	0.69274748	0.867966676
OR9G1	-0.013	0.692538791	0.867896669
KRT17P4	-0.013	0.692524947	0.867896669
ACAD8	-0.013	0.692491233	0.867882742
EEF1A1P12	-0.013	0.692452546	0.867857093
NF1P7	-0.013	0.692439715	0.867857093
CRTAC1	-0.013	0.692424827	0.867857093
AL590822	-0.013	0.692215581	0.867674267
SLC45A1	-0.013	0.692104393	0.86755773
SCNN1B	-0.013	0.692065311	0.867554406
DDX6P1	-0.013	0.692011107	0.867509292
AL359737	-0.014	0.691910855	0.867406445
AOX1	-0.014	0.691796993	0.867309363
RNU6-1163P	-0.014	0.69172484	0.86726185
RNU6-424P	-0.014	0.691712193	0.86726185
RP11-734K21	-0.014	0.691698988	0.86726185
AL137792	-0.014	0.691302444	0.866963201
SYNPR-AS1	-0.014	0.691276047	0.866952926
RNU6-291P	-0.014	0.691205104	0.866917331
SLC25A5P4	-0.014	0.691151159	0.866917331
RNA5SP378	-0.014	0.691144268	0.866917331
RN7SL300P	-0.014	0.691119261	0.866917331
ITIH4-AS1	-0.014	0.691118214	0.866917331
RNU6-601P	-0.014	0.691097911	0.866917331
UFSP2	-0.014	0.691050661	0.866917331
FGD5	-0.014	0.690994688	0.866896821
CTD-3199J23	-0.014	0.690968106	0.866886307
RNA5SP282	-0.014	0.690925932	0.866864501
RP11-16L21	-0.014	0.690873769	0.866836455
SYNJ2BP-COX16	-0.014	0.69086181	0.866836455
SLC9A3R2	-0.014	0.690568705	0.866636339
PPIAP10	-0.014	0.690219973	0.866335661
AC009406	-0.014	0.690184402	0.866313844
SLC25A3	-0.014	0.690154894	0.866299638
MIR503	-0.014	0.69011198	0.866268601
RPS10P27	-0.014	0.690079242	0.866250338
C19orf40	-0.014	0.6900748	0.866250338
RP11-699L21	-0.014	0.690034588	0.866250338
RP11-375D13	-0.014	0.690022184	0.866250338
PITHD1	-0.014	0.689965732	0.866222003
DEFB106B	-0.014	0.689937159	0.866208965
CIRBP-AS1	-0.014	0.689909792	0.866197439
EEF1A1P29	-0.014	0.689839452	0.86617097
RNU6-550P	-0.014	0.689819478	0.86617097
OR4A4P	-0.014	0.689741519	0.866100328
AC012451	-0.014	0.689679713	0.866045552
NPM1P43	-0.014	0.689669803	0.866045552
KB-1247B1	-0.014	0.689635569	0.866045552
KRTAP10-4	-0.014	0.689631014	0.866045552
RP11-85G21	-0.014	0.689530475	0.866035674
RP3-439F8	-0.014	0.689501761	0.866034878
LINC01566	-0.014	0.689457247	0.866017362

SEMA4C	-0.014	0.689414453	0.865986448
HEATR4	-0.014	0.689186746	0.865814596
RN7SL457P	-0.014	0.689099247	0.86579047
CDK7	-0.014	0.689029862	0.865731685
POU5F2	-0.014	0.689017334	0.865731685
SYT6	-0.014	0.688998876	0.865731685
RNU5E-8P	-0.014	0.688858543	0.865630613
IGBP1P5	-0.014	0.688821118	0.865606422
LCE1D	-0.014	0.688803878	0.865606422
SLC7A5	-0.014	0.688702863	0.865505181
CNPY1	-0.014	0.68864382	0.865505181
RP4-594A5	-0.014	0.688438636	0.865331264
TEKT5	-0.014	0.688404958	0.86531177
RP11-131M11	-0.014	0.688375117	0.865297096
RPS26P52	-0.014	0.688318499	0.865271601
RPL5P3	-0.014	0.688196696	0.865210838
RNU7-23P	-0.014	0.688159608	0.865208882
MIR1287	-0.014	0.688092606	0.865147481
PP7080	-0.014	0.688084819	0.865147481
AC125421	-0.014	0.688072308	0.865147481
AC078899	-0.014	0.688058868	0.865147481
RNU6-380P	-0.014	0.687993567	0.865147481
RP11-243A14	-0.014	0.687957166	0.865137062
ARMCX4	-0.014	0.687949335	0.865137062
HNRNPMP2	-0.014	0.687875547	0.865080106
WDR7-OT1	-0.014	0.687861932	0.865080106
RP11-359B20	-0.014	0.687808195	0.865063313
RGS14	-0.014	0.687773664	0.865043344
AL355512	-0.014	0.687727212	0.865007763
RN7SL307P	-0.014	0.687529826	0.864850854
RP11-460I13	-0.014	0.687374986	0.864701755
TMED7-TICAM2	-0.014	0.687374143	0.864701755
IPO4	-0.014	0.687301404	0.864700549
FTO	-0.014	0.687276488	0.864700549
AC004460	-0.014	0.687234207	0.864700549
TCEB1P29	-0.014	0.687217971	0.864700549
RNF113A	-0.014	0.687150709	0.864648
CTB-95D12	-0.014	0.687139856	0.864648
AK7	-0.014	0.68710952	0.864641859
BMF	-0.014	0.687022527	0.864629832
GOLGA6B	-0.014	0.68690033	0.864629832
RP11-166P13	-0.014	0.68689797	0.864629832
RNU2-23P	-0.014	0.686889397	0.864629832
ASH1L-AS1	-0.014	0.686839311	0.864629832
GPR19	-0.014	0.686833686	0.864629832
AL844165	-0.014	0.686818818	0.864629832
FAM231D	-0.014	0.686744759	0.864616898
SEC14L5	-0.014	0.686662715	0.864559312
FGFR2	-0.014	0.686658523	0.864559312
RP11-192N10	-0.014	0.686635129	0.864559312
ATP6V0E1P1	-0.014	0.686552464	0.864530518
KL	-0.014	0.68652435	0.864522229

SOCS5P5	-0.014	0.686515559	0.864522229
FAM154B	-0.014	0.686453653	0.864478916
RP11-408P14	-0.014	0.686444815	0.864478916
PBX2P1	-0.014	0.686356791	0.864478916
HIP1R	-0.014	0.686314273	0.864473445
RAB28P1	-0.014	0.686204576	0.864416667
RP11-226F19	-0.014	0.686196315	0.864416667
CTA-397C4	-0.014	0.686133228	0.864395377
RN7SKP74	-0.014	0.686132065	0.864395377
NLRP13	-0.014	0.686107916	0.864395377
HNRNPCP3	-0.014	0.686059821	0.864394358
RNU6-1183P	-0.014	0.685998443	0.864362757
RP11-639F1	-0.014	0.685955587	0.864331625
USP9YP32	-0.014	0.685945398	0.864331625
RAB41	-0.014	0.685932742	0.864331625
COTL1P1	-0.014	0.68590117	0.864331625
RPL7P24	-0.014	0.685877121	0.864331625
AP000887	-0.014	0.685872212	0.864331625
MIR4455	-0.014	0.685689124	0.864224513
CYP46A4P	-0.014	0.685414299	0.863992452
MAPKAPK5P1	-0.014	0.685206673	0.863822182
CTD-2555I5	-0.014	0.685156596	0.863804277
RP11-136H19	-0.014	0.68513512	0.86380057
RP11-648L3	-0.014	0.685066389	0.863746352
RP11-566H8	-0.014	0.684932913	0.86368249
KCTD19	-0.014	0.684916924	0.86368249
AC144652	-0.014	0.684876116	0.863679794
PCDHAC2	-0.014	0.684801492	0.863608555
ZNF487	-0.014	0.684686294	0.863534311
RP11-359E3	-0.014	0.684559489	0.863461711
OLA1P2	-0.014	0.684556416	0.863461711
RP5-1027G4	-0.014	0.684386184	0.863313402
CCDC25	-0.014	0.684381335	0.863313402
MIR1283-2	-0.014	0.684368485	0.863313402
SYT9	-0.014	0.684362408	0.863313402
AKR1B1P7	-0.014	0.684337437	0.863313402
LINC00467	-0.014	0.684329979	0.863313402
KB-1000E4	-0.014	0.684319267	0.863313402
PI4KB	-0.014	0.684315134	0.863313402
MST1R	-0.014	0.68421684	0.863313402
PGBD4P3	-0.014	0.684205345	0.863313402
RNU6-430P	-0.014	0.684155972	0.863313402
SLC20A1	-0.014	0.684154656	0.863313402
RP13-488H8	-0.014	0.684082446	0.863313402
RP11-686N4	-0.014	0.683938671	0.863232538
ZNF341	-0.014	0.683921186	0.863232538
PLK1	-0.014	0.683901531	0.863232538
AC092117	-0.014	0.683777433	0.86316276
RNU6-61P	-0.014	0.683765036	0.86316276
AC139453	-0.014	0.683727404	0.863145361
RP11-36N20	-0.014	0.683676151	0.863111088
RNU6-167P	-0.014	0.683623216	0.863111088

AC079466	-0.014	0.683612406	0.863111088
TMEM185B	-0.014	0.683545772	0.86309522
C11orf16	-0.014	0.683526259	0.86309522
FAM66D	-0.014	0.683473158	0.863053162
ATP5J2-PTCD1	-0.014	0.683299922	0.862880164
ELFN2	-0.014	0.68327307	0.862869134
SNORD116-7	-0.014	0.683157442	0.862816063
RNU6-541P	-0.014	0.683133156	0.862816063
RP11-305P22	-0.014	0.683130589	0.862816063
AP000688	-0.014	0.683127902	0.862816063
SPINK9	-0.014	0.683104981	0.862816063
DESI1	-0.014	0.683031309	0.862816063
RPS20P10	-0.014	0.683002578	0.862816063
BNIP3P11	-0.014	0.682923062	0.862796107
RNA5SP421	-0.014	0.682897226	0.862796107
RP11-524K14	-0.014	0.682872278	0.862796107
HGSNAT	-0.014	0.682854108	0.862796107
CEP70	-0.014	0.68280961	0.862787151
AC010141	-0.014	0.682773505	0.862775089
SLC7A9	-0.014	0.682683289	0.86274198
HN1L	-0.014	0.682682937	0.86274198
GYG1P3	-0.014	0.682673511	0.86274198
KRTCAP3	-0.014	0.682652472	0.86274198
MIR1269B	-0.014	0.682572437	0.862693454
OR51F5P	-0.014	0.682565138	0.862693454
RP11-545J16	-0.014	0.682476427	0.862640786
C5orf34	-0.014	0.682433625	0.862609578
PET100	-0.014	0.682316597	0.86253783
RN7SKP158	-0.014	0.682268352	0.862515125
RP11-261N11	-0.014	0.682241404	0.862515125
FAM124A	-0.014	0.68216351	0.862497054
AC063944	-0.014	0.681842935	0.862251963
AP000265	-0.014	0.681742986	0.862148461
RP11-557J10	-0.014	0.681696772	0.862112909
SLC35F2	-0.014	0.681616993	0.862057797
PLEKHA5	-0.014	0.681539276	0.862041767
RP11-548H18	-0.014	0.681498032	0.862021798
AC017079	-0.014	0.681497311	0.862021798
RPS19P3	-0.014	0.681421704	0.861992017
U91328	-0.014	0.681343396	0.861992017
ATP6V1B2	-0.014	0.681333382	0.861992017
RNA5SP226	-0.014	0.68120797	0.861929598
RNU6-971P	-0.014	0.68113697	0.861885557
RP11-730N24	-0.014	0.681093618	0.861853598
RP11-338I24	-0.014	0.681064257	0.861853598
EEF1B2P2	-0.014	0.681061951	0.861853598
FER1L6-AS1	-0.014	0.681049434	0.861853598
ABCD4	-0.014	0.681035399	0.861853598
CADM2-AS1	-0.014	0.680996279	0.861853598
ZNF69	-0.014	0.680957307	0.861853598
RP11-534L20	-0.014	0.68081875	0.861751365
MIR3151	-0.014	0.680764215	0.86171151

RP11-11K13	-0.014	0.680738718	0.861702138
OFD1P2Y	-0.014	0.680685483	0.861689339
RNU6-214P	-0.014	0.680631509	0.861658034
VN1R48P	-0.014	0.680476882	0.86148518
H3F3AP6	-0.014	0.680472405	0.86148518
SYMPK	-0.014	0.680468407	0.86148518
YARS2	-0.014	0.680429974	0.86148518
RPS12P31	-0.014	0.680371548	0.86148518
MIR378D1	-0.014	0.680312731	0.861437653
PPIAL4D	-0.014	0.680312485	0.861437653
SNORA58	-0.014	0.680293137	0.861437653
AC016732	-0.014	0.680073223	0.861248867
RN7SKP121	-0.014	0.680043673	0.861234345
CTD-2315A10	-0.014	0.680035604	0.861234345
TINAG	-0.014	0.680017279	0.861234345
UBTFL9	-0.014	0.679943301	0.86119883
FZD6	-0.014	0.67992681	0.86119883
DST	-0.014	0.679664277	0.860959905
RP11-375H17	-0.014	0.679607912	0.860947827
DNAJB8-AS1	-0.014	0.679523039	0.860897009
OR10J3	-0.014	0.679500925	0.860897009
EFHD2	-0.014	0.679498613	0.860897009
VPS35	-0.014	0.67948861	0.860897009
TXNP4	-0.014	0.679472759	0.860897009
TMEM8B	-0.014	0.679434592	0.860897009
MIR378E	-0.014	0.679423618	0.860897009
RN7SL806P	-0.014	0.679253202	0.860874219
AC009052	-0.014	0.679239332	0.860874219
NRAP	-0.014	0.67922717	0.860874219
KCNQ5-AS1	-0.014	0.679220093	0.860874219
OR7E130P	-0.014	0.679200303	0.860874219
AC008937	-0.014	0.679173089	0.860874219
RP11-97E7	-0.014	0.679155186	0.860874219
PPM1H	-0.014	0.679144928	0.860874219
PAQR4	-0.014	0.679132926	0.860874219
TMEM168	-0.014	0.679130526	0.860874219
RP11-688D15	-0.014	0.679094419	0.860874219
AC243980	-0.014	0.679053511	0.860874219
RP11-379B18	-0.014	0.679022915	0.860874219
ART3	-0.014	0.678950921	0.860874219
HMGN1P9	-0.014	0.678662535	0.860779829
HNRNPLP1	-0.014	0.678638052	0.860779829
GAPDHP46	-0.014	0.678579128	0.86072995
RPL15P2	-0.014	0.678551997	0.86072675
CCNJP1	-0.014	0.678451406	0.860682553
OR2T29	-0.014	0.678448998	0.860682553
MIR520B	-0.014	0.678412261	0.860682553
IAH1	-0.014	0.678391781	0.860682553
CA1	-0.014	0.678256487	0.860549888
KRTAP27-1	-0.014	0.678064931	0.860421455
RP1-197J16	-0.014	0.678014382	0.860380234
ACTRT2	-0.014	0.678009304	0.860380234

GS1-204I12	-0.014	0.677970276	0.860380234
CRIM1	-0.014	0.677897191	0.860380234
CTC-339D2	-0.014	0.677797655	0.860288586
RRM2P2	-0.014	0.677754402	0.860256613
AC020922	-0.014	0.677719782	0.860235596
AL139295	-0.014	0.677682085	0.860235596
PMM2P1	-0.014	0.677676449	0.860235596
AC002389	-0.014	0.677637175	0.860235596
NDFIP1	-0.014	0.677620969	0.860235596
IQGAP2	-0.014	0.677529837	0.860177894
AP000255	-0.014	0.677508409	0.860173619
RN7SL849P	-0.014	0.677497316	0.860173619
RP11-698F20	-0.014	0.677495355	0.860173619
RNU6-522P	-0.014	0.677476827	0.860173619
RP11-781M16	-0.014	0.677393296	0.860147571
RP11-759F5	-0.014	0.677253162	0.860032965
RAC1P1	-0.014	0.677154854	0.859934241
RP11-354G16	-0.014	0.677124209	0.859934241
NHSL2	-0.014	0.677119999	0.859934241
RNU6-443P	-0.014	0.676978868	0.859890989
SNORD58	-0.014	0.676961832	0.859890989
B9D1	-0.014	0.6768441	0.859765668
AC104809	-0.014	0.676711363	0.859642913
WDR92	-0.014	0.676681896	0.859628408
RP11-149F8	-0.014	0.67656959	0.859554521
ATP5LP5	-0.014	0.676456007	0.85949812
CACNG1	-0.014	0.676450694	0.85949812
RNU2-39P	-0.014	0.676449285	0.85949812
FAM231A	-0.014	0.676378088	0.859494642
RNU7-175P	-0.014	0.676346198	0.859477051
RNU6ATAC8P	-0.014	0.676283468	0.859443064
RP11-638L3	-0.014	0.676210176	0.859385137
ABHD17B	-0.014	0.67619138	0.859385137
TMEM119	-0.014	0.676093133	0.859338893
RP11-708J19	-0.014	0.676058788	0.859318172
RP11-126F18	-0.014	0.676035395	0.85931137
RP11-989F5	-0.014	0.676034286	0.85931137
KLF2P3	-0.014	0.67594726	0.859245207
RP11-332O19	-0.014	0.675929134	0.859245098
RP11-521A24	-0.014	0.675876105	0.859200621
OR7E96P	-0.014	0.675809902	0.859162326
AC017028	-0.014	0.675781136	0.859148688
ZNF670	-0.014	0.675625939	0.858974309
PCDHGB3	-0.014	0.675563231	0.858917512
LINC00280	-0.014	0.675481289	0.858859185
RP11-375B1	-0.014	0.675400844	0.858848614
RPL5P33	-0.014	0.67517205	0.8586551
RNU6-1195P	-0.014	0.675084891	0.858607315
CYP19A1	-0.014	0.675072356	0.858607315
BCRP8	-0.014	0.675054606	0.858607315
RP11-134L4	-0.014	0.67473928	0.858396979
TMEM64	-0.014	0.674703896	0.858374891

AP001627	-0.014	0.67460479	0.85829466
AC006328	-0.014	0.674585357	0.858292864
PRUNE2	-0.014	0.674475256	0.858198633
TEX13B	-0.014	0.674377246	0.858119776
DSTNP5	-0.014	0.674362763	0.858119776
RP11-589C21	-0.014	0.674133676	0.858004919
AL138721	-0.014	0.674042351	0.857945776
RN7SKP99	-0.014	0.67394465	0.857844344
ZNF138	-0.014	0.673937494	0.857844344
RP11-77B22	-0.014	0.673935526	0.857844344
SNHG8	-0.014	0.67386171	0.857844344
RNA5SP480	-0.014	0.673669333	0.857654352
AL133247	-0.014	0.67360765	0.857598747
MIR6790	-0.014	0.673580607	0.857598747
RP11-275A14	-0.014	0.673469645	0.857537664
RNU6-408P	-0.014	0.673323352	0.857443083
RPS14P7	-0.014	0.673319002	0.857443083
HIST2H3PS2	-0.014	0.673316925	0.857443083
RP11-473A10	-0.014	0.673268353	0.857441823
RP11-291L22	-0.014	0.67324519	0.857435251
RNU1-77P	-0.014	0.673244517	0.857435251
AC083855	-0.014	0.673127796	0.857331592
HMGN2P39	-0.014	0.673070566	0.857327481
RN7SL36P	-0.014	0.672993321	0.857252017
RP11-81H3	-0.014	0.672926285	0.857212479
AP004242	-0.014	0.672898344	0.857212479
RP11-508O18	-0.014	0.672890587	0.857212479
AC005235	-0.014	0.672808584	0.857180285
TXNP1	-0.014	0.672794573	0.857180285
LINC01074	-0.014	0.672752807	0.857180285
CHRNB4	-0.014	0.672607575	0.857127441
AC114973	-0.014	0.672546715	0.857072818
GLRXP	-0.014	0.672443398	0.857001959
CDX4	-0.014	0.672310539	0.856886481
MIR576	-0.014	0.67230336	0.856886481
EIF4EBP2P3	-0.014	0.672273199	0.856886481
RNU6-1073P	-0.014	0.672264067	0.856886481
RNU6-70P	-0.014	0.672231476	0.856886481
CTD-2270F17	-0.014	0.67211444	0.856819979
RP11-400F19	-0.014	0.672072175	0.856796779
ZFAND2A	-0.014	0.672066995	0.856796779
IGSF1	-0.014	0.672059417	0.856796779
OPN5	-0.014	0.672032596	0.856796779
RP11-264F23	-0.014	0.671924809	0.856761696
RP4-535B20	-0.014	0.671796142	0.856713756
RP11-410L14	-0.014	0.67178293	0.856713756
RP6-99M1	-0.014	0.6717753	0.856713756
RP4-737E23	-0.014	0.671728273	0.856713756
L3HYPDH	-0.014	0.671672185	0.856668929
RP4-585I14	-0.014	0.671550679	0.856605715
RPL17P37	-0.014	0.671484585	0.856544348
RP11-473P24	-0.014	0.671470623	0.856544348

C17orf62	-0.014	0.671465055	0.856544348
AC073065	-0.014	0.671408434	0.856544348
AP000322	-0.014	0.671370284	0.85653619
CCDC11P1	-0.014	0.671364055	0.85653619
HMOX1	-0.014	0.671280253	0.856490157
OR10J8P	-0.014	0.671253568	0.856490157
CTD-2010I16	-0.014	0.67124441	0.856490157
RP11-554D14	-0.014	0.671155083	0.856422225
VN2R9P	-0.014	0.671075067	0.85636601
SEPT7P9	-0.014	0.671014715	0.856334883
LINC01431	-0.014	0.670965656	0.856318166
CHP1	-0.014	0.670794842	0.856161473
RP5-912I13	-0.014	0.670756557	0.856154811
RNU6-493P	-0.014	0.67072131	0.856143971
LINC01534	-0.014	0.670696503	0.856143971
RP11-1281K21	-0.014	0.670693986	0.856143971
BPIFA4P	-0.014	0.670674226	0.856143971
CFC1B	-0.014	0.670665646	0.856143971
RP5-874C20	-0.014	0.670644363	0.856143971
MIR4664	-0.014	0.670640357	0.856143971
HIPK1-AS1	-0.014	0.670564602	0.856127501
MIR5702	-0.014	0.670424318	0.855988989
HSP90AB6P	-0.015	0.670340673	0.855956333
LINC01474	-0.015	0.670339112	0.855956333
AC112249	-0.015	0.670232412	0.855890339
SEC31A	-0.015	0.670194143	0.855883961
CLC	-0.015	0.670129755	0.855847631
ZNF727	-0.015	0.670038012	0.855753409
RP11-396J6	-0.015	0.669984461	0.855707962
MTND5P31	-0.015	0.669816186	0.855561871
LILRA5	-0.015	0.669713815	0.855454054
RP3-465N24	-0.015	0.669661093	0.855409652
XPNPEP2	-0.015	0.669650511	0.855409652
USP9YP20	-0.015	0.669634094	0.855409652
NNAT	-0.015	0.669619912	0.855409652
RP11-503G7	-0.015	0.669561527	0.855409652
LYN	-0.015	0.669468728	0.855372905
TAS2R9	-0.015	0.669404318	0.855356946
RN7SKP128	-0.015	0.669378613	0.855347049
AL138963	-0.015	0.669359827	0.855345992
BPNT1	-0.015	0.669280561	0.855267649
NR2F2-AS1	-0.015	0.669213334	0.855208754
SYCP2L	-0.015	0.669169165	0.855208754
MT-CYB	-0.015	0.669127018	0.855208754
DSC1	-0.015	0.668899114	0.855055508
RPL21P23	-0.015	0.668875211	0.855047902
FGF21	-0.015	0.668836346	0.855021168
CKS2	-0.015	0.668830315	0.855021168
RP4-616B8	-0.015	0.66882403	0.855021168
MTNR1A	-0.015	0.66882209	0.855021168
SLC38A2	-0.015	0.668804466	0.855021168
RPL8P2	-0.015	0.668769603	0.855021168

AC087793	-0.015	0.668735927	0.855021168
EZH2P1	-0.015	0.668711425	0.855021168
AL121970	-0.015	0.668682575	0.855021168
OPALIN	-0.015	0.668622883	0.855021168
OR5BT1P	-0.015	0.668617182	0.855021168
RNA5SP477	-0.015	0.668605096	0.855021168
RN7SL601P	-0.015	0.66859196	0.855021168
AL023876	-0.015	0.668525168	0.855021168
RP11-163F15	-0.015	0.668520822	0.855021168
SOX3	-0.015	0.668493914	0.855021168
KRTAP9-3	-0.015	0.668440347	0.855021168
BCCIP	-0.015	0.668430286	0.855021168
EIF3EP1	-0.015	0.66842949	0.855021168
AK4P5	-0.015	0.668382768	0.855021168
PDCL3P3	-0.015	0.668322047	0.855021168
LINC01222	-0.015	0.668267523	0.855005435
RNU6-1060P	-0.015	0.668245277	0.854999941
AL118509	-0.015	0.668214778	0.854999941
GS1-309P15	-0.015	0.668156176	0.854954843
RPL21P54	-0.015	0.668102829	0.854909549
HMGB1P30	-0.015	0.668097886	0.854909549
CYCSP8	-0.015	0.668032765	0.854865831
TSPAN1	-0.015	0.667890434	0.854747377
OR10D5P	-0.015	0.667849372	0.854723009
AC013406	-0.015	0.667795019	0.854676413
GUCY2GP	-0.015	0.667708687	0.854611852
RNU6-258P	-0.015	0.667700498	0.854611852
AKT2	-0.015	0.667632892	0.854560772
PDE5A	-0.015	0.667580038	0.85453905
GPR137C	-0.015	0.667543477	0.854515218
ENO1P2	-0.015	0.667534208	0.854515218
ZFYVE9P2	-0.015	0.667528732	0.854515218
RNU6-757P	-0.015	0.667435086	0.854469662
NDOR1	-0.015	0.667114351	0.854195483
OR10C1	-0.015	0.66709952	0.854195483
OSR2	-0.015	0.667057142	0.854168159
RP11-445N20	-0.015	0.667007464	0.85412751
WARS2-IT1	-0.015	0.666988803	0.854126579
TUBB8P8	-0.015	0.666921995	0.854109921
RNF32	-0.015	0.666808863	0.854010963
RP11-401I19	-0.015	0.666628568	0.853871899
AP000457	-0.015	0.666529036	0.853790334
AC006460	-0.015	0.666294793	0.85360507
RP11-425D17	-0.015	0.66626235	0.853586468
MIR3125	-0.015	0.666204305	0.853566126
RNU2-70P	-0.015	0.666193804	0.853566126
HNRNPCL3	-0.015	0.66615487	0.853566126
SNORD115-25	-0.015	0.666068545	0.853521849
RP4-564F22	-0.015	0.666052529	0.853521849
PCDH9-AS3	-0.015	0.666031497	0.853521849
FAAHP1	-0.015	0.666025716	0.853521849
RN7SL767P	-0.015	0.66591397	0.853438584

RNU6-821P	-0.015	0.665893537	0.853435362
MKNK2	-0.015	0.665761327	0.853311842
AC010525	-0.015	0.665671039	0.853265007
RP11-430C1	-0.015	0.665589734	0.853206714
TGIF1P1	-0.015	0.665581746	0.853206714
BTC	-0.015	0.665571586	0.853206714
NPM1P17	-0.015	0.665507952	0.853170768
KRTAP10-11	-0.015	0.665488998	0.853169435
RP5-832C2	-0.015	0.66546045	0.853155801
RP11-666F17	-0.015	0.665398976	0.853152214
AC005599	-0.015	0.665390331	0.853152214
STAR	-0.015	0.665315716	0.853152214
OR10AC1	-0.015	0.665313613	0.853152214
RP11-43A1	-0.015	0.665285328	0.853152214
CTA-212D2	-0.015	0.66524522	0.853152214
ARHGAP12	-0.015	0.665004473	0.852961544
MRPS36P1	-0.015	0.664983005	0.852956979
NTRK3	-0.015	0.664844573	0.852825352
RP11-103C16	-0.015	0.664762754	0.852789304
RP11-563J2	-0.015	0.664750345	0.852789304
KRCC1	-0.015	0.664664059	0.85275457
RP11-6N13	-0.015	0.664550488	0.852677769
RP11-252P19	-0.015	0.664514645	0.85265475
ZNF460	-0.015	0.664504192	0.85265475
RP11-867G2	-0.015	0.664499859	0.85265475
RNU7-99P	-0.015	0.664438817	0.852626365
RPL6P30	-0.015	0.664388177	0.852584354
RP11-324I22	-0.015	0.664156344	0.852378717
RP11-486B10	-0.015	0.664136515	0.852378717
MFSD5	-0.015	0.664019129	0.852303135
MTND5P6	-0.015	0.66400637	0.852303135
AC090958	-0.015	0.663966508	0.852303135
DNAJC19P4	-0.015	0.663874279	0.852246369
C6orf222	-0.015	0.663848001	0.852246369
MIR591	-0.015	0.663833095	0.852246369
MIR3118-4	-0.015	0.66380682	0.852246369
LINC01579	-0.015	0.663771436	0.852246369
GALR2	-0.015	0.663754069	0.852246369
HGS	-0.015	0.663737198	0.852246369
LHFPL1	-0.015	0.663734849	0.852246369
RP11-92F20	-0.015	0.663720762	0.852246369
LETM1P2	-0.015	0.66370276	0.852246369
BMS1P15	-0.015	0.66363253	0.852211672
RP11-282M24	-0.015	0.66359961	0.852192375
BRDTP1	-0.015	0.663580319	0.85219058
LINC00453	-0.015	0.663562918	0.85219058
ALOX12B	-0.015	0.663558258	0.85219058
TCEAL3-AS1	-0.015	0.66352737	0.85219058
AF224669	-0.015	0.663477976	0.85219058
OR5M2P	-0.015	0.663439913	0.852171114
MIR222	-0.015	0.663439648	0.852171114
AF003626	-0.015	0.663261842	0.851988342

RAD51AP1P1	-0.015	0.663148038	0.851865131
MIR4488	-0.015	0.663099265	0.851825454
OR1AA1P	-0.015	0.663083218	0.851825454
OR10AF1P	-0.015	0.663019323	0.851768708
AC005606	-0.015	0.662995006	0.851768708
MIR5006	-0.015	0.66297081	0.851768708
AL050327	-0.015	0.662821993	0.851712084
THRB	-0.015	0.662816795	0.851712084
MNT	-0.015	0.662800152	0.851712084
CTD-2342N23	-0.015	0.66277855	0.851712084
RP5-879K22	-0.015	0.662754257	0.851712084
RAC1P7	-0.015	0.662722601	0.851712084
C5orf15	-0.015	0.662699898	0.851712084
RNU6-79P	-0.015	0.662625274	0.851712084
RP11-15G8	-0.015	0.662597094	0.851712084
RNU6-482P	-0.015	0.662593433	0.851712084
AL359546	-0.015	0.662567065	0.851712084
RP11-1E4	-0.015	0.662445235	0.851712084
CCT8L2	-0.015	0.662428764	0.851712084
AC010146	-0.015	0.662428052	0.851712084
RP11-147K6	-0.015	0.662345015	0.851683523
CDK9	-0.015	0.66232826	0.851683523
ZNF189	-0.015	0.662304491	0.851677403
SMIM17	-0.015	0.662158473	0.851604601
RP11-424M22	-0.015	0.662080315	0.851527076
RP11-810O3	-0.015	0.662037547	0.851495064
DEFB103B	-0.015	0.662019733	0.851495064
GNPAT	-0.015	0.662018097	0.851495064
RP11-115D19	-0.015	0.661991438	0.851495064
AC093382	-0.015	0.661976285	0.851495064
RP1-197B17	-0.015	0.661968983	0.851495064
GSDMA	-0.015	0.661926125	0.851495064
RP1-60N8	-0.015	0.66191133	0.851495064
RNU6-1170P	-0.015	0.66188816	0.851495064
RIIAD1	-0.015	0.66182808	0.851495064
RDH12	-0.015	0.6618038	0.851493356
MYL5	-0.015	0.661796305	0.851493356
RP11-796E10	-0.015	0.661647202	0.851360872
POU1F1	-0.015	0.661623479	0.851353347
AC008072	-0.015	0.661602162	0.851353347
RN7SL814P	-0.015	0.661579083	0.851353347
HMGB1P39	-0.015	0.661571929	0.851353347
RP11-651P23	-0.015	0.661528609	0.851346278
TSSK6	-0.015	0.66149812	0.851330044
OR3B1P	-0.015	0.66132345	0.851266269
OR2T35	-0.015	0.661181292	0.851152293
SCARNA24	-0.015	0.661034636	0.85102486
ADD2	-0.015	0.661010764	0.85102486
PDCD11	-0.015	0.660866287	0.850930781
AC068282	-0.015	0.660838581	0.850918111
RP5-837J1	-0.015	0.660728524	0.850918111
RNU6-269P	-0.015	0.660725896	0.850918111

RPL9P3	-0.015	0.660689819	0.850918111
AC083810	-0.015	0.660652455	0.850908498
SNORA64	-0.015	0.660626444	0.850898009
FOXD3-AS1	-0.015	0.660569805	0.850871078
RP11-473M20	-0.015	0.660531466	0.850861097
PRR25	-0.015	0.660482921	0.850851209
RAB5CP2	-0.015	0.660469502	0.850851209
FBXL3	-0.015	0.660387427	0.850820245
RNA5SP342	-0.015	0.660369759	0.850820245
FNDC3CP	-0.015	0.660282783	0.850777483
AC104653	-0.015	0.660244987	0.850757295
AC108667	-0.015	0.660219711	0.850757295
VN1R65P	-0.015	0.660210349	0.850757295
AC007040	-0.015	0.660116898	0.850701829
CTD-2037L6	-0.015	0.660052189	0.850664473
CRYGFP	-0.015	0.660026962	0.850662665
ZNF446	-0.015	0.659961319	0.85063784
AP002364	-0.015	0.659948691	0.85063784
RABEPK	-0.015	0.659937188	0.85063784
FKBP4P1	-0.015	0.659899617	0.850628967
RNU12	-0.015	0.659856892	0.850596915
RP11-77H9	-0.015	0.659737429	0.850488957
RP11-332G1	-0.015	0.659732779	0.850488957
DISC1-IT1	-0.015	0.659675458	0.850478128
CEACAM8	-0.015	0.659635476	0.850449604
MGC16275	-0.015	0.659618361	0.850449604
RP11-40H20	-0.015	0.65960589	0.850449604
RP11-7I15	-0.015	0.65942043	0.850264419
RP11-521F1	-0.015	0.659324537	0.850209826
PATE2	-0.015	0.659288762	0.850186712
RP11-54G14	-0.015	0.659277945	0.850186712
CTD-2049O17	-0.015	0.659227316	0.850176534
MIR3680-1	-0.015	0.659178909	0.850137125
HTR5BP	-0.015	0.659129307	0.850119195
AL162381	-0.015	0.659103796	0.850109312
RP11-115L11	-0.015	0.659076371	0.850096961
RP11-44I10	-0.015	0.659055103	0.850092552
AC133965	-0.015	0.659041439	0.850092552
MIR4440	-0.015	0.659037677	0.850092552
RIMS4	-0.015	0.65898437	0.850092552
PDIA3	-0.015	0.658882496	0.850077113
RP11-90P13	-0.015	0.658862992	0.850074978
RP11-14I17	-0.015	0.658743582	0.849943939
COASY	-0.015	0.65871093	0.849924834
RP11-424D14	-0.015	0.658687199	0.849919855
MIR6876	-0.015	0.658512162	0.849743948
PABPC1P12	-0.015	0.658492868	0.849743948
VN1R46P	-0.015	0.658465091	0.849743948
PARS2	-0.015	0.658395122	0.849743948
RP1-31B8	-0.015	0.658390003	0.849743948
TEX11	-0.015	0.658350542	0.849743948
RP13-577H12	-0.015	0.65821998	0.849684352

MIR4530	-0.015	0.658151555	0.849663453
TBC1D21	-0.015	0.658149213	0.849663453
RP5-916O11	-0.015	0.658148409	0.849663453
CASC1	-0.015	0.658131139	0.849663453
RNASEH1P2	-0.015	0.658115867	0.849663453
FAM66B	-0.015	0.658081479	0.849663453
SF3A3P2	-0.015	0.657997166	0.849625349
RP11-710M11	-0.015	0.657903145	0.849573047
RP11-5O17	-0.015	0.657900781	0.849573047
RP11-397E7	-0.015	0.657881817	0.849573047
AP003558	-0.015	0.657854316	0.849573047
FUT8	-0.015	0.657840728	0.849573047
RPL13AP5	-0.015	0.657840333	0.849573047
INO80C	-0.015	0.657700874	0.849573047
LINC01210	-0.015	0.657691463	0.849573047
DUTP5	-0.015	0.657453781	0.849407314
ATP5A1P6	-0.015	0.65745015	0.849407314
HMGB1P48	-0.015	0.657446299	0.849407314
ZDHHC18	-0.015	0.657433054	0.849407314
RP13-329D4	-0.015	0.657413354	0.849407314
CST13P	-0.015	0.657384599	0.849407314
RSRC1	-0.015	0.657344975	0.849407314
AC020626	-0.015	0.657299781	0.849392681
CTD-2125J1	-0.015	0.657238002	0.849358939
GAS2L1P1	-0.015	0.657205576	0.849351297
HNRNPA1P7	-0.015	0.657140834	0.84932555
RP11-1148L6	-0.015	0.65706655	0.849272683
RP5-1096J16	-0.015	0.657014278	0.849243311
LRP1B	-0.015	0.65694618	0.84918919
AC074051	-0.015	0.656907998	0.849162882
APITD1-CORT	-0.015	0.656815811	0.849133781
CTD-2036A18	-0.015	0.656812679	0.849133781
OR4R3P	-0.015	0.656745394	0.849100837
RP11-331K15	-0.015	0.656706403	0.849100837
SKIV2L	-0.015	0.65666578	0.849100837
GAPDHP54	-0.015	0.656639159	0.849100837
RNU1-20P	-0.015	0.656614967	0.849100837
TRMT10C	-0.015	0.656572216	0.849100837
KCNJ1	-0.015	0.656555418	0.849100837
CTD-3064H18	-0.015	0.65638774	0.849100837
RN7SL716P	-0.015	0.656354585	0.849100837
ANKRD20A19P	-0.015	0.656343605	0.849100837
MIR4765	-0.015	0.656322331	0.849100837
RP11-333B6	-0.015	0.656309938	0.849100837
OVGP1	-0.015	0.656286819	0.849100837
RP11-716H6	-0.015	0.656169231	0.849065531
PDDC1	-0.015	0.656105264	0.849023921
MIR6848	-0.015	0.656099016	0.849023921
RPL7AP30	-0.015	0.656095792	0.849023921
RP11-317F20	-0.015	0.656019345	0.84898194
RNU1-96P	-0.015	0.655928589	0.84898194
TTC3P1	-0.015	0.655924519	0.84898194

ZNF404	-0.015	0.655643969	0.848749839
CIRH1A	-0.015	0.655580054	0.848710214
RP11-466I1	-0.015	0.655540491	0.848708156
HAUS1P1	-0.015	0.655500366	0.848679279
OFD1P7Y	-0.015	0.655466025	0.848657887
GLUL	-0.015	0.655443156	0.848657887
RP11-102F4	-0.015	0.655385372	0.848622671
SLC16A1P1	-0.015	0.655337623	0.848583915
C15orf61	-0.015	0.655250256	0.848516924
GRAMD4P2	-0.015	0.655233031	0.848516924
RP11-478B9	-0.015	0.655159555	0.848445607
SCARNA13	-0.015	0.655009627	0.84829758
MAB21L3	-0.015	0.654984421	0.84829758
TULP3	-0.015	0.654913428	0.848265258
ELTD1	-0.015	0.654884244	0.848250528
AC097466	-0.015	0.654831825	0.848205699
AL138999	-0.015	0.654808273	0.84819826
RP11-1105G2	-0.015	0.654771854	0.84819826
RP11-334E6	-0.015	0.654699086	0.848157544
SMCO1	-0.015	0.654687658	0.848157544
CD302	-0.015	0.654676822	0.848157544
CTD-2647E9	-0.015	0.654614826	0.84815529
RNA5SP486	-0.015	0.654545522	0.84811164
LDLRAP1	-0.015	0.654387421	0.847952923
RP13-7D7	-0.015	0.654265694	0.847841324
DOCK9-AS2	-0.015	0.654258508	0.847841324
LGMNP1	-0.015	0.654241703	0.847841324
OR10R1P	-0.015	0.654163131	0.847841324
PGLYRP2	-0.015	0.654156476	0.847841324
EPHA8	-0.015	0.654056194	0.847840715
CLCP1	-0.015	0.654051194	0.847840715
AC073409	-0.015	0.654038189	0.847840715
RPS15P5	-0.015	0.653967073	0.847800364
KB-208E9	-0.015	0.653908382	0.847747353
SLC14A2-AS1	-0.015	0.653875643	0.847727985
MIR218-2	-0.015	0.653697007	0.847596421
DAAM1	-0.015	0.653683455	0.847596421
FAF1	-0.015	0.653627892	0.847568286
CALM1	-0.015	0.653577847	0.847534958
GGT8P	-0.015	0.65352346	0.847525169
NARF	-0.015	0.653397778	0.847431403
TRBV20OR9-2	-0.015	0.653383771	0.847431403
RP11-363J20	-0.015	0.653331187	0.847414266
EEF1GP3	-0.015	0.653329583	0.847414266
UTP14A	-0.015	0.653328041	0.847414266
RNU6-31P	-0.015	0.653227302	0.847414266
PSPH	-0.015	0.653201183	0.847414266
MIR30C2	-0.015	0.653034467	0.847237062
MT-TS1	-0.015	0.653016846	0.847237062
DDX39B-AS1	-0.015	0.652933365	0.847175127
RNU6-1120P	-0.015	0.652843725	0.847104974
KCTD4	-0.015	0.652842455	0.847104974

UBE2DNL	-0.015	0.65282808	0.847104974
CTD-2234B20	-0.015	0.652798892	0.847104974
FAM218BP	-0.015	0.652787262	0.847104974
RNU6-509P	-0.015	0.652747253	0.847095189
AC139103	-0.015	0.652621005	0.846995583
RP5-1120P11	-0.015	0.652585945	0.846995583
AC006014	-0.015	0.652509789	0.846966545
TRBV29OR9-2	-0.015	0.652454375	0.846922802
NOL10	-0.015	0.652450876	0.846922802
RAB14	-0.015	0.652379999	0.846918588
SYTL3	-0.015	0.652351649	0.846904869
RP11-82H13	-0.015	0.652282725	0.846838472
AC079467	-0.015	0.652254085	0.846824374
IGHD3OR15-3B	-0.015	0.652213251	0.846794441
AC007009	-0.015	0.652170336	0.846761807
RNU6-1150P	-0.015	0.65214775	0.846755566
CCL17	-0.015	0.652013331	0.846650278
GNG10	-0.015	0.651927642	0.846608256
NEFLP1	-0.015	0.651893408	0.846600968
MIR5591	-0.015	0.651881818	0.846600968
OR7E21P	-0.015	0.651865777	0.846600968
RP3-336K20__B	-0.015	0.651818207	0.846600968
RPS20P20	-0.015	0.651780041	0.846600968
RP11-566F5	-0.015	0.651677454	0.84658344
SIAH1P1	-0.015	0.651676096	0.84658344
BGLT3	-0.015	0.651638196	0.846578624
XXyac-YR21CG7	-0.015	0.651637473	0.846578624
MIR4786	-0.015	0.651500648	0.846507992
RP11-370I10	-0.015	0.651492721	0.846507992
SSXP10	-0.015	0.651439793	0.846482498
RP11-309N24	-0.015	0.651310503	0.846397206
IGLVIV-53	-0.015	0.651254311	0.846356938
OR10G7	-0.015	0.651173115	0.846292427
PAFAH1B2	-0.015	0.65116416	0.846292427
ASGR2	-0.015	0.651149798	0.846292427
RPSAP1	-0.015	0.651076997	0.846265054
TRDD2	-0.015	0.650993675	0.846202941
COX4I1P1	-0.015	0.650852336	0.846088496
MIR761	-0.015	0.650710167	0.845972952
RP11-177J6	-0.015	0.650649273	0.845916876
AC012493	-0.015	0.650621963	0.845916876
PPP3R1	-0.015	0.650610274	0.845916876
PXT1	-0.015	0.650561497	0.845916876
CYP2F1	-0.015	0.65047985	0.845858238
MIR920	-0.015	0.650426626	0.845829459
SNRPFP3	-0.015	0.650389045	0.845824198
RP11-189E14	-0.015	0.650375112	0.845824198
AL118520	-0.015	0.650352659	0.845824198
LL0XNC01-16G2	-0.015	0.650339023	0.845824198
RNA5SP356	-0.015	0.650317877	0.845824198
KRT18P65	-0.015	0.650220082	0.845751361
MS4A6E	-0.015	0.650214087	0.845751361

TBCE	-0.015	0.650208356	0.845751361
MED28P7	-0.015	0.650158956	0.845741147
RP5-878I13	-0.015	0.650057758	0.8456557
LINC01075	-0.015	0.650040662	0.8456557
DHRS4L1	-0.015	0.650017258	0.84564921
RP11-731D1	-0.015	0.650011729	0.84564921
LINC01476	-0.015	0.649965742	0.845628387
OSBPL8	-0.015	0.649923299	0.845618262
HS3ST5	-0.015	0.649900294	0.845612538
TUBA3E	-0.015	0.649878805	0.845610837
MMP21	-0.015	0.649823278	0.845604734
AC007535	-0.015	0.649576165	0.845352461
UBE2Q2P9	-0.015	0.64930104	0.845033342
RP11-116P24	-0.015	0.649173725	0.844897989
RP11-457K10	-0.015	0.649165819	0.844897989
ASS1P10	-0.015	0.649165614	0.844897989
AC005625	-0.016	0.649050258	0.844806554
UBL4B	-0.016	0.649037937	0.844806554
TYMP	-0.016	0.649027178	0.844806554
AC078842	-0.016	0.648870481	0.844664891
AC026367	-0.016	0.648780834	0.84461745
VDAC1P10	-0.016	0.64877746	0.84461745
LINC00332	-0.016	0.648687581	0.844542218
TMEM212-AS1	-0.016	0.648588524	0.844467999
MZT1P1	-0.016	0.648571453	0.844467999
ZNF213-AS1	-0.016	0.648467359	0.844393999
SCGB1B2P	-0.016	0.648419047	0.844354175
EIF2B5-IT1	-0.016	0.648415641	0.844354175
UGT1A11P	-0.016	0.648378568	0.844354175
WDR82P1	-0.016	0.6483367	0.844354175
AC012368	-0.016	0.648294802	0.844354175
CTD-2275D24	-0.016	0.648288939	0.844354175
RP4-607I7	-0.016	0.648216043	0.844320675
RP11-390E23	-0.016	0.64821364	0.844320675
MTCYBP1	-0.016	0.648164149	0.844320675
OR7E108P	-0.016	0.648156685	0.844320675
YY2	-0.016	0.648122531	0.844320675
MIR7162	-0.016	0.648119379	0.844320675
ASCL2	-0.016	0.648081691	0.844320675
RP11-886P16	-0.016	0.648068701	0.844320675
MT1F	-0.016	0.647945041	0.844221657
ARL2BPP3	-0.016	0.647847477	0.844163821
CAMSAP2	-0.016	0.647730273	0.844080379
AL137855	-0.016	0.647705983	0.844071821
PBX3	-0.016	0.647663924	0.844055757
RNU6-40P	-0.016	0.647614873	0.844022372
RP11-16D22	-0.016	0.647605071	0.844022372
AL390776	-0.016	0.647560066	0.844020232
AC002064	-0.016	0.647499011	0.843986847
RNU2-45P	-0.016	0.647449613	0.843945556
ABLIM3	-0.016	0.647446447	0.843945556
MEIOB	-0.016	0.647416797	0.843945556

RP11-31F19	-0.016	0.64736891	0.843945556
RP11-375N15	-0.016	0.647335241	0.843945556
RP11-781A6	-0.016	0.647319178	0.843945556
AC002519	-0.016	0.647312288	0.843945556
FAM135A	-0.016	0.64727806	0.843945556
AC016597	-0.016	0.647226856	0.843945556
EEF1DP7	-0.016	0.647126372	0.843847545
TRIM64EP	-0.016	0.647092238	0.843826137
HNRNPA1P58	-0.016	0.647076465	0.843826137
NFE2L2	-0.016	0.647059851	0.843826137
NFE2L3P1	-0.016	0.647056084	0.843826137
RP11-1H8	-0.016	0.647052971	0.843826137
RNU2-15P	-0.016	0.646967989	0.843802733
RP3-329E20	-0.016	0.646907439	0.843746867
RP11-143H17	-0.016	0.646788073	0.843737294
ALDH4A1	-0.016	0.646743973	0.843737294
MTCO1P5	-0.016	0.646740451	0.843737294
ZNF449	-0.016	0.646697675	0.843737294
RCC2	-0.016	0.646678058	0.843737294
CICP7	-0.016	0.646657752	0.843737294
AC009014	-0.016	0.646596929	0.843734676
RP1-76C18	-0.016	0.646535411	0.843677517
AL360078	-0.016	0.646447553	0.843585983
CDY2B	-0.016	0.646447553	0.843585983
PRIM2	-0.016	0.646341859	0.843540507
AC024571	-0.016	0.64627013	0.843470007
CCDC136	-0.016	0.646204968	0.843461825
CTAGE8	-0.016	0.646198611	0.843461825
ARHGEF1	-0.016	0.646191633	0.843461825
HIGD1AP10	-0.016	0.646191074	0.843461825
UBE2Q2P11	-0.016	0.646179642	0.843461825
RP11-5P15	-0.016	0.646084883	0.843436251
HMGB3P31	-0.016	0.646027572	0.843384551
RP11-252O18	-0.016	0.645978372	0.843356509
CTD-2545M3	-0.016	0.645969355	0.843356509
RMST	-0.016	0.645852767	0.843271917
AC114296	-0.016	0.645765648	0.843216796
EPS8L3	-0.016	0.64571891	0.843216796
CTD-2653B5	-0.016	0.645668979	0.843216796
RP11-629F19	-0.016	0.645652258	0.843216796
LINC01440	-0.016	0.645595383	0.843216796
ZNF467	-0.016	0.645564602	0.843216796
OR5BB1P	-0.016	0.645520295	0.843207677
OR7E159P	-0.016	0.645433844	0.843164125
GTPBP3	-0.016	0.645308215	0.843069383
RP11-332K15	-0.016	0.645301261	0.843069383
AL360217	-0.016	0.645272341	0.843069383
RNU6-1312P	-0.016	0.645269753	0.843069383
AC013401	-0.016	0.645250686	0.843069383
LINC00538	-0.016	0.645247291	0.843069383
AP006222	-0.016	0.645108685	0.843016829
RP11-257K9	-0.016	0.645064134	0.842981739

AC226119	-0.016	0.645041629	0.84297546
RP11-254B13	-0.016	0.645026003	0.84297546
LINC01509	-0.016	0.644903951	0.842888048
AC005394	-0.016	0.644750146	0.842802668
NUPL1P1	-0.016	0.64461788	0.84272229
CTD-2623H2	-0.016	0.644607142	0.84272229
RN7SKP14	-0.016	0.644601404	0.84272229
GATA5	-0.016	0.644591458	0.84272229
CD27-AS1	-0.016	0.64454205	0.842715684
RP11-80F22	-0.016	0.644494867	0.842677127
RP11-678K21	-0.016	0.644440987	0.842677127
DNAJC21	-0.016	0.644381027	0.842650207
CTB-51A17	-0.016	0.644372153	0.842650207
MRPS33P4	-0.016	0.644353161	0.842650207
RN7SKP78	-0.016	0.644311001	0.842644919
MIR6855	-0.016	0.6442956	0.842644919
RNU6-320P	-0.016	0.644260327	0.842644919
MTND4P13	-0.016	0.644244606	0.842644919
RN7SKP96	-0.016	0.644238765	0.842644919
FAM135B	-0.016	0.644237894	0.842644919
RP11-1096D5	-0.016	0.644176113	0.842644919
SMIM19	-0.016	0.644171447	0.842644919
RP11-660M18	-0.016	0.644167544	0.842644919
C16orf74	-0.016	0.644142069	0.842644919
FTLP10	-0.016	0.644034504	0.84260724
PILRA	-0.016	0.643977171	0.842555374
CCT6B	-0.016	0.643923514	0.842508316
POLR3C	-0.016	0.643887483	0.842484318
PDE7A	-0.016	0.643876126	0.842484318
C20orf181	-0.016	0.643808409	0.842450287
EXTL3-AS1	-0.016	0.643781547	0.842438282
POTEM	-0.016	0.643753989	0.842425367
TBL1XR1-AS1	-0.016	0.643729754	0.842425367
AC004951	-0.016	0.643632938	0.842382686
MAPKAP1	-0.016	0.643601613	0.842364836
MIR6886	-0.016	0.643572605	0.842353648
MIR6893	-0.016	0.643569519	0.842353648
GK-IT1	-0.016	0.643478333	0.842296072
FAR2P3	-0.016	0.64346695	0.842296072
LINC00540	-0.016	0.643393175	0.842273836
RP1-288M22	-0.016	0.643385131	0.842273836
RP11-710F7	-0.016	0.643332152	0.842273836
GPR62	-0.016	0.643220969	0.842236964
AC004850	-0.016	0.643155149	0.842173932
OVOL2	-0.016	0.643088353	0.842132774
RNA5SP181	-0.016	0.642989247	0.842095608
RP11-697H10	-0.016	0.642899726	0.842024674
RP1-266L20	-0.016	0.642835505	0.842010028
ODAM	-0.016	0.642778531	0.841981921
SCAMP4	-0.016	0.642744318	0.841981921
RP11-231C18	-0.016	0.642727994	0.841981921
MYH7B	-0.016	0.642653136	0.841956397

CTC-329D1	-0.016	0.642527916	0.8418155
RP11-79E3	-0.016	0.642524641	0.8418155
RP11-435F17	-0.016	0.642506004	0.8418155
TMEM218	-0.016	0.642447703	0.841779877
AC007161	-0.016	0.642339386	0.841704242
HERC2P8	-0.016	0.642292607	0.841672833
RPA4	-0.016	0.642258436	0.841672833
RP11-67P15	-0.016	0.642215779	0.841661234
AC003006	-0.016	0.642207306	0.841661234
OR10W1	-0.016	0.642202343	0.841661234
ZNF793-AS1	-0.016	0.642167184	0.841661234
VASH1	-0.016	0.64216288	0.841661234
CTD-2236F14	-0.016	0.641993215	0.841515524
RN7SL740P	-0.016	0.641917558	0.841455633
RP11-571E6	-0.016	0.64191026	0.841455633
PGA3	-0.016	0.641892949	0.841455633
RP11-945C19	-0.016	0.641738372	0.841313371
AL138710	-0.016	0.641686836	0.841292123
AL359091	-0.016	0.641659827	0.841279871
RNU7-45P	-0.016	0.641606424	0.841233012
ZNF672	-0.016	0.641573841	0.841213449
RP11-556H2	-0.016	0.641507267	0.841149316
OR13D1	-0.016	0.641485177	0.841143508
AP000949	-0.016	0.641392007	0.84109081
CPB1	-0.016	0.64136001	0.841072008
ZFP28	-0.016	0.64133297	0.841059707
RP11-232C2	-0.016	0.641313319	0.841057094
AC119403	-0.016	0.641288128	0.841053531
ATP6V0E1P2	-0.016	0.641221527	0.841029347
RN7SL362P	-0.016	0.641203654	0.841029067
SNORD105	-0.016	0.641132672	0.840959123
RP11-167B3	-0.016	0.641060313	0.84088737
SWAP70	-0.016	0.641047572	0.84088737
KLHL24	-0.016	0.641015908	0.840875441
AC005559	-0.016	0.640970234	0.840838687
RP11-737F10	-0.016	0.640905837	0.840800527
RNU6-506P	-0.016	0.640765603	0.840718431
INPP1	-0.016	0.640729787	0.840718431
FAM98C	-0.016	0.640676242	0.840684577
GPR68	-0.016	0.64046482	0.840488666
RP11-395F4	-0.016	0.64041055	0.840474877
SNORD114-29	-0.016	0.640352229	0.84046064
TMA16P1	-0.016	0.640333212	0.84046064
AL596092	-0.016	0.640252632	0.840454387
AC243562	-0.016	0.640201746	0.84043243
RP11-1220K2	-0.016	0.640152991	0.84039159
RP11-1113N2	-0.016	0.640095344	0.84036224
SNRPCP1	-0.016	0.640093614	0.84036224
KRT8P35	-0.016	0.64005685	0.840358032
PRKAA2	-0.016	0.639938541	0.84025433
TAS2R1	-0.016	0.639902475	0.84025433
CORO1B	-0.016	0.639860024	0.84025433

EIF4BP4	-0.016	0.639742421	0.840214512
ZDHHC16	-0.016	0.639735813	0.840214512
EIF3E	-0.016	0.639725267	0.840214512
GS1-39E22	-0.016	0.639698341	0.840214512
LINC00700	-0.016	0.639677733	0.840214512
RP11-7M10	-0.016	0.639660623	0.840214512
RN7SL567P	-0.016	0.639640258	0.840214512
RP11-498J9	-0.016	0.639538067	0.840214512
SLC2A7	-0.016	0.639499388	0.840205168
SRSF1P1	-0.016	0.639497724	0.840205168
AC006277	-0.016	0.639463599	0.840205168
RP11-76P2	-0.016	0.639453139	0.840205168
LINC01217	-0.016	0.639408023	0.840205168
VNN1	-0.016	0.639367535	0.840205168
LATS2-AS1	-0.016	0.639283242	0.840176136
RP5-981L23	-0.016	0.639249643	0.840155162
RPS12P27	-0.016	0.639102515	0.839984973
TMEM247	-0.016	0.638938227	0.839815398
EIF1AXP1	-0.016	0.638894316	0.839780857
KRT8P31	-0.016	0.638845799	0.839740261
RN7SL20P	-0.016	0.638617522	0.839463367
HPCAL1	-0.016	0.638614461	0.839463367
HSP90B2P	-0.016	0.638610752	0.839463367
GPHB5	-0.016	0.638588368	0.839463367
AL355593	-0.016	0.638566547	0.839463367
RP1-263J7	-0.016	0.638408319	0.839304193
FEM1A	-0.016	0.638299522	0.83920749
AC069155	-0.016	0.63814499	0.839120139
CTNND1	-0.016	0.638025223	0.839008981
AC026185	-0.016	0.638022135	0.839008981
AC073657	-0.016	0.638021425	0.839008981
C7orf43	-0.016	0.63801781	0.839008981
RP11-839G9	-0.016	0.637864681	0.838890516
POLRMTP1	-0.016	0.63778722	0.838888795
CMP21-97G8	-0.016	0.637747095	0.838888795
RP11-30K9	-0.016	0.637659501	0.838805973
LINC01498	-0.016	0.637628371	0.83878819
ST6GALNAC4	-0.016	0.637406963	0.838566417
PTCHD3P3	-0.016	0.637163738	0.838339061
TANC2	-0.016	0.637079407	0.838320742
AC008060	-0.016	0.63701142	0.838300763
PCGEM1	-0.016	0.636943316	0.838274763
RP11-381O7	-0.016	0.636931112	0.838274763
CTD-2547L16	-0.016	0.636898502	0.838267975
RNASEH2C	-0.016	0.636854448	0.838256324
RN7SL357P	-0.016	0.636734842	0.838122055
POMGNT1	-0.016	0.63673399	0.838122055
UGGT1	-0.016	0.636656029	0.838064639
OPRL1	-0.016	0.636626015	0.838048292
G6PC3	-0.016	0.636613152	0.838048292
MIR3689D2	-0.016	0.636567626	0.838017754
GPATCH2	-0.016	0.636404241	0.837825823

MIR4781	-0.016	0.636359908	0.837790616
LLNLR-470E3	-0.016	0.636302314	0.837737949
NCKAP1	-0.016	0.636261217	0.837706998
RP4-735C1	-0.016	0.636207454	0.837669959
MAK16	-0.016	0.636187566	0.837669959
AKAP4	-0.016	0.636161325	0.837668108
RP11-697E23	-0.016	0.636009792	0.837538042
MIR4270	-0.016	0.635897644	0.837413513
RP11-427E4	-0.016	0.635879952	0.837413368
BAP1	-0.016	0.635749244	0.837310693
RNU6-190P	-0.016	0.635702025	0.837310693
AC100821	-0.016	0.635535793	0.837169452
CYP51A1P2	-0.016	0.635478845	0.837162918
AC099850	-0.016	0.635329354	0.83703544
TVP23CP1	-0.016	0.635327052	0.83703544
AP005227	-0.016	0.635288251	0.83703544
AC004915	-0.016	0.635145565	0.836909056
RP11-218L14	-0.016	0.635141556	0.836909056
MEAF6P1	-0.016	0.635139477	0.836909056
RP11-527J8	-0.016	0.635039628	0.836862078
RPL39P29	-0.016	0.6349845	0.836857744
MCCD1	-0.016	0.634839652	0.836714305
CD47	-0.016	0.634830546	0.836714305
LINC00837	-0.016	0.634794403	0.836714305
RP11-388M20	-0.016	0.634786293	0.836714305
BNIP3P23	-0.016	0.634742584	0.836714305
RP11-795F19	-0.016	0.634733997	0.836714305
AC011498	-0.016	0.63469574	0.836714305
FAM86HP	-0.016	0.634684167	0.836714305
RN7SL648P	-0.016	0.634586928	0.836682213
LINC00265-3P	-0.016	0.634507097	0.836623281
RP11-731J8	-0.016	0.634494036	0.836623281
RNA5SP351	-0.016	0.634437441	0.836600919
UBA52P5	-0.016	0.63443445	0.836600919
AC010082	-0.016	0.634381046	0.836572879
KLHL3	-0.016	0.63433852	0.836563125
RP11-3P17	-0.016	0.634288314	0.836543242
EPHA6	-0.016	0.634286341	0.836543242
CBX3P1	-0.016	0.634227867	0.836533015
RNA5SP415	-0.016	0.634195392	0.836523194
RP11-928F19	-0.016	0.634133418	0.836501103
CTD-2336H13	-0.016	0.634112127	0.836496186
CTD-2023J5	-0.016	0.634007503	0.836385518
RN7SL122P	-0.016	0.633876391	0.836254699
RP11-262M14	-0.016	0.633874538	0.836254699
CTD-2004A9	-0.016	0.633860415	0.836254699
INSL4	-0.016	0.633832486	0.836254699
RP4-555L14	-0.016	0.633802428	0.836249778
RN7SKP177	-0.016	0.633714279	0.836202969
STARD3	-0.016	0.633704262	0.836202969
CTC-430J12	-0.016	0.633678707	0.836202969
RP11-45F15	-0.016	0.63366445	0.836202969

MGAT3		-0.016	0.633639684	0.836202969
RNU6-1322P		-0.016	0.633630584	0.836202969
RN7SL677P		-0.016	0.633578776	0.836186338
RP11-26L16		-0.016	0.633545547	0.836186338
TMCC2		-0.016	0.633520261	0.836178624
RP11-720L2		-0.016	0.63341213	0.836065615
RP11-296A16		-0.016	0.633408947	0.836065615
FOXG1-AS1		-0.016	0.63329447	0.835973273
	6-Mar	-0.016	0.633135215	0.835786216
CTD-3018O17		-0.016	0.633094705	0.835755904
PRKAG2-AS1		-0.016	0.633062884	0.835737062
RP11-242F11		-0.016	0.63305526	0.835737062
DNAJB12		-0.016	0.633032164	0.835737062
AC093802		-0.016	0.632965866	0.835678475
RP11-281B1		-0.016	0.632907357	0.835647557
AC004016		-0.016	0.632845693	0.835647557
AC018865		-0.016	0.632813815	0.835639881
CTAG1A		-0.016	0.63276375	0.835596937
RP11-709P2		-0.016	0.632761663	0.835596937
FABP7P1		-0.016	0.632756108	0.835596937
AL353135		-0.016	0.632704623	0.835588361
SMIM6		-0.016	0.632682697	0.835588361
RP11-451O13		-0.016	0.632516968	0.835479536
GLI1		-0.016	0.63245521	0.835444301
RP11-24C3		-0.016	0.632339132	0.835360146
RNA5SP261		-0.016	0.632328048	0.835360146
TNRC18P1		-0.016	0.632284899	0.835357403
RP11-630H24		-0.016	0.632276828	0.835357403
RP11-536P16		-0.016	0.632190424	0.835349388
RP11-603J24		-0.016	0.632119404	0.83528787
RP11-358N4		-0.016	0.632072569	0.83528787
GS1-279B7		-0.016	0.632068106	0.83528787
PIGFP3		-0.016	0.632036805	0.83528787
TAF9P2		-0.016	0.632011782	0.83528787
PCNA		-0.016	0.631986114	0.83528787
NUCKS1		-0.016	0.631965907	0.83528787
RNU6-719P		-0.016	0.631962736	0.83528787
RP11-85E16		-0.016	0.631941072	0.83528787
MIR6799		-0.016	0.631912978	0.83528787
RP11-571L19		-0.016	0.631880811	0.83528787
AC027124		-0.016	0.631795921	0.835253379
RP11-173D3		-0.016	0.631727095	0.835223783
VN1R88P		-0.016	0.631669856	0.835194476
AC136611		-0.016	0.631581635	0.835101015
NEAT1		-0.016	0.631563781	0.835100592
CST9LP1		-0.016	0.631558371	0.835100592
TRAJ57		-0.016	0.631546873	0.835100592
RNU6-720P		-0.016	0.631508302	0.835100592
HOXB13-AS1_2		-0.016	0.631460533	0.835079992
TXLNA		-0.016	0.631424714	0.835055809
TMEM179B		-0.016	0.631367614	0.835018864
AC068533		-0.016	0.631302739	0.835005317

ADAD1P2	-0.016	0.631292611	0.835005317
KRT18P68	-0.016	0.631225319	0.835000519
RP4-735N21	-0.016	0.631213994	0.835000519
LINC01568	-0.016	0.631176018	0.834991866
RP11-384L8	-0.016	0.631096131	0.834922651
CYP4F23P	-0.016	0.631090563	0.834922651
CHTOP	-0.016	0.630962544	0.83483958
RN7SL692P	-0.016	0.630922532	0.83483958
ZSCAN2	-0.016	0.630893425	0.83483958
RP11-17E13	-0.016	0.630879638	0.83483958
EARS2	-0.016	0.630843723	0.83483958
RP11-397H6	-0.016	0.63082025	0.83483958
ASUN	-0.016	0.630804895	0.83483958
PFN1P10	-0.016	0.630804534	0.83483958
FOXD4L1	-0.016	0.630727455	0.83483958
AC068490	-0.016	0.630684676	0.83483958
FAIM2	-0.016	0.630648428	0.83483958
RNU6-48P	-0.016	0.630607405	0.83483958
ECHDC2	-0.016	0.630585147	0.83483958
RP11-343H19	-0.016	0.630553467	0.834830844
TACC1P1	-0.016	0.630475085	0.834784865
AIFM1	-0.016	0.630329721	0.834650623
CYP2A7P1	-0.016	0.630264434	0.83459814
GLYATL1P3	-0.016	0.630117292	0.834438934
FAM108A9P	-0.016	0.630087997	0.83442334
SPA17	-0.016	0.630087146	0.83442334
CCND2	-0.016	0.630016128	0.834374565
KRTAP4-4	-0.016	0.629982422	0.834353127
CDKN2AIPNLP2	-0.016	0.629901455	0.834292295
RN7SL449P	-0.016	0.629841844	0.83423654
ATP7A	-0.016	0.629782916	0.834181688
RP11-193I22	-0.016	0.629775212	0.834181688
SHE	-0.016	0.629742952	0.834181688
RN7SL234P	-0.016	0.629722847	0.834181688
XKR9	-0.016	0.629682486	0.834164657
MIR4715	-0.016	0.629635507	0.834125624
RP11-196H14	-0.016	0.629612575	0.834118446
RP13-608F4	-0.016	0.629417024	0.833952167
MAPKBP1	-0.016	0.629315052	0.833863454
CTB-33O18	-0.016	0.629302251	0.833863454
HMGB1P18	-0.016	0.629247701	0.833861178
RPS4XP18	-0.016	0.629203532	0.833854878
OR2B11	-0.016	0.629202262	0.833854878
SH3GLB1	-0.016	0.629154089	0.833835758
CRYGC	-0.016	0.629065438	0.833741467
AC034228	-0.016	0.629059397	0.833741467
LINC00840	-0.016	0.629025184	0.833741467
TAF9BP2	-0.016	0.628801949	0.833508222
FAM110D	-0.016	0.628695342	0.833459697
PAWR	-0.016	0.62862788	0.83339346
RN7SL480P	-0.016	0.62860662	0.833388472
AC006026	-0.016	0.62859925	0.833388472

NEK11	-0.016	0.628416984	0.833203747
METTL22	-0.016	0.628376282	0.833203747
HNRNPA1P55	-0.016	0.628371001	0.833203747
RNA5SP130	-0.016	0.628355624	0.833203747
RP11-344B23	-0.016	0.628301308	0.833198316
RNU2-55P	-0.016	0.62822515	0.83313784
RN7SL326P	-0.016	0.628222205	0.83313784
MIR548D2	-0.016	0.628196821	0.83313784
ERCC6-PGBD3	-0.017	0.628143582	0.83313784
RP11-156E8	-0.017	0.628127305	0.83313784
RNU1-65P	-0.017	0.628041264	0.833079551
CTB-99A3	-0.017	0.62795559	0.83303551
ZNF101P2	-0.017	0.627880172	0.832981865
EEF1A1P7	-0.017	0.627852677	0.832972703
RP11-718I15	-0.017	0.627845691	0.832972703
PRKCA-AS1	-0.017	0.627557232	0.832762205
RN7SL566P	-0.017	0.627533049	0.832753317
CTD-2340E1	-0.017	0.627363648	0.832598113
AC007272	-0.017	0.627241596	0.832482527
OR51A5P	-0.017	0.62723821	0.832482527
ZNF174	-0.017	0.627231259	0.832482527
PPP1R1AP1	-0.017	0.627142006	0.832482527
RP11-159A18	-0.017	0.626949987	0.832350645
OR2A42	-0.017	0.626893565	0.83232214
SNX18P23	-0.017	0.62685432	0.83231644
AL049539	-0.017	0.626583229	0.832095679
RP11-813B8	-0.017	0.626356346	0.831956764
AL133153	-0.017	0.626272696	0.831868856
CDK8	-0.017	0.626193183	0.831843045
NANOGP3	-0.017	0.62616288	0.831838986
RN7SL11P	-0.017	0.626153826	0.831838986
RNA5SP77	-0.017	0.626010583	0.831729462
MIR8057	-0.017	0.625935527	0.831699346
VAC14-AS1	-0.017	0.625870789	0.831659731
RNU6-220P	-0.017	0.625866175	0.831659731
REG4	-0.017	0.625736149	0.831527221
LRCH1	-0.017	0.625533009	0.831442861
MIR3690	-0.017	0.625402317	0.831338753
RP11-465N4	-0.017	0.625314049	0.831244619
KRTAP29-1	-0.017	0.625184051	0.831183792
GPR107	-0.017	0.625153525	0.831170424
AC007365	-0.017	0.625122583	0.831152487
STX12	-0.017	0.625093575	0.831152487
MYT1L-AS1	-0.017	0.625055459	0.831152487
ABHD11	-0.017	0.624946224	0.831034002
AC109335	-0.017	0.624942221	0.831034002
RP11-563M4	-0.017	0.624876613	0.830987838
ZNF322P1	-0.017	0.624875362	0.830987838
CACNA1H	-0.017	0.624836784	0.830981278
RNMTL1P2	-0.017	0.624749225	0.830892518
RP11-190P13	-0.017	0.624740937	0.830892518
MT4	-0.017	0.624729599	0.830892518

FAM169B	-0.017	0.624547858	0.830786296
RP11-25I9	-0.017	0.62441289	0.830695886
RP11-83B20	-0.017	0.624382855	0.830679131
ARHGEF28	-0.017	0.624382484	0.830679131
HNRNPCP2	-0.017	0.624345373	0.830675674
RP11-181C3	-0.017	0.624202344	0.830531779
ATP5G1P1	-0.017	0.62418808	0.830531779
EID2B	-0.017	0.624079053	0.830437333
MIR3941	-0.017	0.624028605	0.830403861
METTL21B	-0.017	0.623967359	0.830358306
KIF22	-0.017	0.623964654	0.830358306
RP11-419K12	-0.017	0.623958785	0.830358306
RP11-262H14	-0.017	0.623932191	0.830358306
CTC-554D6	-0.017	0.623901578	0.830358306
MIR3146	-0.017	0.623773807	0.830239922
GUCA1C	-0.017	0.623718871	0.830213204
RAP1B	-0.017	0.623631559	0.830120352
RNU6-769P	-0.017	0.623551734	0.830106732
RP11-266E6	-0.017	0.623531421	0.830102893
NCF2	-0.017	0.623407864	0.829997239
CXCL1P	-0.017	0.623385999	0.829997239
TRAJ51	-0.017	0.623362399	0.829997239
AC009531	-0.017	0.62335293	0.829997239
OR10A4	-0.017	0.623287596	0.829987092
RP11-95O2	-0.017	0.623181854	0.829893762
SNORD116-15	-0.017	0.623163247	0.829893762
MED4	-0.017	0.62314133	0.829893762
AL157904	-0.017	0.623129413	0.829893762
RNU6-318P	-0.017	0.623111184	0.829893762
AC034236	-0.017	0.623026053	0.829870842
NDUFB4P1	-0.017	0.622965994	0.829814051
PNO1	-0.017	0.622955944	0.829814051
RNU6-939P	-0.017	0.622933742	0.829814051
AL356217	-0.017	0.622878678	0.829795487
MED26	-0.017	0.622859398	0.829795487
CTB-7E3	-0.017	0.622656614	0.829681329
KRTAP5-3	-0.017	0.622629337	0.829681329
ISCA2P1	-0.017	0.622613305	0.829681329
CMB9-14B22	-0.017	0.622497908	0.829584976
MIR6839	-0.017	0.622489981	0.829584976
AC007326	-0.017	0.622451053	0.829584976
LINC01153	-0.017	0.622426975	0.829584976
OSCP1	-0.017	0.622396841	0.829584976
NFYAP1	-0.017	0.622328554	0.829584976
LPAL2	-0.017	0.622296054	0.829584976
AC104073	-0.017	0.622281145	0.829584976
GRIK5	-0.017	0.622237413	0.829584976
FCF1P7	-0.017	0.622136184	0.829497495
RP3-477M7	-0.017	0.622112681	0.82948938
RP4-666F24	-0.017	0.622095878	0.82948938
GAPDHP58	-0.017	0.622085748	0.82948938
C17orf61-PLSCR3	-0.017	0.62199493	0.829480001

LINC01047	-0.017	0.621716347	0.82914663
RP1-78B3	-0.017	0.621690297	0.82914663
PRSS21	-0.017	0.621627475	0.829097755
RP1-178F10	-0.017	0.621461754	0.828969754
RPL31P3	-0.017	0.621377941	0.828904216
SNORA7B	-0.017	0.621329864	0.828886514
KRT19P4	-0.017	0.621234749	0.82878284
SUV39H2	-0.017	0.621219112	0.82878284
RP11-24P14	-0.017	0.621205539	0.82878284
COMMD3	-0.017	0.621069071	0.828631609
AC133633	-0.017	0.621049115	0.828631609
LIN37	-0.017	0.62100042	0.828609486
ALG10	-0.017	0.62087624	0.828490213
AC018867	-0.017	0.620829224	0.828473898
RP11-380G5	-0.017	0.620689009	0.828356416
DPRX	-0.017	0.62065576	0.828335255
TCEAL1	-0.017	0.620638707	0.828335255
TVP23BP1	-0.017	0.620594337	0.828335255
CTD-2012B7	-0.017	0.620588581	0.828335255
CTB-55P19	-0.017	0.620556295	0.828335255
SNORD115-37	-0.017	0.620553187	0.828335255
AC093304	-0.017	0.620531714	0.828335255
TRIM51DP	-0.017	0.620485704	0.828335255
XX-2136C77	-0.017	0.620473722	0.828335255
RPS15AP11	-0.017	0.620358262	0.828286365
CENPN	-0.017	0.620157208	0.828134003
NIPA2P5	-0.017	0.620122772	0.828134003
SLC7A4	-0.017	0.620084834	0.828107013
UBTFL5	-0.017	0.619980929	0.827991467
LINC00488	-0.017	0.619970738	0.827991467
SRP54-AS1	-0.017	0.619968354	0.827991467
RP11-243E13	-0.017	0.61995502	0.827991467
RNU6-709P	-0.017	0.619904345	0.827991467
DNM2	-0.017	0.619795323	0.827952539
MIR4515	-0.017	0.619682521	0.827825071
AP001137	-0.017	0.619621239	0.827766423
OR5J1P	-0.017	0.619610114	0.827766423
SGPP2	-0.017	0.619535239	0.827697968
GOLGA7	-0.017	0.619532543	0.827697968
LCE2C	-0.017	0.619498236	0.827697968
RP11-104G3	-0.017	0.61941149	0.827671942
RP11-638I8	-0.017	0.619401941	0.827671942
RNU6-946P	-0.017	0.619231493	0.827563075
MIR3692	-0.017	0.619222636	0.827563075
MTND4P3	-0.017	0.619176622	0.827563075
SH3RF1	-0.017	0.619160802	0.827563075
GID8	-0.017	0.619151943	0.827563075
PRAMEF14	-0.017	0.619143515	0.827563075
RP11-410C4	-0.017	0.619022865	0.827500897
AC009196	-0.017	0.618992064	0.827482949
AC099332	-0.017	0.618983223	0.827482949
C19orf71	-0.017	0.618874647	0.827418882

AC009065	-0.017	0.618822537	0.827372438
RPS12P23	-0.017	0.618794561	0.827372438
CEP83-AS1	-0.017	0.618750652	0.827372438
RPA1	-0.017	0.61869867	0.827372438
POTEJ	-0.017	0.618646688	0.827360649
RP11-63G10	-0.017	0.618611077	0.827360649
TTR	-0.017	0.618609499	0.827360649
RP11-80I15	-0.017	0.618595384	0.827360649
RP11-774I5	-0.017	0.618557931	0.827360649
RP11-99E15	-0.017	0.618467053	0.827354494
RP11-157E14	-0.017	0.618412748	0.827354494
RP11-251G23	-0.017	0.618371032	0.827349415
GPRC5A	-0.017	0.61828057	0.827305123
SSXP3	-0.017	0.618252491	0.827305123
IFNA12P	-0.017	0.618252255	0.827305123
MARK2P9	-0.017	0.618056432	0.827137622
GPR146	-0.017	0.618030385	0.827126005
CCDC159	-0.017	0.617960341	0.827078745
OR4C4P	-0.017	0.617909343	0.827033731
SLCO1A2	-0.017	0.617898732	0.827033731
ERC2-IT1	-0.017	0.617855025	0.827033731
LACE1	-0.017	0.617846046	0.827033731
RP11-403E24	-0.017	0.617823146	0.827033731
RP11-98J9	-0.017	0.617802551	0.827033731
RPL3P10	-0.017	0.617772518	0.827033731
AC011410	-0.017	0.617745949	0.827033731
MIR6129	-0.017	0.617680803	0.827026468
MTHFD2P2	-0.017	0.617578515	0.826939524
PIEZO1P1	-0.017	0.617547822	0.826921674
DUX4L27	-0.017	0.617522646	0.826911212
AC092642	-0.017	0.617485684	0.826911212
XXyac-YR29IB3	-0.017	0.617466012	0.826911212
RP11-159K7	-0.017	0.617419696	0.826911212
RP11-428G2	-0.017	0.617371429	0.826889783
CLK2	-0.017	0.617370108	0.826889783
AC008940	-0.017	0.617345569	0.826889783
RNU5B-4P	-0.017	0.617277447	0.826889783
UBE2L4	-0.017	0.617181788	0.826826728
TPGS2	-0.017	0.617178932	0.826826728
ALG6	-0.017	0.617138931	0.826826728
RP11-14J16	-0.017	0.617073589	0.826798059
SLC5A2	-0.017	0.616998694	0.826744226
MIR1265	-0.017	0.616939248	0.826734351
KRT18P19	-0.017	0.616922747	0.826734351
CTD-2552B11	-0.017	0.616851321	0.826663042
AIP	-0.017	0.616830881	0.826658911
RNU4-31P	-0.017	0.616752514	0.826600404
RBM17	-0.017	0.61673821	0.826600404
PRELID1P3	-0.017	0.616647102	0.826528904
AC004543	-0.017	0.616527644	0.826406812
CICP16	-0.017	0.616345149	0.826285446
AL109618	-0.017	0.616234456	0.82619507

RP11-327L9	-0.017	0.616193423	0.82619507
RNU6-986P	-0.017	0.616177626	0.82619507
APOOP1	-0.017	0.616021212	0.82613173
CCDC42B	-0.017	0.616004551	0.82613173
RP11-351M8	-0.017	0.615981493	0.826124988
Six3os1_5	-0.017	0.615851159	0.82599671
RP11-175D17	-0.017	0.615712937	0.825904355
RP11-47F1	-0.017	0.615704724	0.825904355
AMY1B	-0.017	0.615674048	0.825898712
PRAMEF12	-0.017	0.615657157	0.825898712
KLHL7-AS1	-0.017	0.615628347	0.82589417
SLC25A44	-0.017	0.615596282	0.82588744
TRDN	-0.017	0.615563599	0.825866857
HSD17B1P1	-0.017	0.615529623	0.825844537
APOC3	-0.017	0.615491912	0.825817204
AL132796	-0.017	0.615302726	0.825702937
RP11-133M8	-0.017	0.615288464	0.825702937
RP11-304C12	-0.017	0.61522208	0.825641241
AC090150	-0.017	0.615156518	0.825576518
RP5-1046G13	-0.017	0.615136564	0.825573003
LCE4A	-0.017	0.615017178	0.825482559
ZNF584	-0.017	0.615006272	0.825482559
C14orf79	-0.017	0.614912953	0.825434526
MTND5P19	-0.017	0.614905009	0.825434526
MIR548J	-0.017	0.614886113	0.825434526
TNK1	-0.017	0.614800442	0.825401004
CMTM4	-0.017	0.61473363	0.825334572
RNU6-1237P	-0.017	0.614706264	0.825334572
RP11-143M1	-0.017	0.614577097	0.825263996
EFCC1	-0.017	0.614540125	0.825237616
GIMAP1-GIMAP5	-0.017	0.614534427	0.825237616
ZNF793	-0.017	0.614474093	0.825218747
SMCR2	-0.017	0.614453947	0.825218747
AC097455	-0.017	0.614353458	0.825148572
RP13-152O15	-0.017	0.614345075	0.825148572
RNA5SP233	-0.017	0.614341564	0.825148572
RP5-936J12	-0.017	0.614333807	0.825148572
LRRC72	-0.017	0.61431105	0.825148572
RNU6-251P	-0.017	0.614232515	0.825148572
AL590723	-0.017	0.614215584	0.825148572
RP3-424M6	-0.017	0.614038058	0.824958831
RECQL	-0.017	0.613900673	0.824844059
YWHAG	-0.017	0.613884173	0.824844059
SUMO2P4	-0.017	0.613860424	0.824839712
BRWD1-IT1	-0.017	0.613826238	0.824837125
SPRED2	-0.017	0.613801289	0.824837125
TAF9BP1	-0.017	0.613785976	0.824837125
CTD-2528A14	-0.017	0.613753806	0.824832882
RP11-736N17	-0.017	0.613711731	0.824799611
RP4-630J13	-0.017	0.613624937	0.824706235
RP13-672B3	-0.017	0.613611252	0.824706235
RP11-767L7	-0.017	0.613507201	0.824621003

RN7SL636P	-0.017	0.613387685	0.824550248
RP11-18D7	-0.017	0.613362992	0.824540326
RP11-79O8	-0.017	0.613300161	0.824479134
NDUFB1P2	-0.017	0.613204734	0.824390637
RP11-513D4	-0.017	0.613196554	0.824390637
ZNRF3	-0.017	0.613154573	0.824376492
LINC00955	-0.017	0.613107964	0.824376492
OFD1P17	-0.017	0.613035689	0.824376492
MIR548AL	-0.017	0.613010832	0.824369406
AL158141	-0.017	0.612975525	0.824363284
NATP	-0.017	0.61282086	0.824207022
LINC01054	-0.017	0.612793658	0.824193713
TNK2	-0.017	0.612785214	0.824193713
MIR6859-4	-0.017	0.612670581	0.82414034
LUC7L2	-0.017	0.612667878	0.82414034
RAB11FIP1	-0.017	0.612656004	0.82414034
SSR4	-0.017	0.612610381	0.824133396
MTND5P29	-0.017	0.612486977	0.824060489
RNA5SP228	-0.017	0.612467239	0.824057212
MIR548AG2	-0.017	0.612454219	0.824057212
AP000436	-0.017	0.612318298	0.823903367
ANKDD1B	-0.017	0.612311893	0.823903367
MIR657	-0.017	0.612219229	0.823879314
PIWIL3	-0.017	0.612140838	0.823827508
VN1R28P	-0.017	0.612089084	0.823781135
RP11-119D18	-0.017	0.61206746	0.823775311
TXNP3	-0.017	0.612001483	0.823756349
RPL31P40	-0.017	0.611981115	0.823752214
RN7SKP265	-0.017	0.611918226	0.823690841
ZNF879	-0.017	0.611912419	0.823690841
RP11-359G22	-0.017	0.611906642	0.823690841
BIVM	-0.017	0.611879849	0.823690841
IFI27L1	-0.017	0.611697851	0.823557132
SMAD5-AS1_2	-0.017	0.611666071	0.823557132
AP000361	-0.017	0.611654958	0.823557132
AEBP2	-0.017	0.611644214	0.823557132
ZNF324	-0.017	0.611552653	0.823478037
RP11-806J6	-0.017	0.611548096	0.823478037
ERICH6-AS1	-0.017	0.611490594	0.823441034
DBX1	-0.017	0.611479274	0.823441034
WDR93	-0.017	0.611464545	0.823441034
LINC00383	-0.017	0.611360602	0.823359105
RP11-74D3	-0.017	0.611235585	0.823237295
S1PR3	-0.017	0.611225534	0.823237295
HMGB3P26	-0.017	0.611180669	0.82320989
WFS1	-0.017	0.611042473	0.823093584
PARP4P2	-0.017	0.610977215	0.823052237
CTB-51E13	-0.017	0.610792302	0.82289624
RPS7P10	-0.017	0.61077099	0.822890804
RN7SL846P	-0.017	0.610733837	0.822887305
ZG16	-0.017	0.610718616	0.822887305
PSMC1P4	-0.017	0.610709582	0.822887305

CEACAM6	-0.017	0.610655174	0.822874434
WFDC10A	-0.017	0.610590107	0.822842327
RNU7-143P	-0.017	0.610527846	0.822842327
RP11-148I19	-0.017	0.610519197	0.822842327
RN7SL81P	-0.017	0.610489956	0.822842327
PABPC1P2	-0.017	0.610377317	0.822756077
RP1-85F18	-0.017	0.610300555	0.822699175
CPEB2-AS1	-0.017	0.610281352	0.822696573
RTN1	-0.017	0.610075104	0.82248838
ZNF70P1	-0.017	0.609988422	0.822394796
MIR6802	-0.017	0.6099317	0.822341602
SDF2	-0.017	0.609907501	0.822332255
RNA5SP199	-0.017	0.609896648	0.822332255
ZNF699	-0.017	0.60980649	0.822265896
RP11-727F15	-0.017	0.609801516	0.822265896
MAN2B2	-0.017	0.609752162	0.822262478
TRPT1	-0.017	0.60955782	0.822140074
BCL2L15	-0.017	0.609425852	0.822055202
RP11-670N15	-0.017	0.609383814	0.822021778
HIST1H2BPS3	-0.017	0.609346304	0.82199446
RP1-136J15	-0.017	0.609293143	0.821946028
AC008103	-0.017	0.6092629	0.821928511
RPL21P41	-0.017	0.609168042	0.821893664
SMARCE1P7	-0.017	0.609113813	0.821844564
OR4A47	-0.017	0.609074448	0.821841987
DPH6-AS1	-0.017	0.608917524	0.82171861
AL158069	-0.017	0.608886123	0.821699518
POLR2J2	-0.017	0.60888353	0.821699518
RPL35P4	-0.017	0.60886281	0.821699518
INS	-0.017	0.608785628	0.821699518
PABPC1L2B	-0.017	0.608757157	0.821688454
RP11-973D8	-0.017	0.608696034	0.821629238
GMPS	-0.017	0.608689741	0.821629238
RP1-102H19	-0.017	0.608679549	0.821629238
RP11-1008M1	-0.017	0.608498367	0.821502119
PSG9	-0.017	0.608457292	0.821493237
HIGD1AP9	-0.017	0.608402321	0.821442306
SNRPFP1	-0.017	0.608358503	0.82140643
RP5-898J17	-0.017	0.608336639	0.821400196
IGDCC3	-0.017	0.608302106	0.821376854
AGPAT5	-0.017	0.608203551	0.821267062
RNU4ATAC9P	-0.017	0.608194453	0.821267062
CTA-398F10	-0.017	0.608168781	0.821266679
MIR1288	-0.017	0.608150599	0.821266679
MLLT3	-0.017	0.608108398	0.821266679
RP11-125H8	-0.017	0.608035965	0.821250324
AL161668	-0.017	0.608035541	0.821250324
RP5-897D18	-0.017	0.607965178	0.821201291
DUSP11	-0.017	0.6079567	0.821201291
SDCBPP3	-0.017	0.607906505	0.821191909
EGR2	-0.017	0.607832145	0.821131265
FUT2	-0.017	0.60774122	0.821084168

RP5-1000K24	-0.017	0.607726703	0.821084168
HSPA6	-0.017	0.607672075	0.821061531
CHCHD3P2	-0.017	0.60762579	0.821035701
RP11-52L11	-0.017	0.607568501	0.821035701
RP11-109M17	-0.017	0.607542342	0.821035701
SLC28A2	-0.018	0.607467713	0.820971731
KB-1471A8	-0.018	0.607462687	0.820971731
RP11-795A2	-0.018	0.607442503	0.820971731
OR7E111P	-0.018	0.607384607	0.8209293
RP11-807H22	-0.018	0.60736907	0.8209293
VN1R68P	-0.018	0.607283948	0.820863136
NUTM2G	-0.018	0.607260697	0.820863136
MAGI1-AS1	-0.018	0.607112149	0.82074555
KRTAP9-12P	-0.018	0.607080067	0.820727308
CYP4A44P	-0.018	0.606971563	0.820604316
RP11-267N12	-0.018	0.606915234	0.820574345
RPS4XP11	-0.018	0.606908266	0.820574345
AMYP1	-0.018	0.606862852	0.820554855
PRYP6	-0.018	0.606823418	0.82054338
RP11-22C8	-0.018	0.606717887	0.82050007
RPL12P34	-0.018	0.606717136	0.82050007
AC091096	-0.018	0.606573912	0.820462234
RP11-327P2	-0.018	0.6065147	0.820411173
RP11-111F16	-0.018	0.606501091	0.820411173
LINC00648	-0.018	0.606475127	0.820411173
RP11-158O16	-0.018	0.60643771	0.8204021
SAR1AP4	-0.018	0.606416041	0.8204021
RNF152P1	-0.018	0.606389815	0.820399617
RNU6-237P	-0.018	0.606273179	0.820311725
RP11-554J4	-0.018	0.606212821	0.820273476
RNA5SP514	-0.018	0.606161174	0.820230083
RPL7P3	-0.018	0.606031755	0.82010156
OR4A1P	-0.018	0.605948948	0.820036102
AC138472	-0.018	0.605938623	0.820036102
RP11-349G13	-0.018	0.605897859	0.820036102
RP11-406D1	-0.018	0.605834899	0.819998259
RP11-588G21	-0.018	0.60577264	0.8199839
TOM1L2	-0.018	0.605579897	0.819824462
GAPDHP50	-0.018	0.605561214	0.819824462
CTC-218H9	-0.018	0.605522693	0.819808667
MIR4465	-0.018	0.605234455	0.819484951
RP11-465L8	-0.018	0.605232347	0.819484951
RP11-26P13	-0.018	0.605176697	0.819479999
MGAT2P1	-0.018	0.604941308	0.819301021
RP3-384D21	-0.018	0.604915046	0.81928875
ANKEF1	-0.018	0.604826419	0.819247418
OR7E109P	-0.018	0.604747273	0.819177997
CRB1	-0.018	0.604713489	0.819177997
OR52E5	-0.018	0.604601755	0.819074064
SPCS2P3	-0.018	0.604589618	0.819074064
RNU6-625P	-0.018	0.60455341	0.819055164
C12orf4	-0.018	0.604551977	0.819055164

RN7SKP248	-0.018	0.604471118	0.818990268
RP1-269O5	-0.018	0.604427168	0.818954019
TNFRSF18	-0.018	0.604341228	0.818907468
RNU6-954P	-0.018	0.60426366	0.818872253
RNU1-51P	-0.018	0.604235423	0.818872253
RP11-421L21	-0.018	0.604198334	0.818853624
RP11-461A8	-0.018	0.604183266	0.818853624
AP000998	-0.018	0.604146104	0.818829439
RNU6-339P	-0.018	0.604110621	0.818804649
LINC00845	-0.018	0.604102094	0.818804649
RP11-360A18	-0.018	0.604010126	0.818738338
AC007383	-0.018	0.604000212	0.818738338
DYNC1I2	-0.018	0.603821547	0.818575909
OR4C10P	-0.018	0.603636685	0.81841848
CCDC58P1	-0.018	0.603617184	0.81841848
MIR6808	-0.018	0.603612067	0.81841848
CDH12P3	-0.018	0.603473273	0.818290102
ZBTB8OS	-0.018	0.603471904	0.818290102
MIR27B	-0.018	0.603463122	0.818290102
XXbac-BPG157A10	-0.018	0.603379437	0.818232751
AL591708	-0.018	0.603355743	0.818223917
RPL7L1P5	-0.018	0.603345693	0.818223917
RN7SL671P	-0.018	0.6032419	0.818162717
RP3-399J4	-0.018	0.603237929	0.818162717
CXCR1	-0.018	0.603237639	0.818162717
MARK2P4	-0.018	0.603045136	0.818058922
RP3-437I16	-0.018	0.602938596	0.817960798
OR14J1	-0.018	0.602834115	0.817918387
PQLC3	-0.018	0.602797251	0.817909024
RPL7L1P9	-0.018	0.602723885	0.817832777
RANP6	-0.018	0.602703064	0.817827825
RP1-21P18	-0.018	0.60269037	0.817827825
AC006150	-0.018	0.602660733	0.817816985
IGKV3D-31	-0.018	0.602633584	0.817803445
TEN1	-0.018	0.602586786	0.817803445
VDAC3	-0.018	0.602558995	0.817803445
PRUNEP1	-0.018	0.602540858	0.817803445
RP11-624A21	-0.018	0.602371722	0.817682105
RP11-406A9	-0.018	0.602337394	0.817682105
RP11-317J10	-0.018	0.602261408	0.817647846
RNA5SP354	-0.018	0.602195525	0.817581706
TOPAZ1	-0.018	0.602175956	0.817578444
MIOXP1	-0.018	0.602093178	0.817512666
ARHGEF18	-0.018	0.602082991	0.817512666
GRPR	-0.018	0.60203254	0.817476943
URB1	-0.018	0.601993973	0.817471187
TM4SF18	-0.018	0.601882518	0.817405197
RN7SL4P	-0.018	0.601859424	0.817405007
AP000356	-0.018	0.601835264	0.817395504
RN7SL524P	-0.018	0.601790207	0.817357617
GTF3C1	-0.018	0.601776018	0.817357617
RP4-614O4	-0.018	0.601727715	0.817342665

RPS29P12	-0.018	0.601706465	0.817342665
FREM2-AS1	-0.018	0.60168394	0.817342665
RARRES2P8	-0.018	0.60156363	0.817259635
RN7SL743P	-0.018	0.601527351	0.817233659
RP11-56H16	-0.018	0.601487461	0.817212919
RP11-341B24	-0.018	0.601372622	0.817136192
ZNF497	-0.018	0.601351621	0.817134769
EHD3	-0.018	0.601251246	0.817091625
C6orf48	-0.018	0.601228815	0.817084456
MIR32	-0.018	0.601198353	0.817066373
CTB-179I1	-0.018	0.601196875	0.817066373
ARX	-0.018	0.601128043	0.817017445
COX6CP3	-0.018	0.601122956	0.817017445
RNU6-772P	-0.018	0.601106039	0.817017445
USP9YP11	-0.018	0.601106039	0.817017445
CALM2P3	-0.018	0.601054767	0.817017445
KATNBL1P6	-0.018	0.601022916	0.817014455
RP11-771K4	-0.018	0.601006656	0.817014455
EBF3	-0.018	0.600910255	0.816931257
RP4-672N11	-0.018	0.600831069	0.816846921
CICP9	-0.018	0.600698346	0.816713106
COX5BP3	-0.018	0.600673483	0.816713106
AP1S3	-0.018	0.600659414	0.816713106
TSHB	-0.018	0.600655986	0.816713106
FTLP1	-0.018	0.600613704	0.816713106
SPDYE2B	-0.018	0.600558175	0.816685727
SNORD53_SNORD92	-0.018	0.600442611	0.816598521
RP11-320H14	-0.018	0.600438229	0.816598521
CD200R1L	-0.018	0.600435667	0.816598521
CTC-321K16	-0.018	0.600374595	0.816575972
GS1-122H1	-0.018	0.600343387	0.816556845
SEMA3B	-0.018	0.6002935	0.81651231
RSU1P1	-0.018	0.600253131	0.816504038
MAP10	-0.018	0.600222457	0.816503202
HSD3BP2	-0.018	0.600218903	0.816503202
HMGN1P37	-0.018	0.600180288	0.816498232
RP11-215H22	-0.018	0.60009791	0.816412605
ACTG1P2	-0.018	0.600068787	0.816412605
C7orf31	-0.018	0.600051512	0.816412605
RP11-143P4	-0.018	0.599928267	0.816344762
LINC01514	-0.018	0.599904231	0.816344762
FO538757	-0.018	0.599811847	0.816276791
RGAG1	-0.018	0.59968575	0.81617515
MTCO2P4	-0.018	0.599668408	0.816174871
RP11-351N6	-0.018	0.599588109	0.816112225
AC113188	-0.018	0.599538417	0.816091234
EFTUD2	-0.018	0.599497044	0.81605824
MIR4267	-0.018	0.599476634	0.816053781
RNF212B	-0.018	0.59941047	0.81601036
GNRH1	-0.018	0.599288988	0.815914946
PRTG	-0.018	0.599223693	0.815851437
RNY4P28	-0.018	0.599173574	0.815836452

MIR548H5	-0.018	0.599148817	0.815836452
RP11-305D15	-0.018	0.599142197	0.815836452
TIMM17A	-0.018	0.599126135	0.815836452
VSIG1	-0.018	0.59888474	0.815597721
AL158147	-0.018	0.598707422	0.815426189
VN1R87P	-0.018	0.598638634	0.815355817
ADH5	-0.018	0.598626313	0.815355817
DUTP3	-0.018	0.598584205	0.815328317
PRSS44	-0.018	0.598534424	0.815307145
RP11-654C20	-0.018	0.598480869	0.815280829
PARN	-0.018	0.598447228	0.81525832
HSPE1P5	-0.018	0.598419581	0.815243976
RP11-331N16	-0.018	0.598330886	0.815193098
RP11-165D6	-0.018	0.598249235	0.815138778
NSUN7	-0.018	0.598210508	0.815122361
CTD-2045M21	-0.018	0.598188798	0.815122361
ARHGEF9	-0.018	0.598096716	0.815060582
GRIK3	-0.018	0.598031841	0.815018815
AP005242	-0.018	0.597984535	0.815000986
NPM1P51	-0.018	0.597976634	0.815000986
DUS1L	-0.018	0.597880537	0.814905885
LA16c-329F2	-0.018	0.597772838	0.814805728
PAIP1	-0.018	0.597714445	0.814749453
AC012047	-0.018	0.597596049	0.814668641
PDZD8	-0.018	0.597574534	0.814668641
NFU1P2	-0.018	0.597492286	0.814609828
RP11-332J15	-0.018	0.597361543	0.814520367
GULOP	-0.018	0.597219639	0.814354665
RP11-1081L13	-0.018	0.597140108	0.81428679
RP11-272G22	-0.018	0.597139356	0.81428679
SERBP1P1	-0.018	0.597030907	0.8142417
RP11-932A10	-0.018	0.597016056	0.8142417
ADARB2-AS1	-0.018	0.596881987	0.81422061
RP11-627H22	-0.018	0.596792237	0.814144817
RP11-285F7	-0.018	0.596721363	0.814118089
VN1R85P	-0.018	0.596670464	0.814118089
OR1B1	-0.018	0.596581388	0.814043713
RPL7P34	-0.018	0.596556033	0.814032438
RP11-463M16	-0.018	0.596548494	0.814032438
RP11-350A1	-0.018	0.596480279	0.813999033
GMNC	-0.018	0.596242313	0.813744529
AC002056	-0.018	0.596088933	0.813651485
NXT2	-0.018	0.596000902	0.81355464
OR6C2	-0.018	0.595970415	0.813536341
AC005780	-0.018	0.595966535	0.813536341
RP11-162D16	-0.018	0.595965745	0.813536341
GXYLT1P2	-0.018	0.59589636	0.81352852
OSBPL9P1	-0.018	0.595886394	0.81352852
RP11-33E24	-0.018	0.595868885	0.81352852
SH3BGRL2	-0.018	0.595780524	0.813506089
CTD-2501O3	-0.018	0.595746513	0.813506089
MIR6889	-0.018	0.595670664	0.813476894

OR51A7	-0.018	0.595612864	0.813421285
CASC2	-0.018	0.595610912	0.813421285
COG5	-0.018	0.595571162	0.813421285
TSPY13P	-0.018	0.595540499	0.813421285
RP11-96J15	-0.018	0.595500624	0.813407945
RP11-35J10	-0.018	0.595470282	0.813389827
HYKK	-0.018	0.595444728	0.81337825
GPR17	-0.018	0.595414714	0.813370819
LIN28A	-0.018	0.595383615	0.813370819
LINC00433	-0.018	0.595229361	0.813224004
NEDD8-MDP1	-0.018	0.595015794	0.813048837
RNA5SP276	-0.018	0.594992394	0.813040189
RP11-95I19	-0.018	0.594955698	0.813013371
RNU6-794P	-0.018	0.59495375	0.813013371
PDE1A	-0.018	0.594944033	0.813013371
RP11-8L2	-0.018	0.594923799	0.813013371
HDHD1P2	-0.018	0.594908814	0.813013371
DNAJB1	-0.018	0.594877061	0.813013371
RP11-78I14	-0.018	0.594840382	0.813013371
DDX11-AS1	-0.018	0.594797229	0.813013371
AC134882	-0.018	0.594795655	0.813013371
LRIT2	-0.018	0.594795648	0.813013371
RP11-706D8	-0.018	0.594776108	0.813013371
HTR2B	-0.018	0.594757126	0.813013371
FOXG1	-0.018	0.594607775	0.812981125
TMEM106B	-0.018	0.594574406	0.812958839
UBE2B	-0.018	0.594547171	0.812944939
AP000807	-0.018	0.59454438	0.812944939
PCYT1A	-0.018	0.594525988	0.812944939
HNRNPA1P49	-0.018	0.594486478	0.812944939
HMGN1P35	-0.018	0.594387863	0.812890473
RP11-605F22	-0.018	0.594299071	0.812839063
MRPS18A	-0.018	0.594171228	0.81274134
AC026495	-0.018	0.59413603	0.81274134
NOSTRIN	-0.018	0.594126228	0.81274134
BNIP3P15	-0.018	0.594047898	0.812682232
GREB1L	-0.018	0.594038502	0.812682232
AC023385	-0.018	0.593889117	0.812540254
RP11-494G9	-0.018	0.593814039	0.812525677
AC068538	-0.018	0.593807652	0.812525677
PRRT3	-0.018	0.593705044	0.812446561
RAC1P9	-0.018	0.593689209	0.812446561
RP11-2A4	-0.018	0.593654232	0.812446561
MIR941-1	-0.018	0.593639427	0.812446561
RNU6-1164P	-0.018	0.593532694	0.812444146
RHOBTB3	-0.018	0.593515669	0.812444146
IGHVIII-67-4	-0.018	0.593440302	0.812411074
RNA5SP294	-0.018	0.593383	0.812385308
SLC17A6	-0.018	0.593339688	0.812385308
MIR4669	-0.018	0.593290641	0.81234629
LINC00411	-0.018	0.593051209	0.812205255
AC108059	-0.018	0.593013692	0.812195522

PTGES2-AS1	-0.018	0.593008636	0.812195522
RP11-349I1	-0.018	0.592975454	0.812195522
SERPINI2	-0.018	0.592919061	0.812164398
RNU2-20P	-0.018	0.592852439	0.812154409
CTD-2540F13	-0.018	0.592829085	0.812154409
COG1	-0.018	0.59280453	0.812147663
STH	-0.018	0.592785086	0.812144386
MNX1	-0.018	0.592748107	0.812126705
RP11-119N19	-0.018	0.592716868	0.812126705
RNU6-999P	-0.018	0.592700113	0.812126705
RP5-1044H5	-0.018	0.592625634	0.81206609
EBF2	-0.018	0.592595646	0.812048361
RNU6-448P	-0.018	0.592531419	0.812007075
RP1-97D16	-0.018	0.592459237	0.811954882
AC022073	-0.018	0.592431175	0.811939788
AC044907	-0.018	0.592392714	0.811916303
NDUFB2-AS1	-0.018	0.592316179	0.811899
RNU6-1046P	-0.018	0.592197445	0.811806342
OR51B2	-0.018	0.592167734	0.811788978
TMPRSS11CP	-0.018	0.592097233	0.811715693
HRC	-0.018	0.592072261	0.811715693
KCNJ18	-0.018	0.59203063	0.811715693
SLC25A25	-0.018	0.592003928	0.811715693
GRAMD4P5	-0.018	0.591973636	0.811715693
PGPEP1L	-0.018	0.591908862	0.811691094
RP11-228G3	-0.018	0.591878294	0.811678472
AC011073	-0.018	0.591831449	0.811655046
IL31RA	-0.018	0.591786261	0.811616443
AC144833	-0.018	0.591754283	0.811616443
RNA5SP230	-0.018	0.591748141	0.811616443
RP11-747I9	-0.018	0.591713368	0.811616443
AL139296	-0.018	0.591706529	0.811616443
RNU4-57P	-0.018	0.591689857	0.811616443
RP11-507D14	-0.018	0.591638337	0.811616443
AC103816	-0.018	0.591561363	0.811616443
ZFR2	-0.018	0.591514736	0.811616443
ATP2B1	-0.018	0.591502293	0.811616443
GPR45	-0.018	0.591486122	0.811616443
AC008753	-0.018	0.591267955	0.811396269
FDPSP5	-0.018	0.591201538	0.811395463
AP000857	-0.018	0.591163316	0.811369565
AGAP7P	-0.018	0.591150353	0.811369565
SLC28A3	-0.018	0.591109872	0.81134609
LINC00575	-0.018	0.591069871	0.811334835
RP11-96D24	-0.018	0.590936488	0.811219178
MIR6508	-0.018	0.59089276	0.811208621
MIR944	-0.018	0.590879792	0.811208621
OR4D8P	-0.018	0.590873657	0.811208621
RP11-117O7	-0.018	0.590863014	0.811208621
NEUROD4	-0.018	0.59085126	0.811208621
LINC01349	-0.018	0.590813291	0.811208621
AL158801	-0.018	0.590754545	0.811208621

RP11-636N17	-0.018	0.59073569	0.811208621
TACO1	-0.018	0.590630842	0.811153007
PRR34-AS1	-0.018	0.590502626	0.811070471
RPL29P2	-0.018	0.590365525	0.81095232
RP11-13J10	-0.018	0.590362652	0.81095232
OSBPL9P4	-0.018	0.590337513	0.81095232
SERBP1P4	-0.018	0.590290312	0.810942561
CTD-3057O21	-0.018	0.590225266	0.81087659
AP001171	-0.018	0.590071758	0.810712466
RNA5SP232	-0.018	0.589998543	0.810635258
RP11-21G20	-0.018	0.589977748	0.810630072
WDR27	-0.018	0.589956722	0.810630072
HNRNPCP6	-0.018	0.589741342	0.810445535
CFL1P8	-0.018	0.5896463	0.810361689
HMGCLL1	-0.018	0.589577854	0.810319458
RP11-328P23	-0.018	0.589498741	0.810275801
LINC00637	-0.018	0.589495838	0.810275801
RP5-902P8	-0.018	0.589477738	0.810275801
RP11-185H22	-0.018	0.589420866	0.810262298
CDRT4	-0.018	0.589308573	0.810154699
AC009501	-0.018	0.589308189	0.810154699
GOLGA6L4	-0.018	0.589109764	0.809951524
RNU6-677P	-0.018	0.58899617	0.80988834
KLHL30-AS1	-0.018	0.588986964	0.80988834
AL109763	-0.018	0.588866692	0.809804347
GLRB	-0.018	0.58885657	0.809804347
NETO2	-0.018	0.588849038	0.809804347
CLIC6	-0.018	0.588785394	0.809762691
TGFBR2	-0.018	0.588653221	0.809651052
RNU6-1020P	-0.018	0.588628091	0.809639868
AC018755	-0.018	0.588497337	0.809600308
USP9YP10	-0.018	0.588485619	0.809600308
HNRNPA1P28	-0.018	0.588438704	0.809589801
PPP1R26P3	-0.018	0.588399773	0.80956955
SKP2	-0.018	0.588324166	0.809519384
FAR2	-0.018	0.588235328	0.809449421
RP1-56J10	-0.018	0.588224756	0.809449421
RP11-662M24	-0.018	0.58805586	0.809320246
TRIM72	-0.018	0.587955674	0.809236314
OR5AL1	-0.018	0.58794163	0.809236314
RFPL3-AS1_1	-0.018	0.587833359	0.809201092
INPPL1	-0.018	0.587659161	0.80900806
AUNIP	-0.018	0.587619655	0.80900806
RP11-47A8	-0.018	0.587235951	0.808659187
RP11-715I4	-0.019	0.587077318	0.808487492
RP11-205A8	-0.019	0.587071694	0.808487492
RP11-687M24	-0.019	0.58706157	0.808487492
MT-TS2	-0.019	0.586874983	0.80834909
TSPAN14	-0.019	0.586820779	0.808321183
SURF1	-0.019	0.586812883	0.808321183
RRN3P3	-0.019	0.586673019	0.80821115
RN7SL717P	-0.019	0.586632886	0.808179239

RN7SL454P	-0.019	0.586603401	0.808162435
RCE1	-0.019	0.586447465	0.808017283
RITA1	-0.019	0.586424536	0.808009066
AC108051	-0.019	0.586408955	0.808009066
AC009245	-0.019	0.586407998	0.808009066
HMGN1P28	-0.019	0.586318689	0.807956722
FTCD-AS1	-0.019	0.586307481	0.807956722
RP11-320N7	-0.019	0.586305204	0.807956722
RP11-593F5	-0.019	0.586287893	0.807956722
OR8C1P	-0.019	0.58622117	0.807956722
UBE2Q2P1	-0.019	0.586130254	0.807907435
PYURF	-0.019	0.58608328	0.807866068
COPS8P2	-0.019	0.585978759	0.807792131
PTP4A1P5	-0.019	0.585964553	0.807792131
EPN2	-0.019	0.585919826	0.80775765
RP3-410C9	-0.019	0.585852756	0.807734905
RP11-446P9	-0.019	0.585790089	0.807695691
MIR3914-1	-0.019	0.585748131	0.807695691
RP5-896L10	-0.019	0.585743838	0.807695691
ZNRF4	-0.019	0.585707845	0.807695691
ERV3-1	-0.019	0.585615519	0.807695691
RP11-43N22	-0.019	0.585597711	0.807695691
RP11-253E3	-0.019	0.585531753	0.807666844
CATSPER3	-0.019	0.585491938	0.807635316
ARID1B	-0.019	0.585486304	0.807635316
RP11-126D17	-0.019	0.585466548	0.807635316
CH507-42P11	-0.019	0.585461104	0.807635316
CTC-498M16	-0.019	0.585445667	0.807635316
TERF1P3	-0.019	0.585355662	0.807570479
RP11-788A1	-0.019	0.585306534	0.807562234
AIFM3	-0.019	0.585198624	0.807443777
BX322557	-0.019	0.585167081	0.807443777
RP11-286E11	-0.019	0.585089426	0.807360686
RNU6-1084P	-0.019	0.585071185	0.807358908
AL080285	-0.019	0.585040514	0.807344358
AC104592	-0.019	0.584964828	0.807329099
VN1R110P	-0.019	0.584945104	0.807325271
RPS26P49	-0.019	0.584829562	0.807212587
FUZ	-0.019	0.584817905	0.807212587
GDPD3	-0.019	0.584788999	0.807203387
RP11-419N10	-0.019	0.584732684	0.807149046
OR4K16P	-0.019	0.584709643	0.807140634
RP1-203P18	-0.019	0.584617648	0.807060427
COX6A2	-0.019	0.58461589	0.807060427
PGR	-0.019	0.584567159	0.807037512
TSPY4	-0.019	0.584480425	0.806987946
CRIP3	-0.019	0.584366624	0.806924392
PARD3B	-0.019	0.584302579	0.806882743
CTC-458A3	-0.019	0.584279759	0.806874625
C1GALT1	-0.019	0.584062816	0.806665778
RPL18P11	-0.019	0.584050023	0.806665778
OR8D1	-0.019	0.583928961	0.806553889

RP11-66D17	-0.019	0.583787457	0.806428598
CABP4	-0.019	0.583736077	0.80642278
RPL31P9	-0.019	0.583672476	0.806403827
AC027288	-0.019	0.583542452	0.806270011
SEMA3C	-0.019	0.583521923	0.806270011
RNU6-604P	-0.019	0.583521846	0.806270011
CTD-2410N18	-0.019	0.583517797	0.806270011
ACTR3P2	-0.019	0.583504162	0.806270011
HNRNPCL4	-0.019	0.583345802	0.806216028
RP11-219G17	-0.019	0.58334314	0.806216028
CTC-203F4	-0.019	0.583319054	0.806216028
RNU6-563P	-0.019	0.583212299	0.80619528
RP11-107N7	-0.019	0.583157012	0.806142252
AC005786	-0.019	0.583094468	0.806135916
RP11-867G23	-0.019	0.583057736	0.806135916
ARV1	-0.019	0.583015375	0.806133641
RNA5SP234	-0.019	0.582998201	0.806133299
CTD-3051D23	-0.019	0.582963609	0.80610887
HMBOX1	-0.019	0.582959521	0.80610887
PSG1	-0.019	0.582927808	0.806106173
RN7SL211P	-0.019	0.582888375	0.806098454
GOLGA8G	-0.019	0.582868611	0.806098454
RP5-998G20	-0.019	0.582801062	0.806072675
IVNS1ABP	-0.019	0.582778106	0.806072675
SNHG17	-0.019	0.582776982	0.806072675
AC018643	-0.019	0.582704616	0.806054974
RPS17P8	-0.019	0.58266898	0.806046266
RP3-453P22	-0.019	0.582651213	0.806046266
FMO1	-0.019	0.582651057	0.806046266
CCT6A	-0.019	0.582458446	0.805913109
RN7SL66P	-0.019	0.582377917	0.805860515
RP11-446E24	-0.019	0.582346212	0.805840056
AC087237	-0.019	0.582301351	0.805824803
RP11-976B16	-0.019	0.582246759	0.805796082
SLC6A19	-0.019	0.582069716	0.805597884
SLC25A4	-0.019	0.582027241	0.805585917
KRTAP10-5	-0.019	0.581890715	0.805467175
CDY12P	-0.019	0.581730383	0.805315456
AC079603	-0.019	0.581730383	0.805315456
USP9YP8	-0.019	0.581730383	0.805315456
RP11-729M20	-0.019	0.581724508	0.805315456
CSAG4	-0.019	0.5816906	0.805315456
RNU6-150P	-0.019	0.58167034	0.805315456
RPS7P4	-0.019	0.581640532	0.805315456
MIR185	-0.019	0.581527121	0.805255914
ATXN8OS	-0.019	0.581491068	0.805241622
UBXN11	-0.019	0.581395125	0.805156301
ATP5L2	-0.019	0.581345797	0.805134092
RNU6-1087P	-0.019	0.581325046	0.805134092
RP11-122L9	-0.019	0.581312261	0.805134092
RNU6-153P	-0.019	0.581310401	0.805134092
AC009517	-0.019	0.581304872	0.805134092

FIGLA	-0.019	0.581250168	0.805134092
RNY3P1	-0.019	0.581195513	0.805134092
AL365181	-0.019	0.581151053	0.805118201
MIR5571	-0.019	0.581124499	0.805108547
RP4-740C4	-0.019	0.580941458	0.804972036
RP11-6D1	-0.019	0.580672456	0.804692944
U2AF2	-0.019	0.580624513	0.804663656
MIR466	-0.019	0.580436932	0.804452801
FAM230C	-0.019	0.580424495	0.804452801
RNU6-764P	-0.019	0.58040954	0.804452801
GPANK1	-0.019	0.580330093	0.804429155
RP11-11A9	-0.019	0.580256618	0.804374352
RNU6-1277P	-0.019	0.580223032	0.804374352
CBX3P9	-0.019	0.580189774	0.804374352
OR8J1	-0.019	0.580152547	0.80436115
AP002856	-0.019	0.57999599	0.804223507
LONRF1	-0.019	0.579871206	0.804144127
AC011155	-0.019	0.57977082	0.804028327
RP11-174F8	-0.019	0.579763114	0.804028327
FAM178B	-0.019	0.579735853	0.804026659
CAMK1D	-0.019	0.579518274	0.803841939
RAN	-0.019	0.579477528	0.803808832
RP11-137O10	-0.019	0.579399475	0.80372397
CTB-129O4	-0.019	0.579385576	0.80372397
HMX2	-0.019	0.579205015	0.803571244
AP000889	-0.019	0.579158978	0.803530781
RP1-241P17	-0.019	0.579050588	0.803450614
RPL7P8	-0.019	0.578989001	0.803388567
AC190387	-0.019	0.578980769	0.803388567
RNU4-26P	-0.019	0.57894113	0.803373753
RNU6-606P	-0.019	0.57891017	0.803372804
SCOC-AS1	-0.019	0.578848951	0.803311256
CTD-2354A18	-0.019	0.578812403	0.803283943
PKDREJ	-0.019	0.578805401	0.803283943
ACTR3BP2	-0.019	0.578801969	0.803283943
MIR3118-1	-0.019	0.578705013	0.803228527
RN7SL217P	-0.019	0.57864465	0.803168151
SETMAR	-0.019	0.578644602	0.803168151
FKSG51	-0.019	0.578587591	0.803135764
RP11-3J10	-0.019	0.578586611	0.803135764
GABARAPL3	-0.019	0.578471051	0.803081053
RP11-404E12	-0.019	0.578449493	0.803081053
AC092116	-0.019	0.578388349	0.803046436
CHRM1	-0.019	0.578386797	0.803046436
KRT19P2	-0.019	0.578380755	0.803046436
HLX-AS1	-0.019	0.578338568	0.803046436
RP11-48B3	-0.019	0.578313307	0.803046436
TRPC7-AS2	-0.019	0.57825439	0.803024313
MIR4472-2	-0.019	0.578253176	0.803024313
RNA5SP54	-0.019	0.578191254	0.802984631
LINC01493	-0.019	0.578171959	0.802984631
PTRH2	-0.019	0.577771176	0.802634109

AC006547	-0.019	0.577743926	0.802619665
RP11-27G13	-0.019	0.577671548	0.802597249
RP11-227D13	-0.019	0.577578103	0.802553165
RHO	-0.019	0.577557523	0.802547984
PRPF38AP1	-0.019	0.577511719	0.80250775
RNU6-154P	-0.019	0.577477839	0.802484084
SETP22	-0.019	0.57747493	0.802484084
SPATA6L	-0.019	0.577347569	0.802373809
EIF4BP8	-0.019	0.577244137	0.802323192
RN7SL470P	-0.019	0.577230786	0.802323192
FUNDC1	-0.019	0.577193091	0.802323192
AC093004	-0.019	0.577181972	0.802323192
DHH	-0.019	0.577148166	0.802323192
KB-1125A3	-0.019	0.577147946	0.802323192
ATP5LP4	-0.019	0.577140934	0.802323192
LINC00472	-0.019	0.577129415	0.802323192
GRIA2	-0.019	0.576995996	0.802306093
RP5-1086L22	-0.019	0.576818555	0.802106199
MROH2B	-0.019	0.576706292	0.80202034
TLR5	-0.019	0.576695835	0.80202034
GCSHP5	-0.019	0.576653427	0.802008932
RP4-583K8	-0.019	0.57660594	0.801997872
RP11-159L20	-0.019	0.576548477	0.801941366
AL354828	-0.019	0.576363485	0.801754299
RNU6-1088P	-0.019	0.576357073	0.801754299
AL162614	-0.019	0.576310183	0.801726985
SLC25A1P1	-0.019	0.576306501	0.801726985
FAM200A	-0.019	0.576255822	0.801726985
AC007422	-0.019	0.576221863	0.801726985
ARL8A	-0.019	0.576143607	0.801723709
CCDC13-AS1	-0.019	0.576128633	0.801723709
RYR2	-0.019	0.576127577	0.801723709
RN7SL317P	-0.019	0.576123589	0.801723709
RNA5SP362	-0.019	0.576035866	0.801723709
DNM1P51	-0.019	0.575989147	0.801723709
IGBP1	-0.019	0.575955362	0.801723709
LINC00401	-0.019	0.57581128	0.801645683
CYP2A7	-0.019	0.575778606	0.801643115
HSPBAP1	-0.019	0.575770127	0.801643115
CTD-2006H14	-0.019	0.575650131	0.801511103
LRRC37A6P	-0.019	0.57562591	0.80150081
RP11-432J3	-0.019	0.575599928	0.801488065
MIR202HG	-0.019	0.575518498	0.801398108
KRT18P58	-0.019	0.575435544	0.801329454
FAM162B	-0.019	0.575425618	0.801329454
RP11-325O24	-0.019	0.575397533	0.801329454
AL354775	-0.019	0.57531089	0.801283435
KIAA0895L	-0.019	0.57530586	0.801283435
BCAP31P2	-0.019	0.575303672	0.801283435
SF3A3	-0.019	0.575255941	0.801283435
KRTAP9-7	-0.019	0.575027036	0.800994784
GRIA4	-0.019	0.575011369	0.800994784

FMO6P	-0.019	0.57500905	0.800994784
SLMO2P1	-0.019	0.574986319	0.800994784
GOT2P1	-0.019	0.574961677	0.800994784
RP11-93P10	-0.019	0.574956123	0.800994784
STK32B	-0.019	0.574904705	0.800994784
CYP3A43	-0.019	0.574860505	0.800973648
RP11-375H19	-0.019	0.574692379	0.800809676
PCDH11X	-0.019	0.5746699	0.800801782
CD300C	-0.019	0.574655214	0.800801782
RP11-438D14	-0.019	0.57464407	0.800801782
MIR4305	-0.019	0.574635198	0.800801782
MTMR9	-0.019	0.574634735	0.800801782
CTB-85P21	-0.019	0.574616314	0.800801782
RP11-221H10	-0.019	0.57451879	0.800801782
MYC	-0.019	0.574479856	0.800794688
CMA1	-0.019	0.574477393	0.800794688
AC005013	-0.019	0.574417863	0.800778585
RP11-142L4	-0.019	0.574398713	0.800775327
NDUFA10	-0.019	0.57434063	0.800759705
RP11-81M19	-0.019	0.57426032	0.80069958
MIR5186	-0.019	0.574146999	0.800611891
RP11-439E19	-0.019	0.574041758	0.800532253
RNU1-57P	-0.019	0.573939767	0.800463535
MIR3616	-0.019	0.573897963	0.800463535
P2RY6	-0.019	0.573846767	0.800463535
MRPL37P1	-0.019	0.573677122	0.80026127
RNF180	-0.019	0.573555085	0.800137903
CTCFL	-0.019	0.573520266	0.800114154
RP11-628J14	-0.019	0.573506266	0.800114154
GLS	-0.019	0.573445722	0.800079078
RNF138P2	-0.019	0.573445254	0.800079078
HSD17B3	-0.019	0.573358967	0.800079078
CA6	-0.019	0.573326043	0.800076165
CLDN15	-0.019	0.573302152	0.800066267
OCEL1	-0.019	0.573157854	0.799935213
AC073928	-0.019	0.5730722	0.799872545
AC004070	-0.019	0.573030165	0.799850756
LAMTOR3P2	-0.019	0.573027459	0.799850756
RP11-303E16	-0.019	0.572987918	0.79983867
TSFM	-0.019	0.572818378	0.79967232
RP11-213O5	-0.019	0.572769083	0.799626942
UBE2E1	-0.019	0.572664845	0.799504854
RNU1-44P	-0.019	0.572541252	0.799379169
RP1-278E11	-0.019	0.572513562	0.799363942
KRTAP19-2	-0.019	0.57236953	0.799256564
RP5-955M13	-0.019	0.572350711	0.799253718
ARHGEF15	-0.019	0.572011899	0.799014862
ANGPTL7	-0.019	0.571995264	0.799014862
PLCH1-AS1	-0.019	0.571983868	0.799014862
RNU6-896P	-0.019	0.571901668	0.798954616
HSP90AA5P	-0.019	0.571774108	0.798823275
KHK	-0.019	0.571769318	0.798823275

RP11-360L9	-0.019	0.571736625	0.798823275
MIR543	-0.019	0.571708042	0.798823275
SNRPEP7	-0.019	0.571697665	0.798823275
AC009005	-0.019	0.571696516	0.798823275
MTCO1P4	-0.019	0.571682155	0.798823275
FMO2	-0.019	0.57151713	0.798698543
PSMB3P2	-0.019	0.57142938	0.798622779
RP11-122L4	-0.019	0.571383719	0.798605833
ARF1P2	-0.019	0.571305614	0.798548171
ZNF646	-0.019	0.571253113	0.798517023
AC079154	-0.019	0.571203455	0.798471044
CTC-276P9	-0.019	0.571143842	0.79842781
IDI2	-0.019	0.571090955	0.79842781
RNU6ATAC29P	-0.019	0.571068768	0.798423373
AC025280	-0.019	0.570998538	0.798372056
AL357060	-0.019	0.570997198	0.798372056
CCDC73	-0.019	0.570879645	0.798286849
EGOT	-0.019	0.570779416	0.798229717
PATE4	-0.019	0.57074382	0.79822082
ICT1	-0.019	0.57072414	0.79822082
RP11-552E20	-0.019	0.570529965	0.798115217
RP4-718J7	-0.019	0.570514178	0.798115217
RP11-572H4	-0.019	0.570456426	0.798059223
OR5AZ1P	-0.019	0.570383167	0.798013852
DACH2	-0.019	0.570264082	0.797977654
RPS20P5	-0.019	0.570246597	0.797976632
LINC00879	-0.019	0.570138502	0.797852643
LINC00202-2	-0.019	0.57005501	0.797778854
GUCY2D	-0.019	0.569979827	0.797697076
RP4-595K12	-0.019	0.569957922	0.79768986
STARD7-AS1	-0.019	0.569946138	0.79768986
MIR3158-1	-0.019	0.569931589	0.79768986
RBMS2P1	-0.019	0.569847747	0.797652862
RPLP1P13	-0.019	0.569825684	0.797652862
RP11-289E15	-0.019	0.569812719	0.797652862
TAAR9	-0.019	0.569811115	0.797652862
HIST1H1C	-0.019	0.569600893	0.797484373
RNU6-328P	-0.019	0.569585142	0.797484373
RP11-514A9	-0.019	0.569541248	0.797481655
RPL34P21	-0.019	0.569533723	0.797481655
RNU6-628P	-0.019	0.569492969	0.797481655
EIF1P4	-0.019	0.569479211	0.797481655
HMGB1P10	-0.019	0.5694778	0.797481655
RP11-164C1	-0.019	0.569467686	0.797481655
RP11-128L5	-0.019	0.569421432	0.797481655
RNU6-1197P	-0.019	0.56936532	0.797481655
RP5-877J2	-0.019	0.569351696	0.797481655
AC123768	-0.019	0.569335665	0.797481655
RP11-793L10	-0.019	0.5693217	0.797481655
RN7SL761P	-0.019	0.569321111	0.797481655
RN7SL379P	-0.019	0.569321111	0.797481655
LINC00958	-0.019	0.569264473	0.797481655

CNOT6LP1	-0.019	0.569197101	0.797481655
FAM187B	-0.019	0.56916436	0.797481655
RNY1	-0.019	0.569073203	0.797388922
RP11-790I12	-0.019	0.569072149	0.797388922
RP11-18O11	-0.019	0.569007646	0.797388922
EFCAB11	-0.019	0.568933676	0.797322504
DLGAP3	-0.019	0.568874198	0.797274268
RCBTB2	-0.019	0.568653556	0.797059067
AP3S2	-0.019	0.568613914	0.797059067
ABCB10P1	-0.019	0.568584516	0.797059067
RP11-56O18	-0.019	0.568485811	0.796964464
RP4-676L2	-0.019	0.56843266	0.796913409
CTA-440B3	-0.019	0.568305787	0.79682936
GOLGA2	-0.019	0.568294609	0.79682936
RNA5SP487	-0.019	0.568270092	0.796826229
AC073333	-0.019	0.568202089	0.796754332
AC119674	-0.019	0.568178275	0.796744398
RP11-138E2	-0.019	0.568173394	0.796744398
RN7SL152P	-0.019	0.568113	0.796724243
ADAMTSL3	-0.019	0.568083885	0.796724243
NPM1P35	-0.019	0.568027204	0.796673292
RP1-137K24	-0.019	0.567992092	0.796647506
USH2A	-0.019	0.567975927	0.796647506
RP11-72M10	-0.019	0.567953951	0.796640931
RN7SL26P	-0.019	0.56794428	0.796640931
RP11-65D24	-0.019	0.56792935	0.796640931
LINC00235	-0.019	0.567810259	0.796533215
Z83826	-0.019	0.567704372	0.796455053
CU459202	-0.019	0.567667665	0.796427015
PADI4	-0.019	0.567639196	0.796410533
AL139239	-0.019	0.567629384	0.796410533
CNTN2	-0.019	0.567624864	0.796410533
OR5M3	-0.019	0.5676047	0.796410533
MIR5584	-0.019	0.56754936	0.796410533
CDC42	-0.019	0.567480806	0.796352525
RP11-80I3	-0.019	0.567434788	0.796334875
NDUFA3P6	-0.019	0.567430388	0.796334875
GYG2-AS1	-0.019	0.567404764	0.796334875
FKBP4P7	-0.019	0.567319212	0.796266528
FAM19A2	-0.019	0.567262085	0.796209811
YBX1P8	-0.019	0.567152077	0.796102327
RP11-181B11	-0.019	0.567148103	0.796102327
RP1-218B13	-0.019	0.567146615	0.796102327
RPL7P43	-0.019	0.567074893	0.796086451
RNU4-68P	-0.019	0.567051288	0.796078164
TUBA3C	-0.020	0.566992188	0.796034203
AL121967	-0.020	0.566881906	0.795981995
HMGA1P5	-0.020	0.566833025	0.795981995
RNA5SP405	-0.020	0.566833022	0.795981995
LINC00603	-0.020	0.566767945	0.79596193
MIR548V	-0.020	0.566735584	0.795939953
PTBP1	-0.020	0.566730069	0.795939953

XXbac-BPG308J9	-0.020	0.566622701	0.79585182
RP11-537E18	-0.020	0.56648012	0.795730262
MIR3135A	-0.020	0.566463306	0.795730262
CDCA2	-0.020	0.56633029	0.795589824
RNY3P9	-0.020	0.56631234	0.795589824
ISX-AS1	-0.020	0.566235236	0.795565685
PAFAH1B1	-0.020	0.566184243	0.795565685
RP5-859M6	-0.020	0.566175891	0.795565685
OIT3	-0.020	0.56607777	0.795485245
MMS19	-0.020	0.566006423	0.795408454
RP11-963H1	-0.020	0.565965566	0.795374506
AC012306	-0.020	0.565802383	0.795192106
MIR4797	-0.020	0.565782321	0.795187376
RP11-129O7	-0.020	0.565775996	0.795187376
MRPL49P1	-0.020	0.565762451	0.795187376
CEP57L1	-0.020	0.565726624	0.795187376
NUTM2B-AS1	-0.020	0.565716645	0.795187376
CACNA1C-AS4	-0.020	0.565671571	0.795187376
MIR532	-0.020	0.565609876	0.795156194
AC005160	-0.020	0.565593064	0.795156031
ZNF512B	-0.020	0.565576957	0.795156031
DPP3P2	-0.020	0.56554077	0.795152927
RP11-367N14	-0.020	0.56548347	0.795137067
RNU6-738P	-0.020	0.565422618	0.795080692
RGS1	-0.020	0.565406618	0.795080692
AP000235	-0.020	0.565368131	0.795074496
CDY3P	-0.020	0.565358303	0.795074496
RP11-159D8	-0.020	0.56530619	0.795074496
RP5-1077B9	-0.020	0.56513507	0.794887569
PDGFD	-0.020	0.565105536	0.794887569
SHC1P2	-0.020	0.565062253	0.794855571
SUMO2P12	-0.020	0.565037453	0.794850418
RNU7-70P	-0.020	0.564936345	0.794748879
RP11-526P5	-0.020	0.564858436	0.794686224
RNU6-242P	-0.020	0.564807657	0.794640095
RP11-641C17	-0.020	0.564799985	0.794640095
UPK3A	-0.020	0.5647917	0.794640095
CGB1	-0.020	0.564782212	0.794640095
RP11-485C11	-0.020	0.564720914	0.794640095
RPL21P109	-0.020	0.564670697	0.794609877
RP11-386B1	-0.020	0.56466763	0.794609877
LINC01262	-0.020	0.564638674	0.794609877
C3orf49	-0.020	0.564524579	0.79447468
RPL10L	-0.020	0.564452708	0.794397007
FAM72C	-0.020	0.56444459	0.794397007
LARP7P1	-0.020	0.564424918	0.794397007
RPS19P1	-0.020	0.564413245	0.794397007
CTD-2301A4	-0.020	0.564368641	0.794378798
ADRA2B	-0.020	0.564366644	0.794378798
ZNF747	-0.020	0.564175442	0.794235824
RN7SKP146	-0.020	0.564102743	0.794235824
C10orf131	-0.020	0.564091882	0.794235824

RNU4-50P	-0.020	0.564091411	0.794235824
MED20	-0.020	0.564064722	0.794235824
AC009403	-0.020	0.563911945	0.794128746
MIR1299	-0.020	0.563908503	0.794128746
CHST10	-0.020	0.563881318	0.794128746
ARL2BPP1	-0.020	0.563851964	0.794128746
AC017078	-0.020	0.56379912	0.79408726
KB-1568E2	-0.020	0.56373424	0.794019361
KDR	-0.020	0.563669904	0.794019361
RP11-439A17	-0.020	0.563667511	0.794019361
AC098823	-0.020	0.563635774	0.794019361
ZYG11AP1	-0.020	0.563592275	0.794007255
RP11-44B19	-0.020	0.563567635	0.793996029
RP11-664H7	-0.020	0.563525439	0.793960067
MFN1	-0.020	0.563507587	0.793958402
RP11-484P15	-0.020	0.563475137	0.793936169
FAM47DP	-0.020	0.563424192	0.79391136
RP1-256G22	-0.020	0.563379025	0.793871204
RP11-399L7	-0.020	0.56335333	0.793858484
AC009362	-0.020	0.563320148	0.793835213
RUSC1	-0.020	0.563286652	0.793811498
GDI2P2	-0.020	0.56328182	0.793811498
RP11-6L6	-0.020	0.563117459	0.793690486
MRGPRX4	-0.020	0.563115562	0.793690486
TMTC3	-0.020	0.56309747	0.793690486
ARMCX3	-0.020	0.562868501	0.793424178
AF277315	-0.020	0.562848968	0.793424178
BIRC8	-0.020	0.562839877	0.793424178
KRT18P44	-0.020	0.562827996	0.793424178
RPL36AP21	-0.020	0.562797078	0.793424178
PSMC1P6	-0.020	0.562789168	0.793424178
AC009260	-0.020	0.562739766	0.793424178
RPL5P28	-0.020	0.562713132	0.793424178
SNRPCP11	-0.020	0.562672533	0.793392093
AL033378	-0.020	0.562654693	0.793390428
L3MBTL4	-0.020	0.562552222	0.793307036
CTD-3025N20	-0.020	0.562543745	0.793307036
KLF17P2	-0.020	0.562538977	0.793307036
LINC01194	-0.020	0.562515995	0.793307036
RBM8A	-0.020	0.562449026	0.793307036
GTF3AP1	-0.020	0.562381107	0.7932395
MIR3679	-0.020	0.56234957	0.7932395
ANKRD20A17P	-0.020	0.562323398	0.7932395
MIR194-1	-0.020	0.562235047	0.79319792
BBS5	-0.020	0.562234186	0.79319792
AC005284	-0.020	0.562209908	0.79319792
RNA5SP184	-0.020	0.562178551	0.793188703
TPCN1	-0.020	0.5621756	0.793188703
MIR6510	-0.020	0.562087768	0.793152097
CYFIP1	-0.020	0.562087543	0.793152097
AL354764	-0.020	0.562067068	0.793152097
RNA5SP327	-0.020	0.562031709	0.793145995

RP11-245D16	-0.020	0.561976097	0.793116858
MAP3K15	-0.020	0.561958836	0.793116858
HIST1H2BPS1	-0.020	0.561835456	0.792990449
SLC36A4	-0.020	0.561814202	0.792990449
ATP5F1P6	-0.020	0.561720759	0.792942132
RP3-437C15	-0.020	0.561718592	0.792942132
OR4Q2	-0.020	0.5617164	0.792942132
RP11-51P8	-0.020	0.561686952	0.792942132
RTFDC1	-0.020	0.561665033	0.792942132
CTC-447K7	-0.020	0.561607205	0.792942132
LL22NC03-121E8	-0.020	0.561582944	0.792942132
NEURL2	-0.020	0.561553446	0.792942132
SNORD90	-0.020	0.561523261	0.792942132
RPS27P27	-0.020	0.561514961	0.792942132
RPL29P7	-0.020	0.561466342	0.79293552
ARL6IP1P2	-0.020	0.561327152	0.792808094
RP11-12N13	-0.020	0.561297487	0.792808094
CTD-2376I20	-0.020	0.561253279	0.792775666
RNU6-767P	-0.020	0.561248232	0.792775666
RP11-307E17	-0.020	0.561198223	0.792744922
ANKRD26P4	-0.020	0.561153693	0.79270553
ZNF280C	-0.020	0.561126713	0.792690928
TPT1P12	-0.020	0.561113821	0.792690928
CCDC138	-0.020	0.561082539	0.792690928
ACSM2A	-0.020	0.561054534	0.79268301
MCRS1	-0.020	0.561033213	0.792676402
C5orf17	-0.020	0.561019028	0.792676402
MIR6131	-0.020	0.560915137	0.792650661
RP11-327J17	-0.020	0.560873227	0.792614954
RN7SKP208	-0.020	0.560846962	0.792614954
RNA5SP111	-0.020	0.5608123	0.792613298
Clostridiales-1	-0.020	0.560808196	0.792613298
TRI-TAT2-3	-0.020	0.560747367	0.792578194
GAGE1	-0.020	0.560726718	0.792572529
POLR2C	-0.020	0.560669226	0.792514787
UQCRFS1P2	-0.020	0.56066438	0.792514787
AC000032	-0.020	0.560631793	0.792508916
RPSAP2	-0.020	0.56058517	0.792466531
AC010880	-0.020	0.560438096	0.792365858
RP11-700F16	-0.020	0.56040001	0.792345889
ZSCAN25	-0.020	0.560385917	0.792345889
OR5AK2	-0.020	0.560330716	0.792318478
ZCCHC9	-0.020	0.560199781	0.792227418
RP11-33O4	-0.020	0.560188746	0.792227418
RN7SL698P	-0.020	0.560136785	0.792208898
ZSCAN21	-0.020	0.560108931	0.792193029
GORAB	-0.020	0.560086863	0.792185342
RNU6-57P	-0.020	0.560083105	0.792185342
AC005593	-0.020	0.560053075	0.792185342
RNU7-129P	-0.020	0.560025277	0.792185342
DOCK3	-0.020	0.559887904	0.792068592
HNRNPA1P30	-0.020	0.559878399	0.792068592

NCAM2	-0.020	0.559873563	0.792068592
RP11-182I10	-0.020	0.559856162	0.792068592
RP11-574O16	-0.020	0.559819319	0.792068592
HSPA5	-0.020	0.559805903	0.792068592
RP11-612J15	-0.020	0.559755747	0.792068592
AASS	-0.020	0.559746704	0.792068592
OR5BS1P	-0.020	0.559682266	0.792068592
MOB1AP2	-0.020	0.559663759	0.792068592
FOXJ2	-0.020	0.55954229	0.791979572
RP11-1267H10	-0.020	0.559466339	0.791919141
SCARNA1	-0.020	0.559465725	0.791919141
CTD-2357A8	-0.020	0.55941968	0.791900166
KRT8P47	-0.020	0.559383651	0.791896238
AC142119	-0.020	0.559367715	0.791896238
RP3-414A15	-0.020	0.559331663	0.791869715
RP11-446J8	-0.020	0.559270171	0.791806196
CHEK2P4	-0.020	0.559130255	0.791655171
HOMER2	-0.020	0.559068525	0.791607432
CHIAP2	-0.020	0.559011293	0.791580866
TRIAP1P1	-0.020	0.559006629	0.791580866
C2CD2	-0.020	0.558950206	0.791541432
RNU1-24P	-0.020	0.558908989	0.791506598
RPS15AP6	-0.020	0.55889482	0.791506598
LINC01347	-0.020	0.55885614	0.791478824
RNU7-134P	-0.020	0.558825376	0.79145879
RP3-395P12	-0.020	0.558808935	0.79145879
NACAP3	-0.020	0.558801455	0.79145879
HMGB3P21	-0.020	0.558800708	0.79145879
KLHL10	-0.020	0.558676253	0.791365253
RP11-486O13	-0.020	0.558663716	0.791365253
UHRF2	-0.020	0.558663278	0.791365253
TAS2R16	-0.020	0.558632413	0.791365253
RPL4P6	-0.020	0.558600097	0.791351522
FTHL17	-0.020	0.558556639	0.791313494
SARS	-0.020	0.558531777	0.791301811
MIR598	-0.020	0.558359532	0.791128383
RP1-228H13	-0.020	0.558356222	0.791128383
RP11-324F11	-0.020	0.558338124	0.791128383
GATA2-AS1	-0.020	0.558319132	0.791128383
TPPP	-0.020	0.558299301	0.791128383
CENPL	-0.020	0.558295629	0.791128383
TPM3P6	-0.020	0.558289046	0.791128383
FTSJ2	-0.020	0.558280573	0.791128383
RP11-1084I9	-0.020	0.558185635	0.791117344
SNORA71D	-0.020	0.558111946	0.791036445
RPL12P33	-0.020	0.558108072	0.791036445
RPS20P33	-0.020	0.558060594	0.791010741
OR8H1	-0.020	0.558034893	0.791010741
TMEM71	-0.020	0.557817972	0.790737441
RN7SKP293	-0.020	0.557706031	0.790672891
ARHGAP42P2	-0.020	0.557647916	0.790614035
RN7SL288P	-0.020	0.557545931	0.790492977

AC114877	-0.020	0.557533331	0.790492977
C1orf115	-0.020	0.557522151	0.790492977
CTD-2116N17	-0.020	0.557510592	0.790492977
RP11-108E14	-0.020	0.557385124	0.790359095
UBE2D2	-0.020	0.557377572	0.790359095
RNU6-1105P	-0.020	0.557346032	0.790359095
RP11-1082I11	-0.020	0.557343636	0.790359095
RP11-481E4	-0.020	0.557318774	0.790359095
AC008440	-0.020	0.557316397	0.790359095
MIR4779	-0.020	0.55720123	0.790309269
AC124914	-0.020	0.55719701	0.790309269
RP11-38F22	-0.020	0.557151216	0.790286237
RP11-489C13	-0.020	0.556953568	0.790076475
ACSL3	-0.020	0.556756529	0.789833504
ZDHHC24	-0.020	0.556612047	0.789686087
RNU6-276P	-0.020	0.556551612	0.789647391
TMTC2	-0.020	0.556470149	0.78955533
CTD-2074D8	-0.020	0.556412298	0.78952029
RP11-270C12	-0.020	0.556372384	0.789487175
OR51H1	-0.020	0.556348629	0.789476989
RP11-697E22	-0.020	0.556330098	0.789476989
CHCHD3P3	-0.020	0.55628974	0.789463988
HDAC5	-0.020	0.556280612	0.789463988
OGDH	-0.020	0.556206566	0.789416519
DEFB125	-0.020	0.55613607	0.789363511
ASS1P6	-0.020	0.555955133	0.789224295
PRSS33	-0.020	0.555826678	0.789136028
TLR2	-0.020	0.555701566	0.789052487
OR10A7	-0.020	0.55563837	0.789009799
CTD-2105E13	-0.020	0.555617893	0.789004246
LMOD2	-0.020	0.555440175	0.788798915
AC091770	-0.020	0.555325053	0.788705979
TAL1	-0.020	0.555323146	0.788705979
ZNF679	-0.020	0.555310995	0.788705979
PEX12P1	-0.020	0.555158551	0.788652676
NMNAT1	-0.020	0.555039322	0.78862941
FRMD3	-0.020	0.555036393	0.78862941
GOLGA2P2Y	-0.020	0.555029169	0.78862941
HNRNPCP7	-0.020	0.554949612	0.78862941
RP11-553N19	-0.020	0.554922324	0.788627862
RP11-258O13	-0.020	0.554838436	0.788579236
PDE8A	-0.020	0.554821467	0.788578651
CTB-191D16	-0.020	0.554700995	0.788501545
RN7SKP253	-0.020	0.554522409	0.788353063
NISCH	-0.020	0.554472985	0.788342135
RPL7P52	-0.020	0.55439254	0.788274823
RP11-344A16	-0.020	0.554384786	0.788274823
RNU6ATAC18P	-0.020	0.554318743	0.788216957
AL451006	-0.020	0.554316743	0.788216957
CCBL2	-0.020	0.55412461	0.788011489
RBBP7	-0.020	0.554118024	0.788011489
SNORD62A	-0.020	0.554068178	0.788001825

RNU2-59P	-0.020	0.553964809	0.787901868
RPL5P16	-0.020	0.553934218	0.787881888
C20orf202	-0.020	0.553933597	0.787881888
KRTAP7-1	-0.020	0.553933209	0.787881888
RP11-393N21	-0.020	0.553911468	0.787881888
MCM7	-0.020	0.553820967	0.787861978
LLfos-48D6	-0.020	0.553801025	0.787861978
RP11-275O18	-0.020	0.553733225	0.787854821
NEUROG3	-0.020	0.553677428	0.787798968
AL606923	-0.020	0.553657535	0.787794198
ZNF329	-0.020	0.553611025	0.787751554
TYW1B	-0.020	0.553547757	0.787699113
SNX30	-0.020	0.553545197	0.787699113
RNA5SP436	-0.020	0.553474523	0.787674987
RNA5SP247	-0.020	0.55340953	0.787629562
CA7	-0.020	0.553390056	0.787625383
AC007450	-0.020	0.553337386	0.787573955
MIR4526	-0.020	0.55330293	0.787548448
TUB	-0.020	0.553238419	0.787503697
SYTL5	-0.020	0.553163901	0.787442999
CXorf40B	-0.020	0.553118875	0.787404133
CMTM1	-0.020	0.553070988	0.787398641
AC006480	-0.020	0.553034266	0.787377824
RP3-455J7	-0.020	0.552823389	0.787171715
SLC18A2	-0.020	0.55274883	0.787089081
AC004745	-0.020	0.552733502	0.787089081
FAM210A	-0.020	0.552732179	0.787089081
KRTAP9-6	-0.020	0.552690758	0.787076986
REG3A	-0.020	0.552658161	0.787076986
COPB2	-0.020	0.55262292	0.787074516
NLGN4Y-AS1	-0.020	0.552617318	0.787074516
ICE1	-0.020	0.552507095	0.787027229
SMIM21	-0.020	0.552340532	0.78682298
RNY4P6	-0.020	0.552219025	0.786711003
RP13-238F13	-0.020	0.552101265	0.786613826
RNU1-23P	-0.020	0.552077826	0.786603962
RAB9AP1	-0.020	0.551919397	0.786542941
FAM90A5P	-0.020	0.551886009	0.78654243
FAM83D	-0.020	0.551737906	0.786472563
CTD-3137H5	-0.020	0.55161881	0.786326331
TOE1	-0.020	0.551575443	0.786326331
TBCEL	-0.020	0.551538938	0.78631598
TAF11	-0.020	0.551494768	0.786290715
ZNF284	-0.020	0.551409354	0.786192471
KLF3P2	-0.020	0.551406031	0.786192471
GLUD1P9	-0.020	0.551358538	0.786190625
FAM160B1	-0.020	0.551296573	0.786151099
RP11-537P22	-0.020	0.551288	0.786151099
ADPRHL1	-0.020	0.551229421	0.786124195
EEF1A2	-0.020	0.551186723	0.78608684
RPL30P7	-0.020	0.551138609	0.78604176
BOD1P1	-0.020	0.551083386	0.786010075

RBFA	-0.020	0.551011933	0.785955237
TOR1A	-0.020	0.550899922	0.785819
AC099796	-0.020	0.550827427	0.785739124
PIF1	-0.020	0.550801055	0.785725038
GEMIN8	-0.020	0.550750348	0.785676236
GAGE2E	-0.020	0.550716216	0.785651078
AC091643	-0.020	0.550678122	0.785648689
CTB-50E14	-0.020	0.550638325	0.785634089
TTC13	-0.020	0.550636174	0.785634089
AL121933	-0.020	0.550619993	0.785634089
RP11-467I17	-0.020	0.550611848	0.785634089
AL356289	-0.020	0.550604619	0.785634089
CTD-2122P11	-0.020	0.550586187	0.785634089
RNU6-687P	-0.020	0.550538823	0.785633333
RP11-98O2	-0.020	0.550530914	0.785633333
RP11-355I22	-0.020	0.550495588	0.785618714
RNU6-754P	-0.020	0.550406857	0.785562703
PRKRIRP9	-0.020	0.550401802	0.785562703
MIR4289	-0.020	0.550400501	0.785562703
FXYD7	-0.020	0.550358914	0.785562703
ZEB2	-0.020	0.550355349	0.785562703
RP11-88H12	-0.020	0.550332215	0.785562703
PEAR1	-0.020	0.550323259	0.785562703
AC144449	-0.020	0.550278466	0.785562703
RP11-169K19	-0.020	0.550224861	0.785538353
LINC01243	-0.020	0.550086244	0.785458178
SNORD121A	-0.020	0.550075553	0.785458178
OR4V1P	-0.020	0.550039037	0.785437869
PPARA	-0.020	0.549937159	0.785386581
MIR4326	-0.020	0.549806347	0.785223309
HBQ1	-0.020	0.549795563	0.785223309
SERPINA1	-0.020	0.549737781	0.785172474
RP11-685B24	-0.020	0.549728107	0.785172474
IDNK	-0.020	0.549607676	0.785080823
MAFIP	-0.020	0.549547278	0.785041638
RP11-229M1	-0.020	0.549515748	0.785020143
CAMSAP3	-0.020	0.549448801	0.784995142
SAYSD1	-0.020	0.54943204	0.784995142
RNU6-1080P	-0.020	0.549425488	0.784995142
NUS1P1	-0.020	0.549420498	0.784995142
SIX1	-0.020	0.549342775	0.784981746
GRAMD4	-0.020	0.549312172	0.784981746
AL079306	-0.020	0.549308082	0.784981746
RP11-348P10	-0.020	0.54926809	0.784981746
RNA5SP332	-0.020	0.549186483	0.784926467
RNU4ATAC11P	-0.020	0.549165373	0.784924511
POLB	-0.020	0.549100885	0.784874785
PRELID2P1	-0.020	0.549089781	0.784874785
TUBB8P6	-0.020	0.549040032	0.784834912
OR4A21P	-0.020	0.548948822	0.784728084
RP11-147I3	-0.020	0.548865966	0.78467747
DUSP12	-0.020	0.54883137	0.78467747

GABRG2	-0.020	0.548705481	0.78454506
RP11-571F15	-0.020	0.54863434	0.784490444
SPPL3	-0.020	0.548573207	0.784487425
OTUD7A	-0.020	0.548544445	0.784479665
MIR181C	-0.020	0.548454894	0.784414487
RP11-3K16	-0.020	0.548439586	0.784414487
USP11	-0.020	0.548436685	0.784414487
AC002116	-0.020	0.54842176	0.784414487
RNU6-106P	-0.020	0.548391817	0.784414487
VN1R17P	-0.020	0.548391491	0.784414487
CTC-575C13	-0.020	0.548391255	0.784414487
AP003354	-0.020	0.548382303	0.784414487
ATG4AP1	-0.020	0.5482632	0.784383642
PRR23B	-0.020	0.548200719	0.784317812
CTD-2544M6	-0.020	0.548130581	0.78426184
NAT8L	-0.020	0.54803741	0.78417839
KRT19P3	-0.020	0.548026959	0.78417839
AC004932	-0.020	0.548018558	0.78417839
TCAF2P1	-0.020	0.547936595	0.784128368
RNU6-1248P	-0.020	0.547899459	0.784120868
RP11-236P24	-0.020	0.547801945	0.784029908
HMGN1P12	-0.020	0.547763989	0.784022704
RP3-468B3	-0.020	0.547746301	0.78402095
LINC00309	-0.020	0.547547537	0.783807116
LINC01395	-0.020	0.54754073	0.783807116
MIR3907	-0.020	0.54740029	0.783690552
RP11-537A6	-0.020	0.547373759	0.783676127
KCNC4	-0.020	0.547337477	0.783660755
CBWD2	-0.021	0.547232016	0.783627403
AC015688	-0.021	0.547163127	0.783627403
ZNF334	-0.021	0.547093477	0.783581061
RN7SKP17	-0.021	0.54706749	0.783570653
RNU6-871P	-0.021	0.546995792	0.783535403
RP3-323K23	-0.021	0.546923526	0.783502581
RP11-739A13	-0.021	0.546843508	0.783435079
FANCG	-0.021	0.546799731	0.783395926
EXOSC8	-0.021	0.546731031	0.783355717
PRH1-PRR4	-0.021	0.546718821	0.783355717
MRPS6P2	-0.021	0.546706292	0.783355717
RNU6-369P	-0.021	0.546632262	0.78332094
KRTAP9-2	-0.021	0.546499464	0.783295614
AC096643	-0.021	0.546495861	0.783295614
NDUFV3	-0.021	0.546479898	0.783295614
ELOVL6	-0.021	0.546435564	0.78327536
OXSM	-0.021	0.546388326	0.783254178
SNORD115-45	-0.021	0.546387456	0.783254178
RP11-346D6	-0.021	0.546341026	0.783233522
TNS3	-0.021	0.546292039	0.783186868
LINC01381	-0.021	0.54626389	0.783170087
ABCG2	-0.021	0.54623754	0.783170087
AC009336	-0.021	0.546202476	0.783170087
RP11-360I2	-0.021	0.546194332	0.783170087

RN7SKP123	-0.021	0.546181061	0.783170087
RPL23AP39	-0.021	0.546112325	0.783170087
RNU2-29P	-0.021	0.546083499	0.783170087
RP11-469J4	-0.021	0.546067058	0.783170087
RP11-304L19	-0.021	0.546043548	0.783170087
RP11-138E9	-0.021	0.546000449	0.783170087
HES5	-0.021	0.54594385	0.783170087
RP11-229A12	-0.021	0.545923643	0.783170087
RP11-358M3	-0.021	0.54581102	0.783133743
ZNF749	-0.021	0.545736644	0.783074208
RP13-362E11	-0.021	0.545727325	0.783074208
RP11-846F4	-0.021	0.545675944	0.783074208
VTRNA2-1	-0.021	0.545664019	0.783074208
CSF1	-0.021	0.545659295	0.783074208
AC006548	-0.021	0.545459008	0.78288811
MIR513A1	-0.021	0.545351473	0.782828132
CDH5	-0.021	0.545337252	0.782828132
ACTR10	-0.021	0.545248679	0.782727758
RP11-97F8	-0.021	0.545208039	0.782693009
SUGT1P2	-0.021	0.545155854	0.782641684
RP11-316K19	-0.021	0.545085165	0.782587379
AC096732	-0.021	0.54492946	0.782477159
CTD-2653D5	-0.021	0.544926946	0.782477159
AC138430	-0.021	0.544882438	0.782477159
CCL13	-0.021	0.54482085	0.782443548
AC007966	-0.021	0.544731031	0.782361955
AL353731	-0.021	0.544599262	0.782267065
YBX1P5	-0.021	0.544504416	0.782201599
ARL15	-0.021	0.544501902	0.782201599
RP11-404O13	-0.021	0.544415591	0.78212118
RP11-122A21	-0.021	0.544177607	0.781850037
RP11-69H7	-0.021	0.544116203	0.781785398
RN7SL693P	-0.021	0.544100848	0.781785398
SPOPL	-0.021	0.544095344	0.781785398
RN7SL775P	-0.021	0.544051369	0.781762998
RNU6-484P	-0.021	0.544026903	0.781751427
LA16c-325D7	-0.021	0.543877238	0.781604132
RP11-627K11	-0.021	0.543873088	0.781604132
RPS26P55	-0.021	0.543851184	0.781604132
RNU5B-1	-0.021	0.543788961	0.781574579
CSPG4P5	-0.021	0.543749229	0.781541059
RP11-319G9	-0.021	0.54371136	0.7815338
LINC01031	-0.021	0.543663127	0.7814937
AC104981	-0.021	0.543596989	0.781466559
AL627171	-0.021	0.543546388	0.781466559
GCLM	-0.021	0.54343306	0.781389875
CHERP	-0.021	0.543403344	0.781389875
LINC01182	-0.021	0.543323865	0.78135412
FNTAP2	-0.021	0.543280623	0.781315525
RP11-529K1	-0.021	0.543277969	0.781315525
RP5-996D20	-0.021	0.543192179	0.781259102
ASS1P1	-0.021	0.543188627	0.781259102

RNU6-1241P	-0.021	0.543148031	0.781257189
ZCCHC13	-0.021	0.543080868	0.781216965
RNU2-68P	-0.021	0.543051978	0.781216965
AC002543	-0.021	0.543021085	0.781201753
H1FOO	-0.021	0.543013897	0.781201753
LRRC37A3	-0.021	0.542934165	0.781168861
RP11-552M14	-0.021	0.542894316	0.781168861
MDFIC	-0.021	0.542891424	0.781168861
RP11-716D19	-0.021	0.542867796	0.781168861
LYZL2	-0.021	0.542767648	0.781096685
FAM205BP	-0.021	0.542671742	0.780991419
PHF19	-0.021	0.542665429	0.780991419
PTPRVP	-0.021	0.542656632	0.780991419
RP13-467H17	-0.021	0.542610664	0.780988761
CTD-2369P2	-0.021	0.542605316	0.780988761
MRPS14	-0.021	0.542505015	0.780940364
AC104802	-0.021	0.542357487	0.780813133
RPL7P48	-0.021	0.542237736	0.780687928
RP5-1163L11	-0.021	0.54223589	0.780687928
RP11-606D9	-0.021	0.542196018	0.780675064
C1orf101	-0.021	0.542181001	0.780675064
CAAP1	-0.021	0.542145529	0.780649568
IL10RB	-0.021	0.542141909	0.780649568
BCL2L2-PABPN1	-0.021	0.542132491	0.780649568
FAM174A	-0.021	0.542127793	0.780649568
TFF2	-0.021	0.54197523	0.780569546
MED28P8	-0.021	0.541932053	0.780530963
RP11-215I16	-0.021	0.541866904	0.78050794
MTND2P11	-0.021	0.541798901	0.780437851
RPS20P25	-0.021	0.541724915	0.780397826
ATP5F1P2	-0.021	0.541692712	0.780375039
RP11-1167A19	-0.021	0.541651247	0.780338907
THUMPD1P1	-0.021	0.541616403	0.780312313
AC104387	-0.021	0.541523381	0.780225499
G6PC2	-0.021	0.541361039	0.780038791
FAM45A	-0.021	0.541295976	0.779992238
RNU6-1139P	-0.021	0.541295203	0.779992238
POU3F3	-0.021	0.541283453	0.779992238
GTF2IRD2P1	-0.021	0.541271859	0.779992238
OLR1	-0.021	0.541232323	0.779992238
RP11-332E19	-0.021	0.541219003	0.779992238
MT1HL1	-0.021	0.541144096	0.779938564
LINC00665	-0.021	0.540871087	0.77968346
CSPG4P8	-0.021	0.540802398	0.779611229
TRIM71	-0.021	0.540801213	0.779611229
PDE6B	-0.021	0.540742749	0.779596032
SH3KBP1	-0.021	0.540607198	0.779471392
TAS2R42	-0.021	0.540562259	0.779471207
GAPDHP76	-0.021	0.540551599	0.779471207
CDKN2AIPNLP3	-0.021	0.540485334	0.779437261
RP11-373A6	-0.021	0.540464016	0.779430119
ATG12P2	-0.021	0.54045074	0.779430119

RP11-124N2	-0.021	0.540418338	0.779416379
RNU6-1131P	-0.021	0.54040965	0.779416379
RP11-296K13	-0.021	0.540343454	0.779374248
AL513487	-0.021	0.540334887	0.779374248
OR1D3P	-0.021	0.540267697	0.779335784
RP1-249C1	-0.021	0.540234312	0.779311231
GOLGA8T	-0.021	0.54019928	0.779284299
VIPAS39	-0.021	0.540071663	0.779171002
CACNG4	-0.021	0.540010972	0.779165536
AC018628	-0.021	0.539998367	0.779165536
OR5BM1P	-0.021	0.539904548	0.779104285
MTM1	-0.021	0.539884542	0.779104285
RPL32P26	-0.021	0.539835928	0.779090504
MAVS	-0.021	0.539810858	0.779090504
IPO8P1	-0.021	0.539746581	0.779055987
HSFY4P	-0.021	0.539746305	0.779055987
RNA5SP281	-0.021	0.539649323	0.778986431
AC090602	-0.021	0.539633648	0.778986431
ATP5HP1	-0.021	0.539562119	0.7789786
AC004053	-0.021	0.539554601	0.7789786
RP11-457D13	-0.021	0.53943167	0.778837486
MTND1P31	-0.021	0.539391416	0.77881278
GDAP1L1	-0.021	0.539390201	0.77881278
NPPC	-0.021	0.539382454	0.77881278
RNA5SP448	-0.021	0.539337002	0.77881278
FMO8P	-0.021	0.539302902	0.778793224
TEX2	-0.021	0.539293407	0.778793224
KB-1083B1	-0.021	0.539214476	0.778712752
ZEB2P1	-0.021	0.539172604	0.778712752
RP11-500G10	-0.021	0.539137443	0.778712752
RNU6-703P	-0.021	0.539094272	0.778704443
SNRPFP2	-0.021	0.539081874	0.778704443
RP11-662J14	-0.021	0.538992046	0.778674862
GLUD1P7	-0.021	0.53898442	0.778674862
RNA5SP134	-0.021	0.538952616	0.778665136
LDLRAD1	-0.021	0.538940507	0.778665136
HAPLN4	-0.021	0.538858164	0.778579566
LINC00353	-0.021	0.538848562	0.778579566
NMNAT1P2	-0.021	0.538814635	0.778579566
NAPSA	-0.021	0.538711617	0.778482255
SLC25A20	-0.021	0.538597245	0.778482255
TTTY5	-0.021	0.538573068	0.778482255
TRIP13	-0.021	0.538551066	0.778482255
H3F3BP2	-0.021	0.538548865	0.778482255
RP11-215P8	-0.021	0.538507054	0.778482255
ARHGAP27	-0.021	0.538409297	0.778470539
MYL2	-0.021	0.538405008	0.778470539
OR9G3P	-0.021	0.53840311	0.778470539
STMN4	-0.021	0.538224749	0.778289015
JAK1	-0.021	0.538114274	0.778162092
SNORA19	-0.021	0.538086827	0.778146029
PRKRIR	-0.021	0.53805261	0.778120174

RNA5SP518	-0.021	0.538026493	0.77810603
CENPVP1	-0.021	0.538013945	0.77810603
RP11-90M2	-0.021	0.537921111	0.778071757
OR8G2P	-0.021	0.537913569	0.778071757
SH2B2	-0.021	0.537873771	0.77805968
RP3-473B4	-0.021	0.537712826	0.777983133
HMGN3	-0.021	0.537677659	0.777955884
RNA5SP413	-0.021	0.537662644	0.777955884
SNORA80E	-0.021	0.537613549	0.777920164
UBQLN3	-0.021	0.537588272	0.777920164
KRTAP5-8	-0.021	0.537547133	0.777920164
RP4-568C11	-0.021	0.537427523	0.777782997
MPC1	-0.021	0.537364458	0.777715359
RPL6P12	-0.021	0.537346077	0.777715359
CBX3P8	-0.021	0.537308679	0.777705529
AGPAT9	-0.021	0.537297072	0.777705529
RNU6-1089P	-0.021	0.537270393	0.777705529
UNC80	-0.021	0.537241415	0.777705529
RP11-644L4	-0.021	0.537182763	0.777694583
RPL35AP26	-0.021	0.537157611	0.777694583
RAPH1	-0.021	0.537081251	0.777636308
RPL7P53	-0.021	0.537068768	0.777636308
RNU6-752P	-0.021	0.5370203	0.777605649
RP11-524O24	-0.021	0.536962711	0.77758287
MIR363	-0.021	0.536949708	0.77758287
RP11-51C14	-0.021	0.536935875	0.77758287
MIR6740	-0.021	0.536864049	0.777534561
MT-TL2	-0.021	0.536738706	0.777400309
ITLN1	-0.021	0.53669456	0.777360009
RP11-14N4	-0.021	0.536690371	0.777360009
OR52N2	-0.021	0.536687533	0.777360009
AC108205	-0.021	0.536683138	0.777360009
FOXI3	-0.021	0.536671038	0.777360009
OR14A2	-0.021	0.536662828	0.777360009
GNAT2	-0.021	0.536636885	0.777360009
RP11-162D9	-0.021	0.536633247	0.777360009
Z83851	-0.021	0.536584558	0.777360009
BMI1P1	-0.021	0.536567723	0.777360009
RP1-209B9	-0.021	0.536417086	0.777265403
FAM166A	-0.021	0.536350969	0.777193249
RNU6-926P	-0.021	0.536335603	0.777193249
OR1G1	-0.021	0.536305571	0.777193249
RP11-344E13	-0.021	0.536280152	0.777185211
SWT1	-0.021	0.536247558	0.777161623
MND1	-0.021	0.536244318	0.777161623
RNU6-1266P	-0.021	0.53618793	0.777122502
CLUL1	-0.021	0.536139567	0.777099704
BNIP3P22	-0.021	0.536132359	0.777099704
CLP1	-0.021	0.53612265	0.777099704
POLR3G	-0.021	0.536098894	0.777099704
MIR7150	-0.021	0.535995526	0.776985509
TMEM26	-0.021	0.535964286	0.776963872

LINC00689	-0.021	0.535942548	0.776963872
RNA5SP522	-0.021	0.535929903	0.776963872
RN7SKP52	-0.021	0.535835935	0.776919693
RNU6-1316P	-0.021	0.535788281	0.776874249
AC002550	-0.021	0.535748014	0.776857775
RP11-129I19	-0.021	0.535738197	0.776857775
RP11-336N8	-0.021	0.535706497	0.776850268
FFAR4	-0.021	0.535660942	0.776831512
AL138726	-0.021	0.535632135	0.776830763
KIAA0087	-0.021	0.535604138	0.776830763
FAM92A1	-0.021	0.535518115	0.776789952
RP11-977G19	-0.021	0.535365597	0.77664979
CLNS1A	-0.021	0.535330348	0.77664979
SPOCK3	-0.021	0.535086702	0.776447942
RP11-1016B18	-0.021	0.535062957	0.776437143
RN7SKP114	-0.021	0.534970921	0.776350896
SNX18P8	-0.021	0.534902287	0.776298603
HIGD1AP17	-0.021	0.534858917	0.77628297
CYP2A6	-0.021	0.53480323	0.776227319
RP3-358H7	-0.021	0.534753482	0.776200905
CCT5P2	-0.021	0.534716806	0.776171324
FAM209B	-0.021	0.534686249	0.776150625
RP11-10C24	-0.021	0.534676298	0.776150625
TRIM59	-0.021	0.534649698	0.776144879
PAX8	-0.021	0.534628851	0.776144879
AKAP8P1	-0.021	0.534622581	0.776144879
AC061992	-0.021	0.534520269	0.776084187
NDUFB8P2	-0.021	0.534496892	0.776084187
CHCHD2P5	-0.021	0.534492761	0.776084187
IMMP1LP1	-0.021	0.534474418	0.776084187
MTND6P17	-0.021	0.534464816	0.776084187
RP11-203P23	-0.021	0.534387444	0.776048018
RP11-259G18	-0.021	0.534377595	0.776048018
RP11-25L19	-0.021	0.534313604	0.775988109
GLTP	-0.021	0.534300015	0.775988109
AL139319	-0.021	0.534275553	0.775980171
AC119751	-0.021	0.534175028	0.775857829
DET1	-0.021	0.534105996	0.775857829
RN7SL564P	-0.021	0.534097795	0.775857829
COX7A2P1	-0.021	0.534041026	0.775852482
TATDN2P1	-0.021	0.533969865	0.775796429
PHBP13	-0.021	0.533947165	0.775787114
CISH	-0.021	0.533911653	0.775759184
RP4-569M23	-0.021	0.533894771	0.775758321
CTD-2292P10	-0.021	0.533698671	0.775591695
EIF2S2	-0.021	0.533644086	0.775559698
MIR196A1	-0.021	0.533599512	0.775542249
AK2P2	-0.021	0.533576251	0.775532107
AC099757	-0.021	0.533574024	0.775532107
RBM8B	-0.021	0.53355362	0.775532107
RP11-241G9	-0.021	0.533533442	0.775532107
ZNF66	-0.021	0.533441929	0.775455201

RPL23AP83	-0.021	0.53331679	0.775339465
OR12D2	-0.021	0.533313105	0.775339465
RP5-1118D24	-0.021	0.533236394	0.775339465
ELK1P1	-0.021	0.533226734	0.775339465
ZNF613	-0.021	0.533220786	0.775339465
RP11-653B10	-0.021	0.53309506	0.77528225
RNA5SP408	-0.021	0.533014391	0.775188604
OR7E26P	-0.021	0.532917698	0.775095316
BACH1-IT3	-0.021	0.532820786	0.775001697
SNORD116-27	-0.021	0.532820307	0.775001697
MIR942	-0.021	0.532749767	0.774993072
RP11-223P11	-0.021	0.532674529	0.774930961
CR381670	-0.021	0.532433993	0.774710661
SNORD30	-0.021	0.532317823	0.774601312
RNU6-49P	-0.021	0.532179481	0.774447328
DNAJC25	-0.021	0.532172063	0.774447328
SRR	-0.021	0.532145168	0.77444472
LELP1	-0.021	0.532014836	0.774349691
NEK4P2	-0.021	0.531954919	0.774286144
AHCY	-0.021	0.531876972	0.774220012
P2RX4	-0.021	0.531841481	0.774192011
DBIL5P	-0.021	0.531757956	0.774165072
CTB-31N19	-0.021	0.531673003	0.774065055
PTGER4P3	-0.021	0.531650012	0.774055244
RP1-39G22	-0.021	0.531624616	0.77404193
MSI1	-0.021	0.531578023	0.773997752
RP11-145B3	-0.021	0.531560225	0.7739955
IMP3	-0.021	0.53155616	0.7739955
RP11-801I18	-0.021	0.531493406	0.77394553
ADAM32	-0.021	0.531460832	0.773921758
TMEM59	-0.021	0.531365153	0.77382975
EPB41L4B	-0.021	0.531301731	0.773808371
RP11-799O21	-0.021	0.53124813	0.773777628
RP11-174C7	-0.021	0.531160619	0.773673827
ZNF274	-0.021	0.531082828	0.773631498
RCC2P5	-0.021	0.531049446	0.773630196
TDGF1P1	-0.021	0.530968454	0.773583193
RP11-1391J7	-0.021	0.530936321	0.77356004
NCOR1P3	-0.021	0.530872697	0.773491003
LL22NC03-N64E9	-0.021	0.530852821	0.773485707
RP11-1180F24	-0.021	0.530838809	0.773485707
RP3-344J20	-0.021	0.530789017	0.773440064
MIR7159	-0.021	0.530637287	0.773351388
CROCCP2	-0.021	0.530592879	0.773343546
PRR27	-0.021	0.530533302	0.773280376
CTAGE4	-0.021	0.530523253	0.773280376
RP11-766N7	-0.021	0.530508088	0.773280376
RN7SL250P	-0.021	0.530488313	0.773280376
AC113610	-0.021	0.530452362	0.773280376
RP11-739P1	-0.021	0.530426518	0.773266721
RP11-129H15	-0.021	0.53027428	0.773115778
AC090573	-0.021	0.530246658	0.773099174

C5orf28	-0.021	0.530245971	0.773099174
AC243836	-0.021	0.530162028	0.773070446
RP11-728E14	-0.021	0.530148168	0.773070446
POU6F1	-0.021	0.530074171	0.772989667
AC019055	-0.021	0.530000814	0.772930025
RP11-435D7	-0.021	0.529949042	0.772901856
MIR6811	-0.021	0.529886687	0.772882925
RNA5SP512	-0.021	0.529825483	0.772882925
RP11-776H12	-0.021	0.529816349	0.772882925
Z70272	-0.021	0.529806607	0.772882925
RP11-702L6	-0.021	0.529750824	0.772863328
CFTRP1	-0.021	0.529733801	0.772863328
DAPK1	-0.021	0.529721353	0.772863328
RP11-241F15	-0.021	0.529682479	0.772863328
RP11-181K3	-0.021	0.529646549	0.772863328
OR56B3P	-0.021	0.529642388	0.772863328
RP11-208N20	-0.021	0.52960598	0.772863328
MGAT4EP	-0.021	0.529557829	0.772828274
SUB1P4	-0.021	0.529540269	0.772828274
L3MBTL4-AS1	-0.021	0.529404419	0.772722779
RNU6-141P	-0.021	0.529257438	0.772543394
TJP2	-0.021	0.529123695	0.772455071
RP11-32D4	-0.021	0.529072815	0.772428146
PKNOX2-AS1	-0.021	0.529068735	0.772428146
RNY1P4	-0.021	0.528994729	0.772385173
AC104058	-0.021	0.52895695	0.77238122
RP11-701H16	-0.021	0.528845867	0.77230988
EIF3F	-0.021	0.528782994	0.772265421
RP11-561O23	-0.021	0.528781523	0.772265421
C3orf67-AS1	-0.021	0.528686977	0.772219912
RNU7-176P	-0.021	0.528618857	0.772167774
RP11-812I20	-0.021	0.528595674	0.772167774
RP11-684B2	-0.021	0.528541655	0.772126048
RP11-1415C14	-0.021	0.528503779	0.772094399
RNU6-421P	-0.021	0.528455108	0.772077647
OR4K3	-0.021	0.528407664	0.77204871
ACVR1B	-0.021	0.528385948	0.772040666
GPN3P1	-0.021	0.52824204	0.771901441
RN7SKP147	-0.021	0.5281986	0.771885328
C9orf172	-0.021	0.528140506	0.771847797
AC002127	-0.021	0.528135421	0.771847797
RP11-323I1	-0.021	0.528111602	0.771847797
GEMIN2P1	-0.021	0.528107441	0.771847797
ZNF804B	-0.021	0.52803989	0.771820415
AC079145	-0.021	0.528035092	0.771820415
SKAP2	-0.021	0.528008341	0.771820415
PHOX2B	-0.022	0.527822324	0.771714316
OR2Y1	-0.022	0.527738594	0.771639273
CKMT2-AS1	-0.022	0.527731921	0.771639273
OR2AG1	-0.022	0.527686138	0.771630949
SERPINB8P1	-0.022	0.527665123	0.771630949
ZSCAN20	-0.022	0.527653366	0.771630949

AC063979	-0.022	0.52752011	0.77153299
TAF9B	-0.022	0.527509482	0.77153299
PALD1	-0.022	0.527461287	0.771518035
RNA5SP272	-0.022	0.527388792	0.771459381
SRPR	-0.022	0.5273467	0.771421502
MIR522	-0.022	0.527282134	0.771350745
RP11-561G1	-0.022	0.527219129	0.771282267
AL136985	-0.022	0.52721549	0.771282267
BCAS3	-0.022	0.527166455	0.771282267
FGF2	-0.022	0.526998374	0.771148813
KRT18P17	-0.022	0.526901898	0.77103133
STON2	-0.022	0.52673685	0.770813493
LINC00976	-0.022	0.526623932	0.770720101
RP11-445P19	-0.022	0.526603764	0.770720101
RP11-410D17	-0.022	0.526491296	0.77066729
MIR1268A	-0.022	0.52648377	0.77066729
RNU6-905P	-0.022	0.526465562	0.77066729
MIR597	-0.022	0.526433431	0.77066729
RTCA	-0.022	0.526432595	0.77066729
MIR4699	-0.022	0.526432391	0.77066729
IL20RA	-0.022	0.526412087	0.77066729
MIR4641	-0.022	0.526272541	0.770631278
RNA5SP30	-0.022	0.526230082	0.770627325
TMEM205	-0.022	0.526202591	0.770623621
RP11-1E1	-0.022	0.526078514	0.770560382
RN7SL510P	-0.022	0.52599286	0.770506012
RP11-99J16__A	-0.022	0.525824704	0.770283377
RN7SL608P	-0.022	0.525787659	0.770276495
DNAJC5	-0.022	0.525674666	0.770173253
MARVELD3	-0.022	0.525652928	0.770173253
GM2AP1	-0.022	0.525587305	0.77010999
RNA5SP262	-0.022	0.525586161	0.77010999
RNU6-951P	-0.022	0.525499956	0.770044502
RNU6-1008P	-0.022	0.525490286	0.770044502
QRSL1P1	-0.022	0.525478347	0.770044502
CNIH2	-0.022	0.525406352	0.770002102
LACC1	-0.022	0.525361461	0.7699837
NCKIPSD	-0.022	0.525259931	0.769858585
SIKE1	-0.022	0.525167671	0.769791091
GPS1	-0.022	0.525132408	0.769766434
RP11-165D20	-0.022	0.525042277	0.769681693
CXorf58	-0.022	0.524993129	0.76964684
RPL36AP15	-0.022	0.524967659	0.76964684
HHATL	-0.022	0.524909611	0.769629346
RNU6-306P	-0.022	0.524676863	0.769453903
PAQR9	-0.022	0.524647613	0.769447437
MAP6	-0.022	0.524583317	0.769387787
CYP2D6	-0.022	0.524579401	0.769387787
WRBP1	-0.022	0.524575996	0.769387787
KIF2C	-0.022	0.5245139	0.769387787
RP11-318C2	-0.022	0.524448663	0.769380144
AP001063	-0.022	0.524412762	0.769374569

CTD-2049O4	-0.022	0.524383943	0.769374316
LINC00477	-0.022	0.52438152	0.769374316
MLKL	-0.022	0.524346607	0.769372311
DNAJC19	-0.022	0.524332371	0.769372311
RP11-694I15	-0.022	0.524256565	0.769287593
ENGASE	-0.022	0.524138419	0.769183322
RP11-219J21	-0.022	0.523986758	0.769002239
RP11-372E1	-0.022	0.52398008	0.769002239
AC003080	-0.022	0.523927465	0.768971691
RP11-570L15	-0.022	0.523785366	0.768833039
RNU4-14P	-0.022	0.52370764	0.768772871
PNPLA8	-0.022	0.523705096	0.768772871
ALKBH5	-0.022	0.523677709	0.768772871
RNU1-56P	-0.022	0.523533974	0.768673945
LA16c-306A4	-0.022	0.523523347	0.768673945
RNU6ATAC21P	-0.022	0.523406222	0.768563348
REM1	-0.022	0.523386092	0.768563348
DHX38	-0.022	0.523359779	0.768563348
CACNB1	-0.022	0.523339868	0.768558104
RP11-315D13	-0.022	0.523208527	0.768460004
GIPC2	-0.022	0.523102734	0.768346389
RP11-570P14	-0.022	0.522925649	0.768234057
RN7SL525P	-0.022	0.522893238	0.768210138
PIDD1	-0.022	0.522885914	0.768210138
RP11-433A10	-0.022	0.522818344	0.768210138
MIR6507	-0.022	0.522812646	0.768210138
OR2A2	-0.022	0.522759203	0.768179092
SCARNA14	-0.022	0.522756153	0.768179092
C9	-0.022	0.522731544	0.768179092
RP11-114M5	-0.022	0.522678396	0.768131448
MIR6736	-0.022	0.522594997	0.768039186
NME1P1	-0.022	0.522580297	0.768039186
POC1A	-0.022	0.522541229	0.768039186
RP11-1113C11	-0.022	0.522483304	0.767986924
CTD-2139B15	-0.022	0.522474003	0.767986924
FEM1AP2	-0.022	0.522472932	0.767986924
SPATA31E2P	-0.022	0.522464104	0.767986924
RNU6-1319P	-0.022	0.522324821	0.767919872
RP11-28A22	-0.022	0.522223315	0.767865452
XKRX	-0.022	0.522216906	0.767865452
RNU6-1286P	-0.022	0.522210633	0.767865452
KCNJ11	-0.022	0.522177838	0.767865452
RN7SKP48	-0.022	0.522121434	0.767815088
CH17-451C3	-0.022	0.522115841	0.767815088
RP4-660H19	-0.022	0.521959795	0.767667577
RNU6-919P	-0.022	0.521794507	0.767471881
AC110056	-0.022	0.521778905	0.767471881
ASNS	-0.022	0.521771263	0.767471881
EIF4A1P8	-0.022	0.521735687	0.767471881
RP11-138B4	-0.022	0.521664526	0.7674229
KPNA4	-0.022	0.52156076	0.767293949
AC063980	-0.022	0.521456688	0.767188239

RP11-497K15	-0.022	0.521327161	0.767045063
DNAJB6	-0.022	0.521322621	0.767045063
RFX3	-0.022	0.521275426	0.767040032
GHRH	-0.022	0.521216126	0.766976469
AC005251	-0.022	0.521212441	0.766976469
LINC01465	-0.022	0.521172172	0.766976469
TANGO2	-0.022	0.521033452	0.766849821
INSM2	-0.022	0.520866813	0.766675641
UBTFL7	-0.022	0.52078513	0.766618084
NUFIP1	-0.022	0.520782314	0.766618084
RCCD1	-0.022	0.520723086	0.766582542
RP5-968P14	-0.022	0.520680884	0.766544108
CTD-2154I11	-0.022	0.520646823	0.766544108
HS3ST3B1	-0.022	0.520644002	0.766544108
OR4K8P	-0.022	0.520568387	0.766544108
MIR3180-5	-0.022	0.520522782	0.766544108
CTB-25J19	-0.022	0.52043106	0.766460624
RN7SL744P	-0.022	0.520415223	0.766460624
SETP12	-0.022	0.520365415	0.766411343
RP11-10N16	-0.022	0.520365218	0.766411343
RNU7-113P	-0.022	0.520267837	0.766315024
SNORA18	-0.022	0.520208583	0.766252185
CTD-2210A23	-0.022	0.520189899	0.766252185
ERVFRD-3	-0.022	0.520136777	0.766252185
PRPF38AP2	-0.022	0.520086305	0.766238826
MARK2P14	-0.022	0.520077993	0.766238826
RHEBP1	-0.022	0.520054009	0.766238826
SPRYD7	-0.022	0.519977887	0.766238826
PRDM13	-0.022	0.519915953	0.766175844
PKD1P1	-0.022	0.519914281	0.766175844
BCL9	-0.022	0.519886813	0.766175844
AC018690	-0.022	0.519864327	0.766175844
RPL17P40	-0.022	0.51986402	0.766175844
SCAND3P1	-0.022	0.519825396	0.766175844
MST1P2	-0.022	0.519791141	0.766175844
AC068039	-0.022	0.519673869	0.766079836
MYOM2	-0.022	0.519657925	0.766079836
LINC01529	-0.022	0.519573402	0.766002861
LINC00917	-0.022	0.519571099	0.766002861
RN7SL482P	-0.022	0.519378951	0.765858426
RNU6-492P	-0.022	0.519368375	0.765858426
EIF4A1P13	-0.022	0.519366796	0.765858426
HSPA1A	-0.022	0.519312211	0.765858426
RNA5SP420	-0.022	0.519305974	0.765858426
TKTL2	-0.022	0.519277254	0.765858426
PHACTR1	-0.022	0.5192729	0.765858426
GTF2H2B	-0.022	0.519235323	0.765858426
MIR4316	-0.022	0.519227222	0.765858426
RP11-603K19	-0.022	0.51919335	0.765858426
TCEAL4P1	-0.022	0.519182084	0.765858426
MIR5188	-0.022	0.519165826	0.765858426
RNU1-8P	-0.022	0.519147852	0.765858426

RP11-111A21	-0.022	0.519128066	0.765858426
RP11-526H11	-0.022	0.51908157	0.765858426
LSM6	-0.022	0.51905796	0.765858426
OR52N5	-0.022	0.518915154	0.765719792
EIF2AP4	-0.022	0.518904228	0.765719792
RGS12	-0.022	0.518895457	0.765719792
SLC44A4	-0.022	0.518866074	0.765718543
GNAQ	-0.022	0.51876342	0.765638222
RN7SL635P	-0.022	0.518664687	0.7655874
RP13-895J2	-0.022	0.51863913	0.7655874
ARMC8P1	-0.022	0.518610666	0.7655874
RGS8	-0.022	0.518601321	0.7655874
RP11-274J15	-0.022	0.518456493	0.765493611
BOD1	-0.022	0.518426859	0.765493611
RP11-252P18	-0.022	0.518345416	0.765448255
MIR548I3	-0.022	0.518345364	0.765448255
MIR3131	-0.022	0.518316162	0.765448255
PYCARD-AS1	-0.022	0.518282828	0.765427029
RNA5SP121	-0.022	0.518274644	0.765427029
AL109612	-0.022	0.518233965	0.765402333
ANK2	-0.022	0.518223774	0.765402333
RP11-104E19	-0.022	0.518195225	0.765402333
SLC6A13	-0.022	0.518125699	0.765364465
RN7SL466P	-0.022	0.518121968	0.765364465
CTD-2127O16	-0.022	0.518097293	0.765364465
RPL12P3	-0.022	0.51806321	0.765363748
ZNF519P1	-0.022	0.518062934	0.765363748
RNA5-8SP7	-0.022	0.518061037	0.765363748
RP11-511H9	-0.022	0.517973819	0.765302904
PITPNA	-0.022	0.517935845	0.765270537
RP11-613M10	-0.022	0.517833993	0.765161343
SUCLG2-AS1	-0.022	0.517817019	0.765161343
RP11-131J4	-0.022	0.517745101	0.765154867
RPS26P24	-0.022	0.517725114	0.765149072
ATL3	-0.022	0.517565267	0.764960308
NANOGP8	-0.022	0.517505351	0.764923913
FOXP1	-0.022	0.517458504	0.76489746
GCM1	-0.022	0.517383893	0.764834648
CTC-412M14	-0.022	0.517320436	0.764764577
RP11-61I13	-0.022	0.517207156	0.764644583
AL137224	-0.022	0.517059248	0.764497107
RP11-436P7	-0.022	0.516973951	0.764418453
RNU6-1176P	-0.022	0.516889219	0.764316894
GCOM2	-0.022	0.51678689	0.764272784
DUSP13	-0.022	0.516771407	0.764272784
RNU6-643P	-0.022	0.516675125	0.764190124
AP002498	-0.022	0.516587061	0.764162004
GGACT	-0.022	0.516445174	0.764063565
RP11-818C3	-0.022	0.516397674	0.764017025
RP3-403L10	-0.022	0.516345462	0.763994749
RNA5SP460	-0.022	0.516339806	0.763994749
ATP5A1P10	-0.022	0.516326072	0.763994749

RP11-1036F1	-0.022	0.516205149	0.763920654
HSPE1	-0.022	0.516182469	0.763912202
RP11-383B24	-0.022	0.516168976	0.763912202
SENCR	-0.022	0.516159696	0.763912202
USP24P1	-0.022	0.516146335	0.763912202
AL133228	-0.022	0.516134459	0.763912202
RNU6ATAC3P	-0.022	0.516081758	0.763912202
RNU7-25P	-0.022	0.515975433	0.763843154
CTA-415G2	-0.022	0.515948808	0.763827481
RP4-675G8	-0.022	0.515935171	0.763827481
AC009494	-0.022	0.515912392	0.763827481
AC244230	-0.022	0.515906936	0.763827481
CTB-187M2	-0.022	0.515852725	0.763827481
EEF1A1P24	-0.022	0.515828423	0.763815455
RP11-319E16	-0.022	0.515760709	0.763738933
RP11-676M6	-0.022	0.51574443	0.763738572
CEP19	-0.022	0.515558824	0.763534939
IMMP1LP2	-0.022	0.515499496	0.763470816
GLYATL3	-0.022	0.515389324	0.763355123
CTD-2366F13	-0.022	0.515317518	0.763296244
RP11-406H23	-0.022	0.515235443	0.763198409
CTD-2108O9	-0.022	0.515208418	0.763182114
OCLN	-0.022	0.515149273	0.763120734
RP11-180O5	-0.022	0.515097343	0.763088779
EIF2A	-0.022	0.515044531	0.763034276
ARHGAP22-IT1	-0.022	0.514964236	0.762999031
BAAT	-0.022	0.514960447	0.762999031
RNU6-603P	-0.022	0.514952638	0.762999031
NEDD8	-0.022	0.514939095	0.762999031
OR7E157P	-0.022	0.514926499	0.762999031
MIR3156-3	-0.022	0.514901651	0.762999031
DPYSL5	-0.022	0.514857716	0.762994849
NIPSNAP3A	-0.022	0.514773735	0.76291787
RBMXL3	-0.022	0.514706314	0.762841687
MTCH2	-0.022	0.514544956	0.762626272
RP11-15N24	-0.022	0.514498958	0.762581827
RP11-74K19	-0.022	0.514342167	0.762420616
SLC27A3	-0.022	0.514323982	0.762420616
ATP9A	-0.022	0.51431341	0.762420616
OR2P1P	-0.022	0.514284275	0.762420616
RP11-785H20	-0.022	0.514254625	0.76241074
MIR599	-0.022	0.514125182	0.762312508
ISPD-AS1	-0.022	0.514121015	0.762312508
RN7SL151P	-0.022	0.514033951	0.762224696
GLRA3	-0.022	0.513989914	0.762199405
CHD3	-0.022	0.513878307	0.762153849
TMPRSS7	-0.022	0.513859761	0.762153849
AC009987	-0.022	0.51385639	0.762153849
MTND4P5	-0.022	0.513779295	0.762153849
ACER2	-0.022	0.513745877	0.762153466
TFAP2C	-0.022	0.513734255	0.762153466
ANKS1B	-0.022	0.51372052	0.762153466

AC060226	-0.022	0.513677585	0.762123371
C1QTNF8	-0.022	0.513641729	0.762093914
RN7SKP142	-0.022	0.513629095	0.762093914
RP11-881M11	-0.022	0.513483885	0.761978408
ISCA1P3	-0.022	0.513473107	0.761978408
RP11-341G2	-0.022	0.513415332	0.761928841
AP003305	-0.022	0.513360944	0.761927187
WBP1LP10	-0.022	0.513241428	0.761784763
RP11-291I6	-0.022	0.513196609	0.761784763
RPLP2P1	-0.022	0.513189819	0.761784763
SREK1IP1	-0.022	0.513118481	0.761784763
SLC25A15P2	-0.022	0.513112849	0.761784763
AC073055	-0.022	0.513099398	0.761784763
SNORA68	-0.022	0.513090279	0.761784763
RNU6-263P	-0.022	0.513081624	0.761784763
RPL39P31	-0.022	0.513026416	0.761784763
PPIAP32	-0.022	0.51300956	0.761784763
MTND5P7	-0.022	0.512976685	0.761784763
RP11-101O6	-0.022	0.512944904	0.761784763
snoMe28S-Am2634	-0.022	0.512935878	0.761784763
AKIRIN2	-0.022	0.512908031	0.761784763
RP11-973F15	-0.022	0.512832182	0.761770837
RP11-4B16	-0.022	0.512820767	0.761770837
AL121753	-0.022	0.512727703	0.76168691
ATP8B3	-0.022	0.512713808	0.76168691
DEFB114	-0.022	0.512602935	0.76159658
RP11-726G1	-0.022	0.512531935	0.761514849
CTD-2568P8	-0.022	0.512434581	0.761441464
AC083867	-0.022	0.512413817	0.761434366
FAM41AY1	-0.022	0.51233262	0.761337463
TBR1	-0.022	0.512286446	0.761306279
RGL3	-0.022	0.512156335	0.761218006
RNU4ATAC16P	-0.022	0.512147958	0.761218006
F8A2	-0.022	0.512140216	0.761218006
KNOP1P3	-0.022	0.512000975	0.761082099
OR5D15P	-0.022	0.51196524	0.761076486
ARAFP3	-0.022	0.511930912	0.76104921
RP11-66C24	-0.022	0.511874516	0.760989124
RNU6-575P	-0.022	0.511874241	0.760989124
RP11-717H13	-0.022	0.511724021	0.760860388
TAF13P2	-0.022	0.511714685	0.760860388
HIST2H2BC	-0.022	0.511657859	0.760857027
FAM60A	-0.022	0.511651541	0.760857027
TDGF1P6	-0.022	0.511613488	0.760857027
AL157359	-0.022	0.511604005	0.760857027
RNU6-747P	-0.022	0.51154815	0.760857027
LINC00581	-0.022	0.511519586	0.760857027
PKMP1	-0.022	0.511482312	0.760857027
ZNF519P3	-0.022	0.511453879	0.760838764
WIPI1	-0.022	0.51136235	0.760726367
RPS20P4	-0.022	0.511300643	0.76068209
RP11-572M11	-0.022	0.511245604	0.760623966

RPS27AP3	-0.022	0.511181524	0.760552387
C22orf34	-0.022	0.511107666	0.760490012
RNY4P20	-0.022	0.511058349	0.760464148
TMEM191C	-0.022	0.511043391	0.760464148
TMEM225	-0.022	0.510995823	0.760418624
KCTD7	-0.022	0.510961911	0.760391918
RP11-84C13	-0.022	0.510895565	0.760388221
TOR3A	-0.022	0.510889919	0.760388221
ATP6V1A	-0.022	0.510859017	0.760388221
MIR3152	-0.022	0.510790874	0.760327446
FGD3	-0.022	0.510727695	0.76027702
RP11-7F18	-0.022	0.510657476	0.760223069
MIR6826	-0.022	0.510513043	0.760080174
WDR83	-0.022	0.510390339	0.759921242
AL158051	-0.022	0.510378932	0.759921242
RP11-428I12	-0.022	0.510291787	0.759822013
ZNF736P10Y	-0.022	0.510268182	0.759822013
AC008691	-0.022	0.510176361	0.759768912
RP11-522I20	-0.022	0.510147487	0.759768912
U40455	-0.022	0.510064537	0.759697399
PHF5A	-0.022	0.510007489	0.759636185
AC083862	-0.022	0.509904075	0.75951758
RP11-305K5	-0.022	0.509901538	0.75951758
BMP6	-0.022	0.509878034	0.75951758
CXorf57	-0.022	0.509873474	0.75951758
RP11-148M9	-0.022	0.509856758	0.75951758
RNU6-1287P	-0.022	0.509735085	0.759444205
CCDC116	-0.022	0.509684825	0.759407706
RREB1	-0.022	0.509673889	0.759407706
RPP40P1	-0.022	0.509604911	0.759369035
SHQ1	-0.022	0.509593225	0.759369035
RP11-395N3	-0.022	0.50956541	0.75935769
ENPP7P1	-0.022	0.509473643	0.759244695
OSM	-0.022	0.509334443	0.759108509
RN7SL596P	-0.022	0.509282982	0.75907932
NDUFA12	-0.022	0.509276244	0.75907932
AC092835	-0.022	0.509096584	0.758872743
RP1-302D9	-0.022	0.509081012	0.758872743
RP11-717D12	-0.022	0.509071482	0.758872743
RP11-251M1	-0.022	0.509066483	0.758872743
PRB3	-0.022	0.508999027	0.758869828
RPS3AP43	-0.022	0.508973633	0.758855723
RP4-613A2	-0.023	0.508932628	0.758818341
RP11-95P2	-0.023	0.508854471	0.758749316
AC009404	-0.023	0.508840214	0.758749316
CTD-2533K21	-0.023	0.508836129	0.758749316
SSPO	-0.023	0.508817787	0.758749316
RP11-613M5	-0.023	0.508760843	0.758740054
RP1-265C24	-0.023	0.50870602	0.758717992
CKS1BP6	-0.023	0.508688694	0.758717992
RP11-111G20	-0.023	0.508664051	0.758717992
ABTB2	-0.023	0.508515771	0.758553014

TRAJ55	-0.023	0.508447286	0.75847461
LINC01504	-0.023	0.508423274	0.758462546
PTPMT1	-0.023	0.508383363	0.758462546
RP11-433J22	-0.023	0.508220381	0.758302384
AL354820	-0.023	0.508149795	0.758244574
ARL4P	-0.023	0.508143097	0.758244574
MIR545	-0.023	0.507944253	0.758032867
LINC01127	-0.023	0.507937015	0.758032867
HIST3H3	-0.023	0.507927368	0.758032867
GNS	-0.023	0.507922875	0.758032867
RP3-393K13	-0.023	0.507905944	0.758032867
FAM86GP	-0.023	0.507896616	0.758032867
LL22NC01-116C6	-0.023	0.507834872	0.758017282
RP11-669C19	-0.023	0.507826555	0.758017282
RN7SKP252	-0.023	0.50778112	0.758017282
MTND4P6	-0.023	0.507764444	0.758017282
MIR4487	-0.023	0.507708898	0.757966637
PPP5D1	-0.023	0.50759139	0.757862476
MIR8083	-0.023	0.507541842	0.757836013
CYB5R1	-0.023	0.507521397	0.757829244
MFSD11	-0.023	0.507481503	0.757793433
C15orf62	-0.023	0.507357633	0.757679731
JAM2	-0.023	0.507342937	0.757679731
EIF4BP5	-0.023	0.507279272	0.757629373
MKNK2P1	-0.023	0.50726081	0.757629373
RNU6-133P	-0.023	0.507174206	0.757572085
C21orf91	-0.023	0.50704243	0.757422759
TDO2	-0.023	0.507014971	0.757405496
RP11-562L8	-0.023	0.507001678	0.757405496
RP11-218I21	-0.023	0.506938506	0.757338778
CLMN	-0.023	0.506920015	0.757334909
TOMM20	-0.023	0.506911622	0.757334909
TTTY2	-0.023	0.506774908	0.75721313
MRPS33	-0.023	0.506706701	0.757158725
RP11-1029J19	-0.023	0.506705187	0.757158725
RNU6-297P	-0.023	0.50660545	0.757078688
RBM22P4	-0.023	0.506505778	0.756953488
HGF	-0.023	0.506438201	0.756908114
RP13-26D14	-0.023	0.50613832	0.756616208
AC106901	-0.023	0.506094485	0.756576228
RNU6-818P	-0.023	0.506086094	0.756576228
RP11-503E24	-0.023	0.506085315	0.756576228
VANGL1	-0.023	0.506060749	0.756576228
RP13-13A3	-0.023	0.506056706	0.756576228
RP11-408H1	-0.023	0.505988543	0.756576228
AC093376	-0.023	0.505860202	0.756439682
ERP44	-0.023	0.505822472	0.756412423
C1orf56	-0.023	0.505650613	0.756221245
WDR61	-0.023	0.505543392	0.756132121
MIR518C	-0.023	0.505518047	0.756117958
ZFYVE16	-0.023	0.505479833	0.756084546
MIR572	-0.023	0.505466579	0.756084546

HAUS6P2	-0.023	0.505459978	0.756084546
AC008671	-0.023	0.505444857	0.756084546
MIR3178	-0.023	0.505431549	0.756084546
RIC8A	-0.023	0.505388344	0.756084546
METTL18	-0.023	0.505350982	0.756058027
NCK1	-0.023	0.505154073	0.755810915
PHOSPHO1	-0.023	0.505094901	0.755769867
LINC00939	-0.023	0.504984068	0.755651508
RP11-533N14	-0.023	0.504926131	0.755588552
C3orf70	-0.023	0.50491602	0.755588552
PRSS57	-0.023	0.504889828	0.755581708
SNORA70	-0.023	0.504819318	0.7555268
RAB6A	-0.023	0.504813068	0.7555268
OR8K1	-0.023	0.504784512	0.7555268
DNAJB4	-0.023	0.504670699	0.755447724
MT-TH	-0.023	0.504670349	0.755447724
FAM120AOS	-0.023	0.504520676	0.755361548
RP3-348I23	-0.023	0.504520061	0.755361548
IGKV2D-14	-0.023	0.504422435	0.755326475
MAP2K7	-0.023	0.504341027	0.755282524
CHEK1	-0.023	0.504337191	0.755282524
GOLGA6L11P	-0.023	0.504245812	0.755225552
LHFPL3-AS2	-0.023	0.504244564	0.755225552
CCDC96	-0.023	0.504217909	0.755225552
AL163760	-0.023	0.504166063	0.755225552
BTF3P5	-0.023	0.504139793	0.75521863
ZNF684	-0.023	0.504132817	0.75521863
RP11-381I15	-0.023	0.504079852	0.755209196
KRT8P13	-0.023	0.504073014	0.755209196
RPL18AP2	-0.023	0.50402427	0.755188093
USP28	-0.023	0.503901293	0.755027587
RP13-1032I1	-0.023	0.503891531	0.755027587
SNX18P13	-0.023	0.503821257	0.754955166
RP11-313C15	-0.023	0.503725482	0.754859151
KCNMB3	-0.023	0.503724898	0.754859151
AC133041	-0.023	0.503652788	0.754821465
OR51J1	-0.023	0.503637317	0.754821465
MIR520E	-0.023	0.503569222	0.754814985
LARGE-IT1	-0.023	0.503556642	0.754814985
NME5	-0.023	0.503501449	0.754760906
SNORD116-18	-0.023	0.503494856	0.754760906
MRPL38	-0.023	0.503449565	0.754730639
RP11-451F14	-0.023	0.503419269	0.754708978
RNU6-176P	-0.023	0.503288376	0.754584004
MIR301B	-0.023	0.503266495	0.754574952
DIAPH1	-0.023	0.503195561	0.754516103
GTF3AP2	-0.023	0.503154453	0.754478217
BAATP1	-0.023	0.503129528	0.754464595
EP300-AS1	-0.023	0.503047987	0.754366073
SUCLG2P4	-0.023	0.502987641	0.754299329
LYSMD1	-0.023	0.502982724	0.754299329
HPVC1	-0.023	0.502911252	0.754279773

RP11-885B4	-0.023	0.502781678	0.754109182
RP11-465K16	-0.023	0.502779074	0.754109182
RP11-72I2	-0.023	0.502763255	0.754109182
PCBD2	-0.023	0.502746952	0.754109182
LINC01059	-0.023	0.502707353	0.754109182
RP11-189B4	-0.023	0.502698672	0.754109182
TMEM256P2	-0.023	0.502674087	0.754109182
RP11-183E9	-0.023	0.502662322	0.754109182
RP11-398B16	-0.023	0.502602464	0.754101614
VIPR1	-0.023	0.502582956	0.754101614
CLVS1	-0.023	0.502535887	0.754096748
RNU6-775P	-0.023	0.502391028	0.753998157
CFH	-0.023	0.502326398	0.753996198
RNU6-553P	-0.023	0.50232553	0.753996198
RNU1-72P	-0.023	0.502308477	0.753996198
RP4-736I12	-0.023	0.502288513	0.753996198
UNC13B	-0.023	0.502174307	0.753934234
RNU7-66P	-0.023	0.50217279	0.753934234
ASTN2-AS1	-0.023	0.502161323	0.753934234
RP11-522B15	-0.023	0.502142987	0.753934234
RP11-4G2	-0.023	0.502124143	0.753934234
RP11-152K4	-0.023	0.502103832	0.753934234
NRAS	-0.023	0.502067126	0.753915913
SPINK2	-0.023	0.50193055	0.753734592
AC092910	-0.023	0.501924422	0.753734592
RP11-386G11	-0.023	0.501815231	0.75365647
PBX4	-0.023	0.501743919	0.753573133
RP5-925F19	-0.023	0.501721213	0.753573133
RP5-1057I20	-0.023	0.501709482	0.753573133
MED28P1	-0.023	0.501699593	0.753573133
RP11-124O11	-0.023	0.501645155	0.753573133
SLC9A7	-0.023	0.501602354	0.75355062
HNRNPA1P43	-0.023	0.501385982	0.753273089
AC121336	-0.023	0.501383789	0.753273089
CTB-162J7	-0.023	0.50133288	0.75324083
AC108519	-0.023	0.501300778	0.753216359
PDCD4-AS1	-0.023	0.501276871	0.7532042
OR52H2P	-0.023	0.501191876	0.753124009
ARRDC4	-0.023	0.501181959	0.753124009
RNU6-711P	-0.023	0.501145747	0.753123251
RNA5SP245	-0.023	0.501043094	0.753019231
SLC16A5	-0.023	0.500951847	0.752953375
RP11-332P22	-0.023	0.500908515	0.752935769
CLUHP5	-0.023	0.500805908	0.752876581
MIR1278	-0.023	0.500745079	0.752832662
BRD9P1	-0.023	0.500725407	0.75282685
TNIP1	-0.023	0.500554669	0.75266519
KRT18P7	-0.023	0.500518612	0.752634734
AL589843	-0.023	0.500513982	0.752634734
MIR4464	-0.023	0.500489998	0.752634734
BRINP1	-0.023	0.500468643	0.752634734
AC005626	-0.023	0.500457139	0.752634734

PRRC2B	-0.023	0.500380978	0.752594103
STK4-AS1	-0.023	0.500343465	0.752561447
C11orf85	-0.023	0.500298842	0.752541861
IGHVIII-22-2	-0.023	0.500264832	0.752538239
AB019440	-0.023	0.500163678	0.752452698
CCDC185	-0.023	0.500050965	0.752369307
RNU6-1328P	-0.023	0.500026654	0.752369307
RN7SKP46	-0.023	0.500008071	0.752365867
MAN2B1	-0.023	0.49998786	0.752359224
CPB2	-0.023	0.499987199	0.752359224
DNAAF5	-0.023	0.499958001	0.752359224
KRT8P32	-0.023	0.499924111	0.75235838
RPL35AP30	-0.023	0.499883936	0.75235838
CTC-250I14	-0.023	0.499875159	0.75235838
HIST2H2BE	-0.023	0.4998719	0.75235838
FAM204BP	-0.023	0.499865753	0.75235838
COQ7	-0.023	0.499830589	0.75235838
AC026403	-0.023	0.49982267	0.75235838
MIR550A2	-0.023	0.499796359	0.75235838
DYNC1I2P1	-0.023	0.499788409	0.75235838
C5orf66-AS2	-0.023	0.499786237	0.75235838
LINC00365	-0.023	0.499784049	0.75235838
CTD-2024D23	-0.023	0.499630067	0.752343773
AL357095	-0.023	0.499623406	0.752343773
TEX28	-0.023	0.499583053	0.752343773
CLPTM1	-0.023	0.499565058	0.752343773
ST18	-0.023	0.499520258	0.75233882
RP11-452D2	-0.023	0.499452519	0.752266694
MCMDC2	-0.023	0.49929648	0.752103025
AL133167	-0.023	0.499179927	0.751973862
FAM177A1P1	-0.023	0.499133399	0.751928718
RN7SL561P	-0.023	0.499120681	0.751928718
DCDC2	-0.023	0.499104046	0.751928718
CH17-228N4	-0.023	0.499047887	0.751928718
RSPH3	-0.023	0.499035609	0.751928718
AP001372	-0.023	0.499016185	0.751928718
AC020636	-0.023	0.499008195	0.751928718
TIFA	-0.023	0.499006821	0.751928718
AC005776	-0.023	0.498914504	0.751924901
LINC01585	-0.023	0.498849148	0.751904815
AC096633	-0.023	0.498787032	0.751834984
BMPR1B-AS1	-0.023	0.498773185	0.751834984
ARPP21	-0.023	0.498669859	0.751753533
TMEM62	-0.023	0.498639238	0.751753533
BTF3P9	-0.023	0.498536033	0.75168331
RP11-707E21	-0.023	0.498503224	0.75167939
RP4-555N2	-0.023	0.498470616	0.751667301
MIR4279	-0.023	0.498463348	0.751667301
RN7SKP83	-0.023	0.498355541	0.751565162
RPL7P29	-0.023	0.498314733	0.751527416
SNX18P24	-0.023	0.498262327	0.751495973
HNRNPA1P2	-0.023	0.498213277	0.751445791

ZNF415	-0.023	0.498202486	0.751445791
RP11-394J1	-0.023	0.4980085	0.751232092
FOXD4L4	-0.023	0.497963096	0.751211186
VN1R3	-0.023	0.497934834	0.751192345
AC118463	-0.023	0.497837435	0.751108965
DNAJC28	-0.023	0.49780612	0.751093331
TBC1D13	-0.023	0.497695278	0.75098959
IDH1	-0.023	0.497646443	0.750971361
CTD-2245E15	-0.023	0.497644407	0.750971361
TTC28-AS1_3	-0.023	0.497439536	0.750778071
OR4A9P	-0.023	0.497295849	0.750632568
DNAJC27-AS1	-0.023	0.497293587	0.750632568
RP11-178C3	-0.023	0.497256543	0.750632568
UNC93B1	-0.023	0.497216039	0.750607255
RP11-114H21	-0.023	0.497214514	0.750607255
AC145141	-0.023	0.497212028	0.750607255
RP13-766D20	-0.023	0.497191106	0.750607255
LINC01317	-0.023	0.497076172	0.750529193
RP11-384A12	-0.023	0.496880403	0.750290827
IQCF3	-0.023	0.496877666	0.750290827
RP11-562F9	-0.023	0.496852587	0.750290827
FXYD6P3	-0.023	0.496828564	0.750283912
C6orf99	-0.023	0.496816281	0.750283912
PSME4	-0.023	0.496748117	0.750281374
C8orf33	-0.023	0.496701061	0.750257887
AL121988	-0.023	0.496687694	0.750257887
SLC26A4-AS1	-0.023	0.496610975	0.750193191
RP11-46E17	-0.023	0.496581594	0.750193191
ALG8	-0.023	0.496543302	0.750186139
ZNF154	-0.023	0.496477231	0.750110113
RP11-17E3	-0.023	0.496469907	0.750110113
TAS2R31	-0.023	0.496453817	0.750110113
VCP	-0.023	0.496412203	0.750083248
ZNF530	-0.023	0.496339224	0.750020566
KRTAP16-1	-0.023	0.496222533	0.749915615
AC021078	-0.023	0.496182785	0.749879339
RP11-93O14	-0.023	0.496151846	0.749879339
RPSAP31	-0.023	0.496102904	0.749829996
CTC-311G1	-0.023	0.496056538	0.749783712
RP11-776A13	-0.023	0.4959969	0.749717361
NAP1L6	-0.023	0.495984986	0.749717361
SSU72P1	-0.023	0.49591261	0.749661331
RP11-16F15	-0.023	0.49572069	0.749490153
RELN	-0.023	0.495713319	0.749490153
AP001055	-0.023	0.495560156	0.749392489
OR52K3P	-0.023	0.495534378	0.749392489
AC002059	-0.023	0.495520216	0.749392489
MTURN	-0.023	0.495503661	0.749392489
RP11-16O9	-0.023	0.49549633	0.749392489
RP11-946L16	-0.023	0.495496058	0.749392489
OR4C12	-0.023	0.495437506	0.749392489
PRSS55	-0.023	0.495385712	0.749364311

RNU7-167P	-0.023	0.495370273	0.749364311
MORC1-AS1	-0.023	0.495361406	0.749364311
LINC01231	-0.023	0.495337133	0.749364311
ATRNL1	-0.023	0.495256515	0.749311669
RPL7AP66	-0.023	0.495141252	0.749184879
LINC01124	-0.023	0.495100108	0.749146424
FOXN4	-0.023	0.494870471	0.748854924
RNU6-3P	-0.023	0.494778425	0.748802416
TCAF2	-0.023	0.494688527	0.748713947
RP11-501J20	-0.023	0.494685774	0.748713947
ADAM10	-0.023	0.494670229	0.748713947
HSPA8P7	-0.023	0.494627235	0.748692557
COX6CP13	-0.023	0.494619572	0.748692557
ENPP7P4	-0.023	0.494594428	0.748690488
TMEM52	-0.023	0.494572877	0.748690488
SLC27A4	-0.023	0.49451385	0.748639896
CTD-2275D10	-0.023	0.494413932	0.748560014
CALR3	-0.023	0.494347166	0.748506516
RP5-1128N12	-0.023	0.494257634	0.74841854
RP11-478K15	-0.023	0.494172272	0.748313074
NIFK	-0.023	0.494140441	0.748299635
INHBC	-0.023	0.494025585	0.748186101
AC018902	-0.023	0.493999488	0.748170369
RP11-432B6	-0.023	0.493995161	0.748170369
AL031768	-0.023	0.493992026	0.748170369
PLCH1-AS2	-0.023	0.493955515	0.748170369
RN7SL40P	-0.023	0.493945921	0.748170369
AC104057	-0.023	0.493870426	0.748117641
CTD-2396E7	-0.023	0.493823059	0.748093478
RP11-39C10	-0.023	0.493768297	0.748063518
AC022007	-0.023	0.493734764	0.748063518
CHMP7	-0.023	0.49371683	0.748063518
GRM7-AS2	-0.023	0.493708022	0.748063518
RP11-408O19	-0.023	0.493699986	0.748063518
AP006216	-0.023	0.493680626	0.748063518
HAND1	-0.023	0.493662624	0.748063518
NLRP1	-0.023	0.493647166	0.748063518
RN7SL416P	-0.023	0.493621767	0.748063518
SLC38A6	-0.023	0.493599232	0.748063518
SESN3	-0.023	0.493561991	0.748031088
MIR4774	-0.023	0.493548005	0.748031088
RPS23P2	-0.023	0.493409843	0.747848092
AC016753	-0.023	0.493400516	0.747848092
DEPDC4	-0.023	0.493375115	0.747843054
UBE2D1	-0.023	0.493364083	0.747843054
RP11-399B17	-0.023	0.493310985	0.747817247
CTD-3116E22	-0.023	0.493308866	0.747817247
AC097465	-0.023	0.493273314	0.747807745
RP11-812D23	-0.023	0.493186773	0.747700349
DHX40P1	-0.023	0.493131677	0.747640618
AF241726	-0.023	0.493130115	0.747640618
RPS2P2	-0.023	0.493112432	0.747640618

OR1Q1	-0.023	0.493063946	0.747633129
MIR548AD	-0.023	0.493019379	0.747589353
AL645728	-0.023	0.492978531	0.747551212
PLEKHO2	-0.023	0.492923942	0.747492234
RP11-553K8	-0.023	0.492917253	0.747492234
FO393414	-0.023	0.492907549	0.747492234
ORC3	-0.023	0.492847103	0.747492234
PSMD14	-0.023	0.492711357	0.747336427
RNA5SP389	-0.023	0.492634917	0.74726808
MIR6070	-0.023	0.492634119	0.74726808
RP11-422J15	-0.023	0.492536036	0.747189483
TFAP2A-AS1	-0.023	0.492472301	0.747116592
DDAH1	-0.023	0.492463691	0.747116592
ENTPD1	-0.023	0.492454392	0.747116592
XRCC6	-0.023	0.492419951	0.747116592
IGHVII-65-1	-0.023	0.492376398	0.747090086
RNU7-105P	-0.023	0.492318082	0.7470254
NKIRAS1	-0.023	0.49222952	0.746938614
OR4A2P	-0.023	0.492189365	0.746901478
PLAC4	-0.023	0.492044809	0.746729698
C5orf42	-0.023	0.492019505	0.74671509
RP4-713A8	-0.023	0.491960308	0.746672836
RP11-27N21	-0.023	0.491952735	0.746672836
RP11-292B1	-0.023	0.491925599	0.746667745
RP11-626P14	-0.023	0.491901001	0.746654204
AC073107	-0.023	0.491858635	0.74662936
STRA13	-0.023	0.491782349	0.746560788
RP11-696F10	-0.023	0.491780043	0.746560788
TUBA1B	-0.023	0.491739675	0.746552086
MIR2681	-0.023	0.491676168	0.746503264
POU5F1P4	-0.023	0.491675913	0.746503264
CTD-2033A16	-0.023	0.4916299	0.746480609
AL359983	-0.023	0.491618857	0.746480609
RNA5SP353	-0.023	0.491611736	0.746480609
CYP2AB1P	-0.023	0.491556837	0.746464863
NUTF2P8	-0.023	0.491531235	0.746449784
C2orf83	-0.023	0.491517688	0.746449784
RN7SL201P	-0.023	0.491437206	0.746366366
ATP1B1P1	-0.023	0.491374488	0.746306931
XXbac-BPG116M5	-0.023	0.491357431	0.746304825
RP11-408B11	-0.023	0.491345576	0.746304825
C6orf165	-0.023	0.491334021	0.746304825
RP11-258B16	-0.023	0.491324112	0.746304825
AC147651	-0.023	0.491322325	0.746304825
RP11-273B20	-0.023	0.491310286	0.746304825
C5orf30	-0.023	0.491288271	0.746304825
RPS27P16	-0.023	0.491231591	0.746304825
PIGA	-0.023	0.491096699	0.746218175
RP11-111N20	-0.023	0.49108168	0.746218175
MYOCD	-0.023	0.491080094	0.746218175
RP3-514P16	-0.023	0.491065576	0.746218175
RP11-328K15	-0.023	0.491048478	0.746218175

RP11-1292F20	-0.023	0.49103989	0.746218175
CTD-2345H1	-0.023	0.49101489	0.746218175
TRAJ50	-0.023	0.491012337	0.746218175
GSTT2	-0.023	0.490998005	0.746218175
KIAA0101	-0.023	0.490893132	0.746146907
PIGPP2	-0.023	0.490764069	0.745974542
TXNL1P1	-0.023	0.490725557	0.745974542
RHOU	-0.023	0.490712986	0.745974542
VSTM2A-OT1	-0.023	0.490594138	0.745859068
RNA5SP371	-0.023	0.49053177	0.745788055
RP5-1006K12	-0.023	0.490519111	0.745788055
AC009973	-0.023	0.490513433	0.745788055
AC104451	-0.023	0.490485103	0.745788055
CTD-2537L20	-0.023	0.490477046	0.745788055
RPL7P11	-0.023	0.490471112	0.745788055
ATP6V1B1-AS1	-0.023	0.490422048	0.745787888
RP11-363E7	-0.023	0.490419465	0.745787888
TRPC5	-0.023	0.490399086	0.745787888
C17orf89	-0.023	0.490327028	0.745738636
RNU6-693P	-0.024	0.49020325	0.745645626
RP11-452J6	-0.024	0.49018797	0.745645626
TRAPPC12	-0.024	0.490101304	0.745609621
ZCCHC14	-0.024	0.490035692	0.745557434
SNORD116-6	-0.024	0.490015564	0.745550627
PYDC2	-0.024	0.489980168	0.745529811
CASP7	-0.024	0.489965277	0.745529811
RP11-80A15	-0.024	0.489850806	0.745452002
ZNF716	-0.024	0.489826653	0.745452002
RP11-944L7	-0.024	0.489810452	0.745452002
DRD5P2	-0.024	0.489729519	0.745401163
MIR4802	-0.024	0.489574618	0.745236842
APTX	-0.024	0.48954393	0.745213947
TMPRSS6	-0.024	0.489526763	0.745211632
HMGN2P47	-0.024	0.489520638	0.745211632
RPL9P31	-0.024	0.489462235	0.745184856
RP11-91K11	-0.024	0.489418733	0.745142446
CD177	-0.024	0.489387377	0.745118526
RPL9P30	-0.024	0.489327952	0.745075685
UBE2SP2	-0.024	0.489315889	0.745075685
AC003009	-0.024	0.489263657	0.745068198
ABCF2P1	-0.024	0.489203153	0.745028574
RP11-680E19	-0.024	0.489199385	0.745028574
RP11-796L2	-0.024	0.489174828	0.745028574
C15orf27	-0.024	0.489158124	0.745028574
RPL4P3	-0.024	0.489133666	0.745028574
RN7SL47P	-0.024	0.48911634	0.745028574
FAM86LP	-0.024	0.489101593	0.745028574
FBXO44	-0.024	0.489063414	0.74500634
MIR4778	-0.024	0.489015264	0.744956818
MYO5BP3	-0.024	0.488948299	0.744878631
FOXP4	-0.024	0.488888775	0.744835599
CDK5RAP3	-0.024	0.48871657	0.744597059

CTB-120L21	-0.024	0.488645721	0.744536751
AC008278	-0.024	0.488634655	0.744536751
GGA1	-0.024	0.488627393	0.744536751
ZNF570	-0.024	0.48862705	0.744536751
HIBCH	-0.024	0.488583151	0.744536751
RP11-69A21	-0.024	0.48858199	0.744536751
ATP6V0A1	-0.024	0.488557087	0.744536751
RP11-38L15	-0.024	0.488511646	0.744536751
FAM25A	-0.024	0.488426441	0.744464624
SMAD5	-0.024	0.488360258	0.74443522
SPATA31A6	-0.024	0.488336765	0.74443522
AC079804	-0.024	0.48831361	0.74443522
OR5H1	-0.024	0.488286036	0.74443522
CTB-187L3	-0.024	0.488104007	0.744218895
HSPA8P9	-0.024	0.488098761	0.744218895
RP11-540N6	-0.024	0.487888788	0.743978462
NEURL3	-0.024	0.487858093	0.743978462
WDR75	-0.024	0.487799839	0.743914279
DDTL	-0.024	0.487726856	0.743826794
RP4-646N3	-0.024	0.487726516	0.743826794
RP1-272E8	-0.024	0.487711312	0.743826794
HLA-DPB2	-0.024	0.487683572	0.743826794
RP11-234K24	-0.024	0.487673215	0.743826794
MTND2P25	-0.024	0.487595464	0.74374549
PPIAP19	-0.024	0.487548056	0.743696995
RNU6ATAC22P	-0.024	0.487520354	0.743678557
FKSG61	-0.024	0.487515551	0.743678557
LIPA	-0.024	0.487487046	0.743675386
CTA-249B10	-0.024	0.487422867	0.743601297
TRIM64FP	-0.024	0.487401085	0.743591885
VSX2	-0.024	0.487321389	0.743494115
SLC25A27	-0.024	0.487138066	0.743238232
ABHD17AP4	-0.024	0.487089719	0.743188276
NOP14-AS1	-0.024	0.48701378	0.743120024
AC104417	-0.024	0.486959765	0.743109027
RP11-77I22	-0.024	0.486911547	0.743092274
CREB3L3	-0.024	0.486769638	0.743034729
HOXB-AS1	-0.024	0.486749859	0.743034729
RP4-808O4	-0.024	0.486719515	0.743034729
AC097635	-0.024	0.486632538	0.743034729
LA16c-17H1	-0.024	0.486424818	0.742768669
AC105342	-0.024	0.486419409	0.742768669
CTC-241F20	-0.024	0.486310772	0.742676176
NELFA	-0.024	0.48625235	0.742645257
HBP1	-0.024	0.486229773	0.742637508
ZMYND10	-0.024	0.486196936	0.742628932
PTENP1	-0.024	0.486195568	0.742628932
TBCAP3	-0.024	0.486178414	0.742628932
TPMTP3	-0.024	0.486175225	0.742628932
DYX1C1	-0.024	0.48617094	0.742628932
RP11-184A2	-0.024	0.48607977	0.742575096
TBC1D3H	-0.024	0.48607045	0.742575096

EPCAM	-0.024	0.486066966	0.742575096
OR7E105P	-0.024	0.485990187	0.742509692
SMIM5	-0.024	0.485917524	0.742470129
AC097452	-0.024	0.485840917	0.742376893
RN7SKP134	-0.024	0.485763681	0.742330322
RNU1-88P	-0.024	0.485700885	0.742281994
RP11-25O10	-0.024	0.485595214	0.742191947
ARHGAP26	-0.024	0.485363403	0.742000793
MS4A13	-0.024	0.48531955	0.742000793
CTD-2325K12	-0.024	0.48527085	0.741958098
PSMD10P3	-0.024	0.485231792	0.741935845
OR7K1P	-0.024	0.485222362	0.741935845
RNU6-158P	-0.024	0.485220968	0.741935845
FAM168B	-0.024	0.48522057	0.741935845
AC073257	-0.024	0.485203813	0.741935845
REEP5	-0.024	0.485181278	0.741935845
SDAD1P2	-0.024	0.485068832	0.741863564
PRDX2	-0.024	0.485008071	0.741818279
ST13P14	-0.024	0.484955474	0.741785476
RP11-297D21	-0.024	0.484916527	0.741749725
AL161908	-0.024	0.484801237	0.741692477
GRPEL2P3	-0.024	0.484711095	0.741578391
ASAP1	-0.024	0.484560688	0.741442601
RP11-408J6	-0.024	0.484552603	0.741442601
STK33	-0.024	0.484531084	0.741442601
CTD-2526A2	-0.024	0.484507365	0.741442601
CTD-2050N2	-0.024	0.484478572	0.741442601
RP11-69M1	-0.024	0.484308067	0.741295164
MRPL19	-0.024	0.484270863	0.741262041
RNU6-1306P	-0.024	0.484237354	0.741234572
RP1-191L6	-0.024	0.48423053	0.741234572
LRRC10	-0.024	0.484032029	0.741087004
LYPLAL1	-0.024	0.483996486	0.741056408
DSCR8	-0.024	0.483969906	0.741039534
QRICH2	-0.024	0.483892081	0.741015663
RP11-672L10	-0.024	0.483796899	0.740893727
LRRC8E	-0.024	0.483741295	0.740832396
MIR6885	-0.024	0.483735647	0.740832396
IMPAD1	-0.024	0.483728333	0.740832396
KRTAP15-1	-0.024	0.483701675	0.740832396
RP5-1111A8	-0.024	0.483693589	0.740832396
RP11-300E4	-0.024	0.483680713	0.740832396
LUC7L	-0.024	0.483647494	0.740832396
CRYZP1	-0.024	0.483592156	0.740832396
LINC01581	-0.024	0.483588867	0.740832396
PIGUP1	-0.024	0.483580379	0.740832396
TRIM53AP	-0.024	0.483521096	0.740832396
SUSD4	-0.024	0.483276136	0.740691633
FAM64A	-0.024	0.483179996	0.740606237
AL157823	-0.024	0.483172493	0.740606237
NPM1P37	-0.024	0.483163474	0.740606237
MIR604	-0.024	0.483163339	0.740606237

MT-ND5	-0.024	0.483007176	0.740493877
TYRO3P	-0.024	0.482982742	0.740480252
OR12D1	-0.024	0.482901772	0.740379948
MAP2K1P1	-0.024	0.482897767	0.740379948
MTND1P11	-0.024	0.482852778	0.740376333
RNA5SP159	-0.024	0.482834861	0.740372696
HADHAP1	-0.024	0.482828562	0.740372696
GOLGA8Q	-0.024	0.482808936	0.740372696
CYP4F10P	-0.024	0.482767618	0.740341093
AK4P3	-0.024	0.482743781	0.740328631
FAM103A2P	-0.024	0.482711835	0.740328631
RP11-25C15	-0.024	0.482620981	0.740259234
PGDP1	-0.024	0.482616549	0.740259234
TDGF1P5	-0.024	0.482577837	0.740259234
GAMT	-0.024	0.482557388	0.740257054
LNX1-AS2	-0.024	0.482535466	0.740257054
RP11-33M22	-0.024	0.482379142	0.740102809
RP11-58B2	-0.024	0.482376778	0.740102809
AC023283	-0.024	0.482374949	0.740102809
OR8B10P	-0.024	0.482365602	0.740102809
MIR208B	-0.024	0.482198711	0.739945163
AL592528	-0.024	0.482189164	0.739945163
PM20D2	-0.024	0.482179674	0.739945163
RP11-2A1	-0.024	0.482079831	0.73992962
RPPH1-2P	-0.024	0.482074898	0.73992962
SCGN	-0.024	0.482056604	0.73992962
UBBP2	-0.024	0.482049024	0.73992962
DSCAS	-0.024	0.482031363	0.73992962
SSX5	-0.024	0.482020577	0.73992962
RP11-744I24	-0.024	0.481981078	0.73992112
TRIM49D2	-0.024	0.481971454	0.73992112
MIR105-1	-0.024	0.481971454	0.73992112
PTP4A1P4	-0.024	0.481936293	0.73992112
RP11-185O21	-0.024	0.481859289	0.739901098
OR7E100P	-0.024	0.481674619	0.739712929
RMDN2	-0.024	0.481670406	0.739712929
RPS6P20	-0.024	0.481616627	0.739695424
RNY3P10	-0.024	0.481529464	0.739585403
RP11-850F7	-0.024	0.481370823	0.739413281
CSRNP1	-0.024	0.481353622	0.739410707
PDCD2L	-0.024	0.481288306	0.73933422
RP5-842K16	-0.024	0.481282667	0.73933422
AC005532	-0.024	0.481159522	0.739243614
KLF2	-0.024	0.481044094	0.739125949
AC095040	-0.024	0.480968319	0.739033363
AC092933	-0.024	0.480893597	0.739002098
MAN1A2	-0.024	0.480843128	0.738960203
RNU6-549P	-0.024	0.480743925	0.738926965
RP11-545M17	-0.024	0.480675753	0.738869871
MIR4306	-0.024	0.480648085	0.738869871
PAK6	-0.024	0.480631426	0.738869871
MRPS17P3	-0.024	0.480620997	0.738869871

GK5	-0.024	0.480535817	0.738821686
RP11-739N10	-0.024	0.480505415	0.738798793
RP11-347L18	-0.024	0.480472267	0.738795528
NABP2	-0.024	0.480433517	0.738759795
RNU6-686P	-0.024	0.480296202	0.738572491
IGKV2-10	-0.024	0.480294099	0.738572491
PTGR2	-0.024	0.480174252	0.738528034
RP13-507P19	-0.024	0.480137515	0.73849538
RP11-656D10	-0.024	0.480101549	0.738463909
NDUFA5P10	-0.024	0.480067318	0.738435104
RP11-509A17	-0.024	0.480046764	0.738435104
AC024590	-0.024	0.480027365	0.738435104
LINC01495	-0.024	0.480008939	0.738435104
POU5F1P7	-0.024	0.479929369	0.738389845
CDNF	-0.024	0.47991812	0.738389845
EGLN2	-0.024	0.479879061	0.738389845
FCGR1C	-0.024	0.479870665	0.738389845
SERF1A	-0.024	0.479838735	0.738389845
TSPAN7	-0.024	0.479750689	0.738329598
RP11-616M22	-0.024	0.479707959	0.738311552
RP11-8H2	-0.024	0.479647775	0.738306057
MRPL48P1	-0.024	0.479630132	0.738306057
SDR42E2	-0.024	0.479606356	0.738298327
RP3-423B22	-0.024	0.479531177	0.738230319
RNU6-545P	-0.024	0.479513119	0.738226381
TTC12	-0.024	0.479443758	0.73815966
LINC01412	-0.024	0.479428594	0.73815966
FAM154A	-0.024	0.479362796	0.73811425
RNGTTP1	-0.024	0.479357514	0.73811425
FAM228B	-0.024	0.479173925	0.737894998
HIVEP2	-0.024	0.47914983	0.737884939
DPY19L2P4	-0.024	0.47911065	0.737869132
AC008759	-0.024	0.47908245	0.737869132
AL022574	-0.024	0.479055447	0.737869132
MIR4685	-0.024	0.479053788	0.737869132
AC006037	-0.024	0.47894825	0.737809888
RP11-308K2	-0.024	0.478918949	0.737788614
LRRC49	-0.024	0.478888907	0.737786588
RP1-21O18	-0.024	0.478855345	0.737786087
RP11-159N11	-0.024	0.478716584	0.737622084
PGAM4P1	-0.024	0.478664889	0.73758809
RP11-334L9	-0.024	0.478604828	0.737519401
RP11-401E14	-0.024	0.478588941	0.737518783
CACHD1	-0.024	0.478545651	0.737475934
ZNF425	-0.024	0.478370005	0.737324539
RP11-622A1	-0.024	0.478355393	0.737324539
MIR221	-0.024	0.478353834	0.737324539
LARP7P2	-0.024	0.478343064	0.737324539
MRFAP1	-0.024	0.478203886	0.737187781
AC008074	-0.024	0.478125914	0.737115299
OR2M1P	-0.024	0.478116864	0.737115299
WBP1LP3	-0.024	0.478093898	0.73711366

AC116614	-0.024	0.47802512	0.737031479
CTD-2195B23	-0.024	0.47801128	0.737031479
RP11-190C22	-0.024	0.477939878	0.736971625
AC008565	-0.024	0.477726016	0.736689557
MRPL22P1	-0.024	0.477703994	0.736689557
MIATNB	-0.024	0.477696231	0.736689557
RNU6-803P	-0.024	0.47764251	0.736680046
RNU2-6P	-0.024	0.477537203	0.736565334
WASF2	-0.024	0.477497722	0.736552145
RNU4-56P	-0.024	0.477468501	0.736530925
TCEB1P3	-0.024	0.477362709	0.736433532
DRG1	-0.024	0.477295195	0.736358986
RPL38P1	-0.024	0.477285935	0.736358986
SUV420H2	-0.024	0.477224423	0.736327102
RP11-399C16	-0.024	0.477142782	0.736243091
SMPD4	-0.024	0.47713402	0.736243091
CTD-2194A8	-0.024	0.477026603	0.736135373
DMRTB1	-0.024	0.476931279	0.736096147
C11orf45	-0.024	0.476897389	0.736095176
A1CF	-0.024	0.476878586	0.736095176
RNU4ATAC15P	-0.024	0.47682037	0.736079469
AL354928	-0.024	0.476807683	0.736079469
ZMPSTE24	-0.024	0.476770468	0.736079469
AC084854	-0.024	0.476733387	0.736064481
POMT1	-0.024	0.476677405	0.736049629
IL3RA	-0.024	0.476660823	0.736047889
RP11-599J14	-0.024	0.476638639	0.736037496
TMEM70	-0.024	0.476558298	0.735961156
RP11-133L14	-0.024	0.476495561	0.735935859
RP11-6O2	-0.024	0.476433794	0.735864324
ATG10-AS1	-0.024	0.47636637	0.735784047
RNA5SP109	-0.024	0.476315577	0.735753315
AC092652	-0.024	0.476281934	0.735725209
RP11-74E22	-0.024	0.476281234	0.735725209
AC005740	-0.024	0.476200059	0.735646456
A2M-AS1	-0.024	0.476113482	0.735584287
RP11-442J17	-0.024	0.475959083	0.735465033
UPF1	-0.024	0.47562056	0.735085035
AC003984	-0.024	0.475584835	0.735053672
CTD-2224J9	-0.024	0.475511024	0.734963444
AC025016	-0.024	0.475472765	0.73492816
TPTE2P6	-0.024	0.475469679	0.73492816
KATNAL1	-0.024	0.475444488	0.73492816
OR4C2P	-0.024	0.475381368	0.734882288
RP11-385N17	-0.024	0.475368598	0.734882288
RP11-72M17	-0.024	0.475334348	0.734881159
RNY4P24	-0.024	0.475332098	0.734881159
CCDC149	-0.024	0.4752296	0.734790773
RP11-147G16	-0.024	0.475214312	0.734790773
MIR4682	-0.024	0.475170286	0.734752237
AC106870	-0.024	0.475161646	0.734752237
MIR3183	-0.024	0.475100859	0.734710976

CELA2B	-0.024	0.475019831	0.734648004
PHKA1P1	-0.024	0.474996468	0.734644956
AP001255	-0.024	0.474957783	0.734632838
RP11-65E22	-0.024	0.474822305	0.734490538
CH507-254M2	-0.024	0.474743998	0.734421425
POLR1A	-0.024	0.474722662	0.734421425
NUMB	-0.024	0.474685185	0.734402005
ANKHD1-EIF4EBP3	-0.024	0.474632518	0.734344376
CH17-78J1	-0.024	0.474629835	0.734344376
PTPN5	-0.024	0.474526214	0.734227607
AC007952	-0.024	0.474460875	0.734150361
XXyac-YR12DB5	-0.024	0.474331083	0.734068773
RP11-265D19	-0.024	0.474320359	0.734068773
RP11-144C15	-0.024	0.474294242	0.734059464
HIST1H2BO	-0.024	0.474259562	0.734053498
RNA5SP68	-0.024	0.474245457	0.734053498
RNU6-682P	-0.024	0.474181685	0.733980666
RP11-544P1	-0.024	0.474149983	0.733973651
RPL10P1	-0.024	0.474112289	0.733944808
CACNA1C-IT1	-0.024	0.473901682	0.733714173
RNU6-935P	-0.024	0.473870582	0.733689874
ARAP1	-0.024	0.473809841	0.733643528
AP001205	-0.024	0.47375831	0.733611439
RIPK2	-0.024	0.473705628	0.733553711
COX6CP2	-0.024	0.473701929	0.733553711
RNU4-8P	-0.024	0.473700451	0.733553711
RP11-244C20	-0.024	0.473658613	0.733552459
RP1-106C24	-0.024	0.47354917	0.733454514
MIR1269A	-0.024	0.473471148	0.73338137
MIR320D1	-0.024	0.47336172	0.733272611
MRPS21P2	-0.024	0.473336473	0.733268156
RP11-358D14	-0.024	0.473335298	0.733268156
RNA5SP285	-0.024	0.473307498	0.733268156
RNA5SP396	-0.024	0.473305569	0.733268156
DDX28	-0.024	0.473241039	0.733263418
RP11-543D10	-0.024	0.472923117	0.732913879
C15orf56	-0.024	0.47289334	0.732891579
RP1-90L6	-0.024	0.472856039	0.732887155
RPA2	-0.024	0.472800955	0.732843789
CT45A5	-0.024	0.472743102	0.732815903
AC000362	-0.024	0.472706857	0.732793329
CYP4F8	-0.024	0.472689395	0.732790112
DYNC2H1	-0.024	0.47266756	0.732780113
MIR6793	-0.024	0.472610341	0.73273911
RP11-14K2	-0.024	0.472592591	0.732735443
SFXN5	-0.024	0.472577967	0.732735443
RP11-35P15	-0.024	0.472563737	0.732735443
SNORD12B	-0.024	0.472532699	0.732735443
AC007390	-0.024	0.472503899	0.732735443
LARP1BP2	-0.024	0.472455963	0.732714431
RPL30P6	-0.024	0.472409799	0.732666693
LDB1	-0.024	0.472397463	0.732666693

PNMA3	-0.024	0.472328989	0.732612935
BSX	-0.024	0.472273313	0.732550435
GS1-18A18	-0.024	0.472218776	0.732489697
C1orf54	-0.024	0.47220959	0.732489697
RPL12P15	-0.024	0.472195921	0.732489697
ABCA9	-0.024	0.472183835	0.732489697
ARL6	-0.024	0.472157074	0.732489412
HFM1	-0.024	0.472148608	0.732489412
KHDRBS2	-0.024	0.472109451	0.732489412
CTD-2350J17	-0.024	0.47208012	0.732489327
KLHL17	-0.024	0.472028154	0.732448928
RP13-977E11	-0.024	0.472013396	0.732448928
AC079779	-0.025	0.472006253	0.732448928
FOXD3	-0.025	0.47200211	0.732448928
PRSS51	-0.025	0.471918137	0.732425848
LINC00670	-0.025	0.471852596	0.732351026
AC144634	-0.025	0.471844925	0.732351026
RN7SL108P	-0.025	0.471774681	0.732325557
AC015923	-0.025	0.471759256	0.732325481
OTUD7B	-0.025	0.471749173	0.732325481
PRAMEF26	-0.025	0.471739735	0.732325481
RP11-667F9	-0.025	0.47170417	0.732325481
IBA57-AS1	-0.025	0.471609884	0.732284548
RNU4-87P	-0.025	0.471609741	0.732284548
RP5-1092L12	-0.025	0.471555643	0.732284548
AC013267	-0.025	0.471516826	0.73225942
RNU6-828P	-0.025	0.471273782	0.731953577
MTCYBP4	-0.025	0.47118992	0.73187106
RN7SL507P	-0.025	0.471127789	0.731838184
RP11-304C16	-0.025	0.471121639	0.731838184
MIR4513	-0.025	0.471121042	0.731838184
SCP2	-0.025	0.471101709	0.731838184
RP11-71J4	-0.025	0.471031954	0.731816633
C3orf58	-0.025	0.470998833	0.731789046
RP11-13E1	-0.025	0.470926456	0.731700463
RP13-57D9	-0.025	0.470914413	0.731700463
LINC01111	-0.025	0.470907137	0.731700463
BNIP3P28	-0.025	0.47089375	0.731700463
TAS2R8	-0.025	0.470863932	0.7316988
NPM1	-0.025	0.470828072	0.731666947
OR52B6	-0.025	0.470810912	0.731666947
FILIP1	-0.025	0.470772616	0.731666947
DUX4L19	-0.025	0.470682035	0.731559354
RP11-123K3	-0.025	0.470598595	0.731477408
RNU6-246P	-0.025	0.470465995	0.731366777
RP11-793H13	-0.025	0.470429538	0.731356411
AC017084	-0.025	0.470424681	0.731356411
MIR4708	-0.025	0.470384106	0.73133496
TAF1A-AS1	-0.025	0.470353952	0.731311951
TPT1P9	-0.025	0.470350593	0.731311951
STX3	-0.025	0.470293152	0.731265164
AC002551	-0.025	0.470286502	0.731265164

LRRC63	-0.025	0.47028027	0.731265164
IFNA22P	-0.025	0.470248145	0.731265164
DOCK1	-0.025	0.470173245	0.731221954
LINC00449	-0.025	0.470143502	0.731199575
OR4C15	-0.025	0.470123663	0.731192597
DEAR	-0.025	0.469952629	0.730998199
RNU1-143P	-0.025	0.469925908	0.730983153
MAPK8IP3	-0.025	0.469924414	0.730983153
SRRM4	-0.025	0.469915459	0.730983153
RAB26	-0.025	0.469896547	0.730983153
TMEM235	-0.025	0.469821018	0.730981048
SCGB1C2	-0.025	0.469710479	0.730891187
LINC01032	-0.025	0.469631979	0.730833598
ROGDI	-0.025	0.469450797	0.730694895
RP6-109B7	-0.025	0.469435025	0.730694227
AL139398	-0.025	0.469298023	0.730504851
AL162759	-0.025	0.469188807	0.730406462
AP000781	-0.025	0.469039568	0.730245742
AC024162	-0.025	0.468967614	0.730157586
FASTKD5	-0.025	0.468916406	0.730125594
SLC35E1	-0.025	0.468793339	0.73000557
PDE6D	-0.025	0.468772885	0.729997587
RNU6-102P	-0.025	0.468702275	0.729935362
RP3-417L20	-0.025	0.468693098	0.729935362
RP11-1080G15	-0.025	0.468547933	0.729790451
RPS6P21	-0.025	0.468539409	0.729790451
AC000099	-0.025	0.46852961	0.729790451
RNU1-82P	-0.025	0.468515601	0.729790451
RP11-277B15	-0.025	0.468398525	0.729662415
LLNLR-268E12	-0.025	0.46837084	0.729662415
RP11-54C4	-0.025	0.46831566	0.729619559
ST6GALNAC3	-0.025	0.468247298	0.729536918
RN7SL277P	-0.025	0.468206275	0.729496868
RPL19P14	-0.025	0.468150515	0.729481583
LINC00314	-0.025	0.468133121	0.729478346
SCARNA23	-0.025	0.468080984	0.729442567
RP11-121M22	-0.025	0.468064684	0.729442567
MTND4P20	-0.025	0.468017807	0.729401323
LLNLF-18A12	-0.025	0.467993096	0.729401323
RP11-310E22	-0.025	0.467926436	0.729323309
RP13-238N7	-0.025	0.467791815	0.729185075
AC090324	-0.025	0.467751322	0.729145819
PRSS3P4	-0.025	0.467748755	0.729145819
RP11-354A14	-0.025	0.467743461	0.729145819
OR5P2	-0.025	0.467735091	0.729145819
RP11-149P24	-0.025	0.46762941	0.729075092
RP11-154B12	-0.025	0.467555051	0.729030755
RNU2-44P	-0.025	0.467539388	0.7290302
GLIDR	-0.025	0.46753854	0.7290302
GOLGA8H	-0.025	0.467516577	0.7290302
CTD-2507G9	-0.025	0.467496171	0.7290302
AC011933	-0.025	0.467485935	0.7290302

COX7CP1	-0.025	0.467284014	0.728847601
AC007036	-0.025	0.467260007	0.728847601
MT-TW	-0.025	0.46719865	0.728843203
SPINK5	-0.025	0.46719039	0.728843203
TAPBPL	-0.025	0.467083988	0.728749561
RNU6-341P	-0.025	0.467033789	0.728700748
XXbac-BPG118E17	-0.025	0.467010009	0.728700748
DKFZp313A047	-0.025	0.46697301	0.728671895
RORA-AS2	-0.025	0.466928633	0.728626519
SLC17A2	-0.025	0.466900008	0.728605724
AAAS	-0.025	0.466884572	0.728605508
CTD-2561J22	-0.025	0.466740392	0.728475984
RIOK1	-0.025	0.466727347	0.728475984
SDF2L1	-0.025	0.466719319	0.728475984
LRRC61	-0.025	0.466718985	0.728475984
RPS28P8	-0.025	0.466616367	0.72841695
CAMK2N1	-0.025	0.466606605	0.72841695
TRIM49B	-0.025	0.466573156	0.72840593
AL513547	-0.025	0.466546725	0.72840593
KCNK17	-0.025	0.466480154	0.728356236
RP11-286H14	-0.025	0.466451102	0.728341933
RP11-84C10	-0.025	0.466450427	0.728341933
SPATA9	-0.025	0.466438063	0.728341933
MEMO1P3	-0.025	0.466390436	0.728341933
RP11-433P17	-0.025	0.466341091	0.728341933
RP4-586O15	-0.025	0.466299553	0.728313017
MIR1471	-0.025	0.466239761	0.728267393
AC073326	-0.025	0.466222174	0.728263808
FAM8A6P	-0.025	0.466205501	0.728261648
RP3-331H24	-0.025	0.466188656	0.728261648
RP11-544I20	-0.025	0.466105591	0.728177228
HLA-DQB3	-0.025	0.466064609	0.728150889
OR5M10	-0.025	0.466056417	0.728150889
HMGN2P40	-0.025	0.465945308	0.728022347
PLCB4	-0.025	0.465788957	0.727801931
AL603766	-0.025	0.465742909	0.727753856
RP11-462L8	-0.025	0.465742174	0.727753856
AC010633	-0.025	0.465705413	0.727743018
AGFG1	-0.025	0.465652649	0.727684441
CAPN9	-0.025	0.465647304	0.727684441
AC129778	-0.025	0.465563988	0.727641389
EIF5P2	-0.025	0.46553583	0.727621258
RP11-408A13	-0.025	0.465525715	0.727621258
RP3-365I19	-0.025	0.465400587	0.727481498
RP11-324H9	-0.025	0.465400129	0.727481498
RP11-24D15	-0.025	0.465302023	0.727399049
TRAV28	-0.025	0.46528845	0.727399049
RP3-400B16	-0.025	0.465251708	0.727368143
AC100828	-0.025	0.465141306	0.727267161
RPL7P54	-0.025	0.464996893	0.727160724
RP11-339B9	-0.025	0.464964996	0.727134718
CMC4	-0.025	0.464916922	0.727083412

KRT8P30	-0.025	0.46490468	0.727083412
RDH11	-0.025	0.464885991	0.727083412
RP11-203L2	-0.025	0.464883992	0.727083412
FOXN1	-0.025	0.464869346	0.727083412
RP11-286N3	-0.025	0.464818252	0.727083412
RNA5SP337	-0.025	0.464734368	0.727036649
RN7SL835P	-0.025	0.464607428	0.726861938
RP11-894J14	-0.025	0.464575321	0.726835583
NOXO1	-0.025	0.464563172	0.726835583
C18orf15	-0.025	0.464461003	0.726728348
ABCA3	-0.025	0.464457557	0.726728348
EEF1A1P17	-0.025	0.464387074	0.726660419
TCEB1P32	-0.025	0.464385364	0.726660419
MTND2P2	-0.025	0.464379098	0.726660419
RN7SKP103	-0.025	0.464354183	0.726660419
DDI1	-0.025	0.464306181	0.72665321
DLG5	-0.025	0.464280775	0.726637325
ARL5C	-0.025	0.464241341	0.726599486
SKA2P1	-0.025	0.464231714	0.726599486
RPL36P16	-0.025	0.464173153	0.726599486
SLC25A41	-0.025	0.464135604	0.726599486
C14orf39	-0.025	0.464088918	0.726551932
PLA2G4D	-0.025	0.464028645	0.726542475
RP11-128I7	-0.025	0.464008349	0.726542475
AC137723	-0.025	0.464005881	0.726542475
SLC7A2	-0.025	0.463968995	0.726542475
RGPD1	-0.025	0.463967818	0.726542475
PRRC1	-0.025	0.463931656	0.726542475
GRAPL	-0.025	0.463915437	0.726542475
XRCC3	-0.025	0.463863984	0.726534134
FKBP5	-0.025	0.463861984	0.726534134
SYS1-DBNDD2	-0.025	0.463785317	0.726458702
PFN1P1	-0.025	0.46376861	0.726456424
RBM34	-0.025	0.463698992	0.726395155
FABP5P2	-0.025	0.46366009	0.726358105
GLRX3P2	-0.025	0.463528359	0.726199512
RP11-800A18	-0.025	0.463482	0.726153626
RNU6ATAC38P	-0.025	0.463474622	0.726153626
DDX19B	-0.025	0.463473723	0.726153626
RP11-1094H24	-0.025	0.463421645	0.726153626
RP11-777B9	-0.025	0.463420801	0.726153626
ALOX5AP	-0.025	0.463371105	0.726153626
RP11-199F6	-0.025	0.463310836	0.726153626
ADAD1	-0.025	0.463286321	0.726153626
RN7SL79P	-0.025	0.463236847	0.726148877
RHPN1-AS1	-0.025	0.463218202	0.72614355
MIR5680	-0.025	0.463207976	0.72614355
SRCAP	-0.025	0.463165576	0.726132752
OR13C4	-0.025	0.463131991	0.726104
RNA5SP238	-0.025	0.463129002	0.726104
RP11-456A14	-0.025	0.462956318	0.725971967
RN7SKP73	-0.025	0.462926379	0.72594892

RN7SL586P	-0.025	0.462910705	0.725948243
CTD-2026A21	-0.025	0.462906942	0.725948243
MRPS35	-0.025	0.462900494	0.725948243
CTC-250P20	-0.025	0.462885195	0.725948243
RNU6-527P	-0.025	0.462866919	0.725948243
MIR340	-0.025	0.462632043	0.725750208
CPXM2	-0.025	0.462583573	0.725698073
RNU7-50P	-0.025	0.462548362	0.725690643
EEF1A1P42	-0.025	0.46254776	0.725690643
PHF20	-0.025	0.46243514	0.725560811
DPPA2P2	-0.025	0.462342191	0.725500096
FTH1P18	-0.025	0.462200709	0.725398556
ZNF559-ZNF177	-0.025	0.462146755	0.725347289
CCDC90B	-0.025	0.46214135	0.725347289
NUTM2D	-0.025	0.462130869	0.725347289
RP11-439H13	-0.025	0.462126433	0.725347289
RP11-574E24	-0.025	0.462121566	0.725347289
AJ009632	-0.025	0.462089184	0.725347289
UBQLN4P2	-0.025	0.462039698	0.725322677
SLC6A4	-0.025	0.461895548	0.725168093
NEPNP	-0.025	0.46181207	0.725132648
NR4A1	-0.025	0.461762536	0.72510331
MIR548W	-0.025	0.461722707	0.725101416
C2orf16	-0.025	0.461720974	0.725101416
DBIP2	-0.025	0.461656592	0.725073412
HMGB3P24	-0.025	0.461620526	0.72504702
RABL2A	-0.025	0.461494246	0.724920398
RP11-128B16	-0.025	0.461488348	0.724920398
OR7A19P	-0.025	0.461480923	0.724920398
CCNI2	-0.025	0.461474302	0.724920398
AC005307	-0.025	0.461443096	0.724920398
RP11-452L6	-0.025	0.461426439	0.724920398
ZNF962P	-0.025	0.461384454	0.724895457
RP11-476C8	-0.025	0.461344492	0.724885334
BNIP3P35	-0.025	0.461336589	0.724885334
CABS1	-0.025	0.461292899	0.724867099
ADCY7	-0.025	0.461256282	0.72485739
ERICH3-AS1	-0.025	0.461249368	0.72485739
RP11-490H24	-0.025	0.461234872	0.72485739
MIPEPP2	-0.025	0.461211174	0.72485739
TRBV12-1	-0.025	0.461175129	0.72485739
AC106875	-0.025	0.46114449	0.724849129
ATP5LP6	-0.025	0.461087245	0.724806992
TRAPPC2P3	-0.025	0.461053898	0.724778493
RP11-624A4	-0.025	0.461013961	0.724739632
TRMT11	-0.025	0.461009748	0.724739632
RAB17	-0.025	0.460961912	0.724705651
RORC	-0.025	0.460955601	0.724705651
SNORA80B	-0.025	0.46091385	0.724677932
OR5B1P	-0.025	0.460881991	0.724675687
RNU1-105P	-0.025	0.460879624	0.724675687
SYNE1-AS1	-0.025	0.460852014	0.724675687

PSMD7P1	-0.025	0.460765762	0.724636474
TRIM64DP	-0.025	0.460708065	0.724593587
LINC00526	-0.025	0.460693217	0.724593587
RP3-352A20	-0.025	0.460610518	0.724495022
SAR1P1	-0.025	0.460568964	0.724470508
RNU2-9P	-0.025	0.460495127	0.724402214
AL031229	-0.025	0.460488634	0.724402214
RP11-61F5	-0.025	0.460451202	0.724380967
AC004062	-0.025	0.460370214	0.724373193
SERHL2	-0.025	0.460321705	0.724320796
AC091069	-0.025	0.460313279	0.724320796
EIF3S5P1	-0.025	0.460298575	0.724320796
CT83	-0.025	0.460178303	0.724214786
RN7SL12P	-0.025	0.460057653	0.724049678
CTD-2312P21	-0.025	0.460056918	0.724049678
RP11-344A7	-0.025	0.459964174	0.723973486
RBBP8P1	-0.025	0.459908822	0.723928819
MIR6816	-0.025	0.459839529	0.723849063
IL36A	-0.025	0.459812755	0.723830841
RP11-231N9	-0.025	0.459731522	0.723750809
RP11-11C20	-0.025	0.459697708	0.723733834
RPS18P13	-0.025	0.45966209	0.723733834
AC009955	-0.025	0.459643046	0.723731134
FITM1	-0.025	0.459623581	0.723724413
DPAGT1	-0.025	0.459600408	0.723711851
RAB1B	-0.025	0.459492633	0.723626638
SNPH	-0.025	0.459473069	0.723626638
RPL6P5	-0.025	0.459461768	0.723626638
NECAP2	-0.025	0.459281766	0.723449288
ROCK2	-0.025	0.459267842	0.723449288
AC097483	-0.025	0.459263547	0.723449288
RP11-1006G14	-0.025	0.459219443	0.723449288
CRYZ	-0.025	0.459199224	0.723449288
C2orf27AP2	-0.025	0.459177068	0.723449288
AC116407	-0.025	0.45914918	0.723449288
RP11-778H2	-0.025	0.459092642	0.723449288
CMYA5	-0.025	0.459077913	0.723449288
MIR3139	-0.025	0.459022091	0.723399131
RPL21P134	-0.025	0.458899195	0.72330504
FBXO18	-0.025	0.458898962	0.72330504
AL512791	-0.025	0.458856411	0.72330504
AL139328	-0.025	0.458817152	0.723291556
IFNNP1	-0.025	0.458750423	0.72322958
AC009236	-0.025	0.458703149	0.72320759
RPS15AP7	-0.025	0.458667627	0.723199466
AVPR2	-0.025	0.458568998	0.723093484
CYP4F2	-0.025	0.458534585	0.723085448
CSPG4P13	-0.025	0.458398247	0.722969802
HNRNPA1P10	-0.025	0.458388503	0.722969802
RNU6-944P	-0.025	0.458302261	0.722958458
CPLX3	-0.025	0.458296535	0.722958458
SDCCAG8	-0.025	0.458289826	0.722958458

NBPF10	-0.025	0.458264662	0.722958458
HIGD1AP14	-0.025	0.458254354	0.722958458
DROSHA	-0.025	0.458229567	0.722958458
VRK1	-0.025	0.458192486	0.722958458
RN7SKP188	-0.025	0.458056512	0.722786254
RP11-473J6	-0.025	0.458038422	0.722781656
CTD-2013N24	-0.025	0.457995193	0.722777605
PACS1	-0.025	0.457944553	0.722752819
HSPD1P18	-0.025	0.457889835	0.722714791
KDM6B	-0.025	0.457879024	0.722714791
RPL32P34	-0.025	0.457861979	0.722714791
RP11-683O4	-0.025	0.457835574	0.72270101
SNX2P2	-0.025	0.457777497	0.722660027
CDK2AP2	-0.025	0.457716547	0.722608923
HSPE1P3	-0.025	0.457714324	0.722608923
RNU6-401P	-0.025	0.457686912	0.722608923
GPN2	-0.025	0.457649051	0.722598175
RP11-814M22	-0.025	0.457593475	0.722534377
PHBP3	-0.025	0.457568325	0.72251862
UBA52P9	-0.025	0.457537742	0.722517472
RP5-1018K9	-0.025	0.457526358	0.722517472
LINC01447	-0.025	0.457471584	0.722461672
PABPC1P5	-0.025	0.457384179	0.7223973
IGLVVII-41-1	-0.025	0.457327191	0.722377358
VENTXP4	-0.025	0.457281711	0.722329477
RN7SL42P	-0.025	0.457249813	0.722303046
AL391380	-0.025	0.457192687	0.72223676
TUBA3FP	-0.025	0.457182448	0.72223676
SYNJ2-IT1	-0.025	0.457179357	0.72223676
SUSD3	-0.025	0.457165823	0.72223676
EDEM2	-0.025	0.457156565	0.72223676
HNF4A-AS1	-0.025	0.45714685	0.72223676
RP11-118F19	-0.025	0.457125355	0.72223676
CERS3-AS1	-0.025	0.457079129	0.72223676
CCDC58P2	-0.025	0.457055184	0.722235144
CICP23	-0.025	0.457051372	0.722235144
MIR4532	-0.025	0.456964863	0.7221594
RP11-779P15	-0.025	0.456962104	0.7221594
CDK20	-0.025	0.456919854	0.722141095
CTC-480C2	-0.025	0.456883542	0.722141095
TPT1P3	-0.025	0.456867033	0.722141095
KCNC4-AS1	-0.025	0.456819851	0.722102856
RP11-443I9	-0.025	0.456817691	0.722102856
RP11-694D5	-0.025	0.456803471	0.722102856
MRGPRD	-0.025	0.456765106	0.722088299
MTND4P12	-0.025	0.456728156	0.722077738
HIST1H1PS1	-0.025	0.456714697	0.722077738
WDFY3-AS2	-0.025	0.456705353	0.722077738
DRD3	-0.025	0.456698539	0.722077738
REM2	-0.025	0.456654463	0.722077738
RP11-1024P17	-0.025	0.456636619	0.722077738
RNU5E-7P	-0.025	0.456611437	0.722077738

RNU6-780P	-0.025	0.456542798	0.722077738
CACYBP	-0.025	0.456491239	0.722062711
GPBAR1	-0.025	0.456339609	0.721894543
SENP3-EIF4A1	-0.025	0.456336545	0.721894543
RNU6-1097P	-0.025	0.456275678	0.721841612
RP11-756K15	-0.025	0.456241678	0.721811798
SNX4	-0.025	0.456141772	0.721701687
RNU6-470P	-0.025	0.456030009	0.721548827
RP11-549B18	-0.025	0.456021863	0.721548827
PCBP1	-0.025	0.456020584	0.721548827
AC016723	-0.025	0.455935599	0.721471353
RP11-19G24	-0.025	0.455880999	0.721432892
RP11-160C18	-0.025	0.455854429	0.721414816
MYO5B	-0.025	0.455831408	0.721402355
AC096574	-0.025	0.455800305	0.721377102
TM9SF3	-0.025	0.455762748	0.721341632
EEF1A1P13	-0.025	0.455723139	0.721341632
PPP1R17	-0.025	0.455723063	0.721341632
PRR7-AS1	-0.025	0.455722348	0.721341632
AC010608	-0.025	0.455687044	0.721341632
RN7SL44P	-0.025	0.455676076	0.721341632
CKBP1	-0.025	0.455669583	0.721341632
OR11G2	-0.025	0.45566082	0.721341632
KIAA0825	-0.025	0.455626765	0.721341632
PTX4	-0.025	0.455609926	0.721341632
PLRG1	-0.025	0.455579242	0.721341632
ZNF92P2	-0.025	0.455469783	0.721213491
DOCK7	-0.025	0.45544461	0.721197607
POTEE	-0.025	0.455418041	0.721197607
GOT1L1	-0.025	0.455412052	0.721197607
UBQLNL	-0.025	0.4551948	0.720921878
SERP2	-0.025	0.455128905	0.720846252
TMEM26-AS1	-0.025	0.455058446	0.720801803
ZFYVE9	-0.025	0.454979178	0.720724184
RP11-740N7	-0.025	0.454967032	0.720724184
FERP1	-0.025	0.454925347	0.720698231
RNU6-1059P	-0.025	0.454912381	0.720698231
KDM4E	-0.025	0.454882458	0.72069456
UBE3D	-0.025	0.454804092	0.720662558
RP11-313P13	-0.025	0.454732385	0.720601467
RP13-317D12	-0.025	0.454722907	0.720601467
SPDYE5	-0.025	0.454658709	0.720576031
RP11-618P13	-0.025	0.454657121	0.720576031
CTC-398G3	-0.025	0.45456608	0.720489729
RNA5SP279	-0.025	0.45451635	0.720446308
FAM27L	-0.025	0.454507273	0.720446308
GALC	-0.025	0.454457989	0.720446308
AC007773	-0.025	0.454412182	0.720401072
BOLA3P2	-0.025	0.454367653	0.720354456
ZNF296	-0.025	0.454178577	0.720136951
PHKBP1	-0.025	0.45414872	0.720127217
HUS1B	-0.026	0.454074434	0.720057371

RN7SKP24	-0.026	0.454042724	0.720036547
ATP4B	-0.026	0.453986456	0.720013751
NCEH1	-0.026	0.453911447	0.719918761
MTFR1	-0.026	0.453790921	0.719775543
TCF12	-0.026	0.453752397	0.719746735
ZNF436-AS1	-0.026	0.453744824	0.719746735
GOLGA6L22	-0.026	0.453722459	0.719746735
LRP2	-0.026	0.453703329	0.719746735
CTA-268H5	-0.026	0.453573437	0.719718211
OR5M6P	-0.026	0.453532551	0.719677311
RP11-687E1	-0.026	0.453497867	0.71965007
RP11-305L7	-0.026	0.453489029	0.71965007
BMS1P16	-0.026	0.453485589	0.71965007
RN7SL612P	-0.026	0.453480782	0.71965007
AC093084	-0.026	0.453357982	0.719592101
RP11-337C18	-0.026	0.453318873	0.719554006
ANKRD22	-0.026	0.453208965	0.719475464
TP53TG3C	-0.026	0.453131702	0.719424748
RPS23P10	-0.026	0.453112209	0.719417782
SNORD116-23	-0.026	0.453109571	0.719417782
FATE1	-0.026	0.453086186	0.719417782
CTB-52I2	-0.026	0.45306156	0.71941778
AC069285	-0.026	0.453016386	0.71941778
ECEL1P1	-0.026	0.452975377	0.719392386
U2AF1L4	-0.026	0.45295334	0.719381377
MNX1-AS1	-0.026	0.452940508	0.719381377
SPRTN	-0.026	0.452917816	0.719372935
HHATL-AS1	-0.026	0.452907558	0.719372935
RP11-717A5	-0.026	0.452898699	0.719372935
BX682235	-0.026	0.452870575	0.719369873
NEURL1-AS1	-0.026	0.452866739	0.719369873
MIR548AR	-0.026	0.452834754	0.719360956
RP11-155G15	-0.026	0.452813505	0.719360956
FAM169A	-0.026	0.452767512	0.719326116
SAMD7	-0.026	0.452750996	0.719326116
RP11-608O8	-0.026	0.45268623	0.71926896
RP11-59J16	-0.026	0.452668385	0.719264601
RP3-377O6	-0.026	0.452604053	0.71921037
HMX3	-0.026	0.452583666	0.71921037
TPI1P2	-0.026	0.452562329	0.71921037
RP11-139H14	-0.026	0.452557458	0.71921037
RN7SL523P	-0.026	0.452536879	0.71921037
AC091856	-0.026	0.452515366	0.71921037
LINC00841	-0.026	0.452430974	0.719175289
GSTP1P1	-0.026	0.452425526	0.719175289
COL6A6	-0.026	0.452416029	0.719175289
RP11-814P5	-0.026	0.452376575	0.719175289
AC092535	-0.026	0.452199449	0.718999237
ADIPOR2	-0.026	0.452148177	0.71896572
CYSRT1	-0.026	0.452130104	0.718960985
ATXN7L3	-0.026	0.452089687	0.718920719
HSPE1P2	-0.026	0.452049164	0.718920719

MIR548N	-0.026	0.451912535	0.718783005
ACACA	-0.026	0.451797302	0.718671724
CTD-2050I18	-0.026	0.451796089	0.718671724
CTD-2335O3	-0.026	0.451690323	0.718573553
RP1-90J20	-0.026	0.451649802	0.718533089
AL022578	-0.026	0.451559481	0.718437395
POLR1D	-0.026	0.451484994	0.718342881
OR6C69P	-0.026	0.45147293	0.718342881
SPDYA	-0.026	0.451426224	0.718297366
RRP15	-0.026	0.451420314	0.718297366
RP5-994D16	-0.026	0.451343822	0.718214241
HSD17B11	-0.026	0.45124436	0.718103955
TRAF3IP2-AS1	-0.026	0.451234513	0.718103955
AC011477	-0.026	0.451227415	0.718103955
LINC01478	-0.026	0.451172245	0.718103955
RP11-385E5	-0.026	0.451065779	0.718011693
MIR5010	-0.026	0.451033393	0.718004558
AC019109	-0.026	0.451013021	0.717999705
AL357874	-0.026	0.450960243	0.717939681
SORCS1	-0.026	0.45095265	0.717939681
RP13-130D24	-0.026	0.450946325	0.717939681
OSTM1	-0.026	0.450894865	0.717923881
ATP5H	-0.026	0.450875508	0.717923881
RN7SL57P	-0.026	0.450832318	0.717923881
AC073934	-0.026	0.450823879	0.717923881
NIPSNAP1	-0.026	0.450822032	0.717923881
TEX12	-0.026	0.450797485	0.717923881
CASC11	-0.026	0.450788476	0.717923881
RNU7-157P	-0.026	0.45071176	0.71790405
MERTK	-0.026	0.450682618	0.71788164
MTND6P3	-0.026	0.450666898	0.71788061
CBX5P1	-0.026	0.45065002	0.717877734
RP11-16P20	-0.026	0.450639695	0.717877734
RNU6-672P	-0.026	0.450483991	0.717685267
RP11-600L4	-0.026	0.450343982	0.717486212
ZNF355P	-0.026	0.450298407	0.717437603
WDR26	-0.026	0.450268782	0.717437603
RP11-661G16	-0.026	0.450202012	0.717404016
KRT19P1	-0.026	0.450194399	0.717404016
CALCOCO1	-0.026	0.450177322	0.717404016
PCGF2	-0.026	0.450165521	0.717404016
LINC01125	-0.026	0.450136348	0.717404016
RBMY1J	-0.026	0.450107382	0.717397238
AC069082	-0.026	0.450107382	0.717397238
ZSCAN22	-0.026	0.45010226	0.717397238
NIPA2P2	-0.026	0.449941342	0.717252612
FLJ42969	-0.026	0.449804833	0.717059003
RP11-70F11	-0.026	0.449702626	0.716920065
EIF4E2P1	-0.026	0.449665321	0.716897431
DAP3	-0.026	0.449589143	0.716844867
ANKRD20A7P	-0.026	0.449579714	0.716844867
NOB1	-0.026	0.449542304	0.716844867

CTD-2582D11	-0.026	0.449448889	0.716731475
RP11-27P7	-0.026	0.44943687	0.716731475
HCG11	-0.026	0.449278027	0.716530977
LINC00176	-0.026	0.449218765	0.716460455
DPY19L3	-0.026	0.449170638	0.716407688
LOXL1-AS1	-0.026	0.449136264	0.716376853
GAPDHP55	-0.026	0.449105515	0.716375791
FLJ35934	-0.026	0.44909708	0.716375791
USP9YP1	-0.026	0.449067079	0.716362465
AC116035	-0.026	0.448903844	0.716150043
SNRPEP10	-0.026	0.44889466	0.716150043
AC010139	-0.026	0.448772498	0.715988468
RP11-193P11	-0.026	0.448691327	0.715882947
FOXD4L5	-0.026	0.448589278	0.715828802
RP3-323A16	-0.026	0.448582349	0.715828802
OR2T32P	-0.026	0.448394644	0.715601371
KF511026	-0.026	0.448319029	0.715528648
MTND6P1	-0.026	0.448308183	0.715528648
CLUAP1	-0.026	0.448238474	0.71547201
RP11-31K23	-0.026	0.448232454	0.71547201
RP11-206F17	-0.026	0.448229634	0.71547201
SLC29A4P2	-0.026	0.448208765	0.71547201
MIR5700	-0.026	0.448144547	0.715465955
PROKR2	-0.026	0.448131033	0.715465955
RP11-654F3	-0.026	0.448128615	0.715465955
OR51A4	-0.026	0.448128447	0.715465955
TTLL8	-0.026	0.448026202	0.71539692
RP11-108N13	-0.026	0.447958827	0.715313319
FMNL3	-0.026	0.447895856	0.715278327
RNF115	-0.026	0.447863171	0.715256497
RN7SL832P	-0.026	0.447785713	0.715156774
RP11-22A3	-0.026	0.447779655	0.715156774
AC009276	-0.026	0.447760262	0.715156774
NANOGP4	-0.026	0.447726078	0.715156774
OR5AU1	-0.026	0.447724288	0.715156774
RN7SL683P	-0.026	0.447703105	0.715156774
NCOR1P2	-0.026	0.447681625	0.715156774
STAC3	-0.026	0.447676893	0.715156774
CAPN10	-0.026	0.447660555	0.715156774
AC022681	-0.026	0.44763688	0.715156774
EFCAB3	-0.026	0.447588	0.715156774
OR51F4P	-0.026	0.447503118	0.715113106
BTF3L4P2	-0.026	0.447430224	0.71508167
AC005757	-0.026	0.447318328	0.714961743
RNF8	-0.026	0.447300223	0.714957592
RP11-701I24	-0.026	0.447274922	0.714957592
TRMT61B	-0.026	0.4469554	0.714597497
RP11-655H13	-0.026	0.446937183	0.714597497
OR52P1P	-0.026	0.446895748	0.714574087
ZBTB38	-0.026	0.44688554	0.714574087
RP11-191H23	-0.026	0.446879034	0.714574087
CRY2	-0.026	0.446848327	0.714574087

OR6W1P	-0.026	0.44676858	0.714538674
RP11-248J18	-0.026	0.446761503	0.714538674
APC2	-0.026	0.446740615	0.714538674
RNU6-272P	-0.026	0.446740114	0.714538674
RP11-245C17	-0.026	0.446689683	0.714532462
AIF1L	-0.026	0.446681543	0.714532462
MAPK12	-0.026	0.446670121	0.714532462
RP11-266K22	-0.026	0.446650683	0.714532462
RP11-473C19	-0.026	0.446537828	0.714433523
ZCCHC5	-0.026	0.44647856	0.714386695
RP11-492O8	-0.026	0.446469865	0.714386695
KRTAP5-7	-0.026	0.446440572	0.714386695
RNU7-152P	-0.026	0.446410864	0.714374377
LIN7B	-0.026	0.446343189	0.714314081
NUTF2P4	-0.026	0.446323982	0.714307345
RP11-187C18	-0.026	0.446215792	0.714158194
RP11-1145L24	-0.026	0.446160981	0.714128818
WTAP	-0.026	0.446011828	0.713999709
TMEM167B	-0.026	0.445956397	0.713934969
RPL5P29	-0.026	0.445808423	0.713806826
FLVCR2	-0.026	0.445788173	0.713806826
ERVFRD-1	-0.026	0.445770283	0.713806826
MRPL1	-0.026	0.445656814	0.713671264
AL389915	-0.026	0.445620143	0.713636535
RP4-704D21	-0.026	0.44554875	0.713586452
PPP1R26	-0.026	0.44537168	0.713407533
AC068312	-0.026	0.445358986	0.713407533
RP11-787P24	-0.026	0.44528882	0.713393791
TUFMP1	-0.026	0.445257034	0.713393791
WHAMMP2	-0.026	0.445126711	0.71329735
AL137229	-0.026	0.44510852	0.713296886
OR5V1	-0.026	0.44497883	0.713182024
EIF4E1B	-0.026	0.444888589	0.713088404
RPL21P4	-0.026	0.444887482	0.713088404
SMARCD2	-0.026	0.444887029	0.713088404
ZNF574	-0.026	0.444869121	0.713088404
RP11-439H8	-0.026	0.444834391	0.713088404
RP11-626K17	-0.026	0.444747485	0.713088404
USP9YP7	-0.026	0.444742971	0.713088404
RNU4-38P	-0.026	0.444736701	0.713088404
GPR27	-0.026	0.444722615	0.713088404
AL590762	-0.026	0.444602499	0.712985603
CTD-3145H4	-0.026	0.444524805	0.712913186
RELA	-0.026	0.444488264	0.712878587
SSR1P2	-0.026	0.444437017	0.712868413
RP11-367E12	-0.026	0.444302608	0.712707654
TOR2A	-0.026	0.444294326	0.712707654
RP11-233G1	-0.026	0.44427383	0.712707654
RP11-578C11	-0.026	0.444263383	0.712707654
snoU83B	-0.026	0.444170319	0.712632636
C8orf87	-0.026	0.444154647	0.712631497
AC016907	-0.026	0.444092086	0.712602852

TFR2	-0.026	0.444032131	0.71253094
MIR320C1	-0.026	0.443980731	0.712496471
IL1RAPL1	-0.026	0.443942044	0.712458393
RP11-791O21	-0.026	0.443914062	0.712437492
NUDT19P3	-0.026	0.443862656	0.712378996
RNU6-1127P	-0.026	0.443860908	0.712378996
CITF22-1A6	-0.026	0.443802864	0.712355048
RPL36AL	-0.026	0.443672813	0.712213451
LL0XNC01-73E8	-0.026	0.443632497	0.712177596
MIR6769A	-0.026	0.443623328	0.712177596
SAMD15	-0.026	0.443588292	0.712177596
MYOC	-0.026	0.44353358	0.712114813
TBPL2	-0.026	0.44352413	0.712114813
RP11-269M20	-0.026	0.443411109	0.711990185
GML	-0.026	0.443402166	0.711990185
AC004603	-0.026	0.443367335	0.711967904
CST4	-0.026	0.443364589	0.711967904
DUSP16	-0.026	0.443310087	0.711923982
RP11-46O21	-0.026	0.443273836	0.71188977
RP11-321A17	-0.026	0.443226255	0.711837358
RP11-380N8	-0.026	0.443200273	0.711819634
RP11-479O16	-0.026	0.443157566	0.711775045
OR5H2	-0.026	0.443061985	0.711645526
EIF2B5	-0.026	0.443045625	0.711643249
COX16	-0.026	0.443026785	0.711636987
IPO11	-0.026	0.443009569	0.711633335
CHMP1AP1	-0.026	0.443005765	0.711633335
RP4-671G15	-0.026	0.442934741	0.711561132
MIR3174	-0.026	0.442851807	0.711475899
OPN1LW	-0.026	0.442800932	0.711418162
SLC8A3	-0.026	0.442757875	0.711372983
RP5-1164C1	-0.026	0.442744604	0.711372983
AL512505	-0.026	0.442738405	0.711372983
AL358354	-0.026	0.442692914	0.711364601
ZNF773	-0.026	0.442628646	0.711285328
CTC-505O3	-0.026	0.442602048	0.711266584
XXbac-BPGBPG24O18	-0.026	0.442559987	0.711222989
RP11-801G16	-0.026	0.44255045	0.711222989
GLULP3	-0.026	0.442393104	0.711050768
RNU6-836P	-0.026	0.442344932	0.71103427
RP11-588F10	-0.026	0.442291491	0.710983423
MS4A8	-0.026	0.442170874	0.710837515
TMEM183A	-0.026	0.442083405	0.71072089
RNU6-613P	-0.026	0.442045155	0.710683385
CSRP3	-0.026	0.441897324	0.710517668
SLC9B1P3	-0.026	0.4418765	0.710508172
RP11-335O4	-0.026	0.441854658	0.710497039
RP11-424M24	-0.026	0.441807743	0.710445587
RNU7-133P	-0.026	0.441801214	0.710445587
TRMT2B-AS1	-0.026	0.441766651	0.710427482
OR4A18P	-0.026	0.441756365	0.710427482
AP000580	-0.026	0.441752187	0.710427482

RCN2	-0.026	0.441722719	0.710427482
TWISTNB	-0.026	0.441658189	0.71039698
RP11-569A2	-0.026	0.441621229	0.710361522
OR2Z1	-0.026	0.441599152	0.710350001
RP11-30H9	-0.026	0.441571603	0.710329677
FAM138D	-0.026	0.441548768	0.710316936
RP11-312J18	-0.026	0.441514558	0.710285892
PRB4	-0.026	0.441505345	0.710285892
MIR6763	-0.026	0.441448706	0.710227933
RP11-167P22	-0.026	0.441429871	0.710221622
FAM206BP	-0.026	0.441409245	0.710221622
ZNRD1	-0.026	0.441395587	0.710221622
MTND5P22	-0.026	0.441353598	0.710218867
OR5BN2P	-0.026	0.441353598	0.710218867
AL596268	-0.026	0.441353598	0.710218867
CCNH	-0.026	0.441257514	0.71013623
RP5-991O23	-0.026	0.441243613	0.71013623
AC007387	-0.026	0.441200446	0.710097602
AC234031	-0.026	0.441191381	0.710097602
RN7SKP30	-0.026	0.44111908	0.710081402
NANOGNBP2	-0.026	0.44103937	0.709977086
RN7SL774P	-0.026	0.440942218	0.709892676
RP11-114B7	-0.026	0.440924916	0.709888816
AC109589	-0.026	0.440885484	0.709860996
ZNF385D-AS2	-0.026	0.440845656	0.709833194
LINC01387	-0.026	0.440692917	0.709659236
TPD52L1	-0.026	0.440687007	0.709659236
PBOV1	-0.026	0.440655592	0.709647119
HIST1H1D	-0.026	0.440651394	0.709647119
WBP1LP12	-0.026	0.44054826	0.70952225
GRK7	-0.026	0.440456491	0.709439924
RP5-1053E7	-0.026	0.440450482	0.709439924
GRAP	-0.026	0.440357724	0.709335318
RP11-843A23	-0.026	0.440272187	0.709287579
RP11-576D8	-0.026	0.440260355	0.709287579
POM121L1P	-0.026	0.440222035	0.70928467
RN7SKP151	-0.026	0.440216365	0.70928467
USP29	-0.026	0.440206842	0.70928467
HIST1H4K	-0.026	0.440166327	0.70928467
CSNK2A2	-0.026	0.440140546	0.70928467
SLC23A2	-0.026	0.440022111	0.709202474
MKRN3	-0.026	0.439995944	0.709184298
PSEN1	-0.026	0.439992667	0.709184298
LINC00587	-0.026	0.439948919	0.709180503
U5	-0.026	0.439876564	0.70908787
DYDC1	-0.026	0.439838025	0.709049744
RP11-310A13	-0.026	0.439709895	0.708963173
IMPG1	-0.026	0.439706876	0.708963173
NDFIP2-AS1	-0.026	0.439542113	0.70883663
RP11-474D14	-0.026	0.439532755	0.70883663
HIPK4	-0.026	0.439513376	0.70883663
CLEC4M	-0.026	0.439383553	0.708748915

ATP5JP1	-0.026	0.439381525	0.708748915
KCNT1	-0.026	0.439374878	0.708748915
TMEM78	-0.026	0.439257623	0.708675627
RP11-543F8	-0.026	0.439230072	0.708675627
RP11-700N1	-0.026	0.439218797	0.708675627
MTND3P6	-0.026	0.439161371	0.708654536
RTP3	-0.026	0.439127527	0.708623931
KB-1592A4	-0.026	0.439116359	0.708623931
HNRNPDLP3	-0.026	0.439031886	0.708541616
OR4A48P	-0.026	0.439016808	0.708541616
FAM27E3	-0.026	0.439013825	0.708541616
RP11-434J24	-0.026	0.439004586	0.708541616
OR7E154P	-0.026	0.438979894	0.708541616
COPG1	-0.026	0.438963481	0.708541616
LINC00630	-0.026	0.438948941	0.708541616
RP11-264J4	-0.026	0.438893362	0.708541616
RP11-104D3	-0.026	0.438875592	0.708541616
AC013467	-0.026	0.438864227	0.708541616
DDTP1	-0.026	0.438764729	0.708541616
OR7E129P	-0.026	0.438698434	0.708479426
ADAM22	-0.026	0.438616527	0.708399381
RNU6-388P	-0.026	0.438615283	0.708399381
CISTR	-0.026	0.438604605	0.708399381
ARHGAP26-IT1	-0.026	0.438555768	0.70839734
AC095061	-0.026	0.438553815	0.70839734
MED28P6	-0.026	0.438491635	0.708365821
TPMTP2	-0.026	0.438442923	0.708311152
RP11-554I8	-0.026	0.438408403	0.708303436
GMPR	-0.026	0.438364003	0.708255728
OR10R2	-0.026	0.43832417	0.708215397
AC090955	-0.026	0.438323148	0.708215397
RP11-193M21	-0.026	0.438238876	0.708149656
RP11-38J22	-0.026	0.438183211	0.708083731
TCEB1P12	-0.026	0.438176764	0.708083731
OR7E10P	-0.026	0.437962411	0.707895057
NDUFA5P9	-0.026	0.437830661	0.70773612
KRT18P61	-0.026	0.437766821	0.707688572
TYMSOS	-0.026	0.437733056	0.707688572
KRTAP19-11P	-0.026	0.437503172	0.707440877
STK24P1	-0.026	0.43750164	0.707440877
CTD-2530N21	-0.026	0.437468217	0.707440877
NOS1AP	-0.026	0.437460829	0.707440877
TLR1	-0.026	0.437414393	0.707440877
PRMT7	-0.026	0.437378922	0.707440877
AC064848	-0.026	0.437370806	0.707440877
HNRNPA1P15	-0.026	0.437359488	0.707440877
RNU6-1249P	-0.026	0.437349492	0.707440877
HSFX2	-0.026	0.437315401	0.707440877
RP11-728K20	-0.026	0.437286773	0.707440877
AF186192	-0.026	0.437243505	0.707405272
RNU6-593P	-0.026	0.437161673	0.707368995
CTD-2540L5	-0.026	0.436957948	0.70711142

RN7SL652P	-0.026	0.436928447	0.707087705
DHRS2	-0.026	0.436917634	0.707087705
PDS5B	-0.026	0.436852219	0.707036417
HMGN2P17	-0.026	0.436851575	0.707036417
ADRB1	-0.026	0.43682809	0.707036417
IFIT1B	-0.026	0.436692313	0.706945777
RP11-497D6	-0.026	0.436630028	0.706889925
RNA5SP492	-0.027	0.436568737	0.706817801
MAMDC2-AS1	-0.027	0.436554217	0.706817801
SNRPEP9	-0.027	0.436506865	0.70676568
RP11-642M2	-0.027	0.436429436	0.70668836
RN7SL703P	-0.027	0.436426053	0.70668836
TJAP1	-0.027	0.436413149	0.70668836
RN7SL756P	-0.027	0.436351536	0.706682352
RP11-421N8	-0.027	0.43626122	0.706584136
CCZ1	-0.027	0.436224134	0.706548199
LINC00866	-0.027	0.436119486	0.70642665
NFU1P1	-0.027	0.436100943	0.70642665
RP5-837I24	-0.027	0.436097315	0.70642665
DLST	-0.027	0.436050617	0.706411195
LTV1P1	-0.027	0.436014037	0.706375962
HMGB1P37	-0.027	0.435978169	0.706341881
TRIM62	-0.027	0.43593893	0.706302334
AL158089	-0.027	0.435892203	0.706250653
RP11-62N21	-0.027	0.435820614	0.70618271
MIR3133	-0.027	0.435759469	0.706143463
RP4-581O6	-0.027	0.435712112	0.706102989
LINC00974	-0.027	0.435703075	0.706102989
RNU6-1121P	-0.027	0.435693394	0.706102989
CTD-3179P9	-0.027	0.435685973	0.706102989
RP11-811I15	-0.027	0.435599038	0.706015837
RP11-341N2	-0.027	0.435593865	0.706015837
NCAN	-0.027	0.435586451	0.706015837
RSL24D1P1	-0.027	0.435481928	0.705946147
LL0YNC03-29C1	-0.027	0.435306364	0.705781657
SNORA42	-0.027	0.435258564	0.705728183
PTH2	-0.027	0.435256772	0.705728183
CDCA4P4	-0.027	0.43521459	0.705704933
HSP90AB7P	-0.027	0.435210299	0.705704933
MIR504	-0.027	0.435206189	0.705704933
PLCB1-IT1	-0.027	0.435199088	0.705704933
RP5-903E17	-0.027	0.435187919	0.705704933
CXorf67	-0.027	0.435179775	0.705704933
RP11-397P13	-0.027	0.435167085	0.705704933
AC002314	-0.027	0.435083423	0.705704933
PRAMEF25	-0.027	0.435075648	0.705704933
RNA5SP197	-0.027	0.434990157	0.705605266
RP11-38M15	-0.027	0.434989593	0.705605266
HIGD1AP5	-0.027	0.434987078	0.705605266
SIGLEC17P	-0.027	0.434937363	0.705605266
NDUFA5P3	-0.027	0.434920832	0.705605266
EFCAB14P1	-0.027	0.434873582	0.705570041

TJP3	-0.027	0.434819432	0.705520564
AL158151	-0.027	0.434784451	0.70548784
ZNRF2P1	-0.027	0.434673399	0.705355707
AC104434	-0.027	0.434661811	0.705355707
RNU6-309P	-0.027	0.434661811	0.705355707
RNU6-978P	-0.027	0.434661811	0.705355707
TVP23BP2	-0.027	0.434661331	0.705355707
AC068643	-0.027	0.434645583	0.705355707
CHCHD2P11	-0.027	0.434626527	0.705355707
RN7SL231P	-0.027	0.43457353	0.705355707
CTC-366B18	-0.027	0.434533151	0.705320373
PXMP4	-0.027	0.434400169	0.705152585
CTB-47B11	-0.027	0.434354412	0.705102341
RP11-885L14	-0.027	0.434336762	0.705097722
INTS4P2	-0.027	0.434303811	0.705093807
TSEN15P2	-0.027	0.434302538	0.705093807
RP11-109G23	-0.027	0.434281198	0.705093807
ABCB4	-0.027	0.434278128	0.705093807
RN7SL520P	-0.027	0.434230431	0.705093807
ZNF687	-0.027	0.43421551	0.705093807
RP13-221M14	-0.027	0.434212457	0.705093807
PAPD7	-0.027	0.434160924	0.705093807
DNAJB7	-0.027	0.434143757	0.705093807
HNRNPA1P45	-0.027	0.434019904	0.704991849
EFCAB2	-0.027	0.433932668	0.70491865
PRUNE	-0.027	0.433828034	0.704800374
STT3A-AS1	-0.027	0.433792249	0.704766278
IPO9	-0.027	0.433708974	0.704703101
CTC-508F8	-0.027	0.433678515	0.704678653
RP11-523H20	-0.027	0.433668864	0.704678653
TMIGD1	-0.027	0.433588516	0.704635618
USP53	-0.027	0.433434663	0.704496857
AC135999	-0.027	0.433430561	0.704496857
AP000719	-0.027	0.433380734	0.704481267
RAD17P2	-0.027	0.433365844	0.704481267
COPA	-0.027	0.433330739	0.704449026
RNU6-917P	-0.027	0.433276207	0.704416902
IFNB1	-0.027	0.433187072	0.704335678
USP12PY	-0.027	0.433162478	0.704319734
FEM1C	-0.027	0.43314506	0.704315459
MIR33B	-0.027	0.433073645	0.704257647
EIF4EP5	-0.027	0.432974439	0.704182271
MIR6780A	-0.027	0.432890951	0.704118247
MAGEC2	-0.027	0.432831862	0.704046553
VIMP	-0.027	0.432776724	0.703986644
ZBTB4	-0.027	0.432710385	0.703971752
RP1-169I5	-0.027	0.432706928	0.703971752
CUL1P1	-0.027	0.432681826	0.703971752
CPSF1P1	-0.027	0.43266914	0.703971752
TGOLN2	-0.027	0.432653025	0.703971752
RP11-55K13	-0.027	0.432618939	0.703971752
RP11-31H5	-0.027	0.43261201	0.703971752

ZRANB3	-0.027	0.432607203	0.703971752
SRGNP1	-0.027	0.432589203	0.703971752
HEATR1	-0.027	0.432511404	0.703909945
NDE1P2	-0.027	0.432507625	0.703909945
CYP3A7	-0.027	0.432452744	0.703886635
NPIPA1	-0.027	0.432370451	0.703800795
SNORD32B	-0.027	0.432202472	0.703599506
RNU6-1254P	-0.027	0.432193142	0.703599506
DCAF13P1	-0.027	0.432165273	0.703587046
YRDCP3	-0.027	0.432157544	0.703587046
RP11-24M17	-0.027	0.43201981	0.70344641
CTD-3037G24	-0.027	0.432012857	0.70344641
ODF2	-0.027	0.432009219	0.70344641
NPM1P33	-0.027	0.431893627	0.703337133
MEF2AP1	-0.027	0.431841948	0.703301069
CYP4A11	-0.027	0.431837637	0.703301069
RSL24D1P3	-0.027	0.431804395	0.703301069
AC008171	-0.027	0.431801857	0.703301069
TRPC7	-0.027	0.431751624	0.703301069
AC118282	-0.027	0.431657664	0.703241416
AP003550	-0.027	0.43163286	0.703241416
RP11-371M22	-0.027	0.431599819	0.703241416
HIST1H2BJ	-0.027	0.431587363	0.703241416
LINC01584	-0.027	0.431559022	0.703241416
TBC1D8	-0.027	0.431496493	0.703241416
TGFBRAP1	-0.027	0.431489455	0.703241416
KIAA0040	-0.027	0.431440825	0.703200855
STX2	-0.027	0.431404355	0.703196721
RNU2-47P	-0.027	0.43137407	0.703188308
LA16c-429E7	-0.027	0.431359932	0.703188308
AC018512	-0.027	0.431352044	0.703188308
AC005037	-0.027	0.431345538	0.703188308
KRTAP10-10	-0.027	0.431336108	0.703188308
AC004447	-0.027	0.431304232	0.703188308
TMTC1	-0.027	0.431293677	0.703188308
MRPS27	-0.027	0.431293037	0.703188308
HMGN2P36	-0.027	0.431273605	0.703188308
RP11-33I11	-0.027	0.431193456	0.703158622
RPN2	-0.027	0.431137283	0.703091094
ADPRM	-0.027	0.431132806	0.703091094
HMGN2P25	-0.027	0.431130534	0.703091094
AL353783	-0.027	0.431087955	0.703091094
ASXL1	-0.027	0.431012576	0.703091094
AC005522	-0.027	0.430936316	0.703028154
RP11-315A16	-0.027	0.430896967	0.70298804
GTF2H4	-0.027	0.430828431	0.702943159
RP3-364H10	-0.027	0.430756872	0.702888576
FXN	-0.027	0.430729344	0.702888576
IL1A	-0.027	0.430709342	0.702888576
MIOX	-0.027	0.430704071	0.702888576
RN7SKP135	-0.027	0.430680138	0.702888576
XXbac-B135H6	-0.027	0.430663506	0.702888576

LINC01399	-0.027	0.430599038	0.702887219
AC093843	-0.027	0.430510055	0.702814232
DPPA3P2	-0.027	0.430482142	0.702792752
ZNF215	-0.027	0.430369703	0.702699777
RP11-539E17	-0.027	0.430359884	0.702699777
RP11-624D20	-0.027	0.430326333	0.702699777
RP11-361M10	-0.027	0.430310573	0.702699777
RP11-121A14	-0.027	0.430307011	0.702699777
H2BFS	-0.027	0.430301056	0.702699777
RP11-454C18	-0.027	0.430292808	0.702699777
AC010319	-0.027	0.430266179	0.702699777
RP11-135A24	-0.027	0.430240691	0.702699777
RP11-123N4	-0.027	0.43017571	0.702699777
RP11-313J2	-0.027	0.430127953	0.702672156
LAMTOR5P1	-0.027	0.430043775	0.702558737
RP11-397G17	-0.027	0.429940913	0.702438883
RNU6-1017P	-0.027	0.42992613	0.702438827
OGFRL1	-0.027	0.429911263	0.702438633
EWSAT1	-0.027	0.429865929	0.702388658
C22orf24	-0.027	0.429743155	0.702236233
CRCP	-0.027	0.429601643	0.70212544
RP3-333B15	-0.027	0.429575966	0.70212544
AC020915	-0.027	0.429503104	0.702060759
SMU1	-0.027	0.429462516	0.702018508
RP4-811M8	-0.027	0.429379538	0.701931052
RNU6-1091P	-0.027	0.429337707	0.70188676
OR1F2P	-0.027	0.429284387	0.701823682
AC018799	-0.027	0.429228696	0.701780814
OR7A3P	-0.027	0.42921957	0.701780814
RP11-415G4	-0.027	0.429142286	0.701735894
NSL1	-0.027	0.429131397	0.701735894
TUBA3D	-0.027	0.429078138	0.701697178
ATPAF2	-0.027	0.428999054	0.701622127
ITGAM	-0.027	0.428976096	0.701608672
PYCR2	-0.027	0.428863547	0.701545051
OR2L5	-0.027	0.428862517	0.701545051
OR7L1P	-0.027	0.428827287	0.701545051
OR4A40P	-0.027	0.428818029	0.701545051
CTB-139P11	-0.027	0.428796241	0.701545051
SERBP1P2	-0.027	0.428737381	0.701531427
RP11-755J8	-0.027	0.428726176	0.701531427
RP11-398M15	-0.027	0.428686218	0.701520015
CBR3-AS1	-0.027	0.428631171	0.701475381
RP11-241J12	-0.027	0.428611238	0.701469621
AC000354	-0.027	0.428606392	0.701469621
MT1CP	-0.027	0.42859965	0.701469621
RNU6-169P	-0.027	0.428557433	0.701453875
OR4T1P	-0.027	0.428538028	0.701453875
AP006621	-0.027	0.428337378	0.701237027
ABHD12	-0.027	0.428254551	0.701126793
RP11-15A1	-0.027	0.428250729	0.701126793
CYCSP4	-0.027	0.428186406	0.701087525

RN7SL619P	-0.027	0.428176348	0.701087525
RP11-756H6	-0.027	0.428147272	0.701071655
BIRC5	-0.027	0.428137972	0.701071655
TXNL1	-0.027	0.428135106	0.701071655
RNA5SP347	-0.027	0.428028891	0.700965499
CELA3B	-0.027	0.428011687	0.700965499
DDX39AP1	-0.027	0.42798625	0.700965499
NFRKB	-0.027	0.427979378	0.700965499
ZNF790	-0.027	0.427931933	0.700935935
TTC4	-0.027	0.427905506	0.700916754
RN7SL785P	-0.027	0.427872281	0.700886435
OR10T1P	-0.027	0.427869574	0.700886435
GABRR3	-0.027	0.427817794	0.700886435
KRT73	-0.027	0.427705557	0.700733834
C8orf37-AS1	-0.027	0.427531227	0.700496413
NCBP2	-0.027	0.427502844	0.700474007
PNPLA2	-0.027	0.427496798	0.700474007
RP11-92K15	-0.027	0.42748757	0.700474007
AC143336	-0.027	0.427438423	0.700440744
PLGLB2	-0.027	0.427426005	0.700440744
ARHGAP8	-0.027	0.427385429	0.700419829
POLN	-0.027	0.427360294	0.70040911
CLUHP4	-0.027	0.427344893	0.70040911
RPL38P3	-0.027	0.427314987	0.700407162
PAIP2B	-0.027	0.427289666	0.700389762
P2RX3	-0.027	0.42717126	0.700243877
CDKN2AIPNL	-0.027	0.427137615	0.700212825
CDKN2B-AS_3	-0.027	0.427085337	0.700151223
ARHGAP21	-0.027	0.426929313	0.699943628
RP11-297L17	-0.027	0.426926304	0.699943628
MAP3K7	-0.027	0.426917866	0.699943628
RP11-444D3	-0.027	0.426894213	0.699943628
RNU7-155P	-0.027	0.426876307	0.699943628
TMEM135	-0.027	0.426864767	0.699943628
RN7SL302P	-0.027	0.426640016	0.699691321
RP11-292F9	-0.027	0.426630274	0.699691321
GS1-124K5	-0.027	0.426612679	0.699691321
HCG4B	-0.027	0.426523017	0.699604089
FLJ38576	-0.027	0.426492672	0.699588996
PRRC2A	-0.027	0.426445516	0.699535737
RP11-65B23	-0.027	0.426443899	0.699535737
CST2P1	-0.027	0.426429749	0.699535737
CDK2	-0.027	0.426383554	0.69953047
RP11-464I4	-0.027	0.426341881	0.69953047
MFSD9	-0.027	0.426313338	0.69953047
AC023274	-0.027	0.426267221	0.699508285
ABCB7	-0.027	0.426140843	0.69934909
RPL5P35	-0.027	0.426043931	0.699254288
RP11-688H10	-0.027	0.425911445	0.699141255
RP1-167A14	-0.027	0.425892491	0.699134239
AC076972	-0.027	0.42580883	0.699044194
GOLGA8UP	-0.027	0.425775902	0.699015129

RNU6-315P	-0.027	0.425773192	0.699015129
CLRN1	-0.027	0.425732043	0.699015129
RNU6-934P	-0.027	0.425719003	0.699015129
AC106873	-0.027	0.425692986	0.699015129
LY6G6F	-0.027	0.425692747	0.699015129
RP3-453I5	-0.027	0.425673413	0.699015129
RPL7AP25	-0.027	0.425667226	0.699015129
CLIC4P2	-0.027	0.425656283	0.699015129
STS	-0.027	0.425642648	0.699015129
AL121952	-0.027	0.425618244	0.699015129
HMGB1P38	-0.027	0.425564677	0.69898158
PCDH20	-0.027	0.425457871	0.698902563
IQCF6	-0.027	0.425446224	0.698902563
FIGF	-0.027	0.425437477	0.698902563
MTND5P17	-0.027	0.425433146	0.698902563
STSP1	-0.027	0.425372505	0.69885875
AC093515	-0.027	0.425361679	0.69885875
MTND2P32	-0.027	0.42533953	0.69885875
MIR3689E	-0.027	0.42527289	0.698791511
WI2-88277B6	-0.027	0.425217178	0.698724072
OR5AQ1P	-0.027	0.425070409	0.69856826
RNU6-124P	-0.027	0.425061548	0.69856826
PSMA2P2	-0.027	0.425040784	0.69856826
IRF2BPL	-0.027	0.425022363	0.69856826
ABCA8	-0.027	0.42501697	0.69856826
RP11-506O24	-0.027	0.424827288	0.698372541
SGOL1-AS1	-0.027	0.424808889	0.698366399
RP4-687K1	-0.027	0.424794143	0.698366264
RP11-307A17	-0.027	0.424760073	0.698334357
ZNF29P	-0.027	0.424745816	0.698334357
RP13-259N13	-0.027	0.424434905	0.697992515
SLC13A5	-0.027	0.424409005	0.697992515
FKBP3	-0.027	0.424367434	0.697955834
KCNB1	-0.027	0.42435555	0.697955834
TUBB2BP1	-0.027	0.424344611	0.697955834
PPIF	-0.027	0.424296461	0.697955834
ZNF324B	-0.027	0.424235632	0.697929894
LINC00884	-0.027	0.424231828	0.697929894
FUK	-0.027	0.424108605	0.697769121
MEMO1P4	-0.027	0.42407992	0.697746031
RP11-306O13	-0.027	0.423967644	0.697627271
AAK1	-0.027	0.423952781	0.697627271
RP11-367J11	-0.027	0.423950881	0.697627271
OR52E4	-0.027	0.42379597	0.697447501
IGLVI-68	-0.027	0.423771301	0.697447501
SNORD115-24	-0.027	0.423708637	0.697376067
RP1-102E24	-0.027	0.42370518	0.697376067
TRAPPC2P2	-0.027	0.423410594	0.697030049
GPR37L1	-0.027	0.423410502	0.697030049
RP11-317J9	-0.027	0.423364062	0.697020796
ZDBF2	-0.027	0.423278882	0.697020796
CAPN8	-0.027	0.423239335	0.697013133

AC117422	-0.027	0.423223966	0.697013133
TAX1BP1	-0.027	0.42319283	0.697013133
FLJ40194	-0.027	0.423016587	0.696790861
OR52S1P	-0.027	0.422928853	0.696694538
AJAP1	-0.027	0.422879711	0.696637681
CAMTA1-IT1	-0.027	0.422872362	0.696637681
NAMA_2	-0.027	0.422815074	0.696613604
MIR8081	-0.027	0.422787082	0.696605561
CRYM-AS1	-0.027	0.422583646	0.696432367
EFCAB5	-0.027	0.422425517	0.696298888
GBF1	-0.027	0.422379007	0.696284234
RP11-152L20	-0.027	0.42219807	0.696092619
DTX3	-0.027	0.422110779	0.696020984
RNA5SP468	-0.027	0.422068067	0.69597465
SEPP1	-0.027	0.422035921	0.695967235
RNA5SP286	-0.027	0.422020308	0.695967235
RIMS1	-0.027	0.422000157	0.695967235
RBKS	-0.027	0.421980019	0.695967235
RP11-552E4	-0.027	0.421975069	0.695967235
RPS7P8	-0.027	0.42196179	0.695967235
TRAJ52	-0.027	0.42194633	0.695967235
MAMDC4	-0.027	0.421845177	0.695872134
LINC01052	-0.027	0.421805433	0.695830673
AC012445	-0.027	0.42180088	0.695830673
UTRN	-0.027	0.421781713	0.695830673
AGPAT6	-0.027	0.421704994	0.695737278
TTTY12	-0.027	0.421697856	0.695737278
RP4-736L20	-0.027	0.42169309	0.695737278
RP11-338C15	-0.027	0.421688891	0.695737278
RP11-421N10	-0.027	0.421656285	0.695737278
NMNAT2	-0.027	0.421559472	0.695641777
LINC00184	-0.027	0.421410406	0.695443985
RNU6-419P	-0.027	0.421403404	0.695443985
OR4A42P	-0.027	0.42137658	0.695436357
RNU6-965P	-0.027	0.421284069	0.695331867
POTEH-AS1	-0.027	0.421204108	0.695251662
TACR2	-0.027	0.421127815	0.695218524
RPL7AP4	-0.027	0.421042686	0.695102084
RNA5SP502	-0.027	0.421040561	0.695102084
DYDC2	-0.027	0.421039222	0.695102084
RNU6-749P	-0.027	0.421032541	0.695102084
RP11-88H1	-0.027	0.421015253	0.695102084
CTD-3229J4	-0.027	0.420890722	0.695019847
RPL23AP2	-0.027	0.420878026	0.695019847
AC005017	-0.027	0.420853825	0.695019847
CDC45	-0.027	0.42080298	0.694995442
FKSG59	-0.027	0.420787184	0.694993453
AP003307	-0.027	0.420758479	0.694993453
AP000870	-0.027	0.420756794	0.694993453
UBL7	-0.027	0.420728888	0.694993453
ATG7	-0.027	0.420680023	0.694961064
RPL17P51	-0.027	0.420661049	0.694953824

SFT2D2	-0.027	0.420529351	0.694823656
MIR4491	-0.027	0.420517779	0.694823656
SLC25A1P3	-0.027	0.420489194	0.694823656
KRTAP9-1	-0.027	0.420482652	0.694823656
FAM222B	-0.027	0.420449929	0.694823656
RP3-463P15	-0.027	0.420381755	0.694817776
CTD-2193G5	-0.027	0.420359585	0.694817776
ETV3L	-0.027	0.42029678	0.694785837
LMNTD2	-0.027	0.420249839	0.694732353
OR2T10	-0.027	0.420248073	0.694732353
RGPD4-AS1	-0.027	0.420142746	0.694627645
RP11-320N21	-0.027	0.420054977	0.694530756
TTYH2	-0.027	0.420024292	0.694504131
IZUMO4	-0.027	0.420020513	0.694504131
MYL6P1	-0.027	0.420006882	0.694504131
UBBP3	-0.027	0.419979607	0.694502578
RNA5SP208	-0.027	0.419917536	0.694485949
ZFHX2	-0.027	0.419856124	0.694418933
RP11-275E15	-0.027	0.41985499	0.694418933
RP11-8J9	-0.027	0.419852571	0.694418933
CTD-2323K18	-0.027	0.41980354	0.694406062
CTD-2384A14	-0.027	0.419750767	0.694365245
C20orf78	-0.027	0.419734255	0.694362048
EFTUD1P2	-0.027	0.41968102	0.694320573
RP4-799P18	-0.027	0.419676983	0.694320573
MTND2P17	-0.027	0.419654575	0.694320573
RP11-12D5	-0.027	0.419575646	0.694292588
CDHR1	-0.027	0.419563785	0.694292588
RN7SL592P	-0.027	0.419532824	0.694269973
KCND3-IT1	-0.028	0.419424904	0.694163739
C8orf17	-0.028	0.41942345	0.694163739
C20orf195	-0.028	0.419386444	0.694163739
MIR5681A	-0.028	0.419357085	0.694147982
ZNF720	-0.028	0.419261238	0.694061695
RNA5SP71	-0.028	0.419248067	0.694061695
AC012442	-0.028	0.419212787	0.694029735
RP11-271K21	-0.028	0.419108271	0.693904943
RAB28	-0.028	0.419065883	0.693859405
AC231656	-0.028	0.419028917	0.693859405
PLXNC1	-0.028	0.418960521	0.693780909
RP5-1049G16	-0.028	0.418874038	0.693685936
RP11-457P14	-0.028	0.418801649	0.693638411
KIAA1324	-0.028	0.418737581	0.693556417
CTD-2248H3	-0.028	0.418596899	0.693395747
VAMP8	-0.028	0.418595804	0.693395747
HIST1H2BC	-0.028	0.418522569	0.693365486
FMNL2	-0.028	0.418503083	0.693365486
ZFHX3	-0.028	0.418498683	0.693365486
CTD-2300H10	-0.028	0.418461204	0.693363893
LINC01019	-0.028	0.418456402	0.693363893
RP11-722M1	-0.028	0.418451995	0.693363893
IL20	-0.028	0.418300397	0.693218032

TBL2	-0.028	0.418295054	0.693218032
CTB-105L4	-0.028	0.418255332	0.693215711
RP11-1103G16	-0.028	0.418227076	0.693193003
RP11-347C12	-0.028	0.418142056	0.69312445
CTD-2533G20	-0.028	0.41809295	0.693067173
RP11-118G23	-0.028	0.418077842	0.69306625
CCNB1	-0.028	0.41801296	0.692982814
FAM136A	-0.028	0.417883574	0.692816548
SNORA32	-0.028	0.41783378	0.692782226
RNU6-698P	-0.028	0.417753383	0.69274539
CDCA3	-0.028	0.417647159	0.692641592
LINC01037	-0.028	0.417597083	0.692587798
LINC00899	-0.028	0.417466683	0.692438731
MS4A5	-0.028	0.417460415	0.692438731
AC019185	-0.028	0.417413874	0.692399366
OGFOD2	-0.028	0.417391681	0.692386667
RP11-390B4	-0.028	0.417367989	0.69237148
XXyac-YM21GA2	-0.028	0.417290007	0.692290343
RP11-542A14	-0.028	0.417265214	0.692282492
HINFP	-0.028	0.417257702	0.692282492
LINC01167	-0.028	0.417208516	0.692282492
AC025156	-0.028	0.417180233	0.692282492
AC009237	-0.028	0.417105869	0.692201861
RP11-817G13	-0.028	0.417086397	0.692193666
PLIN5	-0.028	0.417081381	0.692193666
CCAT1	-0.028	0.4170304	0.692193666
TMOD4	-0.028	0.417006086	0.69218098
MIR30D	-0.028	0.416981735	0.692164684
CTA-392C11	-0.028	0.416976643	0.692164684
RP11-2I17	-0.028	0.416882344	0.692072063
PLA2G10	-0.028	0.416851049	0.692044232
RP11-471B18	-0.028	0.416813598	0.692030302
USP25	-0.028	0.41668724	0.69184463
RP11-180D15	-0.028	0.416433424	0.691495526
RP11-183G22	-0.028	0.416427026	0.691495526
RP11-522N14	-0.028	0.416395888	0.691481415
AC010884	-0.028	0.416371785	0.691465498
NPTXR	-0.028	0.416350009	0.691453445
AC008798	-0.028	0.416349739	0.691453445
TAF9P3	-0.028	0.416330351	0.691453445
BRWD1-AS1	-0.028	0.416283019	0.691438632
COPS5	-0.028	0.416274812	0.691438632
RP11-150O12	-0.028	0.416221866	0.691394649
C10orf111	-0.028	0.416196447	0.691391287
RP11-762J3	-0.028	0.416189107	0.691391287
MYADML2	-0.028	0.416160853	0.691380388
ZBTB46	-0.028	0.416074327	0.691260751
CTB-161M19	-0.028	0.416038062	0.691260751
RP11-272M24	-0.028	0.415996189	0.691227384
MIR5580	-0.028	0.415969593	0.691207305
RP11-111J6	-0.028	0.41594525	0.691190972
AC023992	-0.028	0.415942429	0.691190972

AMT	-0.028	0.41591805	0.691190972
CTD-3032H12	-0.028	0.415904898	0.691190972
RNA5SP497	-0.028	0.415772608	0.69107287
OR10AE1P	-0.028	0.415771576	0.69107287
TUBB4BP4	-0.028	0.415742615	0.691071253
GP9	-0.028	0.415683144	0.691020632
RP11-534P6	-0.028	0.415666162	0.691020632
RP11-460N11	-0.028	0.415618132	0.690987494
KRT32	-0.028	0.415573896	0.690959607
RP11-306B9	-0.028	0.415413835	0.690741708
AC083939	-0.028	0.415395618	0.690741708
RPS26P21	-0.028	0.415368959	0.690739434
EXOSC3	-0.028	0.415284528	0.690647259
C1RL-AS1	-0.028	0.415198495	0.690528294
TMEM63A	-0.028	0.415107156	0.690448716
AC115522	-0.028	0.415082421	0.690448716
ANP32C	-0.028	0.415018839	0.690398262
AC106053	-0.028	0.41501783	0.690398262
RP4-730K3	-0.028	0.414974226	0.690398262
RP11-404L6	-0.028	0.414972906	0.690398262
C16orf72	-0.028	0.414938898	0.690398262
FAM207CP	-0.028	0.414890697	0.690353884
AC078855	-0.028	0.414719652	0.690190108
AC092664	-0.028	0.414674034	0.690186273
MIR4711	-0.028	0.414648968	0.690168672
RP11-34F20	-0.028	0.41464001	0.690168672
PGBD4P8	-0.028	0.414636553	0.690168672
TPSG1	-0.028	0.414583329	0.690165033
TEF	-0.028	0.414544993	0.690140324
CEACAMP3	-0.028	0.41449989	0.690089358
MIR6768	-0.028	0.414453844	0.690036819
CDS2	-0.028	0.414330314	0.689855265
MIR3179-1	-0.028	0.414256347	0.689804456
KYNUP1	-0.028	0.414253552	0.689804456
TEKT2	-0.028	0.414215346	0.689804456
RP11-356M17	-0.028	0.414182867	0.689804456
RP11-826F13	-0.028	0.414178793	0.689804456
FNIP1	-0.028	0.414156619	0.689804456
MTND1P19	-0.028	0.414148975	0.689804456
ABCC5	-0.028	0.414058232	0.689715704
EHMT1-IT1	-0.028	0.413785626	0.689382166
RPL21P108	-0.028	0.413757468	0.689359369
MRPS36	-0.028	0.41369947	0.689286851
RP11-322I2	-0.028	0.413648611	0.689237722
RAB3GAP2	-0.028	0.413635168	0.689237722
AP002806	-0.028	0.413537047	0.68911266
LINC01513	-0.028	0.413470075	0.689025167
ZNF726	-0.028	0.413464142	0.689025167
MIR5095	-0.028	0.413441395	0.689025167
IFNA7	-0.028	0.413418621	0.689011746
RP1-41P2	-0.028	0.413351593	0.688924144
GAPDHP26	-0.028	0.413344564	0.688924144

RNU4ATAC10P	-0.028	0.413321709	0.688924144
RPL21P135	-0.028	0.413312025	0.688924144
PABPC1P9	-0.028	0.413281623	0.688924144
NIPAL1	-0.028	0.413266639	0.688924144
CFAP57	-0.028	0.413257904	0.688924144
IGHVII-53-1	-0.028	0.413243449	0.688924144
DUSP8P4	-0.028	0.413194709	0.688903745
RNY1P3	-0.028	0.413164661	0.688877763
RPH3AL	-0.028	0.413164634	0.688877763
THSD7A	-0.028	0.413149974	0.688877763
RBM48P1	-0.028	0.413124161	0.688877763
KIAA0408	-0.028	0.413106439	0.688877153
HIST1H1PS2	-0.028	0.413104256	0.688877153
RNU6-159P	-0.028	0.412986162	0.688748931
AC104133	-0.028	0.412940723	0.688697266
MIR6868	-0.028	0.412838262	0.688574607
SNORD91B	-0.028	0.412779768	0.68854026
ZNF888	-0.028	0.41271125	0.688459202
CREBBP	-0.028	0.412675715	0.688424037
PXN-AS1	-0.028	0.412641428	0.688406737
SNORD116-29	-0.028	0.412614396	0.688394081
OR7E19P	-0.028	0.412609543	0.688394081
Hammerhead_HH10	-0.028	0.412543879	0.68835583
OTULIN	-0.028	0.412518011	0.688353841
KDM4B	-0.028	0.412489654	0.688353841
LINC00524	-0.028	0.41242636	0.688297366
OR52B3P	-0.028	0.41235644	0.688209047
ZC3H11B	-0.028	0.412353907	0.688209047
RN7SKP8	-0.028	0.41233903	0.688209047
RN7SL630P	-0.028	0.412314547	0.688209047
LHX1	-0.028	0.412300482	0.688209047
RP11-195B21	-0.028	0.412277617	0.688209047
AL133230	-0.028	0.412235736	0.688209047
KCTD20	-0.028	0.412234796	0.688209047
PIWIL4	-0.028	0.41222732	0.688209047
RP11-124B13	-0.028	0.412189592	0.688209047
RNU6-669P	-0.028	0.412155937	0.688207791
CAMSAP1	-0.028	0.41202176	0.688032961
CTB-54O9	-0.028	0.411979419	0.688032961
RP11-179D22	-0.028	0.411892983	0.687920157
DGKK	-0.028	0.411866496	0.687917626
AF178030	-0.028	0.411770785	0.687854062
POU4F1	-0.028	0.41172282	0.687798062
DCAF5	-0.028	0.411704506	0.687791592
RP11-129B9	-0.028	0.411659996	0.687741358
YWHAZP8	-0.028	0.41161541	0.687690992
RP11-216L13	-0.028	0.411596816	0.687684051
B3GALNT2	-0.028	0.411585923	0.687684051
NDUFA7	-0.028	0.411499411	0.687666051
AC012593	-0.028	0.411446615	0.687601949
RNU6-10P	-0.028	0.411335515	0.687440402
AC104781	-0.028	0.411185474	0.687233078

RNA5SP463	-0.028	0.41110086	0.687120571
HCAR3	-0.028	0.410863295	0.686816493
ELFN1-AS1	-0.028	0.410828878	0.686816493
PGAP2	-0.028	0.410822623	0.686816493
CTC-339F2	-0.028	0.410814564	0.686816493
RPL6P2	-0.028	0.410751511	0.68680179
CYP17A1	-0.028	0.410709615	0.686755849
RP11-561P12	-0.028	0.410706983	0.686755849
MTPN	-0.028	0.410705435	0.686755849
HEXDC	-0.028	0.410452163	0.68651818
SNORA13	-0.028	0.410437959	0.68651818
AC073648	-0.028	0.410414741	0.686503806
KRTAP5-11	-0.028	0.410383676	0.686475953
RP11-526P6	-0.028	0.410299463	0.686383298
MAP3K8	-0.028	0.410299002	0.686383298
MIR5693	-0.028	0.410222832	0.686303317
ANKS1A	-0.028	0.410210787	0.686303317
FBXO34	-0.028	0.410162025	0.686273907
CTD-2313J17	-0.028	0.410125378	0.686236698
RP11-1378G18	-0.028	0.410050787	0.686160102
GS1-293C5	-0.028	0.410024228	0.686139768
CNN2P11	-0.028	0.409968835	0.686095285
RP11-195O1	-0.028	0.409955385	0.686095285
RP11-641D5	-0.028	0.409921681	0.686064586
TRMT112	-0.028	0.409850277	0.685969186
KRTAP19-7	-0.028	0.409828082	0.685956144
RN7SL294P	-0.028	0.409756563	0.685911224
CDYL	-0.028	0.409712458	0.685883136
NARS2	-0.028	0.409707802	0.685883136
MAGEB18	-0.028	0.409692586	0.685883136
CCDC14	-0.028	0.40959825	0.685788366
EXOSC10	-0.028	0.409589251	0.685788366
DNAI2	-0.028	0.409550946	0.685782548
AL356019	-0.028	0.409421395	0.685685096
XAGE2B	-0.028	0.409355593	0.685623113
EFCAB9	-0.028	0.409351081	0.685623113
TMCO3	-0.028	0.409348176	0.685623113
AC139143	-0.028	0.409328608	0.685623113
RMST_9	-0.028	0.409280207	0.685620582
MTND1P27	-0.028	0.409251063	0.685616814
CTD-2287N17	-0.028	0.40916256	0.685565231
RN7SL751P	-0.028	0.409106726	0.685537214
RP1-65P5	-0.028	0.409032786	0.685468139
RP11-384B12	-0.028	0.408955329	0.685362449
FMO11P	-0.028	0.408801742	0.685225613
CHAC2	-0.028	0.408737954	0.68516692
CCDC113	-0.028	0.408690225	0.685111026
RP11-169D4	-0.028	0.408615986	0.685039239
STIM1	-0.028	0.408409811	0.684833788
PRKCI	-0.028	0.408376355	0.684801798
RP1-272L16	-0.028	0.408363785	0.684801798
GXYLT1P5	-0.028	0.408362147	0.684801798

RP11-528N21	-0.028	0.408285465	0.684794049
RP11-422P24	-0.028	0.408244095	0.684783803
FAM86C1	-0.028	0.40814004	0.684692244
KB-173C10	-0.028	0.408105825	0.684677888
RP11-556G22	-0.028	0.408080794	0.68466777
AC144522	-0.028	0.408029616	0.68463014
PKNOX2	-0.028	0.407958085	0.684558353
AC103863	-0.028	0.407929723	0.684534879
KCNN1	-0.028	0.407893857	0.684498809
KLF12	-0.028	0.407745021	0.684302058
RBM22P3	-0.028	0.40770349	0.68427579
RNU6-748P	-0.028	0.407509424	0.684036191
RPS12P18	-0.028	0.407468345	0.684025761
OR8L1P	-0.028	0.407390562	0.683919291
RPL36P18	-0.028	0.407390166	0.683919291
PCNPP1	-0.028	0.407378919	0.683919291
RP11-361F19	-0.028	0.407344247	0.683919291
AC098617	-0.028	0.407264692	0.683919291
ZNF321P	-0.028	0.407187911	0.683879206
AP002815	-0.028	0.407157924	0.683866217
CD209	-0.028	0.407055522	0.683766563
RP11-690P14	-0.028	0.406928314	0.68357699
RP11-507M3	-0.028	0.406682064	0.68331691
RP11-401L13	-0.028	0.406594695	0.68320934
RNU6-1165P	-0.028	0.406556454	0.683169186
FNIP2	-0.028	0.40651468	0.68313555
RP11-190J23	-0.028	0.406508553	0.68313555
AL442639	-0.028	0.406499199	0.68313555
RAB40B	-0.028	0.406473284	0.68313555
RPL31P57	-0.028	0.406471787	0.68313555
DEFB127	-0.028	0.406464303	0.68313555
FTCDNL1	-0.028	0.406417682	0.683128814
AL449212	-0.028	0.406389786	0.683106033
LRRC18	-0.028	0.406383371	0.683106033
RP11-112N23	-0.028	0.406336074	0.683088074
RP11-775G23	-0.028	0.406294216	0.683041815
CH17-53B9	-0.028	0.406254412	0.682999008
AC008391	-0.028	0.406245636	0.682999008
ZNF254	-0.028	0.406191924	0.682942806
AC005048	-0.028	0.406191415	0.682942806
BTF3P11	-0.028	0.40612085	0.682942806
OR10A6	-0.028	0.406112943	0.682942806
RN7SL513P	-0.028	0.406074465	0.682942806
AF213884	-0.028	0.406054066	0.682942806
NXPH1	-0.028	0.405960136	0.682914079
CBX6	-0.028	0.40589679	0.682879876
NACC2	-0.028	0.405844321	0.682815723
TAS2R18	-0.028	0.405834946	0.682815723
CAP2	-0.028	0.405778587	0.682753365
RP11-542E6	-0.028	0.405750556	0.68273032
AC006322	-0.028	0.405739746	0.68273032
RP11-690J15	-0.028	0.405703194	0.682722988

INADL	-0.028	0.405484866	0.682500273
KB-1208A12	-0.028	0.405472836	0.682500273
RNA5SP434	-0.028	0.405467775	0.682500273
CTC-325H20	-0.028	0.405451553	0.682500273
RNU7-172P	-0.028	0.405400024	0.682478066
RP11-794A8	-0.028	0.405381704	0.682471348
RP11-13L2	-0.028	0.405368746	0.682471348
CD46P1	-0.028	0.405330227	0.682432929
SNORA14B	-0.028	0.405220465	0.68234898
SNORA34	-0.028	0.405181344	0.68234898
LINC00328-2P	-0.028	0.405162535	0.682343566
MIR548A2	-0.028	0.405045781	0.682195184
RP11-723O4	-0.028	0.405038666	0.682195184
MIR5685	-0.028	0.405038626	0.682195184
MIR4511	-0.028	0.404961668	0.682150757
POGK	-0.028	0.404779159	0.681968078
RNA5SP430	-0.028	0.40474831	0.681968078
FOPNL	-0.028	0.404663924	0.681881053
AC104763	-0.028	0.404630713	0.681881053
ABCC5-AS1	-0.028	0.404623387	0.681881053
RP11-580J4	-0.028	0.404614133	0.681881053
AC090774	-0.028	0.404593588	0.681881053
RP11-114L10	-0.028	0.40455797	0.681855844
RP11-58G13	-0.028	0.404535085	0.681841402
CCDC27	-0.028	0.40452016	0.681840376
RNU6-379P	-0.028	0.404513098	0.681840376
SUCLA2P3	-0.028	0.404484537	0.681828592
QPCT	-0.028	0.404477246	0.681828592
UBQLN4	-0.028	0.404422423	0.681809998
RNU6-1231P	-0.028	0.404336997	0.681748794
RNU6-336P	-0.028	0.404329612	0.681748794
HLA-DQB2	-0.028	0.404328188	0.681748794
MMGT1	-0.028	0.404326956	0.681748794
EIF4A1P3	-0.028	0.404315627	0.681748794
TCTEX1D2	-0.028	0.404306017	0.681748794
TENM1	-0.028	0.404297188	0.681748794
EYA1	-0.028	0.404290349	0.681748794
IGSF5	-0.028	0.404263531	0.681748794
NAP1L1P3	-0.028	0.404178678	0.681748794
AC093165	-0.028	0.404145005	0.681748794
AC110781	-0.028	0.404133996	0.681748794
OR5T3	-0.028	0.404129423	0.681748794
RPS27P29	-0.028	0.404112721	0.681748794
CTD-2027G2	-0.028	0.404109638	0.681748794
RNU6-903P	-0.028	0.404101073	0.681748794
MRPL49P2	-0.028	0.403961676	0.681695169
CEP170P1	-0.028	0.403951385	0.681695169
CFAP69	-0.028	0.403944298	0.681695169
RP11-125A15	-0.028	0.403886708	0.68166527
CTD-2571E19	-0.028	0.403881257	0.68166527
MTX2	-0.028	0.403881209	0.68166527
MBOAT4	-0.028	0.403845057	0.68166527

RP11-124D9	-0.028	0.403698906	0.681493205
BNIP3P38	-0.028	0.403648983	0.681452945
LINC01237	-0.028	0.403590922	0.681383368
RNA5SP210	-0.028	0.403532787	0.681351496
XKRYP2	-0.028	0.403493617	0.681339851
AL589736	-0.028	0.403284392	0.681153237
RP11-424E7	-0.028	0.403254094	0.681128586
BRMS1L	-0.028	0.403226052	0.681105376
DCDC2B	-0.028	0.403188277	0.681065724
PPCDC	-0.028	0.403115384	0.681023882
RPL34P1	-0.028	0.40306786	0.680983077
SRSF8	-0.028	0.403035468	0.680952508
SNORA47	-0.028	0.403004154	0.680923756
RP11-96A1	-0.028	0.402959223	0.680871995
THUMPD1	-0.028	0.402941733	0.680866598
SPATA2P1	-0.028	0.402928957	0.680866598
HIATL1	-0.028	0.40292053	0.680866598
RP11-168J5	-0.028	0.402893342	0.680857297
BPHL	-0.028	0.402883655	0.680857297
PARD6G-AS1	-0.028	0.402816364	0.680775521
HSP90AA6P	-0.028	0.402806895	0.680775521
AC016727	-0.029	0.402737298	0.680693089
XXbac-BPG283O16	-0.029	0.402686717	0.680653025
CTD-2363C16	-0.029	0.40267768	0.680653025
RP11-705O24	-0.029	0.402634893	0.680613737
LINC01120	-0.029	0.402579606	0.68054636
ABCD1P5	-0.029	0.402543227	0.68054636
PUM1	-0.029	0.402431183	0.680510917
RP11-1O2	-0.029	0.402392379	0.680469459
RNU6-264P	-0.029	0.402385015	0.680469459
RP11-29P20	-0.029	0.402382386	0.680469459
RN7SL282P	-0.029	0.402376206	0.680469459
RNA5SP188	-0.029	0.402353397	0.680469459
FAM186B	-0.029	0.402328936	0.680469459
FAM177A1	-0.029	0.402307751	0.680469459
MIR4671	-0.029	0.402251817	0.680414285
MIR217	-0.029	0.402092119	0.68025154
RP11-643G5	-0.029	0.402040842	0.68022438
AC003002	-0.029	0.40201448	0.68022438
HMGB3P17	-0.029	0.401999682	0.68022438
TMCC1-AS1	-0.029	0.401971652	0.68022438
FAM201B	-0.029	0.401921808	0.68020503
RNU2-19P	-0.029	0.401890729	0.680176601
KRT18P64	-0.029	0.401865257	0.680157661
TMEM211	-0.029	0.401850483	0.680156828
PSAT1P2	-0.029	0.401844505	0.680156828
GSX1	-0.029	0.401769331	0.680067808
RNA5SP333	-0.029	0.401733874	0.68003196
PCGF6	-0.029	0.40170504	0.68003149
AMBP	-0.029	0.401642292	0.68002195
CENPM	-0.029	0.401639094	0.68002195
TRAV8-5	-0.029	0.401628677	0.68002195

AC006003	-0.029	0.40157124	0.68002195
RP11-293K20	-0.029	0.401569031	0.68002195
RP11-769N21	-0.029	0.401535919	0.680011066
CTB-61M7	-0.029	0.40150135	0.679976702
RP11-30G8	-0.029	0.401479142	0.679976702
PIGF	-0.029	0.401458465	0.679976702
NUS1	-0.029	0.401409406	0.679966062
RNA5SP183	-0.029	0.401397903	0.679966062
KIF20A	-0.029	0.401326628	0.67992257
RPL17P35	-0.029	0.401315549	0.67992257
SPACA6P-AS	-0.029	0.401259975	0.679882202
FAM90A21P	-0.029	0.401241288	0.679882202
FAM197Y3	-0.029	0.401241288	0.679882202
RNU6-1158P	-0.029	0.401142845	0.679828855
STPG2-AS1	-0.029	0.401140884	0.679828855
GS1-120K12	-0.029	0.401127327	0.679828855
RP11-273B19	-0.029	0.40100281	0.679688274
RP11-158G18	-0.029	0.400966815	0.679651452
UBLCP1	-0.029	0.400964362	0.679651452
HMGN2P22	-0.029	0.400948381	0.679651452
GINS1	-0.029	0.400928514	0.679651452
MIR4693	-0.029	0.400916271	0.679651452
AC018880	-0.029	0.400905196	0.679651452
RP11-9G1	-0.029	0.400836902	0.679624747
TCAF1P1	-0.029	0.400819282	0.679624747
GAGE2A	-0.029	0.400797678	0.679624747
RNU6-773P	-0.029	0.400739841	0.679556947
OR10Q2P	-0.029	0.400733048	0.679556947
ARL11	-0.029	0.40065221	0.679480925
RNU6-441P	-0.029	0.400598333	0.679413746
MKLN1	-0.029	0.400586062	0.679413746
RP5-849L7	-0.029	0.400547879	0.679400757
RP11-529P9	-0.029	0.400516522	0.679392628
RP1-159G19	-0.029	0.400487451	0.679370845
SLC25A5P3	-0.029	0.400437224	0.679358232
CNN3P1	-0.029	0.400335688	0.679234364
RPL21P122	-0.029	0.400320959	0.679233569
LYST	-0.029	0.400304696	0.679230175
RP11-324J13	-0.029	0.400275723	0.67920521
CABIN1	-0.029	0.400271026	0.67920521
SNX18P2	-0.029	0.40023862	0.679190649
AL512290	-0.029	0.400229761	0.679190649
AC005288	-0.029	0.400229747	0.679190649
RP11-665G4	-0.029	0.400148739	0.67912692
RP11-30O15	-0.029	0.400043752	0.679005124
GPR61	-0.029	0.400034079	0.679005124
TRAV37	-0.029	0.400032761	0.679005124
AC010907	-0.029	0.400016251	0.679005124
MIR153-2	-0.029	0.400013873	0.679005124
TUBB8P5	-0.029	0.399988996	0.679005124
SFTPA1	-0.029	0.399946241	0.678984801
FAM149B1P1	-0.029	0.399896123	0.678972323

VAPB	-0.029	0.399855187	0.678927023
LINC00678	-0.029	0.39985489	0.678927023
KRTAP21-4P	-0.029	0.399832593	0.678927023
LINC01491	-0.029	0.399830742	0.678927023
VN1R91P	-0.029	0.399742274	0.678904728
AC084219	-0.029	0.399739108	0.678904728
AL136380	-0.029	0.399728508	0.678904728
CALCR	-0.029	0.39959401	0.678749739
TRIM73	-0.029	0.399536599	0.678676425
RNU7-197P	-0.029	0.399495617	0.678645717
RP3-402G11	-0.029	0.399450612	0.678602972
CTD-2135D7	-0.029	0.399378652	0.678529128
TNFRSF10A	-0.029	0.399358295	0.678518747
TFP1	-0.029	0.399316462	0.678471876
RP11-752P2	-0.029	0.399300367	0.678468733
MIR26A2	-0.029	0.399291614	0.678468733
LINC01518	-0.029	0.399282835	0.678468733
RNA5SP489	-0.029	0.399236883	0.678465537
RPL21P40	-0.029	0.399180823	0.678410828
LSM12P1	-0.029	0.399126641	0.678342951
SPZ1	-0.029	0.399118028	0.678342951
C6orf89	-0.029	0.399074392	0.678342951
RNA5SP143	-0.029	0.398949017	0.678210482
C19orf80	-0.029	0.398886034	0.678151822
RMDN3	-0.029	0.398878268	0.678151822
FTLP16	-0.029	0.39887342	0.678151822
ZNF775	-0.029	0.398849862	0.678151822
MIR5094	-0.029	0.398772071	0.678054889
AK4P2	-0.029	0.398750396	0.678042241
AC016908	-0.029	0.398655055	0.677976943
AC114494	-0.029	0.398604644	0.677939626
RP5-1037N22	-0.029	0.398493026	0.677798198
BLZF2P	-0.029	0.398469166	0.677798198
ELMOD3	-0.029	0.398340292	0.677683626
RP11-192H23	-0.029	0.398332052	0.677683626
RP11-418B12	-0.029	0.398314949	0.677683626
SNORA70F	-0.029	0.39831163	0.677683626
RP11-63P12	-0.029	0.398186946	0.677567961
AC018731	-0.029	0.398152022	0.67755695
AC245470	-0.029	0.398075903	0.67745162
RPL35AP18	-0.029	0.398013107	0.677368956
RP11-745O10	-0.029	0.398001919	0.677368956
RPS15AP12	-0.029	0.397913459	0.677319142
RNU6-580P	-0.029	0.397855044	0.677269361
MBNL2	-0.029	0.397853682	0.677269361
SMG6	-0.029	0.397817391	0.677269361
RPL7P36	-0.029	0.397804901	0.677269361
AC006946	-0.029	0.397725911	0.677243188
OR8B5P	-0.029	0.397716228	0.677243188
RNA5SP384	-0.029	0.397630012	0.677128311
RP11-20F18	-0.029	0.397542104	0.677027026
RP11-480O10	-0.029	0.397520503	0.677014447

RP3-467N11	-0.029	0.397452155	0.67692225
TMEM138	-0.029	0.397415277	0.676883646
NAV2-AS4	-0.029	0.397314609	0.676747979
HMGB1P31	-0.029	0.397308918	0.676747979
SUMO2P14	-0.029	0.397127116	0.676513812
RP1-149C7	-0.029	0.397110528	0.676513812
RP11-134J21	-0.029	0.397092351	0.676513812
EPB41L5	-0.029	0.397043257	0.67646774
RNY4P3	-0.029	0.397039327	0.67646774
MARK2P11	-0.029	0.39702086	0.67646774
ADCY9	-0.029	0.39701493	0.67646774
AC005609	-0.029	0.396666046	0.676091106
C3orf35	-0.029	0.39665653	0.676091106
OR2AG2	-0.029	0.396617706	0.676091106
KTI12	-0.029	0.396564906	0.676063886
AC113608	-0.029	0.39651003	0.67604293
CTC-503K11	-0.029	0.396497694	0.67604293
COX6CP18	-0.029	0.396458618	0.676030069
CTD-2024I7	-0.029	0.396395433	0.675983417
RP11-4B14	-0.029	0.39638139	0.675983417
RN7SKP262	-0.029	0.396378003	0.675983417
AL136456	-0.029	0.396359267	0.675983417
OR10G3	-0.029	0.396353872	0.675983417
ZNF680P1	-0.029	0.396349051	0.675983417
RNU6-536P	-0.029	0.396346835	0.675983417
RN7SL252P	-0.029	0.396312003	0.675983417
RP11-44N17	-0.029	0.396171126	0.675876427
AFM	-0.029	0.396140391	0.675869843
RP11-165E7	-0.029	0.396088975	0.675833111
CXXC5	-0.029	0.39608234	0.675833111
MIF-AS1	-0.029	0.396034885	0.675813453
AC091492	-0.029	0.396027728	0.675813453
OR7E93P	-0.029	0.396009412	0.675813453
RP11-476K15	-0.029	0.395976658	0.675813453
MAT1A	-0.029	0.395939464	0.675813453
UBE2V1P6	-0.029	0.395891259	0.67578628
RNU6-1169P	-0.029	0.395890121	0.67578628
PTPRE	-0.029	0.395831461	0.67576526
RP11-52J3	-0.029	0.395829048	0.67576526
RNU6ATAC34P	-0.029	0.395815961	0.67576526
RN7SL182P	-0.029	0.395785318	0.67576526
HIGD1AP2	-0.029	0.395729159	0.675727543
MIR1301	-0.029	0.395672182	0.675654477
CTA-109P11	-0.029	0.395671755	0.675654477
OR51N1P	-0.029	0.395626122	0.675624272
HOTAIR_4	-0.029	0.395604397	0.675611395
RP5-855F16	-0.029	0.395460165	0.675461959
RN7SL285P	-0.029	0.395440899	0.675453277
RNU6-279P	-0.029	0.395427305	0.675453277
GS1-166A23	-0.029	0.39541544	0.675453277
NUDCD3	-0.029	0.395407465	0.675453277
RP11-436F23	-0.029	0.39529505	0.675325252

APOA1	-0.029	0.395215566	0.675286352
GPR50	-0.029	0.395183925	0.6752632
BPIFA3	-0.029	0.395181333	0.6752632
PLCXD3	-0.029	0.395165986	0.6752632
LCNL1	-0.029	0.3951248	0.6752632
TFB2M	-0.029	0.395117057	0.6752632
MIR3684	-0.029	0.395049922	0.67522134
RP11-24P24	-0.029	0.39503626	0.67522134
GRIP2	-0.029	0.395028684	0.67522134
LTB4R	-0.029	0.394990423	0.67522134
RP11-342A23	-0.029	0.394903244	0.675116006
RNU4-64P	-0.029	0.394791461	0.674997592
EEF1A1P38	-0.029	0.394738422	0.674931137
CDHR5	-0.029	0.394734693	0.674931137
UBL5P3	-0.029	0.3947284	0.674931137
RP11-1102P16	-0.029	0.394566022	0.674733257
UBE2Q2P10	-0.029	0.394506025	0.674679108
CYCSP25	-0.029	0.394468804	0.674639677
NOL8P1	-0.029	0.394463452	0.674639677
RNA5SP207	-0.029	0.394439323	0.674637709
RNU6-516P	-0.029	0.394393815	0.674584099
NPIPA7	-0.029	0.39430814	0.674474297
RNU6-983P	-0.029	0.394235474	0.674433859
AAR2	-0.029	0.394220521	0.674433017
FAM83H-AS1	-0.029	0.394195652	0.674432586
LONP2	-0.029	0.394175333	0.674428386
RP11-380O24	-0.029	0.394121695	0.67438049
CA5A	-0.029	0.394079674	0.674337392
AP000711	-0.029	0.394051173	0.67433072
ACRV1	-0.029	0.394003671	0.674328477
HSPD1P2	-0.029	0.393928123	0.674247635
RPS6KA5	-0.029	0.393914876	0.674247635
RP11-469L4	-0.029	0.393898387	0.6742452
AC090952	-0.029	0.393856648	0.674197986
SNORA80A	-0.029	0.393734929	0.674038082
RNU6-314P	-0.029	0.393671717	0.67397832
KDM5C	-0.029	0.393619373	0.67391293
RP11-441O15	-0.029	0.39361604	0.67391293
CTD-2203A3	-0.029	0.393558362	0.673881146
RP11-180C16	-0.029	0.393487981	0.67378486
TDGF1	-0.029	0.393463691	0.673783791
ZDHHC11B	-0.029	0.393392413	0.673718104
ANKRD34B	-0.029	0.393366134	0.673718104
ASS1P4	-0.029	0.393355068	0.673718104
RFC3P1	-0.029	0.393247904	0.673591726
RP11-17J14	-0.029	0.39324278	0.673591726
SLC35A3	-0.029	0.393237691	0.673591726
TSPYL4	-0.029	0.393221035	0.673591726
RP11-677N16	-0.029	0.393184946	0.673591726
SIGLEC9	-0.029	0.393175613	0.673591726
AC007631	-0.029	0.393126049	0.67355257
RP11-552I14	-0.029	0.393105259	0.67354118

C6orf195	-0.029	0.393101851	0.67354118
RNU6-1055P	-0.029	0.393081956	0.67354118
RP11-63I8	-0.029	0.39307962	0.67354118
USP21	-0.029	0.392938863	0.673352975
RAB6C	-0.029	0.392905524	0.673320069
FTH1P25	-0.029	0.392846217	0.67324266
AC002539	-0.029	0.392766407	0.673178555
RP4-705F19	-0.029	0.392647152	0.673022597
RPIA	-0.029	0.392626255	0.673022597
HSD3BP3	-0.029	0.392574945	0.67297149
RP11-651L5	-0.029	0.392571496	0.67297149
ANKRD19P	-0.029	0.392424184	0.672809915
ZNF720P1	-0.029	0.392416309	0.672809915
ANP32BP3	-0.029	0.392390147	0.672799998
RP11-331O9	-0.029	0.392384475	0.672799998
AC004691	-0.029	0.392382098	0.672799998
BATF	-0.029	0.392338595	0.672784269
CYP4F30P	-0.029	0.392335994	0.672784269
UMPS	-0.029	0.392281744	0.672759448
FLVCR1	-0.029	0.392257553	0.672743223
RP11-298E2	-0.029	0.392176607	0.672651803
POU2F3	-0.029	0.392128149	0.672592911
TMEM241	-0.029	0.392041995	0.672469354
EEF1A1P5	-0.029	0.392029904	0.672469354
RP11-805J14	-0.029	0.392019133	0.672469354
RP11-466J24	-0.029	0.392015388	0.672469354
DDX10P1	-0.029	0.39198236	0.672463938
RP4-710M3	-0.029	0.391819331	0.672281118
SNRNP40	-0.029	0.39178209	0.672265658
AC015726	-0.029	0.391739179	0.672216244
DXO	-0.029	0.39173133	0.672216244
LINC01249	-0.029	0.391722023	0.672216244
CTB-119C2	-0.029	0.391654291	0.67214323
RP11-407P18	-0.029	0.391564678	0.67203787
KRT18P35	-0.029	0.391553699	0.67203787
DMRTC1B	-0.029	0.391517829	0.672030116
AC138123	-0.029	0.391476153	0.671982799
RP11-140H17	-0.029	0.391394897	0.671867536
RNU6-657P	-0.029	0.391392575	0.671867536
RP11-1148O4	-0.029	0.391382055	0.671867536
RP5-1033K19	-0.029	0.391366818	0.671867536
MARK2P10	-0.029	0.391289233	0.671867536
HSPE1P9	-0.029	0.391259884	0.671867536
URI1	-0.029	0.391224251	0.671840968
AC024158	-0.029	0.391221186	0.671840968
AC010184	-0.029	0.391219166	0.671840968
KCNH7	-0.029	0.391200424	0.671840968
C8orf44-SGK3	-0.029	0.391150202	0.671840968
TRIP4	-0.029	0.391057627	0.671797058
CTD-2034I4	-0.029	0.39101925	0.671779591
RP11-391M7	-0.029	0.391016625	0.671779591
MPP2	-0.029	0.391003303	0.671779591

AC090420	-0.029	0.390986529	0.671779591
TDRD12	-0.029	0.390835228	0.671639772
UBE2V1P11	-0.029	0.390773104	0.671574734
FAM219A	-0.029	0.390719682	0.671507156
RP11-508M8	-0.029	0.390663626	0.671459276
SLC35A1	-0.029	0.390629795	0.671425358
SPANXN2	-0.029	0.390618933	0.671425358
AL096855	-0.029	0.39059519	0.671425358
ZSCAN5B	-0.029	0.390577461	0.671425358
RP1-59B16	-0.029	0.390536742	0.671425358
OR52X1P	-0.029	0.390536415	0.671425358
AMY1C	-0.029	0.390459755	0.671399622
RP3-334F4	-0.029	0.390444908	0.671398334
MIR4421	-0.029	0.390412717	0.671391459
LA16c-60G3	-0.029	0.390329038	0.671271795
RNU11-5P	-0.029	0.390326403	0.671271795
CCDC183	-0.029	0.390245651	0.671176861
CICP13	-0.029	0.390168527	0.671116921
RNU7-170P	-0.029	0.390144217	0.671116921
CADM2	-0.029	0.390137417	0.671116921
LINC01531	-0.029	0.390063678	0.671057751
AC007250	-0.029	0.390021149	0.671008823
AC087650	-0.029	0.389931727	0.670889861
MIR1256	-0.029	0.389765338	0.670714086
ZDHHC21	-0.029	0.389713235	0.670672891
CSN1S2AP	-0.029	0.389684624	0.670647886
LINC00638	-0.029	0.389672576	0.670647886
AC105179	-0.029	0.389655397	0.670646054
RP11-158J3	-0.029	0.389586866	0.670576569
AC092812	-0.029	0.389584928	0.670576569
SCGB1D5P	-0.029	0.389513719	0.670547599
LLPHP2	-0.029	0.389499531	0.670547413
LINC00935	-0.029	0.389449551	0.670509841
KLHL22	-0.029	0.38942356	0.670509841
B3GALT5-AS1	-0.029	0.38939648	0.670491185
SAPCD2P1	-0.029	0.389386084	0.670491185
RP11-371A19	-0.029	0.389364303	0.67048426
MIR653	-0.029	0.389363636	0.67048426
RNU6-466P	-0.029	0.389333582	0.670479842
AC062032	-0.029	0.389252272	0.670412539
RPS11P7	-0.029	0.389187386	0.670325026
RNU6-420P	-0.029	0.38909007	0.670205887
BMP2KL	-0.029	0.389036593	0.670162247
PGK2	-0.029	0.389024822	0.670162247
SNORD75	-0.029	0.389023864	0.670162247
IPMK	-0.029	0.388986283	0.670148299
MINK1	-0.029	0.388922207	0.670086385
RP5-855D21	-0.029	0.388887614	0.670051023
MIR4767	-0.029	0.388723147	0.669864582
IMMP1L	-0.029	0.388700244	0.669849351
RP11-789L4	-0.029	0.388691236	0.669849351
MIR8052	-0.029	0.388644116	0.669801096

AL732579	-0.029	0.388603124	0.669754685
TPT1P8	-0.029	0.388598544	0.669754685
HCG20	-0.029	0.388591379	0.669754685
RP11-567J20	-0.029	0.388534686	0.669709438
ESRRB	-0.029	0.388414302	0.669550399
PAPPA-AS2	-0.029	0.388379527	0.669514685
PPP1R10P1	-0.029	0.38835675	0.669499653
AC005772	-0.029	0.388346969	0.669499653
OR8B1P	-0.029	0.388342982	0.669499653
RNU6-50P	-0.029	0.388197238	0.669379543
MIR3142	-0.029	0.388188012	0.669379543
MIR4689	-0.029	0.388177224	0.669379543
C15orf41	-0.029	0.388016795	0.669252739
RP11-379K17	-0.029	0.387909618	0.669140578
MRPL10	-0.029	0.387869321	0.669119537
RP11-269G24	-0.029	0.387752806	0.668942764
RNU6-532P	-0.029	0.387723386	0.66891624
AL021917	-0.029	0.387658723	0.668865873
RNY4P27	-0.029	0.387644273	0.668865873
AC013436	-0.029	0.387635896	0.668865873
PHACTR2	-0.029	0.38738179	0.668569086
MT1A	-0.029	0.387376387	0.668569086
ZNF12	-0.029	0.387354482	0.668569086
CYCSP42	-0.029	0.387347707	0.668569086
TTC39A-AS1	-0.029	0.387301406	0.668551486
CTD-2054N24	-0.029	0.387161376	0.668358218
ZNF70	-0.029	0.387134864	0.668336674
MIR4439	-0.029	0.387020986	0.668188519
AMELY	-0.029	0.386929707	0.668055144
RP11-160E2	-0.029	0.386919408	0.668055144
MRRFP1	-0.029	0.386869583	0.668048209
CPOX	-0.029	0.386851153	0.668040605
RNU6-756P	-0.029	0.386841854	0.668040605
GNPATP	-0.029	0.386779735	0.667989935
YWHAQP7	-0.029	0.38677134	0.667989935
PHKG1P2	-0.029	0.38667917	0.667961584
FABP2	-0.029	0.386658182	0.667961584
AP000569	-0.029	0.386641388	0.667961584
KLHL36	-0.029	0.386568768	0.667891994
RNA5SP174	-0.030	0.386449602	0.667710328
AL050310	-0.030	0.386448346	0.667710328
PPP6R2	-0.030	0.386442604	0.667710328
INO80B-WBP1	-0.030	0.386424857	0.667710328
AC079988	-0.030	0.38642331	0.667710328
PDZK1P2	-0.030	0.386401329	0.667710328
AC026366	-0.030	0.386323072	0.667647093
TTK	-0.030	0.386168188	0.667466241
SAP25	-0.030	0.386110698	0.667402143
KCND3-AS1	-0.030	0.386105343	0.667402143
PSAT1P4	-0.030	0.385993201	0.667302538
ARPC3P2	-0.030	0.38593639	0.667235097
FPGT-TNNI3K	-0.030	0.385875456	0.667178187

SPIN4-AS1		-0.030	0.385867839	0.667178187
NT5C1B		-0.030	0.385864814	0.667178187
AC007790		-0.030	0.385809917	0.667178187
CCDC42		-0.030	0.385762279	0.667128211
NLN		-0.030	0.385717875	0.667099472
C10orf105		-0.030	0.385690845	0.667076946
CTD-3247F14		-0.030	0.385673208	0.667076946
RP11-107G24		-0.030	0.385605213	0.667025732
TAS2R41		-0.030	0.385578755	0.667004191
HADH		-0.030	0.385549091	0.6669771
ASCL3		-0.030	0.385542483	0.6669771
PIGG		-0.030	0.385533531	0.6669771
RP11-712C7		-0.030	0.385419584	0.666874174
GNG5P2		-0.030	0.385418619	0.666874174
RP11-665I14		-0.030	0.38539813	0.666874174
SYNE3		-0.030	0.385369283	0.666859821
HAX1		-0.030	0.38533249	0.66684461
GSX2		-0.030	0.385289495	0.666794432
	5-Mar	-0.030	0.385184742	0.666661592
RP11-574K11		-0.030	0.385172182	0.666661592
CTA-722E9		-0.030	0.385154008	0.666656852
AC010971		-0.030	0.385118162	0.666619034
AC096922		-0.030	0.385098188	0.666608687
UGT2B15		-0.030	0.385087933	0.666608687
NLRP9P		-0.030	0.384992469	0.666474133
RNU4-51P		-0.030	0.384838113	0.666287844
RP11-23I7		-0.030	0.38479203	0.666248228
EMC3		-0.030	0.384781152	0.666248228
BMS1P20		-0.030	0.384780853	0.666248228
NAA35		-0.030	0.384680743	0.666182123
RP3-327A19		-0.030	0.384574713	0.666089893
RPA2P3		-0.030	0.384529498	0.6660358
DALRD3		-0.030	0.384464739	0.665947852
ARHGEF34P		-0.030	0.384402013	0.665887637
TCAF1		-0.030	0.384390365	0.665887637
H2AFZP1		-0.030	0.384344483	0.665836414
MIR518B		-0.030	0.384296363	0.665801487
AC005741		-0.030	0.384223303	0.665723343
ZCWPW1		-0.030	0.384187299	0.665685178
LINC01363		-0.030	0.384158701	0.665659843
RP11-1O10		-0.030	0.384103058	0.665587642
AC023128		-0.030	0.383914656	0.665503306
RNU6-1200P		-0.030	0.38390516	0.665503306
AC107977		-0.030	0.383891612	0.665503306
RP11-190I17		-0.030	0.383854762	0.665477331
OR1N2		-0.030	0.383740574	0.665373903
LINC01202		-0.030	0.383714431	0.665373903
C11orf65		-0.030	0.383705281	0.665373903
ACP1		-0.030	0.383649194	0.665333727
CFAP43		-0.030	0.383637857	0.665333727
HNRNPA1P56		-0.030	0.38359613	0.665290153
RP11-535N6		-0.030	0.38357556	0.665278704

FOLR2	-0.030	0.383513472	0.665219466
POLR3GP2	-0.030	0.383505407	0.665219466
RNU7-51P	-0.030	0.383495402	0.665219466
CECR9	-0.030	0.383494942	0.665219466
EHHADH-AS1	-0.030	0.383392129	0.665148666
RP11-767N15	-0.030	0.383367263	0.665148666
NDC1	-0.030	0.383277594	0.665076765
RP11-168P8	-0.030	0.383271211	0.665076765
TMEM18	-0.030	0.38309903	0.664863809
LMBR1	-0.030	0.383085916	0.664863809
ZNF840P	-0.030	0.382994792	0.664763819
RP11-93H10	-0.030	0.382989331	0.664763819
SPATA1	-0.030	0.382926477	0.66475813
RP11-299G20	-0.030	0.382916048	0.66475813
ZNF772	-0.030	0.382895488	0.66475813
MINA	-0.030	0.382875226	0.66475813
CTA-299D3	-0.030	0.38267365	0.664585688
SLC25A21	-0.030	0.382668769	0.664585688
YAE1D1	-0.030	0.38265329	0.664585688
RP11-452H21	-0.030	0.382522338	0.664395586
RP11-234A1	-0.030	0.382436791	0.664343913
MTMR9LP	-0.030	0.382416843	0.66433349
RP11-84A14	-0.030	0.382411737	0.66433349
RN7SL474P	-0.030	0.38239298	0.66433349
DIO1	-0.030	0.382375288	0.66433349
MORF4L1P6	-0.030	0.382315153	0.664302217
RP11-93B21	-0.030	0.382261117	0.664232559
RP11-98C1	-0.030	0.382222039	0.664213122
RP11-632F7	-0.030	0.382200715	0.664200301
ACTG1P1	-0.030	0.382200426	0.664200301
RP11-603B13	-0.030	0.382199829	0.664200301
RPL7AP10	-0.030	0.382134251	0.664200301
GPR50-AS1	-0.030	0.382116866	0.6642
AL137006	-0.030	0.382078086	0.66415683
EIF4BP7	-0.030	0.382067399	0.66415683
RNU6-65P	-0.030	0.381998692	0.664067296
OR2L13	-0.030	0.381973553	0.664047832
HIST2H2BA	-0.030	0.381946092	0.664024329
TBC1D22B	-0.030	0.381943622	0.664024329
RP11-64P14	-0.030	0.381928328	0.664024329
H3F3BP1	-0.030	0.381918891	0.664024329
HLF	-0.030	0.381912087	0.664024329
RP11-363G15	-0.030	0.381885055	0.664024329
RP11-303G3	-0.030	0.381714233	0.663766613
FSBP	-0.030	0.381607931	0.663654456
RP11-295B17	-0.030	0.381518543	0.663547459
PMS2P4	-0.030	0.381509706	0.663547459
RN7SKP120	-0.030	0.38149964	0.663547459
MIR2909	-0.030	0.381452809	0.663505823
WBP1LP5	-0.030	0.381404949	0.66349527
AC016831	-0.030	0.381392587	0.66349527
RP11-109N23	-0.030	0.381367314	0.663478267

AP000721	-0.030	0.381366127	0.663478267
LA16c-360H6	-0.030	0.381355721	0.663478267
RNU2-41P	-0.030	0.381245299	0.663435634
RN7SL505P	-0.030	0.38109797	0.663324672
H2AFB3	-0.030	0.381071768	0.663303308
KB-1254G8	-0.030	0.381058146	0.663303308
RN7SKP163	-0.030	0.381025317	0.663270934
CELF4	-0.030	0.380948351	0.663185434
CTXN2	-0.030	0.380885952	0.663125283
HAO1	-0.030	0.380831318	0.663078642
CYLC1	-0.030	0.380802651	0.663052968
C7orf33	-0.030	0.380767706	0.663035587
ARF4P2	-0.030	0.380647217	0.662879264
MAPKAPK3	-0.030	0.3805273	0.662718898
RPS18P12	-0.030	0.380432617	0.662602459
KRT8P44	-0.030	0.380411851	0.662590522
TNPO1P2	-0.030	0.380369713	0.662541356
RP11-267C16	-0.030	0.380304048	0.662499667
FUT10	-0.030	0.380246262	0.662423231
CMB9-52H4	-0.030	0.38020682	0.662423231
FAM198B	-0.030	0.380199385	0.662423231
NUS1P2	-0.030	0.380102271	0.662293511
MIR184	-0.030	0.380043202	0.662237713
RNU6-1289P	-0.030	0.380018528	0.662237713
AC133680	-0.030	0.380007585	0.662237713
RP11-171I2	-0.030	0.379974545	0.662237713
RNU6-774P	-0.030	0.379826973	0.662065699
AC138965	-0.030	0.379817814	0.662065699
TCEA1P4	-0.030	0.379793769	0.662065699
RP11-455J15	-0.030	0.379714544	0.662029673
THEMIS2	-0.030	0.379532814	0.661737053
IL6RP1	-0.030	0.379525766	0.661737053
CBL	-0.030	0.379389986	0.661560681
SNORA20	-0.030	0.379294836	0.661491638
BX088563	-0.030	0.379258616	0.661452692
RP11-517A5	-0.030	0.379174508	0.661363936
RFPL4AP1	-0.030	0.379069719	0.661220096
MIR670	-0.030	0.378994844	0.661137917
XXyac-YX60D10	-0.030	0.378981309	0.661137917
ATP2C2	-0.030	0.378873474	0.660998826
AIG1P1	-0.030	0.37885124	0.660984248
RPL35AP22	-0.030	0.378815258	0.660957003
BEND2	-0.030	0.378788251	0.660946988
RNA5SP418	-0.030	0.378788194	0.660946988
HIGD1AP12	-0.030	0.378748161	0.660946988
AC010747	-0.030	0.378747938	0.660946988
IRF7	-0.030	0.378730207	0.660946988
OR6A2	-0.030	0.378606033	0.660919604
RNU6-1211P	-0.030	0.378560683	0.660871296
MIR1296	-0.030	0.378488926	0.660808238
EPHX2	-0.030	0.378477498	0.660808238
CTB-91J4	-0.030	0.378469494	0.660808238

RN7SKP154	-0.030	0.378442647	0.660803928
AC134300	-0.030	0.378423734	0.660795131
RP11-644K8	-0.030	0.378413256	0.660795131
MIR4725	-0.030	0.378367938	0.660754715
SNRPCP14	-0.030	0.378306842	0.660712136
AK4P4	-0.030	0.378285191	0.660700673
MIR548AW	-0.030	0.378220512	0.660658268
ZDHHC8P1	-0.030	0.378210403	0.660658268
FGF12-AS1	-0.030	0.378171966	0.660621927
AC092375	-0.030	0.378084374	0.660517369
WDR90	-0.030	0.378037725	0.660517369
RNA5SP307	-0.030	0.378035372	0.660517369
RANP3	-0.030	0.378015448	0.660517369
AC005052	-0.030	0.377907309	0.660457967
QDPR	-0.030	0.377798599	0.660406027
INPP4B	-0.030	0.377763137	0.660392193
RP11-3L23	-0.030	0.377761683	0.660392193
PLCB1	-0.030	0.377748657	0.660392193
RNU6ATAC7P	-0.030	0.3777182	0.660392193
COQ10BP2	-0.030	0.37770744	0.660392193
CTB-158E9	-0.030	0.377696873	0.660392193
MYRFL	-0.030	0.37761096	0.660320062
RP11-32K4	-0.030	0.377610803	0.660320062
RP11-577G20	-0.030	0.377608296	0.660320062
COX6CP7	-0.030	0.377453414	0.660117275
CHRNB2	-0.030	0.377440518	0.660117275
RP5-1100I6	-0.030	0.377400262	0.660077348
TAF1C	-0.030	0.377343364	0.660021756
LLNLR-276H7	-0.030	0.377291726	0.65998036
RPL5P5	-0.030	0.377268314	0.65998036
RP11-65B7	-0.030	0.377193728	0.659929678
PAPLN	-0.030	0.377188938	0.659929678
SHCBP1L	-0.030	0.37705248	0.659779501
CEP164P1	-0.030	0.376975244	0.659692826
CTC-251I16	-0.030	0.376897024	0.659652895
NEU4	-0.030	0.376891937	0.659652895
AC074375	-0.030	0.376858293	0.659633588
MEI4	-0.030	0.376840211	0.659633588
AC092573	-0.030	0.376825699	0.659633588
MRPL45	-0.030	0.376808504	0.659633588
ZCCHC18	-0.030	0.376774279	0.659607741
VN2R10P	-0.030	0.376720147	0.659561461
OPTN	-0.030	0.376717341	0.659561461
AC018688	-0.030	0.376712086	0.659561461
RNU6-540P	-0.030	0.376706333	0.659561461
MTAPP2	-0.030	0.376551457	0.659448275
SRCIN1	-0.030	0.376541543	0.659448275
AC087499	-0.030	0.376509917	0.659445225
HMGN1P20	-0.030	0.376451924	0.659382729
RAB22A	-0.030	0.376396755	0.659310344
PRDM7	-0.030	0.376347353	0.659248056
EI24P1	-0.030	0.376347209	0.659248056

CDRT15	-0.030	0.37634379	0.659248056
RP11-642D6	-0.030	0.376330059	0.659248056
MTND5P8	-0.030	0.376293628	0.659248056
TM9SF2	-0.030	0.376255539	0.659232702
AC073873	-0.030	0.376237494	0.659225338
RP11-488P3	-0.030	0.376184602	0.659156913
WDR66	-0.030	0.376177132	0.659156913
CHD1L	-0.030	0.376108755	0.65912101
RNA5SP38	-0.030	0.3761085	0.65912101
NAA11	-0.030	0.376098441	0.65912101
RP11-536N17	-0.030	0.376017317	0.659068715
RN7SL118P	-0.030	0.376011623	0.659068715
ALDH6A1	-0.030	0.375945802	0.659053693
RN7SKP35	-0.030	0.375824157	0.658913202
AC012497	-0.030	0.375794959	0.658910521
RN7SKP225	-0.030	0.375748867	0.658853961
AC093074	-0.030	0.375692564	0.65877949
RNA5SP290	-0.030	0.37567247	0.65877949
TAF5L	-0.030	0.375639462	0.65877949
RPL3P8	-0.030	0.375593629	0.658727271
IQCE	-0.030	0.375582335	0.658727271
ATP5A1P2	-0.030	0.375578896	0.658727271
DENND1A	-0.030	0.375568202	0.658727271
SNORD94	-0.030	0.375497793	0.658656209
RPL17	-0.030	0.375411575	0.658624735
GGTA2P	-0.030	0.375400946	0.658624735
MIR548F2	-0.030	0.375324481	0.658624735
RN7SL862P	-0.030	0.375324466	0.658624735
ST13P21	-0.030	0.375314949	0.658624735
FAAH2	-0.030	0.375269998	0.658624735
SFXN4	-0.030	0.375267643	0.658624735
MIR196A2	-0.030	0.375217264	0.658624735
EEF1A1P8	-0.030	0.375208482	0.658624735
RP4-799G3	-0.030	0.375093281	0.658528741
TBL1XR1	-0.030	0.375040483	0.65846032
AC009967	-0.030	0.374930498	0.658291485
RP11-331H2	-0.030	0.374902076	0.65826585
CHRNG	-0.030	0.374884123	0.658258595
AC133644	-0.030	0.374785762	0.658110147
RNA5SP404	-0.030	0.374732004	0.658064273
OSBPL9P3	-0.030	0.374697756	0.658028392
KIF6	-0.030	0.37467785	0.658019248
QRSL1	-0.030	0.374668442	0.658019248
RPS27P1	-0.030	0.374637073	0.658019248
PCDH8P1	-0.030	0.374617184	0.658019248
HNRNPRP1	-0.030	0.374548065	0.6579596
SWSAP1	-0.030	0.37452351	0.6579596
NAPEPLD	-0.030	0.374456495	0.657895805
RP5-1173P7	-0.030	0.374422897	0.657861043
AC073476	-0.030	0.374408082	0.657859283
ENO3	-0.030	0.374357749	0.657795111
Hammerhead_HH9	-0.030	0.374232167	0.657622972

RP1-57E3	-0.030	0.374212769	0.657622972
CTA-21C21	-0.030	0.374189551	0.657622972
TOMM34	-0.030	0.37412476	0.657604072
RP11-552D4	-0.030	0.3740559	0.657520736
AL355885	-0.030	0.374049883	0.657520736
GC	-0.030	0.374043503	0.657520736
CPLX4	-0.030	0.374034282	0.657520736
OR4S2	-0.030	0.373996452	0.657520736
UPF3AP3	-0.030	0.373976891	0.657514023
PPP2R5D	-0.030	0.373975847	0.657514023
CD300E	-0.030	0.373908011	0.657465733
AC093375	-0.030	0.373855084	0.657421212
AL022237	-0.030	0.373776735	0.657307704
PCYOX1	-0.030	0.373631754	0.657101272
RP11-80N2	-0.030	0.373493986	0.65693249
RUFY1	-0.030	0.373463595	0.656926823
AC104837	-0.030	0.373456093	0.656926823
CBY3	-0.030	0.373440072	0.656926823
CT978678	-0.030	0.373428913	0.656926823
RP11-268I9	-0.030	0.373417372	0.656926823
UQCRHP1	-0.030	0.373339813	0.656830382
ARPC3	-0.030	0.373328339	0.656830382
RP11-380F14	-0.030	0.373266223	0.656749434
RPL36AP35	-0.030	0.373178553	0.656643697
RP11-331H13	-0.030	0.373134603	0.656590621
PPIL4	-0.030	0.37308255	0.656523282
RN7SKP18	-0.030	0.372987762	0.656429242
ACTBP13	-0.030	0.372904702	0.656341668
AC117947	-0.030	0.372901064	0.656341668
EDDM3B	-0.030	0.372839968	0.656314649
LINC01555	-0.030	0.372828104	0.656314649
ABTB1	-0.030	0.372642258	0.656104784
AJ003147	-0.030	0.372581894	0.656054344
RP5-919F19	-0.030	0.372564725	0.656048366
LINC00208	-0.030	0.372382802	0.655800758
RP11-459O16	-0.030	0.372352657	0.655771918
RP11-253D19	-0.030	0.372351321	0.655771918
TTC39A	-0.030	0.372307417	0.65574074
ARCN1	-0.030	0.372171542	0.655549911
RP11-342D11	-0.030	0.372163263	0.655549911
HLA-W	-0.030	0.372159983	0.655549911
RP11-712B9	-0.030	0.372105958	0.655515371
HSD17B4	-0.030	0.372064094	0.655515371
ODF3	-0.030	0.372053286	0.655515371
MIR4730	-0.030	0.372018316	0.6554982
PFAS	-0.030	0.371936176	0.655377714
LINC01532	-0.030	0.371897703	0.655334169
MIR3122	-0.030	0.371884795	0.655334169
HNRNPKP3	-0.030	0.37182796	0.655259759
MZT1P2	-0.030	0.371814195	0.655259759
HSPB8	-0.030	0.371785754	0.655259759
UBE2V1P3	-0.030	0.371783102	0.655259759

UCMA	-0.030	0.371759719	0.655259759
NAT1	-0.030	0.371619572	0.655145769
KHSRPP1	-0.030	0.371578351	0.655110762
TXNDC8	-0.030	0.371455545	0.654918492
GGPS1	-0.030	0.371373018	0.65482147
FBXO40	-0.030	0.371281895	0.654709275
CTD-2525I3	-0.030	0.371234422	0.6546498
APOC1P1	-0.030	0.371181635	0.65458095
RPL10P14	-0.030	0.371138723	0.654529511
RNU6-208P	-0.030	0.371132405	0.654529511
SLC35G4	-0.030	0.371113989	0.654529511
AC019178	-0.030	0.371026274	0.654525088
RP11-64I5	-0.030	0.37097513	0.654483349
LINC00554	-0.030	0.37095828	0.654483349
SREBF2	-0.030	0.370874954	0.654388121
HNRNPH2	-0.030	0.370841356	0.654385289
RPSAP4	-0.030	0.370784052	0.65438096
RP11-35L17	-0.030	0.3707358	0.654338198
MAPK6PS3	-0.030	0.370584707	0.654255026
RP5-1098D14	-0.031	0.370552563	0.654222528
AC005351	-0.031	0.370517427	0.654184746
RP11-461L13	-0.031	0.370512743	0.654184746
SRPK2	-0.031	0.370460802	0.65417711
FSCN3	-0.031	0.370413449	0.654146662
RP11-92K2	-0.031	0.370360373	0.654077184
RP11-240A16	-0.031	0.370290864	0.653978676
OR11H7	-0.031	0.370264505	0.653956374
LINC01546	-0.031	0.370240693	0.653938568
RP4-597A16	-0.031	0.370227433	0.653938568
AC004702	-0.031	0.370212109	0.653936584
RP11-214D15	-0.031	0.370125598	0.653808021
RP11-635O16	-0.031	0.370073416	0.65376434
RP11-290K4	-0.031	0.370056721	0.653759096
VN1R67P	-0.031	0.37003541	0.653748381
CSE1L-AS1	-0.031	0.370025932	0.653748381
KRTAP9-10P	-0.031	0.370011622	0.653748381
AL356270	-0.031	0.369844453	0.653553791
LINC00428	-0.031	0.369759389	0.653427718
LINC01267	-0.031	0.369730223	0.65340042
MTND2P29	-0.031	0.369674948	0.653326979
HIRA	-0.031	0.369641812	0.653316903
AP003025	-0.031	0.369603545	0.653311774
GPR89A	-0.031	0.369602452	0.653311774
RP11-496D1	-0.031	0.369591285	0.653311774
DOLK	-0.031	0.369579712	0.653311774
RNU2-27P	-0.031	0.369563927	0.653311774
MGC15885	-0.031	0.369473389	0.653237401
RP11-536P6	-0.031	0.369465083	0.653237401
RP1-273G13	-0.031	0.369401606	0.65315898
AC093928	-0.031	0.369370517	0.653128258
AL353626	-0.031	0.369368029	0.653128258
RP11-119F7	-0.031	0.369330209	0.653128258

OR7E145P	-0.031	0.369293251	0.653128258
YWHAQP6	-0.031	0.369194903	0.653084447
TGIF2LX	-0.031	0.369188052	0.653084447
RNU6-876P	-0.031	0.369157076	0.653084447
AC000068	-0.031	0.369127711	0.65306261
AC078927	-0.031	0.36894663	0.65279074
LINC00052	-0.031	0.368890471	0.652739874
RPS10P18	-0.031	0.368855392	0.652727357
TDGF1P2	-0.031	0.368801918	0.652694136
SLC4A8	-0.031	0.368800072	0.652694136
ATG3	-0.031	0.368791046	0.652694136
RP1-116K23	-0.031	0.368751498	0.652694136
GLDN	-0.031	0.36873311	0.652694136
KATNAL2	-0.031	0.368657714	0.652618977
RP11-72L22	-0.031	0.368617781	0.65257254
RP11-107C16	-0.031	0.368591571	0.652550396
RNA5SP424	-0.031	0.368576527	0.652548018
GDPD2	-0.031	0.368559264	0.652541712
OR2A13P	-0.031	0.368521473	0.65249906
RP11-180D21	-0.031	0.368470409	0.652432901
CLYBL-AS1	-0.031	0.368444777	0.65241177
AC078785	-0.031	0.368439878	0.65241177
MTND2P8	-0.031	0.368377663	0.652408723
MTND6P2	-0.031	0.368373094	0.652408723
FLNB	-0.031	0.368302116	0.652401713
LSMEM2	-0.031	0.368281151	0.65238884
FYCO1	-0.031	0.368280007	0.65238884
LINC01012	-0.031	0.368268805	0.65238884
FMR1-AS1	-0.031	0.368265074	0.65238884
AC092198	-0.031	0.368210938	0.652385787
RP4-697P8	-0.031	0.368157941	0.652316155
RHOXF2	-0.031	0.368152557	0.652316155
DGKA	-0.031	0.368137193	0.652316155
VPS26A	-0.031	0.368120034	0.652316155
RP1-319M7	-0.031	0.36809011	0.652316155
AC092017	-0.031	0.36808853	0.652316155
RAB10	-0.031	0.368052277	0.652316155
C21orf62	-0.031	0.368014094	0.652279679
RN7SL610P	-0.031	0.368001205	0.652279679
ARAF	-0.031	0.36791068	0.652222233
ZNF606	-0.031	0.367896726	0.652222233
RPL5P9	-0.031	0.367726628	0.652037086
RNU6-847P	-0.031	0.367705009	0.652023026
AL118506	-0.031	0.367621506	0.651899228
SORCS3-AS1	-0.031	0.367609176	0.651899228
HOXB1	-0.031	0.367591816	0.651895123
AC016644	-0.031	0.367577459	0.651893935
SYNGR1	-0.031	0.36757151	0.651893935
MIR4500	-0.031	0.367502069	0.651808775
AL161645	-0.031	0.367462976	0.651763711
RP3-363L9	-0.031	0.367450105	0.651763711
RNU6-260P	-0.031	0.367446458	0.651763711

RPL29P3	-0.031	0.367408339	0.651763711
SCGB2B2	-0.031	0.367400981	0.651763711
RN7SL530P	-0.031	0.367397073	0.651763711
RP11-732A19	-0.031	0.367342736	0.651744616
RNA5SP84	-0.031	0.367288685	0.651672994
SLK	-0.031	0.367254313	0.651660563
TPM3P3	-0.031	0.367248905	0.651660563
EFCAB12	-0.031	0.367220628	0.651649348
RNU7-193P	-0.031	0.367218478	0.651649348
SRP9	-0.031	0.367195946	0.651649348
ZDHHC20P1	-0.031	0.367195858	0.651649348
SNORA25	-0.031	0.367166315	0.651649348
AC090043	-0.031	0.367143153	0.651649348
FP236240	-0.031	0.367047735	0.651561032
AC007402	-0.031	0.367031545	0.651556576
INTS7	-0.031	0.367030461	0.651556576
AC005537	-0.031	0.367009959	0.651556576
RP11-172H24	-0.031	0.36694962	0.651508277
AL731561	-0.031	0.366898577	0.651445738
AP000343	-0.031	0.366885264	0.651445738
ABHD14B	-0.031	0.36687721	0.651445738
LINC00571	-0.031	0.366872503	0.651445738
HMGB3P1	-0.031	0.366841765	0.651445738
RP11-518K17	-0.031	0.366811389	0.651432845
FAM71F2	-0.031	0.366786672	0.651413238
HMGN2P10	-0.031	0.366758176	0.651411212
CSGALNACT2	-0.031	0.366747501	0.651411212
ALDOAP2	-0.031	0.36671658	0.651385916
ALDH9A1	-0.031	0.366686283	0.651385916
TAT-AS1	-0.031	0.366670275	0.651385916
RNU6-996P	-0.031	0.36666896	0.651385916
STK39	-0.031	0.366641317	0.651385916
RP11-356O22	-0.031	0.3666264	0.651385916
MRPL50P1	-0.031	0.366588977	0.651385916
BNIP3P31	-0.031	0.366577757	0.651385916
RPL21P10	-0.031	0.366572069	0.651385916
RN7SKP193	-0.031	0.366529186	0.651368864
SRGAP2-AS1	-0.031	0.366442429	0.651263287
KCTD13	-0.031	0.366438866	0.651263287
CDC27P1	-0.031	0.366429888	0.651263287
RNU4ATAC3P	-0.031	0.366237709	0.651093818
RNU6-776P	-0.031	0.366237709	0.651093818
RNU7-183P	-0.031	0.366237709	0.651093818
AATF	-0.031	0.366234456	0.651093818
SETD7	-0.031	0.366045187	0.650945938
AL136090	-0.031	0.366040197	0.650945938
SEPT7P7	-0.031	0.365946798	0.650843875
AC009022	-0.031	0.365938489	0.650843875
HNRNPA1P72	-0.031	0.365930156	0.650843875
AL772400	-0.031	0.365929905	0.650843875
IFITM5	-0.031	0.365845043	0.650784413
CTB-186G2	-0.031	0.365819583	0.650763428

UBE2V2P4	-0.031	0.365724796	0.650619112
RP11-477G18	-0.031	0.365712094	0.650619112
RN7SL584P	-0.031	0.365632658	0.650528095
CTC-215O4	-0.031	0.365568926	0.650463303
OR51M1	-0.031	0.36541672	0.650241066
RP11-134G8	-0.031	0.365396104	0.650228675
CNGA4	-0.031	0.365371363	0.650228675
RPS2P41	-0.031	0.365234471	0.650013907
RNU6-1264P	-0.031	0.365168432	0.64994495
AC002365	-0.031	0.365155953	0.64994495
SKP1P1	-0.031	0.365144022	0.64994495
RNA5SP182	-0.031	0.365130465	0.64994495
KLLN	-0.031	0.365116245	0.64994495
KB-1674E1	-0.031	0.36501028	0.649809165
AC007006	-0.031	0.365000122	0.649809165
RNU6-1258P	-0.031	0.365000122	0.649809165
AC096763	-0.031	0.365000122	0.649809165
RNA5SP406	-0.031	0.365000122	0.649809165
LINC00898	-0.031	0.36496797	0.649809165
EBAG9P1	-0.031	0.364966624	0.649809165
MIR3621	-0.031	0.364927251	0.649809165
RPS7P6	-0.031	0.364753551	0.649667815
RP5-1023B21	-0.031	0.364739601	0.649667264
GEMIN6	-0.031	0.364686731	0.649597386
PPIL1	-0.031	0.3646769	0.649597386
NPIPB7	-0.031	0.364599182	0.649514316
UBAP2L	-0.031	0.364529466	0.649438704
LIPN	-0.031	0.364493909	0.649399649
CTD-2310F14	-0.031	0.364440156	0.649328169
PRDM1	-0.031	0.364416747	0.649314818
OR2L3	-0.031	0.364381336	0.64929624
RNA5SP394	-0.031	0.364339288	0.649269897
AC017104	-0.031	0.364333233	0.649269897
AC013286	-0.031	0.364314453	0.649269897
SLC35D1	-0.031	0.364289025	0.649269897
ABCC10	-0.031	0.364260999	0.649251847
SLC25A42	-0.031	0.364242797	0.649243702
MRPS10	-0.031	0.36423673	0.649243702
AKIRIN1	-0.031	0.364226908	0.649243702
RP11-5N11	-0.031	0.364170687	0.649236658
ZNF528-AS1	-0.031	0.364112225	0.649181033
AC105760	-0.031	0.364009694	0.649069016
RP3-368B9	-0.031	0.363981146	0.64906882
RP11-423F24	-0.031	0.363979712	0.64906882
SETP21	-0.031	0.363950915	0.649063515
RNA5SP263	-0.031	0.363918255	0.649053876
AC253578	-0.031	0.363909439	0.649053876
PPP1R9A	-0.031	0.36382172	0.649027532
LA16c-321D4	-0.031	0.363817936	0.649027532
OR5B21	-0.031	0.363797067	0.649027532
APOL4	-0.031	0.363794294	0.649027532
RNU6-236P	-0.031	0.363792739	0.649027532

CTD-2380F24	-0.031	0.363670287	0.64890322
HSPE1P8	-0.031	0.36366649	0.64890322
GALNT4	-0.031	0.36366339	0.64890322
RP11-307L3	-0.031	0.363448692	0.648702251
ELOVL2-AS1	-0.031	0.363326021	0.648531909
OR9L1P	-0.031	0.363296822	0.648504094
RP1-225L15	-0.031	0.363291406	0.648504094
SPI1	-0.031	0.363259025	0.648485235
FBXO27	-0.031	0.363238548	0.648485235
EEF1A1P1	-0.031	0.363181925	0.648469128
RP1-111J24	-0.031	0.363174257	0.648469128
RP11-492M19	-0.031	0.363116731	0.648411337
RPL5P12	-0.031	0.363106373	0.648411337
CH17-351M24	-0.031	0.362999973	0.648363012
RNU4-36P	-0.031	0.362974969	0.648342666
DEFB124	-0.031	0.362938019	0.648342666
RP11-138H11	-0.031	0.362929663	0.648342666
RP11-219E7	-0.031	0.362924796	0.648342666
AC123900	-0.031	0.362864781	0.648316048
HLA-F-AS1	-0.031	0.36280465	0.648232933
AP002022	-0.031	0.362777059	0.648232272
MPP1	-0.031	0.362589407	0.647995124
EEF1A1P10	-0.031	0.362534673	0.647945019
IL32	-0.031	0.362531216	0.647945019
RPL7AP57	-0.031	0.362429482	0.647805635
AC074391	-0.031	0.362417968	0.647805635
SIGLEC11	-0.031	0.362408723	0.647805635
RP11-466P24	-0.031	0.362361048	0.647804874
AL353602	-0.031	0.362291458	0.647729092
RP11-402J6	-0.031	0.362253592	0.647685706
TSPAN5	-0.031	0.362137283	0.64752637
bP-21201H5	-0.031	0.362101029	0.647485854
RP11-76E17	-0.031	0.362097353	0.647485854
RP11-173A16	-0.031	0.362064229	0.647485854
RP11-1078H9	-0.031	0.362042297	0.647485854
ATP5HP3	-0.031	0.361915604	0.647348724
RP11-398J16	-0.031	0.361911868	0.647348724
LINC00336	-0.031	0.361904836	0.647348724
LINC00919	-0.031	0.361867163	0.647348724
TUBA8P2	-0.031	0.361837201	0.647330041
RPS6KL1	-0.031	0.361817554	0.647322296
RP5-850E9	-0.031	0.36179288	0.647322296
PPIL1P1	-0.031	0.361732445	0.647239881
MCCC1-AS1	-0.031	0.361676838	0.647164698
RP11-179A16	-0.031	0.361645695	0.647133285
MIR548I1	-0.031	0.361629364	0.647128376
LINC01293	-0.031	0.361609029	0.647116301
PDE6C	-0.031	0.361537394	0.647061045
MIR1200	-0.031	0.361522124	0.64705803
SLC30A7	-0.031	0.36144912	0.64700031
ARPP21-AS1	-0.031	0.36138869	0.646940769
CTB-36O1	-0.031	0.36129387	0.646819649

EMC7	-0.031	0.361251051	0.646767303
RP11-345K9	-0.031	0.361178806	0.646710891
ARHGAP26-AS1	-0.031	0.361175113	0.646710891
OR7E43P	-0.031	0.361163021	0.646710891
NPIPB6	-0.031	0.361110033	0.646684998
LPPR4	-0.031	0.361094426	0.646681366
CACNG5	-0.031	0.361090926	0.646681366
RP11-294J22	-0.031	0.361076677	0.646681366
RP11-466F5	-0.031	0.361018859	0.646666093
THBS4	-0.031	0.360949909	0.646568427
CTA-732E4	-0.031	0.360948386	0.646568427
CAB39L	-0.031	0.360941004	0.646568427
OR7E14P	-0.031	0.360915725	0.646568427
LINC00589	-0.031	0.360911715	0.646568427
MECP2	-0.031	0.360852819	0.646540411
RNU6-1285P	-0.031	0.360816746	0.6465001
MIR555	-0.031	0.360762799	0.646427759
IFNWP9	-0.031	0.360735102	0.646402449
SRRM2-AS1	-0.031	0.360682807	0.646357378
GS1-433O24	-0.031	0.360577603	0.646193164
KTN1	-0.031	0.360406644	0.645935396
RP4-803J11	-0.031	0.36034611	0.645890359
FAM186A	-0.031	0.360257419	0.645765153
OR11K2P	-0.031	0.360215591	0.64574392
IFNA20P	-0.031	0.360168133	0.645702314
PCNP	-0.031	0.36012631	0.645651639
ZYG11B	-0.031	0.359972572	0.645424602
SLC22A9	-0.031	0.35993068	0.645373786
AC004840	-0.031	0.359893173	0.645330829
RPL3P2	-0.031	0.359869912	0.645330829
RP11-680G24	-0.031	0.359742043	0.645181287
AC118465	-0.031	0.359736245	0.645181287
ZNF16	-0.031	0.359732296	0.645181287
ALG1L6P	-0.031	0.359679529	0.645142053
RNU6-374P	-0.031	0.359658676	0.645128945
RP11-265N7	-0.031	0.359634446	0.64510978
RPS3AP4	-0.031	0.359594747	0.645062862
RP1-305G21	-0.031	0.3595521	0.64503495
CACUL1	-0.031	0.359495558	0.644980696
AL390119	-0.031	0.359313147	0.644776279
RP1-62D2	-0.031	0.359283498	0.644747369
DHRSX-IT1	-0.031	0.359280971	0.644747369
RP11-474B16	-0.031	0.359271953	0.644747369
MIR5194	-0.031	0.359079076	0.644550528
MIR367	-0.031	0.359004366	0.644440711
HLTF-AS1	-0.031	0.359000129	0.644440711
AC226150	-0.031	0.35897148	0.644432757
AC002366	-0.031	0.358948285	0.644432757
CTA-215D11	-0.031	0.358931874	0.644432757
RPS4XP4	-0.031	0.358905754	0.644432757
MIR148B	-0.031	0.358893436	0.644432757
MIR4653	-0.031	0.358888157	0.644432757

RNU6-483P	-0.031	0.358849507	0.644418037
AMER2	-0.031	0.358828503	0.644416482
DHX33	-0.031	0.358819418	0.644416482
RNA5SP355	-0.031	0.358815712	0.644416482
RP11-314A15	-0.031	0.358785142	0.644416482
CNOT7	-0.031	0.358669464	0.644283312
ZNF484	-0.031	0.358649721	0.644283312
S100PBP	-0.031	0.358647718	0.644283312
VN1R64P	-0.031	0.358625465	0.644283312
AC067957	-0.031	0.358604462	0.644283312
AC016737	-0.031	0.358603127	0.644283312
XDH	-0.031	0.358585919	0.644283312
EDNRB-AS1	-0.031	0.358573624	0.644283312
MIR5705	-0.031	0.358499269	0.644262497
PLCG1	-0.031	0.358450327	0.644223159
MIR4311	-0.031	0.358443245	0.644223159
STK17B	-0.031	0.358401705	0.644208703
RP11-124N19	-0.031	0.358385899	0.644204605
AL589986	-0.031	0.358336127	0.644139449
RNU6-4P	-0.031	0.358299802	0.644098463
RP11-733O18	-0.031	0.358230702	0.644057642
C9orf129	-0.031	0.358054397	0.643876035
NANOGP10	-0.031	0.358037947	0.643870764
RP11-373N24	-0.031	0.358025298	0.643870764
MIR661	-0.031	0.357974711	0.643805663
TARS2	-0.031	0.357874564	0.643698472
AC107218	-0.031	0.357789176	0.643624992
VIT	-0.031	0.357750137	0.643600836
RP5-997D24	-0.031	0.357718281	0.643600836
AC083843	-0.031	0.357694007	0.643592462
SSXP9	-0.031	0.357692681	0.643592462
MAS1L	-0.031	0.357690193	0.643592462
CHFR	-0.031	0.357637452	0.643587959
MTOR-AS1	-0.031	0.357620262	0.643581342
LINC01522	-0.031	0.357591303	0.643576459
HSP90AB2P	-0.031	0.357455829	0.643406982
LYZL1	-0.031	0.357446683	0.643406982
RP11-288G3	-0.031	0.357361389	0.643358567
HCFC1	-0.031	0.357355047	0.643358567
AC016722	-0.031	0.357303824	0.643327885
SNRNP35	-0.031	0.357295386	0.643327885
SNORA79	-0.031	0.357282577	0.643327885
MTND4P15	-0.031	0.357264399	0.643327885
RP11-440K22	-0.031	0.357166821	0.643300087
AC005730	-0.031	0.357142298	0.643280244
SPACA1	-0.031	0.357120163	0.643264703
OR7E160P	-0.031	0.357104348	0.643260544
MIR6715A	-0.031	0.357068855	0.643226049
TRIM63	-0.031	0.356892283	0.643047948
RP11-543H12	-0.031	0.356868634	0.643047948
KRTAP4-3	-0.031	0.356830365	0.643047948
TPM3P7	-0.031	0.356793688	0.643017102

RP11-395P13	-0.031	0.356667825	0.642845737
AC007375	-0.031	0.35665729	0.642845737
RP11-51G5	-0.031	0.356631354	0.642845737
RP11-351K23	-0.031	0.356631123	0.642845737
PA2G4P2	-0.031	0.356501665	0.642709698
KIAA2026	-0.031	0.356449955	0.642665127
OR4C9P	-0.031	0.356434619	0.642661806
RP11-472N13	-0.031	0.356311549	0.642488553
RNU6-1206P	-0.031	0.356301796	0.642488553
MDH1	-0.031	0.356182954	0.642353953
HPR	-0.031	0.356153876	0.642325836
B4GALNT2	-0.031	0.356142497	0.642325836
LGALS4	-0.031	0.35612776	0.642325836
SUMO2P10	-0.031	0.356126851	0.642325836
SMC5-AS1	-0.031	0.356116864	0.642325836
RP11-1246C19	-0.031	0.356102337	0.642325836
CGN	-0.031	0.356094361	0.642325836
PSMC1P1	-0.031	0.356078695	0.642325836
MTND1P32	-0.031	0.356068434	0.642325836
CHCHD3P1	-0.031	0.356051589	0.642325836
AC131160	-0.031	0.356012826	0.642325836
LIN28AP1	-0.031	0.35600704	0.642325836
IFNW1	-0.031	0.355815161	0.642128327
OR6Q1	-0.031	0.355809	0.642128327
PRAMEF11	-0.031	0.355786265	0.642124842
SESTD1	-0.031	0.355726181	0.642040731
NIP7	-0.031	0.355684338	0.641989539
PKD1P6	-0.031	0.355683484	0.641989539
FOXD4	-0.031	0.355659268	0.641989539
TMEM245	-0.031	0.355659006	0.641989539
PODXL	-0.031	0.355600946	0.641936332
OR11N1P	-0.031	0.355598579	0.641936332
OR2L6P	-0.031	0.355578841	0.641936332
RP11-707F2	-0.031	0.355570224	0.641936332
SDCBPP2	-0.031	0.355510606	0.641870566
MICD	-0.031	0.355499965	0.641870566
CCL18	-0.031	0.355489603	0.641870566
MIR6760	-0.031	0.3554589	0.641870566
RP11-172C16	-0.031	0.355440971	0.641866502
RP11-335E6	-0.031	0.355435124	0.641866502
RPL5P1	-0.031	0.355351652	0.641753872
ZEB2-AS1	-0.031	0.355341285	0.641753872
ZMIZ2	-0.031	0.355222849	0.641594243
SYT1	-0.031	0.355207207	0.641590323
YAP1P1	-0.031	0.355195019	0.641590323
SLC39A12-AS1	-0.031	0.355185074	0.641590323
RN7SKP16	-0.031	0.3551721	0.641590323
ST7-OT4	-0.031	0.355114358	0.641548154
RP11-453A12	-0.031	0.355098958	0.641548154
CTD-2201G16	-0.032	0.355065916	0.641548154
RNU1-112P	-0.032	0.355060578	0.641548154
RP11-619I22	-0.032	0.354985721	0.641506542

NPHP4	-0.032	0.354946477	0.641487387
RP11-342C20	-0.032	0.354895873	0.641448006
IMMP1LP3	-0.032	0.354873085	0.641448006
AC009970	-0.032	0.354804514	0.641373766
FAM13A-AS1_2	-0.032	0.354757001	0.64133656
ZNF41	-0.032	0.354522232	0.641009467
IZUMO3	-0.032	0.354460253	0.640921734
ZNF398	-0.032	0.354370855	0.640784415
ERCC6L	-0.032	0.354360064	0.640784415
CSK	-0.032	0.354298163	0.640701621
ABCA7	-0.032	0.354285102	0.640701621
BTF3	-0.032	0.354269486	0.640701621
GAB1	-0.032	0.354223492	0.640663892
TPCN2	-0.032	0.354183748	0.640616337
RP11-348M17	-0.032	0.354107436	0.640502634
AZU1P1	-0.032	0.353987581	0.640407449
AC104162	-0.032	0.353976701	0.640407449
EIF4EBP1P1	-0.032	0.353903749	0.640377413
LINC01438	-0.032	0.353871148	0.640342749
RP11-1141N12	-0.032	0.353859755	0.640342749
C17orf99	-0.032	0.353823404	0.64030501
COL2A1	-0.032	0.353782196	0.640254765
AC069303	-0.032	0.353699029	0.640225891
BECN1P2	-0.032	0.353699029	0.640225891
AC069070	-0.032	0.353617151	0.640175006
PITPNM1	-0.032	0.353522703	0.640028348
RP4-681N20	-0.032	0.353501168	0.640013691
MIR320C2	-0.032	0.353484471	0.64000779
TMED7	-0.032	0.353479889	0.64000779
IKBKE	-0.032	0.353464296	0.64000779
RP11-659G9	-0.032	0.353413963	0.639953115
GORASP1	-0.032	0.353364508	0.639921769
MUSK	-0.032	0.353133293	0.63961511
RP4-714D9	-0.032	0.353112849	0.639602406
OR8K5	-0.032	0.353095685	0.63959564
MTRF1L	-0.032	0.353062984	0.639585057
FTX_1	-0.032	0.35298494	0.639500788
RP11-758B24	-0.032	0.352905719	0.639421771
RP11-780M14	-0.032	0.352879485	0.639421771
SLC25A16	-0.032	0.352822222	0.639392107
FGFR1OP2P1	-0.032	0.352739127	0.639290171
ZNF275	-0.032	0.352738687	0.639290171
RP11-407N8	-0.032	0.352653945	0.639184436
SNORD114-19	-0.032	0.352602331	0.639149577
SETD8	-0.032	0.352599397	0.639149577
RN7SL323P	-0.032	0.352527094	0.639149577
RPL7P33	-0.032	0.35251386	0.639149577
RP3-461P17	-0.032	0.352483346	0.639149577
RP11-444J21	-0.032	0.352372708	0.639063819
RP11-204K16	-0.032	0.352323278	0.639022841
WASIR1	-0.032	0.352290503	0.638987729
AL356137	-0.032	0.352270398	0.638975597

BAG1	-0.032	0.35226377	0.638975597
RN7SL472P	-0.032	0.352240872	0.63897071
RN7SL405P	-0.032	0.352234217	0.63897071
ERVK3-1	-0.032	0.352214547	0.63897071
N4BP3	-0.032	0.352189654	0.63897071
RBM11P1	-0.032	0.352110028	0.638910635
RP11-632B21	-0.032	0.352106605	0.638910635
H1FNT	-0.032	0.352095712	0.638910635
PAICSP5	-0.032	0.352089882	0.638910635
RP11-326C3	-0.032	0.352063509	0.638910635
WBP1LP8	-0.032	0.352049724	0.638910635
KRTAP10-3	-0.032	0.352043109	0.638910635
RP11-669B18	-0.032	0.352024847	0.638910635
SNORD116-25	-0.032	0.351989189	0.638910635
SAMSN1-AS1	-0.032	0.351876911	0.638762206
ATP6V1G2	-0.032	0.351769188	0.638625872
PEBP1P1	-0.032	0.351744189	0.63860483
AGTR1	-0.032	0.351716615	0.638588059
ZNF157	-0.032	0.351700836	0.638588059
FUT5	-0.032	0.351585918	0.638463511
RNU6-592P	-0.032	0.351540723	0.638405781
AC112179	-0.032	0.351540723	0.638405781
FREM1	-0.032	0.351496767	0.638374639
TUBAL3	-0.032	0.351452824	0.638319173
RP4-580O19	-0.032	0.351446584	0.638319173
ARMC8	-0.032	0.351430389	0.638319173
RP11-803B1	-0.032	0.3513683	0.638244733
CCDC39-AS1	-0.032	0.351316276	0.638241543
RNU6-346P	-0.032	0.351278812	0.638229935
RP11-96O20	-0.032	0.351274905	0.638229935
AK8	-0.032	0.351258384	0.638229935
CICP5	-0.032	0.351254014	0.638229935
RP1-159A19	-0.032	0.351219133	0.638229935
PHKA1	-0.032	0.351147697	0.638203066
RP11-616M17	-0.032	0.351076809	0.638098581
RP11-613H2	-0.032	0.3510574	0.638098581
RPL36P2	-0.032	0.35104566	0.638098581
IGBP1P3	-0.032	0.350894702	0.637889307
LRRC66	-0.032	0.35080026	0.637766309
SGOL1P1	-0.032	0.350791385	0.637766309
CACNA1F	-0.032	0.350777665	0.637766309
AC007041	-0.032	0.350736725	0.637748176
AC114969	-0.032	0.350722281	0.637746261
RP11-349H17	-0.032	0.350712553	0.637746261
HMGN2P32	-0.032	0.350690524	0.637746261
PRRX2-AS1	-0.032	0.350680226	0.637746261
RP11-727M10	-0.032	0.350579581	0.637657198
RP11-934B9	-0.032	0.350547686	0.637623535
AC084121	-0.032	0.350487211	0.637553629
YWHAEP1	-0.032	0.350475083	0.637553629
CTAGE12P	-0.032	0.350465002	0.637553629
PSMA4	-0.032	0.350463448	0.637553629

RP11-612A1	-0.032	0.350400485	0.637526221
ANKRD24	-0.032	0.350377448	0.637526221
AA06	-0.032	0.350350339	0.637526221
SLC9B1P4	-0.032	0.350271248	0.637437192
RNU6-1294P	-0.032	0.350128847	0.637220804
CTC-503J8	-0.032	0.350119645	0.637220804
LINC00487	-0.032	0.35006443	0.637158199
RPL12P43	-0.032	0.350053696	0.637158199
RN7SL825P	-0.032	0.350014505	0.637116027
AP006294	-0.032	0.350003716	0.637116027
RP11-505O17	-0.032	0.349938733	0.637051144
RP1-101K10	-0.032	0.349926602	0.637051144
SNORD114-13	-0.032	0.349920675	0.637051144
RP11-170J3	-0.032	0.349880246	0.637017718
RP4-621B10	-0.032	0.349864369	0.637017071
MAP7	-0.032	0.34985215	0.637017071
MRPS16P2	-0.032	0.349846765	0.637017071
C10orf95	-0.032	0.3497792	0.636979848
GNL2P1	-0.032	0.349732551	0.63691925
LINC00850	-0.032	0.349726496	0.63691925
RN7SL851P	-0.032	0.349724495	0.63691925
AC215219	-0.032	0.349587853	0.636777477
RP11-175E9	-0.032	0.349544866	0.63673102
RNY3P11	-0.032	0.34951678	0.63673102
RN7SL393P	-0.032	0.349482111	0.63673102
ZNF281	-0.032	0.349412625	0.636653089
PRR23D1	-0.032	0.34940896	0.636653089
MIR4728	-0.032	0.349264115	0.636479893
RP11-26H16	-0.032	0.349252606	0.636479893
AC008697	-0.032	0.349229954	0.636479893
PRSS3P2	-0.032	0.349210915	0.636479893
TMEM262	-0.032	0.349206639	0.636479893
GACAT1	-0.032	0.349193665	0.636479893
POM121L12	-0.032	0.34916168	0.636463692
CTD-2234N14	-0.032	0.349140353	0.636449176
RANBP6	-0.032	0.349051356	0.636330269
AC004535	-0.032	0.349044474	0.636330269
GNG8	-0.032	0.349009003	0.636330269
SOWAHA	-0.032	0.349004947	0.636330269
CTD-2286N8	-0.032	0.34893183	0.63623952
MYL10	-0.032	0.348927502	0.63623952
SMIM15	-0.032	0.348867156	0.636202262
RNU6-890P	-0.032	0.348856788	0.636202262
RP11-421B23	-0.032	0.348851891	0.636202262
TTC30A	-0.032	0.34871759	0.636068043
GAPDHP71	-0.032	0.348678533	0.636021159
RP11-304F15	-0.032	0.348671211	0.636021159
RAX2	-0.032	0.348631168	0.635983478
CTB-73N10	-0.032	0.348534627	0.635953516
TRBVA	-0.032	0.348462862	0.635862053
CD300LG	-0.032	0.348458628	0.635862053
OOSP1P1	-0.032	0.348445117	0.635862053

SLC22A4	-0.032	0.348440778	0.635862053
RP11-168O22	-0.032	0.348371944	0.635862053
CPNE6	-0.032	0.348308652	0.635809136
RN7SL28P	-0.032	0.348290795	0.635802766
RPL23AP76	-0.032	0.348289346	0.635802766
CTC-471F3	-0.032	0.348273352	0.635802766
ZNF337-AS1	-0.032	0.348159611	0.635686534
ZNF221	-0.032	0.348138831	0.635686534
RNU6-461P	-0.032	0.348110076	0.635665922
MIR202	-0.032	0.34806735	0.635612272
MIR6766	-0.032	0.348018535	0.635547499
CDK11B	-0.032	0.348004343	0.635545951
RP11-492M23	-0.032	0.347998451	0.635545951
AC108479	-0.032	0.347997289	0.635545951
LINC00403	-0.032	0.347996894	0.635545951
RP1-22N22	-0.032	0.347802268	0.635371816
RARA	-0.032	0.347771103	0.635348993
CYP2T3P	-0.032	0.347698421	0.635303947
RNU6-332P	-0.032	0.347536337	0.63505652
ANKRD20A8P	-0.032	0.347483124	0.63500801
HSP90AA1	-0.032	0.34747261	0.63500801
OR5AC4P	-0.032	0.347455109	0.635005544
RP11-107K17	-0.032	0.347428338	0.634988983
AC114752	-0.032	0.347363001	0.634912787
KRT18P2	-0.032	0.34732511	0.634912787
RP13-75G22	-0.032	0.347312982	0.634912787
EIF3H	-0.032	0.347234269	0.634855445
OR2U2P	-0.032	0.347070661	0.634692611
MS4A3	-0.032	0.34700291	0.63459308
GAPDHP20	-0.032	0.346957155	0.634559543
MLLT4	-0.032	0.346956243	0.634559543
RP11-398H6	-0.032	0.346944023	0.634559543
RNU6-135P	-0.032	0.346903776	0.634557981
PLCXD2-AS1	-0.032	0.34688006	0.63453897
TP53I11	-0.032	0.346824006	0.634460801
C8orf22	-0.032	0.346770406	0.634411482
NDUFA4P2	-0.032	0.346738273	0.634400214
AP000470	-0.032	0.34669042	0.634338249
MIR548AK	-0.032	0.346664952	0.63431602
RP11-751K21	-0.032	0.346650451	0.634313856
TRAV11	-0.032	0.346571441	0.634218014
GRK5-IT1	-0.032	0.346489338	0.634092131
SDAD1P3	-0.032	0.346443756	0.63408025
SLC5A9	-0.032	0.346441482	0.63408025
ACTG1P10	-0.032	0.346354696	0.634016262
AC007078	-0.032	0.346295757	0.633981474
RNF5P1	-0.032	0.346269912	0.633958527
Six3os1_7	-0.032	0.346252178	0.633950427
RP11-89B16	-0.032	0.346159381	0.633853627
IMP3P2	-0.032	0.346131714	0.633827334
STXBP2	-0.032	0.346061748	0.63372358
ATP6V0E1P3	-0.032	0.346034197	0.633697493

SRP68P3	-0.032	0.345881321	0.633581709
PPCS	-0.032	0.345865967	0.633581709
MOGAT1	-0.032	0.34585408	0.633581709
RN7SL602P	-0.032	0.345802092	0.633564772
AC008536	-0.032	0.345772774	0.6335598
RP11-76E16	-0.032	0.345767932	0.6335598
ATP1A2	-0.032	0.34568705	0.633475842
CFAP53	-0.032	0.345653205	0.633438192
IP6K2	-0.032	0.345639508	0.633438192
RP11-450I1	-0.032	0.345615174	0.633438192
ZNF208	-0.032	0.345546497	0.633383848
AC124276	-0.032	0.345456702	0.63327302
RP11-564C4	-0.032	0.345428931	0.633246486
HNRNPA3P13	-0.032	0.34542101	0.633246486
MIR4425	-0.032	0.345362092	0.633172698
MIR302B	-0.032	0.345336954	0.633172698
LRRC37A11P	-0.032	0.345220296	0.633028643
HNRNPA1P42	-0.032	0.345217504	0.633028643
AL591610	-0.032	0.345156818	0.632991288
RP4-725K1	-0.032	0.345135571	0.632976697
RP11-330M19	-0.032	0.345052206	0.632872546
TAL2	-0.032	0.345032689	0.632861121
RP11-697M17	-0.032	0.345012845	0.632853381
IQCJ-SCHIP1-AS1	-0.032	0.344959958	0.632800828
KRTAP13-6P	-0.032	0.344941231	0.632790848
RP11-168J19	-0.032	0.344925752	0.632786826
ASS1P12	-0.032	0.344828063	0.632680721
ABCB10P4	-0.032	0.344819371	0.632680721
MIR193B	-0.032	0.344788933	0.632679213
RP11-686F15	-0.032	0.344765532	0.632679213
FABP12	-0.032	0.344748022	0.632679213
PTMA	-0.032	0.344691086	0.632599991
HNRNPA1	-0.032	0.344614079	0.632483034
MIR2276	-0.032	0.344583447	0.632483034
ZNF542P	-0.032	0.344548519	0.6324602
TDRD3	-0.032	0.344478227	0.632379914
RP11-267M23	-0.032	0.344461491	0.632373567
RP11-284H18	-0.032	0.344428679	0.632337703
PPT2	-0.032	0.344370852	0.632255911
COPS5P1	-0.032	0.344347669	0.632237719
MEF2B	-0.032	0.34434714	0.632237719
SETP4	-0.032	0.344325214	0.632237719
OR2T2	-0.032	0.344237857	0.63215795
RPL15P20	-0.032	0.344234739	0.63215795
TNPO1P3	-0.032	0.34421382	0.63215795
PIGS	-0.032	0.344143902	0.632126731
CTD-2058B24	-0.032	0.344128541	0.632126731
RP5-1114G22	-0.032	0.344079775	0.632086413
Z83819	-0.032	0.344039747	0.632086413
DDX39A	-0.032	0.344015971	0.632067304
RNU6-840P	-0.032	0.343951264	0.632021562
CFP	-0.032	0.343942404	0.632021562

H2AFZP5	-0.032	0.343930775	0.632021562
C1orf226	-0.032	0.343903395	0.632021562
RNA5SP103	-0.032	0.343834004	0.631976771
CLEC3A	-0.032	0.34377457	0.631923288
SUCLA2P2	-0.032	0.343771375	0.631923288
DIEXF	-0.032	0.343761593	0.631923288
PRSS41	-0.032	0.343730398	0.631923288
RP11-810F22	-0.032	0.343689044	0.631881031
SLC45A4	-0.032	0.343666894	0.631866111
RNA5SP277	-0.032	0.343608357	0.631865513
CA12	-0.032	0.343591703	0.631865513
LGALS9	-0.032	0.343546594	0.63185964
KIZ	-0.032	0.343514295	0.631828043
RP1-139G21	-0.032	0.343510523	0.631828043
CDC42P6	-0.032	0.343500852	0.631828043
OR52Q1P	-0.032	0.343478634	0.631828043
RP11-669N7	-0.032	0.343467771	0.631828043
MIR574	-0.032	0.343457748	0.631828043
PMPCB	-0.032	0.343418526	0.631822696
RP11-474L11	-0.032	0.34339266	0.631799514
MIR548AQ	-0.032	0.343383915	0.631799514
AC010891	-0.032	0.343312322	0.631749323
SCARNA11	-0.032	0.343290884	0.631749323
AC012485	-0.032	0.343255568	0.631718108
RP13-379O24	-0.032	0.343233169	0.631701296
FTH1P1	-0.032	0.343174189	0.631641564
GLULP4	-0.032	0.343112603	0.631624088
PRPF4	-0.032	0.343014477	0.631469618
RPSAP63	-0.032	0.342864058	0.631321684
C9orf116	-0.032	0.342853443	0.631321684
OR6M2P	-0.032	0.342788571	0.631282715
DEFB131	-0.032	0.34278223	0.631282715
RNU6-590P	-0.032	0.342755571	0.631282715
RP11-462G22	-0.032	0.342736898	0.631275824
HRG	-0.032	0.342647097	0.631169056
RP11-63L7	-0.032	0.342640388	0.631169056
AC067718	-0.032	0.342625022	0.631169056
IFIT1P1	-0.032	0.342618182	0.631169056
CTD-2012I17	-0.032	0.342601477	0.631169056
WNK4	-0.032	0.342599335	0.631169056
RP11-506N2	-0.032	0.342419806	0.630984605
STK33P1	-0.032	0.342316389	0.630867262
OR51R1P	-0.032	0.342315817	0.630867262
FDX1	-0.032	0.342308399	0.630867262
RP11-342H21	-0.032	0.342285452	0.630867262
KCTD3	-0.032	0.342205623	0.630809583
AL353141	-0.032	0.342164084	0.630757426
RNU6-304P	-0.032	0.342154861	0.630757426
IRGQ	-0.032	0.342113974	0.630731488
RP11-30M20	-0.032	0.342096832	0.630731102
RP11-996F15	-0.032	0.342010402	0.630620574
CTD-2251F13	-0.032	0.342004018	0.630620574

ARHGDIA	-0.032	0.341910181	0.63050901
RP11-757G1	-0.032	0.341905462	0.63050901
POLE2	-0.032	0.341838567	0.630450179
RN7SL7P	-0.032	0.341778394	0.630412438
SAPCD1	-0.032	0.34177012	0.630412438
PTK6	-0.032	0.341759469	0.630412438
WFDC13	-0.032	0.341695851	0.630382254
RP11-324J3	-0.032	0.341655714	0.630332623
RP11-317B7	-0.032	0.341593223	0.630241746
GOLGA3	-0.032	0.341575507	0.630233475
AC018816	-0.032	0.341561359	0.630231785
SPINK7	-0.032	0.341543953	0.630224085
RP11-750I4	-0.032	0.341483497	0.630136943
AC073342	-0.032	0.341481235	0.630136943
RNU6-1257P	-0.032	0.341434057	0.630099627
NFAM1	-0.032	0.341429604	0.630099627
SPATA24	-0.032	0.341402425	0.630099627
RNU6-334P	-0.032	0.341303537	0.630000139
WBP2NL	-0.032	0.341301209	0.630000139
RPRML	-0.032	0.34129135	0.630000139
CTB-184G21	-0.032	0.341250424	0.629997988
MIR4495	-0.032	0.341243799	0.629997988
SNORD127	-0.032	0.34122227	0.629997988
RN7SL685P	-0.032	0.341199855	0.629997988
RNF170	-0.032	0.341019291	0.629817193
C10orf2	-0.032	0.340963591	0.629807169
ZNF146	-0.032	0.34094641	0.629804704
PLA2G7	-0.032	0.340942531	0.629804704
GDF5OS	-0.032	0.340912145	0.629790261
DNM1P31	-0.032	0.340867877	0.629732908
CTC-137K3	-0.032	0.340763894	0.6296385
MAPRE2	-0.032	0.340760236	0.6296385
AC092299	-0.032	0.340754838	0.6296385
RP11-61D3	-0.032	0.340746699	0.6296385
GAPDHP37	-0.032	0.340743194	0.6296385
RSL1D1	-0.032	0.340703177	0.6296385
RPL7P21	-0.032	0.340690715	0.6296385
RP11-317O24	-0.032	0.340655834	0.629609818
RN7SL515P	-0.032	0.34065326	0.629609818
PPP2R5E	-0.032	0.340639479	0.629609818
RP11-168L7	-0.032	0.34061287	0.629603709
RP11-222G7	-0.032	0.340609876	0.629603709
RP11-775D22	-0.032	0.340502691	0.62947334
LRRC6	-0.032	0.340493001	0.62947334
AC010609	-0.032	0.340468941	0.629463538
PAX2	-0.032	0.340423356	0.629448838
EIF4A1P2	-0.032	0.340410458	0.629448838
CTC-529G1	-0.032	0.34038588	0.629448838
RP11-475F14	-0.032	0.340341279	0.629419256
RP11-2G1	-0.032	0.340260478	0.629318699
RP11-654G14	-0.032	0.340244635	0.629313835
RP11-361F15	-0.032	0.340235062	0.629313835

AL356135	-0.032	0.34020464	0.629313835
HCRT	-0.032	0.340191498	0.629313835
OR2T11	-0.032	0.340189091	0.629313835
RP11-648O15	-0.032	0.340177553	0.629313835
RP11-402G3	-0.032	0.340125338	0.629313835
MIR627	-0.032	0.340107754	0.629313835
RP11-270L13	-0.032	0.340099074	0.629313835
RNU1-120P	-0.032	0.340059773	0.629289615
SCAMP1	-0.032	0.340034444	0.629267192
GTF2A2	-0.032	0.340004293	0.629235845
AC068506	-0.033	0.339982555	0.629227538
KRTAP5-4	-0.033	0.33995257	0.629213471
RP11-366L18	-0.033	0.339921249	0.629179951
MFSD1	-0.033	0.339882325	0.629150907
RNA5SP288	-0.033	0.339842454	0.629131907
RN7SL138P	-0.033	0.339838908	0.629131907
MYO16-AS2	-0.033	0.339795991	0.629094798
PATE3	-0.033	0.339747691	0.629054282
ANXA7	-0.033	0.339593419	0.628841981
SCHLAP1	-0.033	0.339541402	0.628819006
KRT27	-0.033	0.339495839	0.628783525
RP11-196D18	-0.033	0.339466262	0.628753195
RP11-719K4	-0.033	0.339416474	0.628734332
PRDM8	-0.033	0.339390447	0.628734332
CTC-233O10	-0.033	0.339373919	0.628728864
TRAJ49	-0.033	0.3393641	0.628728864
RNA5SP170	-0.033	0.339334064	0.628728864
RNA5SP200	-0.033	0.339302613	0.628728864
MBTPS1	-0.033	0.339125697	0.628562405
LRRC2-AS1	-0.033	0.33909758	0.628534751
MTMR12	-0.033	0.339053904	0.628489784
DAOA-AS1	-0.033	0.339052412	0.628489784
RP11-310N16	-0.033	0.33903596	0.628489784
NF1P4	-0.033	0.339025634	0.628489784
RP11-177G23	-0.033	0.338931432	0.628422444
OSTF1	-0.033	0.33892332	0.628422444
FEZF2	-0.033	0.338795601	0.628243968
AKAP7	-0.033	0.338759102	0.628219725
VN1R11P	-0.033	0.338709943	0.628158501
KLHDC9	-0.033	0.338658664	0.628112316
RP11-451H23	-0.033	0.338558212	0.627999375
GOLGA8F	-0.033	0.338419996	0.627791896
RP11-411G7	-0.033	0.338348536	0.627695229
SPRYD3	-0.033	0.338292116	0.627652459
CTA-256D12	-0.033	0.338257995	0.627613602
ENTPD8	-0.033	0.338233675	0.627592926
AC015922	-0.033	0.338217421	0.627587217
AC021134	-0.033	0.338114624	0.62749426
RNU6-537P	-0.033	0.338099219	0.627490122
RP11-762L8	-0.033	0.338081079	0.627490122
CTD-3105H18	-0.033	0.338078759	0.627490122
RNA5SP299	-0.033	0.338052702	0.627490122

AC004079	-0.033	0.338016151	0.627482663
AL157392	-0.033	0.338005771	0.627482663
FOXR1	-0.033	0.337880756	0.627329128
RP5-963E22	-0.033	0.337751999	0.627226711
ZBTB34	-0.033	0.337720326	0.627202416
EVI5	-0.033	0.337717296	0.627202416
CTD-3193O13	-0.033	0.337703034	0.627202416
LINC00412	-0.033	0.337653938	0.627152488
AC007128	-0.033	0.337650797	0.627152488
GPR141	-0.033	0.337586723	0.627101012
SETP17	-0.033	0.337558936	0.627073854
OR52L2P	-0.033	0.337453688	0.626927243
OR56A1	-0.033	0.337374531	0.626804634
FRA10AC1	-0.033	0.337334615	0.626754923
IDI1	-0.033	0.337257837	0.626661165
VMAC	-0.033	0.337169789	0.626611709
C2orf27A	-0.033	0.337104039	0.62652205
NPM1P14	-0.033	0.337066676	0.626492264
TMED10P2	-0.033	0.337040218	0.626492264
B4GALT6	-0.033	0.337033071	0.626492264
RP11-629P16	-0.033	0.337002168	0.626479402
PPFIBP2	-0.033	0.336976963	0.626479402
LINC00895	-0.033	0.336971222	0.626479402
RP11-856F16	-0.033	0.336918857	0.626422331
MCEE	-0.033	0.336904637	0.626422331
LINC00930	-0.033	0.336880771	0.626422331
LINC01046	-0.033	0.336871735	0.626422331
MIR7160	-0.033	0.336823154	0.626398016
FSD2	-0.033	0.33681106	0.626398016
RP11-10K17	-0.033	0.336754823	0.626361867
RP11-410B16	-0.033	0.336750716	0.626361867
AC024619	-0.033	0.33672639	0.626357904
RP1-178F15	-0.033	0.336722214	0.626357904
RAC1P5	-0.033	0.336718753	0.626357904
RP11-556N4	-0.033	0.336691225	0.626357904
MIR936	-0.033	0.33666161	0.626357904
RP11-364C11	-0.033	0.336577303	0.626227331
DPH5	-0.033	0.33645303	0.626069486
UBQLN2	-0.033	0.336413468	0.626020327
RP11-452K12	-0.033	0.336333254	0.625968707
C22orf29	-0.033	0.336304138	0.625939161
KRTAP4-12	-0.033	0.336257216	0.625878933
MRPS15P1	-0.033	0.336234241	0.625878933
SNORD101	-0.033	0.336210213	0.625862178
ITGAE	-0.033	0.33620966	0.625862178
ARHGAP5	-0.033	0.336066876	0.625717636
TRIM45	-0.033	0.336018833	0.625652643
OR2G6	-0.033	0.335828899	0.625421246
NPM1P6	-0.033	0.335786366	0.62536649
ILKAP	-0.033	0.335761671	0.62536649
RN7SL442P	-0.033	0.335708515	0.62531931
PIPSL	-0.033	0.335697194	0.62531931

RNU6-1282P	-0.033	0.335669823	0.62529615
B4GAT1	-0.033	0.335669759	0.62529615
RP11-502I4	-0.033	0.335660981	0.62529615
RP11-757O6	-0.033	0.335660651	0.62529615
ATP6V1G2-DDX39B	-0.033	0.335654125	0.62529615
AP000362	-0.033	0.335620065	0.62529615
RNU7-19P	-0.033	0.335612574	0.62529615
KRTAP13-5P	-0.033	0.335609419	0.62529615
SNX14	-0.033	0.335603648	0.62529615
CTB-41I6	-0.033	0.33551676	0.625255575
ANO2	-0.033	0.335390284	0.625117719
NPBWR2	-0.033	0.335286797	0.624998211
TLR6	-0.033	0.335235938	0.624927865
GLB1L3	-0.033	0.335222357	0.624927008
ATXN3	-0.033	0.33519126	0.624893496
ZNF512	-0.033	0.335190712	0.624893496
ANKDD1A	-0.033	0.335147956	0.624893496
RN7SL68P	-0.033	0.335146992	0.624893496
C9orf173-AS1	-0.033	0.335122473	0.624893496
PDCL2P1	-0.033	0.335112312	0.624893496
CTSLP2	-0.033	0.33485289	0.624653894
RNA5SP175	-0.033	0.334793768	0.62457685
PDCD2	-0.033	0.334787962	0.62457685
ZNF596	-0.033	0.334724427	0.624512096
ANXA6	-0.033	0.334683626	0.624477779
PIGFP2	-0.033	0.334677788	0.624477779
RNA5SP116	-0.033	0.334665124	0.624477779
LRRC46	-0.033	0.334602034	0.624477779
CDC37P1	-0.033	0.334601343	0.624477779
CHCHD4P2	-0.033	0.334551622	0.624477779
OR6C68	-0.033	0.33451172	0.62443324
CCDC175	-0.033	0.334486644	0.624410903
MIR496	-0.033	0.334485923	0.624410903
PAQR8	-0.033	0.334482408	0.624410903
HMGB1P7	-0.033	0.334480892	0.624410903
RP11-5A1	-0.033	0.334448054	0.624410903
RP11-697B24	-0.033	0.334440691	0.624410903
RNU6-1067P	-0.033	0.33425693	0.624237767
ASH2L	-0.033	0.33415433	0.624100945
VTI1BP4	-0.033	0.334057016	0.623975742
RNF219-AS1	-0.033	0.334022329	0.623935422
AC107068	-0.033	0.333992398	0.623903982
RP11-211N8	-0.033	0.333957321	0.623862927
RN7SL60P	-0.033	0.333927014	0.62385525
CTD-2206N4	-0.033	0.333906288	0.623841001
RP11-271F18	-0.033	0.333845844	0.62375254
AC093609	-0.033	0.33382326	0.623734813
RPP38	-0.033	0.333786549	0.62373246
KRTAP10-8	-0.033	0.333757563	0.623709937
AMBRA1	-0.033	0.333745822	0.623709937
IQCD	-0.033	0.333715484	0.623704718
AC104301	-0.033	0.333693205	0.623687555

LINGO3	-0.033	0.333674219	0.623676544
LINC00200	-0.033	0.333612333	0.623609819
FAM3B	-0.033	0.333563819	0.623543607
CXXC4	-0.033	0.33345372	0.6234112
RP11-897M7	-0.033	0.333453402	0.6234112
AC005300	-0.033	0.333450377	0.6234112
AIMP1P1	-0.033	0.333393754	0.6233725
LL22NC03-123E1	-0.033	0.333371408	0.623355191
RP1-60G11	-0.033	0.333360645	0.623355191
RP11-13J8	-0.033	0.333336483	0.62333883
FARSA-AS1	-0.033	0.333316983	0.62332684
RP4-771M4	-0.033	0.333295779	0.623311662
AP000770	-0.033	0.333184751	0.623177432
SLC15A5	-0.033	0.333183637	0.623177432
RP11-402D21	-0.033	0.333159689	0.623177432
GHc-857G6	-0.033	0.333034528	0.622969854
AC005777	-0.033	0.333026736	0.622969854
MYCT1	-0.033	0.332991175	0.622937692
CTD-3162L10	-0.033	0.332984898	0.622937692
OR1L4	-0.033	0.332783154	0.622597449
OR5AN1	-0.033	0.33277203	0.622597449
FRMPD3	-0.033	0.332762375	0.622597449
RP11-181K12	-0.033	0.332745676	0.622597449
RNU6-1154P	-0.033	0.332676634	0.622520432
RP11-451L19	-0.033	0.332652562	0.622513651
RP11-684B21	-0.033	0.33262328	0.622493966
CTD-2151L9	-0.033	0.332540156	0.622387315
FSHB	-0.033	0.332537114	0.622387315
OR7E149P	-0.033	0.332514531	0.622387315
AC084859	-0.033	0.332474213	0.622387315
RP5-1024G6	-0.033	0.332465786	0.622387315
MIR1197	-0.033	0.332441599	0.622387315
C6orf106	-0.033	0.332402536	0.622374319
BROX	-0.033	0.332399809	0.622374319
FAM120B	-0.033	0.33236328	0.622349753
CHTF8	-0.033	0.332332206	0.622316032
RAB8B	-0.033	0.332275785	0.622259312
SIAH2-AS1	-0.033	0.332272565	0.622259312
PDXDC1	-0.033	0.332225366	0.622213823
BNIP3P36	-0.033	0.332133735	0.622091136
HCN4	-0.033	0.332122762	0.622091136
CTD-3194G12	-0.033	0.332090679	0.622059417
SNRPA1	-0.033	0.332060789	0.622027894
RNU6-519P	-0.033	0.331987195	0.621963422
AL158210	-0.033	0.331952471	0.621922832
HCG4P3	-0.033	0.331926224	0.621898122
PTTG3P	-0.033	0.331757916	0.621656145
MIR1468	-0.033	0.331747599	0.621656145
RNA5SP453	-0.033	0.331710817	0.621616805
RP11-252C15	-0.033	0.331694326	0.621616027
PPP2R2DP1	-0.033	0.331677716	0.621616027
RP11-118M9	-0.033	0.331480444	0.621331776

AC007618	-0.033	0.331386911	0.621180903
HDAC1P2	-0.033	0.331381165	0.621180903
RP11-53B2	-0.033	0.331370903	0.621180903
OR52P2P	-0.033	0.331296877	0.621109917
BLMH	-0.033	0.33126127	0.621067611
RNU6-920P	-0.033	0.33119643	0.620990833
STXBP5	-0.033	0.331166075	0.620962467
MIR4540	-0.033	0.33109315	0.620899061
AL954742	-0.033	0.331001233	0.620800021
RN7SL417P	-0.033	0.330938249	0.620730781
AP001437	-0.033	0.330829671	0.620576003
RTP2	-0.033	0.330825203	0.620576003
FAM120C	-0.033	0.330784113	0.620539426
ZNF736P6Y	-0.033	0.330700414	0.620424102
FAM220CP	-0.033	0.3306482	0.620406643
TMEM120B	-0.033	0.330623801	0.620385303
RP11-367H5	-0.033	0.330583467	0.620368035
IGIP	-0.033	0.330573272	0.620368035
RNU6-902P	-0.033	0.330509404	0.62029284
RP11-93H24	-0.033	0.330485264	0.62029284
RP11-889L3	-0.033	0.330472501	0.62029284
AC114801	-0.033	0.3304449	0.620269549
ADAT2	-0.033	0.33025473	0.61998589
BPIFB5P	-0.033	0.330233638	0.619970731
THEM5	-0.033	0.330200438	0.619932838
RNA5SP513	-0.033	0.330195463	0.619932838
RPS21P1	-0.033	0.330175736	0.619932838
LIME1	-0.033	0.330153479	0.619917988
MRPL35P1	-0.033	0.330074955	0.619827228
DUX4L18	-0.033	0.330070275	0.619827228
CISD3	-0.033	0.329984953	0.619772591
RPS6KA3	-0.033	0.329975457	0.619772591
LINC00500	-0.033	0.329945112	0.619771085
SRD5A3P1	-0.033	0.329942049	0.619771085
GXYLT1P3	-0.033	0.329927173	0.619771085
MIR548AN	-0.033	0.329773736	0.619571349
RP11-400G3	-0.033	0.329766327	0.619571349
TFCP2L1	-0.033	0.329644543	0.619401927
RP11-22N12	-0.033	0.329640317	0.619401927
SMN1	-0.033	0.329578386	0.619350923
PCNPP4	-0.033	0.3295651	0.619350392
Y10196	-0.033	0.32953468	0.619317662
AL355149	-0.033	0.329384031	0.619132262
CTD-2329C7	-0.033	0.329322538	0.619089975
RP11-75N6	-0.033	0.329282546	0.619039229
GAMTP2	-0.033	0.329275545	0.619039229
TBX10	-0.033	0.329266463	0.619039229
SDCBP2P1	-0.033	0.329236011	0.619039229
MIR1324	-0.033	0.329227346	0.619039229
CDC42BPB	-0.033	0.329174581	0.619018503
RP11-126M14	-0.033	0.32916552	0.619018503
RNU6-1100P	-0.033	0.329125942	0.61898915

CH17-262O2	-0.033	0.329118824	0.61898915
OR4C49P	-0.033	0.329017555	0.618907504
RP11-48O20	-0.033	0.328939707	0.618785505
TRAV8-7	-0.033	0.328784567	0.618518093
RNU6-347P	-0.033	0.328748126	0.618473969
RP11-216F5	-0.033	0.328737672	0.618473969
PAX6	-0.033	0.328697204	0.618451456
OR2AK2	-0.033	0.328657085	0.618400402
RN7SKP168	-0.033	0.328551306	0.618274647
RP11-10F11	-0.033	0.328449209	0.618106941
OR7M1P	-0.033	0.328388239	0.618041043
MST1	-0.033	0.328335205	0.617965652
OR5G5P	-0.033	0.328323014	0.617965652
PYGO2	-0.033	0.328320871	0.617965652
RP11-328N19	-0.033	0.328303749	0.617965652
RP11-1112J20	-0.033	0.32826938	0.61793944
RFESD	-0.033	0.328249478	0.617926399
AL096803	-0.033	0.328246964	0.617926399
FAHD1	-0.033	0.328234335	0.617926399
DHX57	-0.033	0.32820324	0.617912627
HSPA8P18	-0.033	0.328180573	0.617894376
TRBV23OR9-2	-0.033	0.328135198	0.617857796
BMX	-0.033	0.328116679	0.617847352
SLC25A38P1	-0.033	0.328086678	0.617815286
RP11-88L20	-0.033	0.328061004	0.617791367
CENPI	-0.033	0.328055361	0.617791367
DDX11L2	-0.033	0.328011935	0.617747811
RP11-159F24	-0.033	0.32794698	0.617649905
AP000897	-0.033	0.327889299	0.617565691
TSPY22P	-0.033	0.327776942	0.617478407
FAM90A22P	-0.033	0.327766253	0.617478407
RP4-782L23	-0.033	0.327748305	0.617478407
CHRM2	-0.033	0.327738182	0.617478407
OLA1P3	-0.033	0.327675945	0.617465381
TAS2R20	-0.033	0.327660055	0.617465381
DGKQ	-0.033	0.327654386	0.617465381
HCST	-0.033	0.327564993	0.617418787
DOK1	-0.033	0.327524488	0.617391308
MIR6747	-0.033	0.327486012	0.617343213
CEND1P1	-0.033	0.32747618	0.617343213
RNY3P7	-0.033	0.327475366	0.617343213
RNU6-23P	-0.033	0.327372734	0.617278856
AC073143	-0.033	0.327359361	0.617278856
TSEN15P3	-0.033	0.327313028	0.617236996
TNP2	-0.033	0.327297726	0.617232579
AC087174	-0.033	0.327227349	0.617124295
RP11-57H14	-0.033	0.32720947	0.617116163
PTDSS1	-0.033	0.327136614	0.617050912
AC084082	-0.033	0.32706317	0.616957181
AC004899	-0.033	0.327053666	0.616957181
TRBV22OR9-2	-0.033	0.327041967	0.616957181
LINC00355	-0.033	0.327025061	0.616957181

TDRG1	-0.033	0.327002751	0.616957181
RPS15AP25	-0.033	0.326942257	0.616904247
UQCRFS1P3	-0.033	0.3268786	0.616857402
AC009262	-0.033	0.326868176	0.616857402
RP11-320G10	-0.033	0.326830708	0.616815904
AC073635	-0.033	0.326813384	0.616807649
RP1-92C4	-0.033	0.326813179	0.616807649
RP11-320P7	-0.033	0.326767559	0.616794489
LINC01121	-0.033	0.32676018	0.616794489
RP13-653N12	-0.033	0.326682008	0.61670633
RP11-598D14	-0.033	0.32666518	0.616699007
RP11-705O1	-0.033	0.326492661	0.61644662
AC013269	-0.033	0.326452877	0.616444817
RP11-907D1	-0.033	0.32642419	0.616439528
FOXD2-AS1	-0.033	0.326409447	0.616436128
XXyac-YX155B6	-0.033	0.326353811	0.616355499
HNRNPLP2	-0.033	0.32634425	0.616355499
RP11-35N6	-0.033	0.326320807	0.616342048
RP11-728F11	-0.033	0.326296048	0.616319726
DRD1	-0.033	0.326242193	0.616266884
C15orf65	-0.033	0.326210919	0.616256691
AK3P3	-0.033	0.326208448	0.616256691
RANP1	-0.033	0.326156124	0.616202059
RP11-186B7	-0.033	0.326145478	0.616202059
TSPAN16	-0.033	0.326083378	0.616132504
MB	-0.033	0.326021084	0.616118013
IFT74	-0.033	0.325978011	0.616085505
NUTM2HP	-0.033	0.325961068	0.616085505
LRRC55	-0.033	0.325886762	0.616010828
RP11-30B1	-0.033	0.32586197	0.615988413
SERPINA9	-0.033	0.325856193	0.615988413
AC019080	-0.033	0.325681551	0.615745117
OR5M13P	-0.033	0.325647638	0.61570544
CTC-558O2	-0.033	0.325637624	0.61570544
AC000036	-0.033	0.325610432	0.615683978
RP11-132J14	-0.033	0.325597133	0.615683276
AC005024	-0.033	0.325504173	0.615556373
CHMP4C	-0.033	0.325473523	0.615535103
RP11-598O8	-0.033	0.325402228	0.615436897
RP11-1029F8	-0.033	0.325379509	0.615418365
AC092069	-0.034	0.325312932	0.615316879
ZFYVE9P1	-0.034	0.325190118	0.615187915
RP11-215H18	-0.034	0.325119597	0.615122196
SNORD95	-0.034	0.32507501	0.615062272
AC078794	-0.034	0.324990538	0.614975738
RP11-101E3	-0.034	0.324964802	0.614975738
LCE2D	-0.034	0.324939917	0.614975738
RNU6-316P	-0.034	0.324930449	0.614975738
RP1-298M8	-0.034	0.324756229	0.614757357
TNFSF10	-0.034	0.32466176	0.614671078
MFAP3	-0.034	0.324613434	0.614604017
RP1-296G17	-0.034	0.324606803	0.614604017

AC108044	-0.034	0.324599577	0.614604017
ACOX2	-0.034	0.32458122	0.614604017
TRIB1	-0.034	0.324419105	0.614455926
RP4-769N13	-0.034	0.324407566	0.614455926
RN7SKP157	-0.034	0.324407374	0.614455926
TFAMP1	-0.034	0.32434151	0.614382257
TISP43	-0.034	0.32433606	0.614382257
UBBP4	-0.034	0.32431231	0.614375816
AC074011	-0.034	0.324306049	0.614375816
RP11-417J1	-0.034	0.324181647	0.614177147
RN7SL838P	-0.034	0.32404713	0.613971144
ZNF713	-0.034	0.324021288	0.613946606
MIR588	-0.034	0.324013067	0.613946606
MIR1251	-0.034	0.323969868	0.613898023
NR2E3	-0.034	0.323953832	0.61389206
ACTG1P3	-0.034	0.323949644	0.61389206
RP11-72I18	-0.034	0.323931487	0.61389206
C14orf142	-0.034	0.323881873	0.613877823
RPL7P26	-0.034	0.323831269	0.613814355
U91324	-0.034	0.323828364	0.613814355
MOB3C	-0.034	0.323799046	0.613814355
AC005539	-0.034	0.323730272	0.613785894
BRINP2	-0.034	0.323717614	0.613785894
LINC00444	-0.034	0.323616801	0.613644041
LOH12CR1	-0.034	0.323609321	0.613644041
KSR1	-0.034	0.323596728	0.613644041
RP11-470E16	-0.034	0.32354115	0.61359831
CTD-3138F19	-0.034	0.32343157	0.61343339
TREX1	-0.034	0.323385655	0.613401116
C16orf52	-0.034	0.323369855	0.613395575
CRYGS	-0.034	0.323360215	0.613395575
RNA5SP319	-0.034	0.323321304	0.613359274
RP4-659I19	-0.034	0.323292976	0.613348555
CICP10	-0.034	0.323263909	0.613341175
LINC00863	-0.034	0.323204386	0.613288813
SLCO2A1	-0.034	0.323184041	0.613287367
MIR4726	-0.034	0.323177949	0.613287367
RNU6-1284P	-0.034	0.323007094	0.613024861
TBC1D3E	-0.034	0.322830737	0.612885538
AC112518	-0.034	0.32279875	0.612873673
SEBOX	-0.034	0.322780303	0.612863079
CDC42EP4	-0.034	0.322764475	0.612863079
ARHGEF12	-0.034	0.322753777	0.612863079
RPL12P37	-0.034	0.322691277	0.612816198
SPATA8-AS1	-0.034	0.322595982	0.612714337
RP11-329A14	-0.034	0.322562926	0.612700315
CSMD3	-0.034	0.322523668	0.612683225
BRD9P2	-0.034	0.322408004	0.612620228
RP11-357H14	-0.034	0.322377304	0.612591976
RNA5SP82	-0.034	0.322236826	0.612490454
COX6CP8	-0.034	0.322204911	0.612454233
VN1R51P	-0.034	0.322188665	0.612447796

CTD-2123J17		-0.034	0.322164078	0.612425503
HIC2		-0.034	0.322104538	0.61233676
CIR1P1		-0.034	0.322096222	0.61233676
CTD-2339L15		-0.034	0.322081399	0.61233676
MIR4476		-0.034	0.322073058	0.61233676
CENPA		-0.034	0.321964592	0.612217336
GNPTAB		-0.034	0.321941627	0.612198111
MIR518F		-0.034	0.32187206	0.612090261
RP11-382J24		-0.034	0.321864281	0.612090261
BAG6		-0.034	0.321782774	0.61196934
AADACL4		-0.034	0.32173597	0.611929199
RNU6-689P		-0.034	0.321710564	0.611905314
AC011467		-0.034	0.321703912	0.611905314
MIR493		-0.034	0.321692382	0.611905314
PAGE2B		-0.034	0.321612431	0.61182651
CHD5		-0.034	0.321598118	0.61182651
LINC01104		-0.034	0.321514075	0.611702586
OTOL1		-0.034	0.321456794	0.611642472
RNU6-1138P		-0.034	0.321437797	0.611630761
RNU7-130P		-0.034	0.321366592	0.611568573
RP11-79M19		-0.034	0.321362914	0.611568573
PTPN20A		-0.034	0.321314283	0.611548801
DLGAP2		-0.034	0.321230667	0.611456495
TIMM8BP1		-0.034	0.32122902	0.611456495
TCN2		-0.034	0.321228657	0.611456495
RP13-487C10		-0.034	0.321162211	0.611423936
UBA3		-0.034	0.321131077	0.611399804
CR769776		-0.034	0.321073952	0.611399804
C18orf21		-0.034	0.321063636	0.611399804
RP1-15D7		-0.034	0.321032902	0.611397714
NTRK2		-0.034	0.320994338	0.611348717
MIR520D		-0.034	0.320972548	0.611331662
A1BG-AS1		-0.034	0.320971419	0.611331662
CTD-2017F17		-0.034	0.320966622	0.611331662
ENHO		-0.034	0.320884282	0.611285776
ZDHHC22		-0.034	0.32085541	0.611255225
TMEM243		-0.034	0.320852042	0.611255225
ALDH8A1		-0.034	0.320849599	0.611255225
FAM106A		-0.034	0.320755521	0.611138264
RP11-840I19		-0.034	0.320749587	0.611138264
	10-Mar	-0.034	0.320721312	0.611121982
YBX1P7		-0.034	0.320668448	0.611094597
RP11-40A13		-0.034	0.320656403	0.611094597
RNU6-116P		-0.034	0.320626979	0.611094597
LMLN-AS1		-0.034	0.320580182	0.611048638
RP11-650P15		-0.034	0.320520027	0.610958431
KRT85		-0.034	0.320295093	0.610675502
RP11-584P21		-0.034	0.320179762	0.610480864
NPM1P4		-0.034	0.320174651	0.610480864
RP11-510I6		-0.034	0.320107842	0.61039261
MIR1250		-0.034	0.320029203	0.610291525
TSPO2		-0.034	0.319976323	0.610239553

CROCCP5	-0.034	0.319930614	0.610195297
RPS6KA1	-0.034	0.319925728	0.610195297
RNU6-1013P	-0.034	0.319896984	0.610195297
RNU6-335P	-0.034	0.319854733	0.610154259
RBM22P12	-0.034	0.319843089	0.610154259
RP11-648M2	-0.034	0.319832586	0.610154259
RN7SL728P	-0.034	0.319826484	0.610154259
MIR548AJ1	-0.034	0.319819547	0.610154259
ZNF214	-0.034	0.319742801	0.610125831
OR8B8	-0.034	0.319649076	0.610006272
LOXL3	-0.034	0.319640466	0.610006272
RNU11-3P	-0.034	0.319607245	0.610006272
FKBP1AP3	-0.034	0.319552151	0.609967949
RP1-127L4	-0.034	0.319499283	0.609891477
RNU6-982P	-0.034	0.319427799	0.609779462
RNU7-63P	-0.034	0.319360017	0.609723383
SNRPGP18	-0.034	0.319309217	0.609650835
BRD4	-0.034	0.319232935	0.609529626
AC066692	-0.034	0.319230096	0.609529626
C6orf10	-0.034	0.319230053	0.609529626
SNRPCP10	-0.034	0.319230002	0.609529626
APTR	-0.034	0.319217799	0.609529626
RP11-805I24	-0.034	0.319210815	0.609529626
ISCA1P4	-0.034	0.319157371	0.609529626
RNU6-498P	-0.034	0.318985959	0.60930233
MIR151B	-0.034	0.318982143	0.60930233
RP4-809F18	-0.034	0.318976623	0.60930233
ASAH2B	-0.034	0.318883855	0.609205028
HSPD1P15	-0.034	0.318792517	0.609128268
RANBP3	-0.034	0.31878568	0.609128268
CR1L	-0.034	0.318732954	0.609087766
GCA	-0.034	0.318726253	0.609087766
RNU5E-10P	-0.034	0.318721396	0.609087766
SLC34A3	-0.034	0.318719146	0.609087766
RPL17P45	-0.034	0.31864542	0.609042677
AC009541	-0.034	0.318614729	0.609008456
CHTF8P1	-0.034	0.31859683	0.608998685
RNU6-434P	-0.034	0.318519374	0.608899503
MIR554	-0.034	0.318517515	0.608899503
TFB1M	-0.034	0.318506121	0.608899503
TUBB4BP6	-0.034	0.318490657	0.608899503
FAM58DP	-0.034	0.318471609	0.608899503
AP000295	-0.034	0.318467993	0.608899503
USP9X	-0.034	0.318467409	0.608899503
RNU6-878P	-0.034	0.318455625	0.608899503
LINC00320	-0.034	0.318353068	0.608867756
ZNF500	-0.034	0.318264885	0.608779537
RNY1P14	-0.034	0.318247617	0.608777028
RP11-122K13	-0.034	0.318229638	0.608777028
TPT1P6	-0.034	0.318209001	0.60877044
ATP5A1P7	-0.034	0.318207787	0.60877044
LL22NC03-48A11	-0.034	0.318160543	0.60877044

RP11-209K10	-0.034	0.318139209	0.608759185
NUDCD2	-0.034	0.318081785	0.60875319
OR5T1	-0.034	0.31797998	0.608650129
CTA-351J1	-0.034	0.317950108	0.608617411
SRRM1P2	-0.034	0.317928332	0.608600187
AC026703	-0.034	0.317706984	0.608298715
OR8B2	-0.034	0.317623583	0.608212383
CCDC177	-0.034	0.317600338	0.608192324
MED15P7	-0.034	0.317481972	0.608014552
USP6	-0.034	0.317455103	0.607987542
RP11-548K12	-0.034	0.317358726	0.607851847
IL20RB-AS1	-0.034	0.317227373	0.607649135
MIR4323	-0.034	0.317130776	0.607586285
RP11-369E15	-0.034	0.317044876	0.607495024
CBLB	-0.034	0.316928596	0.60729665
AC016142	-0.034	0.316863651	0.607245498
HOXB4	-0.034	0.31676238	0.607100277
GADL1	-0.034	0.31661083	0.60683424
RLIM	-0.034	0.316558718	0.606758775
HKDC1	-0.034	0.316387605	0.606557736
LDLRAD3	-0.034	0.316353821	0.606557736
AC104996	-0.034	0.316350517	0.606557736
RNU6-497P	-0.034	0.316346512	0.606557736
RP11-185J20	-0.034	0.316182667	0.606379635
RP11-730B22	-0.034	0.316131167	0.606305283
MTND5P1	-0.034	0.316125189	0.606305283
RP11-959I15	-0.034	0.316122845	0.606305283
MYLKP1	-0.034	0.31612053	0.606305283
RNY1P2	-0.034	0.316094042	0.606305283
RP11-23D5	-0.034	0.315759038	0.605821881
TTI2	-0.034	0.31569589	0.605738786
RP5-1033E15	-0.034	0.315679914	0.605735015
ATP1B3P1	-0.034	0.315656407	0.605735015
CBX3P5	-0.034	0.315656031	0.605735015
PPIAP1	-0.034	0.315588064	0.605702709
RP11-367O10	-0.034	0.315492289	0.605567696
ZNF615	-0.034	0.315487143	0.605567696
RP11-501E14	-0.034	0.315439897	0.60551594
CROCCP4	-0.034	0.315389189	0.605443003
CTC-450M9	-0.034	0.315313507	0.605346518
RNU6-969P	-0.034	0.315284656	0.605315528
AP001189	-0.034	0.315239201	0.605252658
RP4-545K15	-0.034	0.315215069	0.605230722
RP11-396C23	-0.034	0.315199212	0.605224675
EEA1	-0.034	0.315193154	0.605224675
NUP133	-0.034	0.315164187	0.605206222
ZNF304	-0.034	0.315062024	0.605096882
LA16c-385E7	-0.034	0.314974598	0.605013467
RP11-338H14	-0.034	0.314916411	0.604974342
GBA3	-0.034	0.314847718	0.604866777
LINC00967	-0.034	0.31476928	0.604789273
MAGEB2	-0.034	0.31472345	0.604725612

RNU6-444P	-0.034	0.314659235	0.60462662
PA2G4P1	-0.034	0.314638963	0.60461206
ADAM6	-0.034	0.314513539	0.604492992
ARPC3P5	-0.034	0.314459769	0.604414037
RP11-535C7	-0.034	0.314380927	0.604311274
NEB	-0.034	0.314337239	0.604251682
RP11-530A18	-0.034	0.314323261	0.604249201
OR9S24P	-0.034	0.314302615	0.604233901
RN7SL624P	-0.034	0.314294476	0.604233901
RP11-2G17	-0.034	0.314240878	0.604188378
RP1-156L9	-0.034	0.314237368	0.604188378
CHD4	-0.034	0.314192413	0.604168366
PTPN12	-0.034	0.31403292	0.603959209
RP11-527L4	-0.034	0.313943303	0.603811237
RP11-136B18	-0.034	0.313866618	0.603712508
AC090810	-0.034	0.313831258	0.603693255
RP11-567P17	-0.034	0.313805098	0.603691699
RP11-94I2	-0.034	0.313796482	0.603691699
DHRS7	-0.034	0.313752686	0.603671642
PIP5K1P2	-0.034	0.313702075	0.603671642
RP11-335O13	-0.034	0.313677396	0.603665481
ZAR1L	-0.034	0.313574796	0.603516803
RP11-115H15	-0.034	0.313565395	0.603516803
RNU5D-1	-0.034	0.313542135	0.603516803
SLC12A1	-0.034	0.313508757	0.603487252
CALCRL	-0.034	0.313402035	0.603379364
FAM9B	-0.034	0.313399516	0.603379364
RP11-95I16	-0.034	0.31336355	0.603354051
RUVBL1-AS1	-0.034	0.313326253	0.603306629
LINC00159	-0.034	0.313278483	0.603239036
GCDH	-0.034	0.313224708	0.603159874
TTC33	-0.034	0.313203989	0.603144363
CHM	-0.034	0.313103817	0.602975839
AL161752	-0.034	0.313099918	0.602975839
CTB-189B5	-0.034	0.312968657	0.602764294
RP11-568G11	-0.034	0.312875386	0.602682144
RP1-164F3	-0.034	0.312874856	0.602682144
EEF1A1P3	-0.034	0.312852745	0.602682144
CAMK2G	-0.034	0.312823604	0.602655521
AC011288	-0.034	0.312777613	0.602612251
RNU6-17P	-0.034	0.31274695	0.602580969
TDP1	-0.034	0.312713557	0.602565382
Vax2os1_3	-0.034	0.312647985	0.602463407
BEND7P1	-0.034	0.312630782	0.602454632
MSH5-SAPCD1	-0.034	0.312534129	0.602292747
ZC3H18	-0.034	0.312429908	0.602150183
HSBP1L1	-0.034	0.312387231	0.602107109
RP11-144O23	-0.034	0.31233968	0.602039821
AL117334	-0.034	0.312322688	0.602031433
FAM90A10P	-0.034	0.312241772	0.601924183
LINC00029	-0.034	0.312226911	0.601919899
AMZ1	-0.034	0.31218856	0.601892558

GAPDHP15	-0.034	0.312162703	0.601892558
DEFB108P1	-0.034	0.312062003	0.601845597
NT5DC4	-0.034	0.31203223	0.601812546
RNU6-197P	-0.034	0.312019169	0.601811727
RP11-429P7	-0.034	0.312017302	0.601811727
AP005135	-0.034	0.311991824	0.601807728
RNF19B	-0.034	0.311970354	0.601807728
RNU7-156P	-0.034	0.311937864	0.601776759
RNU6-893P	-0.034	0.311908171	0.601768227
RP11-274M17	-0.034	0.311876462	0.601731426
RP11-844P9	-0.034	0.311855082	0.601726682
MGAT5B	-0.034	0.311754601	0.601569418
AC002981	-0.034	0.311754109	0.601569418
KLHDC10	-0.034	0.311722442	0.601556109
MIR4318	-0.034	0.311639065	0.601419578
SUMO1P3	-0.034	0.311633822	0.601419578
PRR4	-0.034	0.311612388	0.601416834
ZNF733P	-0.034	0.311569969	0.601359333
RP11-205K6	-0.034	0.311512927	0.601273604
RP11-70C1	-0.034	0.311499365	0.601271794
RP11-396B14	-0.034	0.311478851	0.601256565
SUMO2P6	-0.034	0.311373046	0.601125418
RNU6-1208P	-0.034	0.311345924	0.601097422
SCRT1	-0.034	0.311340472	0.601097422
GTPBP2	-0.034	0.311322867	0.601097422
MIR3168	-0.034	0.311289407	0.601061404
RNU6-226P	-0.034	0.311261217	0.60103134
LIPT2	-0.034	0.31125626	0.60103134
RP11-415K20	-0.034	0.311211629	0.600984319
DCUN1D1	-0.034	0.311159893	0.600933142
RP11-91I20	-0.034	0.311140744	0.600933142
RNU6-761P	-0.034	0.311129054	0.600933142
RP11-317B3	-0.035	0.311088622	0.600892963
CTB-131B5	-0.035	0.311065373	0.600892963
OSBPL7	-0.035	0.311040832	0.600892963
CTC-236F12	-0.035	0.310982638	0.600834453
TRIM31	-0.035	0.310847347	0.600621794
RP1-181J22	-0.035	0.310783496	0.600563616
DAPK1-IT1	-0.035	0.310751028	0.600557508
RNU6-21P	-0.035	0.310728167	0.600537694
KPNA6	-0.035	0.310725049	0.600537694
OR7E11P	-0.035	0.310692939	0.600518348
KIF19	-0.035	0.31068985	0.600518348
LIPM	-0.035	0.310686447	0.600518348
ADIRF-AS1	-0.035	0.310670265	0.600518348
RNA5SP322	-0.035	0.310611883	0.600459152
PRR3	-0.035	0.310600621	0.600459152
LBX2-AS1	-0.035	0.310582625	0.600451336
LLPH-AS1	-0.035	0.31048857	0.600318237
RP11-444B24	-0.035	0.310450458	0.600268917
RP11-567M16	-0.035	0.310340914	0.600178938
AL121989	-0.035	0.310324482	0.600171531

OR7E116P	-0.035	0.310315253	0.600171531
ATIC	-0.035	0.31029634	0.600165846
CMIP	-0.035	0.310254898	0.600134434
RP11-149B9	-0.035	0.310254893	0.600134434
HAVCR2	-0.035	0.310220496	0.600116636
MED15P8	-0.035	0.310168365	0.60004016
RP11-312A15	-0.035	0.310153935	0.60004016
AL157773	-0.035	0.310152515	0.60004016
UGT2A3P7	-0.035	0.310125182	0.60004016
DNM3-IT1	-0.035	0.310106377	0.60004016
APBB2	-0.035	0.309975325	0.599837266
RP11-381K7	-0.035	0.30995147	0.599837266
OR52N3P	-0.035	0.309917643	0.599798756
RP5-867C24	-0.035	0.309899301	0.599787631
ZNF148	-0.035	0.309890938	0.599787631
FDPSP3	-0.035	0.309878754	0.599787631
ELDR	-0.035	0.309877317	0.599787631
MIR518A2	-0.035	0.309835593	0.599786196
CTC-204F22	-0.035	0.309769983	0.599683561
NBAS	-0.035	0.309761523	0.599683561
MKRNP2	-0.035	0.309713612	0.599647555
RP11-440E5	-0.035	0.309673015	0.599617705
RP11-731K22	-0.035	0.309491099	0.599338561
CDC73	-0.035	0.309477039	0.599335703
IGHV3-33-2	-0.035	0.309476649	0.599335703
SPATA31A3	-0.035	0.309407401	0.599263511
MRPL2P1	-0.035	0.309402312	0.599263511
SP2-AS1	-0.035	0.309385198	0.599263511
AC004837	-0.035	0.309330796	0.599223046
RP11-467I20	-0.035	0.309273512	0.599136448
LRRC39	-0.035	0.309230175	0.599124332
KRT128P	-0.035	0.309201428	0.599094277
GUCA2A	-0.035	0.3091636	0.599045352
SLC16A12	-0.035	0.309140741	0.599025431
ADRB2	-0.035	0.309132142	0.599025431
MIR548I2	-0.035	0.309081377	0.599007883
MUC5B	-0.035	0.309026805	0.598926494
RP11-412B14	-0.035	0.309023767	0.598926494
SPTBN2	-0.035	0.30888338	0.598745981
SAR1B	-0.035	0.30886415	0.598733073
MRPS18CP4	-0.035	0.308851037	0.598732024
CYB5B	-0.035	0.308779703	0.598618103
RP11-74M11	-0.035	0.308748757	0.598582474
RP11-397K18	-0.035	0.308743064	0.598582474
DNM1P34	-0.035	0.308664778	0.598492749
TBC1D16	-0.035	0.308642543	0.598474
ZFYVE19	-0.035	0.308636309	0.598474
FRG1B	-0.035	0.308599428	0.598463495
RP11-291C6	-0.035	0.308594588	0.598463495
C16orf97	-0.035	0.308588831	0.598463495
LINC00690	-0.035	0.308437393	0.598319799
RP11-143J24	-0.035	0.308397437	0.598266659

MTND3P9	-0.035	0.308338516	0.598201088
RP3-510L9	-0.035	0.308329079	0.598201088
RP11-278L15	-0.035	0.308278682	0.598201088
SERTAD3	-0.035	0.308250506	0.59817654
OR1AB1P	-0.035	0.30824257	0.59817654
RP11-377H23	-0.035	0.308229497	0.59817654
RP1-142O9	-0.035	0.308178654	0.59817654
RP4-742N3	-0.035	0.308102296	0.59810826
HEIH	-0.035	0.308082368	0.59810826
AC074290	-0.035	0.308034881	0.598050519
AC005204	-0.035	0.30803127	0.598050519
PRKAR2A	-0.035	0.308005325	0.598041894
AL354984	-0.035	0.307976258	0.598034216
RNU6-979P	-0.035	0.30792501	0.597959083
LINC00710	-0.035	0.307881705	0.597899366
RNU6-14P	-0.035	0.307827245	0.597869899
RN7SKP61	-0.035	0.307819647	0.597869899
MIR515-2	-0.035	0.307819647	0.597869899
AKR1C8P	-0.035	0.307792331	0.597869899
CCDC162P	-0.035	0.307785162	0.597869899
PHRF1	-0.035	0.307602938	0.597685284
RP4-734G22	-0.035	0.307573654	0.597666684
RNU6-634P	-0.035	0.307564056	0.597666684
RN7SL74P	-0.035	0.307490488	0.597578221
RP11-231I16	-0.035	0.30746248	0.597548173
UGT3A1	-0.035	0.307370143	0.597441857
KB-1930G5	-0.035	0.307360334	0.597441857
RP11-850A17	-0.035	0.307351657	0.597441857
AP000266	-0.035	0.307296283	0.597420201
CTD-2026K11	-0.035	0.307220319	0.597296899
OR2D3	-0.035	0.307187432	0.597268422
AC003682	-0.035	0.307124044	0.597182855
RP4-555D20	-0.035	0.307010242	0.597007822
LYZL6	-0.035	0.306982367	0.597002474
RPL34P33	-0.035	0.306974131	0.597002474
RP11-561N12	-0.035	0.30695915	0.597002474
RP5-884M20	-0.035	0.306918656	0.596954092
CHRDL1	-0.035	0.306914079	0.596954092
MTND4P14	-0.035	0.306898575	0.596954092
CERS2	-0.035	0.306893128	0.596954092
RP11-29O13	-0.035	0.306861343	0.596954092
RNASEL	-0.035	0.306861301	0.596954092
RNU6-664P	-0.035	0.306835712	0.596954092
RP11-67M9	-0.035	0.306698508	0.596778812
IFT122	-0.035	0.306693923	0.596778812
RNA5SP329	-0.035	0.306672367	0.596778812
SLC9A9-AS2	-0.035	0.306643876	0.596778812
TRIM60P17	-0.035	0.306604383	0.596757129
PDCD4	-0.035	0.30659306	0.596757129
ZNF704	-0.035	0.306539793	0.596680183
CENPH	-0.035	0.306390366	0.596486836
HERC2P11	-0.035	0.306350753	0.59645848

ZNF415P1	-0.035	0.306288545	0.596361741
AC068042	-0.035	0.306255727	0.59632222
RNF224	-0.035	0.306203265	0.596293204
MIR3202-1	-0.035	0.30613891	0.596265402
RNA5SP25	-0.035	0.306120894	0.596265402
RNA5-8SP5	-0.035	0.306098937	0.596265402
AC021021	-0.035	0.306091681	0.596265402
KIAA0513	-0.035	0.306069442	0.596265402
PNMA1	-0.035	0.306047745	0.596265402
PTTG2	-0.035	0.306032888	0.596265402
RNA5SP180	-0.035	0.306016302	0.596265402
FBXO30	-0.035	0.305939792	0.596218933
SNURF	-0.035	0.305875486	0.596118003
AL121584	-0.035	0.305850672	0.596094036
ARAFP2	-0.035	0.305840551	0.596094036
CTC-344H19	-0.035	0.305783746	0.596012378
RP11-806N19	-0.035	0.305781347	0.596012378
OR7E87P	-0.035	0.305766806	0.596012378
KLF15	-0.035	0.305730415	0.596005995
HINT2	-0.035	0.305706927	0.595984602
AC007563	-0.035	0.305701003	0.595984602
MIR516A2	-0.035	0.305654349	0.59598407
DDX10P2	-0.035	0.305642392	0.59598407
PCYT2	-0.035	0.305583056	0.595913859
IRS1	-0.035	0.305491464	0.59575964
RP1-111C20	-0.035	0.305480887	0.59575964
RP11-163M18	-0.035	0.305473118	0.59575964
AC013404	-0.035	0.305420445	0.595718713
RP11-815N9	-0.035	0.30541432	0.595718713
CTC-360G5	-0.035	0.305366618	0.595662513
MIR6877	-0.035	0.305360529	0.595662513
NCOA1	-0.035	0.305302661	0.595610938
LINC00498	-0.035	0.305299977	0.595610938
STOX1	-0.035	0.305291315	0.595610938
RPS3P6	-0.035	0.305271656	0.595610938
RNU7-110P	-0.035	0.305222501	0.595576534
AC016582	-0.035	0.305169733	0.595522366
VN1R93P	-0.035	0.305169479	0.595522366
RAD23A	-0.035	0.305167554	0.595522366
AC110620	-0.035	0.305156512	0.595522366
LINC00092	-0.035	0.305150843	0.595522366
TMED2	-0.035	0.305147189	0.595522366
KRTAP20-3	-0.035	0.305090803	0.595522366
TAPBP	-0.035	0.305087128	0.595522366
RP11-25D3	-0.035	0.305041381	0.595491489
RN7SKP169	-0.035	0.305007071	0.595448917
IGLVVI-22-1	-0.035	0.304983006	0.595426342
MIR4280	-0.035	0.304954153	0.595418827
SNORD79	-0.035	0.304909379	0.595380221
RP11-423G4	-0.035	0.304891256	0.595380221
GABBR1	-0.035	0.304885094	0.595380221
RNA5SP284	-0.035	0.304817218	0.59529789

EML2	-0.035	0.304811414	0.59529789
RP3-425C14	-0.035	0.304779731	0.595273497
TUBG1	-0.035	0.304772261	0.595273497
LINC01446	-0.035	0.304730681	0.595226515
POM121L7	-0.035	0.304721785	0.595226515
CH507-338C24	-0.035	0.304717079	0.595226515
RPL18AP8	-0.035	0.3046448	0.595156401
MAP2K5	-0.035	0.304626237	0.595144549
RP11-512M8	-0.035	0.304623205	0.595144549
TRIM49D1	-0.035	0.304609275	0.595144549
RP11-316M21	-0.035	0.304598944	0.595144549
PDZRN3-AS1	-0.035	0.304593476	0.595144549
QTRT1P1	-0.035	0.304590856	0.595144549
ENPP7P10	-0.035	0.304560687	0.595144549
RERGL	-0.035	0.304543274	0.595144549
PLA2G2C	-0.035	0.304537541	0.595144549
AC126755	-0.035	0.304502228	0.595144549
CYP4F12	-0.035	0.304305616	0.594905141
TPX2	-0.035	0.304298047	0.594905141
CTD-2194L12	-0.035	0.304242284	0.594858051
MIR5692C2	-0.035	0.304182405	0.594814231
RPL5P25	-0.035	0.304137389	0.594799466
RP11-520B13	-0.035	0.304112047	0.594774329
OR2B3	-0.035	0.304035781	0.59467401
RP11-342A1	-0.035	0.304033796	0.59467401
TPRX2P	-0.035	0.303994608	0.594642319
RP3-341O5	-0.035	0.303950832	0.594581109
SSU72P8	-0.035	0.303886265	0.59450364
DISC1FP1	-0.035	0.30384126	0.594440013
CNN2P8	-0.035	0.30384123	0.594440013
BAK1P2	-0.035	0.303840501	0.594440013
STK24-AS1	-0.035	0.303758524	0.594351393
RP11-148K1	-0.035	0.303745931	0.59435117
SOGA3	-0.035	0.303689557	0.594265275
RPL17P20	-0.035	0.303615496	0.594169176
EXO1	-0.035	0.303563753	0.594092326
EIF4EBP2	-0.035	0.303517256	0.594025739
MAP1LC3B	-0.035	0.303488744	0.593994346
AP000855	-0.035	0.303475747	0.593993318
RP3-337O18	-0.035	0.303461659	0.593993318
AL672296	-0.035	0.303417468	0.593952476
MIR3929	-0.035	0.303411912	0.593952476
MARK2P12	-0.035	0.303387276	0.593952476
AC137590	-0.035	0.303369744	0.593952476
UBB	-0.035	0.303339534	0.59394639
PEX11B	-0.035	0.303338507	0.59394639
RP11-643A5	-0.035	0.303295217	0.59394639
AC008991	-0.035	0.303163431	0.593727599
OR2L2	-0.035	0.303147477	0.593727599
PKMP3	-0.035	0.303140321	0.593727599
RP11-237N19	-0.035	0.30308259	0.593662946
TIGD1	-0.035	0.302953252	0.593507242

RP11-407A16		-0.035	0.302948419	0.593507242
SNORD11		-0.035	0.302936831	0.593507242
AC123886		-0.035	0.302926074	0.593507242
MIR4673		-0.035	0.302902941	0.59350633
CTD-3154N5		-0.035	0.302893371	0.59350633
RAB36		-0.035	0.302864492	0.593488274
RP11-219C24		-0.035	0.302792052	0.593411121
IFNA14		-0.035	0.302789015	0.593411121
ONECUT1		-0.035	0.302690488	0.5932609
	14-Sep	-0.035	0.302636883	0.593180248
AC073464		-0.035	0.30257585	0.59309882
SEC14L1P1		-0.035	0.302513087	0.593084048
TMEM248P1		-0.035	0.302459426	0.593003255
PGGT1BP1		-0.035	0.302415866	0.592974345
BIRC2		-0.035	0.302376876	0.592939044
CCDC74B		-0.035	0.302356122	0.592922758
RBPJP5		-0.035	0.302317129	0.592895117
TAAR2		-0.035	0.302312161	0.592895117
LINC01169		-0.035	0.302303763	0.592895117
RP11-191G24		-0.035	0.302278751	0.592893093
RP11-388B24		-0.035	0.30210057	0.592641233
RP11-1250I15		-0.035	0.302092652	0.592641233
CT45A10		-0.035	0.301872945	0.592267878
RP11-325B23		-0.035	0.301871088	0.592267878
RP11-474I16		-0.035	0.301870457	0.592267878
FAM174B		-0.035	0.301869818	0.592267878
VTA1		-0.035	0.301866491	0.592267878
CMTM5		-0.035	0.301860088	0.592267878
RP11-51L5		-0.035	0.301860082	0.592267878
AP001630		-0.035	0.301854726	0.592267878
RP11-372B4		-0.035	0.301841409	0.592267878
RNU6-744P		-0.035	0.301752538	0.592267878
SH3BGR		-0.035	0.301683526	0.592262214
NPHP3		-0.035	0.301605757	0.592182767
SLC25A20P1		-0.035	0.30159159	0.592179365
CXorf23		-0.035	0.301547694	0.592117586
NBPF17P		-0.035	0.301539692	0.592117586
GUSBP2		-0.035	0.301497823	0.592068478
MIR4286		-0.035	0.301443443	0.592027911
MIR548F5		-0.035	0.301427956	0.592027911
NMD3P2		-0.035	0.301409654	0.592027911
CHIAP1		-0.035	0.3013663	0.592005477
AC099791		-0.035	0.30133435	0.591998938
RNU6ATAC9P		-0.035	0.301313072	0.591998584
RPL14P1		-0.035	0.301291234	0.591980099
RBM22		-0.035	0.301278708	0.59197991
RP11-28H5		-0.035	0.301253686	0.591955165
RP11-278I4		-0.035	0.301242454	0.591955165
RP11-347J14		-0.035	0.301174882	0.591849154
SPTA1		-0.035	0.301087821	0.591751316
PAK2		-0.035	0.300965434	0.591584016
RNU6ATAC35P		-0.035	0.300868476	0.591442253

KIAA1715	-0.035	0.300863781	0.591442253
CTDSPL	-0.035	0.300821135	0.59139801
PRRT1	-0.035	0.300812197	0.59139801
RP11-101C11	-0.035	0.300807165	0.59139801
RP11-165F24	-0.035	0.300801853	0.59139801
RN7SL659P	-0.035	0.300752519	0.59138517
TMCO2	-0.035	0.300737752	0.591380549
CFAP61	-0.035	0.300697559	0.591325928
AC079781	-0.035	0.30069733	0.591325928
RP11-173A8	-0.035	0.300613202	0.591233275
RFPL4AL1	-0.035	0.30060934	0.591233275
RP11-239C9	-0.035	0.300606037	0.591233275
MIR4309	-0.035	0.300600525	0.591233275
CTA-38K21	-0.035	0.300556474	0.591219362
RNA5SP465	-0.035	0.300517667	0.591191859
MAP3K9	-0.035	0.300499501	0.591180541
RP11-591L14	-0.035	0.300437701	0.591083377
RN7SL62P	-0.035	0.300437016	0.591083377
RPL35AP33	-0.035	0.300416185	0.591083377
RP13-348B13	-0.035	0.300404591	0.591083377
CH17-219N22	-0.035	0.300366224	0.591083377
AC010740	-0.035	0.300268279	0.590920925
MIRLET7BHG	-0.035	0.300229607	0.59087325
HMGN1P16	-0.035	0.300107418	0.59072951
AC023347	-0.035	0.30008112	0.590723465
RPL6P7	-0.035	0.300015823	0.59064375
OR5BK1P	-0.035	0.300012114	0.59064375
SNORA45B	-0.035	0.299979954	0.590621963
AC007948	-0.035	0.299860967	0.590459144
SRBD1	-0.035	0.299845198	0.590454281
AC007680	-0.035	0.299844156	0.590454281
RP11-846C15	-0.035	0.299819906	0.590453303
AC098872	-0.035	0.299816655	0.590453303
OR12D3	-0.035	0.299805083	0.590453303
RNU6-572P	-0.035	0.299649391	0.590215118
SLC9A9-AS1	-0.035	0.299612437	0.590181648
RP4-745E8	-0.035	0.299588283	0.590167975
MROH4P	-0.035	0.299521874	0.59011038
LINC00606	-0.035	0.29939833	0.589891377
CCSER2	-0.035	0.299352166	0.589849226
OR10T2	-0.035	0.29934506	0.589849226
RN7SL213P	-0.035	0.299326534	0.589847525
RP11-29B2	-0.035	0.299233	0.589760814
SMG1P1	-0.035	0.299179298	0.589679376
FABP5P12	-0.035	0.29915596	0.589679376
FOXRED2	-0.035	0.299139942	0.589675015
CECR2	-0.035	0.29913052	0.589675015
EEF1GP6	-0.035	0.299118974	0.589675015
RHBDL3	-0.035	0.299060829	0.589616679
HSPD1P7	-0.035	0.299016309	0.589602126
RP11-753B7	-0.035	0.298994028	0.589602126
RNFT2	-0.035	0.298969523	0.5896

RP11-871F6	-0.035	0.298920428	0.589535104
SEPHS1P4	-0.035	0.29889113	0.589501733
MIR6861	-0.035	0.298852877	0.589450697
ACSF3	-0.035	0.298852302	0.589450697
DUSP8	-0.035	0.298839082	0.589450697
ANP32A	-0.035	0.298827641	0.589450697
RP11-232L2	-0.035	0.298795237	0.589450697
OR5AS1	-0.035	0.298765046	0.589438153
KLHL21	-0.035	0.298761547	0.589438153
RP11-483G21	-0.035	0.298749093	0.589438153
SDHDP6	-0.035	0.29873297	0.589438153
CTC-565M22	-0.035	0.298693547	0.589438153
DCTD	-0.035	0.298637773	0.589392559
HMGB1P13	-0.035	0.298611248	0.589364631
GPCPD1	-0.035	0.298597116	0.589361162
AC024995	-0.035	0.298590937	0.589361162
AC098649	-0.035	0.29849955	0.589290697
C4orf33	-0.035	0.298487359	0.589290697
AL020997	-0.035	0.298409035	0.589160847
CTC-248O19	-0.035	0.298322791	0.589112678
CCT8P1	-0.035	0.298285701	0.589063858
RP11-257I14	-0.035	0.298259587	0.589061139
OTOA	-0.035	0.298219432	0.589030684
ETS1	-0.035	0.298218163	0.589030684
RP11-57G22	-0.035	0.298175369	0.589016934
HSPE1P10	-0.035	0.298132388	0.588956459
AC002306	-0.035	0.29812594	0.588956459
RP11-447M4	-0.035	0.298121378	0.588956459
Z69890	-0.035	0.298092746	0.588951432
FAM24B	-0.035	0.298070681	0.588951432
RP11-39H3	-0.035	0.298064592	0.588951432
HUS1	-0.035	0.298026523	0.58892834
MIR3148	-0.035	0.298024134	0.58892834
MTND5P27	-0.035	0.298002657	0.58892834
OR7E22P	-0.035	0.297886305	0.588812246
ERVWE2	-0.035	0.297884006	0.588812246
CT867976	-0.035	0.297815899	0.58877082
RP11-45J1	-0.035	0.297789518	0.588743103
SVILP1	-0.035	0.297770029	0.588743103
MIR3923	-0.035	0.297749936	0.588743103
KLF17P1	-0.035	0.297739098	0.588743103
RP11-436D10	-0.035	0.297717742	0.588743103
RP11-269C23	-0.035	0.297665004	0.588716878
MEF2D	-0.035	0.297658449	0.588716878
UBC	-0.035	0.297622697	0.588682097
AL590558	-0.035	0.297610033	0.588681495
VIL1	-0.035	0.297562164	0.588611254
DDX39BP2	-0.035	0.297513548	0.588539531
POLHP1	-0.035	0.297479211	0.588496049
XXbac-BPG181B23	-0.035	0.297458431	0.588479384
TRAF4	-0.035	0.297427258	0.5884666
HNRNPH1P2	-0.035	0.29737982	0.588421632

SIGLEC19P		-0.035	0.297331984	0.588375869
RNU5A-4P		-0.035	0.29731201	0.588360789
RP5-1014O16		-0.035	0.29727357	0.58833361
AC016561		-0.036	0.297159441	0.588132177
TMEM14B		-0.036	0.297144263	0.588126576
RPS17P5		-0.036	0.297097649	0.588058754
MTA2		-0.036	0.297043978	0.588001395
MIR1294		-0.036	0.297042631	0.588001395
RP11-113A11		-0.036	0.297027038	0.588001395
INPP5K		-0.036	0.296956374	0.587950166
RP4-738P15		-0.036	0.296916156	0.587923411
AC004947		-0.036	0.296911957	0.587923411
UCHL5		-0.036	0.296900682	0.587923411
	9-Mar	-0.036	0.296870053	0.587923411
RNU6-863P		-0.036	0.296813858	0.587900372
IQGAP3		-0.036	0.296795137	0.587900372
AIFM2		-0.036	0.296784508	0.587900372
RP11-144L1		-0.036	0.296772098	0.587900372
AC025284		-0.036	0.29675517	0.587900372
UBE2N		-0.036	0.296736175	0.587900372
AC093639		-0.036	0.296679851	0.587842546
RP11-576N17		-0.036	0.296578262	0.587739063
RFESDP1		-0.036	0.296565453	0.587738133
ZNF736P2Y		-0.036	0.296547666	0.587727338
RNF220		-0.036	0.296437688	0.587558275
GALR3		-0.036	0.296425529	0.587558275
OR52M2P		-0.036	0.296424389	0.587558275
RNU6-456P		-0.036	0.296413531	0.587558275
KDSR		-0.036	0.296407851	0.587558275
RNU7-140P		-0.036	0.296401653	0.587558275
PHF6		-0.036	0.296395461	0.587558275
RPL7AP11		-0.036	0.296328713	0.587513429
MIR1273G		-0.036	0.296292475	0.587490496
KCNQ5-IT1		-0.036	0.296218392	0.587368056
WRAP73		-0.036	0.296199296	0.587354645
MIR6076		-0.036	0.296191432	0.587354645
OR8H3		-0.036	0.296156787	0.587343714
IGHVIII-5-2		-0.036	0.296140488	0.587335846
RNA5SP303		-0.036	0.296100123	0.587304704
RNA5SP73		-0.036	0.296070191	0.587269793
RPL36P19		-0.036	0.29605159	0.587267643
EIF3A		-0.036	0.296029491	0.587262437
AL139383		-0.036	0.295950731	0.587135048
RP11-420H19		-0.036	0.295950404	0.587135048
AC019011		-0.036	0.295806329	0.586963721
ZNF729		-0.036	0.295745388	0.5868944
CTA-972D3		-0.036	0.295671666	0.58677255
C6orf57		-0.036	0.295654231	0.5867624
RPL5P11		-0.036	0.29561499	0.586708969
LINC00517		-0.036	0.295587099	0.586696003
AC104984		-0.036	0.295560252	0.586673674
AC023818		-0.036	0.295485562	0.586608212

PPP1R12BP1	-0.036	0.295455899	0.586608212
RP11-2F20	-0.036	0.295420267	0.586566934
KIAA1024L	-0.036	0.295300988	0.586403442
SCARNA16	-0.036	0.295287169	0.58640045
ADAM29	-0.036	0.295281769	0.58640045
AC140076	-0.036	0.295275318	0.58640045
CYP3A54P	-0.036	0.295262157	0.58640045
GON4L	-0.036	0.29522532	0.586375425
AMBN	-0.036	0.295169635	0.586289273
RP11-1406H17	-0.036	0.295130192	0.586235378
AL020993	-0.036	0.295080654	0.586161424
TACC1	-0.036	0.295022363	0.586070076
MIR3976HG	-0.036	0.295018842	0.586070076
KRT89P	-0.036	0.295014817	0.586070076
SCUBE1	-0.036	0.294988084	0.586070076
OR10H4	-0.036	0.294976178	0.586070076
USP17L2	-0.036	0.294962249	0.586070076
AC006196	-0.036	0.294957561	0.586070076
AC002984	-0.036	0.294906223	0.586010459
SNORA2B	-0.036	0.294826729	0.58587694
MIR23B	-0.036	0.294812977	0.585874057
CFL1P5	-0.036	0.29479606	0.585864884
RP11-425A23	-0.036	0.294742318	0.585806969
TUBB4BP2	-0.036	0.294741084	0.585806969
REREP2Y	-0.036	0.294696148	0.585764093
AC239859	-0.036	0.294696148	0.585764093
TAS2R15P	-0.036	0.294660055	0.585741244
AC104623	-0.036	0.294628442	0.585727296
CASC4	-0.036	0.294502917	0.585587812
RP11-872J21	-0.036	0.294490328	0.585587812
RP11-518I13	-0.036	0.294420408	0.585509239
SNORD27	-0.036	0.294373744	0.585465332
CTD-2131I18	-0.036	0.294343572	0.585429772
AL356791	-0.036	0.294311825	0.585415524
SNORD1B	-0.036	0.294233614	0.585308844
SNORA62	-0.036	0.294195942	0.585282795
OR2U1P	-0.036	0.294191897	0.585282795
DDX4	-0.036	0.294182715	0.585282795
SHANK2-AS3	-0.036	0.294107626	0.585204876
SLC23A3	-0.036	0.294093224	0.585200668
SIGLEC16	-0.036	0.29404867	0.585160907
RP11-454P7	-0.036	0.293965326	0.585064576
RNA5SP482	-0.036	0.293949045	0.585060436
RP11-760H22	-0.036	0.293936306	0.585060436
SBF1	-0.036	0.293879987	0.584996327
SEZ6L	-0.036	0.293874131	0.584996327
RP11-199F11	-0.036	0.293792254	0.584872606
F13B	-0.036	0.293772396	0.584872606
RP11-452K20	-0.036	0.29372011	0.584800287
AIDA	-0.036	0.293645736	0.58467665
HSPH1	-0.036	0.293627394	0.584664571
MICAL1	-0.036	0.293332509	0.584199523

ENO2	-0.036	0.293320456	0.584199523
RP11-617B3	-0.036	0.293267186	0.584142707
MTND5P30	-0.036	0.293242418	0.584117801
RP11-767C1	-0.036	0.293219778	0.584097134
GAPDHP29	-0.036	0.293219069	0.584097134
MUT	-0.036	0.293208234	0.584097134
L1CAM	-0.036	0.2931874	0.584097134
UBE2V1P5	-0.036	0.293175224	0.584097134
LGALS8-AS1	-0.036	0.293140418	0.584097134
TREML5P	-0.036	0.293081948	0.584017987
RPL12P9	-0.036	0.293025108	0.583953588
CYP2U1	-0.036	0.292957784	0.583868284
HMGB3P19	-0.036	0.292947761	0.583868284
RP11-148L24	-0.036	0.292927434	0.583868284
ZNF7	-0.036	0.292908614	0.583868019
RNU6-786P	-0.036	0.292901525	0.583868019
HSFY5P	-0.036	0.29285238	0.583804793
RP11-796E2	-0.036	0.292812002	0.583764822
MPPED2	-0.036	0.29276239	0.583698691
SLC30A5	-0.036	0.29270823	0.583676292
CH507-152C13	-0.036	0.292707864	0.583676292
HSPE1P20	-0.036	0.292701445	0.583676292
CTB-20D2	-0.036	0.292673382	0.583676292
RP11-577B7	-0.036	0.292655247	0.583676292
SLC7A11	-0.036	0.292625494	0.583645647
MIR577	-0.036	0.29260737	0.58363394
NMTRQ-TTG12-1	-0.036	0.292606657	0.58363394
RP11-141C7	-0.036	0.292529365	0.58357611
RP3-431C21	-0.036	0.292477263	0.583496612
ZNF317P1	-0.036	0.292473886	0.583496612
RP11-282I1	-0.036	0.292427224	0.583445662
AP000402	-0.036	0.292393491	0.583402798
HNRNPA1P63	-0.036	0.292347454	0.583382791
RP4-681L3	-0.036	0.29231098	0.583335918
AGK	-0.036	0.292300972	0.583335918
SPATA32	-0.036	0.29226165	0.583310799
RP11-967K21	-0.036	0.29222182	0.583255747
RD3L	-0.036	0.292062572	0.582968298
RP11-261P13	-0.036	0.291872739	0.582681097
OR52B2	-0.036	0.291850073	0.582660271
INSIG1	-0.036	0.291808761	0.582602217
INMT-FAM188B	-0.036	0.291730975	0.582569021
FSD1	-0.036	0.291707834	0.582547236
TNR-IT1	-0.036	0.291702168	0.582547236
RP5-1166F10	-0.036	0.291696536	0.582547236
PAICSP6	-0.036	0.291682992	0.582547236
ABCA6	-0.036	0.291676165	0.582547236
MIR6775	-0.036	0.291645768	0.582547236
MIR3619	-0.036	0.291598781	0.582547236
MIR664A	-0.036	0.2915558	0.582512294
DAK	-0.036	0.291542609	0.582510991
CTB-147N14	-0.036	0.291479596	0.582433343

RP11-586E1	-0.036	0.291463888	0.582433343
RP11-317B17	-0.036	0.29146012	0.582433343
IGSF11-AS1	-0.036	0.291458733	0.582433343
EPB41L1	-0.036	0.291394844	0.582386162
TRIM60P15	-0.036	0.291348868	0.582382369
CTD-2353F22	-0.036	0.291330961	0.58238068
CHCHD7	-0.036	0.291324605	0.58238068
CTD-2117L12	-0.036	0.291318935	0.58238068
MIR516B1	-0.036	0.291286671	0.58238068
AC004076	-0.036	0.291245381	0.582331812
RNU4-10P	-0.036	0.291221847	0.58232678
ETFDH	-0.036	0.291180663	0.582275745
SMCO3	-0.036	0.291116909	0.582197145
PPM1J	-0.036	0.291027776	0.582067774
TSPY8	-0.036	0.291016359	0.582067774
OFD1P16Y	-0.036	0.291016359	0.582067774
NPY5R	-0.036	0.290957121	0.582024227
RP11-343B5	-0.036	0.290929549	0.582017963
SDHC	-0.036	0.290847979	0.581903663
LINC01435	-0.036	0.290824203	0.581880538
MIR4475	-0.036	0.290820378	0.581880538
MIR4696	-0.036	0.290813358	0.581880538
RP1-251I12	-0.036	0.290773419	0.581880538
RPSAP54	-0.036	0.290752367	0.581880538
AC079630	-0.036	0.290692571	0.581788254
ZNF419	-0.036	0.290621091	0.581669638
FAM149A	-0.036	0.290563951	0.581604157
GPR143	-0.036	0.290550478	0.581601632
USP20	-0.036	0.290537505	0.581600107
CCNG2	-0.036	0.290506819	0.581587569
RP5-933E2	-0.036	0.29048869	0.581575721
SDCCAG3P1	-0.036	0.290439697	0.581502079
GS1-421I3	-0.036	0.290353169	0.581402161
ERVH48-1	-0.036	0.290293716	0.581325238
RNU1-87P	-0.036	0.290273652	0.581316256
KRTAP10-1	-0.036	0.290259927	0.581313215
SHD	-0.036	0.290258404	0.581313215
RAMP2	-0.036	0.290210301	0.581274293
LRRC53	-0.036	0.290128477	0.581147704
CRNDE	-0.036	0.290019629	0.58096459
AC105339	-0.036	0.289991182	0.580945993
RP11-253A20	-0.036	0.289983777	0.580945993
RP11-470C13	-0.036	0.289978028	0.580945993
LRRC7	-0.036	0.289945128	0.580945993
FKSG52	-0.036	0.289942577	0.580945993
CCDC15	-0.036	0.289932671	0.580945993
GPR20	-0.036	0.289890165	0.580945993
RP3-514A23	-0.036	0.289869459	0.580945993
DYNLRB1	-0.036	0.289865907	0.580945993
AQP3	-0.036	0.289832529	0.58092138
PDE4DIP1	-0.036	0.289802215	0.580885068
CHIC1	-0.036	0.289790436	0.580885068

RPL18P10	-0.036	0.289774876	0.580879163
SRD5A3	-0.036	0.289735398	0.580843941
RNU6-299P	-0.036	0.289696457	0.5808279
ZNF610	-0.036	0.28961414	0.580742013
KLF3-AS1	-0.036	0.289611651	0.580742013
SNORA59A	-0.036	0.289608095	0.580742013
RNY1P8	-0.036	0.289605438	0.580742013
RP11-1157N2__B	-0.036	0.289581258	0.580742013
CDK3	-0.036	0.289546679	0.580742013
MIR632	-0.036	0.28954169	0.580742013
MYO5BP2	-0.036	0.289515918	0.580742013
PRPS1L1	-0.036	0.289419198	0.580630502
GS1-345D13	-0.036	0.289388349	0.580617529
NDUFS5P5	-0.036	0.289338494	0.580581558
FHL5	-0.036	0.28932184	0.580581558
TGIF1	-0.036	0.28929684	0.580556212
BCRP3	-0.036	0.289288427	0.580556212
HSPA9P1	-0.036	0.289279798	0.580556212
ALOX15P2	-0.036	0.289265493	0.580556212
LPGAT1	-0.036	0.289262013	0.580556212
PTMAP1	-0.036	0.28925407	0.580556212
RP3-467L1	-0.036	0.289243357	0.580556212
C3	-0.036	0.289176949	0.580533647
WDR44	-0.036	0.289155561	0.580533647
ZNF639	-0.036	0.289126465	0.580527142
EEF1GP7	-0.036	0.289076753	0.580481428
ABCA1	-0.036	0.289061318	0.580474908
RP1-196A12	-0.036	0.289056859	0.580474908
AC004490	-0.036	0.289046591	0.580474908
AL596220	-0.036	0.288951695	0.580352649
ACTR3BP3	-0.036	0.288873166	0.580219395
UBE2C	-0.036	0.288860716	0.580219395
RNF43	-0.036	0.28874194	0.580078137
ELOVL2	-0.036	0.288741692	0.580078137
AL590684	-0.036	0.288738194	0.580078137
KRT18P37	-0.036	0.288704913	0.580078137
LINC01330	-0.036	0.288702123	0.580078137
DFNB59	-0.036	0.288697222	0.580078137
RP4-657D16	-0.036	0.288676851	0.580078137
ANKRD28	-0.036	0.288538011	0.579913063
RN7SL154P	-0.036	0.288529947	0.579913063
PRH2	-0.036	0.288481521	0.579848467
RP5-1182A14	-0.036	0.288478558	0.579848467
SCARA5	-0.036	0.288424181	0.57978215
SLC13A1	-0.036	0.288413611	0.57978215
PIK3R4	-0.036	0.288351803	0.579686587
RP11-536C12	-0.036	0.288351461	0.579686587
HLA-V	-0.036	0.28834527	0.579686587
ACTG1P13	-0.036	0.28832668	0.579686587
PRKRIRP7	-0.036	0.28832319	0.579686587
LGR4	-0.036	0.288286773	0.579686587
SNHG22	-0.036	0.288266668	0.57968572

SEMG2	-0.036	0.288253676	0.579684069
RP11-452N4	-0.036	0.288219068	0.579663425
RNU6-439P	-0.036	0.288193136	0.579635746
AF228730	-0.036	0.288037446	0.579371545
AC005621	-0.036	0.2879882	0.579321422
GDI1	-0.036	0.287914902	0.57919844
NUTM1	-0.036	0.287842884	0.579102482
LINC01562	-0.036	0.287842772	0.579102482
FCHSD1	-0.036	0.287776805	0.579018457
CTD-2233K9	-0.036	0.287761436	0.579011994
CCDC85C	-0.036	0.287698231	0.578933735
PTCH2	-0.036	0.287691539	0.578933735
Z85986	-0.036	0.287682403	0.578933735
AP000244	-0.036	0.28765616	0.578933735
UBA52P6	-0.036	0.287485311	0.578655918
MICB	-0.036	0.287464824	0.578655918
MARK3P1	-0.036	0.287416358	0.578586536
TRAV9-1	-0.036	0.287394392	0.578566773
RP11-597G23	-0.036	0.28739343	0.578566773
XXbac-BPG254F23	-0.036	0.287378461	0.578566773
GAPDHP49	-0.036	0.287366009	0.578566773
COG7	-0.036	0.287361542	0.578566773
SIGLEC26P	-0.036	0.287287637	0.578566773
PMCH	-0.036	0.287274197	0.578566773
AP000563	-0.036	0.287264373	0.578566773
RP11-558F24	-0.036	0.287253255	0.578566773
CWC27	-0.036	0.287250856	0.578566773
ARMC7	-0.036	0.287228327	0.578566773
CDK19	-0.036	0.28722288	0.578566773
SRP19	-0.036	0.287222633	0.578566773
RP11-553E24	-0.036	0.28715379	0.57852257
AL445199	-0.036	0.287138739	0.578516719
TMEM82	-0.036	0.287094528	0.578452115
KLF11	-0.036	0.287046895	0.578380609
RP11-443B9	-0.036	0.287030265	0.578371569
MIR200C	-0.036	0.286935116	0.578326651
DEFB121	-0.036	0.28689655	0.578273393
RP11-617D20	-0.036	0.286892223	0.578273393
C10orf12	-0.036	0.286846246	0.578239277
ASIC1	-0.036	0.286798757	0.578174155
RP13-494C23	-0.036	0.286777058	0.578154883
HNRNPA3P16	-0.036	0.286692796	0.578009473
NSUN5P2	-0.036	0.286634566	0.577941005
ZNF551	-0.036	0.286617755	0.577931576
YWHAEP4	-0.036	0.286392685	0.577624477
RP11-1348G14	-0.036	0.286381481	0.577624477
ZFP64	-0.036	0.286295752	0.577477884
OR2R1P	-0.036	0.286283943	0.577477884
RANP5	-0.036	0.286268639	0.577472107
ATAT1	-0.036	0.286236469	0.57745613
SNORD82	-0.036	0.28620457	0.577435246
MT-ND3	-0.036	0.286200656	0.577435246

AC016903	-0.036	0.286155146	0.577435246
RP5-824H1	-0.036	0.28613536	0.577423363
HBZP1	-0.036	0.286102854	0.57738223
GABPA	-0.036	0.286098421	0.57738223
RP11-863K10	-0.036	0.286051756	0.577328036
RAP2C	-0.036	0.286020156	0.577313187
MIR4742	-0.036	0.285998011	0.577292956
IGKV1D-37	-0.036	0.285991128	0.577292956
AC096753	-0.036	0.285827932	0.577071933
LRIG1	-0.036	0.285823939	0.577071933
CHAF1B	-0.036	0.285812342	0.577071933
RPL36AP48	-0.036	0.285729173	0.576995332
OTOP1	-0.036	0.285725656	0.576995332
GLRX	-0.036	0.285713407	0.576995332
AL360091	-0.036	0.285681227	0.576995332
RP1-163G9	-0.036	0.28564126	0.576958296
RP11-796I2	-0.036	0.28556939	0.576908043
U4atac	-0.036	0.285555388	0.576908043
CTC-451A6	-0.036	0.285544258	0.576908043
CCDC114	-0.036	0.285475417	0.576800343
RP5-1115A15	-0.036	0.28536347	0.576647545
RNA5SP242	-0.036	0.285337991	0.576620524
CLRN2	-0.036	0.285231825	0.576509001
PTPRQ	-0.036	0.285180025	0.576448054
RP3-418A9	-0.036	0.285123438	0.576382597
RP11-498E11	-0.036	0.285064697	0.576361706
CAV3	-0.036	0.284993639	0.576242499
TTLL9	-0.036	0.284918319	0.576139124
PGA4	-0.036	0.284887332	0.576125386
CH17-258A22	-0.036	0.284881395	0.576125386
AKR1C5P	-0.036	0.284827266	0.576101762
IGLVI-38	-0.036	0.284806602	0.576084431
DTWD2	-0.036	0.284714993	0.575923592
ZNF85	-0.036	0.284702441	0.575922663
RP11-22H5	-0.036	0.284682708	0.575922663
B4GALT4-AS1	-0.036	0.284577081	0.57579855
RNU6-1024P	-0.036	0.284493126	0.575694855
PGM5P3-AS1	-0.036	0.284481324	0.575694855
RNU6-662P	-0.036	0.284441994	0.575689225
NRG3	-0.036	0.284434275	0.575689225
RP11-153K16	-0.036	0.284428455	0.575689225
RBMX2P1	-0.036	0.28440283	0.575683352
OR2B6	-0.036	0.284386802	0.575675374
RP11-101K23	-0.036	0.284329919	0.575584693
RPSAP55	-0.036	0.284272052	0.575494342
DCANP1	-0.036	0.28422782	0.575451385
CTD-3022L24	-0.036	0.284222661	0.575451385
CTC-513N18	-0.036	0.28421169	0.575451385
RP11-42D20	-0.036	0.284200965	0.575451385
OR2AJ1	-0.036	0.284174965	0.575442222
ZNF805	-0.036	0.28413777	0.575391368
RPL7L1P10	-0.036	0.284089832	0.575318752

LINC00111		-0.036	0.284040777	0.575243871
ZIM3		-0.036	0.284021572	0.575229436
AMMECR1-IT1		-0.036	0.284020671	0.575229436
RPS3AP29		-0.036	0.284006674	0.575229436
HIAT1		-0.036	0.283997212	0.575229436
RASEF		-0.036	0.283990087	0.575229436
	1-Mar	-0.036	0.28398591	0.575229436
IPO5		-0.036	0.283930635	0.575216486
RN7SKP267		-0.036	0.2838995	0.575177874
CYP17A1-AS1		-0.037	0.28384114	0.575084102
TCF3P1		-0.037	0.283829938	0.575084102
KRT18P43		-0.037	0.283758372	0.575002181
RNU6-582P		-0.037	0.283684815	0.574889656
GREB1		-0.037	0.283655105	0.574853908
RP11-449I17		-0.037	0.283625775	0.574818927
CTD-2201I18		-0.037	0.283604412	0.574800091
AC073137		-0.037	0.283593258	0.574800091
EIF2S2P4		-0.037	0.283584454	0.574800091
EEF1A1P30		-0.037	0.283573991	0.574800091
FXR1		-0.037	0.283557166	0.574800091
MSH6		-0.037	0.283480844	0.574745312
CCNYL2		-0.037	0.283462701	0.574732996
PIGHP1		-0.037	0.283413943	0.57466881
HDAC10		-0.037	0.283396929	0.57466881
AC062028		-0.037	0.283368278	0.574639402
RNA5SP501		-0.037	0.283347823	0.574622388
RP11-159H22		-0.037	0.283345993	0.574622388
SNX25		-0.037	0.283343204	0.574622388
RP11-324E6		-0.037	0.283343201	0.574622388
NF1P6		-0.037	0.283342703	0.574622388
APPL2		-0.037	0.283299296	0.574622388
RP11-968A15		-0.037	0.283277832	0.574622388
ZNF785		-0.037	0.283276976	0.574622388
RNU1-109P		-0.037	0.283276168	0.574622388
RP11-185E12		-0.037	0.283269548	0.574622388
RN7SKP82		-0.037	0.283233914	0.574622388
CREB3L4		-0.037	0.28315534	0.574622388
MUS81		-0.037	0.283068059	0.574519421
AC021016		-0.037	0.282958002	0.574369891
ADGB		-0.037	0.282941672	0.574369891
RBMXP1		-0.037	0.282918822	0.574369891
RP11-407B7		-0.037	0.282900656	0.574369891
PGM5		-0.037	0.28288473	0.574369891
FEN1P1		-0.037	0.282778416	0.574331985
RP11-416A14		-0.037	0.282771487	0.574331985
AC097506		-0.037	0.282752107	0.574331985
RARB		-0.037	0.282714218	0.574291139
RAB35		-0.037	0.282658865	0.574208666
NUP205		-0.037	0.282592256	0.574203256
RP11-723D22		-0.037	0.282442028	0.573934059
ZNF333		-0.037	0.282422842	0.573919559
RNA5SP246		-0.037	0.2824009	0.573899457

RP11-317L10	-0.037	0.28236029	0.573841413
RNU6-602P	-0.037	0.282359852	0.573841413
MIR766	-0.037	0.282320677	0.573809879
MLEC	-0.037	0.282320052	0.573809879
RP11-45P22	-0.037	0.282310553	0.573809879
LINC01099	-0.037	0.282309752	0.573809879
RN7SL801P	-0.037	0.282274383	0.573809879
EPRS	-0.037	0.282236126	0.573784948
RP11-478H16	-0.037	0.282204489	0.573769613
RPL21P126	-0.037	0.282182657	0.573749719
PLA2G4E-AS1	-0.037	0.282178159	0.573749719
RP11-397C18	-0.037	0.282163894	0.573749719
AP000866	-0.037	0.282128694	0.573713475
PPP1R27	-0.037	0.282069262	0.57361711
NMNAT3	-0.037	0.282068569	0.57361711
CH17-132F21	-0.037	0.282056036	0.57361711
RNU6-417P	-0.037	0.28203505	0.57361711
RPL12P44	-0.037	0.28202294	0.57361711
ITPKA	-0.037	0.282016725	0.57361711
AC092601	-0.037	0.281931148	0.57361711
MIR548AY	-0.037	0.281849071	0.573561173
TRBV21OR9-2	-0.037	0.281769299	0.573447749
NDFIP2	-0.037	0.28175736	0.573447749
PALMD	-0.037	0.281657422	0.573293646
RFPL2	-0.037	0.281558945	0.573117696
RP11-71H17	-0.037	0.281530951	0.573085207
TCTE3	-0.037	0.281471781	0.573001202
SNX24	-0.037	0.281379236	0.572874315
RP11-182J1	-0.037	0.281338464	0.572815792
LINC00950	-0.037	0.281320643	0.572810954
SPIN4	-0.037	0.281301357	0.572810954
OR10J7P	-0.037	0.281243189	0.572744232
RNU6-535P	-0.037	0.281206642	0.572700779
RNU6-869P	-0.037	0.281205306	0.572700779
RP5-905H7	-0.037	0.281142609	0.572650482
SNX6P1	-0.037	0.281059587	0.572557341
RP2	-0.037	0.281016092	0.57252656
NDUFA5P5	-0.037	0.281009634	0.57252656
SNORD115-48	-0.037	0.281001079	0.57252656
RAD23BLP	-0.037	0.280973934	0.572514142
RNU6-661P	-0.037	0.280865468	0.572342103
AC093523	-0.037	0.280843877	0.572332257
DEFB128	-0.037	0.280839976	0.572332257
POU6F2-AS2	-0.037	0.280835883	0.572332257
RP11-349F21	-0.037	0.280835188	0.572332257
RP11-316M1	-0.037	0.280785192	0.572332257
ZFRP1	-0.037	0.280778819	0.572332257
SLCO4A1	-0.037	0.280778561	0.572332257
RP11-55L3	-0.037	0.280645186	0.572260489
LINC01366	-0.037	0.280629229	0.572252454
ABAT	-0.037	0.280624263	0.572252454
ISG15	-0.037	0.280530932	0.572100998

RNA5SP37	-0.037	0.280421249	0.571926295
NSRP1P1	-0.037	0.280402311	0.571914365
OR7E53P	-0.037	0.280392207	0.571914365
TRMT2B	-0.037	0.280338001	0.571914365
UBE2G1	-0.037	0.280337758	0.571914365
RP11-316I3	-0.037	0.280327507	0.571914365
MIR4462	-0.037	0.280247093	0.571840464
RNF166	-0.037	0.280242787	0.571840464
RN7SL402P	-0.037	0.280242214	0.571840464
RP11-815I9	-0.037	0.280191658	0.571800844
RP11-1149O23	-0.037	0.280110595	0.57165991
SLC6A6	-0.037	0.280078467	0.571618835
EIF4EBP2P2	-0.037	0.280066338	0.571618576
AC005041	-0.037	0.280055755	0.571618576
MTND6P9	-0.037	0.280051533	0.571618576
LINC00613	-0.037	0.280041731	0.571618576
AC083906	-0.037	0.28002424	0.571618576
RP11-567O16	-0.037	0.279958641	0.571542536
TRAP1	-0.037	0.279932945	0.571517764
ASMT	-0.037	0.279928498	0.571517764
LL09NC01-139C3	-0.037	0.279912746	0.571517764
MTND1P21	-0.037	0.279900908	0.571517764
OR5H7P	-0.037	0.279866298	0.571517764
RP11-95G6	-0.037	0.279828602	0.571500721
RP1-172I22	-0.037	0.279815187	0.571497831
URGCP	-0.037	0.279711598	0.57131076
SUV39H1	-0.037	0.279698224	0.571307943
MIR135B	-0.037	0.279633561	0.57120036
HNRNPA1P24	-0.037	0.279544823	0.571068084
LCN12	-0.037	0.279533148	0.571068084
RPL3P12	-0.037	0.279476599	0.570977692
KIF9-AS1	-0.037	0.279391707	0.570828742
BX255923	-0.037	0.279386523	0.570828742
RNU1-85P	-0.037	0.279357924	0.570828742
WDR72	-0.037	0.279305479	0.57079246
ADCY4	-0.037	0.279295739	0.57079246
UBE2T	-0.037	0.279294524	0.57079246
TMEM52B	-0.037	0.279281164	0.57079246
ADAMTS15	-0.037	0.279275819	0.57079246
RP13-516M14	-0.037	0.279260516	0.57079246
RP11-174J11	-0.037	0.27925743	0.57079246
CPEB3_ribozyme	-0.037	0.279204426	0.570788909
ADAM2	-0.037	0.279189017	0.570781906
METTL8	-0.037	0.279072792	0.570642274
TRGV11	-0.037	0.27907231	0.570642274
AFF4	-0.037	0.279032975	0.570609854
LEPR	-0.037	0.279026906	0.570609854
RP11-632K21	-0.037	0.279009337	0.570609854
RP11-263C24	-0.037	0.278876786	0.570531473
RNU6-957P	-0.037	0.278873524	0.570531473
CSRNP2	-0.037	0.27883395	0.57052131
ICE2P1	-0.037	0.278801723	0.570479879

C21orf59	-0.037	0.27870318	0.570327246
ABC7-42418200C9	-0.037	0.278701054	0.570327246
MIR6865	-0.037	0.278681618	0.570327246
TROVE2	-0.037	0.278662429	0.570317369
AC009941	-0.037	0.278622586	0.570260331
RP11-417O18	-0.037	0.278621519	0.570260331
TTC4P1	-0.037	0.278594511	0.570260331
DPH7	-0.037	0.27853729	0.570183766
OR5J7P	-0.037	0.278464119	0.570131999
ZNF341-AS1	-0.037	0.278410009	0.570051013
RP4-705D16	-0.037	0.278299677	0.569893314
MIR4423	-0.037	0.278292528	0.569893314
AC099684	-0.037	0.278210972	0.569785163
THAP2	-0.037	0.278166706	0.569743505
RP11-647F2	-0.037	0.278144195	0.569721898
ARHGAP42P3	-0.037	0.278098277	0.569676846
RNA5SP172	-0.037	0.278074666	0.56965298
RGS9BP	-0.037	0.277928349	0.569524718
ZNF33BP1	-0.037	0.277915075	0.56952202
IARS2	-0.037	0.277885296	0.5694855
RP11-542C10	-0.037	0.277636805	0.5690742
KRTAP10-6	-0.037	0.277589299	0.569025804
RP11-244H3	-0.037	0.277547473	0.568964555
DENND1B	-0.037	0.277502241	0.568896318
CDY5P	-0.037	0.277465953	0.568870898
TRAM1L1	-0.037	0.277415904	0.568817258
SEMA3G	-0.037	0.277400428	0.568810014
CAT	-0.037	0.277365368	0.56876261
CDY22P	-0.037	0.277286433	0.568686502
DCAF8L2	-0.037	0.277250365	0.568673692
HMGB1P22	-0.037	0.27717334	0.568540189
MIR6781	-0.037	0.277063526	0.568363895
OR5H14	-0.037	0.276940976	0.568262377
KB-1090H4	-0.037	0.276848758	0.568143562
NRIP1	-0.037	0.276820505	0.568143264
SVEP1	-0.037	0.27681302	0.568143264
ATP7B	-0.037	0.276783805	0.568132666
UBOX5-AS1	-0.037	0.276733948	0.568079295
RNU1-100P	-0.037	0.276713852	0.568062527
OR5M5P	-0.037	0.276665533	0.567987816
HTR2C	-0.037	0.276617793	0.567914287
MPI	-0.037	0.276576701	0.567903364
ZNF669	-0.037	0.276572357	0.567903364
MIR510	-0.037	0.276570181	0.567903364
RP11-822P1	-0.037	0.276555543	0.567903364
ALKBH1	-0.037	0.276506001	0.567903364
AP001342	-0.037	0.276491505	0.567899814
PLEKHM1P	-0.037	0.27647536	0.567891145
RP11-73E17	-0.037	0.276449546	0.567869495
RP11-597A11	-0.037	0.276434849	0.567869495
AC104306	-0.037	0.2764118	0.567858786
HSPE1P16	-0.037	0.276394827	0.567858786

NECAP1P1	-0.037	0.276392104	0.567858786
TET3	-0.037	0.276360779	0.567851711
ALOX5	-0.037	0.276313267	0.567778582
RP5-951N9	-0.037	0.276186227	0.567566509
RP11-458I7	-0.037	0.276179462	0.567566509
SMARCE1P3	-0.037	0.276148674	0.567538315
RP11-560L11	-0.037	0.276095959	0.567454462
RP11-321L2	-0.037	0.276087601	0.567454462
THAP11	-0.037	0.276085434	0.567454462
GPR158	-0.037	0.276051163	0.567454462
SMCR5	-0.037	0.275999077	0.567426748
RP11-39K24	-0.037	0.275951246	0.567426388
TMEM181	-0.037	0.275914823	0.567375989
RP1-122K4	-0.037	0.275908428	0.567375989
MIR3136	-0.037	0.275887586	0.567368974
RNU6-1227P	-0.037	0.275850195	0.567341073
MIR5708	-0.037	0.275786207	0.567282962
RP11-256I23	-0.037	0.275774972	0.567282962
TUBAP2	-0.037	0.275736096	0.567228882
MRPS18B	-0.037	0.275712497	0.567204833
RN7SKP183	-0.037	0.275690789	0.567184675
RP11-61K20	-0.037	0.275690789	0.567184675
RNU6-533P	-0.037	0.275690789	0.567184675
OR4R1P	-0.037	0.275690789	0.567184675
RNA5SP142	-0.037	0.275690789	0.567184675
RNA5SP126	-0.037	0.275690789	0.567184675
RNU6-832P	-0.037	0.275690789	0.567184675
RNU6-655P	-0.037	0.275690789	0.567184675
RNU6-812P	-0.037	0.275690789	0.567184675
SNORD115-44	-0.037	0.275690789	0.567184675
RNU2-66P	-0.037	0.275690789	0.567184675
AC012119	-0.037	0.275690789	0.567184675
RNU6-1012P	-0.037	0.275690789	0.567184675
RNU6-1130P	-0.037	0.275690789	0.567184675
SNORD115-2	-0.037	0.275690789	0.567184675
NUDT7	-0.037	0.275641947	0.567184675
RP11-354I10	-0.037	0.275612674	0.567184675
NPDC1	-0.037	0.275599575	0.567184675
AKIP1	-0.037	0.275585132	0.567184675
FBXO4	-0.037	0.275513401	0.567184675
RASGEF1A	-0.037	0.275431816	0.567184675
RPS7P1	-0.037	0.275380564	0.567184675
RP5-916L7	-0.037	0.275372213	0.567184675
AC016696	-0.037	0.275350579	0.567184675
RN7SL750P	-0.037	0.275307273	0.567155133
BBS12	-0.037	0.275250898	0.567112585
PROB1	-0.037	0.275214579	0.567086821
OR51Q1	-0.037	0.275101299	0.566945009
SEC62	-0.037	0.275060443	0.566916381
ACBD4	-0.037	0.275024623	0.566890409
USP9YP14	-0.037	0.274936501	0.566852998
COX6CP12	-0.037	0.274893366	0.566792776

MIR4291	-0.037	0.274840178	0.566756718
AL117341	-0.037	0.274824974	0.566756718
RP11-116O18	-0.037	0.274784603	0.566715728
CTD-2666L21	-0.037	0.274782377	0.566715728
ITPR2	-0.037	0.274727124	0.566656958
RP11-334J6	-0.037	0.274657611	0.566601032
MTND6P15	-0.037	0.274653908	0.566601032
RP11-1035H13	-0.037	0.274647357	0.566601032
AC068715	-0.037	0.274508538	0.5663671
RP4-604A21	-0.037	0.274484654	0.566342356
C3orf18	-0.037	0.274430084	0.566278827
RP11-983C2	-0.037	0.274355856	0.566199252
ST6GALNAC5	-0.037	0.274338399	0.56618776
AKR1C4	-0.037	0.274312268	0.566158362
KIAA1671	-0.037	0.274193474	0.565962233
RNU6-1174P	-0.037	0.274161153	0.565920047
C1orf141	-0.037	0.274112429	0.565868522
HDAC9	-0.037	0.274093928	0.565868522
AL359815	-0.037	0.274050006	0.565837761
OCM	-0.037	0.273989158	0.565810243
EIF2S1	-0.037	0.273971628	0.565798575
AF165138	-0.037	0.273889015	0.565701553
DDX18	-0.037	0.273884123	0.565701553
AF146191	-0.037	0.273881201	0.565701553
ZNF74	-0.037	0.273875493	0.565701553
RP11-83J21	-0.037	0.273822532	0.565701553
GEMIN8P2	-0.037	0.273819368	0.565701553
SPATA19	-0.037	0.273788752	0.565690725
AC092841	-0.037	0.273786041	0.565690725
ABCB11	-0.037	0.273729413	0.565641737
AC024704	-0.037	0.27369853	0.565602459
ZKSCAN5	-0.037	0.273642563	0.56551134
RPL23AP6	-0.037	0.273626357	0.56551134
MACF1	-0.037	0.273623877	0.56551134
PLCE1-AS1	-0.037	0.273583115	0.56551134
FAM65A	-0.037	0.273499804	0.565437131
FCF1P3	-0.037	0.27349919	0.565437131
PGM5-AS1	-0.037	0.273496145	0.565437131
RXRB	-0.037	0.273446642	0.565400852
MARK2	-0.037	0.273429163	0.565389256
RP11-357N13	-0.037	0.273351875	0.565278528
RP11-384E22	-0.037	0.273333082	0.565264207
MIR7856	-0.037	0.273272283	0.565187556
TPH2	-0.037	0.273261511	0.565187556
COX11P1	-0.037	0.27325907	0.565187556
AC013737	-0.037	0.273239057	0.565187556
RNU6-254P	-0.037	0.273234115	0.565187556
RP11-423J7	-0.037	0.273224082	0.565187556
AL591806	-0.037	0.27315188	0.565134864
RP11-1J4	-0.037	0.273081413	0.565013618
DCLK1	-0.037	0.273065135	0.565004484
AC083900	-0.037	0.273037898	0.564972671

AP2A2	-0.037	0.273030928	0.564972671
GRK6P1	-0.037	0.272880884	0.564721381
GART	-0.037	0.272838175	0.564666422
RP11-566J3	-0.037	0.27279669	0.564646531
RPL21P3	-0.037	0.272796397	0.564646531
XYLB	-0.037	0.27276765	0.564646531
RP11-10E6	-0.037	0.272763747	0.564646531
AC027320	-0.037	0.272749653	0.564646531
CX3CR1	-0.037	0.272746136	0.564646531
AL157884	-0.037	0.27270825	0.564633996
GAK	-0.037	0.272695225	0.564633996
PMFBP1	-0.037	0.272662722	0.564613368
PSMG2	-0.037	0.272614689	0.564538449
RN7SL811P	-0.037	0.272614328	0.564538449
AC092944	-0.037	0.272564157	0.564482893
DPPA4	-0.037	0.272550335	0.564478813
RP11-375O18	-0.037	0.272490752	0.564429045
RP11-121J20	-0.037	0.272473588	0.564429045
CARD17	-0.037	0.272343938	0.564223096
RPS29P2	-0.037	0.272236001	0.56404855
AC093430	-0.037	0.27214998	0.563919385
Z93241	-0.037	0.272136105	0.56391517
TICRR	-0.037	0.27211988	0.563906084
MRPL35P4	-0.037	0.272119474	0.563906084
RP11-489E7	-0.037	0.272114601	0.563906084
RP11-262A16	-0.037	0.272078106	0.563906084
DPM1	-0.037	0.272052106	0.563906084
DHX36	-0.037	0.271986014	0.563800397
FFAR3	-0.037	0.271980011	0.563800397
MIR4736	-0.037	0.271978219	0.563800397
DNAL1	-0.037	0.271972177	0.563800397
RNU7-126P	-0.037	0.271963317	0.563800397
MIR6782	-0.037	0.271958683	0.563800397
RP11-35G22	-0.037	0.271927577	0.563800397
AKR1B10P2	-0.037	0.271923412	0.563800397
PABPC1P10	-0.037	0.271916715	0.563800397
CCDC75P1	-0.037	0.27186977	0.563800397
RP1-202O8	-0.037	0.271844693	0.563800397
AC008449	-0.037	0.271830098	0.563800397
SEPT2P1	-0.037	0.271826562	0.563800397
EIF4A1P1	-0.037	0.271764561	0.563783027
RP4-543J13	-0.037	0.271730678	0.563737292
RNU4-79P	-0.037	0.271721094	0.563737292
AC011343	-0.037	0.271694461	0.563735823
MIR4257	-0.037	0.271692196	0.563735823
ARL5B	-0.037	0.271651906	0.563721202
ASNSP3	-0.037	0.271643808	0.563721202
RNU6-600P	-0.037	0.27162129	0.563721202
MYO7B	-0.037	0.271530589	0.563641361
XPO7	-0.037	0.271497704	0.563597662
RNPEP	-0.037	0.271470779	0.563566332
SNORA67	-0.037	0.271430759	0.563532377

RP11-365O16	-0.037	0.271415504	0.563532377
LLNLR-246C6	-0.037	0.271415266	0.563532377
HNRNPA3P1	-0.037	0.271385605	0.563532377
MIR548G	-0.037	0.271359511	0.563532377
RP3-445N2	-0.037	0.271320963	0.563525512
ZNF705E	-0.037	0.271307812	0.563522771
RP11-38O23	-0.037	0.271282385	0.563494534
FGF6	-0.037	0.27128082	0.563494534
PRKCE	-0.037	0.271247106	0.563470403
RP11-430B1	-0.037	0.271205911	0.563415462
ZHX1	-0.037	0.271189958	0.563415462
OR5M14P	-0.037	0.271187379	0.563415462
RNU6-1218P	-0.037	0.271171629	0.563415462
TSPAN33	-0.037	0.271157816	0.563415462
MIR4753	-0.037	0.271133838	0.563415462
RP11-398J10	-0.037	0.271097946	0.563406287
PTCH1	-0.037	0.270940744	0.563118404
RHAG	-0.037	0.270929319	0.563118404
ENO1P3	-0.037	0.270928755	0.563118404
SDPR	-0.037	0.270879595	0.563111422
AP000708	-0.038	0.270864213	0.563111422
PSMD1	-0.038	0.270810844	0.563055313
SLC5A11	-0.038	0.270797032	0.563052084
MTND4P2	-0.038	0.270732926	0.562967039
TACC3	-0.038	0.270714754	0.562953832
RNU7-137P	-0.038	0.270709678	0.562953832
ROPN1L-AS1	-0.038	0.270705965	0.562953832
ACTL9	-0.038	0.270694586	0.562953832
AC135050	-0.038	0.270621807	0.56288343
DYNLL1P1	-0.038	0.270593006	0.562872689
RP11-479F13	-0.038	0.270589732	0.562872689
LINC00945	-0.038	0.27058936	0.562872689
CFAP46	-0.038	0.270586498	0.562872689
CSRP2BP	-0.038	0.270583026	0.562872689
RP5-850O15	-0.038	0.270582807	0.562872689
KIAA0556	-0.038	0.270580174	0.562872689
GPR151	-0.038	0.270571893	0.562872689
LSM6P2	-0.038	0.270564185	0.562872689
PPIAL4B	-0.038	0.270525481	0.562872689
PHF13	-0.038	0.27045871	0.562872689
RNU11-6P	-0.038	0.27043664	0.562872689
CTD-2006O16	-0.038	0.27041472	0.562870586
SNORA37	-0.038	0.270322902	0.562728653
OTUB2	-0.038	0.270298558	0.562702571
PCSK6	-0.038	0.27029642	0.562702571
AC067903	-0.038	0.270233364	0.562640632
MIR8065	-0.038	0.270170371	0.562545254
NAV3	-0.038	0.270088368	0.562437091
MIR8080	-0.038	0.270046048	0.562384051
RNU6-114P	-0.038	0.269987126	0.56232461
AL022476	-0.038	0.269871646	0.562237795
PRKX-AS1	-0.038	0.269829383	0.562192747

NBPF1	-0.038	0.269696551	0.561952435
SNORA51	-0.038	0.26967892	0.561952435
SUPV3L1	-0.038	0.269614487	0.561892494
SLC25A36	-0.038	0.269540739	0.56181255
AL163193	-0.038	0.269519767	0.56181255
GMEB1	-0.038	0.269459656	0.561717295
RETNLB	-0.038	0.269455203	0.561717295
FCF1P2	-0.038	0.269450648	0.561717295
RNU6-189P	-0.038	0.269402046	0.561709004
TMX1	-0.038	0.269345646	0.561651712
RNU7-11P	-0.038	0.269251436	0.561479843
RP11-532N4	-0.038	0.269218801	0.56143637
MID2	-0.038	0.269178839	0.561377615
CCNB1IP1P2	-0.038	0.269143036	0.561327525
CNTNAP5	-0.038	0.269125508	0.56131555
POC1B-GALNT4	-0.038	0.269117489	0.56131555
SF3B3	-0.038	0.269019207	0.561167562
MIR758	-0.038	0.269018518	0.561167562
RNU7-26P	-0.038	0.268912042	0.561017733
SLMAP	-0.038	0.268885914	0.560987797
AC005686	-0.038	0.268876456	0.560987797
SNORD29	-0.038	0.268857106	0.560987797
OR4F4	-0.038	0.268775275	0.56092897
LRRC37A13P	-0.038	0.268761947	0.56092897
SNORD62	-0.038	0.268731506	0.56091136
AC005336	-0.038	0.268698876	0.560867833
AC131012	-0.038	0.268669407	0.560855484
RNU6-637P	-0.038	0.268645178	0.560829486
MIR4794	-0.038	0.268629568	0.56082148
DDX56	-0.038	0.268615916	0.560817562
RP11-9B6	-0.038	0.268594736	0.560797927
RP11-420K10	-0.038	0.268557802	0.560745394
HSPA4	-0.038	0.26844398	0.560536003
LINC00210	-0.038	0.268395056	0.56052845
RN7SL382P	-0.038	0.268337898	0.560436474
SLC1A5	-0.038	0.268329793	0.560436474
FAM221B	-0.038	0.268306999	0.560436474
LINC00563	-0.038	0.268301566	0.560436474
XIAP-AS1	-0.038	0.268287167	0.560436474
AF131217	-0.038	0.268149172	0.560211459
AC079235	-0.038	0.268139953	0.560211459
AC006486	-0.038	0.268110258	0.560179306
TEN1-CDK3	-0.038	0.268037265	0.560075937
AC022153	-0.038	0.268033405	0.560075937
RPUSD4	-0.038	0.267891319	0.559942942
BMS1P7	-0.038	0.267872816	0.55992884
TSN	-0.038	0.267853307	0.559912634
RNU6-1145P	-0.038	0.267851219	0.559912634
FAM161B	-0.038	0.267836545	0.559912634
RP11-362I1	-0.038	0.267823871	0.559912634
MIR4681	-0.038	0.267805246	0.559912634
RP5-1077H22	-0.038	0.26774628	0.559836336

HNRNPA1P35	-0.038	0.267683786	0.55973024
PCDHGB8P	-0.038	0.267630497	0.559692531
RN7SKP81	-0.038	0.267620804	0.559692531
RN7SKP182	-0.038	0.267569248	0.559638161
TSGA13	-0.038	0.267457553	0.559502828
CTD-2345M20	-0.038	0.26744744	0.559502828
MIR548T	-0.038	0.26738247	0.559419474
OR11M1P	-0.038	0.267332126	0.559338715
RN7SL869P	-0.038	0.267328465	0.559338715
KCNU1	-0.038	0.26732554	0.559338715
AC024560	-0.038	0.267273898	0.559338715
MTND6P7	-0.038	0.267247503	0.559322409
RP11-568G20	-0.038	0.267207138	0.559282589
AL121748	-0.038	0.267156021	0.559250059
RPS8P6	-0.038	0.267142629	0.559250059
PIEZO1P2	-0.038	0.267136224	0.559250059
RP11-452I5	-0.038	0.267134601	0.559250059
MCTS2P	-0.038	0.267126815	0.559250059
TTPAL	-0.038	0.267126588	0.559250059
PLK5	-0.038	0.266930156	0.55901339
EEF1A1P14	-0.038	0.266903482	0.558982109
AC019046	-0.038	0.266822214	0.558836482
RPL32P28	-0.038	0.266804834	0.558824655
RNU6-130P	-0.038	0.266789679	0.558817488
ZNF124	-0.038	0.266680756	0.558613906
BAG4	-0.038	0.266559789	0.558385076
FTH1P24	-0.038	0.266548684	0.558385076
FEM1AP4	-0.038	0.266535881	0.558384112
HEMK1	-0.038	0.266500261	0.558383174
LRRC58	-0.038	0.266404753	0.558235587
MESTP3	-0.038	0.266378355	0.558225984
DDX39BP1	-0.038	0.266203703	0.557982738
ACAD11	-0.038	0.266070893	0.557827109
RNA5SP21	-0.038	0.266028194	0.557762141
KRT18P49	-0.038	0.265828218	0.557474724
SNORD114-22	-0.038	0.265809991	0.557474724
MRPS16	-0.038	0.265747653	0.55740398
HSPA8P8	-0.038	0.265721048	0.557403042
DDX3Y	-0.038	0.265690919	0.557398529
AQP2	-0.038	0.2656819	0.557398529
ZBED9	-0.038	0.265583015	0.557245793
GAL3ST2	-0.038	0.265521188	0.557162475
HMGN2P41	-0.038	0.265487521	0.557119059
RP11-613G13	-0.038	0.265478568	0.557119059
GOLGA2P4	-0.038	0.265476881	0.557119059
RP11-487E13	-0.038	0.265305224	0.556858598
DNASE1L2	-0.038	0.265245007	0.556757335
CTC-336P14	-0.038	0.265221487	0.556732503
EME2	-0.038	0.265196935	0.556732503
TRIM53CP	-0.038	0.265135273	0.556674203
RP11-719N22	-0.038	0.265098551	0.556621641
ACCS	-0.038	0.265041272	0.556525908

UBE2NP1	-0.038	0.265028812	0.556524282
STKLD1	-0.038	0.26501385	0.556524282
VTI1B	-0.038	0.264968385	0.556470997
FBXL2	-0.038	0.26495784	0.556470997
RNU6-790P	-0.038	0.2649467	0.556470997
RP11-365N19	-0.038	0.264680761	0.555965008
NPM1P13	-0.038	0.264636619	0.555896805
ERO1LB	-0.038	0.264620779	0.55588805
SCG3	-0.038	0.264608571	0.555886923
ISCA1P1	-0.038	0.26460789	0.555886923
RP5-1173A5	-0.038	0.264593959	0.555886923
RP11-140L24	-0.038	0.264585318	0.555886923
RNU2-58P	-0.038	0.26453608	0.555886923
LL09NC01-251B2	-0.038	0.264527702	0.555886923
RP11-6E9	-0.038	0.264526239	0.555886923
HNRNPCP9	-0.038	0.264465479	0.555886923
KPNA3	-0.038	0.264460668	0.555886923
ZNF350	-0.038	0.264421353	0.55588058
THRA	-0.038	0.264416205	0.55588058
OR10G4	-0.038	0.264410846	0.55588058
RP11-160K17	-0.038	0.264402965	0.55588058
MPPED1	-0.038	0.264344921	0.555847926
DEK	-0.038	0.264295945	0.55579402
ABHD15-AS1	-0.038	0.264285317	0.55579402
MIA3	-0.038	0.264244249	0.555758925
RP11-434D9	-0.038	0.264198909	0.555704709
WDR49	-0.038	0.264126232	0.555608854
CYP3A52P	-0.038	0.264094865	0.555576043
AC233299	-0.038	0.264072987	0.555576043
C17orf53	-0.038	0.264017667	0.555552241
RP5-1063M23	-0.038	0.263993981	0.555526945
RNU11-4P	-0.038	0.263943994	0.555455537
PIGM	-0.038	0.263910619	0.555425145
CTC-428G20	-0.038	0.263858508	0.555340013
N4BP1	-0.038	0.263844722	0.555335538
BTBD7	-0.038	0.263732349	0.555123552
HMGB1	-0.038	0.263627483	0.554951872
RP11-765K14	-0.038	0.263587117	0.554891425
SSR1P1	-0.038	0.263573482	0.554887249
RSPH9	-0.038	0.263567238	0.554887249
NBPF21P	-0.038	0.263499961	0.554781514
CYYR1	-0.038	0.263488794	0.554781514
KRTAP23-1	-0.038	0.263476828	0.554781514
AC074117	-0.038	0.263451913	0.554753923
RPL35AP15	-0.038	0.26341713	0.554705207
CBX3P4	-0.038	0.26340962	0.554705207
AC009161	-0.038	0.263316482	0.554591345
MIR199A1	-0.038	0.263313974	0.554591345
RP5-1049N15	-0.038	0.263299024	0.554591345
VBP1	-0.038	0.263238396	0.554524971
RP11-110L15	-0.038	0.263231542	0.554524971
SUCLG2P2	-0.038	0.263186764	0.554489783

LINC01506	-0.038	0.263148441	0.554433569
RP5-964H19	-0.038	0.263041196	0.55427988
SNRPGP7	-0.038	0.262828784	0.553980637
DDX11L5	-0.038	0.262778331	0.553898808
SPAG4	-0.038	0.262706872	0.553772691
RP11-307F22	-0.038	0.262695758	0.553772691
XXbac-BPG249D20	-0.038	0.26266875	0.553741348
RPL3P11	-0.038	0.262646216	0.553718355
RN7SL218P	-0.038	0.262645038	0.553718355
RP11-1252I4	-0.038	0.262642002	0.553718355
GCSHP4	-0.038	0.262538151	0.553609858
RPL23P10	-0.038	0.262511166	0.553580664
MIR548AP	-0.038	0.262508905	0.553580664
KCTD9P4	-0.038	0.262415009	0.553500427
C1QTNF9B	-0.038	0.262392046	0.553476503
TMEM143	-0.038	0.262376907	0.553469083
ENO1-AS1	-0.038	0.26229581	0.553396055
MIR4429	-0.038	0.262249586	0.553342108
PAX7	-0.038	0.262212507	0.553293832
RP11-814H16	-0.038	0.262202228	0.553293832
RP3-359N14	-0.038	0.262197298	0.553293832
RP1-66C13	-0.038	0.262191199	0.553293832
NPIPB15	-0.038	0.262074557	0.553149786
RP11-529H22	-0.038	0.262073249	0.553149786
COPG2	-0.038	0.262067229	0.553149786
NEURL4	-0.038	0.261983513	0.553031149
RPL36P20	-0.038	0.261975956	0.553031149
AC005387	-0.038	0.261953496	0.553016806
GLYATL1P2	-0.038	0.261930636	0.553000138
KCNN3	-0.038	0.26179336	0.552776748
RPS2P39	-0.038	0.261790421	0.552776748
HSPA8P1	-0.038	0.261730825	0.552718218
LINC01364	-0.038	0.261700368	0.552711835
SIGLEC29P	-0.038	0.261696765	0.552711835
ZNF663P	-0.038	0.261670533	0.552711835
ADAM5	-0.038	0.261627084	0.552646167
CXorf56	-0.038	0.261493678	0.552521112
LINC00654	-0.038	0.261424715	0.552414699
LINC00297	-0.038	0.26142378	0.552414699
GLYCTK	-0.038	0.261419607	0.552414699
RP11-121C1	-0.038	0.261379889	0.552414699
RP11-728G15	-0.038	0.261346314	0.552371567
RN7SKP141	-0.038	0.261288765	0.552298953
SPDEF	-0.038	0.261285434	0.552298953
RP11-444A22	-0.038	0.261236278	0.552298953
UBE2F	-0.038	0.261221382	0.552298953
RP11-618I10	-0.038	0.261155483	0.552188776
RPL15P1	-0.038	0.261083192	0.552060433
MIR139	-0.038	0.261056738	0.552060433
RP11-42A4	-0.038	0.260946136	0.551917648
CTD-2200P10	-0.038	0.260889949	0.551872334
RPS29P25	-0.038	0.260853367	0.551819459

HOXB3	-0.038	0.260836139	0.551819459
AC090514	-0.038	0.260818194	0.551818582
RNA5SP402	-0.038	0.260813807	0.551818582
RP11-434O22	-0.038	0.260799023	0.551818582
ZDHHC19	-0.038	0.260743432	0.551782967
RP11-132A1	-0.038	0.260701246	0.551759888
RP5-1108M17	-0.038	0.260650912	0.551709746
LINC00534	-0.038	0.260552184	0.551549796
RP11-379H8	-0.038	0.260551026	0.551549796
UBFD1	-0.038	0.260520074	0.55153085
RP4-564M11	-0.038	0.26050098	0.551514944
OR10G5P	-0.038	0.260457501	0.551447405
CTD-3193K9	-0.038	0.26040929	0.551369842
RNA5SP300	-0.038	0.260374971	0.5513462
LINC00908	-0.038	0.26036444	0.5513462
PANK4	-0.038	0.260275097	0.551257261
RP11-204M4	-0.038	0.260241873	0.551211407
RP11-288E14	-0.038	0.260239797	0.551211407
RP11-105C20	-0.038	0.260152294	0.551095196
CTC-348L5	-0.038	0.260141265	0.551095196
RN7SKP210	-0.038	0.260135622	0.551095196
RP11-603B24	-0.038	0.260095703	0.551073359
DAGLA	-0.038	0.26005318	0.551056808
MIR575	-0.038	0.260015542	0.551024889
MS4A12	-0.038	0.259931845	0.550873225
MRPS18BP1	-0.038	0.259921166	0.550873225
RP11-662B19	-0.038	0.259893603	0.550841202
RNU6-239P	-0.038	0.2598919	0.550841202
KRT18P24	-0.038	0.25979531	0.55074367
ERLIN2	-0.038	0.259780535	0.55074367
CTD-2547G23	-0.038	0.259776374	0.55074367
PRRG4	-0.038	0.259774062	0.55074367
DEFB115	-0.038	0.259754144	0.55074367
RN7SL265P	-0.038	0.259742655	0.55074367
DLAT	-0.038	0.259736384	0.55074367
RGS9	-0.038	0.259725752	0.55074367
ZNF264	-0.038	0.259652489	0.550723765
OR9G4	-0.038	0.259592836	0.55066934
FAM76A	-0.038	0.259573927	0.550664204
RP1-177I10	-0.038	0.2595616	0.550664204
FBF1	-0.038	0.259524488	0.550646981
RP11-161I10	-0.038	0.25948222	0.550606358
AC013472	-0.038	0.259473505	0.550606358
POLM	-0.038	0.259415236	0.550544667
CTD-2355J17	-0.038	0.259401812	0.550544667
STAU1	-0.038	0.259371821	0.550544667
MIR638	-0.038	0.259355921	0.550544667
RAI1-AS1	-0.038	0.259353766	0.550544667
GRIN2C	-0.038	0.259329797	0.550544667
DUSP8P3	-0.038	0.25929181	0.550496637
LRTOMT	-0.038	0.259229142	0.550405275
KRTAP5-5	-0.038	0.259216729	0.550405275

MIR92A2	-0.038	0.259203394	0.550405275
FAM166B	-0.038	0.259178363	0.550378451
RP5-1002M8	-0.038	0.259148329	0.55036375
MIR643	-0.038	0.259133245	0.550356258
OR5S1P	-0.038	0.259058826	0.550247278
RN7SL149P	-0.038	0.258944217	0.550126515
SNORA44	-0.038	0.25889318	0.550042624
AC132068	-0.038	0.258854994	0.549986027
VILL	-0.038	0.258830713	0.549958972
RP11-134N8	-0.038	0.258786134	0.549888784
LL0XNC01-39B3	-0.038	0.258784791	0.549888784
ORC6	-0.038	0.258737108	0.549833671
AKNAD1	-0.038	0.258723463	0.549829207
CTB-175E5	-0.038	0.258675886	0.549777162
KRTAP4-11	-0.038	0.258662274	0.549772764
IKZF2	-0.038	0.258658046	0.549772764
RP11-124N3	-0.038	0.258651625	0.549772764
MTRNR2L8	-0.038	0.25859444	0.549702181
RN7SKP199	-0.038	0.258570753	0.549677541
SMG1P6	-0.038	0.258570474	0.549677541
RN7SL724P	-0.038	0.258547961	0.549677541
LINC01489	-0.038	0.258490162	0.549627703
AC090133	-0.038	0.258476549	0.549623296
RP11-285J16	-0.038	0.258445664	0.549582159
ARHGEF26	-0.038	0.258411978	0.549559601
MIR4258	-0.038	0.258393597	0.549559601
CDY7P	-0.038	0.258387893	0.549559601
RNU4-86P	-0.038	0.258386296	0.549559601
PRR11	-0.038	0.258380194	0.549559601
RP11-164D18	-0.038	0.258289917	0.549496309
SLC25A15P1	-0.039	0.258258881	0.549454825
MIR150	-0.039	0.258250434	0.549454825
RP11-475D10	-0.039	0.258232572	0.549447942
AL157871	-0.039	0.258173075	0.549345889
CTD-3076O17	-0.039	0.258157494	0.549337279
SEC22B	-0.039	0.258145126	0.549335505
RP11-162K6	-0.039	0.25813113	0.549335505
HMGN1P33	-0.039	0.258114675	0.549335505
RP4-607J2	-0.039	0.258107513	0.549335505
SSX9	-0.039	0.25801879	0.549238442
GPR126	-0.039	0.257968865	0.549156713
HSFY1	-0.039	0.257968685	0.549156713
PRMT6	-0.039	0.257952302	0.549156713
RP13-635I23	-0.039	0.257901358	0.549086634
FAM153B	-0.039	0.25789744	0.549086634
SHISA3	-0.039	0.257885643	0.549086634
GLO1	-0.039	0.257847878	0.549086634
LINC00614	-0.039	0.257823233	0.549067569
PDIA3P1	-0.039	0.257755438	0.548972286
PES1P1	-0.039	0.257714669	0.548910001
RP11-1E11	-0.039	0.25769749	0.548897959
CTD-3126B10	-0.039	0.25764755	0.548816131

RN7SKP13	-0.039	0.257598281	0.548760268
RNU6-804P	-0.039	0.257598281	0.548760268
MIR6130	-0.039	0.257598281	0.548760268
CAPN12	-0.039	0.257549717	0.548760268
IDS	-0.039	0.257536849	0.548760268
RNU6-427P	-0.039	0.257493085	0.548720805
RP11-148B18	-0.039	0.257468815	0.548715192
RP11-567G24	-0.039	0.257403665	0.548615608
FNTA	-0.039	0.257366388	0.548585255
OR5BJ1P	-0.039	0.257333948	0.548540659
AC107982	-0.039	0.25726574	0.548446104
RNU7-60P	-0.039	0.257260724	0.548446104
RPL27AP5	-0.039	0.257256007	0.548446104
RP11-265F19	-0.039	0.25700944	0.548094238
RP11-293M10	-0.039	0.256974073	0.548067895
KRTAP6-3	-0.039	0.256953787	0.548049172
B3GALNT1P1	-0.039	0.256890404	0.547938523
RP11-529A4	-0.039	0.256845602	0.547892467
RP11-365H8	-0.039	0.256837933	0.547892467
OSBP2	-0.039	0.256826115	0.547892467
FAM60CP	-0.039	0.25680609	0.547892467
RNU6-1110P	-0.039	0.25680244	0.547892467
UBTFL6	-0.039	0.25680029	0.547892467
SNORD7	-0.039	0.256707661	0.547867708
RN7SL353P	-0.039	0.256696782	0.547867708
CTD-2555O16	-0.039	0.256660107	0.547815313
LINC00466	-0.039	0.256566991	0.547665654
MIR4514	-0.039	0.256516149	0.54763076
RP11-446E9	-0.039	0.256515712	0.54763076
RP5-1033H2	-0.039	0.256461494	0.547587714
MIR2114	-0.039	0.256446296	0.547579814
AC116154	-0.039	0.256377108	0.547456624
RNU6-1085P	-0.039	0.256374162	0.547456624
RNU6-1177P	-0.039	0.256339764	0.547425968
HMGN2P23	-0.039	0.256251104	0.54728571
AC108456	-0.039	0.256201403	0.5472041
RNU6-597P	-0.039	0.256133492	0.547157202
FLCN	-0.039	0.256092479	0.547094129
MIR1302-4	-0.039	0.256083078	0.547094129
ARIH2P1	-0.039	0.256069898	0.547094129
UBE2Q1	-0.039	0.256008257	0.547012349
SRPK2P	-0.039	0.255962836	0.546974153
RN7SL658P	-0.039	0.255952443	0.546974153
SARAF	-0.039	0.255945561	0.546974153
RBBP4P5	-0.039	0.255939384	0.546974153
HNRNPUP1	-0.039	0.255929827	0.546974153
RP5-837M10	-0.039	0.255905294	0.546974153
COL4A2-AS1	-0.039	0.255757807	0.54674703
GNB1	-0.039	0.255754562	0.54674703
GINS4	-0.039	0.255742442	0.54674703
RNA5SP260	-0.039	0.25572393	0.54674703
PSMD6	-0.039	0.255707914	0.546738533

HIST2H4B	-0.039	0.255690793	0.54672647
RP11-440J4	-0.039	0.255677409	0.546722397
TPTE2P2	-0.039	0.255652949	0.546694639
MSMB	-0.039	0.255568048	0.546562165
RP5-914M6	-0.039	0.255500479	0.546521915
RP11-661P17	-0.039	0.255479033	0.546519039
AC055764	-0.039	0.255466131	0.546515988
PSMC1	-0.039	0.255414213	0.546429463
HIST1H2AC	-0.039	0.255237648	0.546174392
VN1R4	-0.039	0.255235092	0.546174392
USP27X	-0.039	0.255197458	0.546162009
RP11-341A11	-0.039	0.255176039	0.546140709
LALBA	-0.039	0.255146304	0.54610161
AC007528	-0.039	0.2550177	0.545860562
KRT18P67	-0.039	0.25501678	0.545860562
MTFR2	-0.039	0.254896407	0.545738421
AL135784	-0.039	0.254857704	0.545729156
RP4-639J15	-0.039	0.254760269	0.545569574
SUPT4H1P	-0.039	0.254668138	0.545421324
RP11-100N20	-0.039	0.254610782	0.545323007
FYTTD1P1	-0.039	0.254552578	0.545222865
RP11-142G1	-0.039	0.254548638	0.545222865
RP11-318K15	-0.039	0.254508072	0.545205496
GAGE13	-0.039	0.254506324	0.545205496
AK3P2	-0.039	0.254486262	0.545205496
RP11-958N24	-0.039	0.254473663	0.545205496
RLN2	-0.039	0.254395899	0.545092274
Z95704	-0.039	0.254378516	0.545092274
DYNLL1-AS1	-0.039	0.254368851	0.545092274
RN7SL159P	-0.039	0.254342333	0.545069334
AC006482	-0.039	0.254319836	0.545069334
RP11-485O10	-0.039	0.254292128	0.545069334
CSTL1	-0.039	0.254166064	0.544885088
CASQ2	-0.039	0.254068976	0.544799563
HNRNPA3P5	-0.039	0.254065623	0.544799563
LINC00502	-0.039	0.254049813	0.544799563
AP000868	-0.039	0.254048237	0.544799563
PQLC1P1	-0.039	0.254046556	0.544799563
RP11-115P21	-0.039	0.253935344	0.544709211
KLHL20	-0.039	0.253886644	0.544629277
NDNL2	-0.039	0.253780635	0.54447544
SNX1	-0.039	0.253774039	0.54447544
RP11-215D10	-0.039	0.253764071	0.54447544
RP11-112L7	-0.039	0.253718849	0.544433635
RLBP1	-0.039	0.253660983	0.544341338
CTD-2555K7	-0.039	0.25362687	0.544317184
SALRNA2	-0.039	0.253568807	0.544217095
YY1AP1	-0.039	0.253526429	0.544175188
RP11-382A18	-0.039	0.253506363	0.544156642
AC006458	-0.039	0.253469231	0.544101459
RP1-97G4	-0.039	0.253433611	0.544049517
RP11-517H2	-0.039	0.253409054	0.544021322

NKRF	-0.039	0.253313969	0.543890741
SLC39A8	-0.039	0.25325741	0.543867376
RP4-604G5	-0.039	0.253217866	0.543806975
MIR6873	-0.039	0.253174736	0.543738869
DNAJC13	-0.039	0.253170884	0.543738869
EIF2AK1	-0.039	0.253138187	0.54373393
RP11-151G12	-0.039	0.253127093	0.54373393
FTLP4	-0.039	0.253100472	0.543701961
LLNLR-284B4	-0.039	0.252977575	0.54353602
RP11-700P18	-0.039	0.25296113	0.543525208
CTD-2062A1	-0.039	0.252946414	0.543518108
MLC1	-0.039	0.252838147	0.543334495
PFN1P3	-0.039	0.252826574	0.543334141
MYF6	-0.039	0.252769909	0.543236879
VSTM2B	-0.039	0.252745102	0.543208077
RFX3-AS1	-0.039	0.252664725	0.543084342
LINC00322	-0.039	0.252611465	0.543043344
CTD-2631K10	-0.039	0.252557886	0.542977224
MGAT4D	-0.039	0.252513563	0.542930949
RNY4P37	-0.039	0.252496045	0.542917794
AC010243	-0.039	0.252451871	0.542847318
RP11-39E3	-0.039	0.252397867	0.542755698
MIR4776-1	-0.039	0.252359389	0.542721964
RP11-697H9	-0.039	0.252316615	0.542654478
AC092415	-0.039	0.252202031	0.542506036
ATP8B2	-0.039	0.252184676	0.542493206
DONSON	-0.039	0.252136217	0.542413461
DDX43P1	-0.039	0.252119812	0.542402668
AC012353	-0.039	0.252099263	0.542382962
OR4A43P	-0.039	0.252063382	0.542330263
KAL1	-0.039	0.252041356	0.542330263
PHKBP2	-0.039	0.251972957	0.54223369
AC011284	-0.039	0.251970628	0.54223369
PRR13P1	-0.039	0.251923929	0.54217718
ABHD6	-0.039	0.251919364	0.54217718
AC012613	-0.039	0.251841413	0.542073077
RP11-553D4	-0.039	0.251760009	0.541946845
RNA5SP320	-0.039	0.251756504	0.541946845
RNU6-525P	-0.039	0.251737236	0.541946845
SLC35G3	-0.039	0.251663937	0.541902974
PA2G4P4	-0.039	0.251624094	0.541850194
GSTCD	-0.039	0.251601788	0.541826657
TRMT44	-0.039	0.251510683	0.541673502
RP11-622O11	-0.039	0.251502404	0.541673502
SLC7A1	-0.039	0.251423669	0.541639004
RP11-643G16	-0.039	0.251328061	0.541506517
APBB3	-0.039	0.251313632	0.541499926
PPT2-EGFL8	-0.039	0.251305048	0.541499926
LRRD1	-0.039	0.251225744	0.541384034
RN7SL359P	-0.039	0.251223342	0.541384034
RPL9P15	-0.039	0.251206899	0.541384034
bP-2171C21	-0.039	0.251188202	0.541381502

PLCZ1	-0.039	0.251166771	0.541381502
MBD5	-0.039	0.25112843	0.541358062
AC242988	-0.039	0.251110695	0.541358062
GNG2	-0.039	0.251087789	0.541356185
OR52H1	-0.039	0.250972596	0.541239628
RP11-468O2	-0.039	0.250941391	0.541239628
RP1-278C19	-0.039	0.250931323	0.541239628
RNA5SP458	-0.039	0.250836602	0.541133073
H2AFV	-0.039	0.250799593	0.541077741
TOMM20P1	-0.039	0.250781115	0.541062385
SAMM50	-0.039	0.25075741	0.541060261
MIR802	-0.039	0.250742285	0.541052139
CTD-2537O9	-0.039	0.250737735	0.541052139
MYNN	-0.039	0.25071681	0.541052139
C7orf71	-0.039	0.250712538	0.541052139
OXCT1	-0.039	0.250590981	0.540872684
SIDT1	-0.039	0.250570013	0.540851939
PAICSP7	-0.039	0.250541292	0.540814455
PIGV	-0.039	0.250473084	0.540691727
MCHR2-AS1	-0.039	0.250471163	0.540691727
RN7SL674P	-0.039	0.250457305	0.540691727
AP001340	-0.039	0.250445127	0.540691727
RP5-945F2	-0.039	0.250384842	0.54059925
CMAS	-0.039	0.250381138	0.54059925
AC080032	-0.039	0.250322765	0.540514228
RP11-346E8	-0.039	0.250314613	0.540514228
CST1	-0.039	0.250282332	0.540500438
LINC00661	-0.039	0.250265552	0.540488707
C12orf60	-0.039	0.250209252	0.540398305
RP11-25E2	-0.039	0.250182506	0.540382866
RP11-432J9	-0.039	0.250103073	0.540260299
RP11-714M23	-0.039	0.250033253	0.540133975
RP1-86C11	-0.039	0.250013354	0.540115486
RP11-299J5	-0.039	0.250013065	0.540115486
MPP3	-0.039	0.250000307	0.540115486
SLC51A	-0.039	0.249974648	0.540115486
RP11-15F12	-0.039	0.249962872	0.540115486
RPEP3	-0.039	0.249837299	0.53998008
FAM104A	-0.039	0.249821405	0.53998008
RP11-79M21	-0.039	0.249781645	0.539933299
RP11-883G10	-0.039	0.249720874	0.539847787
LINC01360	-0.039	0.249648885	0.539765264
OR4F2P	-0.039	0.249646972	0.539765264
GPR137	-0.039	0.249516689	0.539579719
UBE4A	-0.039	0.249476455	0.539518462
ICA1	-0.039	0.249474656	0.539518462
ZBTB5	-0.039	0.249456676	0.539518462
FAM180B	-0.039	0.249342972	0.539352245
UBE2NL	-0.039	0.249302385	0.539313441
MIR3157	-0.039	0.249300538	0.539313441
AC004166	-0.039	0.249291074	0.539313441
HIST1H2APS6	-0.039	0.249264275	0.539313441

FAM197Y6	-0.039	0.249264275	0.539313441
MIR6134	-0.039	0.249264275	0.539313441
OR51F1	-0.039	0.249264275	0.539313441
TTTY13	-0.039	0.249264275	0.539313441
RNA5SP417	-0.039	0.249188428	0.539311881
MOXD2P	-0.039	0.249158734	0.539272122
ZNRF2	-0.039	0.249097483	0.539196568
KRT18P31	-0.039	0.249082272	0.539196568
RNU6-440P	-0.039	0.249034856	0.539176749
RP11-24P4	-0.039	0.2490184	0.539176749
LINC00442	-0.039	0.248988345	0.539176749
RBM22P13	-0.039	0.248974834	0.539176749
LINC00596	-0.039	0.248960046	0.539176749
SEMA6A	-0.039	0.248893001	0.539137988
RP11-393I2	-0.039	0.248868765	0.539110009
AC105081	-0.039	0.248800965	0.539059654
SLC11A2	-0.039	0.248789121	0.539059654
AC012078	-0.039	0.248651569	0.538884613
DHX40	-0.039	0.248646028	0.538884613
HSBP1P1	-0.039	0.24864158	0.538884613
ST13P12	-0.039	0.248607179	0.538884613
CALR4P	-0.039	0.248561317	0.538811609
AL021546	-0.039	0.24852114	0.538749037
AC048380	-0.039	0.248519123	0.538749037
RP11-499P1	-0.039	0.248483251	0.538749037
POTEC	-0.039	0.248478269	0.538749037
RNU6-277P	-0.039	0.248292571	0.538523169
C14orf93	-0.039	0.248239042	0.538438859
RAB3GAP1	-0.039	0.248217093	0.538433024
RN7SL452P	-0.039	0.248161038	0.538335949
RP11-650K20	-0.039	0.24804521	0.53815822
NR6A1	-0.039	0.248017369	0.538122328
PVALB	-0.039	0.24798314	0.538072574
AL358340	-0.039	0.247921647	0.53801268
ADAMTS19-AS1	-0.039	0.24791423	0.53801268
RP11-412D9	-0.039	0.247907479	0.53801268
RP11-421M1	-0.039	0.247864964	0.538012244
NIP7P1	-0.039	0.247801947	0.537899973
RNU6-152P	-0.039	0.247780874	0.537878746
RP11-550P17	-0.039	0.247772166	0.537878746
OR4K13	-0.039	0.247770309	0.537878746
RN7SL644P	-0.039	0.247748163	0.537878746
TMEM126B	-0.039	0.247687577	0.537823299
RBMX2P2	-0.039	0.247653396	0.537798116
RP11-192M23	-0.039	0.24764548	0.537798116
CELF3	-0.039	0.247637427	0.537798116
MTCYBP2	-0.039	0.247620309	0.537798116
KIF3B	-0.039	0.247509609	0.537608434
RN7SL125P	-0.039	0.247499672	0.537608434
MRPS11P1	-0.039	0.247498284	0.537608434
RP11-299D14	-0.039	0.247490947	0.537608434
RNU2-35P	-0.039	0.24748649	0.537608434

CTB-118N6	-0.039	0.247463714	0.537608434
DCX	-0.039	0.247455465	0.537608434
RP11-460G11	-0.039	0.24733753	0.537528841
RP1-138B7	-0.039	0.247320329	0.537515985
ZPBP2	-0.039	0.247299901	0.537496115
LINC01517	-0.039	0.247287668	0.537494057
EIF4A1P11	-0.039	0.24727183	0.537484161
RP11-191F9	-0.039	0.247269612	0.537484161
STX18-AS1	-0.039	0.247248854	0.537483278
FAM156B	-0.039	0.247224855	0.53745564
LTN1	-0.039	0.247201728	0.537429895
RP11-573G6	-0.039	0.247090975	0.537238158
RP11-57G10	-0.039	0.247072309	0.537222099
SLC5A4P1	-0.039	0.247069218	0.537222099
KRT8P17	-0.039	0.247051152	0.537222099
RP11-815I22	-0.039	0.246967376	0.537167404
DEPDC1	-0.039	0.246936514	0.537147537
CYCSP12	-0.039	0.246871896	0.537105102
RP11-637N19	-0.039	0.246856315	0.537095738
MIR342	-0.039	0.246848644	0.537095738
ATXN10	-0.039	0.246819597	0.537075598
RPS19P6	-0.039	0.246790103	0.537074354
WFIKKN1	-0.039	0.246783012	0.537074354
ELP4	-0.039	0.246756915	0.537074354
HIST1H2BB	-0.039	0.246744526	0.537073331
FAM91A3P	-0.039	0.246717781	0.53703966
LINC00471	-0.039	0.246712977	0.53703966
RP3-507I15	-0.039	0.246704597	0.53703966
KCNT2	-0.039	0.246682682	0.53703966
ZC3HAV1L	-0.039	0.246651429	0.537017948
MIR378F	-0.039	0.246598037	0.536926246
ARHGEF10L	-0.039	0.246549765	0.536894776
RNU7-125P	-0.039	0.246545513	0.536894776
STC1	-0.039	0.246537059	0.536894776
IPO8	-0.039	0.246529926	0.536894776
PPP1R14D	-0.039	0.246482961	0.536847486
AC106874	-0.039	0.246455779	0.536812831
LZIC	-0.039	0.246430748	0.53680741
AC007182	-0.039	0.246390834	0.536745014
ZFP82	-0.039	0.246388187	0.536745014
AC009977	-0.039	0.246353216	0.536712163
CTD-2090I13	-0.039	0.246343388	0.536712163
EDEM3	-0.039	0.24632781	0.536710565
RP11-236J17	-0.039	0.246326423	0.536710565
PYY2	-0.039	0.24631989	0.536710565
NMNAT1P4	-0.039	0.246299191	0.536710565
MT-TF	-0.039	0.246220741	0.536619926
MREGP1	-0.039	0.246190698	0.536619926
AGO1	-0.039	0.246144736	0.536601608
RP11-691F15	-0.039	0.2461341	0.536601608
RPS29P13	-0.040	0.246004711	0.536370005
MIR4436B1	-0.040	0.245983934	0.536370005

STK16	-0.040	0.245978893	0.536370005
AAMP	-0.040	0.245972217	0.536370005
MSH3	-0.040	0.245919254	0.536330996
CDC42P3	-0.040	0.245882428	0.536275238
AC011513	-0.040	0.2458487	0.536226233
NPIPA8	-0.040	0.245794351	0.536200482
HINT1P2	-0.040	0.245782455	0.536200482
GPRC6A	-0.040	0.245781605	0.536200482
MEF2C-AS1	-0.040	0.245773981	0.536200482
CASP1	-0.040	0.245739283	0.536184022
RP11-794C22	-0.040	0.24573334	0.536184022
AL596329	-0.040	0.245681449	0.536106954
SLC9A6	-0.040	0.245639216	0.536039356
CTBP2P7	-0.040	0.245624763	0.536032376
CTD-2218G20	-0.040	0.245595712	0.535993539
RP11-216P6	-0.040	0.245593392	0.535993539
C3orf67	-0.040	0.245562962	0.535971182
CTC-550M4	-0.040	0.245410633	0.535712358
LINC-ROR	-0.040	0.245404655	0.535712358
TBX1	-0.040	0.245394061	0.535712358
RP11-512G4	-0.040	0.24538775	0.535712358
C7	-0.040	0.245326938	0.53570153
AC104772	-0.040	0.245312127	0.535693749
IMMT	-0.040	0.245303421	0.535693749
GPM6BP1	-0.040	0.245247338	0.535625945
LINC01374	-0.040	0.245196768	0.535540055
BLZF1	-0.040	0.245176388	0.535520101
MIR100	-0.040	0.245120042	0.535421586
RP11-15D14	-0.040	0.245010292	0.535255501
RP11-167N5	-0.040	0.244944595	0.535210176
LINC01400	-0.040	0.244799855	0.534967545
KPNA1	-0.040	0.244758745	0.534902252
TERF1P4	-0.040	0.24469821	0.534794495
GS1-114I9	-0.040	0.244682762	0.534785273
MIR6761	-0.040	0.244585807	0.534597899
PPP4R2	-0.040	0.244585799	0.534597899
SMAD3	-0.040	0.244571834	0.534597899
MIR488	-0.040	0.244530594	0.534550814
RNU6-591P	-0.040	0.244530594	0.534550814
AC010739	-0.040	0.244530594	0.534550814
AC098583	-0.040	0.244530594	0.534550814
CYP2AC1P	-0.040	0.244506182	0.534550814
SLC26A1	-0.040	0.244486282	0.534550814
RAP2C-AS1	-0.040	0.244461906	0.534547873
RP11-319C21	-0.040	0.244443557	0.534532291
DYNC2LI1	-0.040	0.244409614	0.534482607
STATH	-0.040	0.244403172	0.534482607
LHX6	-0.040	0.244341602	0.534407489
SHCBP1	-0.040	0.244331191	0.534407489
RP11-280H21	-0.040	0.244217722	0.534259219
SRMP2	-0.040	0.244113929	0.534056689
NFKBIA	-0.040	0.244100011	0.534050771

ARF1P3	-0.040	0.244098324	0.534050771
GP2	-0.040	0.244097348	0.534050771
GS1-590J6	-0.040	0.244081749	0.534050771
KLHL34	-0.040	0.244011869	0.534050771
IL18R1	-0.040	0.243998199	0.534050771
RNU7-200P	-0.040	0.243998163	0.534050771
CST2	-0.040	0.243974186	0.534050771
PALB2	-0.040	0.243927834	0.53401751
RP11-318K12	-0.040	0.243910492	0.53400409
FBXO28	-0.040	0.24383868	0.533895955
PGBD1	-0.040	0.243713505	0.533695485
OR5BE1P	-0.040	0.243700258	0.533691014
NLRP6	-0.040	0.243549559	0.533385516
RP11-718L23	-0.040	0.243414064	0.533113291
MALT1	-0.040	0.243379116	0.533069835
WWOX	-0.040	0.243368071	0.533069835
RP11-17E4	-0.040	0.243306646	0.532976066
MED7	-0.040	0.243300262	0.532976066
SIX6	-0.040	0.243295431	0.532976066
AC091697	-0.040	0.243219016	0.53290666
ZNF582	-0.040	0.243213698	0.53290666
RNU6-925P	-0.040	0.243191701	0.532895842
AHNAK	-0.040	0.243178593	0.532891637
UQCRC2P1	-0.040	0.243172397	0.532891637
USP17L1	-0.040	0.243156195	0.532891593
MIR4999	-0.040	0.243099659	0.532865874
YTHDF1P1	-0.040	0.243085367	0.532865874
KB-1589B1	-0.040	0.243023242	0.532796352
CMBL	-0.040	0.243018346	0.532796352
RP11-505C13	-0.040	0.24297224	0.532733583
CCR3	-0.040	0.242924446	0.532677836
APLP1	-0.040	0.242819355	0.532496427
SLC25A3P2	-0.040	0.242815877	0.532496427
RP4-792G4	-0.040	0.242805637	0.532496427
LINC00989	-0.040	0.242785335	0.532495379
SNORD116-24	-0.040	0.242692715	0.532352686
RP11-395B20	-0.040	0.242677824	0.532352686
ADAM20P2	-0.040	0.242642434	0.532352686
RC3H1-IT1	-0.040	0.242635741	0.532352686
EIF5AP3	-0.040	0.242553288	0.532352686
CRYGD	-0.040	0.242533773	0.532352686
RP11-893G17	-0.040	0.24250838	0.532352686
RNU6-1298P	-0.040	0.242413417	0.532194331
FBXW8	-0.040	0.242324139	0.532071908
RP5-1186N24	-0.040	0.242318787	0.532071908
SNHG4	-0.040	0.242230091	0.531914451
AC008088	-0.040	0.242218847	0.531914286
TPT1P13	-0.040	0.242194497	0.531885338
CYCSP24	-0.040	0.242182052	0.531882533
CTBP2P5	-0.040	0.24191499	0.531437332
RNU1-28P	-0.040	0.241879107	0.531399864
APOBEC3A	-0.040	0.241856982	0.531389143

RP11-139I14	-0.040	0.241814665	0.531320679
RP11-252I14	-0.040	0.241764281	0.531258993
FLJ39080	-0.040	0.241732608	0.531238415
PLD4	-0.040	0.241716614	0.531238415
RPL19P1	-0.040	0.241676744	0.531189177
MORF4L1P1	-0.040	0.241644073	0.531166395
RNU6-980P	-0.040	0.241606603	0.531157574
RP11-230L22	-0.040	0.241590205	0.531157574
NUP210L	-0.040	0.241560267	0.531157574
PPP2R5C	-0.040	0.241546717	0.531157574
BIRC6-AS1	-0.040	0.241492456	0.531078229
GSK3A	-0.040	0.241394694	0.530912264
SNORD56	-0.040	0.241392612	0.530912264
MAST2	-0.040	0.241361969	0.530889323
RP11-159H3	-0.040	0.241286712	0.530748301
PPAPDC2	-0.040	0.241273475	0.530748301
RP3-509L4	-0.040	0.241223258	0.530706767
TRIM49C	-0.040	0.241210746	0.530706767
PPP1R26P4	-0.040	0.241207065	0.530706767
RP11-56P9	-0.040	0.241120818	0.530579432
CDKN2C	-0.040	0.241099846	0.530557796
RNU6-1057P	-0.040	0.241054772	0.53050763
RP11-265B8	-0.040	0.241050657	0.53050763
KDM4C	-0.040	0.240987287	0.530432643
RSPH1	-0.040	0.240983338	0.530432643
AC092839	-0.040	0.24090066	0.530340008
SNORA2	-0.040	0.240795386	0.530157259
RP11-3D4	-0.040	0.240772803	0.530156555
LINC01094	-0.040	0.240734877	0.530097553
RNA5SP478	-0.040	0.240729491	0.530097553
SNRPCP19	-0.040	0.240685654	0.530062686
RP11-98J23	-0.040	0.240677785	0.530062686
ATF4	-0.040	0.240664077	0.530062686
RP11-513I15	-0.040	0.240607098	0.529963198
AL021807	-0.040	0.240582847	0.529934292
EEF1B2P4	-0.040	0.240547932	0.529881891
MICALCL	-0.040	0.24053983	0.529881891
RP11-1C1	-0.040	0.240535836	0.529881891
AL136231	-0.040	0.240535022	0.529881891
IGHV1OR16-4	-0.040	0.240534471	0.529881891
CWF19L1	-0.040	0.240445853	0.529828566
RP11-1360M22	-0.040	0.240415207	0.529785548
CEBPG	-0.040	0.240355405	0.529727296
TCEB1P31	-0.040	0.240329518	0.529719269
OR6C1	-0.040	0.240297427	0.529697563
RN7SKP26	-0.040	0.240165293	0.529479814
ONECUT2	-0.040	0.240076237	0.529381497
Z97352	-0.040	0.240003505	0.529260516
RP11-496H15	-0.040	0.239890104	0.529093544
LINC00404	-0.040	0.239841354	0.529010521
RP11-224O19	-0.040	0.239830128	0.52901026
RP11-300D11	-0.040	0.239794328	0.52895579

FRG2	-0.040	0.239759049	0.528902465
RP13-216E22	-0.040	0.23975153	0.528902465
IGBP1P4	-0.040	0.239737317	0.528902465
UBAC2-AS1	-0.040	0.239694747	0.528858598
AC016987	-0.040	0.239671243	0.528831239
AC008562	-0.040	0.239629763	0.528788709
RNU4-75P	-0.040	0.239623857	0.528788709
FXYD6P2	-0.040	0.239591185	0.528777079
RPSAP56	-0.040	0.239550912	0.528724236
AL590964	-0.040	0.239514175	0.528680615
MTRNR2L13	-0.040	0.239440686	0.528567395
ZNF3	-0.040	0.239438485	0.528567395
ANLN	-0.040	0.239434336	0.528567395
AC011465	-0.040	0.239430509	0.528567395
GPR26	-0.040	0.239428054	0.528567395
CXorf40A	-0.040	0.239348086	0.528485456
RP11-364B14	-0.040	0.239290679	0.528383198
FAM213B	-0.040	0.239270589	0.528383198
AC104297	-0.040	0.239261532	0.528383198
AGO2	-0.040	0.23924265	0.528383198
RP11-291J9	-0.040	0.239233689	0.528383198
FTH1P9	-0.040	0.23922223	0.528383198
SLMO2P11	-0.040	0.239188881	0.528378889
AC009729	-0.040	0.239149815	0.528341602
RP11-233E12	-0.040	0.23914412	0.528341602
HS6ST3	-0.040	0.239138947	0.528341602
SLC20A1P3	-0.040	0.239101126	0.528332066
PABPN1	-0.040	0.239054563	0.528253685
OSBPL9P5	-0.040	0.239036114	0.528237426
RP11-180C1	-0.040	0.238985019	0.528149018
SLC25A5P1	-0.040	0.238982402	0.528149018
TCAIM	-0.040	0.238957069	0.528136264
CLEC4F	-0.040	0.238898997	0.528081431
GAS6-AS1	-0.040	0.238889909	0.528081431
RNA5SP176	-0.040	0.238832998	0.527984553
RNA5SP462	-0.040	0.23880176	0.527939999
RNU1-129P	-0.040	0.238797364	0.527939999
CTD-2335A18	-0.040	0.238733853	0.527912398
RP11-11M20	-0.040	0.238724658	0.527912398
ERI3-IT1	-0.040	0.238612597	0.527776681
IGLVIVOR22-2	-0.040	0.238584245	0.527776681
ZNF140	-0.040	0.238536663	0.527696857
MYO6	-0.040	0.23850138	0.527667823
TMBIM4	-0.040	0.23849158	0.527667823
BTF3P4	-0.040	0.238484429	0.527667823
RNA5SP29	-0.040	0.23825431	0.527194667
AC006070	-0.040	0.238170754	0.527058753
CCDC26	-0.040	0.238159732	0.527058753
IQCF2	-0.040	0.238094659	0.526963814
RP11-67A1	-0.040	0.23808569	0.526963814
CCND3	-0.040	0.238064573	0.526956311
IFI30	-0.040	0.238027817	0.52691382

RP11-194G10	-0.040	0.237902348	0.526685034
MIR584	-0.040	0.237784962	0.5264741
RP11-263C16	-0.040	0.237761794	0.526447278
C1QTNF9B-AS1	-0.040	0.237742585	0.52642922
DDX1	-0.040	0.237740434	0.52642922
RAB9B	-0.040	0.237715664	0.526418557
PNPT1P1	-0.040	0.237697929	0.526403761
N6AMT1	-0.040	0.237604871	0.526222143
MED30	-0.040	0.237552963	0.526131648
ABT1	-0.040	0.237549705	0.526131648
RP13-39P12	-0.040	0.237538847	0.526131648
MYOZ2	-0.040	0.237538806	0.526131648
RNMT	-0.040	0.237519737	0.526131648
L34079	-0.040	0.237469512	0.526093611
CSN2	-0.040	0.237462525	0.526093611
RN7SL404P	-0.040	0.237457616	0.526093611
RP13-455A7	-0.040	0.237397359	0.526041578
RCN1P2	-0.040	0.237369937	0.526019821
MIR4324	-0.040	0.237365268	0.526019821
RP11-148B3	-0.040	0.237344515	0.526019821
RP11-151H2	-0.040	0.237324519	0.526019821
WDR3	-0.040	0.237183973	0.52580344
TMPRSS11GP	-0.040	0.237154385	0.525762318
RN7SL697P	-0.040	0.237044501	0.525567639
RP11-323F24	-0.040	0.23698261	0.525509126
ZDHHC20P2	-0.040	0.236954161	0.525509126
YPEL3	-0.040	0.236923103	0.525469714
OR5AP1P	-0.040	0.236910634	0.525466528
AARSD1	-0.040	0.236852879	0.525435171
RP11-420K8	-0.040	0.236814471	0.525400046
CTAGE9	-0.040	0.236805742	0.525400046
RNA5SP398	-0.040	0.2367062	0.525257708
AC021087	-0.040	0.236685636	0.525236547
AC092832	-0.040	0.236625717	0.525152518
RP11-642C5	-0.040	0.236580511	0.525146925
CNTFR-AS1	-0.040	0.236555638	0.525119336
LAMA3	-0.040	0.236507605	0.525037182
MIR626	-0.040	0.236502672	0.525037182
RP11-386M24	-0.040	0.236431623	0.52494191
RAB11FIP1P1	-0.040	0.23636614	0.524820986
ZNF736P3Y	-0.040	0.236337193	0.524781178
GATA4	-0.040	0.236330733	0.524781178
FA2H	-0.040	0.236214812	0.524596738
MIR3677	-0.040	0.236207964	0.524596738
RNU6-942P	-0.040	0.236169534	0.524555631
RP11-148B6	-0.040	0.236168043	0.524555631
AL713999	-0.040	0.236076574	0.524446988
MIR3145	-0.040	0.236050877	0.524414364
HIST1H2APS4	-0.040	0.235953689	0.524271816
IGHVIII-38-1	-0.040	0.235935165	0.524255113
HDGFP1	-0.040	0.235911696	0.524227422
RP11-719L21	-0.040	0.235871262	0.524162029

RP11-72I8	-0.040	0.235831574	0.524098287
XK	-0.040	0.235808434	0.524071315
RP11-552F3	-0.040	0.235759362	0.523986707
ODF3B	-0.040	0.235750506	0.523986707
AC109815	-0.040	0.23565506	0.523828225
LINC00504	-0.040	0.235638935	0.523816829
RP11-15H20	-0.040	0.235533705	0.523607345
IMPDH1P4	-0.040	0.235463544	0.523500244
RP11-329J18	-0.040	0.235461905	0.523500244
DENND6B	-0.040	0.235415862	0.523467542
CENPV	-0.040	0.235310573	0.523285343
USP17L6P	-0.040	0.235236391	0.523215036
TARM1	-0.040	0.235189748	0.523135725
PRR20E	-0.040	0.235146071	0.523111865
LINC01558	-0.040	0.235093799	0.523044442
RNU7-128P	-0.040	0.235063029	0.523000414
RP13-147D17	-0.040	0.235040061	0.522973745
PRR12	-0.040	0.234967104	0.522835838
OR4N3P	-0.040	0.234901771	0.522714883
AC016543	-0.040	0.234836106	0.522617598
PRKAR2B	-0.040	0.234807888	0.522579219
HERC2P4	-0.040	0.234774708	0.522554211
RP11-123I22	-0.040	0.234741117	0.522528285
OR5T2	-0.040	0.234683259	0.522497175
KIF4A	-0.040	0.234681474	0.522497175
RP11-713M6	-0.040	0.234678792	0.522497175
ATP5G1P8	-0.040	0.234641127	0.522497175
MAGEA9	-0.040	0.234638021	0.522497175
ARL13A	-0.040	0.234625354	0.522497175
CTD-2215E18	-0.040	0.234624207	0.522497175
ITGB5-AS1	-0.040	0.234615102	0.522497175
KAT2A	-0.040	0.234575987	0.522497175
SLX4	-0.040	0.234495279	0.522469436
BCL2A1	-0.040	0.234469672	0.522436822
RNU6-192P	-0.040	0.234438407	0.522391598
MSANTD4	-0.040	0.234418679	0.522372078
ACBD3	-0.040	0.234417603	0.522372078
DFFBP1	-0.040	0.23440503	0.522372078
FAM87B	-0.040	0.234373309	0.522344295
KDM4A-AS1	-0.041	0.23429736	0.522199464
RNU7-92P	-0.041	0.23428817	0.522199464
RP11-809N8	-0.041	0.234270864	0.522189285
OXSR1	-0.041	0.234256285	0.522181226
BCL2L11	-0.041	0.234221235	0.522127534
KDM5C-IT1	-0.041	0.234165907	0.522028631
ENPP7P13	-0.041	0.234152127	0.522028631
NFS1	-0.041	0.234146633	0.522028631
U51561	-0.041	0.234140989	0.522028631
RP11-245A18	-0.041	0.234097395	0.521998064
AL133335	-0.041	0.234030849	0.521892179
CYB5D1	-0.041	0.234025628	0.521892179
RP11-472E5	-0.041	0.23400584	0.521892179

SPCS1	-0.041	0.233956012	0.521878271
RP11-646I6	-0.041	0.233867508	0.52172972
NUP155	-0.041	0.233826486	0.521717673
GFM1	-0.041	0.23371848	0.521519398
MIR4516	-0.041	0.233615022	0.521361823
NPM1P18	-0.041	0.233582571	0.52131383
AL136001	-0.041	0.233568812	0.52130755
AC008780	-0.041	0.233497514	0.521172842
C6orf58	-0.041	0.23344827	0.521111772
BRD7P4	-0.041	0.233440086	0.521111772
MIR4428	-0.041	0.233352227	0.520970633
OR2F1	-0.041	0.233314538	0.520910908
ZNF134	-0.041	0.233294311	0.520890167
AC011450	-0.041	0.233280239	0.520883166
ENTPD1-AS1	-0.041	0.233277802	0.520883166
RN7SL22P	-0.041	0.233233954	0.520864561
C1orf189	-0.041	0.233211218	0.520851145
PCP2	-0.041	0.233204055	0.520851145
SRSF9P1	-0.041	0.233203412	0.520851145
LINC00363	-0.041	0.233186484	0.520851145
AC008581	-0.041	0.233131678	0.520806474
RP11-305F18	-0.041	0.233102083	0.520794723
ZNF8	-0.041	0.233093253	0.520794723
RP11-409O11	-0.041	0.233027463	0.520713312
MIR429	-0.041	0.232988808	0.520696258
S1PR1	-0.041	0.232982701	0.520696258
RP11-372K14	-0.041	0.232976489	0.520696258
AC118344	-0.041	0.232969541	0.520696258
HERC3	-0.041	0.232956997	0.520696258
AL031905	-0.041	0.232900723	0.520696258
AL365232	-0.041	0.23289785	0.520696258
RP11-26J3	-0.041	0.232855699	0.520696258
VAMP4	-0.041	0.232853557	0.520696258
RP11-437B10	-0.041	0.23283749	0.520696258
SMC5	-0.041	0.232709696	0.52056102
ZNF346	-0.041	0.232678234	0.520530889
RP11-543E8	-0.041	0.232639476	0.520477279
CDC16	-0.041	0.232630941	0.520477279
RN7SKP256	-0.041	0.232421762	0.520063485
CTA-444M12	-0.041	0.232420986	0.520063485
C4orf36	-0.041	0.232417884	0.520063485
RP11-483H20	-0.041	0.232396827	0.520063485
RNU6-166P	-0.041	0.232356591	0.520015382
CASP9	-0.041	0.232352241	0.520015382
NPM1P30	-0.041	0.232349926	0.520015382
RNU6-1141P	-0.041	0.232293318	0.519971512
RP11-26N15	-0.041	0.232267166	0.519945643
LYPLA1P2	-0.041	0.232223988	0.519889625
UAP1L1	-0.041	0.232214369	0.519889625
LINC00362	-0.041	0.232104905	0.519683555
RP11-820K3	-0.041	0.232086977	0.519680604
FFAR1	-0.041	0.232058955	0.519666722

RP11-241I20	-0.041	0.232042482	0.519654266
DNAH7	-0.041	0.232039814	0.519654266
RPS24P14	-0.041	0.232031029	0.519654266
VDAC1P4	-0.041	0.232014348	0.519654266
PHF1	-0.041	0.23201098	0.519654266
PCNAP4	-0.041	0.231960876	0.519618111
CH507-528H12	-0.041	0.231951992	0.519618111
CTD-2561F5	-0.041	0.231874579	0.519498097
RP11-74J13	-0.041	0.231813267	0.519385165
AC090338	-0.041	0.231775085	0.519324049
SNORA66	-0.041	0.231732088	0.519252136
AC124864	-0.041	0.231716928	0.51924327
LINC01109	-0.041	0.231655487	0.519153767
MIR4790	-0.041	0.231612499	0.519101916
RP1-207H1	-0.041	0.231608813	0.519101916
HIST1H4D	-0.041	0.231548777	0.519061169
MIR548F1	-0.041	0.231443577	0.518874188
CRTC2	-0.041	0.23144293	0.518874188
NTS	-0.041	0.2314192	0.518868385
CIRBP	-0.041	0.231414148	0.518868385
AC108105	-0.041	0.231373539	0.518839283
TRMT112P4	-0.041	0.231369504	0.518839283
TRBV6-7	-0.041	0.231284221	0.518736689
RNU2-72P	-0.041	0.231235301	0.518664367
AC098680	-0.041	0.231211378	0.518651949
CCDC180	-0.041	0.231197408	0.518651949
RN7SL219P	-0.041	0.231097792	0.518526729
RP11-295P9	-0.041	0.23109005	0.518526729
RP11-166N6	-0.041	0.231060918	0.518504446
KCNQ2	-0.041	0.231059556	0.518504446
KRT28	-0.041	0.231030695	0.518504446
MTND5P12	-0.041	0.230911417	0.518309142
ANKRD27	-0.041	0.230849689	0.518201354
ZNF18	-0.041	0.230846283	0.518201354
RNU7-27P	-0.041	0.230839368	0.518201354
Z84721	-0.041	0.23081611	0.51819925
AL606500	-0.041	0.230800681	0.518189037
RP11-290C10	-0.041	0.230787325	0.518189037
RPL21P9	-0.041	0.230764091	0.518180169
PTCD2	-0.041	0.230745424	0.518180169
AKAP9	-0.041	0.230744786	0.518180169
HECA	-0.041	0.230737552	0.518180169
RP13-539F13	-0.041	0.230660584	0.518083166
AC073415	-0.041	0.230576902	0.517955199
CWF19L2	-0.041	0.230571653	0.517955199
BRD3	-0.041	0.23053253	0.517904378
MRC1	-0.041	0.230493412	0.517904378
SEMA4A	-0.041	0.23046684	0.517903377
RP11-244M2	-0.041	0.230405473	0.517789906
CSAG2	-0.041	0.230384385	0.517766946
LL22NC03-27C5	-0.041	0.230320738	0.51767276
AKR1C7P	-0.041	0.230291806	0.517632161

RNU7-141P	-0.041	0.230257289	0.517603432
MAP3K5	-0.041	0.230256252	0.517603432
PPIAL4E	-0.041	0.230253798	0.517603432
RNU6-402P	-0.041	0.230174695	0.517564335
AC093766	-0.041	0.230163451	0.517563489
AP4B1	-0.041	0.230092722	0.517453304
MIB1	-0.041	0.230080407	0.517450043
RNA5SP153	-0.041	0.230035698	0.517398356
KDM3A	-0.041	0.230005044	0.517378276
LINC01166	-0.041	0.229932009	0.517238418
AP000787	-0.041	0.229930269	0.517238418
CRB2	-0.041	0.22992817	0.517238418
CTD-2184C24	-0.041	0.229913531	0.517238418
AL034449	-0.041	0.229912506	0.517238418
MIR517A	-0.041	0.229805052	0.517148233
HNRNPA1P46	-0.041	0.22977856	0.517113049
TFAP4	-0.041	0.229745759	0.517063664
RIBC1	-0.041	0.229687862	0.51698222
ADM2	-0.041	0.229677047	0.51698222
CYB561A3	-0.041	0.229676411	0.51698222
ACSM5	-0.041	0.229664748	0.51698222
RPS27P25	-0.041	0.229663005	0.51698222
TUBBP11	-0.041	0.229627586	0.51698222
RPL21P121	-0.041	0.229443549	0.516652068
RP11-285A18	-0.041	0.229341907	0.516520878
ZNF192P1	-0.041	0.229275027	0.516419093
EAF2	-0.041	0.229259576	0.516408712
AP001631	-0.041	0.229243984	0.516398016
XXbac-BPG154L12	-0.041	0.229232904	0.51639748
FAM199X	-0.041	0.229208965	0.516367975
ARSDP1	-0.041	0.22916009	0.516367975
FGD4	-0.041	0.229155186	0.516367975
RP1-134E15	-0.041	0.229125729	0.516351429
NPRL2	-0.041	0.229104592	0.516336721
AL160056	-0.041	0.229099427	0.516336721
FOXF1	-0.041	0.229073265	0.516330923
AC105052	-0.041	0.229027077	0.516251247
CTSLP6	-0.041	0.229017211	0.516251247
AGXT2	-0.041	0.228948898	0.516123879
KIAA1841	-0.041	0.228922814	0.516089503
ZDHHC7	-0.041	0.228912659	0.516089503
RP11-388K2	-0.041	0.228862714	0.516010264
AHCYP5	-0.041	0.228739662	0.515823099
CHAF1A	-0.041	0.228656363	0.515659671
RGP1	-0.041	0.228640932	0.515649289
AC021231	-0.041	0.228637026	0.515649289
SLC35G6	-0.041	0.228618199	0.515646854
NBEAP3	-0.041	0.228610674	0.515646854
RANBP2	-0.041	0.228583233	0.515621912
RNA5SP189	-0.041	0.228558054	0.515621912
RP11-285E9	-0.041	0.228513969	0.515558256
RNU6-1244P	-0.041	0.228482207	0.515550876

RP11-21M24	-0.041	0.228459523	0.515550876
MIR6894	-0.041	0.228320319	0.515292225
RP11-467H10	-0.041	0.228290248	0.515270066
DLGAP5	-0.041	0.228285391	0.515270066
COL4A3BP	-0.041	0.228208969	0.51516298
FGF12-AS3	-0.041	0.228184983	0.51513325
RP3-337D23	-0.041	0.228073313	0.514978792
CLIP1	-0.041	0.227998795	0.514859352
IL5	-0.041	0.22798201	0.514845861
SNORD60	-0.041	0.227965956	0.514845861
RNU1-94P	-0.041	0.227861197	0.514646239
KB-226F1	-0.041	0.227780326	0.514512383
AP000462	-0.041	0.227739044	0.514467933
MIR3135B	-0.041	0.227624081	0.514232621
HCG4P7	-0.041	0.2275974	0.514196734
RNU6-740P	-0.041	0.227593292	0.514196734
XX-CR54	-0.041	0.22758556	0.514196734
LINC01588	-0.041	0.227567273	0.514196734
CNIH1	-0.041	0.227548946	0.514184829
ZNF432	-0.041	0.227512926	0.51412783
INPP5J	-0.041	0.227473541	0.514087612
RNU1-16P	-0.041	0.227459771	0.514080886
RP11-328K4	-0.041	0.22741174	0.514021117
MUC6	-0.041	0.227329414	0.513883811
RP11-182M20	-0.041	0.227247113	0.513746534
H2AFZ	-0.041	0.227225336	0.513721689
RNU6-293P	-0.041	0.227221562	0.513721689
CBX5	-0.041	0.227210185	0.513721689
MSANTD2	-0.041	0.227145169	0.513654641
TDGF1P3	-0.041	0.227140176	0.513654641
SRPK1	-0.041	0.22710057	0.513654641
HNRNPA1P47	-0.041	0.227092197	0.513654641
CAPN11	-0.041	0.227074697	0.513649328
RP11-745I13	-0.041	0.227041102	0.513622124
DLG2-AS1_1	-0.041	0.226964196	0.513564126
AC017035	-0.041	0.226918362	0.513515196
ZNF705C	-0.041	0.226837619	0.513430059
WDR64	-0.041	0.226808139	0.513387731
DTX2P1-UPK3BP1-PMS2P11	-0.041	0.226792966	0.513377785
MPP5	-0.041	0.226790444	0.513377785
SLC47A1	-0.041	0.2267543	0.513363457
AATK-AS1	-0.041	0.226749035	0.513363457
AC068946	-0.041	0.226717594	0.513329158
RP3-368A4	-0.041	0.226713958	0.513329158
AP001187	-0.041	0.226694399	0.513329158
MCCC1	-0.041	0.226656185	0.513329158
ATP5G2P3	-0.041	0.226632809	0.513329158
MIR3976	-0.041	0.226629087	0.513329158
CKMT1B	-0.041	0.226563941	0.513225248
CTB-147C13	-0.041	0.226481679	0.513112117
RASA1	-0.041	0.226469493	0.513112117
RP11-234B24	-0.041	0.22636719	0.512999152

RN7SL350P	-0.041	0.22630993	0.512893791
IGKV3-31	-0.041	0.226176489	0.512615763
RP11-631F7	-0.041	0.226149441	0.512603247
ST6GALNAC4P1	-0.041	0.226123532	0.512568913
HNRNPA1P37	-0.041	0.226109232	0.512560895
CTA-390C10	-0.041	0.226099297	0.512560895
MYOD1	-0.041	0.225942818	0.512281186
RP11-759L5	-0.041	0.225942811	0.512281186
RPL22P22	-0.041	0.225880641	0.512213363
AC025335	-0.041	0.225831074	0.512125349
PRPSAP1	-0.041	0.22581557	0.512114574
OR11Q1P	-0.041	0.225811394	0.512114574
CCPG1	-0.041	0.225795249	0.512114574
GPRC5D	-0.041	0.225788907	0.512114574
BAG5	-0.041	0.225781422	0.512114574
RP11-164N20	-0.041	0.225720257	0.512114574
BHLHB9	-0.041	0.225718582	0.512114574
RPS15AP27	-0.041	0.225688523	0.512114574
LRRC25	-0.041	0.225669157	0.512099531
MIR5692A2	-0.041	0.22554207	0.511859914
RN7SKP164	-0.041	0.22554207	0.511859914
SERPINE3	-0.041	0.225520053	0.511858729
MIR513B	-0.041	0.22549932	0.511836064
LINC00608	-0.041	0.225428909	0.511725021
LINGO1-AS1	-0.041	0.22537681	0.511655529
RN7SL583P	-0.041	0.225365486	0.511654208
KRT18P57	-0.041	0.225362516	0.511654208
USP19	-0.041	0.225293486	0.511539514
RNU6-1201P	-0.041	0.225275578	0.51152324
RP11-171G2	-0.041	0.225273054	0.51152324
PTGDS	-0.041	0.2252458	0.511515247
RP11-489G11	-0.041	0.22516839	0.511426148
CAMK2B	-0.041	0.225162367	0.511426148
PPP1R2P3	-0.041	0.225138239	0.511406443
CHD7	-0.041	0.225037155	0.511249981
YPEL1	-0.041	0.22500936	0.51121122
RNU6-1280P	-0.041	0.224988119	0.511187347
RP11-886H22	-0.041	0.224962858	0.511154336
STMN3	-0.041	0.224946256	0.511154336
RP11-354I13	-0.041	0.224904677	0.511095289
AC116533	-0.041	0.224895635	0.511095289
CTC-534B23	-0.041	0.224843271	0.511028894
RNU6-910P	-0.041	0.224802725	0.510961124
RP11-86A5	-0.041	0.224799534	0.510961124
RP11-62C3	-0.041	0.22478153	0.510961124
OR4M1	-0.041	0.22469181	0.510782151
RP11-188D8	-0.041	0.224676699	0.51077218
ZSWIM5P2	-0.041	0.224674119	0.51077218
FAM183B	-0.041	0.224669022	0.51077218
MIR5698	-0.041	0.224638101	0.51077218
RP11-395P16	-0.041	0.224624467	0.51077218
AC139100	-0.041	0.224617566	0.51077218

CASC16	-0.041	0.224598689	0.510765495
OTX2P1	-0.041	0.224592537	0.510765495
AL901608	-0.041	0.224580066	0.510765495
LINC00399	-0.041	0.224565925	0.510765495
TMA16P2	-0.041	0.224557949	0.510765495
RP11-509P12	-0.041	0.224519257	0.510755572
SLC6A15	-0.041	0.224488827	0.510710742
RN7SL272P	-0.041	0.22443836	0.51062032
RNA5SP312	-0.041	0.22441659	0.510595182
ADH5P3	-0.041	0.224376083	0.510551799
RP11-352G9	-0.041	0.224359482	0.510538416
SOD2	-0.041	0.22432967	0.510494968
MIR4499	-0.041	0.224168047	0.510200306
RP11-264B14	-0.041	0.224147106	0.510177023
LIN28AP2	-0.041	0.224129711	0.510161812
RP11-476B13	-0.041	0.224110133	0.510141629
SNORD41	-0.041	0.224052722	0.510084082
HIST1H2APS3	-0.041	0.224030798	0.510082934
USP46-AS1	-0.041	0.2239508	0.509925168
AF254982	-0.041	0.223910779	0.509858418
KATNBL1P4	-0.041	0.223883066	0.509819689
RP11-664I21	-0.041	0.223872345	0.509819689
EGFLAM-AS3	-0.041	0.223856769	0.509819689
CTD-2128F4	-0.041	0.223719436	0.50958294
SNORD11B	-0.041	0.223680894	0.509529842
VN1R76P	-0.041	0.223678335	0.509529842
DDR1	-0.041	0.223675375	0.509529842
RP11-209D14	-0.041	0.223656083	0.509529842
MYZAP	-0.041	0.223636048	0.509525166
RNU6-392P	-0.041	0.223551393	0.509429769
TM2D1	-0.041	0.223544305	0.509429769
TCEB3-AS1	-0.041	0.223534945	0.509429769
CTD-2588J6	-0.041	0.223495856	0.509400704
PSMC1P13	-0.041	0.223463313	0.509350908
AL132768	-0.041	0.223442392	0.509350908
MORN4	-0.041	0.223439489	0.509350908
RNU6-104P	-0.041	0.223350003	0.509214484
OPA1-AS1	-0.041	0.223273688	0.509113364
LINC01544	-0.041	0.223264002	0.509113364
C9orf147	-0.041	0.223215102	0.509046627
RP11-543G18	-0.041	0.223214785	0.509046627
AL158086	-0.041	0.223138313	0.5089755
LINC01507	-0.041	0.223090465	0.50890525
H2AFZP6	-0.041	0.223085356	0.50890525
MIR6895	-0.041	0.223059834	0.50889398
RP11-140I24	-0.042	0.22299143	0.508786665
MED29	-0.042	0.222966589	0.508754359
HAO2	-0.042	0.222936943	0.508711085
AKAP17A	-0.042	0.222869231	0.508613682
RP11-70O5	-0.042	0.222868083	0.508613682
EHMT2	-0.042	0.222863419	0.508613682
RP11-112N13	-0.042	0.222763185	0.508485129

LINC00624	-0.042	0.222715364	0.508449078
GMIP	-0.042	0.222713396	0.508449078
NAP1L4P3	-0.042	0.222658856	0.508368808
ST7-OT4_2	-0.042	0.222638746	0.508347264
CTD-2385L22	-0.042	0.222630955	0.508347264
MTMR1	-0.042	0.222615287	0.50834244
TBC1D3P3	-0.042	0.222611768	0.50834244
ZC3H12A	-0.042	0.222601185	0.50834244
RNU7-120P	-0.042	0.222591355	0.50834244
FOSL1P1	-0.042	0.222591344	0.50834244
LA16c-3G11	-0.042	0.22256938	0.50834244
RNU5E-1	-0.042	0.22253928	0.508339486
RP11-34A14	-0.042	0.222505179	0.50828597
DDX50	-0.042	0.222493783	0.508284314
CT55	-0.042	0.222493524	0.508284314
RP11-259P20	-0.042	0.22249092	0.508284314
REV1	-0.042	0.222482588	0.508284314
AC098826	-0.042	0.222455932	0.508284314
RP11-556O15	-0.042	0.222444523	0.508284314
PI16	-0.042	0.222428597	0.508284314
RP4-803A2	-0.042	0.222355442	0.508212047
HAUS6P3	-0.042	0.222346132	0.508212047
SLC26A4	-0.042	0.222322801	0.508189831
RP11-160H12	-0.042	0.222315131	0.508189831
MAN2A2	-0.042	0.222297527	0.508189831
RN7SL209P	-0.042	0.222259599	0.508139301
SMDT1	-0.042	0.222255059	0.508139301
LINC01337	-0.042	0.222248779	0.508139301
RP11-593P24	-0.042	0.222241225	0.508139301
PIGPP3	-0.042	0.222196845	0.508117786
TRRAP	-0.042	0.222147776	0.508054364
TAF7	-0.042	0.222096426	0.508034507
C9orf171	-0.042	0.222086258	0.508034507
RN7SL444P	-0.042	0.222057675	0.507994663
CTD-2291D10	-0.042	0.221987911	0.507883857
KRT18P40	-0.042	0.221928429	0.50777216
DGKZ	-0.042	0.221857593	0.507658864
SERP1	-0.042	0.221853869	0.507658864
HCG9P5	-0.042	0.221833191	0.507651805
AC005014	-0.042	0.221789384	0.507600336
AC009505	-0.042	0.221784361	0.507600336
MIR219A2	-0.042	0.221726401	0.507563095
WDR31	-0.042	0.221721082	0.507563095
RP11-25H12	-0.042	0.221609088	0.507455937
RP11-256K7	-0.042	0.221608827	0.507455937
RP11-440L16	-0.042	0.221593865	0.507455937
MIR5787	-0.042	0.22158898	0.507455937
CNOT4P1	-0.042	0.221579953	0.507455937
RP3-388M5	-0.042	0.221579499	0.507455937
CTA-833B7	-0.042	0.221571435	0.507455937
STAG3L4	-0.042	0.221530273	0.507455937
AC016743	-0.042	0.221491616	0.50743091

PIK3CA	-0.042	0.221480885	0.507430735
PIEZO2	-0.042	0.221454851	0.507395496
SNX18P25	-0.042	0.221451949	0.507395496
LINC01024	-0.042	0.22128403	0.507142455
PITPNB	-0.042	0.221271846	0.507142455
RNU6-851P	-0.042	0.221232571	0.507105769
RP11-301J16	-0.042	0.22121745	0.507095513
RNU6-534P	-0.042	0.221200016	0.507079956
RPS23P3	-0.042	0.221193914	0.507079956
XX-FW81066F1	-0.042	0.221113866	0.506931265
PSMA3	-0.042	0.221093697	0.50691463
RP11-140A10	-0.042	0.221023296	0.506805408
PPP1R12B	-0.042	0.221001875	0.506796491
LINS	-0.042	0.220831923	0.50645553
snoZ40	-0.042	0.220826615	0.50645553
HNRNPA1P26	-0.042	0.220813844	0.50645553
SAMD12	-0.042	0.220806562	0.50645553
RNA5SP20	-0.042	0.220718384	0.506317044
RP1-12G14	-0.042	0.220597361	0.506136935
TRIM26BP	-0.042	0.220547704	0.506071762
RP11-575G13	-0.042	0.220540042	0.506071762
AL049691	-0.042	0.220500643	0.506012534
C3orf84	-0.042	0.22048726	0.506012534
RP11-326A13	-0.042	0.220486389	0.506012534
CCDC115	-0.042	0.220479323	0.506012534
SETDB1	-0.042	0.220400038	0.505952312
RNA5SP374	-0.042	0.220371361	0.505910866
RP11-165I9	-0.042	0.220323027	0.505848669
NCBP2L	-0.042	0.220302125	0.505825063
ALPI	-0.042	0.220283237	0.505806081
HIST1H4PS1	-0.042	0.220278144	0.505806081
RADIL	-0.042	0.220172183	0.505672978
RP11-820I16	-0.042	0.220153304	0.505654004
MASTL	-0.042	0.220120337	0.505602666
RPL23P2	-0.042	0.220015232	0.505409996
ZBTB21	-0.042	0.219921059	0.505221319
C3orf17	-0.042	0.21984089	0.505106955
FP325331	-0.042	0.219757603	0.50497652
AC004824	-0.042	0.219716239	0.504917989
RN7SL847P	-0.042	0.219695101	0.504893774
AC004067	-0.042	0.219694585	0.504893774
AC015691	-0.042	0.219690951	0.504893774
HCG18	-0.042	0.219688628	0.504893774
RPL7P27	-0.042	0.219688045	0.504893774
RNA5SP70	-0.042	0.219670207	0.504893774
RP13-580B18	-0.042	0.219667186	0.504893774
RP13-483F6	-0.042	0.219633918	0.504893774
Y_RNA	-0.042	0.219559644	0.50477733
RP11-134N1	-0.042	0.219532691	0.504764095
RP11-38C17	-0.042	0.219522776	0.504764095
PAICSP4	-0.042	0.21950524	0.504764095
PPP1R2	-0.042	0.219443851	0.504730396

EHMT1	-0.042	0.219365039	0.504646607
MYO1A	-0.042	0.219352743	0.504646607
CDK5PS	-0.042	0.219348251	0.504646607
CTB-10G5	-0.042	0.219245749	0.504591537
SPATA31A1	-0.042	0.219244041	0.504591537
TDGF1P4	-0.042	0.219238396	0.504591537
CTC-499B15	-0.042	0.21918116	0.504540412
MED24	-0.042	0.21916338	0.504523867
SNORA36A	-0.042	0.219054095	0.504345422
MIR4319	-0.042	0.219047561	0.504345422
MTND2P14	-0.042	0.219036745	0.504345422
ZNF850	-0.042	0.219036462	0.504345422
AC090587	-0.042	0.219015348	0.504345422
AC010207	-0.042	0.219013625	0.504345422
CA11	-0.042	0.21900551	0.504345422
IL19	-0.042	0.218987069	0.504345422
AC016656	-0.042	0.218882814	0.50421919
LINC00350	-0.042	0.218870116	0.504214329
AC007277	-0.042	0.218859612	0.504214329
SNORD116-19	-0.042	0.218851723	0.504214329
SLCO3A1	-0.042	0.218821294	0.504214329
AC093151	-0.042	0.218799626	0.504214329
LINC01176	-0.042	0.218778279	0.504214329
LY6G6E	-0.042	0.21873581	0.504214329
RP11-3L10	-0.042	0.218699167	0.504210728
ALG5	-0.042	0.218670357	0.504168713
ITM2B	-0.042	0.21850558	0.503910771
ASTE1	-0.042	0.218460563	0.50383135
RN7SKP12	-0.042	0.218352669	0.503631292
RP11-18F14	-0.042	0.218290972	0.503513372
GPHA2	-0.042	0.218204568	0.50343598
HCFC2	-0.042	0.218198369	0.50343598
AP000403	-0.042	0.218177997	0.503423452
KRTAP5-1	-0.042	0.218160315	0.503423452
CERS6-AS1	-0.042	0.218146549	0.503423452
RNU6-1136P	-0.042	0.218137835	0.503423452
RP11-735B13	-0.042	0.218054145	0.503308363
MIR7155	-0.042	0.218043277	0.503307671
RN7SL569P	-0.042	0.218030476	0.503302515
RN7SKP98	-0.042	0.218027251	0.503302515
GOLGA8VP	-0.042	0.217980825	0.503236683
CADM3	-0.042	0.217898436	0.50309525
CCDC126	-0.042	0.217855323	0.503020092
C8orf44	-0.042	0.217819794	0.502962441
AC000029	-0.042	0.217805293	0.502953343
AL138751	-0.042	0.217781166	0.502922014
HMGN2P11	-0.042	0.217730464	0.50285369
AC083873	-0.042	0.217703178	0.502839441
ZNF17	-0.042	0.217680577	0.502811623
SARDH	-0.042	0.217646701	0.502782143
C2orf47	-0.042	0.217632417	0.502773532
RP11-1023P17	-0.042	0.217597579	0.502717433

RNA5SP372	-0.042	0.217596784	0.502717433
SMR3B	-0.042	0.217551688	0.502660179
RP11-94C24	-0.042	0.217502463	0.502570823
SLC2A11	-0.042	0.2174888	0.502563634
KLF13	-0.042	0.217483905	0.502563634
NCDN	-0.042	0.217473708	0.502563634
RASSF6	-0.042	0.217414162	0.5024643
MIR3908	-0.042	0.217380022	0.50243416
RP11-339N8	-0.042	0.217375555	0.50243416
RBMY2KP	-0.042	0.217370772	0.50243416
SLC25A6P6	-0.042	0.217358352	0.50243416
REPIN1	-0.042	0.217315896	0.502407854
RP11-388G22	-0.042	0.217306621	0.502407854
CTD-2269E23	-0.042	0.21727293	0.50238168
CNN2P12	-0.042	0.217261381	0.502379365
PRPF3	-0.042	0.217223368	0.502315855
LA16c-395F10	-0.042	0.217194124	0.502272617
CHURC1-FNTB	-0.042	0.217193969	0.502272617
LYPLA2P3	-0.042	0.21716346	0.502250479
NPM1P52	-0.042	0.217158215	0.502250479
TRPC7-AS1	-0.042	0.217113976	0.502211563
CTBP1-AS2	-0.042	0.217113757	0.502211563
PANDAR	-0.042	0.217084061	0.502211563
CD72	-0.042	0.217052653	0.502189298
CTD-2118P12	-0.042	0.217049172	0.502189298
LL0XNC01-177E8	-0.042	0.21703666	0.502189298
RP11-293F5	-0.042	0.216983182	0.502126137
XRCC5	-0.042	0.21697914	0.502126137
CTD-2008A1	-0.042	0.216968257	0.502126137
RP11-124I4	-0.042	0.216967032	0.502126137
RP11-331F9	-0.042	0.216932846	0.502126137
RNU7-85P	-0.042	0.216915907	0.502126137
SSR1	-0.042	0.216911753	0.502126137
RP11-161H23	-0.042	0.216896743	0.502126137
ATP5G2P1	-0.042	0.216873821	0.502126137
CH507-396I9	-0.042	0.216847279	0.502126137
RP11-456J20	-0.042	0.216838974	0.502126137
HMGN2P9	-0.042	0.21679788	0.502126137
CCDC150P1	-0.042	0.21675549	0.502111563
OR10AB1P	-0.042	0.216737633	0.502094617
RP11-772C9	-0.042	0.216692201	0.502045372
MBD4	-0.042	0.216688728	0.502045372
LINC01187	-0.042	0.216647729	0.502025198
RNU6-1016P	-0.042	0.216645204	0.502025198
MIR5703	-0.042	0.216635479	0.502025198
BCRP6	-0.042	0.21658891	0.501994259
AL358133	-0.042	0.216586161	0.501994259
DBR1	-0.042	0.216550844	0.501994259
INTS12	-0.042	0.216408756	0.501723205
FHIT	-0.042	0.216374006	0.501667062
RP11-292K15	-0.042	0.216292605	0.501502745
IRF2BP2	-0.042	0.216286438	0.501502745

RNU6-783P	-0.042	0.21621402	0.501418179
MMADHC	-0.042	0.216160719	0.501318979
AC142528	-0.042	0.216158292	0.501318979
PPM1N	-0.042	0.216111583	0.501278252
HS3ST6	-0.042	0.216035922	0.50117598
UNC5A	-0.042	0.215955903	0.501087975
AC007740	-0.042	0.215871515	0.500996194
AL355075	-0.042	0.215870056	0.500996194
MIR1179	-0.042	0.215833212	0.500974141
HSPE1-MOB4	-0.042	0.215769457	0.500874978
ZBTB33	-0.042	0.215737234	0.500849001
RP11-2J18	-0.042	0.215716189	0.500824557
FBP2P1	-0.042	0.215683105	0.500772155
RP11-622J9	-0.042	0.215650646	0.500721203
OR7A10	-0.042	0.215635394	0.500721203
ZNF35	-0.042	0.215601205	0.500703166
RNF5	-0.042	0.215599786	0.500703166
MIR6846	-0.042	0.215546945	0.500651273
RP1-55C23	-0.042	0.215505642	0.500579751
RP11-15L13	-0.042	0.215497631	0.500579751
IGHD	-0.042	0.215464341	0.50053264
OR10D3	-0.042	0.21544062	0.50050266
RP11-43N5	-0.042	0.215412149	0.500484629
RNU6-496P	-0.042	0.21540351	0.500484629
RP11-239E10	-0.042	0.215400924	0.500484629
NFASC	-0.042	0.215382187	0.500484629
LINC01221	-0.042	0.215350565	0.500463617
CTD-2134P3	-0.042	0.21531845	0.500437821
MTND6P5	-0.042	0.215302795	0.500425855
C1orf95	-0.042	0.215288024	0.500415946
OR4F6	-0.042	0.215287677	0.500415946
AC011385	-0.042	0.215231995	0.500383395
RNU6-517P	-0.042	0.215218053	0.500375407
CYP4F24P	-0.042	0.215211976	0.500375407
SNORD70	-0.042	0.215168191	0.500339353
RP11-67H2	-0.042	0.215078062	0.500220863
RBFOX3	-0.042	0.21507509	0.500220863
AK3P5	-0.042	0.215072471	0.500220863
AC103724	-0.042	0.215008729	0.50013288
RBM17P4	-0.042	0.214930458	0.499975234
ARHGEF33	-0.042	0.214894077	0.49991502
SLC39A9	-0.042	0.214891853	0.49991502
RP11-776K3	-0.042	0.214878642	0.49991502
CTD-2132H18	-0.042	0.214811729	0.499821105
RP11-420K14	-0.042	0.214807165	0.499821105
RP11-544M22	-0.042	0.214714777	0.49964434
RP11-118D22	-0.042	0.214691377	0.499614297
MIR4459	-0.042	0.21455496	0.499345636
IGHVII-49-1	-0.042	0.214514631	0.499300574
DOCK11	-0.042	0.214471975	0.499225686
DHX29	-0.042	0.214457841	0.499217185
GPATCH4	-0.042	0.21445663	0.499217185

MIR1180	-0.042	0.214438453	0.499217185
GNL3LP1	-0.042	0.214435336	0.499217185
RP11-517M22	-0.042	0.214353893	0.499097183
AC016959	-0.042	0.214352555	0.499097183
SATL1	-0.042	0.214325627	0.499097183
PCNXL4	-0.042	0.214325218	0.499097183
FAM200B	-0.042	0.214253178	0.498984651
ADCY3	-0.042	0.21422473	0.498942795
AC064852	-0.042	0.214086227	0.498742161
SATB1	-0.042	0.214039179	0.498701899
RP4-635A23	-0.042	0.214008513	0.498701899
LHFPL4	-0.042	0.213989254	0.498694772
MTFMT	-0.042	0.213946246	0.498635568
BNIP3P26	-0.042	0.213916575	0.498635568
PRY2	-0.042	0.213804468	0.498475885
SNORD115-41	-0.042	0.213803503	0.498475885
RP11-16C18	-0.042	0.213766922	0.498437149
UPRT	-0.042	0.21373851	0.498395301
TOB2P1	-0.042	0.213737863	0.498395301
LINC00969	-0.042	0.213721793	0.498395301
RP11-762N20	-0.042	0.213721282	0.498395301
RP11-385N23	-0.042	0.213697641	0.498395301
LINC00523	-0.042	0.213686931	0.498395301
IQCB1	-0.042	0.213680507	0.498395301
ZFP69B	-0.042	0.213656342	0.498374504
MIR7847	-0.042	0.213643643	0.49836929
ERBB4	-0.042	0.213642998	0.49836929
RP11-256L6	-0.042	0.213637845	0.49836929
DYNC1LI2	-0.042	0.213603389	0.498357948
PA2G4	-0.042	0.213594732	0.498357948
RP1-8B22	-0.042	0.213591823	0.498357948
RP11-3N13	-0.042	0.213590272	0.498357948
MIR4303	-0.042	0.213527054	0.498292566
AC132825	-0.042	0.213428078	0.498134817
UBE2Q2P8	-0.042	0.21335639	0.49801631
RNU7-147P	-0.042	0.21335639	0.49801631
ARHGEF3	-0.042	0.213342382	0.49801631
RP11-219B4	-0.042	0.213255242	0.497951043
MRPL57	-0.042	0.213241221	0.497942716
CTD-3060P21	-0.042	0.213239205	0.497942716
RNU6-510P	-0.042	0.213217787	0.497936822
SNORD59	-0.042	0.213212673	0.497936822
AC072062	-0.042	0.213129266	0.497803323
SSXP1	-0.042	0.213113551	0.497791031
HIPK2	-0.042	0.213096076	0.497774624
JARID2-AS1	-0.042	0.21305006	0.497691545
SGSM1	-0.042	0.213043624	0.497691545
THAP9-AS1	-0.042	0.213022325	0.497675574
KIDINS220	-0.042	0.213016096	0.497675574
ZIK1P1	-0.042	0.212974626	0.497631924
CTC-482H14	-0.042	0.212908742	0.497532243
MAGEE1	-0.042	0.212823067	0.497380843

RN7SL239P	-0.042	0.212810477	0.497375826
CYCSP39	-0.042	0.212786405	0.497343973
RP11-60I3	-0.042	0.212774593	0.497340771
SMG7-AS1	-0.042	0.212770864	0.497340771
WASH3P	-0.042	0.212738343	0.497304852
AP004290	-0.042	0.212693428	0.497257474
RNU4ATAC6P	-0.042	0.212637435	0.497211576
SNX19	-0.042	0.212605684	0.497189969
TAF1L	-0.042	0.212506914	0.497056614
RNU6-126P	-0.042	0.212472639	0.497049678
TMEM147-AS1	-0.042	0.212466466	0.497049678
EFTUD1P1	-0.042	0.21241894	0.496972877
RP11-809O17	-0.042	0.212356583	0.496851394
RP11-356M6	-0.042	0.212272795	0.496679754
INSL3	-0.042	0.212264631	0.496679754
FLRT1	-0.042	0.212228502	0.496659152
RP11-474G23	-0.042	0.21219386	0.49663425
LINC01135	-0.042	0.212181827	0.49663425
GPR142	-0.042	0.212171951	0.49663425
TATDN1	-0.042	0.212162382	0.49663425
RP11-420J11	-0.042	0.212136734	0.49662978
RP11-90P5	-0.042	0.212086289	0.496560499
ZNF260	-0.042	0.21205802	0.496518721
RP11-12D24	-0.042	0.212047218	0.496517839
RP11-490N5	-0.043	0.211995871	0.496470834
TMEM9B	-0.043	0.211983527	0.496466336
AC005624	-0.043	0.211932402	0.496395421
MIR129-1	-0.043	0.211884454	0.496331934
LSP1P3	-0.043	0.211768859	0.496085557
DNASE2B	-0.043	0.211718113	0.495991076
RNU6-249P	-0.043	0.211661712	0.495883337
AC068207	-0.043	0.211649139	0.495883337
NUP43	-0.043	0.211616942	0.495883337
GUCA1B	-0.043	0.211611743	0.495883337
LINC00457	-0.043	0.211611196	0.495883337
AC079896	-0.043	0.211537032	0.495810738
ZNF815P	-0.043	0.211516972	0.49578812
PPP1R11	-0.043	0.211476993	0.495743207
CAMLG	-0.043	0.211456806	0.495720283
MIR3130-1	-0.043	0.211421209	0.495661231
MIR3978	-0.043	0.211393003	0.495619502
FANCE	-0.043	0.211384063	0.495619502
GTF2I	-0.043	0.21136628	0.495619502
RP4-749H3	-0.043	0.211342062	0.495619502
PKP4P1	-0.043	0.211315521	0.495608623
RP4-739H11	-0.043	0.211245075	0.495516606
NACAP5	-0.043	0.211148753	0.495339458
RPL7P4	-0.043	0.211081294	0.495254387
AC110769	-0.043	0.21100928	0.49516264
RP11-73B2	-0.043	0.210973121	0.49512254
GOSR2	-0.043	0.210971533	0.49512254
RASSF4	-0.043	0.210956368	0.49512254

RP3-525N14	-0.043	0.210948729	0.49512254
C1orf27	-0.043	0.210803173	0.494918798
LINC00469	-0.043	0.210770377	0.494890591
MIR7-3HG	-0.043	0.210749032	0.494864869
RP11-701B16	-0.043	0.21068718	0.494744027
CCDC150	-0.043	0.210598241	0.494583947
DNAJA1P3	-0.043	0.210529026	0.494494546
AC068279	-0.043	0.210502149	0.494455802
EIF1	-0.043	0.210492642	0.494455802
MIR6750	-0.043	0.210485506	0.494455802
AC096509	-0.043	0.210476186	0.494455802
SETD3	-0.043	0.210452604	0.494436966
TMEM55A	-0.043	0.210404406	0.49439583
RP4-752I6	-0.043	0.210368732	0.494337459
E2F1	-0.043	0.210321772	0.494251493
B3GALT5	-0.043	0.210249909	0.494155764
RP11-547D23	-0.043	0.210201825	0.494067134
CSNK2B-LY6G5B-562	-0.043	0.210138084	0.493975795
RNU1-141P	-0.043	0.210128521	0.493975795
RP11-282O18	-0.043	0.210038603	0.493854094
HNRNPCP4	-0.043	0.209941947	0.49372433
RP11-554D20	-0.043	0.20991329	0.493681312
MIR345	-0.043	0.209821525	0.493514236
FTX_4	-0.043	0.209815796	0.493514236
C6orf201	-0.043	0.209757427	0.493412209
WI2-3308P17	-0.043	0.209752754	0.493412209
RP11-502N13	-0.043	0.209747373	0.493412209
SSH3	-0.043	0.209718951	0.493394808
KRT18P42	-0.043	0.209708469	0.493394518
MTND5P28	-0.043	0.209698585	0.493394518
CLIC1P1	-0.043	0.20969388	0.493394518
PTP4A1	-0.043	0.209680212	0.493394518
RP11-248G5	-0.043	0.209617105	0.493325784
RNU6-899P	-0.043	0.209538475	0.493165099
CPHL1P	-0.043	0.209537419	0.493165099
RP11-629B11	-0.043	0.209531459	0.493165099
RP11-728D14	-0.043	0.209489005	0.493165099
AC092854	-0.043	0.209480626	0.493165099
MT-TN	-0.043	0.209477667	0.493165099
UGT2B29P	-0.043	0.209473978	0.493165099
SMN2	-0.043	0.209460986	0.493165099
RP3-403A15	-0.043	0.20943418	0.493165099
RN7SL132P	-0.043	0.209424416	0.493165099
TTC30B	-0.043	0.20940124	0.49315892
KIAA1586	-0.043	0.209382188	0.493138437
RPS15AP40	-0.043	0.209325586	0.493053892
UBAP2	-0.043	0.209203441	0.492814933
LINC00656	-0.043	0.20919786	0.492814933
RP11-381K20	-0.043	0.209055684	0.492613055
CC2D2B	-0.043	0.209021712	0.492557373
PCNT	-0.043	0.209020048	0.492557373
ANO1	-0.043	0.208965945	0.49251214

TMEM57	-0.043	0.208927306	0.492456735
C1orf174	-0.043	0.208893427	0.492401249
RP11-349A8	-0.043	0.208882967	0.492400964
RP3-525N10	-0.043	0.208810475	0.492259937
BHLHE23	-0.043	0.20877064	0.492209262
KIFC1	-0.043	0.208755375	0.492197636
RP4-669P10	-0.043	0.208723298	0.492170735
LINC00851	-0.043	0.208692873	0.492147725
CDCA4P1	-0.043	0.208642511	0.492053323
B3GAT1	-0.043	0.208626649	0.492040281
MAZ	-0.043	0.208624119	0.492040281
RNA5SP50	-0.043	0.208538539	0.491905551
RP11-1299A16	-0.043	0.208501098	0.491841596
AC009414	-0.043	0.208449743	0.491769164
AGAP1	-0.043	0.208419336	0.491746146
HIST1H2AG	-0.043	0.208413996	0.491746146
KCNH6	-0.043	0.208404127	0.491746146
SLC25A39P1	-0.043	0.208359699	0.491702874
RP11-20P5	-0.043	0.208260934	0.491494152
GYPE	-0.043	0.208251011	0.491494152
SLC4A1APP1	-0.043	0.208249402	0.491494152
AC144450	-0.043	0.208230561	0.491494152
SPANXA1	-0.043	0.208183639	0.491433495
PAGE5	-0.043	0.208176564	0.491433495
BAHD1	-0.043	0.208172207	0.491433495
GSK3B	-0.043	0.208152644	0.491433495
MIAT	-0.043	0.208090405	0.491368229
LINC01103	-0.043	0.208050007	0.491312856
HNRNPA1P67	-0.043	0.207970497	0.491173801
PRKD2	-0.043	0.207969721	0.491173801
AQP6	-0.043	0.207950956	0.491173801
CDRT15P2	-0.043	0.207852568	0.490992673
SSU72P5	-0.043	0.207850814	0.490992673
C9orf173	-0.043	0.207817701	0.490959011
RNU6-361P	-0.043	0.20779324	0.490959011
RN7SL105P	-0.043	0.207755493	0.490940003
MTCO3P1	-0.043	0.207749133	0.490940003
RN7SL411P	-0.043	0.207692961	0.490884004
CC2D1B	-0.043	0.207679968	0.490884004
EML4	-0.043	0.207675036	0.490884004
RNU6-298P	-0.043	0.207671246	0.490884004
IL12A	-0.043	0.207644012	0.490884004
OR2H1	-0.043	0.207616744	0.490884004
RNU6-624P	-0.043	0.207576445	0.490827293
SULT1A3	-0.043	0.207570584	0.490827293
CTB-191K22	-0.043	0.207550175	0.490827293
AC007256	-0.043	0.207520381	0.490816565
AC093642	-0.043	0.20750602	0.490806974
RP11-797A18	-0.043	0.207372926	0.490540894
AC080002	-0.043	0.20736065	0.490536218
LINC00692	-0.043	0.207354168	0.490536218
CTB-43E15	-0.043	0.207352816	0.490536218

COL6A5	-0.043	0.207344225	0.490536218
AHSP	-0.043	0.207300773	0.490492022
IQSEC2	-0.043	0.207284696	0.490485341
ASF1B	-0.043	0.207280952	0.490485341
RP11-520P18	-0.043	0.207265563	0.490485341
RP11-374I15	-0.043	0.207203065	0.490407015
SNORD71	-0.043	0.207139634	0.490305618
VCX	-0.043	0.207135173	0.490305618
RP11-179K3	-0.043	0.207041253	0.490121466
BMS1	-0.043	0.207034015	0.490121466
RPL36AP13	-0.043	0.207015034	0.490108118
RP11-359H18	-0.043	0.206988857	0.490075163
MTCP1	-0.043	0.206946437	0.490043154
RP11-574F11	-0.043	0.206943295	0.490043154
RN7SL667P	-0.043	0.206930359	0.490043154
RP11-374N7	-0.043	0.206852177	0.490010567
CHKB-AS1	-0.043	0.206809121	0.489973699
CTD-2161E19	-0.043	0.206789965	0.48996496
SLC35B3	-0.043	0.206776278	0.489956904
RPL34-AS1	-0.043	0.206718058	0.489892072
LLGL2	-0.043	0.206717876	0.489892072
AMER3	-0.043	0.206702291	0.489892072
OR2C1	-0.043	0.206647354	0.489822003
MSR1	-0.043	0.206594428	0.489720924
RP1-253P7	-0.043	0.206587737	0.489720924
FUNDC2P3	-0.043	0.206523492	0.489625881
MTND1P20	-0.043	0.206505218	0.48960693
CTD-2089O24	-0.043	0.206460406	0.489554139
IGLVV-66	-0.043	0.206441632	0.489553656
RN7SKP272	-0.043	0.206420306	0.489529433
RINT1	-0.043	0.206406605	0.489529433
LINC00667	-0.043	0.206368113	0.489525556
RP11-153F5	-0.043	0.206345869	0.489497172
CTB-1I21	-0.043	0.206276202	0.489429417
RPL37AP1	-0.043	0.2060942	0.489021945
RP11-432J22	-0.043	0.20607841	0.48900884
RNU6ATAC16P	-0.043	0.206075268	0.48900884
CES2	-0.043	0.206042512	0.488996747
DPPA2	-0.043	0.206017138	0.488980148
TRIM5	-0.043	0.205943277	0.488834311
RP11-861E23	-0.043	0.205919377	0.488814258
ATXN2L	-0.043	0.205889359	0.488786856
NT5DC3	-0.043	0.205691422	0.488455593
LPHN3-AS1	-0.043	0.205644282	0.488367999
PTPRB	-0.043	0.205595052	0.488275433
RNU7-151P	-0.043	0.205551158	0.488219878
MIR16-1	-0.043	0.20548747	0.488117293
RP11-351C21	-0.043	0.205486168	0.488117293
AP000783	-0.043	0.205470039	0.488117293
RN7SL574P	-0.043	0.205465235	0.488117293
OSGEP	-0.043	0.205454863	0.488117293
RP1-28H20	-0.043	0.205453295	0.488117293

MKNK1-AS1		-0.043	0.205445646	0.488117293
IGHVIII-25-1		-0.043	0.205435653	0.488117293
CNN2P7		-0.043	0.205351541	0.487989115
SLCO4C1		-0.043	0.20531702	0.487931425
KRTAP2-5P		-0.043	0.205303788	0.487924327
WIPF3		-0.043	0.205303202	0.487924327
RP11-316J7		-0.043	0.205301005	0.487924327
SMAD7		-0.043	0.205266032	0.487924327
COA7		-0.043	0.205243929	0.487903798
KRT18P29		-0.043	0.205201475	0.487851577
RNU6-313P		-0.043	0.205052618	0.487548867
	8-Mar	-0.043	0.205050547	0.487548867
RP11-526K21		-0.043	0.205007724	0.487548867
RP11-480A16		-0.043	0.20499719	0.487548867
TMPPE		-0.043	0.204991132	0.487548867
RNU7-106P		-0.043	0.204956035	0.487548867
RP11-226L15		-0.043	0.204948142	0.487548867
RP11-63C8		-0.043	0.20492646	0.487548867
GMFBP1		-0.043	0.204888423	0.487496674
SNORD69		-0.043	0.204826918	0.48737468
SLC30A6		-0.043	0.204751838	0.487269065
SRMS		-0.043	0.204750073	0.487269065
ATF4P3		-0.043	0.204721895	0.487246499
RP11-802O23		-0.043	0.204695731	0.487208574
DHRS9		-0.043	0.204693071	0.487208574
MIR25		-0.043	0.204659032	0.487194268
RPS27P14		-0.043	0.2045889	0.487051663
RAB11A		-0.043	0.204563476	0.487023255
RP11-576E20		-0.043	0.204556164	0.487023255
HNRNPA1P39		-0.043	0.204508004	0.486956441
SCRN1		-0.043	0.204471781	0.486894531
SNORD116-4		-0.043	0.204458108	0.486886315
	7-Mar	-0.043	0.204397471	0.486766256
SDHDP2		-0.043	0.204393757	0.486766256
HBBP1		-0.043	0.204388738	0.486766256
CTC-471J1		-0.043	0.20438226	0.486766256
RP11-519C12		-0.043	0.204335342	0.486736369
AL050321		-0.043	0.204325535	0.486736369
RNU6-103P		-0.043	0.204174438	0.486488637
RNU6-415P		-0.043	0.204139441	0.486443646
CHST1		-0.043	0.204062248	0.486308365
BNIP3P39		-0.043	0.20394737	0.486107567
CTD-2587H19		-0.043	0.20394246	0.486107567
KHDC3L		-0.043	0.203913429	0.486075319
CTD-2057J6		-0.043	0.203909628	0.486075319
RNU6-76P		-0.043	0.203908918	0.486075319
AC012531		-0.043	0.203905807	0.486075319
RP11-182B22		-0.043	0.203893895	0.486075319
RP11-235C23		-0.043	0.203867358	0.486075319
NANOGP6		-0.043	0.203853761	0.486075319
AL391001		-0.043	0.203843831	0.486075319
RP11-64K7		-0.043	0.203760497	0.486075319

CEP41	-0.043	0.203693574	0.485952452
RP11-120D5	-0.043	0.203688496	0.485952452
RP11-108K3	-0.043	0.20366982	0.485952452
MIR6838	-0.043	0.203669448	0.485952452
RP11-519M16	-0.043	0.20352973	0.485671148
C19orf54	-0.043	0.203448968	0.485527084
CTC-756D1	-0.043	0.203436045	0.485520572
RP11-598P20	-0.043	0.203391014	0.485437426
RP5-886K2	-0.043	0.203356201	0.485427315
LINC01033	-0.043	0.203282953	0.485276787
AC007792	-0.043	0.203248752	0.485219465
ALG1L15P	-0.043	0.203218672	0.485196296
RNA5SP163	-0.043	0.203172344	0.485110004
LLNLR-249E10	-0.043	0.203100236	0.484986461
OR56B1	-0.043	0.203066751	0.48495513
AC074019	-0.043	0.203013343	0.484876209
RP11-398C13	-0.043	0.202846144	0.48454977
SALL4P7	-0.043	0.202824038	0.484521264
SNX19P2	-0.043	0.202724428	0.4843076
GOLGA8K	-0.043	0.202714145	0.484307328
AC004869	-0.043	0.202710173	0.484307328
RP11-461G12	-0.043	0.202650407	0.48420363
TCF7	-0.043	0.202630182	0.484179596
PCMT1	-0.043	0.202627082	0.484179596
RP4-738P11	-0.043	0.202598772	0.484153122
NRD1	-0.043	0.202596466	0.484153122
MCM5	-0.043	0.202535049	0.484098003
ALS2CR12	-0.043	0.202501336	0.484041712
ZNF619P1	-0.043	0.202498877	0.484041712
RNU2-11P	-0.043	0.202479292	0.484037607
RP11-118B18	-0.043	0.202431987	0.483948812
SCUBE2	-0.043	0.202422714	0.483948812
ANKRD33B	-0.043	0.202404706	0.483932176
URAHP	-0.043	0.202404706	0.483932176
AL021398	-0.043	0.202359192	0.48389623
MIR937	-0.043	0.202351303	0.48389623
FGF22	-0.043	0.20228232	0.483837591
RP11-717F1	-0.043	0.202278119	0.483837591
SP7	-0.043	0.202272874	0.483837591
CTD-2536I1	-0.043	0.202237498	0.483837591
RP11-429B14	-0.043	0.202215238	0.483837591
RNU6-518P	-0.043	0.202148063	0.483804312
RP1-223H12	-0.043	0.202147048	0.483804312
RP11-863N1	-0.043	0.202117889	0.483780716
MARS	-0.043	0.202111898	0.483780716
CTC-287O8	-0.043	0.202079891	0.483738389
C1orf131	-0.043	0.202079211	0.483738389
ZNF789	-0.043	0.202059678	0.483738389
C2orf50	-0.043	0.202051943	0.483738389
RP11-499F3	-0.043	0.202028616	0.483712894
IGFLR1	-0.043	0.202005148	0.483705338
EXOSC3P1	-0.043	0.201991082	0.483695977

OSTCP8	-0.043	0.201974546	0.483680697
RHD	-0.043	0.201974476	0.483680697
AF230666	-0.043	0.201911002	0.483625793
MIR4646	-0.043	0.201857304	0.483570134
RP11-70L8	-0.043	0.201837101	0.483567981
SULT1C4	-0.043	0.201835416	0.483567981
SNORA71A	-0.043	0.20183526	0.483567981
RNU6-77P	-0.043	0.201815339	0.483567981
MGC50722	-0.043	0.201800678	0.483567981
RN7SKP5	-0.043	0.201764088	0.483541398
GLT6D1	-0.043	0.201693234	0.483420243
RP11-379H18	-0.043	0.201690936	0.483420243
AC012181	-0.043	0.201626207	0.483332563
ZNF548	-0.043	0.201608481	0.483314397
GAB2	-0.043	0.201550564	0.483220726
RP11-129J12	-0.043	0.201530661	0.483200805
AC005884	-0.043	0.201524254	0.483200805
ZER1	-0.043	0.201497871	0.483170833
CTD-2185K10	-0.043	0.201487689	0.483170744
GPR133	-0.044	0.201445187	0.483093149
CTD-2168K21	-0.044	0.201327466	0.482859464
RPL12P29	-0.044	0.201283752	0.482827563
NHP2P2	-0.044	0.201271247	0.482821885
ZNF287	-0.044	0.201257885	0.482814149
C6orf229	-0.044	0.201257546	0.482814149
GRAMD1A	-0.044	0.201253257	0.482814149
OR52W1	-0.044	0.201230691	0.482814149
DENND3	-0.044	0.201217394	0.482814149
RP11-529E10	-0.044	0.201213923	0.482814149
RNU4-47P	-0.044	0.201195586	0.482814149
RP11-660L16	-0.044	0.20112313	0.482734027
ING2	-0.044	0.201105013	0.48271487
CCDC168	-0.044	0.201085269	0.482691805
OGFOD1	-0.044	0.20107572	0.482691805
ASAH1	-0.044	0.201065893	0.482691805
SCFD1	-0.044	0.20103699	0.482673222
CSH1	-0.044	0.201024675	0.482673222
C2CD2L	-0.044	0.20100886	0.482673222
AJ239318	-0.044	0.200918963	0.48251147
RP11-640A1	-0.044	0.200905695	0.48251147
TMEM198B	-0.044	0.200897912	0.48251147
MIR1285-1	-0.044	0.200867675	0.482485614
C5orf66	-0.044	0.200850542	0.482478948
RP11-266O15	-0.044	0.200846656	0.482478948
ZNF577	-0.044	0.200649982	0.482157249
CTC-563A5	-0.044	0.200634557	0.482144511
STAT5B	-0.044	0.200580532	0.482063791
AC092755	-0.044	0.200514113	0.481952342
RHBDF2	-0.044	0.200511099	0.481952342
RP11-498M15	-0.044	0.200481756	0.481923213
PTER	-0.044	0.200475524	0.481923213
SLC38A9	-0.044	0.200451298	0.481922966

RNU6-742P	-0.044	0.200440877	0.481922241
VTRNA1-3	-0.044	0.2004163	0.481887476
C10orf113	-0.044	0.200415482	0.481887476
ZWILCH	-0.044	0.200359986	0.481822263
IQCF5	-0.044	0.20032382	0.481786728
MIR4296	-0.044	0.200312413	0.481783621
PRAMEF7	-0.044	0.200303234	0.481783621
MIR640	-0.044	0.200183373	0.481570538
RNU1-92P	-0.044	0.200164704	0.481570538
CENPO	-0.044	0.200122525	0.481521445
MC2R	-0.044	0.200065636	0.481408885
OR2J3	-0.044	0.200057924	0.481408885
ATP5J2P6	-0.044	0.200006457	0.481316006
C6orf3	-0.044	0.199974696	0.481287322
CTA-414D7	-0.044	0.199941562	0.481231896
DTD2	-0.044	0.199938614	0.481231896
RP11-84H6	-0.044	0.199914989	0.481231896
GSKIP	-0.044	0.199883734	0.481214305
SRSF9	-0.044	0.199873757	0.481214305
SNORA70B	-0.044	0.19984711	0.481214305
SH2D6	-0.044	0.199672928	0.480901267
RP13-24G8	-0.044	0.199631296	0.48087395
KRT25	-0.044	0.199616387	0.480866114
KCTD2	-0.044	0.199602404	0.480866114
AC026188	-0.044	0.199596069	0.480866114
RNA5SP56	-0.044	0.199564856	0.480835502
CCDC93	-0.044	0.199562347	0.480835502
RP11-395I14	-0.044	0.199507272	0.480745401
PAOX	-0.044	0.199497579	0.480745401
TEX101	-0.044	0.199454866	0.480692081
FLVCR1-AS1	-0.044	0.199448534	0.480692081
KRT35	-0.044	0.199446296	0.480692081
MIR505	-0.044	0.199430921	0.480692081
RNU6-400P	-0.044	0.19941204	0.480686164
RP11-89F3	-0.044	0.199347001	0.480553707
MIR4762	-0.044	0.199226472	0.48036041
LSM14B	-0.044	0.199190425	0.480297811
CTA-254O6	-0.044	0.199178282	0.480292847
ASB15	-0.044	0.199169864	0.480292847
RP11-348B17	-0.044	0.19910627	0.480265097
PRKRIRP8	-0.044	0.199096537	0.480265097
AC004257	-0.044	0.199082902	0.480257376
ETNPPL	-0.044	0.198990846	0.480059621
PGBD2	-0.044	0.198971951	0.480038352
Z97634	-0.044	0.198956844	0.480026221
KRT8P15	-0.044	0.198901878	0.479990866
DKFZP434K028	-0.044	0.198838368	0.479886235
AC137488	-0.044	0.198830278	0.479886235
TROAP	-0.044	0.198781741	0.479798196
LINC01225	-0.044	0.198778572	0.479798196
OR4D6	-0.044	0.198746933	0.479787124
PRINS	-0.044	0.198728688	0.479767399

TPP1	-0.044	0.198621457	0.479581452
TMEM252	-0.044	0.198617429	0.479581452
TRIM60P16	-0.044	0.19860163	0.479581452
HNRNPA1P76	-0.044	0.198596684	0.479581452
LRRC73	-0.044	0.198525217	0.47949493
UBA5	-0.044	0.198446379	0.479328822
RBM44	-0.044	0.198427866	0.479308414
AC016764	-0.044	0.198406007	0.47929446
LRP2BP	-0.044	0.198390271	0.479290528
MIR1537	-0.044	0.198385983	0.479290528
LPA	-0.044	0.198324932	0.479205597
TBC1D2B	-0.044	0.198290108	0.479170072
OR52K2	-0.044	0.1982306	0.479050578
AC004006	-0.044	0.198178508	0.478997604
RP4-631H13	-0.044	0.19808905	0.478805683
PRR13P3	-0.044	0.198058449	0.478756013
POLDIP2	-0.044	0.198049674	0.478756013
DCAKD	-0.044	0.198046434	0.478756013
CIDEB	-0.044	0.197999744	0.478711294
RP11-428C6	-0.044	0.197933652	0.478648696
FAM13C	-0.044	0.197867528	0.478585992
DNAJB1P1	-0.044	0.197867171	0.478585992
GRTP1-AS1	-0.044	0.197807508	0.478513724
RPS2P45	-0.044	0.197781694	0.478498515
KMT2C	-0.044	0.19775462	0.478473738
AC006926	-0.044	0.197719643	0.478422671
AC068056	-0.044	0.197655663	0.478316461
TLK2P1	-0.044	0.197591135	0.478257508
DNAJC19P5	-0.044	0.197415467	0.4778566
MED12	-0.044	0.197389343	0.477817648
CYP2D8P	-0.044	0.197329549	0.477747485
RNA5SP470	-0.044	0.19731489	0.477747485
RNU6-431P	-0.044	0.197311244	0.477747485
RP11-386I8	-0.044	0.197284445	0.477747485
AC114808	-0.044	0.19725545	0.477747485
ZNF735	-0.044	0.197238953	0.477744984
ZNF526	-0.044	0.197238049	0.477744984
VAMP1	-0.044	0.197179532	0.477673934
RP11-480G3	-0.044	0.197164424	0.477673934
SNORA70G	-0.044	0.197163936	0.477673934
RP11-485M7	-0.044	0.197152748	0.477673934
ATXN1L	-0.044	0.197125775	0.477673934
FAM8A2P	-0.044	0.197095242	0.477673934
RNU6-211P	-0.044	0.197024351	0.477613821
MT-TV	-0.044	0.196971417	0.477558424
HIST1H2APS2	-0.044	0.19695858	0.477551611
C1orf64	-0.044	0.196941906	0.477535494
RNU6-612P	-0.044	0.196858961	0.477358675
SULT1C2P1	-0.044	0.196858739	0.477358675
SPEF2	-0.044	0.196826794	0.477329279
AL450992	-0.044	0.196810857	0.477314936
MRPS31P1	-0.044	0.196806882	0.477314936

DLGAP2-AS1	-0.044	0.196748365	0.477211977
PSMC1P3	-0.044	0.196731446	0.477195241
ZNF418	-0.044	0.196730621	0.477195241
RNA5SP211	-0.044	0.196714818	0.477195241
MLYCD	-0.044	0.196689848	0.477191553
LINC01521	-0.044	0.196619316	0.477069038
RP4-591N18	-0.044	0.196582241	0.477003383
PARP1	-0.044	0.196568893	0.477003383
AC008674	-0.044	0.196561937	0.477003383
EPHA1-AS1	-0.044	0.196514251	0.4769356
RP11-1079H9	-0.044	0.19650982	0.4769356
ZNHIT1P1	-0.044	0.196465746	0.47686648
HMGN1	-0.044	0.196459928	0.47686648
RP5-921G16	-0.044	0.196451142	0.47686648
CTB-180A7	-0.044	0.196377794	0.476750189
AC099506	-0.044	0.196361719	0.476735463
RNU6-743P	-0.044	0.196283905	0.476595134
CTD-2183H9	-0.044	0.196273943	0.476595134
KRTAP26-1	-0.044	0.196266381	0.476595134
MIR1289-1	-0.044	0.196257909	0.476595134
PPIAP31	-0.044	0.196237925	0.476595134
WASL	-0.044	0.196192347	0.476518584
PABPC3	-0.044	0.196171906	0.476517538
ZC2HC1B	-0.044	0.196167454	0.476517538
MIR875	-0.044	0.196155542	0.476517538
GPM6A	-0.044	0.196129994	0.476512943
MAB21L2	-0.044	0.196046517	0.476407341
RP11-145M4	-0.044	0.195982617	0.476300663
SDHB	-0.044	0.195980441	0.476300663
ZNF444P1	-0.044	0.195960967	0.476300663
MIR587	-0.044	0.195959916	0.476300663
RP11-293D9	-0.044	0.19594301	0.476300663
RP11-633G11	-0.044	0.195932627	0.476300663
RP11-99C10	-0.044	0.195901133	0.476272754
PKD1L3	-0.044	0.195824348	0.476134685
HNRNPA1P52	-0.044	0.195823194	0.476134685
RNU6-867P	-0.044	0.195710153	0.475905619
RNU7-38P	-0.044	0.19570336	0.475905619
CUL1	-0.044	0.195691422	0.475905619
TM4SF5	-0.044	0.195642176	0.4758618
MTX3	-0.044	0.195511061	0.475695864
GTF2IP1	-0.044	0.195478024	0.475656867
RP11-206B11	-0.044	0.19541958	0.475563248
RN7SKP190	-0.044	0.195378961	0.475488697
RPL35AP12	-0.044	0.195377463	0.475488697
APEX1	-0.044	0.195357426	0.475488697
LL0XNC01-131B10	-0.044	0.195351395	0.475488697
SNURFL	-0.044	0.195330916	0.475488697
ADAMTS7P1	-0.044	0.1953239	0.475488697
PARP16	-0.044	0.195215383	0.475309188
YY1P2	-0.044	0.195165517	0.475222525
HSPD1P6	-0.044	0.195052862	0.475034909

RANP4	-0.044	0.195020548	0.474980498
TTBK2	-0.044	0.194990682	0.474932046
SERTAD4	-0.044	0.194976551	0.474921914
HERC4	-0.044	0.194923046	0.474864445
RPS2P25	-0.044	0.194905405	0.474864445
ARHGEF39	-0.044	0.194903832	0.474864445
MEN1	-0.044	0.194897992	0.474864445
DDX27	-0.044	0.194872188	0.474861995
ST13P2	-0.044	0.194865525	0.474861995
ACADM	-0.044	0.194818327	0.474779331
RP11-25C19	-0.044	0.194804578	0.474770117
MIR3687-2	-0.044	0.19478986	0.474758542
DUT	-0.044	0.194750546	0.474735605
RP11-72B4	-0.044	0.194739773	0.474735605
C10orf126	-0.044	0.194677527	0.474630488
EEF1A1P15	-0.044	0.194631981	0.474556695
LYRM9	-0.044	0.194555018	0.474428951
AC098820	-0.044	0.194548998	0.474428951
AC011193	-0.044	0.194546891	0.474428951
CCNYL3	-0.044	0.194491239	0.474370575
RP11-552D8	-0.044	0.194441261	0.474272966
RP11-255B23	-0.044	0.194429458	0.474268466
XAGE5	-0.044	0.194416936	0.474262211
HIST2H4A	-0.044	0.194373653	0.474205202
EIF5AL1	-0.044	0.194322793	0.474111748
RNU6-64P	-0.044	0.19432125	0.474111748
OR6X1	-0.044	0.194282228	0.474079295
CTD-2313P7	-0.044	0.194192592	0.473884848
CELF6	-0.044	0.194149847	0.473804815
AC114763	-0.044	0.194149319	0.473804815
RP11-566E18	-0.044	0.194146362	0.473804815
LRRIQ3	-0.044	0.194096216	0.473787173
RP11-803P9	-0.044	0.19409127	0.473787173
BTBD9	-0.044	0.194088517	0.473787173
DCAF8L1	-0.044	0.194083039	0.473787173
ERVW-1	-0.044	0.194076989	0.473787173
ARHGAP42P4	-0.044	0.194032914	0.473786495
PRTN3	-0.044	0.194000654	0.473732011
RP11-366L5	-0.044	0.193993304	0.473732011
AC078881	-0.044	0.193945982	0.473647077
RP11-78B10	-0.044	0.193931729	0.473636555
ZIC3	-0.044	0.193931679	0.473636555
ZFYVE27	-0.044	0.193920875	0.473636555
AC005033	-0.044	0.193900967	0.473636555
RP4-798A10	-0.044	0.193899106	0.473636555
OR1M4P	-0.044	0.193874652	0.473636555
SNORA11	-0.044	0.193873023	0.473636555
ZNF654	-0.044	0.193870021	0.473636555
RNU6-155P	-0.044	0.193776504	0.473562453
AC145343	-0.044	0.193755027	0.473544961
LINC00706	-0.044	0.193732894	0.473539467
MIR4645	-0.044	0.193730875	0.473539467

LINC01391	-0.044	0.193717495	0.473539467
ELL2	-0.044	0.193673454	0.473491381
NUS1P4	-0.044	0.193659126	0.473491381
CRYGEP	-0.044	0.193520304	0.47318983
DERL1	-0.044	0.19347516	0.473128026
AC105393	-0.044	0.193456129	0.473128026
TXNP2	-0.044	0.193453713	0.473128026
AGER	-0.044	0.193430573	0.473128026
MIR3937	-0.044	0.193430162	0.473128026
OR8U1	-0.044	0.193429338	0.473128026
RP11-453N18	-0.044	0.193418553	0.473128026
GAGE12C	-0.044	0.193412271	0.473128026
MTND1P24	-0.044	0.19337523	0.473128026
SUCO	-0.044	0.193371386	0.473128026
CDKN3	-0.044	0.193336555	0.473128026
SLMO1	-0.044	0.19333075	0.473128026
RP11-204C16	-0.044	0.193328711	0.473128026
RP11-798M19	-0.044	0.193303974	0.47312238
RNU6-30P	-0.044	0.193272752	0.473070271
RNU5B-2P	-0.044	0.19312373	0.472788806
RP11-297P16	-0.044	0.193068209	0.472715381
RNU2-13P	-0.044	0.193053072	0.47270262
RP11-169K17	-0.044	0.193035908	0.47270262
RP11-164N3	-0.044	0.193024231	0.47270262
SNORA5A	-0.044	0.192951755	0.472648935
CSF3R	-0.044	0.192928094	0.472615282
CYCSP30	-0.044	0.192836395	0.472414947
RP3-466P17	-0.044	0.192745971	0.472266301
AC104457	-0.044	0.192730489	0.472266301
LINC01413	-0.044	0.19271414	0.472261195
RP11-231E19	-0.044	0.192632902	0.472134996
HMGB3P10	-0.044	0.192632619	0.472134996
TTN-AS1	-0.044	0.192620903	0.472134996
ERP29P1	-0.044	0.19260319	0.472134996
AC024132	-0.044	0.192584278	0.472134996
RP4-536B24	-0.044	0.192555814	0.472116127
TULP3P1	-0.044	0.192537192	0.472094771
RN7SL262P	-0.044	0.192516476	0.472068279
ABHD14A-ACY1	-0.044	0.192511927	0.472068279
RP13-1056D16	-0.044	0.19248997	0.472051893
RP11-1324A7	-0.044	0.192465118	0.472015251
AZU1	-0.044	0.192459304	0.472015251
RP1-161P9	-0.044	0.192333986	0.471766532
RN7SL387P	-0.044	0.192320635	0.471766532
HDAC11	-0.044	0.192270614	0.471708265
RP11-240G22	-0.044	0.192265605	0.471708265
CEP63	-0.044	0.192255508	0.471708265
ZNF836	-0.044	0.192164146	0.471568517
FOXN3-AS2	-0.044	0.19216363	0.471568517
SNX29P2	-0.044	0.192100149	0.471469385
PRPF38A	-0.044	0.192099466	0.471469385
CTC-229L21	-0.044	0.192076698	0.471469385

RNU6-329P	-0.044	0.192060732	0.471460493
RP11-474P12	-0.044	0.192046541	0.471449957
RP11-31L22	-0.044	0.191822097	0.470996083
GRIA1	-0.044	0.191814768	0.470996083
LINC00529	-0.044	0.1918123	0.470996083
RP11-85I21	-0.044	0.191795749	0.470996083
TDPX2	-0.044	0.191757339	0.470948837
RP11-242C24	-0.044	0.191739504	0.470938958
HNRNPA1P17	-0.044	0.191724677	0.470926828
NDUFA5P11	-0.044	0.191696041	0.470905063
FBXO42	-0.044	0.191661292	0.470843986
OR7A17	-0.044	0.191641217	0.470839477
LINC00276	-0.044	0.191608289	0.470786627
IFI35	-0.044	0.191598038	0.470785729
CTC-558O19	-0.044	0.191524713	0.470629838
OR2W2P	-0.044	0.191514018	0.470629838
AGA	-0.044	0.191484406	0.470629838
RNU5F-3P	-0.044	0.191443336	0.470629838
AUH	-0.044	0.191425099	0.470627875
E2F3P2	-0.044	0.191296882	0.470480997
CEP55	-0.045	0.191256176	0.470431125
RP11-102N12	-0.045	0.191248002	0.470431125
LRRFIP2	-0.045	0.191240385	0.470431125
RNU6-1215P	-0.045	0.19117575	0.470330463
TOB2	-0.045	0.191126909	0.470258888
ACTR3BP5	-0.045	0.191101449	0.470220537
IPPK	-0.045	0.1911011	0.470220537
COG2	-0.045	0.191085505	0.470220537
RP11-359K18	-0.045	0.191016635	0.470084704
MIR548AM	-0.045	0.190977563	0.470025027
MIR4435-1	-0.045	0.190940913	0.469971208
CTC-224D3	-0.045	0.190940815	0.469971208
TAB3	-0.045	0.19088749	0.469888281
MAPK14	-0.045	0.190878771	0.469888281
CORO2A	-0.045	0.190840374	0.469861965
ZFP91-CNTF	-0.045	0.190829613	0.469861965
PIGN	-0.045	0.190751731	0.469724042
CCNF	-0.045	0.190705938	0.469635558
ATG4D	-0.045	0.190695927	0.46963519
HNRNPU	-0.045	0.190693147	0.46963519
RP5-1174J21	-0.045	0.190687662	0.46963519
AC110086	-0.045	0.190678548	0.46963519
USP9YP9	-0.045	0.190678548	0.46963519
CTD-2254N19	-0.045	0.190672961	0.46963519
RPL29P33	-0.045	0.190647886	0.46963519
PLCG1-AS1	-0.045	0.190600868	0.469571051
TOMM20P3	-0.045	0.19058457	0.469555187
TMBIM6	-0.045	0.190570274	0.469544255
MIR4714	-0.045	0.190438097	0.469291419
KRT26	-0.045	0.190412777	0.469277584
RSPO3	-0.045	0.19040672	0.469277584
AC090453	-0.045	0.190401141	0.469277584

RP1-101D8	-0.045	0.190391189	0.469277584
RP11-621H8	-0.045	0.190384683	0.469277584
AP001136	-0.045	0.190292897	0.469127792
MIR6749	-0.045	0.190234886	0.469033333
KIAA1257	-0.045	0.190232721	0.469033333
RNU6-360P	-0.045	0.190148216	0.468868189
MIA2	-0.045	0.190145187	0.468868189
RNU6-921P	-0.045	0.190141973	0.468868189
EEF1A1P34	-0.045	0.190091911	0.468802166
AC093700	-0.045	0.190055988	0.468737846
RP11-483P21	-0.045	0.190037389	0.468716247
BTF3P13	-0.045	0.190013239	0.468680953
RP11-1101H11	-0.045	0.189955775	0.46856348
RP11-773H22	-0.045	0.189935212	0.468537025
IDI2-AS1	-0.045	0.189911567	0.468502964
MIR107	-0.045	0.189886324	0.468464956
RP11-302K17	-0.045	0.189883783	0.468464956
OR6K4P	-0.045	0.189860202	0.468464956
AC106801	-0.045	0.189843439	0.468458188
PDX1	-0.045	0.189836667	0.468458188
ZNF816-ZNF321P	-0.045	0.189791954	0.468401979
RNU6-110P	-0.045	0.189789986	0.468401979
GTF2IP3	-0.045	0.189772102	0.468401979
DCUN1D4	-0.045	0.189767202	0.468401979
RP11-354M20	-0.045	0.189729681	0.46839392
ACTN4P1	-0.045	0.189692168	0.468325587
NOS1	-0.045	0.189691096	0.468325587
RP11-419L20	-0.045	0.189690155	0.468325587
ATF1	-0.045	0.189680403	0.468325587
UBXN10	-0.045	0.189649014	0.468325587
AL607148	-0.045	0.18964004	0.468325587
RP11-334E15	-0.045	0.189617011	0.468309966
ASNSD1	-0.045	0.189601385	0.468309966
RP11-12K6	-0.045	0.189580081	0.46829161
AP001468	-0.045	0.189578216	0.46829161
RP11-188C12	-0.045	0.189553027	0.468273356
AC114798	-0.045	0.189527481	0.468234534
RP11-537I16	-0.045	0.189473212	0.468173316
AC006128	-0.045	0.189428113	0.468086164
KIAA1598	-0.045	0.18942719	0.468086164
AP001178	-0.045	0.18939886	0.468062448
RP11-1110F20	-0.045	0.189307131	0.46797058
LINC01122	-0.045	0.189270254	0.46793887
CTD-2245F17	-0.045	0.189270163	0.46793887
NME6	-0.045	0.189246292	0.467928209
LA16c-13E4	-0.045	0.189245794	0.467928209
ST7-AS2	-0.045	0.189226155	0.467927003
STPG1	-0.045	0.189224173	0.467927003
RP11-460N20	-0.045	0.189209427	0.467927003
SCEL-AS1	-0.045	0.189203607	0.467927003
AP3D1	-0.045	0.189202792	0.467927003
LL0XNC01-221F2	-0.045	0.189193385	0.467927003

SEC24A	-0.045	0.189190076	0.467927003
RP11-138E16	-0.045	0.189177655	0.467927003
CCBL1	-0.045	0.189046365	0.467725257
RP11-131K17	-0.045	0.189034248	0.467719576
AKAP1	-0.045	0.189027762	0.467719576
IFNL4P1	-0.045	0.188999425	0.467682009
MIR888	-0.045	0.18896225	0.467614315
NAAA	-0.045	0.188939002	0.467581078
RPSAP32	-0.045	0.188920793	0.467581078
C17orf100	-0.045	0.18890752	0.467581078
MIR603	-0.045	0.188889877	0.467580986
ADD1	-0.045	0.188877699	0.467575142
SNORD115-21	-0.045	0.188851285	0.467558358
RN7SL93P	-0.045	0.188836454	0.467545945
RP11-252E2	-0.045	0.188829914	0.467545945
RP11-19J3	-0.045	0.18882109	0.467545945
ARHGAP33	-0.045	0.188799296	0.467545945
SLC29A4P1	-0.045	0.18874801	0.467497086
RP11-68L1	-0.045	0.188726998	0.467469354
RNU6-607P	-0.045	0.188614204	0.467238568
POM121C	-0.045	0.188407089	0.46686109
RGPD2	-0.045	0.1883571	0.466795899
C9orf47	-0.045	0.188349677	0.466795899
RP11-345I18	-0.045	0.188239511	0.46657732
LYRM1	-0.045	0.188163573	0.466437646
EPHA4	-0.045	0.188161921	0.466437646
RN7SKP64	-0.045	0.188132617	0.466411259
RNF185	-0.045	0.188127598	0.466411259
C16orf87	-0.045	0.188063919	0.466311968
RN7SL614P	-0.045	0.188058937	0.466311968
OR51A1P	-0.045	0.188054168	0.466311968
TSPY16P	-0.045	0.187944474	0.466137138
OR2H5P	-0.045	0.187931581	0.466129435
SOCS5P4	-0.045	0.187930202	0.466129435
ARHGEF3-AS1	-0.045	0.187920307	0.466129435
ZNF252P-AS1	-0.045	0.187916071	0.466129435
RNU6-630P	-0.045	0.187822598	0.465980459
KDM7A	-0.045	0.187821646	0.465980459
AC011718	-0.045	0.187790211	0.465980459
PSMB7P1	-0.045	0.187789464	0.465980459
SMAD2	-0.045	0.187780954	0.465980459
GS1-410F4	-0.045	0.187777997	0.465980459
C10orf54	-0.045	0.187744116	0.465980459
GPR152	-0.045	0.187729819	0.465980459
OR52B1P	-0.045	0.187721361	0.465980459
AL021918	-0.045	0.187670182	0.465917836
RNU6-1074P	-0.045	0.187652516	0.465907784
IL31	-0.045	0.187650339	0.465907784
WDR95P	-0.045	0.187629877	0.465907784
CTD-2653M23	-0.045	0.18760069	0.465907784
LINC01538	-0.045	0.187576164	0.465902996
NRDE2	-0.045	0.187564403	0.465902996

RP11-685B13	-0.045	0.187497748	0.465829695
RP11-29B11	-0.045	0.187493903	0.465829695
RP11-141P6	-0.045	0.187462088	0.465813996
ADAM3A	-0.045	0.187438142	0.465778796
RP11-741G21	-0.045	0.187421862	0.465762641
ACTL7A	-0.045	0.187420198	0.465762641
RP11-68D16	-0.045	0.187379067	0.465729195
CTC-479C5	-0.045	0.187332668	0.465664854
MINOS1P2	-0.045	0.187247264	0.465498769
LINC01076	-0.045	0.187190454	0.465381831
POTED	-0.045	0.187190165	0.465381831
CCDC132	-0.045	0.187096573	0.465221285
RNU6-1204P	-0.045	0.187071628	0.465207833
TKTL1	-0.045	0.186980439	0.465005345
AC021660	-0.045	0.186976567	0.465005345
RPL7P14	-0.045	0.186917726	0.464922213
CTD-2140B24	-0.045	0.186876647	0.464844314
RP11-142O6	-0.045	0.186868152	0.464844314
AC007362	-0.045	0.186859972	0.464844314
ZNF568	-0.045	0.18678985	0.464725498
PLEKHB1	-0.045	0.186782548	0.464725498
IGHVIV-44-1	-0.045	0.186767748	0.46471906
CLEC1A	-0.045	0.186732712	0.464680437
AK9	-0.045	0.186707889	0.464642943
RP11-432M8	-0.045	0.186675771	0.464611567
AANAT	-0.045	0.186666741	0.464611567
GPR135	-0.045	0.186594757	0.464458476
RP11-693I21	-0.045	0.186557658	0.464391555
RP11-333A23	-0.045	0.186519591	0.464344182
RNU6-520P	-0.045	0.186517744	0.464344182
RP11-194N12	-0.045	0.186500561	0.464344182
RP11-456K23	-0.045	0.186399814	0.46414304
RP11-255I10	-0.045	0.186390891	0.46414304
RP11-576C12	-0.045	0.186384628	0.46414304
MIR1226	-0.045	0.18634023	0.464116
SNORD116-2	-0.045	0.186305294	0.464074
AC064865	-0.045	0.186293444	0.464072277
MINPP1	-0.045	0.186272243	0.464043734
AC093844	-0.045	0.186261076	0.464043734
LIFR-AS1	-0.045	0.186255299	0.464043734
RNU6-544P	-0.045	0.186237372	0.464043734
RP4-561L24	-0.045	0.186177518	0.463929087
CD200	-0.045	0.186164587	0.463921138
AP000925	-0.045	0.186164544	0.463921138
TMEM176A	-0.045	0.186127983	0.463878465
FRZB	-0.045	0.186119607	0.463878465
RP11-674C21	-0.045	0.186085052	0.463820012
Z83844	-0.045	0.185886742	0.463422731
MOV10	-0.045	0.18588355	0.463422731
RP11-454E5	-0.045	0.185786607	0.463245836
CTD-3244O18	-0.045	0.185762326	0.463209543
AC018693	-0.045	0.185699345	0.463109578

MCM4	-0.045	0.185693154	0.463109578
RNU6-437P	-0.045	0.185690278	0.463109578
MIPEP	-0.045	0.185603553	0.463007564
CDV3P1	-0.045	0.185595796	0.463007564
NMUR1	-0.045	0.185580001	0.462997314
PTCRA	-0.045	0.185565711	0.462985915
RP11-756G20	-0.045	0.185529821	0.462920621
DSCC1	-0.045	0.185477656	0.462838956
VPS53	-0.045	0.185436067	0.462759423
TMEM154	-0.045	0.185396301	0.462732924
RP11-22M7	-0.045	0.185374102	0.462710362
TMEM131	-0.045	0.185311343	0.462679292
RP1-138A5	-0.045	0.185279639	0.462659957
AC004449	-0.045	0.185246746	0.462602075
RP11-379K22	-0.045	0.185223043	0.462567137
GTF2E1	-0.045	0.185217886	0.462567137
RAB27B	-0.045	0.185212987	0.462567137
RP13-487P22	-0.045	0.185212038	0.462567137
ZNF737	-0.045	0.185106872	0.462373996
MOB1AP1	-0.045	0.185081235	0.462358458
ZNF263	-0.045	0.185078401	0.462358458
RNU5F-4P	-0.045	0.185028182	0.462274419
SNRPCP3	-0.045	0.185023556	0.462274419
RP11-1114A5	-0.045	0.185010772	0.462274419
KCNG4	-0.045	0.184998442	0.462272865
AL357932	-0.045	0.184931809	0.462130608
NIP7P2	-0.045	0.184928934	0.462130608
LGALS2	-0.045	0.184922319	0.462130608
FP565210	-0.045	0.184919528	0.462130608
AF121897	-0.045	0.18486102	0.462050686
LINC00924	-0.045	0.18483193	0.46200222
XRCC6P2	-0.045	0.184799292	0.46196913
TAOK3	-0.045	0.184764887	0.461931615
OR10J9P	-0.045	0.184761644	0.461931615
AB015752	-0.045	0.184742701	0.461924644
LGALS3BP	-0.045	0.184710922	0.461924644
NUP85	-0.045	0.184707663	0.461924644
RP1-72E17	-0.045	0.184621217	0.461790595
H2AFZP3	-0.045	0.184571056	0.461689375
RNU6-1157P	-0.045	0.184568938	0.461689375
RN7SL535P	-0.045	0.184556661	0.461689375
RP11-84D1	-0.045	0.184554307	0.461689375
RP11-153N17	-0.045	0.184544325	0.461689375
HNRNPDLP4	-0.045	0.184507585	0.461689375
AC068014	-0.045	0.184504103	0.461689375
RP11-527D15	-0.045	0.184441645	0.461583849
CACNA1D	-0.045	0.184436572	0.461583849
RP11-854K16	-0.045	0.184426532	0.461583849
RP11-346C20	-0.045	0.184411251	0.461580548
AL589822	-0.045	0.184393624	0.461580548
AC018442	-0.045	0.184388122	0.461580548
RP11-64B16	-0.045	0.184384084	0.461580548

RP5-1050E16	-0.045	0.184376902	0.461580548
MIR3188	-0.045	0.184334959	0.461580548
RNU6-1274P	-0.045	0.184330416	0.461580548
MIR4312	-0.045	0.184218129	0.46136996
DIABLO	-0.045	0.184185469	0.461337999
RP11-526A4	-0.045	0.184167258	0.461333509
RP11-338N10	-0.045	0.184166554	0.461333509
MTND1P26	-0.045	0.184145167	0.4613267
HMGB1P47	-0.045	0.184132847	0.461320101
PKNOX1	-0.045	0.184113927	0.461296964
RP11-234G16	-0.045	0.184099304	0.461284594
RP11-685G9	-0.045	0.184097961	0.461284594
RGPD8	-0.045	0.184071119	0.461284594
LLNLF-187D8	-0.045	0.184057099	0.461275913
NPM1P41	-0.045	0.183995091	0.461193316
IPO7P1	-0.045	0.183965567	0.461151357
ABCA11P	-0.045	0.183924511	0.461089203
H3F3AP5	-0.045	0.183914763	0.461089035
MIR4655	-0.045	0.183881751	0.461054813
EIF1AXP2	-0.045	0.183876471	0.461054813
RP11-610P16	-0.045	0.183870848	0.461054813
Six3os1_2	-0.045	0.183852018	0.461053079
DNMT1	-0.045	0.183848532	0.461053079
EIF3L	-0.045	0.183821287	0.46104884
METTL21EP	-0.045	0.183811311	0.4610481
RP11-1136G11	-0.045	0.183800934	0.461046351
RNU6-438P	-0.045	0.183736916	0.460922126
RNA5SP495	-0.045	0.183728036	0.460922126
LRP5L	-0.045	0.183727484	0.460922126
OR5L1	-0.045	0.1836987	0.460922126
GABRB1	-0.045	0.183679728	0.460922126
NIFKP2	-0.045	0.183641225	0.460865486
RNU6-1156P	-0.045	0.183600766	0.460811218
KLHL18	-0.045	0.183577112	0.460776132
MIR6753	-0.045	0.183512445	0.460638095
CCNB2	-0.045	0.183494392	0.460638095
MIR181D	-0.045	0.183481368	0.460632918
RP11-438C19	-0.045	0.183453073	0.460586163
RP6-127F18	-0.045	0.183436608	0.460584222
NDUFAF4P3	-0.045	0.183388218	0.460569007
AC009310	-0.045	0.183385001	0.460569007
RP5-865N13	-0.045	0.18337704	0.460569007
MIR4502	-0.045	0.183297644	0.460462942
RNU6-817P	-0.045	0.183259069	0.460390318
ANKRD11	-0.045	0.183236319	0.460357448
SAG	-0.045	0.183162555	0.460239061
SSSCA1-AS1	-0.045	0.183126183	0.460177847
AC012513	-0.045	0.183075642	0.460099394
YES1P1	-0.045	0.183071688	0.460099394
RNU6-998P	-0.045	0.183054153	0.460099394
RBM22P1	-0.045	0.183020511	0.460057945
AC027119	-0.045	0.183008501	0.460057945

TAS2R19	-0.045	0.182980476	0.460053695
RP11-220I1	-0.045	0.182915076	0.459914282
AC013738	-0.045	0.182909505	0.459914282
ENPP7P7	-0.045	0.18287548	0.459863279
RBP7	-0.045	0.182786728	0.459724792
GNG5P5	-0.045	0.182724301	0.459604452
FAM157C	-0.045	0.18271121	0.459595797
RPL21P107	-0.045	0.1826885	0.459562942
APOOP3	-0.045	0.182667406	0.45954013
RP11-110A12	-0.045	0.182593996	0.459422276
HSPD1P11	-0.045	0.182544587	0.459360633
AL121588	-0.045	0.182497724	0.459325371
RP11-40B20	-0.045	0.182480165	0.459325371
RP11-429P3	-0.045	0.182440433	0.459302807
RP11-689P11	-0.045	0.182413471	0.45926869
ZNF827	-0.045	0.182385845	0.45926869
MFN2	-0.045	0.18233096	0.459193706
CAPN14	-0.045	0.182304648	0.459155129
RP11-463O9	-0.045	0.182289536	0.459144056
TRBV7-5	-0.045	0.182267392	0.459134135
ALDH3A2	-0.045	0.182253855	0.459124319
SNTB2	-0.045	0.182228578	0.459087532
RP5-1027O15	-0.045	0.182220388	0.459087532
RP11-989E6	-0.045	0.182152212	0.458997353
RPS23P7	-0.045	0.18213931	0.458997353
RNU1-76P	-0.045	0.182050019	0.458853527
AC138035	-0.045	0.182044312	0.458853527
LINC01191	-0.045	0.1820409	0.458853527
LINC00992	-0.045	0.182039476	0.458853527
VTI1BP3	-0.045	0.182031473	0.458853527
TMOD3	-0.045	0.182018567	0.458853527
NHLRC1	-0.045	0.182014268	0.458853527
RP11-248N22	-0.045	0.181988874	0.458853527
RP11-350D23	-0.045	0.181871455	0.458711711
AF064860	-0.045	0.181868862	0.458711711
DOCK9-AS1	-0.045	0.181858312	0.458711711
ZNRF2P3	-0.045	0.181855485	0.458711711
GINS2	-0.045	0.181810167	0.458661626
AC004987	-0.045	0.18180623	0.458661626
AC008810	-0.045	0.18180029	0.458661626
DDHD2	-0.045	0.181768652	0.45865407
ECHDC1	-0.045	0.181767936	0.45865407
ALDH3B2	-0.045	0.181721768	0.45860866
RP11-326I11	-0.045	0.181708538	0.458599573
OR5H3P	-0.045	0.181682272	0.458557582
RNU6-621P	-0.045	0.181631029	0.458452541
RP11-104J23	-0.045	0.181620861	0.458451175
AKAP11	-0.045	0.181565827	0.458360842
CTD-3098H1	-0.045	0.181564162	0.458360842
KRT18P28	-0.045	0.181541938	0.458360842
RNA5SP494	-0.046	0.181470674	0.458266373
RP11-24H2	-0.046	0.18146483	0.458266373

CCNI	-0.046	0.181447501	0.458266373
SYT2	-0.046	0.181414436	0.458245842
PPP2R2C	-0.046	0.181382813	0.458190264
AL121893	-0.046	0.181359059	0.458154559
RNASE8	-0.046	0.181349343	0.458154316
AL136529	-0.046	0.181334157	0.458140252
ZNF366	-0.046	0.181310292	0.458104259
DPP9	-0.046	0.181307621	0.458104259
STRIP2	-0.046	0.181302799	0.458104259
RP11-435I3	-0.046	0.181250079	0.458049322
RP11-218M22	-0.046	0.18124405	0.458049322
RNU6-218P	-0.046	0.181230208	0.458049322
STAG1	-0.046	0.181195668	0.458009026
ARPC3P1	-0.046	0.181159962	0.457943077
MZF1	-0.046	0.181118205	0.45793353
MARVELD2	-0.046	0.18110268	0.457919799
URB2	-0.046	0.181091081	0.457919799
MED6	-0.046	0.18104041	0.457859581
RP11-490E15	-0.046	0.181012479	0.457813251
HSPA4L	-0.046	0.180928579	0.457673967
AC007283	-0.046	0.180910264	0.457665163
HNRNPH1P1	-0.046	0.180898264	0.457665163
CTDSP2	-0.046	0.180897554	0.457665163
CTGLF8P	-0.046	0.180841751	0.457575841
RP11-23B7	-0.046	0.180815462	0.457533631
SIAH2	-0.046	0.180768858	0.457440009
CTPS2	-0.046	0.180756919	0.457434101
TERF1P5	-0.046	0.180732818	0.457397412
USP40	-0.046	0.180708634	0.457386213
XKR6	-0.046	0.180708625	0.457386213
AC106782	-0.046	0.180698728	0.457386213
RP11-117N2	-0.046	0.180690203	0.457386213
RP11-383C6	-0.046	0.18067798	0.457386213
RP1-182O16	-0.046	0.180660442	0.457386213
CTD-2124B8	-0.046	0.180660001	0.457386213
TMEM232	-0.046	0.180633728	0.457386213
AC005550	-0.046	0.180600078	0.457353109
RP11-60E8	-0.046	0.180509077	0.457219901
RPL30P16	-0.046	0.180504262	0.457219901
RP11-111E14	-0.046	0.180499724	0.457219901
C10orf62	-0.046	0.180467549	0.457211978
PSMB10	-0.046	0.180463256	0.457211978
ZNF678	-0.046	0.180430866	0.457192002
ACBD7	-0.046	0.180409422	0.457161987
EIF2B1	-0.046	0.180401505	0.457161987
CAB39	-0.046	0.180363253	0.45709838
CASD1	-0.046	0.180363075	0.45709838
NOTO	-0.046	0.180307042	0.457048458
CCNG1	-0.046	0.180170836	0.456807972
SLCO1C1	-0.046	0.180161257	0.456807972
NOLC1	-0.046	0.180132975	0.456807972
BLVRA	-0.046	0.18007966	0.45673948

VWC2L	-0.046	0.180019325	0.456655648
RP11-317G6	-0.046	0.18000104	0.456655648
NRBF2P3	-0.046	0.179996723	0.456655648
CCDC77	-0.046	0.179985143	0.456655648
CNTLN	-0.046	0.17997342	0.456655648
SLC26A5	-0.046	0.179943554	0.456655648
OR52J3	-0.046	0.179927673	0.456655648
ZNF285B	-0.046	0.179880439	0.456622175
AC087380	-0.046	0.179827655	0.456555921
CCDC88B	-0.046	0.179815035	0.456555921
RP11-56N19	-0.046	0.179802493	0.456555921
CNTN5	-0.046	0.179723339	0.456443422
EIF4EP1	-0.046	0.179678769	0.456354562
NPAP1P7	-0.046	0.179642462	0.456330638
RP4-734P14	-0.046	0.179617099	0.456319602
SLC25A51	-0.046	0.179604784	0.456312655
DDX51	-0.046	0.179594143	0.456309963
RP11-361A23	-0.046	0.179593704	0.456309963
ADIRF	-0.046	0.179577691	0.456309963
RP11-554L12	-0.046	0.179474142	0.456078058
MIR561	-0.046	0.179469136	0.456078058
CTA-125H2	-0.046	0.179468497	0.456078058
KIF9	-0.046	0.179448589	0.456078058
KRT37	-0.046	0.179384325	0.456066601
KIAA1467	-0.046	0.179373276	0.456065076
JMY	-0.046	0.17934794	0.456025004
RP11-1113L8	-0.046	0.179341558	0.456025004
RP11-936I5	-0.046	0.179285818	0.455979048
RSAD2	-0.046	0.179232104	0.455876499
RP11-181C21	-0.046	0.179224753	0.455876499
VTI1BP1	-0.046	0.179211952	0.455873933
OR2G2	-0.046	0.179207514	0.455873933
CTGLF12P	-0.046	0.179158799	0.455787412
CTD-2342J14	-0.046	0.179086237	0.455681686
RP11-96A15	-0.046	0.179086196	0.455681686
RP5-888M10	-0.046	0.179042972	0.455638772
RP11-450H6	-0.046	0.179011934	0.455608476
ZSWIM8	-0.046	0.178910924	0.455375726
ZBTB8OSP1	-0.046	0.178896802	0.455375726
METTL6	-0.046	0.178878388	0.455375726
RP11-109I13	-0.046	0.17883391	0.455325704
RPL5P10	-0.046	0.178797423	0.455257144
RP11-585K6	-0.046	0.178786038	0.455252494
METTL8P1	-0.046	0.178784057	0.455252494
RBMY2WP	-0.046	0.178727702	0.455152619
AL031653	-0.046	0.178727702	0.455152619
RP11-434D11	-0.046	0.178703256	0.455152619
AC009961	-0.046	0.178618783	0.455055524
RP11-429O1	-0.046	0.178610933	0.455055524
RAPGEFL1	-0.046	0.178606773	0.455055524
FAM151B	-0.046	0.17860377	0.455055524
MIR578	-0.046	0.178596457	0.455055524

ATP11A-AS1	-0.046	0.178588734	0.455055524
ZFP2	-0.046	0.178578869	0.455055524
LINC01470	-0.046	0.178562647	0.455055524
RP11-462B18	-0.046	0.178544142	0.455055524
AC004655	-0.046	0.178520457	0.455055524
TTC28-AS1	-0.046	0.178496589	0.455050692
ZNF534	-0.046	0.178482722	0.455039698
YTHDF3	-0.046	0.178475758	0.455039698
ANXA9	-0.046	0.178456871	0.455028456
PTPRJ-AS1	-0.046	0.178410318	0.454989594
LMBRD2	-0.046	0.178385043	0.454961147
DSCR9	-0.046	0.178370564	0.45494858
KIF23	-0.046	0.178368104	0.45494858
CTC-239J10	-0.046	0.178352107	0.45494858
TSTD2	-0.046	0.178347991	0.45494858
TAF15	-0.046	0.178337582	0.45494858
CXXC1	-0.046	0.178321528	0.45494858
TNR	-0.046	0.178282595	0.45494858
RPL34P23	-0.046	0.178234967	0.454894122
COQ4	-0.046	0.178226816	0.454894122
KRT8P1	-0.046	0.178179226	0.454821751
RP11-539G18	-0.046	0.178174625	0.454821751
IFNAR2	-0.046	0.17817392	0.454821751
RP11-498P14	-0.046	0.178163695	0.454821751
RPS24P12	-0.046	0.178069788	0.454712056
RP11-211A18	-0.046	0.178032228	0.454645627
ACP2	-0.046	0.178028869	0.454645627
CDKN2D	-0.046	0.178010898	0.45463991
RNA5SP152	-0.046	0.177994902	0.454623434
HMGN3P1	-0.046	0.177966207	0.45459437
MIR4503	-0.046	0.177928301	0.454526453
RPL34P22	-0.046	0.177924313	0.454526453
RNU6-450P	-0.046	0.17789302	0.454508911
CCDC163P	-0.046	0.177885667	0.454508911
RP11-19H6	-0.046	0.17788314	0.454508911
FMO5	-0.046	0.177822284	0.454426244
PGM5P4	-0.046	0.177816817	0.454426244
AL139821	-0.046	0.177815303	0.454426244
RBMXP2	-0.046	0.177806244	0.454426244
MIR4441	-0.046	0.177717003	0.454254692
RP11-135M8__A	-0.046	0.17768916	0.454207902
AL845472	-0.046	0.177687466	0.454207902
CKS1BP3	-0.046	0.177667004	0.454200021
AC083884	-0.046	0.177643657	0.454181384
MAOA	-0.046	0.177613942	0.454161882
DLGAP1	-0.046	0.177603651	0.454161882
RNU6-1329P	-0.046	0.177504452	0.453979409
RP11-22P6	-0.046	0.177488693	0.453963481
RP5-1012F16	-0.046	0.177447873	0.453907827
RP11-24H1	-0.046	0.177400545	0.453835514
RP11-56H7	-0.046	0.177283587	0.453628348
RN7SL396P	-0.046	0.177269815	0.453628348

KLRF2	-0.046	0.177268785	0.453628348
CTC-499J9	-0.046	0.177259216	0.453628348
RNA5SP173	-0.046	0.17723026	0.453619173
FAM65C	-0.046	0.177205177	0.45357935
AL592063	-0.046	0.177057789	0.453250811
CDK5RAP1	-0.046	0.177033164	0.453212133
CTD-2113L7	-0.046	0.177031427	0.453212133
RP11-905K4	-0.046	0.177013186	0.45320971
RAD18	-0.046	0.176963517	0.453106898
RNU6-25P	-0.046	0.176865116	0.452903645
DEFB105A	-0.046	0.176851377	0.452892814
LIG1	-0.046	0.176808331	0.452831274
TUBB8P1	-0.046	0.176798928	0.452831274
PPP1R11P1	-0.046	0.176777091	0.452799963
RNU6-499P	-0.046	0.176746403	0.452745708
AZGP1P2	-0.046	0.176740519	0.452745708
CST5	-0.046	0.176673218	0.452631272
RNU6-1272P	-0.046	0.176662909	0.452629208
PTCHD3	-0.046	0.176655295	0.452629208
RNU2-14P	-0.046	0.176622524	0.452598781
RP11-323A7	-0.046	0.176612571	0.452598781
RP3-452M16	-0.046	0.176545915	0.452499853
RP11-368I7	-0.046	0.176537481	0.452499853
STAT6	-0.046	0.176501239	0.452458394
THOC2	-0.046	0.176469904	0.45242677
AL117209	-0.046	0.176459841	0.452425327
TRGJ2	-0.046	0.176413112	0.452354221
ZBTB12	-0.046	0.176330784	0.452204222
RP11-517C16	-0.046	0.176326403	0.452204222
RCOR1	-0.046	0.176305419	0.452199806
YWHAB	-0.046	0.176304719	0.452199806
RP11-36B15	-0.046	0.176290308	0.452199806
IK	-0.046	0.176287403	0.452199806
TMEM251	-0.046	0.176269213	0.452199806
TRMT5	-0.046	0.176234761	0.452164679
ZNF485	-0.046	0.17622217	0.452156734
OR4K1	-0.046	0.176213985	0.452156734
AC016629	-0.046	0.176181266	0.452124852
ALPP	-0.046	0.176178634	0.452124852
SPATA3-AS1	-0.046	0.176152874	0.452101767
MIR6857	-0.046	0.176149172	0.452101767
YWHAZP2	-0.046	0.176143251	0.452101767
AC092421	-0.046	0.176099697	0.452101767
RP11-217C7	-0.046	0.176090235	0.452101767
TTC3	-0.046	0.176047024	0.452053941
RP11-137J7	-0.046	0.1760053	0.452014257
SPACA7	-0.046	0.176001456	0.452014257
AC011748	-0.046	0.175989093	0.452014257
RP11-463O12	-0.046	0.175979761	0.452014257
HEXIM2	-0.046	0.175917583	0.451977079
SLC46A3	-0.046	0.17591147	0.451977079
GLIPR1L2	-0.046	0.175827138	0.451824706

AC002070	-0.046	0.175808435	0.451801021
RNY4P17	-0.046	0.175782398	0.451801021
EPM2A	-0.046	0.175747024	0.451765086
RP11-270B14	-0.046	0.175739592	0.451765086
RP11-900F13	-0.046	0.175720295	0.451745142
RP11-177N22	-0.046	0.175696259	0.451707733
HEATR5A	-0.046	0.175693281	0.451707733
TMEM210	-0.046	0.17566832	0.451684665
RP11-87C12	-0.046	0.175667348	0.451684665
HSF2	-0.046	0.175647051	0.451678748
AC026954	-0.046	0.175622386	0.451639707
MFF	-0.046	0.175621759	0.451639707
MBTPS2	-0.046	0.175611294	0.451639707
PGAM1P6	-0.046	0.175610215	0.451639707
RN7SL330P	-0.046	0.175585331	0.451639707
RP11-751L19	-0.046	0.175580643	0.451639707
SEC24B-AS1	-0.046	0.175580077	0.451639707
EIF4A2P4	-0.046	0.175535414	0.451601919
LCN6	-0.046	0.175473504	0.451509599
RP11-550C4	-0.046	0.175467027	0.451509599
ATP2A2	-0.046	0.1754549	0.451509599
RP11-219H23	-0.046	0.175411715	0.451487989
PRDM2	-0.046	0.175407148	0.451487989
ZNF114P1	-0.046	0.175398758	0.451487989
PRR13P4	-0.046	0.175390182	0.451487989
LA16c-23H5	-0.046	0.175383569	0.451487989
RP11-65I12	-0.046	0.175372176	0.451487989
RNU6-922P	-0.046	0.175332898	0.45146181
LIMD2	-0.046	0.175320209	0.45146181
RP11-553P9	-0.046	0.175319529	0.45146181
RP11-8P11	-0.046	0.175295927	0.45146181
RP11-1023L17	-0.046	0.175293558	0.45146181
EEF1E1-BLOC1S5	-0.046	0.175262214	0.45146181
C4BPAP1	-0.046	0.175143618	0.451310127
LINC01040	-0.046	0.175106319	0.451238431
DCP1B	-0.046	0.17509282	0.451228058
MAFG-AS1	-0.046	0.175083423	0.451228058
RNA5SP375	-0.046	0.175048384	0.451162366
RPL21P13	-0.046	0.175035952	0.451154741
RP1-234P15	-0.046	0.175012434	0.451118536
IGHVIII-13-1	-0.046	0.174991136	0.45108805
WDR82	-0.046	0.174944596	0.451016904
MTRR	-0.046	0.174940596	0.451016904
RP11-22K10	-0.046	0.174937965	0.451016904
RP11-349J5	-0.046	0.174909658	0.451000068
AC003101	-0.046	0.174909464	0.451000068
DPYD	-0.046	0.174904599	0.451000068
SNORD36	-0.046	0.174870337	0.450971926
HK2P1	-0.046	0.174835964	0.450907697
SMIM18	-0.046	0.174794373	0.450824845
OR52J2P	-0.046	0.174762885	0.450816874
RP1-90G24	-0.046	0.174735697	0.450771155

C1QTNF9-AS1	-0.046	0.174699438	0.450726396
RORA	-0.046	0.174626892	0.450661337
AC093817	-0.046	0.174586034	0.45058031
CTAGE1	-0.046	0.174576615	0.45058031
RP11-565F19	-0.046	0.174575835	0.45058031
SNX18P14	-0.046	0.174539901	0.450534491
RP11-415C15	-0.046	0.174531095	0.450534491
DFFA	-0.046	0.174465771	0.450514057
ZMYM6NB	-0.046	0.174464903	0.450514057
ADCY6	-0.046	0.174448762	0.450514057
PUS10	-0.046	0.174447374	0.450514057
RP11-63B13	-0.046	0.174420207	0.450496117
RP11-297N6	-0.046	0.174377486	0.450487726
VDAC1P6	-0.046	0.174364893	0.450487726
RP1-240B8	-0.046	0.17433791	0.450476976
RP5-894A10	-0.046	0.174278226	0.450371625
RP11-159G9	-0.046	0.174248746	0.450344313
ATG12	-0.046	0.174202105	0.450248205
RANP9	-0.046	0.174122914	0.450092377
PRR23C	-0.046	0.174096651	0.450048915
FYTTD1	-0.046	0.174060335	0.449979458
RNU6-1333P	-0.046	0.174026212	0.449915665
RNA5SP223	-0.046	0.173979182	0.449867339
SLC25A36P1	-0.046	0.17395757	0.449835877
AC131263	-0.046	0.173888614	0.449706398
PKI55	-0.046	0.173837627	0.449623366
EIF4BP3	-0.046	0.17383105	0.449623366
NEUROD6	-0.046	0.173802795	0.449582105
PMP2	-0.046	0.173769779	0.449569952
FCER1G	-0.046	0.173736907	0.449509326
RELL2	-0.046	0.173721855	0.4494948
BRD7P1	-0.046	0.173680882	0.4494132
BIRC6-AS2	-0.046	0.17363568	0.449320649
AP3M2	-0.046	0.173559801	0.449197514
CMTM6	-0.046	0.173544242	0.449197514
AC131056	-0.046	0.173425325	0.448995867
MRPL35	-0.046	0.173417038	0.448995867
SRD5A3-AS1	-0.046	0.173414792	0.448995867
RNA5SP499	-0.046	0.17340909	0.448995867
XIAP	-0.046	0.173357781	0.448943018
AC004541	-0.046	0.17335489	0.448943018
ANGPTL5	-0.046	0.173333076	0.448927861
MRPL53P1	-0.046	0.173313576	0.44892254
HZGJ	-0.046	0.173286778	0.448905594
AC090954	-0.046	0.173270488	0.448887812
HCCS	-0.046	0.173267111	0.448887812
DPRXP5	-0.046	0.173203914	0.448788576
LL22NC03-75A1	-0.046	0.17319654	0.448788576
PRKAB1	-0.046	0.173176821	0.448767204
MCM2	-0.046	0.173156627	0.44873929
OR10AH1P	-0.046	0.17309252	0.448597564
ZNF410	-0.046	0.173087429	0.448597564

TBC1D3I	-0.046	0.173066613	0.448587741
CTD-2545G14	-0.046	0.173054813	0.448587741
TRUB2	-0.046	0.172895128	0.448351413
C9orf24	-0.046	0.172869003	0.448351413
PARP4P1	-0.046	0.17274477	0.448108558
RP11-436M15	-0.046	0.172597447	0.447801852
RP11-454H13	-0.046	0.172585351	0.447801852
RN7SL200P	-0.046	0.172487217	0.44758901
FAM102A	-0.046	0.172477791	0.447588938
MIR548S	-0.046	0.17245596	0.44755667
DDB1	-0.046	0.172360288	0.447430284
RPL23AP3	-0.046	0.172289026	0.447318438
CCL23	-0.046	0.172255497	0.447255768
DLEU1	-0.046	0.172173059	0.447114846
ERH	-0.046	0.172163607	0.44711468
CMB9-94B1	-0.046	0.172135559	0.447066215
OR2J1	-0.046	0.172120197	0.447050698
IGLVV-58	-0.046	0.172092986	0.447004399
DKFZp434P228	-0.047	0.172061621	0.446947304
RP11-2E11	-0.047	0.172025137	0.446925661
RPL18P13	-0.047	0.17201591	0.446925661
RHOA-IT1	-0.047	0.171972417	0.446849219
CPED1	-0.047	0.171756338	0.44639777
SH3BP5	-0.047	0.171753251	0.44639777
FBXO31	-0.047	0.171738841	0.44639777
LINC01206	-0.047	0.171707516	0.446343952
RPS4XP21	-0.047	0.171641502	0.446234248
OR9I1	-0.047	0.171633791	0.446234248
AC016712	-0.047	0.171567181	0.446100898
BRF2	-0.047	0.171563772	0.446100898
RP11-202K23	-0.047	0.171548509	0.446100898
AC021224	-0.047	0.171532024	0.446100898
CEACAMP5	-0.047	0.171517504	0.446093499
RP11-101O21	-0.047	0.171458133	0.446019257
ARMCX5	-0.047	0.171456157	0.446019257
RP11-157L3	-0.047	0.171438215	0.446019257
CTD-3075F15	-0.047	0.171424684	0.446019257
RP1-28C20	-0.047	0.171421272	0.446019257
RP11-500M10	-0.047	0.171357503	0.445920871
RP11-73M14	-0.047	0.171350953	0.445920871
UHRF1	-0.047	0.171340029	0.445920871
OR5B2	-0.047	0.171336691	0.445920871
MTRF1LP1	-0.047	0.1713362	0.445920871
HNRNPA1P48	-0.047	0.171301847	0.445920871
MTND2P33	-0.047	0.17120646	0.445783972
ZSCAN12	-0.047	0.171205306	0.445783972
RP11-290L1	-0.047	0.171143278	0.445704335
YTHDF2	-0.047	0.171117189	0.445660762
RP11-340I6	-0.047	0.171102366	0.445646527
FBXO15	-0.047	0.171026196	0.445496863
STAM2	-0.047	0.171025172	0.445496863
UBE2V2P2	-0.047	0.171005124	0.445490705

RP11-336K24	-0.047	0.17097781	0.44546828
C5orf47	-0.047	0.170903995	0.445300322
OR8A3P	-0.047	0.1708797	0.445261379
EMCN	-0.047	0.170861583	0.445238531
MIR7849	-0.047	0.170844062	0.445217234
RP11-23N2	-0.047	0.170836936	0.445217234
RNU6-1315P	-0.047	0.170743153	0.445051673
RP11-2L4	-0.047	0.170737129	0.445051673
CTD-3253I12	-0.047	0.170723323	0.445048697
RPS7P5	-0.047	0.170706354	0.44502882
FAM72A	-0.047	0.170671987	0.444987942
MIR6859-3	-0.047	0.170664915	0.444987942
RP11-136F16	-0.047	0.170550747	0.444744876
AC097713	-0.047	0.170529853	0.444714741
RP11-365F18	-0.047	0.170432141	0.444484262
PRKRIRP1	-0.047	0.170413065	0.44445885
SLIT2-IT1	-0.047	0.170410921	0.44445885
AC027806	-0.047	0.17040384	0.44445885
RN7SL477P	-0.047	0.170317433	0.444306753
AC078816	-0.047	0.170242208	0.444134839
CHGA	-0.047	0.170213157	0.444132035
GTF2IRD2B	-0.047	0.170201752	0.444132035
CHCHD2P8	-0.047	0.170167978	0.444111473
ISCA2	-0.047	0.17010823	0.443979871
IGLVIV-64	-0.047	0.170068072	0.443899384
PFDN4	-0.047	0.170054417	0.443888071
RNU6-697P	-0.047	0.170043027	0.443882667
SUMO2P1	-0.047	0.170025064	0.443860103
SPACA5	-0.047	0.170004008	0.443829463
SPATA31C1	-0.047	0.170001169	0.443829463
FAM110C	-0.047	0.169967594	0.44378305
EIF2B5-AS1	-0.047	0.169954803	0.44377398
SCAF8	-0.047	0.169921132	0.443734716
RNA5SP433	-0.047	0.169838777	0.443568295
RNU6-294P	-0.047	0.169824128	0.443568295
DBP	-0.047	0.169815478	0.443568295
BMI1	-0.047	0.169809695	0.443568295
SYNJ2	-0.047	0.169741499	0.443484469
SLC25A21-AS1	-0.047	0.169717693	0.443446596
RP11-459E5	-0.047	0.169689161	0.443420699
GRPEL2	-0.047	0.169655306	0.443356556
RP11-203I16	-0.047	0.169638816	0.443337791
OR51A2	-0.047	0.169590078	0.44330772
LHFPL3	-0.047	0.169560989	0.44325601
RP11-103H7	-0.047	0.169554039	0.44325601
AC092159	-0.047	0.169549189	0.44325601
RP11-46H11	-0.047	0.169537713	0.44325601
RP11-386P4	-0.047	0.169512784	0.44325601
ATP6V1G1P4	-0.047	0.169491568	0.443244832
KCNH3	-0.047	0.169478956	0.443236188
CCT6P4	-0.047	0.169470159	0.443236188
RNU6-1199P	-0.047	0.169436567	0.443222682

RP11-423H2	-0.047	0.169434785	0.443222682
RN7SKP209	-0.047	0.169434093	0.443222682
RP11-863H1	-0.047	0.169353782	0.443079135
RP11-579O24	-0.047	0.169289215	0.44293454
C16orf78	-0.047	0.16923921	0.442852364
RP11-101B14	-0.047	0.169153789	0.442653159
ZC3H7B	-0.047	0.169153416	0.442653159
FLJ27354	-0.047	0.169137128	0.442653159
AC004901	-0.047	0.169130494	0.442653159
RASSF10	-0.047	0.169091802	0.442588221
RP11-455I9	-0.047	0.169066763	0.442547004
RP11-514F3	-0.047	0.169065635	0.442547004
PNPLA5	-0.047	0.168964553	0.442425354
COPZ1	-0.047	0.168931691	0.442387949
FCN1	-0.047	0.168927774	0.442387949
FCAR	-0.047	0.168903396	0.442362499
TBC1D32	-0.047	0.168865453	0.44231665
IL15RA	-0.047	0.168814136	0.442225997
TMEM106A	-0.047	0.16879009	0.442211652
RNU6-640P	-0.047	0.168741372	0.442115322
WDR45B	-0.047	0.16872237	0.442107192
LINC00266-3	-0.047	0.168721254	0.442107192
RNU6ATAC14P	-0.047	0.168718658	0.442107192
ABHD2	-0.047	0.168686825	0.442087021
MIR374B	-0.047	0.168669795	0.442087021
PRKAR1AP	-0.047	0.168640317	0.442086767
AC005088	-0.047	0.168545833	0.441912051
KRT18P4	-0.047	0.168482628	0.441843628
C11orf71	-0.047	0.168478578	0.441843628
SHANK2	-0.047	0.168457611	0.441826679
RNU6ATAC13P	-0.047	0.168449735	0.441826679
CCL2	-0.047	0.168424306	0.441787984
METAP1	-0.047	0.168405542	0.441787428
CTA-342B11	-0.047	0.168368601	0.441763517
MAP3K3	-0.047	0.168348246	0.441763517
LINC01569	-0.047	0.168321716	0.441737842
DAZAP2	-0.047	0.168310902	0.441733803
MIR3973	-0.047	0.168273137	0.441697393
LSM8	-0.047	0.168270523	0.441697393
RP3-391O22	-0.047	0.16825082	0.441697393
RP11-19E18	-0.047	0.168243538	0.441697393
RP11-467M13	-0.047	0.168223878	0.441692722
RRP7B	-0.047	0.168186578	0.441650887
RNU6-862P	-0.047	0.168182931	0.441650887
RP11-459I19	-0.047	0.168180125	0.441650887
PRKRIRP5	-0.047	0.168160723	0.441650887
LINC01150	-0.047	0.168116381	0.441612645
RP11-726G23	-0.047	0.168099383	0.441592351
RERE	-0.047	0.168091186	0.441592351
LINC01160	-0.047	0.167920048	0.441242932
RNU4-15P	-0.047	0.167891082	0.44119116
ISCA1P2	-0.047	0.167842468	0.441112087

ACO2	-0.047	0.167825146	0.441112087
BUD13	-0.047	0.167813007	0.441112087
RP11-143K11	-0.047	0.167781463	0.441073454
STIP1P3	-0.047	0.167751178	0.441018181
WSB2	-0.047	0.167750975	0.441018181
LINC01376	-0.047	0.167746593	0.441018181
BCL2L14	-0.047	0.167686592	0.440945745
SRGAP3-AS3	-0.047	0.167680998	0.440945745
RNA5SP393	-0.047	0.167668625	0.440945745
FKBP6	-0.047	0.167612414	0.440823703
RN7SKP27	-0.047	0.16760525	0.440823703
SOWAHD	-0.047	0.167593105	0.440821606
TGFBR3	-0.047	0.167509245	0.440649701
RP11-724N1	-0.047	0.16748785	0.440617754
PRAMEF19	-0.047	0.167469596	0.440615559
FAM86MP	-0.047	0.167466813	0.440615559
NDUFS5P4	-0.047	0.167457832	0.440615559
RNU6-452P	-0.047	0.167401386	0.440512396
HIF1AP1	-0.047	0.167398699	0.440512396
CEACAM5	-0.047	0.16739507	0.440512396
BC040587	-0.047	0.16734226	0.440502394
RAB30	-0.047	0.167337246	0.440502394
AC109357	-0.047	0.167312092	0.440471671
OR6E1P	-0.047	0.167295196	0.440451538
RP11-571M6	-0.047	0.167153444	0.440175666
PLEKHA2	-0.047	0.167146553	0.440175666
HTRA4	-0.047	0.167114935	0.440124362
RP11-286H15	-0.047	0.16705869	0.440023463
RP11-16B9	-0.047	0.166996535	0.439932739
WDR62	-0.047	0.166995829	0.439932739
RP11-802E16	-0.047	0.166980712	0.439932739
AC091866	-0.047	0.166962775	0.439916801
ADAM8	-0.047	0.166943763	0.439891042
SLMO2P2	-0.047	0.166921693	0.439891042
RP1-215K18	-0.047	0.166915178	0.439891042
LINC00963	-0.047	0.166902623	0.439891042
ODC1	-0.047	0.166835639	0.439800796
IGKV2-23	-0.047	0.166834488	0.439800796
AC079117	-0.047	0.166809705	0.439781114
FPR1	-0.047	0.16680442	0.439781114
CASP2	-0.047	0.16675156	0.439700839
ORC1	-0.047	0.166705255	0.439627419
SNRK-AS1	-0.047	0.166685765	0.439600361
RRH	-0.047	0.166641646	0.439557025
AC080008	-0.047	0.16660358	0.439480958
ZNF709	-0.047	0.166596409	0.439480958
KRT8P33	-0.047	0.166561703	0.439428898
RCC2P4	-0.047	0.166519588	0.439381075
MUC3A	-0.047	0.16651946	0.439381075
HELB	-0.047	0.166480912	0.439327704
TSEN15P1	-0.047	0.166458388	0.439292604
TRAK2	-0.047	0.16639546	0.439199544

AC130462	-0.047	0.166326512	0.439041888
RNU4-23P	-0.047	0.166281429	0.438971543
NEK2	-0.047	0.166266339	0.438971543
LYRM5	-0.047	0.16621662	0.438922098
IGLV3-2	-0.047	0.166191304	0.438879582
RNU6-479P	-0.047	0.166187032	0.438879582
RPL23A	-0.047	0.166175614	0.438879582
RP11-344L21	-0.047	0.166165038	0.438879582
RSF1-IT1	-0.047	0.166147949	0.438879582
TRIM55	-0.047	0.166140373	0.438879582
ATP6V1G1P7	-0.047	0.166125377	0.438879582
APH1B	-0.047	0.166112028	0.438879582
HPRTP3	-0.047	0.166095285	0.438869356
AC068657	-0.047	0.166004378	0.438653491
MAPKAPK5-AS1	-0.047	0.166002971	0.438653491
AC097467	-0.047	0.165954662	0.438595123
OR5AO1P	-0.047	0.165787993	0.4383249
AC002511	-0.047	0.165779611	0.4383249
RP11-116B19	-0.047	0.165771159	0.4383249
RP11-234O6	-0.047	0.165730542	0.438270322
RAD51	-0.047	0.165705012	0.438264241
RNU6-257P	-0.047	0.16570074	0.438264241
AC015600	-0.047	0.165695137	0.438264241
RP11-715F3	-0.047	0.165682126	0.438264241
GTF3C4	-0.047	0.165674453	0.438264241
MX2	-0.047	0.16566193	0.438264241
RP11-435P24	-0.047	0.165569973	0.43811329
RP3-434P1	-0.047	0.165445229	0.437866763
RNU6-41P	-0.047	0.165419646	0.43783714
CTC-472C24	-0.047	0.165410416	0.43783704
SCO1	-0.047	0.165409955	0.43783704
TBC1D3P1-DHX40P1	-0.047	0.165378264	0.437800599
IL6ST	-0.047	0.165337253	0.437716362
RP11-443B7	-0.047	0.165234244	0.437492292
CTAGE5	-0.047	0.165227745	0.437492292
CTNNA3	-0.047	0.16522621	0.437492292
CRYZL1	-0.047	0.165209582	0.437492292
SNX5P1	-0.047	0.165203198	0.437492292
RP11-572O6	-0.047	0.165150327	0.437440332
RP11-432N13	-0.047	0.165145575	0.437440332
RASSF2	-0.047	0.165132449	0.437440332
EAF1-AS1	-0.047	0.165129351	0.437440332
RNF141	-0.047	0.164999737	0.437211665
HTATSF1	-0.047	0.164997906	0.437211665
RP11-716A19	-0.047	0.164996968	0.437211665
ACTL7B	-0.047	0.164974448	0.437211665
KIAA1191	-0.047	0.16497377	0.437211665
RNU7-153P	-0.047	0.16496318	0.437211665
CD5L	-0.047	0.16494711	0.437211665
PRDX3	-0.047	0.164941149	0.437211665
RNA5SP370	-0.047	0.164939836	0.437211665
RP11-506E9	-0.047	0.164893158	0.437172479

AL136221	-0.047	0.164889508	0.437172479
RNU6-1115P	-0.047	0.164868071	0.437154638
OR8H2	-0.047	0.164836654	0.437120007
RP11-380P13	-0.047	0.164814339	0.437109507
CTD-2140G10	-0.047	0.164766073	0.437005833
RABGAP1L-IT1	-0.047	0.164723513	0.436964305
USP51	-0.047	0.164671714	0.436877214
LINC01486	-0.047	0.164671335	0.436877214
EDDM3A	-0.047	0.164663391	0.436877214
GRB7	-0.047	0.164606069	0.436800381
KCNC2	-0.047	0.164587349	0.436775042
ZMYND11	-0.047	0.164546922	0.43669209
RP11-500G9	-0.047	0.164519236	0.436642945
PFKFB1	-0.047	0.164468859	0.436557897
NUDT16	-0.047	0.164466866	0.436557897
KIF27	-0.047	0.164457401	0.436557897
CABYR	-0.047	0.16444146	0.436557897
OTUD6A	-0.047	0.164391165	0.436448966
MCPH1	-0.047	0.164345842	0.436377287
FGF12-AS2	-0.047	0.16434468	0.436377287
OR2AD1P	-0.047	0.164336084	0.436377287
ELL2P1	-0.047	0.164333549	0.436377287
MIR628	-0.047	0.164319906	0.436377287
AL441988	-0.047	0.164287923	0.436369466
RP11-446N19	-0.047	0.164245185	0.436280282
NHP2P1	-0.047	0.164223836	0.436247903
RN7SL604P	-0.047	0.164223368	0.436247903
RNU7-179P	-0.047	0.164210526	0.436247903
RP11-298J20	-0.047	0.164198904	0.436247903
RNU6-578P	-0.047	0.16413756	0.436116015
USP17L10	-0.047	0.164135254	0.436116015
RP11-788A4	-0.047	0.164134279	0.436116015
LZTFL1	-0.047	0.164130853	0.436116015
RP1-102G20	-0.047	0.164121719	0.436116015
RP11-262A12	-0.047	0.164114743	0.436116015
RP11-546B15	-0.047	0.164106256	0.436116015
WBSCR27	-0.047	0.164068519	0.436116015
FAM118A	-0.047	0.164039513	0.436116015
HSDL1	-0.047	0.164028409	0.436116015
CCNB2P1	-0.047	0.163996462	0.436116015
MIR892C	-0.047	0.163980607	0.436112596
AC092657	-0.047	0.163931486	0.436055007
ZCWPW2	-0.047	0.163917566	0.436042332
AP001962	-0.047	0.163897406	0.436037412
C20orf144	-0.047	0.163890229	0.436037412
OR51C4P	-0.047	0.163859084	0.436014297
DNAJA1P1	-0.047	0.163817029	0.435945333
RP13-140E4	-0.047	0.163793235	0.43590637
AP000345	-0.047	0.163785924	0.43590637
ST13P10	-0.047	0.163782441	0.43590637
RP3-435K13	-0.047	0.163766911	0.43590637
GOLGA6L10	-0.047	0.163605157	0.435600559

PHKA2	-0.047	0.163591493	0.435600559
CA5B	-0.047	0.163589674	0.435600559
SSU72P7	-0.047	0.1635353	0.435511952
FUT7	-0.047	0.163528582	0.435511952
AC074191	-0.047	0.163498578	0.435462854
AC092634	-0.047	0.163462004	0.435389792
RP11-373F14	-0.047	0.163449689	0.43538134
NPIPA3	-0.047	0.163430975	0.435355839
C5orf22	-0.047	0.163411122	0.435327303
CTC-295J13	-0.047	0.163376565	0.435273953
TPD52	-0.047	0.163365185	0.435273953
CTA-397H3	-0.047	0.163344221	0.43524646
OR51C1P	-0.047	0.163331039	0.435235686
RP11-392B6	-0.047	0.163217653	0.435128305
DDIAS	-0.047	0.163215215	0.435128305
TRAJ54	-0.047	0.163192159	0.435109051
HNRNPA1P11	-0.047	0.163190516	0.435109051
IFNL2	-0.047	0.163062974	0.434861989
BTNL10	-0.047	0.163050251	0.434861989
CDK5R2	-0.048	0.16292311	0.434635018
RPL7P28	-0.048	0.162913763	0.434635018
ADAM20P1	-0.048	0.162898831	0.434635018
U82670	-0.048	0.162897587	0.434635018
MSANTD1	-0.048	0.162811735	0.43450292
CCDC53	-0.048	0.162809922	0.43450292
RP5-1014D13	-0.048	0.162809428	0.43450292
RP11-355E10	-0.048	0.162797222	0.43450292
SH2D2A	-0.048	0.162792289	0.43450292
PPIAP29	-0.048	0.162781743	0.43450292
RP11-5A11	-0.048	0.162767546	0.43450292
SERPINB9P1	-0.048	0.162756863	0.43450292
CDH12P1	-0.048	0.162755107	0.43450292
ATPAF1	-0.048	0.162718972	0.43450292
RP11-355K23B	-0.048	0.162673872	0.434408102
AL355977	-0.048	0.162651974	0.434398339
RNA5SP399	-0.048	0.162640245	0.434391372
RPS3AP9	-0.048	0.162626477	0.43437896
REV3L	-0.048	0.162619004	0.43437896
DPRXP4	-0.048	0.162464891	0.434019365
RNU6-599P	-0.048	0.162460286	0.434019365
RP11-385F5	-0.048	0.16243434	0.433987453
C1orf111	-0.048	0.162289057	0.433691343
SNORD83A	-0.048	0.162281936	0.433691343
RP11-2H3	-0.048	0.162266855	0.433691343
MIR5589	-0.048	0.162246976	0.433681487
RNA5SP33	-0.048	0.162215895	0.433622745
RP11-438D8	-0.048	0.162211264	0.433622745
PHKB	-0.048	0.162196585	0.433622745
DZANK1	-0.048	0.162184753	0.433622745
NIFKP4	-0.048	0.162151299	0.433596089
VDAC1P11	-0.048	0.162148169	0.433596089
RNA5SP206	-0.048	0.162067047	0.433444026

C16orf96	-0.048	0.162055087	0.433444026
AL603910	-0.048	0.162005958	0.433353428
ZNF592	-0.048	0.16199488	0.433348132
bP-2189O9	-0.048	0.161989055	0.433348132
OAT	-0.048	0.161962539	0.433310294
LYPLA1P3	-0.048	0.161908261	0.433189412
UCKL1	-0.048	0.161884416	0.433149949
RP11-184B22	-0.048	0.161852383	0.433112902
RFPL4AP5	-0.048	0.161840698	0.433112902
SAP30	-0.048	0.161789263	0.433041319
SLC22A13	-0.048	0.161777358	0.43303379
TREML1	-0.048	0.16171798	0.432947849
LRRC34P2	-0.048	0.161689291	0.432904576
KRT8P10	-0.048	0.161629717	0.432784536
CELP	-0.048	0.161583757	0.432713867
RNA5SP464	-0.048	0.161531949	0.432620039
ENSAP2	-0.048	0.161477902	0.432523933
RP11-544H14	-0.048	0.161450691	0.432475369
ZNF630-AS1	-0.048	0.161449636	0.432475369
RP11-1030E3	-0.048	0.161447344	0.432475369
ITIH6	-0.048	0.161411542	0.432443467
DICER1	-0.048	0.161395809	0.432425642
LA16c-361A3	-0.048	0.161392967	0.432425642
NDUFV2-AS1	-0.048	0.161375509	0.432419904
RNU6-898P	-0.048	0.161308834	0.432265561
RNU1-22P	-0.048	0.161266653	0.432197996
RPS3AP2	-0.048	0.161205391	0.432109925
RPL35AP31	-0.048	0.161188193	0.432088146
SAPCD2	-0.048	0.161147917	0.432028811
RP11-327O17	-0.048	0.161109963	0.431956411
RP11-453O5	-0.048	0.161083129	0.431928059
RP11-387H17	-0.048	0.16108229	0.431928059
F11	-0.048	0.161050113	0.431912481
ZNF718	-0.048	0.160998829	0.431823575
SCARNA6	-0.048	0.160968262	0.431765905
LDB3	-0.048	0.16095128	0.431744669
TMX2	-0.048	0.160949156	0.431744669
LINC00272	-0.048	0.160896305	0.43167014
HSPA14	-0.048	0.160892308	0.43167014
HMGN2P20	-0.048	0.160878783	0.43167014
RP3-508I15	-0.048	0.160849518	0.431617556
AC005549	-0.048	0.160837613	0.431609928
XXbac-B476C20	-0.048	0.160739154	0.431394326
USP9YP3	-0.048	0.160735223	0.431394326
RP11-551L14	-0.048	0.160729035	0.431394326
OR4G4P	-0.048	0.160677765	0.431375417
LL0XNC01-237H1	-0.048	0.160666791	0.431370271
GCLC	-0.048	0.160597692	0.431233368
MT-CO2	-0.048	0.160589699	0.431233368
UTP15	-0.048	0.160542078	0.431156962
TRMT112P5	-0.048	0.160532331	0.431155098
TOR1AIP1	-0.048	0.160516642	0.431137271

ZNF30-AS1	-0.048	0.160489807	0.431089507
OR1N1	-0.048	0.160399607	0.430895826
MTF1	-0.048	0.160366365	0.430855129
PRDM11	-0.048	0.160355938	0.430851418
RBBP4P6	-0.048	0.160329496	0.430804677
AC034220	-0.048	0.160327754	0.430804677
RP11-25B7	-0.048	0.160286517	0.430786409
RP11-592N21	-0.048	0.160164588	0.430483004
CTD-2270P14	-0.048	0.160145381	0.430455673
RP11-241K18	-0.048	0.160118767	0.43044803
ACTR5	-0.048	0.160090863	0.430406295
COG6	-0.048	0.160078984	0.430406295
ANKHD1	-0.048	0.160046282	0.430359319
RNU6-904P	-0.048	0.160044708	0.430359319
RNU6-651P	-0.048	0.159965551	0.430219906
PGBD4P4	-0.048	0.159908703	0.430110793
RP11-348J24	-0.048	0.159896983	0.430103557
AC127030	-0.048	0.159864378	0.430040144
CTB-50L17	-0.048	0.159820593	0.42997093
GDF7	-0.048	0.159771898	0.429864207
CTC-820M8	-0.048	0.159753933	0.429840155
ASB13	-0.048	0.159741301	0.429840155
HNRNPCP8	-0.048	0.159727503	0.429840155
OR5C1	-0.048	0.1596543	0.429693452
RP11-632P5	-0.048	0.159645824	0.429693452
YTHDF1	-0.048	0.15964369	0.429693452
RN7SL487P	-0.048	0.159623423	0.429683195
KRT24	-0.048	0.159619979	0.429683195
HMGB3P14	-0.048	0.159581868	0.429659137
PPP1R26P5	-0.048	0.159574562	0.429659137
RP1-66N13	-0.048	0.15956155	0.429659137
ZRANB2-AS1	-0.048	0.159549864	0.429655173
RNU6-560P	-0.048	0.159530198	0.429639258
ACY3	-0.048	0.159519077	0.429639258
RP11-326E22	-0.048	0.159506406	0.429635313
C10orf32	-0.048	0.159339937	0.429235475
RNU6-359P	-0.048	0.1593396	0.429235475
SNRPEP6	-0.048	0.159335405	0.429235475
SNIP1	-0.048	0.15930362	0.429217581
MAGEB16	-0.048	0.159249194	0.429112383
RNU6-196P	-0.048	0.159218509	0.429053975
PRKDC	-0.048	0.159209938	0.429053975
LIM2	-0.048	0.159168457	0.428991914
OR4A45P	-0.048	0.159158954	0.428990576
RP11-354M1	-0.048	0.159144743	0.428976552
RP11-436F21	-0.048	0.159102148	0.42888601
C9orf62	-0.048	0.159057462	0.428809172
AC006378	-0.048	0.159034758	0.428777152
MRRF	-0.048	0.159023183	0.428770219
STXBP5L	-0.048	0.159020864	0.428770219
LRRC56	-0.048	0.15899032	0.428730153
OR2A25	-0.048	0.158971049	0.428702461

LINC00564	-0.048	0.158962981	0.428702461
RP11-122C21	-0.048	0.158947659	0.428687929
C1DP1	-0.048	0.158935731	0.428680035
TRIM31-AS1	-0.048	0.158912287	0.428641076
AC104978	-0.048	0.158875529	0.4285662
OR2AS2P	-0.048	0.158869778	0.4285662
RP5-865E18	-0.048	0.158869778	0.4285662
ACAD10	-0.048	0.158860212	0.4285662
RDH14	-0.048	0.158817346	0.428530607
TUBBP2	-0.048	0.158783536	0.428463653
ZNF394	-0.048	0.158779825	0.428463653
VTI1A	-0.048	0.158772387	0.428463653
RP5-1092A3	-0.048	0.158703995	0.428321822
RNA5SP224	-0.048	0.158702281	0.428321822
PACRG-AS1	-0.048	0.158680979	0.428321822
CD46	-0.048	0.158678294	0.428321822
RP11-365H22	-0.048	0.158550446	0.428108553
ATP6V1G1P2	-0.048	0.158504203	0.428049426
HSP90B1	-0.048	0.158499924	0.428049426
ACOT13	-0.048	0.158492172	0.428049426
RP11-344G13	-0.048	0.158398186	0.427908701
SAAL1	-0.048	0.158393362	0.427908701
RP11-134L10	-0.048	0.158392555	0.427908701
RAG2	-0.048	0.15835982	0.427877867
SH2D3C	-0.048	0.158353335	0.427877867
TFCP2	-0.048	0.158325256	0.427857295
VPS37A	-0.048	0.158314283	0.427854874
IGLV3-26	-0.048	0.158306353	0.427854874
RNU1-54P	-0.048	0.15830532	0.427854874
IGHV2OR16-5	-0.048	0.158286668	0.427854874
HSPE1P26	-0.048	0.158260466	0.427827859
RP11-16L14	-0.048	0.158251511	0.427827859
RNU6-739P	-0.048	0.158244238	0.427827859
CTD-2230M5	-0.048	0.158230299	0.427819157
POTEI	-0.048	0.158226032	0.427819157
MTND5P2	-0.048	0.158179939	0.427755845
ACLY	-0.048	0.158172823	0.427755845
RP1-71H19	-0.048	0.158130729	0.427671337
RN7SL500P	-0.048	0.158114812	0.427652573
RP11-145O15	-0.048	0.158109279	0.427652573
RP5-1042K10	-0.048	0.15808706	0.427639154
RAD54B	-0.048	0.158085446	0.427639154
ART1	-0.048	0.158070346	0.427639154
RP11-714G18	-0.048	0.158066722	0.427639154
SERBP1P6	-0.048	0.158017744	0.427632882
F8A3	-0.048	0.158012165	0.427632882
HLA-DRB5	-0.048	0.157989083	0.427603913
AC073987	-0.048	0.157931013	0.427495331
MT-ATP6	-0.048	0.157898723	0.427475262
FARP2	-0.048	0.157894053	0.427475262
RP11-252M21	-0.048	0.157890685	0.427475262
ALPK1	-0.048	0.157888578	0.427475262

HSPD1P9	-0.048	0.15785705	0.427465189
AP000459	-0.048	0.157753627	0.427282302
RN7SL205P	-0.048	0.157730853	0.427244914
LINC01233	-0.048	0.157725386	0.427244914
RPL30P2	-0.048	0.157706144	0.42722658
MIR4652	-0.048	0.157698908	0.42722658
RXFP2	-0.048	0.157684233	0.42722658
CTD-3051L14	-0.048	0.157680348	0.42722658
TET2	-0.048	0.157669535	0.42722658
PRR23A	-0.048	0.15766716	0.42722658
CCDC88A	-0.048	0.157642835	0.42722658
HMGB1P4	-0.048	0.157641832	0.42722658
RP11-3D23	-0.048	0.157614424	0.42722658
NRBF2P2	-0.048	0.157609933	0.42722658
LINC00905	-0.048	0.157597564	0.42722658
SMIM14	-0.048	0.157532369	0.427168875
CDC23	-0.048	0.157509061	0.427129991
RP11-261P24	-0.048	0.157480372	0.427091725
SSRP1	-0.048	0.157480131	0.427091725
TRPS1	-0.048	0.157479809	0.427091725
C12orf71	-0.048	0.157353261	0.426950593
OSBPL2	-0.048	0.157304547	0.426891376
SUN3	-0.048	0.157295061	0.426889958
CNBP	-0.048	0.157292022	0.426889958
RNU6-1015P	-0.048	0.157279966	0.426889958
PDZD9	-0.048	0.157238977	0.426810712
MCM3	-0.048	0.157230213	0.426810712
SNORD116-14	-0.048	0.157228396	0.426810712
LSINCT5	-0.048	0.157222594	0.426810712
HMGN1P8	-0.048	0.157203419	0.426810712
CDC42P4	-0.048	0.157167432	0.426762444
SCML1	-0.048	0.157155264	0.42675723
MYOT	-0.048	0.157135249	0.42675723
DSCAM-AS1	-0.048	0.15712381	0.42675723
U82671	-0.048	0.157111617	0.42675723
RNA5SP324	-0.048	0.157108514	0.42675723
AC009969	-0.048	0.157083751	0.426754196
FAM114A2	-0.048	0.157016833	0.426596731
EWSR1	-0.048	0.156970496	0.426541005
RNF165	-0.048	0.156948052	0.426527222
FAM60BP	-0.048	0.156928901	0.42650381
USP41	-0.048	0.156851228	0.426414376
TCF23	-0.048	0.156826222	0.426395071
CTD-2319I12	-0.048	0.156821453	0.426395071
RP11-644F5	-0.048	0.156814244	0.426395071
RP11-692N5	-0.048	0.15678717	0.426386258
HMGN2P46	-0.048	0.156749054	0.426331289
FTLP13	-0.048	0.156733135	0.426320813
RP11-423P10	-0.048	0.156694291	0.42625537
HLA-S	-0.048	0.1566932	0.42625537
C1orf100	-0.048	0.15665414	0.426199102
AC109471	-0.048	0.156601876	0.426101323

AL356309	-0.048	0.156592346	0.426099734
MRPL32P1	-0.048	0.156544385	0.425993569
ZNF599	-0.048	0.156540488	0.425993569
RPS24P18	-0.048	0.156537604	0.425993569
TESC	-0.048	0.156528809	0.425993569
FAM74A1	-0.048	0.156509626	0.425993569
AC011372	-0.048	0.156509626	0.425993569
AC135178	-0.048	0.156494543	0.425993569
AC091153	-0.048	0.156447642	0.425900647
RP11-146N23	-0.048	0.15639907	0.425841443
CROCCP1	-0.048	0.156359145	0.425757075
TYW1	-0.048	0.156357386	0.425757075
SH3BP1	-0.048	0.156312314	0.425702573
MOB3A	-0.048	0.156285468	0.4256538
MED15P4	-0.048	0.156223158	0.425532762
FPGT	-0.048	0.156195847	0.425495476
ALDH1A1	-0.048	0.156187382	0.425495476
SNORD3C	-0.048	0.156113821	0.425405249
RECQL5	-0.048	0.156101474	0.425395944
LINC00557	-0.048	0.156061858	0.425312322
OR51A8P	-0.048	0.156040894	0.425279523
ATP6V0E1P4	-0.048	0.156040238	0.425279523
HDAC11-AS1	-0.048	0.156030191	0.425279523
RP11-548M13	-0.048	0.156007437	0.425261344
C14orf183	-0.048	0.155998712	0.425261344
MIR4766	-0.048	0.15596806	0.425202678
ANKUB1	-0.048	0.155949513	0.425176452
LRCH3	-0.048	0.155936307	0.425169252
NELL1	-0.048	0.155904611	0.425146729
FAM90A20P	-0.048	0.155846637	0.425017624
WDR55	-0.048	0.155809051	0.424939454
LRRC8B	-0.048	0.155804747	0.424939454
GIT1	-0.048	0.155779401	0.424907253
CTA-544A11	-0.048	0.15574283	0.424856165
DNPEP	-0.048	0.155669129	0.424698994
LA16c-313D11	-0.048	0.155595859	0.424552505
GKN2	-0.048	0.155586701	0.424552505
OR4A13P	-0.048	0.155512575	0.424465887
ZNF43	-0.048	0.15546197	0.424381684
RP11-183K14	-0.048	0.155444551	0.424369515
MYH15	-0.048	0.155431147	0.424369515
RP11-866E20	-0.048	0.155429934	0.424369515
CTB-134H23	-0.048	0.155355458	0.424261147
RP11-861A13	-0.048	0.155291096	0.424158343
HMGB3P12	-0.048	0.155281366	0.424156092
MIR298	-0.048	0.155238243	0.424062621
AL035461	-0.048	0.155198314	0.424002187
KIAA0141	-0.048	0.15515806	0.423916527
RP11-167J8	-0.048	0.155142592	0.423898585
GOT2P3	-0.048	0.155131058	0.423891389
ZNF385B	-0.048	0.155128754	0.423891389
FAM209A	-0.048	0.155117469	0.423891389

TTC37	-0.048	0.155103718	0.423891389
DYX1C1-CCPG1	-0.048	0.155097955	0.423891389
RNU6-1203P	-0.048	0.155025329	0.423772676
LINC00489	-0.048	0.155021939	0.423772676
RNU105B	-0.048	0.155021331	0.423772676
AC083864	-0.048	0.155015531	0.423772676
MIR548H3	-0.048	0.154915333	0.423617878
RP11-573J24	-0.048	0.154817083	0.423446461
NAE1	-0.048	0.15481541	0.423446461
CCDC158	-0.048	0.154791595	0.423446461
RPS26P5	-0.048	0.154790622	0.423446461
ICAM2	-0.048	0.154752436	0.423415539
LINC00167	-0.048	0.154707859	0.423342211
RNA5SP194	-0.048	0.154685136	0.423328679
SIPA1L3	-0.048	0.15464113	0.423281213
TOMM70A	-0.048	0.154583598	0.423148056
MIR2467	-0.048	0.154572126	0.423140972
RNA5SP473	-0.048	0.154548363	0.423100746
DNAJC6	-0.048	0.154545569	0.423100746
MIR138-2	-0.048	0.154461283	0.422983413
ASB5	-0.048	0.154425243	0.422909036
PPP3R2	-0.048	0.154387088	0.422828859
RNU7-7P	-0.049	0.154322375	0.422700238
EBLN1	-0.049	0.154297854	0.422700238
RNU6-1037P	-0.049	0.154282614	0.422700238
CTD-2349B8	-0.049	0.154265415	0.422690077
RNU7-190P	-0.049	0.154263352	0.422690077
AC003084	-0.049	0.1542347	0.422678869
TNFRSF25	-0.049	0.15420442	0.422620206
LPAR6	-0.049	0.154143691	0.422502393
HDC	-0.049	0.154141	0.422502393
INTS6P1	-0.049	0.154117883	0.422502393
AC103564	-0.049	0.15409054	0.422453957
RP11-816B4	-0.049	0.154083955	0.422453957
AY269186	-0.049	0.154059257	0.422416823
AC008521	-0.049	0.154043524	0.422398004
MIR369	-0.049	0.154039378	0.422398004
ENPP2	-0.049	0.153967299	0.422276925
OR10AD1	-0.049	0.153904903	0.422163728
PRAMEF6	-0.049	0.153885658	0.422135252
RP11-169E6	-0.049	0.153860163	0.422089625
RP11-550H2	-0.049	0.15385799	0.422089625
SP3	-0.049	0.153790745	0.421972106
HSD17B7P2	-0.049	0.153711204	0.42180246
AC064829	-0.049	0.15370468	0.42180246
AC080094	-0.049	0.153635877	0.421692942
SNHG16	-0.049	0.153615448	0.42166117
CICP1	-0.049	0.153611547	0.42166117
GNE	-0.049	0.153570505	0.421586399
DENND6A	-0.049	0.153375549	0.42116602
GAPDHP16	-0.049	0.153368783	0.42116602
WDR35	-0.049	0.153356647	0.42116602

BLOC1S3	-0.049	0.153350662	0.42116602
OR4A11P	-0.049	0.153347017	0.42116602
AKAP3	-0.049	0.153342786	0.42116602
TRAJ31	-0.049	0.153333183	0.42116602
VPS13A	-0.049	0.153330344	0.42116602
AC003956	-0.049	0.153302112	0.42116602
OR7E8P	-0.049	0.153294001	0.42116602
RP11-96H19	-0.049	0.153249409	0.421093268
DEFB134	-0.049	0.153192657	0.420961613
MICF	-0.049	0.153187456	0.420961613
CT45A1	-0.049	0.153183988	0.420961613
RP11-93G5	-0.049	0.153165339	0.420959409
ADORA1	-0.049	0.153116898	0.420923433
PGK1P2	-0.049	0.153068813	0.420815536
KRTAP13-4	-0.049	0.153061333	0.420815536
SLC5A5	-0.049	0.153059382	0.420815536
AC003973	-0.049	0.152956879	0.420629209
RP1-212P9	-0.049	0.152952117	0.420629209
ABI2	-0.049	0.152938883	0.420629209
AC073215	-0.049	0.152923987	0.420629209
RP11-572C21	-0.049	0.152899665	0.420593297
RN7SL804P	-0.049	0.152898508	0.420593297
AC002480	-0.049	0.152786056	0.420353619
RP5-1050D4	-0.049	0.152764541	0.420318706
APPL1	-0.049	0.152730719	0.420249924
AC005162	-0.049	0.152722306	0.420249924
MTND1P2	-0.049	0.152721458	0.420249924
PREB	-0.049	0.152691309	0.420214318
RNU6-1297P	-0.049	0.152651456	0.420177478
AC055758	-0.049	0.152648654	0.420177478
NUP210P3	-0.049	0.152540322	0.419955813
PARD6A	-0.049	0.152503005	0.419914473
RN7SL134P	-0.049	0.152458012	0.419839128
RPEP2	-0.049	0.15239809	0.419722654
C2orf44	-0.049	0.152380546	0.419698604
EGR3	-0.049	0.152368947	0.419690927
CUL2	-0.049	0.152348728	0.419659506
AC108676	-0.049	0.15232954	0.419630922
RP11-452J21	-0.049	0.152323639	0.419630922
MMAA	-0.049	0.152243749	0.41944311
RPL35P3	-0.049	0.152164661	0.419249469
SPDL1	-0.049	0.152103445	0.41910505
RNU6ATAC26P	-0.049	0.152083131	0.419097568
LINC00483	-0.049	0.152069404	0.41908399
AC005702	-0.049	0.152046162	0.419044185
NUBPL	-0.049	0.152025037	0.419024443
ZNF655	-0.049	0.152016119	0.419024443
DLG2	-0.049	0.152004227	0.419024443
CABLES2	-0.049	0.151962302	0.418958526
RP11-46J23	-0.049	0.151949162	0.418947847
RP11-596D21	-0.049	0.151920581	0.418929744
MIR3687-1	-0.049	0.151869092	0.418919799

SCN3A	-0.049	0.15182673	0.418827203
TUBB4A	-0.049	0.151826152	0.418827203
FGFR3P1	-0.049	0.151816365	0.418827203
POM121L4P	-0.049	0.151720522	0.418606957
CLTC	-0.049	0.15171718	0.418606957
CLGN	-0.049	0.151666098	0.418505288
PBLD	-0.049	0.151617529	0.418395506
CTD-2187J20	-0.049	0.151613772	0.418395506
TRMT6	-0.049	0.151607847	0.418395506
RP11-99H8	-0.049	0.151599583	0.418395506
TTC26	-0.049	0.151591921	0.418395506
OR10N1P	-0.049	0.151554514	0.418342811
RP11-413H22	-0.049	0.151509163	0.418290353
KIN	-0.049	0.151503569	0.418290353
ZNF595	-0.049	0.151464245	0.41822316
CATSPER4	-0.049	0.151447002	0.41822316
RP11-369G6	-0.049	0.151421025	0.418216723
TNKS	-0.049	0.151413228	0.418216723
FAT1P1	-0.049	0.151361941	0.418126278
GAPDHP39	-0.049	0.151325999	0.418051238
FENDRR	-0.049	0.151203789	0.417786325
IGHVIII-2-1	-0.049	0.151094078	0.417507407
RBP5	-0.049	0.150988132	0.417306556
RN7SKP92	-0.049	0.150974712	0.417298624
SPCS2	-0.049	0.150971944	0.417298624
MCOLN1	-0.049	0.150950154	0.41727918
UNC93B3	-0.049	0.150946169	0.41727918
RP5-906C1	-0.049	0.150931718	0.417276655
SLC22A20	-0.049	0.150922454	0.417275265
IP6K1	-0.049	0.150863642	0.417136876
ERVH-1	-0.049	0.150856889	0.417136876
MIR4659B	-0.049	0.150794043	0.41700811
ZNF426	-0.049	0.150756178	0.416936558
NEK7	-0.049	0.150681562	0.416778602
HIST1H2BD	-0.049	0.150660892	0.416745633
CH507-513H4	-0.049	0.150641468	0.416721795
AC009695	-0.049	0.150593805	0.416632654
ARHGDIB	-0.049	0.150558507	0.416583398
LINC01284	-0.049	0.150551354	0.416583398
RP11-141B14	-0.049	0.150455555	0.416371104
OPRM1	-0.049	0.150448162	0.416371104
RNU6-16P	-0.049	0.150413138	0.416325436
SLC25A6P2	-0.049	0.150398583	0.416310202
CEP162	-0.049	0.150398279	0.416310202
RNA5SP447	-0.049	0.150389094	0.416310202
AP000959	-0.049	0.150346853	0.41626378
LDOC1L	-0.049	0.150343501	0.41626378
TM7SF2	-0.049	0.150334037	0.41626378
COX10	-0.049	0.150325504	0.41626378
AC104534	-0.049	0.150318452	0.41626378
RP11-361A21	-0.049	0.150308086	0.41626378
AC007899	-0.049	0.1503061	0.41626378

RP11-323I15	-0.049	0.1502813	0.41626378
CSF2RA	-0.049	0.150269662	0.41626378
AC022098	-0.049	0.150266706	0.41626378
LNX1-AS1	-0.049	0.150179306	0.416187003
RP11-639B1	-0.049	0.150149154	0.416187003
CALM2P1	-0.049	0.150148244	0.416187003
DPH1	-0.049	0.150131674	0.416187003
SIGLECL1	-0.049	0.15009152	0.41616166
RNA5SP86	-0.049	0.150049087	0.416110162
CSF1R	-0.049	0.150041646	0.416110162
RPSAP47	-0.049	0.150026858	0.416103493
RP11-274E7	-0.049	0.14999781	0.416047154
FBXW2	-0.049	0.149982322	0.416028424
BRSK2	-0.049	0.149966601	0.416028424
RBM11	-0.049	0.149959789	0.416028424
NR3C1	-0.049	0.149900722	0.415898968
ILF3	-0.049	0.149891124	0.415898968
MRM1	-0.049	0.149802754	0.41579671
MAGEB3	-0.049	0.149793956	0.415796523
CHRM5	-0.049	0.14969362	0.415566453
RAB27A	-0.049	0.149654733	0.415482715
FAM160A2	-0.049	0.149639273	0.41546401
SERINC1	-0.049	0.149616393	0.415424704
MIR610	-0.049	0.14960371	0.415413728
MTERF3	-0.049	0.149595216	0.415413728
DUX4L8	-0.049	0.149588405	0.415413728
RP11-38O14	-0.049	0.14956611	0.415406176
HKR1	-0.049	0.149559051	0.415406176
PCNPP2	-0.049	0.149551666	0.415406176
CCAT2	-0.049	0.149518793	0.415384496
AC009480	-0.049	0.149498131	0.415362705
RP11-479O9	-0.049	0.149488619	0.415360507
SNRPCP18	-0.049	0.149467618	0.415350618
PCSK2	-0.049	0.149445221	0.415312613
CYCSP2	-0.049	0.149355978	0.4151615
NUP88	-0.049	0.149265186	0.415030241
RP11-434P11	-0.049	0.149249386	0.415010539
RP11-775A3	-0.049	0.149167073	0.414830093
DUS4L	-0.049	0.149149799	0.414806275
RNU6-179P	-0.049	0.149138792	0.414799886
RP13-392I16	-0.049	0.149089429	0.41468681
LINC00339	-0.049	0.149058833	0.414650138
SHOC2	-0.049	0.149019838	0.414565878
AL161731	-0.049	0.149007845	0.414556729
CTD-2318O12	-0.049	0.149001155	0.414556729
ZNF81	-0.049	0.148997955	0.414556729
AC020983	-0.049	0.148976649	0.414556729
C1QL3	-0.049	0.148966521	0.414556729
PCAT2	-0.049	0.14896437	0.414556729
XKRY	-0.049	0.148963419	0.414556729
RNU2-34P	-0.049	0.148955905	0.414556729
PEX2	-0.049	0.148919228	0.414528117

LIPT1P1	-0.049	0.148857562	0.414404906
UBQLN4P1	-0.049	0.148825925	0.414389497
TMEM67	-0.049	0.148720933	0.414169798
CCDC28A	-0.049	0.148716625	0.414169798
MIR378J	-0.049	0.148707868	0.414169798
MIR4706	-0.049	0.148595166	0.413916363
RNU6-460P	-0.049	0.148592166	0.413916363
AC078882	-0.049	0.148522478	0.413782807
RNU1-80P	-0.049	0.148494237	0.413756217
CTC-467M3	-0.049	0.148492876	0.413756217
CCNL2	-0.049	0.148467815	0.413731006
CTB-89H12	-0.049	0.148465203	0.413731006
TUBA3GP	-0.049	0.148378753	0.41360382
MTERF1	-0.049	0.148334161	0.413527927
CCL14	-0.049	0.148320357	0.413513648
RPH3A	-0.049	0.148263652	0.413379753
NDUFAF7	-0.049	0.148254627	0.413378791
RP13-270P17	-0.049	0.148248794	0.413378791
CPA6	-0.049	0.148235237	0.413378791
POM121L14P	-0.049	0.148232325	0.413378791
FAM227B	-0.049	0.14820106	0.413378791
AF254983	-0.049	0.148182107	0.413370188
RP11-143A12	-0.049	0.148102194	0.413206097
HIST2H2AA4	-0.049	0.148068333	0.413149597
RP11-345J4	-0.049	0.147997669	0.412976621
AMFR	-0.049	0.147983105	0.412960177
FAM204A	-0.049	0.147978353	0.412960177
HLA-Z	-0.049	0.147971691	0.412960177
CTD-2012J19	-0.049	0.147952441	0.412947193
RP11-823P9	-0.049	0.147928355	0.412928366
RNU6-311P	-0.049	0.147905881	0.412889831
AC093668	-0.049	0.147896684	0.412888357
ARPC4-TTLL3	-0.049	0.147766	0.412547704
RP11-182E14	-0.049	0.14773208	0.412501364
LINC00997	-0.049	0.147702012	0.412441589
RP5-965K10	-0.049	0.147700996	0.412441589
TBC1D9B	-0.049	0.147668645	0.412420957
AD000671	-0.049	0.147663251	0.412420957
DEPDC1B	-0.049	0.147653953	0.412420957
SPINT5P	-0.049	0.14764677	0.412420957
RP11-536C5	-0.049	0.147612026	0.412359551
IRX1P1	-0.049	0.147540309	0.412183381
MELK	-0.049	0.147539211	0.412183381
RP11-517P14	-0.049	0.147499297	0.412141333
RP11-781P14	-0.049	0.147439344	0.412022157
C4orf46	-0.049	0.147437224	0.412022157
RP11-583F2	-0.049	0.147403825	0.41199542
DUTP4	-0.049	0.147375856	0.411942073
VAV3	-0.049	0.147348278	0.411912687
ZBED6	-0.049	0.14734763	0.411912687
PIGO	-0.049	0.147336972	0.411912687
OFD1P8Y	-0.049	0.147333899	0.411912687

ALG1L14P	-0.049	0.147330412	0.411912687
ZRSR1	-0.049	0.14731415	0.411912687
RP11-588L15	-0.049	0.147228096	0.411818437
AC005224	-0.049	0.147216158	0.411809229
AC005227	-0.049	0.147207555	0.411809229
SEC14L1	-0.049	0.147179821	0.411802398
FDPSP2	-0.049	0.147138121	0.411736035
MYCBP2	-0.049	0.147076193	0.411611108
RP11-709D24	-0.049	0.147059794	0.411589401
THSD7B	-0.049	0.147051619	0.411589401
RP11-754N21	-0.049	0.147048443	0.411589401
CAND1	-0.049	0.146988176	0.4115324
SMYD5	-0.049	0.146978263	0.4115324
HTR7P1	-0.049	0.146939684	0.411470857
STAG3L5P	-0.049	0.146889681	0.411379209
RP1-127B20	-0.049	0.146873803	0.411358932
SNORD121B	-0.049	0.146865044	0.411358588
RPL37P4	-0.049	0.146864757	0.411358588
ZNF211	-0.049	0.146847005	0.411356447
CTA-250D10	-0.049	0.146840985	0.411356447
LINC00633	-0.049	0.146840939	0.411356447
LTV1	-0.049	0.146836338	0.411356447
RN7SL222P	-0.049	0.146800203	0.411322113
RP11-317G22	-0.049	0.146783373	0.411299154
DYM	-0.049	0.146780572	0.411299154
AC026271	-0.049	0.146762444	0.411288903
RNU6-63P	-0.049	0.146653011	0.41107898
WDR53	-0.049	0.146600434	0.41096475
RNU6-455P	-0.049	0.146592051	0.41096475
RIOK2	-0.049	0.146544584	0.410895968
AC073046	-0.049	0.146465222	0.410721804
RNU6-1079P	-0.049	0.146404846	0.410600848
YME1L1	-0.049	0.146353723	0.410505816
RN7SKP275	-0.049	0.146345163	0.410505816
RPS5P3	-0.049	0.146323427	0.410469187
RNU6-975P	-0.049	0.146275204	0.410382253
CTB-181H17	-0.049	0.146256381	0.410382253
DISC1	-0.049	0.146254337	0.410382253
RP11-244K5	-0.049	0.14623047	0.41037762
RAE1	-0.049	0.146223499	0.41037762
RN7SL606P	-0.049	0.146218548	0.41037762
RP11-510I5	-0.049	0.146217093	0.41037762
ZNF616	-0.049	0.146205423	0.41037762
MIR3945	-0.049	0.146135117	0.410351819
CST11	-0.049	0.146129089	0.410351819
RP11-27M24	-0.049	0.146088864	0.410294513
TMEM74	-0.049	0.146071693	0.410270481
PPATP1	-0.049	0.1460697	0.410270481
AC003104	-0.050	0.145980364	0.41009147
DHX8	-0.050	0.145920846	0.409967682
SLC14A2	-0.050	0.145911651	0.409966031
AC091199	-0.050	0.145909621	0.409966031

RNU4-44P	-0.050	0.145904725	0.409966031
DCAF13P2	-0.050	0.145845438	0.409852529
SNORA84	-0.050	0.145822997	0.409813648
AC005785	-0.050	0.14580865	0.409797507
MIRLET7D	-0.050	0.145745081	0.409676457
DNM1L	-0.050	0.145717728	0.409638663
CBX1	-0.050	0.145710456	0.409638663
AC010974	-0.050	0.145696037	0.409626044
CALY	-0.050	0.145686073	0.409622209
RN7SKP260	-0.050	0.145664598	0.409610192
CXorf66	-0.050	0.145658492	0.409610192
HIST1H4F	-0.050	0.145628192	0.409580364
RNU6-1068P	-0.050	0.145621766	0.409580364
LOR	-0.050	0.145605366	0.40956454
RP11-960H2	-0.050	0.145572927	0.409497479
DNAJC12	-0.050	0.145521333	0.409384613
GLUDP5	-0.050	0.145517458	0.409384613
DDX47	-0.050	0.145496515	0.409379246
KIRREL2	-0.050	0.1454635	0.409310535
RABEPKP1	-0.050	0.145349462	0.409062153
RNA5SP187	-0.050	0.145283083	0.408899503
KIAA1875	-0.050	0.145205512	0.408753644
GFM2	-0.050	0.145204985	0.408753644
MAP2K4P1	-0.050	0.145183448	0.408753644
ITCH-AS1	-0.050	0.145172106	0.408753644
CFL1P6	-0.050	0.145170005	0.408753644
ATP5LP7	-0.050	0.145111986	0.408635283
RP11-557B9	-0.050	0.145105416	0.408635283
RP11-443K8	-0.050	0.145055196	0.408571991
RP11-605F20	-0.050	0.145014733	0.408506339
MIR548AC	-0.050	0.145011661	0.408506339
HMGN1P30	-0.050	0.144992334	0.408506339
HMGN1P36	-0.050	0.144985114	0.408506339
AC064846	-0.050	0.144974069	0.408506339
RPS15P9	-0.050	0.144889378	0.408322272
FXYD2	-0.050	0.144868723	0.408312385
WDFY3-AS1	-0.050	0.144855136	0.408298253
CTA-292E10	-0.050	0.144845272	0.408294613
RNU2-62P	-0.050	0.144844624	0.408294613
ASS1P2	-0.050	0.144843822	0.408294613
AC005808	-0.050	0.144819348	0.408294031
NBPF13P	-0.050	0.144808477	0.408287553
RNU6-471P	-0.050	0.144759701	0.408222528
H3F3B	-0.050	0.144704472	0.408115111
RNU7-138P	-0.050	0.144693789	0.408109148
IYD	-0.050	0.144666923	0.408077171
AC018878	-0.050	0.144664977	0.408077171
MITD1	-0.050	0.144644204	0.408065957
XPO5	-0.050	0.144643752	0.408065957
CREG1	-0.050	0.144641492	0.408065957
POLG	-0.050	0.144638781	0.408065957
RP11-544L8__B	-0.050	0.144615895	0.408065957

RNU6-1278P	-0.050	0.144595452	0.408049275
POU5F1P2	-0.050	0.144545817	0.407981724
KCTD9P6	-0.050	0.144531375	0.407965138
OR7E128P	-0.050	0.144518435	0.407952792
NOM1	-0.050	0.144442295	0.40783455
RP11-328J6	-0.050	0.144415515	0.407783111
PTPDC1	-0.050	0.144368722	0.407699326
NPAP1P6	-0.050	0.144329226	0.407611958
CTD-2008E3	-0.050	0.144321645	0.407611958
PIGB	-0.050	0.144311719	0.407610856
AC021876	-0.050	0.144250236	0.40746136
RP11-168C9	-0.050	0.14423273	0.407436075
HINT3	-0.050	0.144223917	0.407436075
THAP5P1	-0.050	0.144223515	0.407436075
AC068446	-0.050	0.144205789	0.407436075
EIF5P1	-0.050	0.144202712	0.407436075
ZBTB10	-0.050	0.144197915	0.407436075
CTB-39G8	-0.050	0.144191145	0.407436075
RN7SL187P	-0.050	0.144129189	0.407385216
TMCO4	-0.050	0.144091239	0.407326297
SLC25A51P3	-0.050	0.144059083	0.407259569
PP12613	-0.050	0.144005601	0.40713254
ANKRD18A	-0.050	0.143957278	0.407068412
ATG9B	-0.050	0.143931639	0.407020078
SCN4A	-0.050	0.143922934	0.407019631
USP39	-0.050	0.143918132	0.407019631
HNRNPA1P31	-0.050	0.143914419	0.407019631
TIMM21	-0.050	0.143899225	0.407019631
SMARCC1	-0.050	0.143859915	0.406962235
AC093162	-0.050	0.143843714	0.406962235
AC064871	-0.050	0.143835528	0.406962235
MEF2C	-0.050	0.143830213	0.406962235
RP3-357I16	-0.050	0.143806905	0.406957306
OR7E94P	-0.050	0.143777241	0.406897536
RP11-428O18	-0.050	0.143765563	0.406888665
HIPK1	-0.050	0.143727644	0.40680552
RP11-360A10	-0.050	0.143722915	0.40680552
SRRM5	-0.050	0.143705158	0.406790226
CTD-2501B8	-0.050	0.143607133	0.406585241
RN7SL351P	-0.050	0.14344454	0.406276106
ATP5HP4	-0.050	0.143403424	0.406226918
AC011999	-0.050	0.143321991	0.406091765
RP11-114G22	-0.050	0.143320866	0.406091765
GNPDA1	-0.050	0.143297282	0.406086176
ZNF823	-0.050	0.143277988	0.406068156
RP11-210G22	-0.050	0.143255204	0.406047467
MTND2P22	-0.050	0.143251717	0.406047467
ZSWIM3	-0.050	0.143246734	0.406047467
CTD-2600H12	-0.050	0.143210457	0.40601729
TCTA	-0.050	0.14318155	0.405959499
NUDT6	-0.050	0.14313447	0.405874339
MIR4660	-0.050	0.143117866	0.40585142

MAP9	-0.050	0.143117136	0.40585142
NPM1P21	-0.050	0.143048935	0.405752583
ENTPD5	-0.050	0.143001515	0.405642236
RP11-97C18	-0.050	0.142984697	0.405618688
GS1-306C12	-0.050	0.142971864	0.405606442
BNIP3P41	-0.050	0.142937049	0.40556388
TLCD1	-0.050	0.142927907	0.40556388
INPP5B	-0.050	0.142913727	0.405562298
RP11-787D18	-0.050	0.142905954	0.405562298
LINC00279	-0.050	0.142902799	0.405562298
AP001506	-0.050	0.142902799	0.405562298
RP11-61E11	-0.050	0.142889471	0.405562298
RP11-206M11	-0.050	0.142830851	0.405472071
RP11-2O17	-0.050	0.14279013	0.405404801
MAFG	-0.050	0.142729476	0.405280913
AC007349	-0.050	0.142722151	0.405280913
LSP1P2	-0.050	0.142701233	0.405280913
CDCA5	-0.050	0.142675056	0.405271361
FAM196A	-0.050	0.142660448	0.405254034
ANAPC16	-0.050	0.142590253	0.405102957
LINC01335	-0.050	0.14257759	0.405091146
RP11-433A19	-0.050	0.142557282	0.405081017
RP11-609N14	-0.050	0.142538462	0.405081017
OR51H2P	-0.050	0.142494999	0.40500145
MIR6514	-0.050	0.142484361	0.404995385
RN7SL594P	-0.050	0.14244121	0.404896898
RP11-521P1	-0.050	0.14243108	0.404892267
RP11-166B2	-0.050	0.142422102	0.404890911
CTD-2013M15	-0.050	0.142409571	0.404879455
LINC00293	-0.050	0.142389274	0.404845918
ZNF501	-0.050	0.142380125	0.404844072
IGHD1-20	-0.050	0.142296853	0.404655613
DRICH1	-0.050	0.142243669	0.404552682
GPR119	-0.050	0.142239132	0.404552682
FAM138B	-0.050	0.142163475	0.4043729
RP11-214N15	-0.050	0.142137256	0.40434662
NRG1-IT1	-0.050	0.142115319	0.404320385
MSRB1P1	-0.050	0.14208322	0.404265339
HNRNPABP1	-0.050	0.142076901	0.404265339
FCGR3B	-0.050	0.142037922	0.404208898
DLGAP1-AS1	-0.050	0.142027715	0.404204004
DHX30	-0.050	0.141982115	0.404098373
VWA3A	-0.050	0.141977919	0.404098373
KANSL1L	-0.050	0.141973501	0.404098373
MAPK1	-0.050	0.141842857	0.403822689
DACH1	-0.050	0.141818668	0.4038021
RNU6-1072P	-0.050	0.14181114	0.4038021
KRTAP2-1	-0.050	0.141748108	0.403649454
GLULP6	-0.050	0.141740191	0.403649454
RP1-274L7	-0.050	0.141712594	0.403596587
TXNIP	-0.050	0.141640986	0.403416771
RP11-654A16	-0.050	0.141633161	0.403416771

LRRFIP1P1	-0.050	0.14161912	0.40340274
AC090644	-0.050	0.141609825	0.40340039
SCRN3	-0.050	0.141609265	0.40340039
EIF4A1P5	-0.050	0.141490847	0.403182037
RP5-943J3	-0.050	0.141486869	0.403182037
PMS2P9	-0.050	0.141459991	0.403142353
RNU6-1318P	-0.050	0.141411163	0.403027316
RP11-311H10	-0.050	0.141396801	0.403010499
CTC-484P3	-0.050	0.141393787	0.403010499
IGKV2OR2-7	-0.050	0.141392076	0.403010499
CCSAP	-0.050	0.141359389	0.403010499
FAM105A	-0.050	0.1413178	0.402930009
LA16c-OS12	-0.050	0.141311585	0.402930009
RP11-162G10	-0.050	0.141303734	0.402930009
AC018618	-0.050	0.141293081	0.402930009
NEK2P4	-0.050	0.141290613	0.402930009
ATG3P1	-0.050	0.141267131	0.40290614
FAM206A	-0.050	0.141148731	0.402640789
GPALPP1	-0.050	0.141120937	0.402594994
RNU6-59P	-0.050	0.141118949	0.402594994
NEBL	-0.050	0.141094331	0.402582056
SLC25A29	-0.050	0.141057679	0.402501591
FCGR1B	-0.050	0.141001532	0.402389597
C5orf51	-0.050	0.140978783	0.402372895
INSL6	-0.050	0.140953974	0.402326196
RP11-298O21	-0.050	0.140946594	0.402326196
BAIAP2-AS1	-0.050	0.140938019	0.402326196
RP11-392L5	-0.050	0.140934634	0.402326196
TRAM1	-0.050	0.140904056	0.402326196
BMS1P12	-0.050	0.140884864	0.402321826
ATF4P4	-0.050	0.140853575	0.402256593
TSR1	-0.050	0.140834525	0.40225043
RNU6-617P	-0.050	0.140775948	0.402155476
RP1-177A13	-0.050	0.140735528	0.402064123
RP4-781K5	-0.050	0.140721853	0.402049172
SSX8	-0.050	0.140717852	0.402049172
RBM28	-0.050	0.140714716	0.402049172
RP11-732M18	-0.050	0.140668182	0.40196817
MIR1284	-0.050	0.140659263	0.401966802
NSUN4	-0.050	0.140615475	0.401871144
RP11-649E7	-0.050	0.140597472	0.401862558
ACTR3BP4	-0.050	0.140553706	0.401785688
BCORL1	-0.050	0.140552537	0.401785688
MUSTN1	-0.050	0.140551133	0.401785688
RP4-592A1	-0.050	0.140547077	0.401785688
NUPL2	-0.050	0.140538538	0.401785688
EIF4BP9	-0.050	0.140466713	0.401705785
CDK11A	-0.050	0.140442927	0.401661881
RP11-110J1	-0.050	0.140431418	0.401653084
MIR548K	-0.050	0.140427684	0.401653084
RPL32P3	-0.050	0.140403755	0.401646322
CTC-490G23	-0.050	0.140381846	0.401607771

RNA5SP445	-0.050	0.140376965	0.401607771
MIR509-3	-0.050	0.140345405	0.401575884
PDX1-AS1	-0.050	0.140255952	0.401440518
KIAA2018	-0.050	0.140243029	0.401427653
RP11-256G5	-0.050	0.140238948	0.401427653
RP11-746P2	-0.050	0.140231985	0.401427653
RP11-550A18	-0.050	0.140227998	0.401427653
AC004458	-0.050	0.140208351	0.401424895
MIR4795	-0.050	0.140201409	0.401424895
MICE	-0.050	0.140174772	0.401377013
BTF3L4	-0.050	0.140124194	0.40128044
RP11-30P21	-0.050	0.140110811	0.40126624
RNU6-1022P	-0.050	0.140046776	0.401155218
RCC2P7	-0.050	0.140002438	0.401052336
RP11-622J8	-0.050	0.140000808	0.401052336
RP5-882C2	-0.050	0.139958426	0.400974491
CD101	-0.050	0.139918035	0.400907007
DENND5A	-0.050	0.139890722	0.400876979
CLEC1B	-0.050	0.139862409	0.400819962
TIPIN	-0.050	0.139860182	0.400819962
LRP8	-0.050	0.139812915	0.400766733
AC027506	-0.050	0.139797401	0.400754232
RPS27P18	-0.050	0.139791372	0.400754232
AL022345	-0.050	0.139771785	0.400748501
RNU6-1180P	-0.050	0.139749164	0.400712429
RNU6-348P	-0.050	0.139728366	0.40070104
RAP1A	-0.050	0.13971387	0.400683596
ALG11	-0.050	0.1396838	0.400668598
NRXN3	-0.050	0.139680867	0.400668598
HSP90AA2P	-0.050	0.139627048	0.400627592
RNU6-418P	-0.050	0.139626623	0.400627592
CCDC50	-0.050	0.139589076	0.400591042
LRRC48	-0.050	0.139577789	0.400582787
RP11-183E24	-0.050	0.139569078	0.400581925
MIR425	-0.050	0.139511397	0.400488777
AC083863	-0.050	0.139505636	0.400488777
RN7SL165P	-0.050	0.139500613	0.400488777
RP11-166O4	-0.050	0.139499383	0.400488777
SRFBP1	-0.050	0.139473305	0.400477077
RN7SL502P	-0.050	0.1394545	0.400477077
MTND2P4	-0.050	0.139429433	0.400446577
RNU5A-5P	-0.050	0.13942891	0.400446577
CTC-492K19	-0.050	0.139409685	0.400438156
LINC01180	-0.050	0.13939468	0.400419207
MOCOS	-0.050	0.139373566	0.400382702
BNIP3P4	-0.050	0.139345373	0.400325859
JRK	-0.050	0.139327303	0.400298092
IFT74-AS1	-0.050	0.139324059	0.400298092
KPNA2	-0.050	0.139305933	0.400298092
HOXB2	-0.050	0.139304615	0.400298092
SLC38A1	-0.050	0.139292907	0.400298092
PAFAH2	-0.050	0.139286321	0.400298092

RNA5SP201	-0.050	0.139286077	0.400298092
ZNF767P	-0.050	0.139284126	0.400298092
AP000487	-0.050	0.139279443	0.400298092
SEC24D	-0.050	0.139275784	0.400298092
PTPLAD1	-0.050	0.139248567	0.400298092
RP11-421E17	-0.050	0.139186774	0.400231561
OR5K2	-0.050	0.139183779	0.400231561
TACR3	-0.050	0.139173953	0.400231561
RNU2-38P	-0.050	0.139147464	0.400231561
AJ239322	-0.050	0.139139413	0.400231561
FALEC	-0.050	0.139124669	0.400222916
OR5AC2	-0.050	0.139124187	0.400222916
RP1-32B1	-0.050	0.139118737	0.400222916
BHLHE40	-0.050	0.139079941	0.400190933
MED15P3	-0.050	0.139052656	0.400160776
RP11-10A7	-0.050	0.139041532	0.400152943
MIR6779	-0.050	0.139027508	0.400136761
NPAP1P4	-0.050	0.138956136	0.400003861
RP11-960D24	-0.050	0.138940309	0.399982478
TXNDC16	-0.050	0.138938054	0.399982478
NDST4	-0.050	0.138927783	0.399982478
SNORD37	-0.050	0.138909286	0.399979253
RP11-433O3	-0.050	0.138873347	0.399934751
NMD3P1	-0.050	0.138861642	0.399925225
TIMM9P2	-0.050	0.138850938	0.399918578
RP11-109D24	-0.050	0.138742117	0.39970184
TNPO3	-0.050	0.138728714	0.39969385
RNF169	-0.050	0.138699829	0.399676715
OR5K3	-0.050	0.138571678	0.399355762
DNAI1	-0.050	0.13856622	0.399355762
SLX1A-SULT1A3	-0.050	0.138562108	0.399355762
GAPDHP47	-0.050	0.138543382	0.399346708
PRKCD	-0.050	0.138531859	0.399337663
RP11-133L19	-0.050	0.138523589	0.399337663
NPM1P9	-0.050	0.138514147	0.399334943
TUBB4BP7	-0.050	0.138504458	0.399331184
RN7SL678P	-0.050	0.13848044	0.399286107
RP4-566L20	-0.050	0.138443959	0.399205089
C8orf46	-0.050	0.138437808	0.399205089
CTD-2547H18	-0.050	0.138334604	0.398962224
OR5AK1P	-0.050	0.138305864	0.398903494
EFTUD1	-0.050	0.138297227	0.398902739
CTC-296K1	-0.050	0.13828171	0.398882139
LILRB3	-0.050	0.138280926	0.398882139
RP11-359I18	-0.050	0.138247198	0.398830898
AC068489	-0.050	0.138224493	0.398791065
AL136123	-0.050	0.138204422	0.39877996
RP11-108M11	-0.050	0.138160103	0.398676234
RP11-945F5	-0.050	0.138127557	0.398606468
TMEM87A	-0.050	0.138123671	0.398606468
BNIP3P10	-0.050	0.138115368	0.398606468
OR7E126P	-0.050	0.138090195	0.3985711

ZNF250	-0.050	0.138053888	0.39851461
SEC23IP	-0.050	0.138033395	0.398479604
AC004920	-0.050	0.138027622	0.398479604
IGKV1D-32	-0.051	0.137988425	0.398446388
RP11-182I21	-0.051	0.137981765	0.398446388
AC096559	-0.051	0.137937678	0.39837231
CTC-422A18	-0.051	0.137936684	0.39837231
ZNF558	-0.051	0.137932706	0.39837231
ANKRD36P1	-0.051	0.13792159	0.39837231
OR1X1P	-0.051	0.13791101	0.39837231
AC024475	-0.051	0.137806917	0.398139523
RNU6-454P	-0.051	0.137775514	0.398072942
ABCB6	-0.051	0.137746732	0.398013931
RP11-203F8	-0.051	0.137736746	0.398013931
CTA-373H7	-0.051	0.137728913	0.398013931
AP006477	-0.051	0.137724526	0.398013931
LINC01136	-0.051	0.137717192	0.398013931
CH17-80A12	-0.051	0.137680652	0.39796786
RN7SL280P	-0.051	0.137651983	0.39790914
FAM214A	-0.051	0.137648525	0.39790914
ARRDC5	-0.051	0.137623909	0.397900437
ZNF33AP1	-0.051	0.13761365	0.397894932
PDCL	-0.051	0.13752979	0.397745331
STT3B	-0.051	0.137523703	0.397745331
CHKA	-0.051	0.137514863	0.397745331
MIR17HG	-0.051	0.137507473	0.397745331
DAP3P2	-0.051	0.137497282	0.397745331
RP11-2E17	-0.051	0.137478198	0.397744755
RN7SKP50	-0.051	0.137471441	0.397744755
FRG2GP	-0.051	0.137455237	0.397726644
AC009831	-0.051	0.13734836	0.39746569
PTPN9	-0.051	0.137312242	0.397388783
CIR1P2	-0.051	0.137267995	0.39735219
C2orf88	-0.051	0.137209788	0.397233638
OR7E35P	-0.051	0.137203967	0.397233638
PIBF1	-0.051	0.137193152	0.397233638
ZNF175	-0.051	0.13716972	0.397190068
PAQR5	-0.051	0.137167984	0.397190068
AC093433	-0.051	0.13713489	0.397187278
IGBP1P1	-0.051	0.137061644	0.397081748
SNORA63	-0.051	0.137057222	0.397081748
RP11-255H23	-0.051	0.137047256	0.397081748
CDKAL1	-0.051	0.137017484	0.39706308
AC110615	-0.051	0.137017152	0.39706308
ST8SIA3	-0.051	0.136948686	0.396936164
RPA2P1	-0.051	0.136944452	0.396936164
RP11-265N6	-0.051	0.13692474	0.396936164
RP11-518L10	-0.051	0.136922425	0.396936164
AC114945	-0.051	0.13692221	0.396936164
RTP4	-0.051	0.136897869	0.396933813
RP11-274J7	-0.051	0.136858729	0.396868648
AL162151	-0.051	0.136831588	0.396814105

RP11-44K6	-0.051	0.136826929	0.396814105
LINC01106	-0.051	0.136714983	0.396572538
RP11-145P16	-0.051	0.136707339	0.396572538
TRAPPC4	-0.051	0.136684265	0.396555889
ZDHHC5	-0.051	0.136667389	0.396531084
TDGP1	-0.051	0.136657219	0.396525733
RP11-131N11	-0.051	0.136631977	0.396500803
RP11-464D20	-0.051	0.136624271	0.396500803
TMC6	-0.051	0.13658938	0.396425496
RP11-120K19	-0.051	0.136568979	0.396392903
OR51A3P	-0.051	0.136562733	0.396392903
ZFP1	-0.051	0.136561702	0.396392903
UBR4	-0.051	0.136539506	0.396392903
RNASEH2B-AS1	-0.051	0.136535183	0.396392903
FSTL4	-0.051	0.136534948	0.396392903
FAM201A	-0.051	0.136505915	0.396392903
CDC42EP3P1	-0.051	0.136479278	0.396347473
MAML3	-0.051	0.136392706	0.396120215
RP11-749H17	-0.051	0.136362961	0.39605798
COMMD10	-0.051	0.136342456	0.396022574
DDIT3	-0.051	0.136250344	0.395779161
ABCD1P4	-0.051	0.136212674	0.395693873
AHCYP3	-0.051	0.136202599	0.395688738
RP11-660M5	-0.051	0.136163525	0.395599353
EIF4B	-0.051	0.136160293	0.395599353
PHF2P2	-0.051	0.136157799	0.395599353
SMIM11P1	-0.051	0.136088695	0.395454319
AC091736	-0.051	0.13608645	0.395454319
AC069181	-0.051	0.136041267	0.395364744
RP11-775C24	-0.051	0.135999007	0.395266047
TCP11L2	-0.051	0.135990674	0.395265947
RN7SKP243	-0.051	0.135986784	0.395265947
LINC01526	-0.051	0.135983645	0.395265947
RP11-493P1	-0.051	0.135959179	0.395246771
SMKR1	-0.051	0.135950736	0.39524635
OR7E5P	-0.051	0.135932573	0.395219793
HLA-C	-0.051	0.135909228	0.395212819
CTD-2547E10	-0.051	0.135881476	0.395212819
SMUG1P1	-0.051	0.135877663	0.395212819
HECTD2	-0.051	0.13579122	0.395047855
RNU6-468P	-0.051	0.135752347	0.394975941
SNRPGP5	-0.051	0.135732541	0.39494952
EIF4EP2	-0.051	0.135706765	0.394898643
RP11-204C23	-0.051	0.135687253	0.394898643
AL109658	-0.051	0.135683721	0.394898643
AKR1D1P1	-0.051	0.13566438	0.394895943
RNA5SP292	-0.051	0.135532704	0.394633252
TRY2P	-0.051	0.13552701	0.394633252
WNT9B	-0.051	0.1354908	0.394559483
LOC401913	-0.051	0.135471778	0.394528211
AC114730	-0.051	0.13543366	0.394484633
RNA5SP504	-0.051	0.135395011	0.394401113

AC016970	-0.051	0.135395011	0.394401113
RP11-433J8	-0.051	0.13538581	0.394401113
RP11-71C5	-0.051	0.135385794	0.394401113
AC090627	-0.051	0.135372277	0.394401113
AC011499	-0.051	0.135330311	0.394401113
UMAD1	-0.051	0.135328853	0.394401113
COX6B1P2	-0.051	0.135321102	0.394401113
RSF1	-0.051	0.135310675	0.394401113
CTC-1337H24	-0.051	0.135297221	0.394401113
IRX5	-0.051	0.135285631	0.394401113
AL353664	-0.051	0.135271321	0.394401113
RNU4-39P	-0.051	0.135233015	0.394363386
PPIAL4C	-0.051	0.135231652	0.394363386
PHKG1P1	-0.051	0.135224764	0.394363386
ATAD1	-0.051	0.135205512	0.394355621
METTL21AP1	-0.051	0.135201409	0.394355621
CCDC57	-0.051	0.135189922	0.394355621
EPN3	-0.051	0.135155743	0.394282896
GPR89B	-0.051	0.135148307	0.394282896
CTD-2591A1	-0.051	0.135082397	0.394117208
AC053503	-0.051	0.135077399	0.394117208
MIR8086	-0.051	0.135040972	0.394044625
RNU7-171P	-0.051	0.135036629	0.394044625
DPYS	-0.051	0.13502424	0.394044625
MIR548Y	-0.051	0.135013355	0.394044625
RNU6-1246P	-0.051	0.134978927	0.393984272
CCDC87	-0.051	0.134973232	0.393984272
RP13-150K15	-0.051	0.13496442	0.393984272
ABCD3	-0.051	0.134906393	0.393849672
CYCSP46	-0.051	0.134847033	0.39376822
CTB-111F10	-0.051	0.134842934	0.39376822
CTD-2384B11	-0.051	0.134799049	0.393724659
RP11-500K19	-0.051	0.134748111	0.393645109
FKBP4	-0.051	0.134718437	0.393585756
NXPH3	-0.051	0.1346718	0.393497782
CXADRP2	-0.051	0.134626375	0.393389187
AC019070	-0.051	0.134560039	0.393243601
UBE2V2	-0.051	0.134558755	0.393243601
RNU6-411P	-0.051	0.134548147	0.393243601
Six3os1_1	-0.051	0.134545027	0.393243601
RBM24	-0.051	0.134530468	0.393243601
RPS4XP14	-0.051	0.134527966	0.393243601
RP11-120K18	-0.051	0.134527436	0.393243601
RP11-1085N6	-0.051	0.134491413	0.393243601
KIAA1958	-0.051	0.134479254	0.393243601
CTC-340I23	-0.051	0.134458768	0.393237196
CHCHD2P4	-0.051	0.134456215	0.393237196
AL354896	-0.051	0.134419913	0.393171847
RP11-265E18	-0.051	0.134336394	0.392951692
CTAGE15	-0.051	0.134294374	0.392852907
RP11-243J18	-0.051	0.134275639	0.392822227
AF111168	-0.051	0.13417922	0.392564269

RN7SKP179	-0.051	0.13414272	0.392529819
CYP3A4	-0.051	0.134139681	0.392529819
INTS6-AS1	-0.051	0.134083854	0.392405783
RP11-308D16	-0.051	0.134065948	0.39237749
MIR1539	-0.051	0.134062702	0.39237749
C1DP2	-0.051	0.134055664	0.39237749
RP11-325K19	-0.051	0.134007212	0.392326129
LRRC10B	-0.051	0.133955425	0.392215673
FAF2	-0.051	0.133947331	0.392215673
LSM14A	-0.051	0.133926457	0.392197266
RP11-426A6	-0.051	0.133903354	0.392178772
RP11-345P4	-0.051	0.133886278	0.392178772
AL157372	-0.051	0.133783637	0.391960708
CCDC30	-0.051	0.133739556	0.391870232
MIR8055	-0.051	0.133725251	0.391870232
FBXO22	-0.051	0.133710907	0.391868153
SDCBP2-AS1	-0.051	0.133689352	0.391853208
AC078974	-0.051	0.133686768	0.391853208
ASB16-AS1	-0.051	0.133665107	0.391830371
YIPF6	-0.051	0.133654048	0.391822071
RNU6ATAC42P	-0.051	0.13363677	0.391819652
AE000660	-0.051	0.133623859	0.39180592
CTD-2331H12	-0.051	0.133596359	0.391749401
RP5-1009E24	-0.051	0.133594437	0.391749401
SH3YL1	-0.051	0.133493359	0.391592029
AC024084	-0.051	0.133348877	0.391192298
NR0B2	-0.051	0.13332033	0.391156741
AC091304	-0.051	0.133306792	0.391156741
HMGN2P24	-0.051	0.133120672	0.390715368
ENPP7P9	-0.051	0.133103108	0.390687892
HSPE1P25	-0.051	0.133039728	0.390562064
MIR5003	-0.051	0.132985753	0.390439706
BPIFB9P	-0.051	0.132983957	0.390439706
RP11-430A19	-0.051	0.132955335	0.390422574
SNORA60	-0.051	0.132841708	0.390209202
RP11-648K4	-0.051	0.132832794	0.390207085
RP11-147C23	-0.051	0.132781014	0.390079037
ATF6B	-0.051	0.132738958	0.389979541
OR10G8	-0.051	0.13269206	0.38988986
IGHVII-1-1	-0.051	0.132672723	0.389857093
C12orf40	-0.051	0.132654914	0.389828813
ERBB2IP	-0.051	0.132648026	0.389828813
BTG1	-0.051	0.132639006	0.389828813
LAPTM5	-0.051	0.132593771	0.389761313
RN7SL15P	-0.051	0.132530588	0.389631748
ABCA10	-0.051	0.132514183	0.389607567
TMCO6	-0.051	0.13247236	0.38950865
GRIPAP1	-0.051	0.132458844	0.389492954
PEBP1P3	-0.051	0.132408299	0.389392407
RP11-202D1	-0.051	0.132407267	0.389392407
RNU6-605P	-0.051	0.132352652	0.389276833
TMEM63B	-0.051	0.132349206	0.389276833

RP11-629O1	-0.051	0.132344845	0.389276833
FBP1	-0.051	0.132323039	0.389261851
MIR8056	-0.051	0.132292789	0.389244989
RP11-476H20	-0.051	0.132196248	0.389081122
MRPL32	-0.051	0.132143631	0.388974333
CTD-2647L4	-0.051	0.132134497	0.388974333
RP11-152N13	-0.051	0.132115387	0.388974333
JMJD1C	-0.051	0.132096982	0.388957216
RP11-255C15	-0.051	0.132038754	0.388852676
RP11-447D11	-0.051	0.132034809	0.388852676
SNX27	-0.051	0.132002459	0.388823125
AF015720	-0.051	0.131950751	0.388766991
AC010297	-0.051	0.131931968	0.388735701
RP11-498M14	-0.051	0.131930064	0.388735701
RNU1-32P	-0.051	0.131915485	0.388735701
SCFD2	-0.051	0.131909952	0.388735701
UTP14C	-0.051	0.131892692	0.388735701
OR10S1	-0.051	0.13179417	0.388594128
VN1R79P	-0.051	0.131759688	0.388516509
RNA5SP248	-0.051	0.131720822	0.388425955
MIR6864	-0.051	0.131699805	0.388388025
AL035457	-0.051	0.131662078	0.388324857
SGOL1P2	-0.051	0.131642496	0.388315197
RP11-106A1	-0.051	0.131638056	0.388315197
RNA5SP437	-0.051	0.131589895	0.38823217
PSMD8P1	-0.051	0.131562005	0.388173933
CCL1	-0.051	0.131539591	0.388154434
RP11-343D24	-0.051	0.131507571	0.388099497
PLAGL2	-0.051	0.131500664	0.388099497
RP11-128N14	-0.051	0.131475209	0.388062083
CENPIP1	-0.051	0.131459693	0.388040333
RP11-214O1	-0.051	0.131457094	0.388040333
TTC21A	-0.051	0.131434038	0.388012703
RP11-325L7	-0.051	0.131393338	0.387940643
BRWD3	-0.051	0.131376531	0.387915067
GRIN1	-0.051	0.131269193	0.387646195
RNU6-746P	-0.051	0.131243544	0.387594486
SNORD48	-0.051	0.131232259	0.387594486
RCOR3	-0.051	0.131225091	0.387594486
RP1-151F17	-0.051	0.131182047	0.387508983
TSIX	-0.051	0.131170121	0.387497789
NICN1	-0.051	0.131139293	0.387430748
CH17-52D20	-0.051	0.1311153	0.387383897
ANKRD20A2	-0.051	0.131105466	0.387383897
SEPHS1P7	-0.051	0.131080282	0.387376557
ST3GAL5-AS1	-0.051	0.131065136	0.387355832
KB-1507C5	-0.051	0.131058146	0.387355832
RP11-569G13	-0.051	0.131050973	0.387355832
FAM216A	-0.051	0.131040972	0.387355832
C18orf42	-0.051	0.131012948	0.387297726
RP11-616K22	-0.051	0.130987876	0.387247645
RNU4-77P	-0.051	0.13097276	0.387226993

RP11-480D4	-0.051	0.130966774	0.387226993
RPL7P60	-0.051	0.130955329	0.387223534
ENPP7P6	-0.051	0.130929753	0.387181784
NFU1	-0.051	0.130882299	0.387147043
RNU6-203P	-0.051	0.130820408	0.387088939
RNA5SP132	-0.051	0.13082034	0.387088939
C2orf57	-0.051	0.13081192	0.387088939
RAD1P2	-0.051	0.130759584	0.386981093
RP5-1168A5	-0.051	0.130685102	0.386784699
SNORD115-22	-0.051	0.130658414	0.386729743
SERPINB6	-0.051	0.130589208	0.386572951
HSD3B2	-0.051	0.130566535	0.386529858
RP13-228J13	-0.051	0.130525148	0.386431356
RP5-973N23	-0.051	0.130499401	0.386379147
STAG3L5P-PVRIG2P-PILRB	-0.051	0.130482996	0.386354595
MCM6	-0.051	0.130478821	0.386354595
IMPA1P	-0.051	0.130477594	0.386354595
ACTBP6	-0.051	0.130467644	0.386354595
HAR1B	-0.051	0.130401402	0.386257079
DOC2B	-0.051	0.130396322	0.386257079
SSH1	-0.051	0.130389229	0.386257079
RP11-296O14	-0.052	0.13037852	0.386257079
MNAT1	-0.052	0.130364057	0.386242547
PWP1	-0.052	0.130359607	0.386242547
MRPL57P6	-0.052	0.130334265	0.386225507
RNU6-751P	-0.052	0.130221232	0.385963445
RPL7AP2	-0.052	0.13014687	0.385815075
INTS4	-0.052	0.130138096	0.385813077
SAGE2P	-0.052	0.130135423	0.385813077
AC005822	-0.052	0.130055089	0.385614997
KRT43P	-0.052	0.130021124	0.385562297
NXF5	-0.052	0.130020853	0.385562297
RMDN1	-0.052	0.129916737	0.385372724
MIR1273C	-0.052	0.12989725	0.385338917
RP11-392A14	-0.052	0.12986565	0.385293174
NR1I2	-0.052	0.129839373	0.38523921
TUBGCP5	-0.052	0.129804297	0.385159133
RNU2-33P	-0.052	0.12980342	0.385159133
CYP20A1	-0.052	0.129792516	0.385159133
PRDX3P2	-0.052	0.129787159	0.385159133
HNRNPA1P19	-0.052	0.129733502	0.385127372
RP11-1277A3	-0.052	0.129699688	0.385064645
RNU1-84P	-0.052	0.12969153	0.38506443
RNU6-80P	-0.052	0.129689336	0.38506443
RN7SKP37	-0.052	0.129676383	0.38506443
LNX2	-0.052	0.129620616	0.38492587
SEH1L	-0.052	0.129618263	0.38492587
OR4N5	-0.052	0.129609862	0.38492587
AL512506	-0.052	0.129588227	0.384925687
RNU6-924P	-0.052	0.129586051	0.384925687
RP11-471G13	-0.052	0.129561298	0.384893707
LINC00377	-0.052	0.129492404	0.38473704

RNY1P16	-0.052	0.129484808	0.38473704
TRIM53BP	-0.052	0.129441262	0.384633079
TMUB2	-0.052	0.129438244	0.384633079
RTEL1P1	-0.052	0.129438202	0.384633079
ETF1P2	-0.052	0.129412711	0.384633079
PGM5P4-AS1	-0.052	0.129408374	0.384633079
SORD	-0.052	0.129387912	0.384618531
AC092162	-0.052	0.129383956	0.384618531
RPS23P5	-0.052	0.129368658	0.384609306
MAP4K2	-0.052	0.12933262	0.384550175
SAA3P	-0.052	0.129330683	0.384550175
AC012506	-0.052	0.129308152	0.384525433
AC144530	-0.052	0.12930277	0.384525433
LL22NC03-104C7	-0.052	0.129300863	0.384525433
ADCY1	-0.052	0.129279142	0.384511192
TNXB	-0.052	0.129256485	0.384491826
IQCH	-0.052	0.129210075	0.384377779
ZNF674	-0.052	0.129197646	0.384364813
RP11-2C7	-0.052	0.129153512	0.384281523
TRAJ27	-0.052	0.129127373	0.384251757
RP11-718G2	-0.052	0.129107753	0.384232575
CTD-2302E22	-0.052	0.129090656	0.384232575
ZNF658	-0.052	0.129089552	0.384232575
AP001271	-0.052	0.129059237	0.384193029
TNPO2	-0.052	0.129053398	0.384193029
RP11-688I9	-0.052	0.129022841	0.384156717
EIF2S3	-0.052	0.12901477	0.384156701
RPL23AP69	-0.052	0.129014206	0.384156701
SETP5	-0.052	0.129006548	0.384156701
RP11-276H1	-0.052	0.128980384	0.384128536
CTD-2005H7	-0.052	0.128931643	0.384053247
SS18L2P1	-0.052	0.128924891	0.384053247
RP11-421H10	-0.052	0.128888577	0.383997014
CSNK1D	-0.052	0.128866959	0.383956626
TCEB1P33	-0.052	0.128847564	0.383922857
RP11-328K2	-0.052	0.128834489	0.383907916
CPD	-0.052	0.128806329	0.383848018
MIR4670	-0.052	0.128761783	0.383763294
RP11-274A11	-0.052	0.128760853	0.383763294
LINC00427	-0.052	0.128753384	0.383763294
FUBP3	-0.052	0.128707989	0.383763294
CYCSP29	-0.052	0.128684951	0.383750431
THRAP3	-0.052	0.12867715	0.383750431
U73166	-0.052	0.128669854	0.383750431
RPSAP6	-0.052	0.128666691	0.383750431
ANKRD26P2	-0.052	0.128657978	0.383750431
TAS2R14	-0.052	0.1286148	0.383661347
RPS15AP10	-0.052	0.128608842	0.383661347
SLC30A4	-0.052	0.128538718	0.383506453
RP11-231P20	-0.052	0.128527283	0.383506453
GPR132	-0.052	0.12844961	0.383336648
RP11-503D12	-0.052	0.128412737	0.383250622

KB-1042C11	-0.052	0.128307145	0.382995673
RP3-441A12	-0.052	0.128269228	0.382942299
OR4K7P	-0.052	0.12823775	0.382872323
RP13-619I2	-0.052	0.128235263	0.382872323
RBM19	-0.052	0.128229658	0.382872323
RABGAP1	-0.052	0.128225814	0.382872323
RP11-272K23	-0.052	0.128213447	0.382872323
ZC3H4	-0.052	0.128211125	0.382872323
RN7SKP285	-0.052	0.128189917	0.382872323
RP1-288L1	-0.052	0.12817098	0.382872323
RNU6-1038P	-0.052	0.128154633	0.382872323
ADORA2A-AS1	-0.052	0.128133141	0.382847999
TTC39C-AS1	-0.052	0.128130058	0.382847999
AL391095	-0.052	0.128101663	0.382825996
RN7SL49P	-0.052	0.128083018	0.382794296
MT-TG	-0.052	0.127873047	0.382310709
AC005578	-0.052	0.127832292	0.382212854
CHAD	-0.052	0.127740388	0.382034
USP32	-0.052	0.127685506	0.381893845
RP4-635E18	-0.052	0.127622708	0.381729996
TRIM65	-0.052	0.127605064	0.381722466
ZNF781	-0.052	0.127589199	0.381701682
AP003471	-0.052	0.127588245	0.381701682
CYCSP26	-0.052	0.127572727	0.381700356
RP11-196B3	-0.052	0.127541973	0.381632316
RP11-84G21	-0.052	0.127516732	0.381620892
RP11-350G13	-0.052	0.127514703	0.381620892
ELMOD2	-0.052	0.127468894	0.381557478
SLC25A31	-0.052	0.127466463	0.381557478
RP11-46D1	-0.052	0.127454967	0.381557478
MAN2C1	-0.052	0.127453316	0.381557478
KIAA1524	-0.052	0.127444297	0.381557478
MRPL40P1	-0.052	0.127404844	0.38150962
SUOX	-0.052	0.127388148	0.38148361
RNU6-1036P	-0.052	0.12736798	0.381447196
RPL7AP56	-0.052	0.127359214	0.381444929
RP1-223D17	-0.052	0.127319819	0.381374906
CYB561	-0.052	0.127314541	0.381374906
C1orf194	-0.052	0.127236428	0.381173062
RP11-549J18	-0.052	0.127228277	0.381173062
CARTPT	-0.052	0.12721199	0.381171781
RN7SL581P	-0.052	0.127195129	0.381145238
MIR1295A	-0.052	0.127186906	0.381144578
FANCD2P2	-0.052	0.127149444	0.38105629
RP11-350N15	-0.052	0.127143694	0.38105629
ARHGAP4	-0.052	0.12712801	0.381040007
COPS7B	-0.052	0.127118191	0.381034557
LINC00621	-0.052	0.127109234	0.381031689
CNOT11	-0.052	0.126973569	0.380696894
ELK4	-0.052	0.126939375	0.380642296
RNA5SP516	-0.052	0.126930073	0.380638369
RPS17P17	-0.052	0.126924819	0.380638369

RP11-192B18		-0.052	0.126909262	0.380623894
SLC26A11		-0.052	0.12690832	0.380623894
CDC25C		-0.052	0.126860387	0.3805492
	9-Sep	-0.052	0.126858753	0.3805492
SPDYE6		-0.052	0.126842074	0.3805492
HNRNPA1P53		-0.052	0.12679425	0.380517428
NPPA		-0.052	0.126787301	0.380517428
SLC9B1P1		-0.052	0.12678216	0.380517428
RP11-197P3		-0.052	0.12677013	0.380517428
RP11-21L23		-0.052	0.126754903	0.380517428
OPA1		-0.052	0.126747011	0.380517428
LINC00441		-0.052	0.126736968	0.380517428
RP5-1107A17		-0.052	0.126726292	0.380517428
RN7SL543P		-0.052	0.126709301	0.380517428
RP11-185N2		-0.052	0.126705747	0.380517428
TCTN1		-0.052	0.126636705	0.38038133
MIR6165		-0.052	0.126630712	0.38038133
TEX10		-0.052	0.126624741	0.38038133
WARSP1		-0.052	0.126596114	0.380355356
PAAF1		-0.052	0.12657594	0.380318736
RNA5SP339		-0.052	0.126552624	0.38027267
POU5F1B		-0.052	0.126485853	0.380169976
OR5D2P		-0.052	0.126476007	0.380169976
ISL2		-0.052	0.126457508	0.380169976
RNA5SP209		-0.052	0.126431235	0.380123758
RNA5SP467		-0.052	0.126412779	0.380104943
PRKAR2A-AS1		-0.052	0.126411022	0.380104943
AC110926		-0.052	0.126387937	0.380104943
MKRN2		-0.052	0.126371108	0.380096341
OSBPL11		-0.052	0.126338669	0.380037376
DUSP5		-0.052	0.126326455	0.380024637
PGPEP1		-0.052	0.126263906	0.379860466
RP11-142J21		-0.052	0.126255559	0.379859349
RP11-202H2		-0.052	0.126243977	0.379848499
RP11-384K6		-0.052	0.126243966	0.379848499
FAM19A1		-0.052	0.126230682	0.379848499
MYO18A		-0.052	0.126185787	0.379745387
RP3-433G13		-0.052	0.126165543	0.379708456
C1QA		-0.052	0.126149589	0.379684434
C19orf45		-0.052	0.126079722	0.379546103
PCMTD1P2		-0.052	0.12604039	0.379475671
AL158040		-0.052	0.126022901	0.379447003
ANKIB1		-0.052	0.126017608	0.379447003
RP4-620F22		-0.052	0.126004172	0.379444658
MTND5P23		-0.052	0.125987356	0.379444658
EIF4G2		-0.052	0.125953553	0.3794301
SZT2		-0.052	0.125919843	0.379365828
AC096558		-0.052	0.125908245	0.379365828
RP11-5P22		-0.052	0.125889791	0.379365828
NPM1P34		-0.052	0.125889059	0.379365828
RNU6-5P		-0.052	0.125884736	0.379365828
MTND6P20		-0.052	0.125863466	0.379365828

TTLL10-AS1	-0.052	0.125758223	0.379177443
CTD-2033D15	-0.052	0.125737741	0.37913969
PHACTR3	-0.052	0.125692043	0.379025893
CTD-2373H9	-0.052	0.125623674	0.378867699
PPP5C	-0.052	0.125617715	0.378867699
CICP8	-0.052	0.125615858	0.378867699
AC009974	-0.052	0.125606547	0.378867699
RNF10	-0.052	0.125582994	0.378864969
MIR595	-0.052	0.125564497	0.378833162
REXO1L1P	-0.052	0.125556729	0.378833162
CTB-70G10	-0.052	0.125540041	0.378833162
TSPY26P	-0.052	0.125528834	0.378833162
OOSP1	-0.052	0.125518879	0.378833162
FBXO36	-0.052	0.125483197	0.378755828
HMGN2P28	-0.052	0.125467685	0.378733009
RNU6-101P	-0.052	0.125427964	0.37866111
MMEL1	-0.052	0.125338405	0.378438709
SLC10A2	-0.052	0.125322002	0.378437166
AC007389	-0.052	0.125160638	0.377997826
RP11-524F11	-0.052	0.125159652	0.377997826
RP11-50E11	-0.052	0.125114227	0.377953525
PPP1R2P10	-0.052	0.125090736	0.377918989
RP11-142M10	-0.052	0.125074039	0.377904032
CTD-2002J20	-0.052	0.125073117	0.377904032
AC004237	-0.052	0.124992315	0.377752965
RP11-585P4	-0.052	0.124984058	0.377751983
RP11-946P6	-0.052	0.124977201	0.377751983
AC018647	-0.052	0.124974809	0.377751983
RP11-429O2	-0.052	0.124922056	0.377660451
RP11-775E10	-0.052	0.124848485	0.377461994
RP11-158I3	-0.052	0.124821277	0.377403691
ATP5B	-0.052	0.124797095	0.377374024
RPS4XP3	-0.052	0.124792922	0.377374024
CTD-2116N20	-0.052	0.124775757	0.377361886
ZNF573	-0.052	0.124748575	0.377303636
RP11-62G11	-0.052	0.124692637	0.377182359
HDGFRP3	-0.052	0.124658292	0.377126373
KRBA2	-0.052	0.124605843	0.377039548
BEX5	-0.052	0.124583967	0.377012646
CENPJ	-0.052	0.124565644	0.376989766
RP11-104C4	-0.052	0.124532755	0.376962091
HNF4G	-0.052	0.124511469	0.376921614
ARL6IP1	-0.052	0.124510216	0.376921614
IFNAR1	-0.052	0.124488077	0.376898711
ISG20	-0.052	0.124364421	0.37657221
MESTP1	-0.052	0.12430989	0.376431023
VN1R7P	-0.052	0.124305547	0.376431023
RP11-1212A22	-0.052	0.124227967	0.376278644
SLC2A3P2	-0.052	0.124184627	0.37619522
RP11-503C24	-0.052	0.12414689	0.376104822
C14orf159	-0.052	0.124127304	0.376069408
LY6G5C	-0.052	0.124117933	0.376064942

SUCLA2	-0.052	0.124108028	0.376064942
OR7E25P	-0.052	0.124087223	0.376064942
MIR448	-0.052	0.124050967	0.375981635
SLC22A7	-0.052	0.124002245	0.375881804
ANKRD20A5P	-0.052	0.123991638	0.375881804
OR5H4P	-0.052	0.123968326	0.375850753
AL110292	-0.052	0.123957166	0.375840841
EIF4A2P3	-0.052	0.123949329	0.375840841
FAUP1	-0.052	0.123904505	0.375800795
RPRD2	-0.052	0.12388586	0.375768174
RP11-486O12	-0.052	0.123877573	0.375768174
CDH1	-0.052	0.12386207	0.375767808
MRPS17P5	-0.052	0.123839825	0.375724255
POLR3B	-0.052	0.123831736	0.375723648
MTND1P5	-0.052	0.123810058	0.375697391
AC011995	-0.052	0.123804793	0.375697391
MAGI1-IT1	-0.052	0.123727131	0.375525876
RNU6-595P	-0.052	0.123703924	0.375479369
MTND6P6	-0.052	0.123668009	0.375394281
AL109800	-0.052	0.12365801	0.375387853
PEBP1	-0.052	0.12365546	0.375387853
ACKR1	-0.052	0.123549209	0.375129298
GOLGA8EP	-0.052	0.123525712	0.375105783
GRM7-AS1	-0.052	0.123474873	0.374999219
AL161898	-0.052	0.123445004	0.374932417
DHFRP2	-0.052	0.123413921	0.374861916
PHF2	-0.052	0.123391678	0.374832282
MIR4677	-0.052	0.123381067	0.374832282
PJA2	-0.052	0.123361567	0.374798506
CEACAM3	-0.052	0.123346122	0.374775485
AC022021	-0.052	0.123312546	0.374697375
TRIM33	-0.052	0.123297634	0.374697375
CCNB3	-0.052	0.123269938	0.37463961
RP5-1121E10	-0.052	0.123258683	0.374629311
MIR8077	-0.052	0.123236898	0.374587001
MIR337	-0.052	0.123224011	0.374571737
RP11-445J14	-0.052	0.123217879	0.374571737
AL928742	-0.052	0.123179836	0.374496546
OR2V1	-0.052	0.123117647	0.374457698
EMC9	-0.052	0.123099472	0.374456061
GJA10	-0.052	0.123084255	0.374433688
MTND1P29	-0.053	0.123054501	0.37441492
MIR516B2	-0.053	0.123027244	0.37438532
TRIM24	-0.053	0.123012646	0.374383239
AL138479	-0.053	0.123002969	0.374377711
RN7SL664P	-0.053	0.122967314	0.374293108
TMEM155	-0.053	0.122963085	0.374293108
RP11-42L13	-0.053	0.122939324	0.37427967
ZNF627	-0.053	0.122937959	0.37427967
C5orf60	-0.053	0.122930006	0.37427967
AL137024	-0.053	0.122913214	0.37427967
RNU6-589P	-0.053	0.122909173	0.37427967

RP11-198M15	-0.053	0.122870243	0.374248032
LINC00380	-0.053	0.12286803	0.374248032
RP4-591L5	-0.053	0.122865955	0.374248032
KCNV1	-0.053	0.122799342	0.374164435
BCDIN3D-AS1	-0.053	0.122796123	0.374164435
LRIG2	-0.053	0.122769178	0.374120395
CTC-525D6	-0.053	0.122739696	0.374078421
MED28	-0.053	0.122738523	0.374078421
AC091813	-0.053	0.122723962	0.374078421
NDE1	-0.053	0.122587056	0.373804579
RP11-231C14	-0.053	0.122553555	0.373726351
PAN3-AS1	-0.053	0.122548748	0.373726351
RP11-445F6	-0.053	0.122545003	0.373726351
RPL7AL3	-0.053	0.122500674	0.373660792
OSTCP1	-0.053	0.12249956	0.373660792
KRTAP8-1	-0.053	0.12245675	0.373589347
XXbac-B444P24	-0.053	0.122441431	0.373575785
SSBP3-AS1	-0.053	0.122380251	0.373413044
EIF4BP1	-0.053	0.122360155	0.373397143
ZBTB42	-0.053	0.122352849	0.373397143
USP27X-AS1	-0.053	0.122341201	0.373389579
LIFR	-0.053	0.122318569	0.373344429
GCN1L1	-0.053	0.122313937	0.373344429
MRPL3P1	-0.053	0.1222935	0.373315763
ADAMTS7P4	-0.053	0.122281916	0.373304329
MIR596	-0.053	0.1222564	0.373250358
ALDH7A1P4	-0.053	0.12224798	0.373248577
PREX2	-0.053	0.122223179	0.373220706
AC002542	-0.053	0.122214862	0.373219237
GSTA8P	-0.053	0.122187773	0.373193528
RP11-329A6	-0.053	0.1221731	0.373187419
CHRNA6	-0.053	0.122146451	0.373153883
HLA-A	-0.053	0.122104982	0.373051124
EFR3A	-0.053	0.122095599	0.373046387
MAGOH2P	-0.053	0.12207193	0.37302193
AC073869	-0.053	0.122021455	0.372891616
PIM3	-0.053	0.122013024	0.372891616
SCYL3	-0.053	0.121939735	0.372769703
RP5-823G15	-0.053	0.121915652	0.372735702
RP11-467K18	-0.053	0.121900892	0.372735702
ANKRD63	-0.053	0.121871653	0.372672952
PGBD4P2	-0.053	0.121865187	0.372672952
VN1R104P	-0.053	0.121834323	0.372630574
AP001469	-0.053	0.121834152	0.372630574
NEDD4L	-0.053	0.121833889	0.372630574
LRRC69	-0.053	0.121824126	0.372630574
RP11-330C7	-0.053	0.121817909	0.372630574
RPL7AP15	-0.053	0.121807558	0.372630574
ITFG1-AS1	-0.053	0.121777603	0.372630574
RP11-1228E12	-0.053	0.121759733	0.372617799
RNA5SP491	-0.053	0.121756143	0.372617799
RPL7AP23	-0.053	0.121754113	0.372617799

RP11-65J3	-0.053	0.121743572	0.372617799
MIR149	-0.053	0.12172455	0.372605898
B3GNTL1	-0.053	0.121717172	0.372605898
RP11-425A6	-0.053	0.121681035	0.372520579
HSPA8	-0.053	0.121675649	0.372520579
CTD-2014E2	-0.053	0.121652413	0.372480841
AC104938	-0.053	0.121626824	0.372426433
SETP11	-0.053	0.121624979	0.372426433
AC104841	-0.053	0.121621879	0.372426433
ZC2HC1A	-0.053	0.121612399	0.372426433
COL28A1	-0.053	0.12153337	0.372283882
IGHMBP2	-0.053	0.121531528	0.372283882
RP1-276E15	-0.053	0.121493887	0.372210817
LINC00618	-0.053	0.121485411	0.372210817
ANKRD13A	-0.053	0.121448857	0.372144676
MIR3671	-0.053	0.121415954	0.372115678
RP11-76H14	-0.053	0.12140384	0.372102496
RP11-80H5	-0.053	0.12138961	0.372082826
ZNF491	-0.053	0.121337425	0.371946808
CTD-2161F6	-0.053	0.121328155	0.37194233
CRAMP1L	-0.053	0.121282988	0.371851737
RNU6-853P	-0.053	0.12125985	0.371828667
PSD4	-0.053	0.121107054	0.371431879
KIAA1731NL	-0.053	0.121097251	0.371425732
RAD23B	-0.053	0.120980777	0.3711641
GS1-358P8	-0.053	0.120956505	0.371113942
RPL19P21	-0.053	0.120946438	0.371113942
MIR4755	-0.053	0.120915298	0.37108273
HLCS	-0.053	0.120894596	0.371043106
TBC1D17	-0.053	0.120840183	0.370923911
AC062016	-0.053	0.120762288	0.370755653
RNU6-991P	-0.053	0.120757458	0.370755653
POM121L13P	-0.053	0.120743373	0.370746221
STK26	-0.053	0.120736917	0.370746221
RP11-334C17	-0.053	0.120637255	0.370492039
CTD-2026D20	-0.053	0.120597068	0.370416391
NCAPG2	-0.053	0.120580446	0.370389224
MIR629	-0.053	0.120544042	0.370301284
RP11-138A23	-0.053	0.120419432	0.369994172
FERMT3	-0.053	0.120409753	0.369994172
KRBOX4	-0.053	0.120391322	0.369994172
RP13-210D15	-0.053	0.120339386	0.369863444
REXO1L6P	-0.053	0.120334505	0.369863444
AP001001	-0.053	0.120334505	0.369863444
C7orf72	-0.053	0.12033293	0.369863444
WDR36	-0.053	0.120315136	0.369863444
RNU6-1096P	-0.053	0.12028757	0.369847391
RP11-253I19	-0.053	0.120254639	0.369793885
RP11-678B3	-0.053	0.120218279	0.369705944
AC105389	-0.053	0.120186717	0.369656622
AGBL5	-0.053	0.12017841	0.369654942
RP11-350G24	-0.053	0.120175528	0.369654942

EPS15L1	-0.053	0.120129991	0.369577615
AL022326	-0.053	0.120076277	0.369460097
RP11-506H21	-0.053	0.120031645	0.36934663
CTD-2356P16	-0.053	0.120013262	0.369313927
DHX35	-0.053	0.11994598	0.369178441
RNA5SP451	-0.053	0.11989684	0.369051043
AC073636	-0.053	0.119885006	0.369051043
SHANK2-AS1	-0.053	0.119853345	0.368988705
LGSN	-0.053	0.119850701	0.368988705
FKSG54	-0.053	0.119785302	0.368850753
RNU6-477P	-0.053	0.119756763	0.368786716
AJ239321	-0.053	0.119730835	0.368754558
C3orf52	-0.053	0.119684234	0.368682557
RNU6-195P	-0.053	0.119670728	0.368664799
CHST5	-0.053	0.119642057	0.368600314
EIF1P2	-0.053	0.119628054	0.368581016
RP11-426K3	-0.053	0.119622898	0.368581016
CHMP1B	-0.053	0.119607488	0.368565336
RNA5SP454	-0.053	0.119598643	0.368561926
SNX32	-0.053	0.1195729	0.368554135
C14orf28	-0.053	0.119515313	0.368400481
PSTPIP2	-0.053	0.119514014	0.368400481
RPL9P29	-0.053	0.119511543	0.368400481
TCEA1P2	-0.053	0.119508029	0.368400481
ZNF483	-0.053	0.119468532	0.3683546
NFAT5	-0.053	0.11945606	0.3683546
AC096971	-0.053	0.119445289	0.3683546
RPL23AP50	-0.053	0.11943025	0.368352836
RPS15AP18	-0.053	0.11942707	0.368352836
RP11-75D3	-0.053	0.119379003	0.368266324
RP11-1096G20	-0.053	0.119369534	0.368260967
RP11-61O11	-0.053	0.119297596	0.368062876
CYCSP43	-0.053	0.119294784	0.368062876
MIR1283-1	-0.053	0.11922502	0.367934298
UQCRBP1	-0.053	0.119161098	0.36776086
LINC01229	-0.053	0.11911571	0.367668432
MTATP6P4	-0.053	0.119080867	0.367608534
RP11-170K4	-0.053	0.119072046	0.367605128
RP11-49G10	-0.053	0.119058708	0.367605128
RP11-557B23	-0.053	0.119042469	0.367605128
TMEM116	-0.053	0.119028843	0.36759089
RP11-335F8	-0.053	0.118999651	0.367524569
AC002383	-0.053	0.1189524	0.367402462
RPL7P46	-0.053	0.118938731	0.367384067
RPL17P33	-0.053	0.118890328	0.367258376
OR4D11	-0.053	0.118872971	0.367229285
FKTN	-0.053	0.118855729	0.367222952
RN7SL629P	-0.053	0.118844852	0.367213169
RP11-494O16	-0.053	0.118839301	0.367213169
PLEKHG7	-0.053	0.118787168	0.367130205
GPR84	-0.053	0.118774225	0.367114026
DPEP3	-0.053	0.118749601	0.367104512

ECD	-0.053	0.118715518	0.367027847
TAS2R46	-0.053	0.118709433	0.367027847
RPL23AP22	-0.053	0.118630001	0.366834895
MIR143	-0.053	0.118612842	0.366805651
TCEB1P30	-0.053	0.118607139	0.366805651
PPIL2	-0.053	0.118580603	0.366800812
SSX2IP	-0.053	0.118546635	0.366740627
RNU6-393P	-0.053	0.118422134	0.366406221
RP11-75A5	-0.053	0.118404418	0.366375208
RP11-737G21	-0.053	0.11840026	0.366375208
ZNF32-AS1	-0.053	0.11835877	0.366329162
RNU6-32P	-0.053	0.118316717	0.366294221
MORC3	-0.053	0.118287153	0.366244546
C22orf39	-0.053	0.118253637	0.366194147
GOT2	-0.053	0.118214974	0.366126957
GCNT1P1	-0.053	0.118195842	0.366110391
ZNF572	-0.053	0.118191266	0.366110391
AC068287	-0.053	0.118180109	0.366109278
KB-1639H6	-0.053	0.118177468	0.366109278
RP5-890O3	-0.053	0.118149914	0.36606336
TMEM144	-0.053	0.118140254	0.366057244
BTBD17	-0.053	0.118108327	0.366005941
RP11-73K9	-0.053	0.118083338	0.365952311
FAM220BP	-0.053	0.118070584	0.365936599
RNU6-879P	-0.053	0.118068817	0.365936599
NKX6-3	-0.053	0.118064188	0.365936599
RP13-278H16	-0.053	0.118036211	0.365901499
KIAA0319	-0.053	0.118020464	0.365876499
BCL9P1	-0.053	0.118003504	0.365847733
AC007248	-0.053	0.117984097	0.365811379
RP11-653J6	-0.053	0.1179823	0.365811379
PTP4A1P7	-0.053	0.117971799	0.365811379
OR5AC1	-0.053	0.117969141	0.365811379
MIR654	-0.053	0.117929478	0.365784908
KRT18P39	-0.053	0.117860661	0.365609962
RP11-1144P22	-0.053	0.117756943	0.365321125
MIR6813	-0.053	0.117729569	0.365259994
MIR1227	-0.053	0.117716752	0.365259994
BMPR1APS1	-0.053	0.117702381	0.365247021
SP3P	-0.053	0.117613568	0.365018989
PDCL3P7	-0.053	0.117590759	0.36499577
CCDC78	-0.053	0.117568716	0.364951132
RP11-423C15	-0.053	0.117556205	0.364936083
snosnR66	-0.053	0.11748581	0.364812667
EFR3B	-0.053	0.117480979	0.364812667
CTB-47B8	-0.053	0.117420557	0.364698284
LRRC27	-0.053	0.117404957	0.364698284
ADIG	-0.053	0.117402526	0.364698284
ACTG1P11	-0.053	0.11739929	0.364698284
ATRX	-0.053	0.117355749	0.364622757
ADAP2	-0.053	0.117328461	0.364561756
HTR3D	-0.053	0.117309656	0.364527109

AL135878	-0.053	0.117292013	0.364496064
RNU6-363P	-0.053	0.117257309	0.364411996
AL161452	-0.053	0.117245303	0.364398463
DTWD1	-0.053	0.117240225	0.364398463
CYTH1	-0.053	0.117235488	0.364398463
KCNC1	-0.053	0.117205004	0.364392109
CR392039	-0.053	0.117202187	0.364392109
ZNF20	-0.053	0.117179424	0.364360152
AP001462	-0.053	0.117077255	0.364090005
RNA5SP112	-0.053	0.117075974	0.364090005
CTA-276F8	-0.053	0.11707529	0.364090005
RNA5SP311	-0.053	0.117070632	0.364090005
RP11-275H4	-0.053	0.117042028	0.364090005
RP11-94P14	-0.053	0.116975327	0.363986925
ME2P1	-0.053	0.116966421	0.363982994
RP11-76E12	-0.053	0.116943387	0.363935096
NVL	-0.053	0.116934299	0.363930593
OR52I1	-0.053	0.116933794	0.363930593
RP1-34P24	-0.053	0.116923404	0.363930593
HID1-AS1	-0.053	0.116915278	0.363930593
KRT18P23	-0.053	0.116898459	0.363930593
RP11-811J10	-0.053	0.116890144	0.363930593
LINC01216	-0.053	0.116860007	0.363865818
RP11-946L20	-0.053	0.116821472	0.363769616
CYP3A5	-0.053	0.116800718	0.363728768
FAM66C	-0.053	0.116785911	0.363728768
SNAPC3	-0.053	0.116768298	0.363728768
HLA-DRB9	-0.053	0.116754935	0.363728768
RP11-270M14	-0.053	0.116734193	0.363728768
ZNF112	-0.053	0.116706031	0.363671693
OR2A7	-0.053	0.116698513	0.363671693
ASH2LP1	-0.053	0.116682799	0.363646884
FEN1	-0.053	0.116660879	0.363602361
RPL5P18	-0.053	0.116649702	0.363591319
PRDX3P1	-0.053	0.116633753	0.3635654
MAGEB6P1	-0.053	0.116628094	0.3635654
RP11-427L15	-0.053	0.11660299	0.363517091
EXOC7P1	-0.053	0.116574359	0.363451623
AC096507	-0.053	0.116556958	0.36342116
RPL10AP5	-0.053	0.116545594	0.363417262
RNU6-490P	-0.053	0.116509605	0.363344876
RP5-828H9	-0.053	0.116457975	0.363255222
AF131215	-0.053	0.116410621	0.363131297
KBTBD3	-0.053	0.11639267	0.363099084
RP11-466M21	-0.053	0.116368363	0.363070816
RNA5SP403	-0.053	0.116343312	0.363029274
POMK	-0.053	0.116321654	0.362996423
AMMECR1L	-0.053	0.116311093	0.362987252
OR51G1	-0.053	0.116279918	0.36291374
NPIPA5	-0.053	0.116269175	0.362903992
DSTYK	-0.053	0.116241769	0.36284223
DBT	-0.053	0.11624037	0.36284223

NIPA2P1	-0.053	0.116160111	0.362634871
RP11-1109M24	-0.053	0.116158675	0.362634871
RP5-905N1	-0.054	0.116054323	0.362375876
AP000223	-0.054	0.116035224	0.362363755
AC137674	-0.054	0.116013292	0.36234278
FMO3	-0.054	0.115990439	0.362303456
C9orf41-AS1	-0.054	0.115956358	0.362275481
ZFP69	-0.054	0.115947027	0.362275481
RN7SL434P	-0.054	0.115928296	0.3622436
AC096921	-0.054	0.115903017	0.362188374
HNRNPKP5	-0.054	0.115895381	0.362188275
CTA-223H9	-0.054	0.11586496	0.362116967
PIK3R6	-0.054	0.115838426	0.362057796
MIR567	-0.054	0.11581918	0.3620214
KLHL1	-0.054	0.115800875	0.361987942
AC021613	-0.054	0.115775915	0.361933674
HLA-H	-0.054	0.115762858	0.361916609
RP5-821D11	-0.054	0.115747774	0.361893208
GPSM2	-0.054	0.115690116	0.361770055
RP11-149I9	-0.054	0.115625942	0.361678494
RP11-41O4	-0.054	0.115618676	0.361678494
USP9YP34	-0.054	0.115616223	0.361678494
UBE2V1P7	-0.054	0.115616223	0.361678494
CT45A6	-0.054	0.115616223	0.361678494
AC009508	-0.054	0.115604995	0.361678494
RP11-1079K10	-0.054	0.115559346	0.361636439
CHD9	-0.054	0.115509177	0.361546755
AL034553	-0.054	0.115461018	0.361423722
LINC00880	-0.054	0.115430591	0.361380412
EYA3	-0.054	0.115428087	0.361380412
RP4-785G19	-0.054	0.115422143	0.361380412
VWA7	-0.054	0.115410129	0.361380412
AMDP1	-0.054	0.115387287	0.361359174
CTA-796E4	-0.054	0.115351993	0.361304064
ZNF429	-0.054	0.115333769	0.361286597
RPS3AP23	-0.054	0.115300888	0.361207352
EBLN2	-0.054	0.115298135	0.361207352
AC078828	-0.054	0.115279235	0.361187035
ALG9	-0.054	0.115271472	0.361187035
MIR4752	-0.054	0.115267748	0.361187035
MNS1	-0.054	0.115262502	0.361187035
SETP20	-0.054	0.115259974	0.361187035
ST13	-0.054	0.115255933	0.361187035
AL136040	-0.054	0.115213908	0.361172436
MMP23A	-0.054	0.115211992	0.361172436
CCDC166	-0.054	0.115174905	0.361121473
LINC00317	-0.054	0.115171769	0.361121473
RNU6-652P	-0.054	0.115064669	0.360823345
MT-ND4	-0.054	0.115041088	0.360773155
RP11-67L14	-0.054	0.114999938	0.360667854
RP11-111K18	-0.054	0.114981966	0.360635238
RNU7-67P	-0.054	0.114977805	0.360635238

AP003498	-0.054	0.114945595	0.360577074
RP11-105C19	-0.054	0.114868157	0.360396949
NAP1L2	-0.054	0.114855687	0.360381567
TLX1NB	-0.054	0.114846274	0.360375772
ZNRD1-AS1_2	-0.054	0.114843905	0.360375772
DLG3-AS1	-0.054	0.114833288	0.360375772
MIR8078	-0.054	0.114755617	0.360186225
C10orf82	-0.054	0.114743718	0.360172613
RPL29P32	-0.054	0.114719662	0.360120836
TXNP5	-0.054	0.114695707	0.360069371
USP46	-0.054	0.114661274	0.360032469
CTRL	-0.054	0.114659435	0.360032469
MRPL42	-0.054	0.114659009	0.360032469
SLC25A17	-0.054	0.114650203	0.360032469
RNU6-19P	-0.054	0.114560321	0.359810366
UGT2B10	-0.054	0.114537931	0.359763769
RP11-285E23	-0.054	0.114524237	0.359763769
LA16c-349E10	-0.054	0.114517279	0.359763769
GTPBP10	-0.054	0.11446715	0.359660045
TMPOP1	-0.054	0.114398805	0.359492733
ART2P	-0.054	0.114364735	0.359409385
OR7E24	-0.054	0.114336699	0.359368703
RP11-333O1	-0.054	0.114322985	0.35936
TECPR1	-0.054	0.114309929	0.359355708
RNU6-100P	-0.054	0.114291457	0.359355708
OR5B19P	-0.054	0.114290087	0.359355708
ATF5	-0.054	0.114276924	0.359355708
SNX2	-0.054	0.114226615	0.359236067
RP11-15J22	-0.054	0.114203658	0.359211309
LINC01089	-0.054	0.114187108	0.35919654
CICP6	-0.054	0.114124061	0.359055798
LINC01499	-0.054	0.114121332	0.359055798
RNA5SP296	-0.054	0.11409181	0.35900176
SNRPGP15	-0.054	0.114081551	0.358993196
UBE4B	-0.054	0.114068275	0.358975138
RNU6-1256P	-0.054	0.114045166	0.35892613
TUG1	-0.054	0.114034198	0.35892613
UBE2D4	-0.054	0.114014531	0.358900865
PSMA2P1	-0.054	0.114004096	0.358891736
GUCY1B2	-0.054	0.113972819	0.358816992
AC110754	-0.054	0.11397164	0.358816992
GPR158-AS1	-0.054	0.113954153	0.358815183
ANKLE2	-0.054	0.113950791	0.358815183
AC141586	-0.054	0.113933891	0.358813027
RPL27AP	-0.054	0.113924095	0.358805905
RP13-359K18	-0.054	0.11386007	0.35865169
ASH1L	-0.054	0.11377687	0.358437028
SNORD115-12	-0.054	0.113770065	0.358437028
AC093611	-0.054	0.113770065	0.358437028
AL391867	-0.054	0.113770065	0.358437028
RNU6-1023P	-0.054	0.113770065	0.358437028
MIR5011	-0.054	0.113770065	0.358437028

AC016194	-0.054	0.113770065	0.358437028
RMRPP5	-0.054	0.113770065	0.358437028
RNU6-566P	-0.054	0.113770065	0.358437028
AC105245	-0.054	0.113770065	0.358437028
FABP12P1	-0.054	0.113770065	0.358437028
MIR3169	-0.054	0.113770065	0.358437028
AC091321	-0.054	0.113770065	0.358437028
AC134729	-0.054	0.113770065	0.358437028
AC092925	-0.054	0.113770065	0.358437028
RNU6-180P	-0.054	0.113770065	0.358437028
RNA5SP407	-0.054	0.113770065	0.358437028
MIR526A2	-0.054	0.113770065	0.358437028
RNA5SP380	-0.054	0.113770065	0.358437028
MIR4770	-0.054	0.113770065	0.358437028
KF459411	-0.054	0.113770065	0.358437028
AL590726	-0.054	0.113770065	0.358437028
MIR297	-0.054	0.113770065	0.358437028
AC016113	-0.054	0.113770065	0.358437028
RNU6-1007P	-0.054	0.113770065	0.358437028
AL356739	-0.054	0.113770065	0.358437028
AL354741	-0.054	0.113770065	0.358437028
RNU6ATAC37P	-0.054	0.113770065	0.358437028
MIR513A2	-0.054	0.113770065	0.358437028
RNU6-224P	-0.054	0.113770065	0.358437028
RNA5SP446	-0.054	0.113770065	0.358437028
AC104687	-0.054	0.113770065	0.358437028
RNU6-1230P	-0.054	0.113770065	0.358437028
MIR516A1	-0.054	0.113770065	0.358437028
RNU4-55P	-0.054	0.113770065	0.358437028
AC069243	-0.054	0.113770065	0.358437028
MIR514A3	-0.054	0.113770065	0.358437028
AC078988	-0.054	0.113770065	0.358437028
RNU6-330P	-0.054	0.113770065	0.358437028
AC019051	-0.054	0.113770065	0.358437028
RNU4ATAC8P	-0.054	0.113770065	0.358437028
AP005638	-0.054	0.113770065	0.358437028
MIR4330	-0.054	0.113770065	0.358437028
AF186996	-0.054	0.113770065	0.358437028
AC098847	-0.054	0.113770065	0.358437028
MIR4268	-0.054	0.113770065	0.358437028
AC026305	-0.054	0.113770065	0.358437028
AL109922	-0.054	0.113770065	0.358437028
RP11-656A10	-0.054	0.113770065	0.358437028
RNU6-741P	-0.054	0.113770065	0.358437028
MIR7151	-0.054	0.113770065	0.358437028
RP11-569H17	-0.054	0.113770065	0.358437028
PPATP2	-0.054	0.113770065	0.358437028
DEFB108P4	-0.054	0.113770065	0.358437028
RP11-13M3	-0.054	0.113770065	0.358437028
AP000960	-0.054	0.113770065	0.358437028
BNIP3P6	-0.054	0.113770065	0.358437028
OR6C73P	-0.054	0.113770065	0.358437028

MIR105-2	-0.054	0.113770065	0.358437028
RNU6-283P	-0.054	0.113770065	0.358437028
AC068797	-0.054	0.113770065	0.358437028
SNORD2	-0.054	0.113770065	0.358437028
RN7SKP233	-0.054	0.113770065	0.358437028
RP4-657M3	-0.054	0.113770065	0.358437028
RP11-736K12	-0.054	0.113770065	0.358437028
AC093816	-0.054	0.113770065	0.358437028
AP003972	-0.054	0.113770065	0.358437028
AC097361	-0.054	0.113770065	0.358437028
MIR382	-0.054	0.113770065	0.358437028
AC034205	-0.054	0.113770065	0.358437028
AC004769	-0.054	0.113770065	0.358437028
RNU6-639P	-0.054	0.113770065	0.358437028
AL591415	-0.054	0.113770065	0.358437028
RNU6-72P	-0.054	0.113770065	0.358437028
RPEP5	-0.054	0.113770065	0.358437028
AC108739	-0.054	0.113770065	0.358437028
AC108211	-0.054	0.113770065	0.358437028
MIR3924	-0.054	0.113770065	0.358437028
MIR1912	-0.054	0.113770065	0.358437028
AC106860	-0.054	0.113770065	0.358437028
RP11-534P19	-0.054	0.113770065	0.358437028
C10orf32-ASMT	-0.054	0.113764581	0.358437028
LINC01496	-0.054	0.113715656	0.358437028
RNU6-622P	-0.054	0.113663317	0.358437028
NUDT19P5	-0.054	0.113639706	0.358437028
HMGN2P15	-0.054	0.113563506	0.358437028
C11orf80	-0.054	0.113524368	0.358437028
RPL7P31	-0.054	0.113520342	0.358437028
IGHVII-74-1	-0.054	0.113516818	0.358437028
RNU1-132P	-0.054	0.113483649	0.358437028
MIR1271	-0.054	0.1134447	0.358437028
RNU4-24P	-0.054	0.113428886	0.358437028
RP13-16H11	-0.054	0.11341633	0.358437028
RP11-751A18	-0.054	0.113403002	0.358437028
RPL15P18	-0.054	0.113389868	0.358437028
CLUHP2	-0.054	0.113338263	0.358437028
FAM120A	-0.054	0.113322784	0.358437028
AC005538	-0.054	0.113316088	0.358437028
RN7SL198P	-0.054	0.113262761	0.358437028
FBXO11	-0.054	0.113234929	0.358437028
ATXN7L2	-0.054	0.113166426	0.358437028
AC073343	-0.054	0.113142181	0.358437028
HTR4	-0.054	0.113131656	0.358437028
OR10J5	-0.054	0.113108583	0.358437028
RP11-412P11	-0.054	0.113064378	0.358437028
FDPSP8	-0.054	0.113043302	0.358437028
RP11-338E21	-0.054	0.113017136	0.358437028
RP11-398E10	-0.054	0.112989914	0.358437028
MIR3128	-0.054	0.112929274	0.358437028
DDX18P1	-0.054	0.112840899	0.358437028

DLGAP1-AS3	-0.054	0.112820245	0.358437028
LINC01189	-0.054	0.112741856	0.358328754
LHX3	-0.054	0.112739645	0.358328754
RNU7-74P	-0.054	0.112691931	0.358217886
RPL9P16	-0.054	0.112686337	0.358217886
AC021887	-0.054	0.112682666	0.358217886
RP11-428K3	-0.054	0.112682602	0.358217886
ZNF322	-0.054	0.112680375	0.358217886
MAP3K2	-0.054	0.112652579	0.358212338
RP13-890H12	-0.054	0.112617712	0.358125376
EVL	-0.054	0.112599237	0.358090535
MACROD2	-0.054	0.112597873	0.358090535
SGPP1	-0.054	0.112553726	0.358017517
PDK2	-0.054	0.112536368	0.357986214
RP5-1159O4	-0.054	0.112522979	0.35798173
RPL37P1	-0.054	0.11250887	0.35798173
CLRN1-AS1	-0.054	0.112488598	0.357953795
AC013444	-0.054	0.112461383	0.357891104
KATNBL1	-0.054	0.11242917	0.357860328
TMEM194B	-0.054	0.112366748	0.35770945
RP11-528I4	-0.054	0.112363111	0.35770945
STARD5	-0.054	0.112361547	0.35770945
AC092881	-0.054	0.112357675	0.35770945
CTD-2086O20	-0.054	0.112324345	0.357670092
OR4A49P	-0.054	0.112300764	0.357618916
YRDCP2	-0.054	0.112271253	0.357572931
AC062029	-0.054	0.112253738	0.357564792
EIF4BP6	-0.054	0.112253119	0.357564792
ROBO2	-0.054	0.112172875	0.357378923
RP4-728D4	-0.054	0.112140121	0.357346283
RP11-344J7	-0.054	0.112115333	0.35733902
UPF3AP2	-0.054	0.112092158	0.357289065
MIR3657	-0.054	0.111945102	0.356868097
TNPO1	-0.054	0.111942774	0.356868097
RPL17P44	-0.054	0.111926277	0.356868097
AC118653	-0.054	0.111891627	0.356840936
SYT15	-0.054	0.111844164	0.356737344
RP11-151A6	-0.054	0.111844142	0.356737344
Mico1	-0.054	0.111831631	0.356737344
MIRLET7G	-0.054	0.111723363	0.356471402
EFCAB6	-0.054	0.111707532	0.356468652
RP11-440I14	-0.054	0.111699122	0.356465698
AP1S2	-0.054	0.111645055	0.356364792
RP11-363L24	-0.054	0.111555262	0.35612591
PHLPP1	-0.054	0.111475509	0.355942886
RP11-596C23	-0.054	0.111474959	0.355942886
AL356865	-0.054	0.111471477	0.355942886
RP11-671J11	-0.054	0.111458169	0.355942886
C21orf62-AS1	-0.054	0.111445242	0.355942886
RNU6-144P	-0.054	0.111427285	0.355942886
AL121923	-0.054	0.111401754	0.355922139
RPL17-C18orf32	-0.054	0.111372613	0.355876782

RP11-417F21	-0.054	0.111344097	0.355866871
LRTM2	-0.054	0.111306409	0.355808467
TMEM50A	-0.054	0.111294736	0.355800141
KLHL28	-0.054	0.111269422	0.355761867
RP11-202A13	-0.054	0.111205658	0.355581865
MCCC2	-0.054	0.111178279	0.355542059
AC068121	-0.054	0.111177432	0.355542059
AC132807	-0.054	0.111171085	0.355542059
CT45A3	-0.054	0.111167462	0.355542059
COQ6	-0.054	0.111092962	0.355388535
LYSMD3	-0.054	0.111049403	0.355328369
DEFB106A	-0.054	0.111026859	0.355296394
RP11-585K14	-0.054	0.111022531	0.355296394
RP11-227P3	-0.054	0.110976793	0.355207777
RP11-174N3	-0.054	0.110963979	0.355190631
CD163	-0.054	0.110897726	0.355013798
CTC-359D24	-0.054	0.110888781	0.355013798
RP11-50B3	-0.054	0.1108506	0.354998887
GHRL	-0.054	0.11072932	0.354654024
PREX1	-0.054	0.110712185	0.354622989
RMND5B	-0.054	0.11068049	0.354545311
RP11-322L17	-0.054	0.110625092	0.354391689
CENPF	-0.054	0.110616632	0.354388424
APC	-0.054	0.110607826	0.354384051
MIR3612	-0.054	0.110607824	0.354384051
RP13-88F20	-0.054	0.110569953	0.354338026
TANGO6	-0.054	0.11048998	0.354197098
RP11-476F14	-0.054	0.110487985	0.354197098
RN7SL76P	-0.054	0.110475223	0.354197098
GS1-214D18	-0.054	0.110461034	0.354197098
KLHL6-AS1	-0.054	0.110450538	0.354197098
GLUD2	-0.054	0.110429751	0.354194743
KRT8P51	-0.054	0.110420426	0.354188688
AC007246	-0.054	0.110406179	0.354166842
CTC-261N6	-0.054	0.110377369	0.354098271
DTNA	-0.054	0.110360879	0.354069222
PRM1	-0.054	0.110337663	0.354018586
KRT36	-0.054	0.110330124	0.354018586
AC010329	-0.054	0.110300013	0.353993177
RN7SL368P	-0.054	0.110299011	0.353993177
CTB-3M24	-0.054	0.110297533	0.353993177
RP11-578O24	-0.054	0.110291763	0.353993177
VN1R32P	-0.054	0.110270981	0.353993177
GAS2	-0.054	0.110221408	0.353907841
AC005828	-0.054	0.110218434	0.353907841
RP11-197O8	-0.054	0.110201711	0.353892313
HLA-K	-0.054	0.110198907	0.353892313
RNU7-94P	-0.054	0.110147334	0.353765397
CTD-2515H24	-0.054	0.110100896	0.353687804
ZNF420	-0.054	0.110084879	0.353660204
AC016697	-0.054	0.110022781	0.353508398
TXNDC12-AS1	-0.054	0.110019928	0.353508398

RP11-197M22	-0.054	0.110005005	0.353498978
RP11-203B9	-0.054	0.109975142	0.353426863
AC241377	-0.054	0.109934402	0.353367468
RP11-161N3	-0.054	0.109927916	0.353367468
AP000560	-0.054	0.109910839	0.353363274
MIR1275	-0.054	0.109901426	0.353356862
MIR7848	-0.054	0.109853088	0.353296849
ARHGAP17	-0.054	0.109851747	0.353296849
RP11-264K23	-0.054	0.109844143	0.353296849
SCARNA12	-0.054	0.109828035	0.353296849
USP49	-0.054	0.109817692	0.353296849
HSPE1P23	-0.054	0.109808993	0.353296849
CCNB1IP1P3	-0.054	0.109795561	0.353296849
KDM6A	-0.054	0.109777081	0.353290967
ANKRD42	-0.054	0.10967281	0.353026962
AC093911	-0.054	0.109651396	0.352981888
AC067940	-0.054	0.109569366	0.35278936
AC090607	-0.054	0.109542699	0.352775044
PNPO	-0.054	0.10953172	0.35276354
ZBTB43	-0.054	0.109519949	0.352749482
RPL13AP6	-0.054	0.109489218	0.352674353
ALDOB	-0.054	0.109478729	0.352664418
AC010368	-0.054	0.109468606	0.35265566
RP11-338I21	-0.055	0.109398249	0.352476687
SPRY4-IT1	-0.055	0.109390282	0.352474863
ZNF57	-0.055	0.109350165	0.352393282
RABGGTB	-0.055	0.109300119	0.352268284
ZNF862	-0.055	0.10920916	0.352177174
MIR3927	-0.055	0.109184421	0.352121237
OR6M3P	-0.055	0.109155578	0.352099743
RUSC1-AS1	-0.055	0.109140164	0.352073868
NPM1P32	-0.055	0.109113203	0.352010737
SV2B	-0.055	0.109104106	0.352005233
LINC00271	-0.055	0.108998043	0.351758356
COX6B1P6	-0.055	0.108991584	0.351758356
HMGN2P13	-0.055	0.108961891	0.351689346
AXIN2	-0.055	0.10895175	0.351680446
RNA5SP35	-0.055	0.108909635	0.351568333
SLCO1B1	-0.055	0.10888869	0.351548375
PMS2CL	-0.055	0.108881553	0.351548375
SP140L	-0.055	0.108879017	0.351548375
AC074035	-0.055	0.108878791	0.351548375
RNU6-1025P	-0.055	0.108828266	0.351448593
PAQR3	-0.055	0.108803787	0.351393364
FAM27E4	-0.055	0.108743143	0.351235666
RP11-109D20	-0.055	0.108739081	0.351235666
RP3-341D10	-0.055	0.108665321	0.351065203
RP11-55K22	-0.055	0.108651473	0.351044277
RP11-123K19	-0.055	0.108637335	0.351022413
SEL1L	-0.055	0.108619207	0.350987648
TMEM260	-0.055	0.108597869	0.35094251
NF1P5	-0.055	0.108585127	0.350925143

RNU4-49P	-0.055	0.108564219	0.350889248
CTD-2320G14	-0.055	0.108540974	0.350877688
RP11-188P17	-0.055	0.108529972	0.350865936
MIR3683	-0.055	0.108518584	0.350852937
RP11-668G10	-0.055	0.108460661	0.350713279
AC012476	-0.055	0.108459106	0.350713279
RN7SL647P	-0.055	0.108455574	0.350713279
RP11-299H21	-0.055	0.108414047	0.350705413
STMN1P1	-0.055	0.108399269	0.350681429
MGAT2P2	-0.055	0.10839417	0.350681429
RP4-785J1	-0.055	0.108374426	0.350672518
MIR218-1	-0.055	0.108318409	0.35053889
AC015977	-0.055	0.108211925	0.350266604
GS1-541M1	-0.055	0.1081727	0.350233934
AL121899	-0.055	0.108111188	0.350082373
FAM92A1P2	-0.055	0.108108934	0.350082373
TDRD6	-0.055	0.108101114	0.350082373
RP11-486E2	-0.055	0.108042498	0.349955118
KIAA0895	-0.055	0.108000565	0.349866882
TPMTP1	-0.055	0.107970638	0.349841322
AC083822	-0.055	0.107956391	0.349818956
CH507-9B2	-0.055	0.107927254	0.349748338
TIMM9P1	-0.055	0.107902894	0.349716989
RP1-15D23	-0.055	0.107891642	0.349704317
HIST3H2BA	-0.055	0.10788463	0.349704317
TLR9	-0.055	0.10787706	0.349704317
RNU6-326P	-0.055	0.107873065	0.349704317
MIR641	-0.055	0.107860566	0.349698787
KIF13A	-0.055	0.107843073	0.349665876
MESTP2	-0.055	0.107792347	0.349525197
LINC00102	-0.055	0.107790184	0.349525197
AL035250	-0.055	0.107784372	0.349525197
PDCL2P2	-0.055	0.10757256	0.348978839
ITIH2	-0.055	0.107566972	0.348978839
CCDC40	-0.055	0.10753318	0.348898615
SNORA3	-0.055	0.107525609	0.348898615
MIR558	-0.055	0.107504079	0.348898615
MIR592	-0.055	0.107493214	0.348887782
RN7SL316P	-0.055	0.107493017	0.348887782
ZSCAN5D	-0.055	0.107471414	0.348864574
TRAV7	-0.055	0.107453178	0.348858204
RP5-893G23	-0.055	0.107360654	0.348600073
STAB2	-0.055	0.107294718	0.348409731
GID4	-0.055	0.107286059	0.34840537
AC005301	-0.055	0.107257096	0.348335064
FAM63B	-0.055	0.107235329	0.348288121
AC092687	-0.055	0.107198805	0.348216989
RP4-754E20__A	-0.055	0.107190478	0.348213689
RP11-522L3	-0.055	0.107141082	0.348076965
CTD-2141G3	-0.055	0.107134249	0.348076965
ECT2	-0.055	0.107115918	0.348042695
ZSCAN12P1	-0.055	0.107055477	0.347893778

SNX13	-0.055	0.1070376	0.34785942
PTMAP4	-0.055	0.107026518	0.347847139
LARP4P	-0.055	0.107015954	0.34783654
MIR4672	-0.055	0.107005713	0.34783654
SNORA26	-0.055	0.107005184	0.34783654
RP11-288C18	-0.055	0.106994229	0.34783654
ENPP7P2	-0.055	0.106956521	0.347785774
RP11-11N7	-0.055	0.106911934	0.347688269
SHMT1P1	-0.055	0.106896579	0.347662071
AC073130	-0.055	0.106865154	0.347583601
ETAA1	-0.055	0.106835282	0.347543974
SCARNA15	-0.055	0.106827422	0.347543974
ARFIP1	-0.055	0.106806251	0.347510679
CTD-2587H24	-0.055	0.106795544	0.347499583
ISG20L2	-0.055	0.106780081	0.347473006
RP11-392O1	-0.055	0.106777604	0.347473006
RP11-166N17	-0.055	0.10677211	0.347473006
MTCO2P1	-0.055	0.106762309	0.347473006
CTD-2024F21	-0.055	0.106740976	0.347463862
MIR6812	-0.055	0.10673864	0.347463862
RP11-325P15	-0.055	0.106604677	0.347091942
AP001258	-0.055	0.106604516	0.347091942
RP11-305P14	-0.055	0.106581825	0.347064991
OR10U1P	-0.055	0.10656294	0.347050953
OR4G2P	-0.055	0.106538513	0.34701886
RP11-466G12	-0.055	0.106474282	0.346857087
RP11-343C2	-0.055	0.106438787	0.346765176
KIZ-AS1	-0.055	0.106428863	0.346756565
AKAP8	-0.055	0.106408895	0.346738946
RP11-481J2	-0.055	0.106365963	0.346665836
RNU6-73P	-0.055	0.106341448	0.346614027
SCARNA22	-0.055	0.106312011	0.346552313
OR10J4	-0.055	0.106300382	0.346552313
RP1-197O17	-0.055	0.106222421	0.346415725
PPIAP25	-0.055	0.106219975	0.346415725
RP11-125M16	-0.055	0.106214888	0.346415725
USP34	-0.055	0.10620503	0.346415725
MMS22L	-0.055	0.106203964	0.346415725
ATP9B	-0.055	0.106200411	0.346415725
MBNL1	-0.055	0.106193187	0.346415725
RPL12P18	-0.055	0.106182488	0.346415725
RNU6ATAC36P	-0.055	0.106169538	0.346415725
TRIM51CP	-0.055	0.106140937	0.346387172
RCC2P2	-0.055	0.106133294	0.346385964
ITGB2	-0.055	0.106083806	0.346271911
SIDT1-AS1	-0.055	0.106070717	0.346252914
BTBD7P2	-0.055	0.106037189	0.346190925
TMEM106C	-0.055	0.105957845	0.346026762
FRMPD2	-0.055	0.105912989	0.345903992
STRCP1	-0.055	0.105907036	0.345903992
AC245036	-0.055	0.105900888	0.345903992
CHCHD4	-0.055	0.105888233	0.345903992

ATP8B4	-0.055	0.105864013	0.345862626
POTEB2	-0.055	0.10583732	0.345839265
AC084193	-0.055	0.105821136	0.345817431
C5orf24	-0.055	0.1058098	0.345804114
CCDC183-AS1	-0.055	0.10580673	0.345804114
AL645859	-0.055	0.105800015	0.345804114
OR4D10	-0.055	0.105789312	0.345804114
GS1-466O4	-0.055	0.105778662	0.345797264
AC114302	-0.055	0.105734108	0.34569907
PDCD6IP	-0.055	0.105723271	0.345687368
RP11-757H14	-0.055	0.105702476	0.345643104
CYB561D1	-0.055	0.105690586	0.345627954
FAM157A	-0.055	0.105654756	0.34558197
CYP2B7P	-0.055	0.105635743	0.345543511
AC004156	-0.055	0.105628037	0.345542035
LYPLA1P1	-0.055	0.105519483	0.345305511
KCTD9P3	-0.055	0.105511539	0.345303242
PTK2	-0.055	0.105498885	0.345285556
C1orf167	-0.055	0.105465057	0.345200633
AL713997	-0.055	0.105460263	0.345200633
C17orf85	-0.055	0.105452946	0.345200633
KANTR	-0.055	0.105384303	0.34509521
UPK3BP1	-0.055	0.105334948	0.344962357
CTB-114C7	-0.055	0.105332238	0.344962357
RNVU1-20	-0.055	0.105310041	0.344951947
VPS26B	-0.055	0.105303814	0.344951947
ZNF750	-0.055	0.105292285	0.344941235
RP11-333E1	-0.055	0.1051196	0.344493993
ZNF402P	-0.055	0.105109351	0.344484109
ANKRD18B	-0.055	0.105098064	0.344484109
LYAR	-0.055	0.105070387	0.344427511
MEAF6	-0.055	0.105057117	0.344407718
MKS1	-0.055	0.105045963	0.344394856
RP11-293B7	-0.055	0.105025993	0.344376797
RP11-382H24	-0.055	0.105017485	0.344372608
RP11-139B1	-0.055	0.105007965	0.344365101
PIP5K1P1	-0.055	0.104950539	0.344200476
RP11-151M7	-0.055	0.104928602	0.344152229
BRAF	-0.055	0.104928369	0.344152229
ZCCHC3	-0.055	0.104910774	0.344152229
IGHVII-26-2	-0.055	0.104868836	0.344050979
RN7SL30P	-0.055	0.104860072	0.344050979
RP11-769O8	-0.055	0.104814577	0.343944043
UPF3B	-0.055	0.104810411	0.343944043
MOB4	-0.055	0.104805254	0.343944043
MIR4729	-0.055	0.104776513	0.343913921
RNU4-17P	-0.055	0.104755306	0.343868009
LINC01441	-0.055	0.104746392	0.343868009
AC018804	-0.055	0.104722655	0.343831924
RP11-267D19	-0.055	0.104710424	0.343815469
BTNL8	-0.055	0.104696677	0.343794031
AHCYL1	-0.055	0.104655911	0.343723028

UMOD	-0.055	0.104623964	0.343721151
HMGB1P44	-0.055	0.104605524	0.343684277
SH3GLB2	-0.055	0.104554805	0.343588745
RP11-286B5	-0.055	0.104528283	0.343564509
CPVL	-0.055	0.104522523	0.343564509
HMGN1P11	-0.055	0.104473216	0.343444436
SBDSP1	-0.055	0.104470258	0.343444436
AC027319	-0.055	0.104465479	0.343444436
IL3	-0.055	0.104458239	0.343444436
UBL3	-0.055	0.104449101	0.343444436
RP11-152C15	-0.055	0.104422124	0.343413376
NUP50-AS1	-0.055	0.104421753	0.343413376
IPPKP1	-0.055	0.10441965	0.343413376
MAP2K4	-0.055	0.104419066	0.343413376
MIR325	-0.055	0.104407972	0.343413376
CDC40	-0.055	0.10440037	0.343413376
TMC3	-0.055	0.104386462	0.343413376
RP11-486M3	-0.055	0.104377706	0.343413376
RNU6-633P	-0.055	0.104368264	0.343413376
ZNF34	-0.055	0.104358081	0.343413376
RFC3	-0.055	0.104303896	0.343261586
RNU6-136P	-0.055	0.104277483	0.343198376
MTERF2	-0.055	0.104245066	0.343115394
RP1-150O5	-0.055	0.104201443	0.343019223
SNORA73	-0.055	0.104200384	0.343019223
RP11-476I15	-0.055	0.104190986	0.343019223
MIR3199-2	-0.055	0.104187315	0.343019223
RP1-159M24	-0.055	0.104184702	0.343019223
ARL5A	-0.055	0.104150339	0.342969521
RP11-137N23	-0.055	0.104131184	0.342937261
MIR3170	-0.055	0.104128763	0.342937261
PPP1R8	-0.055	0.104128183	0.342937261
CSE1L	-0.055	0.104102884	0.342931807
NBEA	-0.055	0.104081902	0.342910124
RP11-1152B5	-0.055	0.104039951	0.342795623
AC016683	-0.055	0.104001951	0.342694128
ZBED4	-0.055	0.103995795	0.342694128
HNRNPA3P3	-0.055	0.103967551	0.342628187
HYALP1	-0.055	0.103911068	0.342465743
STIP1	-0.055	0.103906137	0.342465743
ZDHHC20-IT1	-0.055	0.103898048	0.342465743
RP11-342D14	-0.055	0.103859114	0.342412989
RP4-591C20	-0.055	0.103856676	0.342412989
PRAMEF20	-0.055	0.10384786	0.342412989
RP11-54P19	-0.055	0.103819328	0.342352915
MARK2P5	-0.055	0.103769657	0.342236511
RN7SL110P	-0.055	0.103768821	0.342236511
OTOGL	-0.055	0.103737039	0.342210634
C6orf163	-0.055	0.103724446	0.342210634
FANCB	-0.055	0.103691787	0.342169246
VDAC1P13	-0.055	0.103640962	0.342048929
RP11-10O17	-0.055	0.103607562	0.341965078

RNU6-782P	-0.055	0.103567675	0.341901826
GABARAPL2	-0.055	0.103560536	0.341901826
ATG4C	-0.055	0.103552758	0.341899979
RP11-348N5	-0.055	0.103550204	0.341899979
RIPPLY3	-0.055	0.10350517	0.341813945
CPT2	-0.055	0.103483009	0.341808853
ZNF561	-0.055	0.103468507	0.341808853
AMDHD1	-0.055	0.103442407	0.341777742
ABI1	-0.055	0.103424314	0.341777742
RP11-1430O6	-0.055	0.103419752	0.341777742
RP11-463C8	-0.055	0.103377111	0.341675343
ALOX12-AS1	-0.055	0.103374108	0.341675343
AC010092	-0.055	0.103364995	0.341675343
GOLGA8O	-0.055	0.103318224	0.341575529
RP11-6C10	-0.055	0.10326232	0.341414405
NR1H3	-0.055	0.103243755	0.341400426
RP11-43D4	-0.055	0.10324099	0.341400426
FAM133DP	-0.055	0.10322952	0.341400426
RP11-21A7A	-0.055	0.103218816	0.341400426
MTND6P14	-0.055	0.103212648	0.341400426
OR5AN2P	-0.055	0.103154812	0.341295883
RNA5SP341	-0.055	0.103129584	0.341248415
PMS2	-0.055	0.103115037	0.3412354
RP11-1193F23	-0.055	0.10309589	0.341195743
RP11-557C18	-0.055	0.103089231	0.341195743
AC006994	-0.055	0.103069261	0.341178739
RP11-471N19	-0.056	0.103030121	0.341096593
RP11-814E24	-0.056	0.102994916	0.341003743
MED17	-0.056	0.10295039	0.340895708
ZNF292	-0.056	0.102921737	0.340832538
RP11-734B5	-0.056	0.102920764	0.340832538
POLD3	-0.056	0.102863526	0.340734534
RP11-202I11	-0.056	0.102848735	0.340709238
PIGL	-0.056	0.102782143	0.340536009
CDC42SE1	-0.056	0.10276645	0.340507702
RP1-186E20	-0.056	0.102758426	0.340504804
SNX3P1X	-0.056	0.102749231	0.340504804
ST13P4	-0.056	0.102705946	0.340401954
RP5-894D12	-0.056	0.102692864	0.340382282
SLX4IP	-0.056	0.102685244	0.340380714
DLG3	-0.056	0.102683399	0.340380714
MAP3K12	-0.056	0.102676711	0.340380714
AC069513	-0.056	0.102670586	0.340380714
AC124074	-0.056	0.102661038	0.340380714
ZFX-AS1	-0.056	0.10264984	0.340380714
AC090206	-0.056	0.102543025	0.340098652
RP11-181D18	-0.056	0.102539938	0.340098652
PIGW	-0.056	0.102500644	0.34007609
RGSL1	-0.056	0.102469574	0.339997102
EXOC5P1	-0.056	0.10243992	0.339922389
TSPAN31	-0.056	0.102430077	0.339914938
IL1B	-0.056	0.102370016	0.339809179

SIRT5	-0.056	0.10236576	0.339809179
MIR1302-11	-0.056	0.1023484	0.339809179
LANCL1	-0.056	0.102346321	0.339809179
NPIPB1P	-0.056	0.102339848	0.339809179
RP4-641G12	-0.056	0.102332284	0.339809179
RP11-420A21	-0.056	0.102312405	0.339809179
POLK	-0.056	0.102265034	0.339697084
AC093690	-0.056	0.102256704	0.339693106
ENTPD3-AS1	-0.056	0.102256299	0.339693106
FGR	-0.056	0.10223975	0.339684171
RNU6-616P	-0.056	0.102212424	0.339617072
MTND2P23	-0.056	0.102210654	0.339617072
RP11-314D7	-0.056	0.102172239	0.339544223
PCSK6-AS1	-0.056	0.102123937	0.339441466
RNA5SP34	-0.056	0.102112686	0.339435107
AC235953	-0.056	0.10206257	0.339308532
BRD2-IT1	-0.056	0.102023135	0.339201108
RNU6-495P	-0.056	0.102020586	0.339201108
RP11-60A24	-0.056	0.101980789	0.339111826
RP11-487I5	-0.056	0.10197142	0.339111826
SMARCD1	-0.056	0.101959661	0.339111826
AC097662	-0.056	0.101912427	0.338998686
MUC2	-0.056	0.101887399	0.338939103
DSCR10	-0.056	0.101840376	0.338806341
RNU6-157P	-0.056	0.101823074	0.338772446
ZNF385C	-0.056	0.101785487	0.338694712
RP11-90L20	-0.056	0.101745143	0.338584121
TSPY20P	-0.056	0.101711769	0.338517643
PRKACB	-0.056	0.101710277	0.338517643
ACVR2B-AS1	-0.056	0.101650208	0.338433727
RP11-9M16	-0.056	0.101628328	0.338384533
CTC-360P9	-0.056	0.101569782	0.338236891
TCEB1P4	-0.056	0.101569601	0.338236891
IL23R	-0.056	0.10150987	0.338084655
MILR1	-0.056	0.101477826	0.338001567
BTBD10	-0.056	0.101442075	0.337906121
RP11-355F16	-0.056	0.101424261	0.337870412
RP11-246A10	-0.056	0.101377576	0.337738515
ADCK3	-0.056	0.101377339	0.337738515
AC103686	-0.056	0.101368737	0.337738515
KIF28P	-0.056	0.101352812	0.337726886
KISS1R	-0.056	0.101352242	0.337726886
SH3BP2	-0.056	0.101316172	0.337654365
AP006285	-0.056	0.101301791	0.337651368
CTB-181F24	-0.056	0.101299742	0.337651368
BMS1P9	-0.056	0.101298469	0.337651368
ANKFY1	-0.056	0.101281607	0.337651368
CACNA1C-IT3	-0.056	0.101227983	0.337523465
RNU6-288P	-0.056	0.101206291	0.337474762
AC062020	-0.056	0.101203044	0.337474762
LANCL1-AS1	-0.056	0.101197223	0.337474762
OR7D1P	-0.056	0.101174646	0.337440116

CCDC65	-0.056	0.101157002	0.337404896
HSPE1P13	-0.056	0.101124923	0.337321522
DLEU2L	-0.056	0.101122083	0.337321522
RAF1	-0.056	0.101113823	0.337321522
RNU6-286P	-0.056	0.101107504	0.337321522
AL450423	-0.056	0.101105929	0.337321522
CTC-244M17	-0.056	0.101070992	0.337283363
C2orf68	-0.056	0.101058817	0.337266363
CBFB	-0.056	0.100922372	0.336858212
SERF1B	-0.056	0.100840167	0.336678208
TPRG1-AS2	-0.056	0.100832107	0.336674899
RNU6-588P	-0.056	0.100829269	0.336674899
LINC00388	-0.056	0.100803017	0.336624968
EML5	-0.056	0.100794143	0.336618935
CDC27	-0.056	0.100773578	0.336573856
PRRC2C	-0.056	0.10077051	0.336573856
GS1-278J22	-0.056	0.100736539	0.336520946
DGCR8	-0.056	0.10072258	0.336497917
RP11-442O18	-0.056	0.100716795	0.336497917
PBX2	-0.056	0.100698281	0.336463941
PCAT1	-0.056	0.100675575	0.336411677
LINC00474	-0.056	0.100664435	0.336398054
RNU2-7P	-0.056	0.100662788	0.336398054
PPIAP9	-0.056	0.100658927	0.336398054
AL845259	-0.056	0.100626669	0.336366254
EIF4BP2	-0.056	0.100571108	0.336204125
OR2A1-AS1	-0.056	0.100558027	0.336183993
GLCE	-0.056	0.100549522	0.336183993
RP11-300J18	-0.056	0.100515759	0.336113462
ZNF569	-0.056	0.100502468	0.336092618
IGKV3-25	-0.056	0.1004688	0.336003618
SNORD73	-0.056	0.100467943	0.336003618
RP11-64K12	-0.056	0.100452373	0.335995868
RPS4XP1	-0.056	0.100424516	0.335926284
GOLGA8M	-0.056	0.100412572	0.335909922
NCAPD2	-0.056	0.100394369	0.335886639
RP11-313L6	-0.056	0.100385791	0.335886639
AFG3L2P1	-0.056	0.100376233	0.335886639
FAM153C	-0.056	0.100270251	0.335716782
RP11-141M1	-0.056	0.100267501	0.335716782
AC074389	-0.056	0.100236764	0.33569249
AC138623	-0.056	0.100229441	0.33569249
RP1-245M18	-0.056	0.10022335	0.33569249
GTF2IRD2	-0.056	0.10019439	0.335627953
RP11-332H14	-0.056	0.100177542	0.335595118
SLC25A52	-0.056	0.100161018	0.335563365
RNU6-632P	-0.056	0.100122756	0.335505979
OR4C13	-0.056	0.100091826	0.335451941
RNF152	-0.056	0.100086667	0.335451941
RP11-296L22	-0.056	0.100084639	0.335451941
CCDC47	-0.056	0.100076386	0.335451941
EID1	-0.056	0.100043627	0.335406012

DNALI1	-0.056	0.100016635	0.335386345
SCYL2	-0.056	0.099997552	0.335345966
GAS8	-0.056	0.099993487	0.335345966
CSPP1	-0.056	0.099956686	0.335256134
RN7SKP222	-0.056	0.099931771	0.335196173
SPIN3	-0.056	0.099911772	0.335152695
MIR320B2	-0.056	0.099846352	0.334956837
HPYR1	-0.056	0.099755542	0.334793688
U7	-0.056	0.099745747	0.334784403
AC093107	-0.056	0.099729572	0.334753704
VEZF1P1	-0.056	0.099722307	0.334752912
CTC-548H10	-0.056	0.099706132	0.33472371
RNA5SP57	-0.056	0.099692695	0.33472371
TRUB1	-0.056	0.099655796	0.334649383
GOLGA6L5P	-0.056	0.099649329	0.334649383
METAP1D	-0.056	0.099630013	0.334631781
RP11-373D7	-0.056	0.099592368	0.334576123
RP11-708B6	-0.056	0.099546867	0.334494049
ENSAP1	-0.056	0.099544947	0.334494049
VENTXP6	-0.056	0.099533897	0.334494049
ADORA2A	-0.056	0.099518894	0.334494049
MTHFR	-0.056	0.099463061	0.334363808
RP11-497H16	-0.056	0.099458246	0.334363808
DDX31	-0.056	0.099422819	0.334313089
AC087651	-0.056	0.099324992	0.33407849
MIR5587	-0.056	0.099292935	0.333994252
BLOC1S2P1	-0.056	0.099282154	0.333981575
AC073551	-0.056	0.09928069	0.333981575
PHBP10	-0.056	0.099209922	0.33378574
MEGF11	-0.056	0.099123491	0.333612784
C18orf54	-0.056	0.099102021	0.333564095
CHUK	-0.056	0.099099106	0.333564095
MIR6807	-0.056	0.099089718	0.333564095
RP11-81K2	-0.056	0.099056045	0.333503622
UXT-AS1	-0.056	0.099031523	0.333444633
RP11-1134I14	-0.056	0.099025983	0.333444633
LMBRD1	-0.056	0.099009908	0.333418997
RNU6-891P	-0.056	0.09899844	0.333403952
RPL5P24	-0.056	0.098978345	0.333360303
SMTNL1	-0.056	0.098913686	0.333189201
SETBP1	-0.056	0.098883615	0.333135032
RP11-17M16	-0.056	0.098879286	0.333135032
TRIM77BP	-0.056	0.098868751	0.333135032
PLEKHM3	-0.056	0.09880184	0.333000867
HMGB3P4	-0.056	0.098765919	0.332912239
BCS1L	-0.056	0.098748509	0.33289179
RP1-286D6	-0.056	0.098747055	0.33289179
PRAMEF8	-0.056	0.09873041	0.3328779
CRHR1-IT1	-0.056	0.098707715	0.33282532
AL357515	-0.056	0.098702296	0.33282532
EEF1A1P40	-0.056	0.098671294	0.332772816
RNU1-91P	-0.056	0.098666032	0.332772816

RP1-29C18	-0.056	0.098651802	0.332772816
AL136160	-0.056	0.09863438	0.332772816
NPM1P5	-0.056	0.098599707	0.332719871
MGST2	-0.056	0.098588539	0.332705758
SNORA36	-0.056	0.098536463	0.332600718
RNU6-366P	-0.056	0.098515384	0.332576706
SAPCD2P2	-0.056	0.09850464	0.332564011
RP11-711D18	-0.056	0.098426058	0.332392958
TRBV8-1	-0.056	0.098393235	0.33232924
HMGN2P3	-0.056	0.098373042	0.332284602
RP11-175M2	-0.056	0.098351954	0.332236933
DIMT1	-0.056	0.098334043	0.332199992
RAD54L	-0.056	0.098282138	0.332048194
CARD10	-0.056	0.098280913	0.332048194
RP11-424C20	-0.056	0.098198569	0.331883567
RP11-218M11	-0.056	0.098194078	0.331883567
MIR3689D1	-0.056	0.098179743	0.331883567
RP11-449J10	-0.056	0.098168475	0.331883567
NKAIN1	-0.056	0.098133004	0.33180325
AC066612	-0.056	0.098078876	0.331667985
RP11-75N20	-0.056	0.098056786	0.331639722
CSH2	-0.056	0.098037695	0.331600287
CDH23-AS1	-0.056	0.098011559	0.331557392
ZKSCAN1	-0.056	0.097996938	0.331531479
RP11-956A19	-0.056	0.097964744	0.331458059
AIRE	-0.056	0.09795562	0.331458059
MIR3682	-0.056	0.09791716	0.331379278
GACAT2	-0.056	0.097889565	0.33130943
RNU6-397P	-0.056	0.097882779	0.33130943
MRPS23	-0.056	0.097876116	0.33130943
MIR3609	-0.056	0.097822623	0.331200546
NUP98	-0.056	0.097811978	0.331188049
CSNK1G3	-0.056	0.097753861	0.331085419
AC007787	-0.056	0.097726082	0.331061962
PMAIP1	-0.056	0.097722264	0.331061962
MIR760	-0.056	0.097704565	0.331059713
POGZ	-0.056	0.09770342	0.331059713
AJ011931	-0.056	0.097683339	0.331058448
RNU6-117P	-0.056	0.097683161	0.331058448
RP11-333J10	-0.056	0.097680359	0.331058448
RP11-460H9	-0.056	0.097678562	0.331058448
RP11-307O13	-0.056	0.097668924	0.331058448
EXOC5	-0.056	0.097627519	0.331057732
CTD-2622I13	-0.056	0.097574811	0.330926122
MRPL44	-0.056	0.097572922	0.330926122
UNC119	-0.056	0.097566722	0.330926122
HIVEP3	-0.056	0.097557311	0.330926122
BARX2	-0.056	0.097461588	0.330636303
IGKV1-22	-0.056	0.097410024	0.330508457
RP11-1072C15	-0.056	0.097307145	0.330253501
CRYBA2	-0.056	0.097298028	0.330246093
KIF18B	-0.056	0.097287598	0.330234225

RP11-162A12	-0.056	0.097233594	0.330074438
MIR4300	-0.056	0.097162638	0.329880592
RPL7P57	-0.056	0.09715895	0.329880592
SEPT7P8	-0.056	0.097140049	0.329850929
DNMT3A	-0.056	0.097118488	0.329801227
ADAMTS19	-0.056	0.09709884	0.329758017
METTL7A	-0.056	0.097061913	0.329728233
DSCR3	-0.056	0.097017046	0.329691821
LRRTM2	-0.056	0.097015648	0.329691821
CTB-113D17	-0.056	0.097000405	0.329691821
SIGLEC1	-0.056	0.096987953	0.329691821
NYX	-0.057	0.096960749	0.329691821
RASA3-IT1	-0.057	0.096951187	0.329691821
RP11-28G8	-0.057	0.09694458	0.329691821
SYNGR3	-0.057	0.096890694	0.329591677
OR10V1	-0.057	0.096852694	0.329485947
OTUD1	-0.057	0.096847211	0.329485947
RNU7-160P	-0.057	0.096835421	0.329485947
HEATR3	-0.057	0.096806605	0.329423277
HCP5B	-0.057	0.096758746	0.329307473
C14orf177	-0.057	0.096698742	0.329150299
MIR6746	-0.057	0.096691378	0.329148757
RP4-583P15	-0.057	0.096662448	0.329073795
ALMS1	-0.057	0.096621574	0.328975669
CDKN2B-AS1	-0.057	0.096614323	0.328975669
CTD-2285G11	-0.057	0.09656257	0.328921867
OR4F16	-0.057	0.096558102	0.328921867
XXbac-BPG181M17	-0.057	0.096533383	0.328869489
ABHD17C	-0.057	0.096516595	0.328835817
RNU1-136P	-0.057	0.096463974	0.328703563
RP11-116K4	-0.057	0.096437549	0.328637031
CHPT1	-0.057	0.096419385	0.328609049
RP11-368M16	-0.057	0.096418662	0.328609049
LINC00977	-0.057	0.09634646	0.328439141
CTD-2265M8	-0.057	0.096339509	0.328439141
LY86	-0.057	0.096334747	0.328439141
WDR59	-0.057	0.09630998	0.328390279
PRKAG1	-0.057	0.096271745	0.328330425
RP13-254B10	-0.057	0.096258851	0.32830996
RP11-307C19	-0.057	0.096253312	0.32830996
SGTB	-0.057	0.096212498	0.32822238
RP11-398A8	-0.057	0.096138152	0.328015742
CSAG3	-0.057	0.096136677	0.328015742
RP11-335K5	-0.057	0.09610455	0.327951273
RP11-763B22	-0.057	0.096100812	0.327951273
WDR37	-0.057	0.096099343	0.327951273
LINC00294	-0.057	0.096076922	0.327947792
GBP1	-0.057	0.09606533	0.327931727
KNOP1P1	-0.057	0.096044892	0.327908967
SLC7A6OS	-0.057	0.096029795	0.327880928
RBM47	-0.057	0.096025423	0.327880928
RNU6-1273P	-0.057	0.095968735	0.327742934

PASD1	-0.057	0.095949604	0.327701096
KRT18P38	-0.057	0.095918474	0.327618272
SS18L2P2	-0.057	0.095905011	0.327613706
COL9A1	-0.057	0.095821495	0.327357458
MIEN1	-0.057	0.095812446	0.327350026
RAPGEF2	-0.057	0.095807535	0.327350026
CARD16	-0.057	0.095800552	0.327350026
RP11-513M1	-0.057	0.095788303	0.327337986
RP11-346L1	-0.057	0.095752477	0.327239038
JKAMP	-0.057	0.095732486	0.327217677
RP11-366O20	-0.057	0.095713888	0.327177588
OSBPL9P2	-0.057	0.095703048	0.327164014
PHACTR2P1	-0.057	0.095607505	0.32695474
RP11-359N5	-0.057	0.09559428	0.326932985
MIR6833	-0.057	0.095583745	0.326932985
AC008280	-0.057	0.095550164	0.326852522
RP5-1170K4	-0.057	0.095547295	0.326852522
DOCK11P1	-0.057	0.095546937	0.326852522
UBTF	-0.057	0.095541257	0.326852522
SLC4A1	-0.057	0.095537459	0.326852522
SP2	-0.057	0.095532983	0.326852522
SYCE2	-0.057	0.095527431	0.326852522
CTBS	-0.057	0.095502722	0.326852522
TTC6	-0.057	0.095490841	0.326852522
MMRN1	-0.057	0.095489982	0.326852522
RP11-715G18	-0.057	0.095479258	0.326852522
SELT	-0.057	0.09543439	0.326761588
AL356261	-0.057	0.095394333	0.326647917
THNSL1	-0.057	0.095394149	0.326647917
PSME3	-0.057	0.095389582	0.326647917
VOPP1	-0.057	0.095359683	0.326623202
HNRNPA3P4	-0.057	0.095338052	0.326572598
CTB-151G24	-0.057	0.095299133	0.326509732
DHFRL1	-0.057	0.095210735	0.326300761
HCG23	-0.057	0.095205493	0.326300761
IGHVIII-82	-0.057	0.095205203	0.326300761
AC109826	-0.057	0.09516156	0.326202649
HLA-DPA2	-0.057	0.095137723	0.326144413
TGS1	-0.057	0.095102861	0.326048372
TCP11X3P	-0.057	0.095059727	0.325923952
RP4-726N1	-0.057	0.095043726	0.325892552
ZRANB2-AS2	-0.057	0.09499747	0.325757398
AC013248	-0.057	0.094876595	0.325391231
AL392111	-0.057	0.094862262	0.325391231
PI4KAP2	-0.057	0.094843346	0.325369448
HNRNPA3P8	-0.057	0.09478205	0.325206015
MIR4292	-0.057	0.09476306	0.325164285
RN7SKP126	-0.057	0.09473682	0.32513022
OR2AS1P	-0.057	0.094716482	0.325098148
DTX2P1	-0.057	0.094703908	0.325078418
RNU6-138P	-0.057	0.094664635	0.325009499
CCDC176	-0.057	0.094635818	0.324961795

CDK1	-0.057	0.094612698	0.324929258
RP11-2H8	-0.057	0.094610813	0.324929258
RP5-983L19	-0.057	0.094598952	0.324929258
SEMA3B-AS1	-0.057	0.094465849	0.324612194
MCM10	-0.057	0.094446128	0.324567842
MCMBP	-0.057	0.094432752	0.324567842
IGLJ7	-0.057	0.094371803	0.324406054
DNTTIP2	-0.057	0.094364494	0.324404341
RP11-795H16	-0.057	0.094340593	0.324345587
ERCC4	-0.057	0.094331511	0.32434
AC073610	-0.057	0.094315131	0.324328284
RAB5CP1	-0.057	0.094283519	0.32424299
RP11-539L10	-0.057	0.094229065	0.324125935
AC009491	-0.057	0.094227133	0.324125935
SNX6	-0.057	0.094218836	0.324125935
CABP2	-0.057	0.094153199	0.323978593
SLC23A1	-0.057	0.094144676	0.323976048
MIR5008	-0.057	0.09407821	0.323770714
SYNGAP1	-0.057	0.094050779	0.323723094
AC069277	-0.057	0.094034426	0.323690201
RNU7-149P	-0.057	0.094020762	0.323690201
CICP11	-0.057	0.094009967	0.323690201
UNC50	-0.057	0.094007459	0.323690201
RP11-342M3	-0.057	0.093999037	0.323690201
GPHN	-0.057	0.093986288	0.323688259
CTA-363E19	-0.057	0.093966303	0.323642834
NPY1R	-0.057	0.093936224	0.323586038
OR4R2P	-0.057	0.09389735	0.323475523
RP11-944C7	-0.057	0.093882284	0.323460974
MIR3646	-0.057	0.093875291	0.323460974
RAC1P8	-0.057	0.093856319	0.323427747
RP11-206L10	-0.057	0.093850328	0.323427747
AC104590	-0.057	0.093840415	0.323419745
DKFZP434H168	-0.057	0.093825671	0.323392332
CTA-305I2	-0.057	0.093822506	0.323392332
MIR8069-2	-0.057	0.093797735	0.323342844
AC112778	-0.057	0.093780363	0.32330636
AREL1	-0.057	0.093768463	0.32330636
MIR3153	-0.057	0.093723822	0.323228421
CTD-2313N18	-0.057	0.093718473	0.323228421
ZNF730	-0.057	0.093688201	0.323175777
PDPK2P	-0.057	0.09368733	0.323175777
RP11-17G11	-0.057	0.093676078	0.323175777
PANK3	-0.057	0.093672723	0.323175777
ZNF886P	-0.057	0.093649666	0.323136467
ZNF529	-0.057	0.09363597	0.323112617
RP11-641J8	-0.057	0.093630736	0.323112617
OR4Q3	-0.057	0.093582824	0.322999433
NEFHP1	-0.057	0.093566163	0.322965331
OR5AK3P	-0.057	0.093562864	0.322965331
AC079080	-0.057	0.093552935	0.322965331
RP11-405F3	-0.057	0.093525893	0.322896537

RP11-492E3	-0.057	0.093497093	0.322820504
ZNF407	-0.057	0.093475592	0.322769669
FAM183CP	-0.057	0.093440294	0.322671178
MIR3171	-0.057	0.093425933	0.32264498
AC009305	-0.057	0.09337372	0.322511433
PLAG1	-0.057	0.093347379	0.322443835
RHOQP1	-0.057	0.09332425	0.322387324
RP11-103B5	-0.057	0.093314424	0.322376763
AC108462	-0.057	0.093209515	0.322037692
RNU7-40P	-0.057	0.093197303	0.322018859
DDX3P3	-0.057	0.093192516	0.322018859
RP11-133K1	-0.057	0.093191938	0.322018859
MTND3P3	-0.057	0.093183966	0.322018859
AC002128	-0.057	0.093129155	0.321876797
CCP110	-0.057	0.093093525	0.321800355
BIVM-ERCC5	-0.057	0.093086811	0.321800355
PPY	-0.057	0.093084736	0.321800355
MTFR1L	-0.057	0.093017197	0.321699954
MIR302C	-0.057	0.0929981	0.321680624
OR5P4P	-0.057	0.092990831	0.321678843
PPM1M	-0.057	0.092968613	0.321628367
PPIAP3	-0.057	0.092957455	0.321628367
RNU7-18P	-0.057	0.092800669	0.321271555
RP11-824M15	-0.057	0.092799016	0.321271555
GNA14	-0.057	0.092792984	0.321271555
AC091177	-0.057	0.092792879	0.321271555
CLEC7A	-0.057	0.092747045	0.321255528
TFF3	-0.057	0.092728758	0.321215547
RP11-33N14	-0.057	0.092669352	0.321033111
CARD8-AS1	-0.057	0.09266234	0.321032171
RNU6-856P	-0.057	0.092652043	0.321032171
MTPAP	-0.057	0.092575361	0.32082418
APOBEC3F	-0.057	0.092568779	0.32082418
MIR4288	-0.057	0.092537277	0.320762219
LEUTX	-0.057	0.092522932	0.32073584
PIGBOS1	-0.057	0.092519003	0.32073584
AC020951	-0.057	0.092514145	0.32073584
GRAMD1B	-0.057	0.092511469	0.32073584
KYNUP3	-0.057	0.092501558	0.32073584
CTD-2630F21	-0.057	0.092450264	0.320623953
OGFRP1	-0.057	0.092417075	0.320532191
KRT223P	-0.057	0.092385894	0.32044738
TMEM255A	-0.057	0.09236175	0.320410303
AP2B1	-0.057	0.092356028	0.320410303
RN7SL709P	-0.057	0.09235493	0.320410303
HIST1H2BK	-0.057	0.092347233	0.320410303
RP1-172N19	-0.057	0.092323895	0.320372325
MIR3180-3	-0.057	0.092254372	0.320224399
AC087501	-0.057	0.09224902	0.320224399
KRT18P46	-0.057	0.092231794	0.320192698
RN7SL395P	-0.057	0.092211907	0.320146998
ATP13A3	-0.057	0.092150121	0.319979133

IMPACT	-0.057	0.092135102	0.319950311
AC099539	-0.057	0.092104742	0.319897003
ZNF682	-0.057	0.092033727	0.31969151
ORAOV1P1	-0.057	0.092027113	0.31969151
RTN3P1	-0.057	0.091996181	0.319631028
AC011742	-0.057	0.09198112	0.319602013
LINC01502	-0.057	0.091926897	0.319466081
RNU6-1119P	-0.057	0.091916572	0.319466081
GLMN	-0.057	0.091901513	0.319465247
MTMR6	-0.057	0.091894653	0.319465247
PROSER3	-0.057	0.091863946	0.319404597
RNF41	-0.057	0.091862891	0.319404597
DPY19L1P2	-0.057	0.091814247	0.319301726
PIK3CB	-0.057	0.091803296	0.319286956
CPNE7	-0.057	0.091790959	0.319267363
SRRD	-0.057	0.091771243	0.319222101
PPAPDC1B	-0.057	0.091714962	0.319049632
GOLGA5P1	-0.057	0.091681172	0.318978681
SLC35D2	-0.057	0.09164723	0.318907183
C4orf32	-0.057	0.091631751	0.318876618
MIR4632	-0.057	0.091628376	0.318876618
TSEN2P1	-0.057	0.091619555	0.318876618
RP11-16E18	-0.057	0.091614379	0.318876618
ZNF223	-0.057	0.091589115	0.318821429
CTC-661I16	-0.057	0.09157048	0.318779858
MIR548C	-0.057	0.091565269	0.318779858
RNU6-351P	-0.057	0.091563862	0.318779858
BTNL3	-0.057	0.091512308	0.318647222
RN7SL388P	-0.057	0.091502452	0.318636197
SLC25A35	-0.057	0.091500316	0.318636197
HMGN2P21	-0.057	0.091450625	0.318525592
RNA5SP124	-0.057	0.091441208	0.318516085
IARS	-0.057	0.091425248	0.318483784
TAMM41	-0.057	0.091337417	0.318336597
POGLUT1	-0.057	0.091316624	0.318308468
FEM1B	-0.057	0.091284498	0.318226233
SIGLEC22P	-0.057	0.091280313	0.318226233
POLR3E	-0.057	0.091272742	0.318226233
RN7SKP129	-0.057	0.091262919	0.318220885
WDTC1	-0.057	0.091239894	0.31816389
ESRP2	-0.057	0.091221427	0.318146083
RPL21P24	-0.058	0.09119595	0.318080522
DNAJC19P8	-0.058	0.091181635	0.318080522
RP11-775A1	-0.058	0.091177419	0.318080522
DARS2	-0.058	0.091156084	0.318067449
ATG16L1	-0.058	0.091113522	0.318025922
MRPL53	-0.058	0.091112369	0.318025922
MIR6881	-0.058	0.091102438	0.318025922
UTS2R	-0.058	0.091095443	0.318025922
NBPF7	-0.058	0.091094412	0.318025922
L3MBTL2	-0.058	0.091075271	0.318025922
RNU6-1099P	-0.058	0.091054731	0.317983844

AC009947	-0.058	0.09105017	0.317983844
CDKN2AIPNLP1	-0.058	0.091033508	0.317968993
RNU7-180P	-0.058	0.091030743	0.317968993
TBXAS1	-0.058	0.091012002	0.317968993
KIF16B	-0.058	0.090962794	0.31782591
ABHD13	-0.058	0.090912142	0.317672237
RP11-352D13	-0.058	0.090898858	0.317649125
FKBP4P6	-0.058	0.090839147	0.317487052
SYBU	-0.058	0.090827843	0.317470841
LMBR1L	-0.058	0.090821779	0.317470841
DUTP7	-0.058	0.090807721	0.317447102
RP11-370B6	-0.058	0.090803905	0.317447102
CTD-2013N17	-0.058	0.090783418	0.317447102
CELSR1	-0.058	0.090779969	0.317447102
SUMO2P17	-0.058	0.090773175	0.317447102
RP11-10I6	-0.058	0.090727076	0.317351533
RNU6-378P	-0.058	0.090723441	0.317351533
AL139184	-0.058	0.09066553	0.317182842
RP11-507K13	-0.058	0.090652362	0.317182842
BTBD8	-0.058	0.09064633	0.317182842
SRP14-AS1	-0.058	0.090638243	0.317180571
DCAF12	-0.058	0.090592222	0.317042823
AL353803	-0.058	0.090573943	0.317032129
ZNF859P	-0.058	0.090568496	0.317032129
AL035422	-0.058	0.090498944	0.316879371
ZFR	-0.058	0.090446251	0.316718153
ZNF746	-0.058	0.090444375	0.316718153
AC011298	-0.058	0.090368402	0.316492086
TAF4B	-0.058	0.090354178	0.316465541
RP11-127B20	-0.058	0.09031431	0.316349167
KRT8P41	-0.058	0.090303765	0.316335498
ZNF257	-0.058	0.090300781	0.316335498
SLC6A12	-0.058	0.090294225	0.316335498
MIR324	-0.058	0.090201863	0.316187836
JPH4	-0.058	0.090120272	0.315925084
ADAM1B	-0.058	0.090116324	0.315925084
KBTBD11	-0.058	0.090094613	0.315922008
RP4-701O16	-0.058	0.090007784	0.315693404
LINC00620	-0.058	0.09000605	0.315693404
RP11-119D9	-0.058	0.089974133	0.315621863
MIR6809	-0.058	0.089973928	0.315621863
OR5D14	-0.058	0.089955933	0.315604509
RP1-63G5	-0.058	0.089939147	0.315568864
RRN3P2	-0.058	0.089937357	0.315568864
RP11-624L4	-0.058	0.089860806	0.315433418
MIR5695	-0.058	0.089857451	0.315433418
FSCN2	-0.058	0.089847358	0.315433418
POU5F1P6	-0.058	0.089843384	0.315433418
PITPNC1	-0.058	0.08979617	0.315369235
GPC5-AS1	-0.058	0.089757056	0.31525511
RP11-285E18	-0.058	0.089737244	0.315208771
ZNF847P	-0.058	0.08972096	0.315174818

CTC-497E21	-0.058	0.089697024	0.315113975
RP11-699C17	-0.058	0.089675893	0.315086225
POTENP	-0.058	0.089667803	0.315081041
MIR4285	-0.058	0.089640669	0.315008939
TAS2R6P	-0.058	0.089628791	0.31499044
KIAA1429	-0.058	0.08962454	0.31499044
APOF	-0.058	0.089595472	0.314978691
PCDH9-AS2	-0.058	0.089578565	0.314953368
ADPGK-AS1	-0.058	0.089541175	0.314855055
ACTR2	-0.058	0.089521565	0.314822682
SMARCAL1	-0.058	0.089497076	0.314783047
CTD-2104P17	-0.058	0.089492427	0.314783047
RP11-768G7	-0.058	0.089461212	0.314737186
H3F3AP4	-0.058	0.089429423	0.314661283
MED28P5	-0.058	0.089370568	0.314477434
RP11-1365D11	-0.058	0.089355441	0.314447435
MIR1185-1	-0.058	0.089343006	0.314426909
CCNK	-0.058	0.089338079	0.314426909
AC069294	-0.058	0.089329104	0.314426909
CYP2B6	-0.058	0.089305304	0.314410387
RN7SL246P	-0.058	0.089296941	0.314404185
CTD-2008P7	-0.058	0.089237121	0.314263263
RP11-18H7	-0.058	0.089227123	0.31425129
PRYP5	-0.058	0.089210528	0.314239317
RALGPS1	-0.058	0.08916839	0.314114121
RNU6-648P	-0.058	0.089163491	0.314114121
CYP4F32P	-0.058	0.089152454	0.314104447
SRP14	-0.058	0.089122868	0.314046672
ACA59	-0.058	0.089110918	0.314027797
MIR1207	-0.058	0.089073547	0.313957102
RP5-1173I20	-0.058	0.089070585	0.313957102
SIGLEC7	-0.058	0.089024369	0.313862136
TARSL2	-0.058	0.089007086	0.313824437
UNC119B	-0.058	0.088957003	0.313694298
PGGT1BP2	-0.058	0.088942094	0.313688176
RP11-498E2	-0.058	0.088904844	0.313649704
OR52V1P	-0.058	0.088870927	0.313553275
SLC2A10	-0.058	0.088848482	0.313497307
AC012507	-0.058	0.088841856	0.313497153
RP11-341A22	-0.058	0.088840566	0.313497153
RP11-533K9	-0.058	0.088805076	0.313413808
AC009487	-0.058	0.088779889	0.313348139
GALK2	-0.058	0.088763979	0.313316847
KMT2B	-0.058	0.088688362	0.313141122
PHB2P1	-0.058	0.088668089	0.313092754
FASTKD3	-0.058	0.088561559	0.312809359
RN7SL826P	-0.058	0.088502423	0.312646854
RP11-142I2	-0.058	0.088494786	0.312643067
HMGN2P7	-0.058	0.088478937	0.312610262
RNU6-355P	-0.058	0.088462168	0.312574201
GNG4	-0.058	0.088447286	0.312567996
SDC4	-0.058	0.088442885	0.312567996

RN7SL297P		-0.058	0.088402634	0.312479756
PLEKHB2		-0.058	0.088401344	0.312479756
APOOL		-0.058	0.088399195	0.312479756
MCM8		-0.058	0.088370249	0.312434849
OR2AI1P		-0.058	0.088366663	0.312434849
TLK1		-0.058	0.088312788	0.312307389
MLX		-0.058	0.088304406	0.312307389
TPPP2		-0.058	0.088294326	0.312305522
ZNF75A		-0.058	0.088246469	0.312176232
RP11-305E6		-0.058	0.088208065	0.31210948
RP11-572O17		-0.058	0.088184274	0.31205531
AC091132		-0.058	0.088168869	0.312023981
KRTAP5-AS1		-0.058	0.088148108	0.311973689
LINC00566		-0.058	0.088146387	0.311973689
RP11-295G12		-0.058	0.088133863	0.311973689
RN7SKP9		-0.058	0.088121757	0.311973689
TWF1		-0.058	0.088112217	0.311973689
VDAC1P5		-0.058	0.088106692	0.311973689
RP11-214K3		-0.058	0.08810181	0.311973689
RNU6-1113P		-0.058	0.088062776	0.31190346
RP11-305O6		-0.058	0.08805378	0.311894792
MIR548E		-0.058	0.088029013	0.311830258
RP11-626I20		-0.058	0.087999029	0.311747231
MAPK13		-0.058	0.087985671	0.311723092
IGLVIV-66-1		-0.058	0.087974296	0.31170598
RNU4-78P		-0.058	0.087971682	0.31170598
RP11-43F13		-0.058	0.087944608	0.311666366
RHEBL1		-0.058	0.087920975	0.31164886
LINC00434		-0.058	0.087916573	0.31164886
MICC		-0.058	0.087914769	0.31164886
PEX7		-0.058	0.087905189	0.31164886
ARL16		-0.058	0.087896737	0.31164886
LINC00418		-0.058	0.087873533	0.311606231
RP11-58H15		-0.058	0.087843247	0.311542952
VWA8		-0.058	0.087752134	0.311358926
ARG1		-0.058	0.087741094	0.311342949
MIPOL1		-0.058	0.087740846	0.311342949
	3-Sep	-0.058	0.087707397	0.311269762
HSP90AA4P		-0.058	0.087702853	0.311269762
RPS20P22		-0.058	0.087680632	0.311262118
CTD-3184A7		-0.058	0.087675381	0.311262118
SYTL1		-0.058	0.087630473	0.311182266
COG8		-0.058	0.087615339	0.311151721
SLBP		-0.058	0.087609143	0.311151721
EZR-AS1		-0.058	0.087605883	0.311151721
ANKRD23		-0.058	0.087603151	0.311151721
MARK2P8		-0.058	0.087600203	0.311151721
RP11-552M11		-0.058	0.087590661	0.311151721
THEMIS3P		-0.058	0.087587519	0.311151721
RP11-397A15		-0.058	0.087574092	0.311151721
VN1R42P		-0.058	0.087535634	0.311151721
FBXO5		-0.058	0.087530259	0.311151721

AL139139	-0.058	0.087494559	0.311070718
MCHR2	-0.058	0.087494332	0.311070718
ARL8B	-0.058	0.087445347	0.310965396
OR10K2	-0.058	0.087404659	0.310890343
RP11-673D15	-0.058	0.08739066	0.310863767
VCX2	-0.058	0.087355196	0.310760825
AGGF1P4	-0.058	0.087334757	0.310711321
RP1-104O17	-0.058	0.087331392	0.310711321
ZDHHC23	-0.058	0.087330411	0.310711321
RPL23AP78	-0.058	0.087325184	0.310711321
ZSCAN30	-0.058	0.08732403	0.310711321
RPS15AP36	-0.058	0.087287359	0.310658725
RNU1-48P	-0.058	0.087272563	0.310629277
NBPF8	-0.058	0.087260941	0.310611125
GCNT1P5	-0.058	0.087259332	0.310611125
DPP9-AS1	-0.058	0.087244062	0.310597469
RP11-315L6	-0.058	0.087227684	0.310562376
NPM1P24	-0.058	0.087222038	0.310562376
UBE2WP1	-0.058	0.087214735	0.310562376
RP11-154H17	-0.058	0.087203633	0.310546392
AC104383	-0.058	0.087074095	0.310177853
MIR133A1HG	-0.058	0.087069889	0.310177853
DSN1	-0.058	0.087057749	0.310166026
CHRNA10	-0.058	0.087045322	0.310144949
SCN7A	-0.058	0.087023678	0.310091032
RN7SL96P	-0.058	0.087013493	0.310077938
NIFKP6	-0.058	0.087006	0.310074437
RPS6P2	-0.058	0.086979444	0.310002991
CDCA8	-0.058	0.086934544	0.309866152
AC010931	-0.058	0.086879753	0.309719911
RP11-175I17	-0.058	0.086866104	0.309719911
PTTG4P	-0.058	0.086847832	0.309696142
TMED6	-0.058	0.086824586	0.309659618
ZNF165	-0.058	0.086786681	0.309593982
C5	-0.058	0.086749898	0.309485948
STRN	-0.058	0.086727029	0.309427537
INTS3	-0.058	0.086710811	0.309392853
RP11-574F21	-0.058	0.086671839	0.309300141
HMGB1P23	-0.058	0.08664318	0.309238358
AC005329	-0.058	0.086615343	0.309168022
RP11-151E14	-0.058	0.086609071	0.309168022
POLR2CP	-0.058	0.086608963	0.309168022
CNEP1R1	-0.058	0.0866042	0.309168022
AC020550	-0.058	0.086580967	0.309137994
RP11-611L7	-0.058	0.086544798	0.309050162
RP11-29A2	-0.058	0.086538778	0.309050162
RP11-110F24	-0.058	0.086532408	0.309050162
NSMCE4A	-0.058	0.086529666	0.309050162
SETP15	-0.058	0.086507827	0.309050162
LINC01181	-0.058	0.086489438	0.309050162
RP11-671E7	-0.058	0.08644313	0.308970128
CFAP99	-0.058	0.086415905	0.308895999

AC092377	-0.058	0.086402108	0.30889305
TRAPPC2	-0.058	0.086400937	0.30889305
CAPZA1	-0.058	0.086372318	0.308853462
TTLL5	-0.058	0.086346918	0.308788457
LINC01359	-0.058	0.086333477	0.308782567
NOL7	-0.058	0.086321324	0.308766483
BTNL9	-0.058	0.086314099	0.308763827
AC008073	-0.058	0.08628204	0.308695515
PWAR1	-0.058	0.086271492	0.308680967
SATB2	-0.058	0.086266444	0.308680967
POLR2A	-0.058	0.086260626	0.308680967
AC016691	-0.058	0.086260193	0.308680967
DDX25	-0.058	0.086253791	0.308680967
PLSCR5-AS1	-0.058	0.086221821	0.308619168
CTD-2010I22	-0.058	0.086208846	0.308595919
RAVER2	-0.058	0.086196353	0.308590182
AC132479	-0.058	0.086116038	0.308333223
AC004846	-0.058	0.086110298	0.308333223
VN1R69P	-0.058	0.086103643	0.308333223
RP5-837O21	-0.058	0.086084362	0.308333223
LPCAT4	-0.058	0.086061416	0.308323084
FAM149B1	-0.058	0.086057112	0.308323084
IL17A	-0.058	0.086054386	0.308323084
RP11-354H21	-0.058	0.086040518	0.308317791
SLC25A18P1	-0.058	0.08602488	0.308308146
TAF1	-0.058	0.086018377	0.30830804
ZNF25	-0.058	0.086005168	0.308307101
TPRG1	-0.058	0.085999665	0.308307101
MICU2	-0.058	0.085999165	0.308307101
RP11-89A16	-0.058	0.085998294	0.308307101
RP11-505P4	-0.058	0.085994241	0.308307101
GPN3	-0.058	0.085980556	0.308307101
ZNF821	-0.058	0.085972053	0.308307101
ZZZ3	-0.058	0.085963951	0.308307101
RP11-380D15	-0.058	0.085937616	0.308250552
RNA5SP217	-0.058	0.085916686	0.30819869
CELF2	-0.058	0.085902256	0.308193353
BCL2L2	-0.058	0.085885764	0.308157395
RP4-622L5	-0.058	0.085825191	0.308037263
RP4-655J12	-0.058	0.085816301	0.308037263
KRTAP9-9	-0.058	0.085814141	0.308037263
PAPOLB	-0.058	0.085807813	0.308037263
AL603632	-0.058	0.085797321	0.308037263
RP11-733C7	-0.058	0.085762268	0.307946288
PPP3CC	-0.058	0.085753715	0.307938789
RHBG	-0.058	0.085745457	0.307932353
RP11-184E9	-0.058	0.085703109	0.307849911
TTC8	-0.059	0.085695833	0.307846992
ITGAX	-0.059	0.085645361	0.30768889
CTD-3020H12	-0.059	0.085539339	0.307400747
RIPK1	-0.059	0.085470394	0.307176158
PSMA1	-0.059	0.085464719	0.307176158

AC132803	-0.059	0.085444691	0.307130137
MIPEPP3	-0.059	0.085405502	0.307081976
RAP1GAP	-0.059	0.085369888	0.306977099
AC067961	-0.059	0.085338947	0.306889009
UBE2MP1	-0.059	0.085310029	0.306831349
AP001595	-0.059	0.085296843	0.306807093
RP11-204P2	-0.059	0.085287303	0.30679595
CERS6	-0.059	0.085235897	0.306634192
TRAJ59	-0.059	0.085186996	0.306527733
TRBV5-3	-0.059	0.085184968	0.306527733
RNU6-658P	-0.059	0.085142949	0.30641554
OR56A4	-0.059	0.085136645	0.30641554
YY1	-0.059	0.08513095	0.30641554
AL160011	-0.059	0.085121094	0.306406344
RP11-313D6	-0.059	0.085117487	0.306406344
HAR1A	-0.059	0.085107265	0.306402882
MAGI1	-0.059	0.085095329	0.30638307
RNU6-778P	-0.059	0.08506728	0.306305234
AC116606	-0.059	0.08506728	0.306305234
NSMAF	-0.059	0.085066266	0.306305234
FAM107B	-0.059	0.085057638	0.306305234
RP11-452D21	-0.059	0.085043917	0.306305234
MIR635	-0.059	0.085036418	0.306305234
DPP10-AS3	-0.059	0.085036043	0.306305234
RP1-128M12	-0.059	0.085010294	0.306262125
DOPEY1	-0.059	0.085006678	0.306262125
CH507-24F1	-0.059	0.084993276	0.306247144
AC104071	-0.059	0.084986223	0.306244901
AL513011	-0.059	0.084986223	0.306244901
AC078991	-0.059	0.084986223	0.306244901
NIPA2	-0.059	0.084972684	0.306244901
CSPG4P11	-0.059	0.084937087	0.306160963
RP11-76I14	-0.059	0.084839159	0.305902749
OCIAD1-AS1	-0.059	0.084834838	0.305902749
PRDM4	-0.059	0.084810699	0.305888733
RP1-101G11	-0.059	0.084808051	0.305888733
DENR	-0.059	0.084757287	0.305766951
RP11-656A15	-0.059	0.084756426	0.305766951
AL162430	-0.059	0.084705179	0.305625268
GS1-111G14	-0.059	0.084701172	0.305625268
AC074264	-0.059	0.084690654	0.305625268
TRDJ4	-0.059	0.084670643	0.305593263
ITPR1-AS1	-0.059	0.084662666	0.305587631
SPATA4	-0.059	0.08465323	0.305587631
DEFB109P1	-0.059	0.08463832	0.305587631
ATP5BP1	-0.059	0.084618088	0.305587631
RP11-21G15	-0.059	0.084611315	0.305587555
AC073587	-0.059	0.084609259	0.305587555
RP11-536K17	-0.059	0.084600506	0.305587555
AGGF1P2	-0.059	0.084554121	0.305520001
RP11-342M21	-0.059	0.084530087	0.305456331
RNA5SP271	-0.059	0.08449909	0.305367492

YWHAQP5	-0.059	0.084491123	0.305361869
RN7SL354P	-0.059	0.084490681	0.305361869
AC004656	-0.059	0.084485899	0.305361869
AC022120	-0.059	0.084473731	0.305361869
DUX4	-0.059	0.084419668	0.305219427
ATP5A1	-0.059	0.08436879	0.305104964
RP5-1074L1	-0.059	0.084228592	0.304690501
IMPDH1P6	-0.059	0.084226649	0.304690501
FEM1AP3	-0.059	0.084211687	0.304675633
HLA-DRB6	-0.059	0.084175068	0.304589421
RPL5P31	-0.059	0.08414393	0.304523019
OR2AQ1P	-0.059	0.084139598	0.304523019
SLC25A24	-0.059	0.084128481	0.304513388
RP11-455G16	-0.059	0.08412151	0.304511299
GPR160	-0.059	0.08409713	0.304446185
SEC23B	-0.059	0.084062056	0.304342343
HLA-J	-0.059	0.084051938	0.304328848
AC011816	-0.059	0.084028308	0.30426642
PLCH2	-0.059	0.083993709	0.304164264
RP11-306I1	-0.059	0.083978051	0.304130684
CENPBD1	-0.059	0.083973784	0.304130684
MIR891A	-0.059	0.083963737	0.304125097
ARL14EPL	-0.059	0.08391741	0.303992915
IGLJ5	-0.059	0.083910823	0.303992915
MIR8064	-0.059	0.083909806	0.303992915
RP11-400N9	-0.059	0.083883013	0.303992915
RNU6-915P	-0.059	0.083880884	0.303992915
DYNLRB2	-0.059	0.083872666	0.303992915
MB21D1	-0.059	0.083841565	0.303913709
RP11-417N10	-0.059	0.083780999	0.303763524
TTLL13P	-0.059	0.083773577	0.303763524
LINC01300	-0.059	0.083757126	0.303746336
PHKA1-AS1	-0.059	0.083747158	0.303733316
KCND3	-0.059	0.083720969	0.303661458
RNA5SP131	-0.059	0.083712612	0.303654269
RN7SL89P	-0.059	0.083700089	0.303631972
AP000302	-0.059	0.083698941	0.303631972
FLYWCH1P1	-0.059	0.083691477	0.303631972
RP11-65L19	-0.059	0.083659584	0.303554395
RNU6-913P	-0.059	0.083635075	0.303488589
DR1	-0.059	0.083589757	0.303393489
RP11-394B5	-0.059	0.08357178	0.303351358
RN7SL833P	-0.059	0.083501995	0.303121152
AL354981	-0.059	0.083492963	0.303111468
SLC26A7	-0.059	0.08348418	0.303102688
AC093419	-0.059	0.083456234	0.303024325
BACE1-AS	-0.059	0.083405598	0.302909745
UBQLN1	-0.059	0.083382857	0.30285025
TPM3P4	-0.059	0.08338167	0.30285025
USP10	-0.059	0.083328709	0.302745934
RAC1P3	-0.059	0.083305798	0.302708874
DRAM1	-0.059	0.083289702	0.302696576

RP11-9H20	-0.059	0.083265477	0.302631629
C9orf152	-0.059	0.083235311	0.302545077
ZNF623	-0.059	0.083206275	0.302482009
RP11-600F24	-0.059	0.083168677	0.302418277
PEX19	-0.059	0.083167898	0.302418277
AHCYP7	-0.059	0.083123726	0.302300986
PROSER2-AS1	-0.059	0.083115517	0.302294212
RP5-901A4	-0.059	0.083112889	0.302294212
SETP16	-0.059	0.083092168	0.302278544
AC064874	-0.059	0.083047567	0.302162451
RP13-560N11	-0.059	0.08302777	0.3021135
TRIM56	-0.059	0.0830263	0.3021135
MIR6783	-0.059	0.082999828	0.302076466
RP11-513M16	-0.059	0.082982352	0.302040541
RP11-349A22	-0.059	0.082978722	0.302040541
DIS3	-0.059	0.082953582	0.301981981
HERC2P10	-0.059	0.082932069	0.301926743
ZNF563	-0.059	0.082926958	0.301926743
RLN1	-0.059	0.082817089	0.301646491
RIBC2	-0.059	0.082802672	0.301617045
SPINK4	-0.059	0.082781443	0.301562778
AKTIP	-0.059	0.082763767	0.301544516
KRT18P27	-0.059	0.082736918	0.301469756
RP11-883A18	-0.059	0.082694891	0.301384737
RABGAP1L	-0.059	0.08267466	0.301335123
AF003625	-0.059	0.082670433	0.301335123
RPS2P6	-0.059	0.082663574	0.301335123
HIST1H3E	-0.059	0.082609671	0.301198489
ZNF131	-0.059	0.082600351	0.301198489
HNRNPA1P5	-0.059	0.082567115	0.301150559
ZNF384	-0.059	0.082549035	0.301126741
CTD-2227I18	-0.059	0.082539816	0.301120171
RNU6-475P	-0.059	0.082537703	0.301120171
FOXJ3	-0.059	0.08253442	0.301120171
AL590084	-0.059	0.082526542	0.301120171
RPL17P43	-0.059	0.082521843	0.301120171
GATB	-0.059	0.08250384	0.301104246
UVRAG	-0.059	0.08248362	0.30105352
RP11-432F4	-0.059	0.082465496	0.301010438
RPL23AP81	-0.059	0.082464922	0.301010438
AC015969	-0.059	0.082461004	0.301010438
PVT1	-0.059	0.0824606	0.301010438
KCNK10	-0.059	0.082420306	0.300937743
AC068134	-0.059	0.082318693	0.30063587
RP5-940J5	-0.059	0.082298345	0.300584607
AC109638	-0.059	0.082295137	0.300584607
TRAM2-AS1	-0.059	0.082272953	0.30053796
PTAR1	-0.059	0.082236469	0.300450774
SYT10	-0.059	0.082228023	0.300442965
KLF1	-0.059	0.082180204	0.300314322
UGDH-AS1	-0.059	0.08217164	0.300306069
LRRC70	-0.059	0.082135958	0.300221738

CTD-3220F14	-0.059	0.082105118	0.300155085
VPS8	-0.059	0.082061083	0.300017134
KRT8P5	-0.059	0.081981758	0.299773144
AC087163	-0.059	0.081968674	0.299764809
EDRF1	-0.059	0.081966526	0.299764809
AC122718	-0.059	0.081948055	0.299741956
RP11-350F16	-0.059	0.081933844	0.299712995
RP11-433J20	-0.059	0.0819216	0.299691225
SF3A3P1	-0.059	0.081899316	0.299632717
RP11-172E10	-0.059	0.081834685	0.299488284
BAZ2B	-0.059	0.081818801	0.299453165
WDR60	-0.059	0.081762884	0.299271508
AC005534	-0.059	0.081697369	0.299054692
ZNF439	-0.059	0.081694518	0.299054692
AKAP2	-0.059	0.081694468	0.299054692
RNU2-31P	-0.059	0.081680746	0.299054692
RPL39P36	-0.059	0.081665816	0.299031124
CUZD1	-0.059	0.081621211	0.298890774
CUL5	-0.059	0.08158707	0.298811703
RN7SKP249	-0.059	0.081585391	0.298811703
GPS2P2	-0.059	0.08155064	0.298724223
RP11-23P13	-0.059	0.081549561	0.298724223
RP11-16K12	-0.059	0.081547119	0.298724223
RP5-1087E8	-0.059	0.081540733	0.298724223
LPPR3	-0.059	0.08151318	0.298701872
OR5E1P	-0.059	0.081402869	0.298397626
RP11-553K23	-0.059	0.081395003	0.298397626
HIST1H2BN	-0.059	0.081341905	0.298280791
RNA5SP102	-0.059	0.081305713	0.298193993
RAB1C	-0.059	0.081283459	0.298135335
RNU6-54P	-0.059	0.081272245	0.298135335
IFNK	-0.059	0.081259451	0.29811615
RNU6-968P	-0.059	0.081256379	0.29811615
CTD-2309H9	-0.059	0.081252876	0.29811615
BUB1P1	-0.059	0.081216353	0.298049872
LINC01322	-0.059	0.081209769	0.298048676
SKIV2L2	-0.059	0.081191941	0.298022757
FOXK2	-0.059	0.081170072	0.297994837
NPFFR1	-0.059	0.081165428	0.297994837
LINC01256	-0.059	0.081156944	0.297992582
RNU6-1125P	-0.059	0.081156397	0.297992582
RP11-172F10	-0.059	0.081109732	0.297888127
CTBP2P6	-0.059	0.081088437	0.297832884
RP11-179H18	-0.059	0.081085562	0.297832884
AC097262	-0.059	0.0810734	0.29782359
ZSCAN9	-0.059	0.081072532	0.29782359
AC015987	-0.059	0.081062097	0.29782359
RP11-50O21	-0.059	0.0810355	0.29775326
TRIM52-AS1	-0.059	0.081033237	0.29775326
RP11-512F24	-0.059	0.08102129	0.297746988
TOX4	-0.059	0.081019507	0.297746988
GOLGA5	-0.059	0.081012261	0.297746988

CTD-2015B23	-0.059	0.08098566	0.29768496
RP3-416J7	-0.059	0.080977996	0.297679762
RPL21P7	-0.059	0.080951398	0.297627932
RPS7P14	-0.059	0.080922038	0.297565929
RN7SL809P	-0.059	0.080891005	0.297509164
RP11-315E17	-0.059	0.080868054	0.29748226
OR5J2	-0.059	0.080858555	0.297470292
CTD-2326C4	-0.059	0.080852662	0.297470292
PPP1CA	-0.059	0.080798364	0.297294783
RAET1K	-0.059	0.080787993	0.297279592
LINC01516	-0.059	0.080786688	0.297279592
PRPSAP2	-0.059	0.080768401	0.297253431
SAP130	-0.059	0.080760888	0.297248751
TIAF1	-0.059	0.080722857	0.297131734
FCGRT	-0.059	0.080716963	0.297131734
DDX46	-0.059	0.08069957	0.297091939
SATB2-AS1	-0.059	0.080675271	0.297048406
CTD-3113P16	-0.059	0.080616802	0.296901982
RP11-52A20	-0.059	0.080598735	0.296881359
OR4P1P	-0.059	0.080588502	0.296866626
ZNF689	-0.059	0.080583501	0.296866626
AC078950	-0.059	0.080571806	0.29685104
SETD6	-0.059	0.080566985	0.29685104
LECT1	-0.059	0.080543123	0.296791283
NAGA	-0.059	0.080539654	0.296791283
TRMT12	-0.059	0.080537282	0.296791283
RP3-378P9	-0.059	0.080525521	0.296791283
CYP2C59P	-0.059	0.080480247	0.2966514
RP11-111F5	-0.059	0.080478451	0.2966514
RNU6-81P	-0.060	0.080463078	0.2966514
TET2-AS1	-0.060	0.080462428	0.2966514
CANT1	-0.060	0.080425477	0.296564278
ZBTB41	-0.060	0.080417232	0.296556834
RP11-96J19	-0.060	0.080392482	0.29648852
RP11-1258F18	-0.060	0.080369742	0.296427609
RP11-20I23	-0.060	0.080361429	0.296424794
TYMS	-0.060	0.080353	0.296424794
NR4A3	-0.060	0.080351674	0.296424794
RP11-152H18	-0.060	0.080333688	0.296424794
RP5-937E21	-0.060	0.080316541	0.296392059
TRIM38	-0.060	0.080295813	0.296351452
OR9A2	-0.060	0.080295007	0.296351452
MIR615	-0.060	0.080277378	0.29633938
RP3-472M2	-0.060	0.0802721	0.29633938
TMEM50B	-0.060	0.080206218	0.296145556
MIR548AT	-0.060	0.08019847	0.296139909
SLC9A3P2	-0.060	0.080173068	0.29609202
CTD-2210P15	-0.060	0.080101007	0.29594063
GRPEL1	-0.060	0.080086852	0.295911287
RP11-388P9	-0.060	0.080053688	0.2958117
RNY4P19	-0.060	0.080053541	0.2958117
RN7SL704P	-0.060	0.080051635	0.2958117

RNU6-650P	-0.060	0.080041614	0.2958117
ALAS1	-0.060	0.080035672	0.2958117
AZIN1	-0.060	0.080029819	0.2958117
CTB-107G13	-0.060	0.080027714	0.2958117
FCHO1	-0.060	0.079959517	0.295716099
RP11-17E2	-0.060	0.079952461	0.295716099
TATDN2P3	-0.060	0.079940696	0.295715381
TCEAL6	-0.060	0.079914304	0.295663682
AL109843	-0.060	0.079894653	0.29561394
GXYLT1P1	-0.060	0.0798846	0.29559971
CNGB3	-0.060	0.079868155	0.295595242
TUBB8P10	-0.060	0.079851274	0.295568246
CLASP1	-0.060	0.079819253	0.295472684
RNU5E-4P	-0.060	0.079755947	0.295307197
UBE2CP1	-0.060	0.079733022	0.295268226
TIAL1	-0.060	0.079730277	0.295268226
CH17-60O17	-0.060	0.079695854	0.295197684
NOXRED1	-0.060	0.079690664	0.295197684
DUSP8P5	-0.060	0.079665738	0.295156756
AC078864	-0.060	0.079655775	0.295142803
RNU6-663P	-0.060	0.079652082	0.295142803
KRT8P26	-0.060	0.079648548	0.295142803
RARRES2P5	-0.060	0.079638491	0.295142803
RP11-135N5	-0.060	0.079632115	0.295142803
RP11-173B14	-0.060	0.079619538	0.295123337
LINC00430	-0.060	0.079614972	0.295123337
AC079768	-0.060	0.079614691	0.295123337
ELMO1-AS1	-0.060	0.079610833	0.295123337
CASZ1	-0.060	0.079594192	0.295123337
ABI3	-0.060	0.079591279	0.295123337
C1orf43	-0.060	0.079576525	0.295123337
KLHL33	-0.060	0.079537741	0.295026791
SPECC1L	-0.060	0.079531151	0.295026791
SNORD116-16	-0.060	0.079530391	0.295026791
RNU1-1	-0.060	0.079453631	0.294829615
RNU6-75P	-0.060	0.079447352	0.294829286
IGLV5-37	-0.060	0.07938755	0.294676241
RP11-74K11	-0.060	0.079366385	0.294620641
RPP40P2	-0.060	0.079360423	0.294620641
LIN9	-0.060	0.07930422	0.294481686
RP11-78J21	-0.060	0.079283134	0.29442634
TRH	-0.060	0.079233913	0.294335352
AC092578	-0.060	0.07923262	0.294335352
RP11-136G2	-0.060	0.0792237	0.294335352
AC005517	-0.060	0.079213998	0.294330245
NXF1	-0.060	0.07920429	0.294330245
MED28P3	-0.060	0.079159454	0.294219394
RP11-715G15	-0.060	0.079122145	0.294103677
RP11-677I18	-0.060	0.079107754	0.294073135
TPO	-0.060	0.079084893	0.294021625
RP11-165N19	-0.060	0.079056751	0.293952367
MAGI2-AS1	-0.060	0.0790544	0.293952367

MIR299	-0.060	0.079051348	0.293952367
HCG4P11	-0.060	0.079027874	0.293936788
NUP107	-0.060	0.078974195	0.293805966
ASTN2	-0.060	0.078967658	0.293804597
TRIM36	-0.060	0.078946183	0.29379355
DNASE1	-0.060	0.078868072	0.293548741
RNU4-74P	-0.060	0.078860269	0.293542636
RP11-503I22	-0.060	0.078854759	0.293542636
PPM1A	-0.060	0.078840651	0.29351549
SEPT7P6	-0.060	0.078828493	0.293493171
RP11-285M22	-0.060	0.078824251	0.293493171
NOP58	-0.060	0.078824145	0.293493171
RP11-388G3	-0.060	0.078821682	0.293493171
AC195454	-0.060	0.078797296	0.293468989
ACSS3	-0.060	0.078782382	0.293459135
MT1DP	-0.060	0.078747434	0.2933519
AC002072	-0.060	0.078705336	0.293231535
MRPS33P3	-0.060	0.078681623	0.293175534
RP11-191L9	-0.060	0.078663392	0.293153471
LINC00461	-0.060	0.078632833	0.293062519
OR4C45	-0.060	0.078621705	0.293043977
YBX2P1	-0.060	0.078598468	0.292980295
RSL24D1P9	-0.060	0.078591204	0.292976148
RP4-584D14	-0.060	0.078562767	0.292915994
CUL9	-0.060	0.078561651	0.292915994
RP11-454L9	-0.060	0.078549669	0.292913019
AP003356	-0.060	0.078530659	0.292887995
RP11-768G3	-0.060	0.078516438	0.29285789
MAPK8IP1	-0.060	0.078495558	0.29280294
RP11-573M3	-0.060	0.078486783	0.292793141
PZP	-0.060	0.078453522	0.292691988
INTS6	-0.060	0.07843536	0.292647153
RNA5SP36	-0.060	0.078431569	0.292647153
RUNDC3A	-0.060	0.078402628	0.292593793
ZNF607	-0.060	0.078383925	0.292546916
AL391994	-0.060	0.078382701	0.292546916
EDNRB	-0.060	0.078372602	0.292546916
CELA3A	-0.060	0.078361428	0.292546916
UBA2	-0.060	0.078331589	0.29248911
MTND2P30	-0.060	0.078320088	0.292469096
HMGB1P11	-0.060	0.078315574	0.292469096
LINC01067	-0.060	0.078300892	0.292443269
RHOF	-0.060	0.078294674	0.292442981
ACACB	-0.060	0.078271123	0.292400877
RN7SKP160	-0.060	0.078208808	0.292213926
C3orf38	-0.060	0.07819652	0.292190935
MYOG	-0.060	0.078175378	0.292134855
CCND3P2	-0.060	0.078172096	0.292134855
RNA5SP423	-0.060	0.07814968	0.292107582
FSCB	-0.060	0.078140593	0.292107582
FAM151A	-0.060	0.078127803	0.292094581
RPS15AP32	-0.060	0.078125504	0.292094581

HLA-DRB1	-0.060	0.078072557	0.291979714
PTMAP6	-0.060	0.078066304	0.291979258
RPS11P5	-0.060	0.0780646	0.291979258
NMT1	-0.060	0.078032412	0.291898341
GOLGA8S	-0.060	0.077994241	0.291824316
ZMYM4	-0.060	0.077951987	0.291712053
GZF1	-0.060	0.077943949	0.291704891
CFHR3	-0.060	0.07791609	0.291623547
RP11-797J4	-0.060	0.077905245	0.291606673
SPCS2P1	-0.060	0.077887754	0.291606673
OMG	-0.060	0.077863571	0.291541531
IL17RA	-0.060	0.07779909	0.291323001
ANAPC10	-0.060	0.077776582	0.291270945
RP11-700A24	-0.060	0.077760958	0.291270945
RN7SKP105	-0.060	0.077741101	0.291220342
CBLL1	-0.060	0.077727711	0.291193083
SNORD64	-0.060	0.077721768	0.291193083
CTD-2017C7	-0.060	0.077720196	0.291193083
CD97	-0.060	0.077692317	0.291152097
GPR63	-0.060	0.077672967	0.291102487
RNU6-165P	-0.060	0.077663841	0.29109119
TMX2P1	-0.060	0.077644318	0.291053691
CDON	-0.060	0.077638699	0.291053691
SYS1	-0.060	0.077619636	0.291040027
OVCH1	-0.060	0.077618676	0.291040027
RPL31P59	-0.060	0.077617178	0.291040027
ERAL1	-0.060	0.077551611	0.290880718
C17orf78	-0.060	0.077545438	0.290880718
NR2F2	-0.060	0.077535326	0.290880718
TRMT112P6	-0.060	0.077518828	0.290845221
AC091493	-0.060	0.077420927	0.290546566
MAP4K3	-0.060	0.077420577	0.290546566
RP13-977J11	-0.060	0.077389431	0.290474147
NENFP1	-0.060	0.077380492	0.290463486
TRBV26	-0.060	0.077380391	0.290463486
MIR550A3	-0.060	0.077280501	0.290202508
AC010547	-0.060	0.077278952	0.290202508
RP11-425M5	-0.060	0.077250359	0.290135071
SNORD115-14	-0.060	0.077244795	0.290135071
SNORD111	-0.060	0.077225685	0.290111033
RAMP2-AS1	-0.060	0.077223584	0.290111033
LY86-AS1	-0.060	0.07722084	0.290111033
FBXW11P1	-0.060	0.077175768	0.290060786
TRIP11	-0.060	0.077152543	0.29001927
DHTKD1	-0.060	0.077120202	0.289920584
AL137800	-0.060	0.077082269	0.289800855
AC093157	-0.060	0.077081709	0.289800855
CTD-3028N15	-0.060	0.07706073	0.289788503
ERAP1	-0.060	0.07700449	0.289599877
AC007842	-0.060	0.077002706	0.289599877
AOX2P	-0.060	0.076985649	0.28957475
EFCAB14	-0.060	0.076941658	0.289432142

CTD-2531D15	-0.060	0.076922353	0.289418366
AC114755	-0.060	0.076868197	0.28927004
TNFRSF14	-0.060	0.07682799	0.289164428
RNF123	-0.060	0.076818676	0.289164428
RP11-565J7	-0.060	0.076758948	0.288984363
FFAR2	-0.060	0.076751951	0.288984363
C9orf78	-0.060	0.076743467	0.288983327
KRTAP25-1	-0.060	0.076710064	0.288903228
SRRM2	-0.060	0.076685915	0.288880808
RP5-942I16	-0.060	0.07666884	0.288862183
RN7SKP276	-0.060	0.076666989	0.288862183
FAM73A	-0.060	0.076654918	0.288855432
NEK5	-0.060	0.076654007	0.288855432
RSPH4A	-0.060	0.076633227	0.288819396
MIR140	-0.060	0.076602732	0.288727312
DLGAP1-AS5	-0.060	0.076541832	0.288566282
FBXW11	-0.060	0.07653413	0.288560087
KIAA1683	-0.060	0.076419134	0.288263432
CARS	-0.060	0.076407315	0.288241678
BRD7	-0.060	0.076395497	0.288219926
RP11-451N19	-0.060	0.076333912	0.288010397
GHRLOS	-0.060	0.076275587	0.287843817
SNORD89	-0.060	0.076257595	0.287829902
HS6ST2-AS1	-0.060	0.076256838	0.287829902
TCP10L2	-0.060	0.076247263	0.287829902
CTC-484M2	-0.060	0.076239474	0.287829902
SLC6A5	-0.060	0.076217209	0.287775252
RP5-827A12	-0.060	0.076175553	0.287663579
ASPDH	-0.060	0.076147918	0.287594438
AL122019	-0.060	0.076137125	0.287594438
RPS4XP16	-0.060	0.076132893	0.287594438
CTD-3080P12	-0.060	0.076130246	0.287594438
AL160231	-0.060	0.076067831	0.287439113
HSP90AB1	-0.060	0.076052435	0.28740374
LL22NC03-86G7	-0.060	0.076046186	0.287402929
RIMKLA	-0.060	0.076041477	0.287402929
NUDT3	-0.060	0.076024002	0.287364692
ZNF764	-0.060	0.07601187	0.287341641
PURA	-0.060	0.075978109	0.287236816
RCBTB1	-0.060	0.075962365	0.28720009
RP1-59D14	-0.060	0.07594029	0.287139425
AC007556	-0.060	0.075923353	0.287098177
RP11-496B10	-0.060	0.075862597	0.286891212
ARHGAP18	-0.060	0.07585322	0.286878527
POP1	-0.060	0.07584102	0.286855167
CICP17	-0.060	0.075838486	0.286855167
CTD-2213F21	-0.060	0.075831261	0.286855167
UBQLN1P1	-0.060	0.075810527	0.286808163
DGKD	-0.060	0.07578122	0.286720063
POLE3	-0.060	0.075780317	0.286720063
PKIB	-0.060	0.07577526	0.286720063
QTRTD1	-0.060	0.075772912	0.286720063

MIR4505	-0.060	0.075750798	0.286720063
MTTP	-0.060	0.07570052	0.286619658
KLC2	-0.060	0.075634682	0.286415917
MCCD1P1	-0.060	0.075584849	0.286295501
C1orf195	-0.060	0.075581341	0.286295501
MRPS7	-0.060	0.075576546	0.286295501
SNORD116	-0.060	0.075554401	0.286271236
RP11-52I18	-0.061	0.075453594	0.285912033
AC018761	-0.061	0.075444895	0.285901816
WASF3-AS1	-0.061	0.075419477	0.285828237
JADRR	-0.061	0.075410915	0.285818532
C10orf76	-0.061	0.075398129	0.285803041
DIAPH3	-0.061	0.075346433	0.285710555
AC007279	-0.061	0.075334026	0.285686255
MRE11A	-0.061	0.075297281	0.285592387
KLHL15	-0.061	0.075275723	0.285556104
RP11-163O19	-0.061	0.075269674	0.2855559
AP002381	-0.061	0.075263085	0.285553649
RP11-413E1	-0.061	0.07526292	0.285553649
GDAP2	-0.061	0.075260187	0.285553649
CTC-209L16	-0.061	0.075230895	0.2855225
KCNA5	-0.061	0.075219892	0.285503491
MTND4P11	-0.061	0.07520379	0.285465123
RNU6-1179P	-0.061	0.075201989	0.285465123
CTB-32P11	-0.061	0.075201989	0.285465123
CBX3P2	-0.061	0.075196184	0.285465123
RPS3AP24	-0.061	0.075163545	0.285403345
MIR3936	-0.061	0.075153703	0.285388726
MTHFD2P7	-0.061	0.07510759	0.285275706
AC007238	-0.061	0.075100396	0.285275706
RP11-474P2	-0.061	0.075072074	0.285215192
HIST1H3J	-0.061	0.07505946	0.285190019
KRT8P39	-0.061	0.075044088	0.285154363
RPL7P42	-0.061	0.075031555	0.285129489
LINC01318	-0.061	0.074957864	0.284904471
RP3-323P24	-0.061	0.074934702	0.284891773
RP11-290D2	-0.061	0.074923773	0.284879016
FAM8A1	-0.061	0.074915292	0.28486951
RN7SKP259	-0.061	0.074914647	0.28486951
SLC10A5	-0.061	0.074911356	0.28486951
CDV3	-0.061	0.074876831	0.284791475
FAM134C	-0.061	0.074860826	0.284753338
LINC01036	-0.061	0.074852932	0.284753338
HNRNPA3P14	-0.061	0.074838063	0.284734976
CELF2-AS1	-0.061	0.074813334	0.284663629
C10orf88	-0.061	0.074811575	0.284663629
CTD-2235C13	-0.061	0.074804331	0.284663629
MTATP8P1	-0.061	0.074752979	0.284524905
YPEL5	-0.061	0.074741453	0.284503771
RP11-472N19	-0.061	0.074738423	0.284503771
MIR4264	-0.061	0.074737944	0.284503771
RP5-1125A11	-0.061	0.074725292	0.284503771

EEF1A1P39	-0.061	0.074711115	0.284501979
PTOV1-AS2	-0.061	0.074708242	0.284501979
ZNF614	-0.061	0.074698246	0.284498468
RP11-307I2	-0.061	0.074652165	0.284368443
RN7SL430P	-0.061	0.074630335	0.284330772
UEVLD	-0.061	0.074624287	0.284330472
PIGH	-0.061	0.074612337	0.284307688
RP11-21K12	-0.061	0.074606172	0.284307688
SLC48A1	-0.061	0.074604134	0.284307688
AC084290	-0.061	0.074599487	0.284307688
LINC00354	-0.061	0.074585393	0.284307688
IGHD4-17	-0.061	0.074571374	0.284307688
OR6B2	-0.061	0.074566007	0.284307688
TRBV6-8	-0.061	0.074549162	0.284307688
IFNA21	-0.061	0.074486057	0.28414986
NBPF3	-0.061	0.07445447	0.284092537
RNF225	-0.061	0.074384812	0.283917729
RP11-56D16	-0.061	0.074377551	0.28391277
NBPF25P	-0.061	0.074377506	0.28391277
ZCCHC11	-0.061	0.074376884	0.28391277
AC108039	-0.061	0.074372483	0.28391277
FITM2	-0.061	0.074371637	0.28391277
POM121L6P	-0.061	0.074369975	0.28391277
ATP5G2P4	-0.061	0.074356763	0.28391277
RP4-717M23	-0.061	0.074349448	0.28391277
EIF2AK3	-0.061	0.074340427	0.28391277
MTND5P9	-0.061	0.07433597	0.28391277
TUBB8P2	-0.061	0.074335746	0.28391277
BNIP3P8	-0.061	0.074239865	0.283682769
OR4D5	-0.061	0.07422943	0.283665655
GEMIN8P4	-0.061	0.074224572	0.283665655
CLHC1	-0.061	0.074222797	0.283665655
RNU6-453P	-0.061	0.07419505	0.283638042
LINC01411	-0.061	0.074193099	0.283638042
RP11-406H4	-0.061	0.074186368	0.283638042
GUSBP10	-0.061	0.074174052	0.283638042
GPRASP1	-0.061	0.074173267	0.283638042
SCAF4	-0.061	0.074139996	0.283596948
RP11-701H24	-0.061	0.074128595	0.283576113
GPR34	-0.061	0.074083153	0.283493359
RN7SL361P	-0.061	0.074068724	0.283470102
SSU72P4	-0.061	0.074049528	0.283433003
THRAP3P1	-0.061	0.073991836	0.283257709
RP1-20B11	-0.061	0.073984189	0.283251204
SLC24A1	-0.061	0.073982413	0.283251204
TRPV2	-0.061	0.073973381	0.283251204
EMC2	-0.061	0.073971434	0.283251204
RNU6-933P	-0.061	0.073966075	0.283251204
SAPCD1-AS1	-0.061	0.073946073	0.283251204
RP1-224A6	-0.061	0.073923869	0.28322517
PAXIP1-AS1	-0.061	0.073917882	0.283225016
RP11-402L5	-0.061	0.073915237	0.283225016

RP11-1293J14	-0.061	0.073876713	0.283135608
RP11-443N24	-0.061	0.073861173	0.283121617
LINC01490	-0.061	0.073858938	0.283121617
CETN3	-0.061	0.073810431	0.282972663
TIMM23B	-0.061	0.073777558	0.282869406
ALS2	-0.061	0.073715644	0.282654778
ZNF527	-0.061	0.073707875	0.282647743
HAUS6	-0.061	0.073687837	0.282616412
ARID1A	-0.061	0.073687749	0.282616412
AC006978	-0.061	0.073687219	0.282616412
RAB11FIP2	-0.061	0.073682453	0.282616412
ABR	-0.061	0.073655979	0.282608017
AF238378	-0.061	0.073567515	0.282291334
MCOLN2	-0.061	0.073542935	0.282238391
LINC00987	-0.061	0.073532713	0.282226007
GATA2	-0.061	0.073510247	0.282162519
CTD-2373J6	-0.061	0.073502511	0.282155563
RP13-235E23	-0.061	0.073471178	0.282058018
OR5M11	-0.061	0.073463793	0.282052403
SUGT1	-0.061	0.073460402	0.282052403
RP11-526N18	-0.061	0.07344794	0.282037009
GDAP1	-0.061	0.073430296	0.282014734
CTA-714B7	-0.061	0.073425662	0.282014734
AC090181	-0.061	0.073409553	0.281980546
LY75	-0.061	0.073393602	0.281964758
RP11-685M7	-0.061	0.073388588	0.281964758
RP11-27I1	-0.061	0.073301274	0.281739112
XX-2136C48	-0.061	0.073296399	0.281739112
RAMP3	-0.061	0.07327131	0.281676705
B3GALNT1	-0.061	0.073260944	0.281659587
VN1R71P	-0.061	0.073251596	0.281646383
CDADC1	-0.061	0.07324566	0.281646297
RNY4P23	-0.061	0.073245491	0.281646297
RPL17P3	-0.061	0.073239576	0.281646297
RP11-439L8	-0.061	0.073220556	0.281626573
LRRC74A	-0.061	0.073171288	0.281496601
AC016706	-0.061	0.073160657	0.281478521
PPP2R5A	-0.061	0.073146903	0.281478521
RPL7AP6	-0.061	0.073141903	0.281478521
ASIC5	-0.061	0.073136928	0.281478521
RP11-725P16	-0.061	0.073117201	0.281447743
STPG2	-0.061	0.073110882	0.281446167
RP11-354P11	-0.061	0.073089251	0.281385639
RP11-107C10	-0.061	0.073086783	0.281385639
C11orf63	-0.061	0.073063419	0.281365467
AL355537	-0.061	0.073061235	0.281365467
RP11-452D12	-0.061	0.07304244	0.281341874
SPRYD4	-0.061	0.073036776	0.281341874
DPEP2	-0.061	0.073014872	0.281281186
MIR6891	-0.061	0.073011443	0.281281186
CES4A	-0.061	0.072966306	0.281139574
BNIP3P27	-0.061	0.07295533	0.281120024

SRP9P1	-0.061	0.072937744	0.281097744
GOLGB1	-0.061	0.072925821	0.281090074
MANSC4	-0.061	0.072913898	0.281083554
DEFB104A	-0.061	0.072905057	0.281083554
TCEB1P21	-0.061	0.072891249	0.281077768
INMT	-0.061	0.072885658	0.281077768
AP001619	-0.061	0.072843087	0.280960292
C11orf54	-0.061	0.072842543	0.280960292
ANKRD52	-0.061	0.072824795	0.280935242
RP11-554E23	-0.061	0.072796122	0.280870133
AC099668	-0.061	0.072789874	0.28086878
RP11-484D2	-0.061	0.072782462	0.280862934
LST1	-0.061	0.072778999	0.280862934
ANAPC1	-0.061	0.072760128	0.280824276
LOC100421166	-0.061	0.072748308	0.280822149
HNRNPA1P1	-0.061	0.072727995	0.280766492
FAM47E	-0.061	0.072724319	0.280766492
SIRT7	-0.061	0.072719866	0.280766492
RP4-700A9	-0.061	0.072667623	0.280624414
GTF2H2C	-0.061	0.072653356	0.280592067
CU459211	-0.061	0.072651645	0.280592067
OSER1-AS1	-0.061	0.07265004	0.280592067
EPHA10	-0.061	0.072638262	0.280592067
HSPD1	-0.061	0.072587331	0.280473537
RP11-31I22	-0.061	0.072570479	0.280431172
OR1J4	-0.061	0.072551215	0.280379482
ZNF431	-0.061	0.07250611	0.280250651
KIAA0232	-0.061	0.07246605	0.280159457
PURB	-0.061	0.072459695	0.280159457
WDFY1	-0.061	0.072441327	0.280136657
MIR331	-0.061	0.072436463	0.280136657
TSPYL2	-0.061	0.07240321	0.280034732
RN7SL333P	-0.061	0.072394729	0.280024668
FUCA1P1	-0.061	0.072386938	0.280017276
ATP6V0A2	-0.061	0.072370937	0.279978118
RP11-91I11	-0.061	0.072295808	0.279755641
ANKRD31	-0.061	0.072293517	0.279755641
CPNE3	-0.061	0.072287829	0.279755641
AMZ2P1	-0.061	0.072285222	0.279755641
CTB-174D11	-0.061	0.072248567	0.279709193
RP11-486F17	-0.061	0.072244315	0.279709193
RP4-566D2	-0.061	0.072225168	0.279689155
CFAP44	-0.061	0.072189869	0.279595583
AL592546	-0.061	0.072183467	0.279593526
OR52B5P	-0.061	0.072170568	0.2795663
CTC-550B14	-0.061	0.072163747	0.279562614
RP11-143G3	-0.061	0.072156593	0.279557637
AC096670	-0.061	0.072115044	0.279419393
RBBP5	-0.061	0.07210641	0.279408674
ATG2B	-0.061	0.072081614	0.279358045
MARK3	-0.061	0.072049804	0.279302949
RNU6-356P	-0.061	0.072016378	0.279196096

Z98949	-0.061	0.071986053	0.279146696
RP11-325N19	-0.061	0.071979115	0.279142523
FBXL19-AS1	-0.061	0.071979033	0.279142523
AC106775	-0.061	0.071953104	0.279114188
CTSC	-0.061	0.071950723	0.279114188
AGFG2	-0.061	0.071948819	0.279114188
SLC33A1	-0.061	0.071930816	0.279114188
TBC1D3B	-0.061	0.071921393	0.279114188
CCDC74A	-0.061	0.071914202	0.279114188
LINC01355	-0.061	0.07191325	0.279114188
RN7SL255P	-0.061	0.071893386	0.279102077
RP11-589F5	-0.061	0.071874589	0.279100781
AC007091	-0.061	0.071852102	0.279036209
RAB33A	-0.061	0.071832993	0.279007498
SPATA21	-0.061	0.071761284	0.278774441
RNU6-1311P	-0.061	0.071756792	0.278774441
NPTN-IT1	-0.061	0.071748699	0.278774441
TNFSF13	-0.061	0.071741927	0.278774441
NUS1P3	-0.061	0.071741267	0.278774441
CDKN1B	-0.061	0.071681844	0.278647665
COX6CP16	-0.061	0.071591695	0.27836539
LINC00707	-0.061	0.071579962	0.278342493
AL590096	-0.061	0.071566198	0.278311695
AC109333	-0.061	0.071546418	0.278280215
RP11-414J4	-0.061	0.071541335	0.278280215
RP11-365P13	-0.061	0.071528597	0.278256348
SRSF3	-0.061	0.071521997	0.278256348
RP11-274B18	-0.061	0.0715086	0.27824674
FAM215B	-0.061	0.071499388	0.278233628
AC012123	-0.061	0.071480647	0.278183429
RN7SL121P	-0.061	0.071477545	0.278183429
RP11-521C10	-0.061	0.071451615	0.278115894
MIR3143	-0.061	0.071442789	0.278104268
AC099344	-0.061	0.071442203	0.278104268
RP11-279N21	-0.061	0.071428885	0.278095603
RP1-91N13	-0.061	0.071407336	0.278034435
RP11-158C21	-0.061	0.071400216	0.278034435
AC138517	-0.061	0.071391412	0.278034435
SPATA25	-0.061	0.071378779	0.278034435
SNORD113-6	-0.061	0.071377493	0.278034435
LINC00691	-0.061	0.071362228	0.278034435
SHISA7	-0.061	0.071361944	0.278034435
PRICKLE4	-0.061	0.071350663	0.278034435
RP5-1068E13	-0.061	0.071283117	0.277823322
MIR200A	-0.061	0.071276427	0.277823322
SYT7	-0.061	0.071264821	0.277823322
HNRNPA1P51	-0.061	0.071255009	0.277823322
RP11-144G7	-0.061	0.071205164	0.277655832
POLR2K	-0.061	0.071190648	0.277621959
ENPP7P8	-0.061	0.071187428	0.277621959
AHCTF1	-0.061	0.071108307	0.277414429
RP11-661C3	-0.061	0.071051224	0.277259866

MAGEA8	-0.061	0.070963753	0.276941222
RP11-196G18	-0.061	0.070942918	0.276882601
NPM1P19	-0.061	0.070918783	0.276856465
FHDC1	-0.061	0.070857991	0.276641819
RNU6-131P	-0.061	0.070825655	0.276549185
DENND4C	-0.061	0.070815773	0.276544999
CTC-512J12	-0.061	0.070795851	0.276544999
HNRNPA1P6	-0.062	0.07078704	0.276544999
EXOC6	-0.062	0.070780578	0.276543607
KRT18P1	-0.062	0.070745008	0.276427312
HNRNPC	-0.062	0.070736689	0.276417485
CYCSP40	-0.062	0.070725751	0.27639742
RP11-219F10	-0.062	0.070723699	0.27639742
CTD-2619J13	-0.062	0.070701527	0.276348101
AP005530	-0.062	0.070701372	0.276348101
SLC9C1	-0.062	0.070694386	0.276348101
NPIPB3	-0.062	0.070667658	0.276303387
SPATS2	-0.062	0.07066254	0.276303387
RP11-9E13	-0.062	0.07061896	0.2761771
ACTN4P2	-0.062	0.070616653	0.2761771
AC005253	-0.062	0.070586068	0.276100745
AC010528	-0.062	0.070580449	0.276100745
RP11-32D17	-0.062	0.070560311	0.276045336
PSMA5	-0.062	0.070526353	0.275935153
PILRB	-0.062	0.070459702	0.275719678
IRGM	-0.062	0.070413185	0.275650894
AP004371	-0.062	0.070411262	0.275650894
RP11-121C6	-0.062	0.070387581	0.275595964
MIR6819	-0.062	0.070342623	0.27546523
SNORD124	-0.062	0.070329818	0.275437732
HMGB1P19	-0.062	0.070298458	0.275360198
RP11-939C17	-0.062	0.070254019	0.27524475
HMGB1P8	-0.062	0.070217156	0.275154887
RP11-259A24	-0.062	0.070207299	0.275138899
RP11-490B18	-0.062	0.070167574	0.275005845
LINC00854	-0.062	0.070163758	0.275005845
RP11-33E12	-0.062	0.070140865	0.274946419
RP11-234K19	-0.062	0.070074734	0.274732414
DNAJA3	-0.062	0.070053423	0.274671471
OSBP	-0.062	0.070047325	0.274670176
ACER3	-0.062	0.070045293	0.274670176
SNAP47-AS1	-0.062	0.070038978	0.274670176
LINC01276	-0.062	0.070007087	0.274648069
RNU6-886P	-0.062	0.069998795	0.274638157
RNU6-1147P	-0.062	0.069992223	0.274634997
UBP1	-0.062	0.069957525	0.274521466
AC007249	-0.062	0.069954525	0.274521466
RP13-643D4	-0.062	0.069943639	0.27451221
BBS7	-0.062	0.069941031	0.27451221
RNU6-895P	-0.062	0.069936885	0.27451221
RP11-380B4	-0.062	0.069915408	0.274491891
RN7SL784P	-0.062	0.069867308	0.274348278

RP11-299J3	-0.062	0.069835375	0.274312962
RP11-323J4	-0.062	0.069783713	0.274178262
SSTR5-AS1	-0.062	0.069780531	0.274178262
CANX	-0.062	0.069766474	0.274155763
SSTR5	-0.062	0.069766005	0.274155763
RP11-314M24	-0.062	0.069750641	0.274138783
AC244021	-0.062	0.069749549	0.274138783
PIP5K1A	-0.062	0.06974951	0.274138783
NAP1L1	-0.062	0.069729344	0.274138783
RP1-167G20	-0.062	0.06972479	0.274138783
RP11-414C16	-0.062	0.069694545	0.274076634
ALG2	-0.062	0.069688014	0.274073581
RPS12P20	-0.062	0.069672025	0.274066098
RTN3	-0.062	0.069641226	0.273980067
NIFKP7	-0.062	0.069622006	0.273949709
OR5P3	-0.062	0.069606406	0.273914681
UBE2F-SCLY	-0.062	0.069573702	0.273852191
MTND1P28	-0.062	0.069568177	0.273852191
LEKR1	-0.062	0.06956423	0.273852191
RNA5SP218	-0.062	0.069550139	0.273852191
RNU6-1267P	-0.062	0.069549138	0.273852191
S100A7L2	-0.062	0.069534941	0.273852191
SIX4	-0.062	0.069531818	0.273852191
SLC9A3R1	-0.062	0.069501017	0.273790316
AC087294	-0.062	0.069490328	0.273770854
RGS11	-0.062	0.069424988	0.273581325
RNU6-512P	-0.062	0.069411361	0.273550261
RNA5SP123	-0.062	0.069405073	0.273550261
SPINT4	-0.062	0.069341725	0.273388937
ACE	-0.062	0.069310737	0.273289382
RP3-394A18	-0.062	0.069299991	0.273269638
CTD-2302A16	-0.062	0.069272973	0.273185714
RP11-560J1	-0.062	0.069271205	0.273185714
GABRR2	-0.062	0.069252004	0.273170877
PARP8	-0.062	0.069238401	0.273139844
RNY4P36	-0.062	0.069217328	0.273106767
OR10G6	-0.062	0.069193191	0.273051959
SOCS2P2	-0.062	0.069185322	0.273043532
RP11-498M5	-0.062	0.069138221	0.272948112
KIAA2022	-0.062	0.069082642	0.272773916
PRELID1P4	-0.062	0.069030818	0.272614493
CCDC179	-0.062	0.068974014	0.272412752
AC010999	-0.062	0.068953785	0.272355443
HMGB1P41	-0.062	0.068923651	0.272258996
RP11-529F4	-0.062	0.068862286	0.272039159
GPC5	-0.062	0.068861564	0.272039159
NRADDP	-0.062	0.068844205	0.272039159
AC007435	-0.062	0.068842704	0.272039159
MAP3K14	-0.062	0.068840828	0.272039159
PHTF1	-0.062	0.068826637	0.272039159
RP11-102J14	-0.062	0.06880533	0.271994643
RP11-417P24	-0.062	0.068771532	0.271883601

AC064834	-0.062	0.068750036	0.271821181
RP13-20L14	-0.062	0.068747794	0.271821181
YPEL5P2	-0.062	0.068725524	0.271769389
AC004381	-0.062	0.068717695	0.271769389
HMGN2P31	-0.062	0.068714884	0.271769389
Z82214	-0.062	0.068714626	0.271769389
RP11-702H23	-0.062	0.068651196	0.271645424
RP4-753M9	-0.062	0.068647894	0.271645424
CTD-2510F5	-0.062	0.068627712	0.271630687
IZUMO2	-0.062	0.068615843	0.27160628
EIF1B-AS1	-0.062	0.068605315	0.271587179
IZUMO1R	-0.062	0.068576094	0.271494069
STYX	-0.062	0.068529176	0.271359688
NFKB1	-0.062	0.068514788	0.271341577
RP11-62L10	-0.062	0.06849543	0.271310033
PGAM5	-0.062	0.068484939	0.271291041
CSAD	-0.062	0.068418295	0.271072135
SLC1A2	-0.062	0.068384495	0.27096076
RP11-20E23	-0.062	0.068382879	0.27096076
EZH2	-0.062	0.068357265	0.270897943
AC103996	-0.062	0.068346375	0.270877325
EDC3	-0.062	0.06833909	0.270870991
GAS5-AS1	-0.062	0.068333032	0.270869523
RP3-453C12	-0.062	0.068332197	0.270869523
ZNF280B	-0.062	0.068330305	0.270869523
XXbac-BPG32J3	-0.062	0.068324764	0.270869523
ELAVL3	-0.062	0.068300928	0.270854981
GRM4	-0.062	0.068287157	0.270822919
SLC9A3P3	-0.062	0.068278544	0.270811309
TXNL4B	-0.062	0.068273806	0.270811309
APOC2	-0.062	0.068244624	0.270730153
PKP4	-0.062	0.06824396	0.270730153
KRT17P8	-0.062	0.068242349	0.270730153
DCDC1	-0.062	0.068184412	0.27061825
UNK	-0.062	0.068145628	0.270509403
RXFP4	-0.062	0.068141198	0.270509403
RP11-377D9	-0.062	0.068118075	0.270445111
RP11-77M5	-0.062	0.068092114	0.270387119
OR6Y1	-0.062	0.068034114	0.270201862
TRAF5	-0.062	0.067990786	0.270052301
AC092384	-0.062	0.067920134	0.269794182
RP5-892K4	-0.062	0.06786305	0.269612402
SETP8	-0.062	0.067860846	0.269612402
SLC10A7	-0.062	0.067851493	0.269611468
AGBL5-IT1	-0.062	0.06783424	0.269565402
DAOA	-0.062	0.067830685	0.269565402
CNN2P9	-0.062	0.067817879	0.269545365
UBR7	-0.062	0.067794341	0.269474302
NADSYN1	-0.062	0.06776714	0.269388664
PDCD1LG2	-0.062	0.067763596	0.269388664
RNF219	-0.062	0.067759146	0.269388664
ZBTB20-AS4	-0.062	0.067730032	0.269308585

SLMO2P3	-0.062	0.06771194	0.269259129
SLIT1	-0.062	0.067711447	0.269259129
KRT18P56	-0.062	0.067694843	0.2692361
RAB5A	-0.062	0.067691518	0.2692361
IQCB2P	-0.062	0.067683441	0.269235718
CGNL1	-0.062	0.067669207	0.269201582
RP3-380E11	-0.062	0.067658883	0.269182994
CTD-2008O4	-0.062	0.067543725	0.268859591
ENPP7P11	-0.062	0.067543221	0.268859591
PHF10	-0.062	0.067542455	0.268859591
BTF3P10	-0.062	0.067527962	0.268859591
MIER1	-0.062	0.067519609	0.268853477
NTSR2	-0.062	0.067508644	0.26883229
KIF5B	-0.062	0.067497099	0.26880879
RP11-864J10	-0.062	0.067467408	0.268735487
RNU6-930P	-0.062	0.067464523	0.268735487
AC026254	-0.062	0.06745212	0.268719543
CTD-2609K8	-0.062	0.06744643	0.268719543
ZNF853	-0.062	0.067428044	0.268697854
RP11-413B19	-0.062	0.067415841	0.268687388
RP6-159A1	-0.062	0.067405559	0.268668888
TAPT1-AS1	-0.062	0.067380618	0.268591957
RNU6-275P	-0.062	0.067357807	0.268523501
NRARP	-0.062	0.067336663	0.268461681
HSPA9	-0.062	0.067315514	0.268410068
ELAVL1	-0.062	0.067291666	0.268362548
RNU6-845P	-0.062	0.06727994	0.268347852
AC010970	-0.062	0.067198525	0.268045574
EIF4E3	-0.062	0.067188406	0.268027657
RNU6-253P	-0.062	0.067174083	0.268027657
KRT18P25	-0.062	0.067156757	0.268013645
MIR7974	-0.062	0.067145631	0.268013645
OGG1	-0.062	0.067131386	0.26797976
RP5-972B16	-0.062	0.067122899	0.267968343
TP53INP1	-0.062	0.067101133	0.267926365
AC058791	-0.062	0.067100888	0.267926365
OCM2	-0.062	0.06709438	0.267926365
ZNF585A	-0.062	0.067090589	0.267926365
RP11-37B2	-0.062	0.06708266	0.267926365
RP5-1154L15	-0.062	0.067045391	0.267838522
RP1-85D24	-0.062	0.06704446	0.267838522
AC025048	-0.062	0.067028263	0.267815022
RP11-496I9	-0.062	0.066996481	0.267732958
EPM2AIP1	-0.062	0.066990276	0.267730627
GPR180	-0.062	0.066972889	0.267706067
RP11-260A9	-0.062	0.066969041	0.267706067
RP4-809F4	-0.062	0.066961276	0.267704584
RP11-513D5	-0.062	0.066943271	0.267655069
CCDC97	-0.062	0.066941471	0.267655069
KB-1269D1	-0.062	0.066923255	0.267619971
AC024060	-0.062	0.066920968	0.267619971
TEK	-0.062	0.066917896	0.267619971

RP11-291O7	-0.062	0.066880012	0.267514432
RNY4P29	-0.062	0.066865482	0.267478777
RP11-365H23	-0.062	0.066809945	0.267301515
RP11-691N7	-0.062	0.066806344	0.267301515
CEACAMP11	-0.062	0.066774409	0.267224993
AC009410	-0.062	0.066755859	0.267197352
FAM193B	-0.062	0.066751592	0.267197352
ZNF671	-0.062	0.066728071	0.267131027
RP5-899E9	-0.062	0.066716391	0.26710672
DNHD1	-0.062	0.066692193	0.267032287
PRMT9	-0.062	0.066690566	0.267032287
RP11-163E9	-0.062	0.066689671	0.267032287
MIR6731	-0.062	0.066687378	0.267032287
LPIN1	-0.062	0.066679749	0.267032287
FNDC3A	-0.062	0.0666582	0.267030877
ACYP1	-0.062	0.066643404	0.267016522
CCDC108	-0.062	0.066635852	0.267008725
UBR1	-0.062	0.066631211	0.267008725
RP11-391J13	-0.062	0.066620651	0.267008725
CTC-459F4	-0.062	0.066620158	0.267008725
HRH2	-0.062	0.066605153	0.266998034
RP11-362L22	-0.062	0.066579346	0.266917045
TPT1P10	-0.062	0.066549321	0.266819137
SENP5	-0.062	0.066525132	0.266744609
AC005020	-0.062	0.066503442	0.266681799
GP5	-0.062	0.066491198	0.266675899
RP11-343B22	-0.062	0.066448389	0.266549097
EGFL8	-0.062	0.066427623	0.266488242
RPS26P35	-0.062	0.06634097	0.266230316
GABPB1-AS1	-0.062	0.066331122	0.266213227
HIF1AN	-0.063	0.066318572	0.266185938
ST13P19	-0.063	0.066288895	0.26614048
RNASE6	-0.063	0.066236759	0.265991409
NBPF5P	-0.063	0.066228668	0.265981346
AC004383	-0.063	0.066185295	0.26585199
RP11-399D15	-0.063	0.066168629	0.265829886
OLMALINC	-0.063	0.066145495	0.265759363
PDK1	-0.063	0.066127885	0.265711024
RNU2-30P	-0.063	0.066121643	0.26570836
CST3	-0.063	0.066103818	0.265659146
UNCX	-0.063	0.06608181	0.265593111
SULT2A1	-0.063	0.066023478	0.265411762
HCN3	-0.063	0.066009559	0.265411762
SYK	-0.063	0.066008108	0.265411762
AC115283	-0.063	0.065983038	0.26538404
NUDT12	-0.063	0.065968356	0.265361055
NXPE4	-0.063	0.065965074	0.265361055
C2orf42	-0.063	0.065963842	0.265361055
RP4-773N10	-0.063	0.065945868	0.265337828
AC010287	-0.063	0.065940813	0.265337828
GPR139	-0.063	0.065939568	0.265337828
ATP1B3-AS1	-0.063	0.065879595	0.265160776

TANK	-0.063	0.0658749	0.265160776
RNU1-130P	-0.063	0.065865145	0.265147431
TNRC18P3	-0.063	0.06586311	0.265147431
CD1C	-0.063	0.065829086	0.265047078
ZBTB37	-0.063	0.065821776	0.265047078
TMEM75	-0.063	0.065787941	0.265015839
ELOVL7	-0.063	0.065673938	0.264623744
AC004813	-0.063	0.065673839	0.264623744
PSMA2P3	-0.063	0.065649082	0.264590743
CTC-421K24	-0.063	0.065636169	0.264561087
AC116618	-0.063	0.065617274	0.264507315
NOL4L	-0.063	0.065613403	0.264507315
CTD-2591A6	-0.063	0.065606168	0.264507315
ACAP2	-0.063	0.065593502	0.264501035
RP5-1022P6	-0.063	0.065588208	0.264501035
TOMM22P5	-0.063	0.065583942	0.264501035
NACA2	-0.063	0.06556311	0.264445649
EIF3C	-0.063	0.06555973	0.264445649
DAZL	-0.063	0.065556047	0.264445649
ATF7IP	-0.063	0.065552994	0.264445649
RP11-214L13	-0.063	0.065535107	0.264445649
RP11-747E23	-0.063	0.065525302	0.264445649
PCM1	-0.063	0.065505212	0.264413657
EXD1	-0.063	0.065497929	0.264413657
RBM5-AS1	-0.063	0.065494291	0.264413657
BLNK	-0.063	0.065490066	0.264413657
RP5-997D16	-0.063	0.065485739	0.264413657
LINC00431	-0.063	0.065477794	0.264413657
AC011753	-0.063	0.06544154	0.264403141
MIR5007	-0.063	0.065439565	0.264403141
RPL34P17	-0.063	0.065425536	0.264399726
APOA5	-0.063	0.065356931	0.264150928
MPHOSPH10	-0.063	0.065344041	0.264121247
ZNF550	-0.063	0.065337963	0.264121247
RP11-241M13	-0.063	0.065332899	0.264121247
PKD2L1	-0.063	0.065328361	0.264121247
RP11-675F6	-0.063	0.065322499	0.264121247
MIR6719	-0.063	0.065320224	0.264121247
LNX1	-0.063	0.06529071	0.264062555
COTL1P2	-0.063	0.065289631	0.264062555
YWHAZP7	-0.063	0.065287381	0.264062555
MIR106B	-0.063	0.065278172	0.264062555
TNFAIP8	-0.063	0.065276324	0.264062555
RNU6-763P	-0.063	0.06526868	0.264062555
AC009473	-0.063	0.065207824	0.263906614
NAPA	-0.063	0.065187109	0.26386762
KIAA0319L	-0.063	0.065137712	0.263712496
GPR52	-0.063	0.065089918	0.263608632
SUMO2P3	-0.063	0.065059005	0.263505843
TMEM178B	-0.063	0.065052118	0.263500358
AC136289	-0.063	0.06505057	0.263500358
RP11-301N24	-0.063	0.065020257	0.263419145

RP11-739L10	-0.063	0.064999699	0.263377631
EXOG	-0.063	0.064997033	0.263377631
KIAA1107	-0.063	0.064973067	0.263322438
MTND2P16	-0.063	0.064961427	0.26331216
MIR5696	-0.063	0.064898381	0.263133657
TAS1R2	-0.063	0.064824442	0.262891152
DNM1P38	-0.063	0.064804777	0.262833784
AC138761	-0.063	0.064804468	0.262833784
RP6-29D12	-0.063	0.064801079	0.262833784
RNU6-653P	-0.063	0.064780064	0.262823077
AC016768	-0.063	0.064758661	0.262803406
TXLNB	-0.063	0.064750792	0.26279386
AL590410	-0.063	0.064748115	0.26279386
RNU6-462P	-0.063	0.064693796	0.262629681
AC091180	-0.063	0.064689273	0.262629681
XXbac-BPG248L24	-0.063	0.064689054	0.262629681
BSDC1	-0.063	0.064679853	0.262629681
FAXDC2	-0.063	0.064674222	0.262629681
ATXN3L	-0.063	0.064668726	0.262629681
NCRNA00250	-0.063	0.064667578	0.262629681
ZNF440	-0.063	0.064637749	0.262581206
RNU6-611P	-0.063	0.064637498	0.262581206
RP11-80B9	-0.063	0.064621403	0.262581206
CTD-3032J10	-0.063	0.06461551	0.262581206
AC139452	-0.063	0.064605441	0.262581206
RP11-806K15	-0.063	0.064556795	0.262409018
TTC19	-0.063	0.064503578	0.262237466
MKL2	-0.063	0.064451287	0.262047247
RP11-481J8	-0.063	0.064449285	0.262047247
TBRG1	-0.063	0.064423929	0.262003114
MIR1827	-0.063	0.064300367	0.261634649
PCIF1	-0.063	0.064294465	0.261632988
RP11-401N16	-0.063	0.064285331	0.261618172
RP11-161P17	-0.063	0.064268897	0.26159195
RP11-209M4	-0.063	0.064259932	0.261581862
STX7	-0.063	0.064243339	0.261536671
TFIP11	-0.063	0.064166161	0.261334168
RP11-757F18	-0.063	0.064144731	0.261313925
MIR6796	-0.063	0.06413135	0.261281761
RP5-884G6	-0.063	0.064130869	0.261281761
C19orf38	-0.063	0.064129688	0.261281761
SMARCA2	-0.063	0.064122758	0.261281761
RP11-571I18	-0.063	0.064101309	0.26127112
RP11-712C19	-0.063	0.064067488	0.261155618
AC074289	-0.063	0.064067178	0.261155618
RP11-37L2	-0.063	0.064040486	0.261112595
UNGP3	-0.063	0.064029939	0.261091944
NUP54	-0.063	0.063991906	0.2609592
RP11-621L6	-0.063	0.063982622	0.26095497
RP11-250H24	-0.063	0.063962759	0.260907359
FXYD6P1	-0.063	0.063962248	0.260907359
CTB-35F21	-0.063	0.063962015	0.260907359

ENPP7P3	-0.063	0.063945228	0.260904138
DCUN1D2	-0.063	0.063924088	0.260883681
SNORA55	-0.063	0.063919525	0.260883681
TMEM176B	-0.063	0.06391537	0.260883681
UBR5	-0.063	0.06387399	0.260813317
UBN1	-0.063	0.063817492	0.26064966
KIAA1328	-0.063	0.063789101	0.260556049
GNL3	-0.063	0.063781496	0.260547328
TRAPPC3L	-0.063	0.063763036	0.260505075
RP11-490O6	-0.063	0.063760775	0.260505075
MIR765	-0.063	0.063760468	0.260505075
AC098817	-0.063	0.063756846	0.260505075
RNA5SP43	-0.063	0.063750176	0.260505075
ARR3	-0.063	0.063743858	0.260505075
RN7SL352P	-0.063	0.063720874	0.26047842
RP11-279O22	-0.063	0.063714849	0.260476147
AC068535	-0.063	0.063709825	0.260476147
RP11-473C18	-0.063	0.063702331	0.260476147
NUP214	-0.063	0.063673921	0.260398233
GATA3-AS1	-0.063	0.063668231	0.260397322
SSX1	-0.063	0.063657138	0.260374316
RPL3P5	-0.063	0.063642752	0.260360191
POTEG	-0.063	0.063568882	0.260147375
Z69666	-0.063	0.063527549	0.260022909
KB-1552D7	-0.063	0.063521421	0.260020172
RP11-109F19	-0.063	0.063495079	0.259957029
QSER1	-0.063	0.063464529	0.259868587
AC137932	-0.063	0.063460001	0.259868587
AURKA	-0.063	0.063423547	0.259775822
LINC01494	-0.063	0.063410071	0.25974702
RP11-778O17	-0.063	0.063408921	0.25974702
RP11-131L12	-0.063	0.063398811	0.25974702
RERG-AS1	-0.063	0.063382196	0.259718134
GOLGA6A	-0.063	0.063352108	0.259617178
RP6-14C6	-0.063	0.063279942	0.2593884
CDS1	-0.063	0.063279772	0.2593884
SMARCE1P2	-0.063	0.063251388	0.259338312
IFITM4P	-0.063	0.063250521	0.259338312
TTC21B	-0.063	0.063222385	0.259264036
TRIQK	-0.063	0.063215068	0.259256356
KLHL42	-0.063	0.063201683	0.259223781
QRSL1P3	-0.063	0.063198282	0.259223781
SDAD1P4	-0.063	0.063198175	0.259223781
LAMTOR3	-0.063	0.063195847	0.259223781
OR11H3P	-0.063	0.063191151	0.259223781
ALG1L11P	-0.063	0.06318298	0.259223781
SORL1	-0.063	0.063181453	0.259223781
PRC1-AS1	-0.063	0.063130467	0.259154864
FAM192A	-0.063	0.063130203	0.259154864
TCEB1P22	-0.063	0.063115354	0.259137498
AC005007	-0.063	0.063093992	0.259116802
RNA5SP490	-0.063	0.06308373	0.259097

HLA-DMA	-0.063	0.063065314	0.259066045
CRNKL1	-0.063	0.063033318	0.258979281
RP11-69J7	-0.063	0.063013682	0.258943283
NCOA4	-0.063	0.063012279	0.258943283
RP11-379F12	-0.063	0.063009592	0.258943283
RNU2-40P	-0.063	0.062992359	0.258924634
SF3A1	-0.063	0.062992099	0.258924634
MMP25-AS1	-0.063	0.062988918	0.258924634
AL133405	-0.063	0.062954149	0.258854983
SUPT7L	-0.063	0.062928568	0.25877214
DDX21	-0.063	0.062912365	0.258727846
TMEM163	-0.063	0.062886632	0.258689031
RP11-681H10	-0.063	0.062876721	0.258686864
ZNF337	-0.063	0.062856724	0.258633018
PRR21	-0.063	0.0628024	0.258465044
RP11-498B4	-0.063	0.062787633	0.25846038
C19orf84	-0.063	0.062783252	0.25846038
KIAA1377	-0.063	0.062741619	0.258337953
CTD-2037K23	-0.063	0.062724818	0.258291103
TMEM56-RWDD3	-0.063	0.062706202	0.25826474
MIR6503	-0.063	0.062684662	0.258227354
RP11-332H17	-0.063	0.062664406	0.258227354
AIRN	-0.063	0.062660278	0.258227354
RP3-496C20	-0.063	0.062642145	0.258218576
RSPH10B	-0.063	0.062641354	0.258218576
GCAT	-0.063	0.062634412	0.258218576
DPY19L4	-0.063	0.062599663	0.258174148
BMS1P4	-0.063	0.062567707	0.258068084
LINC01264	-0.063	0.062507056	0.257840253
SNORA5C	-0.063	0.062504149	0.257840253
ZFAT	-0.063	0.062486643	0.257800705
VN1R38P	-0.063	0.062485311	0.257800705
OR13H1	-0.063	0.062482727	0.257800705
RP1-302G2	-0.063	0.062418306	0.25760803
MIR4727	-0.063	0.062393282	0.257571711
LINC00345	-0.063	0.062358035	0.257470839
HTR1E	-0.063	0.06229385	0.257295048
AC062021	-0.063	0.062285316	0.257282109
RP11-115E19	-0.063	0.062268551	0.257235171
RP11-521B24	-0.063	0.062259341	0.257219433
RP11-29H23	-0.063	0.062241666	0.257168718
FCHO2	-0.063	0.062240744	0.257168718
FAM72B	-0.063	0.062226607	0.257151115
TRPC5OS	-0.063	0.062197045	0.257051256
LINC00910	-0.063	0.062161565	0.256971517
PLA2G4F	-0.063	0.06211644	0.256829558
PPBPP1	-0.063	0.062095879	0.256766836
GTF2H3	-0.063	0.062085766	0.256747312
SLC22A5	-0.064	0.062067442	0.256712941
NDST2	-0.064	0.062047283	0.256655022
IL27	-0.064	0.062036	0.256630641
ZNF674-AS1	-0.064	0.062005755	0.256527803

KCTD18	-0.064	0.061966699	0.256388497
MTND6P18	-0.064	0.061966571	0.256388497
MED12L	-0.064	0.061963254	0.256388497
RP11-384C4	-0.064	0.061944882	0.25638732
ZSWIM8-AS1	-0.064	0.061942569	0.25638732
SRP72P1	-0.064	0.06190437	0.256308739
NSUN6	-0.064	0.061900658	0.256308739
MTRNR2L4	-0.064	0.061887557	0.256283688
RP11-87N3	-0.064	0.061862376	0.256201687
ZNF77	-0.064	0.061845452	0.256153867
CD1B	-0.064	0.061828049	0.256104062
MFSD4	-0.064	0.061820461	0.256094904
TMSB4XP3	-0.064	0.061808436	0.256067363
AP001628	-0.064	0.061789773	0.256012311
RN7SKP170	-0.064	0.061769519	0.255950661
CECR3	-0.064	0.061768247	0.255950661
AE000658	-0.064	0.061750212	0.255915188
ATP1A4	-0.064	0.061726346	0.255860808
BUB1	-0.064	0.061637455	0.255536828
TEX43	-0.064	0.061634758	0.255536828
RP11-666G4	-0.064	0.061630837	0.255536828
FBXO47	-0.064	0.061621053	0.255535559
NSUN3	-0.064	0.061609258	0.255508894
EIF4HP2	-0.064	0.06157216	0.255399517
IFI27	-0.064	0.06154338	0.255309569
LACTB2	-0.064	0.0615105	0.255254909
RP11-61A14	-0.064	0.061502974	0.255245918
RNU7-88P	-0.064	0.061472144	0.255140203
SNHG1	-0.064	0.061463109	0.255140203
NEK2P2	-0.064	0.061460147	0.255140203
E2F4P1	-0.064	0.061459068	0.255140203
AL109620	-0.064	0.061441085	0.255140203
PRR13	-0.064	0.061432014	0.255129274
POLR2KP2	-0.064	0.061425041	0.255122564
RP11-267J23	-0.064	0.061414761	0.255102112
SNAP29	-0.064	0.061379052	0.254976023
SSUH2	-0.064	0.06135809	0.25491118
OVOL1-AS1	-0.064	0.061357013	0.25491118
RWDD2A	-0.064	0.061322029	0.254805816
TRIM4	-0.064	0.06131277	0.254789571
LCN10	-0.064	0.06130963	0.254789571
AL157931	-0.064	0.061239314	0.254528737
SPATA13-AS1	-0.064	0.061223472	0.254507193
CTD-2655K5	-0.064	0.061204248	0.254483801
TRIM60P19	-0.064	0.061196233	0.254482929
RP11-49L2	-0.064	0.061186883	0.254466267
NPM1P36	-0.064	0.061186073	0.254466267
AL391261	-0.064	0.061149113	0.254353606
TRIM67	-0.064	0.061129013	0.25429221
MIR6512	-0.064	0.061119434	0.25427457
SNORD115-6	-0.064	0.061100742	0.254219013
RP3-471M13	-0.064	0.061066121	0.254097164

JAKMIP3	-0.064	0.061064946	0.254097164
FDCSP	-0.064	0.061057182	0.254097164
PGAM1P3	-0.064	0.061032358	0.254045457
BEND3P2	-0.064	0.060998392	0.253948464
DUTP8	-0.064	0.060964539	0.253829712
CCNA2	-0.064	0.060948104	0.253787265
TNPO1P1	-0.064	0.060929733	0.253773537
OR7C2	-0.064	0.060920436	0.253773537
MIR4793	-0.064	0.060919756	0.253773537
RRN3P1	-0.064	0.060912997	0.253773537
PHF14	-0.064	0.060892544	0.25375179
AC017006	-0.064	0.060867575	0.253714337
RP11-690D19	-0.064	0.06085867	0.253699422
IFIT6P	-0.064	0.060841341	0.253665976
SCARNA20	-0.064	0.060784271	0.253522418
AC006539	-0.064	0.060777496	0.253516359
TPR	-0.064	0.060763319	0.253479424
AC090286	-0.064	0.06070031	0.253260944
RP11-433M22	-0.064	0.060674223	0.253218649
ZNF724P	-0.064	0.060646954	0.253156744
CCR9	-0.064	0.060629428	0.253120421
NUP37	-0.064	0.060627403	0.253120421
UBE2Q2P2	-0.064	0.060617779	0.253116164
RP11-6B19	-0.064	0.060617297	0.253116164
VWA9	-0.064	0.060602294	0.253116164
KCTD9P1	-0.064	0.060590069	0.253089212
PIP4K2C	-0.064	0.060585008	0.253089212
CELSR3	-0.064	0.060522515	0.252940137
RP11-81B23	-0.064	0.060516947	0.252939066
GOLGA8R	-0.064	0.060497964	0.25290411
FAXC	-0.064	0.060470877	0.252835262
APOL2	-0.064	0.060467876	0.252835262
IQCC	-0.064	0.060458124	0.252826328
RN7SKP283	-0.064	0.060436916	0.252782031
PYROXD1	-0.064	0.060433823	0.252782031
TWF1P1	-0.064	0.060416047	0.252739136
RP11-570K4	-0.064	0.060391494	0.252680813
WNT3	-0.064	0.06038015	0.252655543
FPR2	-0.064	0.060363999	0.252610157
KIAA1407	-0.064	0.060362392	0.252610157
C1orf127	-0.064	0.060359871	0.252610157
IWS1	-0.064	0.060328538	0.252563186
RUNDC3B	-0.064	0.060299639	0.252496125
ZMIZ1	-0.064	0.060292679	0.252496125
RP1-74B13	-0.064	0.060261669	0.252463419
MIR3159	-0.064	0.060252596	0.252454509
RN7SL18P	-0.064	0.060239443	0.252439973
AC009263	-0.064	0.060233638	0.252439973
C12orf73	-0.064	0.060202771	0.252334538
MTOR	-0.064	0.060202472	0.252334538
MTR	-0.064	0.060200005	0.252334538
TFDP1	-0.064	0.060186103	0.252331295

RP4-562D20	-0.064	0.060165091	0.25226541
RNU7-79P	-0.064	0.060157435	0.252255515
ZNF248	-0.064	0.06015248	0.252255515
CCT2	-0.064	0.060115885	0.252125685
PIGQP1	-0.064	0.060087279	0.252027905
CTD-2179L22	-0.064	0.060073625	0.252015022
SERPINI1	-0.064	0.060058865	0.252015022
LRRC37A17P	-0.064	0.060020081	0.251879144
KCNAB2	-0.064	0.06001091	0.25186285
CTD-2011G17	-0.064	0.059999112	0.251835526
BTRC	-0.064	0.059989591	0.251817754
SYDE2	-0.064	0.059970364	0.251759234
ARNTL	-0.064	0.059967905	0.251759234
MTND4P8	-0.064	0.059967759	0.251759234
KRT222	-0.064	0.059937671	0.251688542
ANAPC5	-0.064	0.059914723	0.251636555
CSTF3-AS1	-0.064	0.059910425	0.251636555
AC073842	-0.064	0.059907126	0.251636555
Metazoa_SRP	-0.064	0.059867087	0.251525208
TPT1P2	-0.064	0.05983944	0.251431231
PLLP	-0.064	0.059838223	0.251431231
APOBEC2	-0.064	0.059806774	0.251338325
ARHGEF11	-0.064	0.059803348	0.251338325
RP11-47L3	-0.064	0.059795394	0.251334854
XXbac-BPG16N22	-0.064	0.059753284	0.251202196
TP53TG5	-0.064	0.059738403	0.251178633
PDSS1P2	-0.064	0.059717083	0.251116504
CST8	-0.064	0.059702074	0.251080173
CTD-2529O21	-0.064	0.059677166	0.251037307
GPC6-AS1	-0.064	0.059658767	0.250985154
M6PR	-0.064	0.059656821	0.250985154
RP3-462C17	-0.064	0.059655021	0.250985154
LINC00643	-0.064	0.059619844	0.250943988
BNIP2	-0.064	0.059602237	0.250909381
RP11-109A6	-0.064	0.05959213	0.2509012
CTD-3224K15	-0.064	0.059585938	0.250897308
MT-ND4L	-0.064	0.059581112	0.250897308
RP11-462G12	-0.064	0.059499338	0.25059913
TLDC2	-0.064	0.059471804	0.250562466
RP11-1399P15	-0.064	0.059444748	0.250479961
LINC01126	-0.064	0.059433938	0.250456569
RNA5SP317	-0.064	0.059422487	0.250452635
RP11-91P24	-0.064	0.059364806	0.250231667
WNT16	-0.064	0.059341373	0.250155032
AC092071	-0.064	0.059334305	0.250147379
SYP	-0.064	0.059331785	0.250147379
RP11-20I6	-0.064	0.059322553	0.250142117
MTND2P7	-0.064	0.059306943	0.250120586
ACCSL	-0.064	0.059295115	0.25009285
PRPF8	-0.064	0.059280695	0.250054171
CEP68	-0.064	0.05927944	0.250054171
STAM-AS1	-0.064	0.05927013	0.250054171

NEUROG1	-0.064	0.05926752	0.250054171
RNGTT	-0.064	0.059258631	0.250054171
AL450244	-0.064	0.059253728	0.250054171
HAGH	-0.064	0.059246163	0.250054171
FDXACB1	-0.064	0.059210837	0.24993659
NFYA	-0.064	0.059192259	0.249880317
MRPS18C	-0.064	0.059187689	0.249880317
FAM35A	-0.064	0.059182677	0.249880317
ZBTB46-AS1	-0.064	0.059155313	0.249790771
SLC25A5P5	-0.064	0.05915215	0.249790771
LINC00174	-0.064	0.059149667	0.249790771
XRRA1	-0.064	0.059133903	0.249766798
NR3C2	-0.064	0.059130074	0.249766798
RNU6-262P	-0.064	0.059101021	0.249685888
AL031594	-0.064	0.059057028	0.249574896
CPEB4	-0.064	0.059039176	0.24954374
PTGES3L	-0.064	0.059038882	0.24954374
AP5M1	-0.064	0.059006673	0.249450632
RP1-137K2	-0.064	0.059004668	0.249450632
ZDHHC6	-0.064	0.05899616	0.249450473
MEOX2	-0.064	0.058987854	0.249437498
PCBP2	-0.064	0.05896206	0.249350563
LTB	-0.064	0.058959121	0.249350563
PPP2R1B	-0.064	0.058947808	0.249334568
PNLIPP1	-0.064	0.058937151	0.249311635
PI4K2B	-0.064	0.058889431	0.249176167
RP3-428A13	-0.064	0.058880514	0.249160571
CTC-359M8	-0.064	0.058847012	0.249063063
CNOT3	-0.064	0.058837117	0.249063063
RP11-207C16	-0.064	0.058829059	0.249053475
VN1R105P	-0.064	0.058824829	0.249053475
CACYBPP2	-0.064	0.05882231	0.249053475
LRPPRC	-0.064	0.058813137	0.249053475
CTD-2085J24	-0.064	0.05881301	0.249053475
RP11-497J7	-0.064	0.058811224	0.249053475
IQSEC1	-0.064	0.0587866	0.249028674
IL17C	-0.064	0.058781153	0.249027856
AF106564	-0.064	0.058763064	0.248995414
RP11-410E4	-0.064	0.058751072	0.248988905
HIST1H4I	-0.064	0.058742125	0.24897314
TCEA1	-0.064	0.058736851	0.248972945
RN7SL225P	-0.064	0.058724785	0.248943954
RNU1-58P	-0.064	0.058706148	0.248909259
RP11-1003J3	-0.064	0.058668979	0.248773813
GUCD1	-0.064	0.058667452	0.248773813
CLDND1	-0.064	0.058600168	0.248592696
BCL2L13	-0.064	0.058536818	0.24836851
C16orf70	-0.064	0.058534919	0.24836851
AC104131	-0.064	0.058522689	0.24836851
RP11-216B9	-0.064	0.05850177	0.248307977
RNU1-103P	-0.064	0.058500997	0.248307977
HERC2P7	-0.064	0.058495741	0.248307977

PECAM1	-0.064	0.058461122	0.248201805
ZNF416	-0.064	0.058457547	0.248201805
FBXO38	-0.064	0.058456672	0.248201805
RP11-285A1	-0.064	0.058454868	0.248201805
RP11-156P1	-0.064	0.05845069	0.248201805
DCAF4L2	-0.064	0.058404068	0.2481144
EIF2S3L	-0.064	0.05839556	0.24811286
LINC01197	-0.064	0.058366632	0.248021734
SNORA81	-0.064	0.058338369	0.247945877
RP11-419C23	-0.064	0.058322927	0.24793654
AL593848	-0.064	0.058261056	0.247749938
RP4-591B8	-0.064	0.058214905	0.24757579
NKX1-1	-0.064	0.058214413	0.24757579
HLCS-IT1	-0.064	0.058206319	0.24757579
EED	-0.064	0.058197441	0.24757579
WNK1	-0.064	0.058115647	0.247264062
FN3KRP	-0.064	0.05808879	0.247171877
HEXA-AS1	-0.064	0.058078191	0.247148857
RP11-1336O20	-0.065	0.058068604	0.247130142
RNU6-204P	-0.065	0.058055456	0.247096265
RP11-713P17	-0.065	0.058040334	0.247053982
FAM63A	-0.065	0.058040185	0.247053982
LEAP2	-0.065	0.058032135	0.247053982
RNU7-59P	-0.065	0.058031145	0.247053982
ZNF706	-0.065	0.058005746	0.246995052
FIGNL1	-0.065	0.058001614	0.246995052
MAST4	-0.065	0.05798596	0.246977047
SERINC3	-0.065	0.057980816	0.246977047
NOL9	-0.065	0.057928592	0.246842887
NUDCD1	-0.065	0.05792309	0.246841713
RIPK3	-0.065	0.057921714	0.246841713
OR2AE1	-0.065	0.057912409	0.246840369
ADAL	-0.065	0.057908394	0.246840369
Nkx2-2as	-0.065	0.057907356	0.246840369
RP11-317N8	-0.065	0.057865122	0.246727141
ZNF645	-0.065	0.057852306	0.246694582
RP11-740P5	-0.065	0.057819992	0.246600946
MIR3198-1	-0.065	0.057811146	0.246585298
AP000944	-0.065	0.0578008	0.246582855
RP11-16P6	-0.065	0.05779058	0.246563824
RP11-44H4	-0.065	0.057779062	0.246558857
AGGF1	-0.065	0.057771722	0.246549626
LGALS9B	-0.065	0.057749837	0.246507541
IGBP1-AS1	-0.065	0.057726178	0.246465682
CTC-218B8	-0.065	0.057722206	0.246465682
HSDL2	-0.065	0.057702116	0.246409591
RPF1	-0.065	0.05767715	0.246344687
RP11-230F18	-0.065	0.057664526	0.246312857
COX15	-0.065	0.057662145	0.246312857
SCP2D1	-0.065	0.057629023	0.24620537
RP5-1055C14	-0.065	0.057573269	0.246011313
PIP5K1B	-0.065	0.05755951	0.245974589

TRERF1	-0.065	0.057535027	0.245893564
AC006159	-0.065	0.057522519	0.245882699
TUBB8P11	-0.065	0.057502498	0.245841244
IL4	-0.065	0.057482826	0.245803216
XPOT	-0.065	0.057450051	0.24574938
RNA5SP297	-0.065	0.057386776	0.245544872
RP11-186O14	-0.065	0.057364603	0.245472053
GNL3L	-0.065	0.057364534	0.245472053
LINC01535	-0.065	0.05734191	0.245419045
CTD-2339F6	-0.065	0.057301808	0.245313547
CRYL1	-0.065	0.05730129	0.245313547
ZNF32-AS2	-0.065	0.057300105	0.245313547
RDM1	-0.065	0.057281226	0.24529158
MIR4432	-0.065	0.057259961	0.245222566
SPTY2D1	-0.065	0.057236527	0.245145973
CH17-373J23	-0.065	0.057234466	0.245145973
AL358134	-0.065	0.057231459	0.245145973
LINC00305	-0.065	0.057209917	0.245145973
RHOT1P1	-0.065	0.057208916	0.245145973
IGHD2-8	-0.065	0.057187413	0.245110234
FRY	-0.065	0.057177411	0.24508942
CTD-2207O23	-0.065	0.057176583	0.24508942
RP11-27G24	-0.065	0.057167519	0.24508942
RP11-612B6	-0.065	0.057167311	0.24508942
AL133493	-0.065	0.057155706	0.245084616
AC016717	-0.065	0.057154377	0.245084616
RP11-667M19	-0.065	0.057137308	0.245071918
SYTL2	-0.065	0.057136597	0.245071918
RP11-393B14	-0.065	0.057124626	0.245071918
AL445183	-0.065	0.057117539	0.245071918
RP3-510H16	-0.065	0.057109524	0.245063094
DCTN4	-0.065	0.057084004	0.244975659
NCF4	-0.065	0.057076852	0.244967037
MFAP1	-0.065	0.057043889	0.244869472
RP11-266A24	-0.065	0.057037467	0.244869472
PRKCQ-AS1	-0.065	0.057030368	0.244869472
GLOD4	-0.065	0.057029736	0.244869472
OSGEPL1-AS1	-0.065	0.057024364	0.244869472
HMGB1P32	-0.065	0.057018047	0.244869472
DDB2	-0.065	0.057013014	0.244869472
APITD1	-0.065	0.057012871	0.244869472
AHI1	-0.065	0.056968298	0.244809993
UBE2W	-0.065	0.056964514	0.244809993
NBPF26	-0.065	0.056960158	0.244809993
HOGA1	-0.065	0.056946598	0.244805093
CTB-152G17	-0.065	0.056941526	0.244805093
TRIP12	-0.065	0.056940312	0.244805093
AC012314	-0.065	0.056938314	0.244805093
RP11-428L21	-0.065	0.056918965	0.244774677
NLRC4	-0.065	0.056882806	0.24470756
RP11-1379J22	-0.065	0.056869328	0.244671679
COPS2	-0.065	0.056867617	0.244671679

RP11-97C16	-0.065	0.056841026	0.244616206
RP11-383M4	-0.065	0.056785527	0.244443627
WDR43	-0.065	0.056754696	0.244355081
HMGN2P18	-0.065	0.056729634	0.244269258
KLKB1	-0.065	0.056680894	0.244125606
SPAG9	-0.065	0.056671745	0.244108275
FAM215A	-0.065	0.056668093	0.244108275
RP13-616I3	-0.065	0.05666569	0.244108275
RAP1AP	-0.065	0.056663081	0.244108275
WASF5P	-0.065	0.056655289	0.244108275
MIR4688	-0.065	0.056652478	0.244108275
CCR1	-0.065	0.056648358	0.244108275
RP5-1170D6	-0.065	0.05660352	0.24401302
OR2A15P	-0.065	0.056598652	0.24401302
BRI3BP	-0.065	0.056593937	0.24401302
AC079127	-0.065	0.056576907	0.243964542
RP11-430L17	-0.065	0.056576208	0.243964542
RP11-475A13	-0.065	0.05656625	0.243962765
RP11-314B1	-0.065	0.056550993	0.243941141
ADCY5	-0.065	0.056550275	0.243941141
TRIM27	-0.065	0.056546372	0.243941141
FAM20B	-0.065	0.05653665	0.243941141
RP11-124D2	-0.065	0.056523241	0.243941141
MIR548M	-0.065	0.056520463	0.243941141
RP11-355B11	-0.065	0.056503734	0.243914015
ASTL	-0.065	0.056486466	0.243883684
IGLVIV-65	-0.065	0.056476193	0.243867696
AC099676	-0.065	0.056430725	0.243709297
MIR4252	-0.065	0.056410663	0.243666848
TRGJP	-0.065	0.056394568	0.24361942
MDC1	-0.065	0.056373714	0.243551423
SLC24A5	-0.065	0.056354172	0.243533267
RP11-99L13	-0.065	0.056346767	0.243533267
SUPT4H1	-0.065	0.056339745	0.243533267
RNU6-964P	-0.065	0.056300995	0.243458098
PP2672	-0.065	0.056280822	0.243392962
SOCS5P1	-0.065	0.056259735	0.243323865
AC023481	-0.065	0.056229676	0.243215945
AC092905	-0.065	0.056177861	0.243013897
SNORD116-12	-0.065	0.056175491	0.243013897
PATL1	-0.065	0.056160555	0.242983172
AL592466	-0.065	0.056158394	0.242983172
MIR8084	-0.065	0.0561537	0.242983172
FBXO7	-0.065	0.056144186	0.242978568
TRAJ61	-0.065	0.056121274	0.242901484
NHLRC3	-0.065	0.056083613	0.242764811
LDHAP1	-0.065	0.056020676	0.242576301
IGHD6-6	-0.065	0.056017941	0.242576301
SNORA28	-0.065	0.056004609	0.242550835
ABRA	-0.065	0.055999349	0.242550108
KLF3	-0.065	0.055967835	0.242435662
MIR199B	-0.065	0.055918053	0.242264085

SMPD4P2	-0.065	0.055907163	0.242238943
NTAN1P2	-0.065	0.05586441	0.24211978
CPEB2	-0.065	0.055861108	0.24211978
LEPROTL1	-0.065	0.055834909	0.242057174
RPL7L1P12	-0.065	0.055786766	0.241893329
RP11-1000B6	-0.065	0.055753428	0.241792799
AC012365	-0.065	0.055752507	0.241792799
RNU5A-2P	-0.065	0.055741837	0.241792799
GBA2	-0.065	0.055739893	0.241792799
SUGT1P3	-0.065	0.055738139	0.241792799
OR1H1P	-0.065	0.055721796	0.241792799
MYO18B	-0.065	0.055712813	0.241792798
WASF4P	-0.065	0.05571272	0.241792798
MAPK9	-0.065	0.055708694	0.241792798
RP5-1142A6	-0.065	0.055696569	0.241788397
RNU6-768P	-0.065	0.055693358	0.241788397
PGS1	-0.065	0.055668193	0.241759989
RP11-455P21	-0.065	0.05565611	0.241744966
RP11-360K13	-0.065	0.05565492	0.241744966
MIR2053	-0.065	0.055626751	0.241699624
DNAJA2	-0.065	0.055623376	0.241699624
EIF4A1	-0.065	0.055604548	0.241653234
FBXL17	-0.065	0.055599859	0.241653234
AD000092	-0.065	0.055593911	0.241651108
ZNF555	-0.065	0.055592302	0.241651108
RP11-511B23	-0.065	0.055566606	0.241620629
RBAK	-0.065	0.055561242	0.241619363
ZNF225	-0.065	0.055546486	0.241577254
C1QTNF3-AMACR	-0.065	0.055533886	0.241544508
RP11-845C23	-0.065	0.055499558	0.241439299
SNHG14	-0.065	0.055482258	0.241386089
MLPH	-0.065	0.055465286	0.241334292
IGKV2-14	-0.065	0.055443894	0.241263256
RP11-703C10	-0.065	0.055438702	0.241262705
RTEL1-TNFRSF6B	-0.065	0.055393601	0.241088461
AC011516	-0.065	0.05537761	0.241040891
AICDA	-0.065	0.055375956	0.241040891
KIF2A	-0.065	0.055370862	0.241040891
RP11-302M6	-0.065	0.055355284	0.241009791
RNU1-134P	-0.065	0.055300289	0.240858424
EDC4	-0.065	0.055282701	0.240803841
RP11-436I24	-0.065	0.055277232	0.240802044
WWC3-AS1	-0.065	0.055267211	0.240780413
RNU6-880P	-0.065	0.055232096	0.24064944
STK10	-0.065	0.055185963	0.240492438
SLC25A32	-0.065	0.055157734	0.240435418
AIF1	-0.065	0.055154119	0.240435418
INCENP	-0.065	0.055142442	0.240412769
RUFY4	-0.065	0.055116172	0.240320233
[truncated: 376,336 more chars]
